# Whole-Exome Sequencing Analysis of Oral Squamous Cell Carcinoma Delineated by Tobacco Usage Habits

**DOI:** 10.3389/fonc.2021.660696

**Published:** 2021-05-31

**Authors:** Krishna Patel, Firdous Ahmad Bhat, Shankargouda Patil, Samapika Routray, Neeta Mohanty, Bipin Nair, David Sidransky, Mandakulutur S. Ganesh, Jay Gopal Ray, Harsha Gowda, Aditi Chatterjee

**Affiliations:** ^1^ Institute of Bioinformatics, International Technology Park, Bangalore, India; ^2^ Amrita School of Biotechnology, Amrita Vishwa Vidyapeetham, Kollam, India; ^3^ Department of Maxillofacial Surgery and Diagnostic Sciences, Division of Oral Pathology, College of Dentistry, Jazan University, Jazan, Saudi Arabia; ^4^ Department of Oral Pathology & Microbiology, Institute of Dental Sciences, Siksha’ O’ Anusandhan University, Bhubaneswar, India; ^5^ Department of Otolaryngology-Head and Neck Surgery, Johns Hopkins University School of Medicine, Baltimore, MD, United States; ^6^ Department of Surgical Oncology, Vydehi Institute of Oncology, Bangalore, India; ^7^ Department of Oral Pathology, Dr. R. Ahmed Dental College & Hospital, Kolkata, India; ^8^ Manipal Academy of Higher Education (MAHE), Manipal, India; ^9^ Genetics and Computational Biology, QIMR Berghofer Medical Research Institute, Brisbane, QLD, Australia

**Keywords:** oral cancer, mutational signature, M-class cancer, genome aberration, HNSCC

## Abstract

Oral squamous cell carcinoma (OSCC) is a common cancer of the oral cavity in India. Cigarette smoking and chewing tobacco are known risk factors associated with OSCC. However, genomic alterations in OSCC with varied tobacco consumption history are not well-characterized. In this study, we carried out whole-exome sequencing to characterize the mutational landscape of OSCC tumors from subjects with different tobacco consumption habits. We identified several frequently mutated genes, including *TP53*, *NOTCH1*, *CASP8*, *RYR2*, *LRP2*, *CDKN2A*, and *ATM*. *TP53* and *HRAS* exhibited mutually exclusive mutation patterns. We identified recurrent amplifications in the 1q31, 7q35, 14q11, 22q11, and 22q13 regions and observed amplification of *EGFR* in 25% of samples with tobacco consumption history. We observed genomic alterations in several genes associated with PTK6 signaling. We observed alterations in clinically actionable targets including *ERBB4*, *HRAS*, *EGFR*, *NOTCH1*, *NOTCH4*, and *NOTCH3*. We observed enrichment of signature 29 in 40% of OSCC samples from tobacco chewers. Signature 15 associated with defective DNA mismatch repair was enriched in 80% of OSCC samples. *NOTCH1* was mutated in 36% of samples and harbored truncating as well as missense variants. We observed copy number alterations in 67% of OSCC samples. Several genes associated with non-receptor tyrosine kinase signaling were affected in OSCC. These molecules can serve as potential candidates for therapeutic targeting in OSCC.

## Introduction

Oral cancer is a prevalent cancer in the Indian subcontinent among men (1). Usage of tobacco in both smoking and chewing forms is a significant risk factor associated with oral cancer development (2). Alcohol consumption, infection by human papillomavirus (HPV), and poor oral hygiene are other factors responsible for oral malignancies (2). Oral cancer is generally diagnosed at a late stage due to ignorance of early onset lesions. This is common in developing countries due to lack of awareness and poor access to medical care (3, 4). There are an estimated 354,864 new cases of oral cancer worldwide (2% of all cancers). Among them, 227,906 (64.22%) cases are reported from Asia, where India accounted for 107,424 (30.27%) incidences in 2018. The estimated number of deaths is recorded to be approximately 177,757, of which (75,290 deaths) 42% are recorded from India (5).

Several studies have investigated genomic anomalies associated with oral squamous cell carcinoma from patients with a history of alcohol consumption, cigarette smoking, tobacco chewing, and HPV infection. The cancer genome atlas (TCGA) head and neck squamous cell carcinoma study consists of 62% oral cavity tissue samples. It reported inactivating mutations in *TRAF3*, *CASP8*, *NOTCH1*, and *TP53* and activating mutations in *PIK3CA* (6). Similarly, mutations in genes such as *HRAS*, *MET*, *CDKN2A*, and *STKII*, including *TP53* and *PIK3CA* were reported from 80 OSCC patients in a Japanese cohort (7). Mutational landscape is delineated using sequencing techniques to understand the molecular mechanisms involved in oral cancer development (8, 9). Farah et al. reported that reduced expression of *BRCA1* and *BRCA2*, among other DNA repair genes, was responsible for the malignant transformation of oral leukoplakia to OSCC using whole-exome sequencing and other techniques (8). RNA-Seq analysis identified *PKLR*, *CST1*, and *C17orf77* as dysregulated genes between tobacco users and non-users of HNSCC samples (10). Our group recently reported that OKF6/TERT1 cell lines exposed to smoke lead to higher C>A transversions while chewing tobacco treatment results in C>G transversions (11). A recent study by Maitra et al. reported a similar observation of higher C>G transversion in gingiva-buccal OSCC patients with a tobacco-chewing history (12). Another study by our group employed whole-exome and RNA-Seq analyses of oral keratinocytes exposed to Shisha tobacco and demonstrated enrichment of Interferon signaling pathway and activation of MAPK1 pathway (13).

Most genome/exome studies in oral cancers have focused on smoking or chewing tobacco forms. Oral cancer is the predominant form of cancer in India, and studying gene alterations associated with tobacco usage habits in oral cancer patients is crucial. Despite many studies carried out on oral cancer to identify alterations at the gene level, the role of tobacco in cellular transformation is unclear. Here, we have performed whole-exome sequencing of oral cancer patient samples to identify genetic anomalies associated with tobacco usage habits. This study provides novel insights into understanding the role of tobacco in oral cancer and paves the way for the future course of disease management.

## Material and Methods

### Sample Details

Oral cancer samples were acquired with written consent from the patients from Burdwan Dental College and Hospital, Burdwan, Kolkata-700014 for this study. All study subjects voluntarily consented to participate in the study and gave informed consent. The Institutional Human Ethics Committee approved the study. Patients who had not undergone any chemo or radiotherapy before surgical resection were considered for genomic profiling. A small portion of surgically resected tumor tissue from patients was treated with RNA-later stabilization solution (Qiagen) and stored at −80°C until further use. Tumor tissue stored in RNA-later solution was used to sequence tumor DNA, and peripheral blood leukocyte samples were used to sequence germline DNA from 30 patients. The patient samples were stratified into three categories based on tobacco usage habits *i.e.* tobacco smokers hereafter referred to as smokers, tobacco chewers hereafter referred to as chewers, and patients with no-habits hereafter referred to as non-users. From each cohort, ten pairs of samples were used for sequencing. Clinicopathological features of oral cancer patients are presented in **Supplementary Table 1**.

### DNA Extraction

DNA was extracted from surgically resected tumor samples and peripheral blood using AllPrep DNA/RNA Mini Kit to generate libraries for Next-Generation sequencing. DNA quantity was assessed using Nanodrop and 1% agarose Gel Electrophoresis to check the integrity of the extracted DNA. Quantitation of DNA was done using Qubit Fluorometer QIAXPERT and Qubit. A minimum of 40 ng of DNA with a 260/280 absorbance ratio greater than 1.6 was used as input for the whole-exome library preparations using Agilent SureSelectXT Human All Exon V5 kit. The obtained libraries were diluted to a final concentration of 2 nm in 10 ul and were subjected to cluster amplification. Once the cluster generation was completed, the flow cells were loaded onto the sequencer. Sequencing was carried out on HiSeq X10 to generate 2 × 150 bp sequence reads at 100× mean sequencing depth.

### Whole-Exome Sequence Analysis

Raw reads were assessed for Phred score quality using FastQC (14). The open-source fastq-mcf (v1.1.2-806) command-line tool was used to detect and remove sequencing adapters, primers, and low-quality nucleotides at the ends of reads (15). Raw reads were acquired in Fastq format and analyzed using GATK good-practice workflow (Genome Analysis Toolkit, Broad Institute). Trimmed reads were aligned against human genome hg19 using BWA (Burrows-Wheeler Aligner)-MEM (Maximal Exact Matches) (v0.7.12) (16). Aligned reads were further sorted and indexed using Samtools (v1.2) (17). These binary alignment map (BAM) files were assessed for biases due to PCR duplicates, and likely duplicates were flagged using MarkDuplicates tool of Picard package (v1.140) of the GATK tool suite (Genome Analysis Toolkit, Broad Institute). It was further used to realign reads around known indels from population frequency databases and base quality score recalibration. Somatic variants were identified using Strelka (v.2.9.2), and variant annotation pipeline VariMAT—Variation and Mutation Annotation Toolkit (v2.4.1) was used for the annotations (18). Common variants reported in the population frequency databases such as dbSNP, 1,000G, and ExAC were removed (19–21). Exonic variants with alternate allele depth ≥five reads and allele frequency ≥5% were retained for the downstream analysis. Copy number alteration analysis was done using OncoCNV, and alterations with p-value 1 × 10^−5^ were considered to have a statistically significant copy number gain with the fold change threshold of ≥3 and copy number loss of ≤1 (22).

### Bioinformatic Analysis

Whole-exome sequence datasets were evaluated for human papillomavirus infection using VirusFinder and HPVDetector (23, 24). Binary alignment files were analyzed for HPV origin reads using VirusFinder using default parameters, and Fastq files were analyzed using HPVDetector for the presence of HPV. A default background database of HPV genome sequence obtained along with respective packages was used for the analysis. All the samples were analyzed for microsatellite instability using MANTIS with default parameters (25). Gene expression profiles from TCGA-Head and neck squamous cell carcinoma datasets were obtained from UALCAN (26). Single nucleotide variants were further analyzed using the Cancer Genome interpreter (https://www.cancergenomeinterpreter.org/) platform, and a list of experimentally validated oncogenic mutations was obtained. Mutually exclusive single nucleotide variants were identified using the “Mutual Exclusivity” module of the cBioPortal platform. Frequently mutated genes were queried for mutual exclusivity using “Head and Neck Squamous Cell Carcinoma (John Hopkins, Science 2011)”, “Head and Neck Squamous Cell Carcinoma (Broad, Science 2011)”, “Head and Neck Squamous Cell Carcinoma (TCGA, Firehose Legacy)” and “Oral Squamous Cell Carcinoma (MD Anderson, Cancer Discov 2013)” as background datasets. The mutually exclusive mutated gene pairs in the background datasets with a p-value <0.001 were considered statistically significant. Lollipop plot was generated using Mutation Mapper module of cBioPortal (27, 28).

### Druggable Genome Analysis

Genes affected by genomic alterations in at least three samples were further analyzed for the potential druggable target using DGIdb 3.0 (29). This database hosts the gene–drug interactions compiled and curated from different resources such as DrugBank, NCI, and CGI. Genes with recurrent SNVs or CNA in at least three samples were queried using API with gene attribute as main query along with accessory attributes such as source_trust_levels=Expert%20curated, fda_approved_drug=true and anti_neoplastic=true. Following is the link to API.

https://dgidb.org/api/v2/interactions.json?genes=<genelist>&fda_approved_drug=true&anti_neoplastic=true&source_trust_levels=Expert%20curated

### Pathway Enrichment Analysis

Genes with recurrent somatic copy number alterations and single nucleotide variants in at least three out of 30 samples *i.e.* 258 genes, were queried for pathway enrichment using Reactome (30). Pathways with at least five mapped genes with p ≤0.05 were considered.

### Somatic Signature

The high confidence somatic SNVs were analyzed to create trinucleotide mutation signatures using R package SomaticSignatures (31). Decomposing mutation signatures determined the peculiar trinucleotide COSMIC signatures in each sample using Mutalisk (32). Boxplots were generated using R, and heatmap was generated using Morpheus (https://software.broadinstitute.org/morpheus/).

### Statistical Analysis

Statistical significance for mutation load between each pair of cohorts in the study was determined using unpaired Welch’s t-test with a 95% confidence level. In transition and transversion comparison between cohorts, statistical significance was calculated using unpaired two-tailed Mann–Whitney test. Gene expression distribution differences were fetched from the UALCAN portal, and the p-value was calculated using the Comprehensive Perl Archive Network (CPAN) module “Statistics::TTest” (26). Mean differences with the p-value ≤0.05 were considered statistically significant.

## Results

### Clinical Characteristics of the Patient Cohort

Tumor tissue and blood samples were collected at the time of surgical excision from 30 treatment naïve OSCC patients. These samples underwent histopathological staging, and tumor sections with >80% tumor nuclei in total cellular nuclei were used for DNA isolation and library preparation. Most patients were male (60%). As tobacco smoking habit is more common among males in India, they constitute our entire smoker cohort. In the tobacco chewer and non-user cohorts, we had representation from both male and female. In each cohort, 60% of the samples were from females and 40% from males. About 60% of patients were between the age group of 40 and 60 years. A mixture of anatomical sites was included, such as buccal mucosa (36.7%), tongue (26.7%), lip (10%), alveolus (10%), and others (16.7%). Among the enrolled patients, 33% chewed tobacco, 33% smoked tobacco/cigarette, and the rest had no history of tobacco consumption. About 53% of patients were presented at an advanced stage III/IV. Histopathological examination of regional nodes confirmed nodal metastasis in 70% of cases. Clinicopathological details of all the patients are provided in **Supplementary Table 1**.

### Mutational Landscape of Oral Squamous Cell Carcinoma

**Figure 1:**
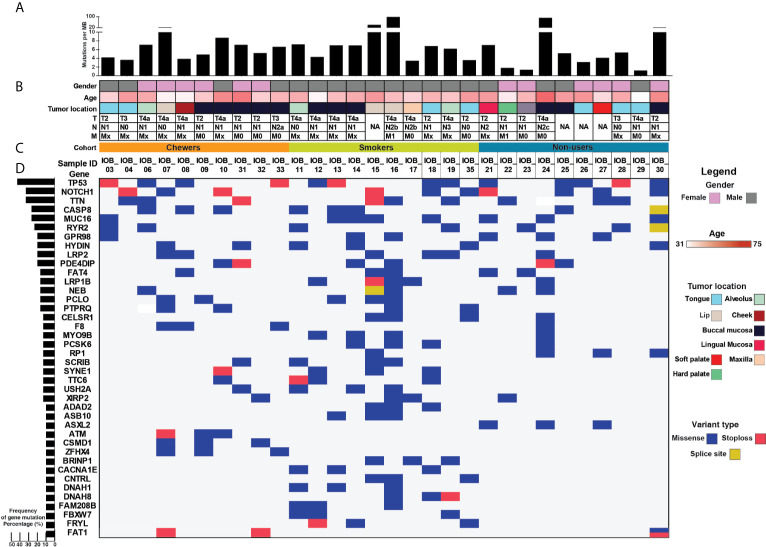
>Mutational landscape of oral squamous cell carcinoma **(A)** Top panel depicts mutation load per Mb. **(B)** Sample details such as age, gender, tumor location, and TNM staging. **(C)** Each row depicts a gene, and each column represents a sample. Somatic mutations are highlighted based on mutation type, and the left panel depicts the percentage of samples that harbor mutations. **(D)** Bar graph represents gene mutation frequency in 30 oral squamous cell carcinoma samples.

**Figure 2:**
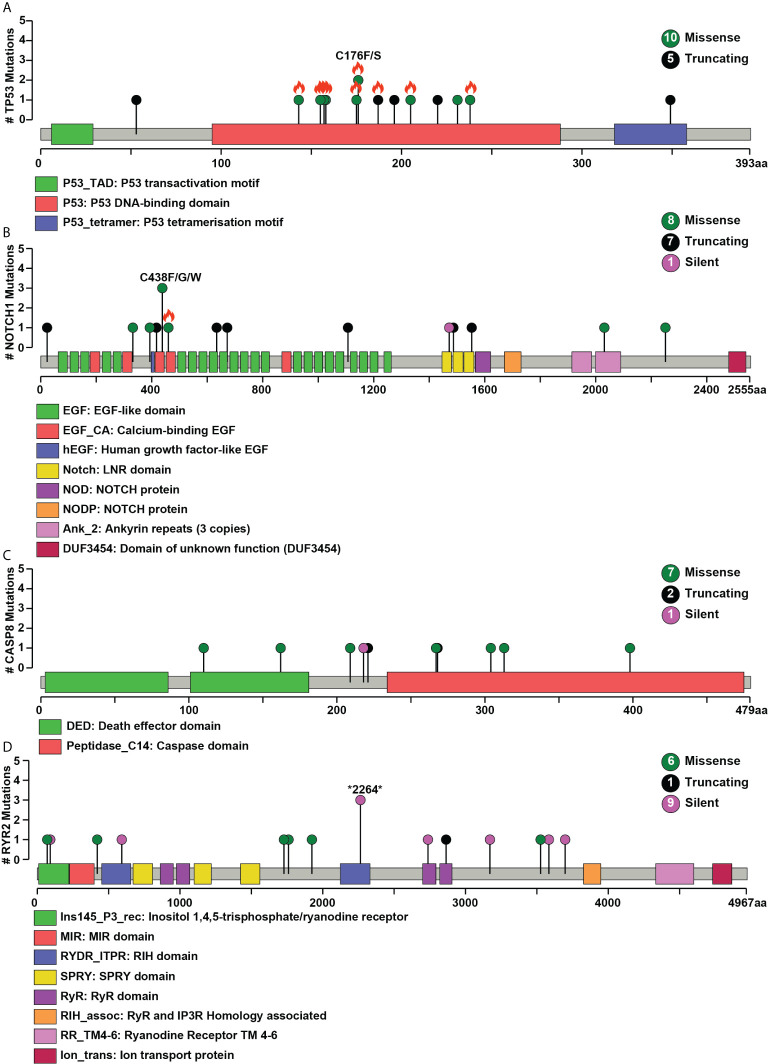
Pictorial representation of single nucleotide variant distribution on proteins’ domain structure **(A)** *TP53*, **(B)** *NOTCH1*, **(C)** *CASP8*, **(D)** *RYR2*. Green represents missense, black represents a splice site, and purple indicates silent mutations. The total number of different types of mutations for each gene is given within the circle on the right side. The x-axis depicts the length of the protein (amino acid), and the y-axis represents the number of samples. Orange flame indicates COSMIC hotspot mutations.

Whole-exome sequence analysis of 30 OSCC samples consisting of three different cohorts *i.e.* smokers, chewers, and tobacco non-users, was carried out using GATK analysis pipeline. An average of 77 million reads was acquired with an average base quality of 38.32. The samples’ on-target coverage ranged from 82.94 to 89.72%, and average panel depth for more than 90% of the samples ranged from 101.25 to 151.49×. Three samples had depth greater than 152×. Sequencing statistics for each patient is provided in **Supplementary Table 2**. Among 30 tumors, we observed 6,179 non-synonymous SNVs in 4,482 genes and 3,070 synonymous SNVs in 2,490 genes **[** **Supplementary Figure 1** **]**. The median mutation load per megabase (MB) per tumor was 5.31 (2.9 non-synonymous variants) in OSCC **[** **Supplementary Table 3** **]**. There are 911 genes mutated in ≥2 tumors, of which 108 are recurrently mutated in >16% of tumor samples **[** **Supplementary Table 4** **]**. The most frequently mutated genes across all the tumors were *NOTCH1* (30%), *CASP8* (26.6%), *RYR2* (23.3%), and *LRP2* (20%), among others **[** **Figure 1** **]**. Mutations in genes involved in P53 signaling pathway such as *TP53* (43.3%), *CDKN2A* (10%), *ATM* (10%), and *CASP8* (26.6%) were also among the top gene mutations across tumors. In the case of *TP53*, eight out of 15 mutations are known hotspot mutations present on the P53 DNA-binding domain of the gene **[** **Figure 2A** **]**. We also observed recurrent variant p.Gly12Ser in *HRAS* (13.3%), p.Leu556Val in *CELSR1* (6.6%), and p.Ala737Thr in *SHANK3* (6.6%). Out of 30, nine samples (30%) had at least one known loss of function/activating mutation in actionable genes such as *PTEN*, *CDKN2A*, *ERBB4*, and *TP53*. We observed 13 SNVs in *NOTCH1* from 11 samples, among which five were truncating and eight were missense variants. Positional recurrent variant p.Cys438Trp/Phe/Gly of *NOTCH1* was observed in smoker and chewer samples, which was predicted to be deleterious by SIFT, Polyphen, and LRT with an allele frequency of 19% **[** **Figure 2B** **]**. We have also observed 10 point mutations in *CASP8* out of which seven are missense and two are truncating mutations **[** **Figure 2C** **]**. *CASP8* mutations have been found increasingly in oral tumor tissues compared to leukoplakia (33). Similarly, for *RYR2* gene, one truncating and six missense mutations were found **[** **Figure 2D** **]**. Mutations in *RYR2* gene are known to occur during transformation of oral dysplasia to cancer (34). *PIK3CA* was also found to be mutated at position p.Glu545Lys and p.His1047Arg in smoker and chewer samples. *FAT1* was observed to have a recurrent truncating mutation in 10%, and *FAT4* had missense variants in 16% of OSCC samples. Genes *MUM1* and *NBPF3* harbored positionally recurrent variants; however, they were predicted to be passenger variants as per Cancer Genome Interpreter mutation analysis (35). In concordance with Biswas et al.’s study, we observed recurrent mutations in gene *XIRP2* in three tumor samples with nodal metastasis (36).

### Single Nucleotide Variants in OSCC Cohorts

We evaluated the mutational landscape to differentiate mutation patterns among the chewer, smoker, and non-user cohorts. Thirteen genes (*ADAD2*, *ASB10*, *BRINP1*, *CACNA1E*, *CNTRL*, *DNAH1*, *DNAH8*, *FAM208B*, *FBXW7*, *FRYL*, *MUM1*, *PCSK6*, and *BRCA2*) were recurrently mutated in the smoker cohort. All these genes, except *FBXW7*, are predicted passenger mutations. We identified hotspot variant p.Arg505Gly located in substrate recognition domain (WD40) of *FBXW7* in two out of 10 OSCC samples from smokers. Similarly, genes *ATM* and *ZFHX4* were recurrently mutated in three samples from the chewer cohort. Non-sense variant p.E1996* and missense variant p.A1014D in tumor suppressor *ATM* are predicted as driver mutations according to OncodriveMUT, whereas alterations in gene *ZFHX4* are predicted to be a passenger mutation (35). Alterations in *ATM*, a DNA repair gene, have been reported in various cancers like prostate, lung, and oropharyngeal cancer (37). All single nucleotide variant harboring genes identified in the non-user cohort are also observed in the chewer and smoker cohorts, except *ASXL2*.

### Mutational Signatures in OSCC Cohorts

**Figure 3:**
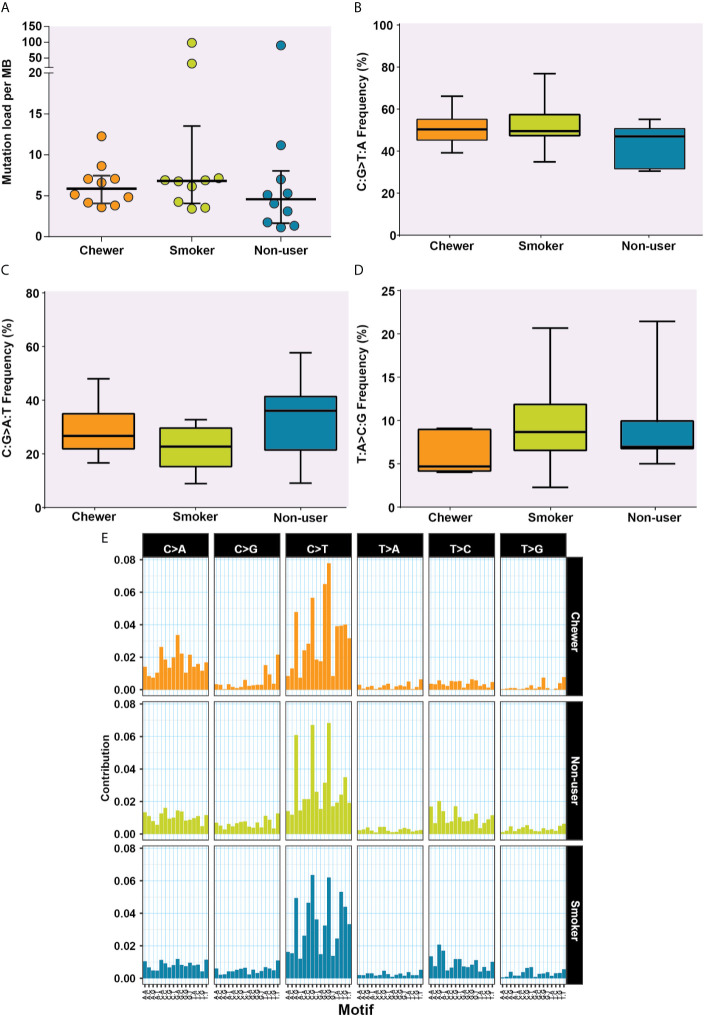
Analysis of mutation signatures enriched in oral squamous cell carcinoma from smokers, chewers, and tobacco non-users. **(A)** Mutation load in chewer, smoker, and non-user cohort. **(B)** C:G>T:A transitions in smokers, chewers, and tobacco non-users. **(C)** C:G>A:T transitions in smokers, chewers, and tobacco non-users. **(D)** T:A>C:G transitions in smokers, chewers, and tobacco non-users. **(E)** Mutation signatures associated with OSCC from smokers, chewers, and tobacco non-users. The height of the bar represents the contribution of the base substitution across different trinucleotide contexts.

We evaluated SNVs identified across three OSCC cohorts for mutational signatures. The median mutation load was 5.88 SNVs per MB in chewers, 6.82 SNVs per MB in smokers and 4.58 SNVs per MB in patients without a history of tobacco consumption **[** **Figure 3A** **]**.

#### Transition and Transversion Mutational Signatures

The transition of C:G>T:A was observed among 50.78, 52.53, and 44% SNVs in chewers, smokers, and non-users, respectively **[** **Figure 3B** **]**, whereas transversion of C:G>A:T was contributed by 28.45, 22.15, and 32.94% SNVs in chewers, smokers, and non-users, respectively **[** **Figure 3C** **]**. The transition of T:A>C:G was contributed by 5.99% in the chewer cohort, whereas it was contributed by 9.63 and 8.99% in the smoker and non-user cohorts respectively; however, the differences were not statistically significant **[** **Figure 3D** **]**. Samples with larger number of variants *i.e.* IOB_24 (n = 3,500), IOB_15 (n = 1,266), and IOB_16 (n = 3,810) had higher proportion of C:G>T:A **[** **Supplementary Figure 2** **]**. In concordance with previous reports (38), we observed higher contribution of C:G>T:A related signature in all cohorts. A relatively higher contribution of C:G>A:T transversion signature was observed in the chewer cohort than in the smoker and non-user cohorts. A slightly higher contribution of T:A>C:G associated signature was observed in the smoker and non-user cohorts **[** **Figure 3E** **]**.

#### COSMIC Mutational Signatures

**Figure 4:**
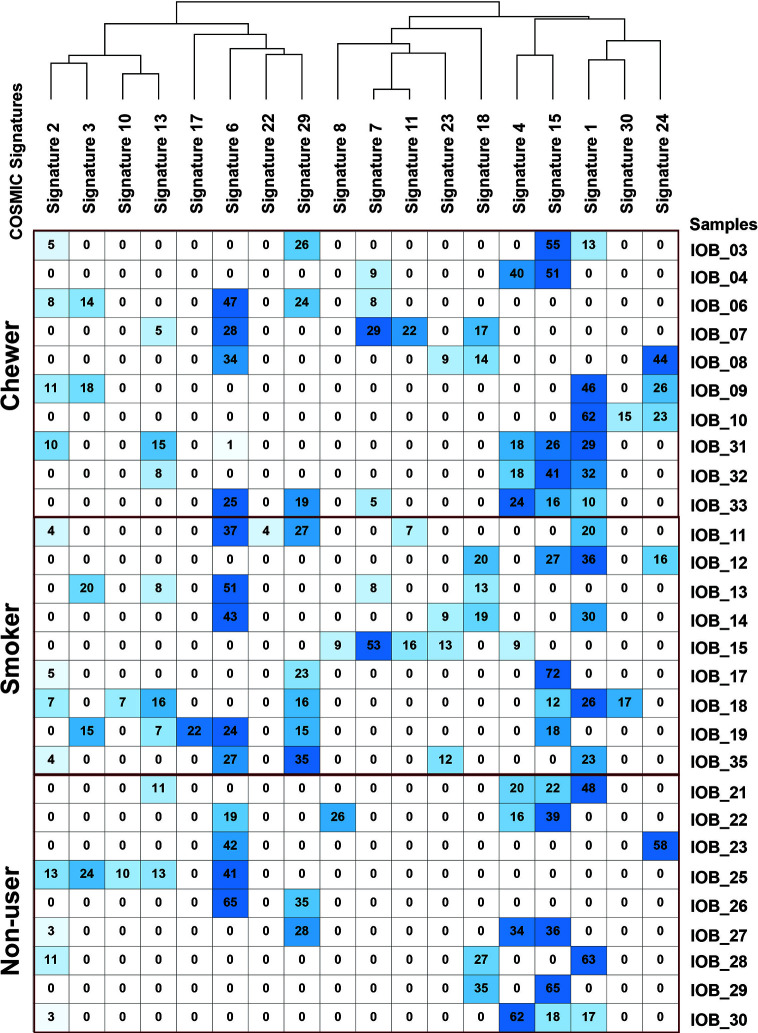
Mutation signature decomposition in oral squamous cell carcinoma samples from chewers, smokers, and tobacco non-users. Columns represent COSMIC signatures, and row represents samples. The percentage contribution of COSMIC signature predicted by Mutalisk is presented in the matrix where the row-wise lowest percentage is depicted by white, and the highest percentage is depicted by blue.

We further deconstructed somatic signatures using COSMIC somatic signatures as background to predict its percentage contribution. Signature 1 was predicted in 47% of the samples with more than 10% contribution. Signature 6 and signature 15, characterized by C:G>T:A, are predicted in 80% of the samples with ≥12% contribution. Signature 15 and signature 6 are predominantly associated with defective DNA mismatch repair, and signature 6 is generally found in microsatellite unstable tumors. Hence, we further assessed the whole-exome dataset for microsatellite instability (MSI) using MANTIS and did not find MSI incidences (25). Signature 29, which is associated with gingivo-buccal oral squamous cell carcinoma, was enriched in 40% of the tumors with a history of tobacco chewing and two samples from a non-user cohort with a contribution of 28 and 35% **[** **Figure 4** and **Supplementary Figure 3** **]**.

### Mutually Exclusive Variants

Single nucleotide variants were analyzed for mutual exclusivity using cBioPortal. We observed a mutually exclusive mutation arrangement in *TP53* and *HRAS* **[** **Supplementary Figure 4A** **]**. *HRAS* harbored a known activating variant p.Gly12Ser in four samples, whereas *TP53* was mutated in 13 samples. A similar mutually exclusive mutation pattern was observed in TCGA-HNSCC samples with primary site annotation of “floor of the mouth,” “base of the tongue,” “lip,” and “other ill-defined sites in the lip, oral cavity, and pharynx” **[** **Supplementary Figure 4B** **]**. Of note, mutual exclusivity was observed between a known oncogene and a tumor suppressor gene.

### Copy Number Alterations in Oral Squamous Cell Carcinoma

Copy number variants (CNVs) were identified using WES of tumor and matched normal coverage results. Recurrent focal copy number variants were filtered for segments with loss of at least one copy or gain of three or more copies in at least two samples with a p-value significance threshold of 1 × 10^−5^. **Supplementary Figure 5** depicts raw copy number estimates for 30 OSCC primary tumors. We report recurrent focal gain in small segments of 1q31, 7q35, 14q11, 22q11, and 22q13 regions containing 31, 9, 9, 17, and 4 genes, respectively **[** **Supplementary Table 5** **]**. There are 20 genes from these regions that are recurrently affected by copy number gain events in ≥ five samples. Out of 30 samples, 10 samples have ≥50 CNA affected genes. We identified more than 50 CNV affected genes in 10 samples out of which six were from the chewer cohort. We also observed 33 genes affected by CNV in 70% of samples from the smoker cohort. In the case of the non-user cohort, 90% of the samples exhibited ≤34 genes affected by copy number alterations. Further, we observed exclusive copy number gain of 23 genes from 11q22 in two out of 10 samples with relatively high number of CNA affected genes. These two samples belonged to patients from the chewer and smoker cohorts. Gene ontology enrichment analysis of these 23 genes revealed enrichment of molecular function metallopeptidase activity using FunRich **[** **Supplementary Figure 6** **]**. *EGFR* was amplified in two chewers and three smoker samples. *EGFR* amplifications have been reported in ESCC and HNSCC and are significantly associated with advanced tumor stages as well as lymph node metastasis in oral squamous cell carcinoma (39, 40).

### Clinically Actionable Gene Targets

We compared our findings with The Drug Gene Interaction Database to identify clinically actionable gene targets. Genes with recurrent alterations in at least three out of 30 samples were mapped against gene–drug interaction database DGIdb **[** **Supplementary Table 6** **]**. This analysis led to the identification of 12 targetable genes. Actionable targets included *TP53*, *NOTCH1*, *CASP8*, *EGFR*, *HRAS*, *ATM*, *ERBB4*, *EPHA2*, *NOTCH4*, *NOTCH3*, *MUC16*, and *POLD1* **[** **Supplementary Table 7** **]**. These genes can serve as potential candidates for targeted therapy in oral cancer, as FDA approved drugs are available targeting these genes. Out of 12, three genes (*ERBB4*, *HRAS*, and *EGFR*) mapped to signaling by PTK6 **[** **Supplementary Table 8** **]**. Expression of *PTK6* is often found co-amplified with members of the EGF receptors including *ERBB4* in breast cancer (41). *PTK6* enhances *EGFR* induced activation of PI3K/AKT pathway which regulates various cellular processes important for carcinogenesis (42). Studying the therapeutic potential of PTK6 signaling in a larger cohort of OSCC patients could be promising. Patients enrolled in the current study underwent standard treatment regimen, and treatment decisions were not based on the outcome of this study. Unfortunately, patient follow-up is extremely poor in India; therefore the genomic alterations could not be assessed for prognostication.

## Discussion

This report presents a catalog of somatic mutations observed in oral squamous cell carcinoma tumors from patients with a history of tobacco chewing and cigarette smoking along with tobacco non-users. Tumors from patients with a history of smoking and chewing tobacco had a higher mutation load than non-users. Tumor biopsy samples were acquired before radiation or chemotherapy; hence the mutation spectrum we report represents alterations in tumors in their natural state.

We identified mutations in genes like *TP53*, *NOTCH1*, *CDKN2A*, *FAT1*, *PIK3CA*, and *HRAS* consistent with the previous oral cancer studies (43). Somatic mutations in *NOTCH1* are generally reported in 11–15% of the HNSCC patients (44, 45). However, we observed them in 36% of cases in this study which is in concordance with previously reported higher rate of *NOTCH1* mutations from Asian cohorts (12, 46). Mutations in genes *NOTCH1*, *NOTCH2*, *NOTCH3*, *FAT1*, *SOX2*, and *TP63* are associated with defective squamous differentiation (47). We observed SNVs in *NOTCH1* in all cohorts, *NOTCH3* in smoker and non-user cohort, and *FAT1* in chewer and non-user cohorts. We observed eight missense and five non-sense SNVs in *NOTCH1*. *NOTCH1* is predicted to have tumor suppressive roles in HNSCC where loss of function mutations in *NOTCH1* were found in domains important for its activation and its translocation to the cell membrane (44). In OSCC patients, mutations in *NOTCH1* were found to have significant correlation with worse overall survival (46). Also, genes regulated by the NOTCH pathway are significantly altered in carcinoma of the oral tongue (OTSCC) from the Asian cohort, and it is significantly associated with poor disease-free survival (9). Interestingly, we observed a deleterious positional recurrent variant p.Cys438Trp/Phe/Gly in *NOTCH1* gene in patients with history of tobacco consumption, and it is not reported earlier in TCGA-HNSC datasets or Indian cohort of OSCC patients (12). This variant is in the calcium binding EGF domain of the protein and is known to play a role in calcium-dependent binding of tetrameric complexes to ligand-expressing cells (48). *NOTCH1* is reported to have oncogenic properties in OSCC, and we speculate the role of this novel variant in the process of tumorigenesis. Further validation to assess the impact of such recurrent variant is vital in studying its role in OSCC. We observed *NOTCH1* gene with both missense and non-sense variants in ten samples. Genes affected by somatic SNVs from samples with *NOTCH1* missense variant were found to be involved in GPCR signaling. GPCRs have been shown to play significant roles in the progression of various cancers including OSCC (49). *FBXW7* targets various proteins for degradation including *NOTCH1*, c-*MYC*, and *Cyclin E*. We identified *FBXW7* mutated at hotspot variant in WD40 domain which blocks the degradation of active *NOTCH1* which eventually results in tumorigenesis (50). These observations confirm the role of the NOTCH signaling pathway in the pathogenesis of oral cancer. *FAT1* and *FAT4* have been reported to be frequently mutated in gingivo-buccal OSCCs in an Indian cohort and are associated with tumorigenesis in various cancers including HNSCC (12). Mutations in tumor suppressor gene *FAT1* were found to be significantly correlated with poor disease-free survival in HNSCC patients (51).

Human papillomavirus is a known risk factor of OSCC, and multiple studies have reported that HPV infection is predominantly observed in wild-type TP53 tumors (52). We observed *TP53* mutation in 43% of the samples. Hence, we further analyzed whole-exome sequencing datasets for reads from human papillomavirus. However, we did not find HPV infection in any of the 30 OSCC samples. Further, we observed *HRAS* mutation in four samples, which is also associated with HPV-ve tumors (47). We observed *HRAS* mutation in 13% of the samples, comparable to the 10% mutation frequency reported in the Indian cohort in contrast to 5% in the TCGA dataset and 8% in the Taiwanese population (53). Mutation in *HRAS* is significantly associated with recurrence-free survival in the Indian cohort (54). *TP53* and *HRAS* mutations alter different pathways which lead to distinctive immune response in HNSCC (55). Hotspot mutation in *PIK3CA* is frequently observed in HPV-induced oropharyngeal carcinoma; however, we observed missense variants in only two samples (6). These mutations are reported to increase kinase activity of *PIK3CA* which constitutively keeps AKT phosphorylated thereby inducing oncogenic transformation of cells (56). Hotspot mutation H1047R was found to be associated with positive response to PI3K pathway inhibitors in HNSCC (57).

Patients recruited in this study were categorized into three cohorts based on the self-reported tobacco consumption habit. We observed that differences in the percentage contribution of transition C:G>T:A and transversion C:G>A:T in three cohorts were statistically insignificant. Signature 15, which is associated with defective DNA mismatch repair, is predicted in 24 samples. Signature 29, characterized by C:G>A:T transversion reported in gingivo-buccal oral squamous cell carcinoma patients with a history of chewing tobacco consumption from the Indian cohort, is observed in all the cohorts. In concordance with previous studies, we do not observe distinct enrichment of tobacco-induced signatures (9, 58).

Downregulation of *DMBT1* is associated with carcinogenesis in OSCC. However, we observed copy number gain in six samples from the tobacco user cohort (59). Genes *CASP8* and *FAT1* are reported to co-occur in TCGA-Head and Neck Squamous Cell Carcinoma (60); however, we observe similar incident in only one out of 11 samples with these SNVs. In concordance with previous reports, we observed higher mutation frequency in *CASP8* gene in oral cancer patients (12). Mutations in *CASP8* are reported to be higher in oral cancer tissues than in leukoplakia tissues and can be used to profile progression of leukoplakia to oral cancer (33). Although loss of function mutations in *CASP8* are a rare event in epithelial cancers, inactivating mutations in *CASP8* have been shown to stimulate tumoral growth, cell migration, and reduced apoptosis in OSCC (61). Out of 30 samples, 20 samples had <50 genes affected by copy number alterations. A subset of oral cavity tumors in the TCGA-HNSCC study with fewer CNAs is broadly categorized as M-class cancer suggesting tumorigenesis driven by point variants instead of copy number alteration. We observed the typical three gene pattern of M-class tumors consisting of activating mutation in *HRAS*, inactivating mutation in *CASP8*, and wild-type *TP53* in one sample out of 20 (6). Besides, patients with fewer CNA affected genes are reported to have better overall survival (62). Further, we observed exclusive copy number gain of 23 genes from 11q22 in two out of 10 samples in the tobacco user cohort with a relatively higher number of CNA affected genes. These two samples belonged to patients from the chewer and smoker cohorts. Out of 23, 15 (65.2%) genes were observed to have overexpressed in TCGA Head and neck squamous cell carcinoma (TCGA-HNSCC) dataset. Among these 15 genes, eight (34.7%) are significantly overexpressed in HPV negative tumors as per UALCAN analysis.

## Conclusions

Here we have investigated the mutation spectrum associated with oral cancer in Indian patients based on their history of tobacco use. Higher contribution of transition C:G>T:A related signature is observed in all the cohorts, and in chewer cohort elevated contribution of C:G>A:T transversion signature was observed compared to smoker and non-user cohorts; however, the change was not statistically significant. Signature 6 and signature 15, which are associated with defective DNA mismatch repair, are predicted in 80% of OSCC samples. Signature 29 associated with gingivo-buccal oral squamous cell carcinoma was enriched in 40% of tumors with a history of tobacco consumption. *NOTCH1* and *HRAS* mutations were more prevalent in the Indian cohort compared to the TCGA-HNSCC and Taiwanese cohorts. *NOTCH1* and *HRAS* mutations were more prevalent in the Indian cohort compared to the TCGA-HNSCC and Taiwanese cohorts. Both missense and non-sense mutations were identified in *NOTCH1*. *EGFR* is amplified in 25% of OSCC samples with a history of tobacco consumption. We report here that 70% of OSCC tumors from Indian patients were M-class (classification proposed based on TCGA studies) cancer suggesting tumorigenesis driven by point variants instead of copy number alterations. This is a pilot study to identify genomic alterations associated with tumors from OSCC patients who smoke tobacco, chew tobacco, and those with no habit of using any tobacco product. One of the major limitations of the study is the small sample size and heterogeneous nature of the samples. Due to the small sample size, we did not observe a significant association of variant types between the three cohorts. A reliable comparison would require data from a larger cohort. Further validation of these genomic anomalies in a larger cohort that is well stratified with due consideration for covariates is warranted. This study provides novel insights about molecular alterations at the gene level which can be further explored for effective treatment modalities of oral cancer.

## Data Availability Statement

The original contributions presented in the study are publicly available. This data can be found here: NCBI repository, accession number: PRJNA700466.

## Ethics Statement

The studies involving human participants were reviewed and approved by the Institutional Human Ethics Committee, Burdwan Dental College and Hospital, Burdwan, Kolkata-700014. The patients/participants provided their written informed consent to participate in this study.

## Author Contributions

KP: Formal analysis, investigation, software, writing original draft, visualization, and data curation. FB: Formal analysis, investigation, writing original draft, and data curation. SP: Writing—review and editing. SR: Writing—review and editing. NM: Writing—review and editing. BN: Writing—review and editing. DS: Writing—review and editing. MG: Methodology. JR: Methodology and resources. HG: Conceptualization, methodology, resources, supervision, project administration, and funding acquisition. AC: Conceptualization, methodology, resources, writing original draft, supervision, project administration, and funding acquisition. All authors contributed to the article and approved the submitted version.

## Funding

We thank the Department of Biotechnology (DBT), Government of India, for research support to the Institute of Bioinformatics (IOB), Bangalore. IOB was supported by DBT Program Support on Neuroproteomics and infrastructure for proteomic data analysis (BT/01/COE/08/05).

## Conflict of Interest

The authors declare that the research was conducted in the absence of any commercial or financial relationships that could be construed as a potential conflict of interest.

## Supplementary Material

Supplementary Figure 1 Distribution of different type of variants identified in OSCC whole-exome sequencing data.

Supplementary Figure 2 Distribution of transitions and transversion in OSCC samples.

Supplementary Figure 3 Mutation signatures deduced by linear decomposition method using Mutalisk in each OSCC sample.

Supplementary Figure 4 Schematic representation of mutual exclusivity of mutant TP53 and HRAS in **(A)** Current study, **(B)** Published TCGA-OSCC studies. Blue circles indicate the mutation in the respective tumor sample, and the gray circle indicates the WT allele. The column represents examined samples, and the row represents genes.

Supplementary Figure 5 The landscape of genomic amplifications and deletions in each oral squamous cell carcinoma sample investigated in the study. Green one-point-outlier, Dark Gray surroundings: frequent one-point-outlier, Brown >1 level gain, Brown surroundings: 1 level gain, Blue: >1 level loss, Blue surroundings: 1 level loss.

Supplementary Figure 6 Gene ontology enrichment analysis of CNA affected genes on cytoband 11q22 using FunRich keeping default human as a background database.

## Abbreviations

OSCC, Oral Squamous Cell Carcinoma; TCGA, The cancer genome atlas; HNSCC, head and neck squamous cell carcinoma; SNV, Single nucleotide variants; CNA, Copy number alterations; COSMIC, Catalogue Of Somatic Mutations In Cancer; BAM, binary alignment map; BWA, Burrows-Wheeler Aligner; MEM, maximal exact matches; WES, whole-exome sequence.
