# Supplementary material for: Whole-Exome Sequencing Analysis of Oral Squamous Cell Carcinoma Delineated by Tobacco Usage Habits
Source: Front Oncol. 2021 May 31;11:660696. doi: 10.3389/fonc.2021.660696 (PMC8200776; doi:10.3389/fonc.2021.660696)
Supplement: Supplementary file 1 [file Table_1.pdf]

Patel *et al.* , 2021. Whole-exome sequencing analysis of oral squamous cell carcinoma delineated by tobacco usage habits

Supplementary Table 1. Clinical characteristics of patients with OSCC that were subjected to whole exome sequencing

Sr. No.	Sample ID	Tobacco-habit	Age	Sex	Tumor location	TNM Staging	Duaration of tobacco usage
1	IOB_03	Chewer	44	M	Tongue	T2N1MX	Tobacco chewing for 19 yrs
2	IOB_04	Chewer	60	M	Tongue	T3N0MX	Tobacco chewing for 30 yrs
3	IOB_06	Chewer	56	F	Alveolus	T4aN1MX	Tobacco chewing for 46 yrs
4	IOB_07	Chewer	35	F	Lip	T4aN0MX	Tobacco chewing for 20 yrs
5	IOB_08	Chewer	35	F	Cheek	T4aN1MX	Tobacco chewing for 10 yrs
6	IOB_09	Chewer	47	F	Buccal mucosa	T2N1M0	Tobacco chewing for 37 yrs
7	IOB_10	Chewer	66	M	Buccal mucosa	T4aN1MX	Tobacco chewing for 46 yrs
8	IOB_31	Chewer	70	F	Buccal mucosa	T2N1M0	Tobacco chewing for 52 yrs
9	IOB_32	Chewer	45	F	Buccal mucosa	T2N1M0	Not reported
10	IOB_33	Chewer	52	M	Buccal mucosa	T3N2aMX	Tobacco chewing for 5 yrs
11	IOB_11	Smoker	55	M	Alveolus	T4aN0MX	Smoking for 35 yrs
12	IOB_12	Smoker	34	M	Buccal mucosa	T4aN1MX	Smoking for 16 yrs
13	IOB_13	Smoker	50	M	Buccal mucosa	T4aN1MX	Smoking for 30 yrs
14	IOB_14	Smoker	55	M	Buccal mucosa	T4aN1MX	Smoking for 37 yrs
15	IOB_15	Smoker	61	M	Lip	NA	Smoking for 31 yrs
16	IOB_16	Smoker	50	M	Lip	T4aN2bM1	Not reported
17	IOB_17	Smoker	44	M	Maxilla	T4aN2bM0	Not reported
18	IOB_18	Smoker	53	M	Tongue	T2N1MX	Smoking for 35 yrs
19	IOB_19	Smoker	48	M	Alveolus	T4aN3MX	Smoking for 13 yrs
20	IOB_35	Smoker	60	M	Tongue	T2N0M0	Not reported
21	IOB_21	Non-user	60	M	Lingual mucosa	T2N2MX	Not applicable
22	IOB_22	Non-user	37	F	Hard palate	T2N1M1	Not applicable
23	IOB_23	Non-user	52	F	Tongue	T2N1M0	Not applicable
24	IOB_24	Non-user	75	M	Buccal mucosa	T4aN2cM0	Not applicable
25	IOB_25	Non-user	60	M	Buccal mucosa	NA	Not applicable
26	IOB_26	Non-user	62	F	Tongue	NA	Not applicable
27	IOB_27	Non-user	42	F	Soft palate	NA	Not applicable
28	IOB_28	Non-user	56	F	Tongue	T3N0MX	Not applicable
29	IOB_29	Non-user	31	M	Tongue	T4aN1M0	Not applicable
30	IOB_30	Non-user	65	F	Buccal mucosa	T2N1MX	Not applicable
NA=Not available							
