# Supplementary material for: Whole-Exome Sequencing Analysis of Oral Squamous Cell Carcinoma Delineated by Tobacco Usage Habits
Source: Front Oncol. 2021 May 31;11:660696. doi: 10.3389/fonc.2021.660696 (PMC8200776; doi:10.3389/fonc.2021.660696)
Supplement: Supplementary file 2 [file Table_2.pdf]

Patel *et al.* , 2021. Whole-exome sequencing analysis of oral squamous cell carcinoma delineated by tobacco usage habits

Supplementary Table 2. Read statistics and QC details of whole-exome sequencing for all samples

Cohort	Sample ID	Sample type	Total reads	Average base quality (Phred)	Alignment (%)	Crossmapped (%)	Unaligned (%)	Panel length	Panel coverage %	Panel avg depth	Read ontarget %
Chewer	IOB_03	Normal	6,63,13,700	38.343	99.996	0.136	0.006	5,03,90,601	99.329	117.134	87.817
Chewer	IOB_03	Tumor	7,37,55,524	38.217	99.996	0.218	0.007	5,03,90,601	99.320	130.300	89.166
Chewer	IOB_04	Normal	10,78,37,250	38.404	99.997	0.188	0.005	5,03,90,601	99.354	187.812	87.756
Chewer	IOB_04	Tumor	7,54,51,740	38.481	99.995	0.167	0.007	5,03,90,601	99.334	129.273	88.187
Chewer	IOB_06	Normal	5,82,89,404	38.725	99.993	0.138	0.009	5,03,90,601	99.216	101.251	86.826
Chewer	IOB_06	Tumor	6,85,84,028	38.945	99.996	0.197	0.005	5,03,90,601	99.198	124.905	89.376
Chewer	IOB_07	Normal	6,99,35,194	38.354	99.996	0.15	0.006	5,03,90,601	99.216	122.789	87.544
Chewer	IOB_07	Tumor	7,27,20,960	38.139	99.997	0.134	0.005	5,03,90,601	99.216	126.322	88.130
Chewer	IOB_08	Normal	8,06,73,194	38.362	99.997	0.158	0.005	5,03,90,601	99.224	141.324	87.619
Chewer	IOB_08	Tumor	7,75,47,422	38.292	99.998	0.343	0.005	5,03,90,601	99.201	137.023	88.310
Chewer	IOB_09	Normal	5,85,06,518	38.346	99.997	0.158	0.005	5,03,90,601	99.225	101.581	87.162
Chewer	IOB_09	Tumor	6,21,26,620	38.058	99.996	0.183	0.006	5,03,90,601	99.215	107.892	87.256
Chewer	IOB_10	Normal	6,80,86,306	38.339	99.997	0.176	0.006	5,03,90,601	99.330	119.446	87.147
Chewer	IOB_10	Tumor	7,60,05,448	38.126	99.996	0.169	0.006	5,03,90,601	99.332	131.521	88.020
Chewer	IOB_31	Normal	7,35,73,540	38.135	99.992	0.251	0.01	5,03,90,601	99.211	131.610	88.320
Chewer	IOB_31	Tumor	8,01,97,088	38.149	99.994	0.38	0.009	5,03,90,601	99.233	143.192	88.987
Chewer	IOB_32	Normal	7,67,31,076	38.172	99.993	0.162	0.009	5,03,90,601	99.237	127.871	82.943
Chewer	IOB_32	Tumor	8,69,54,266	38.174	99.993	0.564	0.012	5,03,90,601	99.240	151.265	87.121
Chewer	IOB_33	Normal	6,77,95,184	38.035	99.993	0.266	0.01	5,03,90,601	99.349	111.042	84.785
Chewer	IOB_33	Tumor	10,30,92,402	38.932	99.96	0.168	0.045	5,03,90,601	99.338	163.656	88.326
Smoker	IOB_11	Normal	7,00,31,568	38.3	99.997	0.157	0.006	5,03,90,601	99.332	120.726	86.744
Smoker	IOB_11	Tumor	7,92,05,932	38.716	99.994	0.15	0.008	5,03,90,601	99.329	139.941	87.822
Smoker	IOB_12	Normal	7,64,57,346	38.448	99.997	0.164	0.005	5,03,90,601	99.319	134.414	88.826
Smoker	IOB_12	Tumor	7,94,92,034	38.308	99.997	0.503	0.005	5,03,90,601	99.309	141.694	88.357
Smoker	IOB_13	Normal	7,37,74,982	38.412	99.997	0.132	0.005	5,03,90,601	99.324	131.266	88.494
Smoker	IOB_13	Tumor	9,29,42,022	38.845	99.997	0.179	0.004	5,03,90,601	99.320	165.618	88.875
Smoker	IOB_14	Normal	6,59,37,612	38.357	99.997	0.163	0.005	5,03,90,601	99.316	117.471	88.231
Smoker	IOB_14	Tumor	8,32,63,242	38.695	99.997	0.198	0.004	5,03,90,601	99.340	149.749	88.086
Smoker	IOB_15	Normal	8,55,31,382	38.657	99.98	0.194	0.026	5,03,90,601	99.341	151.490	88.156
Smoker	IOB_15	Tumor	7,99,16,632	38.772	99.998	0.257	0.004	5,03,90,601	99.333	142.965	88.282
Smoker	IOB_16	Normal	7,58,64,692	38.673	99.974	0.2	0.033	5,03,90,601	99.326	133.343	88.083
Smoker	IOB_16	Tumor	8,39,06,486	38.205	99.997	0.339	0.006	5,03,90,601	99.327	147.472	87.705
Smoker	IOB_17	Normal	7,48,61,494	38.631	99.978	0.166	0.029	5,03,90,601	99.324	131.009	87.228
Smoker	IOB_17	Tumor	7,84,83,462	38.652	99.997	0.197	0.005	5,03,90,601	99.335	138.318	88.291
Smoker	IOB_18	Normal	7,76,83,518	38.125	99.997	0.2	0.005	5,03,90,601	99.323	132.921	87.769
Smoker	IOB_18	Tumor	7,50,44,138	38.64	99.997	0.148	0.005	5,03,90,601	99.318	132.806	87.628
Smoker	IOB_19	Normal	6,52,80,578	38.112	99.997	0.157	0.005	5,03,90,601	99.312	113.750	87.912
Smoker	IOB_19	Tumor	8,53,30,152	38.756	99.997	0.326	0.004	5,03,90,601	99.305	151.468	88.561
Smoker	IOB_35	Normal	7,28,94,424	38.13	99.993	0.245	0.009	5,03,90,601	99.314	127.820	87.094
Smoker	IOB_35	Tumor	7,19,76,050	38.052	99.996	0.502	0.007	5,03,90,601	99.318	122.382	86.790

Patel *et al.* , 2021. Whole-exome sequencing analysis of oral squamous cell carcinoma delineated by tobacco usage habits

Supplementary Table 2. Read statistics and QC details of whole-exome sequencing for all samples

Cohort	Sample ID	Sample type	Total reads	Average base quality (Phred)	Alignment (%)	Crossmapped (%)	Unaligned (%)	Panel length	Panel coverage %	Panel avg depth	Read ontarget %
Non-user	IOB_21	Normal	7,48,21,954	38.263	99.993	0.208	0.009	5,03,90,601	99.327	133.157	88.112
Non-user	IOB_21	Tumor	8,36,23,494	38.223	99.915	0.749	0.104	5,03,90,601	99.324	148.232	88.138
Non-user	IOB_22	Normal	13,06,10,462	38.209	99.994	0.145	0.008	5,03,90,601	99.273	228.315	87.395
Non-user	IOB_22	Tumor	7,43,54,950	38.238	99.994	0.535	0.009	5,03,90,601	99.202	135.943	88.173
Non-user	IOB_23	Normal	7,49,15,178	38.29	99.994	0.3	0.009	5,03,90,601	99.217	133.564	88.212
Non-user	IOB_23	Tumor	5,96,66,354	38.06	99.993	0.167	0.009	5,03,90,601	99.215	105.366	88.147
Non-user	IOB_24	Normal	8,35,78,952	38.174	99.993	0.14	0.009	5,03,90,601	99.343	147.654	87.750
Non-user	IOB_24	Tumor	6,70,21,456	38.23	99.994	0.166	0.008	5,03,90,601	99.305	120.652	89.716
Non-user	IOB_25	Normal	7,50,58,946	38.191	99.993	0.193	0.009	5,03,90,601	99.348	133.143	87.685
Non-user	IOB_25	Tumor	7,70,02,254	38.171	99.994	0.161	0.008	5,03,90,601	99.331	139.041	89.320
Non-user	IOB_26	Normal	8,49,00,946	38.155	99.995	0.119	0.007	5,03,90,601	99.219	150.887	88.423
Non-user	IOB_26	Tumor	7,90,47,204	38.164	99.994	0.174	0.008	5,03,90,601	99.229	141.008	88.592
Non-user	IOB_27	Normal	7,85,85,608	38.229	99.995	0.145	0.007	5,03,90,601	99.214	141.961	88.981
Non-user	IOB_27	Tumor	8,00,70,550	38.139	99.994	0.138	0.008	5,03,90,601	99.222	140.876	88.280
Non-user	IOB_28	Normal	7,31,74,782	38.139	99.994	0.178	0.008	5,03,90,601	99.226	130.596	87.858
Non-user	IOB_28	Tumor	7,70,49,368	38.108	99.99	0.24	0.013	5,03,90,601	99.244	132.781	86.253
Non-user	IOB_29	Normal	8,28,73,806	38.138	99.994	0.158	0.008	5,03,90,601	99.348	150.088	88.853
Non-user	IOB_29	Tumor	7,08,18,104	38.126	99.99	0.62	0.013	5,03,90,601	99.341	121.287	85.898
Non-user	IOB_30	Normal	7,88,38,212	38.149	99.993	0.188	0.009	5,03,90,601	99.235	135.849	87.230
Non-user	IOB_30	Tumor	7,39,02,432	38.135	99.993	0.267	0.009	5,03,90,601	99.222	131.363	88.321
