# Supplementary material for: Whole-Exome Sequencing Analysis of Oral Squamous Cell Carcinoma Delineated by Tobacco Usage Habits
Source: Front Oncol. 2021 May 31;11:660696. doi: 10.3389/fonc.2021.660696 (PMC8200776; doi:10.3389/fonc.2021.660696)
Supplement: Supplementary file 3 [file Table_3.pdf]

Patel *et al.* , 2021. Whole-exome sequencing analysis of oral squamous cell carcinoma delineated by tobacco usage habits

Supplementary Table 3. List of different types of genomic alterations identified in OSCC tumor samples

Sr. No.	Cohort	Sample ID	Total mutations	Non-synonymous	Coding mutations						Non-coding	Mutation load	Genes affected by copy number alterations
					Missense	Nonsense	Nonstop	Startloss	Splice	Silent			
1	Chewer	IOB_03	199	88	85	3	0	0	0	37	74	3.95	58
2	Chewer	IOB_04	171	69	65	3	0	0	1	28	74	3.39	54
3	Chewer	IOB_06	340	173	165	6	1	0	1	54	113	6.75	8
4	Chewer	IOB_07	585	222	203	16	0	1	2	87	276	11.61	15
5	Chewer	IOB_08	169	87	79	5	0	0	3	27	55	3.35	22
6	Chewer	IOB_09	226	88	78	8	0	0	2	37	101	4.48	42
7	Chewer	IOB_10	395	166	145	14	0	1	6	54	175	7.84	606
8	Chewer	IOB_31	328	151	132	18	0	0	1	48	129	6.51	59
9	Chewer	IOB_32	256	105	95	8	0	0	2	38	113	5.08	79
10	Chewer	IOB_33	302	136	123	11	0	1	1	37	129	5.99	672
11	Smoker	IOB_11	329	148	132	12	0	0	4	53	128	6.53	19
12	Smoker	IOB_12	207	86	76	6	0	0	4	33	88	4.11	26
13	Smoker	IOB_13	321	147	131	14	0	0	2	43	131	6.37	50
14	Smoker	IOB_14	317	146	135	8	0	1	2	46	125	6.29	33
15	Smoker	IOB_15	1500	569	527	25	0	1	16	278	653	29.77	31
16	Smoker	IOB_16	4864	1369	1317	31	0	5	16	899	2596	96.53	31
17	Smoker	IOB_17	193	85	73	9	0	0	3	17	91	3.83	33
18	Smoker	IOB_18	296	140	127	8	0	0	5	39	117	5.87	96
19	Smoker	IOB_19	279	124	119	4	1	0	0	36	119	5.54	450
20	Smoker	IOB_35	174	67	66	0	0	0	1	26	81	3.45	23
21	Non-User	IOB_21	320	125	115	6	0	0	4	62	133	6.35	116
22	Non-User	IOB_22	94	42	34	6	0	0	2	12	40	1.87	26
23	Non-User	IOB_23	67	30	26	3	0	0	1	3	34	1.33	19
24	Non-User	IOB_24	4253	1201	1162	22	0	0	17	881	2171	84.4	22
25	Non-User	IOB_25	235	103	98	5	0	0	0	29	103	4.66	48
26	Non-User	IOB_26	149	63	59	2	0	0	2	25	61	2.96	7
27	Non-User	IOB_27	193	81	71	7	0	0	3	18	94	3.83	10
28	Non-User	IOB_28	253	107	94	11	0	0	2	36	110	5.02	34
29	Non-User	IOB_29	50	30	26	3	0	0	1	5	15	0.99	14
30	Non-User	IOB_30	540	231	218	8	0	0	5	82	227	10.72	33
