# Supplementary material for: Whole-Exome Sequencing Analysis of Oral Squamous Cell Carcinoma Delineated by Tobacco Usage Habits
Source: Front Oncol. 2021 May 31;11:660696. doi: 10.3389/fonc.2021.660696 (PMC8200776; doi:10.3389/fonc.2021.660696)
Supplement: Supplementary file 5 [file Table_5.pdf]

Patel *et al.* , 2021. Whole-exome sequencing analysis of oral squamous cell carcinoma delineated by tobacco usage habits

Supplementary Table 5. List of genes affected by copy number alteration events in OSCC patients

Gene	Chromosome	Cytoband	Recurrence	Recurrence in smoker cohort	Recurrence in chewer cohort	Recurrence in No habit cohort	State	Samples with CNA gain	Samples with CNA loss
CFHR3	chr1	1q31.3	17	5	5	7	Amplification/ Deletion	15	2
CFHR1	chr1	1q31.3	16	4	5	7	Amplification/ Deletion	14	2
OR4K2	chr14	14q11.2	8	2	4	2	Amplification	8	0
OR4K5	chr14	14q11.2	8	2	4	2	Amplification	8	0
OR4M1	chr14	14q11.2	8	2	4	2	Amplification	8	0
OR4N2	chr14	14q11.2	8	2	4	2	Amplification	8	0
OR4Q3	chr14	14q11.2	8	2	4	2	Amplification	8	0
OR2A1	chr7	7q35	8	5	1	2	Amplification	8	0
OR2A20P	chr7	7q35	8	5	1	2	Amplification	8	0
OR2A7	chr7	7q35	8	5	1	2	Amplification	8	0
OR2A9P	chr7	7q35	8	5	1	2	Amplification	8	0
GSTT1	chr22	22q11.23	13	4	4	5	Amplification/ Deletion	10	3
LGALS9C	chr17	17p11.2	11	3	4	4	Amplification/ Deletion	9	2
APOBEC3B	chr22	22q13.1	7	1	2	4	Amplification	7	0
ARHGEF35	chr7	7q35	7	4	1	2	Amplification	7	0
ARHGEF5	chr7	7q35	7	5	0	2	Amplification	7	0
DMBT1	chr10	10q26.13	6	3	3	0	Amplification	6	0
ARL17A	chr17	17q21.31	6	3	1	2	Amplification	6	0
CCL3L3	chr17	17q12	6	1	4	1	Amplification	6	0
NSF	chr17	17q21.31	6	3	1	2	Amplification	6	0
LILRA3	chr19	19q13.4	6	1	1	4	Amplification	6	0
CCL4L1	chr17	17q12	7	2	4	1	Amplification/ Deletion	6	1
LRRC37A	chr17	17q21.31	5	2	1	2	Amplification	5	0
DDT	chr22	22q11.23	5	3	1	1	Amplification	5	0
DDTL	chr22	22q11.23	5	3	1	1	Amplification	5	0
GSTT2	chr22	22q11.23	5	3	1	1	Amplification	5	0
GSTT2B	chr22	22q11.23	5	3	1	1	Amplification	5	0
EGFR	chr7	7p11.2	5	3	2	0	Amplification	5	0
OR2A42	chr7	7q35	5	3	1	1	Amplification	5	0
SEC61G	chr7	7p11.2	5	3	2	0	Amplification	5	0
VSTM2A	chr7	7p11.2	5	3	2	0	Amplification	5	0
LRRC37A2	chr17	17q21.31	4	2	0	2	Amplification	4	0
HLA	chr6	0	4	2	0	2	Amplification	4	0
ADAM3A	chr8	8p11.22	15	5	6	4	Amplification/ Deletion	9	6
PDPR	chr16	16q22.1	9	2	5	2	Amplification/ Deletion	6	3
TAS2R43	chr12	12p13.2	7	3	2	2	Amplification/ Deletion	5	2
SULT1A1	chr16	16p11.2	5	2	2	1	Amplification/ Deletion	4	1
CCL3L1	chr17	17q21.1	5	2	2	1	Amplification/ Deletion	4	1
CROCCP2	chr1	1p36.13	3	1	0	2	Amplification	3	0
SKA3	chr13	13q12.11	3	1	1	1	Amplification	3	0
PDXDC1	chr16	16p13.11	3	1	0	2	Amplification	3	0
CCL4L2	chr17	17q12	3	1	1	1	Amplification	3	0
FCGBP	chr19	19q13.2	3	1	2	0	Amplification	3	0
PCDHB8	chr5	5q31.3	3	2	0	1	Amplification	3	0
ZDHHC11B	chr5	5p15.33	3	0	2	1	Amplification	3	0
FKBP9L	chr7	7p11.2	3	2	1	0	Amplification	3	0
LANCL2	chr7	7p11.2	3	2	1	0	Amplification	3	0
VOPP1	chr7	7p11.2	3	2	1	0	Amplification	3	0

Patel *et al.* , 2021. Whole-exome sequencing analysis of oral squamous cell carcinoma delineated by tobacco usage habits

Supplementary Table 5. List of genes affected by copy number alteration events in OSCC patients

Gene	Chromosome	Cytoband	Recurrence	Recurrence in smoker cohort	Recurrence in chewer cohort	Recurrence in No habit cohort	State	Samples with CNA gain	Samples with CNA loss
FCGR2C	chr1	1q23.3	6	2	3	1	Amplification/ Deletion	4	2
FCGR3A	chr1	1q23.3	6	2	3	1	Amplification/ Deletion	4	2
OR4M2	chr15	15q11.2	6	3	1	2	Amplification/ Deletion	4	2
OR4N4	chr15	15q11.2	6	3	1	2	Amplification/ Deletion	4	2
HNRNPCL1	chr1	1p36.21	4	2	2	0	Amplification/ Deletion	3	1
PRAMEF2	chr1	1p36.21	4	2	2	0	Amplification/ Deletion	3	1
TBC1D3H	chr17	17q12	4	2	1	1	Amplification/ Deletion	3	1
CROCC	chr1	1p36.13	2	1	1	0	Amplification	2	0
ANGPTL5	chr11	11q22.1	2	1	1	0	Amplification	2	0
AP000619	chr11	0	2	1	1	0	Amplification	2	0
BIRC2	chr11	11q22.2	2	1	1	0	Amplification	2	0
BIRC3	chr11	11q22.2	2	1	1	0	Amplification	2	0
C11orf70	chr11	11q22.1	2	1	1	0	Amplification	2	0
KIAA1377	chr11	11q22.1	2	1	1	0	Amplification	2	0
MMP1	chr11	11q22.2	2	1	1	0	Amplification	2	0
MMP10	chr11	11q22.2	2	1	1	0	Amplification	2	0
MMP20	chr11	16p13.3	2	1	1	0	Amplification	2	0
MMP27	chr11	11q22.2	2	1	1	0	Amplification	2	0
MMP3	chr11	11q22.2	2	1	1	0	Amplification	2	0
MMP7	chr11	11q22.2	2	1	1	0	Amplification	2	0
MMP8	chr11	11q22.2	2	1	1	0	Amplification	2	0
TMEM123	chr11	11q22.2	2	1	1	0	Amplification	2	0
TRPC6	chr11	11q22.1	2	1	1	0	Amplification	2	0
WTAPP1	chr11	11q22.2	2	1	1	0	Amplification	2	0
YAP1	chr11	11q22.1	2	1	1	0	Amplification	2	0
KLRC2	chr12	12p13.2	2	0	2	0	Amplification	2	0
AL928742	chr14	0	2	1	0	1	Amplification	2	0
CLEC18C	chr16	16q22.1	2	0	2	0	Amplification	2	0
CCDC144A	chr17	17p11.2	2	1	0	1	Amplification	2	0
KRT16P2	chr17	17p11.2	2	1	0	1	Amplification	2	0
ADCYAP1	chr18	18p11.32	2	0	2	0	Amplification	2	0
ARHGAP28	chr18	18p11.31	2	0	2	0	Amplification	2	0
C18orf42	chr18	18p11.31	2	0	2	0	Amplification	2	0
C18orf56	chr18	18p11.32	2	0	2	0	Amplification	2	0
CETN1	chr18	18p11.32	2	0	2	0	Amplification	2	0
CLUL1	chr18	18p11.32	2	0	2	0	Amplification	2	0
DLGAP1	chr18	18p11.31	2	0	2	0	Amplification	2	0
EMILIN2	chr18	18p11.32-p11.31	2	0	2	0	Amplification	2	0
ENOSF1	chr18	18p11.32	2	0	2	0	Amplification	2	0
EPB41L3	chr18	18p11.31	2	0	2	0	Amplification	2	0
L3MBTL4	chr18	18p11.31	2	0	2	0	Amplification	2	0
LAMA1	chr18	18p11.31	2	0	2	0	Amplification	2	0
LINC00470	chr18	18p11.32	2	0	2	0	Amplification	2	0
LINC00667	chr18	18p11.31	2	0	2	0	Amplification	2	0
LINC00668	chr18	18p11.31	2	0	2	0	Amplification	2	0
LPIN2	chr18	18p11.31	2	0	2	0	Amplification	2	0
METTL4	chr18	18p11.32	2	0	2	0	Amplification	2	0
MYL12A	chr18	18p11.31	2	0	2	0	Amplification	2	0
MYL12B	chr18	18p11.31	2	0	2	0	Amplification	2	0
MYOM1	chr18	18p11.31	2	0	2	0	Amplification	2	0
NDC80	chr18	18p11.32	2	0	2	0	Amplification	2	0

Patel *et al.*, 2021. Whole-exome sequencing analysis of oral squamous cell carcinoma delineated by tobacco usage habits

Supplementary Table 5. List of genes affected by copy number alteration events in OSCC patients

Gene	Chromosome	Cytoband	Recurrence	Recurrence in smoker cohort	Recurrence in chewer cohort	Recurrence in No habit cohort	State	Samples with CNA gain	Samples with CNA loss
SMCHD1	chr18	18p11.32	2	0	2	0	Amplification	2	0
TGIF1	chr18	18p11.31	2	0	2	0	Amplification	2	0
TMEM200C	chr18	18p11.31	2	0	2	0	Amplification	2	0
TYMS	chr18	18p11.32	2	0	2	0	Amplification	2	0
YES1	chr18	18p11.32	2	0	2	0	Amplification	2	0
ZBTB14	chr18	18p11.31	2	0	2	0	Amplification	2	0
KIR3DL1	chr19	19q13.42	2	2	0	0	Amplification	2	0
RRP7A	chr22	22q13.2	2	2	0	0	Amplification	2	0
SERHL	chr22	22q13.2	2	2	0	0	Amplification	2	0
ACTRT3	chr3	3q26.2	2	0	2	0	Amplification	2	0
FBLN2	chr3	3p25.1	2	1	1	0	Amplification	2	0
LINC00969	chr3	3q29	2	0	1	1	Amplification	2	0
LRRC31	chr3	3q26.2	2	0	2	0	Amplification	2	0
LRRC34	chr3	3q26.2	2	0	2	0	Amplification	2	0
LRRIQ4	chr3	3q26.2	2	0	2	0	Amplification	2	0
MUC20	chr3	3q29	2	0	1	1	Amplification	2	0
MYNN	chr3	3q26.2	2	0	2	0	Amplification	2	0
SAMD7	chr3	3q26.2	2	0	2	0	Amplification	2	0
TERC	chr3	3q26.2	2	0	2	0	Amplification	2	0
ZDHHC11	chr5	5p15.33	2	0	1	1	Amplification	2	0
AL035588	chr6	0	2	2	0	0	Amplification	2	0
BYSL	chr6	6p21.1	2	2	0	0	Amplification	2	0
C6orf132	chr6	6p21.1	2	2	0	0	Amplification	2	0
CCND3	chr6	6p21.1	2	2	0	0	Amplification	2	0
FOXP4	chr6	6p21.1	2	2	0	0	Amplification	2	0
FRS3	chr6	6p21.1	2	2	0	0	Amplification	2	0
GUCA1A	chr6	6p21.1	2	2	0	0	Amplification	2	0
GUCA1B	chr6	6p21.1	2	2	0	0	Amplification	2	0
MDFI	chr6	6p21.1	2	2	0	0	Amplification	2	0
MED20	chr6	6p21.1	2	2	0	0	Amplification	2	0
MRPS10	chr6	6p21.1	2	2	0	0	Amplification	2	0
NCR2	chr6	6p21.1	2	2	0	0	Amplification	2	0
PGC	chr6	6p21.1	2	2	0	0	Amplification	2	0
PRICKLE4	chr6	6p21.1	2	2	0	0	Amplification	2	0
TAF8	chr6	6p21.1	2	2	0	0	Amplification	2	0
TFEB	chr6	6p21.1	2	2	0	0	Amplification	2	0
TOMM6	chr6	6p21.1	2	2	0	0	Amplification	2	0
TREM1	chr6	6p21.1	2	2	0	0	Amplification	2	0
TREM2	chr6	6p21.1	2	2	0	0	Amplification	2	0
TREML1	chr6	6p21.1	2	2	0	0	Amplification	2	0
TREML2	chr6	6p21.1	2	2	0	0	Amplification	2	0
TREML4	chr6	6p21.1	2	2	0	0	Amplification	2	0
TRERF1	chr6	6p21.1	2	2	0	0	Amplification	2	0
USP49	chr6	6p21.1	2	2	0	0	Amplification	2	0
Sep-14	chr7	7p11.2	2	1	1	0	Amplification	2	0
AC093668	chr7	0	2	1	1	0	Amplification	2	0
CCT6A	chr7	7p11.2	2	1	1	0	Amplification	2	0
CHCHD2	chr7	7p11.2	2	1	1	0	Amplification	2	0
GBAS	chr7	7p11.2	2	1	1	0	Amplification	2	0
GIGYF1	chr7	7q22.1	2	2	0	0	Amplification	2	0
GNB2	chr7	7q22.1	2	2	0	0	Amplification	2	0
MRPS17	chr7	7p11.2	2	1	1	0	Amplification	2	0
NUPR1L	chr7	7p11.2	2	1	1	0	Amplification	2	0
PHKG1	chr7	7p11.2	2	1	1	0	Amplification	2	0
PSPH	chr7	7p11.2	2	1	1	0	Amplification	2	0
SUMF2	chr7	7p11.2	2	1	1	0	Amplification	2	0
ZNF479	chr7	7p11.2	2	1	1	0	Amplification	2	0
ZNF713	chr7	7p11.2	2	1	1	0	Amplification	2	0
ZNF716	chr7	7p11.2	2	1	1	0	Amplification	2	0

Patel *et al.* , 2021. Whole-exome sequencing analysis of oral squamous cell carcinoma delineated by tobacco usage habits

Supplementary Table 5. List of genes affected by copy number alteration events in OSCC patients

Gene	Chromosome	Cytoband	Recurrence	Recurrence in smoker cohort	Recurrence in chewer cohort	Recurrence in No habit cohort	State	Samples with CNA gain	Samples with CNA loss
AC022182	chr8	0	2	0	2	0	Amplification	2	0
AC084082	chr8	0	2	0	2	0	Amplification	2	0
ADHFE1	chr8	8q13.1	2	0	2	0	Amplification	2	0
ARFGEF1	chr8	8q13.2	2	0	2	0	Amplification	2	0
ARMC1	chr8	8q13.1	2	0	2	0	Amplification	2	0
ASPH	chr8	8q12.3	2	0	2	0	Amplification	2	0
ATP6V0D2	chr8	8q21.3	2	0	2	0	Amplification	2	0
BHLHE22	chr8	8q12.3	2	0	2	0	Amplification	2	0
C8orf44	chr8	8q13.1	2	0	2	0	Amplification	2	0
C8orf46	chr8	8q13.1	2	0	2	0	Amplification	2	0
C8orf59	chr8	8q21.2	2	0	2	0	Amplification	2	0
CA1	chr8	8q21.2	2	0	2	0	Amplification	2	0
CA13	chr8	8q21.2	2	0	2	0	Amplification	2	0
CA2	chr8	8q21.2	2	0	2	0	Amplification	2	0
CA3	chr8	8q21.2	2	0	2	0	Amplification	2	0
CA8	chr8	8q12.1	2	0	2	0	Amplification	2	0
CALB1	chr8	8q21.3	2	0	2	0	Amplification	2	0
CHCHD7	chr8	8q12.1	2	0	2	0	Amplification	2	0
CHD7	chr8	8q12.2	2	0	2	0	Amplification	2	0
CHMP4C	chr8	8q21.13	2	0	2	0	Amplification	2	0
CLVS1	chr8	8q12.2-q12.3	2	0	2	0	Amplification	2	0
CNBD1	chr8	8q21.3	2	0	2	0	Amplification	2	0
CNGB3	chr8	8q21.3	2	0	2	0	Amplification	2	0
COPS5	chr8	8q13.1	2	0	2	0	Amplification	2	0
CPA6	chr8	8q13.2	2	0	2	0	Amplification	2	0
CPNE3	chr8	8q21.3	2	0	2	0	Amplification	2	0
CRH	chr8	8q13.1	2	0	2	0	Amplification	2	0
CRISPLD1	chr8	8q21.13	2	0	2	0	Amplification	2	0
CSPP1	chr8	8q13.1-q13.2	2	0	2	0	Amplification	2	0
CYP7A1	chr8	8q12.1	2	0	2	0	Amplification	2	0
CYP7B1	chr8	8q12.3	2	0	2	0	Amplification	2	0
DCAF4L2	chr8	8q21.3	2	0	2	0	Amplification	2	0
DECR1	chr8	8q21.3	2	0	2	0	Amplification	2	0
DNAJC5B	chr8	8q13.1	2	0	2	0	Amplification	2	0
E2F5	chr8	8q21.2	2	0	2	0	Amplification	2	0
EYA1	chr8	8q13.3	2	0	2	0	Amplification	2	0
FABP12	chr8	8q21.13	2	0	2	0	Amplification	2	0
FABP4	chr8	8q21.13	2	0	2	0	Amplification	2	0
FABP5	chr8	13q22.1	2	0	2	0	Amplification	2	0
FABP9	chr8	8q21.13	2	0	2	0	Amplification	2	0
FAM110B	chr8	8q12.1	2	0	2	0	Amplification	2	0
GDAP1	chr8	8q21.11	2	0	2	0	Amplification	2	0
GGH	chr8	8q12.3	2	0	2	0	Amplification	2	0
HEY1	chr8	8q21.13	2	0	2	0	Amplification	2	0
HNF4G	chr8	8q21.13	2	0	2	0	Amplification	2	0
IL7	chr8	8q21.13	2	0	2	0	Amplification	2	0
IMPA1	chr8	8q21.13	2	0	2	0	Amplification	2	0
IMPAD1	chr8	8q12.1	2	0	2	0	Amplification	2	0
JPH1	chr8	8q21.11	2	0	2	0	Amplification	2	0
KCNB2	chr8	8q21.11	2	0	2	0	Amplification	2	0
LACTB2	chr8	8q13.3	2	0	2	0	Amplification	2	0
LINC00966	chr8	8q12.3	2	0	2	0	Amplification	2	0
LINC00967	chr8	8q13.1	2	0	2	0	Amplification	2	0
LRRCC1	chr8	8q21.2	2	0	2	0	Amplification	2	0
LY96	chr8	8q21.11	2	0	2	0	Amplification	2	0
LYN	chr8	8q12.1	2	0	2	0	Amplification	2	0
MCMDC2	chr8	8q13.1	2	0	2	0	Amplification	2	0

Patel *et al.* , 2021. Whole-exome sequencing analysis of oral squamous cell carcinoma delineated by tobacco usage habits

Supplementary Table 5. List of genes affected by copy number alteration events in OSCC patients

Gene	Chromosome	Cytoband	Recurrence	Recurrence in smoker cohort	Recurrence in chewer cohort	Recurrence in No habit cohort	State	Samples with CNA gain	Samples with CNA loss
MMP16	chr8	8q21.3	2	0	2	0	Amplification	2	0
MOS	chr8	18q12.2	2	0	2	0	Amplification	2	0
MRPS28	chr8	12p11.22	2	0	2	0	Amplification	2	0
MSC	chr8	8p21.2	2	0	2	0	Amplification	2	0
MTFR1	chr8	8q13.1	2	0	2	0	Amplification	2	0
MYBL1	chr8	8q13.1	2	0	2	0	Amplification	2	0
NBN	chr8	1p34.1	2	0	2	0	Amplification	2	0
NCOA2	chr8	8q13.3	2	0	2	0	Amplification	2	0
NKAIN3	chr8	8q12.3	2	0	2	0	Amplification	2	0
NSMAF	chr8	8q12.1	2	0	2	0	Amplification	2	0
OSGIN2	chr8	8q21.3	2	0	2	0	Amplification	2	0
PAG1	chr8	8q21.13	2	0	2	0	Amplification	2	0
PDE7A	chr8	8q13.1	2	0	2	0	Amplification	2	0
PENK	chr8	8q12.1	2	0	2	0	Amplification	2	0
PEX2	chr8	8q21.13	2	0	2	0	Amplification	2	0
PI15	chr8	8q21.13	2	0	2	0	Amplification	2	0
PKIA	chr8	8q21.13	2	0	2	0	Amplification	2	0
PLAG1	chr8	8q12.1	2	0	2	0	Amplification	2	0
PMP2	chr8	8q21.13	2	0	2	0	Amplification	2	0
PPP1R42	chr8	8q13.1	2	0	2	0	Amplification	2	0
PRDM14	chr8	8q13.3	2	0	2	0	Amplification	2	0
PREX2	chr8	8q13.2	2	0	2	0	Amplification	2	0
PSKH2	chr8	8q21.3	2	0	2	0	Amplification	2	0
RAB2A	chr8	8q12.1-q12.2	2	0	2	0	Amplification	2	0
RALYL	chr8	8q21.2	2	0	2	0	Amplification	2	0
RDH10	chr8	8q21.11	2	0	2	0	Amplification	2	0
RIPK2	chr8	8q21.3	2	0	2	0	Amplification	2	0
RMDN1	chr8	8q21.3	2	0	2	0	Amplification	2	0
RPL7	chr8	8q21.11	2	0	2	0	Amplification	2	0
RPS20	chr8	8q12.1	2	0	2	0	Amplification	2	0
RRS1	chr8	8q13.1	2	0	2	0	Amplification	2	0
SBSPON	chr8	8q21.11	2	0	2	0	Amplification	2	0
SDCBP	chr8	8q12.1	2	0	2	0	Amplification	2	0
SDR16C5	chr8	8q12.1	2	0	2	0	Amplification	2	0
SDR16C6P	chr8	8q12.1	2	0	2	0	Amplification	2	0
SGK3	chr8	8q13.1	2	0	2	0	Amplification	2	0
SLC10A5	chr8	8q21.13	2	0	2	0	Amplification	2	0
SLC7A13	chr8	8q21.3	2	0	2	0	Amplification	2	0
SLCO5A1	chr8	8q13.3	2	0	2	0	Amplification	2	0
SNHG6	chr8	8q13.1|8q13	2	0	2	0	Amplification	2	0
SNX16	chr8	8q21.13	2	0	2	0	Amplification	2	0
SOX17	chr8	8q11.23	2	0	2	0	Amplification	2	0
SPAG11A	chr8	8p23.1	2	1	1	0	Amplification	2	0
STAU2	chr8	8q21.11	2	0	2	0	Amplification	2	0
STMN2	chr8	8q21.13	2	0	2	0	Amplification	2	0
SULF1	chr8	8q13.2-q13.3	2	0	2	0	Amplification	2	0
TCEB1	chr8	8q21.11	2	0	2	0	Amplification	2	0
TCF24	chr8	8q13.1	2	0	2	0	Amplification	2	0
TERF1	chr8	8q21.11	2	0	2	0	Amplification	2	0
TGS1	chr8	1q42.12	2	0	2	0	Amplification	2	0
TMEM68	chr8	8q12.1	2	0	2	0	Amplification	2	0
TMEM70	chr8	8q21.11	2	0	2	0	Amplification	2	0
TOX	chr8	8q12.1	2	0	2	0	Amplification	2	0
TPD52	chr8	8q21.13	2	0	2	0	Amplification	2	0
TRAM1	chr8	8q13.3	2	0	2	0	Amplification	2	0
TRIM55	chr8	8q13.1	2	0	2	0	Amplification	2	0
TRPA1	chr8	8q21.11	2	0	2	0	Amplification	2	0

Patel *et al.* , 2021. Whole-exome sequencing analysis of oral squamous cell carcinoma delineated by tobacco usage habits

Supplementary Table 5. List of genes affected by copy number alteration events in OSCC patients

Gene	Chromosome	Cytoband	Recurrence	Recurrence in smoker cohort	Recurrence in chewer cohort	Recurrence in No habit cohort	State	Samples with CNA gain	Samples with CNA loss
TTPA	chr8	8q12.3	2	0	2	0	Amplification	2	0
UBE2W	chr8	8q21.11	2	0	2	0	Amplification	2	0
UBXN2B	chr8	8q12.1	2	0	2	0	Amplification	2	0
VCPIP1	chr8	8q13.1	2	0	2	0	Amplification	2	0
WWP1	chr8	8q21.3	2	0	2	0	Amplification	2	0
XKR4	chr8	8q12.1	2	0	2	0	Amplification	2	0
XKR9	chr8	8q13.3	2	0	2	0	Amplification	2	0
YTHDF3	chr8	8q12.3	2	0	2	0	Amplification	2	0
ZBTB10	chr8	8q21.13	2	0	2	0	Amplification	2	0
ZC2HC1A	chr8	8q21.13	2	0	2	0	Amplification	2	0
ZFAND1	chr8	8q21.13	2	0	2	0	Amplification	2	0
ZFHX4	chr8	8q21.13	2	0	2	0	Amplification	2	0
ZNF704	chr8	8q21.13	2	0	2	0	Amplification	2	0
CEL	chr9	9q34.13	2	1	1	0	Amplification	2	0
CER1	chr9	3p22-p21.2	2	1	0	1	Amplification	2	0
FREM1	chr9	9p22.3	2	1	0	1	Amplification	2	0
LINC00583	chr9	9p23	2	1	0	1	Amplification	2	0
LURAP1L	chr9	9p23	2	1	0	1	Amplification	2	0
MPDZ	chr9	4q12	2	1	0	1	Amplification	2	0
NFIB	chr9	9p23-p22.3	2	1	0	1	Amplification	2	0
PSIP1	chr9	9p22.3	2	1	0	1	Amplification	2	0
SNAPC3	chr9	9p22.3	2	1	0	1	Amplification	2	0
SPATA31D3	chr9	9q21.32	2	0	0	2	Amplification	2	0
SPATA31D4	chr9	9q21.32	2	0	0	2	Amplification	2	0
TTC39B	chr9	9p22.3	2	1	0	1	Amplification	2	0
TYRP1	chr9	9p23	2	1	0	1	Amplification	2	0
ZDHHC21	chr9	9p22.3	2	1	0	1	Amplification	2	0
INADL	chr1	1p31.3	1	0	1	0	Amplification	2	0
ARHGAP42	chr11	11q22.1	1	1	0	0	Amplification	2	0
FCGR3B	chr1	1q23.3	5	3	2	0	Amplification/ Deletion	3	2
CHEK2P2	chr15	15q11.1	5	2	1	2	Amplification/ Deletion	3	2
CYP2D6	chr22	-	5	2	2	1	Amplification/ Deletion	3	2
POLR2J2	chr7	7q22.1	5	0	3	2	Amplification/ Deletion	3	2
UPK3BL	chr7	7q22.1	5	0	3	2	Amplification/ Deletion	3	2
CFHR4	chr1	1q31.3	3	1	1	1	Amplification/ Deletion	2	1
HSPA6	chr1	1q23.3	3	1	1	1	Amplification/ Deletion	2	1
KIR2DL4	chr19	19q13.42	3	2	1	0	Amplification/ Deletion	2	1
AL645922	chr6	0	3	0	1	2	Amplification/ Deletion	2	1
CCZ1B	chr7	7p22.1	3	1	0	2	Amplification/ Deletion	2	1
AC084121	chr8	0	3	1	1	1	Amplification/ Deletion	2	1
C8orf34	chr8	8q13.2	3	0	2	1	Amplification/ Deletion	2	1
DEFB103B	chr8	8p23.1	3	1	1	1	Amplification/ Deletion	2	1
DEFB104A	chr8	8p23.1	3	1	1	1	Amplification/ Deletion	2	1
DEFB104B	chr8	8p23.1	3	1	1	1	Amplification/ Deletion	2	1

Patel *et al.* , 2021. Whole-exome sequencing analysis of oral squamous cell carcinoma delineated by tobacco usage habits

Supplementary Table 5. List of genes affected by copy number alteration events in OSCC patients

Gene	Chromosome	Cytoband	Recurrence	Recurrence in smoker cohort	Recurrence in chewer cohort	Recurrence in No habit cohort	State	Samples with CNA gain	Samples with CNA loss
DEFB105A	chr8	8p23.1	3	1	1	1	Amplification/ Deletion	2	1
DEFB105B	chr8	8p23.1	3	1	1	1	Amplification/ Deletion	2	1
DEFB106A	chr8	8p23.1	3	1	1	1	Amplification/ Deletion	2	1
DEFB106B	chr8	8p23.1	3	1	1	1	Amplification/ Deletion	2	1
DEFB107A	chr8	8p23.1	3	1	1	1	Amplification/ Deletion	2	1
DEFB107B	chr8	8p23.1	3	1	1	1	Amplification/ Deletion	2	1
SPAG11B	chr8	8p23.1	3	1	1	1	Amplification/ Deletion	2	1
AIM2	chr1	1q23.1-q23.2	1	0	1	0	Amplification	1	0
AL021920	chr1	0	1	0	0	1	Amplification	1	0
AL137798	chr1	0	1	0	0	1	Amplification	1	0
AL645608	chr1	0	1	0	0	1	Amplification	1	0
ALG6	chr1	1p31.3	1	0	1	0	Amplification	1	0
AMY1A	chr1	1p21.1	1	0	0	1	Amplification	1	0
AMY2A	chr1	1p21.1	1	0	0	1	Amplification	1	0
AMY2B	chr1	1p21.1	1	0	0	1	Amplification	1	0
ANGPTL3	chr1	1p31.3	1	0	1	0	Amplification	1	0
APCS	chr1	1q23.2	1	0	1	0	Amplification	1	0
ASPM	chr1	1q31.3	1	0	1	0	Amplification	1	0
ATG4C	chr1	1p31.3	1	0	1	0	Amplification	1	0
ATP6V1G3	chr1	1q31.3	1	0	1	0	Amplification	1	0
C1orf180	chr1	1p22.3	1	0	1	0	Amplification	1	0
C1orf204	chr1	1q23.2	1	0	1	0	Amplification	1	0
C1orf27	chr1	1q31.1	1	0	1	0	Amplification	1	0
C1orf53	chr1	1q31.3	1	0	1	0	Amplification	1	0
C1orf68	chr1	1q21.3	1	0	1	0	Amplification	1	0
C1orf87	chr1	1p32.1	1	0	1	0	Amplification	1	0
CADM3	chr1	1q23.2	1	0	1	0	Amplification	1	0
CD1A	chr1	1q23.1	1	0	1	0	Amplification	1	0
CD1B	chr1	1q23.1	1	0	1	0	Amplification	1	0
CD1C	chr1	1q23.1	1	0	1	0	Amplification	1	0
CD1D	chr1	1q23.1	1	0	1	0	Amplification	1	0
CD1E	chr1	1q23.1	1	0	1	0	Amplification	1	0
CD5L	chr1	1q23.1	1	0	1	0	Amplification	1	0
CDC73	chr1	1q31.2	1	0	1	0	Amplification	1	0
CDK11B	chr1	1p36.33	1	0	1	0	Amplification	1	0
CFH	chr1	1q31.3	1	0	1	0	Amplification	1	0
CFHR2	chr1	1q31.3	1	0	1	0	Amplification	1	0
CFHR5	chr1	1q31.3	1	0	1	0	Amplification	1	0
CRB1	chr1	1q31.3	1	0	1	0	Amplification	1	0
CRCT1	chr1	1q21.3	1	0	1	0	Amplification	1	0
CRNN	chr1	1q21.3	1	0	1	0	Amplification	1	0
CROCCP3	chr1	1p36.13	1	0	0	1	Amplification	1	0
CRP	chr1	20q13.2	1	0	1	0	Amplification	1	0
CTBS	chr1	1p22.3	1	0	1	0	Amplification	1	0
CYP2J2	chr1	1p32.1	1	0	1	0	Amplification	1	0
DARC	chr1	1q23.2	1	0	1	0	Amplification	1	0
DENND1B	chr1	1q31.3	1	0	1	0	Amplification	1	0
DNASE2B	chr1	1p31.1-p22.3	1	0	1	0	Amplification	1	0
DOCK7	chr1	1p31.3	1	0	1	0	Amplification	1	0
DUSP23	chr1	11p11.2	1	0	1	0	Amplification	1	0

Patel *et al.* , 2021. Whole-exome sequencing analysis of oral squamous cell carcinoma delineated by tobacco usage habits

Supplementary Table 5. List of genes affected by copy number alteration events in OSCC patients

**Gene**	**Chromosome**	**Cytoband**	**Recurrence**	**Recurrence in smoker cohort**	**Recurrence in chewer cohort**	**Recurrence in No habit cohort**	**State**	**Samples with CNA gain**	**Samples with CNA loss**
EFCAB7	chr1	1p31.3	1	0	1	0	Amplification	1	0
ELTD1	chr1	1p31.1	1	0	1	0	Amplification	1	0
ERVMER61	chr1	0	1	0	1	0	Amplification	1	0
ESPNP	chr1	1p36.13	1	0	0	1	Amplification	1	0
ETV3	chr1	1q23.1	1	0	1	0	Amplification	1	0
ETV3L	chr1	1q23.1	1	0	1	0	Amplification	1	0
F13B	chr1	1q31.3	1	0	1	0	Amplification	1	0
FAM5C	chr1	1q31.1	1	0	1	0	Amplification	1	0
FCER1A	chr1	1q23.2	1	0	1	0	Amplification	1	0
FCGR2B	chr1	1q23.3	1	1	0	0	Amplification	1	0
FCRL1	chr1	1q23.1	1	0	1	0	Amplification	1	0
FCRL2	chr1	1q23.3	1	0	1	0	Amplification	1	0
FCRL3	chr1	1q23.1	1	0	1	0	Amplification	1	0
FCRL4	chr1	1q23.1	1	0	1	0	Amplification	1	0
FCRL5	chr1	1q23.1	1	0	1	0	Amplification	1	0
FCRL6	chr1	1q23.2	1	0	1	0	Amplification	1	0
FGGY	chr1	1p32.1	1	0	1	0	Amplification	1	0
FLG	chr1	1q21.3	1	0	1	0	Amplification	1	0
FLG2	chr1	1q21.3	1	0	1	0	Amplification	1	0
FOXD3	chr1	1p31.3	1	0	1	0	Amplification	1	0
GLRX2	chr1	1q31.2	1	0	1	0	Amplification	1	0
GNG5	chr1	1p22.3	1	0	1	0	Amplification	1	0
HMCN1	chr1	1q25.3-q31.1	1	0	1	0	Amplification	1	0
HOOK1	chr1	1p32.1	1	0	1	0	Amplification	1	0
IFI16	chr1	1q23.1	1	0	1	0	Amplification	1	0
IFI44	chr1	1p31.1	1	0	1	0	Amplification	1	0
IFI44L	chr1	1p31.1	1	0	1	0	Amplification	1	0
ITGB3BP	chr1	1p31.3	1	0	1	0	Amplification	1	0
IVL	chr1	1q21.3	1	0	1	0	Amplification	1	0
KANK4	chr1	1p31.3	1	0	1	0	Amplification	1	0
KCNK2	chr1	1q41	1	1	0	0	Amplification	1	0
KCNT2	chr1	1q31.3	1	0	1	0	Amplification	1	0
KCTD3	chr1	1q41	1	1	0	0	Amplification	1	0
KIRREL	chr1	1q23.1	1	0	1	0	Amplification	1	0
KLHL17	chr1	1p36.33	1	0	0	1	Amplification	1	0
L1TD1	chr1	1p31.3	1	0	1	0	Amplification	1	0
LCE1A	chr1	1q21.3	1	0	1	0	Amplification	1	0
LCE1B	chr1	1q21.3	1	0	1	0	Amplification	1	0
LCE1C	chr1	1q21.3	1	0	1	0	Amplification	1	0
LCE1D	chr1	1q21.3	1	0	1	0	Amplification	1	0
LCE1E	chr1	1q21.3	1	0	1	0	Amplification	1	0
LCE1F	chr1	1q21.3	1	0	1	0	Amplification	1	0
LCE2A	chr1	1q21.3	1	0	1	0	Amplification	1	0
LCE2B	chr1	1q21.3	1	0	1	0	Amplification	1	0
LCE2C	chr1	1q21.3	1	0	1	0	Amplification	1	0
LCE2D	chr1	1q21.3	1	0	1	0	Amplification	1	0
LCE3A	chr1	1q21.3	1	0	1	0	Amplification	1	0
LCE3D	chr1	1q21.3	1	0	1	0	Amplification	1	0
LCE3E	chr1	1q21.3	1	0	1	0	Amplification	1	0
LCE4A	chr1	1q21.3	1	0	1	0	Amplification	1	0
LCE5A	chr1	1q21.3	1	0	1	0	Amplification	1	0
LCE6A	chr1	1q21.3	1	0	1	0	Amplification	1	0
LELP1	chr1	1q21.3	1	0	1	0	Amplification	1	0
LHX9	chr1	1q31.3	1	0	1	0	Amplification	1	0
LINC00115	chr1	1p36.33	1	0	0	1	Amplification	1	0
LOR	chr1	8p21.3	1	0	1	0	Amplification	1	0
LPAR3	chr1	1p22.3	1	0	1	0	Amplification	1	0
LPHN2	chr1	1p31.1	1	0	1	0	Amplification	1	0

Patel *et al.* , 2021. Whole-exome sequencing analysis of oral squamous cell carcinoma delineated by tobacco usage habits

Supplementary Table 5. List of genes affected by copy number alteration events in OSCC patients

Gene	Chromosome	Cytoband	Recurrence	Recurrence in smoker cohort	Recurrence in chewer cohort	Recurrence in No habit cohort	State	Samples with CNA gain	Samples with CNA loss
MIR181A1HG	chr1	1q32.1	1	0	1	0	Amplification	1	0
MMP23B	chr1	1p36.33	1	0	1	0	Amplification	1	0
MNDA	chr1	1q23.1	1	0	1	0	Amplification	1	0
MST1L	chr1	1p36.13	1	0	0	1	Amplification	1	0
MYSM1	chr1	1p32.1	1	0	1	0	Amplification	1	0
NEK7	chr1	1q31.3	1	0	1	0	Amplification	1	0
NFIA	chr1	1p31.3	1	0	1	0	Amplification	1	0
NOC2L	chr1	1p36.33	1	0	0	1	Amplification	1	0
OCLM	chr1	1q31.1	1	0	1	0	Amplification	1	0
OLFM3	chr1	1p21.1	1	0	1	0	Amplification	1	0
OMA1	chr1	1p32.2-p32.1	1	0	1	0	Amplification	1	0
OR10J1	chr1	1q23.2	1	0	1	0	Amplification	1	0
OR10J3	chr1	1q23.2	1	0	1	0	Amplification	1	0
OR10J5	chr1	1q23.2	1	0	1	0	Amplification	1	0
OR10K1	chr1	1q23.1	1	0	1	0	Amplification	1	0
OR10K2	chr1	1q23.1	1	0	1	0	Amplification	1	0
OR10R2	chr1	1q23.1	1	0	1	0	Amplification	1	0
OR10T2	chr1	1q23.1	1	0	1	0	Amplification	1	0
OR10X1	chr1	1q23.1	1	0	1	0	Amplification	1	0
OR10Z1	chr1	1q23.1	1	0	1	0	Amplification	1	0
OR11L1	chr1	1q44	1	0	1	0	Amplification	1	0
OR13G1	chr1	1q44	1	0	1	0	Amplification	1	0
OR14A16	chr1	1q44	1	0	1	0	Amplification	1	0
OR14A2	chr1	1q44	1	0	1	0	Amplification	1	0
OR14C36	chr1	1q44	1	0	1	0	Amplification	1	0
OR14I1	chr1	1q44	1	0	1	0	Amplification	1	0
OR14K1	chr1	1q44	1	0	1	0	Amplification	1	0
OR1C1	chr1	1q44	1	0	1	0	Amplification	1	0
OR2AK2	chr1	1q44	1	0	1	0	Amplification	1	0
OR2G2	chr1	1q44	1	0	1	0	Amplification	1	0
OR2G6	chr1	1q44	1	0	1	0	Amplification	1	0
OR2L13	chr1	1q44	1	0	1	0	Amplification	1	0
OR2L2	chr1	1q44	1	0	1	0	Amplification	1	0
OR2L3	chr1	1q44	1	0	1	0	Amplification	1	0
OR2L5	chr1	1q44	1	0	1	0	Amplification	1	0
OR2L8	chr1	1q44	1	0	1	0	Amplification	1	0
OR2M2	chr1	1q44	1	0	1	0	Amplification	1	0
OR2M3	chr1	1q44	1	0	1	0	Amplification	1	0
OR2M4	chr1	1q44	1	0	1	0	Amplification	1	0
OR2M5	chr1	1q44	1	0	1	0	Amplification	1	0
OR2M7	chr1	1q44	1	0	1	0	Amplification	1	0
OR2T1	chr1	1q44	1	0	1	0	Amplification	1	0
OR2T10	chr1	1q44	1	0	1	0	Amplification	1	0
OR2T11	chr1	1q44	1	0	1	0	Amplification	1	0
OR2T12	chr1	1q44	1	0	1	0	Amplification	1	0
OR2T2	chr1	1q44	1	0	1	0	Amplification	1	0
OR2T27	chr1	1q44	1	0	1	0	Amplification	1	0
OR2T29	chr1	1q44	1	0	1	0	Amplification	1	0
OR2T3	chr1	1q44	1	0	1	0	Amplification	1	0
OR2T32P	chr1	1q44	1	0	1	0	Amplification	1	0
OR2T33	chr1	1q44	1	0	1	0	Amplification	1	0
OR2T34	chr1	1q44	1	0	1	0	Amplification	1	0
OR2T35	chr1	1q44	1	0	1	0	Amplification	1	0
OR2T4	chr1	1q44	1	0	1	0	Amplification	1	0
OR2T5	chr1	1q44	1	0	1	0	Amplification	1	0
OR2T6	chr1	1q44	1	0	1	0	Amplification	1	0
OR2T7	chr1	1q44	1	0	1	0	Amplification	1	0
OR2T8	chr1	1q44	1	0	1	0	Amplification	1	0

Patel *et al.* , 2021. Whole-exome sequencing analysis of oral squamous cell carcinoma delineated by tobacco usage habits

Supplementary Table 5. List of genes affected by copy number alteration events in OSCC patients

**Gene**	**Chromosome**	**Cytoband**	**Recurrence**	**Recurrence in smoker cohort**	**Recurrence in chewer cohort**	**Recurrence in No habit cohort**	**State**	**Samples with CNA gain**	**Samples with CNA loss**
OR2W3	chr1	1q44	1	0	1	0	Amplification	1	0
OR4F5	chr1	1p36.33	1	0	0	1	Amplification	1	0
OR6F1	chr1	1q44	1	0	1	0	Amplification	1	0
OR6K2	chr1	1q23.1	1	0	1	0	Amplification	1	0
OR6K3	chr1	1q23.1	1	0	1	0	Amplification	1	0
OR6K6	chr1	1q23.1	1	0	1	0	Amplification	1	0
OR6N1	chr1	1q23.1	1	0	1	0	Amplification	1	0
OR6N2	chr1	1q23.1	1	0	1	0	Amplification	1	0
OR6P1	chr1	1q23.1	1	0	1	0	Amplification	1	0
OR6Y1	chr1	1q23.1	1	0	1	0	Amplification	1	0
PDC	chr1	2q35	1	0	1	0	Amplification	1	0
PGBD2	chr1	1q44	1	0	1	0	Amplification	1	0
PGLYRP3	chr1	1q21.3	1	0	1	0	Amplification	1	0
PLA2G4A	chr1	1q31.1	1	0	1	0	Amplification	1	0
PLEKHN1	chr1	1p36.33	1	0	0	1	Amplification	1	0
PRG4	chr1	19p13.2	1	0	1	0	Amplification	1	0
PRKACB	chr1	1p31.1	1	0	1	0	Amplification	1	0
PRR9	chr1	1q21.3	1	0	1	0	Amplification	1	0
PTGFR	chr1	1p31.1	1	0	1	0	Amplification	1	0
PTGS2	chr1	1q31.1	1	0	1	0	Amplification	1	0
PTPRC	chr1	1q31.3-q32.1	1	0	1	0	Amplification	1	0
PYHIN1	chr1	1q23.1	1	0	1	0	Amplification	1	0
RGS1	chr1	1q31.2	1	0	1	0	Amplification	1	0
RGS13	chr1	1q31.2	1	0	1	0	Amplification	1	0
RGS18	chr1	1q31.2	1	0	1	0	Amplification	1	0
RGS2	chr1	13q14	1	0	1	0	Amplification	1	0
RGS21	chr1	1q31.2	1	0	1	0	Amplification	1	0
RPF1	chr1	1p22.3	1	0	1	0	Amplification	1	0
SAMD11	chr1	1p36.33	1	0	0	1	Amplification	1	0
SAMD13	chr1	1p31.1	1	0	1	0	Amplification	1	0
SH3BP5L	chr1	1q44	1	0	1	0	Amplification	1	0
SLAMF8	chr1	1q23.2	1	0	1	0	Amplification	1	0
SLC35E2B	chr1	1p36.33	1	0	1	0	Amplification	1	0
SMCP	chr1	1q21.3	1	0	1	0	Amplification	1	0
SPATA1	chr1	1p22.3	1	0	1	0	Amplification	1	0
SPRR1A	chr1	1q21.3	1	0	1	0	Amplification	1	0
SPRR1B	chr1	1q21.3	1	0	1	0	Amplification	1	0
SPRR2B	chr1	1q21.3	1	0	1	0	Amplification	1	0
SPRR2G	chr1	1q21.3	1	0	1	0	Amplification	1	0
SPRR3	chr1	1q21.3	1	0	1	0	Amplification	1	0
SPRR4	chr1	1q21.3	1	0	1	0	Amplification	1	0
SPTA1	chr1	1q23.1	1	0	1	0	Amplification	1	0
SRGAP2B	chr1	1q21.1	1	0	0	1	Amplification	1	0
SSX2IP	chr1	1p22.3	1	0	1	0	Amplification	1	0
TM2D1	chr1	1p31.3	1	0	1	0	Amplification	1	0
TPR	chr1	1q31.1	1	0	1	0	Amplification	1	0
TRIM58	chr1	1q44	1	0	1	0	Amplification	1	0
TROVE2	chr1	1q31.2	1	0	1	0	Amplification	1	0
TTLL7	chr1	1p31.1	1	0	1	0	Amplification	1	0
U1	chr1	1p36.13	1	0	1	0	Amplification	1	0
UCHL5	chr1	1q31.2	1	0	1	0	Amplification	1	0
USH2A	chr1	1q41	1	1	0	0	Amplification	1	0
USP1	chr1	1p31.3	1	0	1	0	Amplification	1	0
VSIG8	chr1	1q23.2	1	0	1	0	Amplification	1	0
ZBTB41	chr1	1q31.3	1	0	1	0	Amplification	1	0
ZNF672	chr1	1q44	1	0	1	0	Amplification	1	0
ZNF692	chr1	1q44	1	0	1	0	Amplification	1	0
ANTXRL	chr10	10q11.22	1	1	0	0	Amplification	1	0

Patel *et al.* , 2021. Whole-exome sequencing analysis of oral squamous cell carcinoma delineated by tobacco usage habits

Supplementary Table 5. List of genes affected by copy number alteration events in OSCC patients

Gene	Chromosome	Cytoband	Recurrence	Recurrence in smoker cohort	Recurrence in chewer cohort	Recurrence in No habit cohort	State	Samples with CNA gain	Samples with CNA loss
ANXA8L1	chr10	10q11.22	1	1	0	0	Amplification	1	0
GPRIN2	chr10	10q11.22	1	1	0	0	Amplification	1	0
NPY4R	chr10	10q11.22	1	1	0	0	Amplification	1	0
POLR3A	chr10	10q22.3	1	0	1	0	Amplification	1	0
RPS24	chr10	10q22.3	1	0	1	0	Amplification	1	0
SYT15	chr10	10q11.22	1	1	0	0	Amplification	1	0
ACCS	chr11	11p11.2	1	0	1	0	Amplification	1	0
ACCSL	chr11	11p11.2	1	0	1	0	Amplification	1	0
ALKBH3	chr11	11p11.2	1	0	1	0	Amplification	1	0
ALX4	chr11	11p11.2	1	0	1	0	Amplification	1	0
AMBRA1	chr11	11p11.2	1	0	1	0	Amplification	1	0
ANO1	chr11	11q13.3	1	1	0	0	Amplification	1	0
ARHGAP1	chr11	11p11.2	1	0	1	0	Amplification	1	0
ATG13	chr11	11p11.2	1	0	1	0	Amplification	1	0
ATL3	chr11	11q13.1	1	0	1	0	Amplification	1	0
BBS1	chr11	11q13.2	1	0	1	0	Amplification	1	0
C11orf49	chr11	11p11.2	1	0	1	0	Amplification	1	0
C11orf84	chr11	11q13.1	1	0	1	0	Amplification	1	0
C11orf94	chr11	11p11.2	1	0	1	0	Amplification	1	0
C11orf95	chr11	11q13.1	1	0	1	0	Amplification	1	0
C11orf96	chr11	11p11.2	1	0	1	0	Amplification	1	0
CCND1	chr11	11q13.3	1	1	0	0	Amplification	1	0
CD82	chr11	11p11.2	1	0	1	0	Amplification	1	0
CHRM4	chr11	11p11.2	1	0	1	0	Amplification	1	0
CKAP5	chr11	11p11.2	1	0	1	0	Amplification	1	0
CNTN5	chr11	11q22.1	1	1	0	0	Amplification	1	0
CREB3L1	chr11	11p11.2	1	0	1	0	Amplification	1	0
CRY2	chr11	11p11.2	1	0	1	0	Amplification	1	0
CTSF	chr11	11q13.2	1	0	1	0	Amplification	1	0
CTTN	chr11	11q13.3	1	1	0	0	Amplification	1	0
DCUN1D5	chr11	11q22.3	1	1	0	0	Amplification	1	0
DGKZ	chr11	11p11.2	1	0	1	0	Amplification	1	0
DPP3	chr11	11q13.2	1	0	1	0	Amplification	1	0
EXT2	chr11	19p	1	0	1	0	Amplification	1	0
F2	chr11	11p11.2	1	0	1	0	Amplification	1	0
FADD	chr11	11q13.3	1	1	0	0	Amplification	1	0
FGF19	chr11	11q13.3	1	1	0	0	Amplification	1	0
FGF3	chr11	11q13.3	1	1	0	0	Amplification	1	0
FGF4	chr11	11q13.3	1	1	0	0	Amplification	1	0
GYLTL1B	chr11	11p11.2	1	0	1	0	Amplification	1	0
HARBI1	chr11	11p11.2	1	0	1	0	Amplification	1	0
HRASLS2	chr11	11q12.3	1	0	1	0	Amplification	1	0
HSD17B12	chr11	11p11.2	1	0	1	0	Amplification	1	0
LGALS12	chr11	11q12.3	1	0	1	0	Amplification	1	0
LRP4	chr11	11p11.2	1	0	1	0	Amplification	1	0
MAPK8IP1	chr11	11p11.2	1	0	1	0	Amplification	1	0
MARK2	chr11	11q13.1	1	0	1	0	Amplification	1	0
MDK	chr11	2q24-q32	1	0	1	0	Amplification	1	0
MMP12	chr11	11q22.2	1	1	0	0	Amplification	1	0
MMP13	chr11	11q22.2	1	1	0	0	Amplification	1	0
MRPL11	chr11	11q13.2	1	0	1	0	Amplification	1	0
MYEOV	chr11	11q13.3	1	1	0	0	Amplification	1	0
NPAS4	chr11	11q13.2	1	0	1	0	Amplification	1	0
ORAOV1	chr11	11q13.3	1	1	0	0	Amplification	1	0
PELI3	chr11	11q13.2	1	0	1	0	Amplification	1	0
PEX16	chr11	11p11.2	1	0	1	0	Amplification	1	0
PGR	chr11	11q22.1	1	1	0	0	Amplification	1	0
PHF21A	chr11	11p11.2	1	0	1	0	Amplification	1	0

Supplementary Table 5. List of genes affected by copy number alteration events in OSCC patients

Gene	Chromosome	Cytoband	Recurrence	Recurrence in smoker cohort	Recurrence in chewer cohort	Recurrence in No habit cohort	State	Samples with CNA gain	Samples with CNA loss
PLA2G16	chr11	11q12.3-q13.1	1	0	1	0	Amplification	1	0
PPFIA1	chr11	11q13.3	1	1	0	0	Amplification	1	0
PRDM11	chr11	11p11.2	1	0	1	0	Amplification	1	0
RARRES3	chr11	11q12.3	1	0	1	0	Amplification	1	0
RCOR2	chr11	11q13.1	1	0	1	0	Amplification	1	0
RHOD	chr11	11q13.2	1	0	0	1	Amplification	1	0
RTN3	chr11	11q13.1	1	0	1	0	Amplification	1	0
SLC35C1	chr11	11p11.2	1	0	1	0	Amplification	1	0
SYT13	chr11	11p11.2	1	0	1	0	Amplification	1	0
TMEM133	chr11	11q22.1	1	1	0	0	Amplification	1	0
TP53I11	chr11	11p11.2	1	0	1	0	Amplification	1	0
TSPAN18	chr11	11p11.2	1	0	1	0	Amplification	1	0
ZDHHC24	chr11	11q13.2	1	0	1	0	Amplification	1	0
ZNF408	chr11	16q24.2	1	0	1	0	Amplification	1	0
NANOGP1	chr12	12p13.31	1	0	1	0	Amplification	1	0
SLC2A3	chr12	12p13.31	1	0	1	0	Amplification	1	0
MPHOSPH8	chr13	13q12.11	1	0	0	1	Amplification	1	0
PSPC1	chr13	13q12.11	1	0	0	1	Amplification	1	0
ZMYM2	chr13	13q12.11	1	0	0	1	Amplification	1	0
ZMYM5	chr13	13q12.11	1	0	0	1	Amplification	1	0
ADAM6	chr14	14q32.33	1	0	1	0	Amplification	1	0
AE000661	chr14	0	1	0	1	0	Amplification	1	0
AHNAK2	chr14	14q32.33	1	0	0	1	Amplification	1	0
AKAP6	chr14	14q12	1	1	0	0	Amplification	1	0
AP4S1	chr14	14q12	1	1	0	0	Amplification	1	0
ARHGAP5	chr14	14q12	1	1	0	0	Amplification	1	0
BAZ1A	chr14	14q13.1-q13.2	1	1	0	0	Amplification	1	0
BNIP3P1	chr14	14q12	1	1	0	0	Amplification	1	0
C14orf23	chr14	14q12	1	1	0	0	Amplification	1	0
CFL2	chr14	14q13.1	1	1	0	0	Amplification	1	0
COCH	chr14	14q12	1	1	0	0	Amplification	1	0
DTD2	chr14	14q12	1	1	0	0	Amplification	1	0
EAPP	chr14	14q13.1	1	1	0	0	Amplification	1	0
EGLN3	chr14	14q13.1	1	1	0	0	Amplification	1	0
FAM177A1	chr14	14q13.2	1	1	0	0	Amplification	1	0
FOXG1	chr14	14q12	1	1	0	0	Amplification	1	0
G2E3	chr14	14q12	1	1	0	0	Amplification	1	0
HEATR5A	chr14	14q12	1	1	0	0	Amplification	1	0
HECTD1	chr14	14q12	1	1	0	0	Amplification	1	0
INSM2	chr14	14q13.2	1	1	0	0	Amplification	1	0
KIAA0125	chr14	14q32.33	1	0	1	0	Amplification	1	0
KIAA0391	chr14	14q13.2	1	1	0	0	Amplification	1	0
LINC00221	chr14	14q32.33	1	0	1	0	Amplification	1	0
NFKBIA	chr14	14q13.2	1	1	0	0	Amplification	1	0
NOVA1	chr14	14q12	1	1	0	0	Amplification	1	0
NPAS3	chr14	14q13.1	1	1	0	0	Amplification	1	0
NUBPL	chr14	14q12	1	1	0	0	Amplification	1	0
OR10G2	chr14	14q11.2	1	0	0	1	Amplification	1	0
OR4K13	chr14	14q11.2	1	0	1	0	Amplification	1	0
OR4K14	chr14	14q11.2	1	0	1	0	Amplification	1	0
OR4K15	chr14	14q11.2	1	0	1	0	Amplification	1	0
PPP2R3C	chr14	14q13.2	1	1	0	0	Amplification	1	0
PRKD1	chr14	14q12	1	1	0	0	Amplification	1	0
PSMA6	chr14	14q13.2	1	1	0	0	Amplification	1	0
RALGAPA1	chr14	14q13.2	1	1	0	0	Amplification	1	0
SCFD1	chr14	14q12	1	1	0	0	Amplification	1	0
SNX6	chr14	14q13.1	1	1	0	0	Amplification	1	0

Supplementary Table 5. List of genes affected by copy number alteration events in OSCC patients

Gene	Chromosome	Cytoband	Recurrence	Recurrence in smoker cohort	Recurrence in chewer cohort	Recurrence in No habit cohort	State	Samples with CNA gain	Samples with CNA loss
SPTSSA	chr14	14q13.1	1	1	0	0	Amplification	1	0
SRP54	chr14	14q13.2	1	1	0	0	Amplification	1	0
STRN3	chr14	14q12	1	1	0	0	Amplification	1	0
CES1	chr16	16q12.2	1	0	1	0	Amplification	1	0
CLEC18B	chr16	16q23.1	1	0	1	0	Amplification	1	0
EIF3C	chr16	16p11.2	1	1	0	0	Amplification	1	0
NPIPB15	chr16	16q23.1	1	0	1	0	Amplification	1	0
SLC6A10P	chr16	16p11.2	1	0	1	0	Amplification	1	0
GH1	chr17	17q23.3	1	0	0	1	Amplification	1	0
KRT34	chr17	17q21.2	1	1	0	0	Amplification	1	0
SHMT1	chr17	17p11.2	1	0	1	0	Amplification	1	0
SLFN11	chr17	17q12	1	0	1	0	Amplification	1	0
SLFN12	chr17	17q12	1	0	1	0	Amplification	1	0
SMCR8	chr17	17p11.2	1	0	1	0	Amplification	1	0
TOP3A	chr17	17p11.2	1	0	1	0	Amplification	1	0
ACAA2	chr18	18q21.1	1	0	0	1	Amplification	1	0
AFG3L2	chr18	18p11.21	1	0	1	0	Amplification	1	0
ANKRD12	chr18	18p11.22	1	0	1	0	Amplification	1	0
ANKRD30B	chr18	18p11.21	1	0	1	0	Amplification	1	0
ANKRD62	chr18	18p11.21	1	0	1	0	Amplification	1	0
AP005482	chr18	0	1	0	1	0	Amplification	1	0
APCDD1	chr18	18p11.22	1	0	1	0	Amplification	1	0
C18orf32	chr18	18q21.1	1	0	0	1	Amplification	1	0
CEP192	chr18	18p11.21	1	0	1	0	Amplification	1	0
CEP76	chr18	18p11.21	1	0	1	0	Amplification	1	0
CHMP1B	chr18	18p11.21	1	0	1	0	Amplification	1	0
CIDEA	chr18	18p11.21|18	1	0	1	0	Amplification	1	0
COLEC12	chr18	18p11.32	1	0	1	0	Amplification	1	0
DYM	chr18	18q21.1	1	0	0	1	Amplification	1	0
FAM210A	chr18	18p11.21	1	0	1	0	Amplification	1	0
GNAL	chr18	18p11.21	1	0	1	0	Amplification	1	0
IMPA2	chr18	18p11.21	1	0	1	0	Amplification	1	0
LDLRAD4	chr18	18p11.21	1	0	1	0	Amplification	1	0
LIPG	chr18	18q21.1	1	0	0	1	Amplification	1	0
LRRC30	chr18	18p11.23	1	0	1	0	Amplification	1	0
MC2R	chr18	18p11.21	1	0	1	0	Amplification	1	0
MC5R	chr18	18p11.21	1	0	1	0	Amplification	1	0
MPPE1	chr18	18p11.21	1	0	1	0	Amplification	1	0
MYO5B	chr18	18q21.1	1	0	0	1	Amplification	1	0
NAPG	chr18	18p11.22	1	0	1	0	Amplification	1	0
NDUFV2	chr18	18p11.22	1	0	1	0	Amplification	1	0
NPIPB1P	chr18	18p11.21	1	0	1	0	Amplification	1	0
PIEZO2	chr18	18p11.22-p11.21	1	0	1	0	Amplification	1	0
POTEC	chr18	18p11.21	1	0	1	0	Amplification	1	0
PPP4R1	chr18	18p11.22	1	0	1	0	Amplification	1	0
PSMG2	chr18	18p11.21	1	0	1	0	Amplification	1	0
PTPN2	chr18	18p11.21	1	0	1	0	Amplification	1	0
PTPRM	chr18	18p11.23	1	0	1	0	Amplification	1	0
RAB12	chr18	18p11.22	1	0	1	0	Amplification	1	0
RAB31	chr18	18p11.22	1	0	1	0	Amplification	1	0
RALBP1	chr18	6q24.1	1	0	1	0	Amplification	1	0
RNMT	chr18	18p11.21	1	0	1	0	Amplification	1	0
ROCK1P1	chr18	18p11.32	1	0	1	0	Amplification	1	0
RPL17	chr18	18q21.1	1	0	0	1	Amplification	1	0
SEH1L	chr18	18p11.21	1	0	1	0	Amplification	1	0
SLMO1	chr18	18p11.21	1	0	1	0	Amplification	1	0
SMAD7	chr18	18q21.1	1	0	0	1	Amplification	1	0
SOGA2	chr18	18p11.22	1	0	1	0	Amplification	1	0

Patel *et al.* , 2021. Whole-exome sequencing analysis of oral squamous cell carcinoma delineated by tobacco usage habits

Supplementary Table 5. List of genes affected by copy number alteration events in OSCC patients

Gene	Chromosome	Cytoband	Recurrence	Recurrence in smoker cohort	Recurrence in chewer cohort	Recurrence in No habit cohort	State	Samples with CNA gain	Samples with CNA loss
SPIRE1	chr18	18p11.21	1	0	1	0	Amplification	1	0
THOC1	chr18	18p11.32	1	0	1	0	Amplification	1	0
TUBB6	chr18	18p11.21	1	0	1	0	Amplification	1	0
TWSG1	chr18	18p11.22	1	0	1	0	Amplification	1	0
TXNDC2	chr18	18p11.22	1	0	1	0	Amplification	1	0
USP14	chr18	18p11.32	1	0	1	0	Amplification	1	0
VAPA	chr18	18p11.22	1	0	1	0	Amplification	1	0
ZNF519	chr18	18p11.21	1	0	1	0	Amplification	1	0
GTPBP3	chr19	19p13.11	1	0	0	1	Amplification	1	0
KIR2DL1	chr19	19q13.42	1	0	0	1	Amplification	1	0
LILRB2	chr19	19q13.42	1	1	0	0	Amplification	1	0
ZNF468	chr19	19q13.42	1	0	1	0	Amplification	1	0
ZNF761	chr19	19q13.42	1	0	0	1	Amplification	1	0
ZNF813	chr19	19q13.42	1	0	0	1	Amplification	1	0
ANKRD44	chr2	2q33.1	1	0	0	1	Amplification	1	0
C2orf66	chr2	2q33.1	1	0	0	1	Amplification	1	0
COQ10B	chr2	2q33.1	1	0	0	1	Amplification	1	0
FAM126B	chr2	2q33.1	1	0	0	1	Amplification	1	0
GTF3C3	chr2	2q33.1	1	0	0	1	Amplification	1	0
PGAP1	chr2	2q33.1	1	0	0	1	Amplification	1	0
PRKRA	chr2	2q31.2	1	1	0	0	Amplification	1	0
SF3B1	chr2	2q33.1	1	0	0	1	Amplification	1	0
SP140L	chr2	2q37.1	1	0	0	1	Amplification	1	0
AHCY	chr20	20q11.22	1	1	0	0	Amplification	1	0
ASIP	chr20	10p11.22-p11.21	1	1	0	0	Amplification	1	0
CDH26	chr20	20q13.33	1	0	0	1	Amplification	1	0
DYNLRB1	chr20	20q11.22	1	1	0	0	Amplification	1	0
EIF2S2	chr20	20q11.22	1	1	0	0	Amplification	1	0
ESF1	chr20	20p12.1	1	0	0	1	Amplification	1	0
ITCH	chr20	20q11.22	1	1	0	0	Amplification	1	0
NDUFAF5	chr20	20p12.1	1	0	0	1	Amplification	1	0
RALY	chr20	20q11.22	1	1	0	0	Amplification	1	0
STAU1	chr20	20q13.13	1	1	0	0	Amplification	1	0
ABCC13	chr21	21q11.2	1	1	0	0	Amplification	1	0
AF165138	chr21	0	1	1	0	0	Amplification	1	0
AIRE	chr21	21q22.3	1	0	0	1	Amplification	1	0
AL050302	chr21	0	1	1	0	0	Amplification	1	0
AL050303	chr21	0	1	1	0	0	Amplification	1	0
ANKRD20A11P	chr21	21q11.2	1	1	0	0	Amplification	1	0
AP001062	chr21	0	1	0	0	1	Amplification	1	0
AP001347	chr21	0	1	1	0	0	Amplification	1	0
C21orf2	chr21	21q22.3	1	0	0	1	Amplification	1	0
C21orf37	chr21	21q21.1	1	1	0	0	Amplification	1	0
CXADR	chr21	21q21.1	1	1	0	0	Amplification	1	0
DNMT3L	chr21	21q22.3	1	0	0	1	Amplification	1	0
HSPA13	chr21	21q11.2	1	1	0	0	Amplification	1	0
ICOSLG	chr21	21q22.3	1	0	0	1	Amplification	1	0
LINC00478	chr21	21q21.1	1	1	0	0	Amplification	1	0
LIPI	chr21	21q11.2	1	1	0	0	Amplification	1	0
NRIP1	chr21	21q11.2-q21.1	1	1	0	0	Amplification	1	0
PFKL	chr21	21q22.3	1	0	0	1	Amplification	1	0
POTED	chr21	21q11.2	1	1	0	0	Amplification	1	0
RBM11	chr21	21q11.2	1	1	0	0	Amplification	1	0
SAMSN1	chr21	21q11.2	1	1	0	0	Amplification	1	0
TEKT4P2	chr21	21p11.2	1	0	1	0	Amplification	1	0
TPTE	chr21	21p11.2	1	0	1	0	Amplification	1	0
USP25	chr21	21q21.1	1	1	0	0	Amplification	1	0

Patel *et al.* , 2021. Whole-exome sequencing analysis of oral squamous cell carcinoma delineated by tobacco usage habits

Supplementary Table 5. List of genes affected by copy number alteration events in OSCC patients

Gene	Chromosome	Cytoband	Recurrence	Recurrence in smoker cohort	Recurrence in chewer cohort	Recurrence in No habit cohort	State	Samples with CNA gain	Samples with CNA loss
ANKRD62P1	chr22	22q11.1	1	1	0	0	Amplification	1	0
CCDC116	chr22	22q11.21	1	0	1	0	Amplification	1	0
CRYBB2P1	chr22	22q11.23	1	1	0	0	Amplification	1	0
CYP2D7P1	chr22	22q13.2	1	1	0	0	Amplification	1	0
LRP5L	chr22	22q11.23	1	1	0	0	Amplification	1	0
MAPK1	chr22	22q11.22	1	0	1	0	Amplification	1	0
PPIL2	chr22	22q11.21	1	0	1	0	Amplification	1	0
SDF2L1	chr22	22q11.21	1	0	1	0	Amplification	1	0
TPTEP1	chr22	22q11.1	1	1	0	0	Amplification	1	0
UBE2L3	chr22	22q11.21	1	0	1	0	Amplification	1	0
XKR3	chr22	22q11.1	1	1	0	0	Amplification	1	0
YDJC	chr22	22q11.21	1	0	1	0	Amplification	1	0
YPEL1	chr22	22q11.21-q11.22	1	0	1	0	Amplification	1	0
A4GNT	chr3	3q22.3	1	0	1	0	Amplification	1	0
AADAC	chr3	3q25.1	1	0	1	0	Amplification	1	0
AADACL2	chr3	3q25.1	1	0	1	0	Amplification	1	0
ABCC5	chr3	3q27.1	1	0	1	0	Amplification	1	0
ABCF3	chr3	3q27.1	1	0	1	0	Amplification	1	0
AC022498	chr3	0	1	0	1	0	Amplification	1	0
AC092964	chr3	0	1	0	1	0	Amplification	1	0
AC104472	chr3	0	1	0	1	0	Amplification	1	0
AC107021	chr3	0	1	0	1	0	Amplification	1	0
ACAD11	chr3	3q22.1	1	0	1	0	Amplification	1	0
ACAP2	chr3	3q29	1	0	1	0	Amplification	1	0
ACKR4	chr3	3q22.1	1	0	1	0	Amplification	1	0
ACPL2	chr3	3q23	1	0	1	0	Amplification	1	0
ACPP	chr3	3q22.1	1	0	1	0	Amplification	1	0
ACTL6A	chr3	3q26.33	1	0	1	0	Amplification	1	0
ADIPOQ	chr3	3q27.3	1	0	1	0	Amplification	1	0
AGTR1	chr3	3q24	1	0	1	0	Amplification	1	0
AHSG	chr3	3q27.3	1	0	1	0	Amplification	1	0
ALG3	chr3	3q27.1	1	0	1	0	Amplification	1	0
AMOTL2	chr3	3q22.2	1	0	1	0	Amplification	1	0
ANAPC13	chr3	3q22.2	1	0	1	0	Amplification	1	0
ANKUB1	chr3	3q25.1	1	0	1	0	Amplification	1	0
AP2M1	chr3	3q27.1	1	0	1	0	Amplification	1	0
APOD	chr3	3q29	1	0	1	0	Amplification	1	0
ARHGEF26	chr3	3q25.2	1	0	1	0	Amplification	1	0
ARL14	chr3	3q25.33	1	0	1	0	Amplification	1	0
ARMC8	chr3	3q22.3	1	0	1	0	Amplification	1	0
ATP11B	chr3	3q26.33	1	0	1	0	Amplification	1	0
ATP13A3	chr3	3q29	1	0	1	0	Amplification	1	0
ATP13A4	chr3	3q29	1	0	1	0	Amplification	1	0
ATP13A5	chr3	3q29	1	0	1	0	Amplification	1	0
ATP1B3	chr3	3q23	1	0	1	0	Amplification	1	0
ATR	chr3	14q32.13	1	0	1	0	Amplification	1	0
B3GALNT1	chr3	3q26.1	1	0	1	0	Amplification	1	0
B3GNT5	chr3	3q27.1	1	0	1	0	Amplification	1	0
BCHE	chr3	3q26.1	1	0	1	0	Amplification	1	0
BCL6	chr3	3q27.3	1	0	1	0	Amplification	1	0
BDH1	chr3	3q29	1	0	1	0	Amplification	1	0
BFSP2	chr3	3q22.1	1	0	1	0	Amplification	1	0
C3orf33	chr3	3q25.31	1	0	1	0	Amplification	1	0
C3orf36	chr3	3q22.1	1	0	1	0	Amplification	1	0
C3orf38	chr3	3p11.1	1	0	1	0	Amplification	1	0
C3orf55	chr3	3q25.32	1	0	1	0	Amplification	1	0
C3orf58	chr3	3q24	1	0	1	0	Amplification	1	0
C3orf65	chr3	3q27.2	1	0	1	0	Amplification	1	0

Patel *et al.* , 2021. Whole-exome sequencing analysis of oral squamous cell carcinoma delineated by tobacco usage habits

Supplementary Table 5. List of genes affected by copy number alteration events in OSCC patients

Gene	Chromosome	Cytoband	Recurrence	Recurrence in smoker cohort	Recurrence in chewer cohort	Recurrence in No habit cohort	State	Samples with CNA gain	Samples with CNA loss
C3orf70	chr3	3q27.2	1	0	1	0	Amplification	1	0
C3orf72	chr3	3q22.3	1	0	1	0	Amplification	1	0
C3orf79	chr3	3q25.2	1	0	1	0	Amplification	1	0
C3orf80	chr3	3q25.33	1	0	1	0	Amplification	1	0
CADM2	chr3	3p12.1	1	0	1	0	Amplification	1	0
CAMK2N2	chr3	3q27.1	1	0	1	0	Amplification	1	0
CCDC39	chr3	3q26.33	1	0	1	0	Amplification	1	0
CCDC50	chr3	3q28	1	0	1	0	Amplification	1	0
CCNL1	chr3	3q25.31	1	0	1	0	Amplification	1	0
CDV3	chr3	3q22.1	1	0	1	0	Amplification	1	0
CEP19	chr3	3q29	1	0	1	0	Amplification	1	0
CEP63	chr3	3q22.2	1	0	1	0	Amplification	1	0
CEP70	chr3	3q22.3	1	0	1	0	Amplification	1	0
CGGBP1	chr3	3p11.1	1	0	1	0	Amplification	1	0
CHMP2B	chr3	3p11.2	1	0	1	0	Amplification	1	0
CHRD	chr3	3q27.1	1	0	1	0	Amplification	1	0
CLCN2	chr3	3q27.1	1	0	1	0	Amplification	1	0
CLDN1	chr3	3q28	1	0	1	0	Amplification	1	0
CLDN11	chr3	3q26.2	1	0	1	0	Amplification	1	0
CLDN16	chr3	3q28	1	0	1	0	Amplification	1	0
CLDN18	chr3	3q22.3	1	0	1	0	Amplification	1	0
CLRN1	chr3	3q25.1	1	0	1	0	Amplification	1	0
CLSTN2	chr3	3q23	1	0	1	0	Amplification	1	0
COMMD2	chr3	3q25.1	1	0	1	0	Amplification	1	0
COPB2	chr3	3q23	1	0	1	0	Amplification	1	0
CP	chr3	3q24-q25.1	1	0	1	0	Amplification	1	0
CPA3	chr3	7q32.2	1	0	1	0	Amplification	1	0
CPB1	chr3	3q24	1	0	1	0	Amplification	1	0
CPN2	chr3	16q13	1	0	1	0	Amplification	1	0
CPNE4	chr3	3q22.1	1	0	1	0	Amplification	1	0
CRYGS	chr3	3q27.3	1	0	1	0	Amplification	1	0
DBR1	chr3	3q22.3	1	0	1	0	Amplification	1	0
DCUN1D1	chr3	3q26.33	1	0	1	0	Amplification	1	0
DGKG	chr3	3q27.2-q27.3	1	0	1	0	Amplification	1	0
DHX36	chr3	3q25.2	1	0	1	0	Amplification	1	0
DLG1	chr3	3q29	1	0	1	0	Amplification	1	0
DNAJB11	chr3	3q27.3	1	0	1	0	Amplification	1	0
DNAJC13	chr3	3q22.1	1	0	1	0	Amplification	1	0
DNAJC19	chr3	3q26.33	1	0	1	0	Amplification	1	0
DVL3	chr3	3q27.1	1	0	1	0	Amplification	1	0
DZIP1L	chr3	3q22.3	1	0	1	0	Amplification	1	0
ECE2	chr3	3q27.1	1	0	1	0	Amplification	1	0
ECT2	chr3	3q26.31	1	0	1	0	Amplification	1	0
EGFEM1P	chr3	3q26.2	1	0	1	0	Amplification	1	0
EHHADH	chr3	3q27.2	1	0	1	0	Amplification	1	0
EIF2A	chr3	3q25.1	1	0	1	0	Amplification	1	0
EIF2B5	chr3	3q27.1	1	0	1	0	Amplification	1	0
EIF4A2	chr3	3q27.3	1	0	1	0	Amplification	1	0
EIF4G1	chr3	3q27.1	1	0	1	0	Amplification	1	0
EIF5A2	chr3	3q26.2	1	0	1	0	Amplification	1	0
EPHA3	chr3	3p11.1	1	0	1	0	Amplification	1	0
EPHB1	chr3	3q22.2	1	0	1	0	Amplification	1	0
EPHB3	chr3	3q27.1	1	0	1	0	Amplification	1	0
ESYT3	chr3	3q22.3	1	0	1	0	Amplification	1	0
ETV5	chr3	3q27.2	1	0	1	0	Amplification	1	0
FAIM	chr3	3q22.3	1	0	1	0	Amplification	1	0
FAM131A	chr3	3q27.1	1	0	1	0	Amplification	1	0
FAM188B2	chr3	3q25.1	1	0	1	0	Amplification	1	0

Patel *et al.* , 2021. Whole-exome sequencing analysis of oral squamous cell carcinoma delineated by tobacco usage habits

Supplementary Table 5. List of genes affected by copy number alteration events in OSCC patients

**Gene**	**Chromosome**	**Cytoband**	**Recurrence**	**Recurrence in smoker cohort**	**Recurrence in chewer cohort**	**Recurrence in No habit cohort**	**State**	**Samples with CNA gain**	**Samples with CNA loss**
FAM194A	chr3	3q25.1	1	0	1	0	Amplification	1	0
FBXO45	chr3	3q29	1	0	1	0	Amplification	1	0
FETUB	chr3	3q27.3	1	0	1	0	Amplification	1	0
FGF12	chr3	3q28-q29	1	0	1	0	Amplification	1	0
FNDC3B	chr3	3q26.31	1	0	1	0	Amplification	1	0
FOXL2	chr3	3q22.3	1	0	1	0	Amplification	1	0
FXR1	chr3	3q26.33	1	0	1	0	Amplification	1	0
FYTTD1	chr3	3q29	1	0	1	0	Amplification	1	0
GFM1	chr3	3q25.32	1	0	1	0	Amplification	1	0
GHSR	chr3	3q26.31	1	0	1	0	Amplification	1	0
GK5	chr3	3q23	1	0	1	0	Amplification	1	0
GMNC	chr3	3q28	1	0	1	0	Amplification	1	0
GMPS	chr3	3q25.31	1	0	1	0	Amplification	1	0
GNB4	chr3	3q26.33	1	0	1	0	Amplification	1	0
GOLIM4	chr3	3q26.2	1	0	1	0	Amplification	1	0
GP5	chr3	3q29	1	0	1	0	Amplification	1	0
GPR128	chr3	3q12.2	1	0	0	1	Amplification	1	0
GPR149	chr3	3q25.2	1	0	1	0	Amplification	1	0
GPR160	chr3	3q26.2	1	0	1	0	Amplification	1	0
GPR171	chr3	3q25.1	1	0	1	0	Amplification	1	0
GPR87	chr3	3q25.1	1	0	1	0	Amplification	1	0
GRK7	chr3	3q23	1	0	1	0	Amplification	1	0
GYG1	chr3	3q24	1	0	1	0	Amplification	1	0
HES1	chr3	21q22.3	1	0	1	0	Amplification	1	0
HLTF	chr3	3q24	1	0	1	0	Amplification	1	0
HPS3	chr3	3q24	1	0	1	0	Amplification	1	0
HRASLS	chr3	3q29	1	0	1	0	Amplification	1	0
HRG	chr3	3q27.3	1	0	1	0	Amplification	1	0
HTR1F	chr3	3p11.2-p11.1	1	0	1	0	Amplification	1	0
HTR3C	chr3	3q27.1	1	0	1	0	Amplification	1	0
HTR3D	chr3	3q27.1	1	0	1	0	Amplification	1	0
HTR3E	chr3	3q27.1	1	0	1	0	Amplification	1	0
IFT80	chr3	3q25.33	1	0	1	0	Amplification	1	0
IGF2BP2	chr3	3q27.2	1	0	1	0	Amplification	1	0
IGSF10	chr3	3q25.1	1	0	1	0	Amplification	1	0
IL12A	chr3	3q25.33	1	0	1	0	Amplification	1	0
IL1RAP	chr3	3q28	1	0	1	0	Amplification	1	0
IL20RB	chr3	3q22.3	1	0	1	0	Amplification	1	0
IQCG	chr3	3q29	1	0	1	0	Amplification	1	0
IQCJ	chr3	3q25.32	1	0	1	0	Amplification	1	0
KCNAB1	chr3	3q25.31	1	0	1	0	Amplification	1	0
KCNMB2	chr3	3q26.32	1	0	1	0	Amplification	1	0
KCNMB3	chr3	3q26.32	1	0	1	0	Amplification	1	0
KIAA0226	chr3	3q29	1	0	1	0	Amplification	1	0
KLHL24	chr3	3q27.1	1	0	1	0	Amplification	1	0
KLHL6	chr3	3q27.1	1	0	1	0	Amplification	1	0
KNG1	chr3	3q27.3	1	0	1	0	Amplification	1	0
KPNA4	chr3	3q25.33	1	0	1	0	Amplification	1	0
KY	chr3	3q22.2	1	0	1	0	Amplification	1	0
LAMP3	chr3	3q27.1	1	0	1	0	Amplification	1	0
LEKR1	chr3	3q25.31	1	0	1	0	Amplification	1	0
LEPREL1	chr3	3q28	1	0	1	0	Amplification	1	0
LIPH	chr3	3q27.2	1	0	1	0	Amplification	1	0
LMLN	chr3	3q29	1	0	1	0	Amplification	1	0
LPP	chr3	3q27.3-q28	1	0	1	0	Amplification	1	0
LRCH3	chr3	3q29	1	0	1	0	Amplification	1	0
LRRC15	chr3	3q29	1	0	1	0	Amplification	1	0
LSG1	chr3	3q29	1	0	1	0	Amplification	1	0

Patel *et al.* , 2021. Whole-exome sequencing analysis of oral squamous cell carcinoma delineated by tobacco usage habits

Supplementary Table 5. List of genes affected by copy number alteration events in OSCC patients

Gene	Chromosome	Cytoband	Recurrence	Recurrence in smoker cohort	Recurrence in chewer cohort	Recurrence in No habit cohort	State	Samples with CNA gain	Samples with CNA loss
LXN	chr3	3q25.32	1	0	1	0	Amplification	1	0
MAGEF1	chr3	3q27.1	1	0	1	0	Amplification	1	0
MAP3K13	chr3	3q27.2	1	0	1	0	Amplification	1	0
MAP6D1	chr3	3q27.1	1	0	1	0	Amplification	1	0
MASP1	chr3	3q27.3	1	0	1	0	Amplification	1	0
MB21D2	chr3	3q29	1	0	1	0	Amplification	1	0
MBNL1	chr3	3q25.1-q25.2	1	0	1	0	Amplification	1	0
MCCC1	chr3	3q27.1	1	0	1	0	Amplification	1	0
MCF2L2	chr3	3q27.1	1	0	1	0	Amplification	1	0
MECOM	chr3	3q26.2	1	0	1	0	Amplification	1	0
MED12L	chr3	3q25.1	1	0	1	0	Amplification	1	0
MFI2	chr3	3q29	1	0	1	0	Amplification	1	0
MFN1	chr3	3q26.33	1	0	1	0	Amplification	1	0
MFSD1	chr3	3q25.32	1	0	1	0	Amplification	1	0
MLF1	chr3	3q25.32	1	0	1	0	Amplification	1	0
MME	chr3	11q22.2	1	0	1	0	Amplification	1	0
MRAS	chr3	3q22.3	1	0	1	0	Amplification	1	0
MRPL47	chr3	3q26.33	1	0	1	0	Amplification	1	0
MRPS22	chr3	3q23	1	0	1	0	Amplification	1	0
MSL2	chr3	3q22.3	1	0	1	0	Amplification	1	0
MUC4	chr3	3q29	1	0	1	0	Amplification	1	0
NAALADL2	chr3	3q26.31	1	0	1	0	Amplification	1	0
NCBP2	chr3	3q29	1	0	1	0	Amplification	1	0
NCEH1	chr3	3q26.31	1	0	1	0	Amplification	1	0
NCK1	chr3	3q22.3	1	0	1	0	Amplification	1	0
NDUFB5	chr3	3q26.33	1	0	1	0	Amplification	1	0
NLGN1	chr3	3q26.31	1	0	1	0	Amplification	1	0
NMD3	chr3	3q26.1	1	0	1	0	Amplification	1	0
NME9	chr3	3q22.3	1	0	1	0	Amplification	1	0
NMNAT3	chr3	3q23	1	0	1	0	Amplification	1	0
NPHP3	chr3	3q22.1	1	0	1	0	Amplification	1	0
OPA1	chr3	Xq13.1	1	0	1	0	Amplification	1	0
OSTN	chr3	3q28	1	0	1	0	Amplification	1	0
OTOL1	chr3	3q26.1	1	0	1	0	Amplification	1	0
P2RY1	chr3	3q25.2	1	0	1	0	Amplification	1	0
P2RY12	chr3	3q25.1	1	0	1	0	Amplification	1	0
P2RY13	chr3	3q25.1	1	0	1	0	Amplification	1	0
P2RY14	chr3	3q25.1	1	0	1	0	Amplification	1	0
PAK2	chr3	1p22.2	1	0	1	0	Amplification	1	0
PAQR9	chr3	3q23	1	0	1	0	Amplification	1	0
PARL	chr3	3q27.1	1	0	1	0	Amplification	1	0
PBX2P1	chr3	3q24	1	0	1	0	Amplification	1	0
PCCB	chr3	3q22.3	1	0	1	0	Amplification	1	0
PCOLCE2	chr3	3q23	1	0	1	0	Amplification	1	0
PCYT1A	chr3	3q29	1	0	1	0	Amplification	1	0
PDCD10	chr3	3q26.1	1	0	1	0	Amplification	1	0
PEX5L	chr3	3q26.33	1	0	1	0	Amplification	1	0
PFN2	chr3	3q25.1	1	0	1	0	Amplification	1	0
PHC3	chr3	3q26.2	1	0	1	0	Amplification	1	0
PIGX	chr3	3q29	1	0	1	0	Amplification	1	0
PIGZ	chr3	3q29	1	0	1	0	Amplification	1	0
PIK3CA	chr3	3q26.32	1	0	1	0	Amplification	1	0
PIK3CB	chr3	3q22.3	1	0	1	0	Amplification	1	0
PLCH1	chr3	3q25.31	1	0	1	0	Amplification	1	0
PLD1	chr3	19p13.2	1	0	1	0	Amplification	1	0
PLOD2	chr3	3q24	1	0	1	0	Amplification	1	0
PLS1	chr3	3q23	1	0	1	0	Amplification	1	0
PLSCR1	chr3	3q24	1	0	1	0	Amplification	1	0

Supplementary Table 5. List of genes affected by copy number alteration events in OSCC patients

Gene	Chromosome	Cytoband	Recurrence	Recurrence in smoker cohort	Recurrence in chewer cohort	Recurrence in No habit cohort	State	Samples with CNA gain	Samples with CNA loss
PLSCR2	chr3	3q24	1	0	1	0	Amplification	1	0
PLSCR4	chr3	3q24	1	0	1	0	Amplification	1	0
PLSCR5	chr3	3q24	1	0	1	0	Amplification	1	0
POLR2H	chr3	3q27.1	1	0	1	0	Amplification	1	0
POU1F1	chr3	3p11.2	1	0	1	0	Amplification	1	0
PP13439	chr3	0	1	0	1	0	Amplification	1	0
PPM1L	chr3	3q25.33-q26.1	1	0	1	0	Amplification	1	0
PPP1R2	chr3	3q29	1	0	1	0	Amplification	1	0
PPP2R3A	chr3	3q22.2-q22.3	1	0	1	0	Amplification	1	0
PRKCI	chr3	3q26.2	1	0	1	0	Amplification	1	0
PRR23A	chr3	3q23	1	0	1	0	Amplification	1	0
PRR23B	chr3	3q23	1	0	1	0	Amplification	1	0
PRR23C	chr3	3q23	1	0	1	0	Amplification	1	0
PSMD2	chr3	3q27.1	1	0	1	0	Amplification	1	0
PTX3	chr3	3q25.32	1	0	1	0	Amplification	1	0
PYDC2	chr3	3q28	1	0	1	0	Amplification	1	0
RAB6B	chr3	3q22.1	1	0	1	0	Amplification	1	0
RAP2B	chr3	3q25.2	1	0	1	0	Amplification	1	0
RARRES1	chr3	3q25.32	1	0	1	0	Amplification	1	0
RASA2	chr3	3q23	1	0	1	0	Amplification	1	0
RBP1	chr3	3q23	1	0	1	0	Amplification	1	0
RBP2	chr3	12q24.33	1	0	1	0	Amplification	1	0
RFC4	chr3	3q27.3	1	0	1	0	Amplification	1	0
RNF13	chr3	3q25.1	1	0	1	0	Amplification	1	0
RNF168	chr3	3q29	1	0	1	0	Amplification	1	0
RNF7	chr3	3q23	1	0	1	0	Amplification	1	0
RPL22L1	chr3	3q26.2	1	0	1	0	Amplification	1	0
RPL35A	chr3	3q29	1	0	1	0	Amplification	1	0
RPL39L	chr3	3q27.3	1	0	1	0	Amplification	1	0
RSRC1	chr3	3q25.32	1	0	1	0	Amplification	1	0
RTP1	chr3	3q27.3	1	0	1	0	Amplification	1	0
RTP2	chr3	3q27.3	1	0	1	0	Amplification	1	0
RTP4	chr3	3q27.3	1	0	1	0	Amplification	1	0
RYK	chr3	3q22.2	1	0	1	0	Amplification	1	0
SCHIP1	chr3	3q25.32-q25.33	1	0	1	0	Amplification	1	0
SDHAP1	chr3	3q29	1	0	1	0	Amplification	1	0
SEC62	chr3	3q26.2	1	0	1	0	Amplification	1	0
SELT	chr3	3q25.1	1	0	1	0	Amplification	1	0
SENP2	chr3	3q27.2	1	0	1	0	Amplification	1	0
SENP5	chr3	3q29	1	0	1	0	Amplification	1	0
SERP1	chr3	3q25.1	1	0	1	0	Amplification	1	0
SERPINI1	chr3	3q26.1	1	0	1	0	Amplification	1	0
SERPINI2	chr3	3q26.1	1	0	1	0	Amplification	1	0
SHOX2	chr3	3q25.32	1	0	1	0	Amplification	1	0
SI	chr3	12q13.2	1	0	1	0	Amplification	1	0
SIAH2	chr3	3q25.1	1	0	1	0	Amplification	1	0
SKIL	chr3	3q26.2	1	0	1	0	Amplification	1	0
SLC25A36	chr3	3q23	1	0	1	0	Amplification	1	0
SLC2A2	chr3	3q26.2	1	0	1	0	Amplification	1	0
SLC33A1	chr3	3q25.31	1	0	1	0	Amplification	1	0
SLC51A	chr3	3q29	1	0	1	0	Amplification	1	0
SLC7A14	chr3	3q26.2	1	0	1	0	Amplification	1	0
SLC9A9	chr3	3q24	1	0	1	0	Amplification	1	0
SLCO2A1	chr3	3q22.1-q22.2	1	0	1	0	Amplification	1	0
SLITRK3	chr3	3q26.1	1	0	1	0	Amplification	1	0

Patel *et al.* , 2021. Whole-exome sequencing analysis of oral squamous cell carcinoma delineated by tobacco usage habits

Supplementary Table 5. List of genes affected by copy number alteration events in OSCC patients

Gene	Chromosome	Cytoband	Recurrence	Recurrence in smoker cohort	Recurrence in chewer cohort	Recurrence in No habit cohort	State	Samples with CNA gain	Samples with CNA loss
SMC4	chr3	3q25.33	1	0	1	0	Amplification	1	0
SMCO1	chr3	3q29	1	0	1	0	Amplification	1	0
SOX14	chr3	3q22.3	1	0	1	0	Amplification	1	0
SOX2	chr3	3q26.33	1	0	1	0	Amplification	1	0
SPATA16	chr3	3q26.31	1	0	1	0	Amplification	1	0
SPSB4	chr3	3q23	1	0	1	0	Amplification	1	0
SPTSSB	chr3	3q26.1	1	0	1	0	Amplification	1	0
SRPRB	chr3	3q22.1	1	0	1	0	Amplification	1	0
SSR3	chr3	3q25.31	1	0	1	0	Amplification	1	0
SST	chr3	3q27.3	1	0	1	0	Amplification	1	0
ST6GAL1	chr3	3q27.3	1	0	1	0	Amplification	1	0
STAG1	chr3	20q13.31	1	0	1	0	Amplification	1	0
SUCNR1	chr3	3q25.1	1	0	1	0	Amplification	1	0
TBCCD1	chr3	3q27.3	1	0	1	0	Amplification	1	0
TBL1XR1	chr3	3q26.32	1	0	1	0	Amplification	1	0
TCTEX1D2	chr3	3q29	1	0	1	0	Amplification	1	0
TF	chr3	3q22.1	1	0	1	0	Amplification	1	0
TFDP2	chr3	3q23	1	0	1	0	Amplification	1	0
TFRC	chr3	3q29	1	0	1	0	Amplification	1	0
THPO	chr3	3q27.1	1	0	1	0	Amplification	1	0
TIPARP	chr3	3q25.31	1	0	1	0	Amplification	1	0
TM4SF1	chr3	3q25.1	1	0	1	0	Amplification	1	0
TM4SF18	chr3	3q25.1	1	0	1	0	Amplification	1	0
TM4SF19	chr3	3q29	1	0	1	0	Amplification	1	0
TM4SF4	chr3	3q25.1	1	0	1	0	Amplification	1	0
TMEM108	chr3	3q22.1	1	0	1	0	Amplification	1	0
TMEM14E	chr3	3q25.2	1	0	1	0	Amplification	1	0
TMEM207	chr3	3q28	1	0	1	0	Amplification	1	0
TMEM212	chr3	3q26.31	1	0	1	0	Amplification	1	0
TMEM41A	chr3	3q27.2	1	0	1	0	Amplification	1	0
TMEM44	chr3	3q29	1	0	1	0	Amplification	1	0
TNFSF10	chr3	3q26.31	1	0	1	0	Amplification	1	0
TNIK	chr3	3q26.2-q26.31	1	0	1	0	Amplification	1	0
TNK2	chr3	3q29	1	0	1	0	Amplification	1	0
TOPBP1	chr3	3q22.1	1	0	1	0	Amplification	1	0
TP63	chr3	3q28	1	0	1	0	Amplification	1	0
TPRG1	chr3	3q28	1	0	1	0	Amplification	1	0
TRA2B	chr3	3q27.2	1	0	1	0	Amplification	1	0
TRIM42	chr3	3q23	1	0	1	0	Amplification	1	0
TRIM59	chr3	3q25.33	1	0	1	0	Amplification	1	0
TRPC1	chr3	3q23	1	0	1	0	Amplification	1	0
TSC22D2	chr3	3q25.1	1	0	1	0	Amplification	1	0
TTC14	chr3	3q26.33	1	0	1	0	Amplification	1	0
U2SURP	chr3	3q23	1	0	1	0	Amplification	1	0
UBA5	chr3	3q22.1	1	0	1	0	Amplification	1	0
UBXN7	chr3	3q29	1	0	1	0	Amplification	1	0
USP13	chr3	3q26.33	1	0	1	0	Amplification	1	0
UTS2B	chr3	3q28	1	0	1	0	Amplification	1	0
VEPH1	chr3	3q25.31-q25.32	1	0	1	0	Amplification	1	0
VGLL3	chr3	3p12.1	1	0	1	0	Amplification	1	0
VPS8	chr3	3q27.2	1	0	1	0	Amplification	1	0
VWA5B2	chr3	3q27.1	1	0	1	0	Amplification	1	0
WDR49	chr3	3q26.1	1	0	1	0	Amplification	1	0
WDR53	chr3	3q29	1	0	1	0	Amplification	1	0
WWTR1	chr3	3q25.1	1	0	1	0	Amplification	1	0
XRN1	chr3	3q23	1	0	1	0	Amplification	1	0
XXYLT1	chr3	3q29	1	0	1	0	Amplification	1	0

Patel *et al.* , 2021. Whole-exome sequencing analysis of oral squamous cell carcinoma delineated by tobacco usage habits

Supplementary Table 5. List of genes affected by copy number alteration events in OSCC patients

Gene	Chromosome	Cytoband	Recurrence	Recurrence in smoker cohort	Recurrence in chewer cohort	Recurrence in No habit cohort	State	Samples with CNA gain	Samples with CNA loss
YEATS2	chr3	3q27.1	1	0	1	0	Amplification	1	0
ZBBX	chr3	3q26.1	1	0	1	0	Amplification	1	0
ZBTB38	chr3	3q23	1	0	1	0	Amplification	1	0
ZDHHC19	chr3	3q29	1	0	1	0	Amplification	1	0
ZIC1	chr3	3q24	1	0	1	0	Amplification	1	0
ZIC4	chr3	3q24	1	0	1	0	Amplification	1	0
ZMAT3	chr3	3q26.32	1	0	1	0	Amplification	1	0
ZNF639	chr3	3q26.33	1	0	1	0	Amplification	1	0
ZNF654	chr3	3p11.1	1	0	1	0	Amplification	1	0
ZNF717	chr3	3p12.3	1	0	1	0	Amplification	1	0
PCDH18	chr4	4q28.3	1	0	1	0	Amplification	1	0
SLC7A11	chr4	4q28.3	1	0	1	0	Amplification	1	0
UGT2B17	chr4	4q13.2	1	0	1	0	Amplification	1	0
ANXA2R	chr5	5p12	1	1	0	0	Amplification	1	0
C5orf28	chr5	5p12	1	1	0	0	Amplification	1	0
C5orf34	chr5	5p12	1	1	0	0	Amplification	1	0
C5orf51	chr5	5p13.1	1	1	0	0	Amplification	1	0
CCDC152	chr5	5p12	1	1	0	0	Amplification	1	0
CCL28	chr5	5p12	1	1	0	0	Amplification	1	0
CTNND2	chr5	5p15.2	1	1	0	0	Amplification	1	0
DNAH5	chr5	5p15.2	1	1	0	0	Amplification	1	0
FBXO4	chr5	5p13.1	1	1	0	0	Amplification	1	0
FGF10	chr5	5p12	1	1	0	0	Amplification	1	0
GHR	chr5	5p13.1-p12	1	1	0	0	Amplification	1	0
HCN1	chr5	5p12	1	1	0	0	Amplification	1	0
HMGCS1	chr5	5p12	1	1	0	0	Amplification	1	0
MRPS30	chr5	5p12	1	1	0	0	Amplification	1	0
NIM1	chr5	5p12	1	1	0	0	Amplification	1	0
NNT	chr5	5p12	1	1	0	0	Amplification	1	0
OXCT1	chr5	5p13.1	1	1	0	0	Amplification	1	0
PAIP1	chr5	5p12	1	1	0	0	Amplification	1	0
PLCXD3	chr5	5p13.1	1	1	0	0	Amplification	1	0
SEPP1	chr5	5p12	1	1	0	0	Amplification	1	0
ZNF131	chr5	5p12	1	1	0	0	Amplification	1	0
ABCC10	chr6	6p21.1	1	1	0	0	Amplification	1	0
ADCY10P1	chr6	6p21.1	1	1	0	0	Amplification	1	0
ADGB	chr6	6q24.3	1	0	0	1	Amplification	1	0
APOBEC2	chr6	6p21.1	1	1	0	0	Amplification	1	0
BAG2	chr6	6p12.1	1	1	0	0	Amplification	1	0
BEND6	chr6	6p12.1	1	1	0	0	Amplification	1	0
BMP5	chr6	6p12.1	1	1	0	0	Amplification	1	0
C6orf223	chr6	6p21.1	1	1	0	0	Amplification	1	0
C6orf226	chr6	6p21.1	1	1	0	0	Amplification	1	0
CLPS	chr6	6p21.31	1	1	0	0	Amplification	1	0
CLPSL1	chr6	6p21.31	1	1	0	0	Amplification	1	0
CNPY3	chr6	6p21.1	1	1	0	0	Amplification	1	0
COL21A1	chr6	6p12.1|6p12.3-p11.2	1	1	0	0	Amplification	1	0
CRIP3	chr6	6p21.1	1	1	0	0	Amplification	1	0
CUL7	chr6	6p21.1	1	1	0	0	Amplification	1	0
CUL9	chr6	6p21.1	1	1	0	0	Amplification	1	0
DLK2	chr6	6p21.1	1	1	0	0	Amplification	1	0
DNPH1	chr6	6p21.1	1	1	0	0	Amplification	1	0
DST	chr6	6p12.1	1	1	0	0	Amplification	1	0
EFHC1	chr6	6p12.2	1	1	0	0	Amplification	1	0
ELOVL5	chr6	6p12.1	1	1	0	0	Amplification	1	0
FAM83B	chr6	6p12.1	1	1	0	0	Amplification	1	0
FBXO9	chr6	6p12.1	1	1	0	0	Amplification	1	0
GCLC	chr6	6p12.1	1	1	0	0	Amplification	1	0

Patel *et al.* , 2021. Whole-exome sequencing analysis of oral squamous cell carcinoma delineated by tobacco usage habits

Supplementary Table 5. List of genes affected by copy number alteration events in OSCC patients

Gene	Chromosome	Cytoband	Recurrence	Recurrence in smoker cohort	Recurrence in chewer cohort	Recurrence in No habit cohort	State	Samples with CNA gain	Samples with CNA loss
GCM1	chr6	6p12.1	1	1	0	0	Amplification	1	0
GFRAL	chr6	6p12.1	1	1	0	0	Amplification	1	0
GLTSCR1L	chr6	6p21.1	1	1	0	0	Amplification	1	0
GNMT	chr6	6p21.1	1	1	0	0	Amplification	1	0
GSTA1	chr6	6p12.2	1	1	0	0	Amplification	1	0
GSTA2	chr6	6p12.2	1	1	0	0	Amplification	1	0
GSTA3	chr6	6p12.2	1	1	0	0	Amplification	1	0
GSTA4	chr6	6p12.2	1	1	0	0	Amplification	1	0
GSTA5	chr6	6p12.2	1	1	0	0	Amplification	1	0
GTPBP2	chr6	6p21.1	1	1	0	0	Amplification	1	0
HCRTR2	chr6	3q13.2	1	1	0	0	Amplification	1	0
HIST1H2AK	chr6	6p22.1	1	0	0	1	Amplification	1	0
HIST1H2BN	chr6	6p22.1	1	0	0	1	Amplification	1	0
HIST1H4K	chr6	6p22.1	1	0	0	1	Amplification	1	0
HMGCLL1	chr6	6p12.1	1	1	0	0	Amplification	1	0
ICK	chr6	6p12.1	1	1	0	0	Amplification	1	0
IL17A	chr6	6p12.2	1	1	0	0	Amplification	1	0
IL17F	chr6	6p12.2	1	1	0	0	Amplification	1	0
KIAA1586	chr6	6p12.1	1	1	0	0	Amplification	1	0
KLC4	chr6	6p21.1	1	1	0	0	Amplification	1	0
KLHDC3	chr6	6p21.1	1	1	0	0	Amplification	1	0
KLHL31	chr6	6p12.1	1	1	0	0	Amplification	1	0
LRFN2	chr6	6p21.2-p21.1	1	1	0	0	Amplification	1	0
LRRC1	chr6	6p12.1	1	1	0	0	Amplification	1	0
LRRC73	chr6	6p21.1	1	1	0	0	Amplification	1	0
MAD2L1BP	chr6	6p21.1	1	1	0	0	Amplification	1	0
MCM3	chr6	6p12.2	1	1	0	0	Amplification	1	0
MEA1	chr6	6p21.1	1	1	0	0	Amplification	1	0
MLIP	chr6	6p12.1	1	1	0	0	Amplification	1	0
MRPL2	chr6	1p32.3	1	1	0	0	Amplification	1	0
MRPS18A	chr6	6p21.1	1	1	0	0	Amplification	1	0
NFYA	chr6	6p21.1	1	1	0	0	Amplification	1	0
OARD1	chr6	6p21.1	1	1	0	0	Amplification	1	0
PAQR8	chr6	6p12.2	1	1	0	0	Amplification	1	0
PEX6	chr6	6p21.1	1	1	0	0	Amplification	1	0
PKHD1	chr6	6p12.3-p12.2	1	1	0	0	Amplification	1	0
POLH	chr6	6p21.1	1	1	0	0	Amplification	1	0
POLR1C	chr6	13q12.2	1	1	0	0	Amplification	1	0
PPP2R5D	chr6	6p21.1	1	1	0	0	Amplification	1	0
PRPH2	chr6	6p21.1	1	1	0	0	Amplification	1	0
PTCRA	chr6	6p21.1	1	1	0	0	Amplification	1	0
PTK7	chr6	6p21.1	1	1	0	0	Amplification	1	0
RAB23	chr6	6p12.1-p11.2	1	1	0	0	Amplification	1	0
RAB32	chr6	6q24.3	1	0	0	1	Amplification	1	0
RPL7L1	chr6	6p21.1	1	1	0	0	Amplification	1	0
RRP36	chr6	6p21.1	1	1	0	0	Amplification	1	0
RSPH9	chr6	6p21.1	1	1	0	0	Amplification	1	0
SLC22A7	chr6	6p21.1	1	1	0	0	Amplification	1	0
SRF	chr6	6p21.1	1	1	0	0	Amplification	1	0
STXBP5	chr6	6q24.3	1	0	0	1	Amplification	1	0
TBCC	chr6	6p21.1	1	1	0	0	Amplification	1	0
TFAP2B	chr6	6p12.3	1	1	0	0	Amplification	1	0
TINAG	chr6	6p12.1	1	1	0	0	Amplification	1	0
TJAP1	chr6	6p21.1	1	1	0	0	Amplification	1	0
TMEM14A	chr6	6p12.2	1	1	0	0	Amplification	1	0
TRAM2	chr6	6p12.2	1	1	0	0	Amplification	1	0

Patel *et al.* , 2021. Whole-exome sequencing analysis of oral squamous cell carcinoma delineated by tobacco usage habits

Supplementary Table 5. List of genes affected by copy number alteration events in OSCC patients

Gene	Chromosome	Cytoband	Recurrence	Recurrence in smoker cohort	Recurrence in chewer cohort	Recurrence in No habit cohort	State	Samples with CNA gain	Samples with CNA loss
TSPO2	chr6	6p21.1	1	1	0	0	Amplification	1	0
TTBK1	chr6	6p21.1	1	1	0	0	Amplification	1	0
UBR2	chr6	6p21.1	1	1	0	0	Amplification	1	0
UNC5CL	chr6	6p21.1	1	1	0	0	Amplification	1	0
VEGFA	chr6	6p21.1	1	1	0	0	Amplification	1	0
XPO5	chr6	6p21.1	1	1	0	0	Amplification	1	0
YIPF3	chr6	6p21.1	1	1	0	0	Amplification	1	0
ZNF318	chr6	6p21.1	1	1	0	0	Amplification	1	0
ZNF451	chr6	6p12.1	1	1	0	0	Amplification	1	0
ABCA13	chr7	7p12.3	1	1	0	0	Amplification	1	0
AC002467	chr7	0	1	1	0	0	Amplification	1	0
AC004899	chr7	0	1	1	0	0	Amplification	1	0
AC005008	chr7	0	1	1	0	0	Amplification	1	0
AC005076	chr7	0	1	1	0	0	Amplification	1	0
AC011294	chr7	0	1	1	0	0	Amplification	1	0
AC068533	chr7	0	1	1	0	0	Amplification	1	0
ACHE	chr7	7q22.1	1	1	0	0	Amplification	1	0
ACN9	chr7	7q21.3	1	1	0	0	Amplification	1	0
ACTL6B	chr7	7q22.1	1	1	0	0	Amplification	1	0
ADCY1	chr7	7p12.3	1	1	0	0	Amplification	1	0
AEBP1	chr7	7p13	1	1	0	0	Amplification	1	0
AGFG2	chr7	7q22.1	1	1	0	0	Amplification	1	0
AIMP2	chr7	7p22.1	1	1	0	0	Amplification	1	0
AKAP9	chr7	7q21.2	1	1	0	0	Amplification	1	0
ALKBH4	chr7	7q22.1	1	1	0	0	Amplification	1	0
ANKIB1	chr7	7q21.2	1	1	0	0	Amplification	1	0
ANKRD61	chr7	7p22.1	1	1	0	0	Amplification	1	0
AP1S1	chr7	7q22.1	1	1	0	0	Amplification	1	0
AP4M1	chr7	7q22.1	1	1	0	0	Amplification	1	0
ARMC10	chr7	7q22.1	1	1	0	0	Amplification	1	0
ARPC1A	chr7	7q22.1	1	1	0	0	Amplification	1	0
ARPC1B	chr7	7q22.1	1	1	0	0	Amplification	1	0
ASB4	chr7	7q21.3	1	1	0	0	Amplification	1	0
ASL	chr7	7q11.21	1	1	0	0	Amplification	1	0
ASNS	chr7	7q21.3	1	1	0	0	Amplification	1	0
ATP5J2	chr7	7q22.1	1	1	0	0	Amplification	1	0
ATXN7L1	chr7	7q22.3	1	1	0	0	Amplification	1	0
AZGP1	chr7	7q22.1	1	1	0	0	Amplification	1	0
AZGP1P1	chr7	7q22.1	1	1	0	0	Amplification	1	0
BAIAP2L1	chr7	7q21.3-q22.1	1	1	0	0	Amplification	1	0
BCAP29	chr7	7q22.3	1	1	0	0	Amplification	1	0
BET1	chr7	7q21.3	1	1	0	0	Amplification	1	0
BHLHA15	chr7	7q21.3	1	1	0	0	Amplification	1	0
BLVRA	chr7	7p13	1	1	0	0	Amplification	1	0
BRI3	chr7	2q37.1	1	1	0	0	Amplification	1	0
BUD31	chr7	7q22.1	1	1	0	0	Amplification	1	0
C7orf43	chr7	7q22.1	1	1	0	0	Amplification	1	0
C7orf57	chr7	7p12.3	1	1	0	0	Amplification	1	0
C7orf61	chr7	7q22.1	1	1	0	0	Amplification	1	0
C7orf62	chr7	7q21.13	1	1	0	0	Amplification	1	0
C7orf63	chr7	7q21.13	1	1	0	0	Amplification	1	0
C7orf65	chr7	7p12.3	1	1	0	0	Amplification	1	0
C7orf69	chr7	7p12.3	1	1	0	0	Amplification	1	0
C7orf72	chr7	7p12.2	1	1	0	0	Amplification	1	0
C7orf76	chr7	7q21.3	1	1	0	0	Amplification	1	0
CACNA2D1	chr7	7q21.11	1	1	0	0	Amplification	1	0
CALCR	chr7	7q21.3	1	1	0	0	Amplification	1	0
CAMK2B	chr7	7p13	1	1	0	0	Amplification	1	0

Patel *et al.* , 2021. Whole-exome sequencing analysis of oral squamous cell carcinoma delineated by tobacco usage habits

Supplementary Table 5. List of genes affected by copy number alteration events in OSCC patients

Gene	Chromosome	Cytoband	Recurrence	Recurrence in smoker cohort	Recurrence in chewer cohort	Recurrence in No habit cohort	State	Samples with CNA gain	Samples with CNA loss
CASD1	chr7	7q21.3	1	1	0	0	Amplification	1	0
CBLL1	chr7	7q22.3	1	1	0	0	Amplification	1	0
CCDC132	chr7	7q21.2-q21.3	1	1	0	0	Amplification	1	0
CCDC71L	chr7	7q22.3	1	1	0	0	Amplification	1	0
CCM2	chr7	7p13	1	1	0	0	Amplification	1	0
CCT6P1	chr7	7q11.21	1	1	0	0	Amplification	1	0
CCT6P3	chr7	7q11.21	1	1	0	0	Amplification	1	0
CDHR3	chr7	7q22.3	1	1	0	0	Amplification	1	0
CDK14	chr7	7q21.13	1	1	0	0	Amplification	1	0
CDK6	chr7	7q21.2	1	1	0	0	Amplification	1	0
CLDN15	chr7	7q22.1	1	1	0	0	Amplification	1	0
CNPY4	chr7	7q22.1	1	1	0	0	Amplification	1	0
COA1	chr7	7p13	1	1	0	0	Amplification	1	0
COBL	chr7	7p12.1	1	1	0	0	Amplification	1	0
COG5	chr7	7q22.3	1	1	0	0	Amplification	1	0
COL1A2	chr7	7q21.3	1	1	0	0	Amplification	1	0
COL26A1	chr7	7q22.1	1	1	0	0	Amplification	1	0
COPS6	chr7	7q22.1	1	1	0	0	Amplification	1	0
CPSF4	chr7	7q22.1	1	1	0	0	Amplification	1	0
CRCP	chr7	7q11.21	1	1	0	0	Amplification	1	0
CUX1	chr7	7q22.1	1	1	0	0	Amplification	1	0
CYP3A4	chr7	7q22.1	1	1	0	0	Amplification	1	0
CYP3A43	chr7	7q22.1	1	1	0	0	Amplification	1	0
CYP3A5	chr7	7q22.1	1	1	0	0	Amplification	1	0
CYP3A7	chr7	7q22.1	1	1	0	0	Amplification	1	0
CYP51A1	chr7	7q21.2	1	1	0	0	Amplification	1	0
DBNL	chr7	7p13	1	1	0	0	Amplification	1	0
DDC	chr7	7p12.2-p12.1	1	1	0	0	Amplification	1	0
DDX56	chr7	7p13	1	1	0	0	Amplification	1	0
DLD	chr7	16q23.1	1	1	0	0	Amplification	1	0
DLX5	chr7	7q21.3	1	1	0	0	Amplification	1	0
DLX6	chr7	7q21.3	1	1	0	0	Amplification	1	0
DMTF1	chr7	7q21.12	1	1	0	0	Amplification	1	0
DNAJC2	chr7	7q22.1	1	1	0	0	Amplification	1	0
DPY19L2P2	chr7	7q22.1	1	1	0	0	Amplification	1	0
DUS4L	chr7	7q22.3	1	1	0	0	Amplification	1	0
DYNC1I1	chr7	7q21.3	1	1	0	0	Amplification	1	0
EFCAB10	chr7	7q22.3	1	1	0	0	Amplification	1	0
EIF2AK1	chr7	7p22.1	1	1	0	0	Amplification	1	0
EPHB4	chr7	7q22.1	1	1	0	0	Amplification	1	0
EPO	chr7	17q22	1	1	0	0	Amplification	1	0
ERV3	chr7	7q11.21	1	1	0	0	Amplification	1	0
FAM115A	chr7	7q35	1	0	1	0	Amplification	1	0
FAM115C	chr7	7q35	1	0	1	0	Amplification	1	0
FAM133B	chr7	7q21.2	1	1	0	0	Amplification	1	0
FAM185A	chr7	7q22.1	1	1	0	0	Amplification	1	0
FAM200A	chr7	7q22.1	1	1	0	0	Amplification	1	0
FBXL13	chr7	7q22.1	1	1	0	0	Amplification	1	0
FBXO24	chr7	7q22.1	1	1	0	0	Amplification	1	0
FIS1	chr7	7q22.1	1	1	0	0	Amplification	1	0
FZD1	chr7	7q21.13	1	1	0	0	Amplification	1	0
GAL3ST4	chr7	7q22.1	1	1	0	0	Amplification	1	0
GATAD1	chr7	7q21.2	1	1	0	0	Amplification	1	0
GATS	chr7	7q22.1	1	1	0	0	Amplification	1	0
GCK	chr7	11q13.1	1	1	0	0	Amplification	1	0
GJC3	chr7	7q22.1	1	1	0	0	Amplification	1	0
GNG11	chr7	7q21.3	1	1	0	0	Amplification	1	0

Patel *et al.* , 2021. Whole-exome sequencing analysis of oral squamous cell carcinoma delineated by tobacco usage habits

Supplementary Table 5. List of genes affected by copy number alteration events in OSCC patients

**Gene**	**Chromosome**	**Cytoband**	**Recurrence**	**Recurrence in smoker cohort**	**Recurrence in chewer cohort**	**Recurrence in No habit cohort**	**State**	**Samples with CNA gain**	**Samples with CNA loss**
GNGT1	chr7	7q21.3	1	1	0	0	Amplification	1	0
GPC2	chr7	7q22.1	1	1	0	0	Amplification	1	0
GRB10	chr7	7p12.1	1	1	0	0	Amplification	1	0
GRM3	chr7	7q21.11-q21.12	1	1	0	0	Amplification	1	0
GS1	chr7	Xp22.31	1	1	0	0	Amplification	1	0
GTPBP10	chr7	7q21.13	1	1	0	0	Amplification	1	0
GUSB	chr7	7q11.21	1	1	0	0	Amplification	1	0
H2AFV	chr7	7p13	1	1	0	0	Amplification	1	0
HBP1	chr7	7q22.3	1	1	0	0	Amplification	1	0
HECW1	chr7	7p14.1-p13	1	1	0	0	Amplification	1	0
HEPACAM2	chr7	7q21.2	1	1	0	0	Amplification	1	0
HGF	chr7	2p22.1	1	1	0	0	Amplification	1	0
HUS1	chr7	7p12.3	1	1	0	0	Amplification	1	0
IGFBP1	chr7	7p12.3	1	1	0	0	Amplification	1	0
IGFBP3	chr7	7p12.3	1	1	0	0	Amplification	1	0
IKZF1	chr7	7p12.2	1	1	0	0	Amplification	1	0
INTS4L1	chr7	7q11.21	1	1	0	0	Amplification	1	0
KIAA1324L	chr7	7q21.12	1	1	0	0	Amplification	1	0
KMT2E	chr7	7q22.3	1	1	0	0	Amplification	1	0
KPNA7	chr7	7q22.1	1	1	0	0	Amplification	1	0
KRIT1	chr7	7q21.2	1	1	0	0	Amplification	1	0
LAMB1	chr7	7q31.1	1	1	0	0	Amplification	1	0
LAMB4	chr7	7q31.1	1	1	0	0	Amplification	1	0
LAMTOR4	chr7	7q22.1	1	1	0	0	Amplification	1	0
LHFPL3	chr7	7q22.2-q22.3	1	1	0	0	Amplification	1	0
LMTK2	chr7	7q21.3	1	1	0	0	Amplification	1	0
LRCH4	chr7	7q22.1	1	1	0	0	Amplification	1	0
LRRC17	chr7	7q22.1	1	1	0	0	Amplification	1	0
LRRD1	chr7	7q21.2	1	1	0	0	Amplification	1	0
LRWD1	chr7	7q22.1	1	1	0	0	Amplification	1	0
MBLAC1	chr7	7q22.1	1	1	0	0	Amplification	1	0
MCM7	chr7	7q22.1	1	1	0	0	Amplification	1	0
MEPCE	chr7	7q22.1	1	1	0	0	Amplification	1	0
MOGAT3	chr7	7q22.1	1	1	0	0	Amplification	1	0
MOSPD3	chr7	7q22.1	1	1	0	0	Amplification	1	0
MRPL32	chr7	7p14.1	1	1	0	0	Amplification	1	0
MRPS24	chr7	7p13	1	1	0	0	Amplification	1	0
MTERF	chr7	7q21.2	1	1	0	0	Amplification	1	0
MUC12	chr7	7q22.1	1	1	0	0	Amplification	1	0
MUC3A	chr7	7q22	1	1	0	0	Amplification	1	0
MYH16	chr7	7q22.1	1	1	0	0	Amplification	1	0
MYL10	chr7	7q22.1	1	1	0	0	Amplification	1	0
MYL7	chr7	7p13	1	1	0	0	Amplification	1	0
MYO1G	chr7	7p13	1	1	0	0	Amplification	1	0
NACAD	chr7	7p13	1	1	0	0	Amplification	1	0
NAMPT	chr7	7q22.3	1	1	0	0	Amplification	1	0
NAPEPLD	chr7	7q22.1	1	1	0	0	Amplification	1	0
NAT16	chr7	7q22.1	1	1	0	0	Amplification	1	0
NPC1L1	chr7	7p13	1	1	0	0	Amplification	1	0
NPTX2	chr7	7q22.1	1	1	0	0	Amplification	1	0
NRCAM	chr7	7q31.1	1	1	0	0	Amplification	1	0
NUDCD3	chr7	7p13	1	1	0	0	Amplification	1	0
NYAP1	chr7	7q22.1	1	1	0	0	Amplification	1	0
OCM2	chr7	7q21.3	1	1	0	0	Amplification	1	0
OGDH	chr7	7p13	1	1	0	0	Amplification	1	0
OR2AE1	chr7	7q22.1	1	1	0	0	Amplification	1	0
ORAI2	chr7	7q22.1	1	1	0	0	Amplification	1	0

Patel *et al.* , 2021. Whole-exome sequencing analysis of oral squamous cell carcinoma delineated by tobacco usage habits

Supplementary Table 5. List of genes affected by copy number alteration events in OSCC patients

**Gene**	**Chromosome**	**Cytoband**	**Recurrence**	**Recurrence in smoker cohort**	**Recurrence in chewer cohort**	**Recurrence in No habit cohort**	**State**	**Samples with CNA gain**	**Samples with CNA loss**
ORC5	chr7	7q22.1-q22.2	1	1	0	0	Amplification	1	0
PCLO	chr7	7q21.11	1	1	0	0	Amplification	1	0
PCOLCE	chr7	7q22.1	1	1	0	0	Amplification	1	0
PDAP1	chr7	7q22.1	1	1	0	0	Amplification	1	0
PDK4	chr7	7q21.3	1	1	0	0	Amplification	1	0
PEG10	chr7	7q21.3	1	1	0	0	Amplification	1	0
PEX1	chr7	19q13.12	1	1	0	0	Amplification	1	0
PIK3CG	chr7	7q22.3	1	1	0	0	Amplification	1	0
PILRA	chr7	7q22.1	1	1	0	0	Amplification	1	0
PILRB	chr7	7q22.1	1	1	0	0	Amplification	1	0
PKD1L1	chr7	7p12.3	1	1	0	0	Amplification	1	0
PLOD3	chr7	7q22.1	1	1	0	0	Amplification	1	0
PMPCB	chr7	7q22.1	1	1	0	0	Amplification	1	0
POLD2	chr7	7p13	1	1	0	0	Amplification	1	0
POLM	chr7	7p13	1	1	0	0	Amplification	1	0
POLR2J	chr7	7q22.1	1	1	0	0	Amplification	1	0
POLR2J4	chr7	7p13	1	1	0	0	Amplification	1	0
POM121L12	chr7	7p12.1	1	1	0	0	Amplification	1	0
PON1	chr7	7q21.3	1	1	0	0	Amplification	1	0
PON2	chr7	7q21.3	1	1	0	0	Amplification	1	0
PON3	chr7	7q21.3	1	1	0	0	Amplification	1	0
POP7	chr7	7q22.1	1	1	0	0	Amplification	1	0
PPIA	chr7	7p13	1	1	0	0	Amplification	1	0
PPP1R35	chr7	7q22.1	1	1	0	0	Amplification	1	0
PPP1R9A	chr7	7q21.3	1	1	0	0	Amplification	1	0
PRKAR2B	chr7	7q22.3	1	1	0	0	Amplification	1	0
PRKRIP1	chr7	7q22.1	1	1	0	0	Amplification	1	0
PSMA2	chr7	7p14.1	1	1	0	0	Amplification	1	0
PSMC2	chr7	7q22.1	1	1	0	0	Amplification	1	0
PTCD1	chr7	7q22.1	1	1	0	0	Amplification	1	0
PURB	chr7	7p13	1	1	0	0	Amplification	1	0
PUS7	chr7	7q22.3	1	1	0	0	Amplification	1	0
PVRIG	chr7	7q22.1	1	1	0	0	Amplification	1	0
RABL5	chr7	7q22.1	1	1	0	0	Amplification	1	0
RAMP3	chr7	7p13	1	1	0	0	Amplification	1	0
RASA4CP	chr7	7p13	1	1	0	0	Amplification	1	0
RBM48	chr7	7q21.2	1	1	0	0	Amplification	1	0
RELN	chr7	7q22.1	1	1	0	0	Amplification	1	0
RINT1	chr7	7q22.3	1	1	0	0	Amplification	1	0
SAMD9	chr7	7q21.2	1	1	0	0	Amplification	1	0
SAMD9L	chr7	7q21.2	1	1	0	0	Amplification	1	0
SAP25	chr7	7q22.1	1	1	0	0	Amplification	1	0
SEMA3A	chr7	7q21.11	1	1	0	0	Amplification	1	0
SEMA3C	chr7	7q21.11	1	1	0	0	Amplification	1	0
SEMA3D	chr7	7q21.11	1	1	0	0	Amplification	1	0
SEMA3E	chr7	7q21.11	1	1	0	0	Amplification	1	0
SEPT7P2	chr7	7p12.3	1	1	0	0	Amplification	1	0
SERPINE1	chr7	7q22.1	1	1	0	0	Amplification	1	0
SGCE	chr7	7q21.3	1	1	0	0	Amplification	1	0
SH2B2	chr7	7q22.1	1	1	0	0	Amplification	1	0
SHFM1	chr7	7q21.3	1	1	0	0	Amplification	1	0
SLC12A9	chr7	7q22.1	1	1	0	0	Amplification	1	0
SLC25A13	chr7	7q21.3	1	1	0	0	Amplification	1	0
SLC26A3	chr7	7q22.3-q31.1	1	1	0	0	Amplification	1	0
SLC26A4	chr7	7q22.3	1	1	0	0	Amplification	1	0
SLC26A5	chr7	7q22.1	1	1	0	0	Amplification	1	0
SMURF1	chr7	7q22.1	1	1	0	0	Amplification	1	0

Patel *et al.* , 2021. Whole-exome sequencing analysis of oral squamous cell carcinoma delineated by tobacco usage habits

Supplementary Table 5. List of genes affected by copy number alteration events in OSCC patients

Gene	Chromosome	Cytoband	Recurrence	Recurrence in smoker cohort	Recurrence in chewer cohort	Recurrence in No habit cohort	State	Samples with CNA gain	Samples with CNA loss
SNHG15	chr7	7p13	1	1	0	0	Amplification	1	0
SPDYE3	chr7	7q22.1	1	1	0	0	Amplification	1	0
SRPK2	chr7	7q22.3	1	1	0	0	Amplification	1	0
SRRT	chr7	7q22.1	1	1	0	0	Amplification	1	0
STAG3	chr7	7q22.1	1	1	0	0	Amplification	1	0
STEAP1	chr7	7q21.13	1	1	0	0	Amplification	1	0
STEAP2	chr7	7q21.13	1	1	0	0	Amplification	1	0
STK17A	chr7	7p13	1	1	0	0	Amplification	1	0
SUN3	chr7	7p12.3	1	1	0	0	Amplification	1	0
SYPL1	chr7	7q22.3	1	1	0	0	Amplification	1	0
TAC1	chr7	7q21.3	1	1	0	0	Amplification	1	0
TAF6	chr7	7q22.1	1	1	0	0	Amplification	1	0
TBRG4	chr7	7p13	1	1	0	0	Amplification	1	0
TECPR1	chr7	7q21.3	1	1	0	0	Amplification	1	0
TFPI2	chr7	7q21.3	1	1	0	0	Amplification	1	0
TFR2	chr7	7q22.1	1	1	0	0	Amplification	1	0
TMED4	chr7	7p13	1	1	0	0	Amplification	1	0
TMEM130	chr7	7q22.1	1	1	0	0	Amplification	1	0
TMEM243	chr7	7q21.12	1	1	0	0	Amplification	1	0
TNS3	chr7	7p12.3	1	1	0	0	Amplification	1	0
TPST1	chr7	7q11.21	1	1	0	0	Amplification	1	0
TRIM4	chr7	7q22.1	1	1	0	0	Amplification	1	0
TRIP6	chr7	7q22.1	1	1	0	0	Amplification	1	0
TRRAP	chr7	7q22.1	1	1	0	0	Amplification	1	0
TSC22D4	chr7	7q22.1	1	1	0	0	Amplification	1	0
TYW1B	chr7	7q11.22-q11.23	1	1	0	0	Amplification	1	0
UBE2D4	chr7	7p13	1	1	0	0	Amplification	1	0
UFSP1	chr7	7q22.1	1	1	0	0	Amplification	1	0
UPP1	chr7	7p12.3	1	1	0	0	Amplification	1	0
URGCP	chr7	7p13	1	1	0	0	Amplification	1	0
VGF	chr7	7q22.1	1	1	0	0	Amplification	1	0
VKORC1L1	chr7	7q11.21	1	1	0	0	Amplification	1	0
VWC2	chr7	7p12.2	1	1	0	0	Amplification	1	0
YKT6	chr7	7p13	1	1	0	0	Amplification	1	0
ZCWPW1	chr7	7q22.1	1	1	0	0	Amplification	1	0
ZKSCAN1	chr7	7q22.1	1	1	0	0	Amplification	1	0
ZKSCAN5	chr7	7q22.1	1	1	0	0	Amplification	1	0
ZMIZ2	chr7	7p13	1	1	0	0	Amplification	1	0
ZNF107	chr7	7q11.21	1	1	0	0	Amplification	1	0
ZNF117	chr7	7q11.21	1	1	0	0	Amplification	1	0
ZNF138	chr7	7q11.21	1	1	0	0	Amplification	1	0
ZNF273	chr7	7q11.21	1	1	0	0	Amplification	1	0
ZNF3	chr7	7q22.1	1	1	0	0	Amplification	1	0
ZNF394	chr7	7q22.1	1	1	0	0	Amplification	1	0
ZNF655	chr7	7q22.1	1	1	0	0	Amplification	1	0
ZNF679	chr7	7q11.21	1	1	0	0	Amplification	1	0
ZNF680	chr7	7q11.21	1	1	0	0	Amplification	1	0
ZNF727	chr7	7q11.21	1	1	0	0	Amplification	1	0
ZNF733P	chr7	7q11.21	1	0	1	0	Amplification	1	0
ZNF736	chr7	7q11.21	1	1	0	0	Amplification	1	0
ZNF789	chr7	7q22.1	1	1	0	0	Amplification	1	0
ZNF804B	chr7	7q21.13	1	1	0	0	Amplification	1	0
ZNHIT1	chr7	7q22.1	1	1	0	0	Amplification	1	0
ZPBP	chr7	7p12.2	1	1	0	0	Amplification	1	0
ZSCAN21	chr7	7q22.1	1	1	0	0	Amplification	1	0
ZSCAN25	chr7	7q22.1	1	1	0	0	Amplification	1	0
AARD	chr8	8q24.11	1	0	1	0	Amplification	1	0
ABRA	chr8	8q23.1	1	0	1	0	Amplification	1	0

Patel *et al.* , 2021. Whole-exome sequencing analysis of oral squamous cell carcinoma delineated by tobacco usage habits

Supplementary Table 5. List of genes affected by copy number alteration events in OSCC patients

**Gene**	**Chromosome**	**Cytoband**	**Recurrence**	**Recurrence in smoker cohort**	**Recurrence in chewer cohort**	**Recurrence in No habit cohort**	**State**	**Samples with CNA gain**	**Samples with CNA loss**
AC023590	chr8	0	1	0	1	0	Amplification	1	0
AC023632	chr8	0	1	0	1	0	Amplification	1	0
AC090186	chr8	0	1	0	1	0	Amplification	1	0
AC138647	chr8	0	1	0	1	0	Amplification	1	0
ADCK5	chr8	8q24.3	1	0	1	0	Amplification	1	0
ADCY8	chr8	8q24.22	1	0	1	0	Amplification	1	0
AF186192	chr8	0	1	0	1	0	Amplification	1	0
AGO2	chr8	8q24.3	1	0	1	0	Amplification	1	0
ANGPT1	chr8	8q23.1	1	0	1	0	Amplification	1	0
ANKRD46	chr8	8q22.3	1	0	1	0	Amplification	1	0
ANXA13	chr8	8q24.13	1	0	1	0	Amplification	1	0
ARC	chr8	8q24.3	1	0	1	0	Amplification	1	0
ARHGAP39	chr8	8q24.3	1	0	1	0	Amplification	1	0
ASAP1	chr8	8q24.21-q24.22	1	0	1	0	Amplification	1	0
ATAD2	chr8	8q24.13	1	0	1	0	Amplification	1	0
ATP6V1C1	chr8	8q22.3	1	0	1	0	Amplification	1	0
ATP6V1H	chr8	8q11.23	1	0	1	0	Amplification	1	0
AZIN1	chr8	8q22.3	1	0	1	0	Amplification	1	0
BAALC	chr8	8q22.3	1	0	1	0	Amplification	1	0
BAI1	chr8	8q24.3	1	0	1	0	Amplification	1	0
BOP1	chr8	8q24.3	1	0	1	0	Amplification	1	0
C8orf17	chr8	8q24.3	1	0	1	0	Amplification	1	0
C8orf22	chr8	8q11.21	1	0	1	0	Amplification	1	0
C8orf31	chr8	8q24.3	1	0	1	0	Amplification	1	0
C8orf33	chr8	8q24.3	1	0	1	0	Amplification	1	0
C8orf37	chr8	8q22.1	1	0	1	0	Amplification	1	0
C8orf47	chr8	8q22.2	1	0	1	0	Amplification	1	0
C8orf56	chr8	8q22.3	1	0	1	0	Amplification	1	0
C8orf76	chr8	8q24.13	1	0	1	0	Amplification	1	0
C8orf82	chr8	8q24.3	1	0	1	0	Amplification	1	0
C8orf87	chr8	8q22.1	1	0	1	0	Amplification	1	0
CCDC166	chr8	8q24.3	1	0	1	0	Amplification	1	0
CCDC26	chr8	8q24.21	1	0	1	0	Amplification	1	0
CCNE2	chr8	8q22.1	1	0	1	0	Amplification	1	0
CDH17	chr8	8q22.1	1	0	1	0	Amplification	1	0
CEBPD	chr8	8q11.21	1	0	1	0	Amplification	1	0
CHRAC1	chr8	8q24.3	1	0	1	0	Amplification	1	0
COL14A1	chr8	8q24.12	1	0	1	0	Amplification	1	0
COL22A1	chr8	8q24.23-q24.3	1	0	1	0	Amplification	1	0
COLEC10	chr8	8q24.12	1	0	1	0	Amplification	1	0
COMMD5	chr8	8q24.3	1	0	1	0	Amplification	1	0
COX6C	chr8	8q22.2	1	0	1	0	Amplification	1	0
CPQ	chr8	8q22.1	1	0	1	0	Amplification	1	0
CPSF1	chr8	8q24.3	1	0	1	0	Amplification	1	0
CSMD3	chr8	8q23.3	1	0	1	0	Amplification	1	0
CTHRC1	chr8	8q22.3	1	0	1	0	Amplification	1	0
CYC1	chr8	4q21.1	1	0	1	0	Amplification	1	0
CYHR1	chr8	8q24.3	1	0	1	0	Amplification	1	0
CYP11B1	chr8	8q24.3	1	0	1	0	Amplification	1	0
CYP11B2	chr8	8q24.3	1	0	1	0	Amplification	1	0
DCAF13	chr8	8q22.3	1	0	1	0	Amplification	1	0
DCSTAMP	chr8	8q22.3	1	0	1	0	Amplification	1	0
DEFB4B	chr8	8p23.1	1	0	1	0	Amplification	1	0
DENND3	chr8	8q24.3	1	0	1	0	Amplification	1	0
DEPTOR	chr8	8q24.12	1	0	1	0	Amplification	1	0
DERL1	chr8	8q24.13	1	0	1	0	Amplification	1	0
DGAT1	chr8	8q24.3	1	0	1	0	Amplification	1	0

Patel *et al.* , 2021. Whole-exome sequencing analysis of oral squamous cell carcinoma delineated by tobacco usage habits

Supplementary Table 5. List of genes affected by copy number alteration events in OSCC patients

Gene	Chromosome	Cytoband	Recurrence	Recurrence in smoker cohort	Recurrence in chewer cohort	Recurrence in No habit cohort	State	Samples with CNA gain	Samples with CNA loss
DPY19L4	chr8	8q22.1	1	0	1	0	Amplification	1	0
DPYS	chr8	8q22.3	1	0	1	0	Amplification	1	0
DSCC1	chr8	8q24.12	1	0	1	0	Amplification	1	0
EBAG9	chr8	8q23.2	1	0	1	0	Amplification	1	0
EEF1D	chr8	8q24.3	1	0	1	0	Amplification	1	0
EFCAB1	chr8	8q11.21	1	0	1	0	Amplification	1	0
EFR3A	chr8	8q24.22	1	0	1	0	Amplification	1	0
EIF3E	chr8	8q23.1	1	0	1	0	Amplification	1	0
EIF3H	chr8	8q23.3-q24.11	1	0	1	0	Amplification	1	0
EMC2	chr8	8q23.1	1	0	1	0	Amplification	1	0
ENPP2	chr8	8q24.12	1	0	1	0	Amplification	1	0
ENY2	chr8	8q23.1	1	0	1	0	Amplification	1	0
EPPK1	chr8	8q24.3	1	0	1	0	Amplification	1	0
ESRP1	chr8	8q22.1	1	0	1	0	Amplification	1	0
EXOSC4	chr8	8q24.3	1	0	1	0	Amplification	1	0
EXT1	chr8	8q24.11	1	0	1	0	Amplification	1	0
FAM135B	chr8	8q24.23	1	0	1	0	Amplification	1	0
FAM150A	chr8	8q11.23	1	0	1	0	Amplification	1	0
FAM203A	chr8	8q24.3	1	0	1	0	Amplification	1	0
FAM203B	chr8	8q24.3	1	0	1	0	Amplification	1	0
FAM49B	chr8	8q24.21	1	0	1	0	Amplification	1	0
FAM83A	chr8	8q24.13	1	0	1	0	Amplification	1	0
FAM83H	chr8	8q24.3	1	0	1	0	Amplification	1	0
FAM84B	chr8	8q24.21	1	0	1	0	Amplification	1	0
FAM91A1	chr8	8q24.13	1	0	1	0	Amplification	1	0
FAM92A1	chr8	8q22.1	1	0	1	0	Amplification	1	0
FBXL6	chr8	8q24.3	1	0	1	0	Amplification	1	0
FBXO32	chr8	8q24.13	1	0	1	0	Amplification	1	0
FBXO43	chr8	8q22.2	1	0	1	0	Amplification	1	0
FER1L6	chr8	8q24.13	1	0	1	0	Amplification	1	0
FOXH1	chr8	8q24.3	1	0	1	0	Amplification	1	0
FSBP	chr8	8q22.1	1	0	1	0	Amplification	1	0
FZD6	chr8	8q22.3	1	0	1	0	Amplification	1	0
GDF6	chr8	8q22.1	1	0	1	0	Amplification	1	0
GEM	chr8	8q22.1	1	0	1	0	Amplification	1	0
GLI4	chr8	8q24.3	1	0	1	0	Amplification	1	0
GML	chr8	8q24.3	1	0	1	0	Amplification	1	0
GPAA1	chr8	8q24.3	1	0	1	0	Amplification	1	0
GPIHBP1	chr8	8q24.3	1	0	1	0	Amplification	1	0
GPR20	chr8	8q24.3	1	0	1	0	Amplification	1	0
GPT	chr8	8q24.3	1	0	1	0	Amplification	1	0
GRHL2	chr8	8q22.3	1	0	1	0	Amplification	1	0
GRINA	chr8	8q24.3	1	0	1	0	Amplification	1	0
GSDMC	chr8	8q24.21	1	0	1	0	Amplification	1	0
GSDMD	chr8	8q24.3	1	0	1	0	Amplification	1	0
HAS2	chr8	8q24.13	1	0	1	0	Amplification	1	0
HHLA1	chr8	8q24.22	1	0	1	0	Amplification	1	0
HRSP12	chr8	8q22.2	1	0	1	0	Amplification	1	0
HSF1	chr8	8q24.3	1	0	1	0	Amplification	1	0
INTS8	chr8	8q22.1	1	0	1	0	Amplification	1	0
JRK	chr8	8q24.3	1	0	1	0	Amplification	1	0
KB	chr8	0	1	0	1	0	Amplification	1	0
KCNK9	chr8	8q24.3	1	0	1	0	Amplification	1	0
KCNQ3	chr8	8q24.22	1	0	1	0	Amplification	1	0
KCNS2	chr8	8q22.2	1	0	1	0	Amplification	1	0
KCNV1	chr8	8q23.2	1	0	1	0	Amplification	1	0
KHDRBS3	chr8	8q24.23	1	0	1	0	Amplification	1	0
KIAA0196	chr8	8q24.13	1	0	1	0	Amplification	1	0

Patel *et al.* , 2021. Whole-exome sequencing analysis of oral squamous cell carcinoma delineated by tobacco usage habits

Supplementary Table 5. List of genes affected by copy number alteration events in OSCC patients

Gene	Chromosome	Cytoband	Recurrence	Recurrence in smoker cohort	Recurrence in chewer cohort	Recurrence in No habit cohort	State	Samples with CNA gain	Samples with CNA loss
KIAA1429	chr8	8q22.1	1	0	1	0	Amplification	1	0
KIAA1875	chr8	8q24.3	1	0	1	0	Amplification	1	0
KIFC2	chr8	8q24.3	1	0	1	0	Amplification	1	0
KLF10	chr8	8q22.3	1	0	1	0	Amplification	1	0
KLHL38	chr8	8q24.13	1	0	1	0	Amplification	1	0
KM	chr8	0	1	0	1	0	Amplification	1	0
LAPTM4B	chr8	8q22.1	1	0	1	0	Amplification	1	0
LINC00293	chr8	8q11.1	1	0	1	0	Amplification	1	0
LINC00964	chr8	8q24.13	1	0	1	0	Amplification	1	0
LRP12	chr8	8q22.3	1	0	1	0	Amplification	1	0
LRRC14	chr8	8q24.3	1	0	1	0	Amplification	1	0
LRRC6	chr8	8q24.22	1	0	1	0	Amplification	1	0
LRRC69	chr8	8q21.3	1	0	1	0	Amplification	1	0
LY6D	chr8	8q24.3	1	0	1	0	Amplification	1	0
LY6E	chr8	8q24.3	1	0	1	0	Amplification	1	0
LY6H	chr8	8q24.3	1	0	1	0	Amplification	1	0
LY6K	chr8	8q24.3	1	0	1	0	Amplification	1	0
LYNX1	chr8	8q24.3	1	0	1	0	Amplification	1	0
LYPD2	chr8	8q24.3	1	0	1	0	Amplification	1	0
LYPLA1	chr8	8q11.23	1	0	1	0	Amplification	1	0
MAF1	chr8	8q24.3	1	0	1	0	Amplification	1	0
MAFA	chr8	8q24.3	1	0	1	0	Amplification	1	0
MAL2	chr8	8q24.12	1	0	1	0	Amplification	1	0
MAPK15	chr8	-	1	0	1	0	Amplification	1	0
MATN2	chr8	8q22.1-q22.2	1	0	1	0	Amplification	1	0
MCM4	chr8	8q11.21	1	0	1	0	Amplification	1	0
MED30	chr8	8q24.11	1	0	1	0	Amplification	1	0
MFSD3	chr8	8q24.3	1	0	1	0	Amplification	1	0
MIR378D2	chr8	8q22.1	1	0	1	0	Amplification	1	0
MROH1	chr8	8q24.3	1	0	1	0	Amplification	1	0
MROH5	chr8	8q24.3	1	0	1	0	Amplification	1	0
MROH6	chr8	8q24.3	1	0	1	0	Amplification	1	0
MRPL13	chr8	8q24.12	1	0	1	0	Amplification	1	0
MRPL15	chr8	8q11.23	1	0	1	0	Amplification	1	0
MTBP	chr8	8q24.12	1	0	1	0	Amplification	1	0
MTDH	chr8	8q22.1	1	0	1	0	Amplification	1	0
MTERFD1	chr8	8q22.1	1	0	1	0	Amplification	1	0
MTSS1	chr8	8q24.13	1	0	1	0	Amplification	1	0
MYC	chr8	8q24.21	1	0	1	0	Amplification	1	0
NACAP1	chr8	8q22.3	1	0	1	0	Amplification	1	0
NAPRT1	chr8	8q24.3	1	0	1	0	Amplification	1	0
NCALD	chr8	8q22.3	1	0	1	0	Amplification	1	0
NDRG1	chr8	8q24.22	1	0	1	0	Amplification	1	0
NDUFAF6	chr8	8q22.1	1	0	1	0	Amplification	1	0
NDUFB9	chr8	8q24.13	1	0	1	0	Amplification	1	0
NECAB1	chr8	8q21.3	1	0	1	0	Amplification	1	0
NIPAL2	chr8	8q22.2	1	0	1	0	Amplification	1	0
NOV	chr8	Xq28	1	0	1	0	Amplification	1	0
NPBWR1	chr8	8q11.23	1	0	1	0	Amplification	1	0
NRBP2	chr8	8q24.3	1	0	1	0	Amplification	1	0
NSMCE2	chr8	8q24.13	1	0	1	0	Amplification	1	0
NUDCD1	chr8	8q23.1	1	0	1	0	Amplification	1	0
OC90	chr8	8q24.22	1	0	1	0	Amplification	1	0
ODF1	chr8	8q22.3	1	0	1	0	Amplification	1	0
OPLAH	chr8	8q24.3	1	0	1	0	Amplification	1	0
OPRK1	chr8	8q11.23	1	0	1	0	Amplification	1	0
OSR2	chr8	8q22.2	1	0	1	0	Amplification	1	0
OTUD6B	chr8	8q21.3	1	0	1	0	Amplification	1	0

Patel *et al.* , 2021. Whole-exome sequencing analysis of oral squamous cell carcinoma delineated by tobacco usage habits

Supplementary Table 5. List of genes affected by copy number alteration events in OSCC patients

Gene	Chromosome	Cytoband	Recurrence	Recurrence in smoker cohort	Recurrence in chewer cohort	Recurrence in No habit cohort	State	Samples with CNA gain	Samples with CNA loss
OXR1	chr8	8q23.1	1	0	1	0	Amplification	1	0
PABPC1	chr8	8q22.3	1	0	1	0	Amplification	1	0
PARP10	chr8	8q24.3	1	0	1	0	Amplification	1	0
PCMTD1	chr8	8q11.23	1	0	1	0	Amplification	1	0
PDP1	chr8	9p24.1	1	0	1	0	Amplification	1	0
PHF20L1	chr8	8q24.22	1	0	1	0	Amplification	1	0
PKHD1L1	chr8	8q23.1-q23.2	1	0	1	0	Amplification	1	0
PLEC	chr8	8q24.3	1	0	1	0	Amplification	1	0
PLEKHF2	chr8	8q22.1	1	0	1	0	Amplification	1	0
POLR2K	chr8	8q22.2	1	0	1	0	Amplification	1	0
POP1	chr8	16p11.2	1	0	1	0	Amplification	1	0
POU5F1B	chr8	8q24.21	1	0	1	0	Amplification	1	0
PPP1R16A	chr8	8q24.3	1	0	1	0	Amplification	1	0
PRKDC	chr8	8q11.21	1	0	1	0	Amplification	1	0
PSCA	chr8	8q24.3	1	0	1	0	Amplification	1	0
PTDSS1	chr8	8q22.1	1	0	1	0	Amplification	1	0
PTK2	chr8	8q24.3	1	0	1	0	Amplification	1	0
PTP4A3	chr8	8q24.3	1	0	1	0	Amplification	1	0
PUF60	chr8	8q24.3	1	0	1	0	Amplification	1	0
PVT1	chr8	8q24.21	1	0	1	0	Amplification	1	0
PXDNL	chr8	8q11.22-q11.23	1	0	1	0	Amplification	1	0
PYCRL	chr8	8q24.3	1	0	1	0	Amplification	1	0
RAD21	chr8	8q24.11	1	0	1	0	Amplification	1	0
RAD54B	chr8	8q22.1	1	0	1	0	Amplification	1	0
RB1CC1	chr8	8q11.23	1	0	1	0	Amplification	1	0
RBM12B	chr8	8q22.1	1	0	1	0	Amplification	1	0
RECQL4	chr8	8q24.3	1	0	1	0	Amplification	1	0
RGS20	chr8	8q11.23	1	0	1	0	Amplification	1	0
RGS22	chr8	8q22.2	1	0	1	0	Amplification	1	0
RHPN1	chr8	8q24.3	1	0	1	0	Amplification	1	0
RIMS2	chr8	8q22.3	1	0	1	0	Amplification	1	0
RNF139	chr8	8q24.13	1	0	1	0	Amplification	1	0
RNF19A	chr8	8q22.2	1	0	1	0	Amplification	1	0
RPL30	chr8	8q22.2	1	0	1	0	Amplification	1	0
RPL8	chr8	8q24.3	1	0	1	0	Amplification	1	0
RRM2B	chr8	8q22.3	1	0	1	0	Amplification	1	0
RSPO2	chr8	8q23.1	1	0	1	0	Amplification	1	0
RUNX1T1	chr8	8q21.3	1	0	1	0	Amplification	1	0
SAMD12	chr8	8q24.11-q24.12	1	0	1	0	Amplification	1	0
SCRIB	chr8	8q24.3	1	0	1	0	Amplification	1	0
SCRT1	chr8	8q24.3	1	0	1	0	Amplification	1	0
SCXA	chr8	8q24.3	1	0	1	0	Amplification	1	0
SCXB	chr8	8q24.3	1	0	1	0	Amplification	1	0
SDC2	chr8	8q22.1	1	0	1	0	Amplification	1	0
SHARPIN	chr8	8q24.3	1	0	1	0	Amplification	1	0
SLA	chr8	4p15.2	1	0	1	0	Amplification	1	0
SLC25A32	chr8	8q22.3	1	0	1	0	Amplification	1	0
SLC26A7	chr8	8q21.3	1	0	1	0	Amplification	1	0
SLC30A8	chr8	8q24.11	1	0	1	0	Amplification	1	0
SLC39A4	chr8	8q24.3	1	0	1	0	Amplification	1	0
SLC45A4	chr8	8q24.3	1	0	1	0	Amplification	1	0
SLC52A2	chr8	8q24.3	1	0	1	0	Amplification	1	0
SLURP1	chr8	8q24.3	1	0	1	0	Amplification	1	0
SNAI2	chr8	8q11.21	1	0	1	0	Amplification	1	0
SNTB1	chr8	8q24.12	1	0	1	0	Amplification	1	0
SNTG1	chr8	8q11.21	1	0	1	0	Amplification	1	0

Patel *et al.* , 2021. Whole-exome sequencing analysis of oral squamous cell carcinoma delineated by tobacco usage habits

Supplementary Table 5. List of genes affected by copy number alteration events in OSCC patients

Gene	Chromosome	Cytoband	Recurrence	Recurrence in smoker cohort	Recurrence in chewer cohort	Recurrence in No habit cohort	State	Samples with CNA gain	Samples with CNA loss
SNX31	chr8	8q22.3	1	0	1	0	Amplification	1	0
SPAG1	chr8	8q22.2	1	0	1	0	Amplification	1	0
SPATC1	chr8	8q24.3	1	0	1	0	Amplification	1	0
SPIDR	chr8	8q11.21	1	0	1	0	Amplification	1	0
SQLE	chr8	8q24.13	1	0	1	0	Amplification	1	0
ST18	chr8	8q11.23	1	0	1	0	Amplification	1	0
ST3GAL1	chr8	8q24.22	1	0	1	0	Amplification	1	0
STK3	chr8	13q32.2	1	0	1	0	Amplification	1	0
SYBU	chr8	8q23.2	1	0	1	0	Amplification	1	0
TAF2	chr8	8q24.12	1	0	1	0	Amplification	1	0
TATDN1	chr8	8q24.13	1	0	1	0	Amplification	1	0
TBC1D31	chr8	8q24.13	1	0	1	0	Amplification	1	0
TCEA1	chr8	8q11.23	1	0	1	0	Amplification	1	0
TG	chr8	8q24.22	1	0	1	0	Amplification	1	0
THEM6	chr8	8q24.3	1	0	1	0	Amplification	1	0
TIGD5	chr8	8q24.3	1	0	1	0	Amplification	1	0
TMEM249	chr8	8q24.3	1	0	1	0	Amplification	1	0
TMEM55A	chr8	8q21.3	1	0	1	0	Amplification	1	0
TMEM64	chr8	8q21.3	1	0	1	0	Amplification	1	0
TMEM65	chr8	8q24.13	1	0	1	0	Amplification	1	0
TMEM67	chr8	8q22.1	1	0	1	0	Amplification	1	0
TMEM71	chr8	8q24.22	1	0	1	0	Amplification	1	0
TMEM74	chr8	8q23.1	1	0	1	0	Amplification	1	0
TMEM75	chr8	8q24.21	1	0	1	0	Amplification	1	0
TNFRSF11B	chr8	8q24.12	1	0	1	0	Amplification	1	0
TONSL	chr8	8q24.3	1	0	1	0	Amplification	1	0
TOP1MT	chr8	8q24.3	1	0	1	0	Amplification	1	0
TP53INP1	chr8	8q22.1	1	0	1	0	Amplification	1	0
TRAPPC9	chr8	8q24.3	1	0	1	0	Amplification	1	0
TRHR	chr8	8q23.1	1	0	1	0	Amplification	1	0
TRIB1	chr8	8q24.13	1	0	1	0	Amplification	1	0
TRIQK	chr8	8q22.1	1	0	1	0	Amplification	1	0
TRPS1	chr8	8q23.3	1	0	1	0	Amplification	1	0
TSNARE1	chr8	8q24.3	1	0	1	0	Amplification	1	0
TSTA3	chr8	8q24.3	1	0	1	0	Amplification	1	0
UBE2V2	chr8	8q11.21	1	0	1	0	Amplification	1	0
UBR5	chr8	8q22.3	1	0	1	0	Amplification	1	0
UQCRB	chr8	8q22.1	1	0	1	0	Amplification	1	0
UTP23	chr8	8q24.11	1	0	1	0	Amplification	1	0
VPS13B	chr8	8q22.2	1	0	1	0	Amplification	1	0
VPS28	chr8	8q24.3	1	0	1	0	Amplification	1	0
WDYHV1	chr8	8q24.13	1	0	1	0	Amplification	1	0
WISP1	chr8	8q24.22	1	0	1	0	Amplification	1	0
YWHAZ	chr8	8q22.3	1	0	1	0	Amplification	1	0
ZC3H3	chr8	8q24.3	1	0	1	0	Amplification	1	0
ZFAT	chr8	8q24.22	1	0	1	0	Amplification	1	0
ZFP41	chr8	8q24.3	1	0	1	0	Amplification	1	0
ZFPM2	chr8	8q23.1	1	0	1	0	Amplification	1	0
ZHX1	chr8	8q24.13	1	0	1	0	Amplification	1	0
ZHX2	chr8	8q24.13	1	0	1	0	Amplification	1	0
ZNF16	chr8	8q24.3	1	0	1	0	Amplification	1	0
ZNF250	chr8	8q24.3	1	0	1	0	Amplification	1	0
ZNF251	chr8	8q24.3	1	0	1	0	Amplification	1	0
ZNF252P	chr8	8q24.3	1	0	1	0	Amplification	1	0
ZNF34	chr8	8q24.3	1	0	1	0	Amplification	1	0
ZNF517	chr8	8q24.3	1	0	1	0	Amplification	1	0
ZNF572	chr8	8q24.13	1	0	1	0	Amplification	1	0
ZNF623	chr8	8q24.3	1	0	1	0	Amplification	1	0
ZNF696	chr8	8q24.3	1	0	1	0	Amplification	1	0

Patel *et al.* , 2021. Whole-exome sequencing analysis of oral squamous cell carcinoma delineated by tobacco usage habits

Supplementary Table 5. List of genes affected by copy number alteration events in OSCC patients

**Gene**	**Chromosome**	**Cytoband**	**Recurrence**	**Recurrence in smoker cohort**	**Recurrence in chewer cohort**	**Recurrence in No habit cohort**	**State**	**Samples with CNA gain**	**Samples with CNA loss**
ZNF7	chr8	8q24.3	1	0	1	0	Amplification	1	0
ZNF706	chr8	8q22.3	1	0	1	0	Amplification	1	0
ZNF707	chr8	8q24.3	1	0	1	0	Amplification	1	0
AAED1	chr9	9q22.33	1	0	1	0	Amplification	1	0
ACER2	chr9	9p22.1	1	0	0	1	Amplification	1	0
ACTL7A	chr9	9q31.3	1	0	1	0	Amplification	1	0
ACTL7B	chr9	9q31.3	1	0	1	0	Amplification	1	0
ADAMTSL1	chr9	9p22.2-p22.1	1	0	0	1	Amplification	1	0
AK3	chr9	9p24.1	1	0	0	1	Amplification	1	0
AL365202	chr9	0	1	0	0	1	Amplification	1	0
ANKRD19P	chr9	9q22.31	1	0	1	0	Amplification	1	0
ARID3C	chr9	9p13.3	1	0	1	0	Amplification	1	0
ASPN	chr9	9q22.31	1	0	1	0	Amplification	1	0
AUH	chr9	9q22.31	1	0	1	0	Amplification	1	0
BARX1	chr9	9q22.32	1	0	1	0	Amplification	1	0
BICD2	chr9	9q22.31	1	0	1	0	Amplification	1	0
BNC2	chr9	9p22.3-p22.2	1	0	0	1	Amplification	1	0
C9orf123	chr9	9p24.1	1	0	0	1	Amplification	1	0
C9orf129	chr9	9q22.31	1	0	1	0	Amplification	1	0
C9orf131	chr9	9p13.3	1	0	1	0	Amplification	1	0
C9orf24	chr9	9p13.3	1	0	1	0	Amplification	1	0
C9orf3	chr9	9q22.32	1	0	1	0	Amplification	1	0
C9orf38	chr9	0	1	0	0	1	Amplification	1	0
C9orf53	chr9	9p21.3	1	0	0	1	Amplification	1	0
C9orf72	chr9	9p21.2	1	0	0	1	Amplification	1	0
C9orf89	chr9	9q22.31	1	0	1	0	Amplification	1	0
C9orf92	chr9	9p22.3	1	0	0	1	Amplification	1	0
CAAP1	chr9	9p21.2	1	0	0	1	Amplification	1	0
CBWD1	chr9	9p24.3	1	0	0	1	Amplification	1	0
CCDC171	chr9	9p22.3	1	0	0	1	Amplification	1	0
CCL19	chr9	9p13.3	1	0	1	0	Amplification	1	0
CCL21	chr9	9p13.3	1	0	1	0	Amplification	1	0
CCL27	chr9	9p13.3	1	0	1	0	Amplification	1	0
CD274	chr9	9p24.1	1	0	0	1	Amplification	1	0
CDC14B	chr9	9q22.32|9q22.32	1	0	1	0	Amplification	1	0
CDC37L1	chr9	9p24.1	1	0	0	1	Amplification	1	0
CDKN2A	chr9	9p21.3	1	0	0	1	Amplification	1	0
CDKN2B	chr9	9p21.3	1	0	0	1	Amplification	1	0
CENPP	chr9	9q22.31	1	0	1	0	Amplification	1	0
CNTFR	chr9	9p13.3	1	0	1	0	Amplification	1	0
CNTLN	chr9	9p22.2	1	0	0	1	Amplification	1	0
CTNNAL1	chr9	9q31.3	1	0	1	0	Amplification	1	0
DCAF12	chr9	9p13.3	1	0	1	0	Amplification	1	0
DCTN3	chr9	9p13.3	1	0	1	0	Amplification	1	0
DENND4C	chr9	9p22.1	1	0	0	1	Amplification	1	0
DMRT1	chr9	9p24.3	1	0	0	1	Amplification	1	0
DMRT2	chr9	9p24.3	1	0	0	1	Amplification	1	0
DMRT3	chr9	9p24.3	1	0	0	1	Amplification	1	0
DMRTA1	chr9	9p21.3	1	0	0	1	Amplification	1	0
DNAI1	chr9	9p13.3	1	0	1	0	Amplification	1	0
DNAJB5	chr9	9p13.3	1	0	1	0	Amplification	1	0
DOCK8	chr9	9p24.3	1	0	0	1	Amplification	1	0
ECM2	chr9	9q22.31	1	0	1	0	Amplification	1	0
ELAVL2	chr9	9p21.3	1	0	0	1	Amplification	1	0
ENHO	chr9	9p13.3	1	0	1	0	Amplification	1	0
EPB41L4B	chr9	9q31.3	1	0	1	0	Amplification	1	0

Patel *et al.* , 2021. Whole-exome sequencing analysis of oral squamous cell carcinoma delineated by tobacco usage habits

Supplementary Table 5. List of genes affected by copy number alteration events in OSCC patients

Gene	Chromosome	Cytoband	Recurrence	Recurrence in smoker cohort	Recurrence in chewer cohort	Recurrence in No habit cohort	State	Samples with CNA gain	Samples with CNA loss
EQTN	chr9	9p21.2	1	0	0	1	Amplification	1	0
ERCC6L2	chr9	9q22.32	1	0	1	0	Amplification	1	0
ERMP1	chr9	9p24.1	1	0	0	1	Amplification	1	0
FAM120A	chr9	9q22.31	1	0	1	0	Amplification	1	0
FAM120AOS	chr9	9q22.31	1	0	1	0	Amplification	1	0
FAM154A	chr9	9p22.1	1	0	0	1	Amplification	1	0
FAM166B	chr9	9p13.3	1	0	1	0	Amplification	1	0
FAM205A	chr9	9p13.3	1	0	1	0	Amplification	1	0
FAM205B	chr9	9p13.3	1	0	1	0	Amplification	1	0
FAM206A	chr9	9q31.3	1	0	1	0	Amplification	1	0
FAM214B	chr9	9p13.3	1	0	1	0	Amplification	1	0
FAM219A	chr9	9p13.3	1	0	1	0	Amplification	1	0
FANCC	chr9	9q22.32	1	0	1	0	Amplification	1	0
FANCG	chr9	9p13.3	1	0	1	0	Amplification	1	0
FBP1	chr9	9q22.32	1	0	1	0	Amplification	1	0
FBP2	chr9	14q23.1	1	0	1	0	Amplification	1	0
FGD3	chr9	8q11.2-q13.2	1	0	1	0	Amplification	1	0
FOCAD	chr9	9p21.3	1	0	0	1	Amplification	1	0
FRRS1L	chr9	9q31.3	1	0	1	0	Amplification	1	0
GALT	chr9	9p13.3	1	0	1	0	Amplification	1	0
GLDC	chr9	9p24.1	1	0	0	1	Amplification	1	0
GLIS3	chr9	9p24.2	1	0	0	1	Amplification	1	0
HABP4	chr9	9q22.32	1	0	1	0	Amplification	1	0
HAUS6	chr9	9p22.1	1	0	0	1	Amplification	1	0
HIATL1	chr9	9q22.32	1	0	1	0	Amplification	1	0
HIATL2	chr9	9q22.33	1	0	1	0	Amplification	1	0
HSD17B3	chr9	9q22.32	1	0	1	0	Amplification	1	0
IARS	chr9	9q22.31	1	0	1	0	Amplification	1	0
IFNA1	chr9	9p21.3	1	0	0	1	Amplification	1	0
IFNA13	chr9	9p21.3	1	0	0	1	Amplification	1	0
IFNA14	chr9	9p21.3	1	0	0	1	Amplification	1	0
IFNA16	chr9	9p21.3	1	0	0	1	Amplification	1	0
IFNA2	chr9	9p21.3	1	0	0	1	Amplification	1	0
IFNA21	chr9	9p21.3	1	0	0	1	Amplification	1	0
IFNA5	chr9	9p21.3	1	0	0	1	Amplification	1	0
IFNA6	chr9	9p21.3	1	0	0	1	Amplification	1	0
IFNA7	chr9	9p21.3	1	0	0	1	Amplification	1	0
IFNA8	chr9	9p21.3	1	0	0	1	Amplification	1	0
IFNB1	chr9	9p21.3	1	0	0	1	Amplification	1	0
IFNE	chr9	9p21.3	1	0	0	1	Amplification	1	0
IFNK	chr9	9p21.2	1	0	0	1	Amplification	1	0
IFNW1	chr9	9p21.3	1	0	0	1	Amplification	1	0
IFT74	chr9	9p21.2	1	0	0	1	Amplification	1	0
IKBKAP	chr9	9q31.3	1	0	1	0	Amplification	1	0
IL11RA	chr9	9p13.3	1	0	1	0	Amplification	1	0
IL33	chr9	9p24.1	1	0	0	1	Amplification	1	0
INSL4	chr9	9p24.1	1	0	0	1	Amplification	1	0
INSL6	chr9	9p24.1	1	0	0	1	Amplification	1	0
IPPK	chr9	9q22.31	1	0	1	0	Amplification	1	0
IZUMO3	chr9	9p21.3	1	0	0	1	Amplification	1	0
JAK2	chr9	9p24.1	1	0	0	1	Amplification	1	0
KANK1	chr9	9p24.3	1	0	0	1	Amplification	1	0
KCNV2	chr9	9p24.2	1	0	0	1	Amplification	1	0
KDM4C	chr9	9p24.1	1	0	0	1	Amplification	1	0
KIAA0020	chr9	9p24.2	1	0	0	1	Amplification	1	0
KIAA1045	chr9	0	1	0	1	0	Amplification	1	0
KIAA1432	chr9	9p24.1	1	0	0	1	Amplification	1	0
KIAA2026	chr9	9p24.1	1	0	0	1	Amplification	1	0

Patel *et al.* , 2021. Whole-exome sequencing analysis of oral squamous cell carcinoma delineated by tobacco usage habits

Supplementary Table 5. List of genes affected by copy number alteration events in OSCC patients

Gene	Chromosome	Cytoband	Recurrence	Recurrence in smoker cohort	Recurrence in chewer cohort	Recurrence in No habit cohort	State	Samples with CNA gain	Samples with CNA loss
KIF24	chr9	9p13.3	1	0	1	0	Amplification	1	0
KLF4	chr9	9q31.2	1	0	1	0	Amplification	1	0
KLHL9	chr9	9p21.3	1	0	0	1	Amplification	1	0
LINC00092	chr9	9q22.32	1	0	1	0	Amplification	1	0
LINGO2	chr9	9p21.2-p21.1	1	0	0	1	Amplification	1	0
LRRC19	chr9	9p21.2	1	0	0	1	Amplification	1	0
MIRLET7DHG	chr9	9q22.32	1	0	1	0	Amplification	1	0
MLANA	chr9	9p24.1	1	0	0	1	Amplification	1	0
MLLT3	chr9	9p21.3	1	0	0	1	Amplification	1	0
MOB3B	chr9	9p21.2	1	0	0	1	Amplification	1	0
MTAP	chr9	9p21.3	1	0	0	1	Amplification	1	0
NFIL3	chr9	9q22.31	1	0	1	0	Amplification	1	0
NINJ1	chr9	9q22.31	1	0	1	0	Amplification	1	0
NOL8	chr9	9q22.31	1	0	1	0	Amplification	1	0
NUDT2	chr9	9p13.3	1	0	1	0	Amplification	1	0
NUTM2F	chr9	9q22.32	1	0	1	0	Amplification	1	0
NUTM2G	chr9	9q22.33	1	0	1	0	Amplification	1	0
OGN	chr9	9q22.31	1	0	1	0	Amplification	1	0
OMD	chr9	9q22.31	1	0	1	0	Amplification	1	0
PAX5	chr9	9p13.2	1	0	1	0	Amplification	1	0
PDCD1LG2	chr9	9p24.1	1	0	0	1	Amplification	1	0
PHF2	chr9	9q22.31	1	0	1	0	Amplification	1	0
PIGO	chr9	9p13.3	1	0	1	0	Amplification	1	0
PLAA	chr9	9p21.2	1	0	0	1	Amplification	1	0
PLGRKT	chr9	9p24.1	1	0	0	1	Amplification	1	0
PLIN2	chr9	9p22.1	1	0	0	1	Amplification	1	0
PRSS3	chr9	9p13.3	1	0	1	0	Amplification	1	0
PTCH1	chr9	9q22.32	1	0	1	0	Amplification	1	0
PTENP1	chr9	9p13.3	1	0	1	0	Amplification	1	0
PTPDC1	chr9	9q22.32	1	0	1	0	Amplification	1	0
PTPLAD2	chr9	9p21.3	1	0	0	1	Amplification	1	0
PTPN3	chr9	9q31.3	1	0	1	0	Amplification	1	0
PTPRD	chr9	9p24.1-p23	1	0	0	1	Amplification	1	0
RAD23B	chr9	9q31.2	1	0	1	0	Amplification	1	0
RANBP6	chr9	9p24.1	1	0	0	1	Amplification	1	0
RCL1	chr9	9p24.1	1	0	0	1	Amplification	1	0
RFX3	chr9	9p24.2	1	0	0	1	Amplification	1	0
RLN1	chr9	9p24.1	1	0	0	1	Amplification	1	0
RLN2	chr9	9p24.1	1	0	0	1	Amplification	1	0
ROR2	chr9	15q22.2	1	0	1	0	Amplification	1	0
RPP25L	chr9	9p13.3	1	0	1	0	Amplification	1	0
RPS6	chr9	9p22.1	1	0	0	1	Amplification	1	0
RRAGA	chr9	9p22.1	1	0	0	1	Amplification	1	0
RUSC2	chr9	9p13.3	1	0	1	0	Amplification	1	0
SH3GL2	chr9	9p22.2	1	0	0	1	Amplification	1	0
SIGMAR1	chr9	9p13.3	1	0	1	0	Amplification	1	0
SLC1A1	chr9	9p24.2	1	0	0	1	Amplification	1	0
SLC24A2	chr9	14q32.12	1	0	0	1	Amplification	1	0
SLC35D2	chr9	9q22.32	1	0	1	0	Amplification	1	0
SMARCA2	chr9	9p24.3	1	0	0	1	Amplification	1	0
SPATA6L	chr9	9p24.2-p24.1	1	0	0	1	Amplification	1	0
SPTLC1	chr9	9q22.31	1	0	1	0	Amplification	1	0
STOML2	chr9	9p13.3	1	0	1	0	Amplification	1	0
SUSD3	chr9	9q22.31	1	0	1	0	Amplification	1	0
SYK	chr9	9q22.2	1	0	1	0	Amplification	1	0
TEK	chr9	9p21.2	1	0	0	1	Amplification	1	0
TESK1	chr9	9p13.3	1	0	1	0	Amplification	1	0

Patel *et al.* , 2021. Whole-exome sequencing analysis of oral squamous cell carcinoma delineated by tobacco usage habits

Supplementary Table 5. List of genes affected by copy number alteration events in OSCC patients

Gene	Chromosome	Cytoband	Recurrence	Recurrence in smoker cohort	Recurrence in chewer cohort	Recurrence in No habit cohort	State	Samples with CNA gain	Samples with CNA loss
TMEM245	chr9	9q31.3	1	0	1	0	Amplification	1	0
TPD52L3	chr9	9p24.1	1	0	0	1	Amplification	1	0
TUSC1	chr9	9p21.2	1	0	0	1	Amplification	1	0
UBAP1	chr9	9p13.3	1	0	1	0	Amplification	1	0
UBAP2	chr9	9p13.3	1	0	1	0	Amplification	1	0
UBE2R2	chr9	9p13.3	1	0	1	0	Amplification	1	0
UHRF2	chr9	9p24.1	1	0	0	1	Amplification	1	0
UNC13B	chr9	9p13.3	1	0	1	0	Amplification	1	0
VCP	chr9	9p13.3	1	0	1	0	Amplification	1	0
VLDLR	chr9	9p24.2	1	0	0	1	Amplification	1	0
WNK2	chr9	9q22.31	1	0	1	0	Amplification	1	0
ZNF169	chr9	9q22.32	1	0	1	0	Amplification	1	0
ZNF367	chr9	9q22.32|9q22.32	1	0	1	0	Amplification	1	0
ZNF462	chr9	9q31.2	1	0	1	0	Amplification	1	0
ZNF484	chr9	9q22.31	1	0	1	0	Amplification	1	0
ZNF510	chr9	9q22.33	1	0	1	0	Amplification	1	0
ZNF782	chr9	9q22.33	1	0	1	0	Amplification	1	0
MRGPRX1	chr11	11p15.1|11	6	2	2	2	Amplification/ Deletion	3	3
RASA4	chr7	7q22.1	6	0	4	2	Amplification/ Deletion	3	3
HERC2P3	chr15	15q11.1-q11.2	4	2	1	1	Amplification/ Deletion	2	2
POTEB	chr15	15q11.2	4	2	1	1	Amplification/ Deletion	2	2
POTEB2	chr15	15q11.2	4	2	1	1	Amplification/ Deletion	2	2
TBC1D3	chr17	17q12	4	0	3	1	Amplification/ Deletion	2	2
PRAMEF1	chr1	1p36.21	2	2	0	0	Amplification/ Deletion	1	1
PRAMEF11	chr1	1p36.21	2	2	0	0	Amplification/ Deletion	1	1
GOLGA8B	chr15	15q14	2	0	2	0	Amplification/ Deletion	1	1
C4A	chr6	6p21.33	2	0	1	1	Amplification/ Deletion	1	1
C4B	chr6	6p21.33	2	0	1	1	Amplification/ Deletion	1	1
INTS4L2	chr7	7q11.21	2	1	1	0	Amplification/ Deletion	1	1
MUC17	chr7	7q22.1	2	1	1	0	Amplification/ Deletion	1	1
PMS2	chr7	7p22.1	2	1	1	0	Amplification/ Deletion	1	1
ZAN	chr7	7q22.1	2	1	1	0	Amplification/ Deletion	1	1
DEFB103A	chr8	8p23.1	2	0	1	1	Amplification/ Deletion	1	1
DEFB4A	chr8	8p23.1	2	0	1	1	Amplification/ Deletion	1	1
POLR2J3	chr7	7q22.1	9	1	5	3	Amplification/ Deletion	4	5
RASA4B	chr7	7q22.1	9	1	5	3	Amplification/ Deletion	4	5
PGA3	chr11	11q12.2	7	3	2	2	Amplification/ Deletion	3	4
PGA4	chr11	11q12.2	7	3	2	2	Amplification/ Deletion	3	4

Patel *et al.* , 2021. Whole-exome sequencing analysis of oral squamous cell carcinoma delineated by tobacco usage habits

Supplementary Table 5. List of genes affected by copy number alteration events in OSCC patients

Gene	Chromosome	Cytoband	Recurrence	Recurrence in smoker cohort	Recurrence in chewer cohort	Recurrence in No habit cohort	State	Samples with CNA gain	Samples with CNA loss
GSTM1	chr1	1p13.3	5	2	2	1	Amplification/ Deletion	2	3
GOLGA8A	chr15	15q14	3	0	3	0	Amplification/ Deletion	1	2
ZNF92	chr7	7q11.21	3	1	2	0	Amplification/ Deletion	1	2
ATAD3B	chr1	1p36.33	1	1	0	0	Deletion	0	1
ATAD3C	chr1	1p36.33	1	1	0	0	Deletion	0	1
ERC1	chr12	12p13.33	1	1	0	0	Deletion	0	1
HCAR3	chr12	12q24.31	1	1	0	0	Deletion	0	1
CRYL1	chr13	13q12.11	1	1	0	0	Deletion	0	1
GJB6	chr13	13q12.11	1	1	0	0	Deletion	0	1
DHRS4L2	chr14	14q11.2	1	0	1	0	Deletion	0	1
CHRFAM7A	chr15	15q13.2	1	0	0	1	Deletion	0	1
GOLGA8R	chr15	15q13.2	1	0	0	1	Deletion	0	1
STRC	chr15	15q15.3	1	0	1	0	Deletion	0	1
AURKC	chr19	19q13.43	1	0	1	0	Deletion	0	1
CYP2A7	chr19	19q13.2	1	1	0	0	Deletion	0	1
ZNF264	chr19	19q13.43	1	0	1	0	Deletion	0	1
ALPP	chr2	2q37.1	1	1	0	0	Deletion	0	1
SIRPB1	chr20	20p13	1	1	0	0	Deletion	0	1
MYH9	chr22	22q12.3	1	0	0	1	Deletion	0	1
HGFAC	chr4	4p16.3	1	0	0	1	Deletion	0	1
DUSP22	chr6	6p25.3	1	1	0	0	Deletion	0	1
ARL17B	chr17	17q21.31	6	3	1	2	Amplification/ Deletion	2	4
RP1	chr1	18q12.1-q12.2	3	1	0	2	Amplification/ Deletion	1	3
CATSPER2	chr15	15q15.3	2	1	1	0	Deletion	0	2
GRAP	chr17	17p11.2	2	2	0	0	Deletion	0	2
PSG1	chr19	19q13.2	2	1	1	0	Deletion	0	2
PSG10P	chr19	19q13.2	2	1	1	0	Deletion	0	2
PSG11	chr19	19q13.31	2	1	1	0	Deletion	0	2
PSG6	chr19	19q13.31	2	1	1	0	Deletion	0	2
ALPPL2	chr2	2q37.1	2	1	1	0	Deletion	0	2
BTNL3	chr5	5q35.3	2	0	0	2	Deletion	0	2
PCDHA10	chr5	5q31.3	2	0	0	2	Deletion	0	2
PCDHA9	chr5	5q31.3	2	0	0	2	Deletion	0	2
MGAM	chr7	7q34	2	1	0	1	Deletion	0	2
SLC35E2	chr1	1p36.33	3	1	1	1	Deletion	0	3
RHD	chr1	1p36.11	7	1	4	2	Deletion	0	7
