# Supplementary material for: Whole-Exome Sequencing Analysis of Oral Squamous Cell Carcinoma Delineated by Tobacco Usage Habits
Source: Front Oncol. 2021 May 31;11:660696. doi: 10.3389/fonc.2021.660696 (PMC8200776; doi:10.3389/fonc.2021.660696)
Supplement: Supplementary file 6 [file Table_6.pdf]

Patel *et al.* , 2021. Whole-exome sequencing analysis of oral squamous cell carcinoma delineated by tobacco usage habits

Supplementary Table 6. List of genes screened for potential druggability using DGIdb resource

**Genes with single nucleotide variants**		
TP53	SBNO2	CNTRL
NOTCH1	ASH1L	FBXW7
TTN	ZNF407	ADAD2
CASP8	L3MBTL4	FAM208B
MUC16	ZAN	MUM1
RYR2	SPTA1	ASB10
LRP2	ZNF530	DNAH8
HYDIN	PRRC2A	BRINP1
VCAN	DNAH3	FRYL
PDE4DIP	TRBC2	PRR23A
GPR98	FAT1	TTC3
PTPRQ	PXDNL	SRRM2
PCLO	COL2A1	PCDHA12
PLEC	ABCA13	CFHR1
OBSCN	GDF6	KIAA0907
DNAH9	BSN	WNK2
NEB	ABCA2	LILRA2
FAT4	KHDRBS2	MTTP
LRP1B	SLITRK3	MUC3A
F8	GRID1	TBC1D28
MACC1	FREM2	DENND1B
DLC1	KMT2B	CNTN3
HRAS	SCN5A	MYH9
SHANK3	VPS13D	STK11IP
SYNE1	PLEKHH2	MYOCD
SCRIB	GPR179	NOTCH4
USH2A	PAPPA2	CDKN2A
TTC6	EPHA2	RP11-766F14.2
ATP13A5	NRXN1	DPCR1
MON2	ATRX	RADIL
PTPN14	RNF213	NOTCH3
TECTA	LIMD1	SYNE2
DNAH6	PIGG	SVEP1
XIRP2	FLNA	ZNF445
ASXL3	BEND4	NBPF3
CYFIP2	GBF1	IGHD
HTT	CPAMD8	EPG5
ADAMTSL1	OSBP	ZNF429
OR4C15	SH3TC2	LOC285556
GREB1L	COL17A1	RP1L1
FSIP2	DCC	PDE12
DST	POLD1	EPPK1
CACNA1H	CASC5	STAB2
USP48	IRF2BP2	SLC26A8
SSPO	OR4C11	MPRIP

Patel *et al.* , 2021. Whole-exome sequencing analysis of oral squamous cell carcinoma delineated by tobacco usage habits

Supplementary Table 6. List of genes screened for potential druggability using DGIdb resource

**Genes with single nucleotide variants**		
MYO5B	PLEKHH1	TEKT4
PCSK6	DOCK2	DNAH10
CELSR1	RECQL4	SLC24A3
MYO9B	NPY1R	HIVEP2
CACNA1B	GRIP2	NLRC3
KMT2C	LTBP1	EXOC6B
HMCN2	C12orf42	SNRNP200
KALRN	AHNAK	FMN1
PCDHA11	MGAM	BCAR1
RP1	ETV5	ANK3
ZFHX4	IGFN1	ASPM
ATM	DSCAM	CFTR
CSMD1	PCNT	MUC6
ERBB4	SLX4	SLC12A7
DNAH14	OR51M1	KCNT2
LPHN3	HUWE1	DCTD
KIF2B	CACNA1E	POLQ
DOPEY2	DNAH1	ASXL2

**Genes affected by copy number alterations**		
CFHR3	EGFR	TBC1D3H
CFHR1	OR2A42	FCGR3B
OR4K2	SEC61G	CHEK2P2
OR4K5	VSTM2A	CYP2D6
OR4M1	LRRC37A2	POLR2J2
OR4N2	HLA	UPK3BL
OR4Q3	ADAM3A	MRGPRX1
OR2A1	PDPR	RASA4
OR2A20P	TAS2R43	POLR2J3
OR2A7	SULT1A1	RASA4B
OR2A9P	CCL3L1	PGA3
GSTT1	CROCCP2	PGA4
LGALS9C	SKA3	GSTT1
APOBEC3B	PDXDC1	ADAM3A
ARHGEF35	CCL4L2	PDPR
ARHGEF5	FCGBP	MRGPRX1
DMBT1	PCDHB8	RASA4
ARL17A	ZDHHC11B	POLR2J3
CCL3L3	FKBP9L	RASA4B
NSF	LANCL2	PGA3
LILRA3	VOPP1	PGA4
CCL4L1	FCGR2C	GSTM1
LRRC37A	FCGR3A	ARL17B
DDT	OR4M2	RP1
DDTL	OR4N4	SLC35E2
GSTT2	HNRNPCL1	RHD
GSTT2B	PRAMEF2	
