# Supplementary material for: Whole-Exome Sequencing Analysis of Oral Squamous Cell Carcinoma Delineated by Tobacco Usage Habits
Source: Front Oncol. 2021 May 31;11:660696. doi: 10.3389/fonc.2021.660696 (PMC8200776; doi:10.3389/fonc.2021.660696)
Supplement: Supplementary file 7 [file Table_7.pdf]

Patel *et al.* , 2021. Whole-exome sequencing analysis of oral squamous cell carcinoma delineated by tobacco usage habits

Supplementary Table 7. List of identified genes with an available FDA-approved anti-neoplastic drug using DGIdb resource

Gene symbol	Gene name	Drug	Source	PMID
TP53	TUMOR PROTEIN P53	BORTEZOMIB	TALC	28679691
TP53	TUMOR PROTEIN P53	CARBOPLATIN	JAX-CKB	25567130
TP53	TUMOR PROTEIN P53	PACLITAXEL	JAX-CKB	16459017
TP53	TUMOR PROTEIN P53	DOXORUBICIN	DTC	25658463
TP53	TUMOR PROTEIN P53	CETUXIMAB	CIViC	24957073
TP53	TUMOR PROTEIN P53	ETOPOSIDE	JAX-CKB	25964101
TP53	TUMOR PROTEIN P53	CISPLATIN	JAX-CKB	25376608
TP53	TUMOR PROTEIN P53	DOCETAXEL	JAX-CKB	21399868
TP53	TUMOR PROTEIN P53	TAMOXIFEN	DTC	10786679
TP53	TUMOR PROTEIN P53	TEMOZOLOMIDE	JAX-CKB	21730979
TP53	TUMOR PROTEIN P53	OXALIPLATIN	JAX-CKB	24957073
TP53	TUMOR PROTEIN P53	CAPECITABINE	CIViC	24957073
TP53	TUMOR PROTEIN P53	PAZOPANIB	JAX-CKB	26646755
TP53	TUMOR PROTEIN P53	MITOMYCIN	CIViC	14514923
TP53	TUMOR PROTEIN P53	METHOTREXATE	DTC	17363498
TP53	TUMOR PROTEIN P53	SELUMETINIB	JAX-CKB	26343583
TP53	TUMOR PROTEIN P53	GRANISETRON	ClearityFoundationClinicalTrial	
TP53	TUMOR PROTEIN P53	VORINOSTAT	JAX-CKB	26009011
TP53	TUMOR PROTEIN P53	RO-5045337	JAX-CKB	26459177
NOTCH1	NOTCH 1	RG-4733	MyCancerGenome	27154916
NOTCH1	NOTCH 1	NIROGACESTAT	MyCancerGenome	26202948
CASP8	CASPASE 8	CONATUMUMAB	CIViC	26291055
MUC16	MUCIN 16	ABAGOVOMAB	TdgClinicalTrial	
MUC16	MUCIN 16	OREGOVOMAB	DrugBank	17073521
HRAS	HRAS PROTO-ONCOGENE	PELAREOREP	TdgClinicalTrial	
HRAS	HRAS PROTO-ONCOGENE	EVEROLIMUS	JAX-CKB	26544513
HRAS	HRAS PROTO-ONCOGENE	SELUMETINIB	JAX-CKB	26544513
HRAS	HRAS PROTO-ONCOGENE	BINIMETINIB	JAX-CKB	26544513
ATM	ATM SERINE/THREONINE KINASE	RUCAPARIB	ClearityFoundationBiomarkers	
ATM	ATM SERINE/THREONINE KINASE	VELIPARIB	ClearityFoundationBiomarkers	21300883
ATM	ATM SERINE/THREONINE KINASE	SELUMETINIB	CIViC	27922010
ATM	ATM SERINE/THREONINE KINASE	NIRAPARIB	ClearityFoundationBiomarkers	
ATM	ATM SERINE/THREONINE KINASE	OLAPARIB	ClearityFoundationBiomarkers	20739657
ATM	ATM SERINE/THREONINE KINASE	DOXORUBICIN	CIViC	23585524
ATM	ATM SERINE/THREONINE KINASE	TEMOZOLOMIDE	CGI	23960094
ATM	ATM SERINE/THREONINE KINASE	2X-121	ClearityFoundationBiomarkers	26513298
ATM	ATM SERINE/THREONINE KINASE	TRAMETINIB	CIViC	27922010
ERBB4	ERB-B2 RECEPTOR TYROSINE KINASE 4	PELITINIB	TALC	
ERBB4	ERB-B2 RECEPTOR TYROSINE KINASE 4	DACOMITINIB	TALC	
ERBB4	ERB-B2 RECEPTOR TYROSINE KINASE 4	AC-480	TALC	
ERBB4	ERB-B2 RECEPTOR TYROSINE KINASE 4	GEFITINIB	DTC	
ERBB4	ERB-B2 RECEPTOR TYROSINE KINASE 4	POZIOTINIB	ChemblInteractions	
ERBB4	ERB-B2 RECEPTOR TYROSINE KINASE 4	BMS-690514	TALC	
ERBB4	ERB-B2 RECEPTOR TYROSINE KINASE 4	VANDETANIB	ChemblInteractions	
ERBB4	ERB-B2 RECEPTOR TYROSINE KINASE 4	LAPATINIB	DTC	25590338
EPHA2	EPH RECEPTOR A2	DASATINIB	DTC	19010823
EPHA2	EPH RECEPTOR A2	VANDETANIB	ChemblInteractions	
EPHA2	EPH RECEPTOR A2	REGORAFENIB	DrugBank	
POLD1	DNA POLYMERASE DELTA 1	CLOFARABINE	ChemblInteractions	
POLD1	DNA POLYMERASE DELTA 1	CYTARABINE	ChemblInteractions	
NOTCH4	NOTCH 4	RG-4733	MyCancerGenome	
NOTCH4	NOTCH 4	NIROGACESTAT	MyCancerGenome	
NOTCH3	NOTCH 3	TAREXTUMAB	TdgClinicalTrial	25934888
NOTCH3	NOTCH 3	RG-4733	MyCancerGenome	
NOTCH3	NOTCH 3	NIROGACESTAT	MyCancerGenome	
EGFR	EPIDERMAL GROWTH FACTOR RECEPTOR	DACOMITINIB	TALC	24857124
EGFR	EPIDERMAL GROWTH FACTOR RECEPTOR	PANITUMUMAB	TALC	17355997
EGFR	EPIDERMAL GROWTH FACTOR RECEPTOR	NECITUMUMAB	TALC	20197484
EGFR	EPIDERMAL GROWTH FACTOR RECEPTOR	SAPITINIB	TALC	24886365
EGFR	EPIDERMAL GROWTH FACTOR RECEPTOR	POZIOTINIB	JAX-CKB	
EGFR	EPIDERMAL GROWTH FACTOR RECEPTOR	AC-480	TALC	19821562
EGFR	EPIDERMAL GROWTH FACTOR RECEPTOR	NIMOTUZUMAB	TALC	25185971
EGFR	EPIDERMAL GROWTH FACTOR RECEPTOR	CHEMBL3397300	TALC	27836716
EGFR	EPIDERMAL GROWTH FACTOR RECEPTOR	AFATINIB	TALC	26619011

Patel *et al.* , 2021. Whole-exome sequencing analysis of oral squamous cell carcinoma delineated by tobacco usage habits

Supplementary Table 7. List of identified genes with an available FDA-approved anti-neoplastic drug using DGIdb resource

Gene symbol	Gene name	Drug	Source	PMID
EGFR	EPIDERMAL GROWTH FACTOR RECEPTOR	IMGATUZUMAB	TALC	23209031
EGFR	EPIDERMAL GROWTH FACTOR RECEPTOR	MP-412	DrugBank	17888033
EGFR	EPIDERMAL GROWTH FACTOR RECEPTOR	ERLOTINIB	TALC	26619011
EGFR	EPIDERMAL GROWTH FACTOR RECEPTOR	GEFITINIB	TALC	24533047
EGFR	EPIDERMAL GROWTH FACTOR RECEPTOR	VANDETANIB	TALC	18681783
EGFR	EPIDERMAL GROWTH FACTOR RECEPTOR	LAPATINIB	TALC	25305330
EGFR	EPIDERMAL GROWTH FACTOR RECEPTOR	ROCILETINIB	MyCancerGenome	24065731
EGFR	EPIDERMAL GROWTH FACTOR RECEPTOR	NERATINIB	TALC	18681783
EGFR	EPIDERMAL GROWTH FACTOR RECEPTOR	PELITINIB	TALC	16710023
EGFR	EPIDERMAL GROWTH FACTOR RECEPTOR	CANERTINIB	DTC	23930994
EGFR	EPIDERMAL GROWTH FACTOR RECEPTOR	CETUXIMAB	TALC	26619011
EGFR	EPIDERMAL GROWTH FACTOR RECEPTOR	TESEVATINIB	TALC	22722787
EGFR	EPIDERMAL GROWTH FACTOR RECEPTOR	ZALUTUMUMAB	TALC	15383606
EGFR	EPIDERMAL GROWTH FACTOR RECEPTOR	AEE-788	MyCancerGenome	19147750
EGFR	EPIDERMAL GROWTH FACTOR RECEPTOR	ICOTINIB	TALC	24533047
EGFR	EPIDERMAL GROWTH FACTOR RECEPTOR	BMS-690514	TALC	23490650
EGFR	EPIDERMAL GROWTH FACTOR RECEPTOR	DULIGOTUZUMAB	MyCancerGenome	
EGFR	EPIDERMAL GROWTH FACTOR RECEPTOR	DEPATUXIZUMAB	MyCancerGenome	25895099
EGFR	EPIDERMAL GROWTH FACTOR RECEPTOR	CHEMBL2347958	CancerCommons	
EGFR	EPIDERMAL GROWTH FACTOR RECEPTOR	OSIMERTINIB	DrugBank	31825714
EGFR	EPIDERMAL GROWTH FACTOR RECEPTOR	BRIGATINIB	DrugBank	23239810
EGFR	EPIDERMAL GROWTH FACTOR RECEPTOR	OLMUTINIB	DrugBank	26898616
EGFR	EPIDERMAL GROWTH FACTOR RECEPTOR	VARLITINIB	DrugBank	
EGFR	EPIDERMAL GROWTH FACTOR RECEPTOR	CUDC-101	TALC	20388807
EGFR	EPIDERMAL GROWTH FACTOR RECEPTOR	PEMETREXED	CIViC	31605797
EGFR	EPIDERMAL GROWTH FACTOR RECEPTOR	PACLITAXEL	JAX-CKB	24886365
EGFR	EPIDERMAL GROWTH FACTOR RECEPTOR	PEMBROLIZUMAB	JAX-CKB	31086949
EGFR	EPIDERMAL GROWTH FACTOR RECEPTOR	TALAZOPARIB	CIViC	31852834
EGFR	EPIDERMAL GROWTH FACTOR RECEPTOR	AVELUMAB	CIViC	31086949
EGFR	EPIDERMAL GROWTH FACTOR RECEPTOR	NIVOLUMAB	JAX-CKB	31086949
EGFR	EPIDERMAL GROWTH FACTOR RECEPTOR	SIROLIMUS	JAX-CKB	24934779
EGFR	EPIDERMAL GROWTH FACTOR RECEPTOR	DURVALUMAB	JAX-CKB	31086949
EGFR	EPIDERMAL GROWTH FACTOR RECEPTOR	CRIZOTINIB	JAX-CKB	27595477
EGFR	EPIDERMAL GROWTH FACTOR RECEPTOR	PACRITINIB	CIViC	32226941
EGFR	EPIDERMAL GROWTH FACTOR RECEPTOR	ATEZOLIZUMAB	JAX-CKB	31086949
EGFR	EPIDERMAL GROWTH FACTOR RECEPTOR	CISPLATIN	JAX-CKB	23764753
EGFR	EPIDERMAL GROWTH FACTOR RECEPTOR	TRASTUZUMAB	TEND	11752352
EGFR	EPIDERMAL GROWTH FACTOR RECEPTOR	RINDOPEPIMUT	DrugBank	25586468
EGFR	EPIDERMAL GROWTH FACTOR RECEPTOR	CARBOPLATIN	CIViC	22370314
