# Supplementary material for: Whole-Exome Sequencing Analysis of Oral Squamous Cell Carcinoma Delineated by Tobacco Usage Habits
Source: Front Oncol. 2021 May 31;11:660696. doi: 10.3389/fonc.2021.660696 (PMC8200776; doi:10.3389/fonc.2021.660696)
Supplement: Supplementary file 8 [file Table_8.pdf]

Patel *et al.* , 2021. Whole-exome sequencing analysis of oral squamous cell carcinoma delineated by tobacco usage habits
Supplementary Table 8. List of pathways enriched using genes harbouring single nucleotide variant or affected by copy number alteration using Reactome

Pathway name	Entities found (Uniprot)	Entities Total	Entities ratio	Entities pValue	Entities FDR	Reactions found	Reactions total	Reactions ratio	Species name
Signaling by Non-Receptor Tyrosine Kinases	8	58	0.005	1.47E-04	1.32E-02	13	53	0.004	Homo Sapiens
Signaling by PTK6	8	58	0.005	1.47E-04	1.32E-02	13	53	0.004	Homo Sapiens
Diseases associated with O-glycosylation of proteins	8	71	0.006	5.57E-04	3.01E-02	5	9	0.001	Homo Sapiens

Gene mapped to pathway
ERBB4;KHDRBS2;BCAR1;HRAS;EGFR
ERBB4;KHDRBS2;BCAR1;HRAS;EGFR
SSPO;MUC16;NOTCH4;ADAMTSL1;NOTCH3;NOTCH1;MUC3A;MUC6
