# Supplementary material for: Whole-Exome Sequencing Analysis of Oral Squamous Cell Carcinoma Delineated by Tobacco Usage Habits
Source: Front Oncol. 2021 May 31;11:660696. doi: 10.3389/fonc.2021.660696 (PMC8200776; doi:10.3389/fonc.2021.660696)

Supplementary Figure 1

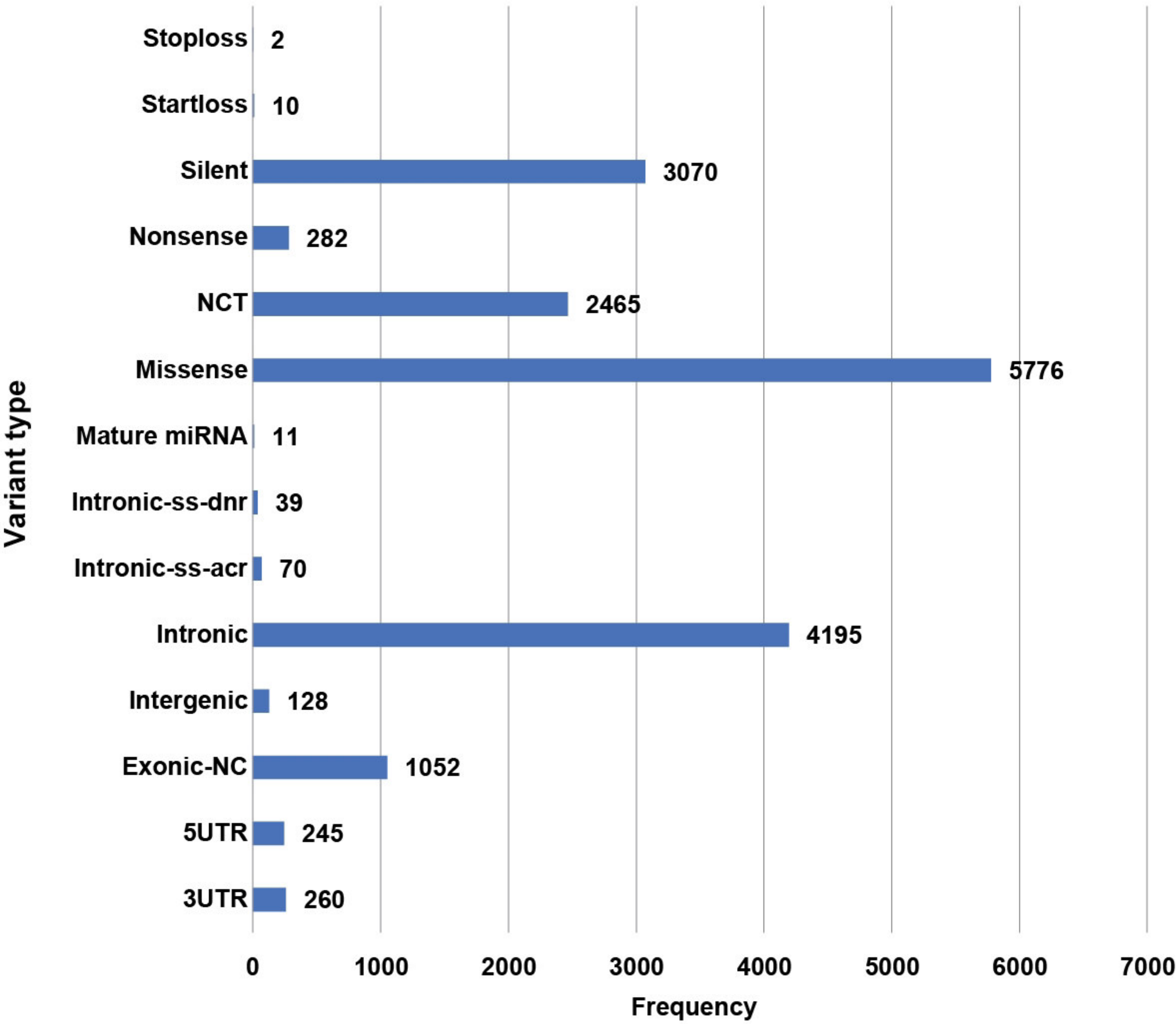

Supplementary figure 2

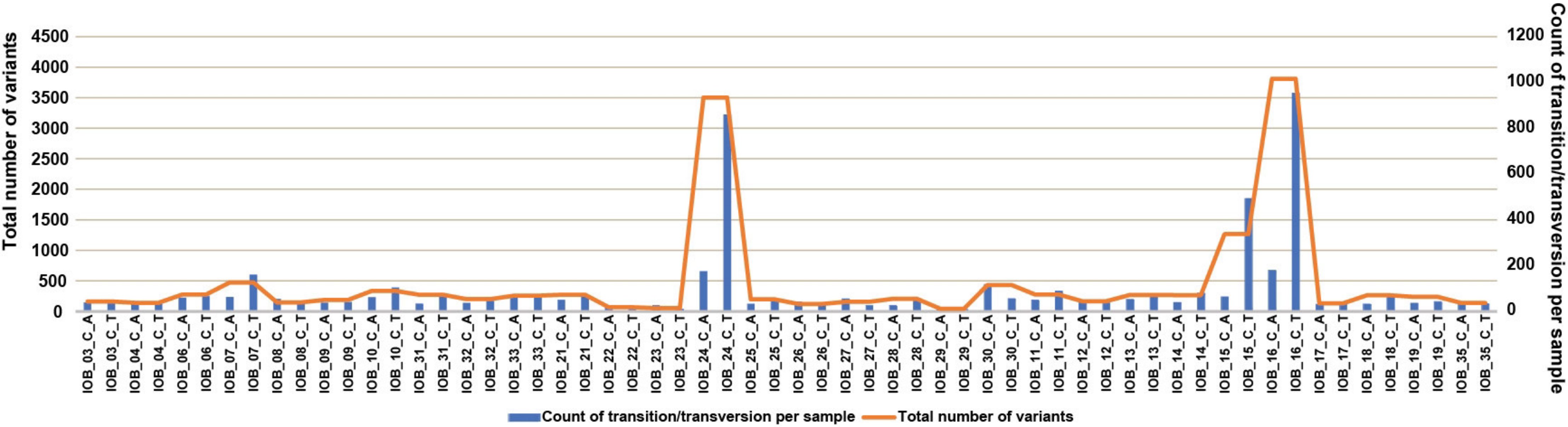

Supplementary figure 3

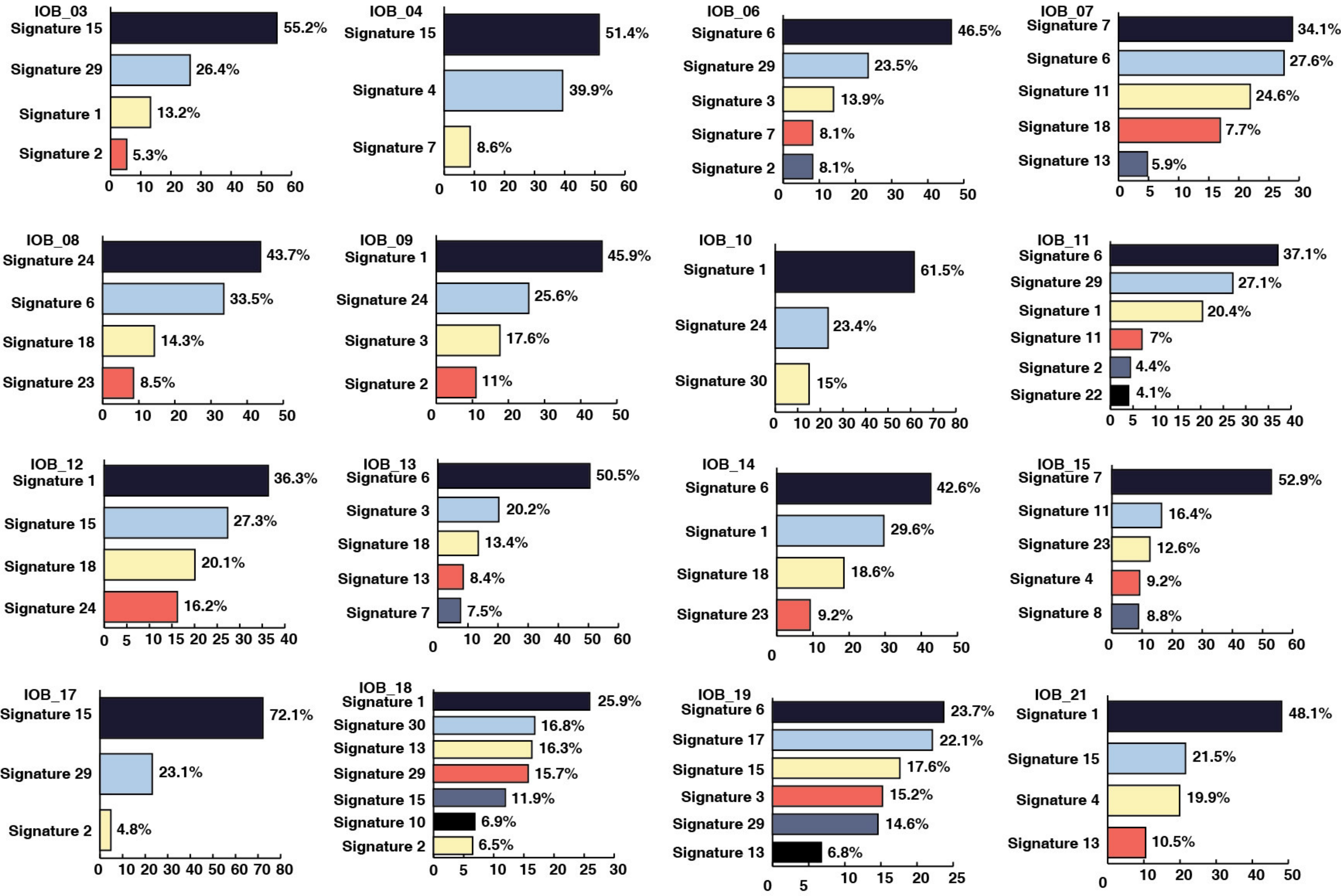

Supplementary figure 3

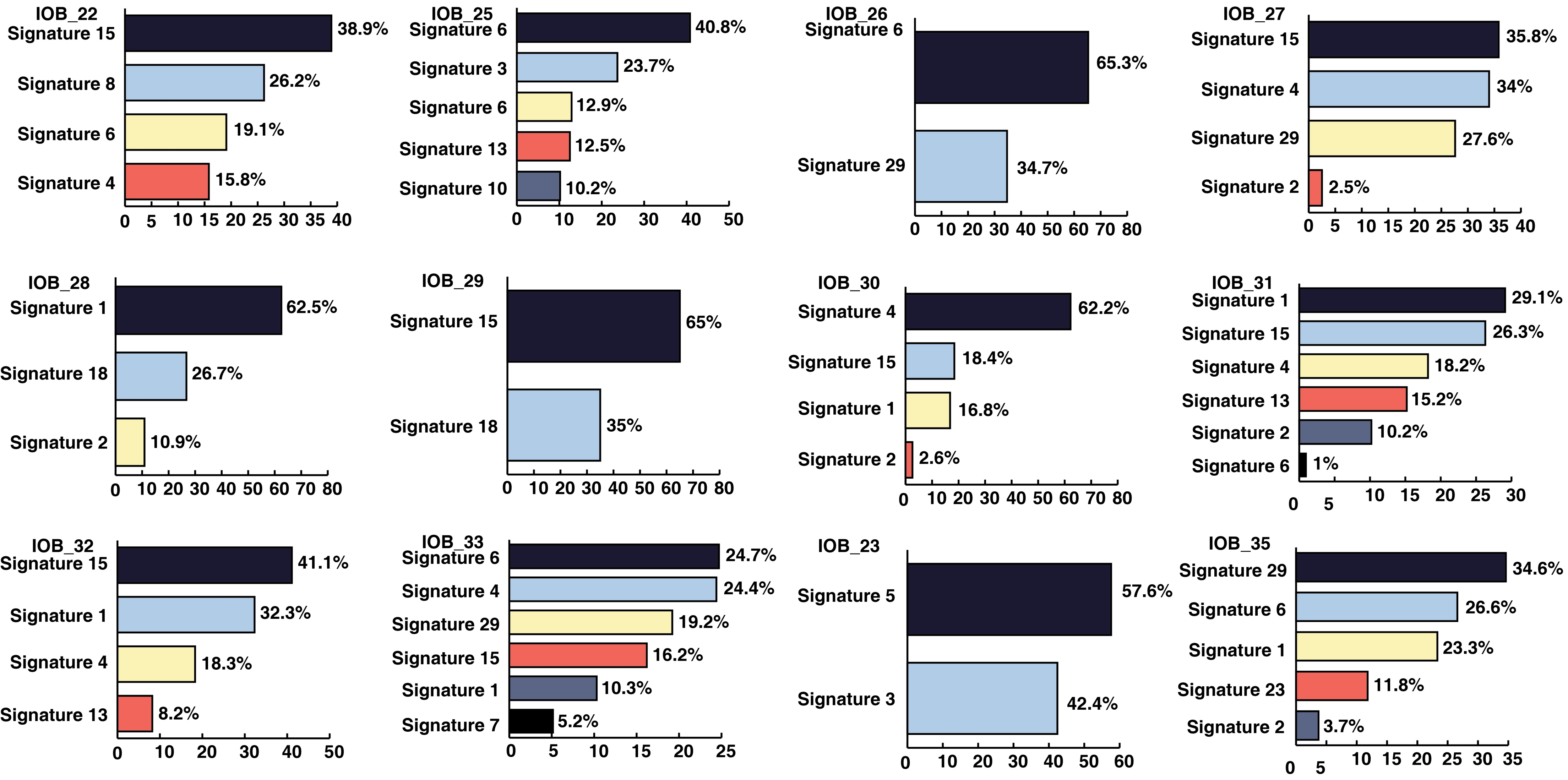

Supplementary figure 4

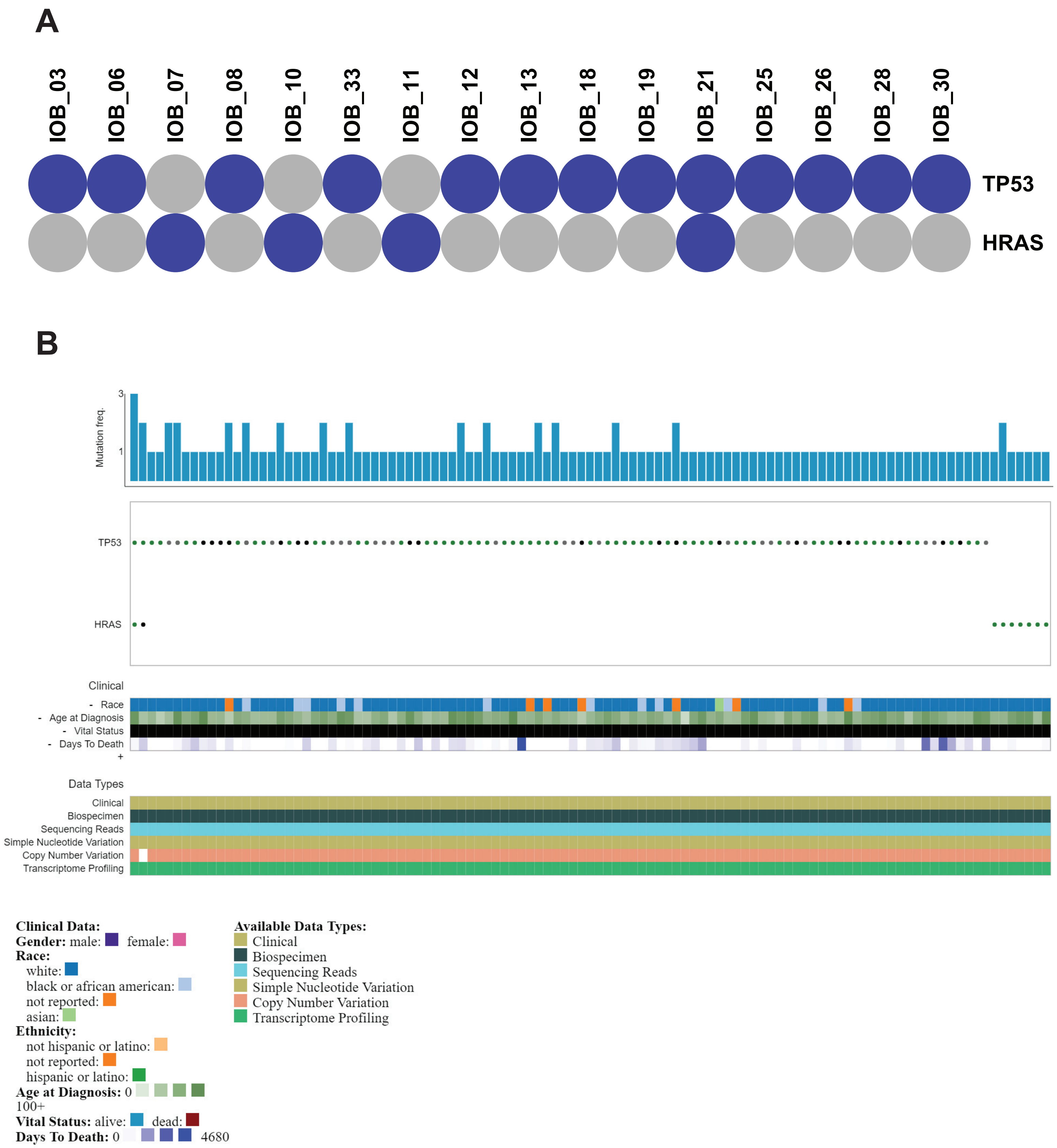

Supplementary figure 5

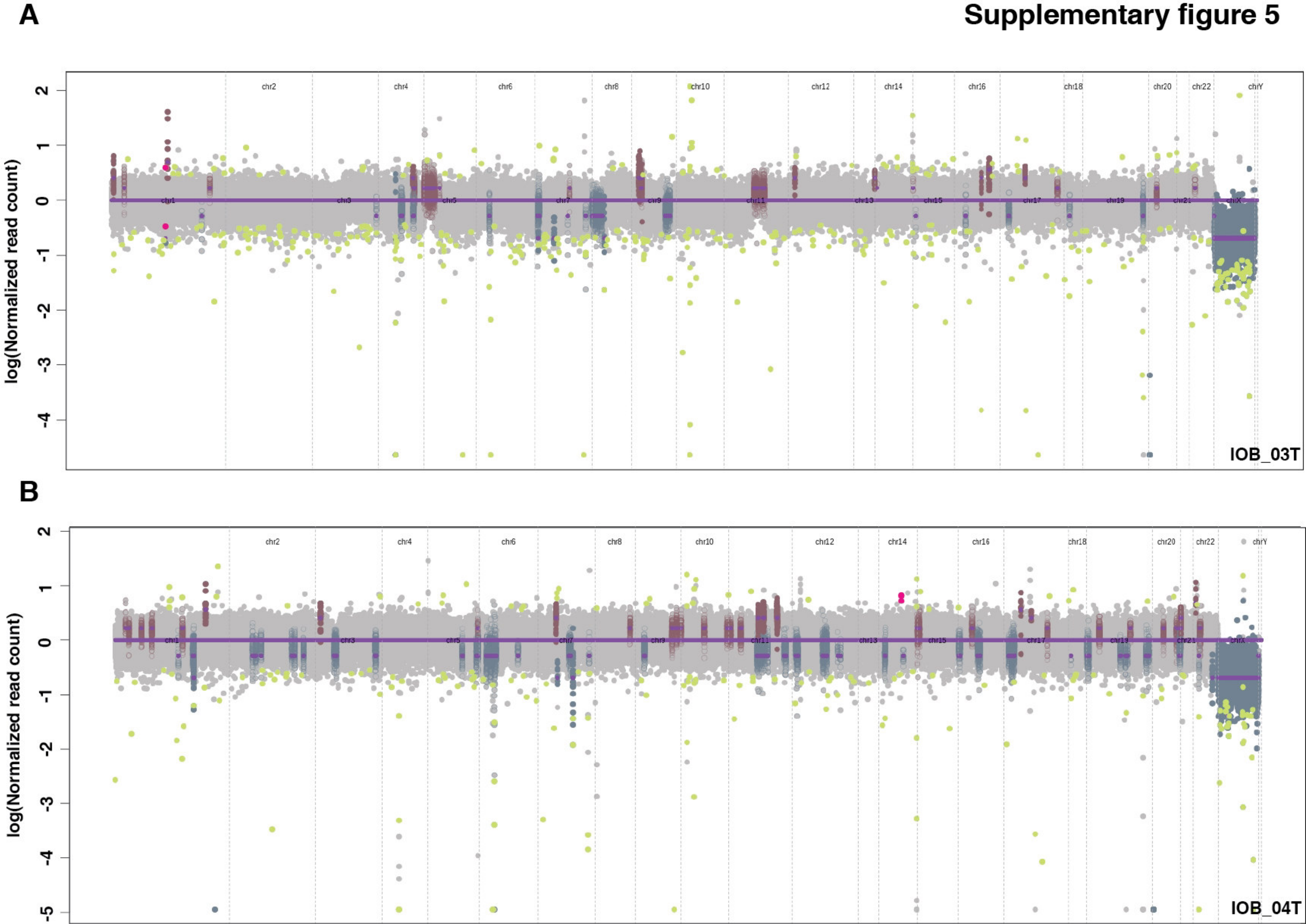

Supplementary figure 5

C

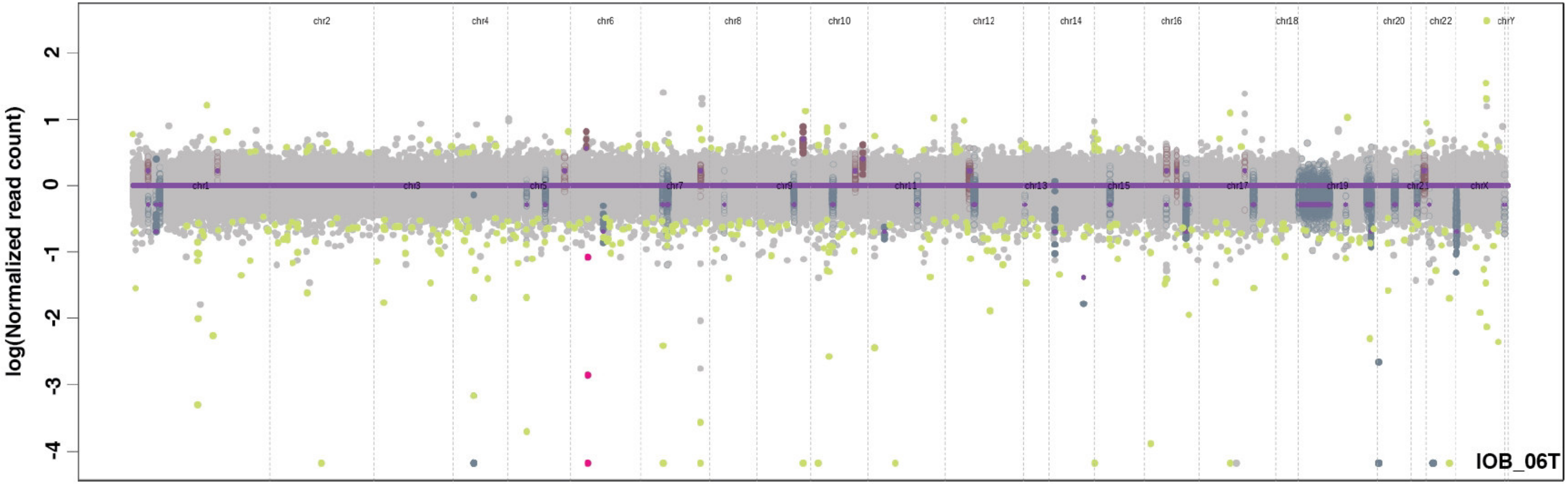

D

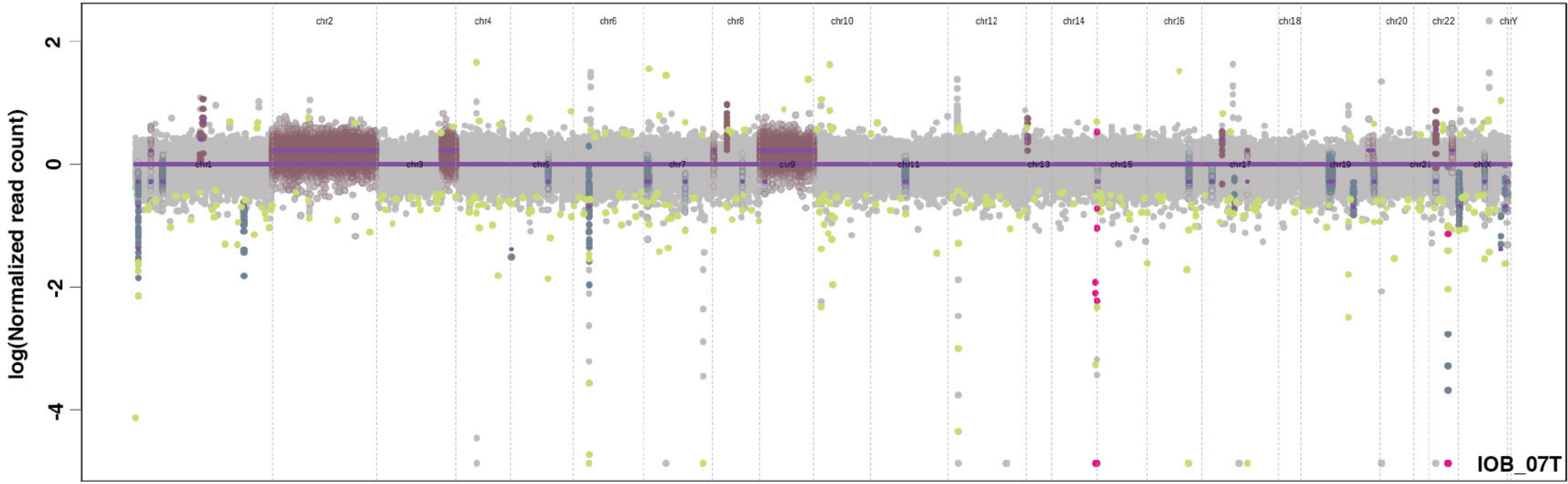

Supplementary figure 5

E

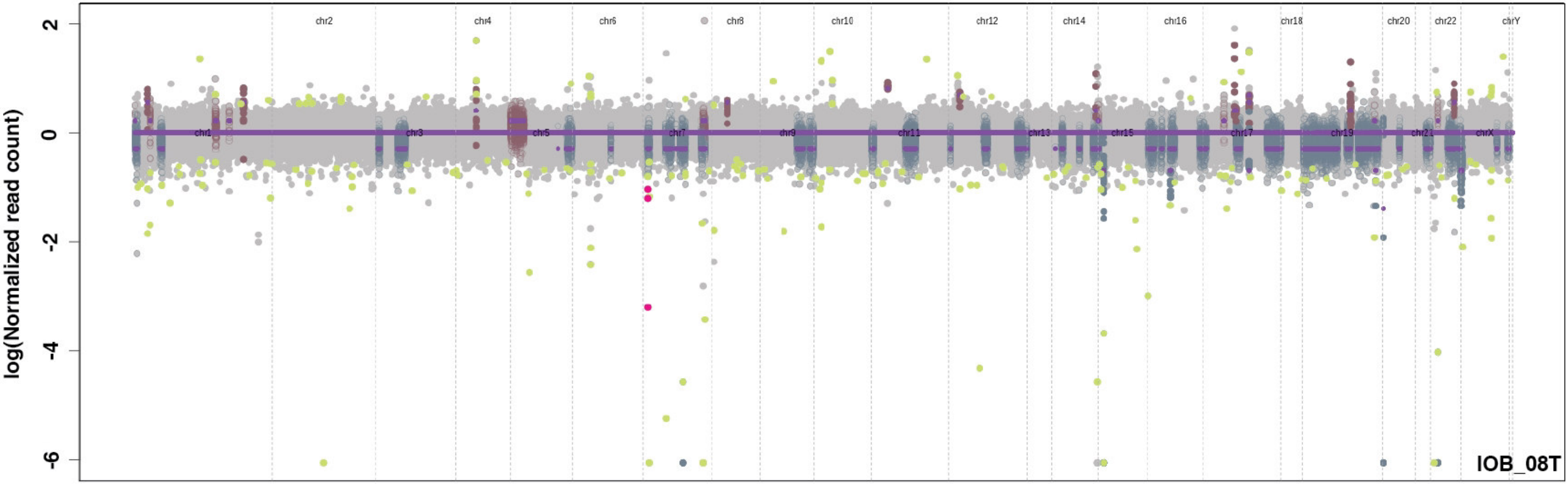

F

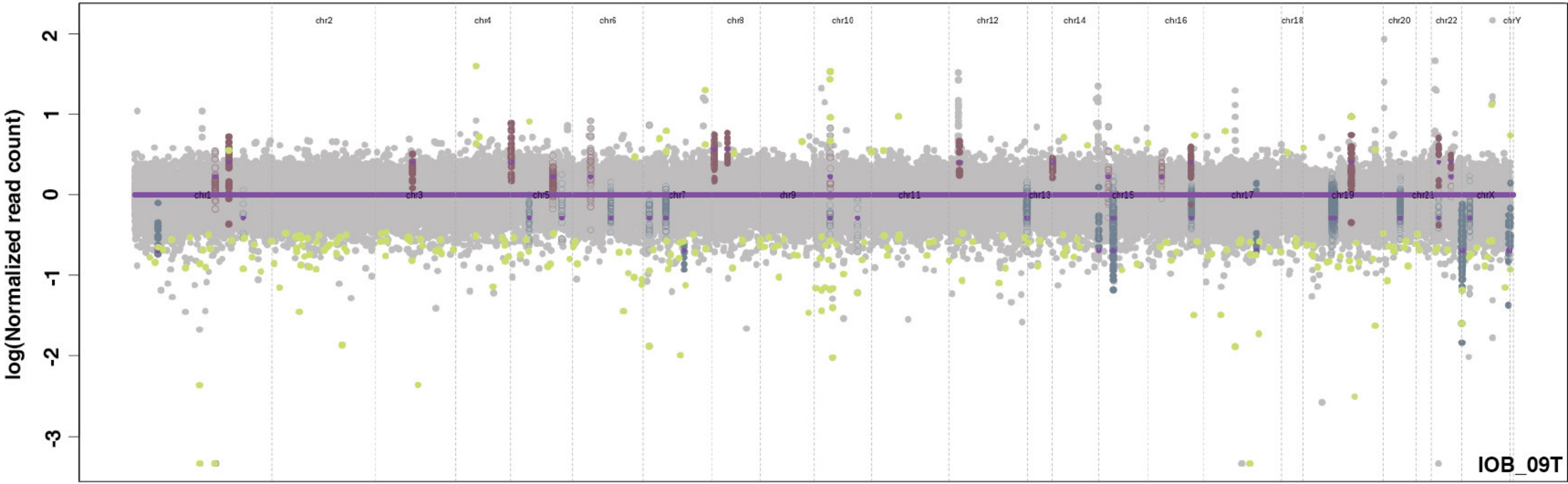

Supplementary figure 5

G

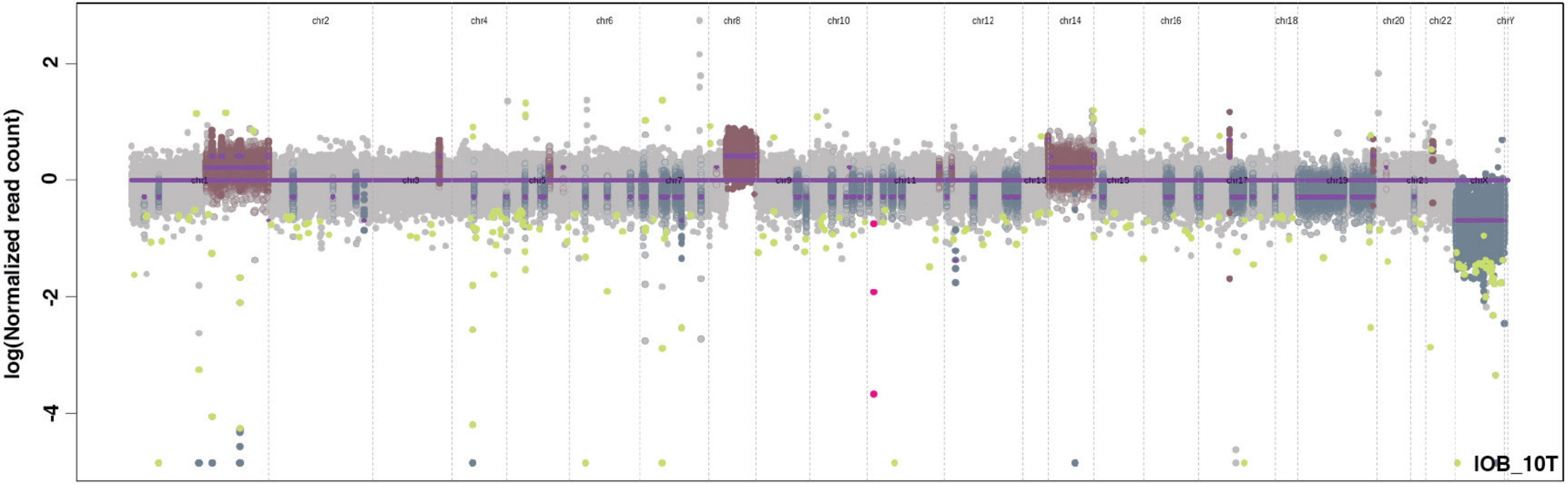

H

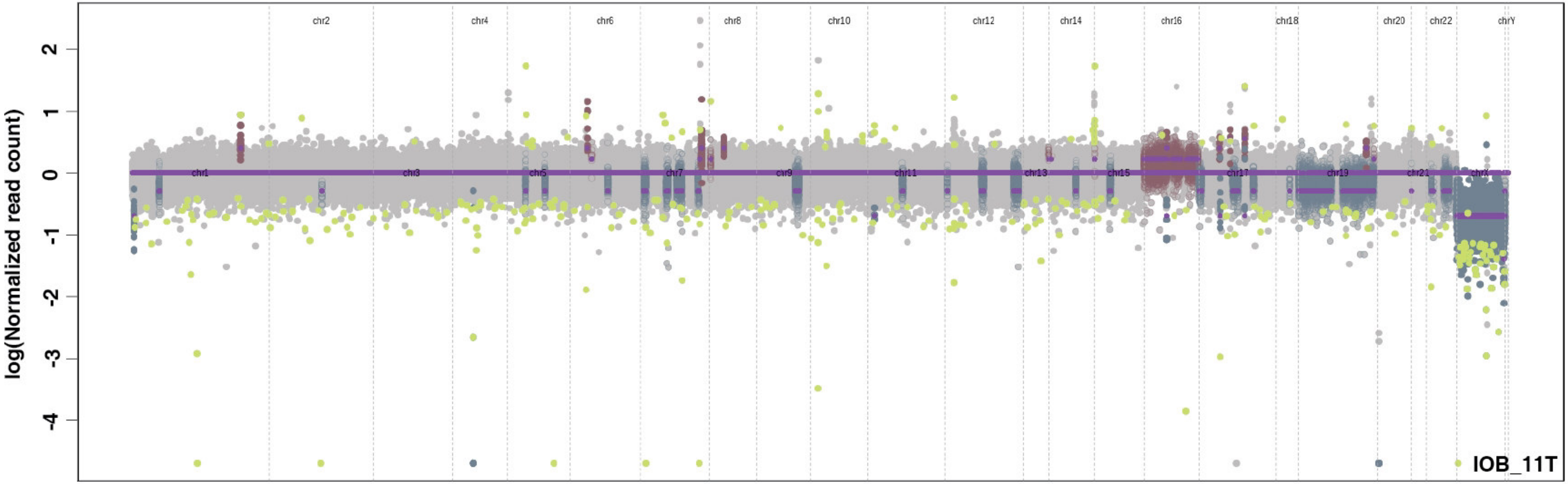

I

Supplementary figure 5

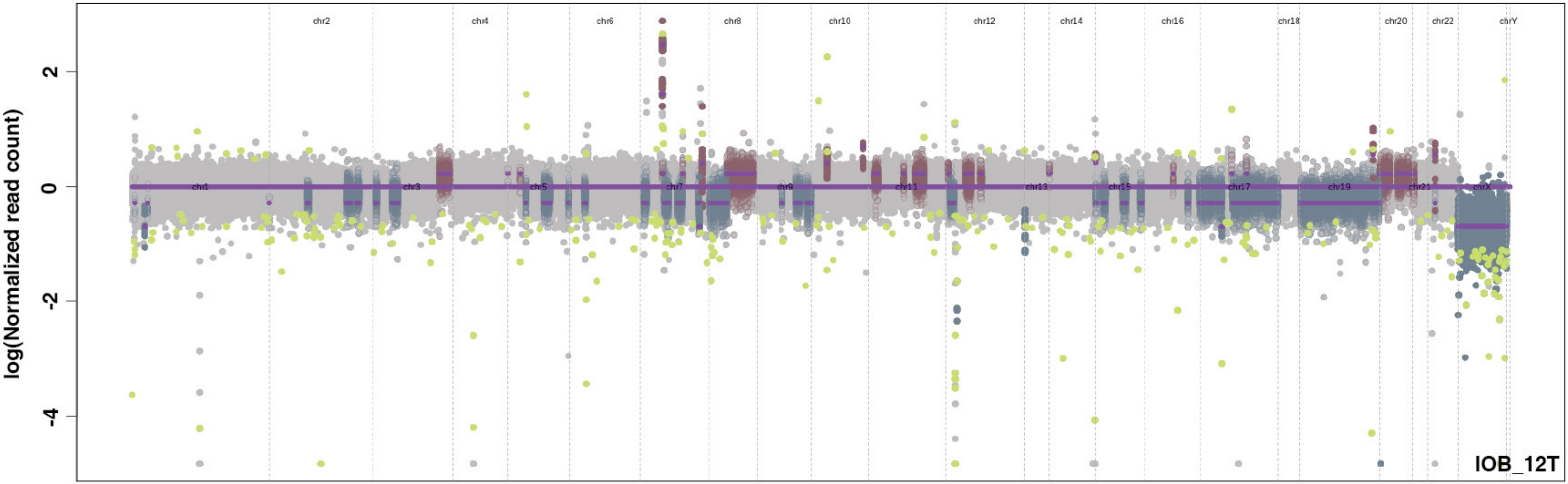

J

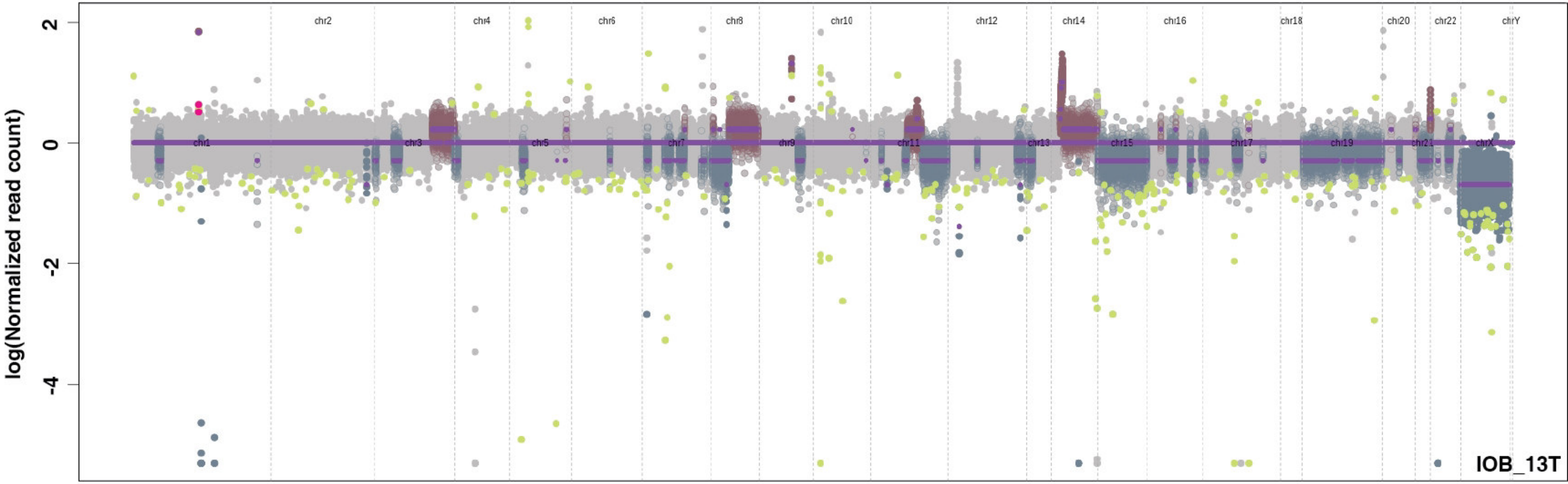

K

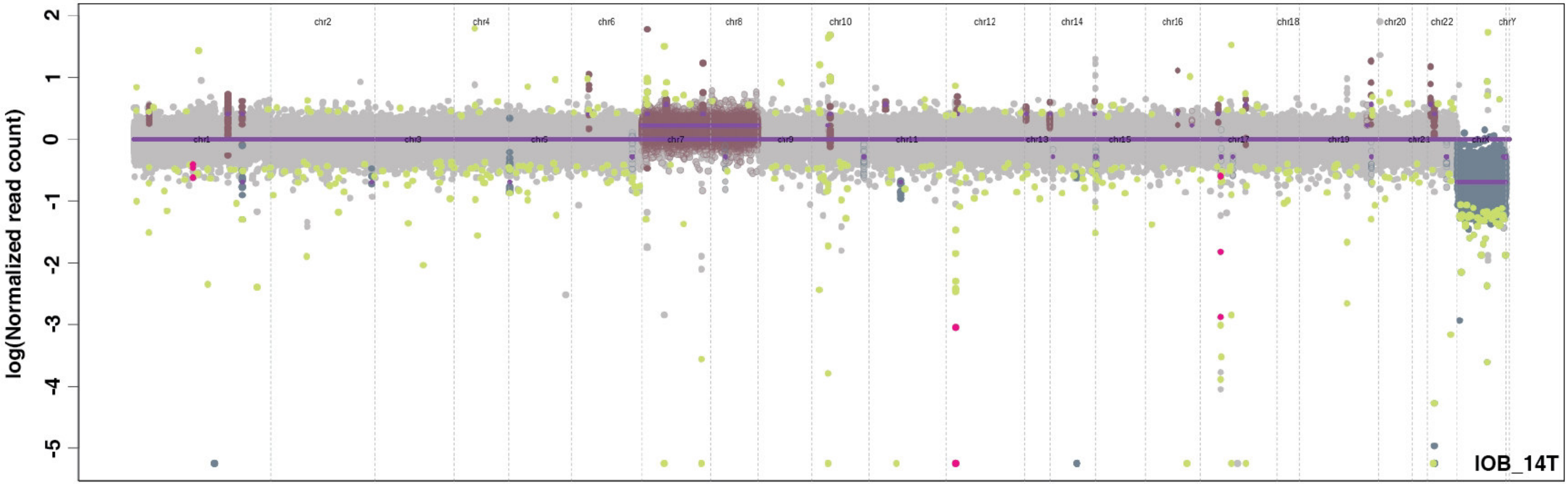
log(Normalized read count)
chr1
chr2
chr3
chr4
chr5
chr6
chr7
chr8
chr9
chr10
chr11
chr12
chr13
chr14
chr15
chr16
chr17
chr18
chr19
chr20
chr21
chr22
chrX
chrY
IOB_14T

L

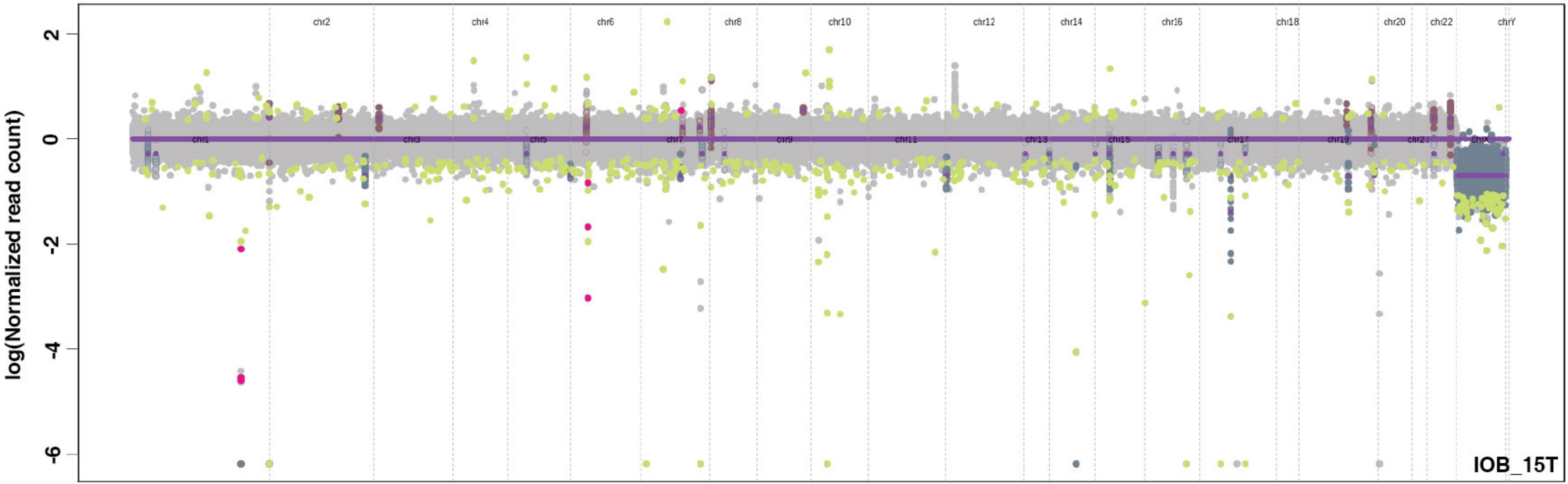
log(Normalized read count)
chr1
chr2
chr3
chr4
chr5
chr6
chr7
chr8
chr9
chr10
chr11
chr12
chr13
chr14
chr15
chr16
chr17
chr18
chr19
chr20
chr21
chr22
chrX
chrY
IOB_15T

M

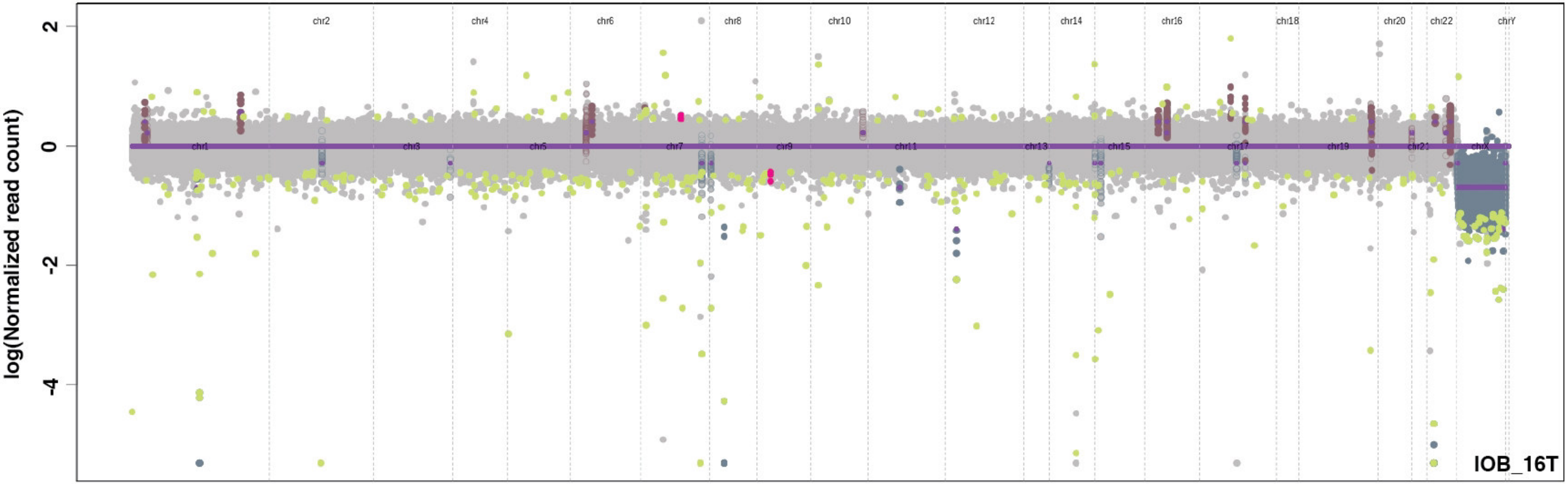
log(Normalized read count)
chr1
chr2
chr3
chr4
chr5
chr6
chr7
chr8
chr9
chr10
chr11
chr12
chr13
chr14
chr15
chr16
chr17
chr18
chr19
chr20
chr21
chr22
chrX
chrY
IOB_16T

N

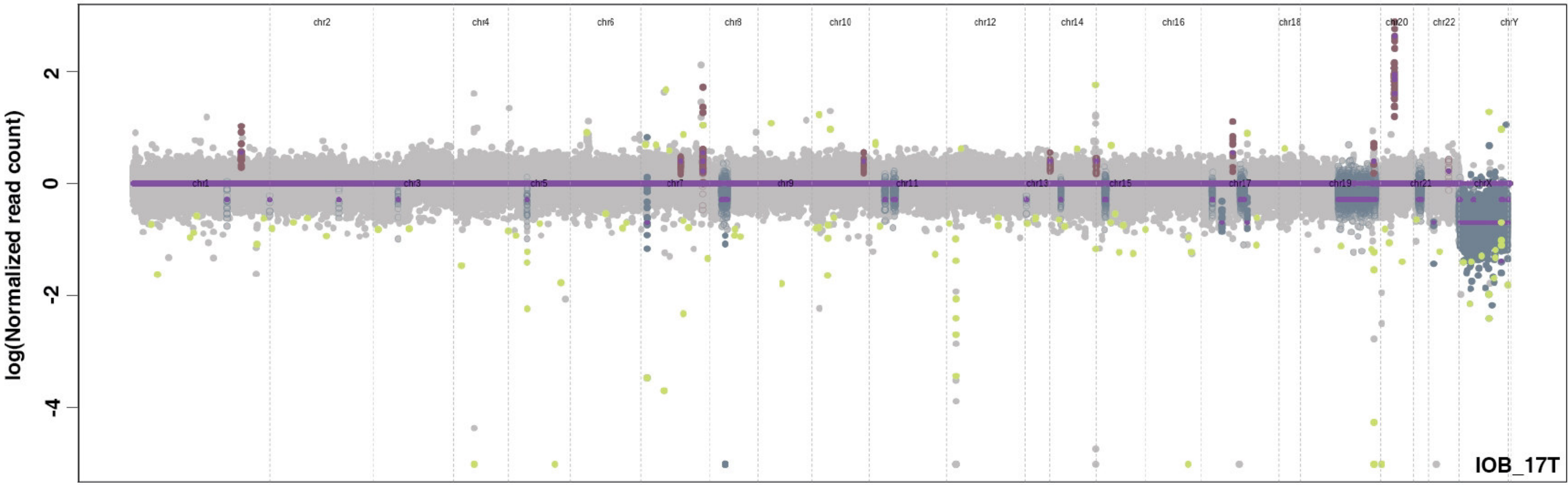
log(Normalized read count)
chr1
chr2
chr3
chr4
chr5
chr6
chr7
chr8
chr9
chr10
chr11
chr12
chr13
chr14
chr15
chr16
chr17
chr18
chr19
chr20
chr21
chr22
chrX
chrY
IOB_17T

Supplementary figure 5

O

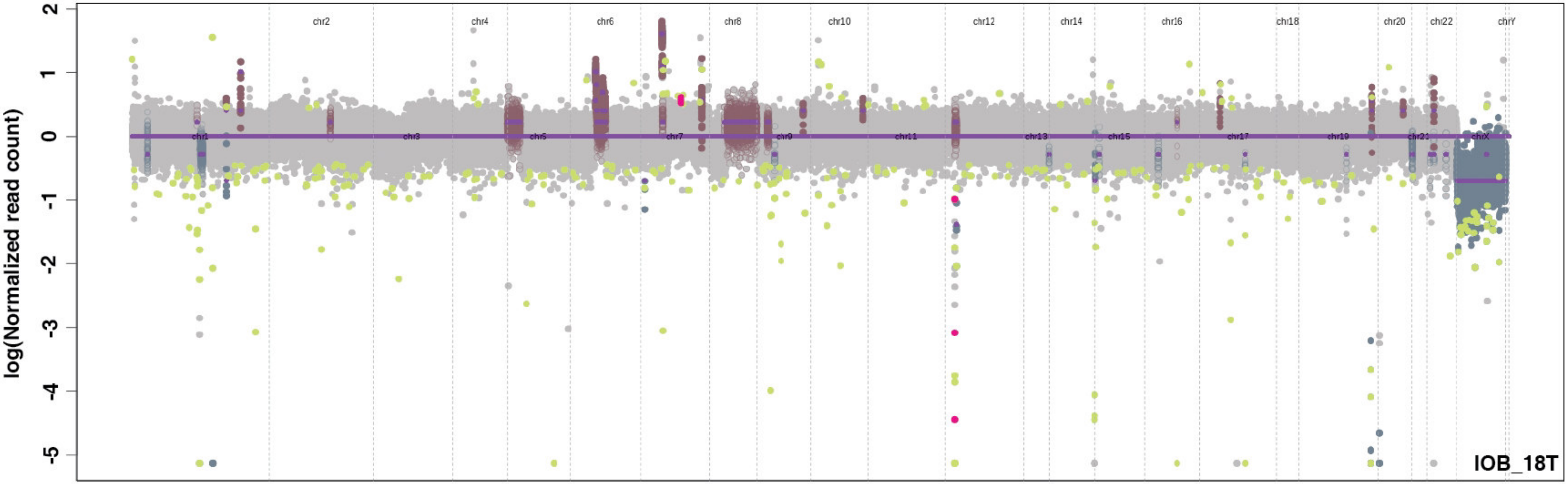

P

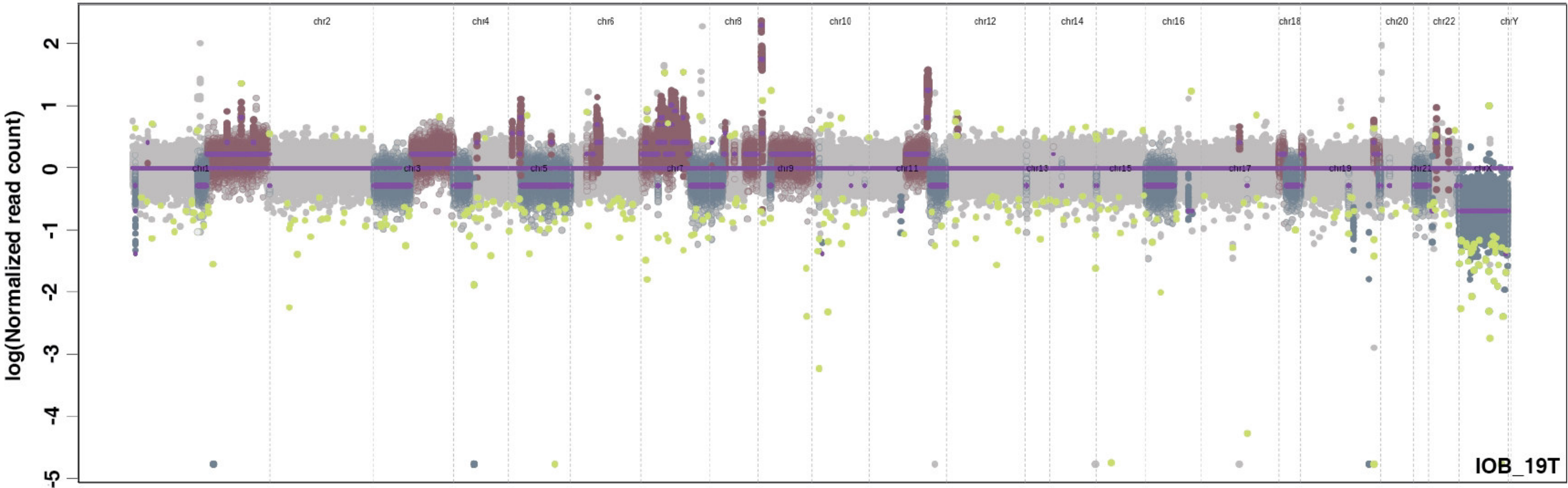

Q

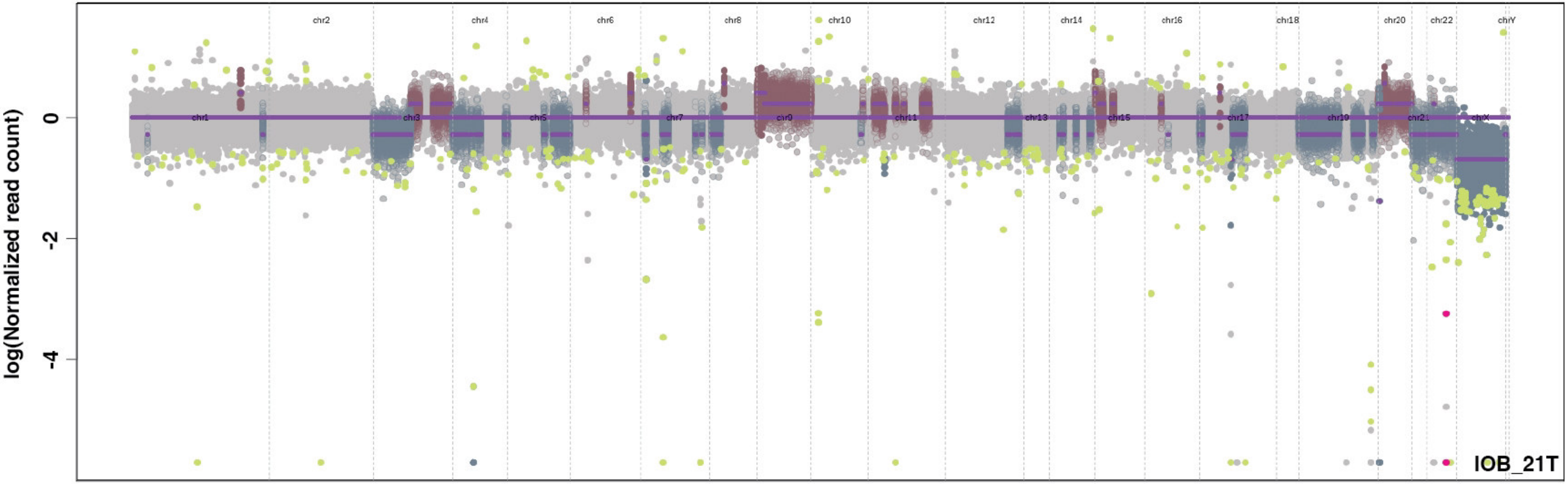
log(Normalized read count)
chr1
chr2
chr3
chr4
chr5
chr6
chr7
chr8
chr9
chr10
chr11
chr12
chr13
chr14
chr15
chr16
chr17
chr18
chr19
chr20
chr21
chr22
chrX
chrY
IOB_21T

R

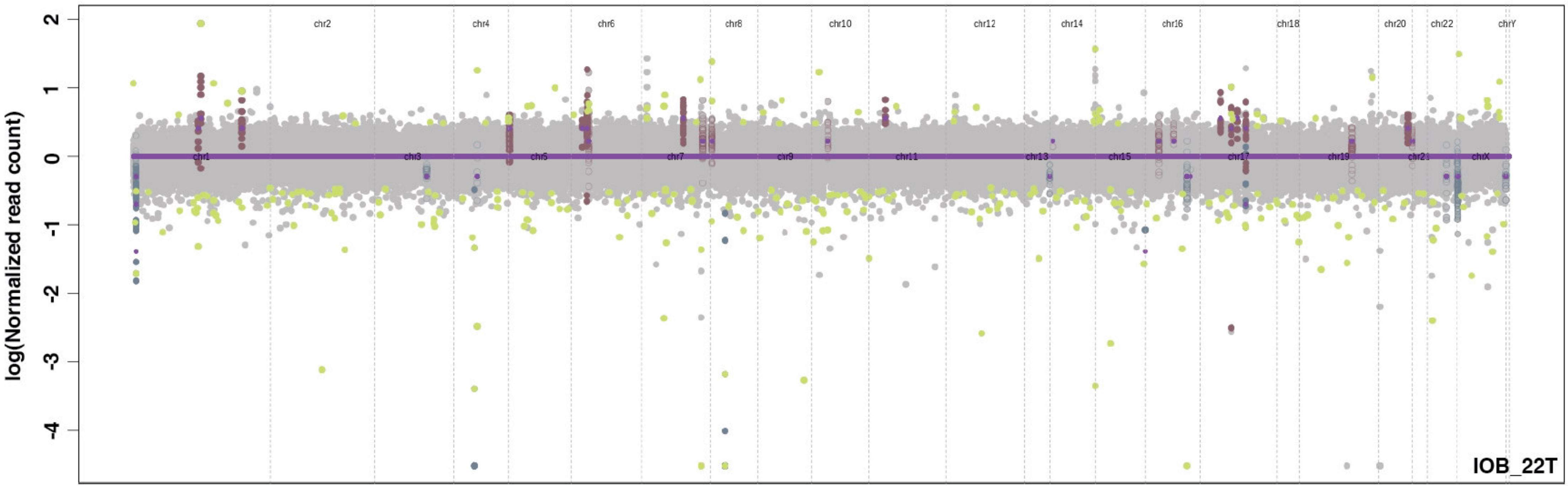
log(Normalized read count)
chr1
chr2
chr3
chr4
chr5
chr6
chr7
chr8
chr9
chr10
chr11
chr12
chr13
chr14
chr15
chr16
chr17
chr18
chr19
chr20
chr21
chr22
chrX
chrY
IOB_22T

Supplementary figure 5

S

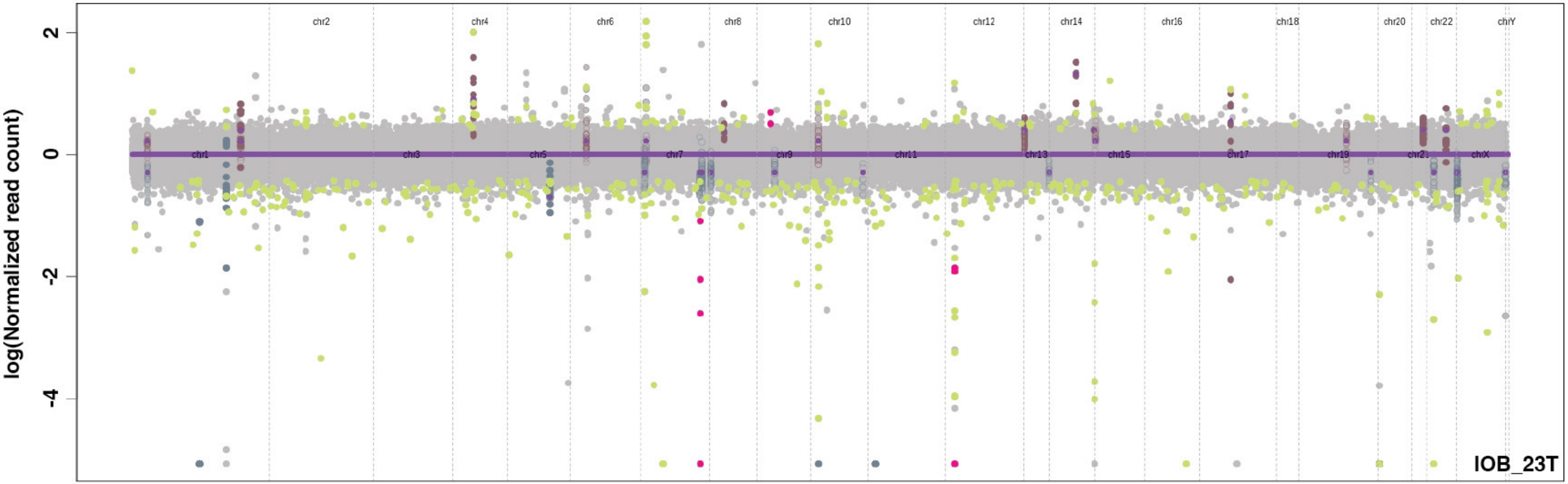

T

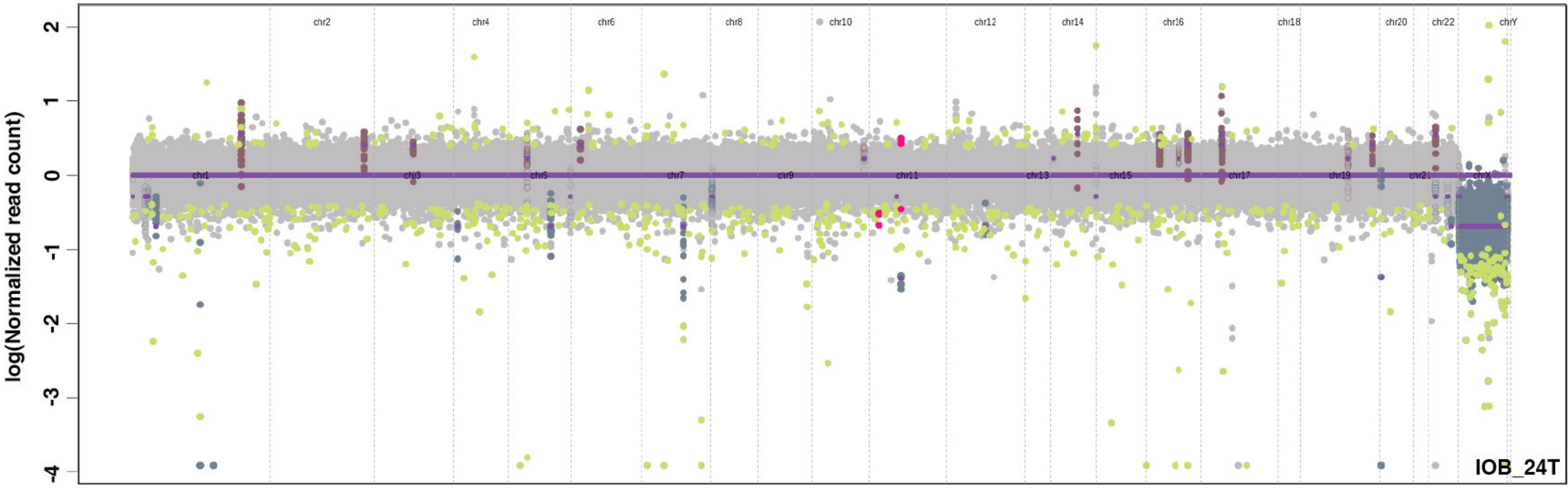

U

Supplementary figure 5

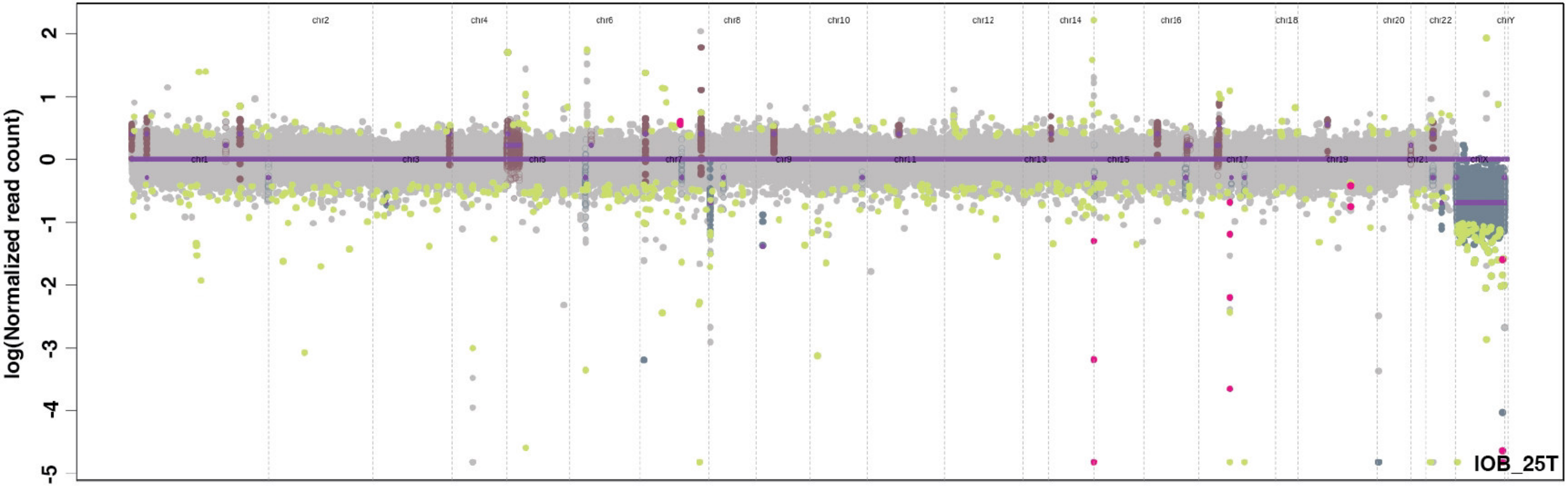

V

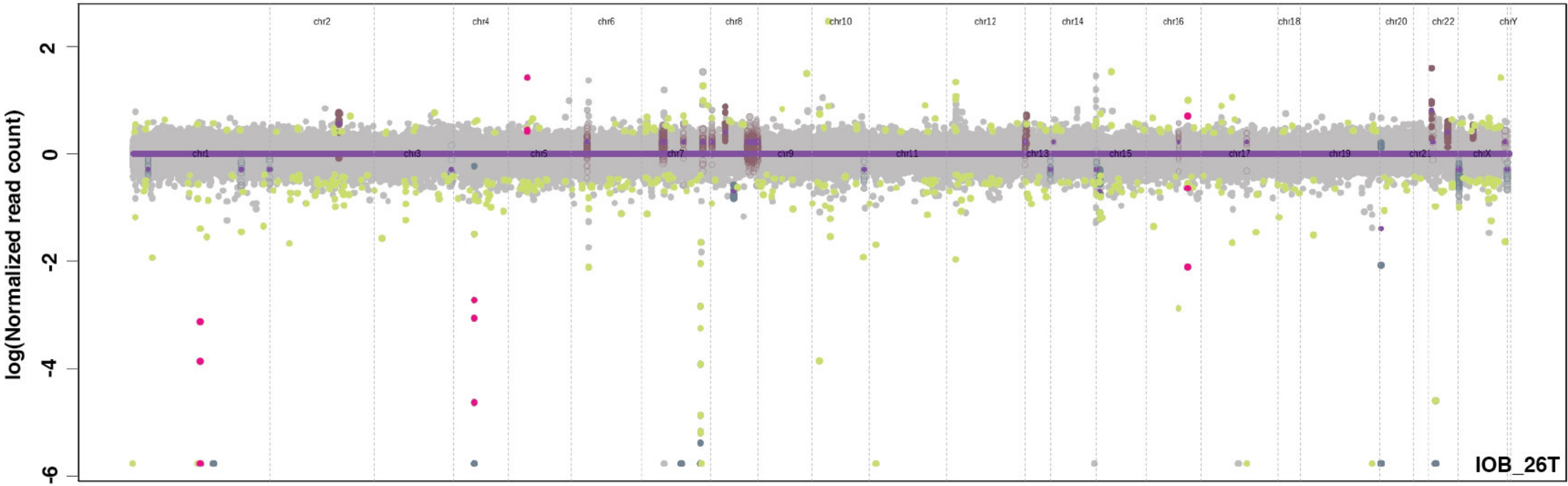

Supplementary figure 5

X

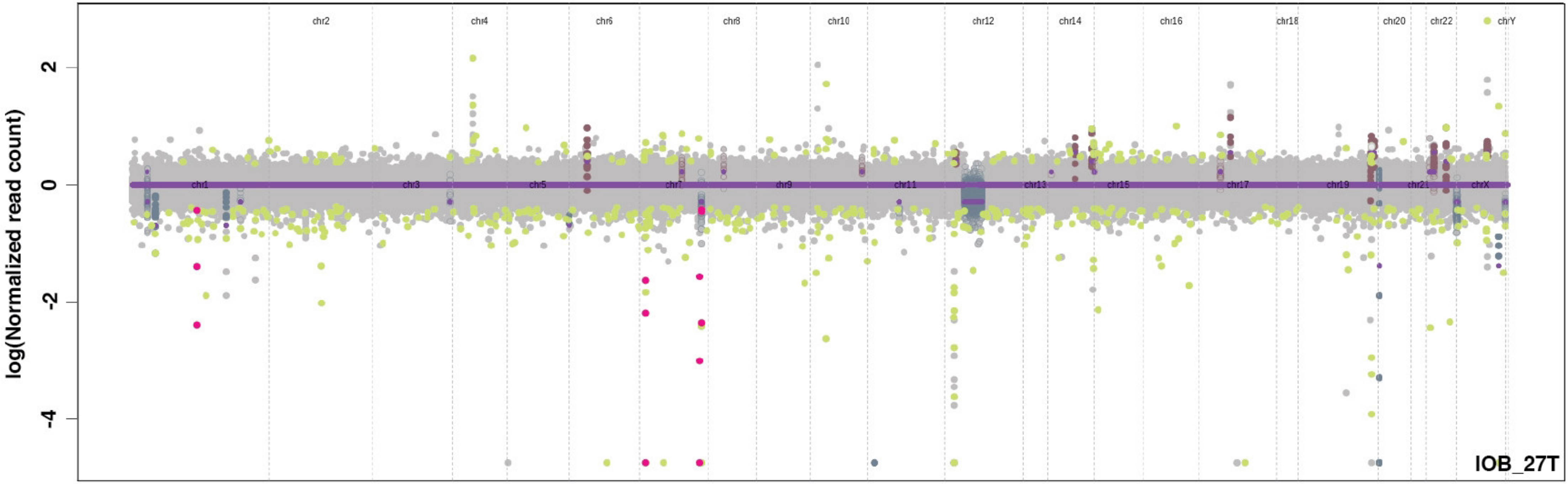

Y

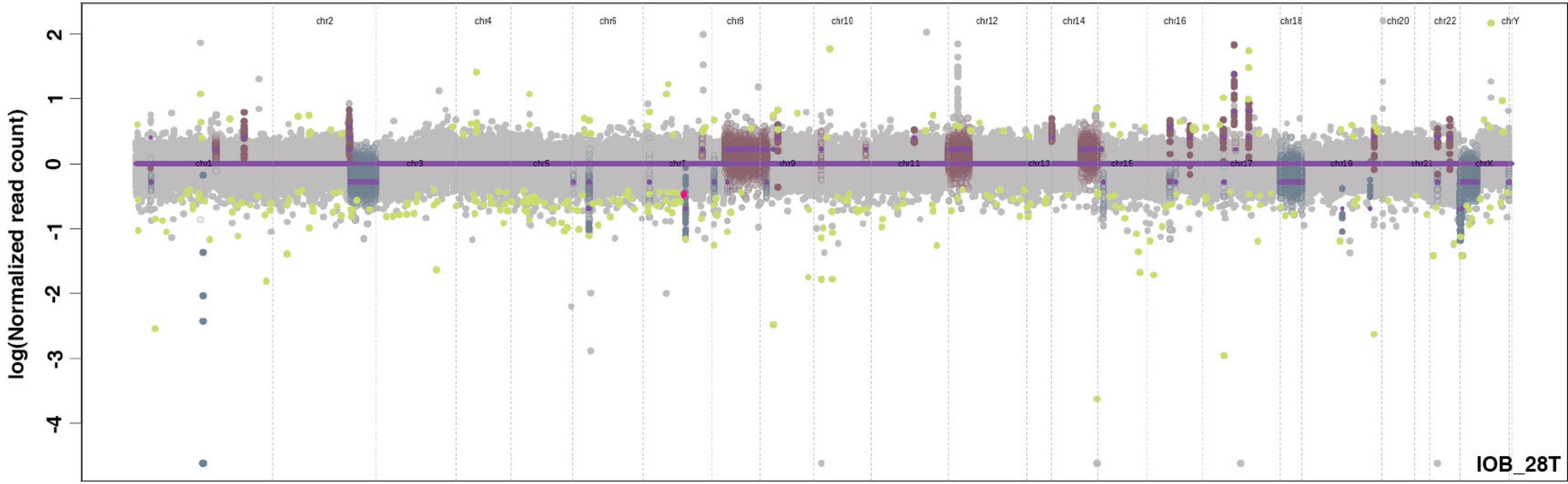

Z

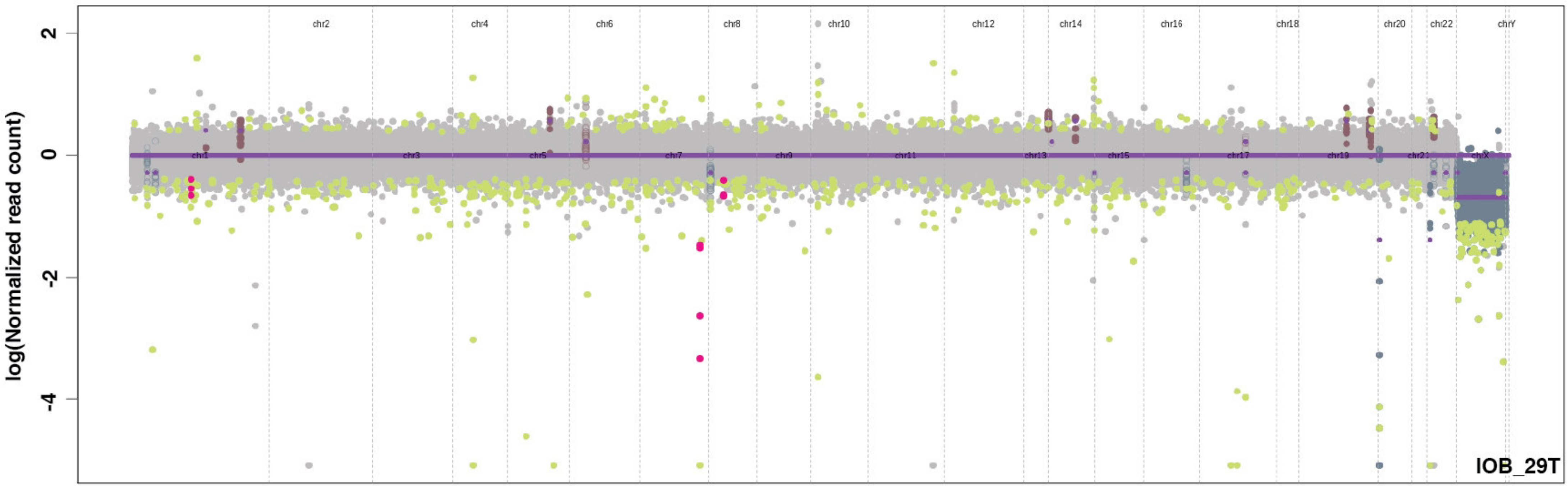
log(Normalized read count)
2
0
-2
-4
chr1
chr2
chr3
chr4
chr5
chr6
chr7
chr8
chr9
chr10
chr11
chr12
chr13
chr14
chr15
chr16
chr17
chr18
chr19
chr20
chr21
chr22
chrX
chrY
IOB_29T

AA

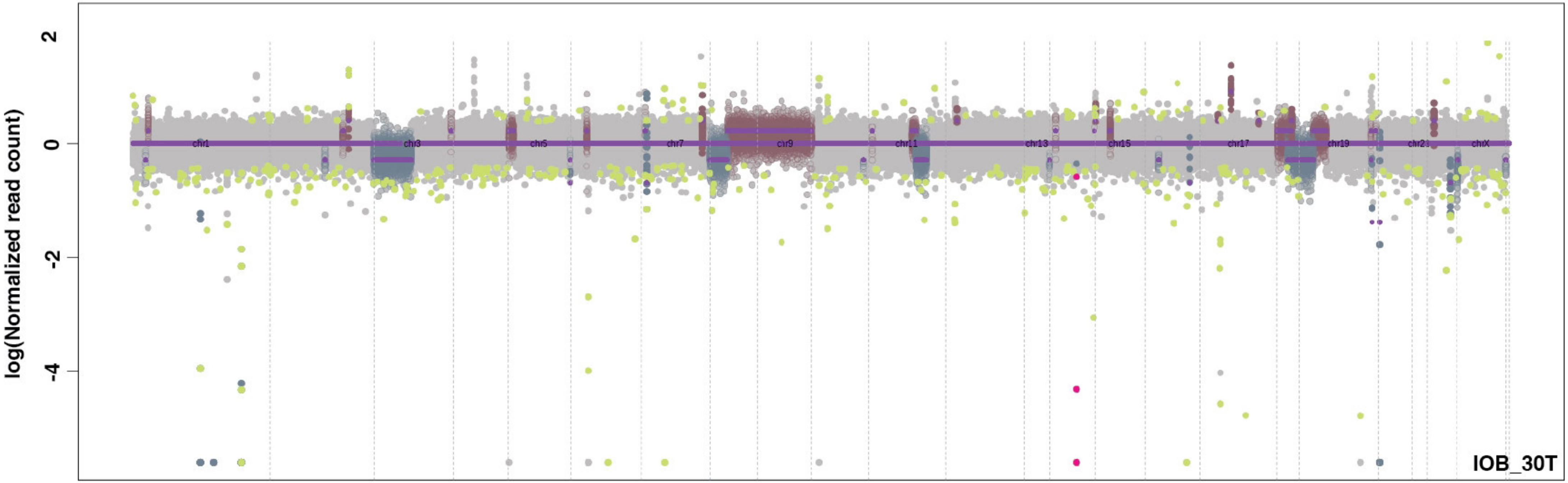
log(Normalized read count)
2
0
-2
-4
chr1
chr3
chr5
chr7
chr9
chr11
chr13
chr15
chr17
chr19
chr21
chrX
IOB_30T

Supplementary figure 5

AB

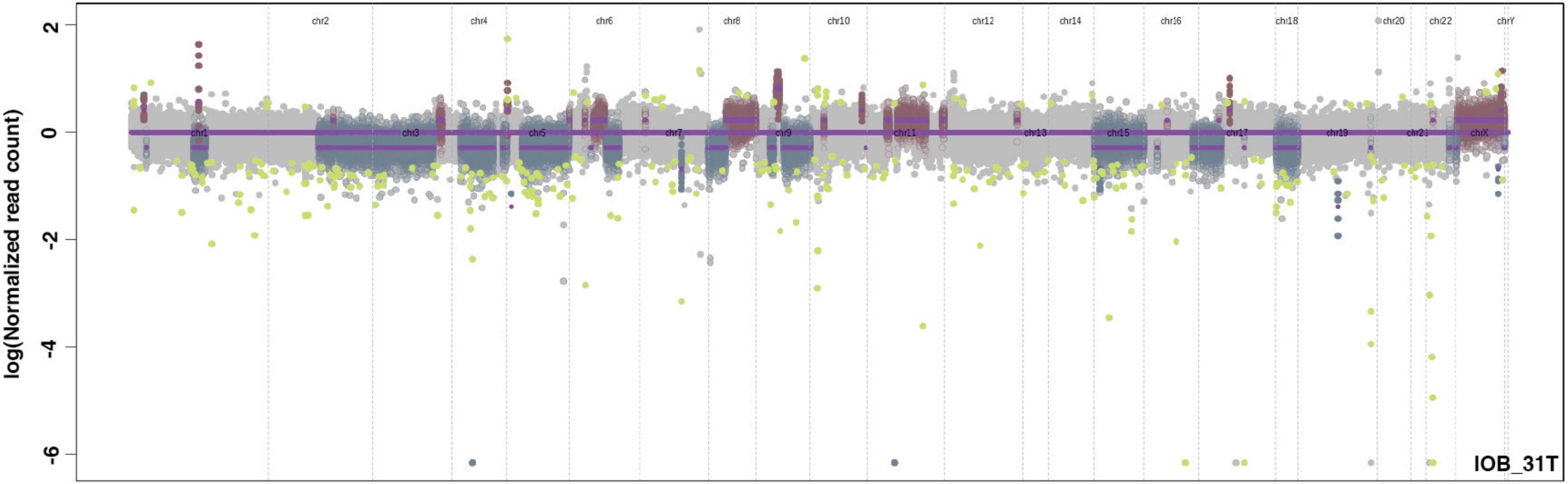

AC

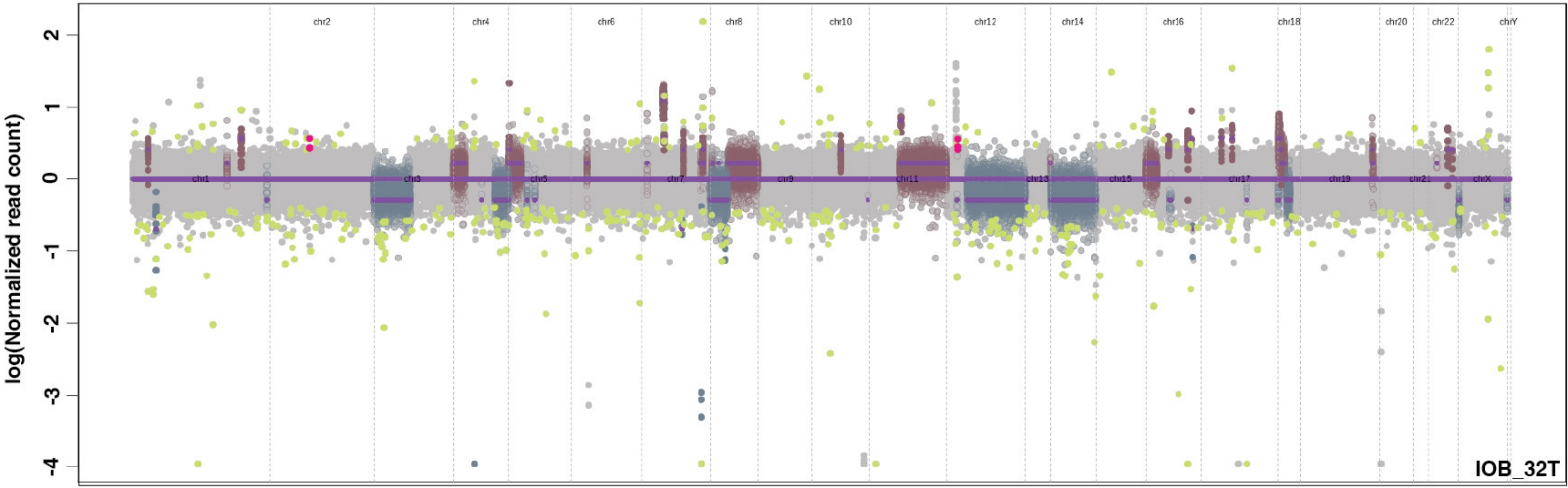

AD **Supplementary figure 5**

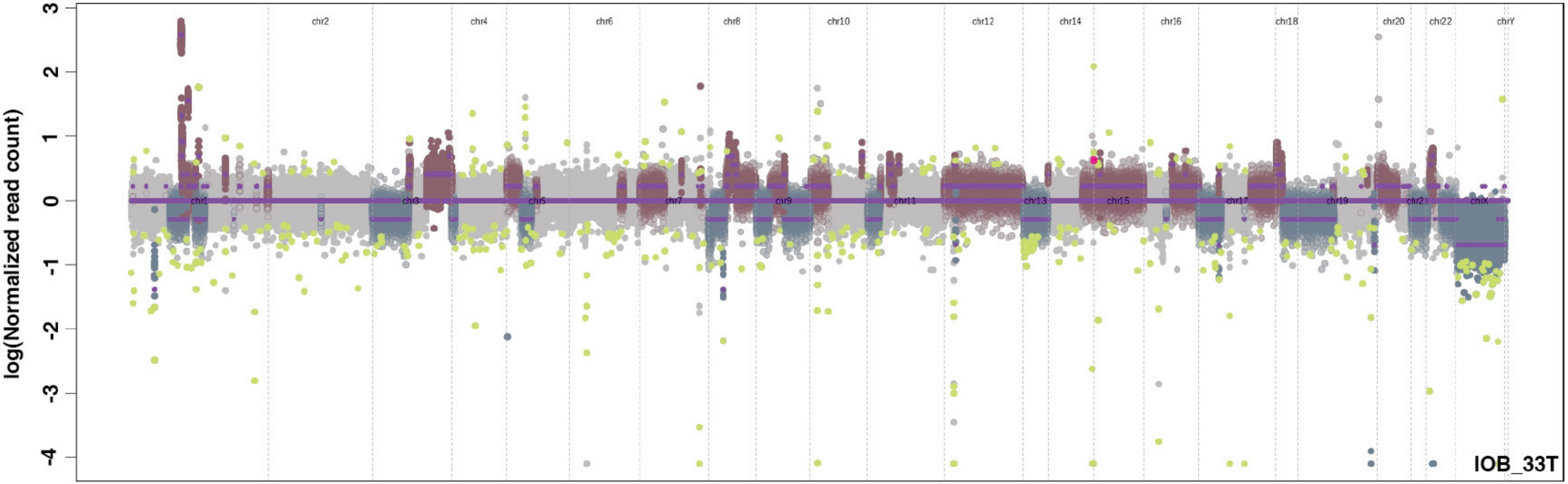

AE

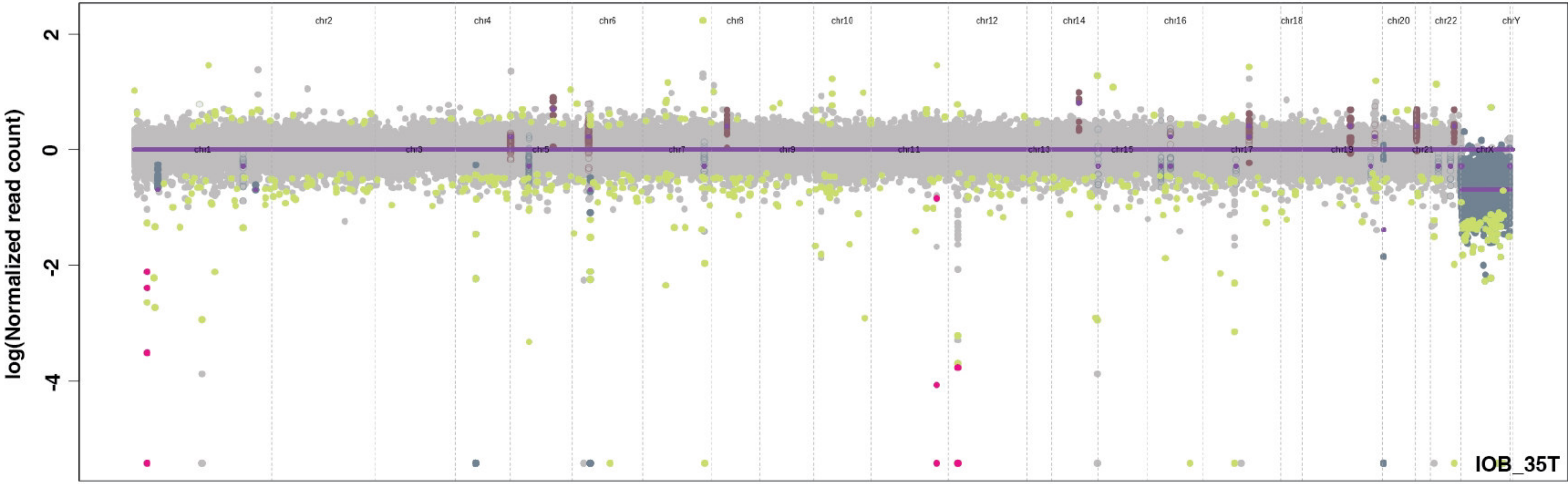

Supplementary figure 6

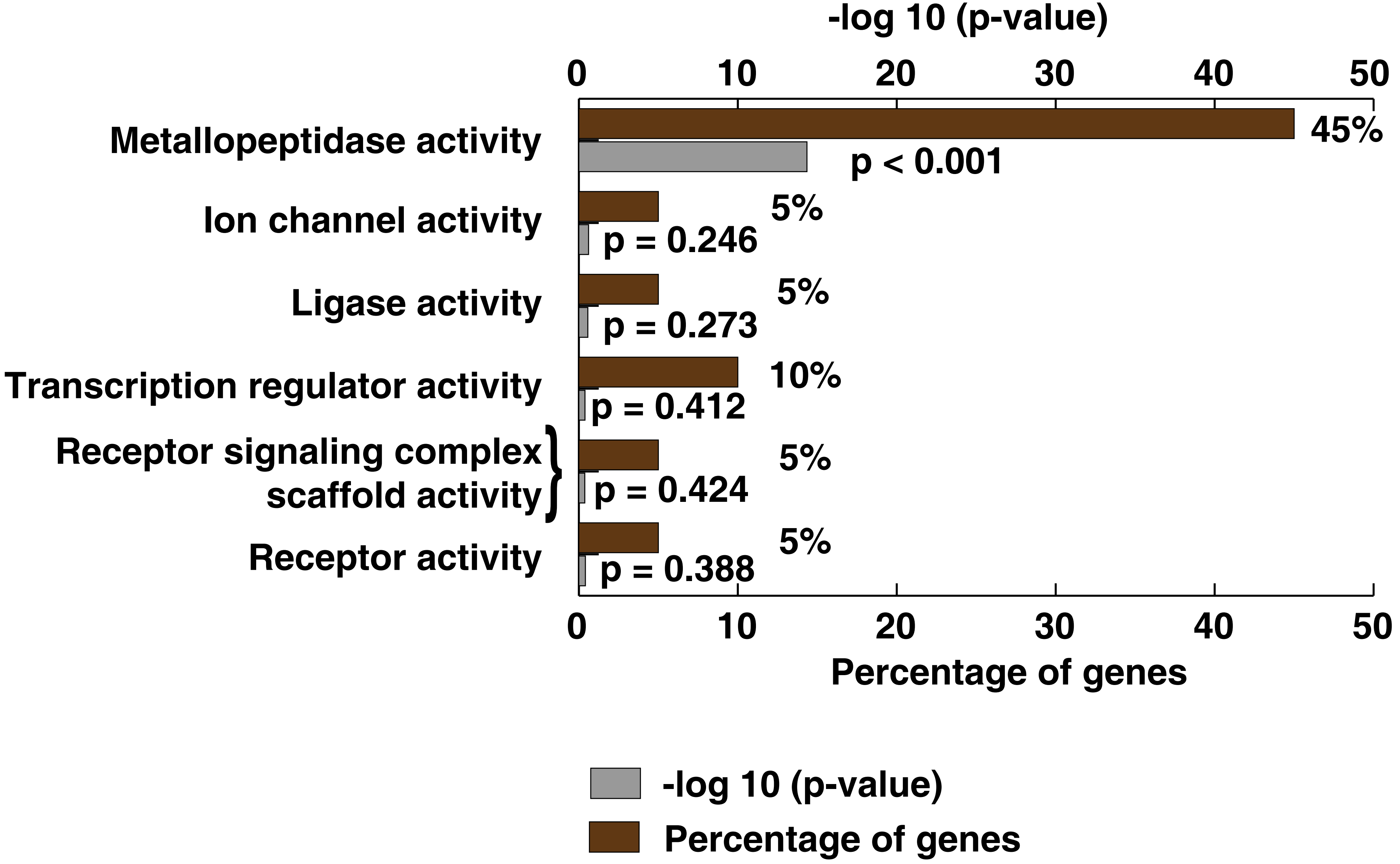

Supplement: Supplementary Figure 1 — Distribution of different type of variants identified in OSCC whole-exome sequencing data. [file DataSheet_1.pdf]
