# Supplementary material for: Whole-Exome Sequencing Analysis of Oral Squamous Cell Carcinoma Delineated by Tobacco Usage Habits
Source: Front Oncol. 2021 May 31;11:660696. doi: 10.3389/fonc.2021.660696 (PMC8200776; doi:10.3389/fonc.2021.660696)
Supplement: Supplementary file 4 [file Table_4.pdf]

Patel *et al.* , 2021. Whole-exome sequencing analysis of oral squamous cell carcinoma delineated by tobacco usage habits
Supplementary Table 4. List of somatic single nucleotide variants identified using whole exome sequencing of OSCC patients

Cohort	Sample	Chromosome	Genomic position	Reference allele	Alternate allele	Genesymbol	Variant type	Variant description	OncoMD	COSMIC	dbSNP	ExAC	ExAC_SAS	1000G	1000G_SAS	SIFT	Polyphen2	CONDEL	VEP	LRT	PhastCon7Way	Total reads in Normal	Reference read support in Normal	Alternative read support in Normal	Total reads in Tumor	Reference read support in Tumor	Alternate read support in Tumor	Alternate allele frequency in Tumor
Chewer	IOB_03	chr1	1229831	G	A	ACAP3	MISSENSE	ENSG00000131584:[ENST00000354700(c.2090C>T,gCg>gTg):ENSP00000346733(p.Ala697Val)]	NA	NA	NA	NA	NA	NA	NA	NA	NA	NA	MODIFIER	NA	NA	289	289	0	453	445	8	1.77
Chewer	IOB_03	chr1	2451739	G	T	PANK4	INTRONIC	ENSG00000157881:[ENST00000486396(c.*478+22C>A)]	NA	NA	NA	NA	NA	NA	NA	NA	NA	NA	MODIFIER	NA	NA	147	147	0	167	145	22	13.17
Chewer	IOB_03	chr1	11561667	G	C	PTCHD2	MISSENSE	ENSG00000204624:[ENST00000294484(c.618G>C,gaG>gaC):ENSP00000294484(p.Glu206Asp)]	NA	NA	NA	NA	NA	NA	NA	T_lc	BN	NA	MODERATE	NA	NA	291	291	0	328	282	44	13.41
Chewer	IOB_03	chr1	43317157	C	A	ZNF691	MISSENSE	ENSG00000164011:[ENST00000372506(c.528C>A,agC>agA):ENSP00000361584(p.Ser176Arg)]	NA	NA	NA	NA	NA	NA	NA	D	PoD	NA	MODERATE	D	1	256	255	1	344	337	7	2.03
Chewer	IOB_03	chr1	68697988	C	T	WLS	INTRONIC	ENSG00000116729:[ENST00000530486(c.-30+355G>A)]	NA	NA	NA	2.51E-05	0.00E+00	NA	NA	NA	NA	NA	MODIFIER	NA	NA	153	153	0	180	175	5	2.78
Chewer	IOB_03	chr1	152191949	C	T	FLG-AS1	NCT	ENSG00000237975:[ENST00000593011(n.296+51053C>T)]	NA	NA	NA	8.24E-06	0.00E+00	NA	NA	NA	NA	NA	MODIFIER	NA	NA	172	171	0	199	194	5	2.51
Chewer	IOB_03	chr1	152191949	C	T	HRNR	MISSENSE	ENSG00000197915:[ENST00000368801(c.2156G>A,gGc>gAc):ENSP00000357791(p.Gly719Asp)]	NA	NA	NA	8.24E-06	0.00E+00	NA	NA	NA	NA	NA	MODIFIER	NA	NA	172	171	0	199	194	5	2.51
Chewer	IOB_03	chr1	158644141	G	T	SPTA1	MISSENSE	ENSG00000163554:[ENST00000368147(c.1328C>A,gCc>gAc):ENSP00000357129(p.Ala443Asp)]	NA	NA	NA	8.28E-06	6.06E-05	NA	NA	T	PoD	NA	MODERATE	N	1	279	278	1	277	268	7	2.53
Chewer	IOB_03	chr1	161683056	C	A	FCRLA	SILENT	ENSG00000132185:[ENST00000367949(c.465C>A,ggC>ggA):ENSP00000356926(p.Gly155(=))]	NA	NA	chr1*161683056*161683056*C*G,T*rs765950399*NA*NA*NA	NA	NA	NA	NA	NA	NA	NA	MODIFIER	NA	NA	99	99	0	113	108	5	4.42
Chewer	IOB_03	chr1	162039948	C	T	NOS1AP	5UTR	ENSG00000198929:[ENST00000361897(c.-20C>T)]	NA	NA	NA	NA	NA	NA	NA	NA	NA	NA	MODIFIER	NA	NA	252	251	1	290	283	7	2.41
Chewer	IOB_03	chr1	196658735	C	A	CFH	MISSENSE	ENSG00000000971:[ENST00000359637(c.958C>A,Cca>Aca):ENSP00000352658(p.Pro320Thr)]	NA	NA	chr1*196658735*196658735*C*T*rs778180494*NA*NA*NA	NA	NA	NA	NA	D	PrD	NA	MODERATE	NA	NA	110	110	0	127	122	5	3.94
Chewer	IOB_03	chr1	201338437	G	A	TNNT2	NCT	ENSG00000118194:[ENST00000494095(n.2285-1082C>T)]	NA	NA	1.20E-03	1.16E-03	2.40E-03	1.20E-03	0.00E+00	NA	NA	NA	MODIFIER	NA	NA	155	155	0	186	181	5	2.69
Chewer	IOB_03	chr1	208041125	G	A	C1orf132	NCT	ENSG00000203709:[ENST00000415882(n.221+146C>T)]	NA	NA	chr1*208041125*208041125*G*C*rs975539763*NA*NA*NA	NA	NA	NA	NA	NA	NA	NA	MODIFIER	NA	NA	179	178	1	265	257	7	2.64
Chewer	IOB_03	chr1	228468099	G	A	OBSCN	MISSENSE	ENSG00000154358:[ENST00000359599(c.4424G>A,cGc>cAc):ENSP00000352613(p.Arg1475His)]	NA	NA	NA	2.13E-05	0.00E+00	NA	NA	NA	NA	NA	MODERATE	NA	0	157	157	0	208	202	5	2.4
Chewer	IOB_03	chr1	228503748	C	T	OBSCN	MISSENSE	ENSG00000154358:[ENST00000570156(c.16084C>T,Cgg>Tgg):ENSP00000455507(p.Arg5362Trp)]	NA	NA	chr1*228503748*228503748*C*A,G,T*rs765073723*NA*NA*NA	1.17E-04	0.00E+00	NA	NA	NA	PrD	NA	MODERATE	N	0.09	213	213	0	336	328	7	2.08
Chewer	IOB_03	chr1	237780641	C	A	RYR2	MISSENSE	ENSG00000198626:[ENST00000366574(c.5771C>A,gCc>gAc):ENSP00000355533(p.Ala1924Asp)]	NA	NA	chr1*237780641*237780641*C*G*rs776358808*NA*NA*NA	NA	NA	NA	NA	NA	PrD	NA	MODERATE	D	1	182	181	0	222	215	6	2.7
Chewer	IOB_03	chr1	246823585	C	T	CNST	MISSENSE	ENSG00000162852:[ENST00000366513(c.1921C>T,Cca>Tca):ENSP00000355470(p.Pro641Ser)]	NA	NA	NA	NA	NA	NA	NA	T	BN	NA	MODERATE	N	0.646	132	132	0	151	136	15	9.93
Chewer	IOB_03	chr10	18804063	C	T	CACNB2	INTRONIC	783:[XM_005252587.1(c.570+93C>T)]	NA	NA	NA	NA	NA	NA	NA	NA	NA	NA	MODIFIER	NA	NA	183	182	0	159	153	5	3.14
Chewer	IOB_03	chr10	18804063	C	T	RP11-499P20.2	NCT	ENSG00000240291:[ENST00000425669(n.483-1474G>A)]	NA	NA	NA	NA	NA	NA	NA	NA	NA	NA	MODIFIER	NA	NA	183	182	0	159	153	5	3.14
Chewer	IOB_03	chr10	33469042	C	T	NRP1	MISSENSE	ENSG00000099250:[ENST00000413802(c.280G>A,Gac>Aac):ENSP00000390447(p.Asp94Asn)]	NA	NA	NA	NA	NA	NA	NA	T	PrD	NA	MODERATE	D	1	294	293	0	287	216	68	23.69
Chewer	IOB_03	chr10	86273981	C	A	CCSER2	SILENT	ENSG00000107771:[ENST00000372088(c.3102C>A,gcC>gcA):ENSP00000361160(p.Ala1034(=))]	NA	NA	NA	NA	NA	NA	NA	NA	NA	NA	MODIFIER	NA	NA	80	80	0	94	89	5	5.32
Chewer	IOB_03	chr10	105967550	C	T	WDR96	SILENT	ENSG00000197748:[ENST00000278064(c.558G>A,ccG>ccA):ENSP00000278064(p.Pro186(=))]	NA	NA	NA	NA	NA	NA	NA	NA	NA	NA	LOW	NA	NA	220	220	0	211	202	5	2.37
Chewer	IOB_03	chr11	824399	C	T	AP006621.8	NCT	ENSG00000255108:[ENST00000532946(n.307-536G>A)]	NA	chr11*824399*824399*COSM1605286*C*T*1*liver*1	chr11*824399*824399*C*A,T*rs767225998*NA*NA*NA	6.04E-05	0.00E+00	NA	NA	D	PoD	NA	MODERATE	N	0.993	158	158	0	210	204	5	2.38
Chewer	IOB_03	chr11	824399	C	T	PNPLA2	MISSENSE	ENSG00000177666:[ENST00000336615(c.1138C>T,Cgc>Tgc):ENSP00000337701(p.Arg380Cys)]	NA	chr11*824399*824399*COSM1605286*C*T*1*liver*1	chr11*824399*824399*C*A,T*rs767225998*NA*NA*NA	6.04E-05	0.00E+00	NA	NA	D	PoD	NA	MODERATE	N	0.993	158	158	0	210	204	5	2.38
Chewer	IOB_03	chr11	824399	C	T	RPLP2	INTRONIC	6181:[XM_005253056.1(c.272-3096C>T)]	NA	chr11*824399*824399*COSM1605286*C*T*1*liver*1	chr11*824399*824399*C*A,T*rs767225998*NA*NA*NA	6.04E-05	0.00E+00	NA	NA	D	PoD	NA	MODERATE	N	0.993	158	158	0	210	204	5	2.38
Chewer	IOB_03	chr11	2016581	C	A	H19	EXONIC-NC	ENSG00000130600:[ENST00000439725(n.1754G>T)]	NA	NA	NA	NA	NA	NA	NA	NA	NA	NA	MODIFIER	NA	NA	114	114	0	111	106	5	4.5
Chewer	IOB_03	chr11	2016581	C	A	HOTS	MISSENSE	103344718:[NM_001293171.2(c.155C>A,cCa>cAa):NP_001280100.1(p.Pro52Gln)]	NA	NA	NA	NA	NA	NA	NA	NA	NA	NA	MODIFIER	NA	NA	114	114	0	111	106	5	4.5
Chewer	IOB_03	chr11	43391620	T	C	TTC17	INTRONIC	ENSG00000052841:[ENST00000039989(c.160-9163T>C)]	NA	NA	NA	NA	NA	NA	NA	NA	NA	NA	MODIFIER	NA	NA	81	81	0	71	56	15	21.13
Chewer	IOB_03	chr11	65156968	C	T	FRMD8	SILENT	ENSG00000126391:[ENST00000526201(c.198C>T,gtC>gtT):ENSP00000435991(p.Val66(=))]	NA	NA	NA	NA	NA	NA	NA	NA	NA	NA	MODIFIER	NA	NA	128	126	2	193	183	10	5.18
Chewer	IOB_03	chr11	66105637	C	A	BRMS1	NCT	ENSG00000174744:[ENST00000530238(n.1296+77G>T)]	NA	NA	chr11*66105637*66105637*C*G*rs937139659*NA*NA*NA	NA	NA	NA	NA	NA	NA	NA	MODIFIER	NA	NA	115	114	1	121	116	5	4.13
Chewer	IOB_03	chr11	66105637	C	A	RP11-867G23.12	NCT	ENSG00000254756:[ENST00000526655(n.424-1617C>A)]	NA	NA	chr11*66105637*66105637*C*G*rs937139659*NA*NA*NA	NA	NA	NA	NA	NA	NA	NA	MODIFIER	NA	NA	115	114	1	121	116	5	4.13
Chewer	IOB_03	chr11	72409146	G	A	ARAP1	SILENT	ENSG00000186635:[ENST00000429686(c.1629C>T,ttC>ttT):ENSP00000403127(p.Phe543(=))]	NA	NA	NA	5.75E-04	1.03E-04	NA	NA	NA	NA	NA	LOW	NA	NA	81	79	0	121	116	5	4.13
Chewer	IOB_03	chr11	73717905	G	C	UCP3	INTRONIC	ENSG00000175564:[ENST00000314032(c.126+57C>G)]	NA	NA	chr11*73717905*73717905*G*T*rs536504852*0*0.9998*0.0001997	NA	NA	NA	NA	NA	NA	NA	MODIFIER	NA	NA	156	156	0	174	169	5	2.87
Chewer	IOB_03	chr11	108562629	C	T	DDX10	SILENT	ENSG00000178105:[ENST00000322536(c.1002C>T,tgC>tgT):ENSP00000314348(p.Cys334(=))]	NA	NA	NA	8.24E-06	0.00E+00	NA	NA	NA	NA	NA	LOW	NA	1	164	163	0	121	114	5	4.13
Chewer	IOB_03	chr11	111680449	C	T	ALG9	MISSENSE	ENSG00000086848:[ENST00000532425(c.384G>A,Gaa>Aaa):ENSP00000432442(p.Glu129Lys)]	NA	NA	NA	NA	NA	NA	NA	NA	NA	NA	MODIFIER	NA	NA	128	128	0	151	132	18	11.92
Chewer	IOB_03	chr11	111680451	C	T	ALG9	MISSENSE	ENSG00000086848:[ENST00000398006(c.1115G>A,aGa>aAa):ENSP00000381090(p.Arg372Lys)]	NA	NA	NA	NA	NA	NA	NA	T	BN	NA	MODERATE	NA	0	130	130	0	150	131	17	11.33
Chewer	IOB_03	chr12	14836161	G	T	GUCY2C	SILENT	ENSG00000070019:[ENST00000261170(c.426C>A,atC>atA):ENSP00000261170(p.Ile142(=))]	NA	NA	NA	NA	NA	NA	NA	NA	NA	NA	MODIFIER	NA	NA	183	182	0	202	174	27	13.37
Chewer	IOB_03	chr12	14836161	G	T	RP11-174G6.1	NCT	ENSG00000214772:[ENST00000501178(n.247+10913G>T)]	NA	NA	NA	NA	NA	NA	NA	NA	NA	NA	MODIFIER	NA	NA	183	182	0	202	174	27	13.37
Chewer	IOB_03	chr12	48886734	G	A	C12orf54	MISSENSE	ENSG00000177627:[ENST00000549398(c.30G>A,atG>atA):ENSP00000450103(p.Met10Ile)]	NA	NA	chr12*48886734*48886734*G*C*rs755348383*NA*NA*NA	NA	NA	NA	NA	NA	NA	NA	MODIFIER	NA	NA	76	75	0	98	79	19	19.39
Chewer	IOB_03	chr12	48886734	G	A	RP11-722P11.4	NCT	ENSG00000258121:[ENST00000551847(n.141-6270C>T)]	NA	NA	chr12*48886734*48886734*G*C*rs755348383*NA*NA*NA	NA	NA	NA	NA	NA	NA	NA	MODIFIER	NA	NA	76	75	0	98	79	19	19.39
Chewer	IOB_03	chr12	50187566	C	T	NCKAP5L	MISSENSE	ENSG00000167566:[ENST00000335999(c.3209G>A,cGg>cAg):ENSP00000337998(p.Arg1070Gln)]	NA	NA	5.99E-04	3.95E-04	1.50E-03	5.99E-04	0.00E+00	T	BN	NA	MODERATE	NA	NA	96	96	0	110	100	9	8.18
Chewer	IOB_03	chr12	52307453	C	T	ACVRL1	MISSENSE	ENSG00000139567:[ENST00000388922(c.424C>T,Cat>Tat):ENSP00000373574(p.His142Tyr)]	NA	NA	NA	NA	NA	NA	NA	T	BN	NA	MODERATE	N	0.988	112	112	0	153	145	7	4.58
Chewer	IOB_03	chr12	53189528	C	T	KRT3	MISSENSE	ENSG00000186442:[ENST00000309505(c.299G>A,gGc>gAc):ENSP00000312206(p.Gly100Asp)]	NA	NA	chr12*53189509*53189533*ACCAAAGCCACCTCCATAGCCGCTC*A*rs750279802*NA*NA*NA	NA	NA	NA	NA	NA	NA	NA	MODERATE	NA	0.005	157	157	0	153	131	20	13.07
Chewer	IOB_03	chr12	53207700	C	A	KRT4	MISSENSE	ENSG00000170477:[ENST00000293774(c.365G>T,gGc>gTc):ENSP00000293774(p.Gly122Val)]	NA	NA	NA	NA	NA	NA	NA	D	PoD	NA	MODERATE	NA	NA	207	206	0	224	216	6	2.68
Chewer	IOB_03	chr12	54423439	G	T	HOXC4	INTRONIC	3221:[XM_005268829.1(c.-123-23505G>T)]	NA	NA	NA	NA	NA	NA	NA	NA	NA	NA	MODIFIER	NA	NA	149	148	1	197	170	25	12.69
Chewer	IOB_03	chr12	54423439	G	T	HOXC5	NCT	3222:[NR_003084.2(n.564-4623G>T)]	NA	NA	NA	NA	NA	NA	NA	NA	NA	NA	MODIFIER	NA	NA	149	148	1	197	170	25	12.69
Chewer	IOB_03	chr12	54423439	G	T	HOXC6	MISSENSE	ENSG00000197757:-SS-PRX[ENST00000394331(c.155G>T,gGg>gTg):ENSP00000377864(p.Gly52Val)]	NA	NA	NA	NA	NA	NA	NA	NA	NA	NA	MODIFIER	NA	NA	149	148	1	197	170	25	12.69
Chewer	IOB_03	chr12	54423439	G	T	RP11-834C11.12	INTRONIC	ENSG00000273049:[ENST00000513209(c.167-4623G>T)]	NA	NA	NA	NA	NA	NA	NA	NA	NA	NA	MODIFIER	NA	NA	149	148	1	197	170	25	12.69
Chewer	IOB_03	chr12	54423439	G	T	RP11-834C11.14	NCT	ENSG00000273046:[ENST00000512206(n.306-4623G>T)]	NA	NA	NA	NA	NA	NA	NA	NA	NA	NA	MODIFIER	NA	NA	149	148	1	197	170	25	12.69
Chewer	IOB_03	chr12	57605205	C	A	LRP1	INTRONIC	ENSG00000123384:[ENST00000243077(c.13092-65C>A)]	NA	NA	NA	NA	NA	NA	NA	NA	NA	NA	MODIFIER	NA	NA	176	176	0	227	221	5	2.2
Chewer	IOB_03	chr12	81226241	C	A	LIN7A	NCT	8825:[NR_136887.1(n.634+13268G>T)]	NA	NA	NA	NA	NA	NA	NA	NA	NA	NA	MODIFIER	NA	NA	152	152	0	160	142	17	10.62
Chewer	IOB_03	chr12	106839930	C	T	POLR3B	INTRONIC	ENSG00000013503:[ENST00000228347(c.2083+1562C>T)]	NA	NA	NA	NA	NA	NA	NA	NA	NA	NA	MODIFIER	NA	NA	148	148	0	148	143	5	3.38
Chewer	IOB_03	chr13	20657069	C	T	ZMYM2	SILENT	ENSG00000121741:[ENST00000382869(c.3717C>T,tcC>tcT):ENSP00000372322(p.Ser1239(=))]	NA	NA	NA	NA	NA	NA	NA	NA	NA	NA	LOW	NA	NA	230	229	0	145	114	31	21.38
Chewer	IOB_03	chr13	32676100	G	T	FRY	NONSENSE-SS-PRX	ENSG00000073910:[ENST00000380250(c.271G>T,Gaa>Taa):ENSP00000369600(p.Glu91Ter)]	NA	NA	NA	NA	NA	NA	NA	NA	NA	NA	HIGH	NA	NA	107	107	0	91	72	18	19.78
Chewer	IOB_03	chr13	113768093	G	A	F7	SILENT	ENSG00000057593:[ENST00000444337(c.216G>A,gtG>gtA):ENSP00000387669(p.Val72(=))]	NA	chr13*113768093*113768093*COSM3700384*G*A*1*liver*1	NA	NA	NA	NA	NA	NA	NA	NA	MODIFIER	NA	NA	143	143	0	187	165	22	11.76
Chewer	IOB_03	chr13	113976637	C	A	LAMP1	SILENT	ENSG00000185896:[ENST00000397181(c.996C>A,ccC>ccA):ENSP00000415354(p.Pro332(=))]	NA	NA	NA	NA	NA	NA	NA	NA	NA	NA	MODIFIER	NA	NA	138	138	0	160	153	6	3.75
Chewer	IOB_03	chr14	21161863	A	T	AL163636.6	INTRONIC	ENSG00000259171:[ENST00000553909(c.86+54A>T)]	NA	NA	NA	NA	NA	NA	NA	NA	NA	NA	MODIFIER	NA	NA	113	112	0	120	98	22	18.33
Chewer	IOB_03	chr14	21161863	A	T	ANG	MISSENSE	ENSG00000214274:[ENST00000336811(c.140A>T,gAc>gTc):ENSP00000336762(p.Asp47Val)]	NA	NA	NA	NA	NA	NA	NA	NA	NA	NA	MODIFIER	NA	NA	113	112	0	120	98	22	18.33
Chewer	IOB_03	chr14	21161863	A	T	RNASE4	INTRONIC	ENSG00000258818:[ENST00000397995(c.-17-5651A>T)]	NA	NA	NA	NA	NA	NA	NA	NA	NA	NA	MODIFIER	NA	NA	113	112	0	120	98	22	18.33
Chewer	IOB_03	chr14	21161863	A	T	RP11-903H12.3	EXONIC-NC	ENSG00000258451:[ENST00000554286(n.691T>A)]	NA	NA	NA	NA	NA	NA	NA	NA	NA	NA	MODIFIER	NA	NA	113	112	0	120	98	22	18.33
Chewer	IOB_03	chr14	22580774	G	A	NA	NCT	101929755:[XR_245743.1(n.35-6656C>T)]	NA	NA	chr14*22580774*22580774*G*T*rs186457642*0*0.9998*0.0001997	NA	NA	NA	NA	NA	NA	NA	LOW	NA	NA	155	154	0	194	153	40	20.62
Chewer	IOB_03	chr14	22580774	G	A	TRAV25	SILENT	ENSG00000211806:[ENST00000390454(c.117G>A,acG>acA):ENSP00000452100(p.Thr39(=))]	NA	NA	chr14*22580774*22580774*G*T*rs186457642*0*0.9998*0.0001997	NA	NA	NA	NA	NA	NA	NA	LOW	NA	NA	155	154	0	194	153	40	20.62
Chewer	IOB_03	chr14	22670450	C	A	NA	NCT	101929755:[XR_245743.1(n.34+61272G>T)]	NA	NA	NA	NA	NA	NA	NA	NA	NA	NA	MODIFIER	NA	NA	99	99	0	115	109	5	4.35
Chewer	IOB_03	chr14	24600593	G	A	FITM1	5UTR	ENSG00000139914:[ENST00000267426(c.-180G>A)]	NA	NA	NA	NA	NA	NA	NA	NA	NA	NA	MODIFIER	NA	NA	265	265	0	340	331	7	2.06
Chewer	IOB_03	chr14	24600593	G	A	RP11-468E2.6	MISSENSE	ENSG00000259371:[ENST00000558325(c.383G>A,Gca>Aca):ENSP00000453089(p.Ala129Thr)]	NA	NA	NA	NA	NA	NA	NA	NA	NA	NA	MODIFIER	NA	NA	265	265	0	340	331	7	2.06
Chewer	IOB_03	chr14	50099995	G	A	DNAAF2	INTRONIC-SS-DNR-PRX	ENSG00000165506:[ENST00000298292(c.1863+10C>T)]	NA	NA	chr14*50099995*50099995*G*T*rs1000254573*NA*NA*NA	NA	NA	NA	NA	NA	NA	NA	MODIFIER	NA	NA	198	198	0	187	181	5	2.67

Patel *et al.* , 2021. Whole-exome sequencing analysis of oral squamous cell carcinoma delineated by tobacco usage habits
Supplementary Table 4. List of somatic single nucleotide variants identified using whole exome sequencing of OSCC patients

Cohort	Sample	Chromosome	Genomic position	Reference allele	Alternate allele	Genesymbol	Variant type	Variant description	OncoMD	COSMIC	dbSNP	ExAC	ExAC_SAS	1000G	1000G_SAS	SIFT	Polyphen2	CONDEL	VEP	LRT	PhastCon7Way	Total reads in Normal	Reference read support in Normal	Alternative read support in Normal	Total reads in Tumor	Reference read support in Tumor	Alternate read support in Tumor	Alternate allele frequency in Tumor
Chewer	IOB_03	chr14	68191116	T	G	RDH12	INTRONIC	ENSG00000139988:[ENST00000267502(c.69-74T>G)]	NA	NA	chr14*68191116*68191116*T*A,G*rs776319260*NA*NA*NA	NA	NA	NA	NA	NA	NA	NA	MODIFIER	NA	NA	72	69	1	88	82	5	5.68
Chewer	IOB_03	chr14	75514673	G	A	MLH3	SILENT	ENSG00000119684:[ENST00000238662(c.1686C>T,tgC>tgT):ENSP00000238662(p.Cys562(=))]	NA	NA	NA	NA	NA	NA	NA	NA	NA	NA	LOW	NA	0	190	187	0	178	170	6	3.37
Chewer	IOB_03	chr14	93649649	C	A	MOAP1	SILENT	ENSG00000165943:[ENST00000298894(c.939G>T,ctG>ctT):ENSP00000298894(p.Leu313(=))]	NA	NA	NA	NA	NA	NA	NA	NA	NA	NA	LOW	NA	NA	89	89	0	85	80	5	5.88
Chewer	IOB_03	chr14	105223660	C	T	SIVA1	INTRONIC	ENSG00000184990:[ENST00000329967(c.470+563C>T)]	NA	NA	NA	NA	NA	NA	NA	NA	NA	NA	MODIFIER	NA	NA	168	167	1	186	168	16	8.6
Chewer	IOB_03	chr14	106552411	G	A	IGHV3-9	SILENT	ENSG00000211940:[ENST00000390600(c.228C>T,agC>agT):ENSP00000375009(p.Ser76(=))]	NA	NA	NA	2.22E-04	1.63E-04	NA	NA	NA	NA	NA	LOW	NA	NA	292	291	1	417	408	8	1.92
Chewer	IOB_03	chr15	22440664	G	A	RP11-2F9.3	EXONIC-NC	ENSG00000223877:[ENST00000460876(n.185C>T)]	NA	NA	9.98E-04	NA	NA	9.98E-04	5.10E-03	NA	NA	NA	MODIFIER	NA	NA	347	346	0	423	398	22	5.2
Chewer	IOB_03	chr15	42137866	G	A	JMJD7-PLA2G4B	MISSENSE	ENSG00000168970:[ENST00000342159(c.2081G>A,gGc>gAc):ENSP00000342785(p.Gly694Asp)]	NA	NA	NA	NA	NA	NA	NA	NA	NA	NA	MODIFIER	NA	NA	88	86	0	121	115	6	4.96
Chewer	IOB_03	chr15	42137866	G	A	PLA2G4B	MISSENSE	ENSG00000243708:[ENST00000452633(c.1388G>A,gGc>gAc):ENSP00000396045(p.Gly463Asp)]	NA	NA	NA	NA	NA	NA	NA	NA	NA	NA	MODIFIER	NA	NA	88	86	0	121	115	6	4.96
Chewer	IOB_03	chr15	44876054	C	A	SPG11	MISSENSE	ENSG00000104133:[ENST00000559822(c.367G>T,ttG>ttT):ENSP00000452744(p.Leu123Phe)]	NA	NA	NA	NA	NA	NA	NA	NA	NA	NA	HIGH	NA	NA	72	72	0	99	86	12	12.12
Chewer	IOB_03	chr15	57810626	C	T	CGNL1	SILENT	ENSG00000128849:[ENST00000281282(c.2646C>T,caC>caT):ENSP00000281282(p.His882(=))]	NA	NA	9.98E-04	6.51E-04	7.19E-05	9.98E-04	0.00E+00	NA	NA	NA	LOW	NA	0	88	87	1	112	86	26	23.21
Chewer	IOB_03	chr15	78882791	G	A	CHRNA5	MISSENSE	ENSG00000169684:[ENST00000559576(c.88G>A,cGc>cAc):ENSP00000452641(p.Arg30His)]	NA	NA	NA	4.12E-05	0.00E+00	NA	NA	NA	NA	NA	MODIFIER	NA	NA	348	346	2	284	275	7	2.46
Chewer	IOB_03	chr15	78882791	G	A	RP11-650L12.2	EXONIC-NC	ENSG00000261762:[ENST00000567141(n.828C>T)]	NA	NA	NA	4.12E-05	0.00E+00	NA	NA	NA	NA	NA	MODIFIER	NA	NA	348	346	2	284	275	7	2.46
Chewer	IOB_03	chr15	85633927	A	C	PDE8A	NCT	ENSG00000073417:[ENST00000560789(n.2322+7052A>C)]	NA	NA	NA	NA	NA	NA	NA	NA	NA	NA	MODIFIER	NA	NA	39	36	1	28	18	7	25
Chewer	IOB_03	chr16	3021765	C	T	PAQR4	MISSENSE	ENSG00000162073:[ENST00000318782(c.638C>T,gCg>gTg):ENSP00000321804(p.Ala213Val)]	NA	NA	NA	NA	NA	NA	NA	D	PrD	NA	MODERATE	D	1	200	198	1	253	244	6	2.37
Chewer	IOB_03	chr16	3021765	C	T	PKMYT1	EXONIC-NC	ENSG00000127564:[ENST00000574333(n.405G>A)]	NA	NA	NA	NA	NA	NA	NA	D	PrD	NA	MODERATE	D	1	200	198	1	253	244	6	2.37
Chewer	IOB_03	chr16	20994224	T	A	DNAH3	MISSENSE	ENSG00000158486:[ENST00000261383(c.7678A>T,Atc>Ttc):ENSP00000261383(p.Ile2560Phe)]	NA	NA	NA	NA	NA	NA	NA	NA	NA	NA	MODIFIER	NA	NA	28	28	0	38	32	6	15.79
Chewer	IOB_03	chr16	30529022	C	A	ITGAL	MISSENSE	ENSG00000005844:[ENST00000433423(c.731C>A,gCt>gAt):ENSP00000409377(p.Ala244Asp)]	NA	NA	chr16*30529022*30529022*C*T*rs764335051*NA*NA*NA	NA	NA	NA	NA	T	BN	NA	MODERATE	N	0.004	76	76	0	100	93	5	5
Chewer	IOB_03	chr16	66643324	C	A	CMTM3	NCT	123920:[NR_037613.1(n.830-14C>A)]	NA	NA	NA	NA	NA	NA	NA	NA	NA	NA	MODIFIER	NA	NA	113	112	0	171	164	6	3.51
Chewer	IOB_03	chr16	67196991	C	T	FBXL8	SILENT	ENSG00000135722:[ENST00000258200(c.393C>T,tgC>tgT):ENSP00000258200(p.Cys131(=))]	NA	NA	NA	NA	NA	NA	NA	NA	NA	NA	LOW	NA	0.059	134	134	0	220	213	5	2.27
Chewer	IOB_03	chr16	71026098	C	T	HYDIN	SILENT	ENSG00000157423:[ENST00000448089(c.3516G>A,ccG>ccA):ENSP00000398544(p.Pro1172(=))]	NA	NA	2.00E-04	1.31E-04	0.00E+00	2.00E-04	0.00E+00	NA	NA	NA	LOW	NA	NA	83	72	11	82	49	31	37.8
Chewer	IOB_03	chr16	83998804	G	T	OSGIN1	MISSENSE	ENSG00000140961:[ENST00000343939(c.875G>T,aGc>aTc):ENSP00000343376(p.Ser292Ile)]	NA	NA	NA	NA	NA	NA	NA	T	BN	NA	MODERATE	N	0	259	259	0	342	331	10	2.92
Chewer	IOB_03	chr17	7579528	C	T	TP53	NONSENSE	ENSG00000141510:[ENST00000269305(c.159G>A,tgG>tgA):ENSP00000269305(p.Trp53Ter)]	NA	chr17*7579498*7579538*COSM43644*BigSeq*A*1*pancreas*1|chr17*7579510*7579535*COSM391510*BigSeq*A*1*lung*1|chr17*7579510*7579535*COSM391511*BigSeq*A*1*lung*1|chr17*7579510*7579535*COSM391509*BigSeq*A*1*lung*1|chr17*7579520*7579533*COSM44839*TCAGTGAACCATTG*T*1*endometrium*1|chr17*7579528*7579528*COSM1324762*C*T*1*ovary*1|chr17*7579528*7579528*COSM45808*C*A*1*haematopoietic_and_lymphoid_tissue*1|chr17*7579528*7579528*COSM44488*C*T*7*upper_aerodigestive_tract,ovary*4,3|chr17*7579528*7579528*COSM45445*C*G*1*skin*1|chr17*7579528*7579528*COSM1162687*C*CCATT*1*pancreas*1|chr17*7579528*7579528*COSM1162686*C*CCATT*1*pancreas*1|chr17*7579528*7579528*COSM1162685*C*CCATT*1*pancreas*1|chr17*7579528*7	NA	NA	NA	NA	NA	NA	NA	NA	HIGH	NA	0	108	108	0	70	61	9	12.86
Chewer	IOB_03	chr17	42082454	C	A	NAGS	SILENT	ENSG00000161653:[ENST00000293404(c.423C>A,atC>atA):ENSP00000293404(p.Ile141(=))]	NA	NA	NA	NA	NA	NA	NA	NA	NA	NA	LOW	NA	NA	192	192	0	268	261	6	2.24
Chewer	IOB_03	chr17	51902102	G	A	KIF2B	MISSENSE	ENSG00000141200:[ENST00000268919(c.1708G>A,Gga>Aga):ENSP00000268919(p.Gly570Arg)]	NA	chr17*51902102*51902102*COSM312344*G*A*1*lung*1	NA	NA	NA	NA	NA	T	BN	NA	MODERATE	N	0	118	117	0	154	134	20	12.99
Chewer	IOB_03	chr17	61607871	C	T	KCNH6	MISSENSE	ENSG00000173826:[ENST00000314672(c.643C>T,Cgg>Tgg):ENSP00000318212(p.Arg215Trp)]	NA	NA	NA	1.65E-05	0.00E+00	NA	NA	NA	NA	NA	MODIFIER	NA	NA	198	195	1	285	256	29	10.18
Chewer	IOB_03	chr17	73089835	G	A	SLC16A5	MISSENSE	ENSG00000170190:[ENST00000329783(c.104G>A,gGc>gAc):ENSP00000330141(p.Gly35Asp)]	NA	NA	NA	NA	NA	NA	NA	NA	NA	NA	MODIFIER	NA	NA	334	331	3	472	464	8	1.69
Chewer	IOB_03	chr17	73750727	G	A	GALK1	NCT	ENSG00000108479:[ENST00000589643(n.255-2909C>T)]	NA	NA	2.00E-04	3.31E-05	0.00E+00	2.00E-04	0.00E+00	NA	NA	NA	LOW	NA	NA	228	227	0	327	299	23	7.03
Chewer	IOB_03	chr17	73750727	G	A	ITGB4	SILENT	ENSG00000132470:[ENST00000583327(c.372G>A,acG>acA):ENSP00000464473(p.Thr124(=))]	NA	NA	2.00E-04	3.31E-05	0.00E+00	2.00E-04	0.00E+00	NA	NA	NA	LOW	NA	NA	228	227	0	327	299	23	7.03
Chewer	IOB_03	chr19	2290717	C	T	LINGO3	SILENT	ENSG00000220008:[ENST00000404279(c.1059G>A,ccG>ccA):ENSP00000384979(p.Pro353(=))]	NA	NA	NA	NA	NA	NA	NA	NA	NA	NA	LOW	NA	NA	338	337	0	466	455	5	1.07
Chewer	IOB_03	chr19	9047570	G	A	MUC16	MISSENSE	ENSG00000181143:[ENST00000397910(c.34061C>T,cCt>cTt):ENSP00000381008(p.Pro11354Leu)]	NA	NA	chr19*9047569*9047570*AG*A*rs754086754*NA*NA*NA	NA	NA	NA	NA	NA	PoD	NA	MODERATE	NA	NA	145	143	0	135	126	9	6.67
Chewer	IOB_03	chr19	33870476	C	A	CEBPG	MISSENSE	ENSG00000153879:[ENST00000284000(c.331C>A,Ctg>Atg):ENSP00000284000(p.Leu111Met)]	NA	NA	NA	NA	NA	NA	NA	D	PrD	NA	MODERATE	D	1	227	227	0	368	358	8	2.17
Chewer	IOB_03	chr19	36303156	C	T	PRODH2	SILENT	ENSG00000250799:[ENST00000301175(c.618G>A,ggG>ggA):ENSP00000301175(p.Gly206(=))]	NA	NA	NA	NA	NA	NA	NA	NA	NA	NA	LOW	NA	NA	166	163	0	209	198	10	4.78
Chewer	IOB_03	chr19	41119778	C	T	LTBP4	SILENT	ENSG00000090006:[ENST00000243562(c.9C>T,ggC>ggT):ENSP00000243562(p.Gly3(=))]	NA	NA	NA	9.81E-06	0.00E+00	NA	NA	NA	NA	NA	MODIFIER	NA	NA	131	131	0	182	177	5	2.75
Chewer	IOB_03	chr19	42315007	G	A	CEACAM3	INTRONIC	ENSG00000170956:[ENST00000221999(c.*1+72G>A)]	NA	NA	NA	NA	NA	NA	NA	NA	NA	NA	MODIFIER	NA	NA	79	79	0	115	110	5	4.35
Chewer	IOB_03	chr19	48389454	C	T	SULT2A1	MISSENSE	ENSG00000105398:[ENST00000222002(c.61G>A,Gaa>Aaa):ENSP00000222002(p.Glu21Lys)]	NA	NA	2.00E-04	7.41E-05	1.82E-04	2.00E-04	0.00E+00	D	PoD	NA	MODERATE	D	0.089	234	232	0	198	174	24	12.12
Chewer	IOB_03	chr19	56320410	C	G	NLRP11	MISSENSE	ENSG00000179873:[ENST00000590409(c.1269G>C,aaG>aaC):ENSP00000466582(p.Lys423Asn)]	NA	NA	NA	NA	NA	NA	NA	NA	NA	NA	MODIFIER	NA	NA	297	296	0	265	237	26	9.81
Chewer	IOB_03	chr2	60687642	C	A	BCL11A	MISSENSE	ENSG00000119866:[ENST00000358510(c.2303G>T,tGt>tTt):ENSP00000351307(p.Cys768Phe)]	NA	NA	NA	NA	NA	NA	NA	NA	NA	NA	MODIFIER	NA	NA	226	225	0	208	202	5	2.4
Chewer	IOB_03	chr2	61719221	A	G	XPO1	SILENT	ENSG00000082898:[ENST00000401558(c.1836T>C,gaT>gaC):ENSP00000384863(p.Asp612(=))]	NA	NA	NA	NA	NA	NA	NA	NA	NA	NA	LOW	NA	1	72	72	0	77	62	14	18.18
Chewer	IOB_03	chr2	70524576	C	G	FAM136A	MISSENSE	ENSG00000035141:[ENST00000438759(c.472G>C,Gac>Cac):ENSP00000390840(p.Asp158His)]	NA	NA	NA	5.31E-02	4.53E-02	NA	NA	NA	NA	NA	MODERATE	NA	1	108	107	0	121	102	18	14.88
Chewer	IOB_03	chr2	70524606	G	A	FAM136A	MISSENSE	ENSG00000035141:[ENST00000438759(c.442C>T,Cgc>Tgc):ENSP00000390840(p.Arg148Cys)]	NA	NA	NA	1.25E-02	1.03E-02	NA	NA	NA	NA	NA	MODERATE	NA	1	122	122	0	119	105	14	11.76
Chewer	IOB_03	chr2	71304615	C	A	NAGK	INTRONIC	ENSG00000124357:[ENST00000455662(c.904-73C>A)]	NA	NA	1.60E-03	NA	NA	1.60E-03	0.00E+00	NA	NA	NA	MODIFIER	NA	NA	95	95	0	106	97	6	5.66
Chewer	IOB_03	chr2	80136842	C	T	CTNNA2	SILENT	ENSG00000066032:[ENST00000361291(c.1077C>T,gaC>gaT):ENSP00000355398(p.Asp359(=))]	NA	NA	NA	8.37E-06	0.00E+00	NA	NA	NA	NA	NA	LOW	NA	0.447	245	245	0	277	201	75	27.08
Chewer	IOB_03	chr2	172187274	T	A	METTL8	NCT	ENSG00000123600:[ENST00000477130(n.81-6402A>T)]	NA	NA	NA	NA	NA	NA	NA	NA	NA	NA	MODIFIER	NA	NA	77	77	0	68	63	5	7.35
Chewer	IOB_03	chr2	202990555	G	A	AC079354.1	MISSENSE	ENSG00000182329:[ENST00000541917(c.1941G>A,atG>atA):ENSP00000437957(p.Met647Ile)]	NA	NA	NA	NA	NA	NA	NA	NA	NA	NA	MODIFIER	NA	NA	62	60	0	40	30	10	25
Chewer	IOB_03	chr2	202990555	G	A	KIAA2012	INTRONIC	100652824:[NM_001277372.1(c.1936+550G>A)]	NA	NA	NA	NA	NA	NA	NA	NA	NA	NA	MODIFIER	NA	NA	62	60	0	40	30	10	25
Chewer	IOB_03	chr2	234731479	A	G	MROH2A	INTRONIC	ENSG00000185038:[ENST00000389758(c.3867-48A>G)]	NA	NA	NA	NA	NA	NA	NA	NA	NA	NA	MODIFIER	NA	NA	105	104	0	158	134	22	13.92
Chewer	IOB_03	chr20	3171440	G	A	DDRGK1	SILENT	ENSG00000198171:[ENST00000354488(c.804C>T,ttC>ttT):ENSP00000346483(p.Phe268(=))]	NA	NA	NA	NA	NA	NA	NA	NA	NA	NA	LOW	NA	1	98	97	0	114	103	11	9.65
Chewer	IOB_03	chr20	3758912	G	A	SPEF1	MISSENSE	ENSG00000101222:[ENST00000379756(c.658C>T,Cgg>Tgg):ENSP00000369080(p.Arg220Trp)]	NA	NA	NA	NA	NA	NA	NA	D	PrD	NA	MODERATE	U	1	199	197	0	264	257	7	2.65
Chewer	IOB_03	chr20	34828408	C	A	AAR2	SILENT	ENSG00000131043:[ENST00000320849(c.618C>A,atC>atA):ENSP00000313674(p.Ile206(=))]	NA	NA	NA	NA	NA	NA	NA	NA	NA	NA	LOW	NA	1	145	145	0	158	152	5	3.16
Chewer	IOB_03	chr20	42683033	C	G	TOX2	MISSENSE	ENSG00000124191:[ENST00000372999(c.620C>G,cCt>cGt):ENSP00000362090(p.Pro207Arg)]	NA	NA	NA	NA	NA	NA	NA	NA	PoD	NA	MODERATE	NA	NA	63	63	0	57	52	5	8.77
Chewer	IOB_03	chr20	57576564	C	T	CTSZ	MISSENSE	ENSG00000101160:[ENST00000217131(c.443G>A,gGc>gAc):ENSP00000217131(p.Gly148Asp)]	NA	NA	NA	NA	NA	NA	NA	D	PrD	NA	MODERATE	D	1	195	195	0	294	287	5	1.7
Chewer	IOB_03	chr20	57829236	C	A	ZNF831	MISSENSE	ENSG00000124203:[ENST00000371030(c.4472C>A,gCt>gAt):ENSP00000360069(p.Ala1491Asp)]	NA	NA	NA	NA	NA	NA	NA	D	BN	NA	MODERATE	N	0	195	194	1	147	131	16	10.88
Chewer	IOB_03	chr20	61511322	C	T	DIDO1	MISSENSE	ENSG00000101191:[ENST00000266070(c.5986G>A,Gca>Aca):ENSP00000266070(p.Ala1996Thr)]	NA	NA	chr20*61511322*61511322*C*G,T*rs866633267*NA*NA*NA	NA	NA	NA	NA	T	BN	NA	MODERATE	N	0	124	124	0	188	183	5	2.66
Chewer	IOB_03	chr20	62187789	G	A	C20orf195	MISSENSE	ENSG00000125531:[ENST00000370097(c.773G>A,tGt>tAt):ENSP00000359115(p.Cys258Tyr)]	NA	NA	NA	NA	NA	NA	NA	NA	NA	NA	MODERATE	NA	0.166	267	267	0	395	365	28	7.09
Chewer	IOB_03	chr20	62421195	G	A	ZBTB46	MISSENSE	ENSG00000130584:[ENST00000245663(c.916C>T,Ccg>Tcg):ENSP00000245663(p.Pro306Ser)]	NA	NA	NA	NA	NA	NA	NA	T	BN	NA	MODERATE	D	1	140	139	0	167	158	7	4.19

Patel *et al.*, 2021. Whole-exome sequencing analysis of oral squamous cell carcinoma delineated by tobacco usage habits
Supplementary Table 4. List of somatic single nucleotide variants identified using whole exome sequencing of OSCC patients

Cohort	Sample	Chromosome	Genomic position	Reference allele	Alternate allele	Genesymbol	Variant type	Variant description	OncoMD	COSMIC	dbSNP	ExAC	ExAC_SAS	1000G	1000G_SAS	SIFT	Polyphen2	CONDEL	VEP	LRT	PhastCon7Way	Total reads in Normal	Reference read support in Normal	Alternative read support in Normal	Total reads in Tumor	Reference read support in Tumor	Alternate read support in Tumor	Alternate allele frequency in Tumor
Chewer	IOB_03	chr20	62904845	G	A	PCMTD2	SILENT	ENSG00000203880:[ENST00000369758(c.897G>A,ccG>ccA):ENSP00000358773(p.Pro299(=))]	NA	NA	8.59E-03	3.00E-03	4.16E-04	8.59E-03	0.00E+00	NA	NA	NA	MODIFIER	NA	NA	58	57	1	55	49	6	10.91
Chewer	IOB_03	chr21	19167559	C	T	AL109761.5	NCT	ENSG00000244676:[ENST00000428689(n.71+1684C>T)]	NA	NA	NA	NA	NA	NA	NA	NA	NA	NA	LOW	NA	NA	128	126	0	143	135	8	5.59
Chewer	IOB_03	chr21	19167559	C	T	C21orf91	SILENT	ENSG00000154642:[ENST00000284881(c.693G>A,gaG>gaA):ENSP00000284881(p.Glu231(=))]	NA	NA	NA	NA	NA	NA	NA	NA	NA	NA	LOW	NA	NA	128	126	0	143	135	8	5.59
Chewer	IOB_03	chr21	27270017	C	A	APP	INTRONIC	351:[NM_001204303.1(c.1685-32G>T)]	NA	NA	NA	NA	NA	NA	NA	NA	NA	NA	MODIFIER	NA	NA	146	145	0	134	129	5	3.73
Chewer	IOB_03	chr21	33725126	C	T	URB1	MISSENSE	ENSG00000142207:[ENST00000382751(c.2506G>A,Gca>Aca):ENSP00000372199(p.Ala836Thr)]	NA	NA	NA	9.30E-05	0.00E+00	NA	NA	T	BN	NA	MODERATE	N	1	177	176	1	232	223	7	3.02
Chewer	IOB_03	chr22	31301945	C	A	OSBP2	MISSENSE	ENSG00000184792:[ENST00000452656(c.1205C>A,cCg>cAg):ENSP00000409838(p.Pro402Gln)]	NA	NA	chr22*31301945*31301945*C*T*rs776910799*NA*NA*NA	NA	NA	NA	NA	T	BN	NA	MODERATE	N	0	145	145	0	195	190	5	2.56
Chewer	IOB_03	chr22	32011108	C	A	SFI1	MISSENSE	ENSG00000198089:[ENST00000400289(c.2897C>A,gCa>gAa):ENSP00000383146(p.Ala966Glu)]	NA	NA	chr22*32011108*32011108*C*T*rs759721678*NA*NA*NA	NA	NA	NA	NA	NA	NA	NA	MODIFIER	NA	NA	167	165	0	210	203	6	2.86
Chewer	IOB_03	chr22	40574202	C	A	TNRC6B	NCT	ENSG00000100354:[ENST00000489500(n.216+58C>A)]	NA	NA	NA	NA	NA	NA	NA	NA	NA	NA	MODIFIER	NA	NA	205	205	0	170	164	5	2.94
Chewer	IOB_03	chr3	38622617	C	G	SCN5A	SILENT	ENSG00000183873:[ENST00000333535(c.3033G>C,ccG>ccC):ENSP00000328968(p.Pro1011(=))]	NA	chr3*38622617*38622617*COSM1422797*C*T*1*large_intestine*1|chr3*38622617*38622617*COSM1422798*C*T*1*large_intestine*1|chr3*38622617*38622617*COSM1422796*C*T*1*large_intestine*1	NA	8.89E-06	0.00E+00	NA	NA	NA	NA	NA	LOW	NA	NA	158	152	4	166	151	7	4.22
Chewer	IOB_03	chr3	52558621	C	T	NT5DC2	SILENT	ENSG00000168268:[ENST00000422318(c.1539G>A,ctG>ctA):ENSP00000406933(p.Leu513(=))]	NA	NA	NA	NA	NA	NA	NA	NA	NA	NA	LOW	NA	NA	160	160	0	198	192	6	3.03
Chewer	IOB_03	chr3	88175302	C	T	CGGBP1	INTRONIC	ENSG00000163320:[ENST00000467332(c.-24+14818G>A)]	NA	NA	1.52E-02	2.54E-03	0.00E+00	1.52E-02	0.00E+00	NA	NA	NA	MODIFIER	NA	NA	87	87	0	93	69	23	24.73
Chewer	IOB_03	chr3	88175302	C	T	RP11-159G9.5	MISSENSE	ENSG00000229729:[ENST00000473136(c.433C>T,Cgc>Tgc):ENSP00000419057(p.Arg145Cys)]	NA	NA	1.52E-02	2.54E-03	0.00E+00	1.52E-02	0.00E+00	NA	NA	NA	MODIFIER	NA	NA	87	87	0	93	69	23	24.73
Chewer	IOB_03	chr3	88175302	C	T	ZNF654	5UTR	55279:[XM_005264766.1(c.-204C>T)]	NA	NA	1.52E-02	2.54E-03	0.00E+00	1.52E-02	0.00E+00	NA	NA	NA	MODIFIER	NA	NA	87	87	0	93	69	23	24.73
Chewer	IOB_03	chr3	121341891	G	C	FBXO40	MISSENSE	ENSG00000163833:[ENST00000338040(c.1615G>C,Gag>Cag):ENSP00000337510(p.Glu539Gln)]	NA	NA	NA	NA	NA	NA	NA	T	BN	NA	MODERATE	N	1	64	64	0	71	63	8	11.27
Chewer	IOB_03	chr3	122133645	C	A	WDR5B	MISSENSE	ENSG00000196981:[ENST00000330689(c.731G>T,tGc>tTc):ENSP00000330381(p.Cys244Phe)]	NA	NA	NA	NA	NA	NA	NA	T	PrD	NA	MODERATE	D	1	296	296	0	344	334	6	1.74
Chewer	IOB_03	chr3	184049170	G	T	EIF2B5	INTRONIC	ENSG00000145191:[ENST00000444495(c.2106+186675G>T)]	NA	NA	NA	NA	NA	NA	NA	NA	NA	NA	MODIFIER	NA	NA	134	132	1	162	156	5	3.09
Chewer	IOB_03	chr3	184049170	G	T	EIF4G1	INTRONIC	1981:[XM_005247193.1(c.4083+18G>T)]	NA	NA	NA	NA	NA	NA	NA	NA	NA	NA	MODIFIER	NA	NA	134	132	1	162	156	5	3.09
Chewer	IOB_03	chr3	184552483	C	T	VPS8	MISSENSE	ENSG00000156931:[ENST00000426319(c.21C>T,Cgc>Tgc):ENSP00000416101(p.Arg8Cys)]	NA	NA	NA	3.86E-05	0.00E+00	NA	NA	D	PrD	NA	MODERATE	NA	NA	260	255	0	260	249	5	1.92
Chewer	IOB_03	chr3	185216603	G	C	TMEM41A	INTRONIC	ENSG00000163900:[ENST00000296254(c.119+28C>G)]	NA	NA	NA	NA	NA	NA	NA	NA	NA	NA	MODIFIER	NA	NA	140	140	0	191	172	18	9.42
Chewer	IOB_03	chr3	195686891	T	C	SDHAP1	EXONIC-NC	ENSG00000185485:[ENST00000427841(n.2392A>G)]	NA	NA	NA	NA	NA	NA	NA	NA	NA	NA	MODIFIER	NA	NA	47	44	3	21	12	9	42.86
Chewer	IOB_03	chr3	195785462	G	T	TFRC	MISSENSE	ENSG00000072274:[ENST00000535031(c.732C>A,agC>agA):ENSP00000437753(p.Ser244Arg)]	NA	NA	NA	NA	NA	NA	NA	D	PrD	NA	MODERATE	N	1	166	166	0	149	144	5	3.36
Chewer	IOB_03	chr4	494301	C	T	PIGG	MISSENSE	ENSG00000174227:[ENST00000296306(c.4C>T,Ccc>Tcc):ENSP00000296306(p.Pro2Ser)]	NA	NA	NA	NA	NA	NA	NA	NA	NA	NA	MODIFIER	NA	NA	148	145	0	141	133	6	4.26
Chewer	IOB_03	chr4	8588990	G	A	GPR78	MISSENSE	ENSG00000155269:[ENST00000382487(c.992G>A,gGc>gAc):ENSP00000371927(p.Gly331Asp)]	NA	NA	NA	NA	NA	NA	NA	NA	NA	NA	MODIFIER	NA	NA	225	224	0	232	223	8	3.45
Chewer	IOB_03	chr5	7885913	G	A	MTRR	MISSENSE	ENSG00000124275:[ENST00000440940(c.1003G>A,Gat>Aat):ENSP00000402510(p.Asp335Asn)]	NA	NA	NA	NA	NA	NA	NA	NA	NA	NA	MODIFIER	NA	NA	167	167	0	211	204	5	2.37
Chewer	IOB_03	chr5	19473798	C	T	CDH18	MISSENSE	ENSG00000145526:[ENST00000274170(c.1910G>A,aGg>aAg):ENSP00000274170(p.Arg637Lys)]	NA	NA	NA	7.56E-05	0.00E+00	NA	NA	NA	NA	NA	MODIFIER	NA	NA	104	103	0	111	78	33	29.73
Chewer	IOB_03	chr5	89938782	C	T	ADGRV1	EXONIC-NC	84059:[NR_003149.1(n.2573C>T)]	NA	NA	NA	8.29E-06	0.00E+00	NA	NA	NA	NA	NA	MODIFIER	NA	NA	151	150	0	148	114	33	22.3
Chewer	IOB_03	chr5	89938782	C	T	GPR98	MISSENSE	ENSG00000164199:[ENST00000504142(c.1242C>T,aCg>aTg):ENSP00000424736(p.Thr415Met)]	NA	NA	NA	8.29E-06	0.00E+00	NA	NA	NA	NA	NA	MODIFIER	NA	NA	151	150	0	148	114	33	22.3
Chewer	IOB_03	chr5	112174248	A	G	APC	MISSENSE	ENSG00000134982:[ENST00000507379(c.2903A>G,tAt>tGt):ENSP00000423224(p.Tyr968Cys)]	NA	NA	NA	NA	NA	NA	NA	NA	NA	NA	MODIFIER	NA	NA	151	151	0	122	98	24	19.67
Chewer	IOB_03	chr5	112174248	A	G	CTC-554D6.1	INTRONIC	ENSG00000258864:[ENST00000520401(c.230+9579A>G)]	NA	NA	NA	NA	NA	NA	NA	NA	NA	NA	MODIFIER	NA	NA	151	151	0	122	98	24	19.67
Chewer	IOB_03	chr5	147510910	G	A	SPINK5	MISSENSE	ENSG00000133710:[ENST00000256084(c.3053G>A,gGc>gAc):ENSP00000256084(p.Gly1018Asp)]	NA	NA	NA	NA	NA	NA	NA	D	BN	NA	MODERATE	N	1	278	276	1	229	222	6	2.62
Chewer	IOB_03	chr5	178552067	G	A	ADAMTS2	SILENT	ENSG00000087116:[ENST00000251582(c.2865C>T,caC>caT):ENSP00000251582(p.His955(=))]	NA	chr5*178552067*178552067*COSM1436474*G*A*1*large_intestine*1	NA	4.16E-05	6.06E-05	NA	NA	NA	NA	NA	LOW	NA	1	135	133	0	165	154	8	4.85
Chewer	IOB_03	chr5	180048772	G	A	FLT4	MISSENSE	ENSG00000037280:[ENST00000261937(c.1790C>T,aCg>aTg):ENSP00000261937(p.Thr597Met)]	NA	NA	NA	8.33E-06	0.00E+00	NA	NA	NA	NA	NA	MODIFIER	NA	NA	303	301	2	445	369	73	16.4
Chewer	IOB_03	chr6	4130970	C	A	C6orf201	EXONIC-NC	404220:[XR_241897.1(n.1241C>A)]	NA	NA	NA	NA	NA	NA	NA	NA	NA	NA	MODIFIER	NA	NA	137	136	0	139	133	5	3.6
Chewer	IOB_03	chr6	4130970	C	A	ECI2	INTRONIC	ENSG00000198721:[ENST00000495548(c.453+31G>T)]	NA	NA	NA	NA	NA	NA	NA	NA	NA	NA	MODIFIER	NA	NA	137	136	0	139	133	5	3.6
Chewer	IOB_03	chr6	30523458	C	A	GNL1	MISSENSE	ENSG00000204590:[ENST00000433809(c.107G>T,aGc>aTc):ENSP00000404728(p.Ser36Ile)]	NA	NA	NA	NA	NA	NA	NA	D	PoD	NA	MODERATE	D	1	126	125	0	183	177	6	3.28
Chewer	IOB_03	chr6	31599996	C	T	PRRC2A	SILENT	ENSG00000204469:[ENST00000376007(c.3546C>T,tgC>tgT):ENSP00000365175(p.Cys1182(=))]	NA	NA	chr6*31599996*31599996*C*G*rs750209152*NA*NA*NA	NA	NA	NA	NA	NA	NA	NA	LOW	NA	0.017	94	94	0	110	104	5	4.55
Chewer	IOB_03	chr6	32939959	C	A	BRD2	INTRONIC	ENSG00000204256:[ENST00000456339(c.35-2280C>A)]	NA	NA	chr6*32939959*32939959*C*G*rs547700086*0*0.9998*0.0001997	NA	NA	NA	NA	NA	NA	NA	MODIFIER	NA	NA	124	123	0	243	235	7	2.88
Chewer	IOB_03	chr6	32939959	C	A	XXbac-BPG181M17.6	EXONIC-NC	ENSG00000263756:[ENST00000580587(n.460G>T)]	NA	NA	chr6*32939959*32939959*C*G*rs547700086*0*0.9998*0.0001997	NA	NA	NA	NA	NA	NA	NA	MODIFIER	NA	NA	124	123	0	243	235	7	2.88
Chewer	IOB_03	chr6	46658801	C	A	TDRD6	MISSENSE	ENSG00000180113:[ENST00000316081(c.2936C>A,aCa>aAa):ENSP00000346065(p.Thr979Lys)]	NA	NA	NA	8.27E-06	6.08E-05	NA	NA	NA	NA	NA	MODIFIER	NA	NA	172	171	1	197	191	6	3.05
Chewer	IOB_03	chr6	53409404	C	A	GCLC	NONSENSE	ENSG00000001084:[ENST00000513939(c.3G>T,Gaa>Taa):ENSP00000424211(p.Glu2Ter)]	NA	NA	NA	NA	NA	NA	NA	NA	NA	NA	HIGH	NA	1	263	263	0	334	326	7	2.1
Chewer	IOB_03	chr6	53706975	C	A	LRRC1	MISSENSE	ENSG00000137269:[ENST00000370882(c.227C>A,cCa>cAa):ENSP00000359919(p.Pro76Gln)]	NA	NA	NA	NA	NA	NA	NA	T	PoD	NA	MODERATE	NA	NA	190	190	0	185	178	6	3.24
Chewer	IOB_03	chr6	117114080	C	G	GPRC6A	MISSENSE	ENSG00000173612:[ENST00000530250(c.1481G>C,aGc>aCc):ENSP00000433465(p.Ser494Thr)]	NA	NA	chr6*117114080*117114080*C*T*rs777273960*NA*NA*NA	NA	NA	NA	NA	D	PrD	NA	MODERATE	D	1	236	235	0	232	188	43	18.53
Chewer	IOB_03	chr6	122766313	G	A	SERINC1	MISSENSE	ENSG00000111897:[ENST00000339697(c.1238C>T,tCt>tTt):ENSP00000342962(p.Ser413Phe)]	NA	NA	NA	NA	NA	NA	NA	NA	NA	NA	MODERATE	NA	1	136	136	0	138	123	15	10.87
Chewer	IOB_03	chr6	153077285	G	A	VIP	MISSENSE	ENSG00000146469:[ENST00000431366(c.200G>A,Gac>Aac):ENSP00000410356(p.Asp68Asn)]	NA	chr6*153077285*153077285*COSM1075164*G*T*1*endometrium*1	NA	NA	NA	NA	NA	D	BN	NA	MODERATE	N	1	206	204	0	176	153	22	12.5
Chewer	IOB_03	chr6	168465635	G	A	FRMD1	SILENT	ENSG00000153303:[ENST00000283309(c.564C>T,tgC>tgT):ENSP00000283309(p.Cys188(=))]	NA	NA	NA	9.83E-06	0.00E+00	NA	NA	NA	NA	NA	MODIFIER	NA	NA	139	139	0	165	159	5	3.03
Chewer	IOB_03	chr7	38810881	A	T	VPS41	INTRONIC	ENSG00000006715:[ENST00000395969(c.1054-27T>A)]	NA	NA	2.96E-02	3.69E-01	4.75E-01	2.96E-02	1.02E-02	NA	NA	NA	MODIFIER	NA	NA	9	9	0	8	2	6	75
Chewer	IOB_03	chr7	76629387	A	G	DTX2P1	NCT	ENSG00000186704:[ENST00000425797(n.1169-241A>G)]	NA	NA	NA	NA	NA	NA	NA	NA	NA	NA	MODIFIER	NA	NA	16	16	0	20	13	7	35
Chewer	IOB_03	chr7	76629387	A	G	DTX2P1-UPK3BP1-PMS2P11	NCT	ENSG00000265479:[ENST00000579700(n.261-239A>G)]	NA	NA	NA	NA	NA	NA	NA	NA	NA	NA	MODIFIER	NA	NA	16	16	0	20	13	7	35
Chewer	IOB_03	chr7	76629387	A	G	LOC100505767	INTRONIC	100505767:[XM_005250741.1(c.829+29163T>C)]	NA	NA	NA	NA	NA	NA	NA	NA	NA	NA	MODIFIER	NA	NA	16	16	0	20	13	7	35
Chewer	IOB_03	chr7	76629387	A	G	UPK3B	INTRONIC	ENSG00000243566:[ENST00000443097(c.*268-18754A>G)]	NA	NA	NA	NA	NA	NA	NA	NA	NA	NA	MODIFIER	NA	NA	16	16	0	20	13	7	35
Chewer	IOB_03	chr7	100547349	A	G	MUC3A	NCT	ENSG00000169894:[ENST00000483366(n.131+32A>G)]	NA	NA	NA	9.84E-04	0.00E+00	NA	NA	NA	NA	NA	MODIFIER	NA	NA	24	23	0	30	21	6	20
Chewer	IOB_03	chr7	104702674	A	G	KMT2E	SILENT	ENSG00000005483:[ENST00000257745(c.135A>G,ttA>ttG):ENSP00000257745(p.Leu45(=))]	NA	chr7*104702672*104702674*COSM1083667*TTA*T*1*endometrium*1	NA	8.24E-06	0.00E+00	NA	NA	NA	NA	NA	MODIFIER	NA	NA	458	458	0	365	300	65	17.81
Chewer	IOB_03	chr7	123595132	C	A	SPAM1	SILENT	ENSG00000106304:[ENST00000223028(c.1036C>A,Cga>Aga):ENSP00000223028(p.Arg346(=))]	NA	NA	chr7*123595132*123595132*C*T*rs768838553*NA*NA*NA	NA	NA	NA	NA	NA	NA	NA	LOW	NA	0	173	172	0	114	109	5	4.39
Chewer	IOB_03	chr7	141464419	T	C	SSBP1	INTRONIC	ENSG00000106028:[ENST00000465582(c.*30+14235T>C)]	NA	NA	NA	NA	NA	NA	NA	D	PoD	NA	MODERATE	N	0	108	108	0	93	81	11	11.83
Chewer	IOB_03	chr7	141464419	T	C	TAS2R3	MISSENSE	ENSG00000127362:[ENST00000247879(c.461T>C,cTc>cCc):ENSP00000247879(p.Leu154Pro)]	NA	NA	NA	NA	NA	NA	NA	D	PoD	NA	MODERATE	N	0	108	108	0	93	81	11	11.83
Chewer	IOB_03	chr7	154379601	T	G	DPP6	MISSENSE	ENSG00000130226:[ENST00000406326(c.869T>G,cTg>cGg):ENSP00000384393(p.Leu290Arg)]	NA	NA	NA	NA	NA	NA	NA	NA	NA	NA	MODIFIER	NA	NA	198	198	0	260	249	9	3.46
Chewer	IOB_03	chr8	25364246	T	A	CDCA2	MISSENSE	ENSG00000184661:[ENST00000380665(c.2019T>A,aaT>aaA):ENSP00000370040(p.Asn673Lys)]	NA	NA	NA	NA	NA	NA	NA	NA	NA	NA	MODIFIER	NA	NA	89	88	0	64	58	6	9.38
Chewer	IOB_03	chr8	28575315	C	A	EXTL3	MISSENSE	ENSG00000012232:[ENST00000523149(c.587C>A,aCg>aAg):ENSP00000428691(p.Thr196Lys)]	NA	NA	chr8*28575315*28575315*C*T*rs923182549*NA*NA*NA	NA	NA	NA	NA	NA	NA	NA	MODIFIER	NA	NA	247	247	0	276	269	5	1.81
Chewer	IOB_03	chr8	100146981	T	C	VPS13B	INTRONIC	ENSG00000132549:[ENST00000355155(c.1302+26T>C)]	NA	NA	NA	NA	NA	NA	NA	NA	NA	NA	MODIFIER	NA	NA	29	29	0	32	23	9	28.12
Chewer	IOB_03	chr8	133036796	C	T	OC90	MISSENSE	ENSG00000258417:[ENST00000262283(c.2002G>A,Ggc>Agc):ENSP00000262283(p.Gly668Ser)]	NA	NA	7.99E-04	2.76E-04	8.57E-04	7.99E-04	0.00E+00	T	BN	NA	MODERATE	NA	0.022	60	59	0	75	55	20	26.67
Chewer	IOB_03	chr8	143745973	C	T	JRK	SILENT	8629:[NM_003724.3(c.1503G>A,gcG>gcA):NP_003715.3(p.Ala501(=))]	NA	NA	NA	2.10E-05	7.13E-05	NA	NA	NA	NA	NA	MODIFIER	NA	NA	151	151	0	223	186	37	16.59
Chewer	IOB_03	chr8	145535737	C	T	HSF1	MISSENSE	ENSG00000185122:[ENST00000528838(c.949C>T,Cgc>Tgc):ENSP00000431512(p.Arg317Cys)]	NA	NA	2.00E-04	3.69E-05	0.00E+00	2.00E-04	0.00E+00	NA	NA	NA	MODIFIER	NA	NA	125	123	0	196	185	5	2.55
Chewer	IOB_03	chr9	5300103	A	G	RLN2	MISSENSE	ENSG00000107014:[ENST00000381627(c.553T>C,Tgc>Cgc):ENSP00000371040(p.Cys185Arg)]	NA	NA	NA	NA	NA	NA	NA	NA	NA	NA	MODERATE	NA	0.902	124	122	0	90	84	6	6.67
Chewer	IOB_03	chr9	14819293	C	T	FREM1	MISSENSE	ENSG00000164946:[ENST00000380875(c.2485G>A,Gga>Aga):ENSP00000370257(p.Gly829Arg)]	NA	NA	NA	NA	NA	NA	NA	D	PrD	NA	MODERATE	D	0.862	67	67	0	58	51	7	12.07
Chewer	IOB_03	chr9	34665117	C	A	LOC730098	EXONIC-NC	730098:[NR_135148.1(n.401G>T)]	NA	NA	NA	NA	NA	NA	NA	NA	NA	NA	MODIFIER	NA	NA	94	94	0	178	171	5	2.81
Chewer	IOB_03	chr9	34665117	C	A	RP11-195F19.30	EXONIC-NC	ENSG00000261215:[ENST00000564224(n.251G>T)]	NA	NA	NA	NA	NA	NA	NA	NA	NA	NA	MODIFIER	NA	NA	94	94	0	178	171	5	2.81
Chewer	IOB_03	chr9	34665117	C	A	RP11-195F19.5	MISSENSE	ENSG00000187186:[ENST00000421828(c.178G>T,Ggt>Tgt):ENSP00000457701(p.Gly60Cys)]	NA	NA	NA	NA	NA	NA	NA	NA	NA	NA	MODIFIER	NA	NA	94	94	0	178	171	5	2.81
Chewer	IOB_03	chr9	37801287	C	T	DCAF10	MISSENSE	ENSG00000122741:[ENST00000242323(c.424C>T,Cgg>Tgg):ENSP00000242323(p.Arg142Trp)]	NA	NA	NA	NA	NA	NA	NA	NA	NA	NA	MODIFIER	NA	NA	205	205	0	304	297	6	1.97
Chewer	IOB_03	chr9	37801287	C	T	EXOSC3	NCT	ENSG00000107371:[ENST00000482614(n.85+63G>A)]	NA	NA	NA	NA	NA	NA	NA	NA	NA	NA	MODIFIER	NA	NA	205	205	0	304	297	6	1.97

Patel *et al.* , 2021. Whole-exome sequencing analysis of oral squamous cell carcinoma delineated by tobacco usage habits
Supplementary Table 4. List of somatic single nucleotide variants identified using whole exome sequencing of OSCC patients

Cohort	Sample	Chromosome	Genomic position	Reference allele	Alternate allele	Genesymbol	Variant type	Variant description	OncoMD	COSMIC	dbSNP	ExAC	ExAC_SAS	1000G	1000G_SAS	SIFT	Polyphen2	CONDEL	VEP	LRT	PhastCon7Way	Total reads in Normal	Reference read support in Normal	Alternative read support in Normal	Total reads in Tumor	Reference read support in Tumor	Alternate read support in Tumor	Alternate allele frequency in Tumor
Chewer	IOB_03	chr9	37801287	C	T	RP11-613M10.9	INTRONIC	ENSG00000255872:[ENST00000540557(c.*761-17227G>A)]	NA	NA	NA	NA	NA	NA	NA	NA	NA	NA	MODIFIER	NA	NA	205	205	0	304	297	6	1.97
Chewer	IOB_03	chr9	68415334	C	T	MIR4477A	EXONIC-NC	100616184:[NR_039688.1(n.55G>A)]	NA	NA	NA	6.86E-03	2.59E-02	NA	NA	NA	NA	NA	MODIFIER	NA	NA	208	206	0	283	273	7	2.47
Chewer	IOB_03	chr9	86924550	T	G	SLC28A3	MISSENSE	ENSG00000197506:[ENST00000376238(c.236A>C,cAa>cCa):ENSP00000365413(p.Gln79Pro)]	NA	NA	NA	NA	NA	NA	NA	NA	NA	NA	MODIFIER	NA	NA	106	104	1	97	85	11	11.34
Chewer	IOB_03	chr9	141000133	G	A	CACNA1B	INTRONIC	774:[XM_005266103.1(c.5326-18G>A)]	NA	NA	NA	2.55E-04	2.58E-04	NA	NA	NA	NA	NA	MODIFIER	NA	NA	185	185	0	216	166	50	23.15
Chewer	IOB_03	chrX	54574763	C	T	GNL3L	SILENT	ENSG00000130119:[ENST00000336470(c.732C>T,tgC>tgT):ENSP00000338573(p.Cys244(=))]	NA	NA	NA	NA	NA	NA	NA	NA	NA	NA	LOW	NA	0.935	86	86	0	67	62	5	7.46
Chewer	IOB_03	chrX	78427141	C	A	GPR174	MISSENSE	ENSG00000147138:[ENST00000276077(c.637C>A,Caa>Aaa):ENSP00000276077(p.Gln213Lys)]	NA	NA	NA	NA	NA	NA	NA	T	BN	NA	MODERATE	N	1	98	98	0	122	117	5	4.1
Chewer	IOB_03	chrX	84526229	C	T	ZNF711	MISSENSE	ENSG00000147180:[ENST00000360700(c.1819C>T,Cca>Tca):ENSP00000353922(p.Pro607Ser)]	NA	chrX*84526229*84526229*COSM1180352*C*T*1*prostate*1|chrX*84526229*84526229*COSM1180353*C*T*1*prostate*1	NA	NA	NA	NA	NA	D	PoD	NA	MODERATE	D	1	88	87	1	97	92	5	5.15
Chewer	IOB_03	chrX	118925635	C	A	NA	INTERGENIC	NA	NA	NA	NA	NA	NA	NA	NA	NA	NA	NA	MODIFIER	NA	NA	42	42	0	63	56	5	7.94
Chewer	IOB_03	chrX	129149524	C	A	BCORL1	MISSENSE	ENSG00000085185:[ENST00000441294(c.1082C>A,Ctc>Atc):ENSP00000416520(p.Leu362Ile)]	NA	NA	NA	NA	NA	NA	NA	D_lc	PoD	NA	MODERATE	NA	NA	135	135	0	119	110	7	5.88
Chewer	IOB_04	chr1	1250351	C	A	CPSF3L	NCT	ENSG00000127054:[ENST00000488042(n.585-9G>T)]	NA	NA	chr1*1250351*1250351*C*T*rs200181216*0*0.9998*0.0001997|chr1*1250351*1250352*CG*C*rs772214806*NA*NA*NA	NA	NA	NA	NA	NA	NA	NA	MODIFIER	NA	NA	245	244	1	124	117	7	5.65
Chewer	IOB_04	chr1	6156810	G	A	KCNAB2	MISSENSE	ENSG00000069424:[ENST00000458166(c.718G>A,Gcc>Acc):ENSP00000396167(p.Ala240Thr)]	NA	NA	NA	NA	NA	NA	NA	D	BN	NA	MODERATE	D	1	98	98	0	85	80	5	5.88
Chewer	IOB_04	chr1	11708291	C	T	FBXO2	NCT	26232:[XR_244788.1(n.1392-320G>A)]	NA	NA	NA	NA	NA	NA	NA	NA	NA	NA	MODIFIER	NA	NA	122	122	0	98	92	6	6.12
Chewer	IOB_04	chr1	22211919	G	A	HSPG2	SILENT	ENSG00000142798:[ENST00000374695(c.1104C>T,tgC>tgT):ENSP00000363827(p.Cys368(=))]	NA	NA	NA	NA	NA	NA	NA	NA	NA	NA	LOW	NA	0	132	132	0	89	83	5	5.62
Chewer	IOB_04	chr1	27587502	G	A	WDTC1	5UTR	ENSG00000142784:[ENST00000319394(c.-44G>A)]	NA	NA	NA	NA	NA	NA	NA	NA	NA	NA	MODIFIER	NA	NA	190	190	0	99	93	6	6.06
Chewer	IOB_04	chr1	38453497	C	A	SF3A3	MISSENSE	ENSG00000183431:[ENST00000373019(c.156G>T,gaG>gaT):ENSP00000362110(p.Glu52Asp)]	NA	NA	chr1*38453497*38453497*C*T*rs375274659*NA*NA*NA	NA	NA	NA	NA	NA	NA	NA	MODIFIER	NA	NA	348	345	0	200	193	5	2.5
Chewer	IOB_04	chr1	55119637	C	T	MROH7	SILENT	ENSG00000184313:[ENST00000339553(c.1038C>T,agC>agT):ENSP00000343211(p.Ser346(=))]	NA	NA	NA	8.28E-06	0.00E+00	NA	NA	NA	NA	NA	MODIFIER	NA	NA	290	290	0	208	201	7	3.37
Chewer	IOB_04	chr1	55119637	C	T	MROH7-TTC4	SILENT	ENSG00000271723:[ENST00000414150(c.1038C>T,agC>agT):ENSP00000410192(p.Ser346(=))]	NA	NA	NA	8.28E-06	0.00E+00	NA	NA	NA	NA	NA	MODIFIER	NA	NA	290	290	0	208	201	7	3.37
Chewer	IOB_04	chr1	74901882	A	T	FPGT-TNNI3K	INTRONIC	ENSG00000259030:[ENST00000557284(c.2167+66A>T)]	NA	NA	NA	NA	NA	NA	NA	NA	NA	NA	MODIFIER	NA	NA	185	178	1	125	118	6	4.8
Chewer	IOB_04	chr1	74901882	A	T	TNNI3K	INTRONIC	ENSG00000116783:[ENST00000534020(c.464-276A>T)]	NA	NA	NA	NA	NA	NA	NA	NA	NA	NA	MODIFIER	NA	NA	185	178	1	125	118	6	4.8
Chewer	IOB_04	chr1	79002151	C	A	PTGFR	MISSENSE	ENSG00000122420:[ENST00000370757(c.859C>A,Ctt>Att):ENSP00000359793(p.Leu287Ile)]	NA	NA	NA	NA	NA	NA	NA	NA	NA	NA	MODIFIER	NA	NA	346	343	0	293	266	26	8.87
Chewer	IOB_04	chr1	82432201	G	C	LPHN2	MISSENSE	ENSG00000117114:[ENST00000319517(c.2206G>C,Gtg>Ctg):ENSP00000322270(p.Val736Leu)]	NA	NA	NA	NA	NA	NA	NA	T	PrD	NA	MODERATE	D	1	311	311	0	257	237	20	7.78
Chewer	IOB_04	chr1	82456392	G	A	ADGRL2	MISSENSE	23266:[XM_005270668.1(c.3904G>A,Ggg>Agg):XP_005270725.1(p.Gly1302Arg)]	NA	NA	NA	NA	NA	NA	NA	NA	NA	NA	MODIFIER	NA	NA	182	182	0	127	112	13	10.24
Chewer	IOB_04	chr1	82456392	G	A	LPHN2	MISSENSE	ENSG00000117114:[ENST00000370728(c.3943G>A,Ggg>Agg):ENSP00000359763(p.Gly1315Arg)]	NA	NA	NA	NA	NA	NA	NA	NA	NA	NA	MODIFIER	NA	NA	182	182	0	127	112	13	10.24
Chewer	IOB_04	chr1	99753690	C	T	LPPR4	MISSENSE	ENSG00000117600:[ENST00000370185(c.392C>T,gCt>gTt):ENSP00000359204(p.Ala131Val)]	NA	NA	NA	NA	NA	NA	NA	D	BN	NA	MODERATE	N	1	313	311	0	206	201	5	2.43
Chewer	IOB_04	chr1	114515802	G	A	HIPK1	MISSENSE	ENSG00000163349:[ENST00000369561(c.3199G>A,Gcc>Acc):ENSP00000358574(p.Ala1067Thr)]	NA	NA	chr1*114515802*114515802*G*T*rs1025837734*NA*NA*NA	NA	NA	NA	NA	NA	NA	NA	MODERATE	NA	1	314	314	0	217	211	6	2.76
Chewer	IOB_04	chr1	120510261	C	T	NOTCH2	INTRONIC	4853:[XM_005270902.1(c.1148-17G>A)]	NA	NA	chr1*120510261*120510261*C*G,T*rs782463239*NA*NA*NA	8.26E-06	6.06E-05	NA	NA	NA	NA	NA	MODIFIER	NA	NA	183	183	0	133	125	8	6.02
Chewer	IOB_04	chr1	168054935	T	C	GPR161	MISSENSE	ENSG00000143147:[ENST00000537209(c.1484A>G,aAa>aGa):ENSP00000441039(p.Lys495Arg)]	NA	NA	NA	NA	NA	NA	NA	NA	NA	NA	MODIFIER	NA	NA	400	399	0	299	288	10	3.34
Chewer	IOB_04	chr1	173638537	C	A	ANKRD45	INTRONIC	ENSG00000183831:[ENST00000333279(c.-16+419G>T)]	NA	NA	chr1*173638537*173638537*C*CA*rs537758244*NA*NA*NA|chr1*173638537*173638538*CA*C*rs58611776*NA*NA*NA|chr1*173638537*173638538*CA*C*rs759046558*NA*NA*NA	NA	NA	NA	NA	NA	NA	NA	MODIFIER	NA	NA	77	70	4	43	34	7	16.28
Chewer	IOB_04	chr1	197396888	A	C	CRB1	SILENT	ENSG00000134376:[ENST00000367400(c.2433A>C,tcA>tcC):ENSP00000356370(p.Ser811(=))]	NA	NA	NA	NA	NA	NA	NA	NA	NA	NA	LOW	NA	NA	416	415	0	246	232	13	5.28
Chewer	IOB_04	chr1	214638071	G	A	PTPN14	MISSENSE	ENSG00000152104:[ENST00000366956(c.76C>T,Cgc>Tgc):ENSP00000355923(p.Arg26Cys)]	NA	NA	NA	NA	NA	NA	NA	NA	NA	NA	MODIFIER	NA	NA	500	500	0	392	379	10	2.55
Chewer	IOB_04	chr1	228528833	G	A	OBSCN	MISSENSE	ENSG00000154358:[ENST00000570156(c.20606G>A,cGc>cAc):ENSP00000455507(p.Arg6869His)]	NA	NA	NA	7.18E-05	0.00E+00	NA	NA	NA	PrD	NA	MODERATE	D	1	489	487	2	417	408	6	1.44
Chewer	IOB_04	chr10	6258743	G	T	PFKFB3	MISSENSE-SS-PRX	ENSG00000170525:[ENST00000540253(c.483G>T,aaG>aaT):ENSP00000446384(p.Lys161Asn)]	NA	NA	NA	NA	NA	NA	NA	NA	PoD	NA	MODERATE	D	1	240	240	0	187	179	6	3.21
Chewer	IOB_04	chr10	50533995	G	A	C10orf71	SILENT	ENSG00000177354:[ENST00000374144(c.3405G>A,cgG>cgA):ENSP00000363259(p.Arg1135(=))]	NA	NA	NA	NA	NA	NA	NA	NA	NA	NA	MODIFIER	NA	NA	392	392	0	377	363	12	3.18
Chewer	IOB_04	chr10	72015183	C	T	NPFFR1	MISSENSE	ENSG00000148734:[ENST00000277942(c.823G>A,Gtg>Atg):ENSP00000277942(p.Val275Met)]	NA	NA	NA	NA	NA	NA	NA	D	PoD	NA	MODERATE	D	1	670	670	0	522	507	13	2.49
Chewer	IOB_04	chr10	72015184	C	T	NPFFR1	MISSENSE	ENSG00000148734:[ENST00000277942(c.822G>A,atG>atA):ENSP00000277942(p.Met274Ile)]	NA	NA	NA	NA	NA	NA	NA	T	BN	NA	MODERATE	N	0.017	668	668	0	518	504	13	2.51
Chewer	IOB_04	chr10	89272874	C	T	MINPP1	INTRONIC-SS-ACR-PRX	ENSG00000107789:[ENST00000536010(c.233-7C>T)]	NA	NA	NA	NA	NA	NA	NA	NA	NA	NA	LOW	NA	NA	247	246	0	148	142	6	4.05
Chewer	IOB_04	chr10	96749286	T	G	CYP2C9	MISSENSE	1559:[XM_005269575.1(c.1338T>G,ttT>ttG):XP_005269632.1(p.Phe446Leu)]	NA	NA	chr10*96749276*96749291*GGAAGCTATTTTGGGT*G*rs752098372*NA*NA*N[illegible]	NA	NA	NA	NA	NA	NA	NA	MODERATE	NA	NA	182	180	1	127	120	6	4.72
Chewer	IOB_04	chr10	98116282	C	A	OPALIN	INTRONIC	93377:[NM_001284322.1(c.-109-1235G>T)]	NA	NA	NA	NA	NA	NA	NA	NA	NA	NA	MODIFIER	NA	NA	143	142	0	103	98	5	4.85
Chewer	IOB_04	chr10	117221444	C	T	ATRNL1	INTRONIC-SS-ACR-PRX	ENSG00000107518:[ENST00000526373(c.573-7C>T)]	NA	NA	NA	NA	NA	NA	NA	NA	NA	NA	MODIFIER	NA	NA	155	155	0	114	109	5	4.39
Chewer	IOB_04	chr11	4870363	G	A	MMP26	INTRONIC	ENSG00000167346:[ENST00000380390(c.-145+81792G>A)]	NA	NA	NA	8.26E-06	0.00E+00	NA	NA	NA	NA	NA	MODIFIER	NA	NA	218	218	0	143	135	5	3.5
Chewer	IOB_04	chr11	4870363	G	A	OR51S1	MISSENSE	ENSG00000176922:[ENST00000322101(c.76C>T,Cca>Tca):ENSP00000322754(p.Pro26Ser)]	NA	NA	NA	8.26E-06	0.00E+00	NA	NA	NA	NA	NA	MODIFIER	NA	NA	218	218	0	143	135	5	3.5
Chewer	IOB_04	chr11	8940861	C	A	AKIP1	INTRONIC	ENSG00000166452:[ENST00000309377(c.490-23C>A)]	NA	NA	chr11*8940861*8940861*C*T*rs764779602*NA*NA*NA	NA	NA	NA	NA	NA	NA	NA	MODIFIER	NA	NA	65	64	0	73	67	6	8.22
Chewer	IOB_04	chr11	62383716	A	C	B3GAT3	NCT	26229:[NR_109991.1(n.1127+262T>G)]	NA	NA	NA	2.06E-01	1.25E-01	NA	NA	NA	NA	NA	MODIFIER	NA	NA	82	64	7	40	27	9	22.5
Chewer	IOB_04	chr11	63277885	G	A	LGALS12	INTRONIC	ENSG00000133317:[ENST00000415491(c.376-50G>A)]	NA	NA	NA	NA	NA	NA	NA	NA	NA	NA	MODIFIER	NA	NA	94	94	0	60	50	10	16.67
Chewer	IOB_04	chr11	69469211	G	T	CCND1	3UTR	ENSG00000110092:[ENST00000227507(c.*3161G>T)]	NA	NA	NA	NA	NA	NA	NA	NA	NA	NA	MODIFIER	NA	NA	161	161	0	118	113	5	4.24
Chewer	IOB_04	chr11	69469211	G	T	ORAOV1	NCT	ENSG00000149716:[ENST00000542515(n.1832-59C>A)]	NA	NA	NA	NA	NA	NA	NA	NA	NA	NA	MODIFIER	NA	NA	161	161	0	118	113	5	4.24
Chewer	IOB_04	chr11	73961958	C	A	P4HA3	NCT	283208:[XR_247196.1(n.2136-2912G>T)]	NA	NA	NA	NA	NA	NA	NA	NA	NA	NA	MODIFIER	NA	NA	91	91	0	57	48	8	14.04
Chewer	IOB_04	chr11	73961958	C	A	PPME1	NCT	ENSG00000214517:[ENST00000541340(n.1645-41C>A)]	NA	NA	NA	NA	NA	NA	NA	NA	NA	NA	MODIFIER	NA	NA	91	91	0	57	48	8	14.04
Chewer	IOB_04	chr11	103044807	A	G	DYNC2H1	MISSENSE	ENSG00000187240:[ENST00000375735(c.5582A>G,cAt>cGt):ENSP00000364887(p.His1861Arg)]	NA	NA	NA	NA	NA	NA	NA	D	PrD	NA	MODERATE	NA	NA	315	312	1	227	189	38	16.74
Chewer	IOB_04	chr11	123810963	C	A	OR4D5	MISSENSE	ENSG00000171014:[ENST00000307033(c.640C>A,Ctt>Att):ENSP00000305970(p.Leu214Ile)]	NA	chr11*123810963*123810963*COSM1197476*C*A*2*lung*2	NA	NA	NA	NA	NA	D	PoD	NA	MODERATE	N	0	532	530	1	406	387	17	4.19
Chewer	IOB_04	chr12	464388	G	A	KDM5A	MISSENSE	ENSG00000073614:[ENST00000382815(c.806C>T,aCc>aTc):ENSP00000372265(p.Thr269Ile)]	NA	NA	.	NA	NA	NA	NA	T	BN	NA	MODERATE	N	1	342	341	0	207	202	5	2.42
Chewer	IOB_04	chr12	7528396	G	A	CD163L1	SILENT	ENSG00000177675:[ENST00000313599(c.2586C>T,ttC>ttT):ENSP00000315945(p.Phe862(=))]	NA	NA	NA	NA	NA	NA	NA	NA	NA	NA	LOW	NA	0	472	472	0	339	316	19	5.6
Chewer	IOB_04	chr12	132905744	G	A	GALNT9	SILENT	ENSG00000182870:[ENST00000328957(c.46C>T,Ctg>Ttg):ENSP00000329846(p.Leu16(=))]	NA	NA	NA	NA	NA	NA	NA	NA	NA	NA	LOW	NA	0.97	593	591	0	536	513	20	3.73
Chewer	IOB_04	chr13	32812163	T	G	FRY	INTRONIC	ENSG00000073910:[ENST00000380250(c.6418+40T>G)]	NA	NA	NA	NA	NA	NA	NA	NA	NA	NA	MODIFIER	NA	NA	113	113	0	58	45	13	22.41
Chewer	IOB_04	chr13	39454710	C	A	FREM2	MISSENSE	ENSG00000150893:[ENST00000280481(c.9296C>A,cCc>cAc):ENSP00000280481(p.Pro3099His)]	NA	NA	chr13*39454710*39454710*C*T*rs777019274*NA*NA*NA	NA	NA	NA	NA	T_lc	PoD	NA	MODERATE	D	0.995	218	217	1	166	161	5	3.01
Chewer	IOB_04	chr14	23236249	C	T	OXA1L	MISSENSE	ENSG00000155463:[ENST00000358043(c.4C>T,Cgg>Tgg):ENSP00000350740(p.Arg2Trp)]	NA	NA	NA	NA	NA	NA	NA	NA	NA	NA	MODIFIER	NA	NA	381	381	0	242	235	6	2.48
Chewer	IOB_04	chr15	22935922	C	A	CYFIP1	SILENT	ENSG00000068793:[ENST00000313077(c.858C>A,gcC>gcA):ENSP00000324549(p.Ala286(=))]	NA	NA	NA	NA	NA	NA	NA	NA	NA	NA	LOW	NA	NA	296	293	1	210	204	6	2.86
Chewer	IOB_04	chr15	33023007	C	A	GREM1	MISSENSE	ENSG00000166923:[ENST00000300177(c.116C>A,gCc>gAc):ENSP00000300177(p.Ala39Asp)]	NA	NA	chr15*33023007*33023007*C*T*rs768170664*NA*NA*NA	NA	NA	NA	NA	NA	NA	NA	MODIFIER	NA	NA	158	158	0	157	149	6	3.82
Chewer	IOB_04	chr15	35193002	G	A	AQR	SILENT	ENSG00000021776:[ENST00000156471(c.2064C>T,caC>caT):ENSP00000156471(p.His688(=))]	NA	NA	NA	4.98E-05	6.15E-05	NA	NA	NA	NA	NA	LOW	NA	1	319	317	0	229	217	12	5.24
Chewer	IOB_04	chr15	41387976	C	A	INO80	SILENT	ENSG00000128908:[ENST00000361937(c.294G>T,ctG>ctT):ENSP00000355205(p.Leu98(=))]	NA	NA	NA	NA	NA	NA	NA	NA	NA	NA	MODIFIER	NA	NA	266	265	0	181	174	6	3.31
Chewer	IOB_04	chr15	86182678	C	T	AKAP13	MISSENSE-SS-PRX	ENSG00000170776:[ENST00000560340(n.451C>T)]	NA	NA	NA	NA	NA	NA	NA	NA	NA	NA	MODIFIER	NA	NA	145	145	0	81	75	5	6.17
Chewer	IOB_04	chr15	86182678	C	T	RP11-815J21.4	NCT	ENSG00000259367:[ENST00000558980(n.265-19429G>A)]	NA	NA	NA	NA	NA	NA	NA	NA	NA	NA	MODIFIER	NA	NA	145	145	0	81	75	5	6.17
Chewer	IOB_04	chr15	101120900	C	T	LINS	MISSENSE	ENSG00000140471:[ENST00000314742(c.148G>A,Gca>Aca):ENSP00000318423(p.Ala50Thr)]	NA	NA	NA	NA	NA	NA	NA	NA	NA	NA	MODIFIER	NA	NA	142	141	0	80	73	6	7.5
Chewer	IOB_04	chr16	2946615	G	A	FLYWCH2	SILENT	ENSG00000162076:[ENST00000293981(c.165G>A,gcG>gcA):ENSP00000293981(p.Ala55(=))]	NA	NA	chr16*2946614*2946615*CG*C*rs768211139*NA*NA*NA	NA	NA	NA	NA	NA	NA	NA	LOW	NA	NA	609	606	0	453	431	21	4.64
Chewer	IOB_04	chr16	6704547	C	T	RBFOX1	INTRONIC	ENSG00000078328:[ENST00000547605(c.-63-57C>T)]	NA	NA	NA	NA	NA	NA	NA	NA	NA	NA	MODIFIER	NA	NA	355	355	0	265	252	13	4.91
Chewer	IOB_04	chr16	6704547	C	T	RP11-420N3.2	NCT	ENSG00000260411:[ENST00000569895(n.500-57C>T)]	NA	NA	NA	NA	NA	NA	NA	NA	NA	NA	MODIFIER	NA	NA	355	355	0	265	252	13	4.91

Patel *et al.* , 2021. Whole-exome sequencing analysis of oral squamous cell carcinoma delineated by tobacco usage habits
Supplementary Table 4. List of somatic single nucleotide variants identified using whole exome sequencing of OSCC patients

Cohort	Sample	Chromosome	Genomic position	Reference allele	Alternate allele	Genesymbol	Variant type	Variant description	OncoMD	COSMIC	dbSNP	ExAC	ExAC_SAS	1000G	1000G_SAS	SIFT	Polyphen2	CONDEL	VEP	LRT	PhastCon7Way	Total reads in Normal	Reference read support in Normal	Alternative read support in Normal	Total reads in Tumor	Reference read support in Tumor	Alternate read support in Tumor	Alternate allele frequency in Tumor
Chewer	IOB_04	chr16	7759108	C	G	RBFOX1	MISSENSE	ENSG00000078328:[ENST00000355637(c.1162C>G,Ccc>Gcc):ENSP00000347855(p.Pro388Ala)]	NA	chr16*7759107*7759108*COSM1379974*GC*G*1*large_intestine*1|chr16*7759107*7759108*COSM1379975*GC*G*1*large_intestine*1|chr16*7759107*7759108*COSM1379976*GC*G*1*large_intestine*1	NA	NA	NA	NA	NA	D_lc	UN	NA	MODERATE	NA	NA	185	185	0	152	145	6	3.95
Chewer	IOB_04	chr16	20810717	C	A	ERI2	INTRONIC	ENSG00000196678:[ENST00000389345(c.-174-22G>T)]	NA	NA	NA	NA	NA	NA	NA	NA	NA	NA	MODIFIER	NA	NA	368	367	0	190	185	5	2.63
Chewer	IOB_04	chr16	48242421	A	G	ABCC11	INTRONIC	ENSG00000121270:[ENST00000353782(c.1609-14T>C)]	NA	NA	NA	NA	NA	NA	NA	NA	NA	NA	MODIFIER	NA	NA	184	184	0	121	116	5	4.13
Chewer	IOB_04	chr16	85024131	C	T	ZDHHC7	MISSENSE	ENSG00000153786:[ENST00000313732(c.94G>A,Gag>Aag):ENSP00000315604(p.Glu32Lys)]	NA	NA	chr16*85024131*85024131*C*CGGA*rs913300297*NA*NA*NA|chr16*85024131*85024134*CGGA*C*rs762809085*NA*NA*NA	NA	NA	NA	NA	NA	NA	NA	MODIFIER	NA	NA	501	500	0	378	371	7	1.85
Chewer	IOB_04	chr17	1960974	C	A	HIC1	SILENT	ENSG00000177374:[ENST00000322941(c.1047C>A,cgC>cgA):ENSP00000314080(p.Arg349(=))]	NA	NA	chr17*1960974*1960974*C*T*rs77393586*1*0.8562*0.1438	NA	NA	NA	NA	NA	NA	NA	LOW	NA	NA	296	296	0	190	184	6	3.16
Chewer	IOB_04	chr17	21199083	T	A	MAP2K3	INTRONIC-SS-DNR	ENSG00000034152:NCT[ENST00000583508(n.260-2642T>A)]	NA	NA	NA	4.86E-01	4.87E-01	NA	NA	NA	NA	NA	MODIFIER	NA	NA	236	196	30	217	142	72	33.18
Chewer	IOB_04	chr17	56676403	C	T	TEX14	MISSENSE	ENSG00000121101:[ENST00000240361(c.2321G>A,cGc>cAc):ENSP00000240361(p.Arg774His)]	NA	NA	2.00E-04	4.12E-05	0.00E+00	2.00E-04	0.00E+00	T	BN	NA	MODERATE	N	0	351	348	1	249	241	7	2.81
Chewer	IOB_04	chr17	65074524	C	T	HELZ	SILENT	ENSG00000198265:[ENST00000580168(c.5676G>A,aaG>aaA):ENSP00000464512(p.Lys1892(=))]	NA	NA	NA	NA	NA	NA	NA	NA	NA	NA	LOW	NA	0.99	150	149	0	123	118	5	4.07
Chewer	IOB_04	chr17	72943373	C	T	OTOP3	MISSENSE	ENSG00000182938:[ENST00000328801(c.1423C>T,Cgc>Tgc):ENSP00000328090(p.Arg475Cys)]	NA	NA	NA	5.90E-05	6.09E-05	NA	NA	NA	NA	NA	MODIFIER	NA	NA	311	307	1	263	254	9	3.42
Chewer	IOB_04	chr17	72947566	C	A	HID1	3UTR	ENSG00000167861:[ENST00000425042(c.*99G>T)]	NA	NA	NA	NA	NA	NA	NA	NA	NA	NA	MODIFIER	NA	NA	209	208	1	139	133	5	3.6
Chewer	IOB_04	chr17	75199713	G	A	SEC14L1	SILENT	ENSG00000129657:[ENST00000431431(c.969G>A,gcG>gcA):ENSP00000389838(p.Ala323(=))]	NA	NA	NA	2.67E-05	0.00E+00	NA	NA	NA	NA	NA	LOW	NA	NA	189	187	1	86	81	5	5.81
Chewer	IOB_04	chr17	80320487	C	A	TEX19	MISSENSE	ENSG00000182459:[ENST00000333437(c.461C>A,aCc>aAc):ENSP00000331500(p.Thr154Asn)]	NA	NA	NA	NA	NA	NA	NA	T	BN	NA	MODERATE	NA	NA	345	344	0	253	245	6	2.37
Chewer	IOB_04	chr18	19024324	C	A	GREB1L	MISSENSE	ENSG00000141449:[ENST00000269218(c.1347C>A,agC>agA):ENSP00000269218(p.Ser449Arg)]	NA	NA	chr18*19024324*19024324*C*T*rs769947250*NA*NA*NA	NA	NA	NA	NA	NA	NA	NA	MODIFIER	NA	NA	332	332	0	226	218	6	2.65
Chewer	IOB_04	chr18	19024324	C	A	RP11-296E23.1	NCT	ENSG00000265751:[ENST00000584611(n.135+6520G>T)]	NA	NA	chr18*19024324*19024324*C*T*rs769947250*NA*NA*NA	NA	NA	NA	NA	NA	NA	NA	MODIFIER	NA	NA	332	332	0	226	218	6	2.65
Chewer	IOB_04	chr18	47363917	A	G	MYO5B	MISSENSE	ENSG00000167306:[ENST00000324581(c.2453T>C,gTc>gCc):ENSP00000315531(p.Val818Ala)]	NA	chr18*47363917*47363917*COSM473883*A*G*6*lung,kidney,endometrium,large_intestine*1,2,2,1	NA	7.12E-02	1.70E-01	NA	NA	NA	NA	NA	MODIFIER	NA	NA	149	149	0	92	86	6	6.52
Chewer	IOB_04	chr18	47363917	A	G	RP11-886H22.1	MISSENSE	ENSG00000266997:[ENST00000590532(c.77T>C,gTc>gCc):ENSP00000467396(p.Val26Ala)]	NA	chr18*47363917*47363917*COSM473883*A*G*6*lung,kidney,endometrium,large_intestine*1,2,2,1	NA	7.12E-02	1.70E-01	NA	NA	NA	NA	NA	MODIFIER	NA	NA	149	149	0	92	86	6	6.52
Chewer	IOB_04	chr18	47363917	A	G	SCARNA17	NCT	ENSG00000267322:[ENST00000589499(n.41-1729A>G)]	NA	chr18*47363917*47363917*COSM473883*A*G*6*lung,kidney,endometrium,large_intestine*1,2,2,1	NA	7.12E-02	1.70E-01	NA	NA	NA	NA	NA	MODIFIER	NA	NA	149	149	0	92	86	6	6.52
Chewer	IOB_04	chr19	1556743	C	A	MEX3D	MISSENSE	ENSG00000181588:[ENST00000605173(c.247G>T,Ggt>Tgt):ENSP00000475059(p.Gly83Cys)]	NA	NA	2.00E-04	8.87E-06	0.00E+00	2.00E-04	0.00E+00	D	PoD	NA	MODERATE	U	0.686	381	380	0	286	279	5	1.75
Chewer	IOB_04	chr19	1905311	C	T	ADAT3	5UTR	113179:[NM_138422.3(c.-286C>T)]	NA	NA	NA	NA	NA	NA	NA	NA	NA	NA	MODIFIER	NA	NA	75	75	0	47	42	5	10.64
Chewer	IOB_04	chr19	1905311	C	T	SCAMP4	5UTR	113178:[NM_001329539.1(c.-253C>T)]	NA	NA	NA	NA	NA	NA	NA	NA	NA	NA	MODIFIER	NA	NA	75	75	0	47	42	5	10.64
Chewer	IOB_04	chr19	4234700	C	A	EBI3	MISSENSE	ENSG00000105246:[ENST00000221847(c.416C>A,cCc>cAc):ENSP00000221847(p.Pro139His)]	NA	NA	chr19*4234700*4234700*C*G,T*rs542032229*0*0.9998*0.0001997,.	NA	NA	NA	NA	T	BN	NA	MODERATE	N	0	298	298	0	226	221	5	2.21
Chewer	IOB_04	chr19	6008014	C	A	CTC-232P5.1	NCT	ENSG00000266983:[ENST00000587836(n.113-4117C>A)]	NA	NA	NA	NA	NA	NA	NA	NA	NA	NA	MODIFIER	NA	NA	189	188	1	134	127	6	4.48
Chewer	IOB_04	chr19	6008014	C	A	RFX2	INTRONIC	ENSG00000087903:[ENST00000303657(c.1134+103G>T)]	NA	NA	NA	NA	NA	NA	NA	NA	NA	NA	MODIFIER	NA	NA	189	188	1	134	127	6	4.48
Chewer	IOB_04	chr19	12845360	C	T	C19orf43	MISSENSE	ENSG00000123144:[ENST00000242784(c.112G>A,Gag>Aag):ENSP00000242784(p.Glu38Lys)]	NA	NA	NA	NA	NA	NA	NA	T	BN	NA	MODERATE	N	0.002	308	306	0	255	233	20	7.84
Chewer	IOB_04	chr19	19004203	G	A	CERS1	INTRONIC	ENSG00000223802:[ENST00000542296(c.115+22C>T)]	NA	NA	NA	NA	NA	NA	NA	NA	NA	NA	MODIFIER	NA	NA	150	150	0	112	107	5	4.46
Chewer	IOB_04	chr19	19004203	G	A	GDF1	INTRONIC	ENSG00000130283:[ENST00000247005(c.-914+22C>T)]	NA	NA	NA	NA	NA	NA	NA	NA	NA	NA	MODIFIER	NA	NA	150	150	0	112	107	5	4.46
Chewer	IOB_04	chr19	21299810	A	C	ZNF714	MISSENSE	ENSG00000160352:[ENST00000596143(c.340A>C,Aaa>Caa):ENSP00000472368(p.Lys114Gln)]	NA	NA	NA	NA	NA	NA	NA	NA	NA	NA	MODIFIER	NA	NA	186	185	0	119	112	7	5.88
Chewer	IOB_04	chr19	39200085	C	A	ACTN4	NONSENSE	ENSG00000130402:[ENST00000252699(c.702C>A,taC>taA):ENSP00000252699(p.Tyr234Ter)]	NA	NA	NA	NA	NA	NA	NA	NA	NA	NA	MODIFIER	NA	NA	399	397	0	225	218	6	2.67
Chewer	IOB_04	chr19	40172053	C	A	LGALS17A	NCT	ENSG00000226025:[ENST00000458539(n.42+1979C>A)]	NA	NA	NA	NA	NA	NA	NA	NA	NA	NA	MODIFIER	NA	NA	130	130	0	100	94	5	5
Chewer	IOB_04	chr19	47700695	C	A	SAE1	INTRONIC	ENSG00000142230:[ENST00000596995(c.*809+61C>A)]	NA	NA	NA	NA	NA	NA	NA	NA	NA	NA	MODIFIER	NA	NA	170	170	0	86	81	5	5.81
Chewer	IOB_04	chr19	50962301	C	A	MYBPC2	INTRONIC	ENSG00000086967:[ENST00000357701(c.2595+38C>A)]	NA	NA	NA	NA	NA	NA	NA	NA	NA	NA	MODIFIER	NA	NA	242	240	0	153	146	6	3.92
Chewer	IOB_04	chr19	51170224	G	A	SHANK1	MISSENSE	ENSG00000161681:[ENST00000391814(c.5017C>T,Ccg>Tcg):ENSP00000375690(p.Pro1673Ser)]	NA	NA	NA	NA	NA	NA	NA	T	BN	NA	MODERATE	U	0.127	179	178	0	121	115	5	4.13
Chewer	IOB_04	chr19	51170224	G	A	SYT3	INTRONIC	ENSG00000213023:[ENST00000544769(c.-518+1386C>T)]	NA	NA	NA	NA	NA	NA	NA	T	BN	NA	MODERATE	U	0.127	179	178	0	121	115	5	4.13
Chewer	IOB_04	chr19	56758677	C	A	ZSCAN5A	INTRONIC	79149:[NM_001322078.1(c.34-23513G>T)]	NA	NA	chr19*56758677*56758677*C*T*rs759111798*NA*NA*NA	NA	NA	NA	NA	NA	NA	NA	MODIFIER	NA	NA	188	188	0	155	150	5	3.23
Chewer	IOB_04	chr19	56758677	C	A	ZSCAN5D	SILENT	ENSG00000267908:[ENST00000600684(c.1305C>A,ccC>ccA):ENSP00000470691(p.Pro435(=))]	NA	NA	chr19*56758677*56758677*C*T*rs759111798*NA*NA*NA	NA	NA	NA	NA	NA	NA	NA	MODIFIER	NA	NA	188	188	0	155	150	5	3.23
Chewer	IOB_04	chr19	58773664	C	A	CTD-3138B18.4	INTRONIC	ENSG00000269545:[ENST00000600029(c.*57+15504C>A)]	NA	NA	NA	NA	NA	NA	NA	NA	NA	NA	MODIFIER	NA	NA	204	204	0	146	140	6	4.11
Chewer	IOB_04	chr19	58773664	C	A	CTD-3138B18.5	NCT	ENSG00000268516:[ENST00000597230(n.287-2607G>T)]	NA	NA	NA	NA	NA	NA	NA	NA	NA	NA	MODIFIER	NA	NA	204	204	0	146	140	6	4.11
Chewer	IOB_04	chr19	58773664	C	A	ZNF544	SILENT	ENSG00000198131:[ENST00000269829(c.1692C>A,gtC>gtA):ENSP00000269829(p.Val564(=))]	NA	NA	NA	NA	NA	NA	NA	NA	NA	NA	MODIFIER	NA	NA	204	204	0	146	140	6	4.11
Chewer	IOB_04	chr2	1652534	G	A	PXDN	SILENT	ENSG00000130508:[ENST00000252804(c.3018C>T,aaC>aaT):ENSP00000252804(p.Asn1006(=))]	NA	NA	NA	NA	NA	NA	NA	NA	NA	NA	MODIFIER	NA	NA	338	337	0	241	232	8	3.32
Chewer	IOB_04	chr2	10952761	C	T	PDIA6	INTRONIC	ENSG00000143870:[ENST00000540494(c.-169+43G>A)]	NA	NA	NA	NA	NA	NA	NA	NA	NA	NA	MODIFIER	NA	NA	199	198	1	179	173	5	2.79
Chewer	IOB_04	chr2	20135333	G	A	WDR35	MISSENSE	ENSG00000118965:[ENST00000453014(c.1084C>T,Cgg>Tgg):ENSP00000404409(p.Arg362Trp)]	NA	chr2*20135333*20135333*COSM3695183*G*A*1*large_intestine*1	NA	2.49E-05	0.00E+00	NA	NA	D	PrD	NA	MODERATE	D	0.998	242	241	0	166	161	5	3.01
Chewer	IOB_04	chr2	32702460	C	T	BIRC6	MISSENSE	ENSG00000115760:[ENST00000421745(c.6877C>T,Cgt>Tgt):ENSP00000393596(p.Arg2293Cys)]	NA	NA	NA	NA	NA	NA	NA	NA	NA	NA	MODIFIER	NA	NA	262	260	0	164	157	5	3.05
Chewer	IOB_04	chr2	38525643	C	T	ATL2	SILENT	ENSG00000119787:[ENST00000402054(c.762G>A,gcG>gcA):ENSP00000384062(p.Ala254(=))]	NA	NA	chr2*38525643*38525643*C*G,T*rs984426120*NA*NA*NA	NA	NA	NA	NA	NA	NA	NA	LOW	NA	NA	804	800	0	489	480	9	1.84
Chewer	IOB_04	chr2	73635885	C	A	ALMS1	INTRONIC	ENSG00000116127:[ENST00000409009(c.325-10366C>A)]	NA	NA	NA	NA	NA	NA	NA	NA	NA	NA	MODIFIER	NA	NA	261	255	0	207	199	6	2.9
Chewer	IOB_04	chr2	179494072	C	T	TTN	MISSENSE	ENSG00000155657:[ENST00000589042(c.44380G>A,Gtg>Atg):ENSP00000467141(p.Val14794Met)]	NA	NA	NA	NA	NA	NA	NA	NA	NA	NA	MODIFIER	NA	NA	127	127	0	97	92	5	5.15
Chewer	IOB_04	chr2	179494072	C	T	TTN-AS1	NCT	ENSG00000237298:[ENST00000592630(n.519+31664C>T)]	NA	NA	NA	NA	NA	NA	NA	NA	NA	NA	MODIFIER	NA	NA	127	127	0	97	92	5	5.15
Chewer	IOB_04	chr2	179650444	G	A	TTN	MISSENSE	ENSG00000155657:[ENST00000342992(c.2396C>T,aCg>aTg):ENSP00000343764(p.Thr799Met)]	NA	chr2*179650444*179650444*COSM719204*G*A*1*lung*1|chr2*179650444*179650444*COSM20389*G*A*2*large_intestine,lung*1,1|chr2*179650444*179650444*COSM719205*G*A*1*lung*1|chr2*179650444*179650444*COSM719207*G*A*1*lung*1|chr2*179650444*179650444*COSM1148839*G*A*1*lung*1|chr2*179650444*179650444*COSM719206*G*A*1*lung*1	7.99E-04	4.94E-05	0.00E+00	7.99E-04	0.00E+00	NA	BN	NA	MODERATE	NA	NA	183	181	1	110	105	5	4.55
Chewer	IOB_04	chr2	179669253	A	C	TTN	INTRONIC	ENSG00000155657:[ENST00000342175(c.91+26T>G)]	NA	NA	chr2*179669249*179669254*AAAAAC*A*rs763294010*NA*NA*NA	NA	NA	NA	NA	NA	NA	NA	MODIFIER	NA	NA	72	71	0	74	68	6	8.11
Chewer	IOB_04	chr2	223158483	G	A	PAX3	INTRONIC-SS-ACR-PRX	ENSG00000135903:[ENST00000258387(c.587-7C>T)]	NA	NA	NA	NA	NA	NA	NA	NA	NA	NA	MODIFIER	NA	NA	97	95	0	117	110	5	4.27
Chewer	IOB_04	chr2	227872812	C	T	COL4A4	SILENT	ENSG00000081052:[ENST00000329662(c.4722G>A,gcG>gcA):ENSP00000328553(p.Ala1574(=))]	NA	NA	5.99E-04	2.12E-03	0.00E+00	5.99E-04	0.00E+00	NA	NA	NA	LOW	NA	NA	185	185	0	169	157	12	7.1
Chewer	IOB_04	chr2	228416636	C	A	AGFG1	INTRONIC	ENSG00000173744:[ENST00000409979(c.1451-39C>A)]	NA	NA	NA	NA	NA	NA	NA	NA	NA	NA	MODIFIER	NA	NA	349	346	0	242	234	5	2.07
Chewer	IOB_04	chr20	35443769	G	A	SOGA1	SILENT	ENSG00000149639:[ENST00000237536(c.2076C>T,ttC>ttT):ENSP00000237536(p.Phe692(=))]	NA	NA	NA	NA	NA	NA	NA	NA	NA	NA	LOW	NA	0.029	826	818	0	496	479	13	2.62
Chewer	IOB_04	chr20	55033690	G	T	CASS4	MISSENSE	ENSG00000087589:[ENST00000434344(c.937G>T,Gcc>Tcc):ENSP00000410027(p.Ala313Ser)]	NA	NA	NA	NA	NA	NA	NA	D	PrD	NA	MODERATE	D	1	243	241	0	195	188	5	2.56
Chewer	IOB_04	chr21	9825820	C	T	NA	INTERGENIC	NA	NA	NA	NA	NA	NA	NA	NA	NA	NA	NA	MODIFIER	NA	NA	644	638	2	410	395	7	1.71
Chewer	IOB_04	chr21	40608721	G	T	BRWD1	INTRONIC	ENSG00000185658:[ENST00000445245(c.*910-11C>A)]	NA	NA	chr21*40608721*40608721*G*A*rs768110545*NA*NA*NA	NA	NA	NA	NA	NA	NA	NA	MODIFIER	NA	NA	199	198	0	184	178	5	2.72
Chewer	IOB_04	chr22	24621306	G	A	GGT5	SILENT	ENSG00000099998:[ENST00000327365(c.1410C>T,tcC>tcT):ENSP00000330080(p.Ser470(=))]	NA	NA	NA	NA	NA	NA	NA	NA	NA	NA	LOW	NA	NA	611	608	0	358	349	8	2.23
Chewer	IOB_04	chr22	24621307	G	A	GGT5	MISSENSE	ENSG00000099998:[ENST00000327365(c.1409C>T,tCc>tTc):ENSP00000330080(p.Ser470Phe)]	NA	NA	NA	NA	NA	NA	NA	D	PoD	NA	MODERATE	D	1	608	605	0	357	348	8	2.24
Chewer	IOB_04	chr22	28389421	C	T	TTC28	MISSENSE	ENSG00000100154:[ENST00000431039(c.191G>A,gGc>gAc):ENSP00000410244(p.Gly64Asp)]	NA	NA	NA	NA	NA	NA	NA	NA	NA	NA	MODIFIER	NA	NA	278	278	0	214	206	6	2.8

Patel *et al.* , 2021. Whole-exome sequencing analysis of oral squamous cell carcinoma delineated by tobacco usage habits
Supplementary Table 4. List of somatic single nucleotide variants identified using whole exome sequencing of OSCC patients

Cohort	Sample	Chromosome	Genomic position	Reference allele	Alternate allele	Genesymbol	Variant type	Variant description	OncoMD	COSMIC	dbSNP	ExAC	ExAC_SAS	1000G	1000G_SAS	SIFT	Polyphen2	CONDEL	VEP	LRT	PhastCon7Way	Total reads in Normal	Reference read support in Normal	Alternative read support in Normal	Total reads in Tumor	Reference read support in Tumor	Alternate read support in Tumor	Alternate allele frequency in Tumor
Chewer	IOB_04	chr22	28389421	C	T	TTC28-AS1	NCT	ENSG00000235954:[ENST00000452612(n.136-3978C>T)]	NA	NA	NA	NA	NA	NA	NA	NA	NA	NA	MODIFIER	NA	NA	278	278	0	214	206	6	2.8
Chewer	IOB_04	chr22	32004030	C	A	SFI1	NCT	ENSG00000198089:[ENST00000466991(n.380+11C>A)]	NA	NA	chr22*32004030*32004030*C*T*rs770837805*NA*NA*NA	NA	NA	NA	NA	NA	NA	NA	MODIFIER	NA	NA	168	167	1	157	150	5	3.18
Chewer	IOB_04	chr22	36122860	G	A	APOL5	MISSENSE	ENSG00000128313:[ENST00000249044(c.745G>A,Gcc>Acc):ENSP00000249044(p.Ala249Thr)]	NA	NA	NA	NA	NA	NA	NA	T	PrD	NA	MODERATE	U	0.012	309	306	1	240	233	5	2.08
Chewer	IOB_04	chr22	46652819	G	A	PKDREJ	MISSENSE	ENSG00000130943:[ENST00000253255(c.6401C>T,tCa>tTa):ENSP00000253255(p.Ser2134Leu)]	NA	NA	NA	NA	NA	NA	NA	D	PoD	NA	MODERATE	N	1	308	306	0	173	166	6	3.47
Chewer	IOB_04	chr22	50965036	C	T	TYMP	SILENT	ENSG00000025708:[ENST00000252029(c.897G>A,ccG>ccA):ENSP00000252029(p.Pro299(=))]	NA	NA	NA	NA	NA	NA	NA	NA	NA	NA	MODIFIER	NA	NA	237	237	0	113	108	5	4.42
Chewer	IOB_04	chr3	49698113	C	A	BSN	MISSENSE	ENSG00000164061:[ENST00000296452(c.8835C>A,gaC>gaA):ENSP00000296452(p.Asp2945Glu)]	NA	NA	NA	NA	NA	NA	NA	D	PoD	NA	MODERATE	D	1	286	285	0	200	193	5	2.5
Chewer	IOB_04	chr3	97806176	G	A	OR5AC2	MISSENSE	ENSG00000196578:[ENST00000358642(c.160G>A,Gac>Aac):ENSP00000351466(p.Asp54Asn)]	NA	NA	NA	8.24E-06	0.00E+00	NA	NA	D	BN	NA	MODERATE	N	0.034	303	302	0	211	205	5	2.37
Chewer	IOB_04	chr3	183433026	C	T	YEATS2	MISSENSE	ENSG00000163872:[ENST00000305135(c.76C>T,Cgg>Tgg):ENSP00000306983(p.Arg26Trp)]	NA	NA	NA	1.66E-05	6.06E-05	NA	NA	D	PoD	NA	MODERATE	D	1	346	345	0	235	228	6	2.55
Chewer	IOB_04	chr4	62761502	C	A	LPHN3	MISSENSE	ENSG00000150471:[ENST00000502815(c.44C>A,Cca>Aca):ENSP00000424402(p.Pro16Thr)]	NA	NA	NA	NA	NA	NA	NA	D	PoD	NA	MODERATE	D	1	371	369	0	287	274	13	4.53
Chewer	IOB_04	chr4	95377067	C	A	PDLIM5	NCT	ENSG00000163110:[ENST00000509333(n.206+532C>A)]	NA	NA	NA	NA	NA	NA	NA	NA	NA	NA	MODIFIER	NA	NA	215	215	0	127	116	8	6.3
Chewer	IOB_04	chr4	108935771	T	C	HADH	INTRONIC	ENSG00000138796:[ENST00000309522(c.419+27T>C)]	NA	NA	6.39E-03	1.49E-01	2.52E-01	6.39E-03	1.00E-03	NA	NA	NA	MODIFIER	NA	NA	120	117	2	78	68	10	12.82
Chewer	IOB_04	chr4	155312422	C	A	DCHS2	NONSENSE	ENSG00000197410:[ENST00000357232(c.28G>T,Gag>Tag):ENSP00000349768(p.Glu10Ter)]	NA	NA	NA	NA	NA	NA	NA	NA	NA	NA	HIGH	NA	0.001	240	237	1	134	127	5	3.73
Chewer	IOB_04	chr4	160251604	G	A	RAPGEF2	MISSENSE	ENSG00000109756:[ENST00000264431(c.938G>A,gGc>gAc):ENSP00000264431(p.Gly313Asp)]	NA	NA	NA	8.28E-06	0.00E+00	NA	NA	NA	NA	NA	MODIFIER	NA	NA	271	271	0	177	172	5	2.82
Chewer	IOB_04	chr4	175897692	G	T	ADAM29	MISSENSE	ENSG00000168594:[ENST00000359240(c.1016G>T,gGc>gTc):ENSP00000352177(p.Gly339Val)]	NA	NA	NA	NA	NA	NA	NA	D	PrD	NA	MODERATE	U	1	463	461	0	250	233	17	6.8
Chewer	IOB_04	chr5	52084343	G	A	ITGA1	INTRONIC	ENSG00000213949:[ENST00000282588(c.61+95G>A)]	NA	NA	NA	NA	NA	NA	NA	NA	NA	NA	MODIFIER	NA	NA	214	212	0	103	95	8	7.77
Chewer	IOB_04	chr5	52084343	G	A	PELO	INTRONIC	ENSG00000152684:[ENST00000274311(c.-511+95G>A)]	NA	NA	NA	NA	NA	NA	NA	NA	NA	NA	MODIFIER	NA	NA	214	212	0	103	95	8	7.77
Chewer	IOB_04	chr5	140187422	G	T	PCDHA1	INTRONIC	ENSG00000204970:[ENST00000504120(c.2394+19153G>T)]	NA	NA	NA	1.65E-05	0.00E+00	NA	NA	D_lc	PrD	NA	MODERATE	U	1	259	257	0	212	202	9	4.25
Chewer	IOB_04	chr5	140187422	G	T	PCDHA2	INTRONIC	ENSG00000204969:[ENST00000526136(c.2388+10485G>T)]	NA	NA	NA	1.65E-05	0.00E+00	NA	NA	D_lc	PrD	NA	MODERATE	U	1	259	257	0	212	202	9	4.25
Chewer	IOB_04	chr5	140187422	G	T	PCDHA3	INTRONIC	ENSG00000255408:[ENST00000522353(c.2394+4246G>T)]	NA	NA	NA	1.65E-05	0.00E+00	NA	NA	D_lc	PrD	NA	MODERATE	U	1	259	257	0	212	202	9	4.25
Chewer	IOB_04	chr5	140187422	G	T	PCDHA4	MISSENSE	ENSG00000204967:[ENST00000356878(c.650G>T,gGa>gTa):ENSP00000349344(p.Gly217Val)]	NA	NA	NA	1.65E-05	0.00E+00	NA	NA	D_lc	PrD	NA	MODERATE	U	1	259	257	0	212	202	9	4.25
Chewer	IOB_04	chr6	7585115	C	A	DSP	MISSENSE	ENSG00000096696:[ENST00000418664(c.5823C>A,gaC>gaA):ENSP00000396591(p.Asp1941Glu)]	NA	NA	NA	NA	NA	NA	NA	NA	NA	NA	MODERATE	NA	0.985	299	295	2	204	197	7	3.43
Chewer	IOB_04	chr6	22147382	C	T	CASC14	EXONIC-NC	ENSG00000260455:[ENST00000566912(n.41G>A)]	NA	NA	NA	NA	NA	NA	NA	NA	NA	NA	MODIFIER	NA	NA	143	143	0	124	119	5	4.03
Chewer	IOB_04	chr6	22147382	C	T	CASC15	EXONIC-NC	ENSG00000272168:[ENST00000606197(n.500C>T)]	NA	NA	NA	NA	NA	NA	NA	NA	NA	NA	MODIFIER	NA	NA	143	143	0	124	119	5	4.03
Chewer	IOB_04	chr6	31604645	C	T	PRRC2A	MISSENSE	ENSG00000204469:[ENST00000376007(c.6070C>T,Cgg>Tgg):ENSP00000365175(p.Arg2024Trp)]	NA	chr6*31604645*31604645*COSM595965*C*T*1*lung*1	chr6*31604645*31604645*C*A,T*rs373808174*NA*NA*NA	8.64E-06	0.00E+00	NA	NA	NA	NA	NA	MODIFIER	NA	NA	226	225	1	143	138	5	3.5
Chewer	IOB_04	chr6	35587984	C	A	FKBP5	SILENT	ENSG00000096060:[ENST00000357266(c.318G>T,ctG>ctT):ENSP00000349811(p.Leu106(=))]	NA	NA	NA	NA	NA	NA	NA	NA	NA	NA	MODIFIER	NA	NA	356	356	0	222	215	6	2.7
Chewer	IOB_04	chr6	46107621	C	A	ENPP4	MISSENSE	ENSG00000001561:[ENST00000321037(c.301C>A,Cac>Aac):ENSP00000318066(p.His101Asn)]	NA	NA	NA	NA	NA	NA	NA	T	BN	NA	MODERATE	N	0.002	380	380	0	243	237	6	2.47
Chewer	IOB_04	chr6	47678522	C	A	GPR115	MISSENSE	ENSG00000153294:[ENST00000283303(c.200C>A,gCt>gAt):ENSP00000283303(p.Ala67Asp)]	NA	NA	NA	NA	NA	NA	NA	T	BN	NA	MODERATE	N	0.484	371	370	0	298	289	5	1.68
Chewer	IOB_04	chr6	49411582	C	A	MUT	INTRONIC	ENSG00000146085:[ENST00000274813(c.1676+770G>T)]	NA	NA	NA	NA	NA	NA	NA	NA	NA	NA	MODIFIER	NA	NA	73	73	0	61	56	5	8.2
Chewer	IOB_04	chr6	117639472	G	A	GOPC	NCT	ENSG00000047932:[ENST00000467125(n.889-57C>T)]	NA	NA	NA	NA	NA	NA	NA	NA	NA	NA	MODIFIER	NA	NA	198	198	0	149	144	5	3.36
Chewer	IOB_04	chr6	117639472	G	A	ROS1	INTRONIC	ENSG00000047936:[ENST00000368508(c.5941-57C>T)]	NA	NA	NA	NA	NA	NA	NA	NA	NA	NA	MODIFIER	NA	NA	198	198	0	149	144	5	3.36
Chewer	IOB_04	chr6	137814928	A	T	OLIG3	MISSENSE	ENSG00000177468:[ENST00000367734(c.380T>A,cTc>cAc):ENSP00000356708(p.Leu127His)]	NA	NA	NA	NA	NA	NA	NA	D	PrD	NA	MODERATE	D	1	507	504	0	414	402	11	2.66
Chewer	IOB_04	chr6	146276215	G	C	SHPRH	MISSENSE	ENSG00000146414:[ENST00000275233(c.244C>G,Cca>Gca):ENSP00000275233(p.Pro82Ala)]	NA	NA	NA	NA	NA	NA	NA	T	BN	NA	MODERATE	NA	NA	124	123	0	86	80	6	6.98
Chewer	IOB_04	chr6	150016278	T	C	LATS1	MISSENSE	ENSG00000131023:[ENST00000458696(c.266A>G,tAc>tGc):ENSP00000441265(p.Tyr89Cys)]	NA	NA	NA	NA	NA	NA	NA	D_lc	PoD	NA	MODERATE	D	1	167	167	0	130	125	5	3.85
Chewer	IOB_04	chr7	1733430	C	T	AC074389.6	SILENT	ENSG00000205971:[ENST00000382528(c.102G>A,ccG>ccA):ENSP00000371968(p.Pro34(=))]	NA	NA	NA	NA	NA	NA	NA	NA	NA	NA	MODIFIER	NA	NA	280	280	0	235	229	5	2.13
Chewer	IOB_04	chr7	1733430	C	T	ELFN1	INTRONIC	ENSG00000225968:[ENST00000424383(c.-456+5644C>T)]	NA	NA	NA	NA	NA	NA	NA	NA	NA	NA	MODIFIER	NA	NA	280	280	0	235	229	5	2.13
Chewer	IOB_04	chr7	1733430	C	T	NA	EXONIC-NC	401296:[XR_108728.1(n.439G>A)]	NA	NA	NA	NA	NA	NA	NA	NA	NA	NA	MODIFIER	NA	NA	280	280	0	235	229	5	2.13
Chewer	IOB_04	chr7	89854878	C	T	STEAP2	MISSENSE	ENSG00000157214:[ENST00000287908(c.482C>T,gCc>gTc):ENSP00000287908(p.Ala161Val)]	NA	chr7*89854876*89854879*COSM453528*TGCC*T*1*breast*1|chr7*89854876*89854879*COSM1488755*TGCC*T*1*breast*1	NA	NA	NA	NA	NA	NA	NA	NA	MODIFIER	NA	NA	253	251	0	161	156	5	3.11
Chewer	IOB_04	chr7	137230741	G	C	DGKI	INTRONIC	ENSG00000157680:[ENST00000424189(c.2148-33C>G)]	NA	NA	NA	NA	NA	NA	NA	NA	NA	NA	MODIFIER	NA	NA	104	103	0	115	108	7	6.09
Chewer	IOB_04	chr8	39606869	C	T	ADAM2	MISSENSE	ENSG00000104755:[ENST00000347580(c.1919G>A,gGc>gAc):ENSP00000343854(p.Gly640Asp)]	NA	chr8*39606869*39606869*COSM3432425*C*T*1*large intestine*1	NA	NA	NA	NA	NA	NA	NA	NA	MODERATE	NA	NA	164	161	1	109	102	6	5.5
Chewer	IOB_04	chr8	55647350	C	T	RP1	INTRONIC	6101:[XM_005251278.1(c.2151+46C>T)]	NA	NA	chr8*55647350*55647350*C*A*rs950160995*NA*NA*NA	NA	NA	NA	NA	NA	NA	NA	MODIFIER	NA	NA	133	133	0	116	110	6	5.17
Chewer	IOB_04	chr8	55647350	C	T	RP11-56A10.1	NCT	ENSG00000254608:[ENST00000521465(n.571-4153C>T)]	NA	NA	chr8*55647350*55647350*C*A*rs950160995*NA*NA*NA	NA	NA	NA	NA	NA	NA	NA	MODIFIER	NA	NA	133	133	0	116	110	6	5.17
Chewer	IOB_04	chr8	56077008	C	T	RP11-386G21.2	3UTR	ENSG00000253857:[ENST00000522559(c.*155C>T)]	NA	NA	NA	NA	NA	NA	NA	NA	NA	NA	MODIFIER	NA	NA	175	174	0	171	165	6	3.51
Chewer	IOB_04	chr8	56077008	C	T	XKR4	INTRONIC	ENSG00000206579:[ENST00000327381(c.806+61154C>T)]	NA	NA	NA	NA	NA	NA	NA	NA	NA	NA	MODIFIER	NA	NA	175	174	0	171	165	6	3.51
Chewer	IOB_04	chr8	57307714	C	A	SDR16C6P	EXONIC-NC	ENSG00000253542:[ENST00000524136(n.211G>T)]	NA	NA	NA	NA	NA	NA	NA	NA	NA	NA	MODIFIER	NA	NA	431	430	0	348	331	16	4.6
Chewer	IOB_04	chr8	74171632	C	A	C8orf89	MISSENSE	100130301:[NM_001243237.1(c.62G>T,gGc>gTc):NP_001230166.1(p.Gly21Val)]	NA	NA	chr8*74171632*74171632*C*T*rs183049268*0*0.9998*0.0001997	NA	NA	NA	NA	T	PoD	NA	MODERATE	NA	NA	102	102	0	72	67	5	6.94
Chewer	IOB_04	chr9	12775915	C	A	LURAP1L	SILENT	ENSG00000153714:[ENST00000319264(c.201C>A,ccC>ccA):ENSP00000321026(p.Pro67(=))]	NA	NA	NA	NA	NA	NA	NA	NA	NA	NA	MODIFIER	NA	NA	128	127	0	130	125	5	3.85
Chewer	IOB_04	chr9	12775915	C	A	LURAP1L-AS1	NCT	101929467:[NR_125775.1(n.243+14704G>T)]	NA	NA	NA	NA	NA	NA	NA	NA	NA	NA	MODIFIER	NA	NA	128	127	0	130	125	5	3.85
Chewer	IOB_04	chr9	12775915	C	A	RP11-3L8.3	NCT	ENSG00000235448:[ENST00000417638(n.199+14704G>T)]	NA	NA	NA	NA	NA	NA	NA	NA	NA	NA	MODIFIER	NA	NA	128	127	0	130	125	5	3.85
Chewer	IOB_04	chr9	21077369	A	T	IFNB1	MISSENSE	ENSG00000171855:[ENST00000380232(c.500T>A,gTc>gAc):ENSP00000369581(p.Val167Asp)]	NA	NA	NA	NA	NA	NA	NA	D	PrD	NA	MODERATE	N	0.039	239	239	0	179	174	5	2.79
Chewer	IOB_04	chr9	77377717	C	T	TRPM6	SILENT	ENSG00000119121:[ENST00000361255(c.3855G>A,cgG>cgA):ENSP00000354962(p.Arg1285(=))]	NA	NA	NA	NA	NA	NA	NA	NA	NA	NA	LOW	NA	NA	278	276	0	180	174	5	2.78
Chewer	IOB_04	chr9	111848258	C	A	TMEM245	INTRONIC	ENSG00000106771:[ENST00000374586(c.1344+19G>T)]	NA	NA	NA	NA	NA	NA	NA	NA	NA	NA	MODIFIER	NA	NA	109	109	0	108	103	5	4.63
Chewer	IOB_04	chr9	139438549	G	A	NOTCH1	NONSENSE	ENSG00000148400:[ENST00000277541(c.67C>T,Cga>Tga):ENSP00000277541(p.Arg23Ter)]	NA	NA	NA	NA	NA	NA	NA	NA	NA	NA	HIGH	NA	0.999	47	46	0	39	34	5	12.82
Chewer	IOB_04	chrX	2867445	C	A	ARSE	MISSENSE	ENSG00000157399:[ENST00000381134(c.754G>T,Gat>Tat):ENSP00000370526(p.Asp252Tyr)]	NA	NA	chrX*2867445*2867445*C*T*rs753447542*NA*NA*NA	NA	NA	NA	NA	NA	NA	NA	MODERATE	NA	0.846	169	167	0	124	118	5	4.03
Chewer	IOB_04	chrX	69282358	G	T	OTUD6A	5UTR	ENSG00000189401:[ENST00000338352(c.-17G>T)]	NA	NA	NA	NA	NA	NA	NA	NA	NA	NA	MODIFIER	NA	NA	89	89	0	65	60	5	7.69
Chewer	IOB_04	chrX	83723759	C	T	HDX	SILENT	ENSG00000165259:[ENST00000297977(c.972G>A,tcG>tcA):ENSP00000297977(p.Ser324(=))]	NA	NA	NA	NA	NA	NA	NA	NA	NA	NA	LOW	NA	NA	201	200	0	134	122	12	8.96
Chewer	IOB_04	chrX	106367537	C	A	NUP62CL	SILENT	ENSG00000198088:[ENST00000432145(c.529G>T,gtG>gtT):ENSP00000408612(p.Val177(=))]	NA	NA	NA	NA	NA	NA	NA	NA	NA	NA	MODIFIER	NA	NA	249	247	0	160	154	6	3.75
Chewer	IOB_06	chr1	1223010	G	A	SCNN1D	MISSENSE	ENSG00000162572:[ENST00000379099(c.314G>A,Ggg>Agg):ENSP00000368393(p.Gly106Arg)]	NA	NA	NA	NA	NA	NA	NA	NA	NA	NA	MODIFIER	NA	NA	149	148	0	167	140	27	16.17
Chewer	IOB_06	chr1	1861606	C	T	C1orf222	MISSENSE	ENSG00000142609:[ENST00000493964(c.1030G>A,Ggc>Agc):ENSP00000417061(p.Gly344Ser)]	NA	NA	NA	NA	NA	NA	NA	NA	NA	NA	MODERATE	NA	NA	146	145	1	232	207	24	10.34
Chewer	IOB_06	chr1	1861606	C	T	CFAP74	MISSENSE	85452:[NM_001304360.1(c.3181G>A,Ggc>Agc):NP_001291289.1(p.Gly1061Ser)]	NA	NA	NA	NA	NA	NA	NA	NA	NA	NA	MODERATE	NA	NA	146	145	1	232	207	24	10.34
Chewer	IOB_06	chr1	23191445	G	A	EPHB2	MISSENSE	ENSG00000133216:[ENST00000374630(c.1043G>A,cGc>cAc):ENSP00000363761(p.Arg348His)]	NA	chr1*23191445*23191445*COSM357691*G*A*2*pancreas,lung*1,1|chr1*23191445*23191445*COSM3385892*G*A*1*pancreas*1|chr1*23191445*23191445*COSM3385893*G*A*1*pancreas*1	2.00E-04	8.82E-06	7.69E-05	2.00E-04	1.00E-03	T	PrD	NA	MODERATE	D	0.999	155	155	0	225	198	20	8.89
Chewer	IOB_06	chr1	24172243	C	G	FUCA1	MISSENSE	ENSG00000179163:[ENST00000374479(c.1363G>C,Gag>Cag):ENSP00000363603(p.Glu455Gln)]	NA	NA	NA	NA	NA	NA	NA	NA	NA	NA	MODERATE	NA	0	107	106	0	112	102	10	8.93
Chewer	IOB_06	chr1	26800219	C	G	HMGN2	MISSENSE	ENSG00000198830:[ENST00000361427(c.86C>G,tCt>tGt):ENSP00000355228(p.Ser29Cys)]	NA	NA	chr1*26800219*26800219*C*A*rs760851265*NA*NA*NA	NA	NA	NA	NA	NA	NA	NA	MODIFIER	NA	NA	180	179	0	202	179	23	11.39
Chewer	IOB_06	chr1	32498845	G	A	KHDRBS1	SILENT	ENSG00000121774:[ENST00000492989(c.564G>A,ctG>ctA):ENSP00000417731(p.Leu188(=))]	NA	NA	NA	NA	NA	NA	NA	NA	NA	NA	MODIFIER	NA	NA	165	164	0	176	170	6	3.41
Chewer	IOB_06	chr1	33235563	C	T	KIAA1522	MISSENSE	57648:[XM_005271073.1(c.74C>T,gCc>gTc):XP_005271130.1(p.Ala25Val)]	NA	NA	NA	NA	NA	NA	NA	NA	NA	NA	LOW	NA	NA	172	172	0	192	185	6	3.12
Chewer	IOB_06	chr1	53732197	C	A	LRP8	MISSENSE	ENSG00000157193:[ENST00000465675(c.34G>T,Gtt>Ttt):ENSP00000437009(p.Val12Phe)]	NA	NA	chr1*53732197*53732197*C*T*rs578053398*0*0.9998*0.0001997	NA	NA	NA	NA	T	PrD	NA	MODERATE	NA	NA	101	101	0	144	137	6	4.17
Chewer	IOB_06	chr1	84372043	G	A	TTLL7	MISSENSE	ENSG00000137941:[ENST00000474957(c.1151C>T,tCa>tTa):ENSP00000434146(p.Ser384Leu)]	NA	NA	NA	NA	NA	NA	NA	NA	NA	NA	MODIFIER	NA	NA	118	118	0	153	141	12	7.84
Chewer	IOB_06	chr1	101705298	C	T	S1PR1	MISSENSE	ENSG00000170989:[ENST00000305352(c.758C>T,gCg>gTg):ENSP00000305416(p.Ala253Val)]	NA	chr1*101705298*101705298*COSM1497571*C*T*1*lung*1	NA	NA	NA	NA	NA	D	PrD	NA	MODERATE	D	1	111	110	0	140	125	15	10.71
Chewer	IOB_06	chr1	109883405	G	A	SORT1	MISSENSE	ENSG00000134243:[ENST00000256637(c.1205C>T,aCg>aTg):ENSP00000256637(p.Thr402Met)]	NA	NA	NA	1.65E-05	0.00E+00	NA	NA	NA	NA	NA	MODERATE	NA	1	87	87	0	127	101	26	20.47

Patel *et al.* , 2021. Whole-exome sequencing analysis of oral squamous cell carcinoma delineated by tobacco usage habits
Supplementary Table 4. List of somatic single nucleotide variants identified using whole exome sequencing of OSCC patients

Cohort	Sample	Chromosome	Genomic position	Reference allele	Alternate allele	Genesymbol	Variant type	Variant description	OncoMD	COSMIC	dbSNP	ExAC	ExAC_SAS	1000G	1000G_SAS	SIFT	Polyphen2	CONDEL	VEP	LRT	PhastCon7Way	Total reads in Normal	Reference read support in Normal	Alternative read support in Normal	Total reads in Tumor	Reference read support in Tumor	Alternate read support in Tumor	Alternate allele frequency in Tumor
Chewer	IOB_06	chr1	110170174	C	A	AMPD2	INTRONIC	ENSG00000116337:[ENST00000369840(c.1023+34C>A)]	NA	NA	NA	NA	NA	NA	NA	NA	NA	NA	MODIFIER	NA	NA	105	105	0	231	225	6	2.6
Chewer	IOB_06	chr1	152281100	C	A	FLG	MISSENSE	ENSG00000143631:[ENST00000368799(c.6262G>T,Ggg>Tgg):ENSP00000357789(p.Gly2088Trp)]	NA	NA	NA	NA	NA	NA	NA	D	PrD	NA	MODERATE	NA	NA	120	120	0	220	214	6	2.73
Chewer	IOB_06	chr1	152281100	C	A	FLG-AS1	NCT	ENSG00000237975:[ENST00000593011(n.377-6282C>A)]	NA	NA	NA	NA	NA	NA	NA	D	PrD	NA	MODERATE	NA	NA	120	120	0	220	214	6	2.73
Chewer	IOB_06	chr1	155262969	G	A	PKLR	MISSENSE-SS-PRX	ENSG00000143627:[ENST00000392414(c.1342C>T,Cgc>Tgc):ENSP00000376214(p.Arg448Cys)]	NA	NA	3.99E-04	2.47E-05	6.06E-05	3.99E-04	0.00E+00	D	PrD	NA	MODERATE	D	1	115	111	0	136	127	5	3.68
Chewer	IOB_06	chr1	155451610	C	A	ASH1L	MISSENSE	ENSG00000116539:[ENST00000368346(c.1051G>T,Gtg>Ttg):ENSP00000357330(p.Val351Leu)]	NA	NA	NA	NA	NA	NA	NA	T_lc	BN	NA	MODERATE	N	0.609	271	270	1	353	309	44	12.46
Chewer	IOB_06	chr1	158368390	G	C	OR10T2	SILENT	ENSG00000186306:[ENST00000334438(c.867C>G,gtC>gtG):ENSP00000334115(p.Val289(=))]	NA	NA	NA	NA	NA	NA	NA	NA	NA	NA	LOW	NA	NA	104	104	0	84	62	22	26.19
Chewer	IOB_06	chr1	161993071	G	T	OLFML2B	MISSENSE	ENSG00000162745:[ENST00000294794(c.150C>A,gaC>gaA):ENSP00000294794(p.Asp50Glu)]	NA	NA	chr1*161993071*161993071*G*A*rs777349963*NA*NA*NA	NA	NA	NA	NA	T	BN	NA	MODERATE	NA	NA	119	119	0	218	182	35	16.06
Chewer	IOB_06	chr1	163142395	G	A	LOC101928404	NCT	101928404:[XR_241139.1(n.261+9729G>A)]	NA	NA	NA	NA	NA	NA	NA	NA	NA	NA	MODIFIER	NA	NA	78	78	0	72	65	7	9.72
Chewer	IOB_06	chr1	163142395	G	A	RGS5	NCT	ENSG00000143248:[ENST00000428971(n.447-4237C>T)]	NA	NA	NA	NA	NA	NA	NA	NA	NA	NA	MODIFIER	NA	NA	78	78	0	72	65	7	9.72
Chewer	IOB_06	chr1	163142395	G	A	RP11-267N12.1	NCT	ENSG00000232892:[ENST00000415437(n.258+9729G>A)]	NA	NA	NA	NA	NA	NA	NA	NA	NA	NA	MODIFIER	NA	NA	78	78	0	72	65	7	9.72
Chewer	IOB_06	chr1	196285017	C	T	KCNT2	NCT-SS-PRX	ENSG00000162687:[ENST00000610076(n.2608+5G>A)]	NA	NA	NA	NA	NA	NA	NA	NA	NA	NA	LOW	NA	NA	145	144	0	184	177	5	2.72
Chewer	IOB_06	chr1	234742995	C	A	IRF2BP2	MISSENSE	ENSG00000168264:[ENST00000366609(c.1652G>T,aGt>aTt):ENSP00000355568(p.Ser551Ile)]	NA	NA	NA	NA	NA	NA	NA	NA	NA	NA	MODIFIER	NA	NA	125	125	0	206	200	6	2.91
Chewer	IOB_06	chr1	234742995	C	A	RP4-781K5.2	EXONIC-NC	ENSG00000228830:[ENST00000436039(n.242C>A)]	NA	NA	NA	NA	NA	NA	NA	NA	NA	NA	MODIFIER	NA	NA	125	125	0	206	200	6	2.91
Chewer	IOB_06	chr1	237777706	C	T	RYR2	MISSENSE	ENSG00000198626:[ENST00000366574(c.5278C>T,Cgg>Tgg):ENSP00000355533(p.Arg1760Trp)]	NA	NA	chr1*237777706*237777706*C*G,T*rs776550479*NA*NA*NA	1.66E-05	0.00E+00	NA	NA	NA	PrD	NA	MODERATE	D	0.209	155	155	0	165	150	15	9.09
Chewer	IOB_06	chr1	244641217	G	T	C1orf101	SILENT	ENSG00000179397:[ENST00000428042(c.168G>T,acG>acT):ENSP00000395796(p.Thr56(=))]	NA	NA	NA	NA	NA	NA	NA	NA	NA	NA	MODIFIER	NA	NA	115	115	0	91	81	10	10.99
Chewer	IOB_06	chr10	1263026	C	A	ADARB2	MISSENSE	ENSG00000185736:[ENST00000381312(c.1547G>T,cGc>cTc):ENSP00000370713(p.Arg516Leu)]	NA	NA	chr10*1263026*1263026*C*T*rs747329974*NA*NA*NA	NA	NA	NA	NA	NA	NA	NA	MODIFIER	NA	NA	121	121	0	177	171	5	2.82
Chewer	IOB_06	chr10	24810788	G	A	KIAA1217	MISSENSE	ENSG00000120549:[ENST00000376462(c.2146G>A,Gag>Aag):ENSP00000365645(p.Glu716Lys)]	NA	NA	NA	NA	NA	NA	NA	NA	NA	NA	MODERATE	NA	1	86	86	0	113	101	11	9.73
Chewer	IOB_06	chr10	45429166	G	T	TMEM72	SILENT	ENSG00000187783:[ENST00000389583(c.291G>T,ctG>ctT):ENSP00000374234(p.Leu97(=))]	NA	NA	NA	NA	NA	NA	NA	NA	NA	NA	MODIFIER	NA	NA	84	84	0	97	83	14	14.43
Chewer	IOB_06	chr10	45429166	G	T	TMEM72-AS1	NCT	220980:[XR_246521.1(n.712+3574C>A)]	NA	NA	NA	NA	NA	NA	NA	NA	NA	NA	MODIFIER	NA	NA	84	84	0	97	83	14	14.43
Chewer	IOB_06	chr10	50167832	C	T	WDFY4	MISSENSE	ENSG00000128815:[ENST00000325239(c.8197C>T,Cgg>Tgg):ENSP00000320563(p.Arg2733Trp)]	NA	NA	NA	NA	NA	NA	NA	D	PoD	NA	MODERATE	NA	NA	68	68	0	83	73	9	10.84
Chewer	IOB_06	chr10	73122110	C	A	SLC29A3	SILENT	ENSG00000198246:[ENST00000373189(c.1173C>A,ccC>ccA):ENSP00000362285(p.Pro391(=))]	NA	NA	chr10*73122110*73122110*C*T*rs781693842*NA*NA*NA	NA	NA	NA	NA	NA	NA	NA	MODIFIER	NA	NA	224	224	0	324	317	6	1.85
Chewer	IOB_06	chr10	77818501	C	T	C10orf11	MISSENSE	ENSG00000148655:[ENST00000372499(c.392C>T,gCg>gTg):ENSP00000361577(p.Ala131Val)]	NA	NA	NA	NA	NA	NA	NA	D	PrD	NA	MODERATE	NA	NA	54	54	0	72	57	15	20.83
Chewer	IOB_06	chr10	87482804	G	T	GRID1	MISSENSE	ENSG00000182771:[ENST00000536331(c.666C>A,aaC>aaA):ENSP00000444455(p.Asn222Lys)]	NA	NA	NA	NA	NA	NA	NA	D	PoD	NA	MODERATE	NA	NA	93	93	0	121	108	12	9.92
Chewer	IOB_06	chr10	95987092	C	T	PLCE1	SILENT	ENSG00000138193:[ENST00000371375(c.915C>T,ctC>ctT):ENSP00000360426(p.Leu305(=))]	NA	NA	NA	NA	NA	NA	NA	NA	NA	NA	LOW	NA	NA	105	105	0	124	115	8	6.45
Chewer	IOB_06	chr10	97175488	G	A	SORBS1	MISSENSE	10580:[XM_005269412.1(c.800C>T,gCg>gTg):XP_005269469.1(p.Ala267Val)]	NA	NA	NA	NA	NA	NA	NA	NA	NA	NA	MODERATE	NA	NA	100	99	0	99	94	5	5.05
Chewer	IOB_06	chr10	104130037	G	A	GBF1	INTRONIC	ENSG00000107862:[ENST00000369983(c.3408+35G>A)]	NA	NA	NA	NA	NA	NA	NA	NA	NA	NA	MODIFIER	NA	NA	67	67	0	122	117	5	4.1
Chewer	IOB_06	chr10	133946935	C	G	JAKMIP3	SILENT	ENSG00000188385:[ENST00000298622(c.753C>G,tcC>tcG):ENSP00000298622(p.Ser251(=))]	NA	NA	NA	NA	NA	NA	NA	NA	NA	NA	MODIFIER	NA	NA	90	90	0	140	122	18	12.86
Chewer	IOB_06	chr11	2167635	A	G	IGF2	INTRONIC	ENSG00000167244:[ENST00000300632(c.-7+1161T>C)]	NA	NA	NA	NA	NA	NA	NA	NA	NA	NA	MODIFIER	NA	NA	66	66	0	170	165	5	2.94
Chewer	IOB_06	chr11	2167635	A	G	IGF2-AS	EXONIC-NC	ENSG00000099869:[ENST00000445504(n.497A>G)]	NA	NA	NA	NA	NA	NA	NA	NA	NA	NA	MODIFIER	NA	NA	66	66	0	170	165	5	2.94
Chewer	IOB_06	chr11	2167635	A	G	INS-IGF2	INTRONIC	ENSG00000129965:[ENST00000356578(c.*46+1161T>C)]	NA	NA	NA	NA	NA	NA	NA	NA	NA	NA	MODIFIER	NA	NA	66	66	0	170	165	5	2.94
Chewer	IOB_06	chr11	2909437	G	A	SLC22A18AS	SILENT	ENSG00000254827:[ENST00000455942(c.426C>T,tcC>tcT):ENSP00000434027(p.Ser142(=))]	NA	NA	NA	1.71E-04	0.00E+00	NA	NA	NA	NA	NA	LOW	NA	NA	73	73	0	64	59	5	7.81
Chewer	IOB_06	chr11	6579116	G	A	DNHD1	MISSENSE	ENSG00000179532:[ENST00000254579(c.8591G>A,cGg>cAg):ENSP00000254579(p.Arg2864Gln)]	NA	NA	NA	5.47E-05	0.00E+00	NA	NA	NA	NA	NA	MODIFIER	NA	NA	148	147	0	239	227	5	2.09
Chewer	IOB_06	chr11	31131762	C	G	DCDC1	INTRONIC	ENSG00000170959:[ENST00000597505(c.1587+45G>C)]	NA	NA	chr11*31131762*31131762*C*T*rs754168459*NA*NA*NA	NA	NA	NA	NA	NA	NA	NA	MODIFIER	NA	NA	37	37	0	50	45	5	10
Chewer	IOB_06	chr11	33369271	G	A	HIPK3	MISSENSE	ENSG00000110422:[ENST00000303296(c.2116G>A,Gct>Act):ENSP00000304226(p.Ala706Thr)]	NA	NA	NA	NA	NA	NA	NA	T	BN	NA	MODERATE	D	1	70	68	0	89	78	11	12.36
Chewer	IOB_06	chr11	47599553	C	T	KBTBD4	MISSENSE	ENSG00000123444:[ENST00000533290(c.74G>A,aGc>aAc):ENSP00000436713(p.Ser25Asn)]	NA	NA	chr11*47599553*47599553*C*G,T*rs772029487*NA*NA*NA	8.29E-06	0.00E+00	NA	NA	T	BN	NA	MODERATE	NA	NA	68	68	0	85	73	11	12.94
Chewer	IOB_06	chr11	47599553	C	T	NDUFS3	NCT	ENSG00000213619:[ENST00000533507(n.816-2524C>T)]	NA	NA	chr11*47599553*47599553*C*G,T*rs772029487*NA*NA*NA	8.29E-06	0.00E+00	NA	NA	T	BN	NA	MODERATE	NA	NA	68	68	0	85	73	11	12.94
Chewer	IOB_06	chr11	56431428	C	A	OR5AR1	MISSENSE	ENSG00000172459:[ENST00000302969(c.267C>A,caC>caA):ENSP00000302639(p.His89Gln)]	NA	NA	NA	NA	NA	NA	NA	T	BN	NA	MODERATE	NA	0	157	156	0	188	173	15	7.98
Chewer	IOB_06	chr11	62434093	C	T	C11orf48	INTRONIC	ENSG00000162194:[ENST00000415855(c.147+902G>A)]	NA	NA	NA	NA	NA	NA	NA	T	PrD	NA	MODERATE	U	0.115	175	175	0	142	124	18	12.68
Chewer	IOB_06	chr11	62434093	C	T	METTL12	MISSENSE	ENSG00000214756:[ENST00000532971(c.293C>T,cCa>cTa):ENSP00000431287(p.Pro98Leu)]	NA	NA	NA	NA	NA	NA	NA	T	PrD	NA	MODERATE	U	0.115	175	175	0	142	124	18	12.68
Chewer	IOB_06	chr11	64127733	G	A	RPS6KA4	MISSENSE	ENSG00000162302:[ENST00000294261(c.226G>A,Gcc>Acc):ENSP00000294261(p.Ala76Thr)]	NA	NA	NA	NA	NA	NA	NA	T	PoD	NA	MODERATE	NA	NA	60	60	0	61	55	6	9.84
Chewer	IOB_06	chr11	64601782	T	C	CDC42BPG	MISSENSE	ENSG00000171219:[ENST00000342711(c.2369A>G,gAg>gGg):ENSP00000345133(p.Glu790Gly)]	NA	NA	NA	NA	NA	NA	NA	D	PrD	NA	MODERATE	D	0.005	46	46	0	42	32	10	23.81
Chewer	IOB_06	chr11	66637784	G	A	PC	MISSENSE	ENSG00000173599:[ENST00000355677(c.892C>T,Ctc>Ttc):ENSP00000347900(p.Leu298Phe)]	NA	NA	NA	NA	NA	NA	NA	D_lc	PrD	NA	MODERATE	D	1	45	44	0	62	51	11	17.74
Chewer	IOB_06	chr11	68305308	C	T	PPP6R3	MISSENSE	ENSG00000110075:[ENST00000265636(c.176C>T,tCa>tTa):ENSP00000265636(p.Ser59Leu)]	NA	NA	NA	NA	NA	NA	NA	NA	NA	NA	MODIFIER	NA	NA	101	101	0	129	112	17	13.18
Chewer	IOB_06	chr11	71634375	C	T	LOC100133315	EXONIC-NC	100133315:[NR_029192.1(n.402G>A)]	NA	NA	chr11*71634375*71634375*C*A*rs758749268*NA*NA*NA	NA	NA	NA	NA	NA	NA	NA	MODIFIER	NA	NA	101	101	0	104	98	6	5.77
Chewer	IOB_06	chr11	71634375	C	T	RP11-849H4.2	MISSENSE	ENSG00000254469:[ENST00000528184(c.25G>A,Gtg>Atg):ENSP00000435720(p.Val9Met)]	NA	NA	chr11*71634375*71634375*C*A*rs758749268*NA*NA*NA	NA	NA	NA	NA	NA	NA	NA	MODIFIER	NA	NA	101	101	0	104	98	6	5.77
Chewer	IOB_06	chr11	82874805	G	C	PCF11	MISSENSE	ENSG00000165494:[ENST00000298281(c.403G>C,Gat>Cat):ENSP00000298281(p.Asp135His)]	NA	NA	NA	NA	NA	NA	NA	D	PrD	NA	MODERATE	NA	NA	289	287	0	242	194	46	19.01
Chewer	IOB_06	chr11	119992793	G	A	TRIM29	INTRONIC	ENSG00000137699:[ENST00000528870(c.34+869C>T)]	NA	NA	NA	NA	NA	NA	NA	NA	NA	NA	MODIFIER	NA	NA	88	88	0	133	124	6	4.51
Chewer	IOB_06	chr11	122953898	G	A	CLMP	NONSENSE	ENSG00000166250:[ENST00000448775(c.574C>T,Cga>Tga):ENSP00000405577(p.Arg192Ter)]	NA	NA	NA	NA	NA	NA	NA	NA	NA	NA	MODIFIER	NA	NA	111	111	0	106	100	5	4.72
Chewer	IOB_06	chr12	26348838	G	A	SSPN	MISSENSE	ENSG00000123096:[ENST00000242729(c.233G>A,aGc>aAc):ENSP00000242729(p.Ser78Asn)]	NA	NA	NA	NA	NA	NA	NA	NA	NA	NA	MODIFIER	NA	NA	116	115	0	174	164	9	5.17
Chewer	IOB_06	chr12	30792654	C	A	IPO8	MISSENSE	ENSG00000133704:[ENST00000544829(c.1669G>T,Gtt>Ttt):ENSP00000444520(p.Val557Phe)]	NA	chr12*30792654*30792654*COSM1639110*C*T*1*stomach*1	chr12*30792654*30792654*C*T*rs774490198*NA*NA*NA	NA	NA	NA	NA	D	PoD	NA	MODERATE	D	1	111	111	0	172	166	5	2.91
Chewer	IOB_06	chr12	48134117	C	T	RAPGEF3	MISSENSE	ENSG00000079337:[ENST00000389212(c.2293G>A,Gcc>Acc):ENSP00000373864(p.Ala765Thr)]	NA	NA	NA	NA	NA	NA	NA	NA	NA	NA	MODIFIER	NA	NA	119	119	0	205	199	6	2.93
Chewer	IOB_06	chr12	48134117	C	T	RP1-197B17.3	EXONIC-NC	ENSG00000257433:[ENST00000547799(n.1339C>T)]	NA	NA	NA	NA	NA	NA	NA	NA	NA	NA	MODIFIER	NA	NA	119	119	0	205	199	6	2.93
Chewer	IOB_06	chr12	49445027	C	T	KMT2D	SILENT	ENSG00000167548:[ENST00000301067(c.2439G>A,ccG>ccA):ENSP00000301067(p.Pro813(=))]	NA	NA	NA	1.90E-05	1.10E-04	NA	NA	NA	NA	NA	LOW	NA	NA	206	196	1	261	242	7	2.68
Chewer	IOB_06	chr12	52164411	C	T	SCN8A	MISSENSE	ENSG00000196876:[ENST00000354534(c.3589C>T,Cac>Tac):ENSP00000346534(p.His1197Tyr)]	NA	NA	NA	NA	NA	NA	NA	NA	NA	NA	MODERATE	NA	1	92	92	0	122	115	6	4.92
Chewer	IOB_06	chr12	71095026	G	A	PTPRR	MISSENSE	ENSG00000153233:[ENST00000342084(c.749C>T,cCa>cTa):ENSP00000339605(p.Pro250Leu)]	NA	NA	NA	NA	NA	NA	NA	D	PrD	NA	MODERATE	NA	NA	110	110	0	104	83	21	20.19
Chewer	IOB_06	chr12	80613585	C	T	OTOGL	INTRONIC-SS-ACR-PRX	ENSG00000165899:[ENST00000458043(c.209-9C>T)]	NA	NA	chr12*80613585*80613585*C*G*rs773199567*NA*NA*NA	NA	NA	NA	NA	NA	NA	NA	MODIFIER	NA	NA	120	118	1	122	115	6	4.92
Chewer	IOB_06	chr12	81043440	G	A	PTPRQ	MISSENSE	ENSG00000139304:[ENST00000547881(c.273G>A,Gaa>Aaa):ENSP00000449526(p.Glu92Lys)]	NA	NA	NA	NA	NA	NA	NA	D	PrD	NA	MODERATE	NA	NA	140	140	0	166	137	28	16.87
Chewer	IOB_06	chr12	81064253	C	A	PTPRQ	MISSENSE	ENSG00000139304:[ENST00000266688(c.6572C>A,cCt>cAt):ENSP00000266688(p.Pro2191His)]	NA	NA	NA	NA	NA	NA	NA	D	PrD	NA	MODERATE	N	1	113	113	0	114	109	5	4.39
Chewer	IOB_06	chr12	81653446	T	C	PPFIA2	INTRONIC-SS-ACR-PRX	ENSG00000139220:[ENST00000549325(c.3671-7A>G)]	NA	NA	NA	NA	NA	NA	NA	NA	NA	NA	LOW	NA	NA	115	115	0	88	83	5	5.68
Chewer	IOB_06	chr12	104096958	C	T	STAB2	SILENT	ENSG00000136011:[ENST00000388887(c.3747C>T,acC>acT):ENSP00000373539(p.Thr1249(=))]	NA	NA	NA	NA	NA	NA	NA	NA	NA	NA	LOW	NA	NA	141	141	0	178	172	5	2.81
Chewer	IOB_06	chr12	118582423	C	A	PEBP1	MISSENSE	ENSG00000089220:[ENST00000261313(c.379C>A,Cag>Aag):ENSP00000261313(p.Gln127Lys)]	NA	NA	NA	NA	NA	NA	NA	D	PrD	NA	MODERATE	D	1	76	73	0	75	69	6	8
Chewer	IOB_06	chr12	120901793	G	A	SRSF9	MISSENSE	ENSG00000111786:[ENST00000229390(c.482C>T,gCc>gTc):ENSP00000229390(p.Ala161Val)]	NA	NA	NA	NA	NA	NA	NA	D	PrD	NA	MODERATE	NA	NA	115	115	0	137	131	6	4.38
Chewer	IOB_06	chr12	131605446	G	T	GPR133	SILENT	ENSG00000111452:[ENST00000543617(c.720G>T,ctG>ctT):ENSP00000438021(p.Leu240(=))]	NA	NA	NA	NA	NA	NA	NA	NA	NA	NA	MODIFIER	NA	NA	104	104	0	172	155	17	9.88
Chewer	IOB_06	chr13	34392250	C	A	RFC3	5UTR	ENSG00000133119:[ENST00000380071(c.-66C>A)]	NA	NA	NA	NA	NA	NA	NA	NA	NA	NA	MODIFIER	NA	NA	264	264	0	285	278	6	2.11
Chewer	IOB_06	chr13	38164607	G	A	POSTN	MISSENSE	ENSG00000133110:[ENST00000379742(c.343C>T,Cgc>Tgc):ENSP00000369066(p.Arg115Cys)]	NA	NA	NA	NA	NA	NA	NA	T	PoD	NA	MODERATE	N	0	117	117	0	90	76	14	15.56
Chewer	IOB_06	chr13	61985598	A	G	PCDH20	SILENT	ENSG00000197991:[ENST00000409186(c.2634T>C,caT>caC):ENSP00000386653(p.His878(=))]	NA	NA	NA	NA	NA	NA	NA	NA	NA	NA	LOW	NA	0.073	115	114	0	150	126	22	14.67
Chewer	IOB_06	chr13	73340156	C	A	DIS3	MISSENSE	ENSG00000083520:[ENST00000545453(c.1438G>T,Gac>Tac):ENSP00000440058(p.Asp480Tyr)]	NA	NA	NA	NA	NA	NA	NA	D	PrD	NA	MODERATE	D	1	135	134	0	122	117	5	4.1
Chewer	IOB_06	chr13	101318315	C	T	TMTC4	MISSENSE	ENSG00000125247:[ENST00000440120(c.209G>A,cGt>cAt):ENSP00000393600(p.Arg70His)]	NA	NA	chr13*101318315*101318315*C*G,T*rs748632078*NA*NA*NA	NA	NA	NA	NA	D_lc	UN	NA	MODERATE	NA	NA	122	122	0	127	117	9	7.09

Patel *et al.* , 2021. Whole-exome sequencing analysis of oral squamous cell carcinoma delineated by tobacco usage habits
Supplementary Table 4. List of somatic single nucleotide variants identified using whole exome sequencing of OSCC patients

Cohort	Sample	Chromosome	Genomic position	Reference allele	Alternate allele	Genesymbol	Variant type	Variant description	OncoMD	COSMIC	dbSNP	ExAC	ExAC_SAS	1000G	1000G_SAS	SIFT	Polyphen2	CONDEL	VEP	LRT	PhastCon7Way	Total reads in Normal	Reference read support in Normal	Alternative read support in Normal	Total reads in Tumor	Reference read support in Tumor	Alternate read support in Tumor	Alternate allele frequency in Tumor
Chewer	IOB_06	chr13	103343183	C	T	METTL21C	MISSENSE	ENSG00000139780:[ENST00000267273(c.262G>A,Gga>Aga):ENSP00000267273(p.Gly88Arg)]	NA	NA	NA	1.90E-04	0.00E+00	NA	NA	D	PrD	NA	MODERATE	D	1	87	86	0	114	94	20	17.54
Chewer	IOB_06	chr14	23574048	T	C	NA	EXONIC-NC	100128908:[XR_110266.2(n.124A>G)]	NA	NA	NA	NA	NA	NA	NA	NA	NA	NA	MODIFIER	NA	NA	52	52	0	52	43	9	17.31
Chewer	IOB_06	chr14	30068245	A	G	PRKD1	SILENT	ENSG00000184304:[ENST00000415220(c.2178T>C,gaT>gaC):ENSP00000390535(p.Asp726(=))]	NA	NA	NA	NA	NA	NA	NA	NA	NA	NA	LOW	NA	0.996	165	165	0	211	183	27	12.8
Chewer	IOB_06	chr14	55621434	C	T	DLGAP5	MISSENSE	ENSG00000126787:[ENST00000247191(c.1964G>A,gGc>gAc):ENSP00000247191(p.Gly655Asp)]	NA	NA	NA	NA	NA	NA	NA	T	BN	NA	MODERATE	N	0	134	133	1	151	135	15	9.93
Chewer	IOB_06	chr14	67646323	G	A	GPHN	MISSENSE	ENSG00000171723:[ENST00000478722(c.2108G>A,cGt>cAt):ENSP00000417901(p.Arg703His)]	NA	NA	NA	NA	NA	NA	NA	NA	NA	NA	MODERATE	NA	1	49	49	0	55	46	9	16.36
Chewer	IOB_06	chr14	77493135	G	C	IRF2BPL	MISSENSE	ENSG00000119669:[ENST00000238647(c.1001C>G,tCg>tGg):ENSP00000238647(p.Ser334Trp)]	NA	NA	NA	NA	NA	NA	NA	D	PrD	NA	MODERATE	U	1	348	345	1	460	404	53	11.52
Chewer	IOB_06	chr14	78226933	C	A	SLIRP	INTRONIC	ENSG00000119705:[ENST00000557431(c.265-63C>A)]	NA	NA	NA	NA	NA	NA	NA	NA	NA	NA	MODIFIER	NA	NA	71	71	0	83	78	5	6.02
Chewer	IOB_06	chr14	78226933	C	A	SNW1	INTRONIC	ENSG00000100603:[ENST00000261531(c.14+524G>T)]	NA	NA	NA	NA	NA	NA	NA	NA	NA	NA	MODIFIER	NA	NA	71	71	0	83	78	5	6.02
Chewer	IOB_06	chr14	95081322	G	A	RP11-986E7.7	3UTR	ENSG00000273259:[ENST00000553947(c.*1370G>A)]	NA	NA	3.99E-04	2.47E-05	0.00E+00	3.99E-04	0.00E+00	T	BN	NA	MODERATE	NA	NA	70	70	0	81	71	9	11.11
Chewer	IOB_06	chr14	95081322	G	A	SERPINA3	MISSENSE	ENSG00000196136:[ENST00000393078(c.544G>A,Gac>Aac):ENSP00000376793(p.Asp182Asn)]	NA	NA	3.99E-04	2.47E-05	0.00E+00	3.99E-04	0.00E+00	T	BN	NA	MODERATE	NA	NA	70	70	0	81	71	9	11.11
Chewer	IOB_06	chr15	23890243	C	T	AC124309.1	EXONIC-NC	ENSG00000179449:[ENST00000314233(n.736G>A)]	NA	NA	NA	NA	NA	NA	NA	D	BN	NA	MODERATE	NA	NA	124	123	0	256	225	31	12.11
Chewer	IOB_06	chr15	23890243	C	T	MAGEL2	MISSENSE	ENSG00000254585:[ENST00000532292(c.838G>A,Ggc>Agc):ENSP00000433433(p.Gly280Ser)]	NA	NA	NA	NA	NA	NA	NA	D	BN	NA	MODERATE	NA	NA	124	123	0	256	225	31	12.11
Chewer	IOB_06	chr15	35174773	G	A	AQR	MISSENSE	ENSG00000021776:[ENST00000156471(c.3095C>T,gCc>gTc):ENSP00000156471(p.Ala1032Val)]	NA	NA	NA	NA	NA	NA	NA	D	PoD	NA	MODERATE	D	1	136	136	0	136	120	16	11.76
Chewer	IOB_06	chr15	41786597	G	A	ITPKA	MISSENSE	ENSG00000137825:[ENST00000425927(c.157G>A,Ggc>Agc):ENSP00000396560(p.Gly53Ser)]	NA	NA	NA	NA	NA	NA	NA	T	BN	NA	MODERATE	N	0	63	63	0	117	106	11	9.4
Chewer	IOB_06	chr15	64665960	C	A	CSNK1G1	5UTR	53944:[XM_005254466.1(c.-431G>T)]	NA	NA	NA	NA	NA	NA	NA	NA	NA	NA	MODIFIER	NA	NA	122	122	0	141	133	8	5.67
Chewer	IOB_06	chr15	64665960	C	A	CTD-2116N17.1	5UTR	ENSG00000259316:[ENST00000560278(c.-390G>T)]	NA	NA	NA	NA	NA	NA	NA	NA	NA	NA	MODIFIER	NA	NA	122	122	0	141	133	8	5.67
Chewer	IOB_06	chr15	64665960	C	A	KIAA0101	MISSENSE	ENSG00000166803:[ENST00000558008(c.294G>T,caG>caT):ENSP00000453527(p.Gln98His)]	NA	NA	NA	NA	NA	NA	NA	NA	NA	NA	MODIFIER	NA	NA	122	122	0	141	133	8	5.67
Chewer	IOB_06	chr15	64665960	C	A	PCLAF	NCT	9768:[NR_109934.1(n.485+2982G>T)]	NA	NA	NA	NA	NA	NA	NA	NA	NA	NA	MODIFIER	NA	NA	122	122	0	141	133	8	5.67
Chewer	IOB_06	chr15	83357512	G	T	AP3B2	SILENT	ENSG00000103723:[ENST00000541693(c.204C>A,atC>atA):ENSP00000441961(p.Ile68(=))]	NA	NA	NA	NA	NA	NA	NA	NA	NA	NA	MODIFIER	NA	NA	133	133	0	155	141	14	9.03
Chewer	IOB_06	chr15	83357512	G	T	RP11-752G15.3	NCT	ENSG00000259462:[ENST00000560650(n.1329-3251G>T)]	NA	NA	NA	NA	NA	NA	NA	NA	NA	NA	MODIFIER	NA	NA	133	133	0	155	141	14	9.03
Chewer	IOB_06	chr15	84116341	G	A	SH3GL3	INTRONIC	ENSG00000140600:[ENST00000324537(c.-445+11G>A)]	NA	NA	chr15*84116332*84116347*TAGGGAGGCGCAGAGG*T*rs777565549*NA*NA*NA	NA	NA	NA	NA	NA	NA	NA	MODIFIER	NA	NA	171	171	0	221	210	11	4.98
Chewer	IOB_06	chr15	87572130	G	T	AGBL1	3UTR	ENSG00000166748:[ENST00000441037(c.*59G>T)]	NA	NA	NA	NA	NA	NA	NA	NA	NA	NA	MODIFIER	NA	NA	144	143	0	129	101	22	17.05
Chewer	IOB_06	chr15	91434223	G	A	FES	SILENT	ENSG00000182511:[ENST00000328850(c.1332G>A,ccG>ccA):ENSP00000331504(p.Pro444(=))]	NA	NA	2.00E-04	2.80E-05	0.00E+00	2.00E-04	0.00E+00	NA	NA	NA	MODIFIER	NA	NA	89	89	0	109	95	14	12.84
Chewer	IOB_06	chr15	91452583	G	A	MAN2A2	MISSENSE	ENSG00000196547:[ENST00000560616(c.135G>A,Gga>Aga):ENSP00000452866(p.Gly46Arg)]	NA	NA	NA	2.48E-05	0.00E+00	NA	NA	D	PrD	NA	MODERATE	NA	NA	85	84	0	92	79	13	14.13
Chewer	IOB_06	chr16	1251780	G	T	CACNA1H	MISSENSE	ENSG00000196557:[ENST00000348261(c.1330G>T,Gac>Tac):ENSP00000334198(p.Asp444Tyr)]	NA	NA	chr16*1251780*1251780*G*A*rs879352720*NA*NA*NA	NA	NA	NA	NA	D	PrD	NA	MODERATE	N	1	102	100	0	133	110	22	16.54
Chewer	IOB_06	chr16	1991223	G	A	MSRB1	MISSENSE	ENSG00000198736:[ENST00000399753(c.239C>T,gCg>gTg):ENSP00000382657(p.Ala80Val)]	NA	NA	NA	2.28E-05	0.00E+00	NA	NA	NA	NA	NA	MODIFIER	NA	NA	99	99	0	128	120	7	5.47
Chewer	IOB_06	chr16	53737970	T	A	FTO	5UTR	ENSG00000140718:[ENST00000471389(c.-127T>A)]	NA	NA	NA	NA	NA	NA	NA	NA	NA	NA	MODIFIER	NA	NA	61	61	0	74	60	13	17.57
Chewer	IOB_06	chr16	67980055	C	A	SLC12A4	INTRONIC	ENSG00000124067:[ENST00000422611(c.2613+19G>T)]	NA	NA	NA	NA	NA	NA	NA	NA	NA	NA	MODIFIER	NA	NA	180	180	0	217	211	5	2.3
Chewer	IOB_06	chr16	76587325	C	T	CNTNAP4	MISSENSE	85445:[NM_033401.3(c.3587C>T,aCg>aTg):NP_207837.2(p.Thr1196Met)]	NA	NA	1.14E-02	3.68E-03	2.73E-04	1.14E-02	0.00E+00	NA	NA	NA	MODIFIER	NA	NA	58	57	0	74	56	16	21.62
Chewer	IOB_06	chr16	76587325	C	T	RP11-58C22.1	SILENT	ENSG00000261833:[ENST00000563764(c.12C>T,caC>caT):ENSP00000455258(p.His4(=))]	NA	NA	1.14E-02	3.68E-03	2.73E-04	1.14E-02	0.00E+00	NA	NA	NA	MODIFIER	NA	NA	58	57	0	74	56	16	21.62
Chewer	IOB_06	chr16	85100809	G	A	KIAA0513	SILENT	ENSG00000135709:[ENST00000258180(c.132G>A,gaG>gaA):ENSP00000258180(p.Glu44(=))]	NA	NA	NA	NA	NA	NA	NA	NA	NA	NA	LOW	NA	1	213	213	0	347	332	14	4.03
Chewer	IOB_06	chr16	85112537	C	T	KIAA0513	MISSENSE	ENSG00000135709:[ENST00000562580(c.221C>T,gCg>gTg):ENSP00000454547(p.Ala74Val)]	NA	NA	2.00E-04	1.07E-04	3.63E-04	2.00E-04	1.00E-03	T_lc	BN	NA	MODERATE	NA	NA	120	120	0	191	175	16	8.38
Chewer	IOB_06	chr16	88496189	C	A	ZNF469	MISSENSE	ENSG00000225614:[ENST00000437464(c.2311C>A,Cct>Act):ENSP00000402343(p.Pro771Thr)]	NA	NA	NA	NA	NA	NA	NA	NA	BN	NA	MODERATE	NA	NA	141	141	0	210	201	6	2.86
Chewer	IOB_06	chr17	2995841	G	A	OR1D2	SILENT	ENSG00000184166:[ENST00000331459(c.450C>T,gtC>gtT):ENSP00000327585(p.Val150(=))]	NA	NA	NA	NA	NA	NA	NA	NA	NA	NA	LOW	NA	NA	70	69	0	132	121	11	8.33
Chewer	IOB_06	chr17	7578403	C	A	TP53	MISSENSE	ENSG00000141510:[ENST00000269305(c.527G>T,tGc>tTc):ENSP00000269305(p.Cys176Phe)]	NA	chr17*7578370*7578416*COSM45583*BigSeq*C*2*upper_aerodigestive_tract,liver*1,1|chr17*7578373*7578419*COSM44264*BigSeq*T*1*large_intestine*1|chr17*7578375*7578413*COSM45676*BigSeq*G*1*lung*1|chr17*7578377*7578409*COSM100003*BigSeq*T*1*upper_aerodigestive_tract*1|chr17*7578377*7578409*COSM100005*BigSeq*T*1*upper_aerodigestive_tract*1|chr17*7578377*7578409*COSM100001*BigSeq*T*1*upper_aerodigestive_tract*1|chr17*7578377*7578409*COSM100002*BigSeq*T*1*upper_aerodigestive_tract*1|chr17*7578377*7578409*COSM100004*BigSeq*T*1*upper_aerodigestive_tract*1|chr17*7578385*7578404*COSM45537*CAGCGCTCATGGTGGGGCA*C*1*ovary*1|chr17*7578386*7578404*COSM45399*AGCGCTCATGGTGGGGGCA*A*3*ovary,oesophagus*1,2|chr17*7578389*75784	NA	NA	NA	NA	NA	NA	NA	NA	MODIFIER	NA	NA	125	123	0	101	94	7	6.93
Chewer	IOB_06	chr17	8473091	G	C	MYH10	MISSENSE	ENSG00000133026:[ENST00000269243(c.673C>G,Ctg>Gtg):ENSP00000269243(p.Leu225Val)]	NA	NA	NA	NA	NA	NA	NA	D	PrD	NA	MODERATE	NA	1	127	127	0	127	109	18	14.17
Chewer	IOB_06	chr17	18152748	C	T	FLII	INTRONIC	ENSG00000177731:[ENST00000545457(c.1612-26G>A)]	NA	NA	NA	NA	NA	NA	NA	NA	NA	NA	MODIFIER	NA	NA	30	30	0	31	23	6	19.35
Chewer	IOB_06	chr17	32963160	C	T	TMEM132E	SILENT	ENSG00000181291:[ENST00000321639(c.1842C>T,agC>agT):ENSP00000316532(p.Ser614(=))]	NA	NA	NA	NA	NA	NA	NA	NA	NA	NA	MODIFIER	NA	NA	72	71	0	115	94	21	18.26
Chewer	IOB_06	chr17	34100284	C	G	MMP28	MISSENSE	ENSG00000129270:[ENST00000587639(c.504G>C,tgG>tgC):ENSP00000464731(p.Trp168Cys)]	NA	NA	NA	NA	NA	NA	NA	D_lc	UN	NA	MODERATE	NA	NA	185	185	0	188	168	20	10.64
Chewer	IOB_06	chr17	36490638	C	T	GPR179	MISSENSE	ENSG00000188888:[ENST00000342292(c.1733G>A,gGc>gAc):ENSP00000345060(p.Gly578Asp)]	NA	NA	NA	NA	NA	NA	NA	D	PoD	NA	MODERATE	NA	0.998	82	81	0	124	118	5	4.03
Chewer	IOB_06	chr17	40855758	C	A	EZH1	MISSENSE-SS-PRX	ENSG00000108799:[ENST00000588897(c.*1605G>T)]	NA	NA	NA	NA	NA	NA	NA	NA	NA	NA	MODIFIER	NA	NA	196	196	0	191	185	5	2.62
Chewer	IOB_06	chr17	40932983	C	T	WNK4	SILENT	ENSG00000126562:[ENST00000246914(c.267C>T,tcC>tcT):ENSP00000246914(p.Ser89(=))]	NA	NA	NA	1.17E-04	0.00E+00	NA	NA	NA	NA	NA	MODIFIER	NA	NA	84	84	0	105	96	9	8.57
Chewer	IOB_06	chr17	42170917	C	T	HDAC5	INTRONIC	ENSG00000108840:[ENST00000225983(c.357+26G>A)]	NA	NA	NA	NA	NA	NA	NA	NA	NA	NA	MODIFIER	NA	NA	192	192	0	227	220	5	2.2
Chewer	IOB_06	chr17	56355299	C	T	MPO	MISSENSE	ENSG00000005381:[ENST00000340482(c.1189G>A,Gtc>Atc):ENSP00000344419(p.Val397Ile)]	NA	NA	NA	NA	NA	NA	NA	NA	NA	NA	MODIFIER	NA	NA	140	140	0	150	145	5	3.33
Chewer	IOB_06	chr17	57430790	C	T	YPEL2	MISSENSE	ENSG00000175155:[ENST00000312655(c.20C>T,tCg>tTg):ENSP00000312272(p.Ser7Leu)]	NA	chr17*57430790*57430790*COSM561517*C*G*1*lung*1	NA	8.92E-06	1.00E-04	NA	NA	NA	NA	NA	MODIFIER	NA	NA	47	47	0	52	47	5	9.62
Chewer	IOB_06	chr17	76503508	G	A	DNAH17	NCT	ENSG00000187775:[ENST00000587177(n.636+32C>T)]	NA	NA	chr17*76503508*76503508*G*C*rs752844488*NA*NA*NA	NA	NA	NA	NA	NA	NA	NA	MODIFIER	NA	NA	146	145	0	255	227	27	10.59
Chewer	IOB_06	chr18	1269757	C	A	LINC00470	EXONIC-NC	56651:[NR_023927.1(n.445G>T)]	NA	NA	NA	NA	NA	NA	NA	NA	NA	NA	MODIFIER	NA	NA	43	43	0	47	42	5	10.64
Chewer	IOB_06	chr18	22040855	C	A	HRH4	MISSENSE	ENSG00000134489:[ENST00000256906(c.163C>A,Ctt>Att):ENSP00000256906(p.Leu55Ile)]	NA	NA	chr18*22040854*22040859*TCTTAA*T*rs765452459*NA*NA*NA	NA	NA	NA	NA	D	PrD	NA	MODERATE	D	1	75	75	0	65	60	5	7.69
Chewer	IOB_06	chr18	31318507	G	A	ASXL3	MISSENSE	ENSG00000141431:[ENST00000269197(c.1139G>A,gGa>gAa):ENSP00000269197(p.Gly380Glu)]	NA	NA	NA	NA	NA	NA	NA	NA	NA	NA	MODERATE	NA	1	79	79	0	74	68	6	8.11
Chewer	IOB_06	chr18	74672700	C	T	ZNF236	MISSENSE	ENSG00000130856:[ENST00000320610(c.5308C>T,Cac>Tac):ENSP00000322361(p.His1770Tyr)]	NA	NA	NA	NA	NA	NA	NA	D	PoD	NA	MODERATE	D	1	123	123	0	160	143	17	10.62
Chewer	IOB_06	chr18	74680110	G	A	ZNF236	INTRONIC	ENSG00000130856:[ENST00000253159(c.5414-61G>A)]	NA	NA	NA	NA	NA	NA	NA	NA	NA	NA	MODIFIER	NA	NA	42	42	0	30	23	7	23.33
Chewer	IOB_06	chr19	5892707	C	T	AC024592.12	INTRONIC	ENSG00000267740:[ENST00000585661(c.308+3757G>A)]	NA	NA	NA	NA	NA	NA	NA	NA	NA	NA	MODIFIER	NA	NA	76	74	1	88	72	16	18.18
Chewer	IOB_06	chr19	5892707	C	T	FUT5	INTRONIC	ENSG00000130383:[ENST00000252675(c.-172+3757G>A)]	NA	NA	NA	NA	NA	NA	NA	NA	NA	NA	MODIFIER	NA	NA	76	74	1	88	72	16	18.18
Chewer	IOB_06	chr19	5892707	C	T	NDUFA11	3UTR	ENSG00000174886:[ENST00000418389(c.*221G>A)]	NA	NA	NA	NA	NA	NA	NA	NA	NA	NA	MODIFIER	NA	NA	76	74	1	88	72	16	18.18
Chewer	IOB_06	chr19	8933287	C	T	CTD-2529P6.3	NCT	ENSG00000269300:[ENST00000594006(n.185+926C>T)]	NA	NA	NA	NA	NA	NA	NA	NA	NA	NA	MODIFIER	NA	NA	117	117	0	183	174	8	4.37
Chewer	IOB_06	chr19	8933287	C	T	ZNF558	NCT	ENSG00000167785:[ENST00000597304(n.530+18G>A)]	NA	NA	NA	NA	NA	NA	NA	NA	NA	NA	MODIFIER	NA	NA	117	117	0	183	174	8	4.37
Chewer	IOB_06	chr19	8979379	T	A	MUC16	NCT	94025:[XR_244090.1(n.17944+2778A>T)]	NA	NA	NA	NA	NA	NA	NA	NA	NA	NA	MODIFIER	NA	NA	26	26	0	27	22	5	18.52
Chewer	IOB_06	chr19	12940664	G	T	CTD-2265O21.3	NCT	ENSG00000267424:[ENST00000588469(n.311-2705G>T)]	NA	NA	chr19*12940664*12940664*G*A*rs779628160*NA*NA*NA	NA	NA	NA	NA	D	PrD	NA	MODERATE	NA	NA	110	110	0	139	134	5	3.6
Chewer	IOB_06	chr19	12940664	G	T	HOOK2	NCT	ENSG00000095066:[ENST00000589765(n.33-3350C>A)]	NA	NA	chr19*12940664*12940664*G*A*rs779628160*NA*NA*NA	NA	NA	NA	NA	D	PrD	NA	MODERATE	NA	NA	110	110	0	139	134	5	3.6

Patel *et al.* , 2021. Whole-exome sequencing analysis of oral squamous cell carcinoma delineated by tobacco usage habits
Supplementary Table 4. List of somatic single nucleotide variants identified using whole exome sequencing of OSCC patients

Cohort	Sample	Chromosome	Genomic position	Reference allele	Alternate allele	Genesymbol	Variant type	Variant description	OncoMD	COSMIC	dbSNP	ExAC	ExAC_SAS	1000G	1000G_SAS	SIFT	Polyphen2	CONDEL	VEP	LRT	PhastCon7Way	Total reads in Normal	Reference read support in Normal	Alternative read support in Normal	Total reads in Tumor	Reference read support in Tumor	Alternate read support in Tumor	Alternate allele frequency in Tumor
Chewer	IOB_06	chr19	12940664	G	T	RTBDN	MISSENSE	ENSG00000132026:[ENST00000322912(c.226C>A,Ctg>Atg):ENSP00000326253(p.Leu76Met)]	NA	NA	chr19*12940664*12940664*G*A*rs779628160*NA*NA*NA	NA	NA	NA	NA	D	PrD	NA	MODERATE	NA	NA	110	110	0	139	134	5	3.6
Chewer	IOB_06	chr19	17412239	C	T	ABHD8	MISSENSE	ENSG00000127220:[ENST00000247706(c.187G>A,Gca>Aca):ENSP00000247706(p.Ala63Thr)]	NA	chr19*17412239*17412239*COSM1391437*C*T*1*large_intestine*1	chr19*17412237*17412261*TGCGGATGATGGTGGAGGTGGGGCA*T*rs768033957*NA*NA*NA|chr19*17412239*17412239*C*G,T*rs374386420*NA*NA*NA	8.74E-06	0.00E+00	NA	NA	T	BN	NA	MODERATE	NA	NA	98	98	0	102	96	6	5.88
Chewer	IOB_06	chr19	17412239	C	T	MRPL34	INTRONIC	ENSG00000130312:[ENST00000600434(c.-63+3053C>T)]	NA	chr19*17412239*17412239*COSM1391437*C*T*1*large_intestine*1	chr19*17412237*17412261*TGCGGATGATGGTGGAGGTGGGGCA*T*rs768033957*NA*NA*NA|chr19*17412239*17412239*C*G,T*rs374386420*NA*NA*NA	8.74E-06	0.00E+00	NA	NA	T	BN	NA	MODERATE	NA	NA	98	98	0	102	96	6	5.88
Chewer	IOB_06	chr19	18974343	G	A	UPF1	SILENT	ENSG00000005007:[ENST00000599848(c.2730G>A,gtG>gtA):ENSP00000470142(p.Val910(=))]	NA	NA	NA	NA	NA	NA	NA	NA	NA	NA	LOW	NA	NA	79	79	0	91	86	5	5.49
Chewer	IOB_06	chr19	37210298	C	A	ZNF567	SILENT	ENSG00000189042:[ENST00000536254(c.672C>A,ggC>ggA):ENSP00000441838(p.Gly224(=))]	NA	NA	chr19*37210298*37210298*C*T*rs1048363715*NA*NA*NA	NA	NA	NA	NA	NA	NA	NA	MODIFIER	NA	NA	92	91	1	67	53	14	20.9
Chewer	IOB_06	chr19	37210298	C	A	ZNF850	INTRONIC	ENSG00000267041:[ENST00000589390(c.236-4896G>T)]	NA	NA	chr19*37210298*37210298*C*T*rs1048363715*NA*NA*NA	NA	NA	NA	NA	NA	NA	NA	MODIFIER	NA	NA	92	91	1	67	53	14	20.9
Chewer	IOB_06	chr19	38103514	G	T	ZNF540	MISSENSE	ENSG00000171817:[ENST00000589117(c.1237G>T,Ggg>Tgg):ENSP00000467118(p.Gly413Trp)]	NA	NA	chr19*38103514*38103514*G*A*rs777388477*NA*NA*NA	NA	NA	NA	NA	D	PrD	NA	MODERATE	NA	NA	105	104	0	104	99	5	4.81
Chewer	IOB_06	chr19	41306923	G	A	EGLN2	MISSENSE	ENSG00000269858:[ENST00000303961(c.446G>A,gGc>gAc):ENSP00000307080(p.Gly149Asp)]	NA	NA	NA	NA	NA	NA	NA	T_lc	BN	NA	MODERATE	N	0	202	202	0	258	250	6	2.33
Chewer	IOB_06	chr19	41306923	G	A	RAB4B-EGLN2	EXONIC-NC	100529264:[NR_037791.1(n.1494G>A)]	NA	NA	NA	NA	NA	NA	NA	T_lc	BN	NA	MODERATE	N	0	202	202	0	258	250	6	2.33
Chewer	IOB_06	chr19	53612314	G	T	ZNF415	NONSENSE	ENSG00000170954:[ENST00000421033(c.1020C>A,taC>taA):ENSP00000395055(p.Tyr340Ter)]	NA	NA	chr19*53612314*53612314*G*A*rs755668523*NA*NA*NA	NA	NA	NA	NA	NA	NA	NA	HIGH	NA	NA	93	93	0	138	123	15	10.87
Chewer	IOB_06	chr19	54074929	G	A	ZNF331	SILENT	ENSG00000130844:[ENST00000253144(c.81G>A,caG>caA):ENSP00000253144(p.Gln27(=))]	NA	NA	NA	NA	NA	NA	NA	NA	NA	NA	LOW	NA	NA	91	91	0	113	97	15	13.27
Chewer	IOB_06	chr19	58384640	G	C	ZNF814	MISSENSE	ENSG00000204514:[ENST00000435989(c.2118C>G,caC>caG):ENSP00000410545(p.His706Gln)]	NA	NA	chr19*58384640*58384640*G*A*rs767832799*NA*NA*NA	NA	NA	NA	NA	NA	NA	NA	MODIFIER	NA	NA	171	171	0	145	119	26	17.93
Chewer	IOB_06	chr2	27664699	C	G	NRBP1	EXONIC-NC	ENSG00000115216:[ENST00000460499(n.2138C>G)]	NA	NA	NA	NA	NA	NA	NA	NA	NA	NA	MODIFIER	NA	NA	69	69	0	62	57	5	8.06
Chewer	IOB_06	chr2	29295262	C	A	C2orf71	MISSENSE	ENSG00000179270:[ENST00000331664(c.1866G>T,aaG>aaT):ENSP00000332809(p.Lys622Asn)]	NA	NA	NA	NA	NA	NA	NA	D	BN	NA	MODERATE	NA	NA	99	99	0	132	126	5	3.79
Chewer	IOB_06	chr2	33335715	G	T	LTBP1	MISSENSE	ENSG00000049323:[ENST00000354476(c.930G>T,aaG>aaT):ENSP00000346467(p.Lys310Asn)]	NA	NA	NA	NA	NA	NA	NA	D	BN	NA	MODERATE	NA	NA	122	121	0	190	185	5	2.63
Chewer	IOB_06	chr2	84934783	C	A	DNAH6	MISSENSE	ENSG00000115423:[ENST00000237449(c.8991C>A,ttC>ttA):ENSP00000237449(p.Phe2997Leu)]	NA	NA	NA	NA	NA	NA	NA	T	BN	NA	MODERATE	N	0.999	106	106	0	118	113	5	4.24
Chewer	IOB_06	chr2	89292134	G	C	IGKV1-8	MISSENSE	ENSG00000240671:[ENST00000495489(c.139C>G,Cag>Gag):ENSP00000419211(p.Gln47Glu)]	NA	NA	NA	8.33E-06	0.00E+00	NA	NA	NA	NA	NA	MODIFIER	NA	NA	188	188	0	252	218	34	13.49
Chewer	IOB_06	chr2	89292134	G	C	NA	NCT	101928457:[XR_245045.1(n.34-17347G>C)]	NA	NA	NA	8.33E-06	0.00E+00	NA	NA	NA	NA	NA	MODIFIER	NA	NA	188	188	0	252	218	34	13.49
Chewer	IOB_06	chr2	96689175	C	T	GPAT2	MISSENSE	ENSG00000186281:[ENST00000359548(c.1910G>A,cGg>cAg):ENSP00000352547(p.Arg637Gln)]	NA	NA	NA	NA	NA	NA	NA	T	BN	NA	MODERATE	N	0.138	147	146	0	217	191	26	11.98
Chewer	IOB_06	chr2	120889176	T	C	EPB41L5	INTRONIC	57669:[XM_005263720.1(c.1511-18T>C)]	NA	NA	NA	2.38E-02	1.64E-02	NA	NA	NA	NA	NA	MODIFIER	NA	NA	76	75	0	72	65	6	8.33
Chewer	IOB_06	chr2	152419237	C	A	NEB	MISSENSE	ENSG00000183091:[ENST00000413693(c.2969G>T,tGc>tTc):ENSP00000410961(p.Cys990Phe)]	NA	NA	NA	NA	NA	NA	NA	NA	NA	NA	MODERATE	NA	1	74	74	0	108	102	6	5.56
Chewer	IOB_06	chr2	179639817	C	A	TTN	MISSENSE	ENSG00000155657:[ENST00000342992(c.6621G>T,tgG>tgT):ENSP00000343764(p.Trp2207Cys)]	NA	chr2*179639817*179639817*COSM441853*C*G*1*breast*1|chr2*179639817*179639817*COSM441855*C*G*1*breast*1|chr2*179639817*179639817*COSM441854*C*G*1*breast*1|chr2*179639817*179639817*COSM441851*C*G*1*breast*1|chr2*179639817*179639817*COSM1482495*C*G*1*breast*1|chr2*179639817*179639817*COSM441852*C*G*1*breas	NA	NA	NA	NA	NA	NA	PrD	NA	MODERATE	NA	NA	321	320	0	325	316	5	1.54
Chewer	IOB_06	chr2	179666973	C	T	TTN	MISSENSE	ENSG00000155657:[ENST00000342175(c.187G>A,Gct>Act):ENSP00000340554(p.Ala63Thr)]	NA	chr2*179666973*179666973*COSM165291*C*T*2*NS,large_intestine*1,1|chr2*179666973*179666973*COSM165292*C*T*2*NS,large_intestine*1,1|chr2*179666973*179666973*COSM165293*C*T*2*NS,large_intestine*1,1|chr2*179666973*179666973*COSM165294*C*T*2*NS,large_intestine*1,1	NA	4.12E-05	0.00E+00	NA	NA	NA	NA	NA	MODIFIER	NA	NA	89	88	0	107	101	5	4.67
Chewer	IOB_06	chr2	198950131	T	C	PLCL1	SILENT	ENSG00000115896:[ENST00000437704(c.1596T>C,gaT>gaC):ENSP00000414138(p.Asp532(=))]	NA	NA	NA	NA	NA	NA	NA	NA	NA	NA	LOW	NA	NA	90	90	0	120	101	19	15.83
Chewer	IOB_06	chr2	202141689	C	T	CASP8	MISSENSE	ENSG00000064012:-SS-PRX[ENST00000323492(c.755C>T,gCa>gTa):ENSP00000325722(p.Ala252Val)]	NA	chr2*202141689*202141689*COSM209550*C*T*1*large_intestine*1|chr2*202141689*202141689*COSM209551*C*T*1*large_intestine*1	NA	NA	NA	NA	NA	NA	NA	NA	LOW	NA	NA	64	63	1	90	83	6	6.67
Chewer	IOB_06	chr2	206988969	G	A	NDUFS1	SILENT	ENSG00000023228:[ENST00000432169(c.1791C>T,gcC>gcT):ENSP00000409689(p.Ala597(=))]	NA	NA	NA	NA	NA	NA	NA	NA	NA	NA	LOW	NA	NA	121	121	0	140	134	6	4.29
Chewer	IOB_06	chr2	211447354	G	A	CPS1	MISSENSE	ENSG00000021826:[ENST00000430249(c.560G>A,gGg>gAg):ENSP00000402608(p.Gly187Glu)]	NA	NA	NA	NA	NA	NA	NA	D	PrD	NA	MODERATE	D	1	178	177	0	167	152	13	7.78
Chewer	IOB_06	chr2	227662312	G	A	IRS1	SILENT	ENSG00000169047:[ENST00000305123(c.1143C>T,cgC>cgT):ENSP00000304895(p.Arg381(=))]	NA	chr2*227662312*227662312*COSM3695310*G*A*1*large_intestine*1	NA	2.72E-04	0.00E+00	NA	NA	NA	NA	NA	LOW	NA	NA	203	202	0	354	329	25	7.06
Chewer	IOB_06	chr2	227771540	C	T	RHBDD1	MISSENSE	ENSG00000144468:[ENST00000409053(c.100C>T,Ctt>Ttt):ENSP00000387269(p.Leu34Phe)]	NA	NA	NA	NA	NA	NA	NA	D	PrD	NA	MODERATE	N	1	85	85	0	157	130	27	17.2
Chewer	IOB_06	chr2	233385294	C	A	PRSS56	5UTR	ENSG00000237412:[ENST00000449534(c.-15C>A)]	NA	NA	NA	NA	NA	NA	NA	NA	NA	NA	MODIFIER	NA	NA	93	92	0	76	69	7	9.21
Chewer	IOB_06	chr2	234676924	C	A	UGT1A1	MISSENSE	ENSG00000242366:[ENST00000305208(c.1143C>A,agC>agA):ENSP00000304845(p.Ser381Arg)]	NA	NA	chr2*234676924*234676924*C*G*rs72551354*NA*NA*NA	NA	NA	NA	NA	NA	NA	NA	MODIFIER	NA	NA	68	68	0	75	69	6	8
Chewer	IOB_06	chr2	234676924	C	A	UGT1A10	MISSENSE	ENSG00000242515:[ENST00000344644(c.1134C>A,agC>agA):ENSP00000343838(p.Ser378Arg)]	NA	NA	chr2*234676924*234676924*C*G*rs72551354*NA*NA*NA	NA	NA	NA	NA	NA	NA	NA	MODIFIER	NA	NA	68	68	0	75	69	6	8
Chewer	IOB_06	chr2	234676924	C	A	UGT1A3	MISSENSE	ENSG00000243135:[ENST00000482026(c.1146C>A,agC>agA):ENSP00000418532(p.Ser382Arg)]	NA	NA	chr2*234676924*234676924*C*G*rs72551354*NA*NA*NA	NA	NA	NA	NA	NA	NA	NA	MODIFIER	NA	NA	68	68	0	75	69	6	8
Chewer	IOB_06	chr2	234676924	C	A	UGT1A4	MISSENSE	ENSG00000244474:[ENST00000373409(c.1146C>A,agC>agA):ENSP00000362508(p.Ser382Arg)]	NA	NA	chr2*234676924*234676924*C*G*rs72551354*NA*NA*NA	NA	NA	NA	NA	NA	NA	NA	MODIFIER	NA	NA	68	68	0	75	69	6	8
Chewer	IOB_06	chr2	234676924	C	A	UGT1A5	MISSENSE	ENSG00000240224:[ENST00000373414(c.1146C>A,agC>agA):ENSP00000362513(p.Ser382Arg)]	NA	NA	chr2*234676924*234676924*C*G*rs72551354*NA*NA*NA	NA	NA	NA	NA	NA	NA	NA	MODIFIER	NA	NA	68	68	0	75	69	6	8
Chewer	IOB_06	chr2	234676924	C	A	UGT1A6	MISSENSE	ENSG00000167165:[ENST00000305139(c.1140C>A,agC>agA):ENSP00000303174(p.Ser380Arg)]	NA	NA	chr2*234676924*234676924*C*G*rs72551354*NA*NA*NA	NA	NA	NA	NA	NA	NA	NA	MODIFIER	NA	NA	68	68	0	75	69	6	8
Chewer	IOB_06	chr2	234676924	C	A	UGT1A7	MISSENSE	ENSG00000244122:[ENST00000373426(c.1134C>A,agC>agA):ENSP00000362525(p.Ser378Arg)]	NA	NA	chr2*234676924*234676924*C*G*rs72551354*NA*NA*NA	NA	NA	NA	NA	NA	NA	NA	MODIFIER	NA	NA	68	68	0	75	69	6	8
Chewer	IOB_06	chr2	234676924	C	A	UGT1A8	MISSENSE	ENSG00000241635:[ENST00000608381(c.1146C>A,agC>agA):ENSP00000477394(p.Ser382Arg)]	NA	NA	chr2*234676924*234676924*C*G*rs72551354*NA*NA*NA	NA	NA	NA	NA	NA	NA	NA	MODIFIER	NA	NA	68	68	0	75	69	6	8
Chewer	IOB_06	chr2	234676924	C	A	UGT1A9	MISSENSE	ENSG00000241119:[ENST00000354728(c.1134C>A,agC>agA):ENSP00000346768(p.Ser378Arg)]	NA	NA	chr2*234676924*234676924*C*G*rs72551354*NA*NA*NA	NA	NA	NA	NA	NA	NA	NA	MODIFIER	NA	NA	68	68	0	75	69	6	8
Chewer	IOB_06	chr2	238494648	C	A	RAB17	SILENT	ENSG00000124839:[ENST00000264601(c.150G>T,acG>acT):ENSP00000264601(p.Thr50(=))]	NA	NA	chr2*238494645*238494650*CACCGT*C*rs750428436*NA*NA*NA	NA	NA	NA	NA	NA	NA	NA	MODIFIER	NA	NA	146	145	0	181	175	6	3.31
Chewer	IOB_06	chr20	334206	C	T	NRSN2	MISSENSE	ENSG00000125841:[ENST00000382285(c.542C>T,tCa>tTa):ENSP00000371722(p.Ser181Leu)]	NA	NA	NA	NA	NA	NA	NA	D	BN	NA	MODERATE	N	0.002	121	120	1	177	144	32	18.08
Chewer	IOB_06	chr20	3732690	G	A	HSPA12B	SILENT	ENSG00000132622:[ENST00000399701(c.1680G>A,gcG>gcA):ENSP00000382608(p.Ala560(=))]	NA	NA	chr20*3732690*3732690*G*T*rs939965908*NA*NA*NA	NA	NA	NA	NA	NA	NA	NA	LOW	NA	NA	478	476	1	563	549	12	2.13
Chewer	IOB_06	chr20	18163890	G	A	CSRP2BP	SILENT	ENSG00000149474:[ENST00000377681(c.1929G>A,cgG>cgA):ENSP00000366909(p.Arg643(=))]	NA	NA	NA	NA	NA	NA	NA	NA	NA	NA	LOW	NA	NA	121	119	0	205	199	6	2.93
Chewer	IOB_06	chr20	18163890	G	A	KAT14	EXONIC-NC	57325:[NR_028402.2(n.1807G>A)]	NA	NA	NA	NA	NA	NA	NA	NA	NA	NA	LOW	NA	NA	121	119	0	205	199	6	2.93

Patel *et al.*, 2021. Whole-exome sequencing analysis of oral squamous cell carcinoma delineated by tobacco usage habits
Supplementary Table 4. List of somatic single nucleotide variants identified using whole exome sequencing of OSCC patients

Cohort	Sample	Chromosome	Genomic position	Reference allele	Alternate allele	Genesymbol	Variant type	Variant description	OncoMD	COSMIC	dbSNP	ExAC	ExAC_SAS	1000G	1000G_SAS	SIFT	Polyphen2	CONDEL	VEP	LRT	PhastCon7Way	Total reads in Normal	Reference read support in Normal	Alternative read support in Normal	Total reads in Tumor	Reference read support in Tumor	Alternate read support in Tumor	Alternate allele frequency in Tumor
Chewer	IOB_06	chr20	21314405	G	A	XRN2	MISSENSE	ENSG00000088930:[ENST00000539513(c.835G>A,Gat>Aat):ENSP00000441113(p.Asp279Asn)]	NA	NA	NA	NA	NA	NA	NA	D	PrD	NA	MODERATE	D	1	333	330	0	375	327	46	12.27
Chewer	IOB_06	chr20	30102396	G	A	HM13	SILENT	ENSG00000101294:[ENST00000335574(c.42G>A,gaG>gaA):ENSP00000335294(p.Glu14(=))]	NA	NA	NA	NA	NA	NA	NA	NA	NA	NA	MODIFIER	NA	NA	69	69	0	56	49	7	12.5
Chewer	IOB_06	chr20	41385246	C	A	PTPRT	MISSENSE	ENSG00000196090:[ENST00000356100(c.715G>T,Gtc>Ttc):ENSP00000348408(p.Val239Phe)]	NA	NA	NA	NA	NA	NA	NA	T	BN	NA	MODERATE	D	1	121	121	0	130	124	5	3.85
Chewer	IOB_06	chr20	56803296	G	C	ANKRD60	SILENT	ENSG00000124227:[ENST00000371167(c.400C>G,ctC>ctG):ENSP00000360209(p.Leu134(=))]	NA	NA	NA	NA	NA	NA	NA	NA	NA	NA	LOW	NA	NA	108	108	0	143	123	20	13.99
Chewer	IOB_06	chr21	9825821	G	A	NA	INTERGENIC	NA	NA	NA	NA	7.72E-04	9.26E-03	NA	NA	NA	NA	NA	MODIFIER	NA	NA	532	525	2	351	342	7	1.99
Chewer	IOB_06	chr21	15579340	C	A	LIPI	INTRONIC	149998:[XM_005260924.1(c.133+3781G>T)]	NA	NA	NA	NA	NA	NA	NA	NA	NA	NA	MODIFIER	NA	NA	28	28	0	39	33	6	15.38
Chewer	IOB_06	chr21	22371039	G	T	NCAM2	INTRONIC	ENSG00000154654:[ENST00000284894(c.55+103G>T)]	NA	NA	NA	NA	NA	NA	NA	NA	NA	NA	MODIFIER	NA	NA	36	36	0	59	54	5	8.47
Chewer	IOB_06	chr21	34969563	C	A	AP000304.12	INTRONIC	ENSG00000249209:[ENST00000429238(c.287-11076G>T)]	NA	NA	chr21*34969563*34969563*C*T*rs375676725*0*0.9996*0.0003994	NA	NA	NA	NA	NA	NA	NA	MODIFIER	NA	NA	164	163	0	126	120	5	3.97
Chewer	IOB_06	chr21	34969563	C	A	CRYZL1	INTRONIC	ENSG00000205758:[ENST00000290244(c.753+23G>T)]	NA	NA	chr21*34969563*34969563*C*T*rs375676725*0*0.9996*0.0003994	NA	NA	NA	NA	NA	NA	NA	MODIFIER	NA	NA	164	163	0	126	120	5	3.97
Chewer	IOB_06	chr21	41450856	G	A	DSCAM	MISSENSE	ENSG00000171587:[ENST00000400454(c.4469C>T,aCa>aTa):ENSP00000383303(p.Thr1490Ile)]	NA	NA	NA	NA	NA	NA	NA	D	PoD	NA	MODERATE	NA	NA	129	129	0	199	179	20	10.05
Chewer	IOB_06	chr21	42775548	C	A	MX2	NCT	ENSG00000183486:[ENST00000481838(n.271+278C>A)]	NA	NA	NA	NA	NA	NA	NA	NA	NA	NA	MODIFIER	NA	NA	120	119	0	122	115	6	4.92
Chewer	IOB_06	chr22	21982944	G	A	YDJC	SILENT	ENSG00000161179:[ENST00000292778(c.735C>T,caC>caT):ENSP00000292778(p.His245(=))]	NA	NA	NA	2.93E-05	0.00E+00	NA	NA	NA	NA	NA	MODIFIER	NA	NA	122	122	0	146	138	8	5.48
Chewer	IOB_06	chr22	22677315	C	T	BMS1P20	EXONIC-NC	96610:[NR_027293.2(n.2002C>T)]	NA	NA	NA	NA	NA	NA	NA	NA	NA	NA	LOW	NA	NA	164	164	0	289	265	24	8.3
Chewer	IOB_06	chr22	22677315	C	T	IGLV1-51	SILENT	ENSG00000211644:[ENST00000390290(c.343C>T,Ctg>Ttg):ENSP00000374825(p.Leu115(=))]	NA	NA	NA	NA	NA	NA	NA	NA	NA	NA	LOW	NA	NA	164	164	0	289	265	24	8.3
Chewer	IOB_06	chr22	26423301	C	A	MYO18B	MISSENSE	ENSG00000133454:[ENST00000543971(c.1210C>A,cCt>cAt):ENSP00000444262(p.Pro404His)]	NA	NA	NA	NA	NA	NA	NA	NA	NA	NA	MODIFIER	NA	NA	201	201	0	240	219	19	7.92
Chewer	IOB_06	chr22	29490406	C	T	KREMEN1	SILENT-SS-PRX	ENSG00000183762:[ENST00000407188(c.252C>T,tgC>tgT):ENSP00000385431(p.Cys84(=))]	NA	NA	NA	NA	NA	NA	NA	NA	NA	NA	LOW	NA	1	30	29	0	30	25	5	16.67
Chewer	IOB_06	chr3	5024763	G	A	BHLHE40	MISSENSE	ENSG00000134107:[ENST00000256495(c.625G>A,Gcc>Acc):ENSP00000256495(p.Ala209Thr)]	NA	NA	3.20E-03	1.41E-03	0.00E+00	3.19E-03	0.00E+00	NA	NA	NA	MODIFIER	NA	NA	207	206	0	230	222	5	2.17
Chewer	IOB_06	chr3	15084335	T	G	MRPS25	EXONIC-NC	64432:[NR_135246.1(n.3682A>C)]	NA	NA	NA	NA	NA	NA	NA	NA	NA	NA	LOW	NA	NA	40	40	0	44	37	7	15.91
Chewer	IOB_06	chr3	15084335	T	G	NR2C2	INTRONIC	ENSG00000177463:[ENST00000439011(c.458+3601T>G)]	NA	NA	NA	NA	NA	NA	NA	NA	NA	NA	LOW	NA	NA	40	40	0	44	37	7	15.91
Chewer	IOB_06	chr3	30880546	G	C	GADL1	MISSENSE	ENSG00000144644:[ENST00000282538(c.846C>G,gaC>gaG):ENSP00000282538(p.Asp282Glu)]	NA	NA	NA	NA	NA	NA	NA	D	PrD	NA	MODERATE	D	0.995	80	80	0	98	80	18	18.37
Chewer	IOB_06	chr3	37560782	C	A	ITGA9	MISSENSE	ENSG00000144668:[ENST00000264741(c.1173C>A,gaC>gaA):ENSP00000264741(p.Asp391Glu)]	NA	NA	NA	NA	NA	NA	NA	T	BN	NA	MODERATE	D	1	125	125	0	152	147	5	3.29
Chewer	IOB_06	chr3	38157970	G	A	ACAA1	NCT	ENSG00000060971:[ENST00000451419(n.299+5785C>T)]	NA	NA	NA	NA	NA	NA	NA	NA	NA	NA	MODIFIER	NA	NA	210	209	0	298	238	59	19.8
Chewer	IOB_06	chr3	38157970	G	A	DLEC1	MISSENSE	ENSG00000008226:[ENST00000308059(c.3883G>A,Gtt>Att):ENSP00000308597(p.Val1295Ile)]	NA	NA	NA	NA	NA	NA	NA	NA	NA	NA	MODIFIER	NA	NA	210	209	0	298	238	59	19.8
Chewer	IOB_06	chr3	38739316	G	A	SCN10A	MISSENSE	ENSG00000185313:[ENST00000449082(c.5395C>T,Cac>Tac):ENSP00000390600(p.His1799Tyr)]	NA	NA	NA	NA	NA	NA	NA	D	PrD	NA	MODERATE	D	1	121	121	0	110	96	14	12.73
Chewer	IOB_06	chr3	43074801	G	A	FAM198A	MISSENSE	ENSG00000144649:[ENST00000273146(c.1046G>A,cGc>cAc):ENSP00000273146(p.Arg349His)]	NA	NA	NA	NA	NA	NA	NA	D	PrD	NA	MODERATE	D	1	164	164	0	219	213	6	2.74
Chewer	IOB_06	chr3	43074801	G	A	KRBOX1	INTRONIC	ENSG00000273291:[ENST00000443313(c.278-20212G>A)]	NA	NA	NA	NA	NA	NA	NA	D	PrD	NA	MODERATE	D	1	164	164	0	219	213	6	2.74
Chewer	IOB_06	chr3	43344823	C	A	SNRK	MISSENSE	ENSG00000163788:[ENST00000296088(c.128C>A,gCa>gAa):ENSP00000296088(p.Ala43Glu)]	NA	NA	NA	NA	NA	NA	NA	D	PrD	NA	MODERATE	D	1	290	288	1	237	229	5	2.11
Chewer	IOB_06	chr3	47147397	A	C	SETD2	INTRONIC	ENSG00000181555:[ENST00000445387(c.3739+90T>G)]	NA	NA	chr3*47147393*47147397*CAAAA*C*rs752575960*NA*NA*NA|chr3*47147393*47147398*CAAAAA*C*rs749834204*NA*NA*NA|chr3*47147393*47147400*CAAAAAA*C*rs778063807*NA*NA*NA	NA	NA	NA	NA	NA	NA	NA	MODIFIER	NA	NA	56	43	0	64	48	6	9.38
Chewer	IOB_06	chr3	47448687	G	C	PTPN23	INTRONIC	25930:[XM_005265031.1(c.1038+11G>C)]	NA	NA	NA	NA	NA	NA	NA	NA	NA	NA	MODIFIER	NA	NA	133	133	0	189	165	23	12.17
Chewer	IOB_06	chr3	51450537	G	A	DCAF1	INTRONIC	9730:[NM_001171904.1(c.3875-17C>T)]	NA	NA	NA	NA	NA	NA	NA	NA	NA	NA	MODIFIER	NA	NA	158	158	0	197	167	28	14.21
Chewer	IOB_06	chr3	51450537	G	A	VPRBP	INTRONIC	ENSG00000145041:[ENST00000335891(c.2690-17C>T)]	NA	NA	NA	NA	NA	NA	NA	NA	NA	NA	MODIFIER	NA	NA	158	158	0	197	167	28	14.21
Chewer	IOB_06	chr3	51454263	C	T	DCAF1	MISSENSE	9730:[NM_001171904.1(c.3419G>A,gGc>gAc):NP_001165375.1(p.Gly1193Asp)]	NA	NA	NA	NA	NA	NA	NA	D	PrD	NA	MODERATE	NA	1	180	180	0	239	231	6	2.51
Chewer	IOB_06	chr3	51454263	C	T	VPRBP	MISSENSE	ENSG00000145041:[ENST00000423656(c.2294G>A,gGc>gAc):ENSP00000393183(p.Gly765Asp)]	NA	NA	NA	NA	NA	NA	NA	D	PrD	NA	MODERATE	NA	1	180	180	0	239	231	6	2.51
Chewer	IOB_06	chr3	53707106	C	A	CACNA1D	SILENT	ENSG00000157388:[ENST00000350061(c.1173C>A,atC>atA):ENSP00000288133(p.Ile391(=))]	NA	NA	NA	NA	NA	NA	NA	NA	NA	NA	MODIFIER	NA	NA	175	174	0	219	211	7	3.2
Chewer	IOB_06	chr3	56114975	G	A	ERC2	MISSENSE	ENSG00000187672:[ENST00000288221(c.1511C>T,tCt>tTt):ENSP00000288221(p.Ser504Phe)]	NA	NA	NA	8.50E-06	0.00E+00	NA	NA	NA	NA	NA	MODIFIER	NA	NA	205	205	0	250	200	47	18.8
Chewer	IOB_06	chr3	113323818	C	A	SIDT1	MISSENSE	ENSG00000072858:[ENST00000264852(c.1399C>A,Cag>Aag):ENSP00000264852(p.Gln467Lys)]	NA	chr3*113323818*113323818*COSM1484459*C*T*1*breast*1	NA	8.24E-06	0.00E+00	NA	NA	NA	NA	NA	MODIFIER	NA	NA	129	129	0	128	123	5	3.91
Chewer	IOB_06	chr3	118865794	G	A	C3orf30	MISSENSE	ENSG00000163424:[ENST00000460150(c.648G>A,aGa>aAa):ENSP00000418207(p.Arg217Lys)]	NA	NA	chr3*118865753*118865801*TGTACAGATTGACAGTGGATCGTCCGTACCATCTGACCAAAGACCTTCC*T*rs765347384*NA*NA*NA	NA	NA	NA	NA	T	PrD	NA	MODERATE	NA	NA	85	83	0	97	84	11	11.34
Chewer	IOB_06	chr3	119222391	C	T	TIMMDC1	MISSENSE	ENSG00000113845:[ENST00000264244(c.373C>T,Cgt>Tgt):ENSP00000264244(p.Arg125Cys)]	NA	chr3*119222391*119222391*COSM1036749*C*T*1*endometrium*1	NA	3.28E-05	7.93E-05	NA	NA	NA	NA	NA	MODIFIER	NA	NA	79	79	0	86	67	18	20.93
Chewer	IOB_06	chr3	137786577	G	T	DZIP1L	INTRONIC	199221:[XM_005247198.1(c.1917-35C>A)]	NA	NA	chr3*137786577*137786577*G*A*rs763485652*NA*NA*NA	NA	NA	NA	NA	NA	NA	NA	MODIFIER	NA	NA	22	22	0	49	41	7	14.29
Chewer	IOB_06	chr3	195692419	C	G	SDHAP1	NCT-SS-PRX	ENSG00000185485:[ENST00000440850(n.1800-6G>C)]	NA	NA	NA	3.56E-01	3.81E-01	NA	NA	NA	NA	NA	LOW	NA	NA	33	33	0	50	41	9	18
Chewer	IOB_06	chr4	1818528	G	A	LETM1	SILENT	ENSG00000168924:[ENST00000302787(c.1857C>T,atC>atT):ENSP00000305653(p.Ile619(=))]	NA	NA	chr4*1818528*1818528*G*C*rs763264804*NA*NA*NA	NA	NA	NA	NA	NA	NA	NA	LOW	NA	0	172	172	0	229	193	36	15.72
Chewer	IOB_06	chr4	8443110	C	A	TRMT44	SILENT	ENSG00000155275:[ENST00000504134(c.422C>A,atC>atA):ENSP00000434207(p.Ile141(=))]	NA	NA	NA	NA	NA	NA	NA	NA	NA	NA	MODIFIER	NA	NA	99	99	0	167	162	5	2.99
Chewer	IOB_06	chr4	62599171	C	A	LPHN3	MISSENSE	ENSG00000150471:[ENST00000504896(c.1094C>A,gCa>gAa):ENSP00000423434(p.Ala365Glu)]	NA	NA	NA	NA	NA	NA	NA	D_lc	PrD	NA	MODERATE	D	1	276	275	0	302	264	38	12.58
Chewer	IOB_06	chr4	83406768	G	A	TMEM150C	MISSENSE	ENSG00000249242:[ENST00000449862(c.646C>T,Cgg>Tgg):ENSP00000403438(p.Arg216Trp)]	NA	NA	NA	2.43E-05	0.00E+00	NA	NA	NA	NA	NA	MODERATE	NA	NA	25	25	0	31	19	11	35.48
Chewer	IOB_06	chr4	90169209	C	T	GPRIN3	MISSENSE	ENSG00000185477:[ENST00000333209(c.2053G>A,Gtg>Atg):ENSP00000328672(p.Val685Met)]	NA	chr4*90169209*90169209*COSM161379*C*T*1*breast*1	chr4*90169209*90169209*C*G*rs763207541*NA*NA*NA	NA	NA	NA	NA	D	PoD	NA	MODERATE	N	0.993	123	122	0	160	145	15	9.38
Chewer	IOB_06	chr4	114386684	C	T	CAMK2D	INTRONIC-SS-DNR	817:[NM_001321585.1(c.563+1G>A)]	NA	chr4*114386684*114386684*COSM347279*C*T*1*lung*1	NA	NA	NA	NA	NA	NA	NA	NA	HIGH	NA	NA	146	146	0	223	196	27	12.11
Chewer	IOB_06	chr4	122682945	G	C	TMEM155	EXONIC-NC	ENSG00000164112:[ENST00000505198(n.339C>G)]	NA	NA	NA	NA	NA	NA	NA	NA	NA	NA	MODIFIER	NA	NA	182	179	0	184	154	28	15.22
Chewer	IOB_06	chr4	122824036	C	G	TRPC3	MISSENSE	ENSG00000138741:[ENST00000513531(c.2050G>C,Gaa>Caa):ENSP00000426899(p.Glu684Gln)]	NA	NA	chr4*122824036*122824036*C*T*rs749012888*NA*NA*NA	NA	NA	NA	NA	T	BN	NA	MODERATE	D	1	149	149	0	156	143	13	8.33
Chewer	IOB_06	chr4	128811066	G	T	PLK4	MISSENSE	ENSG00000142731:[ENST00000514379(c.1382G>T,cGg>cTg):ENSP00000423582(p.Arg461Leu)]	NA	NA	chr4*128811066*128811066*G*A*rs766659674*NA*NA*NA	NA	NA	NA	NA	T	BN	NA	MODERATE	N	0.844	176	176	0	155	142	13	8.39
Chewer	IOB_06	chr4	145974932	G	A	ANAPC10	MISSENSE-SS-PRX	ENSG00000164162:[ENST00000510270(n.482+10792C>T)]	NA	NA	NA	NA	NA	NA	NA	NA	NA	NA	MODIFIER	NA	NA	37	37	0	33	21	11	33.33
Chewer	IOB_06	chr4	154216662	C	A	TRIM2	MISSENSE	ENSG00000109654:[ENST00000437508(c.903C>A,ttC>ttA):ENSP00000415812(p.Phe301Leu)]	NA	NA	NA	NA	NA	NA	NA	NA	NA	NA	MODIFIER	NA	NA	201	200	0	250	243	5	2
Chewer	IOB_06	chr5	39270698	C	T	FYB	INTRONIC	ENSG00000082074:[ENST00000510188(c.-28+3807G>A)]	NA	NA	NA	NA	NA	NA	NA	NA	NA	NA	MODIFIER	NA	NA	180	180	0	193	166	27	13.99
Chewer	IOB_06	chr5	39383080	C	T	C9	NCT	ENSG00000113600:[ENST00000483232(n.119+5821G>A)]	NA	chr5*39383080*39383080*COSM187556*C*T*1*large_intestine*1	NA	3.30E-05	0.00E+00	NA	NA	NA	NA	NA	LOW	NA	NA	113	113	0	142	115	26	18.31
Chewer	IOB_06	chr5	39383080	C	T	DAB2	SILENT	ENSG00000153071:[ENST00000320816(c.981G>A,tcG>tcA):ENSP00000313391(p.Ser327(=))]	NA	chr5*39383080*39383080*COSM187556*C*T*1*large_intestine*1	NA	3.30E-05	0.00E+00	NA	NA	NA	NA	NA	LOW	NA	NA	113	113	0	142	115	26	18.31
Chewer	IOB_06	chr5	71528331	G	C	MRPS27	MISSENSE	ENSG00000113048:[ENST00000457646(c.362C>G,tCc>tGc):ENSP00000428120(p.Ser121Cys)]	NA	NA	NA	NA	NA	NA	NA	D	PrD	NA	MODERATE	D	1	144	144	0	151	133	18	11.92
Chewer	IOB_06	chr5	82817870	G	C	VCAN	MISSENSE	ENSG00000038427:[ENST00000512590(c.3601G>C,Gta>Cta):ENSP00000425959(p.Val1201Leu)]	NA	NA	NA	NA	NA	NA	NA	T	BN	NA	MODERATE	N	0.839	143	143	0	174	144	29	16.67
Chewer	IOB_06	chr5	96472760	C	T	CTD-2215E18.1	INTRONIC	ENSG00000251606:[ENST00000509481(c.32+33962C>T)]	NA	NA	NA	NA	NA	NA	NA	NA	NA	NA	MODIFIER	NA	NA	59	58	0	67	60	7	10.45
Chewer	IOB_06	chr5	96472760	C	T	LIX1	INTRONIC	ENSG00000145721:[ENST00000274382(c.82+5439G>A)]	NA	NA	NA	NA	NA	NA	NA	NA	NA	NA	MODIFIER	NA	NA	59	58	0	67	60	7	10.45
Chewer	IOB_06	chr5	102513380	G	A	PPIP5K2	MISSENSE	ENSG00000145725:[ENST00000509597(c.364G>A,Gaa>Aaa):ENSP00000424948(p.Glu122Lys)]	NA	NA	NA	NA	NA	NA	NA	NA	NA	NA	MODERATE	NA	1	38	37	0	41	36	5	12.2
Chewer	IOB_06	chr5	111595633	C	A	EPB41L4A	MISSENSE	ENSG00000129595:[ENST00000261486(c.686G>T,gGt>gTt):ENSP00000261486(p.Gly229Val)]	NA	chr5*111595633*111595633*COSM1541298*C*A*1*lung*1	NA	NA	NA	NA	NA	NA	NA	NA	MODIFIER	NA	NA	347	347	0	403	341	62	15.38
Chewer	IOB_06	chr5	120021725	C	T	PRR16	MISSENSE	ENSG00000184838:[ENST00000446965(c.26C>T,aCg>aTg):ENSP00000405491(p.Thr9Met)]	NA	chr5*120021725*120021725*COSM287264*C*T*1*large_intestine*1	2.00E-04	5.77E-05	0.00E+00	2.00E-04	0.00E+00	NA	NA	NA	MODERATE	NA	1	71	71	0	112	103	8	7.14
Chewer	IOB_06	chr5	128983559	G	A	ADAMTS19	SILENT	ENSG00000145808:[ENST00000274487(c.1956G>A,ggG>ggA):ENSP00000274487(p.Gly652(=))]	NA	NA	NA	NA	NA	NA	NA	NA	NA	NA	MODIFIER	NA	NA	46	46	0	55	47	8	14.55
Chewer	IOB_06	chr5	128983559	G	A	CTC-575N7.1	NCT	ENSG00000251680:[ENST00000503616(n.404+87077C>T)]	NA	NA	NA	NA	NA	NA	NA	NA	NA	NA	MODIFIER	NA	NA	46	46	0	55	47	8	14.55
Chewer	IOB_06	chr5	133984862	C	A	SAR1B	5UTR	ENSG00000152700:[ENST00000507419(c.-1274G>T)]	NA	NA	NA	NA	NA	NA	NA	T_lc	BN	NA	MODERATE	N	0.999	180	180	0	172	167	5	2.91

Patel *et al.* , 2021. Whole-exome sequencing analysis of oral squamous cell carcinoma delineated by tobacco usage habits
Supplementary Table 4. List of somatic single nucleotide variants identified using whole exome sequencing of OSCC patients

Cohort	Sample	Chromosome	Genomic position	Reference allele	Alternate allele	Genesymbol	Variant type	Variant description	OncoMD	COSMIC	dbSNP	ExAC	ExAC_SAS	1000G	1000G_SAS	SIFT	Polyphen2	CONDEL	VEP	LRT	PhastCon7Way	Total reads in Normal	Reference read support in Normal	Alternative read support in Normal	Total reads in Tumor	Reference read support in Tumor	Alternate read support in Tumor	Alternate allele frequency in Tumor
Chewer	IOB_06	chr5	133984862	C	A	SEC24A	MISSENSE-SS-PRX	10802:[XM_005271853.1(c.-602C>A)]	NA	NA	NA	NA	NA	NA	NA	T_lc	BN	NA	MODERATE	N	0.999	180	180	0	172	167	5	2.91
Chewer	IOB_06	chr5	151234690	G	A	GLRA1	MISSENSE	ENSG00000145888:[ENST00000274576(c.608C>T,gCc>gTc):ENSP00000274576(p.Ala203Val)]	NA	NA	chr5*151234690*151234690*G*T*rs980887652*NA*NA*NA	NA	NA	NA	NA	D	PoD	NA	MODERATE	D	1	43	43	0	47	42	5	10.64
Chewer	IOB_06	chr5	158141151	G	A	EBF1	SILENT	ENSG00000164330:[ENST00000313708(c.1165C>T,Ctg>Ttg):ENSP00000322898(p.Leu389(=))]	NA	NA	NA	NA	NA	NA	NA	NA	NA	NA	MODIFIER	NA	NA	74	74	0	134	120	13	9.7
Chewer	IOB_06	chr5	169472892	C	A	DOCK2	MISSENSE	ENSG00000134516:[ENST00000540750(c.1132C>A,Ctg>Atg):ENSP00000438827(p.Leu378Met)]	NA	NA	NA	NA	NA	NA	NA	NA	NA	NA	MODIFIER	NA	NA	91	91	0	136	130	6	4.41
Chewer	IOB_06	chr5	176023998	G	A	GPRIN1	SILENT	ENSG00000169258:[ENST00000303991(c.2838C>T,atC>atT):ENSP00000305839(p.Ile946(=))]	NA	NA	NA	NA	NA	NA	NA	NA	NA	NA	LOW	NA	NA	161	161	0	179	164	15	8.38
Chewer	IOB_06	chr5	176919521	G	A	PDLIM7	NCT-SS-PRX	ENSG00000196923:[ENST00000503346(n.445+6C>T)]	NA	NA	NA	1.69E-05	0.00E+00	NA	NA	NA	NA	NA	LOW	NA	NA	123	122	0	207	179	27	13.04
Chewer	IOB_06	chr6	30570673	C	A	PPP1R10	MISSENSE	ENSG00000204569:[ENST00000376511(c.1907G>T,gGc>gTc):ENSP00000365694(p.Gly636Val)]	NA	NA	NA	NA	NA	NA	NA	T	BN	NA	MODERATE	D	1	66	66	0	120	115	5	4.17
Chewer	IOB_06	chr6	31931927	G	A	SKIV2L	INTRONIC	ENSG00000204351:[ENST00000544581(c.1281+25G>A)]	NA	NA	NA	1.79E-05	6.27E-05	NA	NA	NA	NA	NA	MODIFIER	NA	NA	177	177	0	255	241	12	4.71
Chewer	IOB_06	chr6	41001791	G	A	OARD1	3UTR	ENSG00000124596:[ENST00000482853(c.*588C>T)]	NA	NA	NA	NA	NA	NA	NA	D	PrD	NA	MODERATE	D	1	155	154	0	227	199	28	12.33
Chewer	IOB_06	chr6	41001791	G	A	UNC5CL	MISSENSE	ENSG00000124602:[ENST00000244565(c.515C>T,tCc>tTc):ENSP00000244565(p.Ser172Phe)]	NA	NA	NA	NA	NA	NA	NA	D	PrD	NA	MODERATE	D	1	155	154	0	227	199	28	12.33
Chewer	IOB_06	chr6	62604647	C	A	KHDRBS2	MISSENSE	ENSG00000112232:[ENST00000281156(c.703G>T,Gtg>Ttg):ENSP00000281156(p.Val235Leu)]	NA	NA	NA	NA	NA	NA	NA	T	BN	NA	MODERATE	N	1	73	73	0	109	95	14	12.84
Chewer	IOB_06	chr6	74079509	C	A	OOEP	MISSENSE	ENSG00000203907:[ENST00000370359(c.7G>T,Gat>Tat):ENSP00000359384(p.Asp3Tyr)]	NA	NA	chr6*74079509*74079509*C*T*rs369027216*NA*NA*NA	NA	NA	NA	NA	NA	NA	NA	MODIFIER	NA	NA	233	233	0	211	201	10	4.74
Chewer	IOB_06	chr6	74079509	C	A	OOEP-AS1	EXONIC-NC	ENSG00000231332:[ENST00000445350(n.83C>A)]	NA	NA	chr6*74079509*74079509*C*T*rs369027216*NA*NA*NA	NA	NA	NA	NA	NA	NA	NA	MODIFIER	NA	NA	233	233	0	211	201	10	4.74
Chewer	IOB_06	chr6	87966075	C	A	ZNF292	MISSENSE	ENSG00000188994:[ENST00000369577(c.2728C>A,Ctc>Atc):ENSP00000358590(p.Leu910Ile)]	NA	NA	chr6*87966075*87966075*C*T*rs777620610*NA*NA*NA	NA	NA	NA	NA	NA	NA	NA	MODERATE	NA	0.001	164	163	0	195	190	5	2.56
Chewer	IOB_06	chr6	90382029	A	T	LOC101929057	NCT	101929057:[XR_241876.1(n.121-3086A>T)]	NA	chr6*90382029*90382029*COSM1082498*A*C*1*endometrium*1	NA	NA	NA	NA	NA	NA	NA	NA	MODIFIER	NA	NA	89	89	0	126	103	23	18.25
Chewer	IOB_06	chr6	90382029	A	T	MDN1	MISSENSE	ENSG00000112159:[ENST00000369393(c.13684T>A,Ttc>Atc):ENSP00000358400(p.Phe4562Ile)]	NA	chr6*90382029*90382029*COSM1082498*A*C*1*endometrium*1	NA	NA	NA	NA	NA	NA	NA	NA	MODIFIER	NA	NA	89	89	0	126	103	23	18.25
Chewer	IOB_06	chr6	99365331	C	A	FBXL4	MISSENSE	ENSG00000112234:[ENST00000229971(c.777G>T,atG>atT):ENSP00000229971(p.Met259Ile)]	NA	NA	NA	NA	NA	NA	NA	NA	NA	NA	MODIFIER	NA	NA	306	306	0	332	325	5	1.51
Chewer	IOB_06	chr6	116442794	C	T	AL121963.1	INTRONIC	ENSG00000234188:[ENST00000430695(c.215-40C>T)]	NA	NA	NA	NA	NA	NA	NA	D	PrD	NA	MODERATE	NA	NA	119	119	0	150	135	14	9.33
Chewer	IOB_06	chr6	116442794	C	T	COL10A1	MISSENSE	ENSG00000123500:[ENST00000243222(c.485G>A,gGa>gAa):ENSP00000243222(p.Gly162Glu)]	NA	NA	NA	NA	NA	NA	NA	D	PrD	NA	MODERATE	NA	NA	119	119	0	150	135	14	9.33
Chewer	IOB_06	chr6	116442794	C	T	NT5DC1	INTRONIC	ENSG00000178425:[ENST00000460749(c.27+3686C>T)]	NA	NA	NA	NA	NA	NA	NA	D	PrD	NA	MODERATE	NA	NA	119	119	0	150	135	14	9.33
Chewer	IOB_06	chr6	138574962	C	T	KIAA1244	INTRONIC	ENSG00000112379:[ENST00000251691(c.666-55C>T)]	NA	NA	NA	NA	NA	NA	NA	NA	NA	NA	MODIFIER	NA	NA	88	88	0	89	77	12	13.48
Chewer	IOB_06	chr6	142396786	C	G	NMBR	STOPLOSS	ENSG00000135577:[ENST00000258042(c.1172G>C,tGa>tCa):ENSP00000258042(p.Ter391SerextTer?)]	NA	NA	chr6*142396786*142396786*C*T*rs751217987*NA*NA*NA	NA	NA	NA	NA	NA	NA	NA	HIGH	NA	NA	142	141	0	163	147	16	9.82
Chewer	IOB_06	chr6	142409574	C	T	NMBR	MISSENSE	ENSG00000135577:[ENST00000258042(c.222G>A,atG>atA):ENSP00000258042(p.Met74Ile)]	NA	NA	NA	NA	NA	NA	NA	NA	NA	NA	MODIFIER	NA	NA	298	296	1	366	296	69	18.85
Chewer	IOB_06	chr6	142409574	C	T	RP11-137J7.2	NCT	ENSG00000236822:[ENST00000454401(n.142+63C>T)]	NA	NA	NA	NA	NA	NA	NA	NA	NA	NA	MODIFIER	NA	NA	298	296	1	366	296	69	18.85
Chewer	IOB_06	chr7	1784419	G	A	ELFN1	MISSENSE	ENSG00000225968:[ENST00000424383(c.187G>A,Gtg>Atg):ENSP00000456548(p.Val63Met)]	NA	NA	NA	NA	NA	NA	NA	T	PrD	NA	MODERATE	NA	NA	435	434	0	533	506	24	4.5
Chewer	IOB_06	chr7	20198690	C	T	MACC1	MISSENSE	ENSG00000183742:[ENST00000332878(c.1294G>A,Gat>Aat):ENSP00000328410(p.Asp432Asn)]	NA	NA	NA	NA	NA	NA	NA	T	BN	NA	MODERATE	N	1	175	174	1	196	179	17	8.67
Chewer	IOB_06	chr7	47942104	C	A	HUS1	INTRONIC	ENSG00000136273:[ENST00000436444(c.*1170+61679G>T)]	NA	NA	NA	NA	NA	NA	NA	NA	NA	NA	MODIFIER	NA	NA	161	160	0	179	174	5	2.79
Chewer	IOB_06	chr7	47942104	C	A	PKD1L1	NONSENSE	ENSG00000158683:[ENST00000289672(c.1936G>T,Gga>Tga):ENSP00000289672(p.Gly646Ter)]	NA	NA	NA	NA	NA	NA	NA	NA	NA	NA	MODIFIER	NA	NA	161	160	0	179	174	5	2.79
Chewer	IOB_06	chr7	72848705	C	T	FZD9	MISSENSE	ENSG00000188763:[ENST00000344575(c.368C>T,gCg>gTg):ENSP00000345785(p.Ala123Val)]	NA	NA	NA	NA	NA	NA	NA	T	BN	NA	MODERATE	U	1	486	482	4	618	533	79	12.78
Chewer	IOB_06	chr7	87913131	G	T	AC003991.3	NCT	ENSG00000228113:[ENST00000595121(n.546-16G>T)]	NA	NA	NA	NA	NA	NA	NA	NA	NA	NA	MODIFIER	NA	NA	107	106	0	89	73	16	17.98
Chewer	IOB_06	chr7	87913131	G	T	STEAP4	MISSENSE-SS-PRX	ENSG00000127954:[ENST00000301959(c.454C>A,Cag>Aag):ENSP00000305545(p.Gln152Lys)]	NA	NA	NA	NA	NA	NA	NA	NA	NA	NA	MODIFIER	NA	NA	107	106	0	89	73	16	17.98
Chewer	IOB_06	chr7	93073015	A	G	CALCR	MISSENSE	ENSG00000004948:[ENST00000360249(c.751T>C,Ttc>Ctc):ENSP00000353385(p.Phe251Leu)]	NA	NA	NA	NA	NA	NA	NA	D	PrD	NA	MODERATE	NA	NA	174	173	0	208	176	32	15.38
Chewer	IOB_06	chr7	98506564	C	A	TRRAP	MISSENSE	ENSG00000196367:[ENST00000355540(c.1329C>A,gaC>gaA):ENSP00000347733(p.Asp443Glu)]	NA	NA	chr7*98506564*98506564*C*T*rs377467365*NA*NA*NA	NA	NA	NA	NA	T	BN	NA	MODERATE	NA	NA	120	120	0	190	183	5	2.63
Chewer	IOB_06	chr7	99021461	C	T	ATP5J2-PTCD1	SILENT	ENSG00000248919:[ENST00000413834(c.2004G>A,ccG>ccA):ENSP00000400168(p.Pro668(=))]	NA	NA	NA	1.65E-05	0.00E+00	NA	NA	NA	NA	NA	LOW	NA	NA	79	78	0	98	83	15	15.31
Chewer	IOB_06	chr7	99021461	C	T	PTCD1	SILENT	ENSG00000106246:[ENST00000292478(c.1857G>A,ccG>ccA):ENSP00000292478(p.Pro619(=))]	NA	NA	NA	1.65E-05	0.00E+00	NA	NA	NA	NA	NA	LOW	NA	NA	79	78	0	98	83	15	15.31
Chewer	IOB_06	chr7	99022700	G	A	ATP5J2-PTCD1	SILENT	ENSG00000248919:[ENST00000413834(c.1602C>T,ccC>ccT):ENSP00000400168(p.Pro534(=))]	NA	NA	2.00E-04	2.23E-04	1.45E-03	2.00E-04	1.00E-03	NA	NA	NA	LOW	NA	NA	121	121	0	143	116	27	18.88
Chewer	IOB_06	chr7	99022700	G	A	PTCD1	SILENT	ENSG00000106246:[ENST00000555673(c.1602C>T,ccC>ccT):ENSP00000450995(p.Pro534(=))]	NA	NA	2.00E-04	2.23E-04	1.45E-03	2.00E-04	1.00E-03	NA	NA	NA	LOW	NA	NA	121	121	0	143	116	27	18.88
Chewer	IOB_06	chr7	121726132	C	T	AASS	SILENT	ENSG00000008311:[ENST00000358954(c.2118G>A,acG>acA):ENSP00000351834(p.Thr706(=))]	NA	NA	2.00E-04	4.13E-05	0.00E+00	2.00E-04	0.00E+00	NA	NA	NA	LOW	NA	NA	167	166	0	192	157	35	18.23
Chewer	IOB_06	chr7	123671871	G	C	TMEM229A	3UTR	ENSG00000234224:[ENST00000455783(c.*44C>G)]	NA	NA	NA	NA	NA	NA	NA	NA	NA	NA	MODIFIER	NA	NA	70	69	0	93	83	9	9.68
Chewer	IOB_06	chr7	142008554	A	G	TRBV3-1	INTRONIC	ENSG00000237702:[ENST00000390387(c.50-23A>G)]	NA	NA	NA	NA	NA	NA	NA	NA	NA	NA	MODIFIER	NA	NA	92	91	0	111	91	19	17.12
Chewer	IOB_06	chr7	142641490	G	A	KEL	INTRONIC	3792:[XM_005249994.1(c.487-3C>T)]	NA	NA	chr7*142641490*142641490*G*T*rs748507981*NA*NA*NA	NA	NA	NA	NA	NA	NA	NA	LOW	NA	NA	78	78	0	135	114	21	15.56
Chewer	IOB_06	chr7	149489618	C	A	SSPO	NCT	ENSG00000197558:[ENST00000262089(n.6092+49C>A)]	NA	NA	chr7*149489618*149489618*C*T*rs760893607*NA*NA*NA	NA	NA	NA	NA	NA	NA	NA	MODIFIER	NA	NA	135	135	0	254	247	5	1.97
Chewer	IOB_06	chr7	149500869	C	T	SSPO	SILENT	23145:[NM_198455.2(c.8187C>T,cgC>cgT):NP_940857.2(p.Arg2729(=))]	NA	NA	NA	NA	NA	NA	NA	NA	NA	NA	MODIFIER	NA	NA	57	57	0	71	66	5	7.04
Chewer	IOB_06	chr7	154738463	C	T	PAXIP1	MISSENSE	ENSG00000157212:[ENST00000397192(c.2983G>A,Gta>Ata):ENSP00000380376(p.Val995Ile)]	NA	NA	NA	9.63E-06	0.00E+00	NA	NA	NA	NA	NA	MODIFIER	NA	NA	87	87	0	96	81	15	15.62
Chewer	IOB_06	chr7	154738463	C	T	PAXIP1-AS2	EXONIC-NC	100132707:[NR_024476.1(n.1209C>T)]	NA	NA	NA	9.63E-06	0.00E+00	NA	NA	NA	NA	NA	MODIFIER	NA	NA	87	87	0	96	81	15	15.62
**Chewer**	**IOB_06**	**chr8**	**2815273**	**C**	**T**	**CSMD1**	**SILENT**	ENSG00000183117:[ENST00000542608(c.9228G>A,acG>acA):ENSP00000446243(p.Thr3076(=))]	NA	chr8*2815273*2815273*COSM1098871*C*T*1*endometrium*1|chr8*2815273*2815273*COSM1098872*C*T*1*endometrium*1	chr8*2815273*2815273*C*A,T*rs745758896*NA*NA*NA	8.23E-05	1.07E-04	NA	NA	NA	NA	NA	LOW	NA	NA	80	80	0	97	80	17	17.53
Chewer	IOB_06	chr8	12956863	A	C	DLC1	MISSENSE	ENSG00000164741:[ENST00000520226(c.1450T>G,Tcc>Gcc):ENSP00000428028(p.Ser484Ala)]	NA	NA	chr8*12956863*12956863*A*G*rs898453011*NA*NA*NA	NA	NA	NA	NA	T	BN	NA	MODERATE	D	1	160	160	0	156	132	24	15.38
Chewer	IOB_06	chr8	21958231	G	A	FAM160B2	MISSENSE	ENSG00000158863:[ENST00000289921(c.1468G>A,Gag>Aag):ENSP00000289921(p.Glu490Lys)]	NA	NA	NA	NA	NA	NA	NA	NA	NA	NA	MODIFIER	NA	NA	204	203	0	376	346	30	7.98
Chewer	IOB_06	chr8	27321003	C	T	CHRNA2	SILENT	ENSG00000120903:[ENST00000407991(c.957G>A,ccG>ccA):ENSP00000385026(p.Pro319(=))]	NA	chr8*27321003*27321003*COSM1456389*C*T*1*large intestine*1	NA	1.65E-05	0.00E+00	NA	NA	NA	NA	NA	LOW	NA	NA	167	165	0	208	181	24	11.54
Chewer	IOB_06	chr8	27358467	G	T	EPHX2	MISSENSE	ENSG00000120915:[ENST00000517536(c.126G>T,caG>caT):ENSP00000428875(p.Gln42His)]	NA	NA	NA	NA	NA	NA	NA	T	BN	NA	MODERATE	NA	NA	128	126	2	147	124	23	15.65
Chewer	IOB_06	chr8	67514729	G	A	MYBL1	MISSENSE	ENSG00000185697:[ENST00000517885(c.50C>T,gCc>gTc):ENSP00000428265(p.Ala17Val)]	NA	NA	NA	NA	NA	NA	NA	T	BN	NA	MODERATE	N	1	128	127	0	165	159	5	3.03
Chewer	IOB_06	chr8	100791239	G	A	VPS13B	MISSENSE	ENSG00000132549:[ENST00000358544(c.7834G>A,Gca>Aca):ENSP00000351346(p.Ala2612Thr)]	NA	NA	NA	NA	NA	NA	NA	NA	NA	NA	MODERATE	NA	1	112	111	0	139	125	14	10.07
Chewer	IOB_06	chr8	145107040	C	A	OPLAH	NCT	ENSG00000178814:[ENST00000534424(n.3565+32G>T)]	NA	NA	NA	NA	NA	NA	NA	NA	NA	NA	MODIFIER	NA	NA	174	172	1	274	267	5	1.82
Chewer	IOB_06	chr8	145737836	C	T	RECQL4	NONSENSE	ENSG00000160957:[ENST00000534626(c.1165G>A,tgG>tgA):ENSP00000477457(p.Trp389Ter)]	NA	NA	NA	NA	NA	NA	NA	NA	NA	NA	HIGH	NA	NA	197	197	0	251	218	32	12.75
Chewer	IOB_06	chr8	146203374	C	A	ZNF252P	EXONIC-NC	ENSG00000196922:[ENST00000427606(n.2250G>T)]	NA	NA	NA	NA	NA	NA	NA	NA	NA	NA	MODIFIER	NA	NA	171	171	0	219	212	5	2.28
Chewer	IOB_06	chr9	26997942	A	G	IFT74	INTRONIC	ENSG00000096872:[ENST00000380062(c.587+7749A>G)]	NA	NA	NA	NA	NA	NA	NA	T	BN	NA	MODERATE	N	0.068	57	57	0	65	60	5	7.69
Chewer	IOB_06	chr9	26997942	A	G	LRRC19	MISSENSE	ENSG00000184434:[ENST00000380055(c.379T>C,Tat>Cat):ENSP00000369395(p.Tyr127His)]	NA	NA	NA	NA	NA	NA	NA	T	BN	NA	MODERATE	N	0.068	57	57	0	65	60	5	7.69
Chewer	IOB_06	chr9	38596091	C	A	ANKRD18A	NONSENSE	ENSG00000180071:[ENST00000399703(c.1246G>T,Gaa>Taa):ENSP00000382610(p.Glu416Ter)]	NA	NA	NA	NA	NA	NA	NA	NA	NA	NA	HIGH	NA	NA	229	228	0	212	180	32	15.09
Chewer	IOB_06	chr9	97063596	C	G	ZNF169	MISSENSE	ENSG00000175787:[ENST00000395395(c.1756C>G,Cat>Gat):ENSP00000378792(p.His586Asp)]	NA	NA	NA	NA	NA	NA	NA	T	BN	NA	MODERATE	NA	NA	130	130	0	123	117	6	4.88
Chewer	IOB_06	chr9	101778351	G	A	COL15A1	MISSENSE	ENSG00000204291:[ENST00000375001(c.1597G>A,Ggg>Agg):ENSP00000364140(p.Gly533Arg)]	NA	NA	NA	NA	NA	NA	NA	T_lc	BN	NA	MODERATE	N	0.077	80	80	0	115	109	6	5.22
Chewer	IOB_06	chr9	104385548	G	A	GRIN3A	INTRONIC	ENSG00000198785:[ENST00000361820(c.2614+52C>T)]	NA	NA	NA	NA	NA	NA	NA	NA	NA	NA	MODIFIER	NA	NA	185	185	0	233	227	6	2.58
Chewer	IOB_06	chr9	114484868	G	A	C9orf84	INTRONIC	158401:[XM_005251738.1(c.1955-3C>T)]	NA	NA	NA	NA	NA	NA	NA	NA	NA	NA	LOW	NA	NA	106	104	0	111	105	5	4.5
Chewer	IOB_06	chr9	115962126	C	T	FKBP15	MISSENSE	ENSG00000119321:[ENST00000238256(c.616G>A,Ggc>Agc):ENSP00000238256(p.Gly206Ser)]	NA	NA	2.00E-04	1.08E-04	7.22E-05	2.00E-04	0.00E+00	T	BN	NA	MODERATE	NA	NA	40	40	0	59	52	6	10.17
Chewer	IOB_06	chr9	125330207	G	T	OR1L8	MISSENSE	ENSG00000171496:[ENST00000304865(c.550C>A,Cct>Act):ENSP00000306607(p.Pro184Thr)]	NA	chr9*125330207*125330207*COSM1104853*G*A*1*endometrium*1|chr9*125330207*125330207*COSM1104854*G*A*1*endometrium*1	NA	NA	NA	NA	NA	D	PrD	NA	MODERATE	N	0.001	98	98	0	117	112	5	4.27
Chewer	IOB_06	chr9	125797706	C	T	GPR21	SILENT	ENSG00000188394:[ENST00000373642(c.861C>T,ttC>ttT):ENSP00000362746(p.Phe287(=))]	NA	NA	NA	NA	NA	NA	NA	NA	NA	NA	MODIFIER	NA	NA	133	133	0	158	144	14	8.86
Chewer	IOB_06	chr9	125797706	C	T	RABGAP1	INTRONIC	ENSG00000011454:[ENST00000456584(c.1590+14968C>T)]	NA	NA	NA	NA	NA	NA	NA	NA	NA	NA	MODIFIER	NA	NA	133	133	0	158	144	14	8.86

Patel *et al.* , 2021. Whole-exome sequencing analysis of oral squamous cell carcinoma delineated by tobacco usage habits
Supplementary Table 4. List of somatic single nucleotide variants identified using whole exome sequencing of OSCC patients

Cohort	Sample	Chromosome	Genomic position	Reference allele	Alternate allele	Genesymbol	Variant type	Variant description	OncoMD	COSMIC	dbSNP	ExAC	ExAC_SAS	1000G	1000G_SAS	SIFT	Polyphen2	CONDEL	VEP	LRT	PhastCon7Way	Total reads in Normal	Reference read support in Normal	Alternative read support in Normal	Total reads in Tumor	Reference read support in Tumor	Alternate read support in Tumor	Alternate allele frequency in Tumor
Chewer	IOB_06	chr9	131871507	G	A	CRAT	MISSENSE	ENSG00000095321:[ENST00000441796(c.77C>T,tCc>tTc):ENSP00000404405(p.Ser26Phe)]	NA	NA	NA	NA	NA	NA	NA	NA	NA	NA	MODIFIER	NA	NA	53	53	0	68	50	17	25
Chewer	IOB_06	chr9	134008570	C	A	NUP214	MISSENSE	ENSG00000126883:[ENST00000359428(c.805C>A,Cca>Aca):ENSP00000352400(p.Pro269Thr)]	NA	NA	NA	NA	NA	NA	NA	NA	NA	NA	MODIFIER	NA	NA	98	98	0	112	89	22	19.64
Chewer	IOB_06	chr9	134008570	C	A	RP11-544A12.4	EXONIC-NC	ENSG00000236986:[ENST00000588378(n.664G>T)]	NA	NA	NA	NA	NA	NA	NA	NA	NA	NA	MODIFIER	NA	NA	98	98	0	112	89	22	19.64
Chewer	IOB_06	chr9	139409936	T	A	NOTCH1	SILENT-SS-PRX	ENSG00000148400:[ENST00000277541(c.1902A>T,acA>acT):ENSP00000277541(p.Thr634(=))]	NA	NA	NA	NA	NA	NA	NA	NA	NA	NA	LOW	NA	NA	183	183	0	268	236	30	11.19
Chewer	IOB_06	chrX	18775760	T	A	PPEF1	MISSENSE	ENSG00000086717:[ENST00000349874(c.412T>A,Tat>Aat):ENSP00000341892(p.Tyr138Asn)]	NA	NA	NA	NA	NA	NA	NA	D	PrD	NA	MODERATE	NA	NA	226	225	0	237	230	6	2.53
Chewer	IOB_06	chrX	19587158	A	C	SH3KBP1	INTRONIC	30011:[XM_005274495.1(c.1348+63T>G)]	NA	NA	NA	NA	NA	NA	NA	NA	NA	NA	MODIFIER	NA	NA	107	107	0	116	105	11	9.48
Chewer	IOB_06	chrX	19984670	C	T	CXorf23	MISSENSE	ENSG00000173681:[ENST00000356980(c.139G>A,Gtc>Atc):ENSP00000349470(p.Val47Ile)]	NA	NA	NA	1.15E-05	0.00E+00	NA	NA	T	BN	NA	MODERATE	NA	NA	135	135	0	159	140	19	11.95
Chewer	IOB_06	chrX	37586968	C	T	TM4SF2	INTRONIC	ENSG00000250349:[ENST00000465127(c.171+301715C>T)]	NA	NA	NA	NA	NA	NA	NA	NA	NA	NA	LOW	NA	0.002	102	102	0	107	91	15	14.02
Chewer	IOB_06	chrX	37586968	C	T	XK	SILENT	ENSG00000047597:[ENST00000378616(c.588C>T,taC>taT):ENSP00000367879(p.Tyr196(=))]	NA	NA	NA	NA	NA	NA	NA	NA	NA	NA	LOW	NA	0.002	102	102	0	107	91	15	14.02
Chewer	IOB_06	chrX	48560278	C	A	AF196970.3	NCT	ENSG00000232828:[ENST00000416061(n.126-1182G>T)]	NA	NA	NA	NA	NA	NA	NA	NA	NA	NA	MODIFIER	NA	NA	63	63	0	68	63	5	7.35
Chewer	IOB_06	chrX	48560278	C	A	SUV39H1	INTRONIC	ENSG00000101945:[ENST00000376687(c.828+1134C>A)]	NA	NA	NA	NA	NA	NA	NA	NA	NA	NA	MODIFIER	NA	NA	63	63	0	68	63	5	7.35
Chewer	IOB_06	chrX	48564698	G	A	AF196970.3	NCT	ENSG00000232828:[ENST00000416061(n.126-5602C>T)]	NA	NA	NA	NA	NA	NA	NA	NA	NA	NA	LOW	NA	1	119	119	0	149	144	5	3.36
Chewer	IOB_06	chrX	48564698	G	A	SUV39H1	MISSENSE	ENSG00000101945:[ENST00000376687(c.871G>A,Gac>Aac):ENSP00000365877(p.Asp291Asn)]	NA	NA	NA	NA	NA	NA	NA	NA	NA	NA	LOW	NA	1	119	119	0	149	144	5	3.36
Chewer	IOB_06	chrX	48666434	G	T	HDAC6	INTRONIC-SS-ACR-PRX	ENSG00000094631:[ENST00000444343(c.675-6G>T)]	NA	NA	NA	NA	NA	NA	NA	NA	NA	NA	LOW	NA	NA	50	50	0	79	72	7	8.86
Chewer	IOB_06	chrX	55284898	C	T	PAGE3	EXONIC-NC	139793:[NR_033460.1(n.578G>A)]	NA	NA	NA	NA	NA	NA	NA	NA	NA	NA	MODIFIER	NA	NA	82	82	0	61	46	14	22.95
Chewer	IOB_06	chrX	70320556	C	G	FOXO4	MISSENSE	ENSG00000184481:[ENST00000374259(c.476C>G,tCc>tGc):ENSP00000363377(p.Ser159Cys)]	NA	NA	NA	NA	NA	NA	NA	NA	NA	NA	MODIFIER	NA	NA	100	100	0	118	110	6	5.08
Chewer	IOB_06	chrX	99551436	G	A	PCDH19	NONSENSE	ENSG00000165194:[ENST00000255531(c.3145C>T,Cag>Tag):ENSP00000255531(p.Gln1049Ter)]	NA	NA	NA	NA	NA	NA	NA	NA	NA	NA	HIGH	NA	1	89	89	0	141	132	8	5.67
Chewer	IOB_06	chrX	103041071	C	A	PLP1	NCT	ENSG00000123560:[ENST00000464776(n.456-323C>A)]	NA	NA	NA	NA	NA	NA	NA	NA	NA	NA	MODIFIER	NA	NA	42	41	0	62	55	7	11.29
Chewer	IOB_06	chrX	107381268	C	A	ATG4A	INTRONIC	ENSG00000101844:[ENST00000372246(c.*893+47C>A)]	NA	NA	chrX*107381268*107381268*C*T*rs182929540*1*0.9995*0.0005298	NA	NA	NA	NA	NA	NA	NA	MODIFIER	NA	NA	98	98	0	156	150	5	3.21
Chewer	IOB_06	chrX	107979142	G	A	IRS4	MISSENSE	ENSG00000133124:[ENST00000372129(c.433C>T,Cgg>Tgg):ENSP00000361202(p.Arg145Trp)]	NA	NA	chrX*107979142*107979142*G*T*rs778732039*NA*NA*NA	NA	NA	NA	NA	D	PrD	NA	MODERATE	D	0.486	338	336	0	484	434	46	9.5
Chewer	IOB_06	chrX	118121056	T	C	LONRF3	NCT	79836:[NR_110311.1(n.1227-2315T>C)]	NA	NA	NA	NA	NA	NA	NA	NA	NA	NA	MODIFIER	NA	NA	129	129	0	201	173	27	13.43
Chewer	IOB_06	chrX	129299569	C	T	AIFM1	MISSENSE	ENSG00000156709:[ENST00000287295(c.62G>A,cGg>cAg):ENSP00000287295(p.Arg21Gln)]	NA	NA	NA	NA	NA	NA	NA	NA	NA	NA	MODIFIER	NA	NA	136	136	0	192	149	43	22.4
Chewer	IOB_06	chrX	130218906	C	A	ARHGAP36	MISSENSE	ENSG00000147256:[ENST00000370921(c.415C>A,Caa>Aaa):ENSP00000359959(p.Gln139Lys)]	NA	NA	NA	NA	NA	NA	NA	T	BN	NA	MODERATE	N	0.011	38	38	0	80	74	6	7.5
Chewer	IOB_06	chrX	142717119	A	T	SLITRK4	SILENT	ENSG00000179542:[ENST00000338017(c.1806T>A,acT>acA):ENSP00000336627(p.Thr602(=))]	NA	chrX*142717119*142717119*COSM3357394*A*T*2*large_intestine,haematopoietic_and_lymphoid_tissue*1,1	NA	NA	NA	NA	NA	NA	NA	NA	LOW	NA	NA	166	164	0	219	210	8	3.65
Chewer	IOB_06	chrX	149919641	G	A	MTMR1	INTRONIC	ENSG00000063601:[ENST00000370390(c.1656+326G>A)]	NA	NA	NA	NA	NA	NA	NA	NA	NA	NA	MODIFIER	NA	NA	85	85	0	117	101	16	13.68
Chewer	IOB_06	chrX	152990793	C	T	ABCD1	SILENT	ENSG00000101986:[ENST00000218104(c.72C>T,ctC>ctT):ENSP00000218104(p.Leu24(=))]	NA	NA	chrX*152990786*152990803*TGGCCCTCGCGGCCTATG*T*rs74315278*NA*NA*NA	NA	NA	NA	NA	NA	NA	NA	LOW	NA	NA	62	62	0	101	91	8	7.92
Chewer	IOB_06	chrX	153595116	G	A	FLNA	MISSENSE	ENSG00000196924:[ENST00000344736(c.971C>T,gCc>gTc):ENSP00000358863(p.Ala324Val)]	NA	NA	NA	NA	NA	NA	NA	D	PoD	NA	MODERATE	NA	NA	274	273	1	358	350	5	1.4
Chewer	IOB_07	chr1	983026	G	A	AGRN	MISSENSE	ENSG00000188157:[ENST00000379370(c.3590G>A,gGc>gAc):ENSP00000368678(p.Gly1197Asp)]	NA	NA	NA	NA	NA	NA	NA	D	PoD	NA	MODERATE	U	1	289	288	0	292	286	6	2.05
Chewer	IOB_07	chr1	2121260	G	A	AL590822.2	5UTR	ENSG00000269554:[ENST00000597060(c.-230G>A)]	NA	NA	NA	NA	NA	NA	NA	NA	NA	NA	MODIFIER	NA	NA	140	139	0	115	110	5	4.35
Chewer	IOB_07	chr1	2121260	G	A	C1orf86	INTRONIC	ENSG00000162585:[ENST00000400919(c.-476-40C>T)]	NA	NA	NA	NA	NA	NA	NA	NA	NA	NA	MODIFIER	NA	NA	140	139	0	115	110	5	4.35
Chewer	IOB_07	chr1	2121260	G	A	FAAP20	INTRONIC	199990:[NM_001256945.1(c.334+3818C>T)]	NA	NA	NA	NA	NA	NA	NA	NA	NA	NA	MODIFIER	NA	NA	140	139	0	115	110	5	4.35
Chewer	IOB_07	chr1	16475089	C	A	EPHA2	NONSENSE	ENSG00000142627:[ENST00000358432(c.607G>T,Gag>Tag):ENSP00000351209(p.Glu203Ter)]	NA	NA	NA	NA	NA	NA	NA	NA	NA	NA	MODIFIER	NA	NA	229	229	0	248	131	117	47.18
Chewer	IOB_07	chr1	17292467	C	A	CROCC	INTRONIC	ENSG00000058453:[ENST00000375541(c.4576-27C>A)]	NA	NA	chr1*17292467*17292467*C*G*rs759656149*NA*NA*NA	NA	NA	NA	NA	NA	NA	NA	MODIFIER	NA	NA	248	247	0	197	190	5	2.54
Chewer	IOB_07	chr1	27674461	C	T	SYTL1	SILENT	ENSG00000142765:[ENST00000318074(c.417C>T,ctC>ctT):ENSP00000316464(p.Leu139(=))]	NA	NA	chr1*27674461*27674461*C*G,T*rs768537098*NA*NA*NA	8.25E-06	0.00E+00	NA	NA	NA	NA	NA	LOW	NA	NA	120	120	0	117	99	17	14.53
Chewer	IOB_07	chr1	27684019	C	A	MAP3K6	MISSENSE	ENSG00000142733:[ENST00000357582(c.3130G>T,Ggg>Tgg):ENSP00000350195(p.Gly1044Trp)]	NA	NA	chr1*27684019*27684019*C*T*rs778812354*NA*NA*NA	NA	NA	NA	NA	D	PoD	NA	MODERATE	NA	NA	180	180	0	197	191	5	2.54
Chewer	IOB_07	chr1	32745227	C	T	LCK	MISSENSE	ENSG00000182866:[ENST00000333070(c.1010C>T,tCc>tTc):ENSP00000328213(p.Ser337Phe)]	NA	NA	NA	NA	NA	NA	NA	T_lc	BN	NA	MODERATE	N	0	122	122	0	101	83	18	17.82
Chewer	IOB_07	chr1	36550705	G	A	TEKT2	INTRONIC	ENSG00000092850:[ENST00000207457(c.156+27G>A)]	NA	NA	NA	NA	NA	NA	NA	NA	NA	NA	MODIFIER	NA	NA	220	219	0	242	182	57	23.55
Chewer	IOB_07	chr1	37337854	G	A	GRIK3	NONSENSE	ENSG00000163873:[ENST00000373091(c.667C>T,Cga>Tga):ENSP00000362183(p.Arg223Ter)]	NA	chr1*37337854*37337854*COSM287191*G*A*1*large_intestine*1	NA	NA	NA	NA	NA	NA	NA	NA	HIGH	NA	1	215	214	0	216	209	6	2.78
Chewer	IOB_07	chr1	39980792	C	T	BMP8A	INTRONIC	ENSG00000183682:[ENST00000331593(c.673+3216C>T)]	NA	NA	NA	NA	NA	NA	NA	NA	NA	NA	MODIFIER	NA	NA	45	45	0	27	19	8	29.63
Chewer	IOB_07	chr1	39980792	C	T	OXCT2P1	EXONIC-NC	192217:[NR_132962.1(n.255C>T)]	NA	NA	NA	NA	NA	NA	NA	NA	NA	NA	MODIFIER	NA	NA	45	45	0	27	19	8	29.63
Chewer	IOB_07	chr1	43770750	C	T	TIE1	MISSENSE	ENSG00000066056:[ENST00000372476(c.287C>T,cCc>cTc):ENSP00000361554(p.Pro96Leu)]	NA	NA	chr1*43770723*43770753*AGGTCACGCTTCGCGGCTTCTCCAAGCCCTC*A*rs770023620*NA*NA*NA	NA	NA	NA	NA	NA	NA	NA	MODIFIER	NA	NA	339	339	0	346	263	81	23.41
Chewer	IOB_07	chr1	44684127	C	A	DMAP1	MISSENSE	ENSG00000178028:[ENST00000315913(c.538C>A,Cac>Aac):ENSP00000312697(p.His180Asn)]	NA	NA	NA	NA	NA	NA	NA	NA	NA	NA	MODERATE	NA	1	171	171	0	211	203	5	2.37
Chewer	IOB_07	chr1	50885234	G	A	DMRTA2	SILENT	ENSG00000142700:[ENST00000404795(c.732C>T,ggC>ggT):ENSP00000383909(p.Gly244(=))]	NA	NA	chr1*50885234*50885234*G*T*rs757587924*NA*NA*NA	NA	NA	NA	NA	NA	NA	NA	LOW	NA	NA	408	407	0	535	514	20	3.74
Chewer	IOB_07	chr1	52211196	C	T	OSBPL9	EXONIC-NC	ENSG00000117859:[ENST00000467064(n.435C>T)]	NA	NA	NA	NA	NA	NA	NA	NA	NA	NA	MODIFIER	NA	NA	65	65	0	47	41	6	12.77
Chewer	IOB_07	chr1	57602286	C	A	DAB1	MISSENSE	ENSG00000173406:[ENST00000332102(c.236G>T,gGa>gTa):ENSP00000329120(p.Gly79Val)]	NA	NA	NA	NA	NA	NA	NA	NA	NA	NA	MODIFIER	NA	NA	135	134	0	167	114	53	31.74
Chewer	IOB_07	chr1	74574595	T	A	LRRIQ3	INTRONIC	127255:[XM_005270454.1(c.-94+483A>T)]	NA	NA	NA	NA	NA	NA	NA	NA	NA	NA	MODIFIER	NA	NA	42	42	0	31	17	14	45.16
Chewer	IOB_07	chr1	76378365	T	A	MSH4	INTRONIC	ENSG00000057468:[ENST00000263187(c.2620-16T>A)]	NA	NA	1.80E-03	8.25E-04	2.39E-03	1.80E-03	4.10E-03	NA	NA	NA	MODIFIER	NA	NA	50	50	0	36	25	8	22.22
Chewer	IOB_07	chr1	91781371	C	T	HFM1	INTRONIC-SS-DNR	ENSG00000162669:[ENST00000370425(c.3140+1G>A)]	NA	NA	NA	2.57E-05	6.60E-05	NA	NA	NA	NA	NA	HIGH	NA	NA	51	51	0	44	32	12	27.27
Chewer	IOB_07	chr1	91977313	C	T	CDC7	INTRONIC	ENSG00000097046:[ENST00000234626(c.430-25C>T)]	NA	NA	chr1*91977311*91977316*CTCATT*C*rs753858262*NA*NA*NA	NA	NA	NA	NA	NA	NA	NA	MODIFIER	NA	NA	93	92	1	74	54	20	27.03
Chewer	IOB_07	chr1	99771801	C	T	LPPR4	SILENT	ENSG00000117600:[ENST00000457765(c.1353C>T,gtC>gtT):ENSP00000394913(p.Val451(=))]	NA	NA	NA	NA	NA	NA	NA	NA	NA	NA	LOW	NA	NA	67	67	0	81	57	23	28.4
Chewer	IOB_07	chr1	102312393	T	G	OLFM3	NCT-SS-PRX	118427:[NR_110212.1(n.236+8A>C)]	NA	NA	NA	NA	NA	NA	NA	NA	NA	NA	MODIFIER	NA	NA	71	71	0	95	73	22	23.16
Chewer	IOB_07	chr1	150960596	C	A	ANXA9	MISSENSE	ENSG00000143412:[ENST00000368947(c.727C>A,Caa>Aaa):ENSP00000357943(p.Gln243Lys)]	NA	NA	NA	NA	NA	NA	NA	T	BN	NA	MODERATE	N	0.614	170	170	0	138	110	28	20.29
Chewer	IOB_07	chr1	155033268	C	A	ADAM15	MISSENSE	ENSG00000143537:[ENST00000368410(c.1355C>A,cCa>cAa):ENSP00000357395(p.Pro452Gln)]	NA	NA	NA	NA	NA	NA	NA	NA	NA	NA	MODIFIER	NA	NA	120	120	0	124	118	5	4.03
Chewer	IOB_07	chr1	155033268	C	A	DCST1-AS1	NCT	100505666:[NR_040773.1(n.235+2965G>T)]	NA	NA	NA	NA	NA	NA	NA	NA	NA	NA	MODIFIER	NA	NA	120	120	0	124	118	5	4.03
Chewer	IOB_07	chr1	155313412	C	A	ASH1L	MISSENSE	ENSG00000116539:[ENST00000368346(c.8118G>T,aaG>aaT):ENSP00000357330(p.Lys2706Asn)]	NA	NA	NA	NA	NA	NA	NA	NA	NA	NA	MODIFIER	NA	NA	199	198	0	165	159	5	3.03
Chewer	IOB_07	chr1	156551733	G	A	TTC24	MISSENSE	ENSG00000187862:[ENST00000368236(c.577G>A,Gca>Aca):ENSP00000357219(p.Ala193Thr)]	NA	chr1*156551728*156551740*COSM424130*GGGCCGCAGCCCT*G*1*breast*1|chr1*156551733*156551733*COSM1334872*G*A*1*large intestine*1	NA	8.51E-05	1.85E-04	NA	NA	D	PrD	NA	MODERATE	N	0.977	135	134	1	141	135	5	3.55
Chewer	IOB_07	chr1	169519972	C	G	F5	SILENT	ENSG00000198734:[ENST00000546081(c.891G>C,gtG>gtC):ENSP00000439664(p.Val297(=))]	NA	NA	NA	NA	NA	NA	NA	NA	NA	NA	LOW	NA	NA	83	83	0	103	81	22	21.36
Chewer	IOB_07	chr1	173772521	C	A	CENPL	SILENT	ENSG00000120334:[ENST00000356198(c.681G>T,ctG>ctT):ENSP00000348527(p.Leu227(=))]	NA	NA	NA	NA	NA	NA	NA	NA	NA	NA	MODIFIER	NA	NA	196	195	0	178	173	5	2.81
Chewer	IOB_07	chr1	173886300	C	A	SERPINC1	NCT	ENSG00000117601:[ENST00000494024(n.98+57G>T)]	NA	NA	NA	NA	NA	NA	NA	NA	NA	NA	MODIFIER	NA	NA	53	53	0	31	23	8	25.81
Chewer	IOB_07	chr1	179452264	G	A	AL160286.1	INTRONIC	ENSG00000269064:[ENST00000600581(c.68+5474C>T)]	NA	NA	NA	NA	NA	NA	NA	T	BN	NA	MODERATE	NA	NA	56	56	0	88	56	32	36.36
Chewer	IOB_07	chr1	179452264	G	A	AXDND1	MISSENSE-SS-PRX	ENSG00000162779:[ENST00000367618(c.1999G>A,Gta>Ata):ENSP00000356590(p.Val667Ile)]	NA	NA	NA	NA	NA	NA	NA	T	BN	NA	MODERATE	NA	NA	56	56	0	88	56	32	36.36
Chewer	IOB_07	chr1	196871589	C	T	CFHR2	INTRONIC	ENSG00000080910:[ENST00000367421(c.59-46996C>T)]	NA	NA	NA	NA	NA	NA	NA	NA	NA	NA	MODIFIER	NA	NA	89	87	0	64	51	12	18.75
Chewer	IOB_07	chr1	196871589	C	T	CFHR4	SILENT	ENSG00000134365:[ENST00000367416(c.97C>T,Cta>Tta):ENSP00000356386(p.Leu33(=))]	NA	NA	NA	NA	NA	NA	NA	NA	NA	NA	MODIFIER	NA	NA	89	87	0	64	51	12	18.75
Chewer	IOB_07	chr1	196928066	G	A	CFHR2	NONSENSE	ENSG00000080910:[ENST00000367415(c.669G>A,tgG>tgA):ENSP00000356385(p.Trp223Ter)]	NA	chr1*196928066*196928066*COSM531745*G*A*1*lung*1	chr1*196928066*196928066*G*C*rs764178525*NA*NA*NA	NA	NA	NA	NA	NA	NA	NA	MODIFIER	NA	NA	24	24	0	33	18	15	45.45
Chewer	IOB_07	chr1	196946778	G	A	CFHR5	MISSENSE	ENSG00000134389:[ENST00000367414(c.56G>A,cGa>cAa):ENSP00000356384(p.Arg19Gln)]	NA	NA	NA	1.65E-05	0.00E+00	NA	NA	NA	NA	NA	MODIFIER	NA	NA	197	197	0	144	119	24	16.67
Chewer	IOB_07	chr1	200973482	C	A	KIF21B	SILENT	ENSG00000116852:[ENST00000332129(c.1002G>T,tcG>tcT):ENSP00000328494(p.Ser334(=))]	NA	chr1*200973482*200973482*COSM902047*C*T*1*endometrium*1	.	3.82E-05	1.23E-04	NA	NA	NA	NA	NA	LOW	NA	NA	59	59	0	67	49	17	25.37
Chewer	IOB_07	chr1	201437586	G	A	PHLDA3	MISSENSE	ENSG00000174307:[ENST00000367309(c.329C>T,gCc>gTc):ENSP00000356278(p.Ala110Val)]	NA	NA	NA	NA	NA	NA	NA	NA	NA	NA	MODIFIER	NA	NA	238	237	0	261	189	69	26.44
Chewer	IOB_07	chr1	203012614	G	A	PPFIA4	SILENT	ENSG00000143847:[ENST00000367240(c.324G>A,cgG>cgA):ENSP00000356209(p.Arg108(=))]	NA	NA	NA	NA	NA	NA	NA	NA	NA	NA	MODIFIER	NA	NA	128	127	0	125	105	20	16

Patel *et al.* , 2021. Whole-exome sequencing analysis of oral squamous cell carcinoma delineated by tobacco usage habits
Supplementary Table 4. List of somatic single nucleotide variants identified using whole exome sequencing of OSCC patients

Cohort	Sample	Chromosome	Genomic position	Reference allele	Alternate allele	Genesymbol	Variant type	Variant description	OncoMD	COSMIC	dbSNP	ExAC	ExAC_SAS	1000G	1000G_SAS	SIFT	Polyphen2	CONDEL	VEP	LRT	PhastCon7Way	Total reads in Normal	Reference read support in Normal	Alternative read support in Normal	Total reads in Tumor	Reference read support in Tumor	Alternate read support in Tumor	Alternate allele frequency in Tumor
Chewer	IOB_07	chr1	205273168	G	A	NUAK2	MISSENSE	ENSG00000163545:[ENST00000367157(c.1297C>T,Cct>Tct):ENSP00000356125(p.Pro433Ser)]	NA	NA	NA	NA	NA	NA	NA	T	BN	NA	MODERATE	NA	0	157	154	1	135	114	21	15.56
Chewer	IOB_07	chr1	206327671	G	A	CTSE	INTRONIC	ENSG00000196188:[ENST00000358184(c.785+75G>A)]	NA	NA	NA	NA	NA	NA	NA	NA	NA	NA	MODIFIER	NA	NA	69	68	0	50	42	8	16
Chewer	IOB_07	chr1	248202332	G	T	OR2L13	INTRONIC	ENSG00000196071:[ENST00000366478(c.-143-59621G>T)]	NA	NA	NA	NA	NA	NA	NA	NA	NA	NA	MODIFIER	NA	NA	396	395	0	319	164	155	48.59
Chewer	IOB_07	chr1	248202332	G	T	OR2L2	MISSENSE	ENSG00000203663:[ENST00000366479(c.763G>T,Gct>Tct):ENSP00000355435(p.Ala255Ser)]	NA	NA	NA	NA	NA	NA	NA	NA	NA	NA	MODIFIER	NA	NA	396	395	0	319	164	155	48.59
Chewer	IOB_07	chr10	5203897	C	T	AKR1CL1	SILENT	ENSG00000196326:[ENST00000334314(c.300G>A,ccG>ccA):ENSP00000334626(p.Pro100(=))]	NA	NA	chr10*5203897*5203897*C*G,T*rs782099265*NA*NA*NA	1.67E-05	0.00E+00	NA	NA	NA	NA	NA	LOW	NA	NA	107	106	0	79	70	7	8.86
Chewer	IOB_07	chr10	24911637	G	A	ARHGAP21	INTRONIC	57584:[XM_005252545.1(c.231+25C>T)]	NA	NA	NA	NA	NA	NA	NA	NA	NA	NA	MODIFIER	NA	NA	65	65	0	53	40	13	24.53
Chewer	IOB_07	chr10	25684879	T	A	GPR158	MISSENSE	ENSG00000151025:[ENST00000376351(c.1048T>A,Tat>Aat):ENSP00000365529(p.Tyr350Asn)]	NA	NA	NA	NA	NA	NA	NA	D	PrD	NA	MODERATE	D	1	185	183	0	207	200	6	2.9
Chewer	IOB_07	chr10	70715985	C	T	DDX21	MISSENSE	ENSG00000165732:[ENST00000354185(c.4C>T,Ccg>Tcg):ENSP00000346120(p.Pro2Ser)]	NA	NA	NA	NA	NA	NA	NA	D_lc	PoD	NA	MODERATE	N	1	127	127	0	155	150	5	3.23
Chewer	IOB_07	chr10	71902563	G	A	TYSND1	SILENT	ENSG00000156521:[ENST00000287078(c.1344C>T,tgC>tgT):ENSP00000287078(p.Cys448(=))]	NA	NA	NA	8.42E-06	0.00E+00	NA	NA	NA	NA	NA	MODIFIER	NA	NA	108	108	0	123	116	6	4.88
Chewer	IOB_07	chr10	75532217	G	A	AC022400.2	SILENT	ENSG00000267964:[ENST00000595757(c.162C>T,tcC>tcT):ENSP00000472381(p.Ser54(=))]	NA	NA	NA	NA	NA	NA	NA	NA	NA	NA	HIGH	NA	1	70	70	0	69	49	20	28.99
Chewer	IOB_07	chr10	75532217	G	A	FUT11	NONSENSE	ENSG00000196968:[ENST00000372841(c.126G>A,tgG>tgA):ENSP00000361932(p.Trp42Ter)]	NA	NA	NA	NA	NA	NA	NA	NA	NA	NA	HIGH	NA	1	70	70	0	69	49	20	28.99
Chewer	IOB_07	chr10	81049696	C	T	ZMIZ1	INTRONIC	ENSG00000108175:[ENST00000334512(c.540+50C>T)]	NA	NA	NA	NA	NA	NA	NA	NA	NA	NA	MODIFIER	NA	NA	171	170	0	194	146	46	23.71
Chewer	IOB_07	chr10	93390492	C	T	PPP1R3C	MISSENSE	ENSG00000119938:[ENST00000238994(c.146G>A,cGa>cAa):ENSP00000238994(p.Arg49Gln)]	NA	NA	NA	NA	NA	NA	NA	D	BN	NA	MODERATE	D	1	162	162	0	186	146	37	19.89
Chewer	IOB_07	chr10	94835038	G	A	CYP26A1	MISSENSE	ENSG00000095596:[ENST00000371531(c.631G>A,Gag>Aag):ENSP00000360586(p.Glu211Lys)]	NA	NA	chr10*94835038*94835038*G*C*rs758275725*NA*NA*NA	NA	NA	NA	NA	T	BN	NA	MODERATE	D	1	234	233	0	265	208	56	21.13
Chewer	IOB_07	chr10	98064281	G	A	DNTT	SILENT	ENSG00000107447:[ENST00000371174(c.27G>A,ttG>ttA):ENSP00000360216(p.Leu9(=))]	NA	NA	.	8.29E-06	6.07E-05	NA	NA	NA	NA	NA	LOW	NA	0	27	27	0	49	35	14	28.57
Chewer	IOB_07	chr10	98064281	G	A	RP11-35J23.1	NCT	ENSG00000229418:[ENST00000454484(n.187+1985C>T)]	NA	NA	.	8.29E-06	6.07E-05	NA	NA	NA	NA	NA	LOW	NA	0	27	27	0	49	35	14	28.57
Chewer	IOB_07	chr10	100017720	C	T	LOXL4	INTRONIC	ENSG00000138131:[ENST00000260702(c.1105+18G>A)]	NA	NA	NA	NA	NA	NA	NA	NA	NA	NA	MODIFIER	NA	NA	408	404	0	386	281	103	26.68
Chewer	IOB_07	chr10	100185499	C	T	HPS1	NCT	ENSG00000107521:[ENST00000497527(n.287-22G>A)]	NA	NA	chr10*100185499*100185499*C*CG*rs774610151*NA*NA*NA|chr10*100185499*100185500*CG*C*rs769794815*NA*NA*NA	NA	NA	NA	NA	NA	NA	NA	MODIFIER	NA	NA	107	107	0	98	93	5	5.1
Chewer	IOB_07	chr10	105806519	G	A	COL17A1	MISSENSE	ENSG00000065618:[ENST00000353479(c.2348C>T,cCc>cTc):ENSP00000340937(p.Pro783Leu)]	NA	NA	NA	NA	NA	NA	NA	T	BN	NA	MODERATE	D	1	96	96	0	105	96	9	8.57
Chewer	IOB_07	chr10	105806520	G	A	COL17A1	MISSENSE	ENSG00000065618:[ENST00000353479(c.2347C>T,Ccc>Tcc):ENSP00000340937(p.Pro783Ser)]	NA	NA	NA	8.42E-06	0.00E+00	NA	NA	T	BN	NA	MODERATE	D	1	97	97	0	105	96	9	8.57
Chewer	IOB_07	chr10	116046111	C	G	AFAP1L2	INTRONIC	84632:[XM_005270242.1(c.1915-4581G>C)]	NA	chr10*116046111*116046111*COSM915033*C*T*1*endometrium*1	chr10*116046111*116046111*C*T*rs370405238*0*0.9998*0.0001997	NA	NA	NA	NA	NA	NA	NA	MODIFIER	NA	NA	235	234	0	296	231	63	21.28
Chewer	IOB_07	chr10	116046111	C	G	VWA2	MISSENSE	ENSG00000165816:[ENST00000392982(c.1411C>G,Cga>Gga):ENSP00000376708(p.Arg471Gly)]	NA	chr10*116046111*116046111*COSM915033*C*T*1*endometrium*1	chr10*116046111*116046111*C*T*rs370405238*0*0.9998*0.0001997	NA	NA	NA	NA	NA	NA	NA	MODIFIER	NA	NA	235	234	0	296	231	63	21.28
Chewer	IOB_07	chr10	120354413	G	A	PRLHR	MISSENSE	ENSG00000119973:[ENST00000239032(c.344C>T,cCg>cTg):ENSP00000239032(p.Pro115Leu)]	NA	NA	NA	NA	NA	NA	NA	D	PrD	NA	MODERATE	D	1	324	323	1	356	262	90	25.28
Chewer	IOB_07	chr10	123245094	C	A	FGFR2	INTRONIC	2263:[NM_001144916.1(c.1713-48G>T)]	NA	NA	NA	NA	NA	NA	NA	NA	NA	NA	MODIFIER	NA	NA	50	49	0	69	53	16	23.19
Chewer	IOB_07	chr10	124350242	C	T	DMBT1	INTRONIC	ENSG00000187908:[ENST00000344338(c.2107+32C>T)]	NA	NA	NA	1.69E-05	0.00E+00	NA	NA	NA	NA	NA	MODIFIER	NA	NA	128	128	0	103	79	24	23.3
Chewer	IOB_07	chr10	124350243	C	T	DMBT1	INTRONIC	ENSG00000187908:[ENST00000359586(c.670+8902C>T)]	NA	NA	NA	NA	NA	NA	NA	NA	NA	NA	MODIFIER	NA	NA	128	128	0	103	79	24	23.3
Chewer	IOB_07	chr10	131762057	C	A	EBF3	5UTR	ENSG00000108001:[ENST00000355311(c.-25G>T)]	NA	NA	chr10*131762057*131762057*C*T*rs746323254*NA*NA*NA	NA	NA	NA	NA	NA	NA	NA	MODIFIER	NA	NA	200	200	0	245	236	7	2.86
Chewer	IOB_07	chr10	135015973	C	T	KNDC1	INTRONIC	ENSG00000171798:[ENST00000368571(c.3192+571C>T)]	NA	NA	NA	NA	NA	NA	NA	NA	NA	NA	MODIFIER	NA	NA	95	95	0	127	90	37	29.13
Chewer	IOB_07	chr11	534289	C	T	HRAS	MISSENSE	ENSG00000174775:[ENST00000311189(c.34G>A,Ggc>Agc):ENSP00000309845(p.Gly12Ser)]	NA	chr11*534289*534289*COSM1644659*C*A*1*NS*1|chr11*534289*534289*COSM481*C*A*344*urinary_tract,lung,upper_aerodigestive_tract,thyroid,soft_tissue,skin,NS,central_nervous_system*7,2,5,5,1,9,1,314|chr11*534289*534289*COSM482*C*G*327*thyroid,pituitary,upper_aerodigestive_tract,skin,large_intestine,salivary_gland,central_nervous_system*2,3,2,1,1,4,314|chr11*534289*534289*COSM480*C*T*66*lung,urinary_tract,penis,upper_aerodigestive_tract,thyroid,skin,bone,soft_tissue,large_intestine,cervix,salivary_gland,prostate*2,9,1,26,4,9,1,4,1,7,1,1	chr11*534289*534289*C*A,G,T*rs104894229*NA*NA*NA	NA	NA	NA	NA	NA	NA	NA	MODIFIER	NA	NA	163	163	0	168	133	35	20.83
Chewer	IOB_07	chr11	2154748	G	A	IGF2	MISSENSE-SS-PRX	ENSG00000167244:[ENST00000434045(c.473C>T,cCg>cTg):ENSP00000391826(p.Pro158Leu)]	NA	chr11*2154748*2154748*COSM1210346*G*A*3*large_intestine,lung*2,1|chr11*2154748*2154748*COSM1210347*G*A*3*large_intestine,lung*2,1	NA	5.21E-05	1.95E-04	NA	NA	D	BN	NA	MODERATE	U	1	150	150	0	200	159	41	20.5
Chewer	IOB_07	chr11	2154748	G	A	INS-IGF2	3UTR-SS-PRX	ENSG00000129965:[ENST00000356578(c.*357C>T)]	NA	chr11*2154748*2154748*COSM1210346*G*A*3*large_intestine,lung*2,1|chr11*2154748*2154748*COSM1210347*G*A*3*large_intestine,lung*2,1	NA	5.21E-05	1.95E-04	NA	NA	D	BN	NA	MODERATE	U	1	150	150	0	200	159	41	20.5
Chewer	IOB_07	chr11	4470929	G	A	OR52K2	MISSENSE	ENSG00000181963:[ENST00000325719(c.360G>A,atG>atA):ENSP00000318956(p.Met120Ile)]	NA	NA	NA	NA	NA	NA	NA	D	PrD	NA	MODERATE	D	1	172	171	1	170	124	44	25.88
Chewer	IOB_07	chr11	6566864	C	T	DNHD1	SILENT	ENSG00000179532:[ENST00000254579(c.4695C>T,gtC>gtT):ENSP00000254579(p.Val1565(=))]	NA	NA	NA	NA	NA	NA	NA	NA	NA	NA	LOW	NA	NA	225	225	0	286	210	73	25.52
Chewer	IOB_07	chr11	6867239	G	A	OR10A5	MISSENSE	ENSG00000166363:[ENST00000299454(c.326G>A,gGg>gAg):ENSP00000299454(p.Gly109Glu)]	NA	NA	NA	NA	NA	NA	NA	D	PrD	NA	MODERATE	D	0.034	232	228	0	211	156	53	25.12
Chewer	IOB_07	chr11	7721902	G	T	OVCH2	MISSENSE	ENSG00000183378:[ENST00000454689(c.842C>A,cCt>cAt):ENSP00000407158(p.Pro281His)]	NA	NA	NA	NA	NA	NA	NA	D	PrD	NA	MODERATE	NA	NA	130	130	0	151	145	5	3.31
Chewer	IOB_07	chr11	9546833	C	G	ZNF143	NONSENSE	ENSG00000166478:[ENST00000299606(c.1649C>G,tCa>tGa):ENSP00000299606(p.Ser550Ter)]	NA	NA	NA	NA	NA	NA	NA	NA	NA	NA	HIGH	NA	NA	205	203	0	147	113	33	22.45
Chewer	IOB_07	chr11	17568969	C	T	OTOG	MISSENSE	ENSG00000188162:[ENST00000399391(c.50C>T,cCc>cTc):ENSP00000382323(p.Pro17Leu)]	NA	NA	NA	NA	NA	NA	NA	T_lc	BN	NA	MODERATE	NA	NA	29	29	0	28	18	10	35.71
Chewer	IOB_07	chr11	19799440	C	T	NAV2	INTRONIC	ENSG00000166833:[ENST00000360655(c.76-54590C>T)]	NA	NA	NA	9.28E-05	1.33E-04	NA	NA	NA	NA	NA	MODIFIER	NA	NA	142	141	0	112	78	32	28.57
Chewer	IOB_07	chr11	45204642	C	T	PRDM11	NONSENSE	ENSG00000019485:[ENST00000263765(c.556C>T,Cag>Tag):ENSP00000263765(p.Gln186Ter)]	NA	NA	NA	NA	NA	NA	NA	NA	NA	NA	MODIFIER	NA	NA	152	150	0	158	149	7	4.43
Chewer	IOB_07	chr11	47663986	C	T	MTCH2	MISSENSE	ENSG00000109919:[ENST00000302503(c.32G>A,gGc>gAc):ENSP00000303222(p.Gly11Asp)]	NA	chr11*47663986*47663986*COSM3359215*C*T*1*kidney*1	NA	NA	NA	NA	NA	NA	NA	NA	MODIFIER	NA	NA	180	180	0	169	162	6	3.55
Chewer	IOB_07	chr11	47663996	C	T	MTCH2	MISSENSE	ENSG00000109919:[ENST00000302503(c.22G>A,Gtg>Atg):ENSP00000303222(p.Val8Met)]	NA	NA	NA	NA	NA	NA	NA	NA	NA	NA	MODIFIER	NA	NA	172	172	0	159	151	6	3.77
Chewer	IOB_07	chr11	57182827	C	T	RP11-872D17.8	NCT	ENSG00000254979:[ENST00000534081(n.487-248G>A)]	NA	NA	NA	NA	NA	NA	NA	NA	NA	NA	LOW	NA	NA	62	62	0	58	43	15	25.86
Chewer	IOB_07	chr11	57182827	C	T	SLC43A3	SILENT	29015:[XM_005273955.1(c.813G>A,gtG>gtA):XP_005274012.1(p.Val271(=))]	NA	NA	NA	NA	NA	NA	NA	NA	NA	NA	LOW	NA	NA	62	62	0	58	43	15	25.86
Chewer	IOB_07	chr11	57512659	C	A	BTBD18	MISSENSE	ENSG00000233436:[ENST00000422652(c.1086G>T,aaG>aaT):ENSP00000394472(p.Lys362Asn)]	NA	NA	NA	NA	NA	NA	NA	NA	NA	NA	MODIFIER	NA	NA	98	98	0	113	81	32	28.32
Chewer	IOB_07	chr11	57512659	C	A	RP11-691N7.6	INTRONIC	ENSG00000254732:[ENST00000531074(c.*152+2203C>A)]	NA	NA	NA	NA	NA	NA	NA	NA	NA	NA	MODIFIER	NA	NA	98	98	0	113	81	32	28.32
Chewer	IOB_07	chr11	57512659	C	A	TMX2-CTNND1	NCT	100528016:[NR_037646.1(n.346+7519C>A)]	NA	NA	NA	NA	NA	NA	NA	NA	NA	NA	MODIFIER	NA	NA	98	98	0	113	81	32	28.32
Chewer	IOB_07	chr11	63258317	G	A	HRASLS5	INTRONIC	ENSG00000168004:[ENST00000394615(c.79+42C>T)]	NA	NA	NA	NA	NA	NA	NA	NA	NA	NA	MODIFIER	NA	NA	96	95	0	83	60	22	26.51
Chewer	IOB_07	chr11	63656495	C	G	MARK2	INTRONIC	ENSG00000072518:[ENST00000315032(c.55-6136C>G)]	NA	NA	NA	NA	NA	NA	NA	NA	NA	NA	MODIFIER	NA	NA	111	110	0	83	66	17	20.48
Chewer	IOB_07	chr11	64564625	G	A	MAP4K2	MISSENSE	ENSG00000168067:[ENST00000377350(c.1292C>T,cCa>cTa):ENSP00000366567(p.Pro431Leu)]	NA	NA	NA	5.90E-05	0.00E+00	NA	NA	NA	NA	NA	MODIFIER	NA	NA	124	123	0	117	92	25	21.37
Chewer	IOB_07	chr11	64673795	C	T	ATG2A	INTRONIC	23130:[XM_005273851.1(c.2964+11G>A)]	NA	NA	NA	NA	NA	NA	NA	NA	NA	NA	MODIFIER	NA	NA	162	162	0	172	125	47	27.33
Chewer	IOB_07	chr11	65387172	G	A	PCNXL3	INTRONIC	ENSG00000197136:[ENST00000355703(c.1829+41G>A)]	NA	NA	NA	4.68E-05	1.03E-04	NA	NA	NA	NA	NA	MODIFIER	NA	NA	127	127	0	109	104	5	4.59
Chewer	IOB_07	chr11	65484124	C	T	KAT5	INTRONIC	ENSG00000172977:[ENST00000534650(c.298-14C>T)]	NA	NA	chr11*65484124*65484124*C*G*rs371849724*NA*NA*NA	NA	NA	NA	NA	NA	NA	NA	MODIFIER	NA	NA	93	93	0	78	55	23	29.49
Chewer	IOB_07	chr11	65484124	C	T	RNASEH2C	INTRONIC	ENSG00000172922:[ENST00000534482(c.*103-1336G>A)]	NA	NA	chr11*65484124*65484124*C*G*rs371849724*NA*NA*NA	NA	NA	NA	NA	NA	NA	NA	MODIFIER	NA	NA	93	93	0	78	55	23	29.49
Chewer	IOB_07	chr11	66063103	G	A	TMEM151A	SILENT	ENSG00000179292:[ENST00000327259(c.1386G>A,ggG>ggA):ENSP00000326244(p.Gly462(=))]	NA	NA	NA	NA	NA	NA	NA	NA	NA	NA	LOW	NA	NA	72	72	0	46	30	15	32.61
Chewer	IOB_07	chr11	66392241	A	T	RBM14	SILENT	ENSG00000239306:[ENST00000310137(c.894A>T,gcA>gcT):ENSP00000311747(p.Ala298(=))]	NA	NA	NA	NA	NA	NA	NA	NA	NA	NA	MODIFIER	NA	NA	164	163	0	156	117	38	24.36
Chewer	IOB_07	chr11	66392241	A	T	RBM14-RBM4	NCT	ENSG00000248643:[ENST00000511114(n.428+7713A>T)]	NA	NA	NA	NA	NA	NA	NA	NA	NA	NA	MODIFIER	NA	NA	164	163	0	156	117	38	24.36
Chewer	IOB_07	chr11	66392241	A	T	RBM4	INTRONIC	ENSG00000173933:[ENST00000503028(c.-13+7713A>T)]	NA	NA	NA	NA	NA	NA	NA	NA	NA	NA	MODIFIER	NA	NA	164	163	0	156	117	38	24.36

Patel *et al.*, 2021. Whole-exome sequencing analysis of oral squamous cell carcinoma delineated by tobacco usage habits
Supplementary Table 4. List of somatic single nucleotide variants identified using whole exome sequencing of OSCC patients

Cohort	Sample	Chromosome	Genomic position	Reference allele	Alternate allele	Genesymbol	Variant type	Variant description	OncoMD	COSMIC	dbSNP	ExAC	ExAC_SAS	1000G	1000G_SAS	SIFT	Polyphen2	CONDEL	VEP	LRT	PhastCon7Way	Total reads in Normal	Reference read support in Normal	Alternative read support in Normal	Total reads in Tumor	Reference read support in Tumor	Alternate read support in Tumor	Alternate allele frequency in Tumor
Chewer	IOB_07	chr11	66581444	C	A	C11orf80	MISSENSE	ENSG00000173715:[ENST00000532565(c.493C>A,Cat>Aat):ENSP00000434804(p.His165Asn)]	NA	NA	NA	NA	NA	NA	NA	T	BN	NA	MODERATE	N	0.778	159	159	0	126	119	6	4.76
Chewer	IOB_07	chr11	67023114	C	T	KDM2A	EXONIC-NC	22992:[NR_027473.1(n.4489C>T)]	NA	NA	NA	NA	NA	NA	NA	NA	NA	NA	MODIFIER	NA	NA	96	96	0	81	65	16	19.75
Chewer	IOB_07	chr11	67817691	C	T	TCIRG1	MISSENSE	ENSG00000110719:[ENST00000532635(c.1558C>T,Cgc>Tgc):ENSP00000434407(p.Arg520Cys)]	NA	NA	chr11*67817691*67817691*C*A*rs587779413*NA*NA*NA	NA	NA	NA	NA	NA	NA	NA	MODIFIER	NA	NA	250	249	0	242	203	38	15.7
Chewer	IOB_07	chr11	68530235	G	A	CPT1A	INTRONIC	1374:[XM_005273763.1(c.1837-6C>T)]	NA	NA	NA	NA	NA	NA	NA	NA	NA	NA	LOW	NA	NA	149	149	0	133	107	26	19.55
Chewer	IOB_07	chr11	74989825	C	T	ARRB1	INTRONIC	408:[XM_005273998.1(c.495-37G>A)]	NA	NA	NA	NA	NA	NA	NA	NA	NA	NA	MODIFIER	NA	NA	180	179	0	174	153	20	11.49
Chewer	IOB_07	chr11	108183205	G	T	ATM	NONSENSE	ENSG00000149311:[ENST00000278616(c.5986G>T,Gaa>Taa):ENSP00000278616(p.Glu1996Ter)]	NA	NA	NA	NA	NA	NA	NA	NA	NA	NA	MODIFIER	NA	NA	26	25	0	30	23	6	20
Chewer	IOB_07	chr11	108183205	G	T	C11orf65	INTRONIC	ENSG00000166323:[ENST00000525729(c.641-3407C>A)]	NA	NA	NA	NA	NA	NA	NA	NA	NA	NA	MODIFIER	NA	NA	26	25	0	30	23	6	20
Chewer	IOB_07	chr11	108253779	C	G	C11orf65	MISSENSE	ENSG00000166323:[ENST00000393084(c.911G>C,aGa>aCa):ENSP00000376799(p.Arg304Thr)]	NA	NA	NA	NA	NA	NA	NA	NA	NA	NA	MODERATE	NA	0	56	56	0	67	55	12	17.91
Chewer	IOB_07	chr11	108382266	G	A	EXPH5	MISSENSE	ENSG00000110723:[ENST00000428840(c.3740C>T,aCg>aTg):ENSP00000391966(p.Thr1247Met)]	NA	NA	NA	4.19E-05	1.30E-04	NA	NA	D	BN	NA	MODERATE	N	0.006	73	73	0	80	65	15	18.75
Chewer	IOB_07	chr11	108382266	G	A	NA	NCT	101928561:[XR_246464.1(n.267-3772G>A)]	NA	NA	NA	4.19E-05	1.30E-04	NA	NA	D	BN	NA	MODERATE	N	0.006	73	73	0	80	65	15	18.75
Chewer	IOB_07	chr11	117647604	C	T	DSCAML1	MISSENSE	ENSG00000177103:[ENST00000525836(c.118G>A,Gtg>Atg):ENSP00000436387(p.Val40Met)]	NA	chr11*117647604*117647604*COSM541051*C*A*1*lung*1|chr11*117647604*117647604*COSM3383226*C*T*1*pancreas*1	NA	2.48E-05	6.08E-05	NA	NA	T	PrD	NA	MODERATE	NA	NA	58	58	0	59	44	15	25.42
Chewer	IOB_07	chr11	118039266	C	T	SCN2B	INTRONIC	ENSG00000149575:[ENST00000278947(c.237+34G>A)]	NA	NA	NA	NA	NA	NA	NA	NA	NA	NA	MODIFIER	NA	NA	99	99	0	107	81	26	24.3
Chewer	IOB_07	chr11	134241654	C	T	GLB1L2	SILENT	ENSG00000149328:[ENST00000525089(c.1252C>T,acC>acT):ENSP00000432527(p.Thr418(=))]	NA	NA	7.99E-04	7.25E-04	2.60E-03	7.99E-04	3.10E-03	NA	NA	NA	MODIFIER	NA	NA	60	60	0	51	27	24	47.06
Chewer	IOB_07	chr12	306511	C	A	SLC6A12	NCT	ENSG00000111181:[ENST00000545058(n.491+32G>T)]	NA	NA	NA	NA	NA	NA	NA	NA	NA	NA	MODIFIER	NA	NA	86	85	1	103	97	5	4.85
Chewer	IOB_07	chr12	498389	C	A	KDM5A	5UTR	ENSG00000073614:[ENST00000382815(c.-132G>T)]	NA	NA	NA	NA	NA	NA	NA	NA	NA	NA	MODIFIER	NA	NA	107	107	0	103	96	5	4.85
Chewer	IOB_07	chr12	661307	G	A	B4GALNT3	MISSENSE	ENSG00000139044:[ENST00000266383(c.1186G>A,Gaa>Aaa):ENSP00000266383(p.Glu396Lys)]	NA	NA	NA	NA	NA	NA	NA	D	PoD	NA	MODERATE	NA	NA	183	183	0	153	114	39	25.49
Chewer	IOB_07	chr12	3039467	G	A	TULP3	MISSENSE	ENSG00000078246:[ENST00000397132(c.448G>A,Gca>Aca):ENSP00000380321(p.Ala150Thr)]	NA	NA	NA	NA	NA	NA	NA	NA	NA	NA	MODIFIER	NA	NA	183	182	0	131	125	6	4.58
Chewer	IOB_07	chr12	6950533	G	A	GNB3	EXONIC-NC	ENSG00000111664:[ENST00000540458(n.1192G>A)]	NA	NA	NA	1.04E-05	7.28E-05	NA	NA	NA	NA	NA	MODIFIER	NA	NA	104	104	0	104	53	51	49.04
Chewer	IOB_07	chr12	7966975	C	T	SLC2A14	SILENT	ENSG00000173262:[ENST00000539924(c.1545G>A,ggG>ggA):ENSP00000445929(p.Gly515(=))]	NA	NA	NA	2.84E-01	3.84E-01	NA	NA	NA	NA	NA	LOW	NA	NA	59	50	9	57	21	36	63.16
Chewer	IOB_07	chr12	10786777	T	C	STYK1	INTRONIC	ENSG00000060140:[ENST00000075503(c.53-54A>G)]	NA	NA	NA	NA	NA	NA	NA	NA	NA	NA	MODIFIER	NA	NA	44	43	0	39	25	12	30.77
Chewer	IOB_07	chr12	46231174	C	G	ARID2	NONSENSE	ENSG00000189079:[ENST00000334344(c.1094C>G,tCa>tGa):ENSP00000335044(p.Ser365Ter)]	NA	chr12*46231174*46231175*COSM1628605*CA*C*1*liver*1	NA	NA	NA	NA	NA	NA	NA	NA	MODIFIER	NA	NA	46	46	0	41	16	25	60.98
Chewer	IOB_07	chr12	50155613	G	T	TMBIM6	EXONIC-NC	ENSG00000139644:[ENST00000550040(n.645G>T)]	NA	NA	NA	NA	NA	NA	NA	NA	NA	NA	MODIFIER	NA	NA	235	233	0	219	123	93	42.47
Chewer	IOB_07	chr12	52771771	C	T	KRT84	3UTR	ENSG00000161849:[ENST00000257951(c.*47G>A)]	NA	NA	NA	6.24E-05	0.00E+00	NA	NA	NA	NA	NA	MODIFIER	NA	NA	42	42	0	64	49	14	21.88
Chewer	IOB_07	chr12	54688945	C	T	NFE2	MISSENSE	ENSG00000123405:[ENST00000312156(c.88G>A,Gag>Aag):ENSP00000312436(p.Glu30Lys)]	NA	NA	NA	NA	NA	NA	NA	D	PrD	NA	MODERATE	NA	NA	103	101	0	102	74	28	27.45
Chewer	IOB_07	chr12	54688945	C	T	RP11-968A15.8	NCT	ENSG00000258344:[ENST00000553061(n.545+17986C>T)]	NA	NA	NA	NA	NA	NA	NA	D	PrD	NA	MODERATE	NA	NA	103	101	0	102	74	28	27.45
Chewer	IOB_07	chr12	75741388	C	T	CAPS2	NCT	ENSG00000180881:[ENST00000486196(n.475+23471G>A)]	NA	NA	NA	NA	NA	NA	NA	NA	NA	NA	MODIFIER	NA	NA	124	124	0	132	72	60	45.45
Chewer	IOB_07	chr12	75741388	C	T	GLIPR1L1	INTRONIC	256710:[XM_005268772.1(c.676-14C>T)]	NA	NA	NA	NA	NA	NA	NA	NA	NA	NA	MODIFIER	NA	NA	124	124	0	132	72	60	45.45
Chewer	IOB_07	chr12	80828770	G	A	PTPRQ	MISSENSE	ENSG00000139304:[ENST00000547376(c.388G>A,Ggc>Agc):ENSP00000448844(p.Gly130Ser)]	NA	NA	chr12*80828770*80828770*G*T*rs116584119*1*0.9794*0.02057	NA	NA	NA	NA	T	BN	NA	MODERATE	NA	NA	36	36	0	41	32	9	21.95
Chewer	IOB_07	chr12	81741512	C	T	PPFIA2	MISSENSE	ENSG00000139220:[ENST00000553058(c.775G>A,Gct>Act):ENSP00000448941(p.Ala259Thr)]	NA	NA	NA	NA	NA	NA	NA	D	PoD	NA	MODERATE	D	1	79	78	0	29	22	7	24.14
Chewer	IOB_07	chr12	95397395	C	A	NDUFA12	MISSENSE	ENSG00000184752:[ENST00000552205(c.42G>T,cGa>cTa):ENSP00000449144(p.Arg15Leu)]	NA	NA	chr12*95397395*95397396*CG*C*rs748012743*NA*NA*NA	NA	NA	NA	NA	T	BN	NA	MODERATE	N	0.973	248	247	1	236	230	6	2.54
Chewer	IOB_07	chr12	109685435	C	T	ACACB	MISSENSE	ENSG00000076555:[ENST00000538526(c.1595C>T,Ccc>Tcc):ENSP00000443281(p.Pro533Ser)]	NA	NA	NA	NA	NA	NA	NA	T	PrD	NA	MODERATE	D	1	40	40	0	33	25	8	24.24
Chewer	IOB_07	chr12	113515903	C	T	DTX1	MISSENSE	ENSG00000135144:[ENST00000257600(c.934C>T,Ccg>Tcg):ENSP00000257600(p.Pro312Ser)]	NA	NA	NA	NA	NA	NA	NA	T	BN	NA	MODERATE	D	0.999	38	38	0	47	35	12	25.53
Chewer	IOB_07	chr12	113515904	C	T	DTX1	MISSENSE	ENSG00000135144:[ENST00000257600(c.935C>T,cCg>cTg):ENSP00000257600(p.Pro312Leu)]	NA	NA	NA	NA	NA	NA	NA	T	BN	NA	MODERATE	D	1	38	38	0	45	33	12	26.67
Chewer	IOB_07	chr12	113600809	G	A	DDX54	MISSENSE	ENSG00000123064:[ENST00000306014(c.2123C>T,gCa>gTa):ENSP00000304072(p.Ala708Val)]	NA	NA	NA	NA	NA	NA	NA	NA	NA	NA	MODIFIER	NA	NA	114	113	0	73	59	14	19.18
Chewer	IOB_07	chr12	125441781	C	T	DHX37	INTRONIC	ENSG00000150990:[ENST00000544745(c.1519-100G>A)]	NA	NA	NA	NA	NA	NA	NA	NA	NA	NA	MODIFIER	NA	NA	61	60	0	60	50	10	16.67
Chewer	IOB_07	chr13	24468763	T	C	C1QTNF9B	INTRONIC	ENSG00000205863:[ENST00000382057(c.167-423A>G)]	NA	NA	NA	3.70E-02	4.59E-02	NA	NA	NA	NA	NA	MODIFIER	NA	NA	57	56	1	62	49	13	20.97
Chewer	IOB_07	chr13	24468763	T	C	C1QTNF9B-AS1	NCT	ENSG00000205861:[ENST00000417034(n.74-2245T>C)]	NA	NA	NA	3.70E-02	4.59E-02	NA	NA	NA	NA	NA	MODIFIER	NA	NA	57	56	1	62	49	13	20.97
Chewer	IOB_07	chr13	24860400	G	A	RP11-307N16.6	MISSENSE	ENSG00000273167:[ENST00000382141(c.1984G>A,Gtg>Atg):ENSP00000371576(p.Val662Met)]	NA	NA	NA	NA	NA	NA	NA	D	PrD	NA	MODERATE	D	1	63	63	0	48	35	13	27.08
Chewer	IOB_07	chr13	24860400	G	A	SPATA13	MISSENSE	ENSG00000182957:[ENST00000343003(c.307G>A,Gtg>Atg):ENSP00000343631(p.Val103Met)]	NA	NA	NA	NA	NA	NA	NA	D	PrD	NA	MODERATE	D	1	63	63	0	48	35	13	27.08
Chewer	IOB_07	chr13	24860401	T	G	RP11-307N16.6	MISSENSE	ENSG00000273167:[ENST00000382141(c.1985T>G,gTg>gGg):ENSP00000371576(p.Val662Gly)]	NA	NA	NA	NA	NA	NA	NA	NA	NA	NA	MODERATE	NA	1	63	63	0	48	35	13	27.08
Chewer	IOB_07	chr13	24860401	T	G	SPATA13	MISSENSE	ENSG00000182957:[ENST00000399949(c.242T>G,gTg>gGg):ENSP00000382830(p.Val81Gly)]	NA	NA	NA	NA	NA	NA	NA	NA	NA	NA	MODERATE	NA	1	63	63	0	48	35	13	27.08
Chewer	IOB_07	chr13	32760384	T	C	FRY	INTRONIC	ENSG00000073910:[ENST00000380250(c.3320-13T>C)]	NA	NA	NA	NA	NA	NA	NA	NA	NA	NA	MODIFIER	NA	NA	103	102	0	100	78	22	22
Chewer	IOB_07	chr13	42783188	C	A	DGKH	MISSENSE	ENSG00000102780:[ENST00000538674(c.1960C>A,Cag>Aag):ENSP00000441308(p.Gln654Lys)]	NA	NA	NA	NA	NA	NA	NA	NA	NA	NA	MODIFIER	NA	NA	207	207	0	196	190	5	2.55
Chewer	IOB_07	chr13	51417609	C	T	DLEU7	SILENT	ENSG00000186047:[ENST00000400393(c.174G>A,cgG>cgA):ENSP00000420976(p.Arg58(=))]	NA	NA	NA	NA	NA	NA	NA	NA	NA	NA	MODIFIER	NA	NA	44	44	0	69	52	17	24.64
Chewer	IOB_07	chr13	51417609	C	T	DLEU7-AS1	NCT	100874074:[NR_046551.1(n.438+3379C>T)]	NA	NA	NA	NA	NA	NA	NA	NA	NA	NA	MODIFIER	NA	NA	44	44	0	69	52	17	24.64
Chewer	IOB_07	chr13	61989142	C	T	PCDH20	INTRONIC	ENSG00000197991:[ENST00000409186(c.132+18G>A)]	NA	NA	chr13*61989142*61989142*C*A*rs754242610*NA*NA*NA	NA	NA	NA	NA	NA	NA	NA	MODIFIER	NA	NA	40	40	0	59	44	15	25.42
Chewer	IOB_07	chr13	97485157	C	T	HS6ST3	MISSENSE	ENSG00000185352:[ENST00000376705(c.1121C>T,tCc>tTc):ENSP00000365895(p.Ser374Phe)]	NA	NA	chr13*97485157*97485157*C*G,T*rs141432230*NA*NA*NA	NA	NA	NA	NA	D	PrD	NA	MODERATE	N	1	196	194	0	187	141	46	24.6
Chewer	IOB_07	chr13	98018827	T	G	MBNL2	NCT	10150:[XR_243036.1(n.1947+20T>G)]	NA	NA	NA	NA	NA	NA	NA	NA	NA	NA	MODIFIER	NA	NA	108	107	0	118	88	28	23.73
Chewer	IOB_07	chr13	101318251	G	A	TMTC4	SILENT	ENSG00000125247:[ENST00000440120(c.273C>T,acC>acT):ENSP00000393600(p.Thr91(=))]	NA	NA	NA	8.24E-05	1.32E-04	NA	NA	NA	NA	NA	MODIFIER	NA	NA	70	69	1	84	64	19	22.62
Chewer	IOB_07	chr13	114793376	G	C	RASA3	MISSENSE	ENSG00000185989:[ENST00000389544(c.381C>G,atC>atG):ENSP00000374195(p.Ile127Met)]	NA	NA	NA	NA	NA	NA	NA	D_lc	BN	NA	MODERATE	NA	0.024	150	149	0	139	112	26	18.71
Chewer	IOB_07	chr14	20529107	C	T	OR4L1	MISSENSE	ENSG00000176246:[ENST00000315683(c.904C>T,Cgg>Tgg):ENSP00000319217(p.Arg302Trp)]	NA	chr14*20529107*20529107*COSM262621*C*T*3*lung,large_intestine*1,2	NA	2.70E-05	9.79E-05	NA	NA	T	BN	NA	MODERATE	N	0	55	55	0	78	60	18	23.08
Chewer	IOB_07	chr14	20823914	C	T	PARP2	SILENT	ENSG00000129484:[ENST00000429687(c.906C>T,ctC>ctT):ENSP00000392972(p.Leu302(=))]	NA	NA	NA	NA	NA	NA	NA	NA	NA	NA	LOW	NA	NA	70	68	0	78	60	18	23.08
Chewer	IOB_07	chr14	20897409	G	A	KLHL33	SILENT	ENSG00000185271:[ENST00000344581(c.1201C>T,Ctg>Ttg):ENSP00000341549(p.Leu401(=))]	NA	NA	NA	NA	NA	NA	NA	NA	NA	NA	LOW	NA	1	145	144	0	121	88	32	26.45
Chewer	IOB_07	chr14	20898338	C	T	KLHL33	MISSENSE	ENSG00000185271:[ENST00000344581(c.497G>A,cGc>cAc):ENSP00000341549(p.Arg166His)]	NA	NA	NA	1.02E-04	1.26E-04	NA	NA	D	PrD	NA	MODERATE	D	1	188	187	1	186	178	5	2.69
Chewer	IOB_07	chr14	23419650	G	A	HAUS4	NCT	ENSG00000092036:[ENST00000553794(n.424-2408C>T)]	NA	NA	NA	4.25E-05	1.87E-04	NA	NA	NA	NA	NA	MODIFIER	NA	NA	125	125	0	128	99	29	22.66
Chewer	IOB_07	chr14	23419650	G	A	RP11-298I3.1	NCT	ENSG00000257285:[ENST00000548322(n.159-4689G>A)]	NA	NA	NA	4.25E-05	1.87E-04	NA	NA	NA	NA	NA	MODIFIER	NA	NA	125	125	0	128	99	29	22.66
Chewer	IOB_07	chr14	23419650	G	A	RP11-298I3.5	INTRONIC	ENSG00000259132:[ENST00000555074(c.51-2428C>T)]	NA	NA	NA	4.25E-05	1.87E-04	NA	NA	NA	NA	NA	MODIFIER	NA	NA	125	125	0	128	99	29	22.66
Chewer	IOB_07	chr14	23991172	T	C	RP11-66N24.4	NCT	ENSG00000157306:[ENST00000554403(n.1068+7547T>C)]	NA	NA	NA	NA	NA	NA	NA	NA	NA	NA	MODIFIER	NA	NA	67	66	0	80	59	21	26.25
Chewer	IOB_07	chr14	23991172	T	C	THTPA	NCT	79178:[NR_046052.1(n.245+7547T>C)]	NA	NA	NA	NA	NA	NA	NA	NA	NA	NA	MODIFIER	NA	NA	67	66	0	80	59	21	26.25
Chewer	IOB_07	chr14	23991172	T	C	ZFHX2	SILENT	ENSG00000136367:[ENST00000419474(c.7718A>G,tAa>tGa):ENSP00000413418(p.Ter2573Ter)]	NA	NA	NA	NA	NA	NA	NA	NA	NA	NA	MODIFIER	NA	NA	67	66	0	80	59	21	26.25
Chewer	IOB_07	chr14	24679911	C	A	AL136419.6	EXONIC-NC	ENSG00000260669:[ENST00000565988(n.1631G>T)]	NA	NA	NA	8.24E-06	0.00E+00	NA	NA	NA	NA	NA	MODIFIER	NA	NA	128	126	1	91	65	26	28.57
Chewer	IOB_07	chr14	24679911	C	A	CHMP4A	MISSENSE	ENSG00000254505:[ENST00000533011(c.453G>T,caG>caT):ENSP00000432575(p.Gln151His)]	NA	NA	NA	8.24E-06	0.00E+00	NA	NA	NA	NA	NA	MODIFIER	NA	NA	128	126	1	91	65	26	28.57
Chewer	IOB_07	chr14	24679911	C	A	TM9SF1	MISSENSE	ENSG00000254692:[ENST00000530611(c.423G>T,caG>caT):ENSP00000433967(p.Gln141His)]	NA	NA	NA	8.24E-06	0.00E+00	NA	NA	NA	NA	NA	MODIFIER	NA	NA	128	126	1	91	65	26	28.57
Chewer	IOB_07	chr14	24887434	G	A	NYNRIN	3UTR	ENSG00000205978:[ENST00000382554(c.*782G>A)]	NA	NA	NA	NA	NA	NA	NA	NA	NA	NA	MODIFIER	NA	NA	59	57	0	51	42	8	15.69
Chewer	IOB_07	chr14	52520308	G	A	NID2	MISSENSE	ENSG00000087303:[ENST00000216286(c.1418C>T,tCc>tTc):ENSP00000216286(p.Ser473Phe)]	NA	NA	NA	NA	NA	NA	NA	T	BN	NA	MODERATE	N	0.996	114	114	0	141	104	36	25.53
Chewer	IOB_07	chr14	52781935	C	T	PTGER2	SILENT	ENSG00000125384:[ENST00000245457(c.669C>T,atC>atT):ENSP00000245457(p.Ile223(=))]	NA	NA	NA	3.33E-05	2.45E-04	NA	NA	NA	NA	NA	LOW	NA	0.011	128	128	0	151	106	44	29.14
Chewer	IOB_07	chr14	53250174	C	T	GNPNAT1	MISSENSE	ENSG00000100522:[ENST00000216410(c.184G>A,Gag>Aag):ENSP00000216410(p.Glu62Lys)]	NA	NA	NA	NA	NA	NA	NA	T	BN	NA	MODERATE	NA	NA	33	32	0	36	31	5	13.89
Chewer	IOB_07	chr14	55310466	G	A	GCH1	NCT	ENSG00000131979:[ENST00000395521(n.293-694C>T)]	NA	NA	NA	NA	NA	NA	NA	NA	NA	NA	MODIFIER	NA	NA	100	100	0	125	103	21	16.8
Chewer	IOB_07	chr14	60616053	C	A	DHRS7	MISSENSE	ENSG00000100612:[ENST00000554101(c.974G>T,aaG>aaT):ENSP00000450899(p.Lys325Asn)]	NA	NA	NA	NA	NA	NA	NA	NA	NA	NA	MODIFIER	NA	NA	157	156	0	154	148	5	3.25
Chewer	IOB_07	chr14	60616053	C	A	PCNXL4	INTRONIC	ENSG00000126773:[ENST00000406949(c.2566-19357C>A)]	NA	NA	NA	NA	NA	NA	NA	NA	NA	NA	MODIFIER	NA	NA	157	156	0	154	148	5	3.25
Chewer	IOB_07	chr14	67631969	C	T	GPHN	NCT	ENSG00000171723:[ENST00000555527(n.434+26C>T)]	NA	NA	NA	NA	NA	NA	NA	NA	NA	NA	MODIFIER	NA	NA	65	65	0	78	60	17	21.79
Chewer	IOB_07	chr14	68935774	G	A	RAD51B	NCT	ENSG00000182185:[ENST00000557045(n.394+807G>A)]	NA	NA	NA	NA	NA	NA	NA	NA	NA	NA	MODIFIER	NA	NA	50	50	0	45	30	14	31.11
Chewer	IOB_07	chr14	73554750	C	T	RBM25	MISSENSE	ENSG00000119707:[ENST00000261973(c.424C>T,Cgt>Tgt):ENSP00000261973(p.Arg142Cys)]	NA	NA	NA	NA	NA	NA	NA	NA	NA	NA	MODIFIER	NA	NA	166	165	0	153	120	32	20.92
Chewer	IOB_07	chr14	77948953	C	G	ISM2	MISSENSE	ENSG00000100593:[ENST00000412904(c.442G>C,Gag>Cag):ENSP00000416773(p.Glu148Gln)]	NA	NA	NA	NA	NA	NA	NA	D	BN	NA	MODERATE	N	0.958	67	67	0	74	65	8	10.81
Chewer	IOB_07	chr14	91444770	C	T	RPS6KA5	MISSENSE	ENSG00000100784:[ENST00000536315(c.37G>A,Gcc>Acc):ENSP00000442803(p.Ala13Thr)]	NA	NA	chr14*91444768*91444786*GGCCTTTTGAACGATTGTT*G*rs781712449*NA*NA*NA	NA	NA	NA	NA	T_lc	BN	NA	MODERATE	NA	NA	252	252	0	253	245	5	1.98

Patel *et al.* , 2021. Whole-exome sequencing analysis of oral squamous cell carcinoma delineated by tobacco usage habits
Supplementary Table 4. List of somatic single nucleotide variants identified using whole exome sequencing of OSCC patients

Cohort	Sample	Chromosome	Genomic position	Reference allele	Alternate allele	Genesymbol	Variant type	Variant description	OncoMD	COSMIC	dbSNP	ExAC	ExAC_SAS	1000G	1000G_SAS	SIFT	Polyphen2	CONDEL	VEP	LRT	PhastCon7Way	Total reads in Normal	Reference read support in Normal	Alternative read support in Normal	Total reads in Tumor	Reference read support in Tumor	Alternate read support in Tumor	Alternate allele frequency in Tumor
Chewer	IOB_07	chr14	102461057	C	T	DYNC1H1	SILENT	ENSG00000197102:[ENST00000360184(c.3204C>T,atC>atT):ENSP00000348965(p.Ile1068(=))]	NA	NA	NA	NA	NA	NA	NA	NA	NA	NA	LOW	NA	0.978	139	138	0	110	77	33	30
Chewer	IOB_07	chr14	104642189	C	T	KIF26A	MISSENSE	ENSG00000066735:[ENST00000423312(c.3064C>T,Cgg>Tgg):ENSP00000388241(p.Arg1022Trp)]	NA	NA	NA	NA	NA	NA	NA	D	BN	NA	MODERATE	NA	NA	133	133	0	153	148	5	3.27
Chewer	IOB_07	chr14	105344362	C	A	CEP170B	INTRONIC	ENSG00000099814:[ENST00000453495(c.276+60C>A)]	NA	NA	NA	NA	NA	NA	NA	NA	NA	NA	MODIFIER	NA	NA	86	86	0	121	116	5	4.13
Chewer	IOB_07	chr15	25344754	A	C	SNHG14	NCT	ENSG00000224078:[ENST00000546682(n.1475+329A>C)]	NA	NA	NA	NA	NA	NA	NA	NA	NA	NA	MODIFIER	NA	NA	76	76	0	87	64	23	26.44
Chewer	IOB_07	chr15	25456938	G	A	PWAR4	EXONIC-NC	347745:[NR_022010.1(n.100G>A)]	NA	NA	NA	NA	NA	NA	NA	NA	NA	NA	MODIFIER	NA	NA	129	129	0	101	84	16	15.84
Chewer	IOB_07	chr15	25456938	G	A	SNHG14	NCT	ENSG00000224078:[ENST00000424333(n.1874+53G>A)]	NA	NA	NA	NA	NA	NA	NA	NA	NA	NA	MODIFIER	NA	NA	129	129	0	101	84	16	15.84
Chewer	IOB_07	chr15	29346093	C	T	APBA2	SILENT	ENSG00000034053:[ENST00000411764(c.6C>T,gcC>gcT):ENSP00000409312(p.Ala2(=))]	NA	NA	NA	NA	NA	NA	NA	NA	NA	NA	MODIFIER	NA	NA	72	70	0	96	79	17	17.71
Chewer	IOB_07	chr15	31233356	C	A	FAN1	INTRONIC	ENSG00000198690:[ENST00000565280(c.*1899-413C>A)]	NA	NA	chr15*31233352*31233356*GTGAC*G*rs3840035*1*0.7821*0.2179|chr15*31233352*31233356*GTGAC*G*rs71963314*1*0.7821*0.2179	NA	NA	NA	NA	NA	NA	NA	MODIFIER	NA	NA	111	111	0	111	104	6	5.41
Chewer	IOB_07	chr15	31233356	C	A	MTMR10	3UTR	ENSG00000166912:[ENST00000568604(c.*2720G>T)]	NA	NA	chr15*31233352*31233356*GTGAC*G*rs3840035*1*0.7821*0.2179|chr15*31233352*31233356*GTGAC*G*rs71963314*1*0.7821*0.2179	NA	NA	NA	NA	NA	NA	NA	MODIFIER	NA	NA	111	111	0	111	104	6	5.41
Chewer	IOB_07	chr15	42021510	C	T	MGA	MISSENSE	ENSG00000174197:[ENST00000219905(c.3806C>T,tCa>tTa):ENSP00000219905(p.Ser1269Leu)]	NA	NA	NA	NA	NA	NA	NA	T	BN	NA	MODERATE	N	0.27	268	267	0	234	183	51	21.79
Chewer	IOB_07	chr15	42983228	C	A	STARD9	MISSENSE	ENSG00000159433:[ENST00000290607(c.9452C>A,gCt>gAt):ENSP00000290607(p.Ala3151Asp)]	NA	NA	NA	NA	NA	NA	NA	D	PoD	NA	MODERATE	NA	NA	115	114	0	113	108	5	4.42
Chewer	IOB_07	chr15	48786466	G	A	FBN1	INTRONIC	ENSG00000166147:[ENST00000537463(c.637-19619C>T)]	NA	NA	3.20E-03	1.01E-03	1.22E-04	3.19E-03	0.00E+00	NA	NA	NA	MODIFIER	NA	NA	49	49	0	58	47	10	17.24
Chewer	IOB_07	chr15	49903452	G	A	FAM227B	MISSENSE	ENSG00000166262:[ENST00000299338(c.65C>T,cCt>cTt):ENSP00000299338(p.Pro22Leu)]	NA	NA	NA	NA	NA	NA	NA	NA	NA	NA	MODIFIER	NA	NA	66	66	0	74	55	19	25.68
Chewer	IOB_07	chr15	65394522	G	A	UBAP1L	SILENT	ENSG00000246922:[ENST00000502113(c.621C>T,ccC>ccT):ENSP00000440243(p.Pro207(=))]	NA	NA	3.99E-04	NA	NA	3.99E-04	1.00E-03	NA	NA	NA	MODIFIER	NA	NA	23	23	0	11	6	5	45.45
Chewer	IOB_07	chr15	73076086	C	T	ADPGK	5UTR	ENSG00000159322:[ENST00000311669(c.-54G>A)]	NA	NA	NA	NA	NA	NA	NA	NA	NA	NA	MODIFIER	NA	NA	220	219	0	235	175	60	25.53
Chewer	IOB_07	chr15	73076086	C	T	ADPGK-AS1	EXONIC-NC	ENSG00000260898:[ENST00000563592(n.911C>T)]	NA	NA	NA	NA	NA	NA	NA	NA	NA	NA	MODIFIER	NA	NA	220	219	0	235	175	60	25.53
Chewer	IOB_07	chr15	75015161	C	T	CYP1A1	MISSENSE	ENSG00000140465:[ENST00000379727(c.278G>A,cGg>cAg):ENSP00000369050(p.Arg93Gln)]	NA	NA	2.20E-03	2.57E-04	0.00E+00	2.20E-03	0.00E+00	D	BN	NA	MODERATE	NA	NA	189	188	0	176	139	37	21.02
Chewer	IOB_07	chr15	85383164	C	T	ALPK3	SILENT	ENSG00000136383:[ENST00000258888(c.1260C>T,ccC>ccT):ENSP00000258888(p.Pro420(=))]	NA	NA	chr15*85383164*85383164*C*G*rs145729582*NA*NA*NA	NA	NA	NA	NA	NA	NA	NA	LOW	NA	NA	146	146	0	115	82	33	28.7
Chewer	IOB_07	chr15	86270337	T	G	AKAP13	MISSENSE	ENSG00000170776:[ENST00000394518(c.6863T>G,gTg>gGg):ENSP00000378026(p.Val2288Gly)]	NA	NA	NA	NA	NA	NA	NA	NA	NA	NA	MODERATE	NA	NA	205	204	1	150	120	30	20
Chewer	IOB_07	chr15	86270337	T	G	RP11-158M2.2	EXONIC-NC	ENSG00000259544:[ENST00000561417(n.122A>C)]	NA	NA	NA	NA	NA	NA	NA	NA	NA	NA	MODERATE	NA	NA	205	204	1	150	120	30	20
Chewer	IOB_07	chr15	92459282	G	A	SLCO3A1	SILENT	ENSG00000176463:[ENST00000553304(c.66G>A,gtG>gtA):ENSP00000450559(p.Val22(=))]	NA	NA	NA	8.43E-06	0.00E+00	NA	NA	NA	NA	NA	LOW	NA	NA	205	205	0	234	195	38	16.24
Chewer	IOB_07	chr15	98417273	G	A	LINC00923	SILENT	ENSG00000251209:[ENST00000503768(c.243C>T,ttC>ttT):ENSP00000453315(p.Phe81(=))]	NA	NA	NA	NA	NA	NA	NA	NA	NA	NA	MODIFIER	NA	NA	112	112	0	88	62	26	29.55
Chewer	IOB_07	chr15	101514230	C	T	LRRK1	EXONIC-NC	ENSG00000154237:[ENST00000527698(n.556C>T)]	NA	NA	NA	NA	NA	NA	NA	NA	NA	NA	MODIFIER	NA	NA	30	30	0	43	33	10	23.26
Chewer	IOB_07	chr16	613835	G	T	C16orf11	MISSENSE	ENSG00000161992:[ENST00000409413(c.541G>T,Gca>Tca):ENSP00000386499(p.Ala181Ser)]	NA	NA	NA	NA	NA	NA	NA	NA	NA	NA	MODIFIER	NA	NA	104	103	1	117	91	26	22.22
Chewer	IOB_07	chr16	667375	G	C	RAB40C	INTRONIC	ENSG00000197562:[ENST00000509637(c.*182+107G>C)]	NA	NA	NA	NA	NA	NA	NA	NA	NA	NA	MODIFIER	NA	NA	54	54	0	44	32	12	27.27
Chewer	IOB_07	chr16	716061	G	A	WDR90	MISSENSE	ENSG00000161996:[ENST00000549091(c.4552G>A,Gcc>Acc):ENSP00000448122(p.Ala1518Thr)]	NA	NA	NA	NA	NA	NA	NA	T	BN	NA	MODERATE	N	1	166	165	0	200	155	42	21
Chewer	IOB_07	chr16	716290	C	T	WDR90	SILENT	ENSG00000161996:[ENST00000549091(c.4686C>T,ttC>ttT):ENSP00000448122(p.Phe1562(=))]	NA	NA	NA	NA	NA	NA	NA	NA	NA	NA	LOW	NA	0.001	304	303	1	312	233	78	25
Chewer	IOB_07	chr16	2367319	G	A	ABCA3	MISSENSE	ENSG00000167972:[ENST00000301732(c.1076C>T,tCc>tTc):ENSP00000301732(p.Ser359Phe)]	NA	NA	NA	NA	NA	NA	NA	T	BN	NA	MODERATE	N	1	256	254	0	197	149	47	23.86
Chewer	IOB_07	chr16	2550856	G	A	RP11-20I23.1	INTRONIC	ENSG00000260272:[ENST00000564543(c.965+3742G>A)]	NA	NA	NA	3.54E-05	0.00E+00	NA	NA	T	BN	NA	MODERATE	D	1	341	340	1	355	341	11	3.1
Chewer	IOB_07	chr16	2550856	G	A	TBC1D24	MISSENSE	ENSG00000162065:[ENST00000564879(c.446G>A,cGc>cAc):ENSP00000457266(p.Arg149His)]	NA	NA	NA	3.54E-05	0.00E+00	NA	NA	T	BN	NA	MODERATE	D	1	341	340	1	355	341	11	3.1
Chewer	IOB_07	chr16	15489846	G	A	MPV17L	MISSENSE	ENSG00000156968:[ENST00000287594(c.92G>A,gGg>gAg):ENSP00000287594(p.Gly31Glu)]	NA	NA	NA	NA	NA	NA	NA	D	PrD	NA	MODERATE	NA	NA	44	44	0	39	30	9	23.08
Chewer	IOB_07	chr16	15489846	G	A	RP11-1021N1.1	MISSENSE	ENSG00000261130:[ENST00000567442(c.40G>A,gGg>gAg):ENSP00000455081(p.Gly14Glu)]	NA	NA	NA	NA	NA	NA	NA	D	PrD	NA	MODERATE	NA	NA	44	44	0	39	30	9	23.08
Chewer	IOB_07	chr16	22268178	C	G	EEF2K	MISSENSE	ENSG00000103319:[ENST00000263026(c.728C>G,tCt>tGt):ENSP00000263026(p.Ser243Cys)]	NA	chr16*22268178*22268178*COSM1250962*C*T*1*oesophagus*1|chr16*22268178*22268178*COSM1250961*C*T*1*oesophagus*1	NA	NA	NA	NA	NA	D	PoD	NA	MODERATE	NA	NA	78	78	0	66	53	13	19.7
Chewer	IOB_07	chr16	24675283	G	A	AC012317.1	EXONIC-NC	ENSG00000224310:[ENST00000414816(n.367C>T)]	NA	NA	NA	NA	NA	NA	NA	NA	NA	NA	MODIFIER	NA	NA	61	61	0	33	24	9	27.27
Chewer	IOB_07	chr16	24981872	T	G	ARHGAP17	MISSENSE	ENSG00000140750:[ENST00000289968(c.228A>C,caA>caC):ENSP00000289968(p.Gln76His)]	NA	NA	NA	NA	NA	NA	NA	NA	NA	NA	MODIFIER	NA	NA	88	88	0	68	56	12	17.65
Chewer	IOB_07	chr16	25143783	A	C	LCMT1	MISSENSE	ENSG00000205629:[ENST00000564011(c.270A>C,agA>agC):ENSP00000457741(p.Arg90Ser)]	NA	NA	chr16*25143783*25143783*A*G*rs372904846*NA*NA*NA	NA	NA	NA	NA	T	BN	NA	MODERATE	NA	NA	178	177	0	167	122	44	26.35
Chewer	IOB_07	chr16	31384556	C	T	ITGAX	NCT	ENSG00000140678:[ENST00000571644(n.2373+565C>T)]	NA	NA	NA	NA	NA	NA	NA	NA	NA	NA	MODIFIER	NA	NA	24	24	0	39	32	7	17.95
Chewer	IOB_07	chr16	70894087	T	C	HYDIN	MISSENSE	ENSG00000157423:[ENST00000393567(c.12013A>G,Acc>Gcc):ENSP00000377197(p.Thr4005Ala)]	NA	NA	NA	NA	NA	NA	NA	NA	NA	NA	MODIFIER	NA	NA	28	26	1	38	28	10	26.32
Chewer	IOB_07	chr16	72162546	G	T	PMFBP1	INTRONIC-SS-DNR-PRX	ENSG00000118557:[ENST00000537392(c.1497+10C>A)]	NA	NA	NA	NA	NA	NA	NA	NA	NA	NA	MODIFIER	NA	NA	44	44	0	62	30	32	51.61
Chewer	IOB_07	chr16	74923754	G	A	WDR59	INTRONIC	79726:[XM_005256146.1(c.2140-41C>T)]	NA	NA	NA	NA	NA	NA	NA	NA	NA	NA	MODIFIER	NA	NA	22	22	0	27	13	13	48.15
Chewer	IOB_07	chr16	81916985	C	T	PLCG2	INTRONIC	ENSG00000197943:[ENST00000563193(c.73+39C>T)]	NA	NA	9.98E-04	2.02E-04	0.00E+00	9.98E-04	0.00E+00	NA	NA	NA	MODIFIER	NA	NA	104	104	0	104	95	6	5.77
Chewer	IOB_07	chr16	84014369	C	T	NECAB2	INTRONIC	ENSG00000103154:[ENST00000565691(c.113-266C>T)]	NA	NA	NA	NA	NA	NA	NA	NA	NA	NA	MODIFIER	NA	NA	104	104	0	104	50	54	51.92
Chewer	IOB_07	chr16	88793946	G	A	PIEZO1	INTRONIC	ENSG00000103335:[ENST00000301015(c.3301+19C>T)]	NA	NA	NA	NA	NA	NA	NA	NA	NA	NA	MODIFIER	NA	NA	83	77	0	105	46	57	54.29
Chewer	IOB_07	chr17	6946091	G	A	SLC16A11	NCT	ENSG00000174326:[ENST00000573338(n.677+158C>T)]	NA	NA	NA	NA	NA	NA	NA	NA	NA	NA	MODIFIER	NA	NA	73	73	0	73	55	18	24.66
Chewer	IOB_07	chr17	7188346	C	T	SLC2A4	INTRONIC	ENSG00000181856:[ENST00000572485(c.1081+27C>T)]	NA	NA	chr17*7188346*7188346*C*G,T*rs761081733*NA*NA*NA	8.52E-05	0.00E+00	NA	NA	NA	NA	NA	MODIFIER	NA	NA	106	105	0	90	71	18	20
Chewer	IOB_07	chr17	7460131	G	A	TNFSF12	MISSENSE	ENSG00000239697:[ENST00000293825(c.410G>A,aGa>aAa):ENSP00000293825(p.Arg137Lys)]	NA	NA	NA	NA	NA	NA	NA	T_lc	BN	NA	MODERATE	NA	1	108	108	0	76	52	23	30.26
Chewer	IOB_07	chr17	7460131	G	A	TNFSF12-TNFSF13	MISSENSE	ENSG00000248871:[ENST00000293826(c.410G>A,aGa>aAa):ENSP00000293826(p.Arg137Lys)]	NA	NA	NA	NA	NA	NA	NA	T_lc	BN	NA	MODERATE	NA	1	108	108	0	76	52	23	30.26
Chewer	IOB_07	chr17	17035082	C	T	MPRIP	INTRONIC	23164:[XM_005256564.1(c.600+67C>T)]	NA	NA	NA	NA	NA	NA	NA	NA	NA	NA	MODIFIER	NA	NA	40	40	0	47	34	12	25.53
Chewer	IOB_07	chr17	18002492	C	T	DRG2	INTRONIC	ENSG00000108591:[ENST00000582314(c.*98+101C>T)]	NA	NA	NA	NA	NA	NA	NA	NA	NA	NA	MODIFIER	NA	NA	79	77	0	68	56	12	17.65
Chewer	IOB_07	chr17	26073151	C	A	NA	INTERGENIC	NA	NA	NA	NA	NA	NA	NA	NA	NA	NA	NA	MODIFIER	NA	NA	57	57	0	28	22	5	17.86
Chewer	IOB_07	chr17	33255991	G	A	CCT6B	MISSENSE	ENSG00000132141:[ENST00000421975(c.1402C>T,Ctt>Ttt):ENSP00000398044(p.Leu468Phe)]	NA	chr17*33255991*33255991*COSM1382371*G*T*1*large_intestine*1	NA	NA	NA	NA	NA	T	BN	NA	MODERATE	D	1	67	67	0	46	35	11	23.91
Chewer	IOB_07	chr17	33496822	C	A	UNC45B	INTRONIC	ENSG00000141161:[ENST00000268876(c.1453-34C>A)]	NA	NA	chr17*33496822*33496822*C*T*rs376919199*NA*NA*NA	NA	NA	NA	NA	NA	NA	NA	MODIFIER	NA	NA	74	73	0	96	89	6	6.25
Chewer	IOB_07	chr17	35479488	G	A	ACACA	MISSENSE	ENSG00000132142:[ENST00000360679(c.5818C>T,Cca>Tca):ENSP00000353898(p.Pro1940Ser)]	NA	NA	NA	NA	NA	NA	NA	NA	NA	NA	MODIFIER	NA	NA	116	115	0	109	79	29	26.61
Chewer	IOB_07	chr17	37381815	G	A	STAC2	5UTR	ENSG00000141750:[ENST00000333461(c.-60C>T)]	NA	NA	NA	NA	NA	NA	NA	NA	NA	NA	MODIFIER	NA	NA	59	57	0	69	44	25	36.23
Chewer	IOB_07	chr17	37783643	G	A	PPP1R1B	5UTR	ENSG00000131771:[ENST00000254079(c.-3G>A)]	NA	NA	NA	NA	NA	NA	NA	NA	NA	NA	MODIFIER	NA	NA	209	208	0	227	214	7	3.08
Chewer	IOB_07	chr17	39346662	C	T	KRTAP9-1	MISSENSE	ENSG00000240542:[ENST00000398470(c.524C>T,aCc>aTc):ENSP00000381488(p.Thr175Ile)]	NA	NA	chr17*39346662*39346662*C*G*rs753376477*NA*NA*NA	NA	NA	NA	NA	NA	NA	NA	MODIFIER	NA	NA	344	340	0	307	240	61	19.87
Chewer	IOB_07	chr17	39346663	C	T	KRTAP9-1	SILENT	ENSG00000240542:[ENST00000398470(c.525C>T,acC>acT):ENSP00000381488(p.Thr175(=))]	NA	NA	NA	NA	NA	NA	NA	NA	NA	NA	MODIFIER	NA	NA	344	340	0	311	243	63	20.26
Chewer	IOB_07	chr17	40864450	G	A	EZH1	MISSENSE	ENSG00000108799:[ENST00000428826(c.1258C>T,Cct>Tct):ENSP00000404658(p.Pro420Ser)]	NA	NA	NA	NA	NA	NA	NA	NA	NA	NA	MODIFIER	NA	NA	44	42	0	51	34	16	31.37
Chewer	IOB_07	chr17	42390813	A	G	AC003102.3	NCT	ENSG00000267750:[ENST00000588097(n.154+1717T>C)]	NA	NA	NA	NA	NA	NA	NA	D	PrD	NA	MODERATE	D	1	137	134	0	121	101	20	16.53
Chewer	IOB_07	chr17	42390813	A	G	RUNDC3A	MISSENSE	ENSG00000108309:[ENST00000225441(c.400A>G,Atg>Gtg):ENSP00000225441(p.Met134Val)]	NA	NA	NA	NA	NA	NA	NA	D	PrD	NA	MODERATE	D	1	137	134	0	121	101	20	16.53
Chewer	IOB_07	chr17	53027006	G	A	TOM1L1	INTRONIC	ENSG00000141198:[ENST00000571319(c.*912+22G>A)]	NA	NA	2.00E-04	1.66E-05	6.10E-05	2.00E-04	1.00E-03	NA	NA	NA	MODIFIER	NA	NA	189	188	1	207	200	6	2.9
Chewer	IOB_07	chr17	53392714	T	G	HLF	MISSENSE	ENSG00000108924:[ENST00000430986(c.323T>G,tTt>tGt):ENSP00000402496(p.Phe108Cys)]	NA	NA	NA	NA	NA	NA	NA	NA	NA	NA	MODIFIER	NA	NA	79	79	0	87	69	18	20.69
Chewer	IOB_07	chr17	56329242	C	A	LPO	INTRONIC	ENSG00000167419:[ENST00000580890(c.276-409C>A)]	NA	NA	NA	NA	NA	NA	NA	NA	NA	NA	MODIFIER	NA	NA	134	134	0	139	112	26	18.71
Chewer	IOB_07	chr17	56565646	G	A	HSF5	5UTR	ENSG00000176160:[ENST00000323777(c.-11C>T)]	NA	NA	NA	NA	NA	NA	NA	NA	NA	NA	MODIFIER	NA	NA	178	178	0	187	141	45	24.06
Chewer	IOB_07	chr17	58503275	C	T	C17orf64	INTRONIC	ENSG00000141371:[ENST00000474834(c.42+30C>T)]	NA	NA	NA	NA	NA	NA	NA	NA	NA	NA	MODIFIER	NA	NA	203	202	0	190	163	26	13.68
Chewer	IOB_07	chr17	58503276	C	T	C17orf64	INTRONIC	124773:[XM_005257034.1(c.252+31C>T)]	NA	NA	NA	NA	NA	NA	NA	NA	NA	NA	MODIFIER	NA	NA	203	201	0	191	164	26	13.61
Chewer	IOB_07	chr17	60003936	C	A	INTS2	MISSENSE	ENSG00000108506:[ENST00000251334(c.70G>T,Gtt>Ttt):ENSP00000251334(p.Val24Phe)]	NA	NA	NA	NA	NA	NA	NA	T	PoD	NA	MODERATE	D	1	124	124	0	119	114	5	4.2
Chewer	IOB_07	chr17	62076160	C	T	C17orf72	INTRONIC	ENSG00000224383:[ENST00000412177(c.136+18C>T)]	NA	NA	NA	NA	NA	NA	NA	NA	NA	NA	MODIFIER	NA	NA	51	47	0	27	20	5	18.52
Chewer	IOB_07	chr17	62076160	C	T	RP11-214C8.2	NCT	ENSG00000264954:[ENST00000577545(n.99+33G>A)]	NA	NA	NA	NA	NA	NA	NA	NA	NA	NA	MODIFIER	NA	NA	51	47	0	27	20	5	18.52
Chewer	IOB_07	chr17	71391469	G	A	SDK2	SILENT	ENSG00000069188:[ENST00000424778(c.945C>T,agC>agT):ENSP00000407098(p.Ser315(=))]	NA	NA	NA	NA	NA	NA	NA	NA	NA	NA	LOW	NA	0.261	191	191	0	183	178	5	2.73
Chewer	IOB_07	chr17	72915626	G	A	USH1G	SILENT	ENSG00000182040:[ENST00000319642(c.1305C>T,ccC>ccT):ENSP00000320076(p.Pro435(=))]	NA	NA	NA	NA	NA	NA	NA	NA	NA	NA	MODIFIER	NA	NA	58	57	0	75	61	14	18.67
Chewer	IOB_07	chr17	74472995	C	T	RHBDF2	SILENT	ENSG00000129667:[ENST00000389760(c.1032G>A,gtG>gtA):ENSP00000374410(p.Val344(=))]	NA	NA	chr17*74472995*74472995*C*G*rs369565878*NA*NA*NA	NA	NA	NA	NA	NA	NA	NA	LOW	NA	NA	116	115	0	89	73	16	17.98
Chewer	IOB_07	chr18	19034544	G	A	GREB1L	INTRONIC	ENSG00000141449:[ENST00000269218(c.1855+20G>A)]	NA	NA	NA	NA	NA	NA	NA	NA	NA	NA	MODIFIER	NA	NA	143	140	0	113	92	19	16.81
Chewer	IOB_07	chr18	29104495	C	G	DSG2	MISSENSE	ENSG00000046604:[ENST00000261590(c.775C>G,Cag>Gag):ENSP00000261590(p.Gln259Glu)]	NA	NA	NA	NA	NA	NA	NA	T	BN	NA	MODERATE	D	0.989	107	107	0	126	99	27	21.43

Patel *et al.* , 2021. Whole-exome sequencing analysis of oral squamous cell carcinoma delineated by tobacco usage habits
Supplementary Table 4. List of somatic single nucleotide variants identified using whole exome sequencing of OSCC patients

Cohort	Sample	Chromosome	Genomic position	Reference allele	Alternate allele	Genesymbol	Variant type	Variant description	OncoMD	COSMIC	dbSNP	ExAC	ExAC_SAS	1000G	1000G_SAS	SIFT	Polyphen2	CONDEL	VEP	LRT	PhastCon7Way	Total reads in Normal	Reference read support in Normal	Alternative read support in Normal	Total reads in Tumor	Reference read support in Tumor	Alternate read support in Tumor	Alternate allele frequency in Tumor
Chewer	IOB_07	chr18	29672694	C	A	RNF138	EXONIC-NC	ENSG00000134758:[ENST00000585103(n.384C>A)]	NA	NA	NA	NA	NA	NA	NA	NA	NA	NA	MODIFIER	NA	NA	267	267	0	311	239	70	22.51
Chewer	IOB_07	chr18	29672694	C	A	RP11-53I6.2	NCT	ENSG00000263917:[ENST00000583184(n.540-19023C>A)]	NA	NA	NA	NA	NA	NA	NA	NA	NA	NA	MODIFIER	NA	NA	267	267	0	311	239	70	22.51
Chewer	IOB_07	chr18	31250729	T	C	ASXL3	SILENT	ENSG00000141431:[ENST00000592288(c.573T>C,tcT>tcC):ENSP00000465053(p.Ser191(=))]	NA	NA	NA	NA	NA	NA	NA	NA	NA	NA	LOW	NA	NA	162	160	0	144	122	22	15.28
Chewer	IOB_07	chr18	44580851	G	A	KATNAL2	INTRONIC	83473:[XM_005258360.1(c.113+42G>A)]	NA	NA	NA	NA	NA	NA	NA	NA	NA	NA	MODIFIER	NA	NA	122	122	0	90	70	20	22.22
Chewer	IOB_07	chr18	67534608	G	A	CD226	SILENT	ENSG00000150637:[ENST00000280200(c.870C>T,tcC>tcT):ENSP00000280200(p.Ser290(=))]	NA	NA	NA	NA	NA	NA	NA	NA	NA	NA	LOW	NA	NA	62	62	0	39	34	5	12.82
Chewer	IOB_07	chr19	541483	C	T	CDC34	SILENT	ENSG00000099804:[ENST00000607527(c.246C>T,ggC>ggT):ENSP00000476107(p.Gly82(=))]	NA	NA	NA	1.71E-05	0.00E+00	NA	NA	NA	NA	NA	LOW	NA	NA	205	205	0	166	129	37	22.29
Chewer	IOB_07	chr19	1104815	C	T	GPX4	MISSENSE	ENSG00000167468:[ENST00000585362(c.82C>T,Cgg>Tgg):ENSP00000473614(p.Arg28Trp)]	NA	NA	chr19*1104814*1104817*TCGG*T*rs771492860*NA*NA*NA	NA	NA	NA	NA	NA	NA	NA	MODIFIER	NA	NA	53	53	0	107	72	35	32.71
Chewer	IOB_07	chr19	1110766	G	A	SBNO2	SILENT	ENSG00000064932:[ENST00000438103(c.2835C>T,ggC>ggT):ENSP00000400762(p.Gly945(=))]	NA	NA	NA	NA	NA	NA	NA	NA	NA	NA	MODIFIER	NA	NA	133	132	0	129	82	45	34.88
Chewer	IOB_07	chr19	1122256	G	A	SBNO2	MISSENSE	ENSG00000064932:[ENST00000438103(c.860C>T,tCa>tTa):ENSP00000400762(p.Ser287Leu)]	NA	NA	NA	NA	NA	NA	NA	D	PrD	NA	MODERATE	D	1	419	416	1	442	354	85	19.23
Chewer	IOB_07	chr19	1122473	G	A	SBNO2	SILENT	ENSG00000064932:[ENST00000361757(c.999C>T,ctC>ctT):ENSP00000354733(p.Leu333(=))]	NA	NA	NA	NA	NA	NA	NA	NA	NA	NA	LOW	NA	NA	292	292	0	287	218	69	24.04
Chewer	IOB_07	chr19	1122746	G	A	SBNO2	SILENT	ENSG00000064932:[ENST00000438103(c.654C>T,ctC>ctT):ENSP00000400762(p.Leu218(=))]	NA	NA	NA	NA	NA	NA	NA	NA	NA	NA	LOW	NA	NA	221	219	0	199	163	35	17.59
Chewer	IOB_07	chr19	3366688	G	C	NFIC	INTRONIC	ENSG00000141905:[ENST00000346156(c.3+7005G>C)]	NA	NA	chr19*3366677*3366688*CCCGGCCCCCCCG*C*rs755482235*NA*NA*NA|chr19*3366680*3366701*GGCCCCCCGCCGGCGCCCCCGC*G*rs779597561*NA*NA*N	NA	NA	NA	NA	NA	NA	NA	MODIFIER	NA	NA	126	123	3	165	147	12	7.27
Chewer	IOB_07	chr19	4110701	G	A	MAP2K2	INTRONIC	ENSG00000126934:[ENST00000262948(c.304-48C>T)]	NA	NA	NA	NA	NA	NA	NA	NA	NA	NA	MODIFIER	NA	NA	106	106	0	73	55	15	20.55
Chewer	IOB_07	chr19	11319475	C	T	CTC-510F12.2	NCT	ENSG00000267082:[ENST00000588634(n.537+2691C>T)]	NA	NA	NA	8.30E-06	6.06E-05	NA	NA	D	PrD	NA	MODERATE	NA	NA	311	311	0	243	234	7	2.88
Chewer	IOB_07	chr19	11319475	C	T	DOCK6	MISSENSE	ENSG00000130158:[ENST00000319867(c.2992G>A,Gcc>Acc):ENSP00000321556(p.Ala998Thr)]	NA	NA	NA	8.30E-06	6.06E-05	NA	NA	D	PrD	NA	MODERATE	NA	NA	311	311	0	243	234	7	2.88
Chewer	IOB_07	chr19	12812880	A	T	TNPO2	INTRONIC-SS-DNR-PRX	ENSG00000105576:[ENST00000356861(c.2381+6T>A)]	NA	NA	NA	NA	NA	NA	NA	NA	NA	NA	LOW	NA	NA	379	377	0	341	256	83	24.34
Chewer	IOB_07	chr19	14875238	G	A	EMR2	INTRONIC-SS-DNR-PRX	ENSG00000127507:[ENST00000392964(c.301+7C>T)]	NA	NA	chr19*14875238*14875238*G*T*rs762489386*NA*NA*NA	NA	NA	NA	NA	NA	NA	NA	LOW	NA	NA	118	118	0	107	92	15	14.02
Chewer	IOB_07	chr19	15989696	G	C	CYP4F2	MISSENSE	ENSG00000186115:[ENST00000221700(c.1448C>G,gCg>gGg):ENSP00000221700(p.Ala483Gly)]	NA	chr19*15989696*15989696*COSM225400*G*C*3*NS,large_intestine*1.2	chr19*15989696*15989696*G*A,C*rs3952537*NA*NA*NA	9.51E-02	3.82E-02	NA	NA	NA	NA	NA	MODIFIER	NA	NA	58	56	2	48	38	10	20.83
Chewer	IOB_07	chr19	16539448	C	A	EPS15L1	INTRONIC	ENSG00000127527:[ENST00000592031(c.*295+65G>T)]	NA	NA	NA	NA	NA	NA	NA	NA	NA	NA	MODIFIER	NA	NA	78	78	0	44	33	11	25
Chewer	IOB_07	chr19	17741453	G	T	UNC13A	INTRONIC-SS-DNR-PRX	ENSG00000130477:[ENST00000252773(c.3525+10C>A)]	NA	NA	NA	NA	NA	NA	NA	NA	NA	NA	MODIFIER	NA	NA	127	127	0	141	136	5	3.55
Chewer	IOB_07	chr19	18497467	G	A	GDF15	INTRONIC	ENSG00000130513:[ENST00000252809(c.277+191G>A)]	NA	NA	NA	NA	NA	NA	NA	NA	NA	NA	MODIFIER	NA	NA	48	48	0	53	43	10	18.87
Chewer	IOB_07	chr19	19788795	G	A	ZNF101	SILENT	ENSG00000181896:[ENST00000318110(c.126G>A,tcG>tcA):ENSP00000319716(p.Ser42(=))]	NA	NA	NA	3.71E-04	0.00E+00	NA	NA	NA	NA	NA	MODIFIER	NA	NA	91	91	0	105	99	6	5.71
Chewer	IOB_07	chr19	30019301	C	A	VSTM2B	INTRONIC	ENSG00000187135:[ENST00000335523(c.268-39C>A)]	NA	NA	NA	NA	NA	NA	NA	NA	NA	NA	MODIFIER	NA	NA	127	127	0	151	144	5	3.31
Chewer	IOB_07	chr19	35251282	G	A	ZNF599	MISSENSE	ENSG00000153896:[ENST00000329285(c.424C>T,Cac>Tac):ENSP00000333802(p.His142Tyr)]	NA	NA	NA	4.12E-05	0.00E+00	NA	NA	T	BN	NA	MODERATE	NA	NA	150	150	0	194	150	42	21.65
Chewer	IOB_07	chr19	36230896	C	A	AD000671.6	3UTR	ENSG00000267120:[ENST00000589807(c.*930G>T)]	NA	NA	NA	NA	NA	NA	NA	NA	NA	NA	MODIFIER	NA	NA	178	178	0	158	150	6	3.8
Chewer	IOB_07	chr19	36230896	C	A	IGFLR1	MISSENSE	ENSG00000126246:[ENST00000246532(c.436G>T,Gcc>Tcc):ENSP00000246532(p.Ala146Ser)]	NA	NA	NA	NA	NA	NA	NA	NA	NA	NA	MODIFIER	NA	NA	178	178	0	158	150	6	3.8
Chewer	IOB_07	chr19	36370054	G	A	APLP1	MISSENSE	ENSG00000105290:[ENST00000587274(c.705G>A,Gtc>Atc):ENSP00000474762(p.Val236Ile)]	NA	NA	NA	NA	NA	NA	NA	T	BN	NA	MODERATE	NA	NA	178	176	1	140	102	38	27.14
Chewer	IOB_07	chr19	39759982	G	C	IFNL2	INTRONIC	ENSG00000183709:[ENST00000331982(c.270+113G>C)]	NA	NA	NA	NA	NA	NA	NA	NA	NA	NA	MODIFIER	NA	NA	145	141	1	110	87	22	20
Chewer	IOB_07	chr19	42410848	C	T	ARHGEF1	INTRONIC	9138:[XM_005259388.1(c.2672-7C>T)]	NA	NA	NA	NA	NA	NA	NA	NA	NA	NA	LOW	NA	NA	77	77	0	84	69	15	17.86
Chewer	IOB_07	chr19	45026778	C	T	CEACAM20	SILENT	125931:[NM_001102600.2(c.636G>A,acG>acA):NP_001096070.2(p.Thr212(=))]	NA	NA	NA	NA	NA	NA	NA	NA	NA	NA	MODIFIER	NA	NA	118	118	0	140	125	15	10.71
Chewer	IOB_07	chr19	45721619	A	C	EXOC3L2	INTRONIC	ENSG00000130201:[ENST00000252482(c.541-42T>G)]	NA	NA	chr19*45721619*45721619*A*T*rs770432407*NA*NA*NA	NA	NA	NA	NA	NA	NA	NA	MODIFIER	NA	NA	20	19	1	15	8	6	40
Chewer	IOB_07	chr19	45721619	A	C	MARK4	INTRONIC	ENSG00000007047:[ENST00000587566(c.-276-40628A>C)]	NA	NA	chr19*45721619*45721619*A*T*rs770432407*NA*NA*NA	NA	NA	NA	NA	NA	NA	NA	MODIFIER	NA	NA	20	19	1	15	8	6	40
Chewer	IOB_07	chr19	47588308	C	A	ZC3H4	INTRONIC-SS-DNR-PRX	ENSG00000130749:[ENST00000253048(c.1107+5G>T)]	NA	NA	NA	NA	NA	NA	NA	NA	NA	NA	MODIFIER	NA	NA	182	182	0	180	175	5	2.78
Chewer	IOB_07	chr19	47933291	G	A	SLC8A2	3UTR	ENSG00000118160:[ENST00000236877(c.*55C>T)]	NA	NA	NA	NA	NA	NA	NA	NA	NA	NA	MODIFIER	NA	NA	80	80	0	87	59	28	32.18
Chewer	IOB_07	chr19	49183627	G	C	SEC1P	EXONIC-NC	ENSG00000232871:[ENST00000473944(n.290G>C)]	NA	NA	NA	NA	NA	NA	NA	NA	NA	NA	MODIFIER	NA	NA	150	150	0	117	85	32	27.35
Chewer	IOB_07	chr19	49388729	C	T	TULP2	MISSENSE	ENSG00000104804:[ENST00000221399(c.1037G>A,gGg>gAg):ENSP00000221399(p.Gly346Glu)]	NA	NA	NA	NA	NA	NA	NA	D	PrD	NA	MODERATE	D	1	192	189	0	168	127	38	22.62
Chewer	IOB_07	chr19	50249687	C	T	TSKS	INTRONIC	ENSG00000126467:[ENST00000358830(c.392+40G>A)]	NA	NA	chr19*50249687*50249687*C*A*rs771812166*NA*NA*NA	NA	NA	NA	NA	NA	NA	NA	MODIFIER	NA	NA	208	205	1	204	159	45	22.06
Chewer	IOB_07	chr19	50912386	G	A	POLD1	MISSENSE	ENSG00000062822:[ENST00000440232(c.1900G>A,Gag>Aag):ENSP00000406046(p.Glu634Lys)]	NA	NA	NA	NA	NA	NA	NA	T	BN	NA	MODERATE	N	0.001	258	256	0	200	145	53	26.5
Chewer	IOB_07	chr19	51205831	C	T	SHANK1	MISSENSE	ENSG00000161681:[ENST00000293441(c.1640G>A,cGg>cAg):ENSP00000293441(p.Arg547Gln)]	NA	NA	9.98E-04	2.92E-04	2.23E-04	9.98E-04	0.00E+00	D	BN	NA	MODERATE	U	0	153	152	1	157	120	37	23.57
Chewer	IOB_07	chr19	52569713	C	G	ZNF432	NCT	ENSG00000256087:[ENST00000598446(n.165-16953G>C)]	NA	NA	NA	NA	NA	NA	NA	NA	NA	NA	LOW	NA	NA	107	107	0	88	74	13	14.77
Chewer	IOB_07	chr19	52569713	C	G	ZNF841	SILENT	ENSG00000197608:[ENST00000389534(c.1422G>C,ggG>ggC):ENSP00000374185(p.Gly474(=))]	NA	NA	NA	NA	NA	NA	NA	NA	NA	NA	LOW	NA	NA	107	107	0	88	74	13	14.77
Chewer	IOB_07	chr19	54297337	C	T	NLRP12	MISSENSE	ENSG00000142405:[ENST00000391775(c.2981G>A,cGa>cAa):ENSP00000375655(p.Arg994Gln)]	NA	chr19*54297337*54297337*COSM1396137*C*T*1*large_intestine*1	chr19*54297337*54297337*C*G,T*rs777108086*NA*NA*NA	8.24E-06	0.00E+00	NA	NA	T	BN	NA	MODERATE	NA	NA	180	179	1	133	107	26	19.55
Chewer	IOB_07	chr19	55143014	T	G	LILRB1	MISSENSE	ENSG00000104972:[ENST00000427581(c.242T>G,gTg>gGg):ENSP00000395004(p.Val81Gly)]	NA	chr19*55143014*55143014*COSM1213377*T*C*1*large_intestine*1	NA	NA	NA	NA	NA	NA	NA	NA	MODIFIER	NA	NA	115	115	0	126	89	35	27.78
Chewer	IOB_07	chr19	55325392	C	A	KIR2DL4	MISSENSE	ENSG00000189013:[ENST00000346587(c.570C>A,agC>agA):ENSP00000345331(p.Ser190Arg)]	NA	NA	NA	NA	NA	NA	NA	NA	NA	NA	MODIFIER	NA	NA	160	159	0	168	144	23	13.69
Chewer	IOB_07	chr19	55325392	C	A	KIR3DL1	INTRONIC	ENSG00000167633:[ENST00000402254(c.35-3597C>A)]	NA	NA	NA	NA	NA	NA	NA	NA	NA	NA	MODIFIER	NA	NA	160	159	0	168	144	23	13.69
Chewer	IOB_07	chr19	57931294	G	A	AC003002.6	MISSENSE	ENSG00000269533:[ENST00000596400(c.470G>A,aGg>aAg):ENSP00000472277(p.Arg157Lys)]	NA	NA	NA	8.26E-06	0.00E+00	NA	NA	NA	NA	NA	MODIFIER	NA	NA	125	125	0	103	81	22	21.36
Chewer	IOB_07	chr19	57931294	G	A	AC004076.7	INTRONIC	ENSG00000268533:[ENST00000597410(c.42-8533G>A)]	NA	NA	NA	8.26E-06	0.00E+00	NA	NA	NA	NA	NA	MODIFIER	NA	NA	125	125	0	103	81	22	21.36
Chewer	IOB_07	chr19	57931294	G	A	ZNF17	MISSENSE	ENSG00000186272:[ENST00000307658(c.440G>A,aGg>aAg):ENSP00000302455(p.Arg147Lys)]	NA	NA	NA	8.26E-06	0.00E+00	NA	NA	NA	NA	NA	MODIFIER	NA	NA	125	125	0	103	81	22	21.36
Chewer	IOB_07	chr19	58118199	G	A	ZNF530	MISSENSE	ENSG00000183647:[ENST00000332854(c.1306G>A,Gag>Aag):ENSP00000332861(p.Glu436Lys)]	NA	NA	NA	NA	NA	NA	NA	NA	NA	NA	MODIFIER	NA	NA	276	274	0	210	163	45	21.43
Chewer	IOB_07	chr19	58864746	G	A	A1BG	INTRONIC	ENSG00000121410:[ENST00000263100(c.34+24C>T)]	NA	NA	chr19*58864746*58864746*G*T*rs201594936*0*0.9998*0.0001997	NA	NA	NA	NA	NA	NA	NA	MODIFIER	NA	NA	131	128	0	129	107	15	11.63
Chewer	IOB_07	chr19	58864746	G	A	A1BG-AS1	EXONIC-NC	ENSG00000268895:[ENST00000599728(n.152G>A)]	NA	NA	chr19*58864746*58864746*G*T*rs201594936*0*0.9998*0.0001997	NA	NA	NA	NA	NA	NA	NA	MODIFIER	NA	NA	131	128	0	129	107	15	11.63
Chewer	IOB_07	chr19	58864746	G	A	CTD-2619J13.8	NCT	ENSG00000268230:[ENST00000599109(n.700-53C>T)]	NA	NA	chr19*58864746*58864746*G*T*rs201594936*0*0.9998*0.0001997	NA	NA	NA	NA	NA	NA	NA	MODIFIER	NA	NA	131	128	0	129	107	15	11.63
Chewer	IOB_07	chr2	10008583	C	T	TAF1B	NCT	ENSG00000115750:[ENST00000402170(n.613+25C>T)]	NA	NA	.	4.60E-04	0.00E+00	NA	NA	NA	NA	NA	MODIFIER	NA	NA	97	97	0	137	116	19	13.87
Chewer	IOB_07	chr2	20824606	G	A	HS1BP3	MISSENSE	ENSG00000118960:[ENST00000458740(c.127C>T,Ccc>Tcc):ENSP00000392203(p.Pro43Ser)]	NA	NA	NA	NA	NA	NA	NA	D	BN	NA	MODERATE	N	0.984	63	63	0	102	66	36	35.29
Chewer	IOB_07	chr2	28236098	C	T	BRE	INTRONIC	9577:[XM_005264652.1(c.133-11995C>T)]	NA	NA	NA	NA	NA	NA	NA	NA	NA	NA	MODIFIER	NA	NA	158	157	0	180	174	5	2.78
Chewer	IOB_07	chr2	29093061	C	T	TRMT61B	MISSENSE	ENSG00000171103:[ENST00000306108(c.83G>A,gGg>gAg):ENSP00000302801(p.Gly28Glu)]	NA	NA	NA	NA	NA	NA	NA	NA	NA	NA	MODIFIER	NA	NA	212	212	0	272	230	41	15.07
Chewer	IOB_07	chr2	32660720	G	A	BIRC6	INTRONIC	57448:[XM_005264454.1(c.3541+67G>A)]	NA	NA	NA	NA	NA	NA	NA	NA	NA	NA	MODIFIER	NA	NA	111	110	1	146	134	11	7.53
Chewer	IOB_07	chr2	70314890	G	C	PCBP1	SILENT	ENSG00000169564:[ENST00000303577(c.15G>C,gtG>gtC):ENSP00000305556(p.Val5(=))]	NA	NA	chr2*70314890*70314890*G*T*rs752253658*NA*NA*NA	NA	NA	NA	NA	NA	NA	NA	MODIFIER	NA	NA	66	65	0	82	65	16	19.51
Chewer	IOB_07	chr2	70314890	G	C	PCBP1-AS1	NCT	ENSG00000179818:[ENST00000423402(n.120+30C>G)]	NA	NA	chr2*70314890*70314890*G*T*rs752253658*NA*NA*NA	NA	NA	NA	NA	NA	NA	NA	MODIFIER	NA	NA	66	65	0	82	65	16	19.51
Chewer	IOB_07	chr2	73679487	G	A	ALMS1	MISSENSE	ENSG00000116127:[ENST00000423048(c.664G>A,Gag>Aag):ENSP00000399833(p.Glu222Lys)]	NA	NA	NA	NA	NA	NA	NA	D	BN	NA	MODERATE	NA	NA	171	170	0	231	150	81	35.06
Chewer	IOB_07	chr2	79386553	C	A	AC011754.1	NCT	ENSG00000224879:[ENST00000415201(n.153+901C>A)]	NA	NA	NA	NA	NA	NA	NA	NA	NA	NA	MODIFIER	NA	NA	114	112	0	138	113	20	14.49
Chewer	IOB_07	chr2	79386553	C	A	REG3A	NCT	ENSG00000172016:[ENST00000464746(n.18-11G>T)]	NA	NA	NA	NA	NA	NA	NA	NA	NA	NA	MODIFIER	NA	NA	114	112	0	138	113	20	14.49
Chewer	IOB_07	chr2	96259950	C	T	TRIM43	MISSENSE	ENSG00000144015:[ENST00000272395(c.179C>T,cCg>cTg):ENSP00000272395(p.Pro60Leu)]	NA	NA	NA	1.12E-05	0.00E+00	NA	NA	D	BN	NA	MODERATE	NA	NA	48	48	0	72	40	29	40.28
Chewer	IOB_07	chr2	102818090	G	A	IL1RL2	SILENT	ENSG00000115598:[ENST00000441515(c.213G>A,gaG>gaA):ENSP00000413348(p.Glu71(=))]	NA	NA	NA	NA	NA	NA	NA	NA	NA	NA	LOW	NA	0.997	69	69	0	99	73	25	25.25
Chewer	IOB_07	chr2	102958800	C	T	IL18R1	INTRONIC	ENSG00000115604:[ENST00000410040(c.-28-20293C>T)]	NA	NA	NA	NA	NA	NA	NA	NA	NA	NA	MODIFIER	NA	NA	57	57	0	82	66	16	19.51
Chewer	IOB_07	chr2	102958800	C	T	IL1RL1	NCT	ENSG00000115602:[ENST00000482701(n.515+46C>T)]	NA	NA	NA	NA	NA	NA	NA	NA	NA	NA	MODIFIER	NA	NA	57	57	0	82	66	16	19.51
Chewer	IOB_07	chr2	122494243	G	A	NIFK	NCT	ENSG00000155438:[ENST00000477693(n.127+79C>T)]	NA	NA	NA	NA	NA	NA	NA	NA	NA	NA	MODIFIER	NA	NA	149	149	0	155	127	28	18.06
Chewer	IOB_07	chr2	128935970	C	T	UGGT1	NCT	56886:[NR_027671.2(n.4045-72C>T)]	NA	NA	chr2*128935970*128935972*CCT*C*rs765923141*NA*NA*NA	NA	NA	NA	NA	NA	NA	NA	MODIFIER	NA	NA	22	22	0	48	28	19	39.58
Chewer	IOB_07	chr2	167262274	C	T	SCN7A	MISSENSE	ENSG00000136546:[ENST00000409855(c.4865G>A,cGa>cAa):ENSP00000386796(p.Arg1622Gln)]	NA	NA	9.98E-04	1.54E-03	3.03E-04	9.98E-04	1.00E-03	NA	NA	NA	MODIFIER	NA	NA	251	251	0	361	292	66	18.28
Chewer	IOB_07	chr2	167304163	G	A	SCN7A	MISSENSE	ENSG00000136546:[ENST00000409855(c.1346C>T,tCa>tTa):ENSP00000386796(p.Ser449Leu)]	NA	NA	NA	NA	NA	NA	NA	D	BN	NA	MODERATE	NA	NA	61	60	0	90	69	21	23.33

Patel *et al.*, 2021. Whole-exome sequencing analysis of oral squamous cell carcinoma delineated by tobacco usage habits
Supplementary Table 4. List of somatic single nucleotide variants identified using whole exome sequencing of OSCC patients

Cohort	Sample	Chromosome	Genomic position	Reference allele	Alternate allele	Genesymbol	Variant type	Variant description	OncoMD	COSMIC	dbSNP	ExAC	ExAC_SAS	1000G	1000G_SAS	SIFT	Polyphen2	CONDEL	VEP	LRT	PhastCon7Way	Total reads in Normal	Reference read support in Normal	Alternative read support in Normal	Total reads in Tumor	Reference read support in Tumor	Alternate read support in Tumor	Alternate allele frequency in Tumor
Chewer	IOB_07	chr2	170063215	C	A	LRP2	MISSENSE	ENSG00000081479:[ENST00000263816(c.7015G>T,Gca>Tca):ENSP00000263816(p.Ala2339Ser)]	NA	NA	NA	NA	NA	NA	NA	NA	NA	NA	MODERATE	NA	0.994	186	186	0	219	211	6	2.74
Chewer	IOB_07	chr2	173881887	C	T	RAPGEF4	INTRONIC	ENSG00000091428:[ENST00000535187(c.1239-15C>T)]	NA	NA	NA	NA	NA	NA	NA	NA	NA	NA	MODIFIER	NA	NA	101	101	0	128	79	49	38.28
Chewer	IOB_07	chr2	198570325	G	A	AC011997.1	INTRONIC	ENSG00000222017:[ENST00000409845(c.161+6737G>A)]	NA	NA	NA	NA	NA	NA	NA	NA	NA	NA	MODIFIER	NA	NA	162	162	0	282	275	6	2.13
Chewer	IOB_07	chr2	198570325	G	A	MARS2	MISSENSE	ENSG00000247626:[ENST00000282276(c.196G>A,Gca>Aca):ENSP00000282276(p.Ala66Thr)]	NA	NA	NA	NA	NA	NA	NA	NA	NA	NA	MODIFIER	NA	NA	162	162	0	282	275	6	2.13
Chewer	IOB_07	chr2	198949059	C	A	PLCL1	MISSENSE	ENSG00000115896:[ENST00000437704(c.524C>A,cCt>cAt):ENSP00000414138(p.Pro175His)]	NA	NA	NA	NA	NA	NA	NA	NA	NA	NA	MODIFIER	NA	NA	188	188	0	255	249	6	2.35
Chewer	IOB_07	chr2	201438222	G	C	SGOL2	MISSENSE	ENSG00000163535:[ENST00000357799(c.3153G>C,ttG>ttC):ENSP00000350447(p.Leu1051Phe)]	NA	NA	NA	NA	NA	NA	NA	T	PrD	NA	MODERATE	N	0.002	83	83	0	94	83	10	10.64
Chewer	IOB_07	chr2	202068403	C	T	CASP10	INTRONIC	ENSG00000003400:[ENST00000438843(c.*234-50C>T)]	NA	NA	NA	NA	NA	NA	NA	NA	NA	NA	MODIFIER	NA	NA	293	291	1	266	217	47	17.67
Chewer	IOB_07	chr2	202149536	C	G	CASP8	INTRONIC	841:[XM_005246885.1(c.935-3C>G)]	NA	NA	NA	NA	NA	NA	NA	NA	NA	NA	LOW	NA	NA	189	187	1	215	172	41	19.07
Chewer	IOB_07	chr2	203420973	C	T	BMPR2	MISSENSE	ENSG00000204217:[ENST00000374580(c.2585C>T,tCc>tTc):ENSP00000363708(p.Ser862Phe)]	NA	NA	NA	NA	NA	NA	NA	NA	NA	NA	MODIFIER	NA	NA	136	136	0	154	128	23	14.94
Chewer	IOB_07	chr2	212537952	G	T	ERBB4	SILENT	ENSG00000178568:[ENST00000342788(c.1653C>A,atC>atA):ENSP00000342235(p.Ile551(=))]	NA	NA	NA	NA	NA	NA	NA	NA	NA	NA	LOW	NA	NA	188	188	0	218	137	81	37.16
Chewer	IOB_07	chr2	212543895	C	T	ERBB4	MISSENSE	ENSG00000178568:[ENST00000260943(c.1503G>A,Gtg>Atg):ENSP00000260943(p.Val502Met)]	NA	NA	NA	NA	NA	NA	NA	D	BN	NA	MODERATE	D	1	56	56	0	71	53	18	25.35
Chewer	IOB_07	chr2	213921481	G	A	IKZF2	INTRONIC	ENSG00000030419:[ENST00000453575(c.*253+154C>T)]	NA	NA	NA	NA	NA	NA	NA	NA	NA	NA	MODIFIER	NA	NA	99	99	0	162	139	22	13.58
Chewer	IOB_07	chr2	219093410	A	T	ARPC2	NCT	ENSG00000163466:[ENST00000491780(n.224-51A>T)]	NA	NA	NA	NA	NA	NA	NA	NA	NA	NA	MODIFIER	NA	NA	77	75	0	81	60	21	25.93
Chewer	IOB_07	chr2	220047012	G	A	FAM134A	SILENT	ENSG00000144567:[ENST00000430297(c.1293G>A,gaG>gaA):ENSP00000395249(p.Glu431(=))]	NA	NA	NA	NA	NA	NA	NA	NA	NA	NA	LOW	NA	NA	132	131	0	165	127	38	23.03
Chewer	IOB_07	chr2	220326992	C	T	SPEG	INTRONIC	ENSG00000072195:[ENST00000396686(c.70-28C>T)]	NA	NA	NA	NA	NA	NA	NA	NA	NA	NA	MODIFIER	NA	NA	135	135	0	143	125	17	11.89
Chewer	IOB_07	chr2	238785945	C	T	RAMP1	SILENT	ENSG00000132329:[ENST00000403885(c.66C>T,ttC>ttT):ENSP00000386046(p.Phe22(=))]	NA	NA	NA	NA	NA	NA	NA	NA	NA	NA	LOW	NA	0	142	141	0	195	127	67	34.36
Chewer	IOB_07	chr2	241631507	A	G	AQP12A	MISSENSE	ENSG00000184945:[ENST00000429564(c.176A>G,gAg>gGg):ENSP00000405899(p.Glu59Gly)]	NA	NA	NA	NA	NA	NA	NA	D	PrD	NA	MODERATE	D	0.957	55	55	0	69	59	10	14.49
Chewer	IOB_07	chr20	5892249	C	T	CHGB	5UTR	ENSG00000089199:[ENST00000378961(c.-31C>T)]	NA	NA	NA	NA	NA	NA	NA	NA	NA	NA	MODIFIER	NA	NA	50	50	0	41	29	12	29.27
Chewer	IOB_07	chr20	31238495	T	C	C20orf203	SILENT	284805:[NM_182584.2(c.324A>G,ggA>ggG):NP_872390.2(p.Gly108(=))]	NA	NA	NA	NA	NA	NA	NA	NA	NA	NA	LOW	NA	NA	56	55	0	55	49	5	9.09
Chewer	IOB_07	chr20	31571667	C	G	SUN5	MISSENSE	ENSG00000167098:[ENST00000356173(c.1073G>C,cGa>cCa):ENSP00000348496(p.Arg358Pro)]	NA	NA	NA	NA	NA	NA	NA	D	PrD	NA	MODERATE	N	0.98	50	50	0	77	65	11	14.29
Chewer	IOB_07	chr20	33451088	C	T	GGT7	INTRONIC	ENSG00000131067:[ENST00000427420(c.456+28G>A)]	NA	NA	NA	NA	NA	NA	NA	NA	NA	NA	MODIFIER	NA	NA	147	147	0	122	94	22	18.03
Chewer	IOB_07	chr20	33509293	C	A	ACSS2	INTRONIC	ENSG00000131069:[ENST00000481284(c.*1156+28C>A)]	NA	NA	NA	NA	NA	NA	NA	NA	NA	NA	MODIFIER	NA	NA	132	132	0	119	112	5	4.2
Chewer	IOB_07	chr20	43743647	C	A	WFDC5	MISSENSE	ENSG00000175121:[ENST00000307971(c.78G>T,aaG>aaT):ENSP00000312381(p.Lys26Asn)]	NA	NA	NA	NA	NA	NA	NA	T	BN	NA	MODERATE	N	0.337	206	206	0	161	126	35	21.74
Chewer	IOB_07	chr20	43926691	C	A	MATN4	SILENT	ENSG00000124159:[ENST00000353917(c.1200G>T,gtG>gtT):ENSP00000243983(p.Val400(=))]	NA	NA	NA	NA	NA	NA	NA	NA	NA	NA	LOW	NA	NA	223	223	0	270	264	6	2.22
Chewer	IOB_07	chr20	44278080	C	A	WFDC11	INTRONIC	ENSG00000180083:[ENST00000324384(c.101-42G>T)]	NA	NA	NA	NA	NA	NA	NA	NA	NA	NA	MODIFIER	NA	NA	191	191	0	169	163	6	3.55
Chewer	IOB_07	chr20	48098652	C	T	KCNB1	NONSENSE	ENSG00000158445:[ENST00000371741(c.366G>A,tgG>tgA):ENSP00000360806(p.Trp122Ter)]	NA	NA	NA	NA	NA	NA	NA	NA	NA	NA	HIGH	NA	1	197	197	0	214	174	39	18.22
Chewer	IOB_07	chr20	61511439	G	A	DIDO1	MISSENSE	ENSG00000101191:[ENST00000266070(c.5869C>T,Cca>Tca):ENSP00000266070(p.Pro1957Ser)]	NA	chr20*61511439*61511439*COSM1534249*G*A*1*lung*1	NA	NA	NA	NA	NA	D	BN	NA	MODERATE	N	0.134	149	148	0	159	153	5	3.14
Chewer	IOB_07	chr20	61528304	C	T	DIDO1	MISSENSE	ENSG00000101191:[ENST00000266070(c.1633G>A,Gca>Aca):ENSP00000266070(p.Ala545Thr)]	NA	NA	NA	NA	NA	NA	NA	T	BN	NA	MODERATE	N	0	129	128	0	112	94	18	16.07
Chewer	IOB_07	chr20	61869295	C	A	BIRC7	SILENT	ENSG00000101197:[ENST00000217169(c.390C>A,ggC>ggA):ENSP00000217169(p.Gly130(=))]	NA	NA	NA	NA	NA	NA	NA	NA	NA	NA	LOW	NA	NA	79	78	0	115	110	5	4.35
Chewer	IOB_07	chr20	62664325	C	T	PRPF6	SILENT	ENSG00000101161:[ENST00000535781(c.2685C>T,ggC>ggT):ENSP00000446216(p.Gly895(=))]	NA	NA	NA	NA	NA	NA	NA	NA	NA	NA	LOW	NA	NA	169	169	0	141	134	5	3.55
Chewer	IOB_07	chr20	62664325	C	T	ZNF512B	INTRONIC	ENSG00000196700:[ENST00000217130(c.-19+5587G>A)]	NA	NA	NA	NA	NA	NA	NA	NA	NA	NA	LOW	NA	NA	169	169	0	141	134	5	3.55
Chewer	IOB_07	chr21	15013872	G	A	POTED	SILENT	ENSG00000166351:[ENST00000299443(c.1740G>A,acG>acA):ENSP00000299443(p.Thr580(=))]	NA	NA	NA	NA	NA	NA	NA	NA	NA	NA	LOW	NA	NA	44	44	0	62	51	11	17.74
Chewer	IOB_07	chr21	22658681	C	T	NCAM2	MISSENSE	ENSG00000154654:[ENST00000535285(c.505C>T,Cct>Tct):ENSP00000441887(p.Pro169Ser)]	NA	NA	NA	NA	NA	NA	NA	D	PrD	NA	MODERATE	D	1	196	194	1	192	141	50	26.04
Chewer	IOB_07	chr21	37661342	G	A	DOPEY2	INTRONIC-SS-ACR	ENSG00000142197:[ENST00000399151(c.6354-1G>A)]	NA	NA	NA	NA	NA	NA	NA	NA	NA	NA	HIGH	NA	NA	134	133	0	120	83	36	30
Chewer	IOB_07	chr21	38081443	C	T	SIM2	INTRONIC	ENSG00000159263:[ENST00000290399(c.176-25C>T)]	NA	NA	2.00E-04	NA	NA	2.00E-04	0.00E+00	NA	NA	NA	MODIFIER	NA	NA	147	147	0	154	119	34	22.08
Chewer	IOB_07	chr21	44174038	C	A	PDE9A	NCT	ENSG00000160191:[ENST00000495343(n.401-62C>A)]	NA	NA	NA	NA	NA	NA	NA	NA	NA	NA	MODIFIER	NA	NA	88	87	0	79	72	6	7.59
Chewer	IOB_07	chr21	44579774	G	A	AP001631.10	MISSENSE	ENSG00000228120:[ENST00000433840(c.268C>T,Ccg>Tcg):ENSP00000391357(p.Pro90Ser)]	NA	NA	NA	NA	NA	NA	NA	D_lc	PoD	NA	MODERATE	NA	NA	126	126	0	107	100	5	4.67
Chewer	IOB_07	chr21	45107794	C	T	RRP1B	SILENT	ENSG00000160208:[ENST00000340648(c.1539C>T,tcC>tcT):ENSP00000339145(p.Ser513(=))]	NA	NA	chr21*45107794*45107794*C*A*rs766647950*NA*NA*NA	NA	NA	NA	NA	NA	NA	NA	LOW	NA	NA	189	189	0	173	147	25	14.45
Chewer	IOB_07	chr21	45555880	T	G	C21orf33	NCT	ENSG00000160221:[ENST00000480786(n.219-62T>G)]	NA	NA	NA	NA	NA	NA	NA	NA	NA	NA	MODIFIER	NA	NA	79	78	0	127	103	23	18.11
Chewer	IOB_07	chr21	45660759	G	A	ICOSLG	5UTR	ENSG00000160223:[ENST00000400377(c.-59C>T)]	NA	NA	NA	NA	NA	NA	NA	NA	NA	NA	MODIFIER	NA	NA	22	22	0	48	34	14	29.17
Chewer	IOB_07	chr21	47716023	G	A	YBEY	INTRONIC	ENSG00000182362:[ENST00000329319(c.340-53G>A)]	NA	NA	NA	NA	NA	NA	NA	NA	NA	NA	MODIFIER	NA	NA	90	90	0	85	70	15	17.65
Chewer	IOB_07	chr21	47716024	G	A	YBEY	INTRONIC	ENSG00000182362:[ENST00000397692(c.76-52G>A)]	NA	NA	chr21*47716024*47716024*G*C*rs1014566788*NA*NA*NA	NA	NA	NA	NA	NA	NA	NA	MODIFIER	NA	NA	91	91	0	88	72	15	17.05
Chewer	IOB_07	chr21	47855838	C	T	PCNT	NONSENSE	ENSG00000160299:[ENST00000359568(c.8773C>T,Cag>Tag):ENSP00000352572(p.Gln2925Ter)]	NA	NA	NA	NA	NA	NA	NA	NA	NA	NA	HIGH	NA	NA	234	234	0	257	182	74	28.79
Chewer	IOB_07	chr22	18185100	T	A	BCL2L13	MISSENSE	ENSG00000099968:[ENST00000538149(c.176T>A,gTg>gAg):ENSP00000441344(p.Val59Glu)]	NA	NA	NA	NA	NA	NA	NA	NA	NA	NA	MODIFIER	NA	NA	133	133	0	142	107	35	24.65
Chewer	IOB_07	chr22	22317320	G	A	TOP3B	MISSENSE	ENSG00000100038:[ENST00000436282(c.473C>T,cCt>cTt):ENSP00000416451(p.Pro158Leu)]	NA	NA	NA	NA	NA	NA	NA	NA	NA	NA	MODIFIER	NA	NA	66	66	0	66	50	15	22.73
Chewer	IOB_07	chr22	24581537	C	T	SUSD2	SILENT-SS-PRX	ENSG00000099994:[ENST00000358321(c.1068C>T,gcC>gcT):ENSP00000351075(p.Ala356(=))]	NA	NA	NA	NA	NA	NA	NA	NA	NA	NA	LOW	NA	NA	132	132	0	120	101	18	15
Chewer	IOB_07	chr22	31127600	C	T	MIR3200	MATURE-MIRNA	ENSG00000264661:[ENST00000580767(n.57C>T)]	NA	NA	NA	NA	NA	NA	NA	NA	NA	NA	MODIFIER	NA	NA	38	38	0	39	23	16	41.03
Chewer	IOB_07	chr22	31127600	C	T	OSBP2	INTRONIC	ENSG00000184792:[ENST00000403222(c.150-9548C>T)]	NA	NA	NA	NA	NA	NA	NA	NA	NA	NA	MODIFIER	NA	NA	38	38	0	39	23	16	41.03
Chewer	IOB_07	chr22	32084062	C	G	PRR14L	INTRONIC	ENSG00000183530:[ENST00000432485(c.348+80G>C)]	NA	NA	NA	NA	NA	NA	NA	NA	NA	NA	MODIFIER	NA	NA	31	31	0	31	25	6	19.35
Chewer	IOB_07	chr22	32758977	G	A	RFPL3S	MISSENSE	ENSG00000205853:[ENST00000382086(c.193C>T,Cgg>Tgg):ENSP00000371518(p.Arg65Trp)]	NA	NA	5.99E-04	3.01E-04	5.22E-04	5.99E-04	0.00E+00	NA	NA	NA	MODIFIER	NA	NA	69	69	0	49	34	15	30.61
Chewer	IOB_07	chr22	39772151	G	A	SYNGR1	SILENT	ENSG00000100321:[ENST00000381535(c.435G>A,acG>acA):ENSP00000370946(p.Thr145(=))]	NA	NA	NA	4.86E-04	1.21E-04	NA	NA	NA	NA	NA	LOW	NA	NA	156	156	0	169	138	30	17.75
Chewer	IOB_07	chr22	40816585	G	A	MKL1	INTRONIC	57591:[XM_005261692.1(c.963-5C>T)]	NA	NA	NA	NA	NA	NA	NA	NA	NA	NA	LOW	NA	NA	90	89	0	89	66	23	25.84
Chewer	IOB_07	chr22	42805558	G	A	NFAM1	INTRONIC-SS-ACR-PRX	ENSG00000235568:[ENST00000329021(c.452-5C>T)]	NA	NA	NA	NA	NA	NA	NA	NA	NA	NA	LOW	NA	NA	93	92	0	120	97	23	19.17
Chewer	IOB_07	chr22	51159188	C	T	SHANK3	MISSENSE	ENSG00000251322:[ENST00000414786(c.2885C>T,cCc>cTc):ENSP00000464552(p.Pro962Leu)]	NA	NA	NA	NA	NA	NA	NA	T	BN	NA	MODERATE	NA	NA	19	19	0	31	21	10	32.26
Chewer	IOB_07	chr3	14724218	C	A	C3orf20	INTRONIC	ENSG00000131379:[ENST00000412910(c.-105-264C>A)]	NA	NA	chr3*14724218*14724218*C*G*rs775601385*NA*NA*NA	NA	NA	NA	NA	NA	NA	NA	MODIFIER	NA	NA	79	78	0	64	37	25	39.06
Chewer	IOB_07	chr3	39230609	C	T	XIRP1	MISSENSE	ENSG00000168334:[ENST00000340369(c.328G>A,Gag>Aag):ENSP00000343140(p.Glu110Lys)]	NA	NA	2.00E-04	8.25E-06	0.00E+00	2.00E-04	0.00E+00	T	BN	NA	MODERATE	U	0.904	244	244	0	229	172	56	24.45
Chewer	IOB_07	chr3	42916065	C	A	ACKR2	NCT	ENSG00000144648:[ENST00000498111(n.546+9714C>A)]	NA	NA	chr3*42916065*42916065*C*T*rs767458378*NA*NA*NA	NA	NA	NA	NA	T	BN	NA	MODERATE	N	0.786	125	124	0	153	148	5	3.27
Chewer	IOB_07	chr3	42916065	C	A	CYP8B1	MISSENSE	ENSG00000180432:[ENST00000316161(c.1244G>T,cGg>cTg):ENSP00000318867(p.Arg415Leu)]	NA	NA	chr3*42916065*42916065*C*T*rs767458378*NA*NA*NA	NA	NA	NA	NA	T	BN	NA	MODERATE	N	0.786	125	124	0	153	148	5	3.27
Chewer	IOB_07	chr3	42916065	C	A	KRBOX1	NCT	ENSG00000273291:[ENST00000451200(n.168+54862C>A)]	NA	NA	chr3*42916065*42916065*C*T*rs767458378*NA*NA*NA	NA	NA	NA	NA	T	BN	NA	MODERATE	N	0.786	125	124	0	153	148	5	3.27
Chewer	IOB_07	chr3	42916065	C	A	RP11-141M3.5	NCT	ENSG00000273328:[ENST00000487368(n.356-33449C>A)]	NA	NA	chr3*42916065*42916065*C*T*rs767458378*NA*NA*NA	NA	NA	NA	NA	T	BN	NA	MODERATE	N	0.786	125	124	0	153	148	5	3.27
Chewer	IOB_07	chr3	46449303	C	T	CCRL2	INTRONIC	ENSG00000121797:[ENST00000400880(c.-13+171C>T)]	NA	NA	NA	NA	NA	NA	NA	NA	NA	NA	MODIFIER	NA	NA	86	86	0	111	77	34	30.63
Chewer	IOB_07	chr3	46497462	C	T	LTF	MISSENSE	ENSG00000012223:[ENST00000443496(c.284G>A,cGa>cAa):ENSP00000397427(p.Arg95Gln)]	NA	chr3*46497462*46497462*COSM1045293*C*T*2*endometrium*2	NA	NA	NA	NA	NA	T	BN	NA	MODERATE	NA	NA	78	78	0	68	51	17	25
Chewer	IOB_07	chr3	47894794	C	T	MAP4	MISSENSE	ENSG00000047849:[ENST00000360240(c.3310G>A,Ggg>Agg):ENSP00000353375(p.Gly1104Arg)]	NA	NA	NA	NA	NA	NA	NA	NA	NA	NA	MODERATE	NA	NA	67	67	0	87	71	14	16.09
Chewer	IOB_07	chr3	49062563	G	A	IMPDH2	SILENT	ENSG00000178035:[ENST00000326739(c.1146C>T,tcC>tcT):ENSP00000321584(p.Ser382(=))]	NA	NA	NA	NA	NA	NA	NA	NA	NA	NA	MODIFIER	NA	NA	211	209	0	213	175	38	17.84
Chewer	IOB_07	chr3	49163434	G	A	LAMB2	SILENT	ENSG00000172037:[ENST00000305544(c.2310C>T,atC>atT):ENSP00000307156(p.Ile770(=))]	NA	NA	NA	NA	NA	NA	NA	NA	NA	NA	LOW	NA	1	210	210	0	202	154	48	23.76
Chewer	IOB_07	chr3	51982378	C	T	PARP3	MISSENSE	ENSG00000041880:[ENST00000417220(c.1484C>T,cCc>cTc):ENSP00000395951(p.Pro495Leu)]	NA	chr3*51982378*51982378*COSM1424327*C*T*1*large_intestine*1|chr3*51982378*51982378*COSM1424326*C*T*1*large_intestine*1	NA	NA	NA	NA	NA	NA	NA	NA	MODIFIER	NA	NA	200	198	0	183	150	32	17.49
Chewer	IOB_07	chr3	57357167	A	C	DNAH12	SILENT	ENSG00000174844:[ENST00000466540(c.720T>G,acT>acG):ENSP00000420359(p.Thr240(=))]	NA	NA	NA	NA	NA	NA	NA	NA	NA	NA	LOW	NA	NA	342	341	0	304	237	66	21.71
Chewer	IOB_07	chr3	62180698	C	A	PTPRG	INTRONIC	5793:[XM_005265352.1(c.1177-38C>A)]	NA	NA	NA	NA	NA	NA	NA	NA	NA	NA	MODIFIER	NA	NA	327	324	2	308	298	5	1.62
Chewer	IOB_07	chr3	66433731	G	A	LRIG1	SILENT	ENSG00000144749:[ENST00000273261(c.2166C>T,ccC>ccT):ENSP00000273261(p.Pro722(=))]	NA	NA	NA	8.39E-06	0.00E+00	NA	NA	NA	NA	NA	MODIFIER	NA	NA	158	158	0	181	138	40	22.1
Chewer	IOB_07	chr3	66433731	G	A	SLC25A26	INTRONIC	ENSG00000144741:[ENST00000464350(c.*1547+2354G>A)]	NA	NA	NA	8.39E-06	0.00E+00	NA	NA	NA	NA	NA	MODIFIER	NA	NA	158	158	0	181	138	40	22.1
Chewer	IOB_07	chr3	101293189	T	G	PCNP	INTRONIC	ENSG00000081154:[ENST00000469941(c.-91+66T>G)]	NA	NA	NA	NA	NA	NA	NA	NA	NA	NA	MODIFIER	NA	NA	41	39	1	37	26	7	18.92
Chewer	IOB_07	chr3	101390833	C	A	ZBTB11	NONSENSE	ENSG00000066422:[ENST00000312938(c.535G>T,Gaa>Taa):ENSP00000326200(p.Glu179Ter)]	NA	NA	NA	NA	NA	NA	NA	NA	NA	NA	HIGH	NA	1	288	287	0	240	220	20	8.33
Chewer	IOB_07	chr3	108301871	C	A	KIAA1524	MISSENSE	ENSG00000163507:[ENST00000295746(c.310G>T,Gcg>Tcg):ENSP00000295746(p.Ala104Ser)]	NA	NA	NA	NA	NA	NA	NA	D	BN	NA	MODERATE	NA	NA	163	162	0	184	178	5	2.72

Patel *et al.* , 2021. Whole-exome sequencing analysis of oral squamous cell carcinoma delineated by tobacco usage habits
Supplementary Table 4. List of somatic single nucleotide variants identified using whole exome sequencing of OSCC patients

Cohort	Sample	Chromosome	Genomic position	Reference allele	Alternate allele	Genesymbol	Variant type	Variant description	OncoMD	COSMIC	dbSNP	ExAC	ExAC_SAS	1000G	1000G_SAS	SIFT	Polyphen2	CONDEL	VEP	LRT	PhastCon7Way	Total reads in Normal	Reference read support in Normal	Alternative read support in Normal	Total reads in Tumor	Reference read support in Tumor	Alternate read support in Tumor	Alternate allele frequency in Tumor
Chewer	IOB_07	chr3	127702942	C	T	KBTBD12	MISSENSE-SS-PRX	ENSG00000187715:[ENST00000476626(n.313C>T)]	NA	chr3*127702942*127702942*COSM331347*C*A*1*lung*1	NA	8.39E-06	0.00E+00	NA	NA	D	PrD	NA	MODERATE	D	1	125	123	0	162	119	42	25.93
Chewer	IOB_07	chr3	130132907	C	T	COL6A5	NCT-SS-PRX	256076:[NR_022012.2(n.5193-6C>T)]	NA	NA	NA	NA	NA	NA	NA	NA	NA	NA	LOW	NA	NA	149	145	0	165	127	37	22.42
Chewer	IOB_07	chr3	130992338	C	T	NEK11	SILENT	ENSG00000114670:[ENST00000412440(c.1086C>T,acC>acT):ENSP00000411888(p.Thr362(=))]	NA	NA	NA	NA	NA	NA	NA	NA	NA	NA	MODIFIER	NA	NA	116	116	0	146	110	35	23.97
Chewer	IOB_07	chr3	141497645	C	T	GRK7	SILENT	ENSG00000114124:[ENST00000264952(c.519C>T,gaC>gaT):ENSP00000264952(p.Asp173(=))]	NA	NA	chr3*141497645*141497645*C*A*rs367747374*NA*NA*NA	NA	NA	NA	NA	NA	NA	NA	LOW	NA	0.957	211	208	0	199	191	5	2.51
Chewer	IOB_07	chr3	151475243	A	T	AADACL2	MISSENSE	ENSG00000197953:[ENST00000356517(c.1067A>T,aAt>aTt):ENSP00000348911(p.Asn356Ile)]	NA	NA	chr3*151475243*151475243*A*G*rs369762343*NA*NA*NA	NA	NA	NA	NA	NA	NA	NA	MODIFIER	NA	NA	161	160	0	155	131	24	15.48
Chewer	IOB_07	chr3	151475243	A	T	AADACL2-AS1	NCT	101928142:[NR_110203.1(n.380-5944T>A)]	NA	NA	chr3*151475243*151475243*A*G*rs369762343*NA*NA*NA	NA	NA	NA	NA	NA	NA	NA	MODIFIER	NA	NA	161	160	0	155	131	24	15.48
Chewer	IOB_07	chr3	151475243	A	T	RP11-454C18.2	NCT	ENSG00000242908:[ENST00000475855(n.380-2324T>A)]	NA	NA	chr3*151475243*151475243*A*G*rs369762343*NA*NA*NA	NA	NA	NA	NA	NA	NA	NA	MODIFIER	NA	NA	161	160	0	155	131	24	15.48
Chewer	IOB_07	chr3	153220213	C	A	C3orf79	MISSENSE	ENSG00000237787:[ENST00000446603(c.245C>A,aCt>aAt):ENSP00000389475(p.Thr82Asn)]	NA	NA	chr3*153220213*153220213*C*T*rs61742013*1*0.9844*0.01558	NA	NA	NA	NA	NA	NA	NA	MODIFIER	NA	NA	177	176	1	123	118	5	4.07
Chewer	IOB_07	chr3	153220213	C	A	RP11-23D24.2	NCT	ENSG00000238755:[ENST00000493214(n.381+32119G>T)]	NA	NA	chr3*153220213*153220213*C*T*rs61742013*1*0.9844*0.01558	NA	NA	NA	NA	NA	NA	NA	MODIFIER	NA	NA	177	176	1	123	118	5	4.07
Chewer	IOB_07	chr3	183823206	C	T	HTR3E	SILENT	ENSG00000186038:[ENST00000425359(c.666C>T,atC>atT):ENSP00000401900(p.Ile222(=))]	NA	chr3*183823206*183823206*COSM209010*C*T*1*large_intestine*1	NA	1.67E-05	0.00E+00	NA	NA	NA	NA	NA	LOW	NA	0.443	131	130	0	123	112	11	8.94
Chewer	IOB_07	chr3	185393741	A	G	IGF2BP2	INTRONIC	10644:[NM_001291874.2(c.624-40T>C)]	NA	NA	.	NA	NA	NA	NA	NA	NA	NA	MODIFIER	NA	NA	48	48	0	69	63	5	7.25
Chewer	IOB_07	chr3	186506910	A	G	EIF4A2	NCT-SS-PRX	ENSG00000156976:[ENST00000496382(n.108-4A>G)]	NA	NA	NA	NA	NA	NA	NA	NA	NA	NA	LOW	NA	NA	106	106	0	139	134	5	3.6
Chewer	IOB_07	chr3	193080199	C	A	ATP13A5	MISSENSE	ENSG00000187527:[ENST00000342358(c.507G>T,ttG>ttT):ENSP00000341942(p.Leu169Phe)]	NA	NA	NA	NA	NA	NA	NA	D	BN	NA	MODERATE	N	0.882	132	132	0	189	115	72	38.1
Chewer	IOB_07	chr3	193335526	C	T	OPA1	INTRONIC	ENSG00000198836:[ENST00000361510(c.557-47C>T)]	NA	NA	NA	NA	NA	NA	NA	NA	NA	NA	MODIFIER	NA	NA	57	56	0	79	55	23	29.11
Chewer	IOB_07	chr3	195516442	G	A	MUC4	MISSENSE	ENSG00000145113:[ENST00000462323(c.2009C>T,tCc>tTc):ENSP00000417397(p.Ser670Phe)]	NA	NA	NA	NA	NA	NA	NA	NA	NA	NA	MODIFIER	NA	NA	125	125	0	168	126	39	23.21
Chewer	IOB_07	chr3	195789731	C	A	TFRC	MISSENSE	ENSG00000072274:[ENST00000420415(c.1155G>T,tgG>tgT):ENSP00000390133(p.Trp385Cys)]	NA	NA	NA	NA	NA	NA	NA	D	PrD	NA	MODERATE	D	1	125	125	0	136	130	6	4.41
Chewer	IOB_07	chr4	3201468	G	T	HTT	MISSENSE	ENSG00000197386:[ENST00000355072(c.5378G>T,cGg>cTg):ENSP00000347184(p.Arg1793Leu)]	NA	NA	NA	NA	NA	NA	NA	NA	NA	NA	MODIFIER	NA	NA	84	84	0	65	54	11	16.92
Chewer	IOB_07	chr4	3417763	C	T	RGS12	MISSENSE	ENSG00000159788:[ENST00000338806(c.398C>T,cCg>cTg):ENSP00000342133(p.Pro133Leu)]	NA	NA	chr4*3417763*3417763*C*A,T*rs763344869*NA*NA*NA	8.80E-06	0.00E+00	NA	NA	D	PoD	NA	MODERATE	D	1	115	115	0	128	108	20	15.62
Chewer	IOB_07	chr4	47916186	C	T	NFXL1	MISSENSE	ENSG00000170448:[ENST00000329043(c.35G>A,cGa>cAa):ENSP00000333113(p.Arg12Gln)]	NA	NA	chr4*47916186*47916203*CGGCCACCGGCCACCTGG*C*rs756576627*NA*NA*NA	NA	NA	NA	NA	D_lc	PrD	NA	MODERATE	N	1	51	49	0	69	50	16	23.19
Chewer	IOB_07	chr4	47916186	C	T	NIPAL1	EXONIC-NC	ENSG00000163293:[ENST00000513724(n.28C>T)]	NA	NA	chr4*47916186*47916203*CGGCCACCGGCCACCTGG*C*rs756576627*NA*NA*NA	NA	NA	NA	NA	D_lc	PrD	NA	MODERATE	N	1	51	49	0	69	50	16	23.19
Chewer	IOB_07	chr4	74863363	C	T	CXCL5	SILENT	ENSG00000163735:[ENST00000296027(c.336G>A,aaG>aaA):ENSP00000296027(p.Lys112(=))]	NA	NA	NA	1.65E-05	0.00E+00	NA	NA	NA	NA	NA	LOW	NA	0.238	108	106	0	117	83	33	28.21
Chewer	IOB_07	chr4	82088371	A	G	PRKG2	SILENT	ENSG00000138669:[ENST00000264399(c.856T>C,Ttg>Ctg):ENSP00000264399(p.Leu286(=))]	NA	NA	NA	NA	NA	NA	NA	NA	NA	NA	LOW	NA	1	44	44	0	46	33	13	28.26
Chewer	IOB_07	chr4	82088371	A	G	RP11-100N20.1	NCT	ENSG00000251059:[ENST00000512502(n.59+2219A>G)]	NA	NA	NA	NA	NA	NA	NA	NA	NA	NA	LOW	NA	1	44	44	0	46	33	13	28.26
Chewer	IOB_07	chr4	88231338	G	A	HSD17B13	INTRONIC	ENSG00000170509:[ENST00000302219(c.704+57C>T)]	NA	NA	NA	NA	NA	NA	NA	NA	NA	NA	MODIFIER	NA	NA	19	18	1	17	9	7	41.18
Chewer	IOB_07	chr4	88231339	C	G	HSD17B13	INTRONIC	ENSG00000170509:[ENST00000328546(c.812+56G>C)]	NA	NA	.	NA	NA	NA	NA	NA	NA	NA	MODIFIER	NA	NA	20	19	1	17	9	7	41.18
Chewer	IOB_07	chr4	88766217	A	G	MEPE	MISSENSE	ENSG00000152595:[ENST00000395102(c.290A>G,cAg>cGg):ENSP00000378534(p.Gln97Arg)]	NA	NA	NA	NA	NA	NA	NA	NA	NA	NA	MODIFIER	NA	NA	60	60	0	76	55	21	27.63
Chewer	IOB_07	chr4	96761978	G	A	PDHA2	MISSENSE	ENSG00000163114:[ENST00000295266(c.677G>A,gGa>gAa):ENSP00000295266(p.Gly226Glu)]	NA	NA	NA	NA	NA	NA	NA	D	PrD	NA	MODERATE	D	1	237	236	0	251	192	59	23.51
Chewer	IOB_07	chr4	140640586	C	T	MAML3	MISSENSE	ENSG00000196782:[ENST00000509479(c.3308G>A,gGt>gAt):ENSP00000421180(p.Gly1103Asp)]	NA	NA	NA	NA	NA	NA	NA	NA	NA	NA	MODERATE	NA	0.982	176	175	0	174	139	35	20.11
Chewer	IOB_07	chr4	140640586	C	T	MGST2	NCT	ENSG00000085871:[ENST00000515137(n.479+15236C>T)]	NA	NA	NA	NA	NA	NA	NA	NA	NA	NA	MODERATE	NA	0.982	176	175	0	174	139	35	20.11
Chewer	IOB_07	chr4	153875457	C	T	FHDC1	MISSENSE	ENSG00000137460:[ENST00000260008(c.649C>T,Cca>Tca):ENSP00000260008(p.Pro217Ser)]	NA	NA	NA	NA	NA	NA	NA	D	PrD	NA	MODERATE	D	1	181	181	0	215	208	6	2.79
Chewer	IOB_07	chr4	170679067	G	A	C4orf27	5UTR	ENSG00000056050:[ENST00000393381(c.-39C>T)]	NA	NA	NA	NA	NA	NA	NA	NA	NA	NA	MODIFIER	NA	NA	86	86	0	127	122	5	3.94
Chewer	IOB_07	chr4	177067271	C	T	WDR17	MISSENSE	ENSG00000150627:[ENST00000280190(c.1727C>T,cCt>cTt):ENSP00000280190(p.Pro576Leu)]	NA	NA	NA	8.24E-06	0.00E+00	NA	NA	D	BN	NA	MODERATE	D	1	259	258	0	258	205	50	19.38
Chewer	IOB_07	chr4	184186125	C	T	WWC2	INTRONIC-SS-ACR-PRX	ENSG00000151718:[ENST00000403733(c.1910-6C>T)]	NA	NA	NA	NA	NA	NA	NA	NA	NA	NA	LOW	NA	NA	80	80	0	102	73	29	28.43
Chewer	IOB_07	chr4	187532819	G	A	FAT1	NONSENSE	ENSG00000083857:[ENST00000441802(c.9574C>T,Cag>Tag):ENSP00000406229(p.Gln3192Ter)]	NA	NA	NA	NA	NA	NA	NA	NA	NA	NA	HIGH	NA	1	150	150	0	176	135	40	22.73
Chewer	IOB_07	chr4	187532820	G	A	FAT1	SILENT	ENSG00000083857:[ENST00000441802(c.9573C>T,ctC>ctT):ENSP00000406229(p.Leu3191(=))]	NA	NA	NA	NA	NA	NA	NA	NA	NA	NA	LOW	NA	NA	151	151	0	177	136	40	22.6
Chewer	IOB_07	chr5	1881029	C	A	IRX4	INTRONIC	ENSG00000113430:[ENST00000231357(c.298-81G>T)]	NA	NA	chr5*1881013*1881032*TAGGAGCCCCCCACTCCCAA*T*rs975773162*NA*NA*NA	NA	NA	NA	NA	NA	NA	NA	MODIFIER	NA	NA	103	103	0	121	115	5	4.13
Chewer	IOB_07	chr5	16090978	G	A	MARCH11	INTRONIC	ENSG00000183654:[ENST00000507111(c.105-23076C>T)]	NA	NA	NA	8.04E-05	0.00E+00	NA	NA	NA	NA	NA	MODIFIER	NA	NA	31	31	0	41	31	9	21.95
Chewer	IOB_07	chr5	54429297	C	T	CDC20B	MISSENSE	ENSG00000164287:[ENST00000296733(c.640G>A,Gat>Aat):ENSP00000296733(p.Asp214Asn)]	NA	NA	NA	NA	NA	NA	NA	T	BN	NA	MODERATE	NA	NA	134	133	0	148	117	31	20.95
Chewer	IOB_07	chr5	76926455	G	A	OTP	SILENT	ENSG00000171540:[ENST00000306422(c.612C>T,gcC>gcT):ENSP00000302814(p.Ala204(=))]	NA	NA	NA	NA	NA	NA	NA	NA	NA	NA	LOW	NA	NA	126	126	0	150	107	42	28
Chewer	IOB_07	chr5	79285025	C	A	MTX3	MISSENSE	ENSG00000177034:[ENST00000509852(c.271G>T,Gat>Tat):ENSP00000423302(p.Asp91Tyr)]	NA	NA	NA	NA	NA	NA	NA	D	PrD	NA	MODERATE	D	1	97	95	0	101	95	5	4.95
Chewer	IOB_07	chr5	89971295	G	T	ADGRV1	NCT	84059:[NR_003149.1(n.5409+33G>T)]	NA	NA	NA	NA	NA	NA	NA	NA	NA	NA	MODIFIER	NA	NA	88	88	0	90	77	13	14.44
Chewer	IOB_07	chr5	89971295	G	T	GPR98	NCT	ENSG00000164199:[ENST00000450321(n.648+33G>T)]	NA	NA	NA	NA	NA	NA	NA	NA	NA	NA	MODIFIER	NA	NA	88	88	0	90	77	13	14.44
Chewer	IOB_07	chr5	94027971	T	C	ANKRD32	NCT	ENSG00000133302:[ENST00000450932(n.1277+10T>C)]	NA	NA	NA	NA	NA	NA	NA	NA	NA	NA	MODIFIER	NA	NA	82	82	0	93	72	21	22.58
Chewer	IOB_07	chr5	94728598	G	A	FAM81B	SILENT	ENSG00000153347:[ENST00000503361(c.63G>A,aaG>aaA):ENSP00000424883(p.Lys21(=))]	NA	NA	chr5*94728598*94728598*G*T*rs375187381*NA*NA*NA	NA	NA	NA	NA	NA	NA	NA	MODIFIER	NA	NA	78	78	0	123	89	34	27.64
Chewer	IOB_07	chr5	110440019	C	T	WDR36	MISSENSE	ENSG00000134987:[ENST00000506538(c.1042C>T,Ctt>Ttt):ENSP00000423067(p.Leu348Phe)]	NA	NA	NA	NA	NA	NA	NA	D	BN	NA	MODERATE	D	1	211	211	0	189	143	46	24.34
Chewer	IOB_07	chr5	137727698	G	C	KDM3B	MISSENSE	ENSG00000120733:[ENST00000394866(c.1345G>C,Gct>Cct):ENSP00000378335(p.Ala449Pro)]	NA	NA	NA	NA	NA	NA	NA	NA	NA	NA	MODERATE	NA	1	93	93	0	128	110	18	14.06
Chewer	IOB_07	chr5	139283885	G	T	NRG2	NCT	9542:[XR_245860.1(n.891-16789C>A)]	NA	NA	chr5*139283885*139283885*G*A*rs1020971292*NA*NA*NA	NA	NA	NA	NA	NA	NA	NA	LOW	NA	NA	95	94	0	146	111	33	22.6
Chewer	IOB_07	chr5	140166577	C	T	PCDHA1	SILENT	ENSG00000204970:[ENST00000378133(c.702C>T,gaC>gaT):ENSP00000367373(p.Asp234(=))]	NA	NA	NA	4.12E-05	0.00E+00	NA	NA	NA	NA	NA	LOW	NA	0	168	168	0	199	150	48	24.12
Chewer	IOB_07	chr5	140567871	C	T	PCDHB9	MISSENSE	56127:[NM_019119.4(c.979C>T,Ctt>Ttt):NP_061992.3(p.Leu327Phe)]	NA	NA	NA	NA	NA	NA	NA	NA	NA	NA	MODIFIER	NA	NA	168	167	0	199	174	23	11.56
Chewer	IOB_07	chr5	140730527	G	A	PCDHGA1	INTRONIC	ENSG00000204956:[ENST00000517417(c.2421+17855G>A)]	NA	NA	NA	4.14E-05	0.00E+00	NA	NA	NA	NA	NA	MODIFIER	NA	NA	174	174	0	163	121	40	24.54
Chewer	IOB_07	chr5	140730527	G	A	PCDHGA2	INTRONIC	ENSG00000081853:[ENST00000394576(c.2424+9565G>A)]	NA	NA	NA	4.14E-05	0.00E+00	NA	NA	NA	NA	NA	MODIFIER	NA	NA	174	174	0	163	121	40	24.54
Chewer	IOB_07	chr5	140730527	G	A	PCDHGA3	INTRONIC	ENSG00000254245:[ENST00000253812(c.2424+4503G>A)]	NA	NA	NA	4.14E-05	0.00E+00	NA	NA	NA	NA	NA	MODIFIER	NA	NA	174	174	0	163	121	40	24.54
Chewer	IOB_07	chr5	140730527	G	A	PCDHGB1	MISSENSE	ENSG00000254221:[ENST00000523390(c.700G>A,Gat>Aat):ENSP00000429273(p.Asp234Asn)]	NA	NA	NA	4.14E-05	0.00E+00	NA	NA	NA	NA	NA	MODIFIER	NA	NA	174	174	0	163	121	40	24.54
Chewer	IOB_07	chr5	140730835	G	C	PCDHGA1	INTRONIC	ENSG00000204956:[ENST00000517417(c.2421+18163G>C)]	NA	chr5*140730835*140730835*COSM3669411*G*A*1*liver*1|chr5*140730835*140730835*COSM3669410*G*A*1*liver*1	NA	NA	NA	NA	NA	NA	NA	NA	MODIFIER	NA	NA	226	226	0	249	195	53	21.29
Chewer	IOB_07	chr5	140730835	G	C	PCDHGA2	INTRONIC	ENSG00000081853:[ENST00000394576(c.2424+9873G>C)]	NA	chr5*140730835*140730835*COSM3669411*G*A*1*liver*1|chr5*140730835*140730835*COSM3669410*G*A*1*liver*1	NA	NA	NA	NA	NA	NA	NA	NA	MODIFIER	NA	NA	226	226	0	249	195	53	21.29
Chewer	IOB_07	chr5	140730835	G	C	PCDHGA3	INTRONIC	ENSG00000254245:[ENST00000253812(c.2424+4811G>C)]	NA	chr5*140730835*140730835*COSM3669411*G*A*1*liver*1|chr5*140730835*140730835*COSM3669410*G*A*1*liver*1	NA	NA	NA	NA	NA	NA	NA	NA	MODIFIER	NA	NA	226	226	0	249	195	53	21.29
Chewer	IOB_07	chr5	140730835	G	C	PCDHGB1	MISSENSE	ENSG00000254221:[ENST00000523390(c.1008G>C,gaG>gaC):ENSP00000429273(p.Glu336Asp)]	NA	chr5*140730835*140730835*COSM3669411*G*A*1*liver*1|chr5*140730835*140730835*COSM3669410*G*A*1*liver*1	NA	NA	NA	NA	NA	NA	NA	NA	MODIFIER	NA	NA	226	226	0	249	195	53	21.29
Chewer	IOB_07	chr5	140731445	G	A	PCDHGA1	INTRONIC	ENSG00000204956:[ENST00000517417(c.2421+18773G>A)]	NA	NA	NA	8.50E-06	0.00E+00	NA	NA	NA	NA	NA	MODIFIER	NA	NA	79	79	0	92	76	15	16.3
Chewer	IOB_07	chr5	140731445	G	A	PCDHGA2	INTRONIC	ENSG00000081853:[ENST00000394576(c.2424+10483G>A)]	NA	NA	NA	8.50E-06	0.00E+00	NA	NA	NA	NA	NA	MODIFIER	NA	NA	79	79	0	92	76	15	16.3
Chewer	IOB_07	chr5	140731445	G	A	PCDHGA3	INTRONIC	ENSG00000254245:[ENST00000253812(c.2424+5421G>A)]	NA	NA	NA	8.50E-06	0.00E+00	NA	NA	NA	NA	NA	MODIFIER	NA	NA	79	79	0	92	76	15	16.3
Chewer	IOB_07	chr5	140731445	G	A	PCDHGB1	MISSENSE	ENSG00000254221:[ENST00000523390(c.1618G>A,Gcc>Acc):ENSP00000429273(p.Ala540Thr)]	NA	NA	NA	8.50E-06	0.00E+00	NA	NA	NA	NA	NA	MODIFIER	NA	NA	79	79	0	92	76	15	16.3

Patel *et al.* , 2021. Whole-exome sequencing analysis of oral squamous cell carcinoma delineated by tobacco usage habits
Supplementary Table 4. List of somatic single nucleotide variants identified using whole exome sequencing of OSCC patients

Cohort	Sample	Chromosome	Genomic position	Reference allele	Alternate allele	Genesymbol	Variant type	Variant description	OncoMD	COSMIC	dbSNP	ExAC	ExAC_SAS	1000G	1000G_SAS	SIFT	Polyphen2	CONDEL	VEP	LRT	PhastCon7Way	Total reads in Normal	Reference read support in Normal	Alternative read support in Normal	Total reads in Tumor	Reference read support in Tumor	Alternate read support in Tumor	Alternate allele frequency in Tumor
Chewer	IOB_07	chr5	140744563	C	T	PCDHGA1	INTRONIC	ENSG00000204956:[ENST00000517417(c.2421+31891C>T)]	NA	NA	NA	NA	NA	NA	NA	NA	NA	NA	MODIFIER	NA	NA	285	284	0	298	242	54	18.12
Chewer	IOB_07	chr5	140744563	C	T	PCDHGA2	INTRONIC	ENSG00000081853:[ENST00000394576(c.2424+23601C>T)]	NA	NA	NA	NA	NA	NA	NA	NA	NA	NA	MODIFIER	NA	NA	285	284	0	298	242	54	18.12
Chewer	IOB_07	chr5	140744563	C	T	PCDHGA3	INTRONIC	ENSG00000254245:[ENST00000253812(c.2424+18539C>T)]	NA	NA	NA	NA	NA	NA	NA	NA	NA	NA	MODIFIER	NA	NA	285	284	0	298	242	54	18.12
Chewer	IOB_07	chr5	140744563	C	T	PCDHGA4	INTRONIC	56111:[NM_018917.3(c.2514+7375C>T)]	NA	NA	NA	NA	NA	NA	NA	NA	NA	NA	MODIFIER	NA	NA	285	284	0	298	242	54	18.12
Chewer	IOB_07	chr5	140744563	C	T	PCDHGA5	SILENT	ENSG00000253485:[ENST00000518069(c.666C>T,ctC>ctT):ENSP00000429834(p.Leu222(=))]	NA	NA	NA	NA	NA	NA	NA	NA	NA	NA	MODIFIER	NA	NA	285	284	0	298	242	54	18.12
Chewer	IOB_07	chr5	140744563	C	T	PCDHGB1	INTRONIC	ENSG00000254221:[ENST00000523390(c.2409+12327C>T)]	NA	NA	NA	NA	NA	NA	NA	NA	NA	NA	MODIFIER	NA	NA	285	284	0	298	242	54	18.12
Chewer	IOB_07	chr5	140744563	C	T	PCDHGB2	INTRONIC	ENSG00000253910:[ENST00000522605(c.2421+2440C>T)]	NA	NA	NA	NA	NA	NA	NA	NA	NA	NA	MODIFIER	NA	NA	285	284	0	298	242	54	18.12
Chewer	IOB_07	chr5	140778730	C	A	PCDHGA1	INTRONIC	ENSG00000204956:[ENST00000517417(c.2421+66058C>A)]	NA	NA	NA	NA	NA	NA	NA	NA	NA	NA	MODIFIER	NA	NA	269	269	0	228	222	5	2.19
Chewer	IOB_07	chr5	140778730	C	A	PCDHGA2	INTRONIC	ENSG00000081853:[ENST00000394576(c.2424+57768C>A)]	NA	NA	NA	NA	NA	NA	NA	NA	NA	NA	MODIFIER	NA	NA	269	269	0	228	222	5	2.19
Chewer	IOB_07	chr5	140778730	C	A	PCDHGA3	INTRONIC	ENSG00000254245:[ENST00000253812(c.2424+52706C>A)]	NA	NA	NA	NA	NA	NA	NA	NA	NA	NA	MODIFIER	NA	NA	269	269	0	228	222	5	2.19
Chewer	IOB_07	chr5	140778730	C	A	PCDHGA4	INTRONIC	ENSG00000262576:[ENST00000571252(c.2421+41542C>A)]	NA	NA	NA	NA	NA	NA	NA	NA	NA	NA	MODIFIER	NA	NA	269	269	0	228	222	5	2.19
Chewer	IOB_07	chr5	140778730	C	A	PCDHGA5	INTRONIC	ENSG00000253485:[ENST00000518069(c.2421+32412C>A)]	NA	NA	NA	NA	NA	NA	NA	NA	NA	NA	MODIFIER	NA	NA	269	269	0	228	222	5	2.19
Chewer	IOB_07	chr5	140778730	C	A	PCDHGA6	INTRONIC	56109:[XM_005268482.1(c.4935+22656C>A)]	NA	NA	NA	NA	NA	NA	NA	NA	NA	NA	MODIFIER	NA	NA	269	269	0	228	222	5	2.19
Chewer	IOB_07	chr5	140778730	C	A	PCDHGA7	INTRONIC	ENSG00000253537:[ENST00000518325(c.2424+13840C>A)]	NA	NA	NA	NA	NA	NA	NA	NA	NA	NA	MODIFIER	NA	NA	269	269	0	228	222	5	2.19
Chewer	IOB_07	chr5	140778730	C	A	PCDHGA8	INTRONIC	ENSG00000253767:[ENST00000398604(c.2424+3926C>A)]	NA	NA	NA	NA	NA	NA	NA	NA	NA	NA	MODIFIER	NA	NA	269	269	0	228	222	5	2.19
Chewer	IOB_07	chr5	140778730	C	A	PCDHGB1	INTRONIC	ENSG00000254221:[ENST00000523390(c.2409+46494C>A)]	NA	NA	NA	NA	NA	NA	NA	NA	NA	NA	MODIFIER	NA	NA	269	269	0	228	222	5	2.19
Chewer	IOB_07	chr5	140778730	C	A	PCDHGB2	INTRONIC	ENSG00000253910:[ENST00000522605(c.2421+36607C>A)]	NA	NA	NA	NA	NA	NA	NA	NA	NA	NA	MODIFIER	NA	NA	269	269	0	228	222	5	2.19
Chewer	IOB_07	chr5	140778730	C	A	PCDHGB3	INTRONIC	ENSG00000262209:[ENST00000576222(c.2415+26354C>A)]	NA	NA	NA	NA	NA	NA	NA	NA	NA	NA	MODIFIER	NA	NA	269	269	0	228	222	5	2.19
Chewer	IOB_07	chr5	140778730	C	A	PCDHGB4	INTRONIC	ENSG00000253953:[ENST00000519479(c.2397+8882C>A)]	NA	NA	NA	NA	NA	NA	NA	NA	NA	NA	MODIFIER	NA	NA	269	269	0	228	222	5	2.19
Chewer	IOB_07	chr5	140778730	C	A	PCDHGB5	MISSENSE	56101:[NM_032099.1(c.1036C>A,Cat>Aat):NP_115270.1(p.His346Asn)]	NA	NA	NA	NA	NA	NA	NA	NA	NA	NA	MODIFIER	NA	NA	269	269	0	228	222	5	2.19
Chewer	IOB_07	chr5	145239318	G	A	GRXCR2	MISSENSE	ENSG00000204928:[ENST00000377976(c.725C>T,cCt>cTt):ENSP00000367214(p.Pro242Leu)]	NA	NA	NA	NA	NA	NA	NA	D	PrD	NA	MODERATE	NA	NA	97	97	0	103	72	30	29.13
Chewer	IOB_07	chr5	145239319	G	A	GRXCR2	MISSENSE	ENSG00000204928:[ENST00000377976(c.724C>T,Cct>Tct):ENSP00000367214(p.Pro242Ser)]	NA	chr5*145239319*145239319*COSM1696465*G*A*1*skin*1	NA	NA	NA	NA	NA	D	PrD	NA	MODERATE	NA	NA	97	97	0	104	73	31	29.81
Chewer	IOB_07	chr5	149580707	G	A	SLC6A7	MISSENSE	ENSG00000011083:[ENST00000230671(c.779G>A,cGc>cAc):ENSP00000230671(p.Arg260His)]	NA	NA	NA	1.81E-04	6.08E-05	NA	NA	D	PrD	NA	MODERATE	D	1	157	156	0	188	148	38	20.21
Chewer	IOB_07	chr6	24291186	G	T	DCDC2	MISSENSE	ENSG00000146038:[ENST00000378454(c.678C>A,gaC>gaA):ENSP00000367715(p.Asp226Glu)]	NA	NA	NA	NA	NA	NA	NA	D	BN	NA	MODERATE	N	1	177	177	0	145	115	30	20.69
Chewer	IOB_07	chr6	25605032	T	C	LRRC16A	INTRONIC-SS-ACR-PRX	ENSG00000079691:[ENST00000329474(c.3553-8T>C)]	NA	NA	NA	NA	NA	NA	NA	NA	NA	NA	LOW	NA	NA	125	125	0	138	91	44	31.88
Chewer	IOB_07	chr6	30891117	C	T	VARS2	NCT	ENSG00000137411:[ENST00000477052(n.400-13C>T)]	NA	NA	chr6*30891117*30891117*C*G,T*rs760836143*NA*NA*NA	1.74E-05	0.00E+00	NA	NA	NA	NA	NA	MODIFIER	NA	NA	143	143	0	109	87	22	20.18
Chewer	IOB_07	chr6	33651876	G	A	ITPR3	NONSENSE	ENSG00000096433:[ENST00000374316(c.4868G>A,tGg>tAg):ENSP00000363435(p.Trp1623Ter)]	NA	NA	chr6*33651868*33651883*CCTGCACTGGCCTGAG*C*rs775689384*NA*NA*NA	NA	NA	NA	NA	NA	NA	NA	HIGH	NA	1	37	37	0	41	25	16	39.02
Chewer	IOB_07	chr6	35200932	C	A	SCUBE3	INTRONIC	ENSG00000146197:[ENST00000274938(c.596-30C>A)]	NA	NA	NA	NA	NA	NA	NA	NA	NA	NA	MODIFIER	NA	NA	167	166	0	160	154	5	3.12
Chewer	IOB_07	chr6	36270139	C	T	PNPLA1	MISSENSE	ENSG00000180316:[ENST00000388715(c.992C>T,tCc>tTc):ENSP00000373367(p.Ser331Phe)]	NA	NA	NA	NA	NA	NA	NA	T_lc	BN	NA	MODERATE	N	0	158	158	0	171	128	43	25.15
Chewer	IOB_07	chr6	36943357	C	T	MTCH1	NCT	23787:[NR_130739.1(n.1232+77G>A)]	NA	NA	NA	NA	NA	NA	NA	NA	NA	NA	MODIFIER	NA	NA	131	130	0	90	84	6	6.67
Chewer	IOB_07	chr6	39893552	G	A	MOCS1	SILENT	ENSG00000124615:[ENST00000373175(c.201C>T,acC>acT):ENSP00000362270(p.Thr67(=))]	NA	NA	chr6*39893552*39893552*G*C*rs777029725*NA*NA*NA	NA	NA	NA	NA	NA	NA	NA	LOW	NA	NA	115	115	0	111	84	27	24.32
Chewer	IOB_07	chr6	41062175	G	A	NFYA	MISSENSE	ENSG00000001167:[ENST00000353205(c.842G>A,cGg>cAg):ENSP00000229418(p.Arg281Gln)]	NA	NA	NA	NA	NA	NA	NA	D	PrD	NA	MODERATE	D	1	123	123	0	157	126	29	18.47
Chewer	IOB_07	chr6	41062175	G	A	OARD1	INTRONIC	ENSG00000124596:[ENST00000488238(c.-347+3277C>T)]	NA	NA	NA	NA	NA	NA	NA	D	PrD	NA	MODERATE	D	1	123	123	0	157	126	29	18.47
Chewer	IOB_07	chr6	45165549	C	T	SUPT3H	INTRONIC	ENSG00000196284:[ENST00000371460(c.135-91806G>A)]	NA	NA	NA	NA	NA	NA	NA	NA	NA	NA	MODIFIER	NA	NA	18	18	0	27	21	6	22.22
Chewer	IOB_07	chr6	46867886	C	A	GPR116	INTRONIC	ENSG00000069122:[ENST00000265417(c.103-66G>T)]	NA	NA	NA	NA	NA	NA	NA	NA	NA	NA	MODIFIER	NA	NA	100	100	0	109	103	6	5.5
Chewer	IOB_07	chr6	50016250	C	T	DEFB112	MISSENSE-SS-PRX	ENSG00000180872:[ENST00000322246(c.115G>A,Gcc>Acc):ENSP00000319126(p.Ala39Thr)]	NA	NA	chr6*50016250*50016250*C*A,T*rs1853647*NA*NA*NA	NA	NA	NA	NA	T	BN	NA	MODERATE	N	0.276	70	69	0	53	35	18	33.96
Chewer	IOB_07	chr6	54002106	G	A	MLIP	SILENT	ENSG00000146147:[ENST00000503951(c.1083G>A,cgG>cgA):ENSP00000426830(p.Arg361(=))]	NA	NA	NA	NA	NA	NA	NA	NA	NA	NA	LOW	NA	NA	158	157	0	138	131	5	3.62
Chewer	IOB_07	chr6	80382754	G	T	SH3BGRL2	INTRONIC	ENSG00000198478:[ENST00000369838(c.46-577G>T)]	NA	NA	NA	NA	NA	NA	NA	NA	NA	NA	MODIFIER	NA	NA	141	139	0	156	118	38	24.36
Chewer	IOB_07	chr6	84862462	T	A	KIAA1009	MISSENSE	ENSG00000135315:[ENST00000403245(c.3431A>T,aAa>aTa):ENSP00000385215(p.Lys1144Ile)]	NA	NA	chr6*84862462*84862462*T*C*rs750676881*NA*NA*NA	NA	NA	NA	NA	NA	NA	NA	MODIFIER	NA	NA	241	240	0	218	159	59	27.06
Chewer	IOB_07	chr6	100062101	C	T	PRDM13	SILENT	ENSG00000112238:[ENST00000369215(c.1590C>T,tcC>tcT):ENSP00000358217(p.Ser530(=))]	NA	NA	NA	9.46E-06	6.49E-05	NA	NA	NA	NA	NA	MODIFIER	NA	NA	385	383	0	445	328	115	25.84
Chewer	IOB_07	chr6	123332125	G	A	CLVS2	INTRONIC-SS-ACR-PRX	ENSG00000146352:[ENST00000275162(c.390-5G>A)]	NA	NA	NA	8.61E-06	0.00E+00	NA	NA	NA	NA	NA	LOW	NA	NA	160	160	0	150	109	40	26.67
Chewer	IOB_07	chr6	126334219	G	T	TRMT11	MISSENSE	ENSG00000066651:[ENST00000334379(c.1111G>T,Gtc>Ttc):ENSP00000333934(p.Val371Phe)]	NA	NA	NA	NA	NA	NA	NA	D	PrD	NA	MODERATE	NA	NA	232	229	0	205	162	41	20
Chewer	IOB_07	chr6	147012263	T	C	ADGB	SILENT	ENSG00000118492:[ENST00000397944(c.1323T>C,gcT>gcC):ENSP00000381036(p.Ala441(=))]	NA	NA	NA	NA	NA	NA	NA	NA	NA	NA	MODIFIER	NA	NA	50	50	0	40	24	16	40
Chewer	IOB_07	chr7	6426907	C	T	RAC1	MISSENSE	ENSG00000136238:[ENST00000348035(c.100C>T,Cct>Tct):ENSP00000258737(p.Pro34Ser)]	NA	NA	NA	NA	NA	NA	NA	NA	NA	NA	MODIFIER	NA	NA	93	93	0	78	56	21	26.92
Chewer	IOB_07	chr7	43484725	C	T	HECW1	MISSENSE	ENSG00000002746:[ENST00000395891(c.1954C>T,Ccc>Tcc):ENSP00000379228(p.Pro652Ser)]	NA	NA	NA	9.16E-06	0.00E+00	NA	NA	NA	NA	NA	MODERATE	NA	NA	196	195	0	164	123	39	23.78
Chewer	IOB_07	chr7	44736023	C	T	OGDH	INTRONIC-SS-ACR-PRX	ENSG00000105953:[ENST00000447398(c.1805-5C>T)]	NA	NA	chr7*44736020*44736023*TCTC*T*rs753194562*NA*NA*NA	NA	NA	NA	NA	NA	NA	NA	LOW	NA	NA	141	140	0	99	79	20	20.2
Chewer	IOB_07	chr7	44804632	C	T	ZMIZ2	NCT	ENSG00000122515:[ENST00000463056(n.1400+24C>T)]	NA	NA	NA	NA	NA	NA	NA	NA	NA	NA	MODIFIER	NA	NA	163	161	0	165	132	32	19.39
Chewer	IOB_07	chr7	55914293	C	G	SEPT14	MISSENSE	ENSG00000154997:[ENST00000388975(c.92G>C,gGa>gCa):ENSP00000373627(p.Gly31Ala)]	NA	NA	NA	NA	NA	NA	NA	NA	NA	NA	MODIFIER	NA	NA	57	57	0	48	36	12	25
Chewer	IOB_07	chr7	82545527	C	A	PCLO	MISSENSE	ENSG00000186472:[ENST00000437081(c.1935G>T,caG>caT):ENSP00000393760(p.Gln645His)]	NA	NA	NA	NA	NA	NA	NA	T	PrD	NA	MODERATE	NA	NA	216	216	0	224	156	66	29.46
Chewer	IOB_07	chr7	86537057	A	G	KIAA1324L	SILENT	ENSG00000164659:[ENST00000450689(c.2487T>C,aaT>aaC):ENSP00000413445(p.Asn829(=))]	NA	NA	NA	NA	NA	NA	NA	NA	NA	NA	LOW	NA	1	214	214	0	171	133	37	21.64
Chewer	IOB_07	chr7	91771800	T	A	CTB-161K23.1	EXONIC-NC	ENSG00000188693:[ENST00000453068(n.749T>A)]	NA	NA	NA	NA	NA	NA	NA	NA	NA	NA	MODIFIER	NA	NA	83	83	0	105	83	22	20.95
Chewer	IOB_07	chr7	91771800	T	A	CYP51A1	STARTLOSS	ENSG00000001630:[ENST00000435873(c.1A>T,Atg>Ttg):ENSP00000412536(p.Met1?)];	NA	NA	NA	NA	NA	NA	NA	NA	NA	NA	MODIFIER	NA	NA	83	83	0	105	83	22	20.95
Chewer	IOB_07	chr7	91771800	T	A	CYP51A1-AS1	NCT	613126:[NR_122110.1(n.520+7404T>A)]	NA	NA	NA	NA	NA	NA	NA	NA	NA	NA	MODIFIER	NA	NA	83	83	0	105	83	22	20.95
Chewer	IOB_07	chr7	91771800	T	A	LRRD1	3UTR	ENSG00000240720:[ENST00000343318(c.*324A>T)]	NA	NA	NA	NA	NA	NA	NA	NA	NA	NA	MODIFIER	NA	NA	83	83	0	105	83	22	20.95
Chewer	IOB_07	chr7	92764581	C	G	SAMD9L	MISSENSE	ENSG00000177409:[ENST00000318238(c.704G>C,gGc>gCc):ENSP00000326247(p.Gly235Ala)]	NA	NA	NA	NA	NA	NA	NA	D	PrD	NA	MODERATE	D	1	275	272	0	318	236	81	25.47
Chewer	IOB_07	chr7	99258130	G	A	CYP3A5	MISSENSE	ENSG00000106258:[ENST00000222982(c.1018C>T,Ccc>Tcc):ENSP00000222982(p.Pro340Ser)]	NA	NA	NA	NA	NA	NA	NA	NA	NA	NA	MODIFIER	NA	NA	83	82	1	87	82	5	5.75
Chewer	IOB_07	chr7	99258130	G	A	ZSCAN25	INTRONIC	221785:[XM_005250198.1(c.806-16697G>A)]	NA	NA	NA	NA	NA	NA	NA	NA	NA	NA	MODIFIER	NA	NA	83	82	1	87	82	5	5.75
Chewer	IOB_07	chr7	100385657	C	T	ZAN	MISSENSE	ENSG00000146839:[ENST00000538115(c.7123C>T,Cgc>Tgc):ENSP00000445091(p.Arg2375Cys)]	NA	NA	NA	2.53E-05	1.32E-04	NA	NA	NA	NA	NA	LOW	NA	NA	253	252	0	222	178	42	18.92
Chewer	IOB_07	chr7	129357152	G	C	NRF1	MISSENSE	ENSG00000106459:[ENST00000539636(c.676G>C,Gag>Cag):ENSP00000440455(p.Glu226Gln)]	NA	NA	NA	NA	NA	NA	NA	T	BN	NA	MODERATE	D	1	155	153	0	127	107	20	15.75
Chewer	IOB_07	chr7	142032369	G	C	TRBV7-1	MISSENSE	ENSG00000211707:[ENST00000547918(c.189G>C,gaG>gaC):ENSP00000448600(p.Glu63Asp)]	NA	NA	NA	NA	NA	NA	NA	D	BN	NA	MODERATE	NA	NA	186	186	0	173	131	41	23.7
Chewer	IOB_07	chr7	142459713	C	T	PRSS1	MISSENSE	ENSG00000204983:[ENST00000486171(c.331C>T,Ccc>Tcc):ENSP00000417854(p.Pro111Ser)]	NA	NA	NA	NA	NA	NA	NA	T	BN	NA	MODERATE	D	1	194	193	1	223	170	52	23.32
Chewer	IOB_07	chr7	142983139	G	A	AC073342.12	NCT	ENSG00000231840:[ENST00000446192(n.339-208C>T)]	NA	NA	NA	NA	NA	NA	NA	NA	NA	NA	MODIFIER	NA	NA	117	117	0	133	128	5	3.76
Chewer	IOB_07	chr7	142983139	G	A	LOC105375545	EXONIC-NC	105375545:[NR_133932.1(n.289C>T)]	NA	NA	NA	NA	NA	NA	NA	NA	NA	NA	MODIFIER	NA	NA	117	117	0	133	128	5	3.76
Chewer	IOB_07	chr7	142983139	G	A	TMEM139	MISSENSE	ENSG00000178826:[ENST00000359333(c.89G>A,gGc>gAc):ENSP00000352284(p.Gly30Asp)]	NA	NA	NA	NA	NA	NA	NA	NA	NA	NA	MODIFIER	NA	NA	117	117	0	133	128	5	3.76
Chewer	IOB_07	chr7	149490706	C	A	SSPO	MISSENSE-SS-PRX	23145:[NM_198455.2(c.6084C>A,cCt>cAt):NP_940857.2(p.Pro2027His)]	NA	NA	chr7*149490706*149490706*C*T*rs763243674*NA*NA*NA	NA	NA	NA	NA	NA	NA	NA	LOW	NA	NA	57	55	0	81	74	6	7.41
Chewer	IOB_07	chr7	157370929	C	T	PTPRN2	INTRONIC	ENSG00000155093:[ENST00000389413(c.2410-97G>A)]	NA	NA	NA	NA	NA	NA	NA	NA	NA	NA	MODIFIER	NA	NA	122	122	0	136	102	33	24.26
**Chewer**	**IOB_07**	**chr8**	**3063101**	**A**	**C**	**CSMD1**	**MISSENSE**	ENSG00000183117:[ENST00000400186(c.4912T>G,Tta>Gta):ENSP00000383047(p.Leu1638Val)]	NA	NA	NA	NA	NA	NA	NA	NA	NA	NA	MODIFIER	NA	NA	46	46	0	45	32	13	28.89
Chewer	IOB_07	chr8	11172400	C	T	MTMR9	INTRONIC	66036:[XM_005272396.1(c.651-32C>T)]	NA	NA	NA	1.66E-05	0.00E+00	NA	NA	NA	NA	NA	MODIFIER	NA	NA	81	81	0	57	41	16	28.07
Chewer	IOB_07	chr8	12957918	G	A	DLC1	MISSENSE	ENSG00000164741:[ENST00000276297(c.1928C>T,cCc>cTc):ENSP00000276297(p.Pro643Leu)]	NA	NA	NA	NA	NA	NA	NA	D	BN	NA	MODERATE	D	1	236	235	0	209	148	61	29.19
Chewer	IOB_07	chr8	16971574	T	A	MICU3	INTRONIC	286097:[XM_005273478.1(c.730-28T>A)]	NA	NA	NA	8.68E-06	0.00E+00	NA	NA	NA	NA	NA	MODIFIER	NA	NA	84	80	0	56	38	17	30.36
Chewer	IOB_07	chr8	22021735	C	T	SFTPC	INTRONIC	ENSG00000168484:[ENST00000520605(c.277-44C>T)]	NA	NA	NA	NA	NA	NA	NA	NA	NA	NA	MODIFIER	NA	NA	79	79	0	108	80	28	25.93
Chewer	IOB_07	chr8	24366077	C	T	ADAM7	NONSENSE	8756:[XM_005273672.1(c.2212C>T,Caa>Taa):XP_005273729.1(p.Gln738Ter)]	NA	NA	NA	NA	NA	NA	NA	NA	NA	NA	HIGH	NA	NA	177	176	0	191	153	38	19.9
Chewer	IOB_07	chr8	24366077	C	T	LOC101929315	NCT	101929315:[NR_125809.1(n.446+3107G>A)]	NA	NA	NA	NA	NA	NA	NA	NA	NA	NA	HIGH	NA	NA	177	176	0	191	153	38	19.9
Chewer	IOB_07	chr8	24366077	C	T	RP11-561E1.1	NCT	ENSG00000253643:[ENST00000519364(n.406+3107G>A)]	NA	NA	NA	NA	NA	NA	NA	NA	NA	NA	HIGH	NA	NA	177	176	0	191	153	38	19.9
Chewer	IOB_07	chr8	24366077	C	T	RP11-624C23.1	NCT	ENSG00000253535:[ENST00000519689(n.185-120573G>A)]	NA	NA	NA	NA	NA	NA	NA	NA	NA	NA	HIGH	NA	NA	177	176	0	191	153	38	19.9
Chewer	IOB_07	chr8	35544154	G	A	LOC101929550	NCT	101929550:[NR_125819.1(n.4097-6504C>T)]	NA	NA	NA	8.48E-06	0.00E+00	NA	NA	NA	NA	NA	MODIFIER	NA	NA	98	97	0	80	59	20	25
Chewer	IOB_07	chr8	35544154	G	A	UNC5D	SILENT	ENSG00000156687:[ENST00000287272(c.843G>A,ccG>ccA):ENSP00000287272(p.Pro281(=))]	NA	NA	NA	8.48E-06	0.00E+00	NA	NA	NA	NA	NA	MODIFIER	NA	NA	98	97	0	80	59	20	25
Chewer	IOB_07	chr8	41814534	C	G	KAT6A	INTRONIC	ENSG00000083168:[ENST00000265713(c.1483-1605G>C)]	NA	NA	NA	NA	NA	NA	NA	NA	NA	NA	MODIFIER	NA	NA	44	44	0	65	47	18	27.69
Chewer	IOB_07	chr8	60031621	G	A	RP11-25K19.1	EXONIC-NC	ENSG00000167912:[ENST00000523683(n.23G>A)]	NA	NA	NA	NA	NA	NA	NA	NA	NA	NA	MODIFIER	NA	NA	61	60	0	57	39	17	29.82
Chewer	IOB_07	chr8	60031621	G	A	TOX	5UTR	ENSG00000198846:[ENST00000361421(c.-75C>T)]	NA	NA	NA	NA	NA	NA	NA	NA	NA	NA	MODIFIER	NA	NA	61	60	0	57	39	17	29.82
Chewer	IOB_07	chr8	60031622	G	A	RP11-25K19.1	EXONIC-NC	ENSG00000167912:[ENST00000523683(n.24G>A)]	NA	NA	chr8*60031622*60031622*G*C*rs949572108*NA*NA*NA	NA	NA	NA	NA	NA	NA	NA	MODIFIER	NA	NA	58	57	0	56	39	16	28.57
Chewer	IOB_07	chr8	60031622	G	A	TOX	5UTR	ENSG00000198846:[ENST00000361421(c.-76C>T)]	NA	NA	chr8*60031622*60031622*G*C*rs949572108*NA*NA*NA	NA	NA	NA	NA	NA	NA	NA	MODIFIER	NA	NA	58	57	0	56	39	16	28.57

Patel *et al.* , 2021. Whole-exome sequencing analysis of oral squamous cell carcinoma delineated by tobacco usage habits
Supplementary Table 4. List of somatic single nucleotide variants identified using whole exome sequencing of OSCC patients

Cohort	Sample	Chromosome	Genomic position	Reference allele	Alternate allele	Genesymbol	Variant type	Variant description	OncoMD	COSMIC	dbSNP	ExAC	ExAC_SAS	1000G	1000G_SAS	SIFT	Polyphen2	CONDEL	VEP	LRT	PhastCon7Way	Total reads in Normal	Reference read support in Normal	Alternative read support in Normal	Total reads in Tumor	Reference read support in Tumor	Alternate read support in Tumor	Alternate allele frequency in Tumor
Chewer	IOB_07	chr8	67507929	G	A	MYBL1	SILENT	ENSG00000185697:[ENST00000522677(c.576C>T,ggC>ggT):ENSP00000429633(p.Gly192(=))]	NA	NA	NA	NA	NA	NA	NA	NA	NA	NA	MODIFIER	NA	NA	57	57	0	55	48	7	12.73
Chewer	IOB_07	chr8	70501399	C	A	SULF1	INTRONIC	ENSG00000137573:[ENST00000260128(c.734+23C>A)]	NA	NA	chr8*70501399*70501399*C*T*rs767554475*NA*NA*NA	NA	NA	NA	NA	NA	NA	NA	MODIFIER	NA	NA	87	87	0	117	88	29	24.79
Chewer	IOB_07	chr8	77754915	G	A	ZFHX4	MISSENSE	ENSG00000091656:[ENST00000521891(c.3419G>A,gGa>gAa):ENSP00000430497(p.Gly1140Glu)]	NA	NA	NA	NA	NA	NA	NA	NA	NA	NA	MODIFIER	NA	NA	63	63	0	57	46	11	19.3
Chewer	IOB_07	chr8	91094212	G	A	CALB1	INTRONIC	ENSG00000104327:[ENST00000514406(c.-16+42C>T)]	NA	NA	NA	NA	NA	NA	NA	NA	NA	NA	MODIFIER	NA	NA	125	125	0	141	108	32	22.7
Chewer	IOB_07	chr8	95895053	G	A	CCNE2	MISSENSE	ENSG00000175305:[ENST00000308108(c.899C>T,gCc>gTc):ENSP00000309181(p.Ala300Val)]	NA	NA	NA	NA	NA	NA	NA	D	PoD	NA	MODERATE	D	1	191	191	0	227	168	58	25.55
Chewer	IOB_07	chr8	107752688	C	T	OXR1	MISSENSE	ENSG00000164830:[ENST00000442977(c.2284C>T,Cca>Tca):ENSP00000405424(p.Pro762Ser)]	NA	NA	NA	NA	NA	NA	NA	D	PrD	NA	MODERATE	D	1	149	149	0	133	104	29	21.8
Chewer	IOB_07	chr8	113299359	G	A	CSMD3	MISSENSE	ENSG00000164796:[ENST00000352409(c.9055C>T,Cat>Tat):ENSP00000343124(p.His3019Tyr)]	NA	chr8*113299359*113299359*COSM387927*G*A*1*lung*1	NA	8.25E-06	6.06E-05	NA	NA	T	BN	NA	MODERATE	N	0.015	205	205	0	218	172	46	21.1
Chewer	IOB_07	chr8	121353641	C	A	COL14A1	INTRONIC	ENSG00000187955:[ENST00000297848(c.4821+42C>A)]	NA	NA	NA	NA	NA	NA	NA	NA	NA	NA	MODIFIER	NA	NA	184	182	0	180	173	6	3.33
Chewer	IOB_07	chr8	124085124	G	C	TBC1D31	NCT	93594:[XR_242399.1(n.81+38G>C)]	NA	NA	NA	NA	NA	NA	NA	NA	NA	NA	MODIFIER	NA	NA	173	171	1	233	179	53	22.75
Chewer	IOB_07	chr8	142489377	A	C	MROH5	MISSENSE	ENSG00000226807:[ENST00000430863(c.1025T>G,gTc>gGc):ENSP00000431031(p.Val342Gly)]	NA	NA	chr8*142489377*142489377*A*G*rs961938411*NA*NA*NA	NA	NA	NA	NA	NA	NA	NA	MODIFIER	NA	NA	92	92	0	84	55	29	34.52
Chewer	IOB_07	chr8	144687950	C	T	PYCRL	MISSENSE	ENSG00000104524:[ENST00000433751(c.706G>A,Gag>Aag):ENSP00000404493(p.Glu236Lys)]	NA	chr8*144687926*144687953*COSM111750*BigSeq*T*1*ovary*1	NA	NA	NA	NA	NA	D	PrD	NA	MODERATE	NA	NA	54	54	0	48	34	14	29.17
Chewer	IOB_07	chr8	144688362	C	T	PYCRL	NCT	65263:[NR_138144.1(n.729-38G>A)]	NA	NA	chr8*144688362*144688362*C*G*rs1048055622*NA*NA*NA	NA	NA	NA	NA	NA	NA	NA	MODIFIER	NA	NA	110	108	0	104	82	19	18.27
Chewer	IOB_07	chr8	144789081	G	A	CCDC166	MISSENSE	ENSG00000255181:[ENST00000542437(c.1103C>T,tCc>tTc):ENSP00000437468(p.Ser368Phe)]	NA	NA	NA	NA	NA	NA	NA	NA	NA	NA	MODIFIER	NA	NA	297	297	0	287	209	74	25.78
Chewer	IOB_07	chr8	144789081	G	A	ZNF707	NCT	ENSG00000181135:[ENST00000527561(n.1089-2342G>A)]	NA	NA	NA	NA	NA	NA	NA	NA	NA	NA	MODIFIER	NA	NA	297	297	0	287	209	74	25.78
Chewer	IOB_07	chr9	842177	C	T	DMRT1	SILENT	ENSG00000137090:[ENST00000382276(c.339C>T,cgC>cgT):ENSP00000371711(p.Arg113(=))]	NA	NA	NA	NA	NA	NA	NA	NA	NA	NA	MODIFIER	NA	NA	84	83	0	125	71	51	40.8
Chewer	IOB_07	chr9	18906821	G	A	ADAMTSL1	MISSENSE	ENSG00000178031:[ENST00000380548(c.5093G>A,cGc>cAc):ENSP00000369921(p.Arg1698His)]	NA	NA	NA	9.95E-05	0.00E+00	NA	NA	NA	NA	NA	LOW	NA	NA	316	316	0	456	446	5	1.1
Chewer	IOB_07	chr9	27949328	G	A	LINGO2	NONSENSE	ENSG00000174482:[ENST00000308675(c.1342C>T,Cga>Tga):ENSP00000310126(p.Arg448Ter)]	NA	NA	NA	NA	NA	NA	NA	NA	NA	NA	HIGH	NA	1	190	188	0	205	118	86	41.95
Chewer	IOB_07	chr9	32487962	G	A	DDX58	MISSENSE	ENSG00000107201:[ENST00000542096(c.980C>T,tCt>tTt):ENSP00000442160(p.Ser327Phe)]	NA	NA	NA	NA	NA	NA	NA	D	PrD	NA	MODERATE	N	0.997	129	128	1	160	131	29	18.12
Chewer	IOB_07	chr9	72913113	G	T	SMC5	NONSENSE	ENSG00000198887:[ENST00000361138(c.1285G>T,Gaa>Taa):ENSP00000354957(p.Glu429Ter)]	NA	NA	NA	NA	NA	NA	NA	NA	NA	NA	HIGH	NA	1	100	99	0	106	81	25	23.58
Chewer	IOB_07	chr9	77390963	G	A	TRPM6	MISSENSE	ENSG00000119121:[ENST00000360774(c.3239C>T,tCa>tTa):ENSP00000354006(p.Ser1080Leu)]	NA	NA	NA	NA	NA	NA	NA	NA	NA	NA	MODIFIER	NA	NA	178	177	0	237	189	46	19.41
Chewer	IOB_07	chr9	77407512	C	A	TRPM6	INTRONIC	ENSG00000119121:[ENST00000376871(c.1497+18219G>T)]	NA	NA	chr9*77407512*77407512*C*T*rs73650078*1*0.996*0.003994	NA	NA	NA	NA	NA	NA	NA	MODIFIER	NA	NA	109	109	0	135	130	5	3.7
Chewer	IOB_07	chr9	78710821	G	A	PCSK5	MISSENSE	ENSG00000099139:[ENST00000376752(c.910G>A,Ggc>Agc):ENSP00000365943(p.Gly304Ser)]	NA	NA	NA	NA	NA	NA	NA	D	PrD	NA	MODERATE	D	1	80	80	0	88	70	18	20.45
Chewer	IOB_07	chr9	82334990	A	C	TLE4	MISSENSE	ENSG00000106829:[ENST00000376544(c.1413A>C,agA>agC):ENSP00000365727(p.Arg471Ser)]	NA	NA	chr9*82334990*82334990*A*G*rs566099337*0*0.9998*0.0001997	NA	NA	NA	NA	NA	NA	NA	MODERATE	NA	1	101	99	0	129	80	48	37.21
Chewer	IOB_07	chr9	130886138	G	A	PTGES2	NCT	ENSG00000148334:[ENST00000487063(n.258-12C>T)]	NA	NA	NA	NA	NA	NA	NA	NA	NA	NA	LOW	NA	NA	110	110	0	124	98	26	20.97
Chewer	IOB_07	chr9	130982360	G	A	DNM1	MISSENSE	ENSG00000106976:[ENST00000341179(c.683G>A,cGc>cAc):ENSP00000345680(p.Arg228His)]	NA	NA	NA	NA	NA	NA	NA	NA	NA	NA	MODIFIER	NA	NA	176	176	0	193	154	36	18.65
Chewer	IOB_07	chr9	131447476	G	A	SET	SILENT	ENSG00000119335:[ENST00000409104(c.30G>A,ttG>ttA):ENSP00000387321(p.Leu10(=))]	NA	NA	NA	NA	NA	NA	NA	NA	NA	NA	MODIFIER	NA	NA	74	74	0	82	73	7	8.54
Chewer	IOB_07	chr9	135865251	C	T	GFI1B	SILENT	ENSG00000165702:[ENST00000372123(c.633C>T,ttC>ttT):ENSP00000361196(p.Phe211(=))]	NA	NA	NA	NA	NA	NA	NA	NA	NA	NA	LOW	NA	1	323	323	0	381	318	60	15.75
Chewer	IOB_07	chr9	136518044	A	C	DBH	INTRONIC	ENSG00000123454:[ENST00000393056(c.1375-18A>C)]	NA	NA	NA	NA	NA	NA	NA	NA	NA	NA	MODIFIER	NA	NA	81	81	0	104	84	19	18.27
Chewer	IOB_07	chr9	139412333	A	C	NOTCH1	MISSENSE	ENSG00000148400:[ENST00000277541(c.1312T>G,Tgc>Ggc):ENSP00000277541(p.Cys438Gly)]	NA	NA	NA	NA	NA	NA	NA	D	PrD	NA	MODERATE	D	1	140	138	0	218	174	44	20.18
Chewer	IOB_07	chr9	139981574	G	T	MAN1B1	SILENT	ENSG00000177239:[ENST00000544448(c.138G>T,ccG>ccT):ENSP00000444966(p.Pro46(=))]	NA	NA	chr9*139981568*139981574*ACCGCCG*A*rs766871551*NA*NA*NA	NA	NA	NA	NA	NA	NA	NA	MODIFIER	NA	NA	129	128	0	182	156	24	13.19
Chewer	IOB_07	chr9	140110716	C	T	NDOR1	INTRONIC	27158:[XM_005266065.1(c.1806-8C>T)]	NA	NA	NA	1.66E-05	6.06E-05	NA	NA	NA	NA	NA	LOW	NA	NA	275	273	1	415	342	72	17.35
Chewer	IOB_07	chrX	2986222	C	A	ARSF	INTRONIC	ENSG00000062096:[ENST00000537104(c.12-3845C>A)]	NA	NA	NA	NA	NA	NA	NA	NA	NA	NA	MODIFIER	NA	NA	88	88	0	106	99	7	6.6
Chewer	IOB_07	chrX	8270699	C	T	NA	INTERGENIC	NA	NA	NA	NA	NA	NA	NA	NA	NA	NA	NA	MODIFIER	NA	NA	142	141	0	158	122	34	21.52
Chewer	IOB_07	chrX	14862032	T	A	FANCB	MISSENSE	ENSG00000181544:[ENST00000324138(c.2237A>T,aAt>aTt):ENSP00000326819(p.Asn746Ile)]	NA	NA	NA	NA	NA	NA	NA	T	PoD	NA	MODERATE	NA	NA	92	92	0	69	53	16	23.19
Chewer	IOB_07	chrX	14862801	T	C	FANCB	SILENT	ENSG00000181544:[ENST00000324138(c.1989A>G,acA>acG):ENSP00000326819(p.Thr663(=))]	NA	NA	NA	NA	NA	NA	NA	NA	NA	NA	LOW	NA	NA	195	194	0	219	163	56	25.57
Chewer	IOB_07	chrX	27765661	C	T	AC107613.1	EXONIC-NC	ENSG00000231315:[ENST00000545306(n.562C>T)]	NA	NA	NA	NA	NA	NA	NA	D	PrD	NA	MODERATE	NA	NA	200	198	1	168	113	53	31.55
Chewer	IOB_07	chrX	27765661	C	T	DCAF8L2	MISSENSE	ENSG00000189186:[ENST00000451261(c.649C>T,Cgt>Tgt):ENSP00000462745(p.Arg217Cys)]	NA	NA	NA	NA	NA	NA	NA	D	PrD	NA	MODERATE	NA	NA	200	198	1	168	113	53	31.55
Chewer	IOB_07	chrX	46330681	A	G	KRBOX4	INTRONIC	ENSG00000147121:[ENST00000360017(c.*61A>G)]	NA	NA	NA	NA	NA	NA	NA	NA	NA	NA	MODIFIER	NA	NA	64	64	0	43	32	11	25.58
Chewer	IOB_07	chrX	49067054	C	A	CACNA1F	MISSENSE	ENSG00000102001:[ENST00000376251(c.4319G>T,tGc>tTc):ENSP00000365427(p.Cys1440Phe)]	NA	NA	NA	2.86E-05	0.00E+00	NA	NA	D	PrD	NA	MODERATE	D	1	103	102	0	94	72	22	23.4
Chewer	IOB_07	chrX	50053281	G	A	CCNB3	SILENT	ENSG00000147082:[ENST00000276014(c.2112G>A,gaG>gaA):ENSP00000276014(p.Glu704(=))]	NA	NA	NA	NA	NA	NA	NA	NA	NA	NA	MODIFIER	NA	NA	138	138	0	134	108	25	18.66
Chewer	IOB_07	chrX	55187324	G	A	FAM104B	EXONIC-NC	ENSG00000182518:[ENST00000478918(n.51C>T)]	NA	NA	NA	NA	NA	NA	NA	NA	NA	NA	MODIFIER	NA	NA	189	188	0	233	171	59	25.32
Chewer	IOB_07	chrX	79283538	C	T	TBX22	SILENT	ENSG00000122145:[ENST00000373291(c.552C>T,ttC>ttT):ENSP00000362388(p.Phe184(=))]	NA	NA	NA	NA	NA	NA	NA	NA	NA	NA	LOW	NA	1	250	250	0	197	140	57	28.93
Chewer	IOB_07	chrX	91066273	G	C	PCDH11X	MISSENSE	27328:[XM_005262122.1(c.22G>C,Gtt>Ctt):XP_005262179.1(p.Val8Leu)]	NA	NA	NA	NA	NA	NA	NA	NA	NA	NA	MODIFIER	NA	NA	271	269	1	220	100	119	54.09
Chewer	IOB_07	chrX	110395652	G	A	PAK3	MISSENSE	ENSG00000077264:[ENST00000372007(c.445G>A,Gga>Aga):ENSP00000361077(p.Gly149Arg)]	NA	NA	NA	NA	NA	NA	NA	T	BN	NA	MODERATE	D	1	64	63	0	64	51	13	20.31
Chewer	IOB_07	chrX	112058575	G	A	AMOT	INTRONIC	ENSG00000126016:[ENST00000304758(c.165+11C>T)]	NA	NA	NA	4.87E-05	0.00E+00	NA	NA	NA	NA	NA	MODIFIER	NA	NA	142	142	0	140	107	33	23.57
Chewer	IOB_07	chrX	114845209	C	T	PLS3	INTRONIC	ENSG00000102024:[ENST00000539310(c.-445+574C>T)]	NA	NA	NA	NA	NA	NA	NA	NA	NA	NA	MODIFIER	NA	NA	72	72	0	65	56	8	12.31
Chewer	IOB_07	chrX	135056151	G	A	MMGT1	5UTR	93380:[NM_001330000.1(c.-240C>T)]	NA	NA	NA	NA	NA	NA	NA	NA	NA	NA	MODIFIER	NA	NA	70	66	1	74	56	15	20.27
Chewer	IOB_07	chrX	135056152	G	A	MMGT1	5UTR	93380:[XM_005262496.1(c.-46C>T)]	NA	NA	NA	NA	NA	NA	NA	NA	NA	NA	MODIFIER	NA	NA	69	66	0	74	56	15	20.27
Chewer	IOB_07	chrX	136112781	A	G	GPR101	SILENT	ENSG00000165370:[ENST00000298110(c.1053T>C,gaT>gaC):ENSP00000298110(p.Asp351(=))]	NA	NA	NA	NA	NA	NA	NA	NA	NA	NA	LOW	NA	0.717	203	203	0	178	133	45	25.28
Chewer	IOB_07	chrX	140983252	C	G	MAGEC3	SILENT	ENSG00000165509:[ENST00000448920(c.363C>G,gtC>gtG):ENSP00000395092(p.Val121(=))]	NA	NA	1.06E-03	3.18E-04	0.00E+00	1.06E-03	0.00E+00	NA	NA	NA	MODIFIER	NA	NA	117	117	0	122	106	15	12.3
Chewer	IOB_07	chrX	152915638	C	T	DUSP9	MISSENSE	ENSG00000130829:[ENST00000342782(c.1033C>T,Cgg>Tgg):ENSP00000345853(p.Arg345Trp)]	NA	NA	NA	NA	NA	NA	NA	D	PoD	NA	MODERATE	N	1	188	187	1	215	207	6	2.79
Chewer	IOB_07	chrX	154194756	C	T	F8	MISSENSE	ENSG00000185010:[ENST00000360256(c.1216G>A,Gct>Act):ENSP00000353393(p.Ala406Thr)]	NA	chrX*154194756*154194756*COSM1118292*C*A*1*endometrium*1|chrX*154194756*154194756*COSM1118293*C*A*1*endometrium*1	NA	NA	NA	NA	NA	D	PoD	NA	MODERATE	D	1	190	190	0	205	199	6	2.93
Chewer	IOB_08	chr1	1959071	C	A	GABRD	MISSENSE	ENSG00000187730:[ENST00000378585(c.526C>A,Cag>Aag):ENSP00000367848(p.Gln176Lys)]	NA	NA	NA	NA	NA	NA	NA	D	PrD	NA	MODERATE	D	1	167	166	0	154	145	9	5.84
Chewer	IOB_08	chr1	22927869	G	A	EPHA8	MISSENSE	ENSG00000070886:[ENST00000166244(c.2806G>A,Ggg>Agg):ENSP00000166244(p.Gly936Arg)]	NA	NA	NA	1.71E-05	1.21E-04	NA	NA	D	BN	NA	MODERATE	N	1	74	72	0	107	97	10	9.35
Chewer	IOB_08	chr1	43748811	C	A	C1orf210	INTRONIC	ENSG00000253313:[ENST00000423420(c.20-33G>T)]	NA	NA	NA	NA	NA	NA	NA	NA	NA	NA	MODIFIER	NA	NA	105	105	0	199	194	5	2.51
Chewer	IOB_08	chr1	144880864	T	A	PDE4DIP	INTRONIC-SS-ACR-PRX	ENSG00000178104:[ENST00000313382(c.3635-3A>T)]	NA	NA	NA	7.43E-02	1.06E-01	NA	NA	NA	NA	NA	MODIFIER	NA	NA	52	48	4	51	33	18	35.29
Chewer	IOB_08	chr1	151787886	C	T	RORC	MISSENSE	ENSG00000143365:[ENST00000392697(c.476G>A,cGc>cAc):ENSP00000376461(p.Arg159His)]	NA	NA	NA	NA	NA	NA	NA	D	PrD	NA	MODERATE	U	1	120	119	0	126	120	6	4.76
Chewer	IOB_08	chr1	153320411	C	T	PGLYRP4	EXONIC-NC	57115:[XR_241090.1(n.80G>A)]	NA	NA	NA	NA	NA	NA	NA	NA	NA	NA	MODIFIER	NA	NA	86	86	0	96	91	5	5.21
Chewer	IOB_08	chr1	169698742	G	A	C1orf112	NCT	ENSG00000000460:[ENST00000498289(n.851+45669G>A)]	NA	NA	NA	NA	NA	NA	NA	T	BN	NA	MODERATE	N	0	172	172	0	184	170	12	6.52
Chewer	IOB_08	chr1	169698742	G	A	SELE	MISSENSE	ENSG00000007908:[ENST00000333360(c.788C>T,cCa>cTa):ENSP00000331736(p.Pro263Leu)]	NA	NA	NA	NA	NA	NA	NA	T	BN	NA	MODERATE	N	0	172	172	0	184	170	12	6.52
Chewer	IOB_08	chr1	180904979	C	A	KIAA1614	MISSENSE	ENSG00000135835:[ENST00000367588(c.1934C>A,cCg>cAg):ENSP00000356560(p.Pro645Gln)]	NA	NA	chr1*180904979*180904979*C*CGCGACT*rs762591781*NA*NA*NA|chr1*180904979*180904979*C*G,T*rs776463337*NA*NA*NA	NA	NA	NA	NA	T	PrD	NA	MODERATE	N	0.013	75	74	1	96	89	7	7.29
Chewer	IOB_08	chr1	214557782	G	A	PTPN14	SILENT	ENSG00000152104:[ENST00000366956(c.1416C>T,atC>atT):ENSP00000355923(p.Ile472(=))]	NA	NA	NA	NA	NA	NA	NA	NA	NA	NA	MODIFIER	NA	NA	338	338	0	338	324	14	4.14
Chewer	IOB_08	chr1	248262763	T	A	OR2L13	MISSENSE	ENSG00000196071:[ENST00000358120(c.86T>A,cTt>cAt):ENSP00000350836(p.Leu29His)]	NA	NA	NA	NA	NA	NA	NA	D	PoD	NA	MODERATE	N	0	237	236	0	168	158	9	5.36
Chewer	IOB_08	chr10	47701137	C	A	ANTXRL	MISSENSE	ENSG00000198250:[ENST00000537271(c.1555C>A,Caa>Aaa):ENSP00000455843(p.Gln519Lys)]	NA	NA	NA	NA	NA	NA	NA	NA	NA	NA	HIGH	NA	NA	436	431	0	483	451	29	6
Chewer	IOB_08	chr10	102051150	T	A	PKD2L1	MISSENSE	ENSG00000107593:[ENST00000338519(c.1690A>T,Acg>Tcg):ENSP00000345068(p.Thr564Ser)]	NA	NA	chr10*102051150*102051150*T*C*rs971372607*NA*NA*NA	NA	NA	NA	NA	NA	NA	NA	MODIFIER	NA	NA	114	114	0	79	70	9	11.39
Chewer	IOB_08	chr10	104122240	C	A	GBF1	MISSENSE	ENSG00000107862:[ENST00000369983(c.1692C>A,ttC>ttA):ENSP00000359000(p.Phe564Leu)]	NA	NA	NA	NA	NA	NA	NA	T	PoD	NA	MODERATE	D	0.668	90	90	0	101	96	5	4.95
Chewer	IOB_08	chr10	115601311	C	A	DCLRE1A	MISSENSE	ENSG00000198924:[ENST00000361384(c.2674G>T,Gat>Tat):ENSP00000355185(p.Asp892Tyr)]	NA	NA	NA	NA	NA	NA	NA	D	BN	NA	MODERATE	D	1	143	141	0	110	104	6	5.45
Chewer	IOB_08	chr10	117849281	G	T	GFRA1	MISSENSE	ENSG00000151892:[ENST00000544592(c.805C>A,Cat>Aat):ENSP00000442179(p.His269Asn)]	NA	NA	NA	NA	NA	NA	NA	T	BN	NA	MODERATE	D	1	40	40	0	64	55	8	12.5

Patel *et al.* , 2021. Whole-exome sequencing analysis of oral squamous cell carcinoma delineated by tobacco usage habits
Supplementary Table 4. List of somatic single nucleotide variants identified using whole exome sequencing of OSCC patients

Cohort	Sample	Chromosome	Genomic position	Reference allele	Alternate allele	Genesymbol	Variant type	Variant description	OncoMD	COSMIC	dbSNP	ExAC	ExAC_SAS	1000G	1000G_SAS	SIFT	Polyphen2	CONDEL	VEP	LRT	PhastCon7Way	Total reads in Normal	Reference read support in Normal	Alternative read support in Normal	Total reads in Tumor	Reference read support in Tumor	Alternate read support in Tumor	Alternate allele frequency in Tumor
Chewer	IOB_08	chr10	131641655	G	T	EBF3	INTRONIC	ENSG00000108001:[ENST00000355311(c.1129-189C>A)]	NA	NA	5.59E-03	4.07E-03	0.00E+00	5.59E-03	0.00E+00	NA	NA	NA	MODIFIER	NA	NA	153	152	0	189	178	11	5.82
Chewer	IOB_08	chr10	131666040	G	T	EBF3	INTRONIC-SS-DNR-PRX	ENSG00000108001:[ENST00000368648(c.885+6C>A)]	NA	NA	NA	NA	NA	NA	NA	NA	NA	NA	LOW	NA	NA	289	285	0	221	214	6	2.71
Chewer	IOB_08	chr11	6478177	C	T	TRIM3	MISSENSE	ENSG00000110171:[ENST00000345851(c.779G>A,cGc>cAc):ENSP00000340797(p.Arg260His)]	NA	NA	chr11*6478177*6478177*C*A*rs770574279*NA*NA*NA	NA	NA	NA	NA	NA	NA	NA	MODIFIER	NA	NA	217	214	2	242	233	7	2.89
Chewer	IOB_08	chr11	17531110	G	T	USH1C	SILENT	ENSG00000006611:[ENST00000005226(c.1806C>A,ccC>ccA):ENSP00000005226(p.Pro602(=))]	NA	NA	chr11*17531109*17531110*TG*T*rs765346539*NA*NA*NA|chr11*17531110*17531110*G*C*rs763323363*NA*NA*NA	NA	NA	NA	NA	NA	NA	NA	MODIFIER	NA	NA	36	34	0	68	58	8	11.76
Chewer	IOB_08	chr11	47806440	G	T	NUP160	NCT	23279:[NR_134636.1(n.4129+34C>A)]	NA	NA	NA	NA	NA	NA	NA	NA	NA	NA	MODIFIER	NA	NA	139	139	0	124	118	6	4.84
Chewer	IOB_08	chr11	65304202	C	A	SCYL1	SILENT	ENSG00000142186:[ENST00000527009(c.1368C>A,ccC>ccA):ENSP00000436993(p.Pro456(=))]	NA	NA	NA	NA	NA	NA	NA	NA	NA	NA	LOW	NA	NA	227	227	0	197	189	5	2.54
Chewer	IOB_08	chr11	66392014	C	T	RBM14	NONSENSE	ENSG00000239306:[ENST00000310137(c.667C>T,Cga>Tga):ENSP00000311747(p.Arg223Ter)]	NA	NA	NA	NA	NA	NA	NA	NA	NA	NA	MODIFIER	NA	NA	243	243	0	169	158	11	6.51
Chewer	IOB_08	chr11	66392014	C	T	RBM14-RBM4	INTRONIC	ENSG00000248643:[ENST00000412278(c.337+7486C>T)]	NA	NA	NA	NA	NA	NA	NA	NA	NA	NA	MODIFIER	NA	NA	243	243	0	169	158	11	6.51
Chewer	IOB_08	chr11	66392014	C	T	RBM4	INTRONIC	ENSG00000173933:[ENST00000503028(c.-13+7486C>T)]	NA	NA	NA	NA	NA	NA	NA	NA	NA	NA	MODIFIER	NA	NA	243	243	0	169	158	11	6.51
Chewer	IOB_08	chr11	71634445	C	A	LOC100133315	EXONIC-NC	100133315:[NR_029192.1(n.332G>T)]	NA	NA	NA	NA	NA	NA	NA	NA	NA	NA	MODIFIER	NA	NA	175	175	0	155	146	9	5.81
Chewer	IOB_08	chr11	71634445	C	A	RP11-849H4.2	5UTR	ENSG00000254469:[ENST00000528184(c.-46G>T)]	NA	NA	NA	NA	NA	NA	NA	NA	NA	NA	MODIFIER	NA	NA	175	175	0	155	146	9	5.81
Chewer	IOB_08	chr11	72416919	C	A	ARAP1	SILENT	ENSG00000186635:[ENST00000359373(c.1740G>T,ctG>ctT):ENSP00000352332(p.Leu580(=))]	NA	NA	NA	NA	NA	NA	NA	NA	NA	NA	LOW	NA	NA	274	274	0	185	175	10	5.41
Chewer	IOB_08	chr11	78383262	C	A	TENM4	MISSENSE	ENSG00000149256:[ENST00000530738(c.1001G>T,cGg>cTg):ENSP00000431711(p.Arg334Leu)]	NA	NA	chr11*78383262*78383262*C*T*rs758862894*NA*NA*NA	NA	NA	NA	NA	T	PrD	NA	MODERATE	D	1	262	259	0	270	264	5	1.85
Chewer	IOB_08	chr11	104897123	G	A	CASP1	INTRONIC	ENSG00000137752:[ENST00000353247(c.169-40C>T)]	NA	NA	NA	NA	NA	NA	NA	NA	NA	NA	MODIFIER	NA	NA	130	129	0	129	116	13	10.08
Chewer	IOB_08	chr11	123676718	C	A	OR6M1	MISSENSE	ENSG00000196099:[ENST00000309154(c.340G>T,Gcg>Tcg):ENSP00000311038(p.Ala114Ser)]	NA	NA	NA	NA	NA	NA	NA	D	BN	NA	MODERATE	U	0	155	154	1	200	194	5	2.5
Chewer	IOB_08	chr12	11420132	G	T	PRB3	MISSENSE	5544:[NM_006249.4(c.925C>A,Cca>Aca):NP_006240.4(p.Pro309Thr)]	NA	NA	NA	NA	NA	NA	NA	NA	NA	NA	MODIFIER	NA	NA	46	46	0	62	55	6	9.68
Chewer	IOB_08	chr12	29450004	C	A	FAR2	MISSENSE	ENSG00000064763:[ENST00000182377(c.416C>A,gCt>gAt):ENSP00000182377(p.Ala139Asp)]	NA	NA	NA	NA	NA	NA	NA	D	PrD	NA	MODERATE	NA	NA	87	87	0	75	70	5	6.67
Chewer	IOB_08	chr12	29450004	C	A	RP11-996F15.2	NCT	ENSG00000257176:[ENST00000553105(n.470-3531G>T)]	NA	NA	NA	NA	NA	NA	NA	D	PrD	NA	MODERATE	NA	NA	87	87	0	75	70	5	6.67
Chewer	IOB_08	chr12	52939318	C	A	KRT71	INTRONIC	ENSG00000139648:[ENST00000267119(c.1360+31G>T)]	NA	NA	chr12*52939318*52939318*C*T*rs754170487*NA*NA*NA	NA	NA	NA	NA	NA	NA	NA	MODIFIER	NA	NA	74	74	0	71	65	5	7.04
Chewer	IOB_08	chr12	58131888	C	T	AGAP2	MISSENSE	ENSG00000135439:[ENST00000547588(c.142G>A,Gag>Aag):ENSP00000449241(p.Glu48Lys)]	NA	NA	NA	NA	NA	NA	NA	NA	NA	NA	MODIFIER	NA	NA	149	146	0	181	172	9	4.97
Chewer	IOB_08	chr12	58131888	C	T	TSPAN31	EXONIC-NC	ENSG00000135452:[ENST00000553221(n.93C>T)]	NA	NA	NA	NA	NA	NA	NA	NA	NA	NA	MODIFIER	NA	NA	149	146	0	181	172	9	4.97
Chewer	IOB_08	chr12	123804466	C	A	SBNO1	MISSENSE	ENSG00000139697:[ENST00000420886(c.2780G>T,cGa>cTa):ENSP00000387361(p.Arg927Leu)]	NA	chr12*123804466*123804466*COSM284796*C*T*1*large_intestine*1	NA	NA	NA	NA	NA	T	PoD	NA	MODERATE	D	1	289	288	0	230	218	9	3.91
Chewer	IOB_08	chr12	124796511	T	G	FAM101A	INTRONIC	144347:[XM_005253548.1(c.290+38T>G)]	NA	NA	chr12*124796511*124796511*T*C*rs143823493*1*0.9782*0.02177|chr12*124796511*124796511*T*TG*rs749185607*NA*NA*NA|chr12*124796511*124796512*TG*T*rs777737600*NA*NA*NA	NA	NA	NA	NA	NA	NA	NA	MODIFIER	NA	NA	33	29	2	34	27	5	14.71
Chewer	IOB_08	chr13	43543315	T	C	EPSTI1	INTRONIC-SS-ACR	ENSG00000133106:[ENST00000476830(n.347-2A>G)]	NA	NA	NA	8.42E-06	0.00E+00	NA	NA	NA	NA	NA	HIGH	NA	NA	108	108	0	117	110	6	5.13
Chewer	IOB_08	chr14	24506962	G	A	DHRS4L1	NCT	728635:[NR_102694.1(n.218-571G>A)]	NA	NA	NA	NA	NA	NA	NA	NA	NA	NA	MODIFIER	NA	NA	167	166	0	145	130	15	10.34
Chewer	IOB_08	chr14	24506962	G	A	RP11-468E2.9	NCT	ENSG00000225766:[ENST00000558293(n.177-571G>A)]	NA	NA	NA	NA	NA	NA	NA	NA	NA	NA	MODIFIER	NA	NA	167	166	0	145	130	15	10.34
Chewer	IOB_08	chr14	63174784	C	A	KCNH5	MISSENSE	ENSG00000140015:[ENST00000322893(c.2409G>T,atG>atT):ENSP00000321427(p.Met803Ile)]	NA	NA	NA	NA	NA	NA	NA	T_lc	BN	NA	MODERATE	D	1	235	235	0	228	221	6	2.63
Chewer	IOB_08	chr14	99866505	C	T	SETD3	SILENT	ENSG00000183576:[ENST00000331768(c.1269G>A,gaG>gaA):ENSP00000327436(p.Glu423(=))]	NA	NA	chr14*99866505*99866505*C*G*rs749942885*NA*NA*NA	NA	NA	NA	NA	NA	NA	NA	LOW	NA	0.985	278	276	0	261	255	5	1.92
Chewer	IOB_08	chr15	25420192	C	A	SNHG14	NCT	ENSG00000224078:[ENST00000441592(n.179+175C>A)]	NA	NA	chr15*25420192*25420192*C*T*rs747653287*NA*NA*NA	NA	NA	NA	NA	NA	NA	NA	MODIFIER	NA	NA	181	180	0	196	188	7	3.57
Chewer	IOB_08	chr15	26806276	C	T	GABRB3	MISSENSE	ENSG00000166206:[ENST00000541819(c.1051G>A,Gag>Aag):ENSP00000442408(p.Glu351Lys)]	NA	NA	NA	NA	NA	NA	NA	T	PoD	NA	MODERATE	D	1	137	137	0	167	152	13	7.78
Chewer	IOB_08	chr15	43928451	G	A	CATSPER2	NCT	117155:[NR_110319.1(n.845-34C>T)]	NA	NA	NA	NA	NA	NA	NA	NA	NA	NA	MODIFIER	NA	NA	106	105	0	78	72	6	7.69
Chewer	IOB_08	chr15	43928451	G	A	STRC	INTRONIC	ENSG00000242866:[ENST00000541030(c.-1103-17897C>T)]	NA	NA	NA	NA	NA	NA	NA	NA	NA	NA	MODIFIER	NA	NA	106	105	0	78	72	6	7.69
Chewer	IOB_08	chr15	45387752	C	T	DUOX2	NONSENSE	ENSG00000140279:[ENST00000389039(c.4122G>A,tgG>tgA):ENSP00000373691(p.Trp1374Ter)]	NA	NA	NA	NA	NA	NA	NA	NA	NA	NA	HIGH	NA	1	183	183	0	186	181	5	2.69
Chewer	IOB_08	chr15	50866902	C	T	TRPM7	SILENT	ENSG00000092439:[ENST00000561267(c.172G>A,caG>caA):ENSP00000454066(p.Gln58(=))]	NA	NA	NA	NA	NA	NA	NA	NA	NA	NA	LOW	NA	1	164	164	0	160	145	15	9.38
Chewer	IOB_08	chr15	75653726	C	A	MAN2C1	NCT-SS-PRX	ENSG00000140400:[ENST00000562228(n.436-4G>T)]	NA	NA	NA	NA	NA	NA	NA	NA	NA	NA	LOW	NA	NA	95	94	1	115	109	6	5.22
Chewer	IOB_08	chr15	75674081	C	A	SIN3A	INTRONIC	ENSG00000169375:[ENST00000360439(c.3196-35G>T)]	NA	NA	NA	NA	NA	NA	NA	NA	NA	NA	MODIFIER	NA	NA	104	103	1	94	89	5	5.32
Chewer	IOB_08	chr15	89392790	C	T	ACAN	SILENT	ENSG00000157766:[ENST00000352105(c.1854C>T,ggC>ggT):ENSP00000341615(p.Gly618(=))]	NA	NA	chr15*89392790*89392790*C*G*rs899483264*NA*NA*NA	NA	NA	NA	NA	NA	NA	NA	LOW	NA	NA	240	240	0	252	244	6	2.38
Chewer	IOB_08	chr16	2354046	C	A	ABCA3	MISSENSE	ENSG00000167972:[ENST00000301732(c.1391G>T,gGc>gTc):ENSP00000301732(p.Gly464Val)]	NA	NA	NA	NA	NA	NA	NA	NA	NA	NA	MODIFIER	NA	NA	132	132	0	79	73	5	6.33
Chewer	IOB_08	chr16	10867242	G	A	TVP23A	SILENT	ENSG00000166676:[ENST00000572102(c.57C>T,ggC>ggT):ENSP00000461067(p.Gly19(=))]	NA	NA	NA	NA	NA	NA	NA	NA	NA	NA	LOW	NA	NA	203	203	0	178	160	17	9.55
Chewer	IOB_08	chr16	21261782	C	A	ANKS4B	MISSENSE	ENSG00000175311:[ENST00000311620(c.895C>A,Ccc>Acc):ENSP00000308772(p.Pro299Thr)]	NA	NA	NA	NA	NA	NA	NA	NA	NA	NA	MODIFIER	NA	NA	93	93	0	83	77	6	7.23
Chewer	IOB_08	chr16	21261782	C	A	CRYM	INTRONIC	ENSG00000103316:[ENST00000570401(c.265-6393G>T)]	NA	NA	NA	NA	NA	NA	NA	NA	NA	NA	MODIFIER	NA	NA	93	93	0	83	77	6	7.23
Chewer	IOB_08	chr16	31073483	C	T	ZNF668	MISSENSE	ENSG00000167394:[ENST00000426488(c.835G>A,Gcc>Acc):ENSP00000403975(p.Ala279Thr)]	NA	NA	NA	NA	NA	NA	NA	T	BN	NA	MODERATE	N	0.836	164	164	0	131	126	5	3.82
Chewer	IOB_08	chr16	47703311	G	A	PHKB	MISSENSE	ENSG00000102893:[ENST00000566275(c.534G>A,atG>atA):ENSP00000459287(p.Met178Ile)]	NA	NA	NA	NA	NA	NA	NA	T	BN	NA	MODERATE	D	1	188	188	0	218	209	5	2.29
Chewer	IOB_08	chr17	6945271	G	A	SLC16A11	INTRONIC	162515:[XM_005256488.1(c.1114+44C>T)]	NA	NA	NA	NA	NA	NA	NA	NA	NA	NA	MODIFIER	NA	NA	156	155	0	133	118	15	11.28
Chewer	IOB_08	chr17	7578457	C	A	TP53	MISSENSE	ENSG00000141510:[ENST00000509690(c.77G>T,cGc>cTc):ENSP00000425104(p.Arg26Leu)]	NA	chr17*7578443*7578461*COSM46373*AGATGGCCATGGCGCGGAC*A*1*breast*1|chr17*7578443*7578464*COSM46001*BigSeq*A*2*ovary*2|chr17*7578445*7578458*COSM45887*ATGGCCATGGCGCG*A*1*large_intestine*1|chr17*7578445*7578465*COSM43786*BigSeq*A*1*liver*1|chr17*7578446*7578464*COSM45175*TGGCCATGGCGCGGACGCG*T*1*lung*1|chr17*7578446*7578467*COSM46354*BigSeq*T*1*lung*1|chr17*7578448*7578461*COSM46018*GCCATGGCGCGGAC*G*1*oesophagus*1|chr17*7578448*7578462*COSM675*GCCATGGCGCGGACG*G*2*breast*2|chr17*7578448*7578466*COSM85575*GCCATGGCGCGGACGCGGG*G*1*pancreas*1|chr17*7578449*7578461*COSM45266*CCATGGCGCGGAC*C*1*lung*1|chr17*7578452*7578458*COSM306853*TGGCGCG*TGG*2*central_nervous	chr17*7578457*7578457*C*G,T*rs587782144*NA*NA*NA	NA	NA	NA	NA	D	PrD	NA	MODERATE	NA	NA	127	127	0	104	91	13	12.5
Chewer	IOB_08	chr17	38451657	G	A	CDC6	MISSENSE	ENSG00000094804:[ENST00000209728(c.1133G>A,cGc>cAc):ENSP00000209728(p.Arg378His)]	NA	NA	5.99E-04	7.41E-05	0.00E+00	5.99E-04	0.00E+00	NA	NA	NA	MODIFIER	NA	NA	217	215	0	167	156	6	3.59
Chewer	IOB_08	chr17	41026195	G	A	AOC4P	EXONIC-NC	ENSG00000260105:[ENST00000566825(n.2098G>A)]	NA	NA	2.00E-04	NA	NA	2.00E-04	0.00E+00	NA	NA	NA	MODIFIER	NA	NA	125	125	0	76	70	6	7.89
Chewer	IOB_08	chr17	74010637	G	A	EVPL	MISSENSE	ENSG00000167880:[ENST00000586740(c.2309C>T,gCc>gTc):ENSP00000465630(p.Ala770Val)]	NA	NA	NA	NA	NA	NA	NA	NA	NA	NA	MODIFIER	NA	NA	81	81	0	105	99	6	5.71
Chewer	IOB_08	chr17	79655892	C	A	HGS	INTRONIC	9146:[XM_005257794.1(c.388+35C>A)]	NA	NA	NA	NA	NA	NA	NA	NA	NA	NA	MODIFIER	NA	NA	124	122	0	98	92	5	5.1
Chewer	IOB_08	chr18	908314	C	A	ADCYAP1	MISSENSE	ENSG00000141433:[ENST00000450565(c.291C>A,gaC>gaA):ENSP00000411658(p.Asp97Glu)]	NA	NA	NA	NA	NA	NA	NA	T	PoD	NA	MODERATE	N	1	142	142	0	152	146	5	3.29
Chewer	IOB_08	chr18	6215835	C	A	L3MBTL4	INTRONIC-SS-ACR	ENSG00000154655:[ENST00000535782(c.224-1G>T)]	NA	NA	NA	NA	NA	NA	NA	NA	NA	NA	HIGH	NA	NA	82	82	0	66	61	5	7.58
Chewer	IOB_08	chr18	29782973	G	T	GAREM	INTRONIC	ENSG00000141441:[ENST00000583696(c.66-66550C>A)]	NA	NA	NA	NA	NA	NA	NA	NA	NA	NA	MODIFIER	NA	NA	179	179	0	196	186	9	4.59
Chewer	IOB_08	chr18	29782973	G	T	MEP1B	MISSENSE-SS-PRX	ENSG00000141434:[ENST00000581184(c.272G>T,gGc>gTc):ENSP00000462038(p.Gly91Val)]	NA	NA	NA	NA	NA	NA	NA	NA	NA	NA	MODIFIER	NA	NA	179	179	0	196	186	9	4.59
Chewer	IOB_08	chr18	52265136	T	C	DYNAP	SILENT	ENSG00000178690:[ENST00000321600(c.393T>C,ctT>ctC):ENSP00000315265(p.Leu131(=))]	NA	NA	NA	NA	NA	NA	NA	NA	NA	NA	LOW	NA	NA	291	290	1	342	330	6	1.75
Chewer	IOB_08	chr19	12921085	C	A	HOOK2	NCT	ENSG00000095066:[ENST00000589765(n.41+14907G>T)]	NA	NA	NA	NA	NA	NA	NA	NA	NA	NA	MODIFIER	NA	NA	199	198	0	117	111	5	4.27
Chewer	IOB_08	chr19	12921085	C	A	RNASEH2A	INTRONIC	10535:[XM_005259710.1(c.418-46C>A)]	NA	NA	NA	NA	NA	NA	NA	NA	NA	NA	MODIFIER	NA	NA	199	198	0	117	111	5	4.27

Patel *et al.* , 2021. Whole-exome sequencing analysis of oral squamous cell carcinoma delineated by tobacco usage habits
Supplementary Table 4. List of somatic single nucleotide variants identified using whole exome sequencing of OSCC patients

Cohort	Sample	Chromosome	Genomic position	Reference allele	Alternate allele	Genesymbol	Variant type	Variant description	OncoMD	COSMIC	dbSNP	ExAC	ExAC_SAS	1000G	1000G_SAS	SIFT	Polyphen2	CONDEL	VEP	LRT	PhastCon7Way	Total reads in Normal	Reference read support in Normal	Alternative read support in Normal	Total reads in Tumor	Reference read support in Tumor	Alternate read support in Tumor	Alternate allele frequency in Tumor
Chewer	IOB_08	chr19	37853251	G	A	HKR1	MISSENSE	ENSG00000181666:[ENST00000589392(c.500G>A,gGg>gAg):ENSP00000467562(p.Gly167Glu)]	NA	NA	NA	NA	NA	NA	NA	NA	NA	NA	MODIFIER	NA	NA	109	109	0	103	95	7	6.8
Chewer	IOB_08	chr19	40325415	C	G	FBL	SILENT	ENSG00000105202:[ENST00000595545(c.579G>C,gtG>gtC):ENSP00000472419(p.Val193(=))]	NA	NA	NA	8.24E-06	0.00E+00	NA	NA	NA	NA	NA	LOW	NA	NA	146	146	0	151	140	11	7.28
Chewer	IOB_08	chr19	46996925	C	A	PNMAL2	MISSENSE	ENSG00000204851:[ENST00000599531(c.1798G>T,Ggg>Tgg):ENSP00000473036(p.Gly600Trp)]	NA	NA	chr19*46996925*46996925*C*T*rs182877132*1*0.9912*0.008786	NA	NA	NA	NA	NA	NA	NA	MODIFIER	NA	NA	67	67	0	54	47	5	9.26
Chewer	IOB_08	chr19	46996925	C	A	PPP5D1	INTRONIC	100506012:[NM_001205281.1(c.358-12228G>T)]	NA	NA	chr19*46996925*46996925*C*T*rs182877132*1*0.9912*0.008786	NA	NA	NA	NA	NA	NA	NA	MODIFIER	NA	NA	67	67	0	54	47	5	9.26
Chewer	IOB_08	chr19	50119428	G	T	PRR12	MISSENSE	ENSG00000126464:[ENST00000418929(c.5449G>T,Ggc>Tgc):ENSP00000394510(p.Gly1817Cys)]	NA	NA	NA	NA	NA	NA	NA	T	BN	NA	MODERATE	N	0.995	106	105	0	104	97	6	5.77
Chewer	IOB_08	chr19	50165295	C	A	IRF3	NONSENSE	ENSG00000126456:[ENST00000309877(c.892G>T,Gag>Tag):ENSP00000310127(p.Glu298Ter)]	NA	NA	NA	NA	NA	NA	NA	NA	NA	NA	HIGH	NA	NA	202	202	0	185	176	5	2.7
Chewer	IOB_08	chr19	52620200	C	A	ZNF616	MISSENSE	ENSG00000204611:[ENST00000600228(c.217G>T,Gca>Tca):ENSP00000471000(p.Ala73Ser)]	NA	NA	NA	NA	NA	NA	NA	NA	NA	NA	MODIFIER	NA	NA	148	147	0	117	112	5	4.27
Chewer	IOB_08	chr2	38200579	C	A	RMDN2	INTRONIC	ENSG00000115841:[ENST00000354545(c.453-604C>A)]	NA	NA	NA	NA	NA	NA	NA	NA	NA	NA	MODIFIER	NA	NA	130	130	0	183	176	7	3.83
Chewer	IOB_08	chr2	38200579	C	A	RMDN2-AS1	NCT	ENSG00000235848:[ENST00000414365(n.259-10281G>T)]	NA	NA	NA	NA	NA	NA	NA	NA	NA	NA	MODIFIER	NA	NA	130	130	0	183	176	7	3.83
Chewer	IOB_08	chr2	53943755	C	T	ASB3	MISSENSE	ENSG00000270898:[ENST00000406625(c.835G>A,Gag>Aag):ENSP00000385085(p.Glu279Lys)]	NA	NA	NA	NA	NA	NA	NA	NA	NA	NA	MODIFIER	NA	NA	142	141	0	148	138	9	6.08
Chewer	IOB_08	chr2	53943755	C	T	GPR75-ASB3	MISSENSE	ENSG00000115239:[ENST00000406053(c.707G>A,Gag>Aag):ENSP00000385137(p.Glu237Lys)]	NA	NA	NA	NA	NA	NA	NA	NA	NA	NA	MODIFIER	NA	NA	142	141	0	148	138	9	6.08
Chewer	IOB_08	chr2	79348696	G	A	REG1A	MISSENSE	ENSG00000115386:[ENST00000233735(c.73G>A,Gcc>Acc):ENSP00000233735(p.Ala25Thr)]	NA	NA	NA	NA	NA	NA	NA	NA	NA	NA	MODIFIER	NA	NA	288	288	0	375	354	21	5.6
Chewer	IOB_08	chr2	97357646	C	A	FER1L5	MISSENSE	90342:[NM_001293083.1(c.3160C>A,Cag>Aag):NP_001280012.1(p.Gln1054Lys)]	NA	NA	NA	NA	NA	NA	NA	T	BN	NA	MODERATE	NA	NA	186	185	0	180	162	16	8.89
Chewer	IOB_08	chr2	121747054	G	A	GLI2	SILENT	ENSG00000074047:[ENST00000361492(c.3564G>A,ccG>ccA):ENSP00000354586(p.Pro1188(=))]	NA	NA	.	8.68E-06	6.12E-05	NA	NA	NA	NA	NA	MODIFIER	NA	NA	96	96	0	149	143	5	3.36
Chewer	IOB_08	chr2	135926317	C	A	RAB3GAP1	MISSENSE	ENSG00000115839:-SS-PRX[ENST00000539493(c.2780C>A,gCa>gAa):ENSP00000444306(p.Ala927Glu)]	NA	NA	NA	NA	NA	NA	NA	D	PoD	NA	MODERATE	D	1	101	101	0	66	61	5	7.58
Chewer	IOB_08	chr2	135926317	C	A	ZRANB3	NCT	ENSG00000121988:[ENST00000412849(n.1782-3593G>T)]	NA	NA	NA	NA	NA	NA	NA	D	PoD	NA	MODERATE	D	1	101	101	0	66	61	5	7.58
Chewer	IOB_08	chr2	158397620	G	T	ACVR1C	SILENT	ENSG00000123612:[ENST00000335450(c.967C>A,Cgg>Agg):ENSP00000335178(p.Arg323(=))]	NA	NA	chr2*158397620*158397620*G*A*rs746678781*NA*NA*NA	NA	NA	NA	NA	NA	NA	NA	LOW	NA	1	380	379	0	377	358	19	5.04
Chewer	IOB_08	chr2	169417801	C	A	CERS6	MISSENSE	ENSG00000172292:[ENST00000305747(c.376C>A,Cca>Aca):ENSP00000306579(p.Pro126Thr)]	NA	NA	NA	NA	NA	NA	NA	D	PrD	NA	MODERATE	D	1	126	126	0	129	124	5	3.88
Chewer	IOB_08	chr2	170030639	C	T	LRP2	MISSENSE	ENSG00000081479:[ENST00000263816(c.10804G>A,Gcc>Acc):ENSP00000263816(p.Ala3602Thr)]	NA	chr2*170030639*170030639*COSM441450*C*T*1*breast*1	8.79E-03	2.98E-03	3.64E-04	8.79E-03	1.00E-03	T	PrD	NA	MODERATE	N	0.985	98	98	0	141	128	10	7.09
Chewer	IOB_08	chr2	179425620	A	T	TTN	SILENT	ENSG00000155657:[ENST00000342175(c.58620T>A,acT>acA):ENSP00000340554(p.Thr19540(=))]	NA	NA	NA	NA	NA	NA	NA	NA	NA	NA	LOW	NA	NA	231	231	0	274	266	7	2.55
Chewer	IOB_08	chr2	179425620	A	T	TTN-AS1	NCT	ENSG00000237298:[ENST00000591332(n.442+25065A>T)]	NA	NA	NA	NA	NA	NA	NA	NA	NA	NA	LOW	NA	NA	231	231	0	274	266	7	2.55
Chewer	IOB_08	chr2	198948709	C	A	PLCL1	SILENT	ENSG00000115896:[ENST00000487695(c.246C>A,gcC>gcA):ENSP00000457588(p.Ala82(=))]	NA	NA	NA	NA	NA	NA	NA	NA	NA	NA	LOW	NA	NA	398	396	1	410	395	15	3.66
Chewer	IOB_08	chr2	202139642	C	T	CASP8	MISSENSE	ENSG00000064012:[ENST00000358485(c.803C>T,tCg>tTg):ENSP00000351273(p.Ser268Leu)]	NA	chr2*202139642*202139642*COSM209549*C*T*1*large_intestine*1|chr2*202139642*202139642*COSM209548*C*T*1*large_intestine*1	NA	NA	NA	NA	NA	NA	NA	NA	MODIFIER	NA	NA	110	109	0	102	88	13	12.75
Chewer	IOB_08	chr2	218669135	C	T	TNS1	EXONIC-NC	ENSG00000079308:[ENST00000490566(n.1038G>A)]	NA	NA	NA	8.34E-06	0.00E+00	NA	NA	NA	NA	NA	MODIFIER	NA	NA	202	200	2	230	220	9	3.91
Chewer	IOB_08	chr2	220506365	C	A	SLC4A3	INTRONIC-SS-ACR-PRX	ENSG00000114923:[ENST00000425141(c.*2090-8C>A)]	NA	NA	NA	NA	NA	NA	NA	NA	NA	NA	LOW	NA	NA	158	158	0	159	152	6	3.77
Chewer	IOB_08	chr2	232575287	G	A	PTMA	MISSENSE	ENSG00000187514:[ENST00000412128(c.151G>A,cGg>cAg):ENSP00000406903(p.Arg51Gln)]	NA	NA	NA	NA	NA	NA	NA	NA	NA	NA	MODIFIER	NA	NA	79	79	0	55	49	6	10.91
Chewer	IOB_08	chr2	242438190	C	T	STK25	MISSENSE	ENSG00000115694:[ENST00000450497(c.499G>A,Gcc>Acc):ENSP00000399212(p.Ala167Thr)]	NA	NA	NA	NA	NA	NA	NA	NA	NA	NA	MODIFIER	NA	NA	353	353	0	309	299	7	2.27
Chewer	IOB_08	chr2	242509581	G	A	BOK	MISSENSE	ENSG00000176720:[ENST00000318407(c.391G>A,Gcg>Acg):ENSP00000314132(p.Ala131Thr)]	NA	NA	NA	1.02E-04	2.01E-04	NA	NA	D	PoD	NA	MODERATE	D	1	186	186	0	216	200	16	7.41
Chewer	IOB_08	chr2	242793383	C	A	PDCD1	NONSENSE	ENSG00000188389:[ENST00000334409(c.694G>T,Gag>Tag):ENSP00000335062(p.Glu232Ter)]	NA	NA	NA	NA	NA	NA	NA	NA	NA	NA	HIGH	NA	0.001	181	181	0	191	185	6	3.14
Chewer	IOB_08	chr20	23065702	G	A	CD93	SILENT	ENSG00000125810:[ENST00000246006(c.1128C>T,ggC>ggT):ENSP00000246006(p.Gly376(=))]	NA	chr20*23065702*23065702*COSM3378999*G*A*1*pancreas*1	NA	8.44E-06	6.55E-05	NA	NA	NA	NA	NA	LOW	NA	NA	253	253	0	300	274	23	7.67
Chewer	IOB_08	chr20	44592644	C	T	ZNF335	INTRONIC	63925:[XM_005260504.1(c.1100-15G>A)]	NA	NA	NA	NA	NA	NA	NA	NA	NA	NA	MODIFIER	NA	NA	163	163	0	173	164	5	2.89
Chewer	IOB_08	chr20	44756968	G	T	CD40	INTRONIC-SS-ACR	958:[NM_001322421.1(c.659-1G>T)]	NA	NA	chr20*44756968*44756968*G*A*rs78782129*NA*NA*NA	NA	NA	NA	NA	NA	NA	NA	HIGH	NA	NA	57	48	0	48	36	6	12.5
Chewer	IOB_08	chr21	9826206	G	A	MIR3687	EXONIC-NC	ENSG00000264063:[ENST00000577708(n.4G>A)]	NA	NA	NA	NA	NA	NA	NA	NA	NA	NA	MODIFIER	NA	NA	6895	6816	58	5229	5143	66	1.26
Chewer	IOB_08	chr21	11059897	A	G	BAGE2	INTRONIC	85319:[NM_182482.2(c.117-1574T>C)]	NA	NA	NA	NA	NA	NA	NA	NA	NA	NA	MODIFIER	NA	NA	93	91	0	74	69	5	6.76
Chewer	IOB_08	chr21	15561610	C	A	LIPI	SILENT	ENSG00000188992:[ENST00000536861(c.177G>T,ctG>ctT):ENSP00000440381(p.Leu59(=))]	NA	NA	NA	NA	NA	NA	NA	NA	NA	NA	LOW	NA	NA	99	99	0	78	73	5	6.41
Chewer	IOB_08	chr22	45826944	G	A	RIBC2	SILENT-SS-PRX	26150:[NM_015653.4(c.1065G>A,ttG>ttA):NP_056468.3(p.Leu355(=))]	NA	NA	NA	NA	NA	NA	NA	NA	NA	NA	LOW	NA	0.128	145	144	0	139	132	7	5.04
Chewer	IOB_08	chr22	50704675	C	T	MAPK11	MISSENSE	ENSG00000185386:[ENST00000330651(c.742G>A,Gca>Aca):ENSP00000333685(p.Ala248Thr)]	NA	NA	NA	NA	NA	NA	NA	T	BN	NA	MODERATE	NA	NA	134	134	0	86	81	5	5.81
Chewer	IOB_08	chr22	50946021	C	T	LMF2	SILENT	ENSG00000100258:[ENST00000380796(c.84G>A,acG>acA):ENSP00000370173(p.Thr28(=))]	NA	NA	NA	NA	NA	NA	NA	NA	NA	NA	LOW	NA	NA	143	143	0	122	116	5	4.1
Chewer	IOB_08	chr3	21478694	G	A	ZNF385D	SILENT-SS-PRX	ENSG00000151789:[ENST00000494118(n.860C>T)]	NA	NA	NA	6.67E-05	6.31E-05	NA	NA	NA	NA	NA	LOW	NA	0.742	154	153	1	127	122	5	3.94
Chewer	IOB_08	chr3	37368703	C	A	GOLGA4	MISSENSE	ENSG00000144674:[ENST00000437131(c.4939C>A,Cgc>Agc):ENSP00000405842(p.Arg1647Ser)]	NA	NA	chr3*37368703*37368703*C*T*rs756838815*NA*NA*NA	NA	NA	NA	NA	NA	NA	NA	MODERATE	NA	0.084	152	151	0	136	131	5	3.68
Chewer	IOB_08	chr3	45500310	C	A	LARS2	MISSENSE	ENSG00000011376:[ENST00000265537(c.682C>A,Cgt>Agt):ENSP00000265537(p.Arg228Ser)]	NA	NA	chr3*45500310*45500310*C*T*rs764747411*NA*NA*NA	NA	NA	NA	NA	D	PrD	NA	MODERATE	D	1	109	108	0	130	123	6	4.62
Chewer	IOB_08	chr3	48638427	C	A	UQCRC1	MISSENSE	ENSG00000010256:[ENST00000203407(c.947G>T,tGc>tTc):ENSP00000203407(p.Cys316Phe)]	NA	NA	NA	NA	NA	NA	NA	D	BN	NA	MODERATE	N	1	265	264	0	295	287	6	2.03
Chewer	IOB_08	chr3	121410122	C	A	GOLGB1	MISSENSE	ENSG00000173230:[ENST00000340645(c.8074G>T,Ggg>Tgg):ENSP00000341848(p.Gly2692Trp)]	NA	NA	NA	NA	NA	NA	NA	NA	NA	NA	MODERATE	NA	0.995	399	399	0	445	437	5	1.12
Chewer	IOB_08	chr3	121438475	C	A	GOLGB1	INTRONIC	2804:[NM_001256487.1(c.654+18G>T)]	NA	NA	chr3*121438475*121438475*C*T*rs72955399*1*0.9846*0.01538	NA	NA	NA	NA	NA	NA	NA	MODIFIER	NA	NA	171	171	0	236	229	5	2.12
Chewer	IOB_08	chr3	129304815	C	T	PLXND1	MISSENSE	ENSG00000004399:[ENST00000505237(c.487G>A,Gat>Aat):ENSP00000426241(p.Asp163Asn)]	NA	NA	NA	4.95E-05	6.06E-05	NA	NA	T	BN	NA	MODERATE	NA	NA	231	230	0	272	251	20	7.35
Chewer	IOB_08	chr3	134851573	C	T	EPHB1	MISSENSE	ENSG00000154928:[ENST00000398015(c.979C>T,Cgc>Tgc):ENSP00000381097(p.Arg327Cys)]	NA	chr3*134851573*134851573*COSM268387*C*T*1*large_intestine*1|chr3*134851573*134851573*COSM268386*C*T*1*large_intestine*1	NA	NA	NA	NA	NA	NA	NA	NA	MODIFIER	NA	NA	139	138	1	150	142	8	5.33
Chewer	IOB_08	chr3	145799597	C	A	PLOD2	MISSENSE	ENSG00000152952:[ENST00000461497(c.266G>T,tGg>tTg):ENSP00000419354(p.Trp89Leu)]	NA	NA	NA	NA	NA	NA	NA	D	PrD	NA	MODERATE	D	1	274	274	0	368	329	38	10.33
Chewer	IOB_08	chr3	145799597	C	A	RP11-274H2.2	NCT	ENSG00000243415:[ENST00000480247(n.337+13632C>A)]	NA	NA	NA	NA	NA	NA	NA	D	PrD	NA	MODERATE	D	1	274	274	0	368	329	38	10.33
Chewer	IOB_08	chr4	1941466	C	A	WHSC1	MISSENSE	ENSG00000109685:[ENST00000312087(c.1842C>A,agC>agA):ENSP00000308780(p.Ser614Arg)]	NA	NA	chr4*1941466*1941466*C*T*rs749350863*NA*NA*N	NA	NA	NA	NA	NA	NA	NA	MODIFIER	NA	NA	197	196	1	144	136	6	4.17
Chewer	IOB_08	chr4	79432499	C	A	FRAS1	SILENT	ENSG00000138759:[ENST00000264895(c.9852C>A,ggC>ggA):ENSP00000264895(p.Gly3284(=))]	NA	NA	NA	NA	NA	NA	NA	NA	NA	NA	LOW	NA	NA	82	82	0	98	93	5	5.1
Chewer	IOB_08	chr4	89652458	T	G	FAM13A	NCT	ENSG00000138640:[ENST00000509478(n.695+20A>C)]	NA	NA	NA	1.12E-02	1.26E-02	NA	NA	NA	NA	NA	MODIFIER	NA	NA	33	31	1	33	26	6	18.18
Chewer	IOB_08	chr4	126239754	G	T	FAT4	MISSENSE	ENSG00000196159:[ENST00000394329(c.2188G>T,Gct>Tct):ENSP00000377862(p.Ala730Ser)]	NA	NA	NA	NA	NA	NA	NA	T	PrD	NA	MODERATE	U	1	181	181	0	213	196	17	7.98
Chewer	IOB_08	chr5	14316870	C	T	TRIO	NCT	7204:[NR_134469.1(n.1755+18C>T)]	NA	NA	chr5*14316870*14316870*C*A*rs754017032*NA*NA*NA	NA	NA	NA	NA	NA	NA	NA	MODIFIER	NA	NA	128	128	0	149	144	5	3.36
Chewer	IOB_08	chr5	137854498	C	T	ETF1	MISSENSE	ENSG00000120705:[ENST00000360541(c.145G>A,Gca>Aca):ENSP00000353741(p.Ala49Thr)]	NA	NA	NA	NA	NA	NA	NA	T	BN	NA	MODERATE	NA	NA	297	297	0	240	234	5	2.08
Chewer	IOB_08	chr5	150501764	C	A	ANXA6	MISSENSE	ENSG00000197043:[ENST00000523714(c.1195G>T,Ggg>Tgg):ENSP00000430517(p.Gly399Trp)]	NA	NA	NA	NA	NA	NA	NA	D	PrD	NA	MODERATE	D	1	106	106	0	76	71	5	6.58
Chewer	IOB_08	chr6	44280902	G	T	AARS2	SILENT	ENSG00000124608:[ENST00000244571(c.159C>A,cgC>cgA):ENSP00000244571(p.Arg53(=))]	NA	NA	NA	NA	NA	NA	NA	NA	NA	NA	MODIFIER	NA	NA	100	100	0	117	112	5	4.27
Chewer	IOB_08	chr6	44280902	G	T	RP11-444E17.6	INTRONIC	ENSG00000272442:[ENST00000505802(c.*449+5523G>T)]	NA	NA	NA	NA	NA	NA	NA	NA	NA	NA	MODIFIER	NA	NA	100	100	0	117	112	5	4.27
Chewer	IOB_08	chr6	52656639	C	T	GSTA1	3UTR	ENSG00000243955:[ENST00000334575(c.*17G>A)]	NA	NA	chr6*52656639*52656639*C*G*rs73740641*1*0.9866*0.01338	NA	NA	NA	NA	NA	NA	NA	MODIFIER	NA	NA	310	309	0	351	341	8	2.28
Chewer	IOB_08	chr6	74079400	C	A	OOEP	MISSENSE	ENSG00000203907:[ENST00000370359(c.116G>T,tGg>tTg):ENSP00000359384(p.Trp39Leu)]	NA	NA	NA	NA	NA	NA	NA	NA	NA	NA	MODIFIER	NA	NA	144	144	0	148	141	7	4.73
Chewer	IOB_08	chr6	119238839	T	C	MCM9	MISSENSE	ENSG00000111877:[ENST00000316068(c.791A>G,aAa>aGa):ENSP00000312870(p.Lys264Arg)]	NA	NA	NA	NA	NA	NA	NA	NA	NA	NA	MODERATE	NA	NA	406	405	0	372	358	13	3.49
Chewer	IOB_08	chr6	131931283	C	A	MED23	MISSENSE	ENSG00000112282:[ENST00000545957(c.47G>T,aGc>aTc):ENSP00000439977(p.Ser16Ile)]	NA	NA	chr6*131931283*131931283*C*T*rs751579845*NA*NA*NA	NA	NA	NA	NA	T	PrD	NA	MODERATE	D	1	130	130	0	156	150	6	3.85
Chewer	IOB_08	chr6	144185702	C	T	ZC2HC1B	MISSENSE-SS-PRX	ENSG00000118491:[ENST00000237275(c.26C>T,gCa>gTa):ENSP00000237275(p.Ala9Val)]	NA	NA	NA	NA	NA	NA	NA	NA	NA	NA	LOW	NA	NA	224	224	0	187	178	7	3.74
Chewer	IOB_08	chr7	27135319	G	A	HOTAIRM1	NCT	ENSG00000233429:[ENST00000495032(n.26+28G>A)]	NA	NA	NA	3.88E-02	5.13E-02	NA	NA	NA	NA	NA	LOW	NA	1	203	197	2	218	207	8	3.67

Patel *et al.*, 2021. Whole-exome sequencing analysis of oral squamous cell carcinoma delineated by tobacco usage habits
Supplementary Table 4. List of somatic single nucleotide variants identified using whole exome sequencing of OSCC patients

Cohort	Sample	Chromosome	Genomic position	Reference allele	Alternate allele	Genesymbol	Variant type	Variant description	OncoMD	COSMIC	dbSNP	ExAC	ExAC_SAS	1000G	1000G_SAS	SIFT	Polyphen2	CONDEL	VEP	LRT	PhastCon7Way	Total reads in Normal	Reference read support in Normal	Alternative read support in Normal	Total reads in Tumor	Reference read support in Tumor	Alternate read support in Tumor	Alternate allele frequency in Tumor
Chewer	IOB_08	chr7	27135319	G	A	HOXA1	SILENT	ENSG00000105991:[ENST00000343060(c.213C>T,caC>caT):ENSP00000343246(p.His71(=))]	NA	NA	NA	3.88E-02	5.13E-02	NA	NA	NA	NA	NA	LOW	NA	1	203	197	2	218	207	8	3.67
Chewer	IOB_08	chr7	76958747	G	A	CCDC146	EXONIC-NC	ENSG00000135205:[ENST00000488998(n.459G>A)]	NA	NA	NA	NA	NA	NA	NA	NA	NA	NA	MODIFIER	NA	NA	207	205	0	185	174	10	5.41
Chewer	IOB_08	chr7	76958747	G	A	GSAP	NCT	54103:[XR_242247.1(n.1756-39C>T)]	NA	NA	NA	NA	NA	NA	NA	NA	NA	NA	MODIFIER	NA	NA	207	205	0	185	174	10	5.41
Chewer	IOB_08	chr7	86570728	C	T	KIAA1324L	INTRONIC	ENSG00000164659:[ENST00000444627(c.741+606G>A)]	NA	NA	NA	NA	NA	NA	NA	NA	NA	NA	MODIFIER	NA	NA	191	188	0	234	218	15	6.41
Chewer	IOB_08	chr7	107234476	T	G	BCAP29	NONSENSE	ENSG00000075790:[ENST00000436699(c.10T>G,taT>taG):ENSP00000414164(p.Tyr4Ter)]	NA	NA	NA	NA	NA	NA	NA	NA	NA	NA	MODIFIER	NA	NA	173	173	0	161	150	11	6.83
Chewer	IOB_08	chr7	130147182	G	T	COPG2	SILENT	ENSG00000158623:[ENST00000425248(c.269C>A,ggC>ggA):ENSP00000402346(p.Gly90(=))]	NA	NA	NA	NA	NA	NA	NA	NA	NA	NA	LOW	NA	NA	316	314	0	332	309	19	5.72
Chewer	IOB_08	chr7	138394491	C	T	ATP6V0A4	SILENT	ENSG00000105929:[ENST00000310018(c.2307G>A,acG>acA):ENSP00000308122(p.Thr769(=))]	NA	NA	1.76E-02	6.81E-03	2.38E-02	1.76E-02	2.56E-02	NA	NA	NA	LOW	NA	NA	66	66	0	56	51	5	8.93
Chewer	IOB_08	chr7	138833111	C	A	TTC26	SILENT	ENSG00000105948:[ENST00000430935(c.705C>A,ggC>ggA):ENSP00000410655(p.Gly235(=))]	NA	NA	NA	NA	NA	NA	NA	NA	NA	NA	LOW	NA	NA	287	287	0	325	317	5	1.54
Chewer	IOB_08	chr7	141719069	C	T	MGAM	MISSENSE	ENSG00000257335:[ENST00000465654(c.398C>T,tCc>tTc):ENSP00000419372(p.Ser133Phe)]	NA	chr7*141719069*141719069*COSM1548474*C*T*3*NS,lung*2,1|chr7*141719069*141719069*COSM1548476*C*T*3*NS,lung*2,1|chr7*141719069*141719069*COSM1548473*C*T*3*NS,lung*2,1|chr7*141719069*141719069*COSM1548475*C*T*3*NS,lung*2,1	NA	NA	NA	NA	NA	D	BN	NA	MODERATE	D	0.674	91	91	0	91	82	9	9.89
Chewer	IOB_08	chr7	142749769	T	C	OR6V1	MISSENSE	ENSG00000225781:[ENST00000418316(c.332T>C,cTc>cCc):ENSP00000396085(p.Leu111Pro)]	NA	NA	NA	NA	NA	NA	NA	D	PrD	NA	MODERATE	NA	NA	210	208	0	286	279	6	2.1
Chewer	IOB_08	chr7	146536934	C	T	CNTNAP2	MISSENSE	ENSG00000174469:[ENST00000361727(c.340C>T,Cgg>Tgg):ENSP00000354778(p.Arg114Trp)]	NA	chr7*146536934*146536934*COSM1329995*C*A*1*ovary*1|chr7*146536934*146536934*COSM205712*C*T*1*large_intestine*1	NA	3.30E-05	6.07E-05	NA	NA	T	PoD	NA	MODERATE	D	1	109	109	0	95	89	6	6.32
Chewer	IOB_08	chr7	150324891	C	A	GIMAP6	MISSENSE	ENSG00000133561:[ENST00000328902(c.795G>T,gaG>gaT):ENSP00000330374(p.Glu265Asp)]	NA	chr7*150324891*150324891*COSM746018*C*A*1*lung*1	NA	NA	NA	NA	NA	T	BN	NA	MODERATE	NA	0	203	202	0	217	206	10	4.61
Chewer	IOB_08	chr7	158109593	C	T	PTPRN2	SILENT	ENSG00000155093:[ENST00000404321(c.264G>A,ccG>ccA):ENSP00000385464(p.Pro88(=))]	NA	NA	NA	2.55E-05	6.26E-05	NA	NA	NA	NA	NA	LOW	NA	NA	172	172	0	168	162	6	3.57
Chewer	IOB_08	chr8	20112542	C	A	LZTS1	MISSENSE	ENSG00000061337:[ENST00000265801(c.151G>T,Ggt>Tgt):ENSP00000265801(p.Gly51Cys)]	NA	NA	chr8*20112542*20112542*C*T*rs367756245*NA*NA*NA	NA	NA	NA	NA	T	PoD	NA	MODERATE	N	0	200	200	0	196	186	9	4.59
Chewer	IOB_08	chr8	22442872	C	T	PDLIM2	MISSENSE	ENSG00000120913:[ENST00000308354(c.1250C>T,gCt>gTt):ENSP00000312634(p.Ala417Val)]	NA	NA	NA	NA	NA	NA	NA	NA	NA	NA	MODIFIER	NA	NA	220	220	0	202	192	9	4.46
Chewer	IOB_08	chr8	43046676	C	A	HGSNAT	MISSENSE	ENSG00000165102:[ENST00000522082(c.496C>A,Cct>Act):ENSP00000430151(p.Pro166Thr)]	NA	NA	NA	NA	NA	NA	NA	NA	NA	NA	MODIFIER	NA	NA	227	227	0	290	283	6	2.07
Chewer	IOB_08	chr8	82437324	C	T	FABP12	MISSENSE	ENSG00000197416:[ENST00000360464(c.380G>A,cGa>cAa):ENSP00000353650(p.Arg127Gln)]	NA	NA	NA	NA	NA	NA	NA	NA	NA	NA	MODIFIER	NA	NA	80	78	1	76	67	9	11.84
Chewer	IOB_08	chr8	82437324	C	T	RP11-257P3.3	NCT	ENSG00000253374:[ENST00000518637(n.184-754C>T)]	NA	NA	NA	NA	NA	NA	NA	NA	NA	NA	MODIFIER	NA	NA	80	78	1	76	67	9	11.84
Chewer	IOB_08	chr8	94146979	C	T	C8orf87	SILENT	ENSG00000254318:[ENST00000522116(c.123G>A,cgG>cgA):ENSP00000429724(p.Arg41(=))]	NA	NA	2.00E-04	NA	NA	2.00E-04	0.00E+00	NA	NA	NA	LOW	NA	NA	267	266	0	360	350	8	2.22
Chewer	IOB_08	chr8	110984838	C	T	KCNV1	MISSENSE	ENSG00000164794:[ENST00000297404(c.640G>A,Gtc>Atc):ENSP00000297404(p.Val214Ile)]	NA	chr8*110984838*110984838*COSM220162*C*T*1*haematopoietic_and_lymphoid_tissue*1	NA	8.24E-06	0.00E+00	NA	NA	T	BN	NA	MODERATE	N	0.994	136	135	0	151	145	5	3.31
Chewer	IOB_08	chr8	144550392	A	C	ZC3H3	MISSENSE	ENSG00000014164:[ENST00000262577(c.2162T>G,gTg>gGg):ENSP00000262577(p.Val721Gly)]	NA	NA	NA	NA	NA	NA	NA	D	PrD	NA	MODERATE	D	1	75	74	0	94	80	14	14.89
Chewer	IOB_08	chr8	145619553	G	A	CPSF1	NCT	ENSG00000071894:[ENST00000532935(n.236-38C>T)]	NA	NA	3.99E-04	4.38E-05	0.00E+00	3.99E-04	0.00E+00	NA	NA	NA	MODIFIER	NA	NA	92	91	0	83	75	8	9.64
Chewer	IOB_08	chr9	71081189	T	G	PGM5	INTRONIC	ENSG00000154330:[ENST00000396396(c.1159+1065T>G)]	NA	NA	NA	NA	NA	NA	NA	NA	NA	NA	MODIFIER	NA	NA	41	41	0	52	47	5	9.62
Chewer	IOB_08	chr9	102595641	T	C	NR4A3	MISSENSE	ENSG00000119508:[ENST00000330847(c.1192T>C,Tct>Cct):ENSP00000333122(p.Ser398Pro)]	NA	NA	NA	NA	NA	NA	NA	T	BN	NA	MODERATE	D	1	171	171	0	210	197	13	6.19
Chewer	IOB_08	chr9	125659663	C	A	RC3H2	MISSENSE	ENSG00000056586:[ENST00000335387(c.126G>T,ttG>ttT):ENSP00000335150(p.Leu42Phe)]	NA	NA	NA	NA	NA	NA	NA	NA	NA	NA	MODIFIER	NA	NA	258	258	0	226	219	6	2.65
Chewer	IOB_08	chr9	132084618	G	C	C9orf106	MISSENSE	414318:[NM_001012715.3(c.526G>C,Ggg>Cgg):NP_001012733.2(p.Gly176Arg)]	NA	NA	chr9*132084618*132084618*G*A*rs547992621*0*0.9998*0.0001997	NA	NA	NA	NA	NA	NA	NA	MODIFIER	NA	NA	269	267	1	242	232	7	2.89
Chewer	IOB_08	chrX	23397839	G	T	PTCHD1	MISSENSE	ENSG00000165186:[ENST00000379361(c.483G>T,aaG>aaT):ENSP00000368666(p.Lys161Asn)]	NA	NA	NA	NA	NA	NA	NA	NA	NA	NA	MODIFIER	NA	NA	209	208	0	298	280	18	6.04
Chewer	IOB_08	chrX	47486151	C	A	CFP	NCT	ENSG00000126759:[ENST00000485991(n.2237+21G>T)]	NA	NA	2.65E-04	9.50E-05	1.36E-03	2.65E-04	1.00E-03	NA	NA	NA	MODIFIER	NA	NA	153	153	0	187	180	6	3.21
Chewer	IOB_08	chrX	54482950	G	T	FGD1	MISSENSE	ENSG00000102302:[ENST00000375135(c.1687C>A,Cgc>Agc):ENSP00000364277(p.Arg563Ser)]	NA	chrX*54482950*54482950*COSM141640*G*A*1*upper_aerodigestive_tract*1	NA	NA	NA	NA	NA	D	BN	NA	MODERATE	D	0.994	130	130	0	128	116	12	9.38
Chewer	IOB_08	chrX	70764481	C	A	OGT	MISSENSE	ENSG00000147162:[ENST00000455587(c.406C>A,gCc>gAc):ENSP00000407659(p.Ala136Asp)]	NA	NA	NA	NA	NA	NA	NA	D	PrD	NA	MODERATE	D	1	144	143	1	118	112	5	4.24
Chewer	IOB_08	chrX	108619339	G	C	GUCY2F	MISSENSE	ENSG00000101890:[ENST00000218006(c.3208C>G,Ctt>Gtt):ENSP00000218006(p.Leu1070Val)]	NA	NA	NA	NA	NA	NA	NA	D	PoD	NA	MODERATE	N	0.965	84	84	0	96	91	5	5.21
Chewer	IOB_08	chrX	153073941	C	A	PDZD4	MISSENSE	ENSG00000067840:[ENST00000164640(c.170G>T,cGg>cTg):ENSP00000164640(p.Arg57Leu)]	NA	NA	chrX*153073941*153073941*C*T*rs146249799*0*0.9997*0.0002649	NA	NA	NA	NA	T	PoD	NA	MODERATE	NA	NA	253	252	0	217	207	9	4.15
Chewer	IOB_08	chrX	154091333	G	T	F8	INTRONIC	ENSG00000185010:[ENST00000360256(c.6574+25C>A)]	NA	NA	NA	NA	NA	NA	NA	NA	NA	NA	MODIFIER	NA	NA	145	145	0	155	147	6	3.87
Chewer	IOB_08	chrX	154128171	C	G	F8	MISSENSE	ENSG00000185010:[ENST00000360256(c.6243G>C,tgG>tgC):ENSP00000353393(p.Trp2081Cys)]	NA	NA	NA	NA	NA	NA	NA	D	PrD	NA	MODERATE	D	1	139	139	0	136	127	8	5.88
Chewer	IOB_09	chr1	16042725	C	T	PLEKHM2	NCT-SS-PRX	ENSG00000116786:[ENST00000462455(n.79-7C>T)]	NA	NA	NA	1.26E-02	1.40E-02	NA	NA	NA	NA	NA	LOW	NA	NA	43	42	0	37	32	5	13.51
Chewer	IOB_09	chr1	21137256	C	T	EIF4G3	SILENT	ENSG00000075151:[ENST00000374937(c.4569G>A,tcG>tcA):ENSP00000364073(p.Ser1523(=))]	NA	NA	NA	1.65E-05	0.00E+00	NA	NA	NA	NA	NA	LOW	NA	NA	123	122	0	135	117	18	13.33
Chewer	IOB_09	chr1	29631867	A	G	PTPRU	MISSENSE	ENSG00000060656:[ENST00000345512(c.2777A>G,gAt>gGt):ENSP00000334941(p.Asp926Gly)]	NA	NA	NA	NA	NA	NA	NA	D	PrD	NA	MODERATE	D	1	61	61	0	73	68	5	6.85
Chewer	IOB_09	chr1	40769544	G	A	COL9A2	NCT	ENSG00000049089:[ENST00000482722(n.1672-33C>T)]	NA	NA	NA	NA	NA	NA	NA	NA	NA	NA	MODIFIER	NA	NA	54	54	0	62	49	13	20.97
Chewer	IOB_09	chr1	53332255	G	C	ZYG11A	INTRONIC	ENSG00000203995:[ENST00000371532(c.365+16G>C)]	NA	NA	NA	NA	NA	NA	NA	NA	NA	NA	MODIFIER	NA	NA	224	223	0	230	189	40	17.39
Chewer	IOB_09	chr1	91182564	A	C	BARHL2	SILENT	ENSG00000143032:[ENST00000370445(c.189T>G,tcT>tcG):ENSP00000359474(p.Ser63(=))]	NA	chr1*91182564*91182564*COSM1245960*A*C*1*oesophagus*1	NA	NA	NA	NA	NA	NA	NA	NA	LOW	NA	NA	68	68	0	114	86	28	24.56
Chewer	IOB_09	chr1	150477064	G	A	TARS2	INTRONIC	ENSG00000143374:[ENST00000467982(c.*866-44G>A)]	NA	NA	NA	NA	NA	NA	NA	NA	NA	NA	MODIFIER	NA	NA	97	97	0	91	69	22	24.18
Chewer	IOB_09	chr1	202702730	G	A	KDM5B	SILENT	ENSG00000117139:[ENST00000235790(c.3234C>T,ctC>ctT):ENSP00000235790(p.Leu1078(=))]	NA	NA	NA	2.47E-05	0.00E+00	NA	NA	NA	NA	NA	MODIFIER	NA	NA	86	86	0	72	62	9	12.5
Chewer	IOB_09	chr1	208390716	G	A	PLXNA2	SILENT	ENSG00000076356:[ENST00000367033(c.552C>T,atC>atT):ENSP00000356000(p.Ile184(=))]	NA	NA	NA	9.06E-05	0.00E+00	NA	NA	NA	NA	NA	LOW	NA	NA	171	169	0	166	137	28	16.87
Chewer	IOB_09	chr1	222802645	A	C	MIA3	MISSENSE	ENSG00000154305:[ENST00000354906(c.830A>C,Aag>Cag):ENSP00000355062(p.Lys278Gln)]	NA	NA	NA	NA	NA	NA	NA	NA	NA	NA	MODIFIER	NA	NA	102	102	0	137	118	19	13.87
Chewer	IOB_09	chr1	225742585	C	T	ENAH	INTRONIC	ENSG00000154380:[ENST00000284563(c.349+23G>A)]	NA	NA	NA	8.62E-06	0.00E+00	NA	NA	NA	NA	NA	MODIFIER	NA	NA	100	100	0	112	97	15	13.39
Chewer	IOB_09	chr10	46122355	C	T	ZFAND4	MISSENSE	ENSG00000172671:[ENST00000344646(c.916G>A,Gag>Aag):ENSP00000339484(p.Glu306Lys)]	NA	NA	NA	NA	NA	NA	NA	T	BN	NA	MODERATE	N	0.997	180	180	0	166	136	29	17.47
Chewer	IOB_09	chr10	58117910	C	G	ZWINT	INTRONIC-SS-ACR	ENSG00000122952:EXONIC-NC[ENST00000460654(n.497G>C)]	NA	NA	NA	NA	NA	NA	NA	NA	NA	NA	HIGH	NA	NA	57	57	0	79	58	21	26.58
Chewer	IOB_09	chr10	69926336	A	G	MYPN	MISSENSE	ENSG00000138347:[ENST00000358913(c.1886A>G,cAg>cGg):ENSP00000351790(p.Gln629Arg)]	NA	NA	NA	NA	NA	NA	NA	T	BN	NA	MODERATE	N	0.001	64	64	0	71	57	12	16.9
Chewer	IOB_09	chr10	75331208	C	A	USP54	NONSENSE	ENSG00000166348:[ENST00000319786(c.211G>T,Gga>Tga):ENSP00000326547(p.Gly71Ter)]	NA	NA	NA	NA	NA	NA	NA	NA	NA	NA	MODIFIER	NA	NA	143	143	0	149	143	5	3.36
Chewer	IOB_09	chr11	6651179	A	G	DCHS1	INTRONIC	ENSG00000166341:[ENST00000299441(c.4796-37T>C)]	NA	NA	NA	NA	NA	NA	NA	NA	NA	NA	MODIFIER	NA	NA	336	332	1	364	353	11	3.02
Chewer	IOB_09	chr11	7022476	G	T	ZNF214	SILENT	ENSG00000149050:[ENST00000278314(c.438C>A,acC>acA):ENSP00000278314(p.Thr146(=))]	NA	NA	NA	NA	NA	NA	NA	NA	NA	NA	LOW	NA	NA	195	193	0	234	190	42	17.95
Chewer	IOB_09	chr11	26586935	G	A	ANO3	INTRONIC	63982:[NM_001313726.1(c.1630+5609G>A)]	NA	NA	NA	4.13E-05	6.07E-05	NA	NA	NA	NA	NA	LOW	NA	NA	327	325	0	403	388	12	2.98
Chewer	IOB_09	chr11	26586935	G	A	MUC15	SILENT	ENSG00000169550:[ENST00000455601(c.471C>T,acC>acT):ENSP00000397339(p.Thr157(=))]	NA	NA	NA	4.13E-05	6.07E-05	NA	NA	NA	NA	NA	LOW	NA	NA	327	325	0	403	388	12	2.98
Chewer	IOB_09	chr11	45235098	C	A	LOC101060179	MISSENSE	101060179:[XM_003959932.2(c.463C>A,Ctt>Att):XP_003959981.1(p.Leu155Ile)]	NA	NA	NA	NA	NA	NA	NA	NA	NA	NA	MODIFIER	NA	NA	69	69	0	111	105	6	5.41
Chewer	IOB_09	chr11	45235098	C	A	PRDM11	INTRONIC	ENSG00000019485:[ENST00000424263(c.555-6023C>A)]	NA	NA	NA	NA	NA	NA	NA	NA	NA	NA	MODIFIER	NA	NA	69	69	0	111	105	6	5.41
Chewer	IOB_09	chr11	47356571	G	A	MYBPC3	INTRONIC	ENSG00000134571:[ENST00000399249(c.2905+22C>T)]	NA	NA	NA	NA	NA	NA	NA	NA	NA	NA	MODIFIER	NA	NA	60	60	0	70	57	12	17.14
Chewer	IOB_09	chr11	49426718	C	A	TYRL	EXONIC-NC	ENSG00000123447:[ENST00000531826(n.159G>T)]	NA	NA	NA	NA	NA	NA	NA	NA	NA	NA	MODIFIER	NA	NA	66	66	0	73	61	11	15.07
Chewer	IOB_09	chr11	57564195	C	A	CTNND1	SILENT	ENSG00000198561:[ENST00000534647(c.453C>A,ggC>ggA):ENSP00000434202(p.Gly151(=))]	NA	NA	NA	NA	NA	NA	NA	NA	NA	NA	LOW	NA	NA	141	141	0	136	131	5	3.68
Chewer	IOB_09	chr11	57564195	C	A	TMX2-CTNND1	EXONIC-NC	100528016:[NR_037646.1(n.1246C>A)]	NA	NA	NA	NA	NA	NA	NA	NA	NA	NA	LOW	NA	NA	141	141	0	136	131	5	3.68
Chewer	IOB_09	chr11	60482526	G	A	MS4A8	MISSENSE	ENSG00000166959:[ENST00000529752(c.514G>A,Ggt>Agt):ENSP00000436857(p.Gly172Ser)]	NA	NA	chr11*60482526*60482526*G*T*rs773157361*NA*NA*NA	NA	NA	NA	NA	NA	NA	NA	LOW	NA	NA	71	71	0	91	76	14	15.38
Chewer	IOB_09	chr11	64032756	G	C	PLCB3	INTRONIC	ENSG00000149782:[ENST00000279230(c.2843-26G>C)]	NA	NA	chr11*64032756*64032756*G*A,C*rs776870992*NA*NA*NA	1.99E-04	3.72E-04	NA	NA	NA	NA	NA	MODIFIER	NA	NA	70	66	2	74	66	7	9.46
Chewer	IOB_09	chr11	65810612	G	T	GAL3ST3	MISSENSE	ENSG00000175229:[ENST00000312006(c.662C>A,gCc>gAc):ENSP00000308591(p.Ala221Asp)]	NA	chr11*65810612*65810612*COSM1356132*G*T*3*large_intestine*3	1.32E-02	1.61E-02	8.02E-03	1.32E-02	8.20E-03	T	BN	NA	MODERATE	N	0.618	370	369	0	362	309	52	14.36
Chewer	IOB_09	chr11	65810612	G	T	LOC101927968	INTRONIC	101927968:[XM_005274422.1(c.-137-8379G>T)]	NA	chr11*65810612*65810612*COSM1356132*G*T*3*large_intestine*3	1.32E-02	1.61E-02	8.02E-03	1.32E-02	8.20E-03	T	BN	NA	MODERATE	N	0.618	370	369	0	362	309	52	14.36
Chewer	IOB_09	chr11	76751296	G	A	B3GNT6	MISSENSE	ENSG00000198488:[ENST00000421061(c.434G>A,cGc>cAc):ENSP00000403463(p.Arg145His)]	NA	NA	NA	4.12E-05	0.00E+00	NA	NA	T	BN	NA	MODERATE	N	0	355	354	0	332	256	76	22.89

Patel *et al.* , 2021. Whole-exome sequencing analysis of oral squamous cell carcinoma delineated by tobacco usage habits
Supplementary Table 4. List of somatic single nucleotide variants identified using whole exome sequencing of OSCC patients

Cohort	Sample	Chromosome	Genomic position	Reference allele	Alternate allele	Genesymbol	Variant type	Variant description	OncoMD	COSMIC	dbSNP	ExAC	ExAC_SAS	1000G	1000G_SAS	SIFT	Polyphen2	CONDEL	VEP	LRT	PhastCon7Way	Total reads in Normal	Reference read support in Normal	Alternative read support in Normal	Total reads in Tumor	Reference read support in Tumor	Alternate read support in Tumor	Alternate allele frequency in Tumor
Chewer	IOB_09	chr11	108142097	C	A	ATM	MISSENSE	ENSG00000149311:[ENST00000278616(c.3041C>A,gCt>gAt):ENSP00000278616(p.Ala1014Asp)]	NA	NA	NA	NA	NA	NA	NA	NA	NA	NA	MODERATE	NA	0.984	168	168	0	178	171	5	2.81
Chewer	IOB_09	chr11	124743242	A	G	ROBO3	MISSENSE	ENSG00000154134:[ENST00000538940(c.1507A>G,Agt>Ggt):ENSP00000441797(p.Ser503Gly)]	NA	NA	NA	NA	NA	NA	NA	D	BN	NA	MODERATE	N	1	51	51	0	51	40	10	19.61
Chewer	IOB_09	chr12	20799557	G	T	PDE3A	INTRONIC	ENSG00000172572:[ENST00000359062(c.2365+20G>T)]	NA	NA	NA	NA	NA	NA	NA	NA	NA	NA	MODIFIER	NA	NA	211	210	0	248	240	5	2.02
Chewer	IOB_09	chr12	51034612	C	A	DIP2B	MISSENSE	ENSG00000066084:[ENST00000301180(c.278C>A,gCc>gAc):ENSP00000301180(p.Ala93Asp)]	NA	NA	NA	NA	NA	NA	NA	T	PoD	NA	MODERATE	D	1	60	60	0	68	63	5	7.35
Chewer	IOB_09	chr12	57572322	G	A	LRP1	SILENT	ENSG00000123384:[ENST00000243077(c.4542G>A,gtG>gtA):ENSP00000243077(p.Val1514(=))]	NA	NA	NA	NA	NA	NA	NA	NA	NA	NA	LOW	NA	NA	220	219	0	196	154	41	20.92
Chewer	IOB_09	chr12	113386789	T	C	OAS3	MISSENSE	ENSG00000111331:[ENST00000228928(c.1153T>C,Tca>Cca):ENSP00000228928(p.Ser385Pro)]	NA	NA	NA	NA	NA	NA	NA	T_lc	BN	NA	MODERATE	NA	NA	164	164	0	168	161	5	2.98
Chewer	IOB_09	chr12	113386789	T	C	RP1-71H24.1	NCT	ENSG00000257452:[ENST00000552784(n.354-40306A>G)]	NA	NA	NA	NA	NA	NA	NA	T_lc	BN	NA	MODERATE	NA	NA	164	164	0	168	161	5	2.98
Chewer	IOB_09	chr12	118839782	C	A	SUDS3	INTRONIC-SS-ACR-PRX	ENSG00000111707:[ENST00000543473(c.676-6C>A)]	NA	NA	NA	NA	NA	NA	NA	NA	NA	NA	LOW	NA	NA	230	229	0	274	267	6	2.19
Chewer	IOB_09	chr12	119866413	G	T	CCDC60	NCT	ENSG00000183273:[ENST00000535685(n.57-75G>T)]	NA	NA	NA	NA	NA	NA	NA	NA	NA	NA	MODIFIER	NA	NA	88	88	0	105	88	17	16.19
Chewer	IOB_09	chr12	119866413	G	T	RP11-768F21.1	NCT	ENSG00000248636:[ENST00000509470(n.419-40341C>A)]	NA	NA	NA	NA	NA	NA	NA	NA	NA	NA	MODIFIER	NA	NA	88	88	0	105	88	17	16.19
Chewer	IOB_09	chr13	42390935	C	A	VWA8	INTRONIC	ENSG00000102763:[ENST00000281496(c.1870-24G>T)]	NA	NA	NA	NA	NA	NA	NA	NA	NA	NA	MODIFIER	NA	NA	110	109	0	126	120	5	3.97
Chewer	IOB_09	chr13	60590195	C	A	DIAPH3	INTRONIC-SS-DNR-PRX	ENSG00000139734:[ENST00000377908(c.668+10G>T)]	NA	NA	NA	NA	NA	NA	NA	NA	NA	NA	MODIFIER	NA	NA	63	63	0	79	74	5	6.33
Chewer	IOB_09	chr13	60590195	C	A	DIAPH3-AS1	NCT	100874195:[XR_245372.1(n.103+2690C>A)]	NA	NA	NA	NA	NA	NA	NA	NA	NA	NA	MODIFIER	NA	NA	63	63	0	79	74	5	6.33
Chewer	IOB_09	chr13	72014833	G	A	DACH1	INTRONIC-SS-ACR-PRX	ENSG00000165659:[ENST00000359684(c.2246-9C>T)]	NA	NA	NA	NA	NA	NA	NA	NA	NA	NA	MODIFIER	NA	NA	45	45	0	62	49	13	20.97
Chewer	IOB_09	chr13	73636642	C	G	KLF5	MISSENSE	ENSG00000102554:[ENST00000539231(c.632C>G,cCg>cGg):ENSP00000440407(p.Pro211Arg)]	NA	NA	NA	NA	NA	NA	NA	D	PrD	NA	MODERATE	D	1	90	90	0	120	95	24	20
Chewer	IOB_09	chr13	113086673	C	T	SPACA7	INTRONIC	122258:[XM_005268296.1(c.299-85C>T)]	NA	NA	6.19E-03	NA	NA	6.19E-03	5.10E-03	NA	NA	NA	MODIFIER	NA	NA	80	80	0	93	77	15	16.13
Chewer	IOB_09	chr14	22475820	A	T	TRAV19	5UTR	ENSG00000211799:[ENST00000390447(c.-48A>T)]	NA	NA	NA	NA	NA	NA	NA	NA	NA	NA	MODIFIER	NA	NA	33	33	0	51	46	5	9.8
Chewer	IOB_09	chr14	70924450	C	T	ADAM21	SILENT	ENSG00000139985:[ENST00000267499(c.234C>T,gtC>gtT):ENSP00000267499(p.Val78(=))]	NA	NA	NA	3.77E-01	4.07E-01	NA	NA	NA	NA	NA	MODIFIER	NA	NA	27	27	0	35	30	5	14.29
Chewer	IOB_09	chr14	70924450	C	T	RP11-486O13.4	NCT	ENSG00000257759:[ENST00000556646(n.184-4224G>A)]	NA	NA	NA	3.77E-01	4.07E-01	NA	NA	NA	NA	NA	MODIFIER	NA	NA	27	27	0	35	30	5	14.29
Chewer	IOB_09	chr14	93649810	T	A	MOAP1	NONSENSE	ENSG00000165943:[ENST00000298894(c.778A>T,Aag>Tag):ENSP00000298894(p.Lys260Ter)]	NA	NA	NA	NA	NA	NA	NA	NA	NA	NA	HIGH	NA	NA	248	247	0	187	162	24	12.83
Chewer	IOB_09	chr14	103804753	G	C	EIF5	MISSENSE	ENSG00000100664:[ENST00000216554(c.529G>C,Gag>Cag):ENSP00000216554(p.Glu177Gln)]	NA	NA	NA	NA	NA	NA	NA	T	BN	NA	MODERATE	N	1	73	72	0	52	45	7	13.46
Chewer	IOB_09	chr15	38239925	C	A	TMCO5A	INTRONIC	ENSG00000166069:[ENST00000560653(c.*108+28C>A)]	NA	NA	2.00E-04	8.40E-06	6.10E-05	2.00E-04	1.00E-03	NA	NA	NA	MODIFIER	NA	NA	35	35	0	33	27	6	18.18
Chewer	IOB_09	chr15	40915735	T	A	CASC5	MISSENSE	ENSG00000137812:[ENST00000526913(c.406T>A,agT>agA):ENSP00000432565(p.Ser136Arg)]	NA	NA	NA	NA	NA	NA	NA	D	BN	NA	MODERATE	N	0	68	67	1	69	54	14	20.29
Chewer	IOB_09	chr15	41022205	G	A	RAD51	NCT	ENSG00000051180:[ENST00000530766(n.129+373G>A)]	NA	NA	2.00E-04	8.27E-06	6.09E-05	2.00E-04	0.00E+00	NA	NA	NA	MODIFIER	NA	NA	69	69	0	80	62	18	22.5
Chewer	IOB_09	chr15	89392892	G	C	ACAN	SILENT	ENSG00000157766:[ENST00000352105(c.1956G>C,acG>acC):ENSP00000341615(p.Thr652(=))]	NA	NA	chr15*89392892*89392892*G*A*rs777892820*NA*NA*NA	NA	NA	NA	NA	NA	NA	NA	LOW	NA	NA	295	294	0	281	233	47	16.73
Chewer	IOB_09	chr15	90213374	G	A	PLIN1	SILENT	ENSG00000166819:[ENST00000300055(c.435C>T,gcC>gcT):ENSP00000300055(p.Ala145(=))]	NA	NA	NA	1.18E-04	0.00E+00	NA	NA	NA	NA	NA	LOW	NA	NA	108	106	0	104	98	5	4.81
Chewer	IOB_09	chr16	839967	C	T	CHTF18	INTRONIC	63922:[XM_005255470.1(c.259-210C>T)]	NA	NA	9.78E-03	2.01E-03	6.44E-04	9.78E-03	0.00E+00	NA	NA	NA	MODIFIER	NA	NA	181	179	0	155	123	32	20.65
Chewer	IOB_09	chr16	1396440	C	T	BAIAP3	INTRONIC-SS-ACR-PRX	ENSG00000007516:[ENST00000562208(c.2425-3C>T)]	NA	NA	NA	NA	NA	NA	NA	NA	NA	NA	LOW	NA	NA	205	204	0	215	169	46	21.4
Chewer	IOB_09	chr16	28328848	G	A	SBK1	MISSENSE	ENSG00000188322:[ENST00000341901(c.136G>A,Gcc>Acc):ENSP00000343248(p.Ala46Thr)]	NA	NA	2.00E-04	9.90E-05	6.06E-05	2.00E-04	0.00E+00	NA	NA	NA	MODERATE	NA	1	188	187	0	167	160	6	3.59
Chewer	IOB_09	chr16	31073394	G	A	ZNF668	SILENT	ENSG00000167394:[ENST00000426488(c.924C>T,tgC>tgT):ENSP00000403975(p.Cys308(=))]	NA	chr16*31073394*31073394*COSM1271686*G*A*1*oesophagus*1|chr16*31073394*31073394*COSM1271687*G*A*1*oesophagus*1	NA	NA	NA	NA	NA	NA	NA	NA	LOW	NA	0.085	237	237	0	182	154	26	14.29
Chewer	IOB_09	chr16	49669766	G	A	ZNF423	SILENT	ENSG00000102935:[ENST00000262383(c.3297C>T,agC>agT):ENSP00000262383(p.Ser1099(=))]	NA	NA	2.00E-04	4.70E-05	1.59E-04	2.00E-04	1.00E-03	NA	NA	NA	LOW	NA	0.995	155	155	0	199	185	14	7.04
Chewer	IOB_09	chr16	67572487	G	C	CTD-2012K14.2	EXONIC-NC	ENSG00000261396:[ENST00000567122(n.503C>G)]	NA	NA	NA	NA	NA	NA	NA	NA	NA	NA	MODIFIER	NA	NA	101	101	0	98	80	17	17.35
Chewer	IOB_09	chr16	67572487	G	C	FAM65A	INTRONIC	ENSG00000039523:[ENST00000042381(c.104+34G>C)]	NA	NA	NA	NA	NA	NA	NA	NA	NA	NA	MODIFIER	NA	NA	101	101	0	98	80	17	17.35
Chewer	IOB_09	chr16	70564652	C	T	SF3B3	SILENT	ENSG00000189091:[ENST00000302516(c.402C>T,gcC>gcT):ENSP00000305790(p.Ala134(=))]	NA	NA	NA	NA	NA	NA	NA	NA	NA	NA	LOW	NA	NA	167	167	0	142	112	30	21.13
Chewer	IOB_09	chr16	89347729	C	T	ANKRD11	MISSENSE	ENSG00000167522:[ENST00000301030(c.5221G>A,Gac>Aac):ENSP00000301030(p.Asp1741Asn)]	NA	NA	NA	4.17E-05	1.22E-04	NA	NA	NA	NA	NA	MODIFIER	NA	NA	190	190	0	141	108	33	23.4
Chewer	IOB_09	chr17	4684107	C	T	TM4SF5	SILENT	ENSG00000142484:[ENST00000270560(c.201C>T,gcC>gcT):ENSP00000270560(p.Ala67(=))]	NA	chr17*4684107*4684107*COSM178969*C*T*1*large_intestine*1	NA	5.04E-05	3.67E-04	NA	NA	NA	NA	NA	LOW	NA	NA	43	42	0	45	36	8	17.78
Chewer	IOB_09	chr17	10363427	G	A	CTC-297N7.11	NCT	ENSG00000272975:[ENST00000587182(n.155+53872G>A)]	NA	NA	NA	NA	NA	NA	NA	NA	NA	NA	MODIFIER	NA	NA	236	236	0	229	190	36	15.72
Chewer	IOB_09	chr17	10363427	G	A	MYH4	INTRONIC-SS-ACR-PRX	ENSG00000264424:[ENST00000255381(c.1267-9C>T)]	NA	NA	NA	NA	NA	NA	NA	NA	NA	NA	MODIFIER	NA	NA	236	236	0	229	190	36	15.72
Chewer	IOB_09	chr17	10363427	G	A	MYHAS	NCT	100128560:[NR_125367.1(n.167+53872G>A)]	NA	NA	NA	NA	NA	NA	NA	NA	NA	NA	MODIFIER	NA	NA	236	236	0	229	190	36	15.72
Chewer	IOB_09	chr17	10363427	G	A	RP11-799N11.1	NCT	ENSG00000272736:[ENST00000581304(n.143+53872G>A)]	NA	NA	NA	NA	NA	NA	NA	NA	NA	NA	MODIFIER	NA	NA	236	236	0	229	190	36	15.72
Chewer	IOB_09	chr17	18219285	C	A	SMCR8	MISSENSE	ENSG00000176994:[ENST00000406438(c.182C>A,tCc>tAc):ENSP00000385025(p.Ser61Tyr)]	NA	NA	NA	NA	NA	NA	NA	D	PrD	NA	MODERATE	D	0.999	124	124	0	169	163	5	2.96
Chewer	IOB_09	chr17	21318982	G	A	KCNJ12	MISSENSE	ENSG00000184185:[ENST00000331718(c.328G>A,Ggt>Agt):ENSP00000328150(p.Gly110Ser)]	NA	NA	NA	9.90E-05	6.06E-05	NA	NA	D	PrD	NA	MODERATE	D	1	395	387	0	462	428	23	4.98
Chewer	IOB_09	chr17	33326431	G	T	LIG3	MISSENSE	ENSG00000005156:[ENST00000262327(c.2219G>T,cGc>cTc):ENSP00000262327(p.Arg740Leu)]	NA	NA	2.00E-04	8.34E-06	0.00E+00	2.00E-04	0.00E+00	NA	NA	NA	MODERATE	NA	1	122	122	0	162	122	38	23.46
Chewer	IOB_09	chr17	33767545	C	A	SLFN13	3UTR	ENSG00000154760:[ENST00000530782(c.*1732G>T)]	NA	NA	NA	NA	NA	NA	NA	NA	NA	NA	MODIFIER	NA	NA	203	203	0	223	213	8	3.59
Chewer	IOB_09	chr17	40343049	C	T	GHDC	MISSENSE	ENSG00000167925:[ENST00000428494(c.952G>A,Gcc>Acc):ENSP00000410945(p.Ala318Thr)]	NA	NA	NA	8.91E-06	0.00E+00	NA	NA	NA	NA	NA	MODIFIER	NA	NA	130	130	0	92	82	9	9.78
Chewer	IOB_09	chr17	40722088	C	G	MLX	MISSENSE	ENSG00000108788:[ENST00000246912(c.727C>G,Caa>Gaa):ENSP00000246912(p.Gln243Glu)]	NA	NA	chr17*40722088*40722088*C*A,G*rs761350836*NA*NA*NA	8.24E-06	0.00E+00	NA	NA	D	PoD	NA	MODERATE	NA	NA	96	95	0	102	84	18	17.65
Chewer	IOB_09	chr17	40725027	C	G	MLX	3UTR	ENSG00000108788:[ENST00000246912(c.*1406C>G)]	NA	NA	NA	NA	NA	NA	NA	NA	NA	NA	MODIFIER	NA	NA	59	58	0	49	39	10	20.41
Chewer	IOB_09	chr17	40725027	C	G	PSMC3IP	MISSENSE	ENSG00000131470:[ENST00000253789(c.577G>C,Gag>Cag):ENSP00000253789(p.Glu193Gln)]	NA	NA	NA	NA	NA	NA	NA	NA	NA	NA	MODIFIER	NA	NA	59	58	0	49	39	10	20.41
Chewer	IOB_09	chr17	40725240	C	T	MLX	3UTR	ENSG00000108788:[ENST00000246912(c.*1619C>T)]	NA	NA	NA	NA	NA	NA	NA	NA	NA	NA	MODIFIER	NA	NA	155	154	0	166	137	29	17.47
Chewer	IOB_09	chr17	40725240	C	T	PSMC3IP	NCT	29893:[NR_045669.1(n.963-56G>A)]	NA	NA	NA	NA	NA	NA	NA	NA	NA	NA	MODIFIER	NA	NA	155	154	0	166	137	29	17.47
Chewer	IOB_09	chr17	40725375	C	G	PSMC3IP	MISSENSE	ENSG00000131470:[ENST00000393795(c.491G>C,aGa>aCa):ENSP00000377384(p.Arg164Thr)]	NA	NA	NA	8.24E-06	0.00E+00	NA	NA	NA	NA	NA	MODIFIER	NA	NA	126	126	0	117	99	17	14.53
Chewer	IOB_09	chr17	41607598	G	A	DHX8	INTRONIC	1659:[NM_001322219.1(c.3444-6182G>A)]	NA	NA	NA	NA	NA	NA	NA	NA	NA	NA	MODIFIER	NA	NA	117	117	0	142	118	23	16.2
Chewer	IOB_09	chr17	41607598	G	A	ETV4	5UTR	ENSG00000175832:[ENST00000586826(c.-69C>T)]	NA	NA	NA	NA	NA	NA	NA	NA	NA	NA	MODIFIER	NA	NA	117	117	0	142	118	23	16.2
Chewer	IOB_09	chr17	56657107	A	C	TEX14	INTRONIC	56155:[XM_005257518.1(c.3301-15T>G)]	NA	NA	chr17*56657104*56657109*CAAAAG*C*rs753992797*NA*NA*NA	NA	NA	NA	NA	NA	NA	NA	MODIFIER	NA	NA	74	74	0	64	52	12	18.75
Chewer	IOB_09	chr17	59024556	T	C	BCAS3	INTRONIC	ENSG00000141376:[ENST00000585744(c.401-24T>C)]	NA	NA	NA	NA	NA	NA	NA	NA	NA	NA	MODIFIER	NA	NA	170	169	0	185	150	34	18.38
Chewer	IOB_09	chr17	71427630	T	G	SDK2	NCT	ENSG00000069188:[ENST00000479356(n.511+11A>C)]	NA	NA	NA	NA	NA	NA	NA	NA	NA	NA	MODIFIER	NA	NA	99	98	0	95	79	16	16.84
Chewer	IOB_09	chr17	78247059	C	A	RNF213	SILENT	ENSG00000173821:[ENST00000319921(c.117C>A,tcC>tcA):ENSP00000324392(p.Ser39(=))]	NA	NA	NA	NA	NA	NA	NA	NA	NA	NA	MODIFIER	NA	NA	79	79	0	52	46	5	9.62
Chewer	IOB_09	chr18	2890927	G	A	EMILIN2	MISSENSE	ENSG00000132205:[ENST00000254528(c.802G>A,Gaa>Aaa):ENSP00000254528(p.Glu268Lys)]	NA	NA	NA	NA	NA	NA	NA	NA	NA	NA	MODERATE	NA	0.998	80	79	0	92	73	18	19.57
Chewer	IOB_09	chr18	6215806	C	G	L3MBTL4	MISSENSE	ENSG00000154655:[ENST00000284898(c.813G>C,tgG>tgC):ENSP00000284898(p.Trp271Cys)]	NA	NA	NA	NA	NA	NA	NA	D	PrD	NA	MODERATE	D	1	80	78	0	75	66	9	12
Chewer	IOB_09	chr18	34233282	G	T	FHOD3	INTRONIC	80206:[XM_005258349.1(c.1646+390G>T)]	NA	NA	chr18*34233282*34233282*G*A*rs1040006122*NA*NA*NA	NA	NA	NA	NA	NA	NA	NA	MODIFIER	NA	NA	263	261	0	306	235	70	22.88
Chewer	IOB_09	chr18	65178220	T	G	CTD-2541J13.2	NCT	ENSG00000263424:[ENST00000581951(n.308-67T>G)]	NA	NA	2.00E-04	8.95E-06	0.00E+00	2.00E-04	0.00E+00	NA	NA	NA	MODIFIER	NA	NA	41	41	0	47	35	12	25.53
Chewer	IOB_09	chr18	65178220	T	G	DSEL	MISSENSE	ENSG00000171451:[ENST00000310045(c.3656A>C,aAg>aCg):ENSP00000310565(p.Lys1219Thr)]	NA	NA	2.00E-04	8.95E-06	0.00E+00	2.00E-04	0.00E+00	NA	NA	NA	MODIFIER	NA	NA	41	41	0	47	35	12	25.53
Chewer	IOB_09	chr19	757838	C	T	MISP	MISSENSE	ENSG00000099812:[ENST00000215582(c.892C>T,Cgt>Tgt):ENSP00000215582(p.Arg298Cys)]	NA	NA	chr19*757838*757838*C*G*rs1027483791*NA*NA*NA	NA	NA	NA	NA	NA	NA	NA	MODIFIER	NA	NA	135	134	0	96	81	13	13.54
Chewer	IOB_09	chr19	1388997	G	C	NDUFS7	INTRONIC	374291:[XM_005259556.1(c.483+60G>C)]	NA	NA	chr19*1388997*1388999*GCA*G*rs779830951*NA*NA*NA	NA	NA	NA	NA	NA	NA	NA	MODIFIER	NA	NA	147	147	0	141	134	6	4.26
Chewer	IOB_09	chr19	3982073	A	G	EEF2	INTRONIC	ENSG00000167658:[ENST00000309311(c.792-23T>C)]	NA	NA	NA	NA	NA	NA	NA	NA	NA	NA	MODIFIER	NA	NA	45	45	0	40	33	7	17.5
Chewer	IOB_09	chr19	5207936	T	C	PTPRS	SILENT	ENSG00000105426:[ENST00000262963(c.5715A>G,acA>acG):ENSP00000262963(p.Thr1905(=))]	NA	NA	NA	NA	NA	NA	NA	NA	NA	NA	LOW	NA	NA	80	79	0	58	43	13	22.41
Chewer	IOB_09	chr19	10081986	G	A	COL5A3	INTRONIC	ENSG00000080573:[ENST00000264828(c.3775-52C>T)]	NA	NA	NA	NA	NA	NA	NA	NA	NA	NA	MODIFIER	NA	NA	87	87	0	103	78	25	24.27
Chewer	IOB_09	chr19	11447990	C	A	RAB3D	MISSENSE	ENSG00000105514:[ENST00000222120(c.86G>T,gGc>gTc):ENSP00000222120(p.Gly29Val)]	NA	NA	NA	NA	NA	NA	NA	D	PrD	NA	MODERATE	D	1	199	199	0	170	154	15	8.82
Chewer	IOB_09	chr19	15234038	C	T	ILVBL	MISSENSE	ENSG00000105135:[ENST00000533148(c.152G>A,cGg>cAg):ENSP00000434734(p.Arg51Gln)]	NA	NA	NA	NA	NA	NA	NA	NA	NA	NA	MODIFIER	NA	NA	255	255	0	219	184	35	15.98
Chewer	IOB_09	chr19	17420585	G	A	ABHD8	INTRONIC	ENSG00000127220:[ENST00000593489(c.40+391C>T)]	NA	NA	NA	NA	NA	NA	NA	NA	NA	NA	MODIFIER	NA	NA	145	141	0	106	93	5	4.72
Chewer	IOB_09	chr19	17420585	G	A	DDA1	INTRONIC	ENSG00000130311:[ENST00000359866(c.3+119G>A)]	NA	NA	NA	NA	NA	NA	NA	NA	NA	NA	MODIFIER	NA	NA	145	141	0	106	93	5	4.72
Chewer	IOB_09	chr19	36228979	C	T	KMT2B	NONSENSE	ENSG00000272333:[ENST00000222270(c.7759C>T,Cga>Tga):ENSP00000222270(p.Arg2587Ter)]	NA	chr19*36228979*36228979*COSM566190*C*T*2*lung,endometrium*1,1	NA	NA	NA	NA	NA	NA	NA	NA	MODIFIER	NA	NA	146	145	0	125	99	25	20
Chewer	IOB_09	chr19	40222145	C	T	CLC	MISSENSE-SS-PRX	ENSG00000105205:[ENST00000221804(c.304G>A,Gta>Ata):ENSP00000221804(p.Val102Ile)]	NA	NA	chr19*40222145*40222145*C*A*rs750982874*NA*NA*NA	NA	NA	NA	NA	T	BN	NA	MODERATE	NA	NA	148	146	0	141	116	23	16.31
Chewer	IOB_09	chr19	42814703	G	A	PRR19	SILENT	ENSG00000188368:[ENST00000341747(c.882G>A,acG>acA):ENSP00000342709(p.Thr294(=))]	NA	NA	NA	NA	NA	NA	NA	NA	NA	NA	LOW	NA	NA	211	211	0	179	137	41	22.91
Chewer	IOB_09	chr19	44223542	C	T	IRGC	NONSENSE	ENSG00000124449:[ENST00000244314(c.832C>T,Cag>Tag):ENSP00000244314(p.Gln278Ter)]	NA	NA	NA	NA	NA	NA	NA	NA	NA	NA	HIGH	NA	0.907	152	150	0	147	121	25	17.01
Chewer	IOB_09	chr19	47763526	G	A	CCDC9	INTRONIC	ENSG00000105321:[ENST00000221922(c.109-52G>A)]	NA	NA	3.99E-04	NA	NA	3.99E-04	0.00E+00	NA	NA	NA	MODIFIER	NA	NA	81	81	0	78	64	14	17.95
Chewer	IOB_09	chr19	48613788	T	G	PLA2G4C	5UTR	ENSG00000105499:[ENST00000596352(c.-120A>C)]	NA	NA	NA	6.38E-04	3.12E-04	NA	NA	NA	NA	NA	MODIFIER	NA	NA	28	26	2	22	16	6	27.27
Chewer	IOB_09	chr19	55824405	C	T	TMEM150B	MISSENSE	ENSG00000180061:[ENST00000326652(c.524G>A,tGc>tAc):ENSP00000320757(p.Cys175Tyr)]	NA	NA	NA	NA	NA	NA	NA	NA	NA	NA	MODIFIER	NA	NA	71	69	0	85	79	6	7.06

Patel *et al.* , 2021. Whole-exome sequencing analysis of oral squamous cell carcinoma delineated by tobacco usage habits
Supplementary Table 4. List of somatic single nucleotide variants identified using whole exome sequencing of OSCC patients

Cohort	Sample	Chromosome	Genomic position	Reference allele	Alternate allele	Genesymbol	Variant type	Variant description	OncoMD	COSMIC	dbSNP	ExAC	ExAC_SAS	1000G	1000G_SAS	SIFT	Polyphen2	CONDEL	VEP	LRT	PhastCon7Way	Total reads in Normal	Reference read support in Normal	Alternative read support in Normal	Total reads in Tumor	Reference read support in Tumor	Alternate read support in Tumor	Alternate allele frequency in Tumor
Chewer	IOB_09	chr19	56127225	C	A	ZNF865	MISSENSE	ENSG00000261221:[ENST00000568956(c.2241C>A,agC>agA):ENSP00000457715(p.Ser747Arg)]	NA	NA	NA	NA	NA	NA	NA	T	UN	NA	MODERATE	NA	NA	139	139	0	145	140	5	3.45
Chewer	IOB_09	chr19	58117912	G	A	ZNF530	MISSENSE	ENSG00000183647:[ENST00000332854(c.1019G>A,cGt>cAt):ENSP00000332861(p.Arg340His)]	NA	NA	NA	9.88E-05	3.03E-04	NA	NA	NA	NA	NA	MODIFIER	NA	NA	170	164	1	160	124	35	21.88
Chewer	IOB_09	chr2	1652991	T	G	PXDN	MISSENSE	ENSG00000130508:[ENST00000252804(c.2561A>C,gAc>gCc):ENSP00000252804(p.Asp854Ala)]	NA	NA	chr2*1652991*1652991*T*TCGTTGCTGCACA*rs772449441*NA*NA*NA	NA	NA	NA	NA	NA	NA	NA	MODIFIER	NA	NA	200	191	8	213	193	13	6.1
Chewer	IOB_09	chr2	11959735	G	C	LPIN1	INTRONIC	ENSG00000134324:[ENST00000404113(c.912+11G>C)]	NA	NA	chr2*11959735*11959735*G*T*rs1048583670*NA*NA*NA	NA	NA	NA	NA	NA	NA	NA	MODIFIER	NA	NA	242	242	0	270	214	54	20
Chewer	IOB_09	chr2	11959789	G	A	LPIN1	NCT	ENSG00000134324:[ENST00000475922(n.3446+65G>A)]	NA	NA	NA	NA	NA	NA	NA	NA	NA	NA	MODIFIER	NA	NA	175	174	0	202	158	43	21.29
Chewer	IOB_09	chr2	64120473	A	G	VPS54	EXONIC-NC	51542:[XR_244939.1(n.3601T>C)]	NA	NA	NA	NA	NA	NA	NA	NA	NA	NA	MODIFIER	NA	NA	184	184	0	193	186	6	3.11
Chewer	IOB_09	chr2	74686866	C	A	INO80B	3UTR	ENSG00000115274:[ENST00000441673(c.*50C>A)]	NA	NA	NA	NA	NA	NA	NA	D	PrD	NA	MODERATE	NA	NA	54	53	0	67	62	5	7.46
Chewer	IOB_09	chr2	74686866	C	A	INO80B-WBP1	EXONIC-NC	100532735:[NR_037849.1(n.1258C>A)]	NA	NA	NA	NA	NA	NA	NA	D	PrD	NA	MODERATE	NA	NA	54	53	0	67	62	5	7.46
Chewer	IOB_09	chr2	74686866	C	A	WBP1	MISSENSE	ENSG00000239779:[ENST00000233615(c.166C>A,Ctc>Atc):ENSP00000233615(p.Leu56Ile)]	NA	NA	NA	NA	NA	NA	NA	D	PrD	NA	MODERATE	NA	NA	54	53	0	67	62	5	7.46
Chewer	IOB_09	chr2	77746846	T	A	LRRTM4	MISSENSE	ENSG00000176204:[ENST00000409088(c.149A>T,cAt>cTt):ENSP00000386236(p.His50Leu)]	NA	NA	NA	NA	NA	NA	NA	T	BN	NA	MODERATE	D	0.991	207	207	0	257	206	49	19.07
Chewer	IOB_09	chr2	89160799	T	A	AC096579.13	NCT	ENSG00000240040:[ENST00000452230(n.39+238A>T)]	NA	NA	NA	2.16E-04	6.76E-05	NA	NA	NA	NA	NA	MODIFIER	NA	NA	227	226	0	195	188	6	3.08
Chewer	IOB_09	chr2	89160799	T	A	AC096579.7	NCT	ENSG00000231486:[ENST00000418209(n.38+599A>T)]	NA	NA	NA	2.16E-04	6.76E-05	NA	NA	NA	NA	NA	MODIFIER	NA	NA	227	226	0	195	188	6	3.08
Chewer	IOB_09	chr2	89160799	T	A	NA	NCT	101928457:[XR_245045.1(n.26+35T>A)]	NA	NA	NA	2.16E-04	6.76E-05	NA	NA	NA	NA	NA	MODIFIER	NA	NA	227	226	0	195	188	6	3.08
Chewer	IOB_09	chr2	120858270	C	T	EPB41L5	NCT-SS-PRX	57669:[NR_138472.1(n.1413-5C>T)]	NA	NA	NA	NA	NA	NA	NA	NA	NA	NA	LOW	NA	NA	52	52	0	54	45	9	16.67
Chewer	IOB_09	chr2	128396904	C	A	LIMS2	SILENT	ENSG00000072163:[ENST00000409455(c.963G>T,ctG>ctT):ENSP00000386383(p.Leu321(=))]	NA	NA	NA	NA	NA	NA	NA	NA	NA	NA	MODIFIER	NA	NA	111	111	0	102	86	15	14.71
Chewer	IOB_09	chr2	139537865	G	A	NXPH2	5UTR	ENSG00000144227:[ENST00000272641(c.-54C>T)]	NA	NA	NA	NA	NA	NA	NA	NA	NA	NA	MODIFIER	NA	NA	80	80	0	110	105	5	4.55
Chewer	IOB_09	chr2	160746884	G	A	LY75	SILENT	ENSG00000054219:[ENST00000263636(c.642C>T,aaC>aaT):ENSP00000263636(p.Asn214(=))]	NA	NA	NA	5.78E-05	0.00E+00	NA	NA	NA	NA	NA	LOW	NA	0.994	58	58	0	91	76	14	15.38
Chewer	IOB_09	chr2	160746884	G	A	LY75-CD302	SILENT	ENSG00000248672:[ENST00000504764(c.642C>T,aaC>aaT):ENSP00000423463(p.Asn214(=))]	NA	NA	NA	5.78E-05	0.00E+00	NA	NA	NA	NA	NA	LOW	NA	0.994	58	58	0	91	76	14	15.38
Chewer	IOB_09	chr2	162224309	C	T	PSMD14	SILENT	ENSG00000115233:[ENST00000409682(c.135C>T,ggC>ggT):ENSP00000386541(p.Gly45(=))]	NA	NA	NA	8.56E-06	0.00E+00	NA	NA	NA	NA	NA	LOW	NA	NA	222	221	1	253	245	7	2.77
Chewer	IOB_09	chr2	228886642	C	T	SPHKAP	MISSENSE	ENSG00000153820:[ENST00000344657(c.482G>A,aGa>aAa):ENSP00000339886(p.Arg161Lys)]	NA	NA	NA	8.38E-06	0.00E+00	NA	NA	NA	NA	NA	MODERATE	NA	1	82	82	0	79	66	13	16.46
Chewer	IOB_09	chr2	241969818	C	T	AC005237.4	NCT	ENSG00000225521:[ENST00000458377(n.140+3100G>A)]	NA	NA	NA	NA	NA	NA	NA	NA	NA	NA	MODIFIER	NA	NA	274	273	1	236	180	53	22.46
Chewer	IOB_09	chr2	241969818	C	T	SNED1	MISSENSE	ENSG00000162804:[ENST00000310397(c.331C>T,Cgg>Tgg):ENSP00000308893(p.Arg111Trp)]	NA	NA	NA	NA	NA	NA	NA	NA	NA	NA	MODIFIER	NA	NA	274	273	1	236	180	53	22.46
Chewer	IOB_09	chr20	45354497	G	A	SLC2A10	SILENT	ENSG00000197496:[ENST00000359271(c.822G>A,gtG>gtA):ENSP00000352216(p.Val274(=))]	NA	NA	NA	NA	NA	NA	NA	NA	NA	NA	LOW	NA	NA	129	127	0	144	123	21	14.58
Chewer	IOB_09	chr20	47628505	C	T	ARFGEF2	NONSENSE	ENSG00000124198:[ENST00000371917(c.3802C>T,Cag>Tag):ENSP00000360985(p.Gln1268Ter)]	NA	NA	NA	NA	NA	NA	NA	NA	NA	NA	HIGH	NA	1	324	324	0	289	230	57	19.72
Chewer	IOB_09	chr20	61915259	C	A	ARFGAP1	INTRONIC	ENSG00000101199:[ENST00000353546(c.747+27C>A)]	NA	NA	NA	NA	NA	NA	NA	NA	NA	NA	MODIFIER	NA	NA	133	133	0	168	161	5	2.98
Chewer	IOB_09	chr21	11059897	A	G	BAGE2	INTRONIC	85319:[NM_182482.2(c.117-1574T>C)]	NA	NA	NA	NA	NA	NA	NA	NA	NA	NA	MODIFIER	NA	NA	50	50	0	64	58	6	9.38
Chewer	IOB_09	chr22	22127164	C	T	MAPK1	MISSENSE-SS-PRX	ENSG00000100030:[ENST00000544786(c.832G>A,Gag>Aag):ENSP00000440842(p.Glu278Lys)]	NA	chr22*22127164*22127164*COSM461148*C*T*2*oesophagus,cervix*1,1	NA	NA	NA	NA	NA	D	PrD	NA	MODERATE	D	1	47	47	0	54	45	9	16.67
Chewer	IOB_09	chr22	23481175	C	T	RTDR1	INTRONIC	27156:[XM_005261569.1(c.332-46G>A)]	NA	NA	NA	NA	NA	NA	NA	NA	NA	NA	MODIFIER	NA	NA	145	143	0	162	155	5	3.09
Chewer	IOB_09	chr22	28194495	A	C	MN1	SILENT	ENSG00000169184:[ENST00000302326(c.2037T>G,ccT>ccG):ENSP00000304956(p.Pro679(=))]	NA	NA	NA	NA	NA	NA	NA	NA	NA	NA	LOW	NA	NA	98	98	0	108	101	5	4.63
Chewer	IOB_09	chr22	36597811	C	T	APOL4	NCT	ENSG00000100336:[ENST00000479929(n.180-2389G>A)]	NA	NA	3.59E-03	8.90E-04	2.93E-04	3.59E-03	1.00E-03	NA	NA	NA	MODIFIER	NA	NA	88	88	0	72	62	9	12.5
Chewer	IOB_09	chr22	50436571	G	A	IL17REL	MISSENSE	ENSG00000188263:[ENST00000341280(c.769C>T,Cgc>Tgc):ENSP00000342520(p.Arg257Cys)]	NA	NA	5.99E-04	6.88E-04	0.00E+00	5.99E-04	0.00E+00	D	PoD	NA	MODERATE	U	0.004	120	120	0	134	110	23	17.16
Chewer	IOB_09	chr3	10138021	C	A	FANCD2	MISSENSE	ENSG00000144554:[ENST00000287647(c.4050C>A,gaC>gaA):ENSP00000287647(p.Asp1350Glu)]	NA	NA	NA	NA	NA	NA	NA	NA	NA	NA	MODIFIER	NA	NA	85	83	0	94	88	5	5.32
Chewer	IOB_09	chr3	10138021	C	A	FANCD2OS	NCT	ENSG00000163705:[ENST00000431315(n.71-1447G>T)]	NA	NA	NA	NA	NA	NA	NA	NA	NA	NA	MODIFIER	NA	NA	85	83	0	94	88	5	5.32
Chewer	IOB_09	chr3	13670430	C	A	FBLN2	SILENT	ENSG00000163520:[ENST00000295760(c.2454C>A,tcC>tcA):ENSP00000295760(p.Ser818(=))]	NA	NA	chr3*13670430*13670430*C*G,T*rs374811722*1*0.998*.,0.001997	NA	NA	NA	NA	NA	NA	NA	LOW	NA	NA	135	135	0	159	144	15	9.43
Chewer	IOB_09	chr3	33905590	C	A	PDCD6IP	MISSENSE	ENSG00000170248:[ENST00000307296(c.2213C>A,aCt>aAt):ENSP00000307387(p.Thr738Asn)]	NA	NA	NA	NA	NA	NA	NA	T	BN	NA	MODERATE	N	0.016	131	131	0	153	146	5	3.27
Chewer	IOB_09	chr3	36779025	G	A	DCLK3	MISSENSE	ENSG00000163673:[ENST00000416516(c.1126C>T,Cgc>Tgc):ENSP00000394484(p.Arg376Cys)]	NA	NA	NA	8.28E-05	0.00E+00	NA	NA	T	BN	NA	MODERATE	N	1	146	146	0	159	133	26	16.35
Chewer	IOB_09	chr3	49830482	C	A	CDHR4	SILENT	ENSG00000187492:[ENST00000412678(c.1800G>T,ctG>ctT):ENSP00000391409(p.Leu600(=))]	NA	NA	NA	NA	NA	NA	NA	NA	NA	NA	LOW	NA	NA	240	239	0	177	170	6	3.39
Chewer	IOB_09	chr3	98251385	C	T	CPOX	INTRONIC	ENSG00000080819:[ENST00000512905(c.*71-11099G>A)]	NA	NA	NA	NA	NA	NA	NA	NA	NA	NA	LOW	NA	0.411	121	120	0	134	117	16	11.94
Chewer	IOB_09	chr3	98251385	C	T	GPR15	SILENT	ENSG00000154165:[ENST00000284311(c.508C>T,Ctg>Ttg):ENSP00000284311(p.Leu170(=))]	NA	NA	NA	NA	NA	NA	NA	NA	NA	NA	LOW	NA	0.411	121	120	0	134	117	16	11.94
Chewer	IOB_09	chr3	126707754	C	A	PLXNA1	MISSENSE	ENSG00000114554:[ENST00000251772(c.249C>A,caC>caA):ENSP00000251772(p.His83Gln)]	NA	NA	chr3*126707754*126707754*C*T*rs770280214*NA*NA*NA	NA	NA	NA	NA	T	BN	NA	MODERATE	D	0.006	341	340	0	347	284	61	17.58
Chewer	IOB_09	chr3	128986968	C	A	COPG1	NCT	ENSG00000181789:[ENST00000515725(n.1907+85C>A)]	NA	NA	NA	NA	NA	NA	NA	NA	NA	NA	MODIFIER	NA	NA	70	69	0	60	55	5	8.33
Chewer	IOB_09	chr3	164906236	C	A	SLITRK3	NONSENSE	ENSG00000121871:[ENST00000241274(c.2383G>T,Gag>Tag):ENSP00000241274(p.Glu795Ter)]	NA	NA	chr3*164906236*164906236*C*T*rs747597295*NA*NA*NA	NA	NA	NA	NA	NA	NA	NA	HIGH	NA	0.999	196	194	1	221	183	38	17.19
Chewer	IOB_09	chr3	167034795	C	T	ZBBX	MISSENSE-SS-PRX	ENSG00000169064:[ENST00000469220(n.256G>A)]	NA	NA	NA	NA	NA	NA	NA	NA	NA	NA	MODERATE	NA	1	158	158	0	149	130	19	12.75
Chewer	IOB_09	chr3	178952085	A	G	PIK3CA	MISSENSE	ENSG00000121879:[ENST00000263967(c.3140A>G,cAt>cGt):ENSP00000263967(p.His1047Arg)]	NA	chr3*178952085*178952085*COSM94986*A*G*202*large_intestine,breast,prostate,endometrium,urinary_tract,skin,pancreas,ovary,liver,oesophagus,upper_aerodigestive_tract,kidney,lung,central_nervous_system,stomach*16,133,2,16,1,3,2,3,7,3,6,2,4,3,1|chr3*178952085*178952085*COSM249874*A*C*1*large_intestine*1|chr3*178952085*178952085*COSM776*A*T*226*breast,urinary_tract,large_intestine,thyroid,liver,endometrium,haematopoietic_and_lymphoid_tissue,pituitary,lung,soft_tissue,skin,ovary,central_nervous_system,upper_aerodigestive_tract,gastrointestinal_tract_(site_indeterminate),pancreas,NS,kidney*102,10,45,2,2,32,8,1,2,1,2,6,3,5,1,1,2,1|chr3*178952085*178952085*COSM94987*A*T*36*breast,upper_aerodigestive_tract,large_intestine,urinary_tract,pancreas,liver,lung,central_nervous_system,NS,endometrium,kidney*17,	NA	8.32E-06	0.00E+00	NA	NA	T	BN	NA	MODERATE	D	1	248	245	0	335	275	60	17.91
Chewer	IOB_09	chr3	183907597	G	A	ABCF3	NCT	ENSG00000161204:[ENST00000473311(n.1715-46G>A)]	NA	NA	NA	NA	NA	NA	NA	NA	NA	NA	MODIFIER	NA	NA	137	137	0	133	110	23	17.29
Chewer	IOB_09	chr3	183907597	G	A	EIF2B5	INTRONIC	ENSG00000145191:[ENST00000444495(c.2106+45102G>A)]	NA	NA	NA	NA	NA	NA	NA	NA	NA	NA	MODIFIER	NA	NA	137	137	0	133	110	23	17.29
Chewer	IOB_09	chr3	185783811	C	T	ETV5	MISSENSE	ENSG00000244405:[ENST00000306376(c.701G>A,gGa>gAa):ENSP00000306894(p.Gly234Glu)]	NA	chr3*185783811*185783811*COSM310947*C*A*1*lung*1|chr3*185783811*185783811*COSM310946*C*A*1*lung*1	chr3*185783811*185783811*C*G*rs749793277*NA*NA*NA	NA	NA	NA	NA	NA	NA	NA	MODIFIER	NA	NA	68	68	0	61	51	9	14.75
Chewer	IOB_09	chr4	860943	G	A	GAK	SILENT	ENSG00000178950:[ENST00000511980(c.8C>T,ggC>ggT):ENSP00000421049(p.Gly3(=))]	NA	chr4*860943*860943*COSM1431447*G*A*1*large_intestine*1	NA	9.15E-06	0.00E+00	NA	NA	NA	NA	NA	LOW	NA	NA	48	47	0	56	42	14	25
Chewer	IOB_09	chr4	1976629	C	T	WHSC1	NONSENSE	ENSG00000109685:[ENST00000382891(c.3412C>T,Cga>Tga):ENSP00000372347(p.Arg1138Ter)]	NA	NA	NA	NA	NA	NA	NA	NA	NA	NA	HIGH	NA	0.999	127	127	0	170	131	37	21.76
Chewer	IOB_09	chr4	20736245	C	A	KCNIP4	INTRONIC	80333:[XM_005248190.1(c.486+6G>T)]	NA	NA	NA	NA	NA	NA	NA	NA	NA	NA	LOW	NA	NA	112	111	0	102	96	5	4.9
Chewer	IOB_09	chr4	20736245	C	A	PACRGL	NCT	133015:[NR_133933.1(n.1051+7225C>A)]	NA	NA	NA	NA	NA	NA	NA	NA	NA	NA	LOW	NA	NA	112	111	0	102	96	5	4.9
Chewer	IOB_09	chr4	22820444	C	A	GBA3	MISSENSE	ENSG00000249948:[ENST00000503442(c.387C>A,ttC>ttA):ENSP00000422220(p.Phe129Leu)]	NA	NA	NA	NA	NA	NA	NA	NA	NA	NA	MODIFIER	NA	NA	67	67	0	75	70	5	6.67
Chewer	IOB_09	chr4	41600976	G	A	LIMCH1	MISSENSE	ENSG00000064042:[ENST00000313860(c.277G>A,Ggc>Agc):ENSP00000316891(p.Gly93Ser)]	NA	NA	NA	2.52E-05	6.17E-05	NA	NA	NA	NA	NA	MODIFIER	NA	NA	84	83	0	109	86	23	21.1
Chewer	IOB_09	chr4	41663454	G	A	LIMCH1	SILENT	ENSG00000064042:[ENST00000503057(c.3054G>A,acG>acA):ENSP00000425631(p.Thr1018(=))]	NA	NA	NA	1.70E-05	0.00E+00	NA	NA	NA	NA	NA	LOW	NA	NA	123	121	2	116	99	17	14.66
Chewer	IOB_09	chr4	48018988	G	A	NIPAL1	NCT	ENSG00000163293:[ENST00000513724(n.564-8097G>A)]	NA	NA	chr4*48018988*48018989*GA*G*rs970040371*NA*NA*NA	NA	NA	NA	NA	NA	NA	NA	MODIFIER	NA	NA	40	40	0	52	32	20	38.46
Chewer	IOB_09	chr4	62936428	G	A	ADGRL3	SILENT	23284:[NM_001322402.1(c.4416G>A,ccG>ccA):NP_001309331.1(p.Pro1472(=))]	NA	NA	NA	9.49E-05	2.54E-04	NA	NA	NA	NA	NA	LOW	NA	NA	75	74	1	90	80	10	11.11
Chewer	IOB_09	chr4	62936428	G	A	LPHN3	SILENT	ENSG00000150471:[ENST00000506746(c.4518G>A,ccG>ccA):ENSP00000425884(p.Pro1506(=))]	NA	NA	NA	9.49E-05	2.54E-04	NA	NA	NA	NA	NA	LOW	NA	NA	75	74	1	90	80	10	11.11

Patel *et al.*, 2021. Whole-exome sequencing analysis of oral squamous cell carcinoma delineated by tobacco usage habits
Supplementary Table 4. List of somatic single nucleotide variants identified using whole exome sequencing of OSCC patients

Cohort	Sample	Chromosome	Genomic position	Reference allele	Alternate allele	Genesymbol	Variant type	Variant description	OncoMD	COSMIC	dbSNP	ExAC	ExAC_SAS	1000G	1000G_SAS	SIFT	Polyphen2	CONDEL	VEP	LRT	PhastCon7Way	Total reads in Normal	Reference read support in Normal	Alternative read support in Normal	Total reads in Tumor	Reference read support in Tumor	Alternate read support in Tumor	Alternate allele frequency in Tumor
Chewer	IOB_09	chr4	73003869	C	A	NPFFR2	INTRONIC	ENSG00000056291:[ENST00000395999(c.437+13C>A)]	NA	NA	NA	NA	NA	NA	NA	NA	NA	NA	MODIFIER	NA	NA	204	202	0	189	182	6	3.17
Chewer	IOB_09	chr4	89668059	C	G	FAM13A	INTRONIC	10144:[XM_005262681.1(c.2424+24G>C)]	NA	NA	NA	NA	NA	NA	NA	NA	NA	NA	MODIFIER	NA	NA	58	58	0	95	69	26	27.37
Chewer	IOB_09	chr4	145581029	C	T	HHIP	SILENT	ENSG00000164161:[ENST00000434550(c.870C>T,taC>taT):ENSP00000408587(p.Tyr290(=))]	NA	NA	chr4*145581029*145581029*C*A,T*rs200052054*NA*NA*NA	9.94E-06	0.00E+00	NA	NA	NA	NA	NA	LOW	NA	NA	59	58	1	57	46	11	19.3
Chewer	IOB_09	chr4	145581029	C	T	HHIP-AS1	NCT	ENSG00000248890:[ENST00000512359(n.84-138G>A)]	NA	NA	chr4*145581029*145581029*C*A,T*rs200052054*NA*NA*NA	9.94E-06	0.00E+00	NA	NA	NA	NA	NA	LOW	NA	NA	59	58	1	57	46	11	19.3
Chewer	IOB_09	chr4	164246774	A	T	NPY1R	MISSENSE	ENSG00000164128:[ENST00000296533(c.836T>A,cTt>cAt):ENSP00000354652(p.Leu279His)]	NA	NA	NA	NA	NA	NA	NA	D	PrD	NA	MODERATE	N	1	125	125	0	137	103	33	24.09
Chewer	IOB_09	chr5	39341226	A	T	C9	NCT	ENSG00000113600:[ENST00000509186(n.447+22T>A)]	NA	NA	NA	NA	NA	NA	NA	NA	NA	NA	MODIFIER	NA	NA	49	49	0	57	47	10	17.54
Chewer	IOB_09	chr5	63256400	C	T	HTR1A	MISSENSE	ENSG00000178394:[ENST00000323865(c.1147G>A,Gcc>Acc):ENSP00000316244(p.Ala383Thr)]	NA	chr5*63256400*63256400*COSM1069279*C*T*2*pancreas,endometrium*1,1	NA	NA	NA	NA	NA	NA	NA	NA	MODIFIER	NA	NA	221	218	1	297	235	59	19.87
Chewer	IOB_09	chr5	63256400	C	T	RP11-158J3.2	NCT	ENSG00000248285:[ENST00000502882(n.97-2558G>A)]	NA	chr5*63256400*63256400*COSM1069279*C*T*2*pancreas,endometrium*1,1	NA	NA	NA	NA	NA	NA	NA	NA	MODIFIER	NA	NA	221	218	1	297	235	59	19.87
Chewer	IOB_09	chr5	148386621	C	A	SH3TC2	MISSENSE	ENSG00000169247:[ENST00000538184(c.2131G>T,Ggt>Tgt):ENSP00000441427(p.Gly711Cys)]	NA	NA	NA	NA	NA	NA	NA	NA	NA	NA	LOW	NA	NA	150	150	0	166	159	6	3.61
Chewer	IOB_09	chr5	159840843	C	G	SLU7	INTRONIC	ENSG00000164609:[ENST00000297151(c.405+63G>C)]	NA	NA	chr5*159840843*159840843*C*A*rs920702527*NA*NA*NA	NA	NA	NA	NA	NA	NA	NA	MODIFIER	NA	NA	35	35	0	35	25	10	28.57
Chewer	IOB_09	chr5	178581902	C	T	ADAMTS2	MISSENSE	ENSG00000087116:[ENST00000251582(c.1151G>A,gGc>gAc):ENSP00000251582(p.Gly384Asp)]	NA	NA	NA	NA	NA	NA	NA	D	PrD	NA	MODERATE	D	1	117	117	0	124	118	6	4.84
Chewer	IOB_09	chr5	179201007	C	G	MAML1	NONSENSE	ENSG00000161021:[ENST00000292599(c.2180C>G,tCa>tGa):ENSP00000292599(p.Ser727Ter)]	NA	NA	NA	NA	NA	NA	NA	NA	NA	NA	MODIFIER	NA	NA	203	203	0	179	147	31	17.32
Chewer	IOB_09	chr6	1612318	C	A	FOXC1	MISSENSE	ENSG00000054598:[ENST00000380874(c.1638C>A,ttC>ttA):ENSP00000370256(p.Phe546Leu)]	NA	NA	NA	NA	NA	NA	NA	D	PrD	NA	MODERATE	U	0.989	83	83	0	80	60	20	25
Chewer	IOB_09	chr6	18148134	C	A	TPMT	MISSENSE	ENSG00000137364:[ENST00000309983(c.153G>T,aaG>aaT):ENSP00000312304(p.Lys51Asn)]	NA	NA	NA	NA	NA	NA	NA	T	BN	NA	MODERATE	D	0.994	38	38	0	43	37	5	11.63
Chewer	IOB_09	chr6	20151442	G	A	MBOAT1	SILENT	ENSG00000172197:[ENST00000324607(c.297C>T,gtC>gtT):ENSP00000324944(p.Val99(=))]	NA	NA	NA	NA	NA	NA	NA	NA	NA	NA	LOW	NA	NA	128	128	0	174	136	38	21.84
Chewer	IOB_09	chr6	32497905	G	T	HLA-DRB5	SILENT	ENSG00000198502:[ENST00000374975(c.97C>A,Cga>Aga):ENSP00000364114(p.Arg33(=))]	NA	NA	chr6*32497904*32497905*CG*C*rs765668095*NA*NA*NA|chr6*32497905*32497905*G*A,C,T*rs71549219*NA*NA*NA	3.14E-01	3.22E-01	NA	NA	NA	NA	NA	LOW	NA	0	39	33	0	61	42	11	18.03
Chewer	IOB_09	chr6	35837637	G	A	SRPK1	MISSENSE	ENSG00000096063:[ENST00000423325(c.985C>T,Cgt>Tgt):ENSP00000391069(p.Arg329Cys)]	NA	NA	NA	NA	NA	NA	NA	NA	NA	NA	MODIFIER	NA	NA	204	202	0	232	177	55	23.71
Chewer	IOB_09	chr6	36298227	T	C	C6orf222	MISSENSE	ENSG00000189325:[ENST00000437635(c.241A>G,Acc>Gcc):ENSP00000418983(p.Thr81Ala)]	NA	NA	NA	NA	NA	NA	NA	T	BN	NA	MODERATE	NA	NA	153	153	0	149	122	27	18.12
Chewer	IOB_09	chr6	47682910	C	A	GPR115	MISSENSE	ENSG00000153294:[ENST00000371220(c.2100C>A,ttC>ttA):ENSP00000360264(p.Phe700Leu)]	NA	NA	chr6*47682910*47682910*C*G,T*rs768945108*NA*NA*NA	NA	NA	NA	NA	T	PoD	NA	MODERATE	D	0.751	134	134	0	135	128	7	5.19
Chewer	IOB_09	chr6	56480524	G	C	DST	MISSENSE	ENSG00000151914:[ENST00000370765(c.7741C>G,Caa>Gaa):ENSP00000359801(p.Gln2581Glu)]	NA	NA	NA	NA	NA	NA	NA	NA	NA	NA	MODIFIER	NA	NA	225	224	0	206	160	43	20.87
Chewer	IOB_09	chr6	57498895	C	T	NA	NCT	100996481:[XR_171038.2(n.1325-72C>T)]	NA	NA	NA	NA	NA	NA	NA	NA	NA	NA	MODIFIER	NA	NA	23	22	1	48	40	8	16.67
Chewer	IOB_09	chr6	57498895	C	T	PRIM2	INTRONIC	5558:[NM_000947.4(c.1231-72C>T)]	NA	NA	NA	NA	NA	NA	NA	NA	NA	NA	MODIFIER	NA	NA	23	22	1	48	40	8	16.67
Chewer	IOB_09	chr6	90494717	C	A	MDN1	INTRONIC	ENSG00000112159:[ENST00000439638(c.1231-3406G>T)]	NA	NA	NA	NA	NA	NA	NA	NA	NA	NA	MODIFIER	NA	NA	177	177	0	172	166	5	2.91
Chewer	IOB_09	chr6	90718551	C	T	BACH2	MISSENSE	ENSG00000112182:[ENST00000257749(c.13G>A,Gag>Aag):ENSP00000257749(p.Glu5Lys)]	NA	NA	NA	NA	NA	NA	NA	NA	NA	NA	MODERATE	NA	1	37	37	0	29	23	5	17.24
Chewer	IOB_09	chr6	116325095	G	A	FRK	SILENT	ENSG00000111816:[ENST00000606080(c.411C>T,acC>acT):ENSP00000476145(p.Thr137(=))]	NA	NA	NA	9.08E-05	0.00E+00	NA	NA	NA	NA	NA	LOW	NA	NA	92	91	1	142	117	24	16.9
Chewer	IOB_09	chr6	134210894	C	T	RP3-323P13.2	NCT	ENSG00000227954:[ENST00000607573(n.250+1931G>A)]	NA	NA	NA	NA	NA	NA	NA	NA	NA	NA	MODIFIER	NA	NA	164	162	2	144	116	28	19.44
Chewer	IOB_09	chr6	134210894	C	T	TCF21	MISSENSE	ENSG00000118526:[ENST00000237316(c.359C>T,aCg>aTg):ENSP00000237316(p.Thr120Met)]	NA	NA	NA	NA	NA	NA	NA	NA	NA	NA	MODIFIER	NA	NA	164	162	2	144	116	28	19.44
Chewer	IOB_09	chr6	135375726	A	T	HBS1L	NCT	10767:[XR_245499.1(n.200+61T>A)]	NA	NA	NA	NA	NA	NA	NA	NA	NA	NA	MODIFIER	NA	NA	146	146	0	149	135	14	9.4
Chewer	IOB_09	chr6	146920214	G	A	ADGB	MISSENSE	ENSG00000118492:[ENST00000397944(c.38G>A,cGt>cAt):ENSP00000381036(p.Arg13His)]	NA	NA	NA	NA	NA	NA	NA	NA	NA	NA	MODIFIER	NA	NA	101	100	0	108	95	12	11.11
Chewer	IOB_09	chr6	158497737	C	A	SYNJ2	MISSENSE	ENSG00000078269:[ENST00000355585(c.2372C>A,aCa>aAa):ENSP00000347792(p.Thr791Lys)]	NA	NA	NA	NA	NA	NA	NA	D	PrD	NA	MODERATE	D	1	83	82	1	88	81	6	6.82
Chewer	IOB_09	chr6	160148296	A	G	SOD2	INTRONIC-SS-DNR	ENSG00000112096:[ENST00000545162(c.92+2T>C)]	NA	NA	NA	NA	NA	NA	NA	NA	NA	NA	MODIFIER	NA	NA	107	106	0	142	121	20	14.08
Chewer	IOB_09	chr6	160148296	A	G	WTAP	INTRONIC	9589:[XM_005267232.1(c.43+411A>G)]	NA	NA	NA	NA	NA	NA	NA	NA	NA	NA	MODIFIER	NA	NA	107	106	0	142	121	20	14.08
Chewer	IOB_09	chr6	161097546	G	C	RP1-81D8.4	EXONIC-NC	ENSG00000243831:[ENST00000452651(n.10C>G)]	NA	NA	NA	NA	NA	NA	NA	NA	NA	NA	MODIFIER	NA	NA	129	129	0	117	94	22	18.8
Chewer	IOB_09	chr6	165808670	C	A	PDE10A	MISSENSE-SS-PRX	ENSG00000112541:[ENST00000354448(c.1475G>T,tGc>tTc):ENSP00000346435(p.Cys492Phe)]	NA	NA	NA	NA	NA	NA	NA	NA	NA	NA	LOW	NA	NA	128	128	0	105	100	5	4.76
Chewer	IOB_09	chr7	14733634	C	G	DGKB	INTRONIC	ENSG00000136267:[ENST00000258767(c.711+66G>C)]	NA	NA	chr7*14733634*14733634*C*T*rs185827387*1*0.999*0.0009984	NA	NA	NA	NA	NA	NA	NA	MODIFIER	NA	NA	33	32	0	23	12	11	47.83
Chewer	IOB_09	chr7	47851456	G	A	C7orf69	INTRONIC	ENSG00000136275:[ENST00000258776(c.109-6168G>A)]	NA	NA	NA	6.44E-05	1.82E-04	NA	NA	NA	NA	NA	MODIFIER	NA	NA	101	101	0	135	113	21	15.56
Chewer	IOB_09	chr7	47851456	G	A	HUS1	INTRONIC	ENSG00000136273:[ENST00000436444(c.*1171-116083C>T)]	NA	NA	NA	6.44E-05	1.82E-04	NA	NA	NA	NA	NA	MODIFIER	NA	NA	101	101	0	135	113	21	15.56
Chewer	IOB_09	chr7	47851456	G	A	PKD1L1	MISSENSE	ENSG00000158683:[ENST00000289672(c.7540C>T,Cgc>Tgc):ENSP00000289672(p.Arg2514Cys)]	NA	NA	NA	6.44E-05	1.82E-04	NA	NA	NA	NA	NA	MODIFIER	NA	NA	101	101	0	135	113	21	15.56
Chewer	IOB_09	chr7	76029657	C	T	SRCRB4D	MISSENSE	ENSG00000146700:[ENST00000275560(c.421G>A,Ggc>Agc):ENSP00000275560(p.Gly141Ser)]	NA	NA	NA	1.88E-05	0.00E+00	NA	NA	D	PrD	NA	MODERATE	D	1	252	250	0	200	164	36	18
Chewer	IOB_09	chr7	76029657	C	T	ZP3	INTRONIC	ENSG00000188372:[ENST00000336517(c.-67+2543C>T)]	NA	NA	NA	1.88E-05	0.00E+00	NA	NA	D	PrD	NA	MODERATE	D	1	252	250	0	200	164	36	18
Chewer	IOB_09	chr7	82585561	A	G	PCLO	MISSENSE	ENSG00000186472:[ENST00000333891(c.4708T>C,Tca>Cca):ENSP00000334319(p.Ser1570Pro)]	NA	NA	NA	NA	NA	NA	NA	D	PrD	NA	MODERATE	NA	NA	288	285	0	370	290	79	21.35
Chewer	IOB_09	chr7	148484186	G	A	CUL1	MISSENSE	ENSG00000055130:[ENST00000325222(c.1453G>A,Gaa>Aaa):ENSP00000326804(p.Glu485Lys)]	NA	chr7*148484186*148484186*COSM302016*G*A*4*central_nervous_system,lung,urinary_tract*1,1,2	NA	NA	NA	NA	NA	NA	NA	NA	MODERATE	NA	1	35	35	0	35	19	15	42.86
**Chewer**	**IOB_09**	**chr8**	**3072184**	**C**	**T**	**CSMD1**	**MISSENSE**	ENSG00000183117:[ENST00000537824(c.4702G>A,Gga>Aga):ENSP00000441462(p.Gly1568Arg)]	NA	NA	NA	NA	NA	NA	NA	D	PrD	NA	MODERATE	NA	NA	58	56	0	82	67	15	18.29
Chewer	IOB_09	chr8	24256409	A	T	ADAMDEC1	MISSENSE	ENSG00000134028:[ENST00000522298(c.548A>T,cAa>cTa):ENSP00000428993(p.Gln183Leu)]	NA	NA	NA	NA	NA	NA	NA	T	BN	NA	MODERATE	N	0.026	123	123	0	167	162	5	2.99
Chewer	IOB_09	chr8	24256409	A	T	RP11-624C23.1	NCT	ENSG00000253535:[ENST00000523578(n.80-10905T>A)]	NA	NA	NA	NA	NA	NA	NA	T	BN	NA	MODERATE	N	0.026	123	123	0	167	162	5	2.99
Chewer	IOB_09	chr8	77765144	C	A	ZFHX4	MISSENSE	ENSG00000091656:[ENST00000518282(c.5909C>A,tCt>tAt):ENSP00000430848(p.Ser1970Tyr)]	NA	chr8*77765144*77765144*COSM264821*C*T*1*large_intestine*1	NA	NA	NA	NA	NA	D	BN	NA	MODERATE	U	0.997	130	130	0	159	138	21	13.21
Chewer	IOB_09	chr8	97156855	G	A	GDF6	MISSENSE	ENSG00000156466:[ENST00000287020(c.1304C>T,gCg>gTg):ENSP00000287020(p.Ala435Val)]	NA	chr8*97156855*97156855*COSM1132661*G*A*1*prostate*1	NA	1.89E-04	1.87E-04	NA	NA	D	PrD	NA	MODERATE	D	1	112	112	0	126	106	20	15.87
Chewer	IOB_09	chr8	124253604	G	A	C8orf76	EXONIC-NC	ENSG00000189376:[ENST00000522477(n.14C>T)]	NA	NA	NA	NA	NA	NA	NA	NA	NA	NA	MODIFIER	NA	NA	123	123	0	105	84	21	20
Chewer	IOB_09	chr8	124253604	G	A	ZHX1-C8ORF76	INTRONIC	ENSG00000259305:[ENST00000357082(c.22-2220C>T)]	NA	NA	NA	NA	NA	NA	NA	NA	NA	NA	MODIFIER	NA	NA	123	123	0	105	84	21	20
Chewer	IOB_09	chr8	144996986	C	T	PLEC	MISSENSE	ENSG00000178209:[ENST00000322810(c.7522G>A,Gag>Aag):ENSP00000323856(p.Glu2508Lys)]	NA	NA	NA	2.22E-05	0.00E+00	NA	NA	D_lc	PoD	NA	MODERATE	U	1	66	66	0	71	50	21	29.58
Chewer	IOB_09	chr9	68415322	A	G	MIR4477A	EXONIC-NC	ENSG00000266017:[ENST00000581659(n.15A>G)]	NA	NA	NA	1.39E-02	0.00E+00	NA	NA	NA	NA	NA	MODIFIER	NA	NA	250	248	1	283	272	11	3.89
Chewer	IOB_09	chr9	113252076	A	C	SVEP1	INTRONIC	ENSG00000165124:[ENST00000374461(c.1732-17T>G)]	NA	NA	NA	NA	NA	NA	NA	NA	NA	NA	MODIFIER	NA	NA	74	74	0	67	54	13	19.4
Chewer	IOB_09	chr9	140260802	G	A	EXD3	MISSENSE	ENSG00000187609:[ENST00000479452(c.668C>T,aCt>aTt):ENSP00000431859(p.Thr223Ile)]	NA	NA	NA	NA	NA	NA	NA	NA	NA	NA	MODIFIER	NA	NA	201	201	0	167	138	29	17.37
Chewer	IOB_09	chrX	76937639	T	C	ATRX	MISSENSE	ENSG00000085224:[ENST00000373344(c.3109A>G,Aat>Gat):ENSP00000362441(p.Asn1037Asp)]	NA	NA	NA	NA	NA	NA	NA	NA	BN	NA	MODERATE	N	0.232	129	129	0	112	100	12	10.71
Chewer	IOB_09	chrX	77041428	C	A	ATRX	INTRONIC	ENSG00000085224:[ENST00000373344(c.20+40G>T)]	NA	NA	NA	NA	NA	NA	NA	NA	NA	NA	MODIFIER	NA	NA	191	191	0	163	156	5	3.07
Chewer	IOB_09	chrX	80064918	A	C	BRWD3	INTRONIC	ENSG00000165288:[ENST00000373275(c.31+22T>G)]	NA	NA	NA	6.77E-04	0.00E+00	NA	NA	NA	NA	NA	MODIFIER	NA	NA	50	48	2	55	47	5	9.09
Chewer	IOB_09	chrX	96139888	C	T	DIAPH2	INTRONIC	1730:[XM_005262097.1(c.608+3171C>T)]	NA	chrX*96139888*96139888*COSM1559160*C*T*1*lung*1	NA	1.14E-05	9.88E-05	NA	NA	NA	NA	NA	MODIFIER	NA	NA	112	112	0	118	90	28	23.73
Chewer	IOB_09	chrX	96139888	C	T	RPA4	SILENT	ENSG00000204086:[ENST00000373040(c.579C>T,aaC>aaT):ENSP00000362131(p.Asn193(=))]	NA	chrX*96139888*96139888*COSM1559160*C*T*1*lung*1	NA	1.14E-05	9.88E-05	NA	NA	NA	NA	NA	MODIFIER	NA	NA	112	112	0	118	90	28	23.73
Chewer	IOB_09	chrX	132352231	G	T	TFDP3	MISSENSE	ENSG00000183434:[ENST00000310125(c.57C>A,gaC>gaA):ENSP00000385461(p.Asp19Glu)]	NA	NA	NA	NA	NA	NA	NA	D_lc	PrD	NA	MODERATE	NA	NA	148	146	0	155	139	14	9.03
Chewer	IOB_09	chrX	151532931	C	A	GABRA3	MISSENSE	ENSG00000011677:[ENST00000370314(c.112G>T,Ggg>Tgg):ENSP00000359337(p.Gly38Trp)]	NA	NA	chrX*151532931*151532931*C*T*rs367755490*NA*NA*NA	NA	NA	NA	NA	D_lc	PrD	NA	MODERATE	N	0.996	47	46	0	79	74	5	6.33
Chewer	IOB_09	chrX	154157097	A	G	F8	SILENT	ENSG00000185010:[ENST00000360256(c.4968T>C,tcT>tcC):ENSP00000353393(p.Ser1656(=))]	NA	NA	NA	NA	NA	NA	NA	NA	NA	NA	LOW	NA	NA	236	236	0	262	221	40	15.27
Chewer	IOB_10	chr1	3686378	C	A	CCDC27	MISSENSE	ENSG00000162592:[ENST00000294600(c.1775C>A,gCc>gAc):ENSP00000294600(p.Ala592Asp)]	NA	NA	NA	NA	NA	NA	NA	NA	NA	NA	MODIFIER	NA	NA	119	118	0	122	116	6	4.92
Chewer	IOB_10	chr1	11708857	G	A	FBXO2	MISSENSE	ENSG00000116661:[ENST00000354287(c.785C>T,cCg>cTg):ENSP00000346240(p.Pro262Leu)]	NA	chr1*11708857*11708857*COSM423546*G*C*1*breast*1	NA	NA	NA	NA	NA	NA	NA	NA	MODIFIER	NA	NA	97	97	0	118	80	37	31.36
Chewer	IOB_10	chr1	11828314	G	A	C1orf167	SILENT	ENSG00000215910:[ENST00000433342(c.1707G>A,ctG>ctA):ENSP00000414909(p.Leu569(=))]	NA	NA	NA	NA	NA	NA	NA	NA	NA	NA	MODIFIER	NA	NA	107	107	0	113	108	5	4.42
Chewer	IOB_10	chr1	11907411	G	A	NPPA	MISSENSE	ENSG00000175206:[ENST00000376480(c.209C>T,gCg>gTg):ENSP00000365663(p.Ala70Val)]	NA	NA	NA	2.50E-05	1.82E-04	NA	NA	NA	NA	NA	MODIFIER	NA	NA	88	87	0	127	86	41	32.28
Chewer	IOB_10	chr1	11907411	G	A	NPPA-AS1	NCT	ENSG00000242349:[ENST00000400892(n.434-223G>A)]	NA	NA	NA	2.50E-05	1.82E-04	NA	NA	NA	NA	NA	MODIFIER	NA	NA	88	87	0	127	86	41	32.28
Chewer	IOB_10	chr1	15888713	G	A	DNAJC16	MISSENSE	ENSG00000116138:[ENST00000375838(c.1231G>A,Gca>Aca):ENSP00000364998(p.Ala411Thr)]	NA	NA	NA	NA	NA	NA	NA	NA	NA	NA	MODIFIER	NA	NA	229	228	0	173	168	5	2.89
Chewer	IOB_10	chr1	27621004	G	A	WDTC1	INTRONIC-SS-ACR	ENSG00000142784:[ENST00000319394(c.758-1G>A)]	NA	NA	NA	NA	NA	NA	NA	NA	NA	NA	HIGH	NA	NA	84	84	0	96	65	31	32.29

Patel *et al.* , 2021. Whole-exome sequencing analysis of oral squamous cell carcinoma delineated by tobacco usage habits
Supplementary Table 4. List of somatic single nucleotide variants identified using whole exome sequencing of OSCC patients

Cohort	Sample	Chromosome	Genomic position	Reference allele	Alternate allele	Genesymbol	Variant type	Variant description	OncoMD	COSMIC	dbSNP	ExAC	ExAC_SAS	1000G	1000G_SAS	SIFT	Polyphen2	CONDEL	VEP	LRT	PhastCon7Way	Total reads in Normal	Reference read support in Normal	Alternative read support in Normal	Total reads in Tumor	Reference read support in Tumor	Alternate read support in Tumor	Alternate allele frequency in Tumor
Chewer	IOB_10	chr1	35486079	A	G	RP11-244H3.4	INTRONIC	ENSG00000271741:[ENST00000487874(c.94-11T>C)]	NA	NA	chr1*35486079*35486079*A*AG*rs755592238*NA*NA*NA|chr1*35486079*35486084*AAAAAG*A*rs752098743*NA*NA*NA	NA	NA	NA	NA	NA	NA	NA	MODIFIER	NA	NA	34	34	0	33	20	12	36.36
Chewer	IOB_10	chr1	35486079	A	G	ZMYM6	NCT	ENSG00000163867:[ENST00000493328(n.292-11T>C)]	NA	NA	chr1*35486079*35486079*A*AG*rs755592238*NA*NA*NA|chr1*35486079*35486084*AAAAAG*A*rs752098743*NA*NA*NA	NA	NA	NA	NA	NA	NA	NA	MODIFIER	NA	NA	34	34	0	33	20	12	36.36
Chewer	IOB_10	chr1	36809769	G	A	STK40	MISSENSE	ENSG00000196182:[ENST00000373130(c.851C>T,cCg>cTg):ENSP00000362222(p.Pro284Leu)]	NA	NA	NA	NA	NA	NA	NA	T	PrD	NA	MODERATE	D	1	143	138	0	169	107	55	32.54
Chewer	IOB_10	chr1	45293183	G	A	PTCH2	SILENT	ENSG00000117425:[ENST00000372192(c.2262C>T,cgC>cgT):ENSP00000361266(p.Arg754(=))]	NA	NA	NA	1.16E-04	6.06E-05	NA	NA	NA	NA	NA	LOW	NA	NA	69	68	1	95	59	36	37.89
Chewer	IOB_10	chr1	47283845	C	T	CYP4B1	MISSENSE	ENSG00000142973:[ENST00000452782(c.826C>T,Cgc>Tgc):ENSP00000400413(p.Arg276Cys)]	NA	NA	3.99E-04	2.47E-05	0.00E+00	3.99E-04	0.00E+00	D	PrD	NA	MODERATE	D	0.116	156	156	0	146	104	42	28.77
Chewer	IOB_10	chr1	55168313	G	T	MROH7	SILENT	ENSG00000184313:[ENST00000454855(c.2013G>T,ctG>ctT):ENSP00000401130(p.Leu671(=))]	NA	NA	chr1*55168310*55168318*CCTGTTACG*C*rs750210690*NA*NA*NA	NA	NA	NA	NA	NA	NA	NA	LOW	NA	NA	198	198	0	189	181	6	3.17
Chewer	IOB_10	chr1	55168313	G	T	MROH7-TTC4	SILENT	ENSG00000271723:[ENST00000414150(c.3459G>T,ctG>ctT):ENSP00000410192(p.Leu1153(=))]	NA	NA	chr1*55168310*55168318*CCTGTTACG*C*rs750210690*NA*NA*NA	NA	NA	NA	NA	NA	NA	NA	LOW	NA	NA	198	198	0	189	181	6	3.17
Chewer	IOB_10	chr1	60299736	T	C	HOOK1	NCT	51361:[XR_246272.1(n.602+527T>C)]	NA	NA	NA	NA	NA	NA	NA	NA	NA	NA	MODIFIER	NA	NA	114	112	0	130	85	43	33.08
Chewer	IOB_10	chr1	60503725	G	T	C1orf87	MISSENSE	ENSG00000162598:[ENST00000371201(c.802C>A,Cag>Aag):ENSP00000360244(p.Gln268Lys)]	NA	NA	NA	NA	NA	NA	NA	NA	NA	NA	MODIFIER	NA	NA	92	90	0	105	71	34	32.38
Chewer	IOB_10	chr1	114301263	A	T	PHTF1	NCT	10745:[NR_136566.1(n.708+18T>A)]	NA	NA	2.10E-02	2.24E-02	1.07E-02	2.10E-02	4.10E-03	NA	NA	NA	MODIFIER	NA	NA	47	47	0	45	37	8	17.78
Chewer	IOB_10	chr1	118539104	C	A	SPAG17	MISSENSE	ENSG00000155761:[ENST00000336338(c.4942G>T,Gat>Tat):ENSP00000337804(p.Asp1648Tyr)]	NA	NA	chr1*118539104*118539104*C*T*rs759404757*NA*NA*NA	NA	NA	NA	NA	D	PrD	NA	MODERATE	NA	NA	86	86	0	132	126	6	4.55
Chewer	IOB_10	chr1	120302490	G	A	HMGCS2	NONSENSE	ENSG00000134240:[ENST00000369406(c.682C>T,Cga>Tga):ENSP00000358414(p.Arg228Ter)]	NA	NA	NA	8.25E-06	0.00E+00	NA	NA	NA	NA	NA	HIGH	NA	1	117	115	0	127	85	38	29.92
Chewer	IOB_10	chr1	145014193	A	C	PDE4DIP	MISSENSE	ENSG00000178104:[ENST00000493130(c.349T>G,Ttt>Gtt):ENSP00000432251(p.Phe117Val)]	NA	NA	NA	NA	NA	NA	NA	NA	UN	NA	MODERATE	NA	NA	194	181	0	260	239	10	3.85
Chewer	IOB_10	chr1	149821867	A	G	HIST2H2BC	EXONIC-NC	ENSG00000203819:[ENST00000498492(n.473T>C)]	NA	NA	2.00E-04	1.92E-03	2.41E-04	2.00E-04	0.00E+00	NA	NA	NA	MODIFIER	NA	NA	143	143	0	172	88	80	46.51
Chewer	IOB_10	chr1	154920768	G	A	PBXIP1	MISSENSE	ENSG00000163346:[ENST00000368460(c.484C>T,Cgg>Tgg):ENSP00000357445(p.Arg162Trp)]	NA	NA	NA	NA	NA	NA	NA	NA	NA	NA	MODERATE	NA	0.985	86	85	0	127	56	71	55.91
Chewer	IOB_10	chr1	156563265	C	T	APOA1BP	NONSENSE	ENSG00000163382:[ENST00000368234(c.526C>T,Cga>Tga):ENSP00000357217(p.Arg176Ter)]	NA	NA	NA	3.30E-05	0.00E+00	NA	NA	NA	NA	NA	HIGH	NA	NA	113	113	0	121	100	21	17.36
Chewer	IOB_10	chr1	156818791	C	T	INSRR	MISSENSE	ENSG00000027644:[ENST00000368195(c.1493G>A,cGc>cAc):ENSP00000357178(p.Arg498His)]	NA	NA	NA	3.09E-05	7.43E-05	NA	NA	T	BN	NA	MODERATE	N	1	258	258	0	348	339	7	2.01
Chewer	IOB_10	chr1	156818791	C	T	NTRK1	NCT	ENSG00000198400:[ENST00000489021(n.312+6806C>T)]	NA	NA	NA	3.09E-05	7.43E-05	NA	NA	T	BN	NA	MODERATE	N	1	258	258	0	348	339	7	2.01
Chewer	IOB_10	chr1	160208579	G	T	DCAF8	EXONIC-NC	ENSG00000132716:[ENST00000466253(n.197C>A)]	NA	NA	chr1*160208578*160208580*TGA*T*rs752353308*NA*NA*NA|chr1*160208579*160208579*G*A*rs760927568*NA*NA*NA	NA	NA	NA	NA	NA	NA	NA	MODIFIER	NA	NA	23	22	0	25	18	7	28
Chewer	IOB_10	chr1	176564034	G	A	PAPPA2	MISSENSE	ENSG00000116183:[ENST00000367661(c.1294G>A,Gcc>Acc):ENSP00000356633(p.Ala432Thr)]	NA	chr1*176564034*176564034*COSM3385432*G*A*1*pancreas*1|chr1*176564034*176564034*COSM3385431*G*A*1*pancreas*1	NA	1.67E-05	0.00E+00	NA	NA	D	PrD	NA	MODERATE	N	0.998	279	277	0	457	242	213	46.61
Chewer	IOB_10	chr1	180062692	C	T	CEP350	SILENT	ENSG00000135837:[ENST00000429851(c.1975C>T,tcC>tcT):ENSP00000412460(p.Ser659(=))]	NA	NA	NA	NA	NA	NA	NA	NA	NA	NA	LOW	NA	NA	267	265	0	364	293	69	18.96
Chewer	IOB_10	chr1	180165463	G	T	QSOX1	MISSENSE	ENSG00000116260:[ENST00000367600(c.1535G>T,tGc>tTc):ENSP00000356572(p.Cys512Phe)]	NA	chr1*180165463*180165463*COSM1241642*G*A*1*oesophagus*1	NA	NA	NA	NA	NA	D	PrD	NA	MODERATE	D	1	234	234	0	318	183	132	41.51
Chewer	IOB_10	chr1	186089113	C	T	HMCN1	INTRONIC	ENSG00000143341:[ENST00000271588(c.12095-30C>T)]	NA	NA	NA	1.66E-05	6.06E-05	NA	NA	NA	NA	NA	MODIFIER	NA	NA	182	181	1	270	259	6	2.22
Chewer	IOB_10	chr1	197404076	C	T	CRB1	MISSENSE	ENSG00000134376:[ENST00000367397(c.1226C>T,tCa>tTa):ENSP00000356367(p.Ser409Leu)]	NA	NA	NA	NA	NA	NA	NA	T	BN	NA	MODERATE	NA	NA	206	205	0	323	250	72	22.29
Chewer	IOB_10	chr1	207110845	C	T	PIGR	MISSENSE	ENSG00000162896:[ENST00000356495(c.640G>A,Gat>Aat):ENSP00000348888(p.Asp214Asn)]	NA	NA	NA	NA	NA	NA	NA	T	PrD	NA	MODERATE	D	0.777	106	106	0	146	69	76	52.05
Chewer	IOB_10	chr1	210332830	C	A	SYT14	NCT-SS-PRX	ENSG00000143469:[ENST00000469604(n.1199+8C>A)]	NA	NA	NA	NA	NA	NA	NA	NA	NA	NA	MODIFIER	NA	NA	238	238	0	289	283	6	2.08
Chewer	IOB_10	chr1	211546038	T	C	TRAF5	SILENT	ENSG00000082512:[ENST00000427925(c.1350T>C,gaT>gaC):ENSP00000389891(p.Asp450(=))]	NA	NA	NA	NA	NA	NA	NA	NA	NA	NA	LOW	NA	0.054	124	124	0	151	115	34	22.52
Chewer	IOB_10	chr1	221875921	G	A	DUSP10	MISSENSE	ENSG00000143507:[ENST00000323825(c.256C>T,Cgg>Tgg):ENSP00000322015(p.Arg86Trp)]	NA	NA	NA	NA	NA	NA	NA	NA	NA	NA	MODIFIER	NA	NA	148	146	0	229	129	100	43.67
Chewer	IOB_10	chr1	223986253	G	A	TP53BP2	NONSENSE	ENSG00000143514:[ENST00000343537(c.1612C>T,Cag>Tag):ENSP00000341957(p.Gln538Ter)]	NA	NA	NA	NA	NA	NA	NA	NA	NA	NA	MODIFIER	NA	NA	126	124	1	162	120	42	25.93
Chewer	IOB_10	chr1	225339649	T	C	DNAH14	MISSENSE	ENSG00000185842:[ENST00000445597(c.3602T>C,tTa>tCa):ENSP00000409472(p.Leu1201Ser)]	NA	NA	NA	NA	NA	NA	NA	NA	NA	NA	MODIFIER	NA	NA	41	41	0	103	97	5	4.85
Chewer	IOB_10	chr1	228539182	G	A	OBSCN	INTRONIC	84033:[XM_005273305.1(c.20667+31G>A)]	NA	NA	NA	5.26E-05	1.42E-04	NA	NA	NA	NA	NA	MODIFIER	NA	NA	37	37	0	37	26	9	24.32
Chewer	IOB_10	chr1	228601573	C	T	TRIM17	MISSENSE	ENSG00000162931:[ENST00000479800(c.369G>A,atG>atA):ENSP00000430468(p.Met123Ile)]	NA	NA	NA	NA	NA	NA	NA	T	BN	NA	MODERATE	N	0.001	144	144	0	201	148	52	25.87
Chewer	IOB_10	chr1	237504822	G	T	RYR2	INTRONIC	ENSG00000198626:[ENST00000542537(c.225+10540G>T)]	NA	NA	NA	NA	NA	NA	NA	NA	NA	NA	MODIFIER	NA	NA	128	128	0	157	84	73	46.5
Chewer	IOB_10	chr1	237798310	C	A	RYR2	INTRONIC	ENSG00000198626:[ENST00000366574(c.6792+18C>A)]	NA	NA	NA	NA	NA	NA	NA	NA	NA	NA	MODIFIER	NA	NA	132	132	0	211	205	5	2.37
Chewer	IOB_10	chr10	293415	C	T	ZMYND11	INTRONIC-SS-DNR-PRX	ENSG00000015171:[ENST00000403354(c.987+9C>T)]	NA	NA	NA	NA	NA	NA	NA	NA	NA	NA	MODIFIER	NA	NA	110	110	0	120	113	5	4.17
Chewer	IOB_10	chr10	1421615	C	A	ADARB2	INTRONIC	ENSG00000185736:[ENST00000381312(c.101-260G>T)]	NA	NA	NA	NA	NA	NA	NA	NA	NA	NA	MODIFIER	NA	NA	216	216	0	216	209	5	2.31
Chewer	IOB_10	chr10	18276618	C	A	SLC39A12	INTRONIC	ENSG00000148482:[ENST00000539911(c.867+38C>A)]	NA	NA	NA	NA	NA	NA	NA	NA	NA	NA	MODIFIER	NA	NA	47	47	0	70	55	15	21.43
Chewer	IOB_10	chr10	21098819	C	A	NEBL	MISSENSE	ENSG00000078114:[ENST00000377122(c.2527G>T,Gtt>Ttt):ENSP00000366326(p.Val843Phe)]	NA	NA	NA	NA	NA	NA	NA	NA	NA	NA	MODIFIER	NA	NA	106	103	0	96	88	5	5.21
Chewer	IOB_10	chr10	22207780	C	A	DNAJC1	MISSENSE	ENSG00000136770:[ENST00000376980(c.657G>T,tgG>tgT):ENSP00000366179(p.Trp219Cys)]	NA	NA	NA	NA	NA	NA	NA	D	PrD	NA	MODERATE	D	1	188	188	0	189	182	6	3.17
Chewer	IOB_10	chr10	34671811	G	A	PARD3	SILENT	ENSG00000148498:[ENST00000544292(c.246C>T,atC>atT):ENSP00000444429(p.Ile82(=))]	NA	NA	NA	NA	NA	NA	NA	NA	NA	NA	LOW	NA	1	186	183	0	215	143	71	33.02
Chewer	IOB_10	chr10	45891370	G	A	ALOX5	SILENT	ENSG00000012779:[ENST00000374391(c.417G>A,cgG>cgA):ENSP00000363512(p.Arg139(=))]	NA	NA	NA	NA	NA	NA	NA	NA	NA	NA	LOW	NA	NA	214	214	0	244	236	6	2.46
Chewer	IOB_10	chr10	47087654	C	T	LINC00842	NCT	ENSG00000223477:[ENST00000503031(n.254-14675G>A)]	NA	NA	NA	NA	NA	NA	NA	NA	NA	NA	MODIFIER	NA	NA	455	452	0	530	471	58	10.94
Chewer	IOB_10	chr10	47087654	C	T	NPY4R	MISSENSE	ENSG00000204174:[ENST00000374312(c.871C>T,Cac>Tac):ENSP00000363431(p.His291Tyr)]	NA	NA	NA	NA	NA	NA	NA	NA	NA	NA	MODIFIER	NA	NA	455	452	0	530	471	58	10.94
Chewer	IOB_10	chr10	50574310	C	T	DRGX	MISSENSE	ENSG00000165606:[ENST00000434016(c.658G>A,Gag>Aag):ENSP00000401653(p.Glu220Lys)]	NA	NA	NA	1.66E-05	1.21E-04	NA	NA	D	PrD	NA	MODERATE	D	1	98	98	0	101	76	25	24.75
Chewer	IOB_10	chr10	50901939	G	A	C10orf53	MISSENSE	ENSG00000178645:-SS-PRX[ENST00000374111(c.217G>A,Gga>Aga):ENSP00000363225(p.Gly73Arg)]	NA	NA	NA	1.65E-05	0.00E+00	NA	NA	D_lc	PrD	NA	MODERATE	NA	NA	32	32	0	58	38	20	34.48
Chewer	IOB_10	chr10	70333807	C	A	TET1	MISSENSE	ENSG00000138336:[ENST00000373644(c.1712C>A,aCc>aAc):ENSP00000362748(p.Thr571Asn)]	NA	NA	NA	NA	NA	NA	NA	T	PrD	NA	MODERATE	N	0.007	116	116	0	103	98	5	4.85
Chewer	IOB_10	chr10	78785267	G	A	KCNMA1	INTRONIC	ENSG00000156113:[ENST00000372408(c.1739+2314C>T)]	NA	NA	NA	NA	NA	NA	NA	NA	NA	NA	MODIFIER	NA	NA	33	33	0	31	17	13	41.94
Chewer	IOB_10	chr10	97115906	C	T	SORBS1	MISSENSE	ENSG00000095637:[ENST00000607232(c.2386G>A,Ggc>Agc):ENSP00000475901(p.Gly796Ser)]	NA	NA	NA	NA	NA	NA	NA	NA	NA	NA	MODIFIER	NA	NA	158	156	1	163	114	49	30.06
Chewer	IOB_10	chr10	99220606	T	A	MMS19	NCT	ENSG00000155229:[ENST00000480108(n.460+58A>T)]	NA	NA	NA	NA	NA	NA	NA	NA	NA	NA	MODIFIER	NA	NA	105	105	0	77	44	33	42.86
Chewer	IOB_10	chr10	99371339	C	T	HOGA1	MISSENSE	ENSG00000241935:[ENST00000370647(c.418C>T,Cgc>Tgc):ENSP00000359681(p.Arg140Cys)]	NA	NA	NA	1.65E-05	6.06E-05	NA	NA	D	PrD	NA	MODERATE	D	1	263	263	0	301	214	83	27.57
Chewer	IOB_10	chr10	99371339	C	T	PI4K2A	INTRONIC	ENSG00000249967:[ENST00000370649(c.345+9592C>T)]	NA	NA	NA	1.65E-05	6.06E-05	NA	NA	D	PrD	NA	MODERATE	D	1	263	263	0	301	214	83	27.57
Chewer	IOB_10	chr10	102789979	C	T	PDZD7	5UTR	ENSG00000186862:[ENST00000370215(c.-3G>A)]	NA	NA	chr10*102789979*102789979*C*G*rs762699492*NA*NA*NA	NA	NA	NA	NA	NA	NA	NA	MODIFIER	NA	NA	137	137	0	183	177	6	3.28
Chewer	IOB_10	chr10	104416663	C	T	TRIM8	MISSENSE	ENSG00000171206:[ENST00000302424(c.1208C>T,gCg>gTg):ENSP00000302120(p.Ala403Val)]	NA	NA	NA	NA	NA	NA	NA	T_lc	BN	NA	MODERATE	NA	NA	220	219	1	227	220	6	2.64
Chewer	IOB_10	chr10	105906069	G	A	WDR96	SILENT	ENSG00000197748:[ENST00000357060(c.3807C>T,gaC>gaT):ENSP00000349568(p.Asp1269(=))]	NA	NA	NA	NA	NA	NA	NA	NA	NA	NA	LOW	NA	NA	192	192	0	155	119	36	23.23
Chewer	IOB_10	chr11	534289	C	T	HRAS	MISSENSE	ENSG00000174775:[ENST00000311189(c.34G>A,Ggc>Agc):ENSP00000309845(p.Gly12Ser)]	NA	chr11*534289*534289*COSM1644659*C*A*1*NS*1|chr11*534289*534289*COSM481*C*A*344*urinary_tract,lung,upper_aerodigestive_tract,thyroid,soft_tissue,skin,NS,central_nervous_system*7,2,5,5,1,9,1,314|chr11*534289*534289*COSM482*C*G*327*thyroid,pituitary,upper_aerodigestive_tract,skin,large_intestine,salivary_gland,central_nervous_system*2,3,2,1,1,4,314|chr11*534289*534289*COSM480*C*T*66*lung,urinary_tract,penis,upper_aerodigestive_tract,thyroid,skin,bone,soft_tissue,large_intestine,cervix,salivary_gland,prostate*2,9,1,26,4,9,1,4,1,[illegible]	chr11*534289*534289*C*A,G,T*rs104894229*NA*NA*NA	NA	NA	NA	NA	NA	NA	NA	MODIFIER	NA	NA	174	174	0	121	57	64	52.89
Chewer	IOB_10	chr11	1248256	C	T	MUC5B	INTRONIC-SS-ACR-PRX	ENSG00000117983:[ENST00000447027(c.462-5C>T)]	NA	NA	NA	6.44E-05	1.58E-04	NA	NA	NA	NA	NA	LOW	NA	NA	61	61	0	80	60	20	25

Patel *et al.* , 2021. Whole-exome sequencing analysis of oral squamous cell carcinoma delineated by tobacco usage habits
Supplementary Table 4. List of somatic single nucleotide variants identified using whole exome sequencing of OSCC patients

Cohort	Sample	Chromosome	Genomic position	Reference allele	Alternate allele	Genesymbol	Variant type	Variant description	OncoMD	COSMIC	dbSNP	ExAC	ExAC_SAS	1000G	1000G_SAS	SIFT	Polyphen2	CONDEL	VEP	LRT	PhastCon7Way	Total reads in Normal	Reference read support in Normal	Alternative read support in Normal	Total reads in Tumor	Reference read support in Tumor	Alternate read support in Tumor	Alternate allele frequency in Tumor
Chewer	IOB_10	chr11	1258143	C	G	MUC5B	NCT	ENSG00000117983:[ENST00000525715(n.3116-12C>G)]	NA	NA	NA	NA	NA	NA	NA	NA	NA	NA	MODIFIER	NA	NA	257	254	2	247	240	7	2.83
Chewer	IOB_10	chr11	3243123	C	T	MRGPRG-AS1	EXONIC-NC	ENSG00000236301:[ENST00000434798(n.552C>T)]	NA	NA	chr11*3243123*3243123*C*G*rs1051400978*NA*NA*NA	NA	NA	NA	NA	NA	NA	NA	MODIFIER	NA	NA	296	294	0	356	346	7	1.97
Chewer	IOB_10	chr11	3383117	T	G	ZNF195	MISSENSE-SS-PRX	ENSG00000005801:[ENST00000526598(n.289A>C)]	NA	NA	NA	NA	NA	NA	NA	NA	NA	NA	MODIFIER	NA	NA	28	28	0	28	19	8	28.57
Chewer	IOB_10	chr11	17809851	T	C	SERGEF	SILENT	ENSG00000129158:[ENST00000529151(c.749A>G,ggA>ggG):ENSP00000436111(p.Gly250(=))]	NA	NA	NA	NA	NA	NA	NA	NA	NA	NA	MODIFIER	NA	NA	155	154	0	160	152	5	3.12
Chewer	IOB_10	chr11	19173814	G	A	ZDHHC13	MISSENSE	ENSG00000177054:[ENST00000399351(c.304G>A,Gca>Aca):ENSP00000382288(p.Ala102Thr)]	NA	NA	NA	NA	NA	NA	NA	D	PrD	NA	MODERATE	N	1	231	230	0	262	182	80	30.53
Chewer	IOB_10	chr11	61563149	G	A	FADS2	INTRONIC	ENSG00000134824:[ENST00000574708(c.-55+2649G>A)]	NA	NA	NA	NA	NA	NA	NA	T	PoD	NA	MODERATE	NA	NA	109	109	0	129	90	39	30.23
Chewer	IOB_10	chr11	61563149	G	A	FEN1	MISSENSE	ENSG00000168496:[ENST00000305885(c.316G>A,Gag>Aag):ENSP00000305480(p.Glu106Lys)]	NA	NA	NA	NA	NA	NA	NA	T	PoD	NA	MODERATE	NA	NA	109	109	0	129	90	39	30.23
Chewer	IOB_10	chr11	64072097	C	A	RP11-783K16.10	EXONIC-NC	ENSG00000257069:[ENST00000539086(n.2589C>A)]	NA	NA	NA	NA	NA	NA	NA	T	BN	NA	MODERATE	NA	NA	112	111	0	94	69	25	26.6
Chewer	IOB_10	chr11	64072097	C	A	TEX40	MISSENSE	ENSG00000219435:[ENST00000539943(c.456C>A,agC>agA):ENSP00000443917(p.Ser152Arg)]	NA	NA	NA	NA	NA	NA	NA	T	BN	NA	MODERATE	NA	NA	112	111	0	94	69	25	26.6
Chewer	IOB_10	chr11	64072102	G	C	RP11-783K16.10	EXONIC-NC	ENSG00000257069:[ENST00000539086(n.2594G>C)]	NA	NA	NA	NA	NA	NA	NA	NA	NA	NA	MODIFIER	NA	NA	114	113	0	93	69	24	25.81
Chewer	IOB_10	chr11	64072102	G	C	TEX40	MISSENSE	ENSG00000219435:[ENST00000539943(c.461G>C,aGg>aCg):ENSP00000443917(p.Arg154Thr)]	NA	NA	NA	NA	NA	NA	NA	NA	NA	NA	MODIFIER	NA	NA	114	113	0	93	69	24	25.81
Chewer	IOB_10	chr11	64665293	C	A	ATG2A	INTRONIC	23130:[XM_005273851.1(c.4781+18G>T)]	NA	NA	chr11*64665293*64665293*C*T*rs556046223*0*0.9996*0.0003994	NA	NA	NA	NA	NA	NA	NA	MODIFIER	NA	NA	212	212	0	192	185	6	3.12
Chewer	IOB_10	chr11	64950568	G	T	CAPN1	NCT	ENSG00000014216:[ENST00000527189(n.351+129G>T)]	NA	NA	NA	NA	NA	NA	NA	NA	NA	NA	MODIFIER	NA	NA	253	252	1	236	228	6	2.54
Chewer	IOB_10	chr11	65305576	G	A	SCYL1	MISSENSE	ENSG00000142186:[ENST00000420247(c.2119G>A,Gag>Aag):ENSP00000408192(p.Glu707Lys)]	NA	NA	NA	NA	NA	NA	NA	T	BN	NA	MODERATE	N	1	201	201	0	212	153	59	27.83
Chewer	IOB_10	chr11	65305817	G	A	SCYL1	MISSENSE	ENSG00000142186:[ENST00000524944(c.2324G>A,gGc>gAc):ENSP00000432175(p.Gly775Asp)]	NA	NA	NA	NA	NA	NA	NA	NA	NA	NA	MODIFIER	NA	NA	135	132	0	139	133	6	4.32
Chewer	IOB_10	chr11	66333613	C	A	CTSF	SILENT	ENSG00000174080:[ENST00000310325(c.747G>T,ctG>ctT):ENSP00000310832(p.Leu249(=))]	NA	NA	NA	NA	NA	NA	NA	NA	NA	NA	LOW	NA	NA	210	208	0	238	232	6	2.52
Chewer	IOB_10	chr11	71164328	G	A	NADSYN1	EXONIC-NC	ENSG00000172890:[ENST00000527538(n.42G>A)]	NA	NA	NA	NA	NA	NA	NA	NA	NA	NA	MODIFIER	NA	NA	88	88	0	102	79	22	21.57
Chewer	IOB_10	chr11	108128298	C	A	ATM	MISSENSE	ENSG00000149311:[ENST00000278616(c.2341C>A,Cag>Aag):ENSP00000278616(p.Gln781Lys)]	NA	NA	NA	NA	NA	NA	NA	NA	NA	NA	MODERATE	NA	0.761	75	75	0	58	53	5	8.62
Chewer	IOB_10	chr11	119001593	C	A	HINFP	MISSENSE	ENSG00000172273:[ENST00000350777(c.340C>A,Ctg>Atg):ENSP00000318085(p.Leu114Met)]	NA	NA	NA	NA	NA	NA	NA	NA	NA	NA	MODIFIER	NA	NA	246	245	1	195	190	5	2.56
Chewer	IOB_10	chr11	124006918	C	A	VWA5A	MISSENSE	ENSG00000110002:[ENST00000392748(c.1442C>A,gCt>gAt):ENSP00000376504(p.Ala481Asp)]	NA	NA	chr11*124006918*124006918*C*T*rs768122678*NA*NA*NA	NA	NA	NA	NA	NA	NA	NA	MODIFIER	NA	NA	142	141	1	174	118	54	31.03
Chewer	IOB_10	chr11	124564256	G	T	SIAE	EXONIC-NC	ENSG00000110013:[ENST00000525730(n.261C>A)]	NA	NA	NA	NA	NA	NA	NA	T	BN	NA	MODERATE	N	0.695	31	31	0	52	39	13	25
Chewer	IOB_10	chr11	124564256	G	T	SPA17	MISSENSE	ENSG00000064199:[ENST00000227135(c.370G>T,Gcc>Tcc):ENSP00000227135(p.Ala124Ser)]	NA	NA	NA	NA	NA	NA	NA	T	BN	NA	MODERATE	N	0.695	31	31	0	52	39	13	25
Chewer	IOB_10	chr11	133790994	C	T	IGSF9B	MISSENSE	ENSG00000080854:[ENST00000321016(c.2626G>A,Ggc>Agc):ENSP00000317980(p.Gly876Ser)]	NA	NA	chr11*133790994*133790994*C*G*rs745618211*NA*NA*NA	NA	NA	NA	NA	D	PrD	NA	MODERATE	D	1	199	199	0	200	153	46	23
Chewer	IOB_10	chr11	134238677	C	A	GLB1L2	INTRONIC-SS-DNR	ENSG00000149328:[ENST00000529077(n.3187+2C>A)]	NA	NA	NA	NA	NA	NA	NA	NA	NA	NA	HIGH	NA	NA	77	77	0	91	66	25	27.47
Chewer	IOB_10	chr12	10875417	C	T	YBX3	NCT	ENSG00000060138:[ENST00000540447(n.62+32G>A)]	NA	NA	NA	NA	NA	NA	NA	NA	NA	NA	MODIFIER	NA	NA	227	225	0	368	239	129	35.05
Chewer	IOB_10	chr12	14976415	C	A	C12orf60	SILENT	ENSG00000182993:[ENST00000330828(c.546C>A,acC>acA):ENSP00000331691(p.Thr182(=))]	NA	NA	NA	NA	NA	NA	NA	NA	NA	NA	LOW	NA	NA	162	161	1	151	142	5	3.31
Chewer	IOB_10	chr12	40076548	A	G	C12orf40	SILENT	ENSG00000180116:[ENST00000398716(c.591A>G,ggA>ggG):ENSP00000381701(p.Gly197(=))]	NA	NA	NA	NA	NA	NA	NA	NA	NA	NA	MODIFIER	NA	NA	110	110	0	113	86	27	23.89
Chewer	IOB_10	chr12	48388220	G	A	COL2A1	MISSENSE	ENSG00000139219:[ENST00000337299(c.596C>T,cCg>cTg):ENSP00000338213(p.Pro199Leu)]	NA	NA	5.99E-04	9.18E-05	1.85E-04	5.99E-04	2.00E-03	T	BN	NA	MODERATE	N	0.1	63	63	0	76	58	18	23.68
Chewer	IOB_10	chr12	51079611	C	T	DIP2B	INTRONIC-SS-ACR-PRX	ENSG00000066084:[ENST00000301180(c.1318-5C>T)]	NA	NA	chr12*51079611*51079611*C*G*rs544169547*0*0.9998*0.0001997	NA	NA	NA	NA	NA	NA	NA	LOW	NA	NA	142	141	0	133	107	26	19.55
Chewer	IOB_10	chr12	52452720	C	A	NR4A1	MISSENSE	ENSG00000123358:[ENST00000243050(c.1789C>A,Ccc>Acc):ENSP00000243050(p.Pro597Thr)]	NA	NA	NA	NA	NA	NA	NA	D	BN	NA	MODERATE	D	1	176	175	0	193	186	5	2.59
Chewer	IOB_10	chr12	52452720	C	A	RP11-1100L3.8	NCT	ENSG00000259884:[ENST00000564531(n.381+16G>T)]	NA	NA	NA	NA	NA	NA	NA	D	BN	NA	MODERATE	D	1	176	175	0	193	186	5	2.59
Chewer	IOB_10	chr12	56708712	C	A	CNPY2	MISSENSE	ENSG00000257727:[ENST00000273308(c.127G>T,Gcc>Tcc):ENSP00000273308(p.Ala43Ser)]	NA	NA	NA	NA	NA	NA	NA	NA	NA	NA	MODIFIER	NA	NA	84	84	0	69	48	21	30.43
Chewer	IOB_10	chr12	56708712	C	A	RP11-977G19.10	MISSENSE	ENSG00000144785:[ENST00000547423(c.127G>T,Gcc>Tcc):ENSP00000446506(p.Ala43Ser)]	NA	NA	NA	NA	NA	NA	NA	NA	NA	NA	MODIFIER	NA	NA	84	84	0	69	48	21	30.43
Chewer	IOB_10	chr12	62902163	C	T	MON2	MISSENSE	ENSG00000061987:[ENST00000280379(c.887C>T,tCa>tTa):ENSP00000280379(p.Ser296Leu)]	NA	NA	NA	NA	NA	NA	NA	T	BN	NA	MODERATE	N	1	425	425	0	375	279	96	25.6
Chewer	IOB_10	chr12	65462534	C	T	WIF1	INTRONIC-SS-DNR-PRX	ENSG00000156076:[ENST00000286574(c.538+10G>A)]	NA	NA	NA	1.66E-05	0.00E+00	NA	NA	NA	NA	NA	MODIFIER	NA	NA	165	165	0	213	139	71	33.33
Chewer	IOB_10	chr12	69042539	G	A	RAP1B	MISSENSE	ENSG00000127314:[ENST00000250559(c.35G>A,gGa>gAa):ENSP00000250559(p.Gly12Glu)]	NA	chr12*69042539*69042539*COSM1317973*G*A*1*haematopoietic_and_lymphoid_tissue*1	NA	NA	NA	NA	NA	NA	NA	NA	MODIFIER	NA	NA	196	193	0	223	157	61	27.35
Chewer	IOB_10	chr12	72300797	T	G	TBC1D15	NCT-SS-PRX	ENSG00000121749:[ENST00000479971(n.96-5T>G)]	NA	NA	NA	NA	NA	NA	NA	NA	NA	NA	LOW	NA	NA	69	69	0	95	71	23	24.21
Chewer	IOB_10	chr12	81007460	C	A	PTPRQ	MISSENSE	ENSG00000139304:[ENST00000266688(c.4996C>A,Cct>Act):ENSP00000266688(p.Pro1666Thr)]	NA	NA	NA	NA	NA	NA	NA	D	PrD	NA	MODERATE	NA	NA	124	124	0	137	131	5	3.65
Chewer	IOB_10	chr12	96915719	C	T	C12orf55	NONSENSE	ENSG00000188596:[ENST00000553778(c.883C>T,Cga>Tga):ENSP00000452066(p.Arg295Ter)]	NA	NA	NA	NA	NA	NA	NA	NA	NA	NA	HIGH	NA	0.076	143	143	0	135	90	45	33.33
Chewer	IOB_10	chr12	104682693	C	T	TXNRD1	MISSENSE	ENSG00000198431:[ENST00000378070(c.136C>T,Ctt>Ttt):ENSP00000367310(p.Leu46Phe)]	NA	NA	NA	NA	NA	NA	NA	NA	NA	NA	MODIFIER	NA	NA	188	185	0	216	161	55	25.46
Chewer	IOB_10	chr12	105455520	C	A	ALDH1L2	MISSENSE	ENSG00000136010:[ENST00000258494(c.932G>T,aGa>aTa):ENSP00000258494(p.Arg311Ile)]	NA	NA	NA	NA	NA	NA	NA	NA	NA	NA	MODIFIER	NA	NA	174	174	0	223	215	6	2.69
Chewer	IOB_10	chr12	110221532	T	C	TRPV4	MISSENSE	ENSG00000111199:[ENST00000261740(c.2510A>G,aAc>aGc):ENSP00000261740(p.Asn837Ser)]	NA	NA	NA	NA	NA	NA	NA	T	BN	NA	MODERATE	N	0.055	227	226	0	205	138	63	30.73
Chewer	IOB_10	chr12	111086960	C	A	HVCN1	INTRONIC	ENSG00000122986:[ENST00000548312(c.756+1013G>T)]	NA	NA	NA	NA	NA	NA	NA	NA	NA	NA	MODIFIER	NA	NA	141	140	0	129	123	5	3.88
Chewer	IOB_10	chr12	111086960	C	A	TCTN1	3UTR	ENSG00000204852:[ENST00000551590(c.*114C>A)]	NA	NA	NA	NA	NA	NA	NA	NA	NA	NA	MODIFIER	NA	NA	141	140	0	129	123	5	3.88
Chewer	IOB_10	chr12	117448094	A	C	FBXW8	INTRONIC	26259:[XM_005253866.1(c.1240-13858A>C)]	NA	NA	chr12*117448094*117448094*A*G*rs757453330*NA*NA*NA	NA	NA	NA	NA	NA	NA	NA	MODIFIER	NA	NA	135	135	0	136	94	42	30.88
Chewer	IOB_10	chr12	122667655	C	T	LRRC43	NCT	ENSG00000158113:[ENST00000537113(n.71+36C>T)]	NA	NA	NA	NA	NA	NA	NA	NA	NA	NA	MODIFIER	NA	NA	152	152	0	159	120	39	24.53
Chewer	IOB_10	chr12	124821459	G	A	NCOR2	SILENT	ENSG00000196498:[ENST00000397355(c.5928C>T,gtC>gtT):ENSP00000380513(p.Val1976(=))]	NA	NA	NA	NA	NA	NA	NA	NA	NA	NA	LOW	NA	NA	54	54	0	45	34	10	22.22
Chewer	IOB_10	chr13	21750538	G	A	MRPL57	5UTR	78988:[NM_024026.4(c.-324G>A)]	NA	NA	NA	3.19E-01	2.53E-01	NA	NA	NA	NA	NA	HIGH	NA	NA	64	61	3	76	53	23	30.26
Chewer	IOB_10	chr13	21750538	G	A	SKA3	NONSENSE	ENSG00000165480:[ENST00000298260(c.79C>T,Cga>Tga):ENSP00000298260(p.Arg27Ter)]	NA	NA	NA	3.19E-01	2.53E-01	NA	NA	NA	NA	NA	HIGH	NA	NA	64	61	3	76	53	23	30.26
Chewer	IOB_10	chr13	29292075	C	T	SLC46A3	MISSENSE	ENSG00000139508:[ENST00000266943(c.61G>A,Ggt>Agt):ENSP00000266943(p.Gly21Ser)]	NA	NA	NA	NA	NA	NA	NA	T	BN	NA	MODERATE	N	0	258	258	0	204	144	59	28.92
Chewer	IOB_10	chr13	33315188	G	T	PDS5B	INTRONIC	ENSG00000083642:[ENST00000315596(c.2407-30G>T)]	NA	NA	NA	4.17E-02	9.50E-02	NA	NA	NA	NA	NA	MODIFIER	NA	NA	50	45	0	48	41	5	10.42
Chewer	IOB_10	chr13	35619083	G	A	NBEA	INTRONIC-SS-ACR	26960:[XM_005266351.1(c.404-1G>A)]	NA	NA	NA	NA	NA	NA	NA	NA	NA	NA	HIGH	NA	NA	149	148	0	181	126	55	30.39
Chewer	IOB_10	chr13	58207227	G	A	PCDH17	MISSENSE	ENSG00000118946:[ENST00000377918(c.547G>A,Gtt>Att):ENSP00000367151(p.Val183Ile)]	NA	NA	NA	NA	NA	NA	NA	T	BN	NA	MODERATE	NA	NA	313	313	0	478	339	136	28.45
Chewer	IOB_10	chr14	20228995	C	T	OR11K2P	NCT	ENSG00000258438:[ENST00000593630(n.15+1035G>A)]	NA	NA	3.00E-02	NA	NA	3.00E-02	3.58E-02	NA	NA	NA	MODIFIER	NA	NA	52	45	7	59	38	20	33.9
Chewer	IOB_10	chr14	20228995	C	T	OR4N2	INTRONIC	ENSG00000176294:[ENST00000557414(c.-303-1052C>T)]	NA	NA	3.00E-02	NA	NA	3.00E-02	3.58E-02	NA	NA	NA	MODIFIER	NA	NA	52	45	7	59	38	20	33.9
Chewer	IOB_10	chr14	21868404	C	A	CHD8	MISSENSE	ENSG00000100888:[ENST00000555935(c.2333G>T,Gat>Tat):ENSP00000451442(p.Asp779Tyr)]	NA	NA	NA	NA	NA	NA	NA	D	PoD	NA	MODERATE	D	1	129	128	0	184	178	6	3.26
Chewer	IOB_10	chr14	22322310	T	C	NA	INTERGENIC	NA	NA	NA	chr14*22322309*22322310*CT*C*rs762604540*NA*NA*NA	NA	NA	NA	NA	NA	NA	NA	MODIFIER	NA	NA	76	75	0	159	93	66	41.51
Chewer	IOB_10	chr14	23873621	G	A	MYH6	NCT	ENSG00000197616:[ENST00000557461(n.697-24C>T)]	NA	NA	NA	NA	NA	NA	NA	NA	NA	NA	MODIFIER	NA	NA	168	168	0	276	138	137	49.64
Chewer	IOB_10	chr14	24546936	G	A	CPNE6	SILENT	ENSG00000100884:[ENST00000537691(c.1836G>A,ccG>ccA):ENSP00000440077(p.Pro612(=))]	NA	NA	NA	9.73E-06	0.00E+00	NA	NA	NA	NA	NA	MODIFIER	NA	NA	186	185	0	233	227	6	2.58
Chewer	IOB_10	chr14	24615787	C	T	PSME2	MISSENSE	ENSG00000100911:[ENST00000216802(c.4G>A,Gcc>Acc):ENSP00000216802(p.Ala2Thr)]	NA	NA	NA	NA	NA	NA	NA	T_lc	BN	NA	MODERATE	D	1	159	159	0	215	209	6	2.79
Chewer	IOB_10	chr14	31849709	C	A	HEATR5A	MISSENSE	ENSG00000129493:[ENST00000389961(c.1654G>T,Gct>Tct):ENSP00000374611(p.Ala552Ser)]	NA	NA	NA	NA	NA	NA	NA	NA	NA	NA	MODERATE	NA	1	216	215	0	260	252	6	2.31
Chewer	IOB_10	chr14	31849709	C	A	RP11-176H8.1	3UTR	ENSG00000203546:[ENST00000549185(c.*1768G>T)]	NA	NA	NA	NA	NA	NA	NA	NA	NA	NA	MODERATE	NA	1	216	215	0	260	252	6	2.31
Chewer	IOB_10	chr14	38194200	A	T	TTC6	MISSENSE	ENSG00000139865:[ENST00000553443(c.1811A>T,aAa>aTa):ENSP00000451131(p.Lys604Ile)]	NA	NA	NA	NA	NA	NA	NA	NA	NA	NA	MODIFIER	NA	NA	73	73	0	86	70	16	18.6
Chewer	IOB_10	chr14	52171537	C	T	FRMD6	NONSENSE	ENSG00000139926:[ENST00000344768(c.442C>T,Cga>Tga):ENSP00000343899(p.Arg148Ter)]	NA	NA	chr14*52171537*52171537*C*G*rs771992156*NA*NA*NA	NA	NA	NA	NA	NA	NA	NA	MODIFIER	NA	NA	101	100	0	145	72	72	49.66
Chewer	IOB_10	chr14	61115488	C	A	SIX1	SILENT	ENSG00000126778:[ENST00000247182(c.420G>T,cgG>cgT):ENSP00000247182(p.Arg140(=))]	NA	NA	NA	NA	NA	NA	NA	NA	NA	NA	MODIFIER	NA	NA	269	269	0	553	543	7	1.27
Chewer	IOB_10	chr14	63447927	G	A	KCNH5	MISSENSE	ENSG00000140015:[ENST00000394964(c.431C>T,aCg>aTg):ENSP00000378415(p.Thr144Met)]	NA	chr14*63447927*63447927*COSM1370451*G*A*3*ovary,NS,large_intestine*1,1,1|chr14*63447927*63447927*COSM343214*G*A*4*ovary,NS,lung,large_intestine*1,1,[illegible]	NA	NA	NA	NA	NA	D	PrD	NA	MODERATE	D	1	88	88	0	137	73	64	46.72
Chewer	IOB_10	chr14	71444285	G	A	PCNX	MISSENSE	ENSG00000100731:[ENST00000238570(c.1231G>A,Gag>Aag):ENSP00000238570(p.Glu411Lys)]	NA	NA	NA	NA	NA	NA	NA	NA	NA	NA	MODERATE	NA	1	85	85	0	110	89	21	19.09
Chewer	IOB_10	chr14	74206657	C	T	ELMSAN1	MISSENSE	ENSG00000156030:[ENST00000286523(c.55G>A,Ggg>Agg):ENSP00000286523(p.Gly19Arg)]	NA	NA	2.00E-04	2.74E-05	0.00E+00	2.00E-04	0.00E+00	T_lc	BN	NA	MODERATE	NA	NA	147	145	1	223	120	100	44.84
Chewer	IOB_10	chr14	74404346	G	A	FAM161B	NONSENSE	ENSG00000156050:[ENST00000286544(c.1813C>T,Cga>Tga):ENSP00000286544(p.Arg605Ter)]	NA	NA	NA	8.24E-06	0.00E+00	NA	NA	NA	NA	NA	HIGH	NA	0.988	230	230	0	262	119	142	54.2
Chewer	IOB_10	chr14	74404346	G	A	RP5-1021I20.5	EXONIC-NC	ENSG00000258891:[ENST00000555916(n.226C>T)]	NA	NA	NA	8.24E-06	0.00E+00	NA	NA	NA	NA	NA	HIGH	NA	0.988	230	230	0	262	119	142	54.2

Patel *et al.*, 2021. Whole-exome sequencing analysis of oral squamous cell carcinoma delineated by tobacco usage habits
Supplementary Table 4. List of somatic single nucleotide variants identified using whole exome sequencing of OSCC patients

Cohort	Sample	Chromosome	Genomic position	Reference allele	Alternate allele	Genesymbol	Variant type	Variant description	OncoMD	COSMIC	dbSNP	ExAC	ExAC_SAS	1000G	1000G_SAS	SIFT	Polyphen2	CONDEL	VEP	LRT	PhastCon7Way	Total reads in Normal	Reference read support in Normal	Alternative read support in Normal	Total reads in Tumor	Reference read support in Tumor	Alternate read support in Tumor	Alternate allele frequency in Tumor
Chewer	IOB_10	chr14	93412806	C	G	ITPK1	SILENT	ENSG00000100605:[ENST00000555495(c.414G>C,ccG>ccC):ENSP00000451893(p.Pro138(=))]	NA	NA	chr14*93412806*93412806*C*T*rs148765069*1*0.9986*0.001398	NA	NA	NA	NA	NA	NA	NA	MODIFIER	NA	NA	180	177	0	244	202	41	16.8
Chewer	IOB_10	chr14	95081145	C	T	RP11-986E7.7	3UTR	ENSG00000273259:[ENST00000553947(c.*1193C>T)]	NA	NA	NA	1.65E-05	1.21E-04	NA	NA	NA	NA	NA	MODIFIER	NA	NA	117	116	0	179	142	36	20.11
Chewer	IOB_10	chr14	95081145	C	T	SERPINA3	SILENT	ENSG00000196136:[ENST00000393078(c.367C>T,Ctg>Ttg):ENSP00000376793(p.Leu123(=))]	NA	NA	NA	1.65E-05	1.21E-04	NA	NA	NA	NA	NA	MODIFIER	NA	NA	117	116	0	179	142	36	20.11
Chewer	IOB_10	chr14	100191724	G	A	CYP46A1	INTRONIC-SS-ACR-PRX	ENSG00000036530:[ENST00000554917(c.*299-4G>A)]	NA	chr14*100191724*100191724*COSM280271*G*A*1*large_intestine*1	NA	2.48E-05	0.00E+00	NA	NA	NA	NA	NA	LOW	NA	NA	69	69	0	131	102	29	22.14
Chewer	IOB_10	chr14	105613901	C	T	JAG2	INTRONIC	ENSG00000184916:[ENST00000347004(c.2252-37G>A)]	NA	NA	NA	NA	NA	NA	NA	NA	NA	NA	MODIFIER	NA	NA	90	90	0	114	87	26	22.81
Chewer	IOB_10	chr15	22466228	C	T	IGHV1OR15-3	EXONIC-NC	ENSG00000271288:[ENST00000564926(n.181G>A)]	NA	NA	NA	NA	NA	NA	NA	NA	NA	NA	MODIFIER	NA	NA	521	519	0	581	452	126	21.69
Chewer	IOB_10	chr15	33927949	G	A	RYR3	MISSENSE	ENSG00000198838:[ENST00000389232(c.3310G>A,Gcg>Acg):ENSP00000373884(p.Ala1104Thr)]	NA	NA	NA	NA	NA	NA	NA	D	PoD	NA	MODERATE	D	1	72	72	0	67	44	23	34.33
Chewer	IOB_10	chr15	33927956	C	A	RYR3	MISSENSE	ENSG00000198838:[ENST00000389232(c.3317C>A,cCa>cAa):ENSP00000373884(p.Pro1106Gln)]	NA	NA	NA	NA	NA	NA	NA	D	PrD	NA	MODERATE	D	1	67	67	0	65	44	21	32.31
Chewer	IOB_10	chr15	41476320	T	C	EXD1	MISSENSE	ENSG00000178997:[ENST00000458580(c.1528A>G,Atg>Gtg):ENSP00000415056(p.Met510Val)]	NA	NA	NA	NA	NA	NA	NA	T_lc	BN	NA	MODERATE	N	0.069	161	161	0	113	81	32	28.32
Chewer	IOB_10	chr15	45428614	G	A	DUOX1	INTRONIC-SS-DNR-PRX	ENSG00000137857:[ENST00000321429(c.925+6G>A)]	NA	NA	NA	NA	NA	NA	NA	NA	NA	NA	LOW	NA	NA	51	51	0	41	23	17	41.46
Chewer	IOB_10	chr15	55474500	G	T	RSL24D1	INTRONIC	ENSG00000137876:[ENST00000260443(c.419-77C>A)]	NA	NA	NA	NA	NA	NA	NA	NA	NA	NA	MODIFIER	NA	NA	22	22	0	44	39	5	11.36
Chewer	IOB_10	chr15	77057796	C	A	SCAPER	INTRONIC-SS-ACR	ENSG00000140386:[ENST00000565507(c.*1335-1G>T)]	NA	NA	NA	NA	NA	NA	NA	NA	NA	NA	HIGH	NA	NA	172	172	0	221	212	5	2.26
Chewer	IOB_10	chr15	94913348	C	T	MCTP2	SILENT	ENSG00000140563:[ENST00000331706(c.285C>T,gaC>gaT):ENSP00000329646(p.Asp95(=))]	NA	NA	NA	NA	NA	NA	NA	NA	NA	NA	MODIFIER	NA	NA	201	201	0	254	182	71	27.95
Chewer	IOB_10	chr16	324151	C	T	ARHGDIG	INTRONIC	ENSG00000242173:[ENST00000412541(c.169+5147C>T)]	NA	NA	2.00E-04	3.48E-05	0.00E+00	2.00E-04	0.00E+00	NA	NA	NA	MODIFIER	NA	NA	103	103	0	87	72	15	17.24
Chewer	IOB_10	chr16	324151	C	T	RGS11	INTRONIC	ENSG00000076344:[ENST00000359740(c.338-50G>A)]	NA	NA	2.00E-04	3.48E-05	0.00E+00	2.00E-04	0.00E+00	NA	NA	NA	MODIFIER	NA	NA	103	103	0	87	72	15	17.24
Chewer	IOB_10	chr16	2487253	G	A	CCNF	NONSENSE	ENSG00000162063:[ENST00000397066(c.470G>A,tGg>tAg):ENSP00000380256(p.Trp157Ter)]	NA	NA	NA	NA	NA	NA	NA	NA	NA	NA	MODIFIER	NA	NA	45	44	0	49	37	12	24.49
Chewer	IOB_10	chr16	4039122	G	A	ADCY9	INTRONIC	115:[XM_005255079.1(c.2265-25C>T)]	NA	NA	NA	2.48E-05	0.00E+00	NA	NA	NA	NA	NA	MODIFIER	NA	NA	160	160	0	142	101	39	27.46
Chewer	IOB_10	chr16	7102174	C	A	RBFOX1	NCT	ENSG00000078328:[ENST00000547427(n.411+75C>A)]	NA	NA	chr16*7102174*7102174*C*G*rs142266105*0*0.9996*0.0003994	NA	NA	NA	NA	NA	NA	NA	MODIFIER	NA	NA	107	107	0	142	136	6	4.23
Chewer	IOB_10	chr16	19126462	C	T	CTD-2349B8.1	INTRONIC	ENSG00000261427:[ENST00000564808(c.*104+205C>T)]	NA	NA	NA	NA	NA	NA	NA	NA	NA	NA	MODIFIER	NA	NA	206	206	0	217	143	73	33.64
Chewer	IOB_10	chr16	19126462	C	T	ITPRIPL2	MISSENSE	ENSG00000205730:[ENST00000381440(c.679C>T,Cgg>Tgg):ENSP00000370849(p.Arg227Trp)]	NA	NA	NA	NA	NA	NA	NA	NA	NA	NA	MODIFIER	NA	NA	206	206	0	217	143	73	33.64
Chewer	IOB_10	chr16	20975841	C	A	DNAH3	MISSENSE	ENSG00000158486:[ENST00000261383(c.9365G>T,gGc>gTc):ENSP00000261383(p.Gly3122Val)]	NA	NA	NA	NA	NA	NA	NA	NA	NA	NA	MODIFIER	NA	NA	229	229	0	215	206	7	3.26
Chewer	IOB_10	chr16	48149458	G	A	ABCC12	MISSENSE	ENSG00000140798:[ENST00000416054(c.1784C>T,cCg>cTg):ENSP00000413046(p.Pro595Leu)]	NA	NA	NA	1.65E-05	0.00E+00	NA	NA	NA	NA	NA	MODIFIER	NA	NA	219	217	0	255	184	70	27.45
Chewer	IOB_10	chr16	50829379	C	T	CYLD	INTRONIC	ENSG00000083799:[ENST00000311559(c.2687-856C>T)]	NA	NA	NA	NA	NA	NA	NA	NA	NA	NA	MODIFIER	NA	NA	73	73	0	87	81	6	6.9
Chewer	IOB_10	chr16	50829379	C	T	RP11-327F22.4	NCT	ENSG00000260616:[ENST00000564510(n.456+6655G>A)]	NA	NA	NA	NA	NA	NA	NA	NA	NA	NA	MODIFIER	NA	NA	73	73	0	87	81	6	6.9
Chewer	IOB_10	chr16	58075487	C	T	MMP15	INTRONIC	ENSG00000102996:[ENST00000219271(c.911-34C>T)]	NA	NA	NA	NA	NA	NA	NA	NA	NA	NA	MODIFIER	NA	NA	129	128	1	150	104	45	30
Chewer	IOB_10	chr16	88712949	G	A	CYBA	MISSENSE	ENSG00000051523:[ENST00000568278(c.350C>T,cCg>cTg):ENSP00000455506(p.Pro117Leu)]	NA	NA	NA	NA	NA	NA	NA	NA	NA	NA	MODIFIER	NA	NA	118	116	0	119	78	41	34.45
Chewer	IOB_10	chr16	89351833	T	C	ANKRD11	MISSENSE	ENSG00000167522:[ENST00000301030(c.1117A>G,Aaa>Gaa):ENSP00000301030(p.Lys373Glu)]	NA	NA	NA	NA	NA	NA	NA	NA	NA	NA	MODIFIER	NA	NA	166	165	0	154	107	46	29.87
Chewer	IOB_10	chr17	1579846	G	A	PRPF8	MISSENSE	ENSG00000174231:[ENST00000304992(c.2341C>T,Cgg>Tgg):ENSP00000304350(p.Arg781Trp)]	NA	NA	chr17*1579846*1579846*G*T*rs774774848*NA*NA*NA	NA	NA	NA	NA	D	PrD	NA	MODERATE	NA	NA	140	140	0	105	78	26	24.76
Chewer	IOB_10	chr17	3921115	G	A	ZZEF1	INTRONIC	23140:[XM_005256561.1(c.7644+15C>T)]	NA	NA	NA	NA	NA	NA	NA	NA	NA	NA	MODIFIER	NA	NA	157	157	0	159	112	47	29.56
Chewer	IOB_10	chr17	17163811	A	C	COPS3	INTRONIC	8533:[NM_001316358.1(c.448-23T>G)]	NA	NA	NA	NA	NA	NA	NA	NA	NA	NA	MODIFIER	NA	NA	96	95	0	90	62	28	31.11
Chewer	IOB_10	chr17	28952432	G	C	AC005562.1	NCT	ENSG00000214719:[ENST00000578265(n.287+14472G>C)]	NA	NA	NA	NA	NA	NA	NA	NA	NA	NA	MODIFIER	NA	NA	141	140	0	158	105	53	33.54
Chewer	IOB_10	chr17	28952432	G	C	LOC107133515	NCT	107133515:[NR_015341.2(n.659-6341G>C)]	NA	NA	NA	NA	NA	NA	NA	NA	NA	NA	MODIFIER	NA	NA	141	140	0	158	105	53	33.54
Chewer	IOB_10	chr17	28952432	G	C	SH3GL1P2	EXONIC-NC	ENSG00000264943:[ENST00000582245(n.1002G>C)]	NA	NA	NA	NA	NA	NA	NA	NA	NA	NA	MODIFIER	NA	NA	141	140	0	158	105	53	33.54
Chewer	IOB_10	chr17	29899068	C	T	AC003101.1	INTRONIC	ENSG00000228768:[ENST00000412403(c.217-36C>T)]	NA	NA	NA	NA	NA	NA	NA	NA	NA	NA	MODIFIER	NA	NA	114	109	3	113	99	13	11.5
Chewer	IOB_10	chr17	33687373	C	T	SLFN11	MISSENSE	ENSG00000172716:[ENST00000308377(c.1087G>A,Gaa>Aaa):ENSP00000312402(p.Glu363Lys)]	NA	NA	NA	NA	NA	NA	NA	NA	NA	NA	MODIFIER	NA	NA	75	74	0	86	59	26	30.23
Chewer	IOB_10	chr17	37821535	C	T	TCAP	5UTR	ENSG00000173991:[ENST00000309889(c.-78C>T)]	NA	NA	NA	NA	NA	NA	NA	NA	NA	NA	MODIFIER	NA	NA	47	47	0	56	37	19	33.93
Chewer	IOB_10	chr17	41244445	C	T	BRCA1	MISSENSE	ENSG00000012048:[ENST00000309486(c.2215G>A,Gtt>Att):ENSP00000310938(p.Val739Ile)]	NA	NA	NA	NA	NA	NA	NA	NA	NA	NA	MODIFIER	NA	NA	200	200	0	188	127	60	31.91
Chewer	IOB_10	chr17	43107506	T	C	DCAKD	MISSENSE	ENSG00000172992:[ENST00000342350(c.358A>G,Acc>Gcc):ENSP00000341504(p.Thr120Ala)]	NA	NA	NA	NA	NA	NA	NA	D	BN	NA	MODERATE	NA	NA	118	118	0	103	97	5	4.85
Chewer	IOB_10	chr17	47284139	C	A	GNGT2	MISSENSE	ENSG00000167083:[ENST00000300406(c.190G>T,Ggt>Tgt):ENSP00000300406(p.Gly64Cys)]	NA	NA	chr17*47284139*47284139*C*T*rs201646997*0*0.9998*0.0001997	NA	NA	NA	NA	D	PrD	NA	MODERATE	D	1	116	115	0	112	106	5	4.46
Chewer	IOB_10	chr17	58740668	G	T	PPM1D	NONSENSE	ENSG00000170836:[ENST00000305921(c.1573G>T,Gaa>Taa):ENSP00000306682(p.Glu525Ter)]	NA	chr17*58740668*58740668*COSM982224*G*T*4*endometrium,large_intestine*3,1	NA	8.26E-06	0.00E+00	NA	NA	NA	NA	NA	HIGH	NA	1	209	209	0	197	130	66	33.5
Chewer	IOB_10	chr17	61995219	G	C	CSHL1	INTRONIC	ENSG00000204414:[ENST00000392824(c.10+908C>G)]	NA	chr17*61995219*61995219*COSM1303188*G*A*1*urinary_tract*1	2.00E-04	NA	NA	2.00E-04	0.00E+00	NA	NA	NA	LOW	NA	NA	114	114	0	117	112	5	4.27
Chewer	IOB_10	chr17	61995219	G	C	GH1	SILENT	ENSG00000259384:[ENST00000323322(c.357C>G,ctC>ctG):ENSP00000312673(p.Leu119(=))]	NA	chr17*61995219*61995219*COSM1303188*G*A*1*urinary_tract*1	2.00E-04	NA	NA	2.00E-04	0.00E+00	NA	NA	NA	LOW	NA	NA	114	114	0	117	112	5	4.27
Chewer	IOB_10	chr17	73502450	G	A	CASKIN2	MISSENSE	ENSG00000177303:[ENST00000321617(c.662C>T,aCg>aTg):ENSP00000325355(p.Thr221Met)]	NA	NA	NA	3.35E-05	0.00E+00	NA	NA	T	BN	NA	MODERATE	N	1	60	60	0	55	41	14	25.45
Chewer	IOB_10	chr17	74276406	T	C	QRICH2	MISSENSE	ENSG00000129646:[ENST00000262765(c.4090A>G,Aag>Gag):ENSP00000262765(p.Lys1364Glu)]	NA	NA	NA	NA	NA	NA	NA	NA	NA	NA	MODIFIER	NA	NA	185	185	0	158	115	43	27.22
Chewer	IOB_10	chr17	74276914	C	A	QRICH2	MISSENSE-SS-PRX	ENSG00000129646:[ENST00000524722(c.*650G>T)]	NA	NA	NA	NA	NA	NA	NA	T	BN	NA	MODERATE	NA	NA	91	91	0	81	76	5	6.17
Chewer	IOB_10	chr17	78896549	G	A	RPTOR	MISSENSE	ENSG00000141564:[ENST00000576366(c.26G>A,cGc>cAc):ENSP00000460940(p.Arg9His)]	NA	NA	NA	8.02E-05	0.00E+00	NA	NA	T	PoD	NA	MODERATE	D	1	497	496	0	560	409	151	26.96
Chewer	IOB_10	chr17	79898714	G	A	MYADML2	MISSENSE	ENSG00000185105:[ENST00000330655(c.904C>T,Cgc>Tgc):ENSP00000327718(p.Arg302Cys)]	NA	chr17*79898714*79898714*COSM1387366*G*A*1*large_intestine*1	7.99E-04	1.04E-03	0.00E+00	7.99E-04	0.00E+00	NA	NA	NA	MODIFIER	NA	NA	252	251	0	202	133	69	34.16
Chewer	IOB_10	chr17	79898714	G	A	PYCR1	INTRONIC	ENSG00000183010:[ENST00000582198(c.-24+1458C>T)]	NA	chr17*79898714*79898714*COSM1387366*G*A*1*large_intestine*1	7.99E-04	1.04E-03	0.00E+00	7.99E-04	0.00E+00	NA	NA	NA	MODIFIER	NA	NA	252	251	0	202	133	69	34.16
Chewer	IOB_10	chr17	80223678	C	A	CSNK1D	INTRONIC-SS-ACR-PRX	ENSG00000141551:[ENST00000581660(c.*115-6G>T)]	NA	NA	NA	NA	NA	NA	NA	NA	NA	NA	LOW	NA	NA	141	139	1	128	121	6	4.69
Chewer	IOB_10	chr18	11609868	G	A	RP11-677O4.3	EXONIC-NC	ENSG00000236396:[ENST00000588001(n.325G>A)]	NA	NA	9.98E-04	4.78E-04	6.06E-05	9.98E-04	0.00E+00	T	BN	NA	MODERATE	NA	NA	159	158	0	152	112	37	24.34
Chewer	IOB_10	chr18	11609868	G	A	SLC35G4	MISSENSE	646000:[NM_001282300.1(c.274G>A,Gac>Aac):NP_001269229.1(p.Asp92Asn)]	NA	NA	9.98E-04	4.78E-04	6.06E-05	9.98E-04	0.00E+00	T	BN	NA	MODERATE	NA	NA	159	158	0	152	112	37	24.34
Chewer	IOB_10	chr18	67068529	C	T	DOK6	MISSENSE	ENSG00000206052:[ENST00000382713(c.49C>T,Cgc>Tgc):ENSP00000372160(p.Arg17Cys)]	NA	NA	NA	NA	NA	NA	NA	D	PrD	NA	MODERATE	N	0.92	195	194	0	311	234	76	24.44
Chewer	IOB_10	chr18	74091787	C	A	ZNF516	SILENT	ENSG00000101493:[ENST00000443185(c.2283G>T,ccG>ccT):ENSP00000394757(p.Pro761(=))]	NA	NA	NA	NA	NA	NA	NA	NA	NA	NA	LOW	NA	NA	299	299	0	281	273	6	2.14
Chewer	IOB_10	chr18	74667919	C	T	ZNF236	INTRONIC	7776:[XM_005266762.1(c.4744-22C>T)]	NA	NA	NA	NA	NA	NA	NA	NA	NA	NA	MODIFIER	NA	NA	52	51	0	62	49	12	19.35
Chewer	IOB_10	chr19	1080183	G	T	HMHA1	INTRONIC	ENSG00000180448:[ENST00000313093(c.1703+66G>T)]	NA	NA	chr19*1080183*1080183*G*A*rs538742323*0*0.9998*0.0001997	NA	NA	NA	NA	NA	NA	NA	MODIFIER	NA	NA	258	257	0	250	170	78	31.2
Chewer	IOB_10	chr19	1119102	G	A	SBNO2	MISSENSE	ENSG00000064932:[ENST00000361757(c.1435C>T,Cgc>Tgc):ENSP00000354733(p.Arg479Cys)]	NA	chr19*1119102*1119102*COSM990766*G*A*1*endometrium*1|chr19*1119102*1119102*COSM990767*G*A*1*endometrium*1	NA	NA	NA	NA	NA	D	PrD	NA	MODERATE	D	0.826	305	305	0	234	162	70	29.91
Chewer	IOB_10	chr19	2427399	G	A	TIMM13	INTRONIC	ENSG00000099800:[ENST00000215570(c.120+13C>T)]	NA	NA	NA	NA	NA	NA	NA	NA	NA	NA	MODIFIER	NA	NA	206	206	0	175	167	7	4
Chewer	IOB_10	chr19	10561156	C	T	PDE4A	INTRONIC	ENSG00000065989:[ENST00000440014(c.438-123C>T)]	NA	NA	NA	NA	NA	NA	NA	NA	NA	NA	MODIFIER	NA	NA	36	36	0	35	25	9	25.71
Chewer	IOB_10	chr19	10753110	C	T	SLC44A2	MISSENSE	ENSG00000129353:[ENST00000407327(c.1991C>T,aCg>aTg):ENSP00000385135(p.Thr664Met)]	NA	chr19*10753110*10753110*COSM3718047*C*T*2*liver*2	NA	NA	NA	NA	NA	D	PrD	NA	MODERATE	NA	NA	185	183	0	166	121	45	27.11
Chewer	IOB_10	chr19	11216055	C	T	LDLR	MISSENSE	ENSG00000130164:[ENST00000560467(c.73C>T,tCc>tTc):ENSP00000453513(p.Ser25Phe)]	NA	NA	chr19*11216055*11216055*C*G,T*rs763998635*NA*NA*NA	NA	NA	NA	NA	D	PoD	NA	MODERATE	N	0	214	214	0	197	146	51	25.89
Chewer	IOB_10	chr19	15218247	G	A	SYDE1	STARTLOSS	ENSG00000105137:[ENST00000342784(c.3G>A,atG>atA):ENSP00000341489(p.Met1?)]	NA	NA	NA	NA	NA	NA	NA	D_lc	BN	NA	HIGH	U	1	35	35	0	47	31	16	34.04
Chewer	IOB_10	chr19	17017791	C	T	CPAMD8	MISSENSE	ENSG00000160111:[ENST00000443236(c.4139G>A,cGc>cAc):ENSP00000402505(p.Arg1380His)]	NA	NA	chr19*17017791*17017791*C*A,T*rs764644028*NA*NA*NA	2.03E-05	0.00E+00	NA	NA	D	PoD	NA	MODERATE	U	0.999	157	157	0	135	130	5	3.7
Chewer	IOB_10	chr19	18779335	C	T	KLHL26	SILENT	ENSG00000167487:[ENST00000300976(c.1128C>T,ggC>ggT):ENSP00000300976(p.Gly376(=))]	NA	NA	NA	NA	NA	NA	NA	NA	NA	NA	LOW	NA	NA	152	151	0	166	118	47	28.31
Chewer	IOB_10	chr19	34984257	G	C	WTIP	INTRONIC	ENSG00000142279:[ENST00000590071(c.900+27G>C)]	NA	NA	NA	NA	NA	NA	NA	NA	NA	NA	MODIFIER	NA	NA	226	226	0	217	158	57	26.27
Chewer	IOB_10	chr19	38709270	G	A	DPF1	MISSENSE	8193:[XM_005259294.1(c.8C>T,tCg>tTg):XP_005259351.1(p.Ser3Leu)]	NA	NA	2.00E-04	2.47E-05	0.00E+00	2.00E-04	0.00E+00	NA	NA	NA	MODIFIER	NA	NA	44	44	0	35	23	12	34.29
Chewer	IOB_10	chr19	46888047	C	G	PPP5C	NCT	ENSG00000011485:[ENST00000493347(n.254-14C>G)]	NA	NA	NA	NA	NA	NA	NA	NA	NA	NA	MODIFIER	NA	NA	23	23	0	27	19	7	25.93
Chewer	IOB_10	chr19	47260108	C	T	FKRP	SILENT	ENSG00000181027:[ENST00000318584(c.1401C>T,cgC>cgT):ENSP00000326570(p.Arg467(=))]	NA	NA	NA	NA	NA	NA	NA	NA	NA	NA	LOW	NA	NA	112	112	0	98	67	31	31.63
Chewer	IOB_10	chr19	49363604	C	G	PLEKHA4	INTRONIC-SS-DNR-PRX	ENSG00000105559:[ENST00000596982(c.*187+3G>C)]	NA	NA	NA	NA	NA	NA	NA	NA	NA	NA	LOW	NA	NA	188	188	0	168	113	54	32.14
Chewer	IOB_10	chr19	50962166	C	T	MYBPC2	INTRONIC-SS-ACR-PRX	ENSG00000086967:[ENST00000357701(c.2507-9C>T)]	NA	NA	NA	NA	NA	NA	NA	NA	NA	NA	MODIFIER	NA	NA	118	114	0	117	112	5	4.27
Chewer	IOB_10	chr19	55044235	T	C	KIR3DX1	INTRONIC	ENSG00000104970:[ENST00000221567(c.35-27T>C)]	NA	NA	NA	3.62E-03	2.63E-02	NA	NA	NA	NA	NA	MODIFIER	NA	NA	72	71	0	95	88	5	5.26

Patel *et al.* , 2021. Whole-exome sequencing analysis of oral squamous cell carcinoma delineated by tobacco usage habits
Supplementary Table 4. List of somatic single nucleotide variants identified using whole exome sequencing of OSCC patients

Cohort	Sample	Chromosome	Genomic position	Reference allele	Alternate allele	Genesymbol	Variant type	Variant description	OncoMD	COSMIC	dbSNP	ExAC	ExAC_SAS	1000G	1000G_SAS	SIFT	Polyphen2	CONDEL	VEP	LRT	PhastCon7Way	Total reads in Normal	Reference read support in Normal	Alternative read support in Normal	Total reads in Tumor	Reference read support in Tumor	Alternate read support in Tumor	Alternate allele frequency in Tumor
Chewer	IOB_10	chr19	55044241	C	T	KIR3DX1	NCT	90011:[NR_104097.1(n.141-21C>T)]	NA	NA	chr19*55044240*55044241*TC*T*rs56695343*NA*NA*NA|chr19*55044241*55044241*C*CT,CTT,CTTT*rs775470980*NA*NA*NA|chr19*55044241*55044241*C*CT*rs74177858*NA*NA*NA|chr19*55044241*55044241*C*G,T*rs116763600*NA*NA*NA|chr19*55044241*55044242*CT*C*rs755344239*NA*NA*NA|chr19*55044241*55044243*CTT*C*rs754147239*NA*NA*NA|chr19*55044241*55044244*CTTT*C*rs758990187*NA*NA*NA	NA	NA	NA	NA	NA	NA	NA	MODIFIER	NA	NA	74	74	0	101	91	5	4.95
Chewer	IOB_10	chr19	55148342	C	T	AC009892.10	INTRONIC	ENSG00000224730:[ENST00000456337(c.46-815G>A)]	NA	NA	3.99E-04	8.26E-05	0.00E+00	3.99E-04	0.00E+00	NA	NA	NA	MODIFIER	NA	NA	165	165	0	170	163	5	2.94
Chewer	IOB_10	chr19	55148342	C	T	LILRB1	EXONIC-NC	10859:[NR_103518.2(n.2055C>T)]	NA	NA	3.99E-04	8.26E-05	0.00E+00	3.99E-04	0.00E+00	NA	NA	NA	MODIFIER	NA	NA	165	165	0	170	163	5	2.94
Chewer	IOB_10	chr19	55743059	C	A	PPP6R1	NCT	ENSG00000105063:[ENST00000587457(n.1302-23G>T)]	NA	NA	chr19*55743049*55743062*AGAGGCAGCCCAAG*A*rs552704147*0*0.9992*0.0007987	NA	NA	NA	NA	NA	NA	NA	MODIFIER	NA	NA	174	173	0	174	167	5	2.87
Chewer	IOB_10	chr2	10262911	C	T	RRM2	MISSENSE	ENSG00000171848:[ENST00000360566(c.166C>T,Cgc>Tgc):ENSP00000353770(p.Arg56Cys)]	NA	NA	chr2*10262911*10262911*C*A*rs760561877*NA*NA*NA	NA	NA	NA	NA	NA	NA	NA	MODIFIER	NA	NA	167	167	0	214	139	73	34.11
Chewer	IOB_10	chr2	42990648	G	A	MTA3	INTRONIC	57504:[XM_005264457.1(c.1760-20407G>A)]	NA	chr2*42990648*42990648*COSM307092*G*A*1*central_nervous_system*1	2.00E-04	1.01E-04	0.00E+00	2.00E-04	0.00E+00	NA	NA	NA	MODIFIER	NA	NA	122	122	0	153	95	58	37.91
Chewer	IOB_10	chr2	42990648	G	A	OXER1	SILENT	ENSG00000162881:[ENST00000378661(c.672C>T,aaC>aaT):ENSP00000367930(p.Asn224(=))]	NA	chr2*42990648*42990648*COSM307092*G*A*1*central_nervous_system*1	2.00E-04	1.01E-04	0.00E+00	2.00E-04	0.00E+00	NA	NA	NA	MODIFIER	NA	NA	122	122	0	153	95	58	37.91
Chewer	IOB_10	chr2	43922306	C	T	PLEKHH2	MISSENSE	ENSG00000152527:[ENST00000282406(c.445C>T,Cgt>Tgt):ENSP00000282406(p.Arg149Cys)]	NA	NA	NA	3.96E-05	7.50E-05	NA	NA	NA	NA	NA	MODIFIER	NA	NA	30	30	0	16	9	7	43.75
Chewer	IOB_10	chr2	51255261	C	T	NRXN1	MISSENSE	ENSG00000179915:[ENST00000401669(c.151G>A,Gag>Aag):ENSP00000385017(p.Glu51Lys)]	NA	NA	NA	NA	NA	NA	NA	T	BN	NA	MODERATE	U	1	139	139	0	266	175	91	34.21
Chewer	IOB_10	chr2	72360292	C	T	CYP26B1	MISSENSE	ENSG00000003137:[ENST00000412253(c.433G>A,Ggc>Agc):ENSP00000401465(p.Gly145Ser)]	NA	NA	2.00E-04	3.48E-05	0.00E+00	2.00E-04	0.00E+00	T	BN	NA	MODERATE	N	1	107	107	0	139	91	48	34.53
Chewer	IOB_10	chr2	74642356	C	T	C2orf81	SILENT	ENSG00000159239:[ENST00000517883(c.663G>A,cgG>cgA):ENSP00000431103(p.Arg221(=))]	NA	NA	NA	NA	NA	NA	NA	NA	NA	NA	LOW	NA	NA	168	167	1	211	205	6	2.84
Chewer	IOB_10	chr2	112813365	C	T	TMEM87B	INTRONIC	ENSG00000153214:[ENST00000283206(c.165+32C>T)]	NA	NA	NA	NA	NA	NA	NA	NA	NA	NA	MODIFIER	NA	NA	128	128	0	140	89	51	36.43
Chewer	IOB_10	chr2	121742107	C	T	GLI2	MISSENSE	ENSG00000074047:[ENST00000314490(c.760C>T,Cgg>Tgg):ENSP00000312694(p.Arg254Trp)]	NA	NA	NA	NA	NA	NA	NA	D	PrD	NA	MODERATE	D	1	238	238	0	284	206	76	26.76
Chewer	IOB_10	chr2	128394697	C	T	MYO7B	INTRONIC	ENSG00000169994:[ENST00000409090(c.2809-194C>T)]	NA	NA	NA	NA	NA	NA	NA	NA	NA	NA	MODIFIER	NA	NA	70	69	0	89	84	5	5.62
Chewer	IOB_10	chr2	173662221	G	A	RAPGEF4	NCT	ENSG00000091428:[ENST00000484331(n.276-32G>A)]	NA	NA	NA	NA	NA	NA	NA	NA	NA	NA	MODIFIER	NA	NA	42	42	0	44	33	11	25
Chewer	IOB_10	chr2	177016359	T	C	HOXD3	5UTR	ENSG00000128652:[ENST00000468418(c.-853T>C)]	NA	NA	NA	NA	NA	NA	NA	NA	NA	NA	MODIFIER	NA	NA	82	82	0	119	110	8	6.72
Chewer	IOB_10	chr2	177016359	T	C	HOXD4	5UTR	ENSG00000170166:[ENST00000306324(c.-3T>C)]	NA	NA	NA	NA	NA	NA	NA	NA	NA	NA	MODIFIER	NA	NA	82	82	0	119	110	8	6.72
Chewer	IOB_10	chr2	202149647	A	T	CASP8	MISSENSE	ENSG00000064012:[ENST00000432109(c.911A>T,cAc>cTc):ENSP00000412523(p.His304Leu)]	NA	NA	NA	NA	NA	NA	NA	NA	NA	NA	MODIFIER	NA	NA	409	406	0	355	259	93	26.2
Chewer	IOB_10	chr2	220474047	C	T	STK11IP	INTRONIC	ENSG00000144589:[ENST00000483319(c.69-38C>T)]	NA	NA	NA	NA	NA	NA	NA	NA	NA	NA	MODIFIER	NA	NA	59	59	0	91	73	17	18.68
Chewer	IOB_10	chr2	227661657	G	C	IRS1	MISSENSE	ENSG00000169047:[ENST00000305123(c.1798C>G,Cgc>Ggc):ENSP00000304895(p.Arg600Gly)]	NA	NA	chr2*227661657*227661657*G*A*rs375677810*NA*NA*NA	NA	NA	NA	NA	D	BN	NA	MODERATE	D	1	225	224	0	332	208	122	36.75
Chewer	IOB_10	chr2	228476332	C	A	C2orf83	SILENT	ENSG00000042304:[ENST00000264387(c.231G>T,ggG>ggT):ENSP00000264387(p.Gly77(=))]	NA	NA	NA	NA	NA	NA	NA	NA	NA	NA	MODIFIER	NA	NA	41	41	0	50	43	6	12
Chewer	IOB_10	chr2	230911334	A	G	SLC16A14	MISSENSE	ENSG00000163053:[ENST00000295190(c.508T>C,Ttc>Ctc):ENSP00000295190(p.Phe170Leu)]	NA	NA	NA	NA	NA	NA	NA	NA	NA	NA	MODERATE	NA	0.984	172	171	0	184	178	5	2.72
Chewer	IOB_10	chr2	232153354	C	T	ARMC9	NCT	ENSG00000135931:[ENST00000481520(n.747-2712C>T)]	NA	NA	NA	NA	NA	NA	NA	NA	NA	NA	MODIFIER	NA	NA	125	124	0	134	89	45	33.58
Chewer	IOB_10	chr2	232572406	C	T	PTMA	5UTR	ENSG00000187514:[ENST00000409321(c.-70C>T)]	NA	NA	NA	NA	NA	NA	NA	NA	NA	NA	MODIFIER	NA	NA	201	200	0	193	187	5	2.59
Chewer	IOB_10	chr2	233390035	C	T	PRSS56	MISSENSE	ENSG00000237412:[ENST00000449534(c.1634C>T,cCg>cTg):ENSP00000473410(p.Pro545Leu)]	NA	NA	NA	NA	NA	NA	NA	D	PrD	NA	MODERATE	NA	NA	54	54	0	73	60	12	16.44
Chewer	IOB_10	chr2	233880375	C	A	AC106876.2	INTRONIC	ENSG00000222001:[ENST00000409905(c.227-42C>A)]	NA	NA	NA	NA	NA	NA	NA	NA	NA	NA	MODIFIER	NA	NA	210	210	0	142	114	27	19.01
Chewer	IOB_10	chr2	233880375	C	A	LOC101928881	NCT	101928881:[XR_241427.1(n.336-42C>A)]	NA	NA	NA	NA	NA	NA	NA	NA	NA	NA	MODIFIER	NA	NA	210	210	0	142	114	27	19.01
Chewer	IOB_10	chr2	239976565	A	C	HDAC4	INTRONIC	ENSG00000068024:[ENST00000543185(c.1741-36T>G)]	NA	NA	7.99E-04	2.14E-04	0.00E+00	7.99E-04	0.00E+00	NA	NA	NA	MODIFIER	NA	NA	166	166	0	150	97	52	34.67
Chewer	IOB_10	chr2	241724432	C	A	KIF1A	MISSENSE	ENSG00000130294:[ENST00000428768(c.116G>T,Gca>Tca):ENSP00000395443(p.Ala40Ser)]	NA	NA	NA	NA	NA	NA	NA	T	BN	NA	MODERATE	U	0.79	136	135	0	142	137	5	3.52
Chewer	IOB_10	chr2	242187675	C	A	HDLBP	MISSENSE	ENSG00000115677:[ENST00000310931(c.1601G>T,cGt>cTt):ENSP00000312042(p.Arg534Leu)]	NA	chr2*242187675*242187675*COSM3426298*C*T*1*large_intestine*1|chr2*242187675*242187675*COSM3426297*C*T*1*large_intestine*1	NA	NA	NA	NA	NA	D	PoD	NA	MODERATE	D	1	341	339	0	369	360	6	1.63
Chewer	IOB_10	chr20	1285832	C	T	SNPH	MISSENSE	ENSG00000101298:[ENST00000381873(c.619C>T,Cgc>Tgc):ENSP00000371297(p.Arg207Cys)]	NA	NA	5.99E-04	1.38E-04	0.00E+00	5.99E-04	0.00E+00	D	PrD	NA	MODERATE	D	0.723	138	137	1	202	126	75	37.13
Chewer	IOB_10	chr20	9561425	G	A	PAK7	SILENT	ENSG00000101349:[ENST00000353224(c.357C>T,caC>caT):ENSP00000322957(p.His119(=))]	NA	NA	NA	NA	NA	NA	NA	NA	NA	NA	LOW	NA	0	142	142	0	138	88	50	36.23
Chewer	IOB_10	chr20	33760034	C	A	EDEM2	INTRONIC	ENSG00000088298:[ENST00000540582(c.-16-25296G>T)]	NA	NA	chr20*33760034*33760034*C*T*rs200863394*0*0.9998*0.0001997|chr20*33760034*33760035*CG*C*rs1039551360*NA*NA*NA	NA	NA	NA	NA	NA	NA	NA	LOW	NA	NA	89	89	0	108	103	5	4.63
Chewer	IOB_10	chr20	33760034	C	A	PROCR	INTRONIC-SS-DNR-PRX	ENSG00000101000:[ENST00000216968(c.70+7C>A)]	NA	NA	chr20*33760034*33760034*C*T*rs200863394*0*0.9998*0.0001997|chr20*33760034*33760035*CG*C*rs1039551360*NA*NA*NA	NA	NA	NA	NA	NA	NA	NA	LOW	NA	NA	89	89	0	108	103	5	4.63
Chewer	IOB_10	chr20	35533826	G	A	SAMHD1	MISSENSE	ENSG00000101347:[ENST00000262878(c.1351C>T,Cgt>Tgt):ENSP00000262878(p.Arg451Cys)]	NA	NA	NA	NA	NA	NA	NA	D	PrD	NA	MODERATE	D	1	115	115	0	85	65	20	23.53
Chewer	IOB_10	chr20	43565672	T	A	PABPC1L	INTRONIC	ENSG00000101104:[ENST00000479873(c.390+55T>A)]	NA	NA	NA	NA	NA	NA	NA	NA	NA	NA	MODIFIER	NA	NA	76	75	0	62	43	19	30.65
Chewer	IOB_10	chr20	43565687	C	A	PABPC1L	NCT	ENSG00000101104:[ENST00000476056(n.800+316C>A)]	NA	NA	NA	NA	NA	NA	NA	NA	NA	NA	MODIFIER	NA	NA	66	66	0	54	37	17	31.48
Chewer	IOB_10	chr20	52645453	C	T	BCAS1	SILENT	ENSG00000064787:[ENST00000371435(c.201G>A,acG>acA):ENSP00000360490(p.Thr67(=))]	NA	chr20*52645453*52645453*COSM1533986*C*A*1*lung*1	NA	6.61E-05	6.09E-05	NA	NA	NA	NA	NA	LOW	NA	NA	99	99	0	91	65	24	26.37
Chewer	IOB_10	chr20	61992405	G	A	CHRNA4	INTRONIC	1137:[XM_005260190.1(c.-145-10026C>T)]	NA	NA	NA	NA	NA	NA	NA	NA	NA	NA	MODIFIER	NA	NA	18	18	0	24	17	7	29.17
Chewer	IOB_10	chr20	61992405	G	A	RP11-261N11.8	NCT	ENSG00000203900:[ENST00000370257(n.183-765G>A)]	NA	NA	NA	NA	NA	NA	NA	NA	NA	NA	MODIFIER	NA	NA	18	18	0	24	17	7	29.17
Chewer	IOB_10	chr21	38850578	C	A	DYRK1A	SILENT	ENSG00000157540:[ENST00000321219(c.303C>A,atC>atA):ENSP00000319032(p.Ile101(=))]	NA	NA	NA	NA	NA	NA	NA	NA	NA	NA	LOW	NA	1	182	182	0	230	224	6	2.61
Chewer	IOB_10	chr21	43513977	G	A	UMODL1	INTRONIC	ENSG00000177398:[ENST00000408910(c.931+3429G>A)]	NA	NA	NA	NA	NA	NA	NA	NA	NA	NA	LOW	NA	NA	163	162	0	153	147	5	3.27
Chewer	IOB_10	chr21	46924416	C	T	COL18A1	SILENT	ENSG00000182871:[ENST00000359759(c.4059C>T,ccC>ccT):ENSP00000352798(p.Pro1353(=))]	NA	chr21*46924416*46924416*COSM3708090*C*T*1*liver*1|chr21*46924416*46924416*COSM3708091*C*T*1*liver*1	NA	8.55E-04	7.84E-04	NA	NA	NA	NA	NA	LOW	NA	NA	49	49	0	63	39	24	38.1
Chewer	IOB_10	chr21	46924416	C	T	SLC19A1	INTRONIC	ENSG00000173638:[ENST00000567670(c.1294-5890G>A)]	NA	chr21*46924416*46924416*COSM3708090*C*T*1*liver*1|chr21*46924416*46924416*COSM3708091*C*T*1*liver*1	NA	8.55E-04	7.84E-04	NA	NA	NA	NA	NA	LOW	NA	NA	49	49	0	63	39	24	38.1
Chewer	IOB_10	chr21	47771488	A	G	PCNT	NCT	ENSG00000160299:[ENST00000480896(n.1725+34A>G)]	NA	NA	NA	NA	NA	NA	NA	NA	NA	NA	MODIFIER	NA	NA	88	88	0	84	58	26	30.95
Chewer	IOB_10	chr22	19435147	G	A	C22orf39	INTRONIC	ENSG00000242259:[ENST00000333059(c.24+41C>T)]	NA	NA	NA	NA	NA	NA	NA	NA	NA	NA	MODIFIER	NA	NA	106	106	0	122	94	27	22.13
Chewer	IOB_10	chr22	19435147	G	A	HIRA	INTRONIC	ENSG00000100084:[ENST00000541063(c.-96+41C>T)]	NA	NA	NA	NA	NA	NA	NA	NA	NA	NA	MODIFIER	NA	NA	106	106	0	122	94	27	22.13
Chewer	IOB_10	chr22	19918648	G	A	TXNRD2	INTRONIC	10587:[XM_005261218.1(c.8-27C>T)]	NA	NA	NA	8.30E-06	6.06E-05	NA	NA	NA	NA	NA	MODIFIER	NA	NA	189	186	0	168	161	5	2.98
Chewer	IOB_10	chr22	20138006	C	A	AC006547.14	MISSENSE	ENSG00000234409:[ENST00000439765(c.394G>T,caG>caT):ENSP00000409542(p.Gln132His)]	NA	NA	NA	NA	NA	NA	NA	D_lc	PrD	NA	MODERATE	NA	NA	25	25	0	33	22	11	33.33
Chewer	IOB_10	chr22	20138006	C	A	LOC388849	MISSENSE	388849:[XM_005261241.1(c.504G>T,caG>caT):XP_005261298.1(p.Gln168His)]	NA	NA	NA	NA	NA	NA	NA	D_lc	PrD	NA	MODERATE	NA	NA	25	25	0	33	22	11	33.33
Chewer	IOB_10	chr22	25145713	G	A	PIWIL3	MISSENSE	ENSG00000184571:[ENST00000527701(c.836C>T,aCg>aTg):ENSP00000435718(p.Thr279Met)]	NA	chr22*25145713*25145713*COSM1032752*G*A*1*endometrium*1	2.00E-04	4.94E-05	0.00E+00	2.00E-04	0.00E+00	T	PoD	NA	MODERATE	U	0	140	139	0	143	107	36	25.17
Chewer	IOB_10	chr22	28386733	G	A	TTC28	INTRONIC	23331:[XM_005261405.1(c.5554-738C>T)]	NA	NA	NA	NA	NA	NA	NA	NA	NA	NA	MODIFIER	NA	NA	19	19	0	22	14	8	36.36
Chewer	IOB_10	chr22	28386733	G	A	TTC28-AS1	NCT	ENSG00000235954:[ENST00000434221(n.90-2379G>A)]	NA	NA	NA	NA	NA	NA	NA	NA	NA	NA	MODIFIER	NA	NA	19	19	0	22	14	8	36.36
Chewer	IOB_10	chr22	29092976	C	A	CHEK2	INTRONIC-SS-ACR	ENSG00000183765:[ENST00000328354(c.1009-1G>T)]	NA	NA	NA	NA	NA	NA	NA	NA	NA	NA	HIGH	NA	NA	174	174	0	184	178	5	2.72
Chewer	IOB_10	chr22	29957839	C	T	NIPSNAP1	MISSENSE	ENSG00000184117:[ENST00000415100(c.429G>A,cGa>cAa):ENSP00000407851(p.Arg144Gln)]	NA	NA	NA	8.24E-06	0.00E+00	NA	NA	T	BN	NA	MODERATE	D	1	95	95	0	88	62	25	28.41
Chewer	IOB_10	chr22	38523558	T	A	PLA2G6	INTRONIC	8398:[XM_005261769.1(c.931+718A>T)]	NA	NA	chr22*38523558*38523558*T*C*rs942071652*NA*NA*NA	NA	NA	NA	NA	NA	NA	NA	MODIFIER	NA	NA	93	92	0	83	57	25	30.12
Chewer	IOB_10	chr22	38523560	C	A	PLA2G6	INTRONIC	8398:[XM_005261767.1(c.1132+716G>T)]	NA	NA	NA	NA	NA	NA	NA	NA	NA	NA	MODIFIER	NA	NA	89	89	0	77	54	23	29.87
Chewer	IOB_10	chr3	9945632	G	A	IL17RE	NCT	132014:[XR_245094.1(n.218-84G>A)]	NA	NA	NA	NA	NA	NA	NA	NA	NA	NA	MODIFIER	NA	NA	73	73	0	70	52	18	25.71
Chewer	IOB_10	chr3	12941002	C	A	IQSEC1	INTRONIC	9922:[XM_005265627.1(c.2851-69G>T)]	NA	NA	NA	NA	NA	NA	NA	NA	NA	NA	MODIFIER	NA	NA	228	225	1	247	239	6	2.43
Chewer	IOB_10	chr3	38647460	C	A	SCN5A	MISSENSE	ENSG00000183873:[ENST00000333535(c.1320G>T,atG>atT):ENSP00000328968(p.Met440Ile)]	NA	NA	NA	NA	NA	NA	NA	T	BN	NA	MODERATE	N	1	111	111	0	103	79	24	23.3

Patel *et al.* , 2021. Whole-exome sequencing analysis of oral squamous cell carcinoma delineated by tobacco usage habits
Supplementary Table 4. List of somatic single nucleotide variants identified using whole exome sequencing of OSCC patients

Cohort	Sample	Chromosome	Genomic position	Reference allele	Alternate allele	Genesymbol	Variant type	Variant description	OncoMD	COSMIC	dbSNP	ExAC	ExAC_SAS	1000G	1000G_SAS	SIFT	Polyphen2	CONDEL	VEP	LRT	PhastCon7Way	Total reads in Normal	Reference read support in Normal	Alternative read support in Normal	Total reads in Tumor	Reference read support in Tumor	Alternate read support in Tumor	Alternate allele frequency in Tumor
Chewer	IOB_10	chr3	42597440	G	A	SEC22C	SILENT	ENSG00000093183:[ENST00000536332(c.483C>T,ttC>ttT):ENSP00000439845(p.Phe161(=))]	NA	chr3*42597440*42597440*COSM1044702*G*T*1*endometrium*1	NA	3.34E-05	2.45E-04	NA	NA	NA	NA	NA	LOW	NA	NA	144	143	0	125	117	7	5.6
Chewer	IOB_10	chr3	42727173	C	T	KLHL40	SILENT	ENSG00000157119:[ENST00000287777(c.63C>T,gaC>gaT):ENSP00000287777(p.Asp21(=))]	NA	NA	NA	7.60E-05	0.00E+00	NA	NA	NA	NA	NA	LOW	NA	0.002	321	318	1	317	216	101	31.86
Chewer	IOB_10	chr3	46726951	C	T	ALS2CL	NCT	259173:[NR_033815.1(n.873+64G>A)]	NA	NA	NA	NA	NA	NA	NA	NA	NA	NA	MODIFIER	NA	NA	106	106	0	83	78	5	6.02
Chewer	IOB_10	chr3	48896095	C	T	SLC25A20	INTRONIC	ENSG00000178537:[ENST00000430379(c.500-31G>A)]	NA	NA	NA	NA	NA	NA	NA	NA	NA	NA	MODIFIER	NA	NA	55	55	0	53	32	19	35.85
Chewer	IOB_10	chr3	113672709	G	A	ZDHHC23	NONSENSE	ENSG00000184307:[ENST00000498275(c.306G>A,tgG>tgA):ENSP00000417840(p.Trp102Ter)]	NA	NA	NA	2.47E-05	0.00E+00	NA	NA	NA	NA	NA	MODIFIER	NA	NA	107	107	0	125	119	6	4.8
Chewer	IOB_10	chr3	119101985	C	T	ARHGAP31	SILENT	ENSG00000031081:[ENST00000264245(c.594C>T,gtC>gtT):ENSP00000264245(p.Val198(=))]	NA	chr3*119101985*119101985*COSM1036690*C*T*1*endometrium*1	NA	NA	NA	NA	NA	NA	NA	NA	LOW	NA	NA	57	57	0	68	45	22	32.35
Chewer	IOB_10	chr3	123458732	A	G	MYLK	NCT	ENSG00000065534:[ENST00000510571(n.468+68T>C)]	NA	NA	NA	NA	NA	NA	NA	NA	NA	NA	MODIFIER	NA	NA	47	47	0	70	58	11	15.71
Chewer	IOB_10	chr3	137986115	C	T	ARMC8	NCT	25852:[NR_049770.1(n.1498+32C>T)]	NA	NA	NA	NA	NA	NA	NA	NA	NA	NA	MODIFIER	NA	NA	32	32	0	24	18	5	20.83
Chewer	IOB_10	chr3	137986115	C	T	NME9	INTRONIC	ENSG00000181322:[ENST00000492993(c.*66-4687G>A)]	NA	NA	NA	NA	NA	NA	NA	NA	NA	NA	MODIFIER	NA	NA	32	32	0	24	18	5	20.83
Chewer	IOB_10	chr3	150289902	C	A	EIF2A	MISSENSE	ENSG00000144895:[ENST00000487799(c.894C>A,agC>agA):ENSP00000420537(p.Ser298Arg)]	NA	NA	NA	NA	NA	NA	NA	D	PoD	NA	MODERATE	D	1	299	296	1	313	303	7	2.24
Chewer	IOB_10	chr3	150289902	C	A	NA	NCT	101928061:[XR_241595.1(n.290-5009G>T)]	NA	NA	NA	NA	NA	NA	NA	D	PoD	NA	MODERATE	D	1	299	296	1	313	303	7	2.24
Chewer	IOB_10	chr3	150289902	C	A	SERP1	NCT	ENSG00000120742:[ENST00000491195(n.228-5009G>T)]	NA	NA	NA	NA	NA	NA	NA	D	PoD	NA	MODERATE	D	1	299	296	1	313	303	7	2.24
Chewer	IOB_10	chr3	160118530	G	A	RP11-432B6.3	INTRONIC	ENSG00000248710:[ENST00000483754(c.953-34590C>T)]	NA	NA	NA	NA	NA	NA	NA	NA	NA	NA	MODIFIER	NA	NA	36	36	0	56	40	15	26.79
Chewer	IOB_10	chr3	160118530	G	A	SMC4	INTRONIC	10051:[XM_005247057.1(c.-1409-80G>A)]	NA	NA	NA	NA	NA	NA	NA	NA	NA	NA	MODIFIER	NA	NA	36	36	0	56	40	15	26.79
Chewer	IOB_10	chr4	59433	G	A	ZNF595	SILENT	ENSG00000197701:[ENST00000509152(c.114G>A,agG>agA):ENSP00000434858(p.Arg38(=))]	NA	NA	NA	NA	NA	NA	NA	NA	NA	NA	LOW	NA	NA	867	863	2	949	932	12	1.26
Chewer	IOB_10	chr4	15004692	C	G	CPEB2	MISSENSE	ENSG00000137449:[ENST00000442003(c.395C>G,aCc>aGc):ENSP00000414270(p.Thr132Ser)]	NA	NA	NA	NA	NA	NA	NA	NA	UN	NA	MODERATE	NA	NA	254	253	0	365	268	96	26.3
Chewer	IOB_10	chr4	38022244	C	T	TBC1D1	SILENT	ENSG00000065882:[ENST00000446803(c.618C>T,atC>atT):ENSP00000396877(p.Ile206(=))]	NA	NA	NA	NA	NA	NA	NA	NA	NA	NA	MODIFIER	NA	NA	145	144	0	124	83	40	32.26
Chewer	IOB_10	chr4	40824039	C	T	APBB2	MISSENSE	ENSG00000163697:[ENST00000513140(c.1892G>A,cGt>cAt):ENSP00000426018(p.Arg631His)]	NA	NA	NA	NA	NA	NA	NA	NA	NA	NA	MODERATE	NA	1	84	84	0	64	51	13	20.31
Chewer	IOB_10	chr4	40824039	C	T	RP11-632F7.3	NCT	ENSG00000250906:[ENST00000513127(n.80-3732C>T)]	NA	NA	NA	NA	NA	NA	NA	NA	NA	NA	MODERATE	NA	1	84	84	0	64	51	13	20.31
Chewer	IOB_10	chr4	74270112	G	A	ALB	MISSENSE	ENSG00000163631:[ENST00000295897(c.68G>A,cGt>cAt):ENSP00000295897(p.Arg23His)]	NA	NA	NA	8.24E-06	0.00E+00	NA	NA	NA	NA	NA	MODIFIER	NA	NA	181	180	0	170	163	7	4.12
Chewer	IOB_10	chr4	77940333	G	T	SEPT11	MISSENSE	ENSG00000138758:[ENST00000510641(c.679G>T,Gca>Tca):ENSP00000420839(p.Ala227Ser)]	NA	NA	NA	NA	NA	NA	NA	NA	NA	NA	MODIFIER	NA	NA	60	60	0	100	64	35	35
Chewer	IOB_10	chr4	89708919	C	T	FAM13A	MISSENSE	ENSG00000138640:[ENST00000513837(c.194G>A,cGa>cAa):ENSP00000423252(p.Arg65Gln)]	NA	chr4*89708919*89708919*COSM1251781*C*T*1*oesophagus*1|chr4*89708919*89708919*COSM1251782*C*T*1*oesophagus*1	NA	NA	NA	NA	NA	T	BN	NA	MODERATE	N	0	161	161	0	171	136	35	20.47
Chewer	IOB_10	chr4	103941479	C	T	SLC9B1	5UTR	150159:[XM_005262762.1(c.-398G>A)]	NA	NA	NA	NA	NA	NA	NA	NA	NA	NA	LOW	NA	NA	67	67	0	107	71	33	30.84
Chewer	IOB_10	chr4	103941479	C	T	SLC9B2	SILENT	ENSG00000164038:[ENST00000339611(c.1323G>A,acG>acA):ENSP00000345241(p.Thr441(=))]	NA	NA	NA	NA	NA	NA	NA	NA	NA	NA	LOW	NA	NA	67	67	0	107	71	33	30.84
Chewer	IOB_10	chr4	160251017	G	A	RAPGEF2	MISSENSE	ENSG00000109756:[ENST00000264431(c.674G>A,tGc>tAc):ENSP00000264431(p.Cys225Tyr)]	NA	NA	NA	NA	NA	NA	NA	NA	NA	NA	MODERATE	NA	1	123	123	0	125	119	5	4
Chewer	IOB_10	chr4	186294206	C	A	LRP2BP	MISSENSE	ENSG00000109771:[ENST00000510776(c.529G>T,Ggc>Tgc):ENSP00000424610(p.Gly177Cys)]	NA	NA	NA	NA	NA	NA	NA	D	PrD	NA	MODERATE	D	1	105	104	0	96	69	27	28.12
Chewer	IOB_10	chr4	186294206	C	A	RP11-714G18.1	NCT	ENSG00000250410:[ENST00000514884(n.242+1987C>A)]	NA	NA	NA	NA	NA	NA	NA	D	PrD	NA	MODERATE	D	1	105	104	0	96	69	27	28.12
Chewer	IOB_10	chr4	186378991	C	A	CCDC110	INTRONIC	ENSG00000168491:[ENST00000393540(c.2350+289G>T)]	NA	NA	NA	1.07E-04	1.81E-04	NA	NA	NA	NA	NA	MODIFIER	NA	NA	17	17	0	32	25	6	18.75
Chewer	IOB_10	chr5	1879905	G	A	IRX4	MISSENSE	ENSG00000113430:[ENST00000231357(c.449C>T,aCg>aTg):ENSP00000231357(p.Thr150Met)]	NA	NA	2.00E-04	2.49E-05	6.06E-05	2.00E-04	0.00E+00	NA	UN	NA	MODERATE	NA	NA	183	182	0	197	124	73	37.06
Chewer	IOB_10	chr5	2748565	C	A	IRX2	MISSENSE	ENSG00000170561:[ENST00000302057(c.1257G>T,gaG>gaT):ENSP00000307006(p.Glu419Asp)]	NA	NA	NA	NA	NA	NA	NA	T_lc	BN	NA	MODERATE	N	0.975	37	36	0	75	50	24	32
Chewer	IOB_10	chr5	7709365	C	T	ADCY2	SILENT	ENSG00000078295:[ENST00000537121(c.903C>T,caC>caT):ENSP00000444803(p.His301(=))]	NA	NA	NA	NA	NA	NA	NA	NA	NA	NA	LOW	NA	0.539	78	77	1	101	79	20	19.8
Chewer	IOB_10	chr5	7709365	C	T	RP11-711G10.1	NCT	ENSG00000250761:[ENST00000514105(n.163-1091G>A)]	NA	NA	NA	NA	NA	NA	NA	NA	NA	NA	LOW	NA	0.539	78	77	1	101	79	20	19.8
Chewer	IOB_10	chr5	41934076	G	A	FBXO4	SILENT	ENSG00000151876:[ENST00000281623(c.675G>A,ttG>ttA):ENSP00000281623(p.Leu225(=))]	NA	NA	NA	NA	NA	NA	NA	NA	NA	NA	LOW	NA	0.993	38	38	0	37	15	20	54.05
Chewer	IOB_10	chr5	44388675	C	T	FGF10	MISSENSE	ENSG00000070193:[ENST00000264664(c.110G>A,tGc>tAc):ENSP00000264664(p.Cys37Tyr)]	NA	NA	NA	NA	NA	NA	NA	D_lc	BN	NA	MODERATE	NA	NA	96	95	0	97	92	5	5.15
Chewer	IOB_10	chr5	72469682	C	A	TMEM174	MISSENSE	ENSG00000164325:[ENST00000296776(c.612C>A,gaC>gaA):ENSP00000296776(p.Asp204Glu)]	NA	NA	chr5*72469681*72469682*AC*A*rs1025701320*NA*NA*NA|chr5*72469682*72469682*C*T*rs201696334*NA*NA*NA	NA	NA	NA	NA	T	BN	NA	MODERATE	N	0	116	116	0	123	117	6	4.88
Chewer	IOB_10	chr5	79027464	C	T	CMYA5	MISSENSE	ENSG00000164309:[ENST00000446378(c.2876C>T,cCg>cTg):ENSP00000394770(p.Pro959Leu)]	NA	NA	2.60E-03	5.98E-04	1.21E-04	2.60E-03	0.00E+00	D	PrD	NA	MODERATE	N	0.007	233	231	1	273	266	5	1.83
Chewer	IOB_10	chr5	82817181	A	G	VCAN	MISSENSE	ENSG00000038427:[ENST00000265077(c.3056A>G,gAa>gGa):ENSP00000265077(p.Glu1019Gly)]	NA	NA	NA	NA	NA	NA	NA	NA	NA	NA	MODIFIER	NA	NA	57	57	0	91	62	29	31.87
Chewer	IOB_10	chr5	90281400	C	T	ADGRV1	NCT	84059:[NR_003149.1(n.18165+61C>T)]	NA	NA	NA	NA	NA	NA	NA	NA	NA	NA	MODIFIER	NA	NA	131	131	0	141	134	5	3.55
Chewer	IOB_10	chr5	90281400	C	T	GPR98	INTRONIC	84059:[XM_005248613.1(c.11270+61C>T)]	NA	NA	NA	NA	NA	NA	NA	NA	NA	NA	MODIFIER	NA	NA	131	131	0	141	134	5	3.55
Chewer	IOB_10	chr5	128797210	C	T	ADAMTS19	SILENT	ENSG00000145808:[ENST00000274487(c.489C>T,gaC>gaT):ENSP00000274487(p.Asp163(=))]	NA	NA	chr5*128797210*128797210*C*A,T*rs777419049*NA*NA*NA	2.75E-04	0.00E+00	NA	NA	NA	NA	NA	MODIFIER	NA	NA	83	83	0	133	87	46	34.59
Chewer	IOB_10	chr5	135693037	C	T	TRPC7	SILENT	ENSG00000069018:[ENST00000355180(c.39G>A,cgG>cgA):ENSP00000347312(p.Arg13(=))]	NA	NA	NA	NA	NA	NA	NA	NA	NA	NA	LOW	NA	NA	144	144	0	205	197	5	2.44
Chewer	IOB_10	chr5	139938399	T	C	APBB3	MISSENSE	ENSG00000113108:[ENST00000412920(c.1226A>G,tAc>tGc):ENSP00000402591(p.Tyr409Cys)]	NA	NA	NA	NA	NA	NA	NA	D	PoD	NA	MODERATE	D	1	43	43	0	57	40	17	29.82
Chewer	IOB_10	chr5	140501813	G	A	PCDHB4	MISSENSE	ENSG00000081818:[ENST00000194152(c.233G>A,cGt>cAt):ENSP00000194152(p.Arg78His)]	NA	NA	NA	NA	NA	NA	NA	T_lc	BN	NA	MODERATE	NA	NA	151	151	0	227	166	60	26.43
Chewer	IOB_10	chr5	140615651	G	A	PCDHB18	EXONIC-NC	ENSG00000146001:[ENST00000524813(n.1368G>A)]	NA	NA	NA	NA	NA	NA	NA	NA	NA	NA	MODIFIER	NA	NA	31	31	0	53	29	24	45.28
Chewer	IOB_10	chr5	140615651	G	A	PCDHB18P	EXONIC-NC	54660:[NR_001281.2(n.1747G>A)]	NA	NA	NA	NA	NA	NA	NA	NA	NA	NA	MODIFIER	NA	NA	31	31	0	53	29	24	45.28
Chewer	IOB_10	chr5	156727843	G	A	CYFIP2	MISSENSE	ENSG00000055163:[ENST00000541131(c.283G>A,Gtc>Atc):ENSP00000444645(p.Val95Ile)]	NA	NA	NA	NA	NA	NA	NA	T	BN	NA	MODERATE	D	1	116	115	0	88	65	21	23.86
Chewer	IOB_10	chr5	170737349	C	T	TLX3	MISSENSE	ENSG00000164438:[ENST00000296921(c.617C>T,aCg>aTg):ENSP00000296921(p.Thr206Met)]	NA	chr5*170737349*170737349*COSM41116*C*G*1*central_nervous_system*1	NA	NA	NA	NA	NA	D	PrD	NA	MODERATE	D	1	164	162	0	197	137	59	29.95
Chewer	IOB_10	chr6	7581410	G	A	DSP	MISSENSE	ENSG00000096696:[ENST00000379802(c.4987G>A,Gcc>Acc):ENSP00000369129(p.Ala1663Thr)]	NA	NA	NA	NA	NA	NA	NA	NA	NA	NA	MODIFIER	NA	NA	121	121	0	172	130	40	23.26
Chewer	IOB_10	chr6	12122849	G	A	HIVEP1	MISSENSE	ENSG00000095951:[ENST00000379388(c.2821G>A,Gca>Aca):ENSP00000368698(p.Ala941Thr)]	NA	NA	NA	NA	NA	NA	NA	NA	NA	NA	MODIFIER	NA	NA	106	105	0	137	130	5	3.65
Chewer	IOB_10	chr6	24832554	C	A	FAM65B	MISSENSE	ENSG00000111913:[ENST00000538035(c.2274G>T,caG>caT):ENSP00000441138(p.Gln758His)]	NA	NA	NA	NA	NA	NA	NA	NA	NA	NA	MODERATE	NA	0.997	290	287	0	294	210	84	28.57
Chewer	IOB_10	chr6	26225355	C	T	HIST1H3E	5UTR	8353:[XM_005249443.1(c.-28C>T)]	NA	NA	chr6*26225355*26225355*C*A*rs775955930*NA*NA*NA	NA	NA	NA	NA	NA	NA	NA	MODIFIER	NA	NA	124	124	0	127	93	33	25.98
Chewer	IOB_10	chr6	27356968	G	A	LOC101928873	NONSENSE	101928873:[XM_005249512.1(c.335G>A,tGg>tAg):XP_005249569.1(p.Trp112Ter)]	NA	NA	NA	NA	NA	NA	NA	NA	NA	NA	MODIFIER	NA	NA	205	203	0	276	189	86	31.16
Chewer	IOB_10	chr6	27356968	G	A	ZNF391	INTRONIC	346157:[NM_001322288.1(c.-266-5828G>A)]	NA	NA	NA	NA	NA	NA	NA	NA	NA	NA	MODIFIER	NA	NA	205	203	0	276	189	86	31.16
Chewer	IOB_10	chr6	29598418	G	A	GABBR1	NONSENSE-SS-PRX	ENSG00000204681:[ENST00000467259(n.510C>T)]	NA	NA	NA	NA	NA	NA	NA	NA	NA	NA	HIGH	NA	NA	85	84	0	80	55	24	30
Chewer	IOB_10	chr6	41048601	T	C	NFYA	MISSENSE	ENSG00000001167:[ENST00000341376(c.127T>C,Tcc>Ccc):ENSP00000345702(p.Ser43Pro)]	NA	NA	chr6*41048601*41048601*T*A*rs201057528*0*0.9998*0.0001997	NA	NA	NA	NA	T_lc	BN	NA	MODERATE	D	1	120	120	0	123	88	35	28.46
Chewer	IOB_10	chr6	41048601	T	C	OARD1	INTRONIC	ENSG00000124596:[ENST00000480585(c.-41-9187A>G)]	NA	NA	chr6*41048601*41048601*T*A*rs201057528*0*0.9998*0.0001997	NA	NA	NA	NA	T_lc	BN	NA	MODERATE	D	1	120	120	0	123	88	35	28.46
Chewer	IOB_10	chr6	42985698	G	A	KLHDC3	MISSENSE	ENSG00000124702:[ENST00000332245(c.262G>A,Gag>Aag):ENSP00000331562(p.Glu88Lys)]	NA	NA	NA	8.24E-06	0.00E+00	NA	NA	D	PrD	NA	MODERATE	D	1	64	63	0	81	61	20	24.69
Chewer	IOB_10	chr6	45922804	C	T	CLIC5	INTRONIC	ENSG00000112782:[ENST00000185206(c.650+68G>A)]	NA	NA	NA	NA	NA	NA	NA	NA	NA	NA	MODIFIER	NA	NA	149	148	0	126	87	39	30.95
Chewer	IOB_10	chr6	52129436	G	C	MCM3	MISSENSE	ENSG00000112118:[ENST00000596288(c.2512C>G,Cag>Gag):ENSP00000472940(p.Gln838Glu)]	NA	NA	NA	NA	NA	NA	NA	T	BN	NA	MODERATE	D	1	104	103	0	107	75	32	29.91
Chewer	IOB_10	chr6	76744065	A	G	IMPG1	INTRONIC-SS-ACR-PRX	ENSG00000112706:[ENST00000369963(c.235-5T>C)]	NA	NA	NA	1.65E-05	1.21E-04	NA	NA	NA	NA	NA	LOW	NA	NA	75	75	0	57	38	19	33.33
Chewer	IOB_10	chr6	90418240	C	A	MDN1	MISSENSE	ENSG00000112159:[ENST00000369393(c.7873G>T,Gcc>Tcc):ENSP00000358400(p.Ala2625Ser)]	NA	NA	NA	NA	NA	NA	NA	NA	NA	NA	MODERATE	NA	0	80	80	0	99	93	6	6.06
Chewer	IOB_10	chr6	105307436	G	A	HACE1	INTRONIC-SS-DNR-PRX	ENSG00000085382:[ENST00000521962(c.38+6C>T)]	NA	NA	chr6*105307436*105307436*G*T*rs774984488*NA*NA*NA	NA	NA	NA	NA	NA	NA	NA	LOW	NA	NA	272	272	0	354	346	7	1.98
Chewer	IOB_10	chr6	110423060	C	T	WASF1	MISSENSE	ENSG00000112290:[ENST00000359451(c.1253G>A,gGt>gAt):ENSP00000352425(p.Gly418Asp)]	NA	NA	NA	8.28E-06	0.00E+00	NA	NA	T	BN	NA	MODERATE	N	0.939	82	81	0	88	67	20	22.73
Chewer	IOB_10	chr6	111696190	G	C	REV3L	MISSENSE	ENSG00000009413:[ENST00000358835(c.3368C>G,tCt>tGt):ENSP00000351697(p.Ser1123Cys)]	NA	NA	chr6*111696190*111696190*G*T*rs898731188*NA*NA*NA	NA	NA	NA	NA	NA	NA	NA	MODERATE	NA	1	204	203	0	210	151	59	28.1
Chewer	IOB_10	chr6	123824772	T	G	TRDN	INTRONIC	ENSG00000186439:[ENST00000361029(c.309+92A>C)]	NA	NA	NA	NA	NA	NA	NA	NA	NA	NA	MODIFIER	NA	NA	148	144	1	167	107	60	35.93
Chewer	IOB_10	chr6	146242573	G	A	SHPRH	INTRONIC	ENSG00000146414:[ENST00000519632(c.*667-45C>T)]	NA	NA	NA	NA	NA	NA	NA	NA	NA	NA	MODIFIER	NA	NA	103	102	0	148	103	45	30.41
Chewer	IOB_10	chr6	152540245	G	A	SYNE1	NONSENSE	ENSG00000131018:[ENST00000341594(c.20773C>T,Caa>Taa):ENSP00000341887(p.Gln6925Ter)]	NA	NA	chr6*152540245*152540245*G*T*rs754278549*NA*NA*NA	NA	NA	NA	NA	NA	NA	NA	HIGH	NA	1	55	55	0	52	35	17	32.69
Chewer	IOB_10	chr6	158538778	C	A	SERAC1	MISSENSE	ENSG00000122335:[ENST00000367101(c.1384G>T,Gca>Tca):ENSP00000356068(p.Ala462Ser)]	NA	NA	NA	NA	NA	NA	NA	NA	NA	NA	MODIFIER	NA	NA	209	208	0	172	166	5	2.91
Chewer	IOB_10	chr6	168352343	C	T	AFDN	MISSENSE	4301:[NM_001291964.1(c.4165C>T,Cgg>Tgg):NP_001278893.1(p.Arg1389Trp)]	NA	NA	NA	NA	NA	NA	NA	NA	NA	NA	MODERATE	NA	1	229	229	0	284	277	6	2.11

Patel *et al.* , 2021. Whole-exome sequencing analysis of oral squamous cell carcinoma delineated by tobacco usage habits
Supplementary Table 4. List of somatic single nucleotide variants identified using whole exome sequencing of OSCC patients

Cohort	Sample	Chromosome	Genomic position	Reference allele	Alternate allele	Genesymbol	Variant type	Variant description	OncoMD	COSMIC	dbSNP	ExAC	ExAC_SAS	1000G	1000G_SAS	SIFT	Polyphen2	CONDEL	VEP	LRT	PhastCon7Way	Total reads in Normal	Reference read support in Normal	Alternative read support in Normal	Total reads in Tumor	Reference read support in Tumor	Alternate read support in Tumor	Alternate allele frequency in Tumor
Chewer	IOB_10	chr6	168352343	C	T	MLLT4	MISSENSE	ENSG00000130396:[ENST00000351017(c.4309C>T,Cgg>Tgg):ENSP00000252692(p.Arg1437Trp)]	NA	NA	NA	NA	NA	NA	NA	NA	NA	NA	MODERATE	NA	1	229	229	0	284	277	6	2.11
Chewer	IOB_10	chr7	2414320	C	T	EIF3B	INTRONIC	ENSG00000106263:[ENST00000360876(c.1889+79C>T)]	NA	NA	1.20E-03	NA	NA	1.20E-03	1.00E-03	NA	NA	NA	MODIFIER	NA	NA	110	110	0	104	80	24	23.08
Chewer	IOB_10	chr7	2472403	G	A	CHST12	SILENT	ENSG00000136213:[ENST00000258711(c.129G>A,ccG>ccA):ENSP00000258711(p.Pro43(=))]	NA	NA	2.00E-04	8.33E-06	6.07E-05	2.00E-04	1.00E-03	NA	NA	NA	LOW	NA	NA	252	251	0	249	242	5	2.01
Chewer	IOB_10	chr7	5540134	C	A	FBXL18	MISSENSE	ENSG00000155034:[ENST00000458142(c.1417G>T,cGg>cTg):ENSP00000405896(p.Arg473Leu)]	NA	NA	NA	NA	NA	NA	NA	D	BN	NA	MODERATE	N	1	83	82	0	85	80	5	5.88
Chewer	IOB_10	chr7	6641666	C	A	C7orf26	NCT	ENSG00000146576:[ENST00000477298(n.384-66C>A)]	NA	NA	chr7*6641666*6641666*C*T*rs1006449309*NA*NA*NA	NA	NA	NA	NA	NA	NA	NA	MODIFIER	NA	NA	145	145	0	136	129	6	4.41
Chewer	IOB_10	chr7	20687721	G	A	ABCB5	INTRONIC	ENSG00000004846:[ENST00000258738(c.-130+20G>A)]	NA	NA	2.00E-04	1.09E-04	2.45E-04	2.00E-04	0.00E+00	NA	NA	NA	MODIFIER	NA	NA	29	29	0	23	15	8	34.78
Chewer	IOB_10	chr7	28995293	A	G	AC005013.1	EXONIC-NC	ENSG00000255690:[ENST00000539664(n.2731T>C)]	NA	NA	NA	NA	NA	NA	NA	NA	NA	NA	MODIFIER	NA	NA	188	187	0	309	212	97	31.39
Chewer	IOB_10	chr7	28995293	A	G	TRIL	SILENT	9865:[NM_014817.3(c.2370T>C,agT>agC):NP_055632.2(p.Ser790(=))]	NA	NA	NA	NA	NA	NA	NA	NA	NA	NA	MODIFIER	NA	NA	188	187	0	309	212	97	31.39
Chewer	IOB_10	chr7	31008717	C	T	GHRHR	MISSENSE	ENSG00000106128:[ENST00000326139(c.200C>T,aCg>aTg):ENSP00000320180(p.Thr67Met)]	NA	NA	2.00E-04	3.11E-04	0.00E+00	2.00E-04	0.00E+00	T	BN	NA	MODERATE	NA	NA	87	87	0	82	57	24	29.27
Chewer	IOB_10	chr7	48964712	A	G	AC006024.4	EXONIC-NC	ENSG00000218305:[ENST00000428251(n.822A>G)]	NA	NA	NA	NA	NA	NA	NA	NA	NA	NA	MODIFIER	NA	NA	225	223	0	277	199	77	27.8
Chewer	IOB_10	chr7	48964712	A	G	CDC14C	EXONIC-NC	168448:[NR_003595.1(n.556A>G)]	NA	NA	NA	NA	NA	NA	NA	NA	NA	NA	MODIFIER	NA	NA	225	223	0	277	199	77	27.8
Chewer	IOB_10	chr7	56183837	C	T	NUPR1L	SILENT	ENSG00000185290:[ENST00000329309(c.171G>A,gcG>gcA):ENSP00000455442(p.Ala57(=))]	NA	NA	NA	NA	NA	NA	NA	NA	NA	NA	LOW	NA	NA	170	170	0	194	189	5	2.58
Chewer	IOB_10	chr7	63354915	G	C	RP11-340I6.6	EXONIC-NC	ENSG00000235720:[ENST00000419271(n.1252G>C)]	NA	NA	NA	NA	NA	NA	NA	NA	NA	NA	MODIFIER	NA	NA	61	61	0	73	57	16	21.92
Chewer	IOB_10	chr7	63354915	G	C	RP11-340I6.7	NCT	ENSG00000236299:[ENST00000587704(n.234-1251C>G)]	NA	NA	NA	NA	NA	NA	NA	NA	NA	NA	MODIFIER	NA	NA	61	61	0	73	57	16	21.92
Chewer	IOB_10	chr7	98506403	C	T	TRRAP	SILENT	ENSG00000196367:[ENST00000355540(c.1168C>T,Ctg>Ttg):ENSP00000347733(p.Leu390(=))]	NA	NA	NA	NA	NA	NA	NA	NA	NA	NA	LOW	NA	NA	162	161	1	153	111	42	27.45
Chewer	IOB_10	chr7	100547349	A	G	MUC3A	NCT	ENSG00000169894:[ENST00000483366(n.131+32A>G)]	NA	NA	NA	9.84E-04	0.00E+00	NA	NA	NA	NA	NA	MODIFIER	NA	NA	36	34	1	35	23	5	14.29
Chewer	IOB_10	chr7	106513221	T	C	PIK3CG	MISSENSE	ENSG00000105851:[ENST00000359195(c.2125T>C,Tat>Cat):ENSP00000352121(p.Tyr709His)]	NA	NA	NA	NA	NA	NA	NA	D	PrD	NA	MODERATE	D	1	133	133	0	186	125	61	32.8
Chewer	IOB_10	chr7	120634125	C	T	CPED1	NCT	ENSG00000106034:[ENST00000495036(n.696+4201C>T)]	NA	NA	NA	NA	NA	NA	NA	NA	NA	NA	MODIFIER	NA	NA	118	117	0	107	72	35	32.71
Chewer	IOB_10	chr7	136700423	A	C	CHRM2	MISSENSE	ENSG00000181072:[ENST00000320658(c.811A>C,Act>Cct):ENSP00000319984(p.Thr271Pro)]	NA	chr7*136700413*136700425*COSM1622380*GGATCCTGTGACT*G*1*liver*1	NA	NA	NA	NA	NA	NA	NA	NA	MODIFIER	NA	NA	56	55	0	66	52	13	19.7
Chewer	IOB_10	chr7	136700423	A	C	hsa-mir-490	NCT	ENSG00000234352:[ENST00000425981(n.341+17118T>G)]	NA	chr7*136700413*136700425*COSM1622380*GGATCCTGTGACT*G*1*liver*1	NA	NA	NA	NA	NA	NA	NA	NA	MODIFIER	NA	NA	56	55	0	66	52	13	19.7
Chewer	IOB_10	chr7	137263121	C	T	DGKI	INTRONIC	ENSG00000157680:[ENST00000453654(c.743-50G>A)]	NA	NA	.	8.52E-06	0.00E+00	NA	NA	NA	NA	NA	MODIFIER	NA	NA	65	65	0	73	55	18	24.66
Chewer	IOB_10	chr7	142499508	G	C	TRBC2	INTRONIC	ENSG00000211772:[ENST00000466254(c.388-120G>C)]	NA	NA	NA	NA	NA	NA	NA	NA	NA	NA	MODIFIER	NA	NA	176	175	0	133	88	44	33.08
Chewer	IOB_10	chr7	142499789	A	T	TRBC2	SILENT-SS-PRX	ENSG00000211772:[ENST00000466254(c.406A>T,gAg>gTg):ENSP00000417300(p.Glu136Val)]	NA	NA	NA	1.69E-05	0.00E+00	NA	NA	NA	NA	NA	LOW	NA	NA	97	88	8	101	75	26	25.74
Chewer	IOB_10	chr7	142499793	T	C	TRBC2	SILENT	ENSG00000211772:[ENST00000466254(c.410T>C,tcT>tcC):ENSP00000417300(p.Ser137(=))]	NA	NA	NA	1.69E-05	0.00E+00	NA	NA	NA	NA	NA	LOW	NA	NA	97	88	9	103	77	26	25.24
Chewer	IOB_10	chr7	150269785	C	A	GIMAP4	SILENT	ENSG00000133574:[ENST00000461940(c.669C>A,atC>atA):ENSP00000419545(p.Ile223(=))]	NA	chr7*150269785*150269785*COSM402947*C*A*1*lung*1	NA	NA	NA	NA	NA	NA	NA	NA	MODIFIER	NA	NA	138	138	0	183	177	5	2.73
Chewer	IOB_10	chr7	157926536	C	T	PTPRN2	SILENT	ENSG00000155093:[ENST00000409483(c.1275G>A,tcG>tcA):ENSP00000387114(p.Ser425(=))]	NA	NA	3.00E-03	1.06E-03	0.00E+00	3.00E-03	0.00E+00	NA	NA	NA	LOW	NA	NA	160	159	0	186	137	48	25.81
Chewer	IOB_10	chr8	2054221	C	T	MYOM2	INTRONIC	ENSG00000036448:[ENST00000523438(c.1166+33C>T)]	NA	NA	chr8*2054221*2054221*C*A*rs940446705*NA*NA*NA	NA	NA	NA	NA	NA	NA	NA	MODIFIER	NA	NA	190	188	1	193	123	69	35.75
Chewer	IOB_10	chr8	14095089	C	A	SGCZ	INTRONIC	137868:[NM_001322879.1(c.322+12G>T)]	NA	NA	NA	NA	NA	NA	NA	NA	NA	NA	MODIFIER	NA	NA	261	260	0	244	167	77	31.56
Chewer	IOB_10	chr8	38281975	C	A	FGFR1	NCT	ENSG00000077782:[ENST00000475621(n.251+52G>T)]	NA	NA	NA	NA	NA	NA	NA	NA	NA	NA	MODIFIER	NA	NA	178	178	0	201	192	6	2.99
Chewer	IOB_10	chr8	38281975	C	A	RP11-350N15.4	NCT	ENSG00000255201:[ENST00000528407(n.388-1493C>A)]	NA	NA	NA	NA	NA	NA	NA	NA	NA	NA	MODIFIER	NA	NA	178	178	0	201	192	6	2.99
Chewer	IOB_10	chr8	41530362	G	A	ANK1	MISSENSE	ENSG00000029534:[ENST00000289734(c.4606C>T,Cgt>Tgt):ENSP00000289734(p.Arg1536Cys)]	NA	chr8*41530362*41530362*COSM1582768*G*A*1*stomach*1|chr8*41530362*41530362*COSM1582767*G*A*1*stomach*1	NA	8.63E-04	0.00E+00	NA	NA	D	BN	NA	MODERATE	N	1	57	56	0	72	62	10	13.89
Chewer	IOB_10	chr8	61768539	A	G	CHD7	SILENT	ENSG00000171316:[ENST00000423902(c.6942A>G,agA>agG):ENSP00000392028(p.Arg2314(=))]	NA	NA	NA	NA	NA	NA	NA	NA	NA	NA	LOW	NA	0.998	98	98	0	143	67	76	53.15
Chewer	IOB_10	chr8	69021890	C	A	PREX2	NCT	ENSG00000046889:[ENST00000517617(n.2857+32C>A)]	NA	NA	NA	NA	NA	NA	NA	NA	NA	NA	MODIFIER	NA	NA	57	57	0	99	61	37	37.37
Chewer	IOB_10	chr8	70981712	A	G	PRDM14	SILENT	ENSG00000147596:[ENST00000276594(c.384T>C,gaT>gaC):ENSP00000276594(p.Asp128(=))]	NA	NA	NA	NA	NA	NA	NA	NA	NA	NA	LOW	NA	0	126	125	0	228	112	116	50.88
Chewer	IOB_10	chr8	71572766	A	T	LACTB2	INTRONIC	ENSG00000147592:[ENST00000276590(c.286+1203T>A)]	NA	NA	NA	NA	NA	NA	NA	NA	NA	NA	MODIFIER	NA	NA	226	226	0	338	276	61	18.05
Chewer	IOB_10	chr8	71572766	A	T	LACTB2-AS1	EXONIC-NC	286190:[NR_038881.1(n.968A>T)]	NA	NA	NA	NA	NA	NA	NA	NA	NA	NA	MODIFIER	NA	NA	226	226	0	338	276	61	18.05
Chewer	IOB_10	chr8	71572766	A	T	RP11-382J12.1	5UTR	ENSG00000246366:[ENST00000499227(c.-31A>T)]	NA	NA	NA	NA	NA	NA	NA	NA	NA	NA	MODIFIER	NA	NA	226	226	0	338	276	61	18.05
Chewer	IOB_10	chr8	87437622	G	A	WWP1	INTRONIC	11059:[XM_005250761.1(c.1154+75G>A)]	NA	NA	8.19E-03	NA	NA	8.19E-03	0.00E+00	NA	NA	NA	MODIFIER	NA	NA	25	25	0	41	24	17	41.46
Chewer	IOB_10	chr8	88621973	T	A	AF121898.3	NCT	ENSG00000253500:[ENST00000518494(n.324+37172A>T)]	NA	NA	NA	NA	NA	NA	NA	NA	NA	NA	MODIFIER	NA	NA	62	62	0	116	99	16	13.79
Chewer	IOB_10	chr8	88621973	T	A	CNBD1	INTRONIC	ENSG00000176571:[ENST00000521593(c.342-64T>A)]	NA	NA	NA	NA	NA	NA	NA	NA	NA	NA	MODIFIER	NA	NA	62	62	0	116	99	16	13.79
Chewer	IOB_10	chr8	92982963	G	A	RUNX1T1	MISSENSE	ENSG00000079102:[ENST00000360348(c.1351C>T,Cgc>Tgc):ENSP00000353504(p.Arg451Cys)]	NA	chr8*92982963*92982963*COSM1569507*G*A*1*large_intestine*1|chr8*92982963*92982963*COSM1569505*G*A*1*large_intestine*1|chr8*92982963*92982963*COSM1569506*G*A*1*large_intestine*1	NA	1.65E-05	1.21E-04	NA	NA	NA	NA	NA	MODIFIER	NA	NA	135	135	0	205	122	83	40.49
Chewer	IOB_10	chr8	105727231	C	A	RP11-127H5.1	INTRONIC	ENSG00000253350:[ENST00000518180(c.6+28C>A)]	NA	NA	NA	NA	NA	NA	NA	NA	NA	NA	MODIFIER	NA	NA	97	96	0	149	143	6	4.03
Chewer	IOB_10	chr8	124054277	C	T	DERL1	MISSENSE	ENSG00000136986:[ENST00000259512(c.86G>A,gGc>gAc):ENSP00000259512(p.Gly29Asp)]	NA	NA	NA	4.41E-05	0.00E+00	NA	NA	T	PoD	NA	MODERATE	D	1	179	179	0	352	343	7	1.99
Chewer	IOB_10	chr8	124054277	C	T	TBC1D31	5UTR	ENSG00000156787:[ENST00000520368(c.-451C>T)]	NA	NA	NA	4.41E-05	0.00E+00	NA	NA	T	PoD	NA	MODERATE	D	1	179	179	0	352	343	7	1.99
Chewer	IOB_10	chr8	125568182	A	G	MTSS1	SILENT	9788:[XM_005251113.1(c.1512T>C,ccT>ccC):XP_005251170.1(p.Pro504(=))]	NA	NA	NA	5.90E-03	1.52E-03	NA	NA	NA	NA	NA	MODIFIER	NA	NA	97	95	1	109	101	6	5.5
Chewer	IOB_10	chr8	125568182	A	G	NDUFB9	INTRONIC	ENSG00000147684:[ENST00000522532(c.408+8828A>G)]	NA	NA	NA	5.90E-03	1.52E-03	NA	NA	NA	NA	NA	MODIFIER	NA	NA	97	95	1	109	101	6	5.5
Chewer	IOB_10	chr8	133187777	C	A	KCNQ3	MISSENSE	ENSG00000184156:[ENST00000388996(c.856G>T,Gtc>Ttc):ENSP00000373648(p.Val286Phe)]	NA	NA	.	8.24E-06	0.00E+00	NA	NA	NA	NA	NA	MODIFIER	NA	NA	86	85	0	147	125	21	14.29
Chewer	IOB_10	chr8	139277870	C	G	FAM135B	INTRONIC	51059:[XM_005250929.1(c.240+133G>C)]	NA	NA	NA	NA	NA	NA	NA	NA	NA	NA	MODIFIER	NA	NA	52	51	0	70	57	13	18.57
Chewer	IOB_10	chr9	34623908	G	A	ARID3C	SILENT	ENSG00000205143:[ENST00000378909(c.528C>T,ctC>ctT):ENSP00000368189(p.Leu176(=))]	NA	NA	NA	NA	NA	NA	NA	NA	NA	NA	LOW	NA	NA	125	122	0	147	102	40	27.21
Chewer	IOB_10	chr9	34659883	G	T	IL11RA	MISSENSE	ENSG00000137070:[ENST00000318041(c.938G>T,gGa>gTa):ENSP00000326500(p.Gly313Val)]	NA	NA	NA	NA	NA	NA	NA	NA	NA	NA	MODIFIER	NA	NA	49	49	0	76	61	15	19.74
Chewer	IOB_10	chr9	77749359	C	G	OSTF1	INTRONIC	ENSG00000134996:[ENST00000346234(c.408+48C>G)]	NA	NA	NA	NA	NA	NA	NA	NA	NA	NA	MODIFIER	NA	NA	44	44	0	32	20	11	34.38
Chewer	IOB_10	chr9	90219897	C	T	DAPK1	MISSENSE	ENSG00000196730:[ENST00000358077(c.91C>T,Cgt>Tgt):ENSP00000350785(p.Arg31Cys)]	NA	NA	NA	8.38E-06	0.00E+00	NA	NA	NA	NA	NA	MODIFIER	NA	NA	99	98	1	100	72	28	28
Chewer	IOB_10	chr9	90258282	A	T	DAPK1	NCT	ENSG00000196730:[ENST00000469067(n.1399-9A>T)]	NA	chr9*90258282*90258282*COSM1625207*A*T*1*liver*1|chr9*90258282*90258282*COSM1625206*A*T*1*liver*1	NA	1.42E-04	1.62E-04	NA	NA	NA	NA	NA	MODIFIER	NA	NA	84	69	1	99	37	39	39.39
Chewer	IOB_10	chr9	90502173	C	T	SPATA31E1	MISSENSE	ENSG00000177992:[ENST00000325643(c.2771C>T,cCg>cTg):ENSP00000322640(p.Pro924Leu)]	NA	NA	3.99E-03	1.18E-03	0.00E+00	3.99E-03	0.00E+00	D	BN	NA	MODERATE	N	0	221	219	0	283	207	74	26.15
Chewer	IOB_10	chr9	125391041	G	A	OR1B1	SILENT	ENSG00000171484:[ENST00000304833(c.774C>T,taC>taT):ENSP00000303151(p.Tyr258(=))]	NA	NA	2.00E-04	4.94E-05	1.82E-04	2.00E-04	0.00E+00	NA	NA	NA	LOW	NA	0	149	148	0	173	128	41	23.7
Chewer	IOB_10	chr9	125391041	G	A	RP11-64P14.7	NCT	ENSG00000234156:[ENST00000431442(n.137-8341G>A)]	NA	NA	2.00E-04	4.94E-05	1.82E-04	2.00E-04	0.00E+00	NA	NA	NA	LOW	NA	0	149	148	0	173	128	41	23.7
Chewer	IOB_10	chr9	128201126	G	C	MAPKAP1	EXONIC-NC	ENSG00000119487:[ENST00000496658(n.308C>G)]	NA	NA	NA	NA	NA	NA	NA	NA	NA	NA	MODIFIER	NA	NA	44	44	0	50	40	10	20
Chewer	IOB_10	chr9	131495703	A	C	ZER1	INTRONIC-SS-DNR	10444:[XM_005251646.1(c.2237+2T>G)]	NA	NA	NA	NA	NA	NA	NA	NA	NA	NA	HIGH	NA	NA	55	54	0	51	33	17	33.33
**Chewer**	**IOB_10**	**chr9**	**139399887**	**C**	**T**	**NOTCH1**	**NONSENSE**	ENSG00000148400:[ENST00000277541(c.4461G>A,tgG>tgA):ENSP00000277541(p.Trp1487Ter)]	NA	NA	NA	NA	NA	NA	NA	NA	NA	NA	HIGH	NA	1	266	266	0	183	104	78	42.62
Chewer	IOB_10	chrX	53657931	C	T	HUWE1	MISSENSE	ENSG00000086758:[ENST00000218328(c.818G>A,cGa>cAa):ENSP00000218328(p.Arg273Gln)]	NA	NA	NA	NA	NA	NA	NA	D	BN	NA	MODERATE	N	1	48	48	0	36	6	30	83.33
Chewer	IOB_10	chrX	55103917	G	T	PAGE2B	SILENT	ENSG00000238269:[ENST00000374971(c.279G>T,ggG>ggT):ENSP00000364110(p.Gly93(=))]	NA	chrX*55103917*55103917*COSM1636579*G*T*1*liver*1	NA	4.59E-03	6.25E-03	NA	NA	NA	NA	NA	LOW	NA	NA	104	103	0	45	35	10	22.22
Chewer	IOB_10	chrX	65817993	T	G	EDA2R	INTRONIC	60401:[NM_001324204.1(c.319-58A>C)]	NA	NA	NA	NA	NA	NA	NA	NA	NA	NA	MODIFIER	NA	NA	40	40	0	43	20	21	48.84
Chewer	IOB_10	chrX	118985458	C	T	UPF3B	MISSENSE-SS-PRX	ENSG00000125351:[ENST00000276201(c.370G>A,Ggt>Agt):ENSP00000276201(p.Gly124Ser)]	NA	NA	NA	NA	NA	NA	NA	D	PrD	NA	MODERATE	D	1	36	36	0	30	13	17	56.67
Chewer	IOB_31	chr1	1325889	C	G	CCNL2	MISSENSE	ENSG00000221978:[ENST00000400809(c.814G>C,Gaa>Caa):ENSP00000383611(p.Glu272Gln)]	NA	NA	NA	NA	NA	NA	NA	NA	NA	NA	MODIFIER	NA	NA	307	306	0	347	314	33	9.51
Chewer	IOB_31	chr1	7724116	G	A	CAMTA1	SILENT	ENSG00000171735:[ENST00000303635(c.1509G>A,ctG>ctA):ENSP00000306522(p.Leu503(=))]	NA	NA	NA	NA	NA	NA	NA	NA	NA	NA	LOW	NA	NA	425	424	0	564	383	178	31.56
Chewer	IOB_31	chr1	7724233	G	C	CAMTA1	MISSENSE	ENSG00000171735:[ENST00000303635(c.1626G>C,caG>caC):ENSP00000306522(p.Gln542His)]	NA	NA	NA	NA	NA	NA	NA	D_lc	PrD	NA	MODERATE	D	1	292	291	1	369	265	104	28.18
Chewer	IOB_31	chr1	7724512	G	C	CAMTA1	SILENT	ENSG00000171735:[ENST00000303635(c.1905G>C,ctG>ctC):ENSP00000306522(p.Leu635(=))]	NA	NA	NA	NA	NA	NA	NA	NA	NA	NA	LOW	NA	NA	290	288	0	370	253	113	30.54
Chewer	IOB_31	chr1	9085113	C	A	SLC2A7	MISSENSE	ENSG00000197241:[ENST00000400906(c.72G>T,ttG>ttT):ENSP00000383698(p.Leu24Phe)]	NA	NA	NA	NA	NA	NA	NA	D	BN	NA	MODERATE	N	0	170	168	0	201	191	7	3.48
Chewer	IOB_31	chr1	10321879	C	G	KIF1B	INTRONIC	ENSG00000054523:[ENST00000263934(c.364-84C>G)]	NA	NA	NA	NA	NA	NA	NA	NA	NA	NA	MODIFIER	NA	NA	76	76	0	81	76	5	6.17
Chewer	IOB_31	chr1	12403067	G	C	VPS13D	MISSENSE	ENSG00000048707:[ENST00000356315(c.8769G>C,gaG>gaC):ENSP00000348666(p.Glu2923Asp)]	NA	NA	NA	NA	NA	NA	NA	NA	NA	NA	MODIFIER	NA	NA	25	25	0	35	24	11	31.43
Chewer	IOB_31	chr1	12429582	C	A	VPS13D	MISSENSE	ENSG00000048707:[ENST00000356315(c.10558C>A,Ccc>Acc):ENSP00000348666(p.Pro3520Thr)]	NA	NA	NA	NA	NA	NA	NA	D	PrD	NA	MODERATE	NA	NA	193	193	0	179	172	7	3.91
Chewer	IOB_31	chr1	17682644	C	G	PADI4	INTRONIC	ENSG00000159339:[ENST00000375448(c.1455+22C>G)]	NA	NA	NA	NA	NA	NA	NA	NA	NA	NA	MODIFIER	NA	NA	187	187	0	209	192	17	8.13

Patel *et al.* , 2021. Whole-exome sequencing analysis of oral squamous cell carcinoma delineated by tobacco usage habits
Supplementary Table 4. List of somatic single nucleotide variants identified using whole exome sequencing of OSCC patients

Cohort	Sample	Chromosome	Genomic position	Reference allele	Alternate allele	Genesymbol	Variant type	Variant description	OncoMD	COSMIC	dbSNP	ExAC	ExAC_SAS	1000G	1000G_SAS	SIFT	Polyphen2	CONDEL	VEP	LRT	PhastCon7Way	Total reads in Normal	Reference read support in Normal	Alternative read support in Normal	Total reads in Tumor	Reference read support in Tumor	Alternate read support in Tumor	Alternate allele frequency in Tumor
Chewer	IOB_31	chr1	22032619	C	G	USP48	MISSENSE-SS-PRX	84196:[XM_005246012.1(c.1031G>C,aGa>aCa):XP_005246069.1(p.Arg344Thr)]	NA	chr1*22032619*22032619*COSM1339020*C*T*1*large_intestine*1	NA	NA	NA	NA	NA	D	PoD	NA	MODERATE	D	1	180	180	0	165	145	20	12.12
Chewer	IOB_31	chr1	24402664	G	A	MYOM3	SILENT	ENSG00000142661:[ENST00000329601(c.2686C>T,Ctg>Ttg):ENSP00000328415(p.Leu896(=))]	NA	NA	NA	NA	NA	NA	NA	NA	NA	NA	LOW	NA	1	162	161	0	175	163	12	6.86
Chewer	IOB_31	chr1	24402664	G	A	RP11-293P20.2	NCT	ENSG00000225315:[ENST00000439239(n.405-10576G>A)]	NA	NA	NA	NA	NA	NA	NA	NA	NA	NA	LOW	NA	1	162	161	0	175	163	12	6.86
Chewer	IOB_31	chr1	24402664	G	A	RP11-293P20.4	NCT	ENSG00000230703:[ENST00000429191(n.70-5576G>A)]	NA	NA	NA	NA	NA	NA	NA	NA	NA	NA	LOW	NA	1	162	161	0	175	163	12	6.86
Chewer	IOB_31	chr1	43891648	G	T	SZT2	NCT	ENSG00000198198:[ENST00000470139(n.1660+28G>T)]	NA	NA	NA	NA	NA	NA	NA	NA	NA	NA	MODIFIER	NA	NA	120	120	0	145	88	53	36.55
Chewer	IOB_31	chr1	54661155	C	A	CYB5RL	SILENT	ENSG00000215883:[ENST00000420054(c.90G>T,ctG>ctT):ENSP00000403021(p.Leu30(=))]	NA	NA	chr1*54661155*54661155*C*T*rs895146219*NA*NA*NA	NA	NA	NA	NA	NA	NA	NA	LOW	NA	NA	111	109	1	112	100	6	5.36
Chewer	IOB_31	chr1	54661155	C	A	MRPL37	NCT	ENSG00000116221:[ENST00000487096(n.261-9536C>A)]	NA	NA	chr1*54661155*54661155*C*T*rs895146219*NA*NA*NA	NA	NA	NA	NA	NA	NA	NA	LOW	NA	NA	111	109	1	112	100	6	5.36
Chewer	IOB_31	chr1	54661155	C	A	RP11-446E24.4	SILENT	ENSG00000256407:[ENST00000311841(c.135G>T,ctG>ctT):ENSP00000457656(p.Leu45(=))]	NA	NA	chr1*54661155*54661155*C*T*rs895146219*NA*NA*NA	NA	NA	NA	NA	NA	NA	NA	LOW	NA	NA	111	109	1	112	100	6	5.36
Chewer	IOB_31	chr1	70339170	G	T	LRRC7	INTRONIC	57554:[XM_005271060.1(c.647+35204G>T)]	NA	NA	NA	NA	NA	NA	NA	NA	NA	NA	MODIFIER	NA	NA	148	148	0	83	72	11	13.25
Chewer	IOB_31	chr1	78478992	G	A	DNAJB4	MISSENSE	ENSG00000162616:[ENST00000370763(c.469G>A,Gat>Aat):ENSP00000359799(p.Asp157Asn)]	NA	NA	NA	NA	NA	NA	NA	NA	NA	NA	MODIFIER	NA	NA	300	300	0	228	222	6	2.63
Chewer	IOB_31	chr1	78478992	G	A	GIPC2	NCT	ENSG00000137960:[ENST00000476882(n.78+33689G>A)]	NA	NA	NA	NA	NA	NA	NA	NA	NA	NA	MODIFIER	NA	NA	300	300	0	228	222	6	2.63
Chewer	IOB_31	chr1	87025931	G	C	CLCA4	MISSENSE	ENSG00000016602:[ENST00000370563(c.338G>C,aGa>aCa):ENSP00000359594(p.Arg113Thr)]	NA	chr1*87025923*87025938*COSM214460*CCCAGGTAGAGATGAA*C*1*breast*1	NA	NA	NA	NA	NA	NA	NA	NA	MODIFIER	NA	NA	270	266	0	249	229	19	7.63
Chewer	IOB_31	chr1	111893806	T	G	PIFO	NCT	ENSG00000173947:[ENST00000484512(n.2020-75T>G)]	NA	NA	NA	NA	NA	NA	NA	NA	NA	NA	MODIFIER	NA	NA	90	85	0	71	56	5	7.04
Chewer	IOB_31	chr1	144882724	G	A	PDE4DIP	NONSENSE	ENSG00000178104:[ENST00000369354(c.3295C>T,Cag>Tag):ENSP00000358360(p.Gln1099Ter)]	NA	chr1*144882724*144882724*COSM1294900*G*A*1*urinary_tract*1|chr1*144882724*144882724*COSM1294901*G*A*1*urinary_tract*1	chr1*144882724*144882724*G*T*rs782502294*NA*NA*NA	NA	NA	NA	NA	NA	NA	NA	MODIFIER	NA	NA	484	482	1	409	370	36	8.8
Chewer	IOB_31	chr1	150044214	C	G	VPS45	NCT	11311:[NR_103998.1(n.419-19C>G)]	NA	NA	NA	NA	NA	NA	NA	NA	NA	NA	MODIFIER	NA	NA	43	43	0	27	20	7	25.93
Chewer	IOB_31	chr1	156551371	C	A	TTC24	MISSENSE	ENSG00000187862:[ENST00000368236(c.215C>A,aCc>aAc):ENSP00000357219(p.Thr72Asn)]	NA	NA	chr1*156551371*156551371*C*T*rs755267652*NA*NA*NA	NA	NA	NA	NA	T	BN	NA	MODERATE	N	0	107	107	0	125	76	49	39.2
Chewer	IOB_31	chr1	158637801	C	G	SPTA1	MISSENSE	ENSG00000163554:[ENST00000368147(c.1885G>C,Gag>Cag):ENSP00000357129(p.Glu629Gln)]	NA	chr1*158637801*158637801*COSM1266734*C*A*1*oesophagus*1|chr1*158637801*158637801*COSM1720550*C*T*2*NS*2	NA	NA	NA	NA	NA	D	PrD	NA	MODERATE	N	1	172	170	0	131	90	38	29.01
Chewer	IOB_31	chr1	166889955	C	T	ILDR2	MISSENSE	ENSG00000143195:[ENST00000271417(c.1873G>A,Gcc>Acc):ENSP00000271417(p.Ala625Thr)]	NA	NA	chr1*166889955*166889955*C*A,T*rs375231605*NA*NA*NA	1.10E-04	0.00E+00	NA	NA	T	BN	NA	MODERATE	N	1	169	169	0	253	245	5	1.98
Chewer	IOB_31	chr1	168260594	C	T	TBX19	MISSENSE	ENSG00000143178:[ENST00000431969(c.197C>T,Cac>Tac):ENSP00000397540(p.His67Tyr)]	NA	NA	NA	NA	NA	NA	NA	D	PrD	NA	MODERATE	D	1	171	171	0	171	145	24	14.04
Chewer	IOB_31	chr1	169519969	G	A	F5	SILENT	ENSG00000198734:[ENST00000546081(c.894C>T,ttC>ttT):ENSP00000439664(p.Phe298(=))]	NA	NA	NA	NA	NA	NA	NA	NA	NA	NA	LOW	NA	1	106	106	0	81	51	30	37.04
Chewer	IOB_31	chr1	176918511	C	G	ASTN1	INTRONIC	ENSG00000152092:[ENST00000361833(c.1888-24G>C)]	NA	NA	NA	NA	NA	NA	NA	NA	NA	NA	MODIFIER	NA	NA	47	47	0	55	44	10	18.18
Chewer	IOB_31	chr1	202911257	G	A	ADIPOR1	NONSENSE	ENSG00000159346:[ENST00000340990(c.895C>T,Cag>Tag):ENSP00000341785(p.Gln299Ter)]	NA	NA	NA	NA	NA	NA	NA	NA	NA	NA	HIGH	NA	1	118	118	0	135	117	17	12.59
Chewer	IOB_31	chr1	206942040	C	G	IL10	MISSENSE	ENSG00000136634:[ENST00000423557(c.478G>C,Gag>Cag):ENSP00000412237(p.Glu160Gln)]	NA	NA	NA	NA	NA	NA	NA	D	PrD	NA	MODERATE	D	1	156	154	0	198	183	15	7.58
Chewer	IOB_31	chr1	215914838	C	T	USH2A	MISSENSE	ENSG00000042781:[ENST00000307340(c.11590G>A,Gaa>Aaa):ENSP00000305941(p.Glu3864Lys)]	NA	NA	NA	NA	NA	NA	NA	NA	PrD	NA	MODERATE	D	1	102	101	0	81	74	7	8.64
Chewer	IOB_31	chr1	234367284	C	A	SLC35F3	SILENT	ENSG00000183780:[ENST00000366617(c.198C>A,atC>atA):ENSP00000355576(p.Ile66(=))]	NA	NA	chr1*234367284*234367284*C*G*rs767792477*NA*NA*NA	NA	NA	NA	NA	NA	NA	NA	LOW	NA	0.001	147	146	1	196	190	6	3.06
Chewer	IOB_31	chr1	245019776	G	A	HNRNPU	MISSENSE	ENSG00000153187:[ENST00000444376(c.1838C>T,gCg>gTg):ENSP00000393151(p.Ala613Val)]	NA	NA	NA	NA	NA	NA	NA	D	PrD	NA	MODERATE	D	1	105	105	0	104	99	5	4.81
Chewer	IOB_31	chr1	247014476	G	A	AHCTF1	MISSENSE	ENSG00000153207:[ENST00000391829(c.4832C>T,tCt>tTt):ENSP00000375705(p.Ser1611Phe)]	NA	NA	NA	NA	NA	NA	NA	NA	NA	NA	MODIFIER	NA	NA	265	265	0	275	239	35	12.73
Chewer	IOB_31	chr10	91198577	A	T	SLC16A12	NONSENSE	ENSG00000152779:[ENST00000371790(c.812T>A,tTg>tAg):ENSP00000360855(p.Leu271Ter)]	NA	NA	chr10*91198577*91198577*A*C*rs759066217*NA*NA*NA	NA	NA	NA	NA	NA	NA	NA	HIGH	NA	0.319	172	172	0	216	193	23	10.65
Chewer	IOB_31	chr10	94824229	C	T	CYP26C1	MISSENSE	ENSG00000187553:[ENST00000285949(c.797C>T,gCc>gTc):ENSP00000285949(p.Ala266Val)]	NA	NA	NA	8.25E-06	6.06E-05	NA	NA	T	BN	NA	MODERATE	D	1	155	155	0	206	200	5	2.43
Chewer	IOB_31	chr10	96066409	G	T	PLCE1	MISSENSE	ENSG00000138193:[ENST00000371375(c.4924G>T,Gct>Tct):ENSP00000360426(p.Ala1642Ser)]	NA	NA	NA	NA	NA	NA	NA	NA	NA	NA	MODERATE	NA	1	72	72	0	110	91	18	16.36
Chewer	IOB_31	chr10	102683754	C	A	FAM178A	MISSENSE	ENSG00000119906:[ENST00000238961(c.996C>A,ttC>ttA):ENSP00000238961(p.Phe332Leu)]	NA	NA	chr10*102683754*102683754*C*T*rs771371903*NA*NA*NA	NA	NA	NA	NA	T	PrD	NA	MODERATE	N	0.954	56	56	0	60	54	6	10
Chewer	IOB_31	chr10	133966477	G	T	JAKMIP3	NONSENSE	ENSG00000188385:[ENST00000298622(c.2017G>T,Gag>Tag):ENSP00000298622(p.Glu673Ter)]	NA	NA	NA	NA	NA	NA	NA	NA	NA	NA	HIGH	NA	NA	90	90	0	125	116	9	7.2
Chewer	IOB_31	chr10	135170151	C	T	FUOM	MISSENSE	ENSG00000148803:[ENST00000447176(c.185G>A,gGc>gAc):ENSP00000413379(p.Gly62Asp)]	NA	NA	NA	NA	NA	NA	NA	NA	NA	NA	MODIFIER	NA	NA	107	107	0	165	159	5	3.03
Chewer	IOB_31	chr11	48349235	C	T	NA	INTERGENIC	NA	NA	NA	NA	NA	NA	NA	NA	NA	NA	NA	MODIFIER	NA	NA	83	75	7	84	63	19	22.62
Chewer	IOB_31	chr11	55321822	C	T	OR4C15	MISSENSE	ENSG00000181939:[ENST00000314644(c.40C>T,Ctt>Ttt):ENSP00000324958(p.Leu14Phe)]	NA	NA	NA	NA	NA	NA	NA	T_lc	BN	NA	MODERATE	NA	NA	140	140	0	142	133	9	6.34
Chewer	IOB_31	chr11	59345799	G	A	OSBP	MISSENSE	ENSG00000110048:[ENST00000263847(c.1883C>T,aCg>aTg):ENSP00000263847(p.Thr628Met)]	NA	NA	NA	3.33E-05	0.00E+00	NA	NA	D	PrD	NA	MODERATE	D	1	156	155	0	150	143	6	4
Chewer	IOB_31	chr11	60075645	G	A	MS4A4A	SILENT	ENSG00000110079:[ENST00000355131(c.657G>A,gaG>gaA):ENSP00000347252(p.Glu219(=))]	NA	NA	NA	NA	NA	NA	NA	NA	NA	NA	MODIFIER	NA	NA	236	235	1	304	296	7	2.3
Chewer	IOB_31	chr11	62286756	T	C	AHNAK	MISSENSE	ENSG00000124942:[ENST00000378024(c.15133A>G,Aat>Gat):ENSP00000367263(p.Asn5045Asp)]	NA	NA	NA	NA	NA	NA	NA	NA	NA	NA	MODERATE	NA	0.995	329	328	0	419	410	9	2.15
Chewer	IOB_31	chr11	62365182	C	T	MTA2	MISSENSE-SS-PRX	ENSG00000149480:[ENST00000278823(c.493G>A,Gaa>Aaa):ENSP00000278823(p.Glu165Lys)]	NA	NA	chr11*62365182*62365182*C*G*rs772969797*NA*NA*NA	NA	NA	NA	NA	NA	NA	NA	LOW	NA	NA	113	113	0	168	151	17	10.12
Chewer	IOB_31	chr11	62458116	C	T	BSCL2	MISSENSE	ENSG00000168000:[ENST00000403550(c.1010G>A,gGa>gAa):ENSP00000385561(p.Gly337Glu)]	NA	NA	chr11*62458116*62458116*C*A,T*rs767463971*NA*NA*NA	5.00E-05	0.00E+00	NA	NA	NA	NA	NA	MODIFIER	NA	NA	160	160	0	234	215	19	8.12
Chewer	IOB_31	chr11	62458116	C	T	HNRNPUL2-BSCL2	EXONIC-NC	100534595:[NR_037946.1(n.3722G>A)]	NA	NA	chr11*62458116*62458116*C*A,T*rs767463971*NA*NA*NA	5.00E-05	0.00E+00	NA	NA	NA	NA	NA	MODIFIER	NA	NA	160	160	0	234	215	19	8.12
Chewer	IOB_31	chr11	64569158	G	T	MAP4K2	NCT	ENSG00000168067:[ENST00000482314(n.386+24C>A)]	NA	NA	NA	NA	NA	NA	NA	NA	NA	NA	MODIFIER	NA	NA	154	154	0	250	236	13	5.2
Chewer	IOB_31	chr11	74556213	C	T	XRRA1	MISSENSE	ENSG00000166435:[ENST00000321448(c.983G>A,cGg>cAg):ENSP00000319303(p.Arg328Gln)]	NA	NA	NA	4.97E-05	0.00E+00	NA	NA	NA	NA	NA	MODIFIER	NA	NA	60	60	0	71	46	25	35.21
Chewer	IOB_31	chr11	77921475	G	A	USP35	SILENT	ENSG00000118369:[ENST00000529308(c.2574G>A,tcG>tcA):ENSP00000431876(p.Ser858(=))]	NA	NA	5.99E-04	3.56E-04	1.39E-03	5.99E-04	1.00E-03	NA	NA	NA	LOW	NA	NA	398	398	0	661	649	12	1.82
Chewer	IOB_31	chr11	83585489	A	G	DLG2	SILENT	ENSG00000150672:[ENST00000537455(c.486T>C,acT>acC):ENSP00000443248(p.Thr162(=))]	NA	NA	chr11*83585489*83585489*A*C*rs201862748*NA*NA*NA	NA	NA	NA	NA	NA	NA	NA	MODIFIER	NA	NA	51	51	0	48	41	7	14.58
Chewer	IOB_31	chr11	94696701	G	C	CWC15	NCT	ENSG00000150316:[ENST00000545018(n.683-22C>G)]	NA	NA	NA	NA	NA	NA	NA	NA	NA	NA	MODIFIER	NA	NA	320	320	0	294	277	14	4.76
Chewer	IOB_31	chr11	117710589	C	T	FXYD6	INTRONIC	ENSG00000137726:[ENST00000584394(c.210-44G>A)]	NA	NA	NA	8.25E-06	0.00E+00	NA	NA	NA	NA	NA	MODIFIER	NA	NA	170	169	0	195	166	29	14.87
Chewer	IOB_31	chr11	117710589	C	T	FXYD6-FXYD2	INTRONIC	ENSG00000255245:[ENST00000532984(c.223-44G>A)]	NA	NA	NA	8.25E-06	0.00E+00	NA	NA	NA	NA	NA	MODIFIER	NA	NA	170	169	0	195	166	29	14.87
Chewer	IOB_31	chr11	121028641	C	T	TECTA	MISSENSE	ENSG00000109927:[ENST00000264037(c.4397C>T,tCa>tTa):ENSP00000264037(p.Ser1466Leu)]	NA	NA	NA	NA	NA	NA	NA	T	PoD	NA	MODERATE	D	1	307	304	0	321	260	60	18.69
Chewer	IOB_31	chr11	130343246	C	T	ADAMTS15	MISSENSE	ENSG00000166106:[ENST00000299164(c.2383C>T,Cgc>Tgc):ENSP00000299164(p.Arg795Cys)]	NA	NA	2.00E-04	8.51E-06	0.00E+00	2.00E-04	0.00E+00	NA	NA	NA	MODERATE	NA	NA	173	173	0	328	319	9	2.74
Chewer	IOB_31	chr11	133801084	G	C	IGSF9B	INTRONIC	ENSG00000080854:[ENST00000321016(c.1369-55C>G)]	NA	NA	chr11*133801084*133801084*G*T*rs1038893371*NA*NA*NA	NA	NA	NA	NA	NA	NA	NA	MODIFIER	NA	NA	51	51	0	85	77	8	9.41
Chewer	IOB_31	chr12	7343876	C	G	PEX5	MISSENSE	ENSG00000139197:[ENST00000266563(c.238C>G,Cag>Gag):ENSP00000266563(p.Gln80Glu)]	NA	NA	NA	NA	NA	NA	NA	T	BN	NA	MODERATE	D	1	166	166	0	227	213	14	6.17
Chewer	IOB_31	chr12	8200740	C	A	FOXJ2	SILENT	ENSG00000065970:[ENST00000162391(c.1080C>A,ccC>ccA):ENSP00000162391(p.Pro360(=))]	NA	chr12*8200740*8200740*COSM431911*C*T*1*breast*1	chr12*8200740*8200740*C*T*rs772312089*0*0.9998*0.0001997	NA	NA	NA	NA	NA	NA	NA	LOW	NA	NA	106	104	0	143	136	6	4.2
Chewer	IOB_31	chr12	9089638	C	T	PHC1	INTRONIC	ENSG00000111752:[ENST00000543824(c.2477+78C>T)]	NA	NA	NA	NA	NA	NA	NA	NA	NA	NA	MODIFIER	NA	NA	249	249	0	312	272	39	12.5
Chewer	IOB_31	chr12	53608265	A	T	RARG	MISSENSE	ENSG00000172819:[ENST00000543726(c.535T>A,Ttc>Atc):ENSP00000444335(p.Phe179Ile)]	NA	NA	NA	NA	NA	NA	NA	D	PrD	NA	MODERATE	D	1	67	67	0	78	65	13	16.67
Chewer	IOB_31	chr12	78537150	C	T	NAV3	INTRONIC	ENSG00000067798:[ENST00000550788(c.571-2999C>T)]	NA	NA	NA	NA	NA	NA	NA	NA	NA	NA	MODIFIER	NA	NA	77	77	0	64	59	5	7.81
Chewer	IOB_31	chr12	80083572	G	C	PAWR	MISSENSE	ENSG00000177425:[ENST00000328827(c.453C>G,atC>atG):ENSP00000328088(p.Ile151Met)]	NA	NA	NA	NA	NA	NA	NA	NA	NA	NA	MODIFIER	NA	NA	112	112	0	187	168	18	9.63
Chewer	IOB_31	chr12	83290187	C	A	TMTC2	SILENT	ENSG00000179104:[ENST00000321196(c.1245C>A,gtC>gtA):ENSP00000322300(p.Val415(=))]	NA	NA	chr12*83290187*83290187*C*T*rs139737960*0*0.9998*0.0001997	NA	NA	NA	NA	NA	NA	NA	LOW	NA	NA	355	354	0	290	282	6	2.07
Chewer	IOB_31	chr12	91445323	G	C	KERA	INTRONIC	ENSG00000139330:[ENST00000266719(c.887-28C>G)]	NA	NA	NA	NA	NA	NA	NA	NA	NA	NA	MODIFIER	NA	NA	49	49	0	51	23	27	52.94
Chewer	IOB_31	chr12	116425004	G	A	MED13L	MISSENSE	ENSG00000123066:[ENST00000281928(c.4024C>T,Cgc>Tgc):ENSP00000281928(p.Arg1342Cys)]	NA	NA	NA	NA	NA	NA	NA	T	PrD	NA	MODERATE	D	1	69	69	0	74	68	6	8.11
Chewer	IOB_31	chr13	36686256	G	A	DCLK1	MISSENSE	ENSG00000133083:[ENST00000255448(c.473C>T,tCg>tTg):ENSP00000255448(p.Ser158Leu)]	NA	NA	NA	NA	NA	NA	NA	T	BN	NA	MODERATE	N	1	76	75	0	64	53	11	17.19
Chewer	IOB_31	chr13	41905430	C	T	NAA16	NONSENSE	ENSG00000172766:[ENST00000379367(c.832C>T,Caa>Taa):ENSP00000368674(p.Gln278Ter)]	NA	NA	NA	NA	NA	NA	NA	NA	NA	NA	HIGH	NA	NA	103	102	0	64	59	5	7.81

Patel *et al.* , 2021. Whole-exome sequencing analysis of oral squamous cell carcinoma delineated by tobacco usage habits
Supplementary Table 4. List of somatic single nucleotide variants identified using whole exome sequencing of OSCC patients

Cohort	Sample	Chromosome	Genomic position	Reference allele	Alternate allele	Genesymbol	Variant type	Variant description	OncoMD	COSMIC	dbSNP	ExAC	ExAC_SAS	1000G	1000G_SAS	SIFT	Polyphen2	CONDEL	VEP	LRT	PhastCon7Way	Total reads in Normal	Reference read support in Normal	Alternative read support in Normal	Total reads in Tumor	Reference read support in Tumor	Alternate read support in Tumor	Alternate allele frequency in Tumor
Chewer	IOB_31	chr13	46946147	G	A	KIAA0226L	MISSENSE	ENSG00000102445:[ENST00000417405(c.59C>T,gCc>gTc):ENSP00000402357(p.Ala20Val)]	NA	chr13*46946147*46946147*COSM1493186*G*A*1*kidney*1	NA	NA	NA	NA	NA	NA	NA	NA	MODIFIER	NA	NA	181	181	0	155	149	5	3.23
Chewer	IOB_31	chr13	46946147	G	A	PPP1R2P4	NCT	ENSG00000241353:[ENST00000535073(n.1126-3197C>T)]	NA	chr13*46946147*46946147*COSM1493186*G*A*1*kidney*1	NA	NA	NA	NA	NA	NA	NA	NA	MODIFIER	NA	NA	181	181	0	155	149	5	3.23
Chewer	IOB_31	chr13	46946147	G	A	RUBCNL	MISSENSE	80183:[XM_005266539.1(c.353C>T,gCc>gTc):XP_005266596.1(p.Ala118Val)]	NA	chr13*46946147*46946147*COSM1493186*G*A*1*kidney*1	NA	NA	NA	NA	NA	NA	NA	NA	MODIFIER	NA	NA	181	181	0	155	149	5	3.23
Chewer	IOB_31	chr14	21899180	C	T	CHD8	MISSENSE	ENSG00000100888:[ENST00000399982(c.623G>A,gGc>gAc):ENSP00000382863(p.Gly208Asp)]	NA	NA	NA	NA	NA	NA	NA	D_lc	PrD	NA	MODERATE	U	1	223	223	0	266	259	6	2.26
Chewer	IOB_31	chr14	35099332	G	C	SNX6	SILENT	ENSG00000129515:[ENST00000362031(c.27C>G,ctC>ctG):ENSP00000355217(p.Leu9(=))]	NA	NA	chr14*35099332*35099332*G*A,C*rs756508092*NA*NA*NA	2.04E-03	2.62E-03	NA	NA	NA	NA	NA	MODIFIER	NA	NA	37	37	0	50	43	6	12
Chewer	IOB_31	chr14	73425566	G	A	DCAF4	EXONIC-NC	26094:[XR_245678.1(n.1583G>A)]	NA	NA	NA	NA	NA	NA	NA	NA	NA	NA	MODIFIER	NA	NA	38	38	0	48	35	13	27.08
Chewer	IOB_31	chr14	102484897	C	T	DYNC1H1	MISSENSE	ENSG00000197102:[ENST00000360184(c.8287C>T,Cgg>Tgg):ENSP00000348965(p.Arg2763Trp)]	NA	NA	NA	NA	NA	NA	NA	D	PrD	NA	MODERATE	D	1	232	232	0	199	120	79	39.7
Chewer	IOB_31	chr14	105996034	C	T	TMEM121	MISSENSE	ENSG00000184986:[ENST00000392519(c.863C>T,cCg>cTg):ENSP00000376304(p.Pro288Leu)]	NA	NA	NA	1.17E-04	0.00E+00	NA	NA	T_lc	BN	NA	MODERATE	U	0.007	236	233	0	338	311	26	7.69
Chewer	IOB_31	chr15	25325356	C	T	SNHG14	NCT	ENSG00000224078:[ENST00000549804(n.1378-882C>T)]	NA	NA	3.99E-04	2.75E-04	6.17E-05	3.99E-04	0.00E+00	NA	NA	NA	MODIFIER	NA	NA	311	309	0	291	282	8	2.75
Chewer	IOB_31	chr15	25325356	C	T	SNORD116-14	EXONIC-NC	100033426:[NR_003329.2(n.69C>T)]	NA	NA	3.99E-04	2.75E-04	6.17E-05	3.99E-04	0.00E+00	NA	NA	NA	MODIFIER	NA	NA	311	309	0	291	282	8	2.75
Chewer	IOB_31	chr15	51504687	G	A	CYP19A1	MISSENSE	ENSG00000137869:[ENST00000260433(c.1093C>T,Cgg>Tgg):ENSP00000260433(p.Arg365Trp)]	NA	NA	2.00E-04	1.65E-05	0.00E+00	2.00E-04	0.00E+00	NA	NA	NA	MODIFIER	NA	NA	235	231	2	226	220	6	2.65
Chewer	IOB_31	chr15	51504687	G	A	RP11-108K3.1	NCT	ENSG00000259240:[ENST00000559909(n.195-65493G>A)]	NA	NA	2.00E-04	1.65E-05	0.00E+00	2.00E-04	0.00E+00	NA	NA	NA	MODIFIER	NA	NA	235	231	2	226	220	6	2.65
Chewer	IOB_31	chr15	67649755	C	G	IQCH	NONSENSE	ENSG00000103599:[ENST00000335894(c.710C>G,tCa>tGa):ENSP00000336861(p.Ser237Ter)]	NA	NA	NA	2.49E-05	0.00E+00	NA	NA	NA	NA	NA	MODIFIER	NA	NA	198	196	0	144	124	19	13.19
Chewer	IOB_31	chr15	72766858	C	T	ARIH1	5UTR	ENSG00000166233:[ENST00000379887(c.-123C>T)]	NA	NA	NA	NA	NA	NA	NA	NA	NA	NA	MODIFIER	NA	NA	66	66	0	101	81	20	19.8
Chewer	IOB_31	chr15	72766858	C	T	RP11-1007O24.3	EXONIC-NC	ENSG00000261423:[ENST00000565181(n.652G>A)]	NA	NA	NA	NA	NA	NA	NA	NA	NA	NA	MODIFIER	NA	NA	66	66	0	101	81	20	19.8
Chewer	IOB_31	chr15	74554878	A	G	CCDC33	MISSENSE	ENSG00000140481:[ENST00000321288(c.892A>G,Aat>Gat):ENSP00000325012(p.Asn298Asp)]	NA	NA	NA	NA	NA	NA	NA	T	BN	NA	MODERATE	U	0.961	72	71	1	78	66	9	11.54
Chewer	IOB_31	chr15	75093210	C	A	CSK	NONSENSE	ENSG00000103653:[ENST00000220003(c.669C>A,tgC>tgA):ENSP00000220003(p.Cys223Ter)]	NA	NA	NA	NA	NA	NA	NA	NA	NA	NA	HIGH	NA	1	174	174	0	206	200	5	2.43
Chewer	IOB_31	chr16	681406	C	G	WFIKKN1	SILENT	ENSG00000127578:[ENST00000319070(c.153C>G,cgC>cgG):ENSP00000324763(p.Arg51(=))]	NA	NA	NA	6.90E-05	0.00E+00	NA	NA	NA	NA	NA	LOW	NA	NA	91	91	0	106	85	20	18.87
Chewer	IOB_31	chr16	1537372	C	A	PTX4	SILENT	ENSG00000251692:[ENST00000447419(c.741G>T,ggG>ggT):ENSP00000445277(p.Gly247(=))]	NA	NA	NA	NA	NA	NA	NA	NA	NA	NA	LOW	NA	NA	85	85	0	126	121	5	3.97
Chewer	IOB_31	chr16	3726084	T	C	AC006111.1	EXONIC-NC	ENSG00000178430:[ENST00000323601(n.673T>C)]	NA	NA	NA	NA	NA	NA	NA	D	BN	NA	MODERATE	NA	NA	70	70	0	60	48	11	18.33
Chewer	IOB_31	chr16	3726084	T	C	DNASE1	INTRONIC	ENSG00000213918:[ENST00000575479(c.*22-3666T>C)]	NA	NA	NA	NA	NA	NA	NA	D	BN	NA	MODERATE	NA	NA	70	70	0	60	48	11	18.33
Chewer	IOB_31	chr16	3726084	T	C	NA	EXONIC-NC	101929751:[XR_243343.1(n.674T>C)]	NA	NA	NA	NA	NA	NA	NA	D	BN	NA	MODERATE	NA	NA	70	70	0	60	48	11	18.33
Chewer	IOB_31	chr16	3726084	T	C	TRAP1	MISSENSE	ENSG00000126602:[ENST00000246957(c.767A>G,cAc>cGc):ENSP00000246957(p.His256Arg)]	NA	NA	NA	NA	NA	NA	NA	D	BN	NA	MODERATE	NA	NA	70	70	0	60	48	11	18.33
Chewer	IOB_31	chr16	17235153	G	A	XYLT1	MISSENSE	ENSG00000103489:[ENST00000261381(c.1444C>T,Cgg>Tgg):ENSP00000261381(p.Arg482Trp)]	NA	NA	NA	8.24E-06	6.06E-05	NA	NA	D	PrD	NA	MODERATE	D	0.968	176	176	0	159	153	5	3.14
Chewer	IOB_31	chr16	20334201	G	A	GP2	SILENT	ENSG00000169347:[ENST00000572478(c.441C>T,ctC>ctT):ENSP00000461061(p.Leu147(=))]	NA	NA	NA	NA	NA	NA	NA	NA	NA	NA	LOW	NA	NA	108	108	0	87	76	11	12.64
Chewer	IOB_31	chr16	23223348	C	G	CTC-391G2.1	NCT	ENSG00000260741:[ENST00000563471(n.101+977G>C)]	NA	NA	NA	NA	NA	NA	NA	NA	NA	NA	LOW	NA	NA	55	55	0	63	44	19	30.16
Chewer	IOB_31	chr16	23223348	C	G	SCNN1G	INTRONIC	6340:[XM_005255469.1(c.1078-7C>G)]	NA	NA	NA	NA	NA	NA	NA	NA	NA	NA	LOW	NA	NA	55	55	0	63	44	19	30.16
Chewer	IOB_31	chr16	56536710	C	T	BBS2	MISSENSE	ENSG00000125124:[ENST00000562012(c.236G>A,cGa>cAa):ENSP00000455651(p.Arg79Gln)]	NA	chr16*56536710*56536710*COSM279129*C*T*2*large intestine,breast*1,1	NA	4.14E-05	0.00E+00	NA	NA	NA	NA	NA	MODIFIER	NA	NA	127	126	0	95	87	8	8.42
Chewer	IOB_31	chr16	57095434	C	T	NLRC5	MISSENSE	ENSG00000140853:[ENST00000543030(c.1694C>T,aCg>aTg):ENSP00000440153(p.Thr565Met)]	NA	NA	1.80E-03	6.49E-04	0.00E+00	1.80E-03	0.00E+00	T	BN	NA	MODERATE	NA	NA	233	233	0	237	230	6	2.53
Chewer	IOB_31	chr16	69791435	C	T	CTD-2033A16.2	EXONIC-NC	ENSG00000260290:[ENST00000566277(n.406G>A)]	NA	NA	NA	NA	NA	NA	NA	NA	NA	NA	MODIFIER	NA	NA	88	88	0	100	87	12	12
Chewer	IOB_31	chr16	70894087	T	C	HYDIN	MISSENSE	ENSG00000157423:[ENST00000393567(c.12013A>G,Acc>Gcc):ENSP00000377197(p.Thr4005Ala)]	NA	NA	NA	NA	NA	NA	NA	NA	NA	NA	MODIFIER	NA	NA	34	32	2	41	31	10	24.39
Chewer	IOB_31	chr17	1561689	G	A	PRPF8	INTRONIC	ENSG00000174231:[ENST00000304992(c.5377-14C>T)]	NA	NA	NA	8.24E-06	0.00E+00	NA	NA	NA	NA	NA	MODIFIER	NA	NA	221	221	0	189	184	5	2.65
Chewer	IOB_31	chr17	2227028	C	G	SRR	MISSENSE	ENSG00000167720:[ENST00000344595(c.884C>G,tCt>tGt):ENSP00000339435(p.Ser295Cys)]	NA	NA	NA	NA	NA	NA	NA	NA	NA	NA	MODIFIER	NA	NA	234	234	0	167	145	21	12.57
Chewer	IOB_31	chr17	2227028	C	G	TSR1	3UTR	ENSG00000167721:[ENST00000301364(c.*462G>C)]	NA	NA	NA	NA	NA	NA	NA	NA	NA	NA	MODIFIER	NA	NA	234	234	0	167	145	21	12.57
Chewer	IOB_31	chr17	2888260	C	T	RAP1GAP2	INTRONIC	23108:[XM_005256543.1(c.721-17C>T)]	NA	NA	NA	1.68E-05	0.00E+00	NA	NA	NA	NA	NA	MODIFIER	NA	NA	192	188	3	144	137	6	4.17
Chewer	IOB_31	chr17	3119298	C	T	OR1A1	SILENT	ENSG00000172146:[ENST00000304094(c.384C>T,cgC>cgT):ENSP00000305207(p.Arg128(=))]	NA	NA	NA	NA	NA	NA	NA	NA	NA	NA	LOW	NA	NA	256	256	0	203	122	79	38.92
Chewer	IOB_31	chr17	8053532	C	T	PER1	INTRONIC	ENSG00000179094:[ENST00000577253(c.276-89G>A)]	NA	NA	NA	3.52E-05	0.00E+00	NA	NA	NA	NA	NA	LOW	NA	NA	144	144	0	175	161	14	8
Chewer	IOB_31	chr17	11554600	G	A	DNAH9	MISSENSE	ENSG00000007174:[ENST00000262442(c.2312G>A,cGc>cAc):ENSP00000262442(p.Arg771His)]	NA	chr17*11554600*11554600*COSM1380867*G*A*4*large_intestine*4|chr17*11554600*11554600*COSM32872*G*T*1*breast*1	NA	4.14E-05	0.00E+00	NA	NA	T	BN	NA	MODERATE	N	0	129	127	0	89	54	35	39.33
Chewer	IOB_31	chr17	33504147	C	T	UNC45B	MISSENSE-SS-PRX	ENSG00000141161:[ENST00000268876(c.2143C>T,Cgg>Tgg):ENSP00000268876(p.Arg715Trp)]	NA	NA	.	1.41E-04	6.13E-05	NA	NA	D	PrD	NA	MODERATE	D	1	117	117	0	100	82	16	16
Chewer	IOB_31	chr17	42458345	G	A	ITGA2B	MISSENSE	ENSG00000005961:[ENST00000262407(c.1295C>T,tCa>tTa):ENSP00000262407(p.Ser432Leu)]	NA	NA	NA	NA	NA	NA	NA	NA	NA	NA	MODIFIER	NA	NA	325	323	1	455	419	35	7.69
Chewer	IOB_31	chr17	51901150	G	T	KIF2B	MISSENSE	ENSG00000141200:[ENST00000268919(c.756G>T,aaG>aaT):ENSP00000268919(p.Lys252Asn)]	NA	NA	NA	NA	NA	NA	NA	D	PoD	NA	MODERATE	N	1	181	181	0	226	179	46	20.35
Chewer	IOB_31	chr17	59668212	G	A	AC002994.1	EXONIC-NC	ENSG00000196861:[ENST00000447590(n.330C>T)]	NA	NA	NA	8.24E-06	6.06E-05	NA	NA	NA	NA	NA	LOW	NA	0.999	201	200	0	257	176	79	30.74
Chewer	IOB_31	chr17	59668212	G	A	NACA2	SILENT	ENSG00000253506:[ENST00000521764(c.330C>T,gaC>gaT):ENSP00000427802(p.Asp110(=))]	NA	NA	NA	8.24E-06	6.06E-05	NA	NA	NA	NA	NA	LOW	NA	0.999	201	200	0	257	176	79	30.74
Chewer	IOB_31	chr17	62009574	C	T	CD79B	SILENT	ENSG00000007312:[ENST00000006750(c.48G>A,gcG>gcA):ENSP00000006750(p.Ala16(=))]	NA	NA	chr17*62009574*62009574*C*G,T*rs758062682*NA*NA*NA	1.25E-04	5.45E-04	NA	NA	NA	NA	NA	LOW	NA	NA	142	141	0	148	111	35	23.65
Chewer	IOB_31	chr17	70119003	C	T	FLJ37644	NCT	400618:[XR_243660.1(n.1564-37817G>A)]	NA	NA	NA	NA	NA	NA	NA	NA	NA	NA	MODIFIER	NA	NA	309	307	2	402	392	7	1.74
Chewer	IOB_31	chr17	70119003	C	T	SOX9	MISSENSE	ENSG00000125398:[ENST00000245479(c.575C>T,gCc>gTc):ENSP00000245479(p.Ala192Val)]	NA	NA	NA	NA	NA	NA	NA	NA	NA	NA	MODIFIER	NA	NA	309	307	2	402	392	7	1.74
Chewer	IOB_31	chr17	70119003	C	T	SOX9-AS1	NCT	ENSG00000234899:[ENST00000414600(n.96+18823G>A)]	NA	NA	NA	NA	NA	NA	NA	NA	NA	NA	MODIFIER	NA	NA	309	307	2	402	392	7	1.74
Chewer	IOB_31	chr17	73747031	C	G	ITGB4	INTRONIC	ENSG00000132470:[ENST00000582629(c.266-6676C>G)]	NA	NA	NA	NA	NA	NA	NA	NA	NA	NA	MODIFIER	NA	NA	327	326	1	385	341	43	11.17
Chewer	IOB_31	chr17	74003539	G	A	EVPL	MISSENSE	ENSG00000167880:[ENST00000301607(c.5747C>T,tCg>tTg):ENSP00000301607(p.Ser1916Leu)]	NA	NA	NA	2.49E-05	6.22E-05	NA	NA	D	PoD	NA	MODERATE	D	1	188	183	0	299	266	26	8.7
Chewer	IOB_31	chr17	79966994	G	A	ASPSCR1	MISSENSE	ENSG00000169696:[ENST00000580534(c.784G>A,Gag>Aag):ENSP00000462329(p.Glu262Lys)]	NA	NA	NA	NA	NA	NA	NA	NA	NA	NA	MODIFIER	NA	NA	92	92	0	150	143	7	4.67
Chewer	IOB_31	chr17	79967429	G	C	ASPSCR1	MISSENSE	ENSG00000169696:[ENST00000306729(c.1156G>C,Gag>Cag):ENSP00000306625(p.Glu386Gln)]	NA	NA	NA	NA	NA	NA	NA	NA	NA	NA	MODIFIER	NA	NA	37	37	0	70	63	7	10
Chewer	IOB_31	chr18	210061	A	C	USP14	INTRONIC	ENSG00000101557:[ENST00000383589(c.1087+30A>C)]	NA	NA	NA	NA	NA	NA	NA	NA	NA	NA	MODIFIER	NA	NA	66	65	1	44	32	12	27.27
Chewer	IOB_31	chr18	9513065	C	A	RALBP1	MISSENSE	ENSG00000017797:[ENST00000019317(c.22C>A,Ccc>Acc):ENSP00000019317(p.Pro8Thr)]	NA	NA	NA	NA	NA	NA	NA	D_lc	PrD	NA	MODERATE	NA	NA	92	92	0	60	55	5	8.33
Chewer	IOB_31	chr18	12274252	C	A	CIDEA	MISSENSE	ENSG00000176194:[ENST00000320477(c.491C>A,aCg>aAg):ENSP00000320209(p.Thr164Lys)]	NA	NA	NA	NA	NA	NA	NA	T	PoD	NA	MODERATE	D	0.041	204	203	0	165	159	5	3.03
Chewer	IOB_31	chr18	34298545	C	G	FHOD3	MISSENSE	ENSG00000134775:[ENST00000445677(c.2645C>G,cCt>cGt):ENSP00000411430(p.Pro882Arg)]	NA	NA	NA	NA	NA	NA	NA	T	BN	NA	MODERATE	D	1	119	118	0	106	83	23	21.7
Chewer	IOB_31	chr18	72114226	G	A	FAM69C	MISSENSE	ENSG00000187773:[ENST00000343998(c.491C>T,tCc>tTc):ENSP00000344331(p.Ser164Phe)]	NA	NA	NA	NA	NA	NA	NA	D	BN	NA	MODERATE	N	0.277	159	157	0	210	185	24	11.43
Chewer	IOB_31	chr18	72343565	C	T	ZNF407	MISSENSE	ENSG00000215421:[ENST00000299687(c.590C>T,tCt>tTt):ENSP00000299687(p.Ser197Phe)]	NA	NA	NA	NA	NA	NA	NA	D	PrD	NA	MODERATE	U	0.576	411	409	0	287	265	22	7.67
Chewer	IOB_31	chr19	1111477	C	T	SBNO2	INTRONIC	ENSG00000064932:[ENST00000587024(c.2779+28G>A)]	NA	NA	NA	NA	NA	NA	NA	NA	NA	NA	MODIFIER	NA	NA	57	57	0	104	92	11	10.58
Chewer	IOB_31	chr19	4298269	C	G	TMIGD2	SILENT	ENSG00000167664:[ENST00000301272(c.120G>C,gcG>gcC):ENSP00000301272(p.Ala40(=))]	NA	NA	chr19*4298269*4298269*C*T*rs773648143*NA*NA*NA	NA	NA	NA	NA	NA	NA	NA	LOW	NA	NA	105	105	0	112	102	10	8.93
Chewer	IOB_31	chr19	5693555	G	A	LONP1	NCT-SS-PRX	9361:[NR_076392.1(n.2362+8C>T)]	NA	NA	2.00E-04	6.63E-05	3.64E-04	2.00E-04	0.00E+00	NA	NA	NA	LOW	NA	NA	176	176	0	267	211	56	20.97
Chewer	IOB_31	chr19	7936023	G	C	CTD-3193O13.11	EXONIC-NC	ENSG00000268120:[ENST00000597156(n.508G>C)]	NA	NA	NA	NA	NA	NA	NA	NA	NA	NA	MODIFIER	NA	NA	26	26	0	37	30	7	18.92
Chewer	IOB_31	chr19	7936023	G	C	CTD-3193O13.9	MISSENSE	ENSG00000183248:[ENST00000599142(c.351C>G,Ctc>Gtc):ENSP00000469043(p.Leu118Val)]	NA	NA	NA	NA	NA	NA	NA	NA	NA	NA	MODIFIER	NA	NA	26	26	0	37	30	7	18.92
Chewer	IOB_31	chr19	7936023	G	C	FLJ22184	MISSENSE	80164:[NM_001190467.1(c.2106C>G,Ctc>Gtc):NP_001177396.1(p.Leu703Val)]	NA	NA	NA	NA	NA	NA	NA	NA	NA	NA	MODIFIER	NA	NA	26	26	0	37	30	7	18.92
Chewer	IOB_31	chr19	7936023	G	C	PRR36	MISSENSE	80164:[NM_001190467.1(c.2104C>G,Ctc>Gtc):NP_001177396.1(p.Leu702Val)]	NA	NA	NA	NA	NA	NA	NA	NA	NA	NA	MODIFIER	NA	NA	26	26	0	37	30	7	18.92
Chewer	IOB_31	chr19	8326966	G	A	CERS4	SILENT	ENSG00000090661:[ENST00000559336(c.894G>A,ctG>ctA):ENSP00000453815(p.Leu298(=))]	NA	NA	NA	NA	NA	NA	NA	NA	NA	NA	LOW	NA	NA	115	115	0	122	92	30	24.59
Chewer	IOB_31	chr19	8979187	G	C	MUC16	NCT	94025:[XR_244090.1(n.17944+2970C>G)]	NA	NA	NA	NA	NA	NA	NA	NA	NA	NA	MODIFIER	NA	NA	123	123	0	91	82	8	8.79
Chewer	IOB_31	chr19	9018235	C	G	MUC16	NCT	94025:[XR_244090.1(n.13564-35G>C)]	NA	NA	chr19*9018235*9018235*C*A*rs758401741*NA*NA*NA	NA	NA	NA	NA	NA	NA	NA	MODIFIER	NA	NA	76	75	1	73	59	10	13.7
Chewer	IOB_31	chr19	9057530	G	C	MUC16	SILENT	ENSG00000181143:[ENST00000397910(c.29916C>G,acC>acG):ENSP00000381008(p.Thr9972(=))]	NA	NA	NA	NA	NA	NA	NA	NA	NA	NA	LOW	NA	NA	129	127	0	105	97	7	6.67
Chewer	IOB_31	chr19	12951877	G	A	HOOK2	NCT	ENSG00000095066:[ENST00000589765(n.33-14563C>T)]	NA	NA	NA	NA	NA	NA	NA	NA	NA	NA	MODIFIER	NA	NA	56	50	0	68	49	11	16.18
Chewer	IOB_31	chr19	12951877	G	A	MAST1	MISSENSE	ENSG00000105613:[ENST00000591495(c.233G>A,cGa>cAa):ENSP00000466470(p.Arg78Gln)]	NA	NA	NA	NA	NA	NA	NA	NA	NA	NA	MODIFIER	NA	NA	56	50	0	68	49	11	16.18
Chewer	IOB_31	chr19	13186412	C	G	NFIX	MISSENSE	ENSG00000008441:[ENST00000585575(c.858C>G,ttC>ttG):ENSP00000468794(p.Phe286Leu)]	NA	NA	NA	NA	NA	NA	NA	T	PrD	NA	MODERATE	D	1	298	298	0	324	288	36	11.11
Chewer	IOB_31	chr19	15918868	G	C	OR10H1	5UTR	ENSG00000186723:[ENST00000334920(c.-21C>G)]	NA	NA	chr19*15918868*15918868*G*T*rs779812360*NA*NA*NA	NA	NA	NA	NA	NA	NA	NA	MODIFIER	NA	NA	74	74	0	64	49	15	23.44

Patel *et al.* , 2021. Whole-exome sequencing analysis of oral squamous cell carcinoma delineated by tobacco usage habits
Supplementary Table 4. List of somatic single nucleotide variants identified using whole exome sequencing of OSCC patients

Cohort	Sample	Chromosome	Genomic position	Reference allele	Alternate allele	Genesymbol	Variant type	Variant description	OncoMD	COSMIC	dbSNP	ExAC	ExAC_SAS	1000G	1000G_SAS	SIFT	Polyphen2	CONDEL	VEP	LRT	PhastCon7Way	Total reads in Normal	Reference read support in Normal	Alternative read support in Normal	Total reads in Tumor	Reference read support in Tumor	Alternate read support in Tumor	Alternate allele frequency in Tumor
Chewer	IOB_31	chr19	18507438	C	T	LRRC25	SILENT	ENSG00000175489:[ENST00000339007(c.336G>A,ctG>ctA):ENSP00000340983(p.Leu112(=))]	NA	NA	chr19*18507438*18507438*C*A*rs748187223*NA*NA*NA	NA	NA	NA	NA	NA	NA	NA	LOW	NA	NA	190	190	0	278	271	6	2.16
Chewer	IOB_31	chr19	33490500	C	T	RHPN2	MISSENSE	ENSG00000131941:[ENST00000400226(c.764G>A,cGa>cAa):ENSP00000402244(p.Arg255Gln)]	NA	chr19*33490500*33490500*COSM140214*C*T*2*breast.skin*1.1	NA	7.46E-05	0.00E+00	NA	NA	T	BN	NA	MODERATE	N	0	43	42	0	46	39	7	15.22
Chewer	IOB_31	chr19	38720177	C	G	DPF1	INTRONIC	ENSG00000011332:[ENST00000412732(c.-132+22G>C)]	NA	NA	NA	NA	NA	NA	NA	NA	NA	NA	MODIFIER	NA	NA	13	12	1	55	46	6	10.91
Chewer	IOB_31	chr19	38976717	C	T	RYR1	MISSENSE	ENSG00000196218:[ENST00000355481(c.5422C>T,Cgg>Tgg):ENSP00000347667(p.Arg1808Trp)]	NA	chr19*38976717*38976717*COSM3378933*C*T*1*pancreas*1	NA	8.76E-06	0.00E+00	NA	NA	NA	PoD	NA	MODERATE	U	0.503	161	161	0	200	195	5	2.5
Chewer	IOB_31	chr19	46640200	G	T	AC007193.6	NCT	ENSG00000267922:[ENST00000597989(n.16+35805G>T)]	NA	NA	NA	NA	NA	NA	NA	NA	NA	NA	MODIFIER	NA	NA	187	187	0	213	205	6	2.82
Chewer	IOB_31	chr19	46640200	G	T	TGIF1P1	EXONIC-NC	ENSG00000259339:[ENST00000558095(n.605C>A)]	NA	NA	NA	NA	NA	NA	NA	NA	NA	NA	MODIFIER	NA	NA	187	187	0	213	205	6	2.82
Chewer	IOB_31	chr19	50863138	G	C	NAPSA	NCT	ENSG00000131400:[ENST00000599181(n.1904-19C>G)]	NA	NA	chr19*50863136*50863138*TAG*T*rs773704054*NA*NA*NA|chr19*50863138*50863138*G*A*rs758116701*NA*NA*NA	NA	NA	NA	NA	NA	NA	NA	MODIFIER	NA	NA	178	177	0	164	73	90	54.88
Chewer	IOB_31	chr19	50863138	G	C	NR1H2	NCT	ENSG00000131408:[ENST00000600978(n.150-11474G>C)]	NA	NA	chr19*50863136*50863138*TAG*T*rs773704054*NA*NA*NA|chr19*50863138*50863138*G*A*rs758116701*NA*NA*NA	NA	NA	NA	NA	NA	NA	NA	MODIFIER	NA	NA	178	177	0	164	73	90	54.88
Chewer	IOB_31	chr19	52937315	C	A	ZNF534	NONSENSE	ENSG00000198633:[ENST00000301085(c.123C>A,taC>taA):ENSP00000301085(p.Tyr41Ter)]	NA	NA	NA	NA	NA	NA	NA	NA	NA	NA	HIGH	NA	NA	183	181	1	135	110	25	18.52
Chewer	IOB_31	chr19	55594824	G	A	EPS8L1	SILENT	ENSG00000131037:[ENST00000201647(c.1293G>A,ccG>ccA):ENSP00000201647(p.Pro431(=))]	NA	NA	NA	NA	NA	NA	NA	NA	NA	NA	LOW	NA	NA	156	156	0	249	243	5	2.01
Chewer	IOB_31	chr2	27280181	G	C	AGBL5	MISSENSE	ENSG00000084693:[ENST00000323064(c.1546G>C,Gaa>Caa):ENSP00000323681(p.Glu516Gln)]	NA	NA	NA	NA	NA	NA	NA	NA	NA	NA	MODIFIER	NA	NA	150	150	0	132	117	14	10.61
Chewer	IOB_31	chr2	30379665	G	A	YPEL5	NCT-SS-PRX	ENSG00000119801:[ENST00000492439(n.658+7G>A)]	NA	NA	NA	NA	NA	NA	NA	NA	NA	NA	LOW	NA	NA	196	195	0	164	145	18	10.98
Chewer	IOB_31	chr2	47300918	G	A	C2orf61	NCT	ENSG00000239605:[ENST00000496939(n.416-26860C>T)]	NA	NA	2.00E-04	4.15E-05	0.00E+00	2.00E-04	0.00E+00	NA	NA	NA	LOW	NA	NA	158	158	0	210	189	20	9.52
Chewer	IOB_31	chr2	47300918	G	A	RP11-761B3.1	INTRONIC	ENSG00000273269:[ENST00000422269(c.*656-7642C>T)]	NA	NA	2.00E-04	4.15E-05	0.00E+00	2.00E-04	0.00E+00	NA	NA	NA	LOW	NA	NA	158	158	0	210	189	20	9.52
Chewer	IOB_31	chr2	47300918	G	A	TTC7A	SILENT	ENSG00000068724:[ENST00000394850(c.2505G>A,acG>acA):ENSP00000378320(p.Thr835(=))]	NA	NA	2.00E-04	4.15E-05	0.00E+00	2.00E-04	0.00E+00	NA	NA	NA	LOW	NA	NA	158	158	0	210	189	20	9.52
Chewer	IOB_31	chr2	58374319	A	G	VRK2	INTRONIC	7444:[NM_001288839.1(c.828+710A>G)]	NA	NA	NA	NA	NA	NA	NA	NA	NA	NA	MODIFIER	NA	NA	191	191	0	136	125	11	8.09
Chewer	IOB_31	chr2	91933124	G	T	AC027612.4	EXONIC-NC	ENSG00000223703:[ENST00000445234(n.1041G>T)]	NA	NA	NA	NA	NA	NA	NA	NA	NA	NA	MODIFIER	NA	NA	212	210	1	203	176	27	13.3
Chewer	IOB_31	chr2	100623283	C	T	AFF3	SILENT	ENSG00000144218:[ENST00000356421(c.759G>A,caG>caA):ENSP00000348793(p.Gln253(=))]	NA	NA	NA	NA	NA	NA	NA	NA	NA	NA	LOW	NA	1	282	282	0	306	298	6	1.96
Chewer	IOB_31	chr2	152684612	C	A	ARL5A	INTRONIC	ENSG00000162980:[ENST00000428992(c.-66+291G>T)]	NA	NA	NA	NA	NA	NA	NA	NA	NA	NA	MODIFIER	NA	NA	183	181	0	265	257	5	1.89
Chewer	IOB_31	chr2	166535753	C	T	CSRNP3	SILENT	ENSG00000178662:[ENST00000409420(c.1344C>T,acC>acT):ENSP00000387195(p.Thr448(=))]	NA	NA	NA	NA	NA	NA	NA	NA	NA	NA	LOW	NA	NA	244	244	0	180	167	12	6.67
Chewer	IOB_31	chr2	170411669	C	G	FASTKD1	MISSENSE	ENSG00000138399:[ENST00000453153(c.1179G>C,aaG>aaC):ENSP00000400513(p.Lys393Asn)]	NA	NA	chr2*170411669*170411669*C*T*rs772347512*NA*NA*NA	NA	NA	NA	NA	NA	NA	NA	MODERATE	NA	1	33	33	0	52	47	5	9.62
Chewer	IOB_31	chr2	179428018	G	A	TTN	MISSENSE	ENSG00000155657:[ENST00000342992(c.75137C>T,gCg>gTg):ENSP00000343764(p.Ala25046Val)]	NA	NA	NA	8.30E-06	0.00E+00	NA	NA	NA	NA	NA	MODIFIER	NA	NA	251	251	0	249	177	69	27.71
Chewer	IOB_31	chr2	179428018	G	A	TTN-AS1	NCT	ENSG00000237298:[ENST00000586452(n.75-8009G>A)]	NA	NA	NA	8.30E-06	0.00E+00	NA	NA	NA	NA	NA	MODIFIER	NA	NA	251	251	0	249	177	69	27.71
Chewer	IOB_31	chr2	179476568	T	C	TTN	MISSENSE	ENSG00000155657:[ENST00000591111(c.45545A>G,cAg>cGg):ENSP00000465570(p.Gln15182Arg)]	NA	NA	NA	NA	NA	NA	NA	NA	PrD	NA	MODERATE	NA	NA	243	243	0	210	197	13	6.19
Chewer	IOB_31	chr2	179476568	T	C	TTN-AS1	NCT	ENSG00000237298:[ENST00000589487(n.110+11174T>C)]	NA	NA	NA	NA	NA	NA	NA	NA	PrD	NA	MODERATE	NA	NA	243	243	0	210	197	13	6.19
Chewer	IOB_31	chr2	179560763	C	A	TTN	NONSENSE	ENSG00000155657:[ENST00000589042(c.31036G>T,Gaa>Taa):ENSP00000467141(p.Glu10346Ter)]	NA	NA	NA	NA	NA	NA	NA	NA	NA	NA	MODIFIER	NA	NA	290	289	0	212	141	69	32.55
Chewer	IOB_31	chr2	179560763	C	A	TTN-AS1	NCT	ENSG00000237298:[ENST00000592630(n.520-17718C>A)]	NA	NA	NA	NA	NA	NA	NA	NA	NA	NA	MODIFIER	NA	NA	290	289	0	212	141	69	32.55
Chewer	IOB_31	chr2	196749270	C	T	DNAH7	INTRONIC	ENSG00000118997:[ENST00000312428(c.5763+39G>A)]	NA	NA	chr2*196749270*196749270*C*A*rs957221266*NA*NA*NA	NA	NA	NA	NA	NA	NA	NA	MODIFIER	NA	NA	98	97	1	111	91	18	16.22
Chewer	IOB_31	chr2	202436731	G	A	ALS2CR11	NONSENSE	ENSG00000155754:[ENST00000286195(c.766C>T,Cag>Tag):ENSP00000286195(p.Gln256Ter)]	NA	NA	NA	NA	NA	NA	NA	NA	NA	NA	HIGH	NA	1	143	142	0	97	84	12	12.37
Chewer	IOB_31	chr2	209189776	G	T	PIKFYVE	INTRONIC	ENSG00000115020:[ENST00000264380(c.2458+15G>T)]	NA	NA	NA	NA	NA	NA	NA	NA	NA	NA	MODIFIER	NA	NA	201	200	0	165	104	61	36.97
Chewer	IOB_31	chr2	212530155	G	T	ERBB4	MISSENSE	ENSG00000178568:[ENST00000342788(c.1764C>A,aaC>aaA):ENSP00000342235(p.Asn588Lys)]	NA	NA	NA	NA	NA	NA	NA	NA	NA	NA	MODIFIER	NA	NA	338	329	3	250	221	28	11.2
Chewer	IOB_31	chr2	219110335	C	T	ARPC2	INTRONIC	ENSG00000163466:[ENST00000456575(c.121+66C>T)]	NA	NA	NA	NA	NA	NA	NA	NA	NA	NA	MODIFIER	NA	NA	89	89	0	82	60	22	26.83
Chewer	IOB_31	chr2	219757626	G	A	WNT10A	MISSENSE	ENSG00000135925:[ENST00000258411(c.887G>A,cGc>cAc):ENSP00000258411(p.Arg296His)]	NA	NA	NA	NA	NA	NA	NA	T	BN	NA	MODERATE	N	0.95	137	137	0	184	165	19	10.33
Chewer	IOB_31	chr2	220506403	G	A	SLC4A3	MISSENSE	ENSG00000114923:[ENST00000273063(c.3733G>A,Gat>Aat):ENSP00000273063(p.Asp1245Asn)]	NA	chr2*220506403*220506403*COSM1016954*G*A*2*endometrium*2	NA	NA	NA	NA	NA	D	PoD	NA	MODERATE	D	1	138	138	0	167	151	16	9.58
Chewer	IOB_31	chr20	419843	C	T	TBC1D20	MISSENSE	ENSG00000125875:[ENST00000354200(c.865G>A,Gag>Aag):ENSP00000346139(p.Glu289Lys)]	NA	NA	NA	NA	NA	NA	NA	D	PrD	NA	MODERATE	NA	NA	291	291	0	255	185	66	25.88
Chewer	IOB_31	chr20	31596435	G	T	BPIFB2	MISSENSE	ENSG00000078898:[ENST00000170150(c.55G>T,Ggt>Tgt):ENSP00000170150(p.Gly19Cys)]	NA	NA	.	NA	NA	NA	NA	T	BN	NA	MODERATE	N	0	130	130	0	114	85	28	24.56
Chewer	IOB_31	chr20	33584178	G	A	MYH7B	SILENT	ENSG00000078814:[ENST00000262873(c.3099G>A,gcG>gcA):ENSP00000262873(p.Ala1033(=))]	NA	NA	NA	5.73E-05	0.00E+00	NA	NA	NA	NA	NA	LOW	NA	NA	87	87	0	94	72	21	22.34
Chewer	IOB_31	chr20	51871107	G	A	TSHZ2	NONSENSE	ENSG00000182463:[ENST00000329613(c.1101G>A,tgG>tgA):ENSP00000333114(p.Trp367Ter)]	NA	NA	NA	NA	NA	NA	NA	NA	NA	NA	HIGH	NA	1	139	138	1	197	191	5	2.54
Chewer	IOB_31	chr20	60427875	C	T	CDH4	SILENT	ENSG00000179242:[ENST00000360469(c.798C>T,taC>taT):ENSP00000353656(p.Tyr266(=))]	NA	NA	NA	2.47E-05	6.06E-05	NA	NA	NA	NA	NA	LOW	NA	0	255	254	1	304	287	17	5.59
Chewer	IOB_31	chr20	61429868	C	T	MRGBP	INTRONIC	ENSG00000101189:[ENST00000370487(c.271-71C>T)]	NA	NA	NA	NA	NA	NA	NA	NA	NA	NA	MODIFIER	NA	NA	142	142	0	211	196	14	6.64
Chewer	IOB_31	chr20	62365034	G	A	RP4-583P15.15	MISSENSE	ENSG00000273154:[ENST00000490623(c.531G>A,Gag>Aag):ENSP00000476393(p.Glu178Lys)]	NA	NA	chr20*62365034*62365034*G*C*rs200268567*NA*NA*NA	NA	NA	NA	NA	D	BN	NA	MODERATE	NA	NA	349	348	0	340	316	23	6.76
Chewer	IOB_31	chr20	62365034	G	A	ZGPAT	MISSENSE	ENSG00000197114:[ENST00000328969(c.814G>A,Gag>Aag):ENSP00000332013(p.Glu272Lys)]	NA	NA	chr20*62365034*62365034*G*C*rs200268567*NA*NA*NA	NA	NA	NA	NA	D	BN	NA	MODERATE	NA	NA	349	348	0	340	316	23	6.76
Chewer	IOB_31	chr21	15554084	G	C	LIPI	MISSENSE	ENSG00000188992:[ENST00000536861(c.638C>G,tCc>tGc):ENSP00000440381(p.Ser213Cys)]	NA	NA	NA	NA	NA	NA	NA	D	PoD	NA	MODERATE	N	0	81	80	0	62	55	7	11.29
Chewer	IOB_31	chr21	19666630	C	T	TMPRSS15	MISSENSE	ENSG00000154646:[ENST00000284885(c.2443G>A,Gtc>Atc):ENSP00000284885(p.Val815Ile)]	NA	NA	2.00E-04	4.94E-05	6.06E-05	2.00E-04	0.00E+00	T	BN	NA	MODERATE	N	0.005	98	98	0	81	57	23	28.4
Chewer	IOB_31	chr22	28379153	C	G	TTC28	MISSENSE	ENSG00000100154:[ENST00000397906(c.6502G>C,Gag>Cag):ENSP00000381003(p.Glu2168Gln)]	NA	NA	NA	NA	NA	NA	NA	NA	NA	NA	MODIFIER	NA	NA	159	156	2	171	153	18	10.53
Chewer	IOB_31	chr22	28379153	C	G	TTC28-AS1	NCT	ENSG00000235954:[ENST00000424161(n.155-11308C>G)]	NA	NA	NA	NA	NA	NA	NA	NA	NA	NA	MODIFIER	NA	NA	159	156	2	171	153	18	10.53
Chewer	IOB_31	chr22	30781942	C	A	RNF215	MISSENSE	ENSG00000099999:[ENST00000382363(c.538G>T,Gtc>Ttc):ENSP00000371800(p.Val180Phe)]	NA	NA	chr22*30781942*30781942*C*G,T*rs751258397*NA*NA*NA	NA	NA	NA	NA	D	PrD	NA	MODERATE	NA	NA	161	161	0	182	177	5	2.75
Chewer	IOB_31	chr22	32346271	A	T	YWHAH	INTRONIC	7533:[XM_005261731.1(c.-124+5050A>T)]	NA	NA	NA	NA	NA	NA	NA	NA	NA	NA	MODIFIER	NA	NA	142	142	0	111	105	5	4.5
Chewer	IOB_31	chr22	32620351	C	G	RP1-90G24.10	NCT	ENSG00000242082:[ENST00000434942(n.274+17088C>G)]	NA	NA	NA	NA	NA	NA	NA	D	BN	NA	MODERATE	D	0.999	137	136	0	121	116	5	4.13
Chewer	IOB_31	chr22	32620351	C	G	SLC5A4	MISSENSE	ENSG00000100191:[ENST00000266086(c.1568G>C,gGa>gCa):ENSP00000266086(p.Gly523Ala)]	NA	NA	NA	NA	NA	NA	NA	D	BN	NA	MODERATE	D	0.999	137	136	0	121	116	5	4.13
Chewer	IOB_31	chr22	50968830	G	A	ODF3B	EXONIC-NC	ENSG00000177989:[ENST00000468249(n.1031C>T)]	NA	NA	chr22*50968826*50968830*CAGAG*C*rs369717217*NA*NA*NA	NA	NA	NA	NA	NA	NA	NA	MODIFIER	NA	NA	192	190	0	286	212	72	25.17
Chewer	IOB_31	chr22	51169697	G	A	SHANK3	MISSENSE	ENSG00000251322:[ENST00000445220(c.5186G>A,cGc>cAc):ENSP00000446078(p.Arg1729His)]	NA	NA	NA	NA	NA	NA	NA	D	PrD	NA	MODERATE	NA	NA	107	106	1	169	163	5	2.96
Chewer	IOB_31	chr3	17109586	T	G	PLCL2	INTRONIC	23228:[NM_001144382.1(c.2879+29T>G)]	NA	NA	NA	NA	NA	NA	NA	NA	NA	NA	MODIFIER	NA	NA	230	230	0	145	92	52	35.86
Chewer	IOB_31	chr3	29922177	C	A	RBMS3	INTRONIC	27303:[XM_005265065.1(c.418-3476C>A)]	NA	NA	NA	NA	NA	NA	NA	NA	NA	NA	MODIFIER	NA	NA	202	202	0	160	153	6	3.75
Chewer	IOB_31	chr3	36779074	G	A	DCLK3	SILENT	ENSG00000163673:[ENST00000416516(c.1077C>T,ggC>ggT):ENSP00000394484(p.Gly359(=))]	NA	NA	NA	NA	NA	NA	NA	NA	NA	NA	LOW	NA	NA	246	246	0	201	194	5	2.49
Chewer	IOB_31	chr3	47455828	C	T	SCAP	MISSENSE	ENSG00000114650:[ENST00000441517(c.2668G>A,Gat>Aat):ENSP00000416847(p.Asp890Asn)]	NA	NA	NA	NA	NA	NA	NA	D	PrD	NA	MODERATE	D	1	147	144	0	148	91	57	38.51
Chewer	IOB_31	chr3	52130755	C	G	POC1A	INTRONIC	ENSG00000164087:[ENST00000394970(c.982-20754G>C)]	NA	NA	NA	NA	NA	NA	NA	NA	NA	NA	MODIFIER	NA	NA	39	38	1	45	39	6	13.33
Chewer	IOB_31	chr3	52835146	C	T	ITIH3	MISSENSE	ENSG00000162267:[ENST00000416872(c.1367C>T,gCc>gTc):ENSP00000413922(p.Ala456Val)]	NA	NA	NA	9.95E-06	0.00E+00	NA	NA	NA	NA	NA	MODIFIER	NA	NA	67	67	0	70	64	5	7.14
Chewer	IOB_31	chr3	100549415	C	T	ABI3BP	INTRONIC	ENSG00000154175:[ENST00000533795(c.520+1693G>A)]	NA	NA	NA	NA	NA	NA	NA	NA	NA	NA	LOW	NA	NA	73	73	0	47	40	7	14.89
Chewer	IOB_31	chr3	108746698	C	T	MORC1	MISSENSE	ENSG00000114487:[ENST00000232603(c.1604G>A,gGc>gAc):ENSP00000232603(p.Gly535Asp)]	NA	NA	NA	1.65E-05	6.06E-05	NA	NA	NA	NA	NA	MODERATE	NA	1	185	183	0	138	124	14	10.14
Chewer	IOB_31	chr3	118621721	G	C	IGSF11	SILENT	ENSG00000144847:[ENST00000354673(c.939C>G,tcC>tcG):ENSP00000346700(p.Ser313(=))]	NA	NA	chr3*118621721*118621721*G*A*rs753092282*NA*NA*NA	NA	NA	NA	NA	NA	NA	NA	LOW	NA	NA	426	425	0	358	345	13	3.63
Chewer	IOB_31	chr3	121351266	C	G	HCLS1	MISSENSE	ENSG00000180353:[ENST00000428394(c.1042G>C,Gac>Cac):ENSP00000387645(p.Asp348His)]	NA	NA	chr3*121351266*121351266*C*A*rs764855451*NA*NA*NA	NA	NA	NA	NA	D	BN	NA	MODERATE	N	0	191	190	1	207	156	51	24.64
Chewer	IOB_31	chr3	130104070	C	A	COL6A5	MISSENSE	ENSG00000172752:[ENST00000265379(c.1724C>A,gCa>gAa):ENSP00000265379(p.Ala575Glu)]	NA	NA	NA	NA	NA	NA	NA	D	PrD	NA	MODERATE	NA	NA	177	175	1	132	126	5	3.79
Chewer	IOB_31	chr3	133327472	C	A	TOPBP1	SILENT	ENSG00000163781:[ENST00000260810(c.4332G>T,ctG>ctT):ENSP00000260810(p.Leu1444(=))]	NA	NA	NA	NA	NA	NA	NA	NA	NA	NA	LOW	NA	NA	235	234	1	164	158	6	3.66
Chewer	IOB_31	chr3	137786492	C	A	DZIP1L	MISSENSE	ENSG00000158163:[ENST00000327532(c.1883G>T,cGt>cTt):ENSP00000332148(p.Arg628Leu)]	NA	NA	chr3*137786492*137786492*C*T*rs111285595*NA*NA*NA	NA	NA	NA	NA	NA	NA	NA	MODERATE	NA	0	64	64	0	102	83	19	18.63
Chewer	IOB_31	chr3	149508679	T	C	ANKUB1	MISSENSE	ENSG00000206199:[ENST00000484019(c.95A>G,aAc>aGc):ENSP00000420428(p.Asn32Ser)]	NA	NA	NA	NA	NA	NA	NA	NA	NA	NA	LOW	NA	0.985	299	298	1	296	284	12	4.05
Chewer	IOB_31	chr3	167035281	C	G	ZBBX	MISSENSE	ENSG00000169064:[ENST00000307529(c.1088G>C,aGt>aCt):ENSP00000305065(p.Ser363Thr)]	NA	NA	NA	NA	NA	NA	NA	NA	NA	NA	MODERATE	NA	0.008	136	134	0	90	73	17	18.89

Patel *et al.* , 2021. Whole-exome sequencing analysis of oral squamous cell carcinoma delineated by tobacco usage habits
Supplementary Table 4. List of somatic single nucleotide variants identified using whole exome sequencing of OSCC patients

Cohort	Sample	Chromosome	Genomic position	Reference allele	Alternate allele	Genesymbol	Variant type	Variant description	OncoMD	COSMIC	dbSNP	ExAC	ExAC_SAS	1000G	1000G_SAS	SIFT	Polyphen2	CONDEL	VEP	LRT	PhastCon7Way	Total reads in Normal	Reference read support in Normal	Alternative read support in Normal	Total reads in Tumor	Reference read support in Tumor	Alternate read support in Tumor	Alternate allele frequency in Tumor
Chewer	IOB_31	chr3	167245723	A	T	WDR49	MISSENSE	ENSG00000174776:[ENST00000308378(c.1433T>A,aTa>aAa):ENSP00000311343(p.Ile478Lys)]	NA	NA	NA	NA	NA	NA	NA	NA	NA	NA	MODIFIER	NA	NA	46	46	0	68	59	9	13.24
Chewer	IOB_31	chr3	195968514	G	A	PCYT1A	INTRONIC	ENSG00000161217:[ENST00000292823(c.708+305C>T)]	NA	NA	NA	NA	NA	NA	NA	NA	NA	NA	MODIFIER	NA	NA	179	179	0	113	107	6	5.31
Chewer	IOB_31	chr3	195968514	G	A	SLC51A	INTRONIC	ENSG00000163959:[ENST00000415111(c.58-851G>A)]	NA	NA	NA	NA	NA	NA	NA	NA	NA	NA	MODIFIER	NA	NA	179	179	0	113	107	6	5.31
Chewer	IOB_31	chr3	196675397	G	A	PIGZ	MISSENSE	ENSG00000119227:[ENST00000412723(c.371C>T,gCg>gTg):ENSP00000413405(p.Ala124Val)]	NA	NA	2.40E-03	5.27E-03	2.84E-03	2.40E-03	2.00E-03	NA	NA	NA	MODIFIER	NA	NA	153	153	0	225	163	62	27.56
Chewer	IOB_31	chr4	7705019	G	C	SORCS2	MISSENSE	ENSG00000184985:[ENST00000329016(c.1165G>C,Gag>Cag):ENSP00000329124(p.Glu389Gln)]	NA	NA	NA	NA	NA	NA	NA	D	PrD	NA	MODERATE	D	1	219	218	1	217	177	39	17.97
Chewer	IOB_31	chr4	16507523	C	T	LDB2	MISSENSE	ENSG00000169744:[ENST00000441778(c.974G>A,gGc>gAc):ENSP00000392089(p.Gly325Asp)]	NA	NA	NA	NA	NA	NA	NA	D_lc	BN	NA	MODERATE	NA	NA	177	177	0	133	127	5	3.76
Chewer	IOB_31	chr4	16507523	C	T	RP11-446J8.1	NCT	ENSG00000248138:[ENST00000512370(n.181-6033C>T)]	NA	NA	NA	NA	NA	NA	NA	D_lc	BN	NA	MODERATE	NA	NA	177	177	0	133	127	5	3.76
Chewer	IOB_31	chr4	42145621	C	T	BEND4	NONSENSE	ENSG00000188848:[ENST00000504360(c.866G>A,tGg>tAg):ENSP00000421160(p.Trp289Ter)]	NA	NA	NA	NA	NA	NA	NA	NA	NA	NA	HIGH	NA	1	192	190	0	180	160	19	10.56
Chewer	IOB_31	chr4	79340066	T	A	FRAS1	INTRONIC	ENSG00000138759:[ENST00000264895(c.4426-37T>A)]	NA	NA	NA	NA	NA	NA	NA	NA	NA	NA	MODIFIER	NA	NA	158	157	0	101	76	25	24.75
Chewer	IOB_31	chr4	129208978	G	C	PGRMC2	MISSENSE	ENSG00000164040:[ENST00000520121(c.40C>G,Cgg>Ggg):ENSP00000429301(p.Arg14Gly)]	NA	NA	chr4*129208970*129208986*TCCTCCCCGCCCCCTGC*T*rs755804339*NA*NA*NA	NA	NA	NA	NA	NA	UN	NA	MODERATE	U	1	46	44	1	53	43	5	9.43
Chewer	IOB_31	chr4	145636491	G	T	HHIP	MISSENSE	ENSG00000164161:[ENST00000296575(c.1587G>T,caG>caT):ENSP00000296575(p.Gln529His)]	NA	NA	NA	NA	NA	NA	NA	NA	NA	NA	MODIFIER	NA	NA	145	145	0	132	115	17	12.88
Chewer	IOB_31	chr4	175577907	C	A	GLRA3	MISSENSE	ENSG00000145451:[ENST00000274093(c.1103G>T,cGt>cTt):ENSP00000274093(p.Arg368Leu)]	NA	NA	chr4*175577907*175577907*C*T*rs142149685*0*0.9996*0.0003994	NA	NA	NA	NA	T	BN	NA	MODERATE	U	1	139	139	0	133	127	6	4.51
Chewer	IOB_31	chr5	871573	C	G	BRD9	INTRONIC	ENSG00000028310:[ENST00000490814(c.*1387+68G>C)]	NA	NA	NA	NA	NA	NA	NA	NA	NA	NA	MODIFIER	NA	NA	144	143	1	196	177	18	9.18
Chewer	IOB_31	chr5	7835560	C	A	C5orf49	MISSENSE	ENSG00000215217:[ENST00000399810(c.199G>T,Gat>Tat):ENSP00000382708(p.Asp67Tyr)]	NA	NA	NA	NA	NA	NA	NA	D	PoD	NA	MODERATE	NA	NA	153	152	0	181	176	5	2.76
Chewer	IOB_31	chr5	32057827	C	A	PDZD2	MISSENSE	ENSG00000133401:[ENST00000282493(c.1967C>A,aCc>aAc):ENSP00000282493(p.Thr656Asn)]	NA	NA	NA	NA	NA	NA	NA	D	PrD	NA	MODERATE	NA	NA	88	88	0	97	92	5	5.15
Chewer	IOB_31	chr5	33984266	A	G	SLC45A2	NCT	ENSG00000164175:[ENST00000505056(n.364+38T>C)]	NA	NA	NA	NA	NA	NA	NA	NA	NA	NA	MODIFIER	NA	NA	126	125	0	131	95	36	27.48
Chewer	IOB_31	chr5	34818920	C	T	RAI14	NONSENSE	ENSG00000039560:[ENST00000397449(c.937C>T,Cga>Tga):ENSP00000380591(p.Arg313Ter)]	NA	NA	NA	NA	NA	NA	NA	NA	NA	NA	HIGH	NA	NA	47	47	0	53	46	7	13.21
Chewer	IOB_31	chr5	34914091	G	C	RAD1	NONSENSE	ENSG00000113456:[ENST00000511456(c.239C>G,tCa>tGa):ENSP00000423253(p.Ser80Ter)]	NA	NA	NA	NA	NA	NA	NA	NA	NA	NA	MODIFIER	NA	NA	157	156	0	199	186	13	6.53
Chewer	IOB_31	chr5	39202786	C	T	FYB	MISSENSE	ENSG00000082074:[ENST00000351578(c.277G>A,Gga>Aga):ENSP00000316460(p.Gly93Arg)]	NA	NA	2.00E-04	2.49E-05	0.00E+00	2.00E-04	0.00E+00	D	PrD	NA	MODERATE	D	0.927	82	82	0	97	78	19	19.59
Chewer	IOB_31	chr5	39375066	C	T	C9	NCT	ENSG00000113600:[ENST00000483232(n.119+13835G>A)]	NA	NA	NA	NA	NA	NA	NA	NA	NA	NA	MODIFIER	NA	NA	67	67	0	61	56	5	8.2
Chewer	IOB_31	chr5	39375066	C	T	DAB2	EXONIC-NC	ENSG00000153071:[ENST00000502879(n.167G>A)]	NA	NA	NA	NA	NA	NA	NA	NA	NA	NA	MODIFIER	NA	NA	67	67	0	61	56	5	8.2
Chewer	IOB_31	chr5	42807023	T	G	CTD-2325A15.5	EXONIC-NC	ENSG00000272234:[ENST00000606056(n.528T>G)]	NA	NA	NA	NA	NA	NA	NA	NA	NA	NA	MODIFIER	NA	NA	42	42	0	66	53	13	19.7
Chewer	IOB_31	chr5	42807023	T	G	SELENOP	MISSENSE	6414:[NM_001093726.1(c.481A>C,Aaa>Caa):NP_001087195.1(p.Lys161Gln)]	NA	NA	NA	NA	NA	NA	NA	NA	NA	NA	MODIFIER	NA	NA	42	42	0	66	53	13	19.7
Chewer	IOB_31	chr5	42807023	T	G	SEPP1	MISSENSE	ENSG00000250722:[ENST00000506577(c.391A>C,Aaa>Caa):ENSP00000425915(p.Lys131Gln)]	NA	NA	NA	NA	NA	NA	NA	NA	NA	NA	MODIFIER	NA	NA	42	42	0	66	53	13	19.7
Chewer	IOB_31	chr5	80409346	C	G	CTD-2193P3.2	EXONIC-NC	ENSG00000249772:[ENST00000508993(n.413G>C)]	NA	NA	NA	NA	NA	NA	NA	NA	NA	NA	MODIFIER	NA	NA	195	194	0	174	157	17	9.77
Chewer	IOB_31	chr5	80409346	C	G	RASGRF2	INTRONIC	ENSG00000113319:[ENST00000265080(c.2088-11C>G)]	NA	NA	NA	NA	NA	NA	NA	NA	NA	NA	MODIFIER	NA	NA	195	194	0	174	157	17	9.77
Chewer	IOB_31	chr5	96073661	C	T	CAST	INTRONIC-SS-DNR-PRX	ENSG00000153113:[ENST00000309190(c.483+10C>T)]	NA	NA	NA	NA	NA	NA	NA	NA	NA	NA	MODIFIER	NA	NA	53	53	0	47	39	8	17.02
Chewer	IOB_31	chr5	130785676	G	A	CTC-432M15.3	INTRONIC	ENSG00000273217:[ENST00000514667(c.3350+3071C>T)]	NA	NA	2.00E-04	4.70E-05	1.27E-04	2.00E-04	1.00E-03	NA	NA	NA	MODIFIER	NA	NA	301	299	0	194	189	5	2.58
Chewer	IOB_31	chr5	130785676	G	A	RAPGEF6	INTRONIC	ENSG00000158987:[ENST00000308008(c.3200+3071C>T)]	NA	NA	2.00E-04	4.70E-05	1.27E-04	2.00E-04	1.00E-03	NA	NA	NA	MODIFIER	NA	NA	301	299	0	194	189	5	2.58
Chewer	IOB_31	chr5	139062471	C	T	CXXC5	SILENT	ENSG00000171604:[ENST00000302517(c.948C>T,gcC>gcT):ENSP00000302543(p.Ala316(=))]	NA	NA	NA	2.52E-05	0.00E+00	NA	NA	NA	NA	NA	LOW	NA	NA	84	84	0	117	108	9	7.69
Chewer	IOB_31	chr5	153760011	G	A	GALNT10	MISSENSE	ENSG00000164574:[ENST00000297107(c.758G>A,cGc>cAc):ENSP00000297107(p.Arg253His)]	NA	NA	NA	8.38E-06	0.00E+00	NA	NA	T	PoD	NA	MODERATE	D	1	227	227	0	177	171	5	2.82
Chewer	IOB_31	chr5	153760011	G	A	SAP30L-AS1	NCT	ENSG00000245275:[ENST00000519727(n.202-50494C>T)]	NA	NA	NA	8.38E-06	0.00E+00	NA	NA	T	PoD	NA	MODERATE	D	1	227	227	0	177	171	5	2.82
Chewer	IOB_31	chr5	177649524	G	A	PHYKPL	SILENT	ENSG00000175309:[ENST00000308158(c.759C>T,ggC>ggT):ENSP00000310978(p.Gly253(=))]	NA	NA	NA	NA	NA	NA	NA	NA	NA	NA	MODIFIER	NA	NA	193	193	0	212	205	6	2.83
Chewer	IOB_31	chr6	3850731	C	G	FAM50B	MISSENSE	ENSG00000145945:[ENST00000380272(c.686C>G,tCc>tGc):ENSP00000369625(p.Ser229Cys)]	NA	chr6*3850731*3850731*COSM461774*C*T*1*cervix*1	NA	NA	NA	NA	NA	D	PoD	NA	MODERATE	N	0	456	452	1	549	521	27	4.92
Chewer	IOB_31	chr6	3850731	C	G	RP11-420L9.4	NCT	ENSG00000238158:[ENST00000454396(n.80-4973C>G)]	NA	chr6*3850731*3850731*COSM461774*C*T*1*cervix*1	NA	NA	NA	NA	NA	D	PoD	NA	MODERATE	N	0	456	452	1	549	521	27	4.92
Chewer	IOB_31	chr6	22570077	C	T	HDGFL1	SILENT	ENSG00000112273:[ENST00000230012(c.273C>T,tcC>tcT):ENSP00000230012(p.Ser91(=))]	NA	chr6*22570077*22570077*COSM1442449*C*T*1*large_intestine*1	chr6*22570077*22570077*C*G*rs371956095*NA*NA*NA	NA	NA	NA	NA	NA	NA	NA	LOW	NA	NA	193	192	0	222	210	9	4.05
Chewer	IOB_31	chr6	27834714	C	G	HIST1H1B	SILENT	ENSG00000184357:[ENST00000331442(c.594G>C,ccG>ccC):ENSP00000330074(p.Pro198(=))]	NA	chr6*27834714*27834714*COSM128161*C*T*1*upper_aerodigestive_tract*1	NA	NA	NA	NA	NA	NA	NA	NA	LOW	NA	NA	141	139	1	170	139	30	17.65
Chewer	IOB_31	chr6	29141653	C	T	OR2J2	NONSENSE	ENSG00000204700:[ENST00000377167(c.241C>T,Cag>Tag):ENSP00000366372(p.Gln81Ter)]	NA	NA	NA	NA	NA	NA	NA	NA	NA	NA	HIGH	NA	NA	263	256	0	272	197	66	24.26
Chewer	IOB_31	chr6	30309709	C	T	TRIM39	SILENT	ENSG00000204599:[ENST00000376659(c.1140C>T,tgC>tgT):ENSP00000365847(p.Cys380(=))]	NA	NA	NA	2.58E-05	1.23E-04	NA	NA	NA	NA	NA	MODIFIER	NA	NA	286	284	0	308	300	6	1.95
Chewer	IOB_31	chr6	30309709	C	T	TRIM39-RPP21	INTRONIC	202658:[NM_001199119.1(c.1104+36C>T)]	NA	NA	NA	2.58E-05	1.23E-04	NA	NA	NA	NA	NA	MODIFIER	NA	NA	286	284	0	308	300	6	1.95
Chewer	IOB_31	chr6	31084056	G	A	CDSN	NONSENSE	ENSG00000204539:[ENST00000376288(c.1336C>T,Cag>Tag):ENSP00000365465(p.Gln446Ter)]	NA	NA	NA	NA	NA	NA	NA	NA	NA	NA	MODIFIER	NA	NA	172	172	0	266	255	11	4.14
Chewer	IOB_31	chr6	31084056	G	A	PSORS1C1	NCT	ENSG00000204540:[ENST00000548049(n.119+1388G>A)]	NA	NA	NA	NA	NA	NA	NA	NA	NA	NA	MODIFIER	NA	NA	172	172	0	266	255	11	4.14
Chewer	IOB_31	chr6	31084063	G	A	CDSN	SILENT	ENSG00000204539:[ENST00000376288(c.1329C>T,atC>atT):ENSP00000365465(p.Ile443(=))]	NA	NA	NA	8.25E-06	0.00E+00	NA	NA	NA	NA	NA	MODIFIER	NA	NA	165	164	0	254	244	10	3.94
Chewer	IOB_31	chr6	31084063	G	A	PSORS1C1	NCT	ENSG00000204540:[ENST00000550838(n.58+1395G>A)]	NA	NA	NA	8.25E-06	0.00E+00	NA	NA	NA	NA	NA	MODIFIER	NA	NA	165	164	0	254	244	10	3.94
Chewer	IOB_31	chr6	33632922	C	T	ITPR3	SILENT	ENSG00000096433:[ENST00000374316(c.1341C>T,gaC>gaT):ENSP00000363435(p.Asp447(=))]	NA	NA	1.40E-03	1.89E-03	3.63E-04	1.40E-03	0.00E+00	NA	NA	NA	LOW	NA	0.042	246	245	1	397	388	6	1.51
Chewer	IOB_31	chr6	35565004	C	T	FKBP5	INTRONIC	ENSG00000096060:[ENST00000540787(c.128+21G>A)]	NA	NA	NA	NA	NA	NA	NA	NA	NA	NA	MODIFIER	NA	NA	324	324	0	311	285	26	8.36
Chewer	IOB_31	chr6	39286787	C	A	KCNK16	INTRONIC-SS-DNR-PRX	ENSG00000095981:[ENST00000373227(c.328+8G>T)]	NA	NA	NA	NA	NA	NA	NA	NA	NA	NA	LOW	NA	NA	98	98	0	140	132	8	5.71
Chewer	IOB_31	chr6	46135442	A	G	ENPP5	SILENT	ENSG00000112796:[ENST00000230565(c.558T>C,taT>taC):ENSP00000230565(p.Tyr186(=))]	NA	NA	NA	NA	NA	NA	NA	NA	NA	NA	LOW	NA	0.361	252	252	0	307	290	14	4.56
Chewer	IOB_31	chr6	108093490	G	T	SCML4	SILENT	ENSG00000146285:[ENST00000369020(c.42C>A,ccC>ccA):ENSP00000358016(p.Pro14(=))]	NA	chr6*108093490*108093490*COSM1439576*G*T*1*large_intestine*1	NA	NA	NA	NA	NA	NA	NA	NA	LOW	NA	NA	37	37	0	33	28	5	15.15
Chewer	IOB_31	chr6	109563531	A	T	C6orf183	NCT	ENSG00000243587:[ENST00000453496(n.488+22A>T)]	NA	NA	NA	NA	NA	NA	NA	NA	NA	NA	MODIFIER	NA	NA	75	75	0	45	39	6	13.33
Chewer	IOB_31	chr6	109563531	A	T	LOC100996634	INTRONIC	100996634:[NM_001277339.1(c.441+22A>T)]	NA	NA	NA	NA	NA	NA	NA	NA	NA	NA	MODIFIER	NA	NA	75	75	0	45	39	6	13.33
Chewer	IOB_31	chr6	123892219	G	T	TRDN	SILENT	ENSG00000186439:[ENST00000334268(c.81C>A,tcC>tcA):ENSP00000333984(p.Ser27(=))]	NA	NA	NA	NA	NA	NA	NA	NA	NA	NA	LOW	NA	NA	202	202	0	214	135	79	36.92
Chewer	IOB_31	chr6	131904991	C	A	ARG1	MISSENSE	ENSG00000118520:[ENST00000368087(c.912C>A,ttC>ttA):ENSP00000357066(p.Phe304Leu)]	NA	NA	chr6*131904991*131904991*C*T*rs373480378*0*0.9994*0.000599	NA	NA	NA	NA	T	BN	NA	MODERATE	D	1	145	145	0	209	204	5	2.39
Chewer	IOB_31	chr6	131904991	C	A	MED23	INTRONIC	ENSG00000112282:[ENST00000354577(c.4095+3858G>T)]	NA	NA	chr6*131904991*131904991*C*T*rs373480378*0*0.9994*0.000599	NA	NA	NA	NA	T	BN	NA	MODERATE	D	1	145	145	0	209	204	5	2.39
Chewer	IOB_31	chr6	152861043	G	T	SYNE1	NCT	ENSG00000131018:[ENST00000461872(n.398+52C>A)]	NA	NA	NA	NA	NA	NA	NA	NA	NA	NA	MODIFIER	NA	NA	277	274	0	316	263	53	16.77
Chewer	IOB_31	chr6	166864601	G	C	RPS6KA2	MISSENSE	ENSG00000071242:[ENST00000265678(c.1196C>G,cCa>cGa):ENSP00000265678(p.Pro399Arg)]	NA	NA	NA	NA	NA	NA	NA	D	PoD	NA	MODERATE	D	1	122	122	0	176	121	53	30.11
Chewer	IOB_31	chr7	1024669	G	T	CYP2W1	NONSENSE	ENSG00000073067:[ENST00000340150(c.253G>T,Gag>Tag):ENSP00000344178(p.Glu85Ter)]	NA	NA	chr7*1024663*1024670*GGCCGGGA*G*rs558437445*NA*NA*NA	NA	NA	NA	NA	NA	NA	NA	HIGH	NA	0	98	98	0	150	140	10	6.67
Chewer	IOB_31	chr7	5434199	G	A	TNRC18	MISSENSE	ENSG00000182095:[ENST00000399537(c.215C>T,gCc>gTc):ENSP00000382452(p.Ala72Val)]	NA	NA	NA	NA	NA	NA	NA	NA	NA	NA	MODIFIER	NA	NA	61	61	0	80	73	6	7.5
Chewer	IOB_31	chr7	30197132	G	A	MTURN	MISSENSE	ENSG00000180354:[ENST00000324453(c.364G>A,Gac>Aac):ENSP00000324204(p.Asp122Asn)]	NA	NA	NA	NA	NA	NA	NA	D_lc	PrD	NA	MODERATE	D	1	137	136	0	172	157	14	8.14
Chewer	IOB_31	chr7	44152778	T	G	AEBP1	INTRONIC	ENSG00000106624:[ENST00000223357(c.2709+49T>G)]	NA	NA	NA	NA	NA	NA	NA	NA	NA	NA	MODIFIER	NA	NA	144	144	0	256	200	54	21.09
Chewer	IOB_31	chr7	48556376	G	A	ABCA13	MISSENSE	ENSG00000179869:[ENST00000453246(c.2492G>A,Gtg>Atg):ENSP00000396789(p.Val832Met)]	NA	NA	NA	NA	NA	NA	NA	NA	NA	NA	MODERATE	NA	0.973	351	351	0	333	325	8	2.4
Chewer	IOB_31	chr7	73107007	G	A	WBSCR22	INTRONIC	ENSG00000071462:[ENST00000421744(c.*13+31G>A)]	NA	NA	chr7*73107007*73107007*G*T*rs374849764*NA*NA*NA	NA	NA	NA	NA	NA	NA	NA	MODIFIER	NA	NA	112	111	0	123	111	12	9.76
Chewer	IOB_31	chr7	98649004	G	A	SMURF1	MISSENSE	ENSG00000198742:[ENST00000361125(c.782C>T,aCg>aTg):ENSP00000354621(p.Thr261Met)]	NA	NA	NA	1.65E-05	0.00E+00	NA	NA	D	PrD	NA	MODERATE	D	1	166	166	0	161	150	11	6.83
Chewer	IOB_31	chr7	100172868	C	T	LRCH4	SILENT	ENSG00000077454:[ENST00000310300(c.1914G>A,gcG>gcA):ENSP00000309689(p.Ala638(=))]	NA	NA	NA	3.99E-05	0.00E+00	NA	NA	NA	NA	NA	MODIFIER	NA	NA	108	107	0	129	120	9	6.98
Chewer	IOB_31	chr7	100383643	G	A	ZAN	MISSENSE	ENSG00000146839:[ENST00000546213(c.2278G>A,Ggt>Agt):ENSP00000441117(p.Gly760Ser)]	NA	chr7*100383643*100383643*COSM372311*G*A*1*lung*1|chr7*100383643*100383643*COSM372310*G*A*1*lung*1	NA	2.54E-05	0.00E+00	NA	NA	NA	BN	NA	MODERATE	N	0	121	120	0	129	108	21	16.28
Chewer	IOB_31	chr7	102953492	C	A	DNAJC2	MISSENSE	ENSG00000105821:[ENST00000249270(c.1534G>T,Gct>Tct):ENSP00000249270(p.Ala512Ser)]	NA	NA	NA	NA	NA	NA	NA	NA	NA	NA	MODIFIER	NA	NA	159	158	0	118	98	20	16.95
Chewer	IOB_31	chr7	102953492	C	A	PMPCB	INTRONIC	ENSG00000105819:[ENST00000420236(c.1090+914C>A)]	NA	NA	NA	NA	NA	NA	NA	NA	NA	NA	MODIFIER	NA	NA	159	158	0	118	98	20	16.95
Chewer	IOB_31	chr7	105250950	C	T	ATXN7L1	INTRONIC	ENSG00000146776:[ENST00000477775(c.2175+26G>A)]	NA	NA	NA	NA	NA	NA	NA	NA	NA	NA	MODIFIER	NA	NA	66	65	0	67	61	5	7.46
Chewer	IOB_31	chr7	105250950	C	T	NA	NCT	101927951:[XR_242327.1(n.109-5433C>T)]	NA	NA	NA	NA	NA	NA	NA	NA	NA	NA	MODIFIER	NA	NA	66	65	0	67	61	5	7.46
Chewer	IOB_31	chr7	110764108	G	C	IMMP2L	INTRONIC	ENSG00000184903:[ENST00000450877(c.186-160487C>G)]	NA	NA	chr7*110764108*110764108*G*A*rs140070228*0*0.9998*0.0001997	NA	NA	NA	NA	T	PrD	NA	MODERATE	D	1	303	299	0	298	219	77	25.84
Chewer	IOB_31	chr7	110764108	G	C	LRRN3	MISSENSE	ENSG00000173114:[ENST00000308478(c.1280G>C,aGc>aCc):ENSP00000312001(p.Ser427Thr)]	NA	NA	chr7*110764108*110764108*G*A*rs140070228*0*0.9998*0.0001997	NA	NA	NA	NA	T	PrD	NA	MODERATE	D	1	303	299	0	298	219	77	25.84
Chewer	IOB_31	chr7	114562958	T	A	MDFIC	INTRONIC	29969:[XM_005250300.1(c.-104-27T>A)]	NA	NA	2.40E-03	7.26E-04	3.43E-04	2.40E-03	0.00E+00	NA	NA	NA	MODIFIER	NA	NA	12	12	0	38	18	19	50

Patel *et al.*, 2021. Whole-exome sequencing analysis of oral squamous cell carcinoma delineated by tobacco usage habits
Supplementary Table 4. List of somatic single nucleotide variants identified using whole exome sequencing of OSCC patients

Cohort	Sample	Chromosome	Genomic position	Reference allele	Alternate allele	Genesymbol	Variant type	Variant description	OncoMD	COSMIC	dbSNP	ExAC	ExAC_SAS	1000G	1000G_SAS	SIFT	Polyphen2	CONDEL	VEP	LRT	PhastCon7Way	Total reads in Normal	Reference read support in Normal	Alternative read support in Normal	Total reads in Tumor	Reference read support in Tumor	Alternate read support in Tumor	Alternate allele frequency in Tumor
Chewer	IOB_31	chr7	128414538	C	G	OPN1SW	INTRONIC	ENSG00000128617:[ENST00000249389(c.687+14G>C)]	NA	NA	NA	NA	NA	NA	NA	NA	NA	NA	MODIFIER	NA	NA	65	65	0	86	79	7	8.14
Chewer	IOB_31	chr7	142627053	C	T	TRPV5	INTRONIC	ENSG00000127412:[ENST00000439304(c.331+100G>A)]	NA	NA	NA	NA	NA	NA	NA	NA	NA	NA	MODIFIER	NA	NA	75	74	1	82	75	7	8.54
Chewer	IOB_31	chr7	155499632	C	G	RBM33	MISSENSE	ENSG00000184863:[ENST00000392761(c.133C>G,tCc>tGc):ENSP00000376514(p.Ser45Cys)]	NA	NA	NA	NA	NA	NA	NA	D_lc	PrD	NA	MODERATE	NA	NA	90	90	0	81	69	12	14.81
Chewer	IOB_31	chr8	7320349	C	T	SPAG11B	MISSENSE	ENSG00000164871:[ENST00000297498(c.94G>A,Gcc>Acc):ENSP00000297498(p.Ala32Thr)]	NA	NA	chr8*7320349*7320349*C*A,G*rs370271349*0*0.9998*0.0001997,.	NA	NA	NA	NA	D_lc	PrD	NA	MODERATE	NA	NA	197	196	0	127	105	22	17.32
Chewer	IOB_31	chr8	22210584	C	A	PIWIL2	MISSENSE	ENSG00000197181:[ENST00000356766(c.2516C>A,gCt>gAt):ENSP00000349208(p.Ala839Asp)]	NA	NA	NA	NA	NA	NA	NA	D	BN	NA	MODERATE	D	1	286	285	1	237	231	6	2.53
Chewer	IOB_31	chr8	30643526	C	G	PPP2CB	INTRONIC	ENSG00000104695:[ENST00000518564(c.142-21G>C)]	NA	NA	NA	NA	NA	NA	NA	NA	NA	NA	MODIFIER	NA	NA	70	70	0	34	29	5	14.71
Chewer	IOB_31	chr8	52287373	C	A	PXDNL	NCT	137902:[XR_242454.1(n.3645-82G>T)]	NA	NA	NA	NA	NA	NA	NA	NA	NA	NA	MODIFIER	NA	NA	60	59	1	58	53	5	8.62
Chewer	IOB_31	chr8	52321348	G	A	PXDNL	NONSENSE	ENSG00000147485:[ENST00000356297(c.2836C>T,Cga>Tga):ENSP00000348645(p.Arg946Ter)]	NA	chr8*52321348*52321348*COSM1457434*G*A*3*liver,large_intestine,breast*1,1,1|chr8*52321348*52321348*COSM1457433*G*A*3*liver,large_intestine,breast*1,1,1	NA	NA	NA	NA	NA	NA	NA	NA	HIGH	NA	NA	143	141	0	213	206	6	2.82
Chewer	IOB_31	chr8	65493557	C	A	BHLHE22	SILENT	ENSG00000180828:[ENST00000321870(c.210C>A,ccC>ccA):ENSP00000318799(p.Pro70(=))]	NA	NA	chr8*65493530*65493564*CCTGGGCTGCTTCGAGCCGGCTGACCCCGAGGGGG*C*rs754807406*NA*NA*NA	NA	NA	NA	NA	NA	NA	NA	LOW	NA	NA	64	64	0	111	106	5	4.5
Chewer	IOB_31	chr8	65493557	C	A	RP11-21C4.1	NCT	ENSG00000254102:[ENST00000517909(n.171+718G>T)]	NA	NA	chr8*65493530*65493564*CCTGGGCTGCTTCGAGCCGGCTGACCCCGAGGGGG*C*rs754807406*NA*NA*NA	NA	NA	NA	NA	NA	NA	NA	LOW	NA	NA	64	64	0	111	106	5	4.5
Chewer	IOB_31	chr8	124253629	G	A	C8orf76	5UTR	ENSG00000189376:[ENST00000276704(c.-43C>T)]	NA	NA	NA	NA	NA	NA	NA	NA	NA	NA	MODIFIER	NA	NA	118	118	0	216	208	7	3.24
Chewer	IOB_31	chr8	124253629	G	A	ZHX1-C8ORF76	INTRONIC	ENSG00000259305:[ENST00000357082(c.22-2245C>T)]	NA	NA	NA	NA	NA	NA	NA	NA	NA	NA	MODIFIER	NA	NA	118	118	0	216	208	7	3.24
Chewer	IOB_31	chr8	124265575	G	A	ZHX1	MISSENSE	ENSG00000165156:[ENST00000297857(c.2612C>T,tCa>tTa):ENSP00000297857(p.Ser871Leu)]	NA	NA	NA	NA	NA	NA	NA	NA	NA	NA	MODIFIER	NA	NA	250	249	1	269	243	25	9.29
Chewer	IOB_31	chr8	124265575	G	A	ZHX1-C8ORF76	INTRONIC	ENSG00000259305:[ENST00000357082(c.21+13938C>T)]	NA	NA	NA	NA	NA	NA	NA	NA	NA	NA	MODIFIER	NA	NA	250	249	1	269	243	25	9.29
Chewer	IOB_31	chr8	125531025	C	T	TATDN1	SILENT	ENSG00000147687:[ENST00000520938(c.188G>A,tGa>tAa):ENSP00000430756(p.Ter63Ter)]	NA	NA	NA	NA	NA	NA	NA	NA	NA	NA	MODIFIER	NA	NA	43	42	1	47	41	6	12.77
Chewer	IOB_31	chr8	133984821	G	T	TG	INTRONIC	ENSG00000042832:[ENST00000519543(c.518-22G>T)]	NA	NA	NA	3.95E-01	3.55E-01	NA	NA	NA	NA	NA	MODIFIER	NA	NA	41	39	2	33	27	6	18.18
Chewer	IOB_31	chr8	134251333	G	A	NDRG1	MISSENSE	ENSG00000104419:[ENST00000323851(c.973C>T,Cgg>Tgg):ENSP00000319977(p.Arg325Trp)]	NA	NA	.	4.79E-04	0.00E+00	NA	NA	D	PrD	NA	MODERATE	D	1	115	115	0	235	229	6	2.55
Chewer	IOB_31	chr8	144691082	C	G	PYCRL	INTRONIC	ENSG00000104524:[ENST00000377579(c.-433-36G>C)]	NA	NA	NA	NA	NA	NA	NA	NA	NA	NA	MODIFIER	NA	NA	75	75	0	112	87	24	21.43
Chewer	IOB_31	chr8	144877651	C	T	SCRIB	MISSENSE	ENSG00000180900:[ENST00000526832(c.639G>A,cGg>cAg):ENSP00000431519(p.Arg214Gln)]	NA	NA	NA	6.27E-05	6.45E-05	NA	NA	D	BN	NA	MODERATE	NA	NA	185	185	0	348	329	17	4.89
Chewer	IOB_31	chr9	68415332	T	C	MIR4477A	EXONIC-NC	ENSG00000266017:[ENST00000581659(n.25T>C)]	NA	NA	NA	9.88E-03	0.00E+00	NA	NA	NA	NA	NA	MODIFIER	NA	NA	296	294	0	232	223	8	3.45
Chewer	IOB_31	chr9	91617249	C	A	S1PR3	MISSENSE	ENSG00000213694:[ENST00000358157(c.1134C>A,aaC>aaA):ENSP00000350878(p.Asn378Lys)]	NA	NA	NA	NA	NA	NA	NA	T_lc	BN	NA	MODERATE	N	0.999	228	226	0	337	330	7	2.08
Chewer	IOB_31	chr9	96425255	C	A	PHF2	MISSENSE	ENSG00000197724:[ENST00000359246(c.1765C>A,Ctg>Atg):ENSP00000352185(p.Leu589Met)]	NA	NA	NA	NA	NA	NA	NA	T	PoD	NA	MODERATE	N	1	76	76	0	218	210	7	3.21
Chewer	IOB_31	chr9	109689490	A	C	ZNF462	SILENT	ENSG00000148143:[ENST00000277225(c.3297A>C,ccA>ccC):ENSP00000277225(p.Pro1099(=))]	NA	chr9*109689490*109689490*COSM1645931*A*C*2*NS,kidney*1,1|chr9*109689490*109689490*COSM486873*A*C*2*NS,kidney*1,1|chr9*109689490*109689491*COSM1459166*AC*A*5*large_intestine*5	NA	9.10E-02	9.96E-02	NA	NA	NA	NA	NA	MODIFIER	NA	NA	40	37	3	43	34	7	16.28
Chewer	IOB_31	chr9	114905832	C	G	SUSD1	MISSENSE	ENSG00000106868:[ENST00000355396(c.395G>C,Gaa>Caa):ENSP00000347558(p.Glu133Gln)]	NA	NA	NA	NA	NA	NA	NA	NA	NA	NA	MODIFIER	NA	NA	100	100	0	70	51	18	25.71
Chewer	IOB_31	chr9	130572306	G	A	FPGS	INTRONIC-SS-ACR-PRX	ENSG00000136877:[ENST00000373225(c.1062-4G>A)]	NA	NA	NA	1.24E-05	0.00E+00	NA	NA	NA	NA	NA	LOW	NA	NA	54	54	0	80	49	30	37.5
Chewer	IOB_31	chr9	137623480	C	T	COL5A1	MISSENSE	ENSG00000130635:[ENST00000371817(c.1303C>T,Ccg>Tcg):ENSP00000360882(p.Pro435Ser)]	NA	chr9*137623480*137623480*COSM3413438*C*T*1*central_nervous_system*1	chr9*137623480*137623480*C*G*rs377488010*NA*NA*NA	NA	NA	NA	NA	NA	NA	NA	MODIFIER	NA	NA	346	343	2	406	396	10	2.46
Chewer	IOB_31	chr9	139649813	C	A	LCN8	MISSENSE	138307:[XM_005266056.1(c.382G>T,Gcc>Tcc):XP_005266113.1(p.Ala128Ser)]	NA	NA	NA	NA	NA	NA	NA	NA	NA	NA	MODERATE	NA	NA	137	136	1	125	119	6	4.8
Chewer	IOB_31	chr9	139905043	A	C	ABCA2	MISSENSE	ENSG00000107331:[ENST00000479446(c.4252T>G,cTg>cGg):ENSP00000420084(p.Leu1418Arg)]	NA	NA	NA	NA	NA	NA	NA	NA	NA	NA	MODIFIER	NA	NA	45	41	1	73	59	9	12.33
Chewer	IOB_31	chr9	140123502	C	T	RNF224	SILENT	ENSG00000233198:[ENST00000445101(c.435C>T,agC>agT):ENSP00000406665(p.Ser145(=))]	NA	NA	NA	NA	NA	NA	NA	NA	NA	NA	LOW	NA	NA	113	112	0	156	149	7	4.49
Chewer	IOB_31	chrX	15320941	A	C	ASB11	INTRONIC	ENSG00000165192:[ENST00000380470(c.182-12T>G)]	NA	NA	NA	NA	NA	NA	NA	NA	NA	NA	MODIFIER	NA	NA	17	17	0	35	19	15	42.86
Chewer	IOB_31	chrX	16873113	T	G	RBBP7	INTRONIC	ENSG00000102054:[ENST00000416035(c.358-1148A>C)]	NA	NA	NA	NA	NA	NA	NA	NA	NA	NA	MODIFIER	NA	NA	73	68	0	92	78	6	6.52
Chewer	IOB_31	chrX	16876937	C	G	RBBP7	MISSENSE	ENSG00000102054:[ENST00000404022(c.316G>C,Gaa>Caa):ENSP00000386068(p.Glu106Gln)]	NA	NA	NA	NA	NA	NA	NA	T	BN	NA	MODERATE	NA	NA	256	255	0	286	271	15	5.24
Chewer	IOB_31	chrX	17819065	G	T	RAI2	MISSENSE	ENSG00000131831:[ENST00000331511(c.1066C>A,Ccc>Acc):ENSP00000333456(p.Pro356Thr)]	NA	NA	NA	NA	NA	NA	NA	T	BN	NA	MODERATE	N	0.023	154	152	0	163	96	67	41.1
Chewer	IOB_31	chrX	19905395	G	C	SH3KBP1	INTRONIC	ENSG00000147010:[ENST00000379697(c.4+30C>G)]	NA	NA	NA	NA	NA	NA	NA	NA	NA	NA	MODIFIER	NA	NA	31	31	0	39	13	26	66.67
Chewer	IOB_31	chrX	24483738	C	A	PDK3	INTRONIC	ENSG00000067992:[ENST00000379162(c.106+60C>A)]	NA	NA	NA	NA	NA	NA	NA	NA	NA	NA	MODIFIER	NA	NA	209	208	1	295	281	11	3.73
Chewer	IOB_31	chrX	47058918	G	A	UBA1	MISSENSE	ENSG00000130985:[ENST00000335972(c.385G>A,Gag>Aag):ENSP00000338413(p.Glu129Lys)]	NA	chrX*47058918*47058918*COSM457542*G*A*1*breast*1	NA	1.14E-05	0.00E+00	NA	NA	T	BN	NA	MODERATE	NA	NA	478	475	0	697	655	41	5.88
Chewer	IOB_31	chrX	48758568	G	T	PQBP1	MISSENSE	ENSG00000102103:[ENST00000218224(c.169G>T,Gac>Tac):ENSP00000218224(p.Asp57Tyr)]	NA	NA	NA	NA	NA	NA	NA	NA	NA	NA	MODIFIER	NA	NA	113	113	0	164	158	6	3.66
Chewer	IOB_31	chrX	49028464	G	A	PLP2	INTRONIC	ENSG00000102007:[ENST00000376322(c.96+21G>A)]	NA	NA	NA	NA	NA	NA	NA	NA	NA	NA	MODIFIER	NA	NA	153	151	1	278	258	17	6.12
Chewer	IOB_31	chrX	49962146	C	A	AKAP4	INTRONIC	ENSG00000147081:[ENST00000358526(c.174+49G>T)]	NA	NA	NA	NA	NA	NA	NA	NA	NA	NA	MODIFIER	NA	NA	184	184	0	232	210	18	7.76
Chewer	IOB_31	chrX	74960896	G	A	TTC3P1	EXONIC-NC	ENSG00000215105:[ENST00000535618(n.1008C>T)]	NA	NA	2.65E-04	1.16E-05	1.03E-04	2.65E-04	1.00E-03	NA	NA	NA	MODIFIER	NA	NA	207	207	0	334	302	30	8.98
Chewer	IOB_31	chrX	99956262	G	A	SYTL4	SILENT	ENSG00000102362:[ENST00000263033(c.390C>T,cgC>cgT):ENSP00000263033(p.Arg130(=))]	NA	NA	NA	NA	NA	NA	NA	NA	NA	NA	LOW	NA	NA	80	80	0	90	82	8	8.89
Chewer	IOB_31	chrX	107978641	C	G	IRS4	MISSENSE	ENSG00000133124:[ENST00000372129(c.934G>C,Gat>Cat):ENSP00000361202(p.Asp312His)]	NA	NA	NA	NA	NA	NA	NA	D	PrD	NA	MODERATE	D	1	243	243	0	354	330	24	6.78
Chewer	IOB_31	chrX	123697624	T	G	TENM1	INTRONIC-SS-ACR	ENSG00000009694:[ENST00000371130(c.2264-2A>C)]	NA	NA	chrX*123697624*123697624*T*TA*rs750391237*NA*NA*NA	NA	NA	NA	NA	NA	NA	NA	HIGH	NA	NA	66	66	0	67	51	14	20.9
Chewer	IOB_31	chrX	152083580	G	A	ZNF185	INTRONIC	ENSG00000147394:[ENST00000318504(c.159-102G>A)]	NA	NA	NA	NA	NA	NA	NA	NA	NA	NA	MODIFIER	NA	NA	90	90	0	179	169	9	5.03
Chewer	IOB_31	chrX	153149469	G	A	L1CAM	EXONIC-NC	ENSG00000198910:[ENST00000484587(n.589C>T)]	NA	NA	NA	NA	NA	NA	NA	NA	NA	NA	MODIFIER	NA	NA	138	137	0	241	225	14	5.81
Chewer	IOB_31	chrX	153149469	G	A	LCA10	MISSENSE	ENSG00000196987:[ENST00000452593(c.300G>A,Gat>Aat):ENSP00000389835(p.Asp101Asn)]	NA	NA	NA	NA	NA	NA	NA	NA	NA	NA	MODIFIER	NA	NA	138	137	0	241	225	14	5.81
Chewer	IOB_32	chr1	6634726	G	A	TAS1R1	SILENT	ENSG00000173662:[ENST00000328191(c.534G>A,gtG>gtA):ENSP00000327705(p.Val178(=))]	NA	NA	NA	NA	NA	NA	NA	NA	NA	NA	MODIFIER	NA	NA	165	165	0	230	190	38	16.52
Chewer	IOB_32	chr1	35477663	A	C	RP11-244H3.4	INTRONIC	ENSG00000271741:[ENST00000487874(c.947-57T>G)]	NA	NA	NA	NA	NA	NA	NA	NA	NA	NA	MODIFIER	NA	NA	39	39	0	38	33	5	13.16
Chewer	IOB_32	chr1	35477663	A	C	ZMYM6	INTRONIC	ENSG00000163867:[ENST00000357182(c.947-57T>G)]	NA	NA	NA	NA	NA	NA	NA	NA	NA	NA	MODIFIER	NA	NA	39	39	0	38	33	5	13.16
Chewer	IOB_32	chr1	41303361	G	A	KCNQ4	SILENT	ENSG00000117013:[ENST00000347132(c.1770G>A,ggG>ggA):ENSP00000262916(p.Gly590(=))]	NA	NA	NA	8.98E-06	0.00E+00	NA	NA	NA	NA	NA	LOW	NA	NA	81	81	0	122	95	27	22.13
Chewer	IOB_32	chr1	47842478	T	G	CMPK1	3UTR	ENSG00000162368:[ENST00000371871(c.*161T>G)]	NA	NA	NA	NA	NA	NA	NA	NA	NA	NA	MODIFIER	NA	NA	52	52	0	49	40	9	18.37
Chewer	IOB_32	chr1	74937421	T	A	FPGT-TNNI3K	INTRONIC	ENSG00000259030:[ENST00000557284(c.2463+8187T>A)]	NA	NA	NA	NA	NA	NA	NA	NA	NA	NA	MODIFIER	NA	NA	73	73	0	105	91	14	13.33
Chewer	IOB_32	chr1	74937421	T	A	LRRC53	MISSENSE	ENSG00000162621:[ENST00000294635(c.1885A>T,Acc>Tcc):ENSP00000294635(p.Thr629Ser)]	NA	NA	NA	NA	NA	NA	NA	NA	NA	NA	MODIFIER	NA	NA	73	73	0	105	91	14	13.33
Chewer	IOB_32	chr1	74937421	T	A	TNNI3K	INTRONIC	ENSG00000116783:[ENST00000326637(c.2121+8187T>A)]	NA	NA	NA	NA	NA	NA	NA	NA	NA	NA	MODIFIER	NA	NA	73	73	0	105	91	14	13.33
Chewer	IOB_32	chr1	82456697	C	T	ADGRL2	SILENT	23266:[XM_005270668.1(c.4209C>T,ggC>ggT):XP_005270725.1(p.Gly1403(=))]	NA	NA	NA	NA	NA	NA	NA	NA	NA	NA	MODIFIER	NA	NA	163	162	0	198	190	7	3.54
Chewer	IOB_32	chr1	82456697	C	T	LPHN2	SILENT	ENSG00000117114:[ENST00000370723(c.4254C>T,ggC>ggT):ENSP00000359758(p.Gly1418(=))]	NA	NA	NA	NA	NA	NA	NA	NA	NA	NA	MODIFIER	NA	NA	163	162	0	198	190	7	3.54
Chewer	IOB_32	chr1	90048607	C	T	LRRC8B	MISSENSE	ENSG00000197147:[ENST00000330947(c.398C>T,gCa>gTa):ENSP00000332674(p.Ala133Val)]	NA	NA	NA	NA	NA	NA	NA	T	BN	NA	MODERATE	D	1	258	258	0	325	317	6	1.85
Chewer	IOB_32	chr1	110299633	A	G	EPS8L3	INTRONIC	79574:[NM_001319952.1(c.1019+6T>C)]	NA	NA	NA	NA	NA	NA	NA	NA	NA	NA	MODIFIER	NA	NA	117	117	0	136	130	5	3.68
Chewer	IOB_32	chr1	110299633	A	G	GSTM5	NCT	ENSG00000134201:[ENST00000429410(n.83-18061A>G)]	NA	NA	NA	NA	NA	NA	NA	NA	NA	NA	MODIFIER	NA	NA	117	117	0	136	130	5	3.68
Chewer	IOB_32	chr1	110299633	A	G	NA	NCT	101928585:[XR_246393.1(n.224-2685A>G)]	NA	NA	NA	NA	NA	NA	NA	NA	NA	NA	MODIFIER	NA	NA	117	117	0	136	130	5	3.68
Chewer	IOB_32	chr1	110299633	A	G	RP4-735C1.4	NCT	ENSG00000241720:[ENST00000431955(n.628-18061A>G)]	NA	NA	NA	NA	NA	NA	NA	NA	NA	NA	MODIFIER	NA	NA	117	117	0	136	130	5	3.68
Chewer	IOB_32	chr1	161577660	G	A	FCGR2B	INTRONIC	ENSG00000072694:[ENST00000367960(c.112+26321G>A)]	NA	NA	NA	NA	NA	NA	NA	NA	NA	NA	MODIFIER	NA	NA	77	77	0	108	82	26	24.07
Chewer	IOB_32	chr1	161577660	G	A	FCGR3A	INTRONIC	ENSG00000203747:[ENST00000540048(c.61+22498C>T)]	NA	NA	NA	NA	NA	NA	NA	NA	NA	NA	MODIFIER	NA	NA	77	77	0	108	82	26	24.07
Chewer	IOB_32	chr1	161577660	G	A	HSPA7	EXONIC-NC	3311:[NR_024151.1(n.1812G>A)]	NA	NA	NA	NA	NA	NA	NA	NA	NA	NA	MODIFIER	NA	NA	77	77	0	108	82	26	24.07
Chewer	IOB_32	chr1	168054852	G	A	GPR161	NONSENSE	ENSG00000143147:[ENST00000539777(c.1273C>T,Cga>Tga):ENSP00000437576(p.Arg425Ter)]	NA	NA	NA	8.30E-06	0.00E+00	NA	NA	NA	NA	NA	HIGH	NA	1	257	257	0	321	278	41	12.77
Chewer	IOB_32	chr1	185835059	C	A	HMCN1	INTRONIC	ENSG00000143341:[ENST00000271588(c.621+64C>A)]	NA	NA	NA	NA	NA	NA	NA	NA	NA	NA	MODIFIER	NA	NA	119	118	1	150	143	7	4.67
Chewer	IOB_32	chr1	186344242	C	T	TPR	SILENT	ENSG00000047410:[ENST00000451586(c.147G>A,gcG>gcA):ENSP00000408103(p.Ala49(=))]	NA	NA	chr1*186344242*186344242*C*A,G*rs746897607*NA*NA*NA	NA	NA	NA	NA	NA	NA	NA	MODIFIER	NA	NA	96	93	0	114	93	21	18.42
Chewer	IOB_32	chr1	203194807	G	A	CHIT1	SILENT	ENSG00000133063:[ENST00000535569(c.277C>T,Ctg>Ttg):ENSP00000438078(p.Leu93(=))]	NA	NA	NA	NA	NA	NA	NA	NA	NA	NA	LOW	NA	NA	102	102	0	119	107	12	10.08
Chewer	IOB_32	chr1	212912946	G	A	NSL1	INTRONIC	ENSG00000117697:[ENST00000366978(c.191-12425C>T)]	NA	NA	NA	NA	NA	NA	NA	NA	NA	NA	MODIFIER	NA	NA	58	58	0	78	73	5	6.41
Chewer	IOB_32	chr1	248789931	C	A	OR2T11	MISSENSE	ENSG00000183130:[ENST00000330803(c.499G>T,Ggc>Tgc):ENSP00000328934(p.Gly167Cys)]	NA	NA	NA	NA	NA	NA	NA	D	PoD	NA	MODERATE	NA	0.056	174	173	0	258	251	5	1.94
Chewer	IOB_32	chr10	32103163	C	A	ARHGAP12	INTRONIC	94134:[NM_001270698.1(c.1603+24G>T)]	NA	NA	NA	NA	NA	NA	NA	NA	NA	NA	MODIFIER	NA	NA	59	59	0	65	56	9	13.85
Chewer	IOB_32	chr10	50085201	G	A	WDFY4	MISSENSE	ENSG00000128815:[ENST00000325239(c.7124G>A,cGg>cAg):ENSP00000320563(p.Arg2375Gln)]	NA	NA	NA	NA	NA	NA	NA	T	BN	NA	MODERATE	NA	NA	49	49	0	45	37	8	17.78

Patel *et al.* , 2021. Whole-exome sequencing analysis of oral squamous cell carcinoma delineated by tobacco usage habits
Supplementary Table 4. List of somatic single nucleotide variants identified using whole exome sequencing of OSCC patients

Cohort	Sample	Chromosome	Genomic position	Reference allele	Alternate allele	Genesymbol	Variant type	Variant description	OncoMD	COSMIC	dbSNP	ExAC	ExAC_SAS	1000G	1000G_SAS	SIFT	Polyphen2	CONDEL	VEP	LRT	PhastCon7Way	Total reads in Normal	Reference read support in Normal	Alternative read support in Normal	Total reads in Tumor	Reference read support in Tumor	Alternate read support in Tumor	Alternate allele frequency in Tumor
Chewer	IOB_32	chr10	69672655	G	A	SIRT1	SILENT	ENSG00000096717:[ENST00000403579(c.873G>A,caG>caA):ENSP00000384063(p.Gln291(=))]	NA	NA	chr10*69672655*69672655*G*C*rs759555466*NA*NA*NA	NA	NA	NA	NA	NA	NA	NA	LOW	NA	0.417	231	231	0	228	198	28	12.28
Chewer	IOB_32	chr10	89692922	T	C	PTEN	MISSENSE	ENSG00000171862:[ENST00000371953(c.406T>C,Tgt>Cgt):ENSP00000361021(p.Cys136Arg)]	NA	chr10*89692907*89692927*COSM4946*BigSeq*A*1*endometrium*1|chr10*89692919*89692925*COSM5845*ATATGTG*A*1*central_nervous_system*1|chr10*89692920*89692922*COSM1684693*TAT*T*1*endometrium*1|chr10*89692920*89692939*COSM4925*TATGTGCATATTTATTACAT*T*1*endometrium*1|chr10*89692922*89692922*COSM4987*T*TA*2*endometrium,central_nervous_system*1,1|chr10*89692922*89692922*COSM5096*T*C*5*endometrium,central_nervous_system*	NA	NA	NA	NA	NA	D	PrD	NA	MODERATE	NA	1	259	258	0	313	261	51	16.29
Chewer	IOB_32	chr10	89712017	G	A	PTEN	INTRONIC-SS-DNR	5728:[NM_001304717.2(c.1153+1G>A)]	NA	chr10*89712009*89712034*COSM1321158*BigSeq*A*1*ovary*1|chr10*89712015*89712019*COSM921120*CAGTA*C*1*endometrium*1|chr10*89712017*89712017*COSM5968*G*T*2*endometrium*2|chr10*89712017*89712017*COSM17567*G*A*2*central_nervous_system,upper_aerodigestive_tract*1,1	NA	NA	NA	NA	NA	NA	NA	NA	HIGH	NA	NA	62	62	0	78	61	17	21.79
Chewer	IOB_32	chr10	93600364	G	A	TNKS2	MISSENSE	ENSG00000107854:[ENST00000371627(c.1574G>A,cGt>cAt):ENSP00000360689(p.Arg525His)]	NA	NA	NA	3.30E-05	0.00E+00	NA	NA	D	PrD	NA	MODERATE	D	1	149	148	0	210	186	23	10.95
Chewer	IOB_32	chr10	94333773	G	A	IDE	MISSENSE	ENSG00000119912:[ENST00000265986(c.4C>T,Cgg>Tgg):ENSP00000265986(p.Arg2Trp)]	NA	NA	NA	NA	NA	NA	NA	NA	NA	NA	MODIFIER	NA	NA	76	76	0	69	64	5	7.25
Chewer	IOB_32	chr11	1080999	C	A	MUC2	INTRONIC	ENSG00000198788:[ENST00000359061(c.1368+15C>A)]	NA	NA	chr11*1080996*1081012*TGCCCCCTCCTTCTGGA*T*rs750789302*NA*NA*NA|chr11*1080999*1080999*C*T*rs750320800*NA*NA*N	NA	NA	NA	NA	NA	NA	NA	MODIFIER	NA	NA	253	251	0	237	229	6	2.53
Chewer	IOB_32	chr11	1253869	C	T	MUC5B	INTRONIC-SS-ACR-PRX	ENSG00000117983:[ENST00000447027(c.1948-5C>T)]	NA	NA	2.00E-04	1.92E-04	1.17E-04	2.00E-04	0.00E+00	NA	NA	NA	LOW	NA	NA	198	198	0	208	198	6	2.88
Chewer	IOB_32	chr11	4827121	C	G	MMP26	INTRONIC	ENSG00000167346:[ENST00000380390(c.-145+38550C>G)]	NA	NA	NA	NA	NA	NA	NA	NA	NA	NA	MODIFIER	NA	NA	204	204	0	214	201	13	6.07
Chewer	IOB_32	chr11	4843463	C	A	MMP26	NCT	ENSG00000167346:[ENST00000477339(n.191+54892C>A)]	NA	NA	chr11*4843463*4843463*C*T*rs374318168*NA*NA*NA	NA	NA	NA	NA	NA	NA	NA	MODIFIER	NA	NA	249	248	0	282	234	48	17.02
Chewer	IOB_32	chr11	4843463	C	A	OR51F2	MISSENSE	ENSG00000176925:[ENST00000322110(c.848C>A,cCt>cAt):ENSP00000323952(p.Pro283His)]	NA	NA	chr11*4843463*4843463*C*T*rs374318168*NA*NA*NA	NA	NA	NA	NA	NA	NA	NA	MODIFIER	NA	NA	249	248	0	282	234	48	17.02
Chewer	IOB_32	chr11	5411029	G	A	AC104389.28	NCT	ENSG00000167355:[ENST00000420726(n.303-42889C>T)]	NA	chr11*5411029*5411029*COSM1260365*G*A*1*oesophagus*1	NA	1.66E-05	6.06E-05	NA	NA	NA	NA	NA	MODIFIER	NA	NA	197	197	0	270	224	44	16.3
Chewer	IOB_32	chr11	5411029	G	A	HBE1	INTRONIC	ENSG00000213931:[ENST00000396895(c.-267+115770C>T)]	NA	chr11*5411029*5411029*COSM1260365*G*A*1*oesophagus*1	NA	1.66E-05	6.06E-05	NA	NA	NA	NA	NA	MODIFIER	NA	NA	197	197	0	270	224	44	16.3
Chewer	IOB_32	chr11	5411029	G	A	HBG2	INTRONIC	ENSG00000196565:[ENST00000380252(c.62+115552C>T)]	NA	chr11*5411029*5411029*COSM1260365*G*A*1*oesophagus*1	NA	1.66E-05	6.06E-05	NA	NA	NA	NA	NA	MODIFIER	NA	NA	197	197	0	270	224	44	16.3
Chewer	IOB_32	chr11	5411029	G	A	OR51B5	INTRONIC	282763:[NM_001005567.2(c.-363-42889C>T)]	NA	chr11*5411029*5411029*COSM1260365*G*A*1*oesophagus*1	NA	1.66E-05	6.06E-05	NA	NA	NA	NA	NA	MODIFIER	NA	NA	197	197	0	270	224	44	16.3
Chewer	IOB_32	chr11	5411029	G	A	OR51M1	MISSENSE	ENSG00000184698:[ENST00000328611(c.401G>A,cGc>cAc):ENSP00000333196(p.Arg134His)]	NA	chr11*5411029*5411029*COSM1260365*G*A*1*oesophagus*1	NA	1.66E-05	6.06E-05	NA	NA	NA	NA	NA	MODIFIER	NA	NA	197	197	0	270	224	44	16.3
Chewer	IOB_32	chr11	5423982	C	G	AC104389.28	NCT	ENSG00000167355:[ENST00000420465(n.46-55842G>C)]	NA	NA	NA	NA	NA	NA	NA	NA	NA	NA	MODIFIER	NA	NA	486	486	0	539	447	90	16.7
Chewer	IOB_32	chr11	5423982	C	G	HBE1	INTRONIC	ENSG00000213931:[ENST00000380237(c.-310+102817G>C)]	NA	NA	NA	NA	NA	NA	NA	NA	NA	NA	MODIFIER	NA	NA	486	486	0	539	447	90	16.7
Chewer	IOB_32	chr11	5423982	C	G	HBG2	INTRONIC	ENSG00000196565:[ENST00000380259(c.-564-56853G>C)]	NA	NA	NA	NA	NA	NA	NA	NA	NA	NA	MODIFIER	NA	NA	486	486	0	539	447	90	16.7
Chewer	IOB_32	chr11	5423982	C	G	OR51B5	INTRONIC	282763:[NM_001005567.2(c.-363-55842G>C)]	NA	NA	NA	NA	NA	NA	NA	NA	NA	NA	MODIFIER	NA	NA	486	486	0	539	447	90	16.7
Chewer	IOB_32	chr11	5423982	C	G	OR51J1	MISSENSE	ENSG00000184321:[ENST00000332043(c.156C>G,atC>atG):ENSP00000332473(p.Ile52Met)]	NA	NA	NA	NA	NA	NA	NA	NA	NA	NA	MODIFIER	NA	NA	486	486	0	539	447	90	16.7
Chewer	IOB_32	chr11	20652329	G	A	SLC6A5	MISSENSE	ENSG00000165970:[ENST00000525748(c.1592G>A,cGc>cAc):ENSP00000434364(p.Arg531His)]	NA	chr11*20652329*20652329*COSM182765*G*A*2*large_intestine*2	NA	NA	NA	NA	NA	NA	NA	NA	MODIFIER	NA	NA	154	153	0	199	163	33	16.58
Chewer	IOB_32	chr11	20959484	G	A	NELL1	NCT	ENSG00000165973:[ENST00000530672(n.151+79G>A)]	NA	NA	NA	NA	NA	NA	NA	NA	NA	NA	MODIFIER	NA	NA	100	100	0	113	104	9	7.96
Chewer	IOB_32	chr11	45891196	G	A	CRY2	MISSENSE	ENSG00000121671:[ENST00000443527(c.1085G>A,cGc>cAc):ENSP00000406751(p.Arg362His)]	NA	NA	NA	4.12E-05	6.06E-05	NA	NA	T	BN	NA	MODERATE	N	1	381	381	0	495	487	8	1.62
Chewer	IOB_32	chr11	49175930	G	A	FOLH1	NONSENSE	ENSG00000086205:[ENST00000256999(c.1738C>T,Cga>Tga):ENSP00000256999(p.Arg580Ter)]	NA	NA	NA	NA	NA	NA	NA	NA	NA	NA	MODIFIER	NA	NA	207	207	0	241	202	39	16.18
Chewer	IOB_32	chr11	55371734	C	A	OR4C11	MISSENSE	ENSG00000172188:[ENST00000302231(c.116G>T,gGg>gTg):ENSP00000306651(p.Gly39Val)]	NA	NA	NA	NA	NA	NA	NA	D	PrD	NA	MODERATE	U	0.001	102	102	0	107	77	30	28.04
Chewer	IOB_32	chr11	61276940	G	A	LRRC10B	MISSENSE	ENSG00000204950:[ENST00000378075(c.470G>A,cGc>cAc):ENSP00000367315(p.Arg157His)]	NA	NA	NA	NA	NA	NA	NA	T	BN	NA	MODERATE	U	1	296	294	0	332	323	6	1.81
Chewer	IOB_32	chr11	61507021	C	T	DAGLA	MISSENSE	ENSG00000134780:[ENST00000257215(c.1741C>T,Cgg>Tgg):ENSP00000257215(p.Arg581Trp)]	NA	chr11*61507021*61507021*COSM141343*C*T*1*upper_aerodigestive_tract*1	chr11*61507021*61507021*C*G,T*rs766441182*NA*NA*NA	NA	NA	NA	NA	NA	NA	NA	MODIFIER	NA	NA	201	199	2	281	203	77	27.4
Chewer	IOB_32	chr11	62379869	C	A	EML3	INTRONIC	256364:[XM_005273881.1(c.-292+39G>T)]	NA	NA	NA	NA	NA	NA	NA	NA	NA	NA	MODIFIER	NA	NA	29	29	0	33	28	5	15.15
Chewer	IOB_32	chr11	62379869	C	A	ROM1	INTRONIC	ENSG00000149489:[ENST00000534093(c.-39+477C>A)]	NA	NA	NA	NA	NA	NA	NA	NA	NA	NA	MODIFIER	NA	NA	29	29	0	33	28	5	15.15
Chewer	IOB_32	chr11	63964770	G	T	STIP1	MISSENSE	ENSG00000168439:[ENST00000538945(c.628G>T,Gat>Tat):ENSP00000445957(p.Asp210Tyr)]	NA	NA	chr11*63964770*63964770*G*A*rs149913210*NA*NA*NA	NA	NA	NA	NA	NA	NA	NA	MODIFIER	NA	NA	32	32	0	45	39	6	13.33
Chewer	IOB_32	chr11	64599906	G	C	CDC42BPG	MISSENSE	ENSG00000171219:[ENST00000342711(c.3081C>G,atC>atG):ENSP00000345133(p.Ile1027Met)]	NA	NA	chr11*64599906*64599906*G*A*rs372174582*NA*NA*NA	NA	NA	NA	NA	NA	NA	NA	MODIFIER	NA	NA	73	73	0	115	106	8	6.96
Chewer	IOB_32	chr11	64601325	G	C	CDC42BPG	INTRONIC	ENSG00000171219:[ENST00000342711(c.2467-17C>G)]	NA	NA	NA	8.24E-06	0.00E+00	NA	NA	NA	NA	NA	MODIFIER	NA	NA	155	155	0	189	176	13	6.88
Chewer	IOB_32	chr11	66372745	C	G	CCS	MISSENSE	ENSG00000173992:[ENST00000530961(c.610C>G,Cag>Gag):ENSP00000433609(p.Gln204Glu)]	NA	NA	2.00E-04	NA	NA	2.00E-04	0.00E+00	NA	NA	NA	MODIFIER	NA	NA	234	234	0	351	324	26	7.41
Chewer	IOB_32	chr11	66392698	G	A	RBM14	MISSENSE	ENSG00000239306:[ENST00000310137(c.1351G>A,Gca>Aca):ENSP00000311747(p.Ala451Thr)]	NA	NA	NA	3.41E-05	0.00E+00	NA	NA	NA	NA	NA	MODIFIER	NA	NA	306	303	0	391	381	7	1.79
Chewer	IOB_32	chr11	66392698	G	A	RBM14-RBM4	NCT	ENSG00000248643:[ENST00000511114(n.428+8170G>A)]	NA	NA	NA	3.41E-05	0.00E+00	NA	NA	NA	NA	NA	MODIFIER	NA	NA	306	303	0	391	381	7	1.79
Chewer	IOB_32	chr11	66392698	G	A	RBM4	INTRONIC	ENSG00000173933:[ENST00000514361(c.337+8170G>A)]	NA	NA	NA	3.41E-05	0.00E+00	NA	NA	NA	NA	NA	MODIFIER	NA	NA	306	303	0	391	381	7	1.79
Chewer	IOB_32	chr11	121474974	C	T	SORL1	MISSENSE	ENSG00000137642:[ENST00000532694(c.1130C>T,tCg>tTg):ENSP00000432131(p.Ser377Leu)]	NA	NA	NA	8.24E-06	0.00E+00	NA	NA	T	BN	NA	MODERATE	N	0.01	266	262	0	346	297	46	13.29
Chewer	IOB_32	chr12	22005053	C	T	ABCC9	MISSENSE	ENSG00000069431:[ENST00000544039(c.1628G>A,cGg>cAg):ENSP00000440521(p.Arg543Gln)]	NA	NA	NA	NA	NA	NA	NA	T	BN	NA	MODERATE	D	1	147	147	0	135	115	20	14.81
Chewer	IOB_32	chr12	22005053	C	T	RP11-729I10.2	NCT	ENSG00000257022:[ENST00000539874(n.332-17463C>T)]	NA	NA	NA	NA	NA	NA	NA	T	BN	NA	MODERATE	D	1	147	147	0	135	115	20	14.81
Chewer	IOB_32	chr12	53451341	C	T	TENC1	INTRONIC-SS-ACR-PRX	ENSG00000111077:[ENST00000314276(c.876-10C>T)]	NA	NA	NA	NA	NA	NA	NA	NA	NA	NA	MODIFIER	NA	NA	74	74	0	84	72	12	14.29
Chewer	IOB_32	chr12	58159069	C	A	CYP27B1	INTRONIC	ENSG00000111012:[ENST00000546609(c.501+11G>T)]	NA	NA	NA	NA	NA	NA	NA	NA	NA	NA	MODIFIER	NA	NA	291	290	0	273	267	6	2.2
Chewer	IOB_32	chr12	66308881	C	A	AC090673.2	INTRONIC	ENSG00000267942:[ENST00000601398(c.9+9078G>T)]	NA	NA	NA	NA	NA	NA	NA	T_lc	BN	NA	MODERATE	NA	NA	88	88	0	103	98	5	4.85
Chewer	IOB_32	chr12	66308881	C	A	HMGA2	MISSENSE	ENSG00000149948:[ENST00000354636(c.292C>A,Ctg>Atg):ENSP00000346658(p.Leu98Met)]	NA	NA	NA	NA	NA	NA	NA	T_lc	BN	NA	MODERATE	NA	NA	88	88	0	103	98	5	4.85
Chewer	IOB_32	chr12	90017984	C	A	ATP2B1	SILENT	ENSG00000070961:[ENST00000261173(c.1320G>T,acG>acT):ENSP00000261173(p.Thr440(=))]	NA	NA	chr12*90017984*90017984*C*T*rs752601549*NA*NA*NA	NA	NA	NA	NA	NA	NA	NA	LOW	NA	NA	179	179	0	213	206	5	2.35
Chewer	IOB_32	chr12	100930281	C	A	NR1H4	INTRONIC-SS-ACR-PRX	ENSG00000012504:[ENST00000549996(c.580-9C>A)]	NA	NA	NA	NA	NA	NA	NA	NA	NA	NA	MODIFIER	NA	NA	200	199	0	223	214	6	2.69
Chewer	IOB_32	chr12	102302130	C	A	DRAM1	MISSENSE	ENSG00000136048:[ENST00000549066(c.39C>A,gCt>gAt):ENSP00000447906(p.Ala14Asp)]	NA	NA	NA	NA	NA	NA	NA	D	PoD	NA	MODERATE	D	1	241	241	0	245	238	7	2.86
Chewer	IOB_32	chr12	122326772	G	A	PSMD9	MISSENSE	ENSG00000110801:[ENST00000261817(c.10G>A,Gag>Aag):ENSP00000261817(p.Glu4Lys)]	NA	NA	NA	NA	NA	NA	NA	NA	NA	NA	MODIFIER	NA	NA	66	66	0	70	62	7	10
Chewer	IOB_32	chr12	122326772	G	A	RP11-87C12.2	MISSENSE	ENSG00000256950:[ENST00000546333(c.10G>A,Gag>Aag):ENSP00000477146(p.Glu4Lys)]	NA	NA	NA	NA	NA	NA	NA	NA	NA	NA	MODIFIER	NA	NA	66	66	0	70	62	7	10
Chewer	IOB_32	chr12	124812024	C	T	NCOR2	MISSENSE	ENSG00000196498:[ENST00000404121(c.5797G>A,Gcc>Acc):ENSP00000385618(p.Ala1933Thr)]	NA	NA	NA	NA	NA	NA	NA	NA	NA	NA	MODIFIER	NA	NA	45	45	0	30	24	6	20
Chewer	IOB_32	chr13	42293845	G	A	VWA8	NONSENSE	ENSG00000102763:[ENST00000281496(c.2998C>T,Cga>Tga):ENSP00000281496(p.Arg1000Ter)]	NA	chr13*42293845*42293845*COSM188048*G*A*3*lung,large_intestine*1,2	NA	NA	NA	NA	NA	NA	NA	NA	HIGH	NA	0.937	150	149	0	175	140	34	19.43
Chewer	IOB_32	chr14	24438066	C	T	DHRS4	MISSENSE	ENSG00000157326:[ENST00000559632(c.586C>T,Ccg>Tcg):ENSP00000453983(p.Pro196Ser)]	NA	NA	NA	NA	NA	NA	NA	T	BN	NA	MODERATE	N	0	89	88	0	64	49	15	23.44
Chewer	IOB_32	chr14	24438066	C	T	DHRS4-AS1	NCT	ENSG00000215256:[ENST00000558423(n.111+19872G>A)]	NA	NA	NA	NA	NA	NA	NA	T	BN	NA	MODERATE	N	0	89	88	0	64	49	15	23.44
Chewer	IOB_32	chr14	101526140	C	T	MIR154	EXONIC-NC	ENSG00000207978:[ENST00000385243(n.49C>T)]	NA	NA	2.00E-04	4.13E-05	0.00E+00	2.00E-04	0.00E+00	NA	NA	NA	MODIFIER	NA	NA	93	93	0	104	74	30	28.85
Chewer	IOB_32	chr14	107178940	G	A	IGHV2-70	SILENT	ENSG00000211974:[ENST00000390634(c.237C>T,taC>taT):ENSP00000375043(p.Tyr79(=))]	NA	NA	NA	9.12E-04	3.64E-04	NA	NA	NA	NA	NA	LOW	NA	NA	175	174	0	196	181	5	2.55

Patel *et al.* , 2021. Whole-exome sequencing analysis of oral squamous cell carcinoma delineated by tobacco usage habits
Supplementary Table 4. List of somatic single nucleotide variants identified using whole exome sequencing of OSCC patients

Cohort	Sample	Chromosome	Genomic position	Reference allele	Alternate allele	Genesymbol	Variant type	Variant description	OncoMD	COSMIC	dbSNP	ExAC	ExAC_SAS	1000G	1000G_SAS	SIFT	Polyphen2	CONDEL	VEP	LRT	PhastCon7Way	Total reads in Normal	Reference read support in Normal	Alternative read support in Normal	Total reads in Tumor	Reference read support in Tumor	Alternate read support in Tumor	Alternate allele frequency in Tumor
Chewer	IOB_32	chr15	40268860	C	T	EIF2AK4	SILENT	ENSG00000128829:[ENST00000263791(c.2064C>T,agC>agT):ENSP00000263791(p.Ser688(=))]	NA	NA	chr15*40268860*40268860*C*A*rs774259183*NA*NA*NA	NA	NA	NA	NA	NA	NA	NA	LOW	NA	NA	165	164	0	196	169	27	13.78
Chewer	IOB_32	chr15	43804497	C	A	MAP1A	INTRONIC-SS-DNR-PRX	ENSG00000166963:[ENST00000382031(c.340+8C>A)]	NA	NA	NA	NA	NA	NA	NA	NA	NA	NA	LOW	NA	NA	104	104	0	172	167	5	2.91
Chewer	IOB_32	chr15	67391333	G	C	SMAD3	MISSENSE	ENSG00000166949:[ENST00000559092(c.122G>C,aGa>aCa):ENSP00000453788(p.Arg41Thr)]	NA	NA	NA	NA	NA	NA	NA	NA	NA	NA	MODIFIER	NA	NA	102	102	0	112	85	27	24.11
Chewer	IOB_32	chr15	75651466	C	T	MAN2C1	MISSENSE	ENSG00000140400:[ENST00000565683(c.2126G>A,gGc>gAc):ENSP00000457788(p.Gly709Asp)]	NA	NA	NA	NA	NA	NA	NA	NA	NA	NA	MODIFIER	NA	NA	176	174	0	190	185	5	2.63
Chewer	IOB_32	chr16	2011436	C	G	NDUFB10	INTRONIC	ENSG00000140990:[ENST00000569148(c.237-62C>G)]	NA	NA	chr16*2011436*2011436*C*T*rs957190767*NA*NA*NA	NA	NA	NA	NA	NA	NA	NA	MODIFIER	NA	NA	181	181	0	297	273	22	7.41
Chewer	IOB_32	chr16	3640512	C	A	SLX4	MISSENSE	ENSG00000188827:[ENST00000294008(c.3127G>T,Ggg>Tgg):ENSP00000294008(p.Gly1043Trp)]	NA	NA	NA	8.27E-06	6.06E-05	NA	NA	T	PoD	NA	MODERATE	N	0	172	171	0	222	216	5	2.25
Chewer	IOB_32	chr16	9934691	G	A	GRIN2A	INTRONIC	2903:[XM_005255268.1(c.1654-34C>T)]	NA	NA	NA	2.00E-04	2.66E-04	NA	NA	NA	NA	NA	MODIFIER	NA	NA	96	90	3	120	107	6	5
Chewer	IOB_32	chr16	29990786	C	A	TAOK2	SILENT	ENSG00000149930:[ENST00000279394(c.612C>A,ggC>ggA):ENSP00000279394(p.Gly204(=))]	NA	NA	NA	NA	NA	NA	NA	NA	NA	NA	LOW	NA	NA	163	163	0	257	249	6	2.33
Chewer	IOB_32	chr16	56852590	T	A	NUP93	MISSENSE	ENSG00000102900:[ENST00000308159(c.504T>A,agT>agA):ENSP00000310668(p.Ser168Arg)]	NA	NA	NA	NA	NA	NA	NA	D	BN	NA	MODERATE	NA	NA	107	105	0	114	98	16	14.04
Chewer	IOB_32	chr16	67242587	G	A	LRRC29	SILENT	ENSG00000125122:[ENST00000447579(c.252C>T,taC>taT):ENSP00000414023(p.Tyr84(=))]	NA	NA	NA	NA	NA	NA	NA	NA	NA	NA	LOW	NA	NA	114	113	0	131	117	14	10.69
Chewer	IOB_32	chr16	70563158	C	A	SF3B3	INTRONIC	ENSG00000189091:[ENST00000302516(c.397+56C>A)]	NA	NA	NA	NA	NA	NA	NA	NA	NA	NA	MODIFIER	NA	NA	104	104	0	109	104	5	4.59
Chewer	IOB_32	chr16	88599135	C	T	ZFPM1	INTRONIC	ENSG00000179588:[ENST00000319555(c.1042+18C>T)]	NA	NA	NA	9.03E-06	0.00E+00	NA	NA	NA	NA	NA	MODIFIER	NA	NA	229	229	0	231	188	43	18.61
Chewer	IOB_32	chr17	1960759	G	A	HIC1	MISSENSE	ENSG00000177374:[ENST00000399849(c.775G>A,Gcc>Acc):ENSP00000382742(p.Ala259Thr)]	NA	NA	NA	NA	NA	NA	NA	D	BN	NA	MODERATE	NA	NA	80	80	0	101	82	19	18.81
Chewer	IOB_32	chr17	5328662	T	C	CTC-524C5.2	EXONIC-NC	ENSG00000263272:[ENST00000575890(n.1735A>G)]	NA	NA	NA	7.68E-02	9.46E-02	NA	NA	NA	NA	NA	MODIFIER	NA	NA	49	48	1	58	52	5	8.62
Chewer	IOB_32	chr17	5328662	T	C	RPAIN	NCT	ENSG00000129197:[ENST00000570883(n.340-629T>C)]	NA	NA	NA	7.68E-02	9.46E-02	NA	NA	NA	NA	NA	MODIFIER	NA	NA	49	48	1	58	52	5	8.62
Chewer	IOB_32	chr17	7132844	G	A	DVL2	INTRONIC	1856:[XM_005256504.1(c.738+63C>T)]	NA	NA	NA	2.49E-05	0.00E+00	NA	NA	NA	NA	NA	MODIFIER	NA	NA	210	210	0	211	135	76	36.02
Chewer	IOB_32	chr17	9632336	G	A	USP43	EXONIC-NC	ENSG00000154914:[ENST00000570827(n.3542G>A)]	NA	NA	NA	NA	NA	NA	NA	NA	NA	NA	MODIFIER	NA	NA	104	104	0	140	135	5	3.57
Chewer	IOB_32	chr17	16365683	C	A	C17orf76-AS1	NCT	125144:[XR_243540.1(n.239-464C>A)]	NA	NA	chr17*16365683*16365683*C*T*rs778102342*NA*NA*NA	NA	NA	NA	NA	NA	NA	NA	MODIFIER	NA	NA	144	144	0	182	176	5	2.75
Chewer	IOB_32	chr17	16365683	C	A	FAM211A	SILENT	ENSG00000181350:[ENST00000409083(c.264G>T,tcG>tcT):ENSP00000386504(p.Ser88(=))]	NA	NA	chr17*16365683*16365683*C*T*rs778102342*NA*NA*NA	NA	NA	NA	NA	NA	NA	NA	MODIFIER	NA	NA	144	144	0	182	176	5	2.75
Chewer	IOB_32	chr17	16365683	C	A	FAM211A-AS1	NCT	ENSG00000175061:[ENST00000478103(n.391-464C>A)]	NA	NA	chr17*16365683*16365683*C*T*rs778102342*NA*NA*NA	NA	NA	NA	NA	NA	NA	NA	MODIFIER	NA	NA	144	144	0	182	176	5	2.75
Chewer	IOB_32	chr17	16365683	C	A	LRRC75A-AS1	NCT	125144:[NR_027178.1(n.289-464C>A)]	NA	NA	chr17*16365683*16365683*C*T*rs778102342*NA*NA*NA	NA	NA	NA	NA	NA	NA	NA	MODIFIER	NA	NA	144	144	0	182	176	5	2.75
Chewer	IOB_32	chr17	27902364	G	A	GIT1	MISSENSE	ENSG00000108262:[ENST00000578670(c.394C>T,gCa>gTa):ENSP00000463746(p.Ala132Val)]	NA	NA	NA	NA	NA	NA	NA	NA	NA	NA	MODIFIER	NA	NA	53	52	0	47	40	7	14.89
Chewer	IOB_32	chr17	27902364	G	A	RP11-68I3.2	NCT	ENSG00000264031:[ENST00000581474(n.153+14647G>A)]	NA	NA	NA	NA	NA	NA	NA	NA	NA	NA	MODIFIER	NA	NA	53	52	0	47	40	7	14.89
Chewer	IOB_32	chr17	31324380	C	G	SPACA3	INTRONIC	ENSG00000141316:[ENST00000580599(c.296-83C>G)]	NA	NA	NA	NA	NA	NA	NA	NA	NA	NA	MODIFIER	NA	NA	130	127	2	162	146	6	3.7
Chewer	IOB_32	chr17	39884497	G	A	HAP1	MISSENSE	ENSG00000173805:[ENST00000341193(c.1180C>T,Cgg>Tgg):ENSP00000343170(p.Arg394Trp)]	NA	NA	NA	5.84E-05	0.00E+00	NA	NA	D	PoD	NA	MODERATE	N	0.971	252	251	0	302	253	49	16.23
Chewer	IOB_32	chr17	39884497	G	A	JUP	INTRONIC	ENSG00000173801:[ENST00000540235(c.909+39134C>T)]	NA	NA	NA	5.84E-05	0.00E+00	NA	NA	D	PoD	NA	MODERATE	N	0.971	252	251	0	302	253	49	16.23
Chewer	IOB_32	chr17	43174512	G	A	NMT1	MISSENSE	ENSG00000136448:[ENST00000258960(c.613G>A,Ggc>Agc):ENSP00000258960(p.Gly205Ser)]	NA	NA	NA	5.77E-05	1.21E-04	NA	NA	NA	NA	NA	MODIFIER	NA	NA	101	101	0	120	94	25	20.83
Chewer	IOB_32	chr17	45050437	G	C	GOSR2	INTRONIC	ENSG00000108433:[ENST00000439730(c.676+6445G>C)]	NA	NA	chr17*45050437*45050437*G*A,C*rs879114792*NA*NA*NA	NA	NA	NA	NA	NA	NA	NA	MODIFIER	NA	NA	74	74	0	79	67	11	13.92
Chewer	IOB_32	chr17	45050437	G	C	MIR5089	EXONIC-NC	ENSG00000264999:[ENST00000580047(n.55G>C)]	NA	NA	chr17*45050437*45050437*G*A,C*rs879114792*NA*NA*NA	NA	NA	NA	NA	NA	NA	NA	MODIFIER	NA	NA	74	74	0	79	67	11	13.92
Chewer	IOB_32	chr17	45050437	G	C	RP11-156P1.2	INTRONIC	ENSG00000262633:[ENST00000571841(c.676+6445G>C)]	NA	NA	chr17*45050437*45050437*G*A,C*rs879114792*NA*NA*NA	NA	NA	NA	NA	NA	NA	NA	MODIFIER	NA	NA	74	74	0	79	67	11	13.92
Chewer	IOB_32	chr17	53155557	T	C	STXBP4	INTRONIC-SS-DNR	252983:[XM_005257185.1(c.1359+2T>C)]	NA	NA	NA	NA	NA	NA	NA	NA	NA	NA	HIGH	NA	NA	35	35	0	51	42	9	17.65
Chewer	IOB_32	chr17	56676621	C	T	TEX14	NONSENSE	ENSG00000121101:[ENST00000240361(c.2103G>A,tgG>tgA):ENSP00000240361(p.Trp701Ter)]	NA	NA	NA	NA	NA	NA	NA	NA	NA	NA	HIGH	NA	0	246	246	0	268	262	6	2.24
Chewer	IOB_32	chr17	72764345	G	T	SLC9A3R1	SILENT	ENSG00000109062:[ENST00000413388(c.390G>T,gtG>gtT):ENSP00000464982(p.Val130(=))]	NA	NA	NA	NA	NA	NA	NA	NA	NA	NA	MODIFIER	NA	NA	113	113	0	170	156	14	8.24
Chewer	IOB_32	chr17	72791089	A	G	TMEM104	INTRONIC	ENSG00000109066:[ENST00000417024(c.467-62A>G)]	NA	NA	1.94E-02	NA	NA	1.94E-02	1.23E-02	NA	NA	NA	MODIFIER	NA	NA	65	65	0	91	84	5	5.49
Chewer	IOB_32	chr17	78313136	T	A	RNF213	MISSENSE	ENSG00000173821:[ENST00000582970(c.4969T>A,Ttg>Atg):ENSP00000464087(p.Leu1657Met)]	NA	NA	NA	NA	NA	NA	NA	NA	NA	NA	MODIFIER	NA	NA	277	276	0	314	260	54	17.2
Chewer	IOB_32	chr18	21946573	A	T	OSBPL1A	EXONIC-NC	ENSG00000141447:[ENST00000582618(n.798T>A)]	NA	NA	1.20E-03	NA	NA	1.20E-03	0.00E+00	NA	NA	NA	MODIFIER	NA	NA	23	23	0	34	27	7	20.59
Chewer	IOB_32	chr18	21946573	A	T	RP11-621L6.2	NCT	ENSG00000264365:[ENST00000579347(n.86-894A>T)]	NA	NA	1.20E-03	NA	NA	1.20E-03	0.00E+00	NA	NA	NA	MODIFIER	NA	NA	23	23	0	34	27	7	20.59
Chewer	IOB_32	chr18	60646229	G	T	PHLPP1	SILENT	ENSG00000081913:[ENST00000400316(c.3183G>T,cgG>cgT):ENSP00000383170(p.Arg1061(=))]	NA	chr18*60646229*60646229*COSM1141487*G*T*1*lung*1|chr18*60646229*60646229*COSM1141486*G*T*1*lung*1|chr18*60646229*60646229*COSM564106*G*T*1*lung*1	NA	NA	NA	NA	NA	NA	NA	NA	LOW	NA	NA	306	303	0	372	328	42	11.29
Chewer	IOB_32	chr18	72343898	C	T	ZNF407	MISSENSE	ENSG00000215421:[ENST00000299687(c.923C>T,tCt>tTt):ENSP00000299687(p.Ser308Phe)]	NA	NA	NA	NA	NA	NA	NA	D	PoD	NA	MODERATE	U	0.011	145	144	0	173	135	38	21.97
Chewer	IOB_32	chr18	77470291	G	A	CTDP1	INTRONIC	ENSG00000060069:[ENST00000075430(c.773-55G>A)]	NA	NA	2.00E-04	NA	NA	2.00E-04	0.00E+00	NA	NA	NA	MODIFIER	NA	NA	176	176	0	190	165	23	12.11
Chewer	IOB_32	chr19	2834045	G	A	ZNF554	MISSENSE	ENSG00000172006:[ENST00000317243(c.812G>A,aGa>aAa):ENSP00000321132(p.Arg271Lys)]	NA	chr19*2834045*2834045*COSM178455*G*T*1*large_intestine*1	NA	NA	NA	NA	NA	NA	NA	NA	MODIFIER	NA	NA	258	258	0	260	217	43	16.54
Chewer	IOB_32	chr19	3543913	G	A	C19orf71	MISSENSE	ENSG00000183397:[ENST00000329493(c.535G>A,Ggc>Agc):ENSP00000327950(p.Gly179Ser)]	NA	NA	NA	4.92E-05	1.29E-04	NA	NA	D	BN	NA	MODERATE	NA	NA	92	92	0	83	72	10	12.05
Chewer	IOB_32	chr19	3543913	G	A	MFSD12	INTRONIC	126321:[XM_005259490.1(c.1249+894C>T)]	NA	NA	NA	4.92E-05	1.29E-04	NA	NA	D	BN	NA	MODERATE	NA	NA	92	92	0	83	72	10	12.05
Chewer	IOB_32	chr19	3589834	T	A	GIPC3	MISSENSE	ENSG00000179855:[ENST00000322315(c.711T>A,agT>agA):ENSP00000319254(p.Ser237Arg)]	NA	NA	NA	NA	NA	NA	NA	D	BN	NA	MODERATE	D	0.04	62	60	0	44	36	8	18.18
Chewer	IOB_32	chr19	4054284	G	A	ZBTB7A	MISSENSE	ENSG00000178951:[ENST00000322357(c.947C>T,gCc>gTc):ENSP00000323670(p.Ala316Val)]	NA	NA	NA	NA	NA	NA	NA	NA	NA	NA	MODERATE	NA	1	464	460	2	462	380	80	17.32
Chewer	IOB_32	chr19	5245856	C	T	PTPRS	MISSENSE	ENSG00000105426:[ENST00000348075(c.880G>A,Gcc>Acc):ENSP00000269907(p.Ala294Thr)]	NA	NA	chr19*5245856*5245856*C*A*rs751418551*NA*NA*NA	NA	NA	NA	NA	D	PoD	NA	MODERATE	U	1	214	213	0	266	260	5	1.88
Chewer	IOB_32	chr19	9270856	C	A	ZNF317	MISSENSE	ENSG00000130803:[ENST00000247956(c.535C>A,Cct>Act):ENSP00000247956(p.Pro179Thr)]	NA	NA	NA	NA	NA	NA	NA	T	BN	NA	MODERATE	N	0	184	184	0	251	242	6	2.39
Chewer	IOB_32	chr19	10746059	C	T	SLC44A2	NCT	ENSG00000129353:[ENST00000588393(n.653-48C>T)]	NA	NA	NA	NA	NA	NA	NA	NA	NA	NA	MODIFIER	NA	NA	130	130	0	132	127	5	3.79
Chewer	IOB_32	chr19	16060572	C	T	OR10H4	MISSENSE	ENSG00000176231:[ENST00000322107(c.755C>T,aCg>aTg):ENSP00000318834(p.Thr252Met)]	NA	NA	3.40E-03	5.53E-03	2.18E-03	3.39E-03	0.00E+00	D	BN	NA	MODERATE	U	0.151	115	115	0	154	123	29	18.83
Chewer	IOB_32	chr19	19329904	C	T	NCAN	MISSENSE	ENSG00000130287:[ENST00000252575(c.254C>T,tCg>tTg):ENSP00000252575(p.Ser85Leu)]	NA	NA	NA	3.38E-05	6.50E-05	NA	NA	D	PoD	NA	MODERATE	N	0.033	179	178	0	207	178	29	14.01
Chewer	IOB_32	chr19	33666405	C	A	WDR88	MISSENSE	ENSG00000166359:[ENST00000355868(c.1346C>A,gCa>gAa):ENSP00000348129(p.Ala449Glu)]	NA	NA	NA	NA	NA	NA	NA	NA	NA	NA	MODIFIER	NA	NA	94	94	0	134	126	6	4.48
Chewer	IOB_32	chr19	37100582	G	C	ZNF382	INTRONIC	ENSG00000161298:[ENST00000439428(c.10-247G>C)]	NA	NA	NA	NA	NA	NA	NA	NA	NA	NA	MODIFIER	NA	NA	386	386	0	377	325	48	12.73
Chewer	IOB_32	chr19	48204950	G	T	CTD-2571L23.8	NCT	ENSG00000268746:[ENST00000599924(n.87-30372G>T)]	NA	NA	NA	NA	NA	NA	NA	D	PrD	NA	MODERATE	NA	NA	103	103	0	90	75	12	13.33
Chewer	IOB_32	chr19	48204950	G	T	GLTSCR1	MISSENSE	ENSG00000063169:[ENST00000396720(c.3961G>T,Gtc>Ttc):ENSP00000379946(p.Val1321Phe)]	NA	NA	NA	NA	NA	NA	NA	D	PrD	NA	MODERATE	NA	NA	103	103	0	90	75	12	13.33
Chewer	IOB_32	chr19	50826904	C	T	KCNC3	MISSENSE	ENSG00000131398:[ENST00000376959(c.1306G>A,Gtg>Atg):ENSP00000366158(p.Val436Met)]	NA	chr19*50826904*50826904*COSM1395428*C*T*1*large_intestine*1|chr19*50826904*50826904*COSM386960*C*A*1*lung*1	NA	8.27E-06	0.00E+00	NA	NA	D	PrD	NA	MODERATE	U	1	359	359	0	425	377	45	10.59
Chewer	IOB_32	chr19	52394318	C	T	CTC-429C10.2	NCT	ENSG00000268095:[ENST00000600329(n.102+2939C>T)]	NA	NA	NA	NA	NA	NA	NA	NA	NA	NA	MODIFIER	NA	NA	121	120	1	154	131	23	14.94
Chewer	IOB_32	chr19	52394318	C	T	ZNF649	SILENT	ENSG00000198093:[ENST00000354957(c.1071G>A,caG>caA):ENSP00000347043(p.Gln357(=))]	NA	NA	NA	NA	NA	NA	NA	NA	NA	NA	MODIFIER	NA	NA	121	120	1	154	131	23	14.94
Chewer	IOB_32	chr19	52394318	C	T	ZNF649-AS1	NCT	101928571:[XR_244010.1(n.95+2939C>T)]	NA	NA	NA	NA	NA	NA	NA	NA	NA	NA	MODIFIER	NA	NA	121	120	1	154	131	23	14.94
Chewer	IOB_32	chr19	54584544	G	A	TARM1	INTRONIC	ENSG00000248385:[ENST00000432826(c.34+32C>T)]	NA	NA	NA	NA	NA	NA	NA	NA	NA	NA	MODIFIER	NA	NA	70	70	0	80	74	5	6.25
Chewer	IOB_32	chr19	58549584	G	T	ZSCAN1	MISSENSE	ENSG00000152467:[ENST00000391700(c.380G>T,cGc>cTc):ENSP00000375581(p.Arg127Leu)]	NA	NA	chr19*58549584*58549584*G*A*rs749879977*NA*NA*NA	NA	NA	NA	NA	NA	NA	NA	MODIFIER	NA	NA	86	86	0	126	120	5	3.97
Chewer	IOB_32	chr2	27849997	C	G	CCDC121	MISSENSE	ENSG00000176714:[ENST00000394775(c.1156G>C,Gaa>Caa):ENSP00000412150(p.Glu386Gln)]	NA	NA	chr2*27849991*27850016*TGTTTTCTAAGTGGCTTTGAGTAGCC*T*rs766688052*NA*NA*NA	NA	NA	NA	NA	T	BN	NA	MODERATE	N	0	104	103	0	142	127	15	10.56
Chewer	IOB_32	chr2	27849997	C	G	RP11-158I13.2	NCT	ENSG00000259080:[ENST00000505973(n.1389-2729C>G)]	NA	NA	chr2*27849991*27850016*TGTTTTCTAAGTGGCTTTGAGTAGCC*T*rs766688052*NA*NA*NA	NA	NA	NA	NA	T	BN	NA	MODERATE	N	0	104	103	0	142	127	15	10.56
Chewer	IOB_32	chr2	27849997	C	G	ZNF512	INTRONIC	ENSG00000243943:[ENST00000556601(c.1113-2729C>G)]	NA	NA	chr2*27849991*27850016*TGTTTTCTAAGTGGCTTTGAGTAGCC*T*rs766688052*NA*NA*NA	NA	NA	NA	NA	T	BN	NA	MODERATE	N	0	104	103	0	142	127	15	10.56

Cohort	Sample	Chromosome	Genomic position	Reference allele	Alternate allele	Genesymbol	Variant type	Variant description	OncoMD	COSMIC	dbSNP	ExAC	ExAC_SAS	1000G	1000G_SAS	SIFT	Polyphen2	CONDEL	VEP	LRT	PhastCon7Way	Total reads in Normal	Reference read support in Normal	Alternative read support in Normal	Total reads in Tumor	Reference read support in Tumor	Alternate read support in Tumor	Alternate allele frequency in Tumor
Chewer	IOB_32	chr2	50149262	G	T	NRXN1	SILENT	ENSG00000179915:[ENST00000412315(c.452C>A,ctC>ctA):ENSP00000396738(p.Leu151(=))]	NA	NA	chr2*50149262*50149262*G*A,C*rs111725706*NA*NA*NA	NA	NA	NA	NA	NA	NA	NA	MODIFIER	NA	NA	256	255	1	256	249	6	2.34
Chewer	IOB_32	chr2	80101326	G	A	CTNNA2	MISSENSE	ENSG00000066032:[ENST00000361291(c.812G>A,aGa>aAa):ENSP00000355398(p.Arg271Lys)]	NA	chr2*80101326*80101326*COSM383268*G*T*1*lung*1|chr2*80101326*80101326*COSM383267*G*T*1*lung	NA	NA	NA	NA	NA	T	BN	NA	MODERATE	D	1	108	108	0	127	91	34	26.77
Chewer	IOB_32	chr2	97285361	G	A	KANSL3	INTRONIC	ENSG00000114982:[ENST00000416138(c.216-6005C>T)]	NA	NA	NA	NA	NA	NA	NA	NA	NA	NA	MODIFIER	NA	NA	173	173	0	185	154	31	16.76
Chewer	IOB_32	chr2	166747352	G	T	TTC21B	MISSENSE	ENSG00000123607:[ENST00000243344(c.3097C>A,Ctt>Att):ENSP00000243344(p.Leu1033Ile)]	NA	chr2*166747352*166747352*COSM1008688*G*T*2*haematopoietic_and_lymphoid_tissue,endometrium*1,1	NA	8.40E-06	0.00E+00	NA	NA	T	BN	NA	MODERATE	N	1	157	155	1	159	151	5	3.14
Chewer	IOB_32	chr2	168107605	C	A	XIRP2	MISSENSE	ENSG00000163092:[ENST00000409273(c.9037C>A,Cca>Aca):ENSP00000387255(p.Pro3013Thr)]	NA	NA	NA	NA	NA	NA	NA	NA	NA	NA	MODIFIER	NA	NA	162	162	0	170	146	24	14.12
Chewer	IOB_32	chr2	233246466	C	A	ALPP	SILENT	ENSG00000163283:[ENST00000392027(c.1569C>A,gcC>gcA):ENSP00000375881(p.Ala523(=))]	NA	NA	7.99E-04	4.16E-03	1.08E-02	7.99E-04	2.00E-03	NA	NA	NA	LOW	NA	NA	20	20	0	31	26	5	16.13
Chewer	IOB_32	chr2	241419000	C	A	ANKMY1	3UTR	ENSG00000144504:[ENST00000272972(c.*26G>T)]	NA	NA	NA	NA	NA	NA	NA	NA	NA	NA	MODIFIER	NA	NA	89	89	0	91	71	20	21.98
Chewer	IOB_32	chr20	2641562	G	A	IDH3B	MISSENSE	ENSG00000101365:[ENST00000380843(c.391C>T,Cgg>Tgg):ENSP00000370223(p.Arg131Trp)]	NA	chr20*2641562*2641562*COSM723230*G*A*1*lung*1	NA	8.25E-06	0.00E+00	NA	NA	D	PoD	NA	MODERATE	D	0.911	76	76	0	129	124	5	3.88
Chewer	IOB_32	chr20	2843999	A	G	VPS16	SILENT	ENSG00000215305:[ENST00000380445(c.1431A>G,gaA>gaG):ENSP00000369810(p.Glu477(=))]	NA	NA	NA	NA	NA	NA	NA	NA	NA	NA	LOW	NA	0.996	291	287	0	385	317	65	16.88
Chewer	IOB_32	chr20	2845181	C	A	PTPRA	INTRONIC	ENSG00000132670:[ENST00000380393(c.-484-12C>A)]	NA	NA	chr20*2845181*2845182*CT*C*rs781450688*NA*NA*NA	NA	NA	NA	NA	NA	NA	NA	MODIFIER	NA	NA	200	199	0	243	237	6	2.47
Chewer	IOB_32	chr20	2845181	C	A	VPS16	INTRONIC	ENSG00000215305:[ENST00000380469(c.1387-12C>A)]	NA	NA	chr20*2845181*2845182*CT*C*rs781450688*NA*NA*NA	NA	NA	NA	NA	NA	NA	NA	MODIFIER	NA	NA	200	199	0	243	237	6	2.47
Chewer	IOB_32	chr20	16553947	G	A	KIF16B	5UTR	ENSG00000089177:[ENST00000354981(c.-27C>T)]	NA	NA	NA	NA	NA	NA	NA	NA	NA	NA	MODIFIER	NA	NA	86	86	0	104	89	15	14.42
Chewer	IOB_32	chr20	33519847	C	A	GSS	MISSENSE	ENSG00000100983:[ENST00000216951(c.924G>T,caG>caT):ENSP00000216951(p.Gln308His)]	NA	NA	NA	NA	NA	NA	NA	D	PrD	NA	MODERATE	D	1	78	78	0	119	114	5	4.2
Chewer	IOB_32	chr20	39745037	G	C	PLCG1-AS1	NCT	101927117:[NR_109889.1(n.711-15108C>G)]	NA	NA	NA	NA	NA	NA	NA	NA	NA	NA	LOW	NA	NA	62	62	0	77	66	10	12.99
Chewer	IOB_32	chr20	39745037	G	C	RP1-1J6.2	NCT	ENSG00000226648:[ENST00000454626(n.714-15108C>G)]	NA	NA	NA	NA	NA	NA	NA	NA	NA	NA	LOW	NA	NA	62	62	0	77	66	10	12.99
Chewer	IOB_32	chr20	39745037	G	C	TOP1	INTRONIC	7150:[XM_005260541.1(c.1726+5G>C)]	NA	NA	NA	NA	NA	NA	NA	NA	NA	NA	LOW	NA	NA	62	62	0	77	66	10	12.99
Chewer	IOB_32	chr20	50401180	C	T	SALL4	MISSENSE	ENSG00000101115:[ENST00000371539(c.455G>A,cGt>cAt):ENSP00000360594(p.Arg152His)]	NA	chr20*50401180*50401180*COSM1028036*C*T*1*endometrium*1	NA	NA	NA	NA	NA	D	BN	NA	MODERATE	D	1	98	98	0	119	104	15	12.61
Chewer	IOB_32	chr20	60990769	C	A	RBBP8NL	INTRONIC	ENSG00000130701:[ENST00000252998(c.545-53G>T)]	NA	NA	chr20*60990769*60990769*C*T*rs374843458*1*0.9966*0.003395	NA	NA	NA	NA	NA	NA	NA	MODIFIER	NA	NA	125	125	0	148	143	5	3.38
Chewer	IOB_32	chr20	62202095	C	T	HELZ2	SILENT	ENSG00000130589:[ENST00000467148(c.405G>A,gtG>gtA):ENSP00000417401(p.Val135(=))]	NA	NA	NA	NA	NA	NA	NA	NA	NA	NA	MODIFIER	NA	NA	110	110	0	135	113	22	16.3
Chewer	IOB_32	chr21	11098728	C	T	BAGE2	5UTR	85319:[NM_182482.2(c.-11G>A)]	NA	NA	NA	2.60E-01	2.67E-01	NA	NA	NA	NA	NA	MODIFIER	NA	NA	86	74	9	71	42	26	36.62
Chewer	IOB_32	chr21	33103978	C	A	SCAF4	NONSENSE-SS-PRX	ENSG00000156304:[ENST00000485790(n.297G>T)]	NA	NA	NA	NA	NA	NA	NA	NA	NA	NA	HIGH	NA	1	415	413	1	434	387	46	10.6
Chewer	IOB_32	chr21	37619898	T	A	DOPEY2	MISSENSE	ENSG00000142197:[ENST00000399151(c.4781T>A,cTt>cAt):ENSP00000382104(p.Leu1594His)]	NA	NA	NA	NA	NA	NA	NA	NA	NA	NA	MODIFIER	NA	NA	306	305	0	335	298	36	10.75
Chewer	IOB_32	chr22	25436518	C	T	KIAA1671	MISSENSE	ENSG00000197077:[ENST00000358431(c.3421C>T,Cgt>Tgt):ENSP00000351207(p.Arg1141Cys)]	NA	NA	NA	NA	NA	NA	NA	T	BN	NA	MODERATE	N	0.003	168	168	0	157	130	26	16.56
Chewer	IOB_32	chr22	26937013	C	T	TPST2	MISSENSE	ENSG00000128294:[ENST00000338754(c.584G>A,cGc>cAc):ENSP00000339813(p.Arg195His)]	NA	NA	NA	NA	NA	NA	NA	NA	NA	NA	MODERATE	NA	1	303	303	0	252	246	5	1.98
Chewer	IOB_32	chr22	38005239	G	A	GGA1	INTRONIC	ENSG00000100083:[ENST00000429218(c.-173+329G>A)]	NA	NA	NA	NA	NA	NA	NA	NA	NA	NA	MODIFIER	NA	NA	277	275	0	220	214	5	2.27
Chewer	IOB_32	chr22	44332993	G	A	PNPLA3	MISSENSE	ENSG00000100344:[ENST00000423180(c.808G>A,Gcc>Acc):ENSP00000397987(p.Ala270Thr)]	NA	NA	NA	8.25E-05	6.06E-05	NA	NA	NA	NA	NA	MODIFIER	NA	NA	106	106	0	102	97	5	4.9
Chewer	IOB_32	chr3	7620249	G	C	GRM7	MISSENSE	ENSG00000196277:[ENST00000357716(c.1656G>C,caG>caC):ENSP00000350348(p.Gln552His)]	NA	NA	NA	NA	NA	NA	NA	NA	NA	NA	MODIFIER	NA	NA	108	108	0	100	90	9	9
Chewer	IOB_32	chr3	11850959	G	A	TAMM41	EXONIC-NC	132001:[NR_104314.1(n.1338C>T)]	NA	NA	NA	NA	NA	NA	NA	NA	NA	NA	MODIFIER	NA	NA	334	333	0	311	304	5	1.61
Chewer	IOB_32	chr3	14105766	C	A	TPRXL	SILENT	ENSG00000180438:[ENST00000326972(c.90C>A,ctC>ctA):ENSP00000322097(p.Leu30(=))]	NA	NA	chr3*14105766*14105766*C*T*rs942829850*NA*NA*NA	NA	NA	NA	NA	NA	NA	NA	MODIFIER	NA	NA	88	88	0	59	53	6	10.17
Chewer	IOB_32	chr3	17333395	C	A	TBC1D5	MISSENSE	ENSG00000131374:[ENST00000253692(c.1237G>T,Gat>Tat):ENSP00000253692(p.Asp413Tyr)]	NA	NA	NA	NA	NA	NA	NA	D	PrD	NA	MODERATE	D	1	154	154	0	149	116	33	22.15
Chewer	IOB_32	chr3	48682775	C	A	CELSR3	INTRONIC	ENSG00000008300:[ENST00000544264(c.7812+101G>T)]	NA	NA	NA	NA	NA	NA	NA	NA	NA	NA	MODIFIER	NA	NA	180	179	1	173	167	5	2.89
Chewer	IOB_32	chr3	101568559	G	A	NFKBIZ	SILENT	ENSG00000144802:[ENST00000326151(c.87G>A,ccG>ccA):ENSP00000325593(p.Pro29(=))]	NA	NA	NA	NA	NA	NA	NA	NA	NA	NA	MODIFIER	NA	NA	132	132	0	139	122	16	11.51
Chewer	IOB_32	chr3	169802385	C	A	GPR160	MISSENSE	ENSG00000173890:[ENST00000355897(c.625C>A,Cag>Aag):ENSP00000348161(p.Gln209Lys)]	NA	NA	NA	NA	NA	NA	NA	T	BN	NA	MODERATE	N	0.028	111	111	0	126	101	23	18.25
Chewer	IOB_32	chr3	182623535	G	C	ATP11B	INTRONIC	23200:[XM_005247244.1(c.2658+6975G>C)]	NA	NA	NA	NA	NA	NA	NA	NA	NA	NA	MODIFIER	NA	NA	147	146	0	159	151	8	5.03
Chewer	IOB_32	chr3	182948847	C	A	MCF2L2	NCT	ENSG00000053524:[ENST00000488149(n.2204-42G>T)]	NA	NA	NA	NA	NA	NA	NA	NA	NA	NA	MODIFIER	NA	NA	226	226	0	301	295	5	1.66
Chewer	IOB_32	chr3	195251652	G	T	PPP1R2	SILENT	ENSG00000184203:[ENST00000328432(c.273C>A,gcC>gcA):ENSP00000328178(p.Ala91(=))]	NA	NA	NA	NA	NA	NA	NA	NA	NA	NA	MODIFIER	NA	NA	95	95	0	111	106	5	4.5
Chewer	IOB_32	chr3	195400848	G	A	LINC00969	EXONIC-NC	ENSG00000242086:[ENST00000429897(n.1312G>A)]	NA	NA	NA	1.31E-01	2.49E-01	NA	NA	NA	NA	NA	MODIFIER	NA	NA	64	60	3	67	47	18	26.87
Chewer	IOB_32	chr3	195400848	G	A	SDHAP2	EXONIC-NC	727956:[NR_003265.3(n.1482G>A)]	NA	NA	NA	1.31E-01	2.49E-01	NA	NA	NA	NA	NA	MODIFIER	NA	NA	64	60	3	67	47	18	26.87
Chewer	IOB_32	chr4	59439	G	A	ZNF595	SILENT	ENSG00000197701:[ENST00000509152(c.120G>A,ctG>ctA):ENSP00000434858(p.Leu40(=))]	NA	chr4*59439*59439*COSM587829*G*T*2*lung*2	NA	NA	NA	NA	NA	NA	NA	NA	MODIFIER	NA	NA	1030	1026	2	1174	1153	17	1.45
Chewer	IOB_32	chr4	4317454	G	C	ZBTB49	NCT	166793:[XR_241646.1(n.1504+2613G>C)]	NA	NA	NA	NA	NA	NA	NA	NA	NA	NA	MODIFIER	NA	NA	266	263	1	358	322	35	9.78
Chewer	IOB_32	chr4	4861965	G	A	MSX1	SILENT	ENSG00000163132:[ENST00000382723(c.339G>A,cgG>cgA):ENSP00000372170(p.Arg113(=))]	NA	NA	NA	NA	NA	NA	NA	NA	NA	NA	LOW	NA	NA	126	122	0	113	93	16	14.16
Chewer	IOB_32	chr4	8608572	C	T	CPZ	MISSENSE	ENSG00000109625:[ENST00000382480(c.604C>T,Cgc>Tgc):ENSP00000371920(p.Arg202Cys)]	NA	NA	NA	1.41E-04	1.85E-04	NA	NA	NA	NA	NA	MODIFIER	NA	NA	241	240	0	301	215	85	28.24
Chewer	IOB_32	chr4	8608572	C	T	GPR78	3UTR	ENSG00000155269:[ENST00000514302(c.*923C>T)]	NA	NA	NA	1.41E-04	1.85E-04	NA	NA	NA	NA	NA	MODIFIER	NA	NA	241	240	0	301	215	85	28.24
Chewer	IOB_32	chr4	18023231	C	T	LCORL	MISSENSE	ENSG00000178177:[ENST00000326877(c.144G>A,atG>atA):ENSP00000317566(p.Met48Ile)]	NA	NA	NA	NA	NA	NA	NA	NA	NA	NA	MODIFIER	NA	NA	192	191	0	216	180	36	16.67
Chewer	IOB_32	chr4	25005835	C	A	LGI2	SILENT	ENSG00000153012:[ENST00000382114(c.876G>T,gtG>gtT):ENSP00000371548(p.Val292(=))]	NA	NA	NA	NA	NA	NA	NA	NA	NA	NA	LOW	NA	NA	50	50	0	86	81	5	5.81
Chewer	IOB_32	chr4	40796535	C	A	NSUN7	NCT	ENSG00000179299:[ENST00000423784(n.287+42C>A)]	NA	NA	NA	NA	NA	NA	NA	NA	NA	NA	MODIFIER	NA	NA	174	174	0	170	165	5	2.94
Chewer	IOB_32	chr4	46305465	G	C	GABRA2	INTRONIC	ENSG00000151834:[ENST00000513005(c.*290+6725C>G)]	NA	NA	NA	NA	NA	NA	NA	NA	NA	NA	MODIFIER	NA	NA	158	158	0	197	173	20	10.15
Chewer	IOB_32	chr4	79399081	C	T	FRAS1	MISSENSE	ENSG00000138759:[ENST00000264895(c.7964C>T,gCc>gTc):ENSP00000264895(p.Ala2655Val)]	NA	NA	NA	NA	NA	NA	NA	D	BN	NA	MODERATE	NA	NA	160	159	0	205	199	6	2.93
Chewer	IOB_32	chr4	87691299	C	A	PTPN13	MISSENSE	ENSG00000163629:[ENST00000427191(c.4693C>A,Ctt>Att):ENSP00000408368(p.Leu1565Ile)]	NA	NA	NA	NA	NA	NA	NA	T	BN	NA	MODERATE	N	1	183	183	0	191	184	7	3.66
Chewer	IOB_32	chr4	90170301	C	A	GPRIN3	NONSENSE	ENSG00000185477:[ENST00000333209(c.961G>T,Gag>Tag):ENSP00000328672(p.Glu321Ter)]	NA	NA	chr4*90170301*90170301*C*G*rs780926554*NA*NA*NA	NA	NA	NA	NA	NA	NA	NA	HIGH	NA	1	150	150	0	216	192	23	10.65
Chewer	IOB_32	chr4	105412130	A	T	AC004053.1	EXONIC-NC	ENSG00000245384:[ENST00000500179(n.9A>T)]	NA	NA	NA	NA	NA	NA	NA	NA	NA	NA	MODIFIER	NA	NA	159	159	0	216	177	37	17.13
Chewer	IOB_32	chr4	105412130	A	T	CXXC4	MISSENSE	ENSG00000168772:[ENST00000394767(c.830T>A,cTg>cAg):ENSP00000378248(p.Leu277Gln)]	NA	NA	NA	NA	NA	NA	NA	NA	NA	NA	MODIFIER	NA	NA	159	159	0	216	177	37	17.13
Chewer	IOB_32	chr4	114262932	G	A	ANK2	MISSENSE	ENSG00000145362:[ENST00000514960(c.1019G>A,Gcc>Acc):ENSP00000422853(p.Ala341Thr)]	NA	chr4*114262932*114262932*COSM3409009*G*A*1*central_nervous_system*1|chr4*114262932*114262932*COSM3409008*G*A*1*central_nervous_system*1	NA	NA	NA	NA	NA	D_lc	PrD	NA	MODERATE	NA	NA	262	262	0	259	251	5	1.93
Chewer	IOB_32	chr4	187527363	G	C	FAT1	NONSENSE	ENSG00000083857:[ENST00000441802(c.10211C>G,tCa>tGa):ENSP00000406229(p.Ser3404Ter)]	NA	NA	NA	NA	NA	NA	NA	NA	NA	NA	HIGH	NA	1	57	56	0	72	58	14	19.44
Chewer	IOB_32	chr5	112487064	C	T	MCC	MISSENSE	ENSG00000171444:[ENST00000408903(c.683G>A,cGt>cAt):ENSP00000386227(p.Arg228His)]	NA	chr5*112487064*112487064*COSM124356*C*T*2*upper_aerodigestive_tract,large_intestine*1,1|chr5*112487064*112487064*COSM124355*C*T*2*upper_aerodigestive_tract,large_intestine*1,1	NA	2.54E-05	0.00E+00	NA	NA	NA	NA	NA	MODERATE	NA	1	159	158	0	267	226	41	15.36
Chewer	IOB_32	chr5	129037220	G	T	ADAMTS19	MISSENSE	ENSG00000145808:[ENST00000274487(c.3076G>T,Gcc>Tcc):ENSP00000274487(p.Ala1026Ser)]	NA	chr5*129037220*129037220*COSM242824*G*T*1*prostate*1|chr5*129037220*129037220*COSM1060480*G*A*1*endometrium*1	chr5*129037220*129037220*G*A*rs746700188*NA*NA*NA	NA	NA	NA	NA	T	BN	NA	MODERATE	N	0.626	159	158	0	149	129	20	13.42
Chewer	IOB_32	chr5	129037220	G	T	CTC-575N7.1	NCT	ENSG00000251680:[ENST00000503616(n.404+33416C>A)]	NA	chr5*129037220*129037220*COSM242824*G*T*1*prostate*1|chr5*129037220*129037220*COSM1060480*G*A*1*endometrium*1	chr5*129037220*129037220*G*A*rs746700188*NA*NA*NA	NA	NA	NA	NA	T	BN	NA	MODERATE	N	0.626	159	158	0	149	129	20	13.42
Chewer	IOB_32	chr5	132046646	C	T	AC004237.1	NCT	ENSG00000230612:[ENST00000431165(n.274-10401C>T)]	NA	NA	NA	NA	NA	NA	NA	NA	NA	NA	MODIFIER	NA	NA	276	274	0	280	235	44	15.71
Chewer	IOB_32	chr5	132046646	C	T	KIF3A	INTRONIC-SS-DNR-PRX	ENSG00000131437:[ENST00000378735(c.1228+5G>A)]	NA	NA	NA	NA	NA	NA	NA	NA	NA	NA	MODIFIER	NA	NA	276	274	0	280	235	44	15.71
Chewer	IOB_32	chr5	132046646	C	T	NA	NCT	101927783:[XR_246588.1(n.468-10401C>T)]	NA	NA	NA	NA	NA	NA	NA	NA	NA	NA	MODIFIER	NA	NA	276	274	0	280	235	44	15.71
Chewer	IOB_32	chr5	140202151	A	G	PCDHA1	INTRONIC	ENSG00000204970:[ENST00000504120(c.2394+33882A>G)]	NA	NA	NA	NA	NA	NA	NA	NA	NA	NA	MODIFIER	NA	NA	145	145	0	191	161	30	15.71
Chewer	IOB_32	chr5	140202151	A	G	PCDHA2	INTRONIC	ENSG00000204969:[ENST00000526136(c.2388+25214A>G)]	NA	NA	NA	NA	NA	NA	NA	NA	NA	NA	MODIFIER	NA	NA	145	145	0	191	161	30	15.71
Chewer	IOB_32	chr5	140202151	A	G	PCDHA3	INTRONIC	ENSG00000255408:[ENST00000522353(c.2394+18975A>G)]	NA	NA	NA	NA	NA	NA	NA	NA	NA	NA	MODIFIER	NA	NA	145	145	0	191	161	30	15.71
Chewer	IOB_32	chr5	140202151	A	G	PCDHA4	INTRONIC	ENSG00000204967:[ENST00000512229(c.2385+12994A>G)]	NA	NA	NA	NA	NA	NA	NA	NA	NA	NA	MODIFIER	NA	NA	145	145	0	191	161	30	15.71
Chewer	IOB_32	chr5	140202151	A	G	PCDHA5	MISSENSE	ENSG00000204965:[ENST00000378126(c.791A>G,aAc>aGc):ENSP00000367366(p.Asn264Ser)]	NA	NA	NA	NA	NA	NA	NA	NA	NA	NA	MODIFIER	NA	NA	145	145	0	191	161	30	15.71
Chewer	IOB_32	chr6	18122358	G	A	NHLRC1	SILENT	ENSG00000187566:[ENST00000340650(c.480C>T,tgC>tgT):ENSP00000345464(p.Cys160(=))]	NA	NA	NA	NA	NA	NA	NA	NA	NA	NA	LOW	NA	0	305	302	3	353	341	8	2.27

Patel *et al.* , 2021. Whole-exome sequencing analysis of oral squamous cell carcinoma delineated by tobacco usage habits
Supplementary Table 4. List of somatic single nucleotide variants identified using whole exome sequencing of OSCC patients

Cohort	Sample	Chromosome	Genomic position	Reference allele	Alternate allele	Genesymbol	Variant type	Variant description	OncoMD	COSMIC	dbSNP	ExAC	ExAC_SAS	1000G	1000G_SAS	SIFT	Polyphen2	CONDEL	VEP	LRT	PhastCon7Way	Total reads in Normal	Reference read support in Normal	Alternative read support in Normal	Total reads in Tumor	Reference read support in Tumor	Alternate read support in Tumor	Alternate allele frequency in Tumor
Chewer	IOB_32	chr6	35480041	C	T	TULP1	MISSENSE	ENSG00000112041:[ENST00000229771(c.106G>A,Gcc>Acc):ENSP00000229771(p.Ala36Thr)]	NA	NA	chr6*35480041*35480041*C*A*rs891678786*NA*NA*NA	NA	NA	NA	NA	T	BN	NA	MODERATE	NA	NA	71	70	0	62	53	8	12.9
Chewer	IOB_32	chr6	47846646	G	T	PTCHD4	MISSENSE	ENSG00000244694:[ENST00000339488(c.1934C>A,cCc>cAc):ENSP00000341914(p.Pro645His)]	NA	NA	NA	NA	NA	NA	NA	NA	NA	NA	MODERATE	NA	1	117	117	0	151	125	26	17.22
Chewer	IOB_32	chr6	79770396	C	T	PHIP	MISSENSE	ENSG00000146247:[ENST00000275034(c.329G>A,cGc>cAc):ENSP00000275034(p.Arg110His)]	NA	chr6*79770396*79770396*COSM1446061*C*T*1*large intestine*1	NA	NA	NA	NA	NA	D	PrD	NA	MODERATE	D	1	212	212	0	248	241	5	2.02
Chewer	IOB_32	chr6	110943224	C	T	CDK19	INTRONIC	23097:[NM_001300960.1(c.978+67G>A)]	NA	NA	NA	NA	NA	NA	NA	NA	NA	NA	MODIFIER	NA	NA	128	128	0	118	112	5	4.24
Chewer	IOB_32	chr6	136847001	C	T	MAP7	SILENT	ENSG00000135525:[ENST00000438100(c.102G>A,caG>caA):ENSP00000400790(p.Gln34(=))]	NA	NA	NA	NA	NA	NA	NA	NA	NA	NA	MODIFIER	NA	NA	77	77	0	167	158	5	2.99
Chewer	IOB_32	chr6	138198389	G	A	TNFAIP3	MISSENSE	ENSG00000118503:[ENST00000237289(c.982G>A,Gca>Aca):ENSP00000237289(p.Ala328Thr)]	NA	NA	NA	2.50E-05	0.00E+00	NA	NA	D	PoD	NA	MODERATE	D	1	85	85	0	120	115	5	4.17
Chewer	IOB_32	chr6	138576761	G	A	KIAA1244	MISSENSE	ENSG00000112379:[ENST00000251691(c.959G>A,cGg>cAg):ENSP00000251691(p.Arg320Gln)]	NA	NA	NA	8.29E-06	0.00E+00	NA	NA	D	PrD	NA	MODERATE	D	1	131	129	2	204	173	31	15.2
Chewer	IOB_32	chr6	149700023	C	G	TAB2	MISSENSE	ENSG00000055208:[ENST00000286332(c.972C>G,aaC>aaG):ENSP00000286332(p.Asn324Lys)]	NA	NA	NA	NA	NA	NA	NA	T	PrD	NA	MODERATE	D	1	492	489	0	452	383	68	15.04
Chewer	IOB_32	chr7	31142858	G	T	ADCYAP1R1	MISSENSE	ENSG00000078549:[ENST00000436116(c.287G>T,Gcc>Tcc):ENSP00000416622(p.Ala97Ser)]	NA	NA	NA	NA	NA	NA	NA	D	PrD	NA	MODERATE	N	1	112	111	1	143	125	17	11.89
Chewer	IOB_32	chr7	45009076	G	A	MYO1G	MISSENSE	ENSG00000136286:[ENST00000258787(c.1510C>T,Ccc>Tcc):ENSP00000258787(p.Pro504Ser)]	NA	NA	NA	NA	NA	NA	NA	NA	NA	NA	MODIFIER	NA	NA	125	125	0	213	180	33	15.49
Chewer	IOB_32	chr7	45009076	G	A	NA	NCT	101928927:[XR_242171.1(n.14+310G>A)]	NA	NA	NA	NA	NA	NA	NA	NA	NA	NA	MODIFIER	NA	NA	125	125	0	213	180	33	15.49
Chewer	IOB_32	chr7	80117902	A	C	CD36	INTRONIC	ENSG00000135218:[ENST00000435819(c.-261+805A>C)]	NA	NA	NA	1.62E-05	9.62E-05	NA	NA	T	PrD	NA	MODERATE	U	0.837	212	212	0	247	194	51	20.65
Chewer	IOB_32	chr7	80117902	A	C	GNAT3	MISSENSE	ENSG00000214415:[ENST00000398291(c.252T>G,atT>atG):ENSP00000381339(p.Ile84Met)]	NA	NA	NA	1.62E-05	9.62E-05	NA	NA	T	PrD	NA	MODERATE	U	0.837	212	212	0	247	194	51	20.65
Chewer	IOB_32	chr7	98559010	G	A	TRRAP	MISSENSE	ENSG00000196367:[ENST00000456197(c.5760G>A,Gcc>Acc):ENSP00000394645(p.Ala1921Thr)]	NA	NA	NA	8.24E-06	0.00E+00	NA	NA	T	BN	NA	MODERATE	D	1	139	139	0	168	137	30	17.86
Chewer	IOB_32	chr7	100361656	C	T	ZAN	SILENT	ENSG00000146839:[ENST00000348028(c.4104C>T,tgC>tgT):ENSP00000423579(p.Cys1368(=))]	NA	NA	NA	NA	NA	NA	NA	NA	NA	NA	MODIFIER	NA	NA	229	229	0	253	246	6	2.37
Chewer	IOB_32	chr7	124404272	C	T	GPR37	SILENT	ENSG00000170775:[ENST00000303921(c.759G>A,ccG>ccA):ENSP00000306449(p.Pro253(=))]	NA	NA	NA	NA	NA	NA	NA	NA	NA	NA	LOW	NA	NA	188	187	0	216	189	26	12.04
Chewer	IOB_32	chr7	142499789	A	T	TRBC2	SILENT-SS-PRX	ENSG00000211772:[ENST00000466254(c.406A>T,gAg>gTg):ENSP00000417300(p.Glu136Val)]	NA	NA	NA	1.69E-05	0.00E+00	NA	NA	NA	NA	NA	LOW	NA	NA	101	92	9	125	94	31	24.8
Chewer	IOB_32	chr7	142499793	T	C	TRBC2	SILENT	ENSG00000211772:[ENST00000466254(c.410T>C,tcT>tcC):ENSP00000417300(p.Ser137(=))]	NA	NA	NA	1.69E-05	0.00E+00	NA	NA	NA	NA	NA	LOW	NA	NA	101	91	10	129	97	32	24.81
Chewer	IOB_32	chr7	142499836	T	C	TRBC2	MISSENSE	28638:[NC_000007.13:TRBC2:u_t_1(c.453T>C,cTt>cCt):NC_000007.13:TRBC2:u_t_1(p.Leu152Pro)]	NA	NA	NA	1.64E-02	8.05E-03	NA	NA	NA	NA	NA	LOW	NA	NA	101	91	10	115	84	31	26.96
Chewer	IOB_32	chr7	154002604	G	A	DPP6	MISSENSE	ENSG00000130226:[ENST00000332007(c.25G>A,Gct>Act):ENSP00000328226(p.Ala9Thr)]	NA	NA	NA	NA	NA	NA	NA	T	BN	NA	MODERATE	NA	NA	152	152	0	196	153	42	21.43
Chewer	IOB_32	chr8	3253901	A	T	CSMD1	MISSENSE-SS-PRX	ENSG00000183117:[ENST00000537824(c.2408T>A,tTt>tAt):ENSP00000441462(p.Phe803Tyr)]	NA	NA	NA	NA	NA	NA	NA	D	PrD	NA	MODERATE	D	1	28	28	0	31	23	7	22.58
Chewer	IOB_32	chr8	21608199	C	T	GFRA2	MISSENSE	ENSG00000168546:[ENST00000518077(c.296G>A,cGc>cAc):ENSP00000429206(p.Arg99His)]	NA	NA	NA	8.37E-06	0.00E+00	NA	NA	NA	NA	NA	MODERATE	NA	1	134	133	0	123	96	25	20.33
Chewer	IOB_32	chr8	90926278	C	T	OSGIN2	INTRONIC	ENSG00000164823:[ENST00000451899(c.200-27C>T)]	NA	NA	chr8*90926277*90926278*AC*A*rs960945647*NA*NA*NA	NA	NA	NA	NA	NA	NA	NA	MODIFIER	NA	NA	91	90	0	124	105	19	15.32
Chewer	IOB_32	chr8	95419794	C	T	FSBP	3UTR	ENSG00000265817:[ENST00000517506(c.*334G>A)]	NA	NA	2.40E-03	7.83E-04	0.00E+00	2.40E-03	0.00E+00	NA	NA	NA	MODIFIER	NA	NA	195	195	0	288	281	6	2.08
Chewer	IOB_32	chr8	95419794	C	T	RAD54B	SILENT	ENSG00000197275:[ENST00000336148(c.654G>A,tcG>tcA):ENSP00000336606(p.Ser218(=))]	NA	NA	2.40E-03	7.83E-04	0.00E+00	2.40E-03	0.00E+00	NA	NA	NA	MODIFIER	NA	NA	195	195	0	288	281	6	2.08
Chewer	IOB_32	chr8	97399292	C	G	KB-1448A5.1	NCT	ENSG00000253105:[ENST00000522767(n.335+40G>C)]	NA	NA	NA	NA	NA	NA	NA	NA	NA	NA	MODIFIER	NA	NA	92	92	0	136	116	19	13.97
Chewer	IOB_32	chr8	132991119	C	T	EFR3A	MISSENSE	ENSG00000132294:[ENST00000254624(c.1352C>T,aCg>aTg):ENSP00000254624(p.Thr451Met)]	NA	NA	NA	9.96E-05	6.19E-05	NA	NA	D	PoD	NA	MODERATE	D	0.992	106	106	0	139	125	13	9.35
Chewer	IOB_32	chr8	144993019	A	G	PLEC	MISSENSE	ENSG00000178209:[ENST00000527096(c.11039T>C,aTc>aCc):ENSP00000434583(p.Ile3680Thr)]	NA	NA	NA	NA	NA	NA	NA	NA	NA	NA	MODERATE	NA	1	281	280	0	438	375	60	13.7
Chewer	IOB_32	chr8	145007412	C	T	PLEC	SILENT	ENSG00000178209:[ENST00000436759(c.1452G>A,caG>caA):ENSP00000388180(p.Gln484(=))]	NA	NA	NA	NA	NA	NA	NA	NA	NA	NA	LOW	NA	0.982	150	149	0	204	170	33	16.18
Chewer	IOB_32	chr8	145138640	C	T	GPAA1	MISSENSE	ENSG00000197858:[ENST00000530633(c.134C>T,aCg>aTg):ENSP00000431233(p.Thr45Met)]	NA	NA	chr8*145138640*145138640*C*G*rs368478276*NA*NA*NA	NA	NA	NA	NA	NA	NA	NA	LOW	NA	0	128	128	0	212	153	57	26.89
Chewer	IOB_32	chr9	71538119	G	C	PIP5K1B	INTRONIC	ENSG00000107242:[ENST00000265382(c.1117-99G>C)]	NA	NA	NA	NA	NA	NA	NA	NA	NA	NA	MODIFIER	NA	NA	82	82	0	114	103	11	9.65
Chewer	IOB_32	chr9	114090649	C	A	OR2K2	MISSENSE	ENSG00000171133:[ENST00000302681(c.65G>T,gGg>gTg):ENSP00000305055(p.Gly22Val)]	NA	NA	NA	NA	NA	NA	NA	T	BN	NA	MODERATE	U	0	154	154	0	194	158	36	18.56
Chewer	IOB_32	chr9	134371208	C	T	PRRC2B	NONSENSE	ENSG00000130723:[ENST00000405995(c.4555C>T,Cga>Tga):ENSP00000384606(p.Arg1519Ter)]	NA	NA	NA	1.67E-05	0.00E+00	NA	NA	NA	NA	NA	MODIFIER	NA	NA	106	106	0	180	174	6	3.33
Chewer	IOB_32	chr9	139399932	G	A	NOTCH1	SILENT	ENSG00000148400:[ENST00000277541(c.4416C>T,tgC>tgT):ENSP00000277541(p.Cys1472(=))]	NA	NA	NA	8.50E-06	0.00E+00	NA	NA	NA	NA	NA	LOW	NA	1	238	236	0	297	253	42	14.14
Chewer	IOB_32	chrX	9716535	G	C	GPR143	INTRONIC	ENSG00000101850:[ENST00000431126(c.296+79C>G)]	NA	NA	NA	NA	NA	NA	NA	NA	NA	NA	MODIFIER	NA	NA	100	100	0	96	82	14	14.58
Chewer	IOB_32	chrX	21675548	C	T	KLHL34	MISSENSE	ENSG00000185915:[ENST00000379499(c.359G>A,cGc>cAc):ENSP00000368813(p.Arg120His)]	NA	NA	NA	NA	NA	NA	NA	T	BN	NA	MODERATE	N	1	146	145	0	205	169	36	17.56
Chewer	IOB_32	chrX	49079511	G	A	CACNA1F	SILENT	ENSG00000102001:[ENST00000376251(c.1800C>T,ctC>ctT):ENSP00000365427(p.Leu600(=))]	NA	NA	NA	NA	NA	NA	NA	NA	NA	NA	LOW	NA	NA	49	49	0	72	58	14	19.44
Chewer	IOB_32	chrX	49099474	C	T	CCDC22	NCT	ENSG00000101997:[ENST00000496651(n.559+16C>T)]	NA	NA	NA	NA	NA	NA	NA	NA	NA	NA	MODIFIER	NA	NA	411	409	0	455	398	54	11.87
Chewer	IOB_32	chrX	54949825	A	T	TRO	MISSENSE	ENSG00000067445:[ENST00000173898(c.860A>T,cAg>cTg):ENSP00000173898(p.Gln287Leu)]	NA	NA	NA	NA	NA	NA	NA	NA	NA	NA	MODIFIER	NA	NA	55	55	0	52	44	8	15.38
Chewer	IOB_32	chrX	57618870	G	A	ZXDB	MISSENSE	ENSG00000198455:[ENST00000374888(c.389G>A,gGc>gAc):ENSP00000364023(p.Gly130Asp)]	NA	NA	NA	2.28E-01	1.86E-01	NA	NA	T_lc	BN	NA	MODERATE	N	0	28	26	2	19	12	7	36.84
Chewer	IOB_32	chrX	64936454	C	A	MSN	INTRONIC	ENSG00000147065:[ENST00000360270(c.13-226C>A)]	NA	NA	chrX*64936454*64936454*C*T*rs952877928*NA*NA*NA	NA	NA	NA	NA	NA	NA	NA	MODIFIER	NA	NA	197	195	0	233	225	5	2.15
Chewer	IOB_32	chrX	70519801	A	C	NONO	MISSENSE	ENSG00000147140:[ENST00000535149(c.1024A>C,Aca>Cca):ENSP00000441364(p.Thr342Pro)]	NA	NA	NA	NA	NA	NA	NA	NA	NA	NA	MODIFIER	NA	NA	96	96	0	85	68	17	20
Chewer	IOB_32	chrX	118603818	T	G	SLC25A5	SILENT	ENSG00000005022:[ENST00000317881(c.306T>G,ggT>ggG):ENSP00000360671(p.Gly102(=))]	NA	chrX*118603818*118603818*COSM150817*T*G*1*stomach*1	NA	6.20E-02	4.62E-02	NA	NA	NA	NA	NA	LOW	NA	NA	72	72	0	85	76	9	10.59
Chewer	IOB_32	chrX	128650287	G	C	SMARCA1	INTRONIC	ENSG00000102038:[ENST00000371121(c.428+21C>G)]	NA	NA	NA	NA	NA	NA	NA	NA	NA	NA	MODIFIER	NA	NA	54	54	0	57	48	9	15.79
Chewer	IOB_32	chrX	140985395	A	T	MAGEC3	MISSENSE	ENSG00000165509:[ENST00000409007(c.957A>T,gaA>gaT):ENSP00000386566(p.Glu319Asp)]	NA	NA	NA	NA	NA	NA	NA	NA	NA	NA	MODIFIER	NA	NA	114	114	0	122	112	10	8.2
Chewer	IOB_32	chrX	152751258	C	T	HAUS7	INTRONIC	ENSG00000213397:[ENST00000370210(c.-589+9574G>A)]	NA	NA	NA	NA	NA	NA	NA	NA	NA	NA	MODIFIER	NA	NA	101	101	0	122	116	5	4.1
Chewer	IOB_32	chrX	152751258	C	T	LOC100509091	SILENT	100509091:[XM_003960028.1(c.1212C>T,aaC>aaT):XP_003960077.1(p.Asn404(=))]	NA	NA	NA	NA	NA	NA	NA	NA	NA	NA	MODIFIER	NA	NA	101	101	0	122	116	5	4.1
Chewer	IOB_33	chr1	11301664	C	A	MTOR	MISSENSE	ENSG00000198793:[ENST00000361445(c.1487G>T,gGc>gTc):ENSP00000354558(p.Gly496Val)]	NA	NA	NA	NA	NA	NA	NA	T	BN	NA	MODERATE	D	1	115	115	0	183	177	6	3.28
Chewer	IOB_33	chr1	20674907	C	A	VWA5B1	INTRONIC	ENSG00000158816:[ENST00000375079(c.2893+38C>A)]	NA	NA	NA	NA	NA	NA	NA	NA	NA	NA	MODIFIER	NA	NA	154	152	1	165	149	16	9.7
Chewer	IOB_33	chr1	25599204	A	C	C1orf63	INTRONIC	ENSG00000117616:[ENST00000473314(c.-66-25684T>G)]	NA	NA	NA	1.76E-04	1.95E-04	NA	NA	NA	NA	NA	MODIFIER	NA	NA	85	83	2	53	42	11	20.75
Chewer	IOB_33	chr1	25599204	A	C	RHD	INTRONIC	6007:[NM_001282867.1(c.-347+18A>C)]	NA	NA	NA	1.76E-04	1.95E-04	NA	NA	NA	NA	NA	MODIFIER	NA	NA	85	83	2	53	42	11	20.75
Chewer	IOB_33	chr1	25599204	A	C	RSRP1	NCT	57035:[NR_135788.1(n.278-25684T>G)]	NA	NA	NA	1.76E-04	1.95E-04	NA	NA	NA	NA	NA	MODIFIER	NA	NA	85	83	2	53	42	11	20.75
Chewer	IOB_33	chr1	32208558	C	T	ADGRB2	MISSENSE	576:[XM_005271063.1(c.1235G>A,cGc>cAc):XP_005271120.1(p.Arg412His)]	NA	NA	NA	NA	NA	NA	NA	T	PoD	NA	MODERATE	N	0.469	18	18	0	48	21	27	56.25
Chewer	IOB_33	chr1	32208558	C	T	BAI2	MISSENSE	ENSG00000121753:[ENST00000257070(c.1133G>A,cGc>cAc):ENSP00000257070(p.Arg378His)]	NA	NA	NA	NA	NA	NA	NA	T	PoD	NA	MODERATE	N	0.469	18	18	0	48	21	27	56.25
Chewer	IOB_33	chr1	46543279	C	A	PIK3R3	MISSENSE	ENSG00000117461:[ENST00000540385(c.360G>T,gaG>gaT):ENSP00000439913(p.Glu120Asp)]	NA	NA	NA	NA	NA	NA	NA	NA	NA	NA	MODIFIER	NA	NA	203	202	1	196	189	6	3.06
Chewer	IOB_33	chr1	52520724	G	A	TXNDC12	SILENT	ENSG00000117862:[ENST00000371626(c.45C>T,ttC>ttT):ENSP00000360688(p.Phe15(=))]	NA	NA	NA	8.28E-06	0.00E+00	NA	NA	NA	NA	NA	LOW	NA	0.997	185	185	0	162	156	6	3.7
Chewer	IOB_33	chr1	109143216	T	C	FAM102B	MISSENSE	ENSG00000162636:[ENST00000370035(c.166T>C,Tgg>Cgg):ENSP00000359052(p.Trp56Arg)]	NA	NA	NA	NA	NA	NA	NA	D	PrD	NA	MODERATE	D	1	119	119	0	107	100	6	5.61
Chewer	IOB_33	chr1	116933435	G	A	ATP1A1	NONSENSE	ENSG00000163399:[ENST00000295598(c.1254G>A,tgG>tgA):ENSP00000295598(p.Trp418Ter)]	NA	NA	NA	NA	NA	NA	NA	NA	NA	NA	HIGH	NA	1	198	198	0	263	257	6	2.28
Chewer	IOB_33	chr1	144014027	G	A	SRGAP2B	MISSENSE	647135:[NM_001271870.1(c.194G>A,cGc>cAc):NP_001258799.1(p.Arg65His)]	NA	NA	NA	5.45E-05	0.00E+00	NA	NA	NA	NA	NA	MODERATE	NA	NA	187	187	0	260	253	6	2.31
Chewer	IOB_33	chr1	145539719	C	T	ITGA10	MISSENSE	ENSG00000143127:[ENST00000538811(c.2758C>T,Cac>Tac):ENSP00000440011(p.His920Tyr)]	NA	NA	NA	8.37E-06	0.00E+00	NA	NA	NA	NA	NA	MODERATE	NA	NA	63	63	0	109	95	14	12.84
Chewer	IOB_33	chr1	152130432	G	A	RPTN	INTRONIC	ENSG00000215853:[ENST00000316073(c.-20-47C>T)]	NA	NA	NA	NA	NA	NA	NA	NA	NA	NA	MODIFIER	NA	NA	145	144	1	256	247	8	3.12
Chewer	IOB_33	chr1	160064985	C	T	IGSF8	MISSENSE	ENSG00000162729:[ENST00000314485(c.116G>A,cGc>cAc):ENSP00000316664(p.Arg39His)]	NA	chr1*160064985*160064985*COSM311952*C*A*2*lung*2	2.00E-04	2.52E-05	0.00E+00	2.00E-04	0.00E+00	NA	NA	NA	MODIFIER	NA	NA	136	136	0	163	141	21	12.88
Chewer	IOB_33	chr1	161475747	G	T	FCGR2A	NCT	ENSG00000143226:[ENST00000497474(n.95-30G>T)]	NA	NA	chr1*161475747*161475747*G*A*rs763905176*NA*NA*NA	NA	NA	NA	NA	NA	NA	NA	MODIFIER	NA	NA	93	93	0	175	110	65	37.14
Chewer	IOB_33	chr1	162367150	C	T	SH2D1B	INTRONIC	ENSG00000198574:[ENST00000359567(c.199-45G>A)]	NA	NA	NA	1.65E-05	0.00E+00	NA	NA	NA	NA	NA	MODIFIER	NA	NA	173	172	0	190	164	26	13.68
Chewer	IOB_33	chr1	183520392	A	T	SMG7	INTRONIC	ENSG00000116698:[ENST00000515829(c.3162+67A>T)]	NA	NA	NA	NA	NA	NA	NA	NA	NA	NA	MODIFIER	NA	NA	86	86	0	148	122	26	17.57
Chewer	IOB_33	chr1	183529388	A	G	NCF2	SILENT	ENSG00000116701:[ENST00000367535(c.1311T>C,gaT>gaC):ENSP00000356505(p.Asp437(=))]	NA	NA	NA	NA	NA	NA	NA	NA	NA	NA	LOW	NA	0	85	85	0	87	75	12	13.79
Chewer	IOB_33	chr1	183529388	A	G	SMG7	NCT	ENSG00000116698:[ENST00000495321(n.233+9063A>G)]	NA	NA	NA	NA	NA	NA	NA	NA	NA	NA	LOW	NA	0	85	85	0	87	75	12	13.79
Chewer	IOB_33	chr1	184706771	G	A	EDEM3	SILENT	ENSG00000116406:[ENST00000318130(c.279C>T,cgC>cgT):ENSP00000318147(p.Arg93(=))]	NA	chr1*184706771*184706771*COSM900869*G*A*2*endometrium*2	NA	3.30E-05	1.21E-04	NA	NA	NA	NA	NA	LOW	NA	NA	101	101	0	155	150	5	3.23
Chewer	IOB_33	chr1	201176657	C	G	IGFN1	MISSENSE	ENSG00000163395:[ENST00000335211(c.2636C>G,tCt>tGt):ENSP00000334714(p.Ser879Cys)]	NA	NA	chr1*201176657*201176657*C*T*rs1048753843*NA*NA*NA	NA	NA	NA	NA	NA	NA	NA	MODIFIER	NA	NA	109	109	0	107	64	43	40.19

Patel *et al.*, 2021. Whole-exome sequencing analysis of oral squamous cell carcinoma delineated by tobacco usage habits
Supplementary Table 4. List of somatic single nucleotide variants identified using whole exome sequencing of OSCC patients

Cohort	Sample	Chromosome	Genomic position	Reference allele	Alternate allele	Genesymbol	Variant type	Variant description	OncoMD	COSMIC	dbSNP	ExAC	ExAC_SAS	1000G	1000G_SAS	SIFT	Polyphen2	CONDEL	VEP	LRT	PhastCon7Way	Total reads in Normal	Reference read support in Normal	Alternative read support in Normal	Total reads in Tumor	Reference read support in Tumor	Alternate read support in Tumor	Alternate allele frequency in Tumor
Chewer	IOB_33	chr1	212799906	G	T	FAM71A	MISSENSE	ENSG00000162771:[ENST00000294829(c.1687G>T,Gtg>Ttg):ENSP00000294829(p.Val563Leu)]	NA	NA	chr1*212799906*212799906*G*A*rs753608986*NA*NA*NA	NA	NA	NA	NA	NA	NA	NA	MODIFIER	NA	NA	132	132	0	174	168	6	3.45
Chewer	IOB_33	chr1	212799906	G	T	RP11-338C15.5	EXONIC-NC	ENSG00000235862:[ENST00000427949(n.208C>A)]	NA	NA	chr1*212799906*212799906*G*A*rs753608986*NA*NA*NA	NA	NA	NA	NA	NA	NA	NA	MODIFIER	NA	NA	132	132	0	174	168	6	3.45
Chewer	IOB_33	chr1	225569260	G	A	DNAH14	MISSENSE	ENSG00000185842:[ENST00000445597(c.9726G>A,atG>atA):ENSP00000409472(p.Met3242Ile)]	NA	NA	NA	NA	NA	NA	NA	T	BN	NA	MODERATE	NA	NA	33	33	0	46	38	8	17.39
Chewer	IOB_33	chr1	241675323	C	A	FH	MISSENSE	ENSG00000091483:[ENST00000366560(c.499G>T,Ggt>Tgt):ENSP00000355518(p.Gly167Cys)]	NA	NA	NA	NA	NA	NA	NA	D	PrD	NA	MODERATE	D	1	292	292	0	414	333	80	19.32
Chewer	IOB_33	chr1	246021919	G	A	SMYD3	MISSENSE	ENSG00000185420:[ENST00000388985(c.955C>T,Cgg>Tgg):ENSP00000373637(p.Arg319Trp)]	NA	NA	NA	1.65E-05	6.06E-05	NA	NA	T	BN	NA	MODERATE	NA	0.169	129	128	0	181	149	32	17.68
Chewer	IOB_33	chr1	248487511	A	G	OR2M7	SILENT	ENSG00000177186:[ENST00000317965(c.360T>C,taT>taC):ENSP00000324557(p.Tyr120(=))]	NA	NA	NA	NA	NA	NA	NA	NA	NA	NA	LOW	NA	0.005	311	310	1	569	474	94	16.52
Chewer	IOB_33	chr10	45799721	G	A	OR13A1	SILENT	ENSG00000256574:[ENST00000374401(c.150C>T,ctC>ctT):ENSP00000363522(p.Leu50(=))]	NA	NA	NA	NA	NA	NA	NA	NA	NA	NA	LOW	NA	NA	79	79	0	87	79	8	9.2
Chewer	IOB_33	chr10	45919614	G	T	ALOX5	NCT	ENSG00000012779:[ENST00000483623(n.64+19G>T)]	NA	NA	chr10*45919614*45919614*G*A*rs752896072*NA*NA*NA	NA	NA	NA	NA	NA	NA	NA	MODIFIER	NA	NA	88	87	0	147	133	12	8.16
Chewer	IOB_33	chr10	49634531	C	A	MAPK8	MISSENSE	ENSG00000107643:[ENST00000395611(c.752C>A,cCt>cAt):ENSP00000378974(p.Pro251His)]	NA	NA	NA	NA	NA	NA	NA	NA	NA	NA	MODIFIER	NA	NA	139	139	0	239	231	6	2.51
Chewer	IOB_33	chr10	72015293	C	T	NPFFR1	MISSENSE	ENSG00000148734:[ENST00000277942(c.713G>A,cGc>cAc):ENSP00000277942(p.Arg238His)]	NA	NA	NA	NA	NA	NA	NA	D	PrD	NA	MODERATE	D	1	260	259	0	313	306	6	1.92
Chewer	IOB_33	chr10	77159859	C	A	RP11-399K21.11	NCT	ENSG00000270087:[ENST00000418818(n.142+1004G>T)]	NA	NA	NA	NA	NA	NA	NA	D	PoD	NA	MODERATE	N	0.339	35	35	0	57	50	5	8.77
Chewer	IOB_33	chr10	77159859	C	A	ZNF503	MISSENSE	ENSG00000165655:[ENST00000372524(c.589G>T,Ggt>Tgt):ENSP00000361602(p.Gly197Cys)]	NA	NA	NA	NA	NA	NA	NA	D	PoD	NA	MODERATE	N	0.339	35	35	0	57	50	5	8.77
Chewer	IOB_33	chr10	103825770	G	A	HPS6	MISSENSE	ENSG00000166189:[ENST00000299238(c.539G>A,cGc>cAc):ENSP00000299238(p.Arg180His)]	NA	NA	chr10*103825770*103825770*G*T*rs549561454*0*0.9998*0.0001997	NA	NA	NA	NA	T	BN	NA	MODERATE	N	0.604	128	128	0	187	181	6	3.21
Chewer	IOB_33	chr10	115381684	A	T	NRAP	MISSENSE	ENSG00000197893:[ENST00000359988(c.2713T>A,Ttg>Atg):ENSP00000353078(p.Leu905Met)]	NA	NA	NA	NA	NA	NA	NA	NA	NA	NA	MODERATE	NA	0.019	86	86	0	173	137	36	20.81
Chewer	IOB_33	chr10	118630730	C	T	ENO4	MISSENSE	ENSG00000188316:[ENST00000409522(c.317C>T,gCc>gTc):ENSP00000387194(p.Ala106Val)]	NA	NA	NA	NA	NA	NA	NA	T	BN	NA	MODERATE	N	0	139	138	0	208	202	6	2.88
Chewer	IOB_33	chr10	131640392	C	T	EBF3	MISSENSE	ENSG00000108001:[ENST00000368648(c.1333G>A,Gcc>Acc):ENSP00000357637(p.Ala445Thr)]	NA	NA	NA	NA	NA	NA	NA	T_lc	BN	NA	MODERATE	D	1	108	107	0	153	135	18	11.76
Chewer	IOB_33	chr11	1260114	A	G	MUC5B	MISSENSE	ENSG00000117983:[ENST00000447027(c.3320A>G,aAg>aGg):ENSP00000415793(p.Lys1107Arg)]	NA	NA	NA	NA	NA	NA	NA	T	BN	NA	MODERATE	NA	NA	103	103	0	147	141	5	3.4
Chewer	IOB_33	chr11	11678184	C	A	NA	INTERGENIC	NA	NA	NA	NA	NA	NA	NA	NA	NA	NA	NA	MODIFIER	NA	NA	44	44	0	81	75	6	7.41
Chewer	IOB_33	chr11	12277194	C	T	MICAL2	MISSENSE	ENSG00000133816:[ENST00000256194(c.2852C>T,aCg>aTg):ENSP00000256194(p.Thr951Met)]	NA	NA	NA	5.97E-05	1.26E-04	NA	NA	NA	NA	NA	MODIFIER	NA	NA	124	123	1	163	126	37	22.7
Chewer	IOB_33	chr11	47359326	C	A	MYBPC3	SILENT	ENSG00000134571:[ENST00000399249(c.2328G>T,gcG>gcT):ENSP00000382193(p.Ala776(=))]	NA	NA	NA	NA	NA	NA	NA	NA	NA	NA	LOW	NA	NA	154	154	0	354	346	5	1.41
Chewer	IOB_33	chr11	55432954	C	A	OR4C6	MISSENSE	ENSG00000181903:[ENST00000314259(c.312C>A,ttC>ttA):ENSP00000324769(p.Phe104Leu)]	NA	chr11*55432954*55432954*COSM1508591*C*A*1*lung*1	NA	NA	NA	NA	NA	T_lc	BN	NA	MODERATE	N	0	151	150	1	257	223	31	12.06
Chewer	IOB_33	chr11	58958521	G	T	DTX4	INTRONIC	ENSG00000110042:[ENST00000227451(c.1160-66G>T)]	NA	NA	NA	NA	NA	NA	NA	NA	NA	NA	MODIFIER	NA	NA	43	43	0	44	27	17	38.64
Chewer	IOB_33	chr11	71809978	C	A	LAMTOR1	INTRONIC	ENSG00000149357:[ENST00000278671(c.189-74G>T)]	NA	NA	NA	NA	NA	NA	NA	NA	NA	NA	MODIFIER	NA	NA	75	75	0	108	102	6	5.56
Chewer	IOB_33	chr11	71809978	C	A	LRTOMT	INTRONIC	ENSG00000184154:[ENST00000307198(c.34+3836C>A)]	NA	NA	NA	NA	NA	NA	NA	NA	NA	NA	MODIFIER	NA	NA	75	75	0	108	102	6	5.56
Chewer	IOB_33	chr11	95568380	G	C	MTMR2	3UTR	ENSG00000087053:[ENST00000346299(c.*74C>G)]	NA	NA	chr11*95568380*95568380*G*A*rs570725885*0*0.9998*0.0001997	NA	NA	NA	NA	NA	NA	NA	MODIFIER	NA	NA	50	50	0	59	43	16	27.12
Chewer	IOB_33	chr11	101786222	C	A	ANGPTL5	INTRONIC	ENSG00000187151:[ENST00000334289(c.-93+528G>T)]	NA	NA	NA	NA	NA	NA	NA	NA	NA	NA	MODIFIER	NA	NA	79	79	0	125	120	5	4
Chewer	IOB_33	chr11	101786222	C	A	KIAA1377	INTRONIC	ENSG00000110318:[ENST00000263468(c.128+79C>A)]	NA	NA	NA	NA	NA	NA	NA	NA	NA	NA	MODIFIER	NA	NA	79	79	0	125	120	5	4
Chewer	IOB_33	chr11	103158232	C	G	DYNC2H1	INTRONIC	ENSG00000187240:[ENST00000334267(c.2206-148439C>G)]	NA	NA	NA	1.52E-05	0.00E+00	NA	NA	NA	NA	NA	MODIFIER	NA	NA	65	64	0	80	63	17	21.25
Chewer	IOB_33	chr11	121000568	C	T	TECTA	SILENT	ENSG00000109927:[ENST00000264037(c.2589C>T,ccC>ccT):ENSP00000264037(p.Pro863(=))]	NA	NA	NA	NA	NA	NA	NA	NA	NA	NA	LOW	NA	NA	230	230	0	337	225	112	33.23
Chewer	IOB_33	chr12	1995182	G	A	CACNA2D4	SILENT	ENSG00000151062:[ENST00000585708(c.825C>T,atC>atT):ENSP00000467697(p.Ile275(=))]	NA	NA	2.00E-04	8.47E-06	0.00E+00	2.00E-04	0.00E+00	NA	NA	NA	LOW	NA	0.096	73	73	0	136	95	41	30.15
Chewer	IOB_33	chr12	4716542	A	G	DYRK4	MISSENSE	ENSG00000010219:[ENST00000010132(c.1004A>G,cAg>cGg):ENSP00000010132(p.Gln335Arg)]	NA	NA	NA	NA	NA	NA	NA	T	BN	NA	MODERATE	NA	NA	92	92	0	140	119	21	15
Chewer	IOB_33	chr12	4716542	A	G	RP11-500M8.7	INTRONIC	ENSG00000272921:[ENST00000536588(c.143+2290A>G)]	NA	NA	NA	NA	NA	NA	NA	T	BN	NA	MODERATE	NA	NA	92	92	0	140	119	21	15
Chewer	IOB_33	chr12	7805403	C	T	APOBEC1	MISSENSE	ENSG00000111701:[ENST00000229304(c.73G>A,Gtc>Atc):ENSP00000229304(p.Val25Ile)]	NA	chr12*7805403*7805403*COSM3399118*C*T*1*central_nervous_system*1	2.00E-04	1.13E-04	0.00E+00	2.00E-04	0.00E+00	NA	NA	NA	LOW	NA	NA	79	79	0	140	108	31	22.14
Chewer	IOB_33	chr12	42538320	G	A	GXYLT1	SILENT	ENSG00000151233:[ENST00000280876(c.129C>T,gcC>gcT):ENSP00000280876(p.Ala43(=))]	NA	NA	NA	5.68E-04	1.60E-04	NA	NA	NA	NA	NA	LOW	NA	NA	29	29	0	45	39	6	13.33
Chewer	IOB_33	chr12	49953113	G	A	MCRS1	INTRONIC	ENSG00000187778:[ENST00000551598(c.214-29C>T)]	NA	NA	NA	NA	NA	NA	NA	NA	NA	NA	MODIFIER	NA	NA	152	152	0	279	183	95	34.05
Chewer	IOB_33	chr12	53680290	G	A	ESPL1	MISSENSE	ENSG00000135476:[ENST00000257934(c.3770G>A,cGg>cAg):ENSP00000257934(p.Arg1257Gln)]	NA	NA	NA	NA	NA	NA	NA	NA	NA	NA	MODIFIER	NA	NA	113	113	0	170	165	5	2.94
Chewer	IOB_33	chr12	55759253	G	A	OR6C75	MISSENSE	ENSG00000187857:[ENST00000343399(c.359G>A,cGc>cAc):ENSP00000368987(p.Arg120His)]	NA	NA	NA	1.65E-05	0.00E+00	NA	NA	D_lc	BN	NA	MODERATE	U	0.962	178	177	0	258	252	5	1.94
Chewer	IOB_33	chr12	56713126	C	T	PAN2	MISSENSE	ENSG00000135473:[ENST00000425394(c.3248G>A,gGc>gAc):ENSP00000401721(p.Gly1083Asp)]	NA	chr12*56713126*56713126*COSM941632*C*T*1*endometrium*1	NA	NA	NA	NA	NA	NA	NA	NA	MODERATE	NA	1	126	126	0	221	214	7	3.17
Chewer	IOB_33	chr12	57850403	C	A	INHBE	NONSENSE	ENSG00000139269:[ENST00000547970(c.660C>A,taC>taA):ENSP00000450212(p.Tyr220Ter)]	NA	NA	NA	NA	NA	NA	NA	NA	NA	NA	MODIFIER	NA	NA	149	149	0	256	250	6	2.34
Chewer	IOB_33	chr12	103699881	C	T	C12orf42	MISSENSE	ENSG00000179088:[ENST00000548048(c.301G>A,Gaa>Aaa):ENSP00000449362(p.Glu101Lys)]	NA	NA	NA	NA	NA	NA	NA	NA	NA	NA	MODIFIER	NA	NA	105	105	0	189	161	25	13.23
Chewer	IOB_33	chr12	105538491	G	T	KIAA1033	NCT-SS-PRX	23325:[XR_245916.1(n.2270-5G>T)]	NA	NA	NA	NA	NA	NA	NA	NA	NA	NA	MODIFIER	NA	NA	105	102	2	181	164	11	6.08
Chewer	IOB_33	chr12	105538491	G	T	WASHC4	INTRONIC	23325:[NM_001293640.1(c.2183-5G>T)]	NA	NA	NA	NA	NA	NA	NA	NA	NA	NA	MODIFIER	NA	NA	105	102	2	181	164	11	6.08
Chewer	IOB_33	chr12	124798859	C	T	FAM101A	MISSENSE	ENSG00000178882:[ENST00000389727(c.439C>T,Cgc>Tgc):ENSP00000374377(p.Arg147Cys)]	NA	NA	NA	8.28E-06	0.00E+00	NA	NA	D	PrD	NA	MODERATE	D	1	279	279	0	464	320	143	30.82
Chewer	IOB_33	chr12	131692210	C	T	RP11-638F5.1	NCT	ENSG00000204603:[ENST00000376678(n.373-10C>T)]	NA	NA	2.00E-04	NA	NA	2.00E-04	1.00E-03	NA	NA	NA	MODIFIER	NA	NA	185	185	0	337	277	59	17.51
Chewer	IOB_33	chr12	132626869	G	T	DDX51	MISSENSE	ENSG00000185163:[ENST00000397333(c.769C>A,Cca>Aca):ENSP00000380495(p.Pro257Thr)]	NA	NA	chr12*132626869*132626869*G*C*rs755089192*NA*NA*NA	NA	NA	NA	NA	NA	NA	NA	MODIFIER	NA	NA	133	133	0	206	177	27	13.11
Chewer	IOB_33	chr12	133810859	C	A	ANHX	SILENT	ENSG00000227059:[ENST00000419717(c.84G>T,ctG>ctT):ENSP00000409950(p.Leu28(=))]	NA	NA	NA	NA	NA	NA	NA	NA	NA	NA	LOW	NA	NA	140	140	0	221	156	64	28.96
Chewer	IOB_33	chr13	110823056	G	A	COL4A1	MISSENSE	ENSG00000187498:[ENST00000375820(c.3580C>T,Cca>Tca):ENSP00000364979(p.Pro1194Ser)]	NA	NA	NA	NA	NA	NA	NA	NA	PrD	NA	MODERATE	D	1	57	55	1	79	66	13	16.46
Chewer	IOB_33	chr14	20813493	G	T	PARP2	INTRONIC	ENSG00000129484:[ENST00000429687(c.203-60G>T)]	NA	NA	NA	NA	NA	NA	NA	NA	NA	NA	MODIFIER	NA	NA	45	45	0	90	85	5	5.56
Chewer	IOB_33	chr14	20813493	G	T	RP11-203M5.2	NCT	ENSG00000254846:[ENST00000528210(n.325-958C>A)]	NA	NA	NA	NA	NA	NA	NA	NA	NA	NA	MODIFIER	NA	NA	45	45	0	90	85	5	5.56
Chewer	IOB_33	chr14	55326469	C	T	GCH1	NCT	ENSG00000131979:[ENST00000254299(n.602-15G>A)]	NA	NA	2.00E-04	6.65E-05	4.89E-04	2.00E-04	1.00E-03	NA	NA	NA	MODIFIER	NA	NA	95	95	0	97	83	14	14.43
Chewer	IOB_33	chr14	65009186	C	T	HSPA2	MISSENSE	ENSG00000126803:[ENST00000247207(c.1619C>T,gCg>gTg):ENSP00000247207(p.Ala540Val)]	NA	NA	NA	NA	NA	NA	NA	T_lc	BN	NA	MODERATE	U	0.991	203	201	0	296	288	5	1.69
Chewer	IOB_33	chr14	68029294	C	T	PLEKHH1	MISSENSE	ENSG00000054690:[ENST00000329153(c.946C>T,Cgg>Tgg):ENSP00000330278(p.Arg316Trp)]	NA	NA	NA	3.48E-05	1.27E-04	NA	NA	D	PrD	NA	MODERATE	N	0.943	110	110	0	173	167	5	2.89
Chewer	IOB_33	chr14	74959965	C	T	NPC2	MISSENSE	ENSG00000119655:[ENST00000238633(c.13G>A,Gca>Aca):ENSP00000238633(p.Ala5Thr)]	NA	NA	NA	NA	NA	NA	NA	T	BN	NA	MODERATE	N	0	163	163	0	170	141	29	17.06
Chewer	IOB_33	chr14	96137725	T	C	RP11-1070N10.6	EXONIC-NC	ENSG00000259084:[ENST00000495847(n.2470T>C)]	NA	NA	NA	NA	NA	NA	NA	NA	NA	NA	MODIFIER	NA	NA	71	71	0	98	92	6	6.12
Chewer	IOB_33	chr14	96137725	T	C	TCL6	EXONIC-NC	ENSG00000187621:[ENST00000465501(n.328T>C)]	NA	NA	NA	NA	NA	NA	NA	NA	NA	NA	MODIFIER	NA	NA	71	71	0	98	92	6	6.12
Chewer	IOB_33	chr14	101349021	G	T	RTL1	MISSENSE	ENSG00000254656:[ENST00000534062(c.2105C>A,cCg>cAg):ENSP00000435342(p.Pro702Gln)]	NA	NA	chr14*101349021*101349021*G*A*rs769769599*NA*NA*NA	NA	NA	NA	NA	D	PrD	NA	MODERATE	N	1	146	145	0	331	280	51	15.41
Chewer	IOB_33	chr14	102484827	G	A	DYNC1H1	SILENT	ENSG00000197102:[ENST00000360184(c.8217G>A,ccG>ccA):ENSP00000348965(p.Pro2739(=))]	NA	NA	NA	8.28E-06	0.00E+00	NA	NA	NA	NA	NA	LOW	NA	NA	130	129	0	273	212	61	22.34
Chewer	IOB_33	chr14	104174896	C	T	XRCC3	SILENT	ENSG00000126215:[ENST00000352127(c.156G>A,acG>acA):ENSP00000343392(p.Thr52(=))]	NA	NA	2.00E-04	5.80E-05	6.06E-05	2.00E-04	0.00E+00	NA	NA	NA	LOW	NA	NA	65	64	0	123	116	6	4.88
Chewer	IOB_33	chr14	105618348	C	T	JAG2	SILENT	ENSG00000184916:[ENST00000331782(c.966G>A,acG>acA):ENSP00000328169(p.Thr322(=))]	NA	NA	2.00E-04	8.32E-06	0.00E+00	2.00E-04	0.00E+00	NA	NA	NA	MODIFIER	NA	NA	93	87	6	95	61	34	35.79
Chewer	IOB_33	chr14	105996004	C	T	TMEM121	MISSENSE	ENSG00000184986:[ENST00000392519(c.833C>T,gCg>gTg):ENSP00000376304(p.Ala278Val)]	NA	NA	NA	NA	NA	NA	NA	T_lc	BN	NA	MODERATE	U	0.999	252	252	0	427	366	61	14.29
Chewer	IOB_33	chr15	20453913	C	T	RP11-492D6.3	EXONIC-NC-SS-PRX	ENSG00000258628:[ENST00000557586(n.1684G>A)]	NA	NA	NA	NA	NA	NA	NA	NA	NA	NA	LOW	NA	NA	65	62	3	155	119	35	22.58
Chewer	IOB_33	chr15	26874111	C	A	GABRB3	INTRONIC	ENSG00000166206:[ENST00000299267(c.241-7430G>T)]	NA	NA	NA	NA	NA	NA	NA	NA	NA	NA	MODIFIER	NA	NA	72	72	0	90	71	18	20
Chewer	IOB_33	chr15	40917934	G	T	CASC5	INTRONIC	ENSG00000137812:[ENST00000534204(c.116-3588G>T)]	NA	NA	chr15*40917933*40917934*CG*C*rs1036352540*NA*NA*NA|chr15*40917934*40917934*G*A*rs775188695*NA*NA*NA	NA	NA	NA	NA	NA	NA	NA	MODIFIER	NA	NA	24	24	0	31	12	18	58.06
Chewer	IOB_33	chr15	42974536	A	G	STARD9	MISSENSE	ENSG00000159433:[ENST00000290607(c.2300A>G,aAg>aGg):ENSP00000290607(p.Lys767Arg)]	NA	NA	NA	NA	NA	NA	NA	T	BN	NA	MODERATE	NA	NA	79	78	0	106	79	27	25.47
Chewer	IOB_33	chr15	43924420	C	T	CATSPER2	MISSENSE	ENSG00000166762:[ENST00000450810(c.163G>A,cGt>cAt):ENSP00000388242(p.Arg55His)]	NA	NA	NA	5.25E-02	6.64E-02	NA	NA	NA	NA	NA	MODIFIER	NA	NA	91	87	4	118	90	28	23.73
Chewer	IOB_33	chr15	43924420	C	T	STRC	INTRONIC	161497:[XM_005254193.1(c.-48-13452G>A)]	NA	NA	NA	5.25E-02	6.64E-02	NA	NA	NA	NA	NA	MODIFIER	NA	NA	91	87	4	118	90	28	23.73
Chewer	IOB_33	chr15	43924422	T	C	CATSPER2	SILENT	ENSG00000166762:[ENST00000354127(c.1530A>G,gaA>gaG):ENSP00000339137(p.Glu510(=))]	NA	NA	NA	5.38E-02	6.71E-02	NA	NA	NA	NA	NA	LOW	NA	0.996	91	87	4	122	92	29	23.77
Chewer	IOB_33	chr15	43924422	T	C	STRC	INTRONIC	ENSG00000242866:[ENST00000541030(c.-1103-13868A>G)]	NA	NA	NA	5.38E-02	6.71E-02	NA	NA	NA	NA	NA	LOW	NA	0.996	91	87	4	122	92	29	23.77
Chewer	IOB_33	chr15	71276573	C	T	LRRC49	SILENT	ENSG00000137821:[ENST00000544974(c.1116C>T,ctC>ctT):ENSP00000439600(p.Leu372(=))]	NA	NA	chr15*71276573*71276573*C*G*rs757871855*NA*NA*NA	NA	NA	NA	NA	NA	NA	NA	MODIFIER	NA	NA	110	110	0	128	109	19	14.84

Patel *et al.* , 2021. Whole-exome sequencing analysis of oral squamous cell carcinoma delineated by tobacco usage habits
Supplementary Table 4. List of somatic single nucleotide variants identified using whole exome sequencing of OSCC patients

Cohort	Sample	Chromosome	Genomic position	Reference allele	Alternate allele	Genesymbol	Variant type	Variant description	OncoMD	COSMIC	dbSNP	ExAC	ExAC_SAS	1000G	1000G_SAS	SIFT	Polyphen2	CONDEL	VEP	LRT	PhastCon7Way	Total reads in Normal	Reference read support in Normal	Alternative read support in Normal	Total reads in Tumor	Reference read support in Tumor	Alternate read support in Tumor	Alternate allele frequency in Tumor
Chewer	IOB_33	chr15	78810896	G	C	HYKK	NCT	123688:[XR_243078.1(n.573-34G>C)]	NA	NA	chr15*78810896*78810896*G*A*rs929897336*NA*NA*NA	NA	NA	NA	NA	NA	NA	NA	MODIFIER	NA	NA	101	100	1	121	103	18	14.88
Chewer	IOB_33	chr15	84694029	C	T	ADAMTSL3	SILENT	ENSG00000156218:[ENST00000286744(c.4497C>T,tgC>tgT):ENSP00000286744(p.Cys1499(=))]	NA	chr15*84694029*84694029*COSM1375050*C*T*1*large_intestine*1	3.99E-04	3.40E-05	2.18E-04	3.99E-04	1.00E-03	NA	NA	NA	LOW	NA	0.107	76	76	0	132	90	42	31.82
Chewer	IOB_33	chr16	705376	C	T	LA16c-349E10.1	EXONIC-NC	ENSG00000262528:[ENST00000573609(n.652G>A)]	NA	NA	NA	1.06E-05	7.09E-05	NA	NA	NA	NA	NA	MODIFIER	NA	NA	150	149	1	233	134	98	42.06
Chewer	IOB_33	chr16	705376	C	T	WDR90	SILENT	ENSG00000161996:[ENST00000293879(c.1626C>T,ccC>ccT):ENSP00000293879(p.Pro542(=))]	NA	NA	NA	1.06E-05	7.09E-05	NA	NA	NA	NA	NA	MODIFIER	NA	NA	150	149	1	233	134	98	42.06
Chewer	IOB_33	chr16	1918155	T	C	MEIOB	MISSENSE	ENSG00000162039:[ENST00000325962(c.22A>G,Agg>Ggg):ENSP00000314484(p.Arg8Gly)]	NA	NA	NA	NA	NA	NA	NA	NA	BN	NA	MODERATE	NA	NA	68	68	0	90	69	20	22.22
Chewer	IOB_33	chr16	19195383	G	A	SYT17	MISSENSE	ENSG00000103528:[ENST00000562034(c.682G>A,Gtc>Atc):ENSP00000456252(p.Val228Ile)]	NA	NA	NA	NA	NA	NA	NA	T	BN	NA	MODERATE	D	1	94	94	0	139	90	48	34.53
Chewer	IOB_33	chr16	23533217	G	A	GGA2	5UTR	ENSG00000103365:[ENST00000569300(c.-96C>T)]	NA	NA	NA	NA	NA	NA	NA	NA	NA	NA	MODIFIER	NA	NA	65	65	0	122	117	5	4.1
Chewer	IOB_33	chr16	31437328	C	T	ITGAD	INTRONIC	3681:[NM_001318185.1(c.3376-8C>T)]	NA	NA	chr16*31437328*31437330*CCT*C*rs777250618*NA*NA*NA	NA	NA	NA	NA	NA	NA	NA	LOW	NA	NA	61	61	0	71	48	23	32.39
Chewer	IOB_33	chr16	49670567	C	G	ZNF423	SILENT	ENSG00000102935:[ENST00000535559(c.2145G>C,gtG>gtC):ENSP00000442321(p.Val715(=))]	NA	NA	chr16*49670567*49670567*C*T*rs1042660017*NA*NA*NA	NA	NA	NA	NA	NA	NA	NA	LOW	NA	NA	202	202	0	360	322	38	10.56
Chewer	IOB_33	chr16	67228783	C	A	E2F4	SILENT	ENSG00000205250:[ENST00000379378(c.708C>A,tcC>tcA):ENSP00000368686(p.Ser236(=))]	NA	NA	NA	NA	NA	NA	NA	NA	NA	NA	MODIFIER	NA	NA	137	136	0	229	223	6	2.62
Chewer	IOB_33	chr17	2598490	C	T	CLUH	EXONIC-NC	ENSG00000132361:[ENST00000573641(n.411G>A)]	NA	NA	chr17*2598490*2598490*C*A*rs753571498*NA*NA*NA	NA	NA	NA	NA	NA	NA	NA	MODIFIER	NA	NA	244	243	0	310	302	7	2.26
Chewer	IOB_33	chr17	7573982	C	A	TP53	NONSENSE	ENSG00000141510:[ENST00000269305(c.1045G>T,Gaa>Taa):ENSP00000269305(p.Glu349Ter)]	NA	chr17*7573970*7573989*COSM1133051*CATCCTTGAGTTCCAAGGCC*C*1*haematopoietic_and_lymphoid_tissue*1|chr17*7573981*7573982*COSM46056*TC*T*2*central_nervous_system,lung*1,1|chr17*7573982*7573982*COSM1386549*C*T*2*large_intestine*2|chr17*7573982*7573982*COSM10770*C*A*13*large_intestine,liver,oesophagus,breast,endometrium,lung,ovary,skin*3,3,1,1,1,2,1,1|chr17*7573982*7573982*COSM1386550*C*T*2*large_intestine*2|chr17*7573982*7573982*COSM140784*C*A*9*liver,oesophagus,large_intestine,endometrium,lung,skin*3,1,2,1,1,1	chr17*7573975*7573983*TTGAGTTCC*T*rs1057519625*NA*NA*NA	NA	NA	NA	NA	NA	NA	NA	MODIFIER	NA	NA	90	90	0	91	56	34	37.36
Chewer	IOB_33	chr17	10250132	A	C	MYH13	INTRONIC	ENSG00000006788:[ENST00000252172(c.1145-17T>G)]	NA	NA	NA	NA	NA	NA	NA	NA	NA	NA	MODIFIER	NA	NA	112	112	0	92	77	14	15.22
Chewer	IOB_33	chr17	10431083	G	T	CTC-297N7.11	NCT	ENSG00000272975:[ENST00000587182(n.156-39771G>T)]	NA	NA	NA	1.65E-05	0.00E+00	NA	NA	NA	NA	NA	MODIFIER	NA	NA	44	43	0	59	48	11	18.64
Chewer	IOB_33	chr17	10431083	G	T	MYH2	MISSENSE	ENSG00000125414:[ENST00000245503(c.3853C>A,Cgc>Agc):ENSP00000245503(p.Arg1285Ser)]	NA	NA	NA	1.65E-05	0.00E+00	NA	NA	NA	NA	NA	MODIFIER	NA	NA	44	43	0	59	48	11	18.64
Chewer	IOB_33	chr17	10431083	G	T	MYHAS	NCT	100128560:[NR_125367.1(n.168-39771G>T)]	NA	NA	NA	1.65E-05	0.00E+00	NA	NA	NA	NA	NA	MODIFIER	NA	NA	44	43	0	59	48	11	18.64
Chewer	IOB_33	chr17	10431083	G	T	RP11-799N11.1	NCT	ENSG00000272736:[ENST00000581304(n.144-5558G>T)]	NA	NA	NA	1.65E-05	0.00E+00	NA	NA	NA	NA	NA	MODIFIER	NA	NA	44	43	0	59	48	11	18.64
Chewer	IOB_33	chr17	15539387	C	G	RP11-385D13.1	MISSENSE	ENSG00000251537:[ENST00000455584(c.812G>C,aGg>aCg):ENSP00000402644(p.Arg271Thr)]	NA	chr17*15539387*15539387*COSM2738696*C*G*1*liver*1	NA	1.08E-02	2.19E-02	NA	NA	NA	NA	NA	MODERATE	NA	0.123	48	47	1	36	27	9	25
Chewer	IOB_33	chr17	15539387	C	G	TRIM16	MISSENSE	ENSG00000221926:[ENST00000578744(c.434G>C,aGg>aCg):ENSP00000465907(p.Arg145Thr)]	NA	chr17*15539387*15539387*COSM2738696*C*G*1*liver*1	NA	1.08E-02	2.19E-02	NA	NA	NA	NA	NA	MODERATE	NA	0.123	48	47	1	36	27	9	25
Chewer	IOB_33	chr17	20640413	G	A	AC126365.1	EXONIC-NC	ENSG00000226521:[ENST00000441215(n.1146G>A)]	NA	NA	NA	NA	NA	NA	NA	NA	NA	NA	MODIFIER	NA	NA	79	78	0	98	92	6	6.12
Chewer	IOB_33	chr17	20640413	G	A	LOC100287072	EXONIC-NC	100287072:[NR_073509.1(n.1378G>A)]	NA	NA	NA	NA	NA	NA	NA	NA	NA	NA	MODIFIER	NA	NA	79	78	0	98	92	6	6.12
Chewer	IOB_33	chr17	27188485	A	G	MIR451B	EXONIC-NC	ENSG00000264066:[ENST00000582320(n.500T>C)]	NA	NA	NA	NA	NA	NA	NA	NA	NA	NA	MODIFIER	NA	NA	45	45	0	70	64	6	8.57
Chewer	IOB_33	chr17	28749793	C	T	CPD	MISSENSE	ENSG00000108582:[ENST00000225719(c.1409C>T,aCg>aTg):ENSP00000225719(p.Thr470Met)]	NA	NA	.	7.41E-05	0.00E+00	NA	NA	T	BN	NA	MODERATE	U	0.001	279	278	0	389	336	52	13.37
Chewer	IOB_33	chr17	29206419	T	G	ATAD5	INTRONIC	ENSG00000176208:[ENST00000321990(c.4013-22T>G)]	NA	NA	3.26E-02	3.94E-02	6.20E-02	NA	NA	NA	NA	NA	MODIFIER	NA	NA	52	51	0	75	59	16	21.33
Chewer	IOB_33	chr17	42513868	C	A	GPATCH8	MISSENSE	ENSG00000186566:[ENST00000591680(c.239G>T,gGc>gTc):ENSP00000467556(p.Gly80Val)]	NA	NA	NA	NA	NA	NA	NA	D	PrD	NA	MODERATE	D	1	199	198	0	278	271	5	1.8
Chewer	IOB_33	chr17	47700186	G	A	SPOP	5UTR	ENSG00000121067:[ENST00000347630(c.-14C>T)]	NA	NA	NA	NA	NA	NA	NA	NA	NA	NA	MODIFIER	NA	NA	71	71	0	122	116	6	4.92
Chewer	IOB_33	chr17	56385151	C	T	BZRAP1	INTRONIC	ENSG00000005379:[ENST00000268893(c.4652-28G>A)]	NA	NA	NA	NA	NA	NA	NA	NA	NA	NA	MODIFIER	NA	NA	73	73	0	130	113	17	13.08
Chewer	IOB_33	chr17	61775975	G	A	LIMD2	SILENT	ENSG00000136490:[ENST00000580222(c.174C>T,ggC>ggT):ENSP00000463409(p.Gly58(=))]	NA	NA	NA	NA	NA	NA	NA	NA	NA	NA	MODIFIER	NA	NA	94	94	0	195	188	6	3.08
Chewer	IOB_33	chr17	72357918	C	A	BTBD17	MISSENSE	ENSG00000204347:[ENST00000375366(c.41G>T,aGc>aTc):ENSP00000364515(p.Ser14Ile)]	NA	NA	NA	NA	NA	NA	NA	T_lc	BN	NA	MODERATE	N	1	170	170	0	258	252	6	2.33
Chewer	IOB_33	chr17	73495447	G	A	KIAA0195	EXONIC-NC	ENSG00000177728:[ENST00000585105(n.4908G>A)]	NA	NA	NA	1.66E-05	0.00E+00	NA	NA	NA	NA	NA	MODIFIER	NA	NA	73	73	0	134	99	35	26.12
Chewer	IOB_33	chr17	79639485	C	A	CCDC137	INTRONIC	ENSG00000185298:[ENST00000571916(c.*296-40C>A)]	NA	NA	NA	NA	NA	NA	NA	NA	NA	NA	MODIFIER	NA	NA	90	90	0	129	123	6	4.65
Chewer	IOB_33	chr17	79995536	C	A	DCXR	MISSENSE	ENSG00000169738:[ENST00000581584(c.18G>T,cGc>cTc):ENSP00000464507(p.Arg7Leu)]	NA	NA	NA	NA	NA	NA	NA	T	BN	NA	MODERATE	NA	NA	129	128	0	206	192	14	6.8
Chewer	IOB_33	chr18	346726	T	C	COLEC12	MISSENSE	ENSG00000158270:[ENST00000400256(c.896A>G,aAc>aGc):ENSP00000383115(p.Asn299Ser)]	NA	NA	NA	NA	NA	NA	NA	D	PrD	NA	MODERATE	D	1	138	138	0	371	340	31	8.36
Chewer	IOB_33	chr18	18549252	G	T	ROCK1	EXONIC-NC	ENSG00000067900:[ENST00000584875(n.64C>A)]	NA	NA	chr18*18549252*18549252*G*C*rs1050648395*NA*NA*NA	NA	NA	NA	NA	NA	NA	NA	MODIFIER	NA	NA	33	33	0	54	38	15	27.78
Chewer	IOB_33	chr18	43568573	G	A	PSTPIP2	INTRONIC	ENSG00000152229:[ENST00000409746(c.*8+95C>T)]	NA	NA	NA	NA	NA	NA	NA	NA	NA	NA	MODIFIER	NA	NA	71	71	0	89	78	11	12.36
Chewer	IOB_33	chr18	44416350	T	A	PIAS2	MISSENSE	ENSG00000078043:[ENST00000324794(c.1172A>T,aAa>aTa):ENSP00000317163(p.Lys391Ile)]	NA	NA	NA	NA	NA	NA	NA	NA	NA	NA	MODIFIER	NA	NA	156	156	0	183	131	52	28.42
Chewer	IOB_33	chr18	51053073	C	A	DCC	MISSENSE	ENSG00000187323:[ENST00000581580(c.3097C>A,Cca>Aca):ENSP00000464582(p.Pro1033Thr)]	NA	NA	NA	NA	NA	NA	NA	NA	NA	NA	MODIFIER	NA	NA	108	108	0	84	58	25	29.76
Chewer	IOB_33	chr18	51053073	C	A	RP11-671P2.1	NCT	ENSG00000264263:[ENST00000582064(n.121-21383G>T)]	NA	NA	NA	NA	NA	NA	NA	NA	NA	NA	MODIFIER	NA	NA	108	108	0	84	58	25	29.76
Chewer	IOB_33	chr19	3816803	G	A	ZFR2	NONSENSE	ENSG00000105278:[ENST00000262961(c.1972C>T,Cga>Tga):ENSP00000262961(p.Arg658Ter)]	NA	NA	NA	2.30E-05	0.00E+00	NA	NA	NA	NA	NA	HIGH	NA	1	131	131	0	221	216	5	2.26
Chewer	IOB_33	chr19	3959445	C	T	DAPK3	MISSENSE	ENSG00000167657:[ENST00000301264(c.1019G>A,cGc>cAc):ENSP00000301264(p.Arg340His)]	NA	NA	NA	NA	NA	NA	NA	NA	NA	NA	MODIFIER	NA	NA	96	95	1	112	86	26	23.21
Chewer	IOB_33	chr19	4769615	G	A	MIR7-3HG	NCT	ENSG00000176840:[ENST00000317292(n.213-27G>A)]	NA	NA	chr19*4769615*4769615*G*C*rs755286825*NA*NA*NA	NA	NA	NA	NA	NA	NA	NA	MODIFIER	NA	NA	126	123	0	84	65	19	22.62
Chewer	IOB_33	chr19	5690619	C	T	RPL36	EXONIC-NC	ENSG00000130255:[ENST00000590786(n.125C>T)]	NA	NA	NA	5.02E-05	0.00E+00	NA	NA	NA	NA	NA	LOW	NA	NA	93	93	0	128	122	6	4.69
Chewer	IOB_33	chr19	8398817	C	A	KANK3	MISSENSE	ENSG00000186994:[ENST00000595639(c.318G>T,gaG>gaT):ENSP00000470585(p.Glu106Asp)]	NA	NA	chr19*8398817*8398817*C*G,T*rs760119579*NA*NA*NA	NA	NA	NA	NA	T	BN	NA	MODERATE	NA	NA	75	75	0	113	107	6	5.31
Chewer	IOB_33	chr19	9060407	G	T	MUC16	SILENT	ENSG00000181143:[ENST00000397910(c.27039C>A,atC>atA):ENSP00000381008(p.Ile9013(=))]	NA	NA	chr19*9060407*9060407*G*A,T*rs764689108*NA*NA*NA	8.29E-06	0.00E+00	NA	NA	NA	NA	NA	LOW	NA	NA	130	130	0	168	130	38	22.62
Chewer	IOB_33	chr19	10893731	C	T	DNM2	MISSENSE	ENSG00000079805:[ENST00000587830(c.40C>T,Cac>Tac):ENSP00000466603(p.His14Tyr)]	NA	NA	NA	NA	NA	NA	NA	NA	NA	NA	MODIFIER	NA	NA	108	106	0	144	138	6	4.17
Chewer	IOB_33	chr19	17111414	T	C	CPAMD8	INTRONIC	ENSG00000160111:[ENST00000388925(c.759-82A>G)]	NA	NA	NA	NA	NA	NA	NA	NA	NA	NA	MODIFIER	NA	NA	100	100	0	94	89	5	5.32
Chewer	IOB_33	chr19	33490500	C	T	RHPN2	MISSENSE	ENSG00000131941:[ENST00000400226(c.764G>A,cGa>cAa):ENSP00000402244(p.Arg255Gln)]	NA	chr19*33490500*33490500*COSM140214*C*T*2*breast,skin*1,1	NA	7.46E-05	0.00E+00	NA	NA	T	BN	NA	MODERATE	N	0	45	45	0	60	50	9	15
Chewer	IOB_33	chr19	36109850	G	A	HAUS5	MISSENSE	ENSG00000249115:[ENST00000203166(c.1078G>A,Gat>Aat):ENSP00000439056(p.Asp360Asn)]	NA	NA	2.00E-04	8.37E-05	0.00E+00	2.00E-04	0.00E+00	NA	NA	NA	MODIFIER	NA	NA	128	127	0	157	134	23	14.65
Chewer	IOB_33	chr19	36234635	G	C	AD000671.6	INTRONIC	ENSG00000267120:[ENST00000589807(c.*222+75C>G)]	NA	NA	NA	NA	NA	NA	NA	NA	NA	NA	MODIFIER	NA	NA	30	30	0	43	32	11	25.58
Chewer	IOB_33	chr19	36234635	G	C	U2AF1L4	NCT	ENSG00000161265:[ENST00000592913(n.703+75C>G)]	NA	NA	NA	NA	NA	NA	NA	NA	NA	NA	MODIFIER	NA	NA	30	30	0	43	32	11	25.58
Chewer	IOB_33	chr19	44171863	G	A	PLAUR	INTRONIC	ENSG00000011422:[ENST00000593939(c.41-20C>T)]	NA	NA	NA	NA	NA	NA	NA	NA	NA	NA	MODIFIER	NA	NA	185	185	0	273	173	99	36.26
Chewer	IOB_33	chr19	46832490	G	A	AC007193.10	NCT	ENSG00000269124:[ENST00000596807(n.251-8815C>T)]	NA	NA	NA	NA	NA	NA	NA	NA	NA	NA	MODIFIER	NA	NA	93	93	0	189	183	5	2.65
Chewer	IOB_33	chr19	46832490	G	A	HIF3A	MISSENSE	ENSG00000124440:[ENST00000339613(c.1299G>A,atG>atA):ENSP00000341877(p.Met433Ile)]	NA	NA	NA	NA	NA	NA	NA	NA	NA	NA	MODIFIER	NA	NA	93	93	0	189	183	5	2.65
Chewer	IOB_33	chr19	49122376	G	A	RPL18	5UTR	ENSG00000063177:[ENST00000550973(c.-713C>T)]	NA	NA	NA	NA	NA	NA	NA	NA	NA	NA	MODIFIER	NA	NA	127	122	0	173	167	6	3.47
Chewer	IOB_33	chr19	51021220	C	T	LRRC4B	MISSENSE	ENSG00000131409:[ENST00000389201(c.1750G>A,Gcc>Acc):ENSP00000373853(p.Ala584Thr)]	NA	NA	NA	NA	NA	NA	NA	D	PrD	NA	MODERATE	U	1	200	200	0	324	317	7	2.16
Chewer	IOB_33	chr19	53513082	C	G	CTD-2620I22.3	EXONIC-NC	ENSG00000267943:[ENST00000596769(n.709C>G)]	NA	NA	chr19*53513082*53513082*C*T*rs375982775*NA*NA*NA	NA	NA	NA	NA	NA	NA	NA	MODIFIER	NA	NA	141	140	0	153	129	24	15.69
Chewer	IOB_33	chr19	53513082	C	G	ZNF702P	NCT	ENSG00000242779:[ENST00000594516(n.160-22983G>C)]	NA	NA	chr19*53513082*53513082*C*T*rs375982775*NA*NA*NA	NA	NA	NA	NA	NA	NA	NA	MODIFIER	NA	NA	141	140	0	153	129	24	15.69
Chewer	IOB_33	chr19	53644258	T	A	ZNF347	MISSENSE	ENSG00000197937:[ENST00000452676(c.1826A>T,cAt>cTt):ENSP00000405218(p.His609Leu)]	NA	NA	chr19*53644258*53644258*T*C*rs376138740*NA*NA*NA	NA	NA	NA	NA	NA	NA	NA	MODERATE	NA	NA	142	140	0	197	162	35	17.77
Chewer	IOB_33	chr19	57967796	A	T	AC004076.9	INTRONIC	ENSG00000268163:[ENST00000596831(c.200-246T>A)]	NA	NA	NA	NA	NA	NA	NA	D_lc	PoD	NA	MODERATE	NA	NA	56	56	0	81	64	17	20.99
Chewer	IOB_33	chr19	57967796	A	T	VN1R1	MISSENSE	ENSG00000178201:[ENST00000321039(c.59T>A,cTt>cAt):ENSP00000322339(p.Leu20His)]	NA	NA	NA	NA	NA	NA	NA	D_lc	PoD	NA	MODERATE	NA	NA	56	56	0	81	64	17	20.99

Patel *et al.* , 2021. Whole-exome sequencing analysis of oral squamous cell carcinoma delineated by tobacco usage habits
Supplementary Table 4. List of somatic single nucleotide variants identified using whole exome sequencing of OSCC patients

Cohort	Sample	Chromosome	Genomic position	Reference allele	Alternate allele	Genesymbol	Variant type	Variant description	OncoMD	COSMIC	dbSNP	ExAC	ExAC_SAS	1000G	1000G_SAS	SIFT	Polyphen2	CONDEL	VEP	LRT	PhastCon7Way	Total reads in Normal	Reference read support in Normal	Alternative read support in Normal	Total reads in Tumor	Reference read support in Tumor	Alternate read support in Tumor	Alternate allele frequency in Tumor
Chewer	IOB_33	chr2	3392130	G	A	TRAPPC12	MISSENSE	ENSG00000171853:[ENST00000411973(c.233G>A,Gcc>Acc):ENSP00000405626(p.Ala79Thr)]	NA	chr2*3392130*3392130*COSM721109*G*A*1*lung*1	NA	1.07E-05	0.00E+00	NA	NA	T_lc	BN	NA	MODERATE	N	1	285	283	1	308	301	6	1.95
Chewer	IOB_33	chr2	11595468	C	A	E2F6	3UTR-SS-PRX	ENSG00000169016:[ENST00000455198(c.*214G>T)]	NA	NA	NA	NA	NA	NA	NA	NA	NA	NA	MODIFIER	NA	NA	181	181	0	290	241	49	16.9
Chewer	IOB_33	chr2	26178398	G	A	KIF3C	NCT	ENSG00000084731:[ENST00000475453(n.475+12C>T)]	NA	NA	NA	NA	NA	NA	NA	NA	NA	NA	MODIFIER	NA	NA	135	135	0	201	195	5	2.49
Chewer	IOB_33	chr2	32449565	C	A	NLRC4	MISSENSE	ENSG00000091106:[ENST00000360906(c.3052G>T,Gct>Tct):ENSP00000354159(p.Ala1018Ser)]	NA	NA	NA	NA	NA	NA	NA	T	BN	NA	MODERATE	N	0.054	164	162	1	231	223	7	3.03
Chewer	IOB_33	chr2	55589612	C	A	CCDC88A	INTRONIC	ENSG00000115355:[ENST00000430086(c.262-28G>T)]	NA	NA	NA	NA	NA	NA	NA	NA	NA	NA	MODIFIER	NA	NA	59	59	0	77	72	5	6.49
Chewer	IOB_33	chr2	74076531	C	A	STAMBP	SILENT	ENSG00000124356:[ENST00000339566(c.784C>A,Cgg>Agg):ENSP00000344742(p.Arg262(=))]	NA	NA	chr2*74076531*74076531*C*T*rs747329455*NA*NA*NA	NA	NA	NA	NA	NA	NA	NA	MODIFIER	NA	NA	31	31	0	50	45	5	10
Chewer	IOB_33	chr2	99227264	C	A	UNC50	MISSENSE	ENSG00000115446:[ENST00000409347(c.358C>A,Ctg>Atg):ENSP00000386466(p.Leu120Met)]	NA	NA	NA	NA	NA	NA	NA	T	PoD	NA	MODERATE	D	1	201	200	0	298	292	6	2.01
Chewer	IOB_33	chr2	101889388	C	A	RNF149	INTRONIC	ENSG00000163162:[ENST00000424632(c.*177+201G>T)]	NA	NA	NA	NA	NA	NA	NA	NA	NA	NA	MODIFIER	NA	NA	153	153	0	195	189	5	2.56
Chewer	IOB_33	chr2	102003702	C	T	CREG2	MISSENSE	ENSG00000175874:[ENST00000324768(c.218G>A,aGc>aAc):ENSP00000315203(p.Ser73Asn)]	NA	NA	NA	NA	NA	NA	NA	T	PoD	NA	MODERATE	N	1	90	90	0	116	111	5	4.31
Chewer	IOB_33	chr2	129075919	G	A	HS6ST1	SILENT	ENSG00000136720:[ENST00000259241(c.219C>T,cgC>cgT):ENSP00000259241(p.Arg73(=))]	NA	chr2*129075919*129075919*COSM1641641*G*A*1*stomach*1	NA	4.55E-02	9.79E-02	NA	NA	NA	NA	NA	MODIFIER	NA	NA	51	51	0	80	66	14	17.5
Chewer	IOB_33	chr2	155555662	C	A	KCNJ3	SILENT	ENSG00000162989:[ENST00000295101(c.375C>A,gcC>gcA):ENSP00000295101(p.Ala125(=))]	NA	NA	NA	NA	NA	NA	NA	NA	NA	NA	LOW	NA	NA	191	191	0	311	248	61	19.61
Chewer	IOB_33	chr2	162758698	T	A	SLC4A10	INTRONIC	57282:[XM_005246694.1(c.1475+1177T>A)]	NA	NA	NA	NA	NA	NA	NA	NA	NA	NA	MODIFIER	NA	NA	127	126	0	190	159	31	16.32
Chewer	IOB_33	chr2	162876830	A	C	DPP4	INTRONIC	1803:[XM_005246371.1(c.1174-24T>G)]	NA	NA	NA	4.00E-04	0.00E+00	NA	NA	NA	NA	NA	MODIFIER	NA	NA	47	45	0	49	39	5	10.2
Chewer	IOB_33	chr2	175622377	C	T	AC018890.6	NCT	ENSG00000236449:[ENST00000442996(n.322-15100C>T)]	NA	NA	chr2*175622377*175622377*C*G*rs768240064*NA*NA*NA	NA	NA	NA	NA	NA	NA	NA	HIGH	NA	1	133	131	0	234	195	39	16.67
Chewer	IOB_33	chr2	175622377	C	T	CHRNA1	NONSENSE	ENSG00000138435:[ENST00000348749(c.261G>A,tgG>tgA):ENSP00000261008(p.Trp87Ter)]	NA	NA	chr2*175622377*175622377*C*G*rs768240064*NA*NA*NA	NA	NA	NA	NA	NA	NA	NA	HIGH	NA	1	133	131	0	234	195	39	16.67
Chewer	IOB_33	chr2	185802015	G	T	ZNF804A	MISSENSE	ENSG00000170396:[ENST00000302277(c.1892G>T,gGg>gTg):ENSP00000303252(p.Gly631Val)]	NA	NA	NA	NA	NA	NA	NA	T	BN	NA	MODERATE	N	0.205	89	89	0	60	35	25	41.67
Chewer	IOB_33	chr2	186658179	C	A	AC008174.3	NCT	ENSG00000231646:[ENST00000429929(n.73-4509G>T)]	NA	NA	NA	NA	NA	NA	NA	NA	NA	NA	MODIFIER	NA	NA	87	87	0	118	95	23	19.49
Chewer	IOB_33	chr2	186658179	C	A	FSIP2	MISSENSE	ENSG00000188738:[ENST00000424728(c.6316C>A,Ctg>Atg):ENSP00000401306(p.Leu2106Met)]	NA	NA	NA	NA	NA	NA	NA	NA	NA	NA	MODIFIER	NA	NA	87	87	0	118	95	23	19.49
Chewer	IOB_33	chr2	189434857	T	A	GULP1	NCT	ENSG00000144366:[ENST00000476422(n.537+10T>A)]	NA	NA	NA	NA	NA	NA	NA	NA	NA	NA	MODIFIER	NA	NA	52	52	0	61	48	13	21.31
Chewer	IOB_33	chr2	226491717	C	T	NYAP2	MISSENSE	ENSG00000144460:[ENST00000272907(c.1703C>T,gCg>gTg):ENSP00000272907(p.Ala568Val)]	NA	chr2*226491717*226491717*COSM3046851*C*T*1*large_intestine*1	NA	NA	NA	NA	NA	T	BN	NA	MODERATE	N	0.062	129	128	1	163	127	36	22.09
Chewer	IOB_33	chr2	237172977	C	T	AC079135.1	NCT	ENSG00000233611:[ENST00000415226(n.316+4735C>T)]	NA	NA	chr2*237172977*237172977*C*G,T*rs781251806*NA*NA*NA	2.49E-05	6.06E-05	NA	NA	NA	NA	NA	LOW	NA	NA	61	60	0	95	82	13	13.68
Chewer	IOB_33	chr2	237172977	C	T	ASB18	SILENT	ENSG00000182177:[ENST00000409749(c.12G>A,tcG>tcA):ENSP00000386532(p.Ser4(=))]	NA	NA	chr2*237172977*237172977*C*G,T*rs781251806*NA*NA*NA	2.49E-05	6.06E-05	NA	NA	NA	NA	NA	LOW	NA	NA	61	60	0	95	82	13	13.68
Chewer	IOB_33	chr20	257888	C	A	C20orf96	MISSENSE	ENSG00000196476:[ENST00000382369(c.597G>T,caG>caT):ENSP00000371806(p.Gln199His)]	NA	NA	NA	NA	NA	NA	NA	D	PrD	NA	MODERATE	NA	NA	109	109	0	226	199	27	11.95
Chewer	IOB_33	chr20	4228926	C	T	ADRA1D	MISSENSE	ENSG00000171873:[ENST00000379453(c.679G>A,Gtg>Atg):ENSP00000368766(p.Val227Met)]	NA	NA	chr20*4228926*4228926*C*A*rs367566190*NA*NA*NA	NA	NA	NA	NA	D	PrD	NA	MODERATE	D	1	257	255	0	439	377	62	14.12
Chewer	IOB_33	chr20	39795158	G	T	PLCG1	MISSENSE	ENSG00000124181:[ENST00000244007(c.2043G>T,atG>atT):ENSP00000244007(p.Met681Ile)]	NA	NA	NA	NA	NA	NA	NA	T	BN	NA	MODERATE	N	1	210	210	0	385	327	58	15.06
Chewer	IOB_33	chr20	43355915	C	A	KCNK15-AS1	NCT	106144538:[NR_132377.1(n.438+10237G>T)]	NA	NA	NA	NA	NA	NA	NA	NA	NA	NA	MODIFIER	NA	NA	80	80	0	153	146	7	4.58
Chewer	IOB_33	chr20	43355915	C	A	RP11-445H22.4	NCT	ENSG00000244558:[ENST00000427598(n.78-10481G>T)]	NA	NA	NA	NA	NA	NA	NA	NA	NA	NA	MODIFIER	NA	NA	80	80	0	153	146	7	4.58
Chewer	IOB_33	chr20	43355915	C	A	WISP2	MISSENSE	ENSG00000064205:[ENST00000372865(c.473C>A,cCa>cAa):ENSP00000361956(p.Pro158Gln)]	NA	NA	NA	NA	NA	NA	NA	NA	NA	NA	MODIFIER	NA	NA	80	80	0	153	146	7	4.58
Chewer	IOB_33	chr20	45169989	G	C	OCSTAMP	MISSENSE	ENSG00000149635:[ENST00000279028(c.1625C>G,aCc>aGc):ENSP00000279028(p.Thr542Ser)]	NA	NA	NA	NA	NA	NA	NA	D_lc	BN	NA	MODERATE	NA	NA	72	72	0	118	103	15	12.71
Chewer	IOB_33	chr20	47712581	C	T	CSE1L	INTRONIC	ENSG00000124207:[ENST00000262982(c.2827-305C>T)]	NA	NA	NA	NA	NA	NA	NA	NA	NA	NA	MODIFIER	NA	NA	57	57	0	78	63	15	19.23
Chewer	IOB_33	chr20	61467792	C	T	COL9A3	EXONIC-NC	ENSG00000092758:[ENST00000467819(n.22C>T)]	NA	NA	NA	NA	NA	NA	NA	NA	NA	NA	MODIFIER	NA	NA	314	314	0	485	477	8	1.65
Chewer	IOB_33	chr20	62178624	G	A	SRMS	MISSENSE	ENSG00000125508:[ENST00000217188(c.193C>T,Cgg>Tgg):ENSP00000217188(p.Arg65Trp)]	NA	chr20*62178624*62178624*COSM1413128*G*A*3*liver,large_intestine*2,1	NA	9.54E-06	0.00E+00	NA	NA	D	PrD	NA	MODERATE	D	0.922	80	80	0	86	73	13	15.12
Chewer	IOB_33	chr21	47552269	G	A	COL6A2	MISSENSE	ENSG00000142173:[ENST00000300527(c.2863G>A,Gac>Aac):ENSP00000300527(p.Asp955Asn)]	NA	NA	NA	1.71E-05	0.00E+00	NA	NA	T	BN	NA	MODERATE	N	0.895	186	185	0	290	282	7	2.41
Chewer	IOB_33	chr22	19706128	C	T	SEPT5	INTRONIC	ENSG00000184702:[ENST00000431124(c.169-997C>T)]	NA	NA	NA	NA	NA	NA	NA	NA	NA	NA	MODIFIER	NA	NA	229	229	0	376	368	8	2.13
Chewer	IOB_33	chr22	19706128	C	T	SEPT5-GP1BB	EXONIC-NC	100526833:[NR_037611.1(n.1386C>T)]	NA	NA	NA	NA	NA	NA	NA	NA	NA	NA	MODIFIER	NA	NA	229	229	0	376	368	8	2.13
Chewer	IOB_33	chr22	30394910	C	A	MTMR3	INTRONIC	8897:[XM_005261809.1(c.143+64C>A)]	NA	NA	NA	NA	NA	NA	NA	NA	NA	NA	MODIFIER	NA	NA	146	146	0	150	145	5	3.33
Chewer	IOB_33	chr22	33402605	C	T	SYN3	MISSENSE	ENSG00000185666:[ENST00000332840(c.43G>A,Gcc>Acc):ENSP00000330219(p.Ala15Thr)]	NA	NA	NA	NA	NA	NA	NA	D	BN	NA	MODERATE	NA	NA	127	126	1	165	159	6	3.64
Chewer	IOB_33	chr22	51016274	C	A	CHKB-CPT1B	EXONIC-NC	386593:[NR_027928.2(n.1641G>T)]	NA	NA	NA	NA	NA	NA	NA	NA	NA	NA	MODIFIER	NA	NA	128	128	0	142	136	5	3.52
Chewer	IOB_33	chr22	51016274	C	A	CPT1B	MISSENSE	ENSG00000205560:[ENST00000312108(c.71G>T,aGt>aTt):ENSP00000312189(p.Ser24Ile)]	NA	NA	NA	NA	NA	NA	NA	NA	NA	NA	MODIFIER	NA	NA	128	128	0	142	136	5	3.52
Chewer	IOB_33	chr3	3030156	G	T	CNTN4	MISSENSE-SS-PRX	ENSG00000144619:[ENST00000427741(c.*684G>T)]	NA	NA	NA	NA	NA	NA	NA	NA	NA	NA	MODIFIER	NA	NA	157	157	0	150	123	27	18
Chewer	IOB_33	chr3	14545130	C	A	GRIP2	MISSENSE	80852:[NM_001080423.2(c.2913G>T,gaG>gaT):NP_001073892.2(p.Glu971Asp)]	NA	NA	NA	NA	NA	NA	NA	NA	NA	NA	HIGH	NA	NA	102	102	0	157	150	5	3.18
Chewer	IOB_33	chr3	38051897	A	G	PLCD1	MISSENSE	ENSG00000187091:[ENST00000334661(c.947T>C,cTg>cCg):ENSP00000335600(p.Leu316Pro)]	NA	NA	NA	NA	NA	NA	NA	NA	NA	NA	MODIFIER	NA	NA	112	111	0	139	106	33	23.74
Chewer	IOB_33	chr3	45637560	C	A	LIMD1	MISSENSE	ENSG00000144791:[ENST00000273317(c.1189C>A,Cct>Act):ENSP00000273317(p.Pro397Thr)]	NA	NA	NA	NA	NA	NA	NA	D	PoD	NA	MODERATE	N	0.01	136	136	0	163	158	5	3.07
Chewer	IOB_33	chr3	47034004	G	T	NBEAL2	MISSENSE	ENSG00000160796:[ENST00000292309(c.1072G>T,Gac>Tac):ENSP00000292309(p.Asp358Tyr)]	NA	NA	NA	NA	NA	NA	NA	NA	NA	NA	MODIFIER	NA	NA	72	72	0	80	63	17	21.25
Chewer	IOB_33	chr3	47899025	C	A	MAP4	INTRONIC	4134:[XM_005265158.1(c.2329-23G>T)]	NA	NA	NA	NA	NA	NA	NA	NA	NA	NA	MODIFIER	NA	NA	89	89	0	90	66	24	26.67
Chewer	IOB_33	chr3	48623721	C	A	COL7A1	INTRONIC	ENSG00000114270:[ENST00000328333(c.3551-42G>T)]	NA	NA	NA	NA	NA	NA	NA	NA	NA	NA	MODIFIER	NA	NA	201	198	0	230	202	27	11.74
Chewer	IOB_33	chr3	50387320	C	A	NPRL2	NCT	ENSG00000114388:[ENST00000469839(n.293+42G>T)]	NA	NA	NA	NA	NA	NA	NA	NA	NA	NA	MODIFIER	NA	NA	109	109	0	140	135	5	3.57
Chewer	IOB_33	chr3	56330467	C	A	ERC2	NCT-SS-PRX	26059:[XR_245116.1(n.908-4G>T)]	NA	NA	NA	NA	NA	NA	NA	NA	NA	NA	LOW	NA	NA	64	64	0	78	63	14	17.95
Chewer	IOB_33	chr3	109195630	C	A	FLJ25363	INTRONIC	401082:[NM_001145553.1(c.287+21C>A)]	NA	NA	NA	NA	NA	NA	NA	NA	NA	NA	MODIFIER	NA	NA	44	44	0	46	37	8	17.39
Chewer	IOB_33	chr3	109195630	C	A	RP11-702L6.4	NCT	ENSG00000228980:[ENST00000497996(n.464+21C>A)]	NA	NA	NA	NA	NA	NA	NA	NA	NA	NA	MODIFIER	NA	NA	44	44	0	46	37	8	17.39
Chewer	IOB_33	chr3	125865696	G	A	ALDH1L1	MISSENSE	ENSG00000144908:[ENST00000452905(c.755C>T,gCg>gTg):ENSP00000395881(p.Ala252Val)]	NA	chr3*125865696*125865696*COSM1037718*G*A*1*endometrium*1	NA	2.50E-05	0.00E+00	NA	NA	D_lc	PoD	NA	MODERATE	D	1	57	56	0	120	109	11	9.17
Chewer	IOB_33	chr3	133098683	C	T	NA	NCT	101927432:[XR_241569.1(n.103+1824G>A)]	NA	NA	NA	NA	NA	NA	NA	D_lc	BN	NA	MODERATE	N	0.999	137	137	0	326	287	37	11.35
Chewer	IOB_33	chr3	133098683	C	T	TMEM108	MISSENSE	ENSG00000144868:[ENST00000321871(c.128C>T,tCa>tTa):ENSP00000324651(p.Ser43Leu)]	NA	NA	NA	NA	NA	NA	NA	D_lc	BN	NA	MODERATE	N	0.999	137	137	0	326	287	37	11.35
Chewer	IOB_33	chr3	139071547	C	A	MRPS22	NONSENSE	ENSG00000175110:[ENST00000480644(c.276C>A,tCa>tAa):ENSP00000420229(p.Ser93Ter)]	NA	NA	NA	NA	NA	NA	NA	NA	NA	NA	HIGH	NA	1	45	45	0	92	86	5	5.43
Chewer	IOB_33	chr3	170244619	A	G	CLDN11	INTRONIC	ENSG00000013297:[ENST00000451576(c.391+103504A>G)]	NA	NA	NA	NA	NA	NA	NA	NA	NA	NA	MODIFIER	NA	NA	97	97	0	321	264	57	17.76
Chewer	IOB_33	chr3	170244619	A	G	LOC101928583	NCT	101928583:[NR_135556.1(n.215+49958A>G)]	NA	NA	NA	NA	NA	NA	NA	NA	NA	NA	MODIFIER	NA	NA	97	97	0	321	264	57	17.76
Chewer	IOB_33	chr3	170244619	A	G	SLC7A14	MISSENSE	ENSG00000013293:[ENST00000231706(c.107T>C,cTa>cCa):ENSP00000231706(p.Leu36Pro)]	NA	NA	NA	NA	NA	NA	NA	NA	NA	NA	MODIFIER	NA	NA	97	97	0	321	264	57	17.76
Chewer	IOB_33	chr3	172028552	G	T	FNDC3B	NCT	ENSG00000075420:[ENST00000469491(n.1342-66G>T)]	NA	NA	NA	NA	NA	NA	NA	NA	NA	NA	MODIFIER	NA	NA	47	47	0	95	87	8	8.42
Chewer	IOB_33	chr3	180693187	G	A	FXR1	MISSENSE	ENSG00000114416:[ENST00000357559(c.1690G>A,Gaa>Aaa):ENSP00000350170(p.Glu564Lys)]	NA	NA	chr3*180693187*180693187*G*C*rs777772932*NA*NA*NA	NA	NA	NA	NA	NA	NA	NA	MODERATE	NA	1	23	23	0	76	70	5	6.58
Chewer	IOB_33	chr3	185407214	G	T	IGF2BP2	SILENT	ENSG00000073792:[ENST00000346192(c.606C>A,acC>acA):ENSP00000320204(p.Thr202(=))]	NA	NA	chr3*185407214*185407214*G*C*rs771670912*NA*NA*NA	NA	NA	NA	NA	NA	NA	NA	LOW	NA	NA	41	41	0	78	50	27	34.62
Chewer	IOB_33	chr3	190580311	C	T	GMNC	INTRONIC	647309:[XM_005247707.1(c.28-1664G>A)]	NA	NA	NA	NA	NA	NA	NA	NA	NA	NA	MODIFIER	NA	NA	87	87	0	148	70	78	52.7
Chewer	IOB_33	chr4	5624630	C	T	EVC2	MISSENSE	ENSG00000173040:[ENST00000344408(c.2135G>A,aGc>aAc):ENSP00000342144(p.Ser712Asn)]	NA	NA	NA	8.30E-06	0.00E+00	NA	NA	NA	NA	NA	MODIFIER	NA	NA	107	107	0	150	119	31	20.67
Chewer	IOB_33	chr4	10529731	A	T	CLNK	INTRONIC	ENSG00000109684:[ENST00000442825(c.505-4146T>A)]	NA	NA	chr4*10529730*10529731*TA*T*rs755866213*NA*NA*NA	NA	NA	NA	NA	NA	NA	NA	MODIFIER	NA	NA	68	68	0	76	59	17	22.37
Chewer	IOB_33	chr4	43022299	T	C	GRXCR1	INTRONIC	ENSG00000215203:[ENST00000399770(c.628-72T>C)]	NA	NA	NA	NA	NA	NA	NA	NA	NA	NA	MODIFIER	NA	NA	41	41	0	51	44	7	13.73
Chewer	IOB_33	chr4	46312284	A	T	GABRA2	INTRONIC	ENSG00000151834:[ENST00000356504(c.477-12T>A)]	NA	NA	NA	NA	NA	NA	NA	NA	NA	NA	MODIFIER	NA	NA	194	193	0	232	196	36	15.52
Chewer	IOB_33	chr4	55970801	T	C	KDR	INTRONIC-SS-DNR-PRX	ENSG00000128052:[ENST00000263923(c.1987+9A>G)]	NA	NA	NA	NA	NA	NA	NA	NA	NA	NA	MODIFIER	NA	NA	110	110	0	223	153	69	30.94
Chewer	IOB_33	chr4	74005712	C	A	ANKRD17	MISSENSE	ENSG00000132466:[ENST00000558247(c.2275G>T,cGa>cTa):ENSP00000453434(p.Arg759Leu)]	NA	NA	chr4*74005712*74005712*C*T*rs145777158*NA*NA*NA	NA	NA	NA	NA	NA	NA	NA	MODIFIER	NA	NA	131	130	0	222	186	36	16.22
Chewer	IOB_33	chr4	96141349	T	G	UNC5C	INTRONIC	ENSG00000182168:[ENST00000506749(c.1166-22A>C)]	NA	NA	chr4*96141349*96141349*T*C*rs765434040*NA*NA*NA	NA	NA	NA	NA	NA	NA	NA	MODIFIER	NA	NA	65	65	0	106	88	18	16.98
Chewer	IOB_33	chr4	134071910	C	A	PCDH10	MISSENSE	ENSG00000138650:[ENST00000264360(c.615C>A,gaC>gaA):ENSP00000264360(p.Asp205Glu)]	NA	NA	NA	NA	NA	NA	NA	D	PrD	NA	MODERATE	N	1	195	184	0	325	298	6	1.85
Chewer	IOB_33	chr4	151818817	A	T	LRBA	INTRONIC	ENSG00000198589:[ENST00000357115(c.2004+74T>A)]	NA	NA	NA	NA	NA	NA	NA	NA	NA	NA	MODIFIER	NA	NA	70	70	0	66	59	7	10.61
Chewer	IOB_33	chr4	153601032	G	T	TMEM154	SILENT	ENSG00000170006:[ENST00000304385(c.54C>A,ccC>ccA):ENSP00000302144(p.Pro18(=))]	NA	NA	chr4*153601032*153601032*G*C*rs747637170*NA*NA*NA	NA	NA	NA	NA	NA	NA	NA	MODIFIER	NA	NA	150	149	0	165	147	18	10.91
Chewer	IOB_33	chr4	186329125	T	C	UFSP2	SILENT	ENSG00000109775:[ENST00000511485(c.781A>G,caA>caG):ENSP00000425855(p.Gln261(=))]	NA	NA	NA	1.90E-04	6.06E-05	NA	NA	NA	NA	NA	MODIFIER	NA	NA	161	160	0	217	188	29	13.36
Chewer	IOB_33	chr5	41159223	C	T	C6	MISSENSE	ENSG00000039537:[ENST00000263413(c.1817G>A,cGa>cAa):ENSP00000263413(p.Arg606Gln)]	NA	NA	NA	2.47E-05	0.00E+00	NA	NA	T	BN	NA	MODERATE	N	0	246	245	0	536	461	70	13.06
Chewer	IOB_33	chr5	50685769	G	A	ISL1	INTRONIC-SS-DNR-PRX	ENSG00000016082:[ENST00000230658(c.765+3G>A)]	NA	NA	NA	NA	NA	NA	NA	NA	NA	NA	LOW	NA	NA	141	140	0	217	193	24	11.06

Patel *et al.* , 2021. Whole-exome sequencing analysis of oral squamous cell carcinoma delineated by tobacco usage habits
Supplementary Table 4. List of somatic single nucleotide variants identified using whole exome sequencing of OSCC patients

Cohort	Sample	Chromosome	Genomic position	Reference allele	Alternate allele	Genesymbol	Variant type	Variant description	OncoMD	COSMIC	dbSNP	ExAC	ExAC_SAS	1000G	1000G_SAS	SIFT	Polyphen2	CONDEL	VEP	LRT	PhastCon7Way	Total reads in Normal	Reference read support in Normal	Alternative read support in Normal	Total reads in Tumor	Reference read support in Tumor	Alternate read support in Tumor	Alternate allele frequency in Tumor
Chewer	IOB_33	chr5	58270739	G	A	PDE4D	NONSENSE	ENSG00000113448:[ENST00000360047(c.1774C>T,Cag>Tag):ENSP00000353152(p.Gln592Ter)]	NA	NA	NA	NA	NA	NA	NA	NA	NA	NA	HIGH	NA	1	140	140	0	183	176	7	3.83
Chewer	IOB_33	chr5	74865178	C	T	POLK	MISSENSE	ENSG00000122008:[ENST00000503479(c.71C>T,gCa>gTa):ENSP00000421997(p.Ala24Val)]	NA	NA	NA	NA	NA	NA	NA	NA	NA	NA	MODIFIER	NA	NA	118	118	0	150	143	5	3.33
Chewer	IOB_33	chr5	111496878	G	A	EPB41L4A	INTRONIC	64097:[NM_001347887.1(c.1932+7558C>T)]	NA	NA	NA	NA	NA	NA	NA	NA	NA	NA	MODIFIER	NA	NA	81	81	0	156	151	5	3.21
Chewer	IOB_33	chr5	111496878	G	A	EPB41L4A-AS1	EXONIC-NC	ENSG00000224032:[ENST00000413221(n.656G>A)]	NA	NA	NA	NA	NA	NA	NA	NA	NA	NA	MODIFIER	NA	NA	81	81	0	156	151	5	3.21
Chewer	IOB_33	chr5	129521170	G	A	CHSY3	MISSENSE	ENSG00000198108:[ENST00000305031(c.2335G>A,Gga>Aga):ENSP00000302629(p.Gly779Arg)]	NA	NA	NA	NA	NA	NA	NA	D	PrD	NA	MODERATE	D	1	195	194	0	300	152	148	49.33
Chewer	IOB_33	chr5	132425286	C	A	HSPA4	MISSENSE	ENSG00000170606:[ENST00000304858(c.1277C>A,gCt>gAt):ENSP00000302961(p.Ala426Asp)]	NA	NA	NA	NA	NA	NA	NA	D	PoD	NA	MODERATE	D	1	148	148	0	256	250	6	2.34
Chewer	IOB_33	chr5	169535485	A	T	FOXI1	MISSENSE	ENSG00000168269:[ENST00000306268(c.1007A>T,gAc>gTc):ENSP00000304286(p.Asp336Val)]	NA	chr5*169535485*169535485*COSM3661709*A*C*2*liver*2	NA	NA	NA	NA	NA	D_lc	PoD	NA	MODERATE	N	1	209	206	0	303	160	142	46.86
Chewer	IOB_33	chr5	179297271	C	T	TBC1D9B	SILENT	ENSG00000197226:[ENST00000444477(c.183G>A,gaG>gaA):ENSP00000401585(p.Glu61(=))]	NA	NA	NA	NA	NA	NA	NA	NA	NA	NA	MODIFIER	NA	NA	175	175	0	259	128	130	50.19
Chewer	IOB_33	chr6	17616364	A	G	NUP153	SILENT	ENSG00000124789:[ENST00000537253(c.4485T>C,ggT>ggC):ENSP00000444029(p.Gly1495(=))]	NA	NA	NA	NA	NA	NA	NA	NA	NA	NA	LOW	NA	NA	84	84	0	171	146	23	13.45
Chewer	IOB_33	chr6	24357992	C	T	DCDC2	5UTR	ENSG00000146038:[ENST00000378454(c.-14G>A)]	NA	NA	NA	NA	NA	NA	NA	T_lc	BN	NA	MODERATE	NA	NA	349	348	0	517	466	49	9.48
Chewer	IOB_33	chr6	24357992	C	T	KAAG1	MISSENSE	ENSG00000146049:[ENST00000274766(c.125C>T,cCg>cTg):ENSP00000274766(p.Pro42Leu)]	NA	NA	NA	NA	NA	NA	NA	T_lc	BN	NA	MODERATE	NA	NA	349	348	0	517	466	49	9.48
Chewer	IOB_33	chr6	28359166	G	T	ZSCAN12	MISSENSE	ENSG00000158691:[ENST00000361028(c.901C>A,Ccc>Acc):ENSP00000354305(p.Pro301Thr)]	NA	NA	NA	NA	NA	NA	NA	NA	NA	NA	MODIFIER	NA	NA	138	138	0	179	162	16	8.94
Chewer	IOB_33	chr6	30297371	C	A	TRIM39	MISSENSE	ENSG00000204599:[ENST00000376656(c.277C>A,Cag>Aag):ENSP00000365844(p.Gln93Lys)]	NA	NA	chr6*30297371*30297371*C*G*rs776204161*NA*NA*NA	NA	NA	NA	NA	T	PoD	NA	MODERATE	NA	NA	171	171	0	253	244	6	2.37
Chewer	IOB_33	chr6	30297371	C	A	TRIM39-RPP21	MISSENSE	ENSG00000248167:[ENST00000513556(c.13C>A,Cag>Aag):ENSP00000424048(p.Gln5Lys)]	NA	NA	chr6*30297371*30297371*C*G*rs776204161*NA*NA*NA	NA	NA	NA	NA	T	PoD	NA	MODERATE	NA	NA	171	171	0	253	244	6	2.37
Chewer	IOB_33	chr6	31597469	A	C	PRRC2A	MISSENSE	ENSG00000204469:[ENST00000376007(c.2101A>C,Aag>Cag):ENSP00000365175(p.Lys701Gln)]	NA	NA	NA	7.43E-05	0.00E+00	NA	NA	NA	NA	NA	MODIFIER	NA	NA	36	35	0	63	54	6	9.52
Chewer	IOB_33	chr6	31937885	C	T	DXO	INTRONIC-SS-DNR	ENSG00000204348:[ENST00000478221(n.924+1G>A)]	NA	NA	chr6*31937885*31937885*C*G*rs750822960*NA*NA*NA	NA	NA	NA	NA	NA	NA	NA	HIGH	NA	NA	179	178	0	255	248	5	1.96
Chewer	IOB_33	chr6	39839324	G	A	DAAM2	SILENT	ENSG00000146122:[ENST00000274867(c.1020G>A,ctG>ctA):ENSP00000274867(p.Leu340(=))]	NA	NA	NA	NA	NA	NA	NA	NA	NA	NA	LOW	NA	NA	16	16	0	30	24	6	20
Chewer	IOB_33	chr6	80203454	T	G	LCA5	MISSENSE	ENSG00000135338:[ENST00000369846(c.734A>C,aAc>aCc):ENSP00000358861(p.Asn245Thr)]	NA	NA	NA	NA	NA	NA	NA	D	PoD	NA	MODERATE	D	1	69	68	0	78	60	18	23.08
Chewer	IOB_33	chr6	107955884	C	T	SOBP	SILENT	ENSG00000112320:[ENST00000317357(c.1836C>T,ccC>ccT):ENSP00000318900(p.Pro612(=))]	NA	NA	NA	2.17E-04	0.00E+00	NA	NA	NA	NA	NA	LOW	NA	NA	79	78	0	67	61	6	8.96
Chewer	IOB_33	chr6	136590672	G	A	BCLAF1	MISSENSE	ENSG00000029363:[ENST00000530767(c.1603C>T,Cgg>Tgg):ENSP00000436501(p.Arg535Trp)]	NA	NA	NA	4.95E-05	1.82E-04	NA	NA	NA	NA	NA	MODIFIER	NA	NA	194	194	0	288	240	47	16.32
Chewer	IOB_33	chr6	138550984	G	A	KIAA1244	MISSENSE	ENSG00000112379:[ENST00000251691(c.415G>A,Gcg>Acg):ENSP00000251691(p.Ala139Thr)]	NA	NA	2.00E-04	7.49E-05	0.00E+00	2.00E-04	0.00E+00	T	BN	NA	MODERATE	N	1	120	120	0	236	193	43	18.22
Chewer	IOB_33	chr6	138564094	T	A	KIAA1244	SILENT	ENSG00000112379:[ENST00000251691(c.549T>A,atT>atA):ENSP00000251691(p.Ile183(=))]	NA	NA	NA	NA	NA	NA	NA	NA	NA	NA	LOW	NA	1	141	141	0	204	197	7	3.43
Chewer	IOB_33	chr7	2581859	C	T	BRAT1	NCT	ENSG00000106009:[ENST00000467558(n.1206-14G>A)]	NA	NA	2.00E-02	9.02E-03	0.00E+00	2.00E-02	0.00E+00	NA	NA	NA	MODIFIER	NA	NA	109	109	0	189	152	37	19.58
Chewer	IOB_33	chr7	20198273	C	A	MACC1	NONSENSE	ENSG00000183742:[ENST00000332878(c.1711G>T,Gaa>Taa):ENSP00000328410(p.Glu571Ter)]	NA	NA	NA	NA	NA	NA	NA	NA	NA	NA	HIGH	NA	1	223	222	0	374	355	19	5.08
Chewer	IOB_33	chr7	38475953	C	A	AMPH	MISSENSE	ENSG00000078053:[ENST00000325590(c.1053G>T,ttG>ttT):ENSP00000317441(p.Leu351Phe)]	NA	NA	NA	NA	NA	NA	NA	D	PrD	NA	MODERATE	NA	NA	72	72	0	137	131	6	4.38
Chewer	IOB_33	chr7	50132710	G	A	ZPBP	SILENT	ENSG00000042813:[ENST00000046087(c.81C>T,gcC>gcT):ENSP00000046087(p.Ala27(=))]	NA	NA	chr7*50132709*50132733*TGGCGGCCCGAGAGAGCAGGGAGCC*T*rs773457192*NA*NA*NA	NA	NA	NA	NA	NA	NA	NA	LOW	NA	NA	61	61	0	117	103	14	11.97
Chewer	IOB_33	chr7	75187506	C	A	HIP1	NONSENSE	ENSG00000127946:[ENST00000336926(c.1429G>T,Gag>Tag):ENSP00000336747(p.Glu477Ter)]	NA	NA	chr7*75187506*75187506*C*T*rs782771644*NA*NA*NA	NA	NA	NA	NA	NA	NA	NA	HIGH	NA	1	105	105	0	181	174	6	3.31
Chewer	IOB_33	chr7	82584617	A	G	PCLO	SILENT	ENSG00000186472:[ENST00000333891(c.5652T>C,taT>taC):ENSP00000334319(p.Tyr1884(=))]	NA	NA	NA	NA	NA	NA	NA	NA	NA	NA	LOW	NA	NA	138	138	0	192	173	18	9.38
Chewer	IOB_33	chr7	87179778	C	T	ABCB1	INTRONIC-SS-DNR-PRX	ENSG00000085563:[ENST00000265724(c.1224+6G>A)]	NA	NA	NA	NA	NA	NA	NA	NA	NA	NA	LOW	NA	NA	57	57	0	82	53	29	35.37
Chewer	IOB_33	chr7	94294285	A	G	PEG10	MISSENSE	23089:[NM_001184961.1(c.1519A>G,Act>Gct):NP_001171890.1(p.Thr507Ala)]	NA	NA	NA	NA	NA	NA	NA	NA	NA	NA	MODERATE	NA	NA	156	155	0	307	210	96	31.27
Chewer	IOB_33	chr7	96635665	C	A	DLX6	MISSENSE	ENSG00000006377:[ENST00000518156(c.376C>A,Cct>Act):ENSP00000428480(p.Pro126Thr)]	NA	NA	NA	NA	NA	NA	NA	NA	NA	NA	MODIFIER	NA	NA	86	86	0	145	140	5	3.45
Chewer	IOB_33	chr7	96635665	C	A	DLX6-AS1	NCT	ENSG00000231764:[ENST00000605417(n.580+5472G>T)]	NA	NA	NA	NA	NA	NA	NA	NA	NA	NA	MODIFIER	NA	NA	86	86	0	145	140	5	3.45
Chewer	IOB_33	chr7	99030878	G	A	ATP5J2-PTCD1	INTRONIC	ENSG00000248919:[ENST00000413834(c.741+23C>T)]	NA	NA	2.40E-03	3.54E-04	0.00E+00	2.40E-03	0.00E+00	NA	NA	NA	MODIFIER	NA	NA	126	126	0	168	110	58	34.52
Chewer	IOB_33	chr7	99030878	G	A	PTCD1	INTRONIC	ENSG00000106246:[ENST00000555673(c.741+23C>T)]	NA	NA	2.40E-03	3.54E-04	0.00E+00	2.40E-03	0.00E+00	NA	NA	NA	MODIFIER	NA	NA	126	126	0	168	110	58	34.52
Chewer	IOB_33	chr7	104752556	A	G	KMT2E	SILENT	ENSG00000005483:[ENST00000334877(c.4227A>G,ccA>ccG):ENSP00000335599(p.Pro1409(=))]	NA	NA	NA	3.29E-05	0.00E+00	NA	NA	NA	NA	NA	MODIFIER	NA	NA	241	239	0	366	257	109	29.78
Chewer	IOB_33	chr7	104752556	A	G	SRPK2	NCT	ENSG00000135250:[ENST00000493638(n.231-921T>C)]	NA	NA	NA	3.29E-05	0.00E+00	NA	NA	NA	NA	NA	MODIFIER	NA	NA	241	239	0	366	257	109	29.78
Chewer	IOB_33	chr7	147259278	C	A	CNTNAP2	NONSENSE	ENSG00000174469:[ENST00000361727(c.1826C>A,tCa>tAa):ENSP00000354778(p.Ser609Ter)]	NA	NA	NA	NA	NA	NA	NA	NA	NA	NA	HIGH	NA	1	92	92	0	126	121	5	3.97
Chewer	IOB_33	chr7	148992036	C	A	LOC155060	EXONIC-NC	155060:[NR_036573.1(n.2654C>A)]	NA	NA	NA	NA	NA	NA	NA	NA	NA	NA	MODIFIER	NA	NA	86	86	0	122	117	5	4.1
Chewer	IOB_33	chr7	148992036	C	A	RP4-800G7.2	EXONIC-NC	ENSG00000244560:[ENST00000416232(n.2304C>A)]	NA	NA	NA	NA	NA	NA	NA	NA	NA	NA	MODIFIER	NA	NA	86	86	0	122	117	5	4.1
Chewer	IOB_33	chr7	148992036	C	A	ZNF783	INTRONIC	ENSG00000204946:[ENST00000481519(c.*583+44C>A)]	NA	NA	NA	NA	NA	NA	NA	NA	NA	NA	MODIFIER	NA	NA	86	86	0	122	117	5	4.1
Chewer	IOB_33	chr8	17851051	C	A	PCM1	MISSENSE	ENSG00000078674:[ENST00000325083(c.4750C>A,Cca>Aca):ENSP00000327077(p.Pro1584Thr)]	NA	NA	NA	NA	NA	NA	NA	NA	NA	NA	MODERATE	NA	1	118	118	0	102	97	5	4.9
Chewer	IOB_33	chr8	51363326	A	T	SNTG1	NCT	54212:[NR_135798.1(n.949+37A>T)]	NA	NA	NA	NA	NA	NA	NA	NA	NA	NA	MODIFIER	NA	NA	34	33	0	52	47	5	9.62
Chewer	IOB_33	chr8	52370017	C	A	PXDNL	NCT	137902:[XR_242454.1(n.1069+41G>T)]	NA	NA	NA	NA	NA	NA	NA	NA	NA	NA	MODIFIER	NA	NA	63	63	0	89	77	12	13.48
Chewer	IOB_33	chr8	68934332	T	G	PREX2	MISSENSE	ENSG00000046889:[ENST00000288368(c.398T>G,cTt>cGt):ENSP00000288368(p.Leu133Arg)]	NA	NA	NA	NA	NA	NA	NA	NA	NA	NA	MODIFIER	NA	NA	56	56	0	167	153	14	8.38
Chewer	IOB_33	chr8	69380989	G	C	C8orf34	MISSENSE	ENSG00000165084:[ENST00000337103(c.337G>C,Gat>Cat):ENSP00000337174(p.Asp113His)]	NA	NA	NA	NA	NA	NA	NA	D	PrD	NA	MODERATE	D	1	114	114	0	266	242	24	9.02
Chewer	IOB_33	chr8	73480408	C	A	KCNB2	MISSENSE	ENSG00000182674:[ENST00000523207(c.439C>A,Caa>Aaa):ENSP00000430846(p.Gln147Lys)]	NA	NA	NA	NA	NA	NA	NA	D	PoD	NA	MODERATE	U	1	185	183	1	524	460	60	11.45
Chewer	IOB_33	chr8	74938990	C	A	LY96	INTRONIC	ENSG00000154589:[ENST00000284818(c.332-34C>A)]	NA	NA	chr8*74938990*74938990*C*T*rs372115044*NA*NA*NA	NA	NA	NA	NA	NA	NA	NA	MODIFIER	NA	NA	49	49	0	110	105	5	4.55
Chewer	IOB_33	chr8	77618292	C	A	ZFHX4	MISSENSE	ENSG00000091656:[ENST00000050961(c.1969C>A,Cag>Aag):ENSP00000050961(p.Gln657Lys)]	NA	chr8*77618292*77618292*COSM1458259*C*T*1*large intestine*1	NA	NA	NA	NA	NA	D	PrD	NA	MODERATE	U	1	113	112	1	309	291	17	5.5
Chewer	IOB_33	chr8	101157351	C	A	FBXO43	MISSENSE	ENSG00000156509:[ENST00000428847(c.13G>T,Gac>Tac):ENSP00000403293(p.Asp5Tyr)]	NA	NA	chr8*101157351*101157351*C*T*rs1025824704*NA*NA*NA	NA	NA	NA	NA	NA	NA	NA	MODIFIER	NA	NA	107	107	0	210	204	6	2.86
Chewer	IOB_33	chr8	110631186	G	A	SYBU	SILENT	ENSG00000147642:[ENST00000532779(c.108C>T,tgC>tgT):ENSP00000436266(p.Cys36(=))]	NA	NA	NA	NA	NA	NA	NA	NA	NA	NA	LOW	NA	NA	61	61	0	104	99	5	4.81
Chewer	IOB_33	chr8	113243889	C	A	CSMD3	INTRONIC	ENSG00000164796:[ENST00000455883(c.10234-28G>T)]	NA	NA	NA	NA	NA	NA	NA	NA	NA	NA	MODIFIER	NA	NA	57	57	0	61	46	15	24.59
Chewer	IOB_33	chr8	121522299	G	T	MTBP	INTRONIC	ENSG00000172167:[ENST00000305949(c.1979+30G>T)]	NA	NA	NA	NA	NA	NA	NA	NA	NA	NA	MODIFIER	NA	NA	32	32	0	72	65	7	9.72
Chewer	IOB_33	chr8	125711769	C	T	MTSS1	MISSENSE-SS-PRX	ENSG00000170873:[ENST00000522162(c.190G>A,cGt>cAt):ENSP00000429752(p.Arg64His)]	NA	NA	NA	3.32E-05	6.11E-05	NA	NA	NA	NA	NA	LOW	NA	NA	60	60	0	143	103	40	27.97
Chewer	IOB_33	chr8	131092231	C	T	ASAP1	INTRONIC	ENSG00000153317:[ENST00000357668(c.2573-14G>A)]	NA	NA	chr8*131092231*131092231*C*G,T*rs751906732*NA*NA*NA	8.24E-06	0.00E+00	NA	NA	NA	NA	NA	MODIFIER	NA	NA	61	61	0	126	94	31	24.6
Chewer	IOB_33	chr9	5339659	C	A	NA	NCT	101929382:[XR_242528.1(n.64+1162G>T)]	NA	NA	NA	NA	NA	NA	NA	D	PoD	NA	MODERATE	U	0	59	59	0	80	75	5	6.25
Chewer	IOB_33	chr9	5339659	C	A	RLN1	MISSENSE	ENSG00000107018:[ENST00000223858(c.88G>T,Gat>Tat):ENSP00000223858(p.Asp30Tyr)]	NA	NA	NA	NA	NA	NA	NA	D	PoD	NA	MODERATE	U	0	59	59	0	80	75	5	6.25
Chewer	IOB_33	chr9	21239550	C	A	IFNA14	MISSENSE	ENSG00000228083:[ENST00000380222(c.385G>T,Gtg>Ttg):ENSP00000369571(p.Val129Leu)]	NA	NA	chr9*21239550*21239550*C*T*rs747714944*NA*NA*NA	NA	NA	NA	NA	T	BN	NA	MODERATE	N	0	192	191	0	251	244	7	2.79
Chewer	IOB_33	chr9	35090705	T	A	PIGO	INTRONIC	ENSG00000165282:[ENST00000341666(c.2648-36A>T)]	NA	NA	chr9*35090686*35090708*GCCACGTTACAGTCACTTCTTAT*G*rs757991211*NA*NA*NA	NA	NA	NA	NA	NA	NA	NA	MODIFIER	NA	NA	24	24	0	44	34	10	22.73
Chewer	IOB_33	chr9	35092115	C	A	PIGO	MISSENSE	ENSG00000165282:[ENST00000341666(c.1769G>T,tGg>tTg):ENSP00000339382(p.Trp590Leu)]	NA	NA	NA	NA	NA	NA	NA	NA	NA	NA	MODIFIER	NA	NA	124	124	0	154	147	7	4.55
Chewer	IOB_33	chr9	68415302	A	G	NA	INTERGENIC	NA	NA	NA	NA	2.40E-02	2.33E-02	NA	NA	NA	NA	NA	MODIFIER	NA	NA	230	228	1	339	320	17	5.01
Chewer	IOB_33	chr9	74360211	G	A	TMEM2	MISSENSE	ENSG00000135048:[ENST00000377044(c.757C>T,Ccc>Tcc):ENSP00000366243(p.Pro253Ser)]	NA	NA	NA	8.24E-06	0.00E+00	NA	NA	T	BN	NA	MODERATE	NA	NA	109	108	0	148	143	5	3.38
Chewer	IOB_33	chr9	77693280	C	A	NMRK1	STARTLOSS	;ENSG00000106733:[ENST00000376811(c.3G>T,atG>atT):ENSP00000366007(p.Met1?)]	NA	NA	NA	NA	NA	NA	NA	NA	NA	NA	MODIFIER	NA	NA	92	92	0	164	158	6	3.66
Chewer	IOB_33	chr9	85925498	A	T	FRMD3	INTRONIC	257019:[XM_005251882.1(c.486-35T>A)]	NA	NA	NA	NA	NA	NA	NA	NA	NA	NA	MODIFIER	NA	NA	143	142	0	204	173	30	14.71
Chewer	IOB_33	chr9	86322506	G	A	UBQLN1	MISSENSE	ENSG00000135018:[ENST00000257468(c.89C>T,gCc>gTc):ENSP00000257468(p.Ala30Val)]	NA	NA	chr9*86322504*86322519*AGGCAGCGGCCGCGGG*A*rs902893817*NA*NA*NA	NA	NA	NA	NA	T_lc	UN	NA	MODERATE	NA	NA	176	176	0	243	236	7	2.88
Chewer	IOB_33	chr9	90283621	C	T	DAPK1	NCT	ENSG00000196730:[ENST00000497743(n.222+32C>T)]	NA	NA	chr9*90283613*90283640*GAAGTTCTCTACCTGTGTTTATGTGAAA*G*rs755828307*NA*NA*NA|chr9*90283621*90283621*C*G*rs924052406*NA*NA*NA	NA	NA	NA	NA	NA	NA	NA	MODIFIER	NA	NA	40	40	0	84	75	9	10.71
Chewer	IOB_33	chr9	122001101	A	G	BRINP1	INTRONIC	ENSG00000078725:[ENST00000265922(c.580-63T>C)]	NA	NA	NA	NA	NA	NA	NA	NA	NA	NA	MODIFIER	NA	NA	38	38	0	31	25	6	19.35

Patel *et al.* , 2021. Whole-exome sequencing analysis of oral squamous cell carcinoma delineated by tobacco usage habits
Supplementary Table 4. List of somatic single nucleotide variants identified using whole exome sequencing of OSCC patients

Cohort	Sample	Chromosome	Genomic position	Reference allele	Alternate allele	Genesymbol	Variant type	Variant description	OncoMD	COSMIC	dbSNP	ExAC	ExAC_SAS	1000G	1000G_SAS	SIFT	Polyphen2	CONDEL	VEP	LRT	PhastCon7Way	Total reads in Normal	Reference read support in Normal	Alternative read support in Normal	Total reads in Tumor	Reference read support in Tumor	Alternate read support in Tumor	Alternate allele frequency in Tumor
Chewer	IOB_33	chr9	133943511	C	A	LAMC3	NONSENSE	ENSG00000050555:[ENST00000361069(c.2640C>A,tgC>tgA):ENSP00000354360(p.Cys880Ter)]	NA	NA	chr9*133943511*133943511*C*T*rs770821754*NA*NA*NA	NA	NA	NA	NA	NA	NA	NA	HIGH	NA	0	109	109	0	127	122	5	3.94
Chewer	IOB_33	chr9	138378231	G	T	PPP1R26	MISSENSE	ENSG00000196422:[ENST00000356818(c.1875G>T,caG>caT):ENSP00000349274(p.Gln625His)]	NA	NA	NA	NA	NA	NA	NA	T	BN	NA	MODERATE	N	0	169	169	0	153	131	21	13.73
Chewer	IOB_33	chrX	1718383	C	G	AKAP17A	MISSENSE	ENSG00000197976:[ENST00000381261(c.1210C>G,Ccc>Gcc):ENSP00000370660(p.Pro404Ala)]	NA	NA	chrX*1718383*1718383*C*T*rs745730982*NA*NA*NA	NA	NA	NA	NA	T_lc	BN	NA	MODERATE	NA	NA	31	31	0	67	48	19	28.36
Chewer	IOB_33	chrX	1718383	C	G	ASMT	INTRONIC	438:[NM_004043.2(c.-68+3958C>G)]	NA	NA	chrX*1718383*1718383*C*T*rs745730982*NA*NA*NA	NA	NA	NA	NA	T_lc	BN	NA	MODERATE	NA	NA	31	31	0	67	48	19	28.36
Chewer	IOB_33	chrX	20062940	C	T	MAP7D2	INTRONIC	256714:[XM_005274480.1(c.596-322G>A)]	NA	NA	NA	NA	NA	NA	NA	NA	NA	NA	MODIFIER	NA	NA	63	63	0	108	68	40	37.04
Chewer	IOB_33	chrX	23803466	C	T	SAT1	MISSENSE	ENSG00000130066:[ENST00000379254(c.140C>T,gCc>gTc):ENSP00000368556(p.Ala47Val)]	NA	NA	NA	NA	NA	NA	NA	NA	NA	NA	MODIFIER	NA	NA	92	92	0	139	134	5	3.6
Chewer	IOB_33	chrX	41043926	C	G	USP9X	MISSENSE-SS-PRX	8239:[XM_005272676.1(c.3571C>G,Cag>Gag):XP_005272733.1(p.Gln1191Glu)]	NA	NA	NA	NA	NA	NA	NA	NA	NA	NA	MODIFIER	NA	NA	23	23	0	47	34	13	27.66
Chewer	IOB_33	chrX	118827164	C	A	SEPT6	5UTR	ENSG00000125354:[ENST00000343984(c.-96G>T)]	NA	NA	NA	NA	NA	NA	NA	NA	NA	NA	MODIFIER	NA	NA	70	70	0	125	120	5	4
Chewer	IOB_33	chrX	135291430	C	A	FHL1	MISSENSE	ENSG00000022267:[ENST00000345434(c.717C>A,caC>caA):ENSP00000071281(p.His239Gln)]	NA	NA	NA	NA	NA	NA	NA	NA	NA	NA	MODIFIER	NA	NA	23	23	0	47	31	16	34.04
Chewer	IOB_33	chrX	153760303	C	T	G6PD	MISSENSE-SS-PRX	ENSG00000160211:[ENST00000490651(n.778G>A)]	NA	NA	NA	NA	NA	NA	NA	D	PrD	NA	MODERATE	D	1	23	23	0	40	31	9	22.5
Chewer	IOB_33	chrX	154212980	C	T	F8	MISSENSE	ENSG00000185010:[ENST00000423959(c.664G>A,Gta>Ata):ENSP00000409446(p.Val222Ile)]	NA	NA	NA	NA	NA	NA	NA	T	BN	NA	MODERATE	N	0.419	65	65	0	77	59	18	23.38
Non-user	IOB_21	chr1	894556	C	T	NOC2L	NCT	ENSG00000188976:[ENST00000469563(n.42+39G>A)]	NA	NA	NA	NA	NA	NA	NA	NA	NA	NA	MODIFIER	NA	NA	255	253	0	211	169	40	18.96
Non-user	IOB_21	chr1	1417600	C	T	ATAD3B	SILENT	ENSG00000160072:[ENST00000378741(c.93C>T,gcC>gcT):ENSP00000368015(p.Ala31(=))]	NA	NA	.	2.65E-05	1.25E-04	NA	NA	NA	NA	NA	LOW	NA	NA	44	44	0	37	17	20	54.05
Non-user	IOB_21	chr1	1421916	T	C	ATAD3B	INTRONIC-SS-ACR-PRX	ENSG00000160072:[ENST00000308647(c.1090-8T>C)]	NA	NA	NA	2.47E-01	3.32E-01	NA	NA	NA	NA	NA	LOW	NA	NA	157	150	6	174	132	41	23.56
Non-user	IOB_21	chr1	1560444	C	T	MIB2	SILENT	ENSG00000197530:[ENST00000514234(c.325C>T,agC>agT):ENSP00000427680(p.Ser109(=))]	NA	NA	NA	5.85E-05	0.00E+00	NA	NA	NA	NA	NA	MODIFIER	NA	NA	200	200	0	205	125	75	36.59
Non-user	IOB_21	chr1	6638701	C	A	TAS1R1	INTRONIC	80835:[XM_005263500.1(c.824-12C>A)]	NA	NA	NA	NA	NA	NA	NA	NA	NA	NA	MODIFIER	NA	NA	105	105	0	114	108	6	5.26
Non-user	IOB_21	chr1	25554719	G	A	SYF2	MISSENSE	ENSG00000117614:[ENST00000354361(c.140C>T,gCg>gTg):ENSP00000346330(p.Ala47Val)]	NA	NA	1.36E-02	2.34E-02	1.43E-02	1.36E-02	8.20E-03	T	BN	NA	MODERATE	D	1	114	114	0	123	116	6	4.88
Non-user	IOB_21	chr1	40092608	C	A	HEYL	SILENT	ENSG00000163909:[ENST00000372852(c.558G>T,ctG>ctT):ENSP00000361943(p.Leu186(=))]	NA	NA	NA	NA	NA	NA	NA	NA	NA	NA	LOW	NA	NA	146	146	0	141	136	5	3.55
Non-user	IOB_21	chr1	41847348	A	C	FOXO6	MISSENSE	ENSG00000204060:[ENST00000372591(c.475A>C,Acc>Ccc):ENSP00000361672(p.Thr159Pro)]	NA	NA	NA	NA	NA	NA	NA	D_lc	BN	NA	MODERATE	U	1	217	217	0	215	195	19	8.84
Non-user	IOB_21	chr1	45798828	C	T	MUTYH	MISSENSE	ENSG00000132781:[ENST00000354383(c.322G>A,Gtc>Atc):ENSP00000346354(p.Val108Ile)]	NA	NA	NA	NA	NA	NA	NA	NA	NA	NA	MODIFIER	NA	NA	306	303	1	298	276	20	6.71
Non-user	IOB_21	chr1	45798829	C	T	MUTYH	SILENT	ENSG00000132781:[ENST00000355498(c.318G>A,gaG>gaA):ENSP00000347685(p.Glu106(=))]	NA	NA	NA	NA	NA	NA	NA	NA	NA	NA	MODIFIER	NA	NA	307	303	0	299	277	21	7.02
Non-user	IOB_21	chr1	45798832	T	C	MUTYH	SILENT	ENSG00000132781:[ENST00000372110(c.360A>G,tcA>tcG):ENSP00000361182(p.Ser120(=))]	NA	NA	NA	8.24E-06	0.00E+00	NA	NA	NA	NA	NA	LOW	NA	NA	304	304	0	302	281	21	6.95
Non-user	IOB_21	chr1	109816020	C	T	CELSR2	INTRONIC	ENSG00000143126:[ENST00000271332(c.8510-38C>T)]	NA	NA	NA	NA	NA	NA	NA	NA	NA	NA	MODIFIER	NA	NA	329	327	2	313	241	70	22.36
Non-user	IOB_21	chr1	152192623	G	A	FLG-AS1	NCT	ENSG00000237975:[ENST00000593011(n.296+51727G>A)]	NA	NA	2.00E-04	8.24E-05	0.00E+00	2.00E-04	0.00E+00	NA	NA	NA	MODIFIER	NA	NA	237	236	1	304	265	39	12.83
Non-user	IOB_21	chr1	152192623	G	A	HRNR	SILENT	ENSG00000197915:[ENST00000368801(c.1482C>T,caC>caT):ENSP00000357791(p.His494(=))]	NA	NA	2.00E-04	8.24E-05	0.00E+00	2.00E-04	0.00E+00	NA	NA	NA	MODIFIER	NA	NA	237	236	1	304	265	39	12.83
Non-user	IOB_21	chr1	155294980	C	A	RUSC1	5UTR	23623:[NM_001278228.1(c.-19C>A)]	NA	NA	NA	NA	NA	NA	NA	NA	NA	NA	MODIFIER	NA	NA	141	140	0	170	163	5	2.94
Non-user	IOB_21	chr1	157771835	C	T	FCRL1	SILENT	ENSG00000163534:[ENST00000358292(c.756G>A,agG>agA):ENSP00000351039(p.Arg252(=))]	NA	NA	NA	NA	NA	NA	NA	NA	NA	NA	LOW	NA	0	280	277	0	272	203	68	25
Non-user	IOB_21	chr1	213161756	G	A	VASH2	INTRONIC	ENSG00000143494:[ENST00000366967(c.684-98G>A)]	NA	NA	NA	NA	NA	NA	NA	NA	NA	NA	MODIFIER	NA	NA	114	114	0	185	153	32	17.3
Non-user	IOB_21	chr1	226353538	C	A	ACBD3	INTRONIC	ENSG00000182827:[ENST00000366812(c.428+22G>T)]	NA	NA	NA	NA	NA	NA	NA	NA	NA	NA	MODIFIER	NA	NA	236	235	0	255	248	6	2.35
Non-user	IOB_21	chr10	27112102	G	A	ABI1	MISSENSE	ENSG00000136754:[ENST00000346832(c.301C>T,Cgg>Tgg):ENSP00000279599(p.Arg101Trp)]	NA	NA	NA	1.65E-05	6.06E-05	NA	NA	NA	NA	NA	MODIFIER	NA	NA	177	177	0	179	139	38	21.23
Non-user	IOB_21	chr10	56345379	T	G	PCDH15	INTRONIC	ENSG00000150275:[ENST00000409834(c.-91+78553A>C)]	NA	NA	NA	NA	NA	NA	NA	NA	NA	NA	MODIFIER	NA	NA	84	83	0	105	100	5	4.76
Non-user	IOB_21	chr10	56345379	T	G	RP11-257I14.1	NCT	ENSG00000234173:[ENST00000422842(n.198+16944T>G)]	NA	NA	NA	NA	NA	NA	NA	NA	NA	NA	MODIFIER	NA	NA	84	83	0	105	100	5	4.76
Non-user	IOB_21	chr10	88232106	C	A	WAPAL	INTRONIC-SS-ACR-PRX	ENSG00000062650:[ENST00000298767(c.1894-5G>T)]	NA	NA	NA	NA	NA	NA	NA	NA	NA	NA	LOW	NA	NA	85	85	0	103	98	5	4.85
Non-user	IOB_21	chr10	88232106	C	A	WAPL	INTRONIC	23063:[NM_001318328.1(c.1876-5G>T)]	NA	NA	NA	NA	NA	NA	NA	NA	NA	NA	LOW	NA	NA	85	85	0	103	98	5	4.85
Non-user	IOB_21	chr10	116048875	C	T	AFAP1L2	INTRONIC	84632:[XM_005270232.1(c.2515-7345G>A)]	NA	chr10*116048875*116048875*COSM458902*C*T*1*cervix*1	NA	2.50E-05	6.09E-05	NA	NA	NA	NA	NA	LOW	NA	0	236	236	0	252	244	5	1.98
Non-user	IOB_21	chr10	116048875	C	T	VWA2	SILENT	ENSG00000165816:[ENST00000392982(c.1749C>T,ttC>ttT):ENSP00000376708(p.Phe583(=))]	NA	chr10*116048875*116048875*COSM458902*C*T*1*cervix*1	NA	2.50E-05	6.09E-05	NA	NA	NA	NA	NA	LOW	NA	0	236	236	0	252	244	5	1.98
Non-user	IOB_21	chr10	125804167	C	T	CHST15	MISSENSE	ENSG00000182022:[ENST00000346248(c.815G>A,cGc>cAc):ENSP00000333947(p.Arg272His)]	NA	NA	NA	NA	NA	NA	NA	D	PrD	NA	MODERATE	D	1	152	152	0	138	113	25	18.12
Non-user	IOB_21	chr10	127697736	G	A	FANK1	INTRONIC	92565:[XM_005270278.1(c.1005+36G>A)]	NA	NA	2.00E-04	1.65E-05	0.00E+00	2.00E-04	0.00E+00	NA	NA	NA	MODIFIER	NA	NA	146	145	0	187	129	58	31.02
Non-user	IOB_21	chr10	135473026	T	C	CLUHP5	NCT	ENSG00000230468:[ENST00000438421(n.322-65A>G)]	NA	NA	NA	NA	NA	NA	NA	NA	NA	NA	MODIFIER	NA	NA	79	73	4	115	78	37	32.17
Non-user	IOB_21	chr11	294523	C	T	ATHL1	INTRONIC	80162:[XM_005253145.1(c.2018-33C>T)]	NA	NA	NA	2.47E-03	5.02E-03	NA	NA	NA	NA	NA	MODIFIER	NA	NA	56	56	0	99	39	58	58.59
Non-user	IOB_21	chr11	294537	G	A	ATHL1	INTRONIC	ENSG00000142102:[ENST00000409548(c.2021-19G>A)]	NA	NA	NA	1.67E-03	4.73E-03	NA	NA	NA	NA	NA	MODIFIER	NA	NA	64	62	2	96	54	41	42.71
Non-user	IOB_21	chr11	534289	C	T	HRAS	MISSENSE	ENSG00000174775:[ENST00000311189(c.34G>A,Ggc>Agc):ENSP00000309845(p.Gly12Ser)]	NA	chr11*534289*534289*COSM1644659*C*A*1*NS*1|chr11*534289*534289*COSM481*C*A*344*urinary_tract,lung,upper_aerodigestive_tract,thyroid,soft_tissue,skin,NS,central_nervous_system*7,2,5,5,1,9,1,314|chr11*534289*534289*COSM482*C*G*327*thyroid,pituitary,upper_aerodigestive_tract,skin,large_intestine,salivary_gland,central_nervous_system*2,3,2,1,1,4,314|chr11*534289*534289*COSM480*C*T*66*lung,urinary_tract,penis,upper_aerodigestive_tract,thyroid,skin,bone,soft_tissue,large_intestine,cervix,salivary_gland,prostate*2,9,1,26,4,9,1,4,1,[illegible]	chr11*534289*534289*C*A,G,T*rs104894229*NA*NA*NA	NA	NA	NA	NA	NA	NA	NA	MODIFIER	NA	NA	194	194	0	227	156	70	30.84
Non-user	IOB_21	chr11	5461908	G	T	AC104389.28	NCT	ENSG00000167355:[ENST00000420726(n.302+64673C>A)]	NA	NA	NA	NA	NA	NA	NA	NA	NA	NA	MODIFIER	NA	NA	195	193	1	334	297	37	11.08
Non-user	IOB_21	chr11	5461908	G	T	HBE1	INTRONIC	ENSG00000213931:[ENST00000396895(c.-267+64891C>A)]	NA	NA	NA	NA	NA	NA	NA	NA	NA	NA	MODIFIER	NA	NA	195	193	1	334	297	37	11.08
Non-user	IOB_21	chr11	5461908	G	T	HBG2	INTRONIC	ENSG00000196565:[ENST00000380259(c.-564-94779C>A)]	NA	NA	NA	NA	NA	NA	NA	NA	NA	NA	MODIFIER	NA	NA	195	193	1	334	297	37	11.08
Non-user	IOB_21	chr11	5461908	G	T	OR51B5	INTRONIC	282763:[NM_001005567.2(c.-364+64891C>A)]	NA	NA	NA	NA	NA	NA	NA	NA	NA	NA	MODIFIER	NA	NA	195	193	1	334	297	37	11.08
Non-user	IOB_21	chr11	5461908	G	T	OR51I1	SILENT	ENSG00000167359:[ENST00000380211(c.837C>A,tcC>tcA):ENSP00000369559(p.Ser279(=))]	NA	NA	NA	NA	NA	NA	NA	NA	NA	NA	MODIFIER	NA	NA	195	193	1	334	297	37	11.08
Non-user	IOB_21	chr11	24518705	A	G	LUZP2	5UTR	338645:[NM_001252010.1(c.-85A>G)]	NA	NA	NA	NA	NA	NA	NA	NA	NA	NA	MODIFIER	NA	NA	240	238	2	243	159	80	32.92
Non-user	IOB_21	chr11	47611856	C	T	C1QTNF4	SILENT	ENSG00000172247:[ENST00000302514(c.507G>A,ccG>ccA):ENSP00000302274(p.Pro169(=))]	NA	NA	chr11*47611848*47611863*GGCGCGGGCGGCCCGC*G*rs892757307*NA*NA*NA	NA	NA	NA	NA	NA	NA	NA	LOW	NA	NA	93	93	0	62	43	19	30.65
Non-user	IOB_21	chr11	64504422	G	A	RASGRP2	MISSENSE	ENSG00000068831:[ENST00000354024(c.898C>T,Cgc>Tgc):ENSP00000338864(p.Arg300Cys)]	NA	NA	2.00E-04	3.12E-04	6.09E-04	2.00E-04	1.00E-03	NA	NA	NA	MODIFIER	NA	NA	201	201	0	153	110	43	28.1
Non-user	IOB_21	chr11	64812722	G	A	NAALADL1	SILENT-SS-PRX	ENSG00000168060:[ENST00000529274(n.2413C>T)]	NA	NA	chr11*64812722*64812722*G*T*rs371674868*NA*NA*NA	NA	NA	NA	NA	NA	NA	NA	MODIFIER	NA	NA	64	63	0	59	50	9	15.25
Non-user	IOB_21	chr11	67771153	C	T	UNC93B1	MISSENSE	ENSG00000110057:[ENST00000531152(c.361G>A,Gca>Aca):ENSP00000436758(p.Ala121Thr)]	NA	NA	NA	NA	NA	NA	NA	NA	NA	NA	MODIFIER	NA	NA	85	85	0	66	60	6	9.09
Non-user	IOB_21	chr11	95523867	C	T	CEP57	MISSENSE	ENSG00000166037:[ENST00000325486(c.5C>T,gCg>gTg):ENSP00000317487(p.Ala2Val)]	NA	NA	chr11*95523864*95523867*TGGC*T*rs780763546*NA*NA*NA	NA	NA	NA	NA	NA	NA	NA	MODIFIER	NA	NA	192	192	0	262	255	6	2.29
Non-user	IOB_21	chr11	107286994	C	G	CWF19L2	MISSENSE	ENSG00000152404:[ENST00000431778(c.1201G>C,Gaa>Caa):ENSP00000411736(p.Glu401Gln)]	NA	NA	NA	NA	NA	NA	NA	D	BN	NA	MODERATE	N	1	31	31	0	35	28	7	20
Non-user	IOB_21	chr11	108256575	T	G	C11orf65	NCT	ENSG00000166323:[ENST00000526725(n.271+7360A>C)]	NA	NA	NA	NA	NA	NA	NA	NA	NA	NA	MODIFIER	NA	NA	47	47	0	31	22	9	29.03
Non-user	IOB_21	chr11	118764331	C	A	CXCR5	MISSENSE	ENSG00000160683:[ENST00000292174(c.78C>A,aaC>aaA):ENSP00000292174(p.Asn26Lys)]	NA	NA	chr11*118764331*118764331*C*G*rs146150938*NA*NA*NA	NA	NA	NA	NA	D	BN	NA	MODERATE	N	0.886	186	186	0	226	219	6	2.65
Non-user	IOB_21	chr11	120852931	G	A	GRIK4	MISSENSE-SS-PRX	ENSG00000149403:[ENST00000438375(c.2512G>A,Gag>Aag):ENSP00000404063(p.Glu838Lys)]	NA	NA	NA	NA	NA	NA	NA	T	PoD	NA	MODERATE	N	1	270	269	0	332	226	105	31.63
Non-user	IOB_21	chr12	6030296	G	A	ANO2	SILENT	ENSG00000047617:[ENST00000327087(c.432C>T,ctC>ctT):ENSP00000314048(p.Leu144(=))]	NA	NA	2.00E-04	1.41E-04	0.00E+00	2.00E-04	0.00E+00	NA	NA	NA	LOW	NA	NA	125	124	1	135	103	32	23.7
Non-user	IOB_21	chr12	10775178	A	T	STYK1	INTRONIC	ENSG00000060140:[ENST00000075503(c.967+59T>A)]	NA	NA	NA	NA	NA	NA	NA	NA	NA	NA	MODIFIER	NA	NA	287	285	0	383	307	74	19.32
Non-user	IOB_21	chr12	40499110	C	A	SLC2A13	SILENT	ENSG00000151229:[ENST00000280871(c.501G>T,gcG>gcT):ENSP00000280871(p.Ala167(=))]	NA	NA	chr12*40499110*40499110*C*T*rs768558221*NA*NA*NA	NA	NA	NA	NA	NA	NA	NA	LOW	NA	NA	89	87	0	96	89	5	5.21
Non-user	IOB_21	chr12	42632520	G	A	PPHLN1	INTRONIC	ENSG00000134283:[ENST00000549190(c.34+57G>A)]	NA	NA	NA	NA	NA	NA	NA	NA	NA	NA	MODIFIER	NA	NA	64	64	0	58	52	6	10.34
Non-user	IOB_21	chr12	49488074	G	A	DHH	SILENT	ENSG00000139549:[ENST00000266991(c.222C>T,ttC>ttT):ENSP00000266991(p.Phe74(=))]	NA	NA	NA	NA	NA	NA	NA	NA	NA	NA	LOW	NA	0.998	170	169	0	176	134	42	23.86
Non-user	IOB_21	chr12	50474243	G	C	ASIC1	INTRONIC	ENSG00000110881:[ENST00000550558(c.*496-38G>C)]	NA	NA	NA	NA	NA	NA	NA	NA	NA	NA	MODIFIER	NA	NA	68	68	0	71	58	13	18.31
Non-user	IOB_21	chr12	50474482	G	C	ASIC1	MISSENSE	ENSG00000110881:[ENST00000228468(c.1407G>C,caG>caC):ENSP00000228468(p.Gln469His)]	NA	NA	NA	NA	NA	NA	NA	NA	NA	NA	MODIFIER	NA	NA	98	98	0	82	65	17	20.73
Non-user	IOB_21	chr12	53825083	C	A	AMHR2	SILENT	ENSG00000135409:[ENST00000379791(c.1263C>A,ccC>ccA):ENSP00000369117(p.Pro421(=))]	NA	NA	NA	NA	NA	NA	NA	NA	NA	NA	LOW	NA	NA	150	149	0	152	147	5	3.29

Patel *et al.* , 2021. Whole-exome sequencing analysis of oral squamous cell carcinoma delineated by tobacco usage habits
Supplementary Table 4. List of somatic single nucleotide variants identified using whole exome sequencing of OSCC patients

Cohort	Sample	Chromosome	Genomic position	Reference allele	Alternate allele	Genesymbol	Variant type	Variant description	OncoMD	COSMIC	dbSNP	ExAC	ExAC_SAS	1000G	1000G_SAS	SIFT	Polyphen2	CONDEL	VEP	LRT	PhastCon7Way	Total reads in Normal	Reference read support in Normal	Alternative read support in Normal	Total reads in Tumor	Reference read support in Tumor	Alternate read support in Tumor	Alternate allele frequency in Tumor
Non-user	IOB_21	chr12	57081720	G	C	PTGES3	INTRONIC	ENSG00000110958:[ENST00000262033(c.2+62C>G)]	NA	NA	chr12*57081720*57081720*G*A*rs982853344*NA*NA*NA	NA	NA	NA	NA	NA	NA	NA	MODIFIER	NA	NA	238	237	0	254	207	47	18.5
Non-user	IOB_21	chr12	62918293	A	G	MON2	INTRONIC-SS-ACR	ENSG00000061987:[ENST00000280379(c.985-2A>G)]	NA	NA	NA	NA	NA	NA	NA	NA	NA	NA	HIGH	NA	NA	76	76	0	106	89	17	16.04
Non-user	IOB_21	chr12	78516196	C	A	NAV3	MISSENSE	ENSG00000067798:[ENST00000228327(c.4226C>A,aCt>aAt):ENSP00000228327(p.Thr1409Asn)]	NA	chr12*78516194*78516196*COSM1140294*TAC*TTG*1*lung*1	chr12*78516196*78516196*C*T*rs778854137*NA*NA*NA	NA	NA	NA	NA	NA	NA	NA	MODIFIER	NA	NA	88	88	0	99	93	5	5.05
Non-user	IOB_21	chr12	81696013	T	G	LOC102724663	NCT	102724663:[NR_120491.1(n.593+4071T>G)]	NA	NA	2.00E-04	NA	NA	2.00E-04	0.00E+00	NA	NA	NA	MODIFIER	NA	NA	101	100	0	134	105	28	20.9
Non-user	IOB_21	chr12	81696013	T	G	PPFIA2	INTRONIC	ENSG00000139220:[ENST00000541017(c.294-2852A>C)]	NA	NA	2.00E-04	NA	NA	2.00E-04	0.00E+00	NA	NA	NA	MODIFIER	NA	NA	101	100	0	134	105	28	20.9
Non-user	IOB_21	chr12	81696013	T	G	RP11-121G22.3	NCT	ENSG00000257467:[ENST00000551401(n.429+5465T>G)]	NA	NA	2.00E-04	NA	NA	2.00E-04	0.00E+00	NA	NA	NA	MODIFIER	NA	NA	101	100	0	134	105	28	20.9
Non-user	IOB_21	chr12	109882024	C	T	MYO1H	MISSENSE	ENSG00000174527:[ENST00000543960(c.95C>T,tCc>tTc):ENSP00000474025(p.Ser32Phe)]	NA	NA	NA	NA	NA	NA	NA	D	PrD	NA	MODERATE	NA	NA	135	135	0	166	160	5	3.01
Non-user	IOB_21	chr12	112451219	G	T	ERP29	5UTR	ENSG00000089248:[ENST00000261735(c.-51G>T)]	NA	NA	NA	NA	NA	NA	NA	NA	NA	NA	MODIFIER	NA	NA	89	87	0	69	50	19	27.54
Non-user	IOB_21	chr12	122614111	C	A	MLXIP	NCT	ENSG00000175727:[ENST00000539861(n.172-24C>A)]	NA	NA	NA	NA	NA	NA	NA	NA	NA	NA	MODIFIER	NA	NA	294	291	0	289	279	5	1.73
Non-user	IOB_21	chr13	26144947	G	A	ATP8A2	MISSENSE	ENSG00000132932:[ENST00000255283(c.1396G>A,Gtg>Atg):ENSP00000255283(p.Val466Met)]	NA	chr13*26144947*26144947*COSM1184158*G*A*1*large_intestine*1	NA	NA	NA	NA	NA	T	PrD	NA	MODERATE	NA	NA	248	247	1	340	266	73	21.47
Non-user	IOB_21	chr13	31491466	C	A	MEDAG	INTRONIC	ENSG00000102802:[ENST00000380482(c.279-74C>A)]	NA	NA	NA	NA	NA	NA	NA	NA	NA	NA	MODIFIER	NA	NA	181	181	0	197	192	5	2.54
Non-user	IOB_21	chr13	31491466	C	A	TEX26-AS1	NCT	ENSG00000224743:[ENST00000590344(n.187+13472G>T)]	NA	NA	NA	NA	NA	NA	NA	NA	NA	NA	MODIFIER	NA	NA	181	181	0	197	192	5	2.54
Non-user	IOB_21	chr13	84455054	C	A	SLITRK1	MISSENSE	ENSG00000178235:[ENST00000377084(c.589G>T,Gtc>Ttc):ENSP00000366288(p.Val197Phe)]	NA	NA	chr13*84455054*84455054*C*T*rs145616062*NA*NA*NA	NA	NA	NA	NA	D	BN	NA	MODERATE	D	1	95	95	0	120	98	22	18.33
Non-user	IOB_21	chr13	113742041	G	A	MCF2L	SILENT	ENSG00000126217:[ENST00000413354(c.305G>A,acG>acA):ENSP00000407392(p.Thr102(=))]	NA	chr13*113742041*113742041*COSM147719*G*A*1*stomach*1|chr13*113742041*113742041*COSM147718*G*A*1*stomach*1	NA	2.33E-04	0.00E+00	NA	NA	NA	NA	NA	LOW	NA	NA	213	212	1	231	199	31	13.42
Non-user	IOB_21	chr14	20926511	A	G	TMEM55B	EXONIC-NC	ENSG00000165782:[ENST00000557041(n.2959T>C)]	NA	NA	NA	NA	NA	NA	NA	NA	NA	NA	LOW	NA	NA	104	103	0	114	106	6	5.26
Non-user	IOB_21	chr14	23598984	C	G	SLC7A8	MISSENSE	ENSG00000092068:[ENST00000529705(c.823G>C,Gtc>Ctc):ENSP00000434345(p.Val275Leu)]	NA	NA	NA	NA	NA	NA	NA	NA	NA	NA	MODIFIER	NA	NA	126	125	0	111	100	11	9.91
Non-user	IOB_21	chr14	31575541	C	T	HECTD1	INTRONIC	ENSG00000092148:[ENST00000399332(c.7226+311G>A)]	NA	NA	NA	NA	NA	NA	NA	NA	NA	NA	MODIFIER	NA	NA	91	91	0	65	58	7	10.77
Non-user	IOB_21	chr14	51382644	C	G	PYGL	MISSENSE	ENSG00000100504:[ENST00000544180(c.1036G>C,Gtg>Ctg):ENSP00000443787(p.Val346Leu)]	NA	NA	NA	NA	NA	NA	NA	NA	NA	NA	MODIFIER	NA	NA	144	144	0	176	144	29	16.48
Non-user	IOB_21	chr14	51382645	T	C	PYGL	SILENT	ENSG00000100504:[ENST00000216392(c.1137A>G,acA>acG):ENSP00000216392(p.Thr379(=))]	NA	NA	NA	4.94E-05	0.00E+00	NA	NA	NA	NA	NA	LOW	NA	NA	143	143	0	175	143	29	16.57
Non-user	IOB_21	chr14	61789114	G	A	PRKCH	MISSENSE	ENSG00000027075:[ENST00000332981(c.295G>A,Gcc>Acc):ENSP00000329127(p.Ala99Thr)]	NA	NA	NA	NA	NA	NA	NA	NA	NA	NA	MODIFIER	NA	NA	228	227	0	337	329	6	1.78
Non-user	IOB_21	chr14	61789114	G	A	RP11-902B17.1	NCT	ENSG00000247287:[ENST00000554086(n.260+139C>T)]	NA	NA	NA	NA	NA	NA	NA	NA	NA	NA	MODIFIER	NA	NA	228	227	0	337	329	6	1.78
Non-user	IOB_21	chr14	73029088	C	T	RGS6	INTRONIC	9628:[NM_001204421.2(c.1312-335C>T)]	NA	NA	NA	2.49E-05	0.00E+00	NA	NA	NA	NA	NA	MODIFIER	NA	NA	128	128	0	206	199	5	2.43
Non-user	IOB_21	chr14	73029088	C	T	RP3-514A23.2	NCT	ENSG00000258871:[ENST00000555303(n.452-9598G>A)]	NA	NA	NA	2.49E-05	0.00E+00	NA	NA	NA	NA	NA	MODIFIER	NA	NA	128	128	0	206	199	5	2.43
Non-user	IOB_21	chr14	73436383	C	T	ZFYVE1	3UTR	ENSG00000165861:[ENST00000318876(c.*1207G>A)]	NA	NA	NA	NA	NA	NA	NA	NA	NA	NA	MODIFIER	NA	NA	309	309	0	284	278	6	2.11
Non-user	IOB_21	chr14	75143385	C	T	AREL1	SILENT	ENSG00000119682:[ENST00000356357(c.552G>A,ccG>ccA):ENSP00000348714(p.Pro184(=))]	NA	NA	NA	1.66E-05	1.21E-04	NA	NA	NA	NA	NA	LOW	NA	NA	107	105	0	115	108	6	5.22
Non-user	IOB_21	chr14	77945105	G	A	ISM2	INTRONIC	ENSG00000100593:[ENST00000493585(c.628-47C>T)]	NA	NA	NA	NA	NA	NA	NA	NA	NA	NA	MODIFIER	NA	NA	83	82	0	92	85	6	6.52
Non-user	IOB_21	chr14	103452845	C	A	CDC42BPB	MISSENSE	ENSG00000198752:[ENST00000361246(c.669G>T,ttG>ttT):ENSP00000355237(p.Leu223Phe)]	NA	NA	NA	NA	NA	NA	NA	D	PrD	NA	MODERATE	D	1	276	276	0	206	194	12	5.83
Non-user	IOB_21	chr15	22954288	G	A	CYFIP1	MISSENSE	ENSG00000068793:[ENST00000557859(c.113G>A,Gtc>Atc):ENSP00000453917(p.Val39Ile)]	NA	NA	NA	3.32E-05	6.12E-05	NA	NA	NA	NA	NA	MODERATE	NA	NA	95	95	0	119	95	24	20.17
Non-user	IOB_21	chr15	24922056	C	T	NPAP1	NONSENSE	ENSG00000185823:[ENST00000329468(c.1042C>T,Cga>Tga):ENSP00000333735(p.Arg348Ter)]	NA	chr15*24922056*24922056*COSM1707943*C*T*3*skin*3	NA	NA	NA	NA	NA	NA	NA	NA	HIGH	NA	0	161	161	0	157	128	28	17.83
Non-user	IOB_21	chr15	29418705	A	T	FAM189A1	INTRONIC	ENSG00000104059:[ENST00000261275(c.1004-11T>A)]	NA	NA	NA	NA	NA	NA	NA	NA	NA	NA	MODIFIER	NA	NA	77	77	0	74	66	8	10.81
Non-user	IOB_21	chr15	41036269	C	T	RMDN3	MISSENSE	ENSG00000137824:[ENST00000260385(c.886G>A,Gag>Aag):ENSP00000260385(p.Glu296Lys)]	NA	NA	NA	4.14E-05	0.00E+00	NA	NA	NA	NA	NA	MODERATE	NA	0.999	129	129	0	134	127	7	5.22
Non-user	IOB_21	chr15	69011823	G	C	CORO2B	MISSENSE	ENSG00000103647:[ENST00000261861(c.1228G>C,Gaa>Caa):ENSP00000261861(p.Glu410Gln)]	NA	NA	NA	NA	NA	NA	NA	T	BN	NA	MODERATE	U	1	143	142	0	148	126	21	14.19
Non-user	IOB_21	chr15	74426855	T	C	ISLR2	MISSENSE	ENSG00000167178:[ENST00000361742(c.1760T>C,cTc>cCc):ENSP00000355402(p.Leu587Pro)]	NA	NA	NA	NA	NA	NA	NA	D	PoD	NA	MODERATE	N	0.999	257	254	0	262	181	81	30.92
Non-user	IOB_21	chr15	79323852	C	T	RASGRF1	INTRONIC-SS-ACR	ENSG00000058335:[ENST00000560334(n.1014-1G>A)]	NA	NA	NA	NA	NA	NA	NA	NA	NA	NA	HIGH	NA	NA	70	70	0	97	71	24	24.74
Non-user	IOB_21	chr15	83680323	C	A	C15orf40	MISSENSE	ENSG00000169609:[ENST00000508990(c.15G>T,Gca>Tca):ENSP00000454502(p.Ala6Ser)]	NA	NA	chr15*83680323*83680323*C*G*rs758886923*NA*NA*NA	NA	NA	NA	NA	NA	NA	NA	MODIFIER	NA	NA	132	132	0	139	131	5	3.6
Non-user	IOB_21	chr15	83680323	C	A	RP11-382A20.5	NCT	ENSG00000259805:[ENST00000566841(n.734+84954C>A)]	NA	NA	chr15*83680323*83680323*C*G*rs758886923*NA*NA*NA	NA	NA	NA	NA	NA	NA	NA	MODIFIER	NA	NA	132	132	0	139	131	5	3.6
Non-user	IOB_21	chr16	1020849	G	C	LMF1	SILENT	ENSG00000103227:[ENST00000568964(c.115C>G,ctC>ctG):ENSP00000456631(p.Leu39(=))]	NA	NA	chr16*1020849*1020849*G*A*rs767737264*NA*NA*NA	NA	NA	NA	NA	NA	NA	NA	LOW	NA	NA	193	192	0	190	157	33	17.37
Non-user	IOB_21	chr16	1270476	C	T	CACNA1H	MISSENSE	ENSG00000196557:[ENST00000569107(c.2783C>T,Cgc>Tgc):ENSP00000454990(p.Arg929Cys)]	NA	NA	NA	NA	NA	NA	NA	NA	NA	NA	MODERATE	NA	0.984	231	228	0	289	271	10	3.46
Non-user	IOB_21	chr16	1574607	C	T	IFT140	SILENT	ENSG00000187535:[ENST00000426508(c.3087G>A,gcG>gcA):ENSP00000406012(p.Ala1029(=))]	NA	NA	NA	6.80E-05	4.00E-04	NA	NA	NA	NA	NA	MODIFIER	NA	NA	96	96	0	101	73	27	26.73
Non-user	IOB_21	chr16	3632664	A	C	RP11-461A8.1	NCT	ENSG00000261938:[ENST00000573982(n.199-473A>C)]	NA	NA	NA	NA	NA	NA	NA	NA	NA	NA	MODIFIER	NA	NA	200	198	0	158	120	37	23.42
Non-user	IOB_21	chr16	3632664	A	C	SLX4	MISSENSE	ENSG00000188827:[ENST00000294008(c.5184T>G,ttT>ttG):ENSP00000294008(p.Phe1728Leu)]	NA	NA	NA	NA	NA	NA	NA	NA	NA	NA	MODIFIER	NA	NA	200	198	0	158	120	37	23.42
Non-user	IOB_21	chr16	4835793	A	G	SEPT12	NCT	ENSG00000140623:[ENST00000591861(n.515+15T>C)]	NA	NA	NA	3.20E-02	5.66E-02	NA	NA	NA	NA	NA	MODIFIER	NA	NA	32	31	0	46	38	7	15.22
Non-user	IOB_21	chr16	4835794	G	A	SEPT12	INTRONIC	ENSG00000140623:[ENST00000268231(c.374+14C>T)]	NA	NA	NA	2.66E-02	4.45E-02	NA	NA	NA	NA	NA	MODIFIER	NA	NA	33	32	0	46	37	8	17.39
Non-user	IOB_21	chr16	20043922	A	C	GPR139	MISSENSE	ENSG00000180269:[ENST00000570682(c.197T>G,cTc>cGc):ENSP00000458791(p.Leu66Arg)]	NA	NA	NA	NA	NA	NA	NA	NA	NA	NA	MODIFIER	NA	NA	64	64	0	105	77	27	25.71
Non-user	IOB_21	chr16	21979939	A	G	UQCRC2	EXONIC-NC	ENSG00000140740:[ENST00000567810(n.157A>G)]	NA	NA	NA	NA	NA	NA	NA	NA	NA	NA	MODIFIER	NA	NA	53	52	0	54	47	7	12.96
Non-user	IOB_21	chr16	24835066	C	A	TNRC6A	MISSENSE	ENSG00000090905:[ENST00000395799(c.5827C>A,Cca>Aca):ENSP00000379144(p.Pro1943Thr)]	NA	NA	NA	NA	NA	NA	NA	NA	NA	NA	MODIFIER	NA	NA	196	196	0	247	239	5	2.02
Non-user	IOB_21	chr16	33961983	C	T	LINC00273	EXONIC-NC	ENSG00000256642:[ENST00000539813(n.521G>A)]	NA	NA	NA	NA	NA	NA	NA	NA	NA	NA	MODIFIER	NA	NA	66	36	0	82	38	8	9.76
Non-user	IOB_21	chr16	46562259	C	A	ANKRD26P1	EXONIC-NC-SS-PRX	ENSG00000261239:[ENST00000566201(n.980G>T)]	NA	NA	NA	NA	NA	NA	NA	NA	NA	NA	LOW	NA	NA	35	35	0	54	37	17	31.48
Non-user	IOB_21	chr16	69355092	G	A	COG8	NCT	ENSG00000213380:[ENST00000564419(n.30-143C>T)]	NA	NA	.	NA	NA	NA	NA	NA	NA	NA	LOW	NA	NA	107	107	0	130	124	6	4.62
Non-user	IOB_21	chr16	69355092	G	A	VPS4A	SILENT	ENSG00000132612:[ENST00000254950(c.990G>A,tcG>tcA):ENSP00000254950(p.Ser330(=))]	NA	NA	.	NA	NA	NA	NA	NA	NA	NA	LOW	NA	NA	107	107	0	130	124	6	4.62
Non-user	IOB_21	chr16	71798295	C	T	AP1G1	SILENT	ENSG00000166747:[ENST00000566179(c.87G>A,acG>acA):ENSP00000454932(p.Thr29(=))]	NA	chr16*71798295*71798295*COSM1479070*C*T*1*breast*1	chr16*71798295*71798295*C*A,T*rs147509201*NA*NA*NA	1.50E-04	6.18E-05	NA	NA	NA	NA	NA	LOW	NA	NA	48	48	0	62	52	10	16.13
Non-user	IOB_21	chr16	89257845	G	A	CDH15	MISSENSE	ENSG00000129910:[ENST00000289746(c.1340G>A,gGc>gAc):ENSP00000289746(p.Gly447Asp)]	NA	NA	NA	NA	NA	NA	NA	T	BN	NA	MODERATE	D	0.986	191	188	0	236	196	40	16.95
Non-user	IOB_21	chr17	4800559	C	A	MINK1	MISSENSE	ENSG00000141503:[ENST00000453408(c.3916C>A,Cgt>Agt):ENSP00000406487(p.Arg1306Ser)]	NA	NA	chr17*4800559*4800559*C*T*rs200856469*0*0.9998*0.0001997	NA	NA	NA	NA	NA	NA	NA	MODIFIER	NA	NA	214	214	0	223	217	6	2.69
Non-user	IOB_21	chr17	7340279	G	T	RP11-104H15.7	NCT	ENSG00000262880:[ENST00000575310(n.273-4511G>T)]	NA	NA	NA	NA	NA	NA	NA	NA	NA	NA	MODIFIER	NA	NA	309	308	1	246	239	5	2.03
Non-user	IOB_21	chr17	7340279	G	T	RP11-104H15.9	NCT	ENSG00000262624:[ENST00000570444(n.249+315C>A)]	NA	NA	NA	NA	NA	NA	NA	NA	NA	NA	MODIFIER	NA	NA	309	308	1	246	239	5	2.03
Non-user	IOB_21	chr17	7340279	G	T	TMEM102	MISSENSE	ENSG00000181284:[ENST00000323206(c.981G>T,tgG>tgT):ENSP00000315387(p.Trp327Cys)]	NA	NA	NA	NA	NA	NA	NA	NA	NA	NA	MODIFIER	NA	NA	309	308	1	246	239	5	2.03
Non-user	IOB_21	chr17	7578406	C	T	TP53	MISSENSE	ENSG00000141510:[ENST00000269305(c.524G>A,cGc>cAc):ENSP00000269305(p.Arg175His)]	NA	chr17*7578370*7578416*COSM45583*BigSeq*C*2*upper_aerodigestive_tract,liver*1,1|chr17*7578373*7578419*COSM44264*BigSeq*T*1*large_intestine*1|chr17*7578375*7578413*COSM45676*BigSeq*G*1*lung*1|chr17*7578377*7578409*COSM100003*BigSeq*T*1*upper_aerodigestive_tract*1|chr17*7578377*7578409*COSM100005*BigSeq*T*1*upper_aerodigestive_tract*1|chr17*7578377*7578409*COSM100001*BigSeq*T*1*upper_aerodigestive_tract*1|chr17*7578377*7578409*COSM100002*BigSeq*T*1*upper_aerodigestive_tract*1|chr17*7578377*7578409*COSM100004*BigSeq*T*1*upper_aerodigestive_tract*1|chr17*7578389*7578407*COSM44742*GCTCATGGTGGGGGCAGCG*G*2*large_intestine,breast*1,1|chr17*7578390*7578408*COSM44462*CTCATGGTGGGGGCAGCGC*C*2*haematopoietic_and_lymphoi	chr17*7578406*7578406*C*A,T*rs28934578*NA*NA*NA	8.24E-06	0.00E+00	NA	NA	T	PoD	NA	MODERATE	NA	NA	151	151	0	159	142	17	10.69
Non-user	IOB_21	chr17	18219152	G	A	SMCR8	MISSENSE	ENSG00000176994:[ENST00000406438(c.49G>A,Gaa>Aaa):ENSP00000385025(p.Glu17Lys)]	NA	NA	NA	NA	NA	NA	NA	T	BN	NA	MODERATE	D	1	134	133	1	114	79	35	30.7

Patel *et al.* , 2021. Whole-exome sequencing analysis of oral squamous cell carcinoma delineated by tobacco usage habits
Supplementary Table 4. List of somatic single nucleotide variants identified using whole exome sequencing of OSCC patients

Cohort	Sample	Chromosome	Genomic position	Reference allele	Alternate allele	Genesymbol	Variant type	Variant description	OncoMD	COSMIC	dbSNP	ExAC	ExAC_SAS	1000G	1000G_SAS	SIFT	Polyphen2	CONDEL	VEP	LRT	PhastCon7Way	Total reads in Normal	Reference read support in Normal	Alternative read support in Normal	Total reads in Tumor	Reference read support in Tumor	Alternate read support in Tumor	Alternate allele frequency in Tumor
Non-user	IOB_21	chr17	19534015	C	A	CTD-2104P17.1	EXONIC-NC	ENSG00000231625:[ENST00000574288(n.369C>A)]	NA	NA	NA	NA	NA	NA	NA	NA	NA	NA	MODIFIER	NA	NA	272	271	1	277	268	5	1.81
Non-user	IOB_21	chr17	19534015	C	A	NA	EXONIC-NC	101060602:[XR_243609.1(n.1249C>A)]	NA	NA	NA	NA	NA	NA	NA	NA	NA	NA	MODIFIER	NA	NA	272	271	1	277	268	5	1.81
Non-user	IOB_21	chr17	26926006	G	A	RP11-192H23.4	INTRONIC	ENSG00000258472:[ENST00000481916(c.*1195+5063C>T)]	NA	NA	NA	NA	NA	NA	NA	NA	NA	NA	MODIFIER	NA	NA	248	243	1	254	198	55	21.65
Non-user	IOB_21	chr17	26926006	G	A	SPAG5	5UTR	ENSG00000076382:[ENST00000321765(c.-42C>T)]	NA	NA	NA	NA	NA	NA	NA	NA	NA	NA	MODIFIER	NA	NA	248	243	1	254	198	55	21.65
Non-user	IOB_21	chr17	26926006	G	A	SPAG5-AS1	EXONIC-NC	ENSG00000227543:[ENST00000414744(n.199G>A)]	NA	NA	NA	NA	NA	NA	NA	NA	NA	NA	MODIFIER	NA	NA	248	243	1	254	198	55	21.65
Non-user	IOB_21	chr17	27251241	C	A	PHF12	MISSENSE	ENSG00000109118:[ENST00000578900(c.131G>T,aGt>aTt):ENSP00000468478(p.Ser44Ile)]	NA	NA	chr17*27251240*27251241*AC*A*rs376557999*NA*NA*NA	NA	NA	NA	NA	T	BN	NA	MODERATE	D	1	176	176	0	145	138	6	4.14
Non-user	IOB_21	chr17	27425854	C	T	MYO18A	MISSENSE	ENSG00000196535:[ENST00000354329(c.3770G>A,cGg>cAg):ENSP00000346291(p.Arg1257Gln)]	NA	NA	1.60E-03	2.63E-03	9.76E-04	1.60E-03	0.00E+00	NA	NA	NA	MODERATE	NA	1	101	101	0	118	105	13	11.02
Non-user	IOB_21	chr17	36455379	G	C	MRPL45	MISSENSE	ENSG00000174100:[ENST00000312513(c.307G>C,Gag>Cag):ENSP00000308901(p.Glu103Gln)]	NA	NA	NA	NA	NA	NA	NA	D	BN	NA	MODERATE	NA	NA	241	241	0	223	178	42	18.83
Non-user	IOB_21	chr17	37673793	C	T	CDK12	NONSENSE	ENSG00000167258:[ENST00000430627(c.2947C>T,Cga>Tga):ENSP00000407720(p.Arg983Ter)]	NA	chr17*37673793*37673796*COSM1324002*CGAG*C*1*ovary*1	NA	NA	NA	NA	NA	NA	NA	NA	MODIFIER	NA	NA	189	189	0	142	109	33	23.24
Non-user	IOB_21	chr17	45221251	T	C	CDC27	MISSENSE-SS-PRX	ENSG00000004897:[ENST00000574304(c.*324A>G)]	NA	NA	NA	NA	NA	NA	NA	T	PoD	NA	MODERATE	D	1	29	28	1	36	26	9	25
Non-user	IOB_21	chr17	61666670	C	A	DCAF7	MISSENSE	ENSG00000136485:[ENST00000431926(c.493C>A,Ctc>Atc):ENSP00000402312(p.Leu165Ile)]	NA	NA	NA	NA	NA	NA	NA	NA	NA	NA	MODIFIER	NA	NA	84	83	0	100	95	5	5
Non-user	IOB_21	chr17	67096991	G	C	ABCA6	MISSENSE	ENSG00000154262:[ENST00000284425(c.2959C>G,Caa>Gaa):ENSP00000284425(p.Gln987Glu)]	NA	NA	NA	NA	NA	NA	NA	T	BN	NA	MODERATE	N	0	35	35	0	40	31	9	22.5
Non-user	IOB_21	chr17	67252390	G	T	ABCA5	MISSENSE	ENSG00000154265:[ENST00000392676(c.3766C>A,Cca>Aca):ENSP00000376443(p.Pro1256Thr)]	NA	NA	NA	NA	NA	NA	NA	T	BN	NA	MODERATE	N	1	134	134	0	111	96	15	13.51
Non-user	IOB_21	chr17	73735979	A	T	ITGB4	MISSENSE	ENSG00000132470:[ENST00000200181(c.2273A>T,aAg>aTg):ENSP00000200181(p.Lys758Met)]	NA	NA	NA	NA	NA	NA	NA	D	PrD	NA	MODERATE	D	1	144	143	0	149	131	18	12.08
Non-user	IOB_21	chr17	76420132	C	T	DNAH17	MISSENSE	ENSG00000187775:[ENST00000585328(c.13229G>A,cGc>cAc):ENSP00000465516(p.Arg4410His)]	NA	NA	NA	2.47E-05	0.00E+00	NA	NA	NA	NA	NA	MODIFIER	NA	NA	198	198	0	211	205	6	2.84
Non-user	IOB_21	chr17	76420132	C	T	PGS1	3UTR	9489:[XM_005257828.1(c.*3848C>T)]	NA	NA	NA	2.47E-05	0.00E+00	NA	NA	NA	NA	NA	MODIFIER	NA	NA	198	198	0	211	205	6	2.84
Non-user	IOB_21	chr17	79580427	G	A	NPLOC4	SILENT	ENSG00000182446:[ENST00000331134(c.303C>T,ggC>ggT):ENSP00000331487(p.Gly101(=))]	NA	NA	NA	8.29E-06	0.00E+00	NA	NA	NA	NA	NA	MODIFIER	NA	NA	121	121	0	116	89	26	22.41
Non-user	IOB_21	chr17	80040493	G	A	FASN	SILENT	ENSG00000169710:[ENST00000306749(c.5829C>T,acC>acT):ENSP00000304592(p.Thr1943(=))]	NA	NA	NA	NA	NA	NA	NA	NA	NA	NA	MODIFIER	NA	NA	179	178	1	173	143	28	16.18
Non-user	IOB_21	chr18	8113700	C	A	PTPRM	SILENT	ENSG00000173482:[ENST00000400053(c.1887C>A,ccC>ccA):ENSP00000382927(p.Pro629(=))]	NA	NA	NA	NA	NA	NA	NA	NA	NA	NA	LOW	NA	NA	271	270	0	265	227	38	14.34
Non-user	IOB_21	chr18	44236988	G	A	LOXHD1	SILENT	ENSG00000167210:[ENST00000441551(c.9C>T,ccC>ccT):ENSP00000387621(p.Pro3(=))]	NA	NA	NA	NA	NA	NA	NA	NA	NA	NA	LOW	NA	NA	93	93	0	120	114	5	4.17
Non-user	IOB_21	chr19	2733273	C	T	AC006538.4	INTRONIC	ENSG00000267001:[ENST00000586572(c.210+3773G>A)]	NA	NA	NA	2.62E-05	0.00E+00	NA	NA	T	BN	NA	MODERATE	N	0	290	290	0	266	259	5	1.88
Non-user	IOB_21	chr19	2733273	C	T	SLC39A3	MISSENSE	ENSG00000141873:[ENST00000269740(c.421G>A,Gcg>Acg):ENSP00000269740(p.Ala141Thr)]	NA	NA	NA	2.62E-05	0.00E+00	NA	NA	T	BN	NA	MODERATE	N	0	290	290	0	266	259	5	1.88
Non-user	IOB_21	chr19	2832394	C	A	ZNF554	MISSENSE	ENSG00000172006:[ENST00000317243(c.347C>A,aCg>aAg):ENSP00000321132(p.Thr116Lys)]	NA	NA	chr19*2832394*2832394*C*T*rs577420438*0*0.9998*0.0001997	NA	NA	NA	NA	T	BN	NA	MODERATE	NA	NA	142	142	0	117	112	5	4.27
Non-user	IOB_21	chr19	3207159	C	T	NCLN	INTRONIC	ENSG00000125912:[ENST00000587740(c.861-37C>T)]	NA	NA	2.00E-04	7.44E-05	0.00E+00	2.00E-04	0.00E+00	NA	NA	NA	MODIFIER	NA	NA	135	135	0	157	119	38	24.2
Non-user	IOB_21	chr19	3624039	C	A	CACTIN	MISSENSE	ENSG00000105298:[ENST00000248420(c.289G>T,Ggc>Tgc):ENSP00000248420(p.Gly97Cys)]	NA	NA	NA	NA	NA	NA	NA	D_lc	BN	NA	MODERATE	U	0	173	173	0	160	147	13	8.12
Non-user	IOB_21	chr19	6381051	C	T	GTF2F1	SILENT-SS-PRX	ENSG00000125651:[ENST00000594213(n.462G>A)]	NA	NA	NA	NA	NA	NA	NA	NA	NA	NA	LOW	NA	NA	146	146	0	126	105	21	16.67
Non-user	IOB_21	chr19	9069083	C	A	MUC16	MISSENSE	ENSG00000181143:[ENST00000397910(c.18363G>T,atG>atT):ENSP00000381008(p.Met6121Ile)]	NA	NA	NA	NA	NA	NA	NA	NA	BN	NA	MODERATE	NA	NA	130	130	0	189	161	27	14.29
Non-user	IOB_21	chr19	9965152	G	A	OLFM2	MISSENSE	ENSG00000105088:[ENST00000590841(c.841C>T,Cgg>Tgg):ENSP00000464877(p.Arg281Trp)]	NA	chr19*9965152*9965152*COSM1259906*G*A*1*oesophagus*1	NA	NA	NA	NA	NA	D	PoD	NA	MODERATE	D	0.983	248	248	0	261	255	6	2.3
Non-user	IOB_21	chr19	11526582	C	A	RGL3	NCT	ENSG00000205517:[ENST00000453604(n.461+706G>T)]	NA	NA	NA	NA	NA	NA	NA	NA	NA	NA	MODIFIER	NA	NA	47	47	0	38	28	10	26.32
Non-user	IOB_21	chr19	13249097	C	A	NACC1	MISSENSE	ENSG00000160877:[ENST00000585663(c.162C>A,gaC>gaA):ENSP00000466017(p.Asp54Glu)]	NA	NA	chr19*13249097*13249097*C*T*rs142534277*NA*NA*NA	NA	NA	NA	NA	T_lc	BN	NA	MODERATE	D	0.002	325	323	1	337	329	7	2.08
Non-user	IOB_21	chr19	13947053	C	A	MIR24-2	EXONIC-NC	ENSG00000267519:[ENST00000587762(n.51G>T)]	NA	NA	NA	NA	NA	NA	NA	NA	NA	NA	MODIFIER	NA	NA	106	105	0	100	82	18	18
Non-user	IOB_21	chr19	15131363	C	G	CCDC105	MISSENSE	ENSG00000160994:[ENST00000292574(c.766C>G,Ctg>Gtg):ENSP00000292574(p.Leu256Val)]	NA	NA	NA	NA	NA	NA	NA	D	PrD	NA	MODERATE	N	1	36	36	0	47	36	11	23.4
Non-user	IOB_21	chr19	15131363	C	G	SLC1A6	INTRONIC	ENSG00000105143:[ENST00000595863(c.-8+2353G>C)]	NA	NA	NA	NA	NA	NA	NA	D	PrD	NA	MODERATE	N	1	36	36	0	47	36	11	23.4
Non-user	IOB_21	chr19	15221190	C	A	SYDE1	INTRONIC	ENSG00000105137:[ENST00000342784(c.1075+31C>A)]	NA	NA	NA	NA	NA	NA	NA	NA	NA	NA	MODIFIER	NA	NA	94	93	0	104	98	5	4.81
Non-user	IOB_21	chr19	16793243	G	A	TMEM38A	MISSENSE	ENSG00000072954:[ENST00000187762(c.478G>A,Gcc>Acc):ENSP00000187762(p.Ala160Thr)]	NA	NA	NA	1.65E-05	0.00E+00	NA	NA	T	BN	NA	MODERATE	N	1	171	169	0	163	131	32	19.63
Non-user	IOB_21	chr19	17608081	G	C	CTD-3131K8.2	NCT	ENSG00000269439:[ENST00000596643(n.548-6075C>G)]	NA	NA	NA	NA	NA	NA	NA	NA	NA	NA	MODIFIER	NA	NA	123	123	0	132	124	7	5.3
Non-user	IOB_21	chr19	17608081	G	C	SLC27A1	SILENT	ENSG00000130304:[ENST00000598424(c.477G>C,ggG>ggC):ENSP00000472313(p.Gly159(=))]	NA	NA	NA	NA	NA	NA	NA	NA	NA	NA	MODIFIER	NA	NA	123	123	0	132	124	7	5.3
Non-user	IOB_21	chr19	17608085	A	C	CTD-3131K8.2	NCT	ENSG00000269439:[ENST00000596643(n.548-6079T>G)]	NA	NA	NA	NA	NA	NA	NA	NA	NA	NA	MODIFIER	NA	NA	118	117	0	124	115	7	5.65
Non-user	IOB_21	chr19	17608085	A	C	SLC27A1	MISSENSE	ENSG00000130304:[ENST00000252595(c.1018A>C,Atc>Ctc):ENSP00000252595(p.Ile340Leu)]	NA	NA	NA	NA	NA	NA	NA	NA	NA	NA	MODIFIER	NA	NA	118	117	0	124	115	7	5.65
Non-user	IOB_21	chr19	17608105	G	C	CTD-3131K8.2	NCT	ENSG00000269439:[ENST00000596643(n.548-6099C>G)]	NA	NA	NA	NA	NA	NA	NA	T	BN	NA	MODERATE	N	0.996	114	114	0	120	112	8	6.67
Non-user	IOB_21	chr19	17608105	G	C	SLC27A1	MISSENSE	ENSG00000130304:[ENST00000252595(c.1038G>C,aaG>aaC):ENSP00000252595(p.Lys346Asn)]	NA	NA	NA	NA	NA	NA	NA	T	BN	NA	MODERATE	N	0.996	114	114	0	120	112	8	6.67
Non-user	IOB_21	chr19	18653725	C	T	FKBP8	MISSENSE	ENSG00000105701:[ENST00000453489(c.35G>A,gGg>gAg):ENSP00000388891(p.Gly12Glu)]	NA	NA	NA	NA	NA	NA	NA	NA	NA	NA	MODIFIER	NA	NA	72	72	0	44	32	12	27.27
Non-user	IOB_21	chr19	19369537	G	A	AC138430.4	EXONIC-NC	ENSG00000267629:[ENST00000586064(n.1743C>T)]	NA	NA	NA	NA	NA	NA	NA	NA	NA	NA	LOW	NA	0.1	335	334	0	318	280	38	11.95
Non-user	IOB_21	chr19	19369537	G	A	HAPLN4	SILENT	ENSG00000187664:[ENST00000291481(c.612C>T,gaC>gaT):ENSP00000291481(p.Asp204(=))]	NA	NA	NA	NA	NA	NA	NA	NA	NA	NA	LOW	NA	0.1	335	334	0	318	280	38	11.95
Non-user	IOB_21	chr19	20230133	G	C	ZNF90	MISSENSE	ENSG00000213988:[ENST00000418063(c.1770G>C,caG>caC):ENSP00000410466(p.Gln590His)]	NA	NA	NA	NA	NA	NA	NA	NA	NA	NA	MODIFIER	NA	NA	167	165	0	188	156	32	17.02
Non-user	IOB_21	chr19	21992032	C	T	ZNF43	SILENT	ENSG00000198521:[ENST00000594012(c.789G>A,aaG>aaA):ENSP00000469578(p.Lys263(=))]	NA	NA	chr19*21992032*21992032*C*G*rs1041839753*NA*NA*NA	NA	NA	NA	NA	NA	NA	NA	LOW	NA	NA	63	61	1	78	71	6	7.69
Non-user	IOB_21	chr19	36556873	G	A	WDR62	MISSENSE	ENSG00000075702:[ENST00000270301(c.346G>A,Gct>Act):ENSP00000270301(p.Ala116Thr)]	NA	NA	NA	NA	NA	NA	NA	T	BN	NA	MODERATE	NA	NA	179	179	0	148	142	6	4.05
Non-user	IOB_21	chr19	42730013	C	A	ZNF526	MISSENSE	ENSG00000167625:[ENST00000301215(c.1458C>A,caC>caA):ENSP00000301215(p.His486Gln)]	NA	NA	chr19*42730013*42730013*C*T*rs145696319*NA*NA*NA	NA	NA	NA	NA	D	PrD	NA	MODERATE	N	0	266	266	0	282	275	5	1.77
Non-user	IOB_21	chr19	44983568	C	A	ZNF180	MISSENSE	ENSG00000167384:[ENST00000221327(c.266G>T,tGg>tTg):ENSP00000221327(p.Trp89Leu)]	NA	NA	NA	NA	NA	NA	NA	D_lc	PoD	NA	MODERATE	NA	NA	46	46	0	71	66	5	7.04
Non-user	IOB_21	chr19	45202546	C	A	CEACAM16	INTRONIC-SS-DNR-PRX	ENSG00000213892:[ENST00000587331(c.-97+7C>A)]	NA	NA	NA	NA	NA	NA	NA	NA	NA	NA	MODIFIER	NA	NA	174	174	0	179	170	6	3.35
Non-user	IOB_21	chr19	45202546	C	A	CTB-171A8.1	NCT	ENSG00000266903:[ENST00000590796(n.315-99G>T)]	NA	NA	NA	NA	NA	NA	NA	NA	NA	NA	MODIFIER	NA	NA	174	174	0	179	170	6	3.35
Non-user	IOB_21	chr19	45297532	G	A	CBLC	SILENT	ENSG00000142273:[ENST00000341505(c.1218G>A,ccG>ccA):ENSP00000340250(p.Pro406(=))]	NA	chr19*45297532*45297532*COSM108150*G*A*1*skin*1	NA	4.95E-05	1.82E-04	NA	NA	NA	NA	NA	LOW	NA	NA	77	77	0	106	100	5	4.72
Non-user	IOB_21	chr19	48805978	G	A	CCDC114	MISSENSE	ENSG00000105479:[ENST00000315396(c.1102C>T,Cgg>Tgg):ENSP00000318429(p.Arg368Trp)]	NA	chr19*48805978*48805978*COSM1247420*G*A*2*oesophagus,central_nervous_system*1,1|chr19*48805978*48805978*COSM1247421*G*A*2*oesophagus,central_nervous_system*1,1	NA	1.70E-05	6.18E-05	NA	NA	NA	NA	NA	MODERATE	NA	0	127	125	0	96	90	6	6.25
Non-user	IOB_21	chr19	48994772	C	T	LMTK3	MISSENSE	ENSG00000142235:[ENST00000600059(c.4117G>A,Gtc>Atc):ENSP00000472020(p.Val1373Ile)]	NA	NA	NA	NA	NA	NA	NA	T_lc	PoD	NA	MODERATE	N	0.99	21	21	0	26	20	5	19.23
Non-user	IOB_21	chr19	49316469	C	T	HSD17B14	MISSENSE	ENSG00000087076:[ENST00000599157(c.704G>A,cGg>cAg):ENSP00000472746(p.Arg235Gln)]	NA	NA	chr19*49316469*49316469*C*A,T*rs141811939*NA*NA*NA	NA	NA	NA	NA	T	BN	NA	MODERATE	N	0	39	39	0	40	32	8	20
Non-user	IOB_21	chr19	54625970	C	A	AC012314.8	EXONIC-NC	ENSG00000237017:[ENST00000452097(n.2753G>T)]	NA	NA	NA	NA	NA	NA	NA	NA	NA	NA	LOW	NA	NA	122	122	0	131	126	5	3.82
Non-user	IOB_21	chr19	54625970	C	A	PRPF31	SILENT	ENSG00000105618:[ENST00000321030(c.417C>A,gtC>gtA):ENSP00000324122(p.Val139(=))]	NA	NA	NA	NA	NA	NA	NA	NA	NA	NA	LOW	NA	NA	122	122	0	131	126	5	3.82
Non-user	IOB_21	chr19	55053854	C	T	KIR3DX1	EXONIC-NC-SS-PRX	ENSG00000104970:[ENST00000471931(n.1165C>T)]	NA	NA	NA	NA	NA	NA	NA	NA	NA	NA	LOW	NA	NA	85	85	0	63	54	9	14.29
Non-user	IOB_21	chr19	55512257	C	G	NLRP2	MISSENSE	ENSG00000022556:[ENST00000391721(c.3108C>G,ttC>ttG):ENSP00000375601(p.Phe1036Leu)]	NA	NA	chr19*55512257*55512257*C*T*rs778692647*NA*NA*NA	NA	NA	NA	NA	D	PrD	NA	MODERATE	NA	NA	100	99	0	91	70	21	23.08
Non-user	IOB_21	chr19	57334280	C	A	PEG3	INTRONIC	ENSG00000198300:[ENST00000326441(c.482-76G>T)]	NA	NA	NA	NA	NA	NA	NA	NA	NA	NA	MODIFIER	NA	NA	130	130	0	185	178	5	2.7
Non-user	IOB_21	chr19	57334280	C	A	ZIM2	INTRONIC	ENSG00000269699:[ENST00000221722(c.107-76G>T)]	NA	NA	NA	NA	NA	NA	NA	NA	NA	NA	MODIFIER	NA	NA	130	130	0	185	178	5	2.7
Non-user	IOB_21	chr2	3504354	A	G	ADI1	INTRONIC-SS-DNR	ENSG00000182551:[ENST00000327435(c.420+231T>C)]	NA	NA	NA	NA	NA	NA	NA	NA	NA	NA	MODIFIER	NA	NA	102	102	0	99	72	27	27.27
Non-user	IOB_21	chr2	25180812	C	A	DNAJC27	MISSENSE	ENSG00000115137:[ENST00000534855(c.59G>T,gGt>gTt):ENSP00000440086(p.Gly20Val)]	NA	NA	chr2*25180812*25180812*C*T*rs760914183*NA*NA*NA	NA	NA	NA	NA	NA	NA	NA	MODIFIER	NA	NA	263	263	0	283	276	6	2.12
Non-user	IOB_21	chr2	26101043	C	T	ASXL2	MISSENSE	ENSG00000143970:[ENST00000435504(c.49G>A,Gcc>Acc):ENSP00000391447(p.Ala17Thr)]	NA	chr2*26101043*26101043*COSM243089*C*A*1*prostate*1	NA	NA	NA	NA	NA	NA	NA	NA	MODIFIER	NA	NA	373	370	1	389	381	6	1.54
Non-user	IOB_21	chr2	36970323	C	T	VIT	NONSENSE	ENSG00000205221:[ENST00000404084(c.133C>T,Caa>Taa):ENSP00000384154(p.Gln45Ter)]	NA	NA	NA	NA	NA	NA	NA	NA	NA	NA	MODIFIER	NA	NA	184	184	0	256	188	66	25.78
Non-user	IOB_21	chr2	46211674	C	G	PRKCE	INTRONIC	5581:[XM_005264430.1(c.244-16C>G)]	NA	NA	NA	NA	NA	NA	NA	NA	NA	NA	MODIFIER	NA	NA	128	127	1	149	120	28	18.79
Non-user	IOB_21	chr2	54859798	C	A	SPTBN1	SILENT	ENSG00000115306:[ENST00000356805(c.3660C>A,gcC>gcA):ENSP00000349259(p.Ala1220(=))]	NA	NA	NA	8.24E-06	0.00E+00	NA	NA	NA	NA	NA	LOW	NA	NA	138	138	0	210	203	5	2.38
Non-user	IOB_21	chr2	70933564	C	T	ADD2	MISSENSE	ENSG00000075340:[ENST00000430656(c.25G>A,Gcg>Acg):ENSP00000398112(p.Ala9Thr)]	NA	NA	NA	NA	NA	NA	NA	NA	NA	NA	MODIFIER	NA	NA	198	198	0	259	202	56	21.62
Non-user	IOB_21	chr2	73053032	C	G	EXOC6B	MISSENSE	ENSG00000144036:[ENST00000272427(c.8G>C,cGg>cCg):ENSP00000272427(p.Arg3Pro)]	NA	NA	chr2*73053032*73053032*C*A*rs373687089*NA*NA*NA	NA	NA	NA	NA	NA	NA	NA	MODIFIER	NA	NA	294	294	0	322	272	49	15.22
Non-user	IOB_21	chr2	73227490	C	T	SFXN5	NCT	94097:[XR_244971.1(n.1145+70G>A)]	NA	NA	NA	NA	NA	NA	NA	NA	NA	NA	MODIFIER	NA	NA	106	106	0	110	94	16	14.55

Patel *et al.* , 2021. Whole-exome sequencing analysis of oral squamous cell carcinoma delineated by tobacco usage habits
Supplementary Table 4. List of somatic single nucleotide variants identified using whole exome sequencing of OSCC patients

Cohort	Sample	Chromosome	Genomic position	Reference allele	Alternate allele	Genesymbol	Variant type	Variant description	OncoMD	COSMIC	dbSNP	ExAC	ExAC_SAS	1000G	1000G_SAS	SIFT	Polyphen2	CONDEL	VEP	LRT	PhastCon7Way	Total reads in Normal	Reference read support in Normal	Alternative read support in Normal	Total reads in Tumor	Reference read support in Tumor	Alternate read support in Tumor	Alternate allele frequency in Tumor
Non-user	IOB_21	chr2	74005385	G	C	DUSP11	INTRONIC	ENSG00000144048:[ENST00000377706(c.177+43C>G)]	NA	NA	chr2*74005385*74005385*G*A*rs987982718*NA*NA*NA	NA	NA	NA	NA	NA	NA	NA	MODIFIER	NA	NA	68	68	0	80	59	21	26.25
Non-user	IOB_21	chr2	80136770	G	A	CTNNA2	SILENT	ENSG00000066032:[ENST00000361291(c.1005G>A,ccG>ccA):ENSP00000355398(p.Pro335(=))]	NA	NA	NA	NA	NA	NA	NA	NA	NA	NA	LOW	NA	NA	279	276	1	276	229	45	16.3
Non-user	IOB_21	chr2	98275431	C	A	ACTR1B	SILENT	ENSG00000115073:[ENST00000289228(c.351G>T,ccG>ccT):ENSP00000289228(p.Pro117(=))]	NA	chr2*98275431*98275431*COSM172056*C*T*1*large_intestine*1	chr2*98275431*98275431*C*T*rs900815095*NA*NA*NA	NA	NA	NA	NA	NA	NA	NA	MODIFIER	NA	NA	128	127	0	129	123	6	4.65
Non-user	IOB_21	chr2	101869538	G	A	CNOT11	MISSENSE	ENSG00000158435:[ENST00000289382(c.112G>A,Ggc>Agc):ENSP00000289382(p.Gly38Ser)]	NA	NA	NA	NA	NA	NA	NA	T_lc	BN	NA	MODERATE	N	0.001	67	67	0	69	53	15	21.74
Non-user	IOB_21	chr2	111556538	T	C	ACOXL	INTRONIC	ENSG00000153093:[ENST00000340561(c.461-53T>C)]	NA	NA	NA	NA	NA	NA	NA	NA	NA	NA	MODIFIER	NA	NA	159	158	0	205	162	43	20.98
Non-user	IOB_21	chr2	151331453	G	A	RND3	MISSENSE	ENSG00000115963:[ENST00000263895(c.284C>T,tCg>tTg):ENSP00000263895(p.Ser95Leu)]	NA	chr2*151331453*151331453*COSM175652*G*A*1*large_intestine*1	NA	3.31E-05	0.00E+00	NA	NA	NA	NA	NA	MODIFIER	NA	NA	95	95	0	164	157	5	3.05
Non-user	IOB_21	chr2	171627312	A	T	ERICH2	MISSENSE	285141:[NM_001290031.1(c.121A>T,Acc>Tcc):NP_001276960.1(p.Thr41Ser)]	NA	NA	NA	NA	NA	NA	NA	NA	NA	NA	MODERATE	NA	NA	72	71	0	103	80	22	21.36
Non-user	IOB_21	chr2	171627312	A	T	LOC101926913	EXONIC-NC	101926913:[XR_241349.1(n.20T>A)]	NA	NA	NA	NA	NA	NA	NA	NA	NA	NA	MODERATE	NA	NA	72	71	0	103	80	22	21.36
Non-user	IOB_21	chr2	172965559	C	T	DLX2	SILENT	ENSG00000115844:[ENST00000234198(c.699G>A,ccG>ccA):ENSP00000234198(p.Pro233(=))]	NA	NA	NA	NA	NA	NA	NA	NA	NA	NA	LOW	NA	NA	191	189	1	173	138	35	20.23
Non-user	IOB_21	chr2	196620863	G	A	DNAH7	SILENT	ENSG00000118997:[ENST00000438565(c.60C>T,ttC>ttT):ENSP00000409732(p.Phe20(=))]	NA	NA	NA	NA	NA	NA	NA	NA	NA	NA	LOW	NA	1	97	96	0	112	96	16	14.29
Non-user	IOB_21	chr2	198175347	G	A	ANKRD44	INTRONIC	ENSG00000065413:[ENST00000443014(c.-49+502C>T)]	NA	NA	chr2*198175347*198175347*G*C*rs546671160*NA*NA*NA	NA	NA	NA	NA	NA	NA	NA	MODIFIER	NA	NA	268	266	1	315	308	6	1.9
Non-user	IOB_21	chr2	220366202	C	A	AC053503.11	NCT	ENSG00000227432:[ENST00000429882(n.182+15216G>T)]	NA	NA	NA	NA	NA	NA	NA	D	BN	NA	MODERATE	NA	NA	171	170	0	199	192	6	3.02
Non-user	IOB_21	chr2	220366202	C	A	GMPPA	MISSENSE	ENSG00000144591:[ENST00000313597(c.143C>A,cCt>cAt):ENSP00000315925(p.Pro48His)]	NA	NA	NA	NA	NA	NA	NA	D	BN	NA	MODERATE	NA	NA	171	170	0	199	192	6	3.02
Non-user	IOB_21	chr2	223066851	G	A	PAX3	MISSENSE	ENSG00000135903:[ENST00000350526(c.1232C>T,gCg>gTg):ENSP00000343052(p.Ala411Val)]	NA	chr2*223066851*223066851*COSM720557*G*T*1*lung*1	NA	2.13E-05	1.43E-04	NA	NA	D_lc	PoD	NA	MODERATE	D	1	86	86	0	143	138	5	3.5
Non-user	IOB_21	chr2	233756026	C	A	NGEF	INTRONIC	ENSG00000066248:[ENST00000416114(c.441+42G>T)]	NA	NA	NA	NA	NA	NA	NA	NA	NA	NA	MODIFIER	NA	NA	96	96	0	79	74	5	6.33
Non-user	IOB_21	chr2	242689581	C	A	D2HGDH	MISSENSE	ENSG00000180902:[ENST00000321264(c.869C>A,gCt>gAt):ENSP00000315351(p.Ala290Asp)]	NA	NA	NA	NA	NA	NA	NA	NA	NA	NA	MODIFIER	NA	NA	141	140	0	161	156	5	3.11
Non-user	IOB_21	chr20	3842018	C	T	MAVS	MISSENSE	ENSG00000088888:[ENST00000428216(c.332C>T,tCa>tTa):ENSP00000401980(p.Ser111Leu)]	NA	NA	chr20*3842018*3842018*C*G*rs771288502*NA*NA*NA	NA	NA	NA	NA	NA	NA	NA	MODIFIER	NA	NA	203	201	0	270	217	51	18.89
Non-user	IOB_21	chr20	23066476	G	C	CD93	SILENT	ENSG00000125810:[ENST00000246006(c.354C>G,ggC>ggG):ENSP00000246006(p.Gly118(=))]	NA	chr20*23066476*23066476*COSM443545*G*A*1*breast*1	chr20*23066476*23066476*G*A,C,T*rs764193328*NA*NA*NA	4.53E-03	4.59E-04	NA	NA	NA	NA	NA	LOW	NA	NA	50	49	1	71	65	5	7.04
Non-user	IOB_21	chr20	25604601	G	T	NANP	5UTR	ENSG00000170191:[ENST00000304788(c.-17C>A)]	NA	NA	NA	NA	NA	NA	NA	NA	NA	NA	MODIFIER	NA	NA	127	127	0	122	115	5	4.1
Non-user	IOB_21	chr20	36500361	G	A	CTNNBL1	SILENT	ENSG00000132792:[ENST00000373473(c.1077G>A,ccG>ccA):ENSP00000362572(p.Pro359(=))]	NA	NA	2.00E-04	1.15E-04	6.06E-05	2.00E-04	0.00E+00	NA	NA	NA	MODIFIER	NA	NA	200	200	0	278	234	42	15.11
Non-user	IOB_21	chr20	42143364	C	T	L3MBTL1	SILENT	ENSG00000185513:[ENST00000418998(c.384C>T,taC>taT):ENSP00000398516(p.Tyr128(=))]	NA	NA	NA	NA	NA	NA	NA	NA	NA	NA	MODIFIER	NA	NA	100	99	1	154	148	5	3.25
Non-user	IOB_21	chr20	42935338	G	A	FITM2	MISSENSE	ENSG00000197296:[ENST00000396825(c.716C>T,cCg>cTg):ENSP00000380037(p.Pro239Leu)]	NA	NA	NA	4.12E-05	0.00E+00	NA	NA	T	BN	NA	MODERATE	N	1	71	71	0	119	108	10	8.4
Non-user	IOB_21	chr20	47253084	C	T	PREX1	MISSENSE	ENSG00000124126:[ENST00000371941(c.4084G>A,Gac>Aac):ENSP00000361009(p.Asp1362Asn)]	NA	NA	chr20*47253084*47253084*C*G,T*rs371681138*NA*NA*NA	3.69E-05	0.00E+00	NA	NA	D	PoD	NA	MODERATE	NA	NA	201	200	1	256	186	69	26.95
Non-user	IOB_21	chr20	47260948	A	G	PREX1	SILENT-SS-PRX	ENSG00000124126:[ENST00000482556(c.1565T>C,tcT>tcC):ENSP00000434632(p.Ser522(=))]	NA	NA	NA	8.42E-06	0.00E+00	NA	NA	NA	NA	NA	LOW	NA	NA	180	180	0	175	119	53	30.29
Non-user	IOB_21	chr20	48098892	G	A	KCNB1	SILENT	ENSG00000158445:[ENST00000371741(c.126C>T,caC>caT):ENSP00000360806(p.His42(=))]	NA	NA	NA	NA	NA	NA	NA	NA	NA	NA	LOW	NA	1	185	185	0	213	146	64	30.05
Non-user	IOB_21	chr20	48129552	C	T	PTGIS	NCT	ENSG00000124212:[ENST00000478971(n.1027+65G>A)]	NA	NA	NA	NA	NA	NA	NA	NA	NA	NA	MODIFIER	NA	NA	73	73	0	101	67	34	33.66
Non-user	IOB_21	chr20	62174794	A	G	SRMS	MISSENSE	ENSG00000125508:[ENST00000217188(c.518T>C,aTg>aCg):ENSP00000217188(p.Met173Thr)]	NA	NA	NA	1.67E-05	0.00E+00	NA	NA	T	BN	NA	MODERATE	N	0	114	114	0	143	112	30	20.98
Non-user	IOB_21	chr21	11059861	T	G	BAGE2	INTRONIC	85319:[NM_182482.2(c.117-1538A>C)]	NA	NA	NA	1.09E-01	1.65E-01	NA	NA	NA	NA	NA	MODIFIER	NA	NA	50	46	0	60	53	6	10
Non-user	IOB_21	chr21	43519013	C	A	UMODL1	INTRONIC	ENSG00000177398:[ENST00000400421(c.439-23C>A)]	NA	NA	NA	NA	NA	NA	NA	NA	NA	NA	MODIFIER	NA	NA	77	77	0	34	28	6	17.65
Non-user	IOB_21	chr21	47532464	C	A	COL6A2	MISSENSE	ENSG00000142173:[ENST00000300527(c.687C>A,gaC>gaA):ENSP00000300527(p.Asp229Glu)]	NA	NA	chr21*47532464*47532464*C*T*rs143496259*NA*NA*NA	NA	NA	NA	NA	NA	NA	NA	MODIFIER	NA	NA	256	256	0	164	159	5	3.05
Non-user	IOB_21	chr21	47851746	C	G	PCNT	MISSENSE	ENSG00000160299:[ENST00000359568(c.8368C>G,Ctg>Gtg):ENSP00000352572(p.Leu2790Val)]	NA	NA	chr21*47851746*47851746*C*T*rs758836598*NA*NA*NA	NA	NA	NA	NA	NA	NA	NA	MODERATE	NA	0	164	164	0	136	104	31	22.79
Non-user	IOB_21	chr22	16157940	C	T	AP000525.10	NCT	ENSG00000232775:[ENST00000609679(n.264+598C>T)]	NA	NA	NA	1.07E-01	3.80E-01	NA	NA	NA	NA	NA	MODIFIER	NA	NA	23	22	1	19	10	9	47.37
Non-user	IOB_21	chr22	16157940	C	T	AP000525.9	NCT	ENSG00000206195:[ENST00000413768(n.809+4457G>A)]	NA	NA	NA	1.07E-01	3.80E-01	NA	NA	NA	NA	NA	MODIFIER	NA	NA	23	22	1	19	10	9	47.37
Non-user	IOB_21	chr22	16157940	C	T	DUXAP8	NCT	503637:[NR_122113.1(n.814+4457G>A)]	NA	NA	NA	1.07E-01	3.80E-01	NA	NA	NA	NA	NA	MODIFIER	NA	NA	23	22	1	19	10	9	47.37
Non-user	IOB_21	chr22	21409399	C	T	AC002472.13	SILENT	ENSG00000187905:[ENST00000342608(c.861C>T,atC>atT):ENSP00000341179(p.Ile287(=))]	NA	NA	NA	NA	NA	NA	NA	NA	NA	NA	LOW	NA	NA	233	233	0	207	201	6	2.9
Non-user	IOB_21	chr22	21409399	C	T	LRRC74B	EXONIC-NC	400891:[NR_027006.1(n.2155C>T)]	NA	NA	NA	NA	NA	NA	NA	NA	NA	NA	LOW	NA	NA	233	233	0	207	201	6	2.9
Non-user	IOB_21	chr22	26688852	C	T	SEZ6L	MISSENSE	ENSG00000100095:[ENST00000248933(c.575C>T,gCg>gTg):ENSP00000248933(p.Ala192Val)]	NA	NA	chr22*26688852*26688852*C*A,G,T*rs138721720*NA*NA*NA	8.36E-06	0.00E+00	NA	NA	NA	NA	NA	MODIFIER	NA	NA	285	284	1	297	288	7	2.36
Non-user	IOB_21	chr22	26895106	G	C	TFIP11	SILENT	ENSG00000100109:[ENST00000405938(c.1293C>G,ctC>ctG):ENSP00000384297(p.Leu431(=))]	NA	NA	NA	NA	NA	NA	NA	NA	NA	NA	LOW	NA	NA	139	138	0	132	91	40	30.3
Non-user	IOB_21	chr22	30405105	C	T	CTA-85E5.10	EXONIC-NC	ENSG00000227117:[ENST00000429350(n.528G>A)]	NA	NA	NA	NA	NA	NA	NA	NA	NA	NA	MODERATE	NA	1	199	199	0	213	205	6	2.82
Non-user	IOB_21	chr22	30405105	C	T	HORMAD2-AS1	EXONIC-NC	101929664:[NR_110541.1(n.778G>A)]	NA	NA	NA	NA	NA	NA	NA	NA	NA	NA	MODERATE	NA	1	199	199	0	213	205	6	2.82
Non-user	IOB_21	chr22	30405105	C	T	MTMR3	MISSENSE	ENSG00000100330:[ENST00000333027(c.1108C>T,Cca>Tca):ENSP00000331649(p.Pro370Ser)]	NA	NA	NA	NA	NA	NA	NA	NA	NA	NA	MODERATE	NA	1	199	199	0	213	205	6	2.82
Non-user	IOB_21	chr22	32546976	T	A	C22orf42	INTRONIC	150297:[XM_005261369.1(c.535+3A>T)]	NA	NA	NA	NA	NA	NA	NA	NA	NA	NA	LOW	NA	NA	100	100	0	120	104	15	12.5
Non-user	IOB_21	chr22	38934430	G	A	DMC1	INTRONIC	ENSG00000100206:[ENST00000216024(c.661-16C>T)]	NA	NA	NA	8.27E-06	6.06E-05	NA	NA	NA	NA	NA	MODIFIER	NA	NA	148	148	0	130	105	25	19.23
Non-user	IOB_21	chr22	43687140	G	A	SCUBE1	SILENT	ENSG00000159307:[ENST00000290460(c.396C>T,tgC>tgT):ENSP00000290460(p.Cys132(=))]	NA	NA	NA	2.51E-05	1.23E-04	NA	NA	NA	NA	NA	LOW	NA	0.012	108	106	2	116	92	24	20.69
Non-user	IOB_21	chr3	14939592	G	A	FGD5	MISSENSE	ENSG00000154783:[ENST00000543601(c.2333G>A,cGt>cAt):ENSP00000445949(p.Arg778His)]	NA	NA	NA	2.49E-05	6.06E-05	NA	NA	NA	NA	NA	MODIFIER	NA	NA	198	197	0	175	142	33	18.86
Non-user	IOB_21	chr3	49011254	G	A	ARIH2	INTRONIC-SS-DNR-PRX	ENSG00000177479:[ENST00000356401(c.888+5G>A)]	NA	NA	NA	NA	NA	NA	NA	NA	NA	NA	LOW	NA	NA	125	124	0	101	80	21	20.79
Non-user	IOB_21	chr3	50379263	C	T	ZMYND10	MISSENSE	ENSG00000004838:[ENST00000360165(c.1084G>A,Gac>Aac):ENSP00000353289(p.Asp362Asn)]	NA	NA	NA	NA	NA	NA	NA	T	BN	NA	MODERATE	D	1	163	162	1	111	83	28	25.23
Non-user	IOB_21	chr3	50379263	C	T	ZMYND10-AS1	NCT	ENSG00000235058:[ENST00000440013(n.123+604C>T)]	NA	NA	NA	NA	NA	NA	NA	T	BN	NA	MODERATE	D	1	163	162	1	111	83	28	25.23
Non-user	IOB_21	chr3	51378735	C	G	DOCK3	MISSENSE	ENSG00000088538:[ENST00000266037(c.3834C>G,ttC>ttG):ENSP00000266037(p.Phe1278Leu)]	NA	NA	chr3*51378735*51378735*C*T*rs1045080246*NA*NA*NA	NA	NA	NA	NA	NA	NA	NA	MODERATE	NA	1	87	87	0	53	46	7	13.21
Non-user	IOB_21	chr3	100074025	G	C	NIT2	SILENT	ENSG00000114021:[ENST00000394140(c.744G>C,ctG>ctC):ENSP00000377696(p.Leu248(=))]	NA	NA	NA	NA	NA	NA	NA	NA	NA	NA	MODIFIER	NA	NA	189	188	0	186	156	29	15.59
Non-user	IOB_21	chr3	100473479	G	A	ABI3BP	MISSENSE	ENSG00000154175:[ENST00000284322(c.2774C>T,aCa>aTa):ENSP00000284322(p.Thr925Ile)]	NA	NA	NA	NA	NA	NA	NA	NA	NA	NA	MODERATE	NA	0.999	201	201	0	277	209	65	23.47
Non-user	IOB_21	chr3	119545583	G	A	GSK3B	EXONIC-NC	ENSG00000082701:[ENST00000473886(n.403C>T)]	NA	NA	NA	NA	NA	NA	NA	NA	NA	NA	MODIFIER	NA	NA	53	53	0	82	60	21	25.61
Non-user	IOB_21	chr3	119545583	G	A	NR1I2	INTRONIC	8856:[XM_005247866.1(c.996-6240G>A)]	NA	NA	NA	NA	NA	NA	NA	NA	NA	NA	MODIFIER	NA	NA	53	53	0	82	60	21	25.61
Non-user	IOB_21	chr3	124456602	C	T	UMPS	SILENT	ENSG00000114491:[ENST00000536109(c.222C>T,caC>caT):ENSP00000443577(p.His74(=))]	NA	NA	NA	6.59E-05	6.06E-05	NA	NA	NA	NA	NA	MODIFIER	NA	NA	150	150	0	191	161	29	15.18
Non-user	IOB_21	chr3	126185092	C	T	ZXDC	SILENT	ENSG00000070476:[ENST00000336332(c.1347G>A,ccG>ccA):ENSP00000337694(p.Pro449(=))]	NA	NA	2.60E-03	4.31E-04	1.21E-04	2.60E-03	0.00E+00	NA	NA	NA	LOW	NA	NA	85	84	0	78	62	15	19.23
Non-user	IOB_21	chr3	195596413	C	T	TNK2	INTRONIC-SS-ACR	ENSG00000061938:EXONIC-NC[ENST00000420716(n.149G>A)]	NA	NA	NA	NA	NA	NA	NA	NA	NA	NA	MODIFIER	NA	NA	63	63	0	79	56	22	27.85
Non-user	IOB_21	chr3	195686868	G	A	SDHAP1	EXONIC-NC	ENSG00000185485:[ENST00000354937(n.2060C>T)]	NA	NA	NA	NA	NA	NA	NA	NA	NA	NA	MODIFIER	NA	NA	38	36	2	45	30	14	31.11
Non-user	IOB_21	chr3	195686899	C	T	SDHAP1	EXONIC-NC	ENSG00000185485:[ENST00000427149(n.426G>A)]	NA	NA	NA	NA	NA	NA	NA	NA	NA	NA	MODIFIER	NA	NA	40	38	2	49	32	17	34.69
Non-user	IOB_21	chr4	1730128	C	T	TACC3	SILENT	ENSG00000013810:[ENST00000313288(c.999C>T,agC>agT):ENSP00000326550(p.Ser333(=))]	NA	NA	NA	NA	NA	NA	NA	NA	NA	NA	LOW	NA	0	225	224	1	153	148	5	3.27
Non-user	IOB_21	chr4	2826410	A	C	SH3BP2	MISSENSE	ENSG00000087266:[ENST00000356331(c.310A>C,Aag>Cag):ENSP00000348685(p.Lys104Gln)]	NA	NA	NA	NA	NA	NA	NA	NA	NA	NA	MODIFIER	NA	NA	266	260	1	251	221	30	11.95
Non-user	IOB_21	chr4	3255110	C	T	MSANTD1	MISSENSE	ENSG00000188981:[ENST00000507492(c.458C>T,tCc>tTc):ENSP00000423547(p.Ser153Phe)]	NA	NA	NA	NA	NA	NA	NA	D	PrD	NA	MODERATE	N	1	441	441	0	419	319	97	23.15
Non-user	IOB_21	chr4	42153796	G	A	BEND4	MISSENSE	ENSG00000188848:[ENST00000502486(c.365C>T,gCg>gTg):ENSP00000421169(p.Ala122Val)]	NA	NA	NA	NA	NA	NA	NA	T_lc	BN	NA	MODERATE	U	0.066	104	103	0	105	94	11	10.48
Non-user	IOB_21	chr4	126411458	G	A	FAT4	MISSENSE	ENSG00000196159:[ENST00000394329(c.13481G>A,gGc>gAc):ENSP00000377862(p.Gly4494Asp)]	NA	NA	chr4*126411458*126411458*G*T*rs747737170*NA*NA*NA	NA	NA	NA	NA	T	BN	NA	MODERATE	U	0.004	161	161	0	221	202	16	7.24
Non-user	IOB_21	chr4	169176945	C	T	DDX60	SILENT	ENSG00000137628:[ENST00000393743(c.3474G>A,agG>agA):ENSP00000377344(p.Arg1158(=))]	NA	NA	chr4*169176945*169176945*C*G*rs760912611*NA*NA*NA	NA	NA	NA	NA	NA	NA	NA	LOW	NA	0	141	141	0	116	102	13	11.21
Non-user	IOB_21	chr4	177649058	C	A	VEGFC	MISSENSE	ENSG00000150630:[ENST00000280193(c.426G>T,aaG>aaT):ENSP00000280193(p.Lys142Asn)]	NA	chr4*177649058*177649058*COSM586624*C*A*1*lung*1	chr4*177649058*177649058*C*T*rs867744061*NA*NA*NA	NA	NA	NA	NA	D	PrD	NA	MODERATE	D	1	112	112	0	181	175	5	2.76
Non-user	IOB_21	chr4	183837946	C	A	DCTD	MISSENSE	ENSG00000129187:[ENST00000507543(c.68G>T,gGa>gTa):ENSP00000422386(p.Gly23Val)]	NA	NA	NA	NA	NA	NA	NA	NA	NA	NA	MODIFIER	NA	NA	103	103	0	103	93	9	8.74
Non-user	IOB_21	chr5	1245958	C	T	SLC6A18	INTRONIC-SS-ACR-PRX	ENSG00000164363:[ENST00000324642(c.1657-5C>T)]	NA	NA	NA	1.12E-05	8.02E-05	NA	NA	NA	NA	NA	LOW	NA	NA	121	120	0	104	77	27	25.96
Non-user	IOB_21	chr5	55422911	G	C	ANKRD55	MISSENSE	ENSG00000164512:[ENST00000341048(c.635C>G,tCc>tGc):ENSP00000342295(p.Ser212Cys)]	NA	NA	NA	NA	NA	NA	NA	D	PrD	NA	MODERATE	D	1	92	92	0	106	82	22	20.75
Non-user	IOB_21	chr5	55422911	G	C	NA	EXONIC-NC	101928419:[XR_241794.1(n.768G>C)]	NA	NA	NA	NA	NA	NA	NA	D	PrD	NA	MODERATE	D	1	92	92	0	106	82	22	20.75
Non-user	IOB_21	chr5	64592990	G	A	ADAMTS6	NCT	ENSG00000049192:[ENST00000470597(n.1391+2822C>T)]	NA	NA	NA	NA	NA	NA	NA	NA	NA	NA	MODIFIER	NA	NA	80	80	0	96	82	14	14.58
Non-user	IOB_21	chr5	89971159	G	A	ADGRV1	EXONIC-NC	84059:[NR_003149.1(n.5306G>A)]	NA	NA	NA	NA	NA	NA	NA	NA	NA	NA	MODIFIER	NA	NA	93	93	0	129	98	31	24.03

Patel *et al.* , 2021. Whole-exome sequencing analysis of oral squamous cell carcinoma delineated by tobacco usage habits
Supplementary Table 4. List of somatic single nucleotide variants identified using whole exome sequencing of OSCC patients

Cohort	Sample	Chromosome	Genomic position	Reference allele	Alternate allele	Genesymbol	Variant type	Variant description	OncoMD	COSMIC	dbSNP	ExAC	ExAC_SAS	1000G	1000G_SAS	SIFT	Polyphen2	CONDEL	VEP	LRT	PhastCon7Way	Total reads in Normal	Reference read support in Normal	Alternative read support in Normal	Total reads in Tumor	Reference read support in Tumor	Alternate read support in Tumor	Alternate allele frequency in Tumor
Non-user	IOB_21	chr5	89971159	G	A	GPR98	MISSENSE	ENSG00000164199:[ENST00000405460(c.5210G>A,gGa>gAa):ENSP00000384582(p.Gly1737Glu)]	NA	NA	NA	NA	NA	NA	NA	NA	NA	NA	MODIFIER	NA	NA	93	93	0	129	98	31	24.03
Non-user	IOB_21	chr5	137426524	G	A	WNT8A	MISSENSE	ENSG00000061492:[ENST00000361560(c.818G>A,cGt>cAt):ENSP00000354726(p.Arg273His)]	NA	chr5*137426524*137426524*COSM1061226*G*A*1*endometrium*1	NA	1.66E-05	0.00E+00	NA	NA	D	PrD	NA	MODERATE	D	1	170	169	0	188	150	38	20.21
Non-user	IOB_21	chr5	138860899	G	A	TMEM173	MISSENSE	ENSG00000184584:[ENST00000330794(c.256C>T,Cgg>Tgg):ENSP00000331288(p.Arg86Trp)]	NA	NA	NA	NA	NA	NA	NA	NA	NA	NA	MODIFIER	NA	NA	164	163	0	159	154	5	3.14
Non-user	IOB_21	chr5	140683289	G	A	SLC25A2	SILENT	ENSG00000120329:[ENST00000239451(c.144C>T,acC>acT):ENSP00000239451(p.Thr48(=))]	NA	NA	NA	1.65E-05	1.21E-04	NA	NA	NA	NA	NA	LOW	NA	NA	264	263	0	298	215	83	27.85
Non-user	IOB_21	chr5	141488599	G	C	NDFIP1	MISSENSE	ENSG00000131507:[ENST00000253814(c.60G>C,caG>caC):ENSP00000253814(p.Gln20His)]	NA	NA	chr5*141488599*141488599*G*A*rs566265381*1*0.997*0.002995	NA	NA	NA	NA	T	BN	NA	MODERATE	N	1	59	59	0	72	59	12	16.67
Non-user	IOB_21	chr5	150481135	C	A	ANXA6	INTRONIC	ENSG00000197043:[ENST00000356496(c.1945-67G>T)]	NA	NA	NA	NA	NA	NA	NA	NA	NA	NA	MODIFIER	NA	NA	154	153	0	144	137	5	3.47
Non-user	IOB_21	chr5	176831589	G	A	F12	SILENT	ENSG00000131187:[ENST00000253496(c.711C>T,ccC>ccT):ENSP00000253496(p.Pro237(=))]	NA	NA	9.19E-03	2.86E-02	2.95E-03	9.19E-03	1.00E-03	NA	NA	NA	MODIFIER	NA	NA	249	248	0	149	143	5	3.36
Non-user	IOB_21	chr5	176831589	G	A	GRK6	INTRONIC	ENSG00000198055:[ENST00000502598(c.-45+1062G>A)]	NA	NA	9.19E-03	2.86E-02	2.95E-03	9.19E-03	1.00E-03	NA	NA	NA	MODIFIER	NA	NA	249	248	0	149	143	5	3.36
Non-user	IOB_21	chr6	73830181	A	G	KCNQ5	INTRONIC	ENSG00000185760:[ENST00000342056(c.1126-25A>G)]	NA	NA	NA	NA	NA	NA	NA	NA	NA	NA	MODIFIER	NA	NA	189	188	0	219	173	44	20.09
Non-user	IOB_21	chr6	75834065	C	T	COL12A1	SILENT	ENSG00000111799:[ENST00000322507(c.6630G>A,ctG>ctA):ENSP00000325146(p.Leu2210(=))]	NA	chr6*75834065*75834065*COSM1445934*C*A*1*large intestine*1	NA	NA	NA	NA	NA	NA	NA	NA	LOW	NA	NA	80	79	0	115	93	21	18.26
Non-user	IOB_21	chr6	110679207	C	A	METTL24	MISSENSE	ENSG00000053328:[ENST00000338882(c.269G>T,gGg>gTg):ENSP00000344071(p.Gly90Val)]	NA	NA	chr6*110679204*110679210*GTCCCGC*G*rs952340854*NA*NA*NA	NA	NA	NA	NA	T	BN	NA	MODERATE	N	0.275	29	29	0	34	29	5	14.71
Non-user	IOB_21	chr6	110679208	C	A	METTL24	MISSENSE	ENSG00000053328:[ENST00000338882(c.268G>T,Ggg>Tgg):ENSP00000344071(p.Gly90Trp)]	NA	NA	chr6*110679204*110679210*GTCCCGC*G*rs952340854*NA*NA*NA	NA	NA	NA	NA	T	BN	NA	MODERATE	N	0.264	29	29	0	34	29	5	14.71
Non-user	IOB_21	chr6	143074731	C	T	HIVEP2	MISSENSE	ENSG00000010818:[ENST00000012134(c.6854G>A,cGa>cAa):ENSP00000012134(p.Arg2285Gln)]	NA	NA	NA	NA	NA	NA	NA	NA	NA	NA	MODIFIER	NA	NA	126	125	0	171	161	10	5.85
Non-user	IOB_21	chr6	143074731	C	T	RP1-67K17.3	EXONIC-NC	ENSG00000233138:[ENST00000437067(n.184C>T)]	NA	NA	NA	NA	NA	NA	NA	NA	NA	NA	MODIFIER	NA	NA	126	125	0	171	161	10	5.85
Non-user	IOB_21	chr6	168947818	G	A	SMOC2	SILENT	ENSG00000112562:[ENST00000356284(c.531G>A,gcG>gcA):ENSP00000348630(p.Ala177(=))]	NA	NA	NA	2.47E-05	6.06E-05	NA	NA	NA	NA	NA	LOW	NA	NA	60	58	0	80	68	12	15
Non-user	IOB_21	chr7	21469853	G	A	SP4	MISSENSE	ENSG00000105866:[ENST00000222584(c.1070G>A,cGc>cAc):ENSP00000222584(p.Arg357His)]	NA	NA	NA	NA	NA	NA	NA	T	BN	NA	MODERATE	D	1	109	109	0	136	108	28	20.59
Non-user	IOB_21	chr7	38467626	C	A	AMPH	NCT	ENSG00000078053:[ENST00000467580(n.536+724G>T)]	NA	NA	NA	NA	NA	NA	NA	NA	NA	NA	MODIFIER	NA	NA	166	165	1	196	189	5	2.55
Non-user	IOB_21	chr7	47408570	C	T	TNS3	MISSENSE	ENSG00000136205:[ENST00000355730(c.953G>A,cGa>cAa):ENSP00000347968(p.Arg318Gln)]	NA	chr7*47408570*47408570*COSM1230049*C*T*1*large intestine*1	NA	NA	NA	NA	NA	NA	NA	NA	MODERATE	NA	0.033	282	282	0	269	263	5	1.86
Non-user	IOB_21	chr7	50135813	G	A	C7orf72	SILENT	ENSG00000164500:[ENST00000297001(c.132G>A,gtG>gtA):ENSP00000297001(p.Val44(=))]	NA	NA	NA	NA	NA	NA	NA	NA	NA	NA	LOW	NA	NA	240	239	0	305	246	59	19.34
Non-user	IOB_21	chr7	50135813	G	A	ZPBP	INTRONIC	ENSG00000042813:[ENST00000450231(c.11-6508C>T)]	NA	NA	NA	NA	NA	NA	NA	NA	NA	NA	LOW	NA	NA	240	239	0	305	246	59	19.34
Non-user	IOB_21	chr7	75629698	C	T	STYXL1	INTRONIC	ENSG00000127952:[ENST00000360591(c.*325+510G>A)]	NA	NA	NA	NA	NA	NA	NA	NA	NA	NA	MODIFIER	NA	NA	127	126	0	93	88	5	5.38
Non-user	IOB_21	chr7	94268633	G	A	SGCE	INTRONIC	ENSG00000127990:[ENST00000265735(c.110-9480C>T)]	NA	NA	NA	NA	NA	NA	NA	NA	NA	NA	MODIFIER	NA	NA	274	274	0	289	234	55	19.03
Non-user	IOB_21	chr7	128033186	C	T	IMPDH1	INTRONIC	ENSG00000106348:[ENST00000338791(c.1779-104G>A)]	NA	NA	NA	NA	NA	NA	NA	NA	NA	NA	MODIFIER	NA	NA	126	126	0	133	107	25	18.8
Non-user	IOB_21	chr7	128483348	C	T	FLNC	SILENT	ENSG00000128591:[ENST00000325888(c.2616C>T,gcC>gcT):ENSP00000327145(p.Ala872(=))]	NA	NA	chr7*128483348*128483348*C*A,T*rs769947935*NA*NA*NA	NA	NA	NA	NA	NA	NA	NA	LOW	NA	NA	111	111	0	104	78	26	25
Non-user	IOB_21	chr7	141730254	C	A	MGAM	MISSENSE	ENSG00000257335:[ENST00000475668(c.1314C>A,aaC>aaA):ENSP00000417515(p.Asn438Lys)]	NA	NA	chr7*141730254*141730254*C*T*rs367803242*NA*NA*NA	NA	NA	NA	NA	T	BN	NA	MODERATE	N	0	236	236	0	242	235	5	2.07
Non-user	IOB_21	chr7	142231950	G	A	TRBV10-1	INTRONIC	ENSG00000211717:[ENST00000390364(c.49+24C>T)]	NA	NA	NA	8.45E-06	0.00E+00	NA	NA	NA	NA	NA	MODIFIER	NA	NA	59	59	0	57	50	7	12.28
Non-user	IOB_21	chr7	142231955	T	A	TRBV10-1	INTRONIC	ENSG00000211717:[ENST00000390364(c.49+19A>T)]	NA	NA	NA	8.44E-06	0.00E+00	NA	NA	NA	NA	NA	MODIFIER	NA	NA	60	60	0	55	48	7	12.73
Non-user	IOB_21	chr7	142334809	G	T	TRBV20-1	MISSENSE	ENSG00000211747:[ENST00000390394(c.231G>T,gaG>gaT):ENSP00000374917(p.Glu77Asp)]	NA	NA	NA	NA	NA	NA	NA	T	BN	NA	MODERATE	NA	NA	229	229	0	274	267	6	2.19
Non-user	IOB_21	chr7	150775723	G	A	FASTK	MISSENSE	ENSG00000164896:[ENST00000482571(c.670C>T,Cgg>Tgg):ENSP00000418516(p.Arg224Trp)]	NA	NA	NA	NA	NA	NA	NA	D	PrD	NA	MODERATE	D	1	118	118	0	115	86	26	22.61
Non-user	IOB_21	chr7	150841001	G	A	AGAP3	MISSENSE	ENSG00000133612:[ENST00000397238(c.2707G>A,Gag>Aag):ENSP00000380413(p.Glu903Lys)]	NA	chr7*150841001*150841001*COSM1087398*G*T*1*endometrium*1	NA	NA	NA	NA	NA	NA	NA	NA	MODIFIER	NA	NA	188	188	0	182	143	39	21.43
Non-user	IOB_21	chr7	152027726	C	T	KMT2C	MISSENSE	ENSG00000055609:[ENST00000452749(c.352G>A,Gca>Aca):ENSP00000410239(p.Ala118Thr)]	NA	NA	NA	NA	NA	NA	NA	T	BN	NA	MODERATE	NA	NA	297	296	1	257	249	7	2.72
Non-user	IOB_21	chr8	10285783	G	C	MSRA	SILENT	ENSG00000175806:[ENST00000317173(c.669G>C,ggG>ggC):ENSP00000313921(p.Gly223(=))]	NA	NA	NA	NA	NA	NA	NA	NA	NA	NA	LOW	NA	NA	168	168	0	224	170	47	20.98
Non-user	IOB_21	chr8	22481373	G	C	BIN3	NCT	ENSG00000147439:[ENST00000520489(n.2456+55C>G)]	NA	NA	1.60E-03	NA	NA	1.60E-03	8.20E-03	NA	NA	NA	MODIFIER	NA	NA	29	26	3	25	7	18	72
Non-user	IOB_21	chr8	26371961	G	A	DPYSL2	SILENT	1808:[NM_001197293.2(c.120G>A,ccG>ccA):NP_001184222.1(p.Pro40(=))]	NA	NA	NA	2.65E-04	4.00E-04	NA	NA	NA	NA	NA	LOW	NA	NA	177	176	0	132	127	5	3.79
Non-user	IOB_21	chr8	36746323	A	G	KCNU1	NCT	157855:[NR_134267.1(n.2097-16903A>G)]	NA	NA	chr8*36746319*36746323*CAGAA*C*rs755129802*NA*NA*NA|chr8*36746323*36746325*AAG*A*rs778979901*NA*NA*NA	NA	NA	NA	NA	NA	NA	NA	MODIFIER	NA	NA	110	110	0	101	72	28	27.72
Non-user	IOB_21	chr8	36746323	A	G	TPT1P8	EXONIC-NC	ENSG00000219491:[ENST00000510120(n.324A>G)]	NA	NA	chr8*36746319*36746323*CAGAA*C*rs755129802*NA*NA*NA|chr8*36746323*36746325*AAG*A*rs778979901*NA*NA*NA	NA	NA	NA	NA	NA	NA	NA	MODIFIER	NA	NA	110	110	0	101	72	28	27.72
Non-user	IOB_21	chr8	69018583	G	A	PREX2	INTRONIC	ENSG00000046889:[ENST00000288368(c.2716-1761G>A)]	NA	NA	NA	NA	NA	NA	NA	NA	NA	NA	MODIFIER	NA	NA	75	75	0	86	57	28	32.56
Non-user	IOB_21	chr8	81083474	G	C	TPD52	MISSENSE	ENSG00000076554:[ENST00000520795(c.145C>G,tCt>tGt):ENSP00000430865(p.Ser49Cys)]	NA	NA	chr8*81083474*81083474*G*A*rs557606187*0*0.9998*0.0001997	NA	NA	NA	NA	NA	NA	NA	MODIFIER	NA	NA	242	240	0	206	163	42	20.39
Non-user	IOB_21	chr8	85774547	G	A	RALYL	MISSENSE	ENSG00000184672:[ENST00000521268(c.430G>A,Ggg>Agg):ENSP00000430367(p.Gly144Arg)]	NA	NA	NA	6.44E-05	4.04E-04	NA	NA	NA	NA	NA	MODERATE	NA	1	70	69	0	77	51	26	33.77
Non-user	IOB_21	chr8	104898359	C	T	RIMS2	MISSENSE	ENSG00000176406:[ENST00000406091(c.1532C>T,tCg>tTg):ENSP00000384892(p.Ser511Leu)]	NA	chr8*104898359*104898359*COSM125866*C*T*3*NS,upper_aerodigestive_tract*2,1|chr8*104898359*104898359*COSM125865*C*T*3*NS,upper_aerodigestive_tract*2,1|chr8*104898359*104898359*COSM1722614*C*T*2*NS*2|chr8*104898359*104898359*COSM1722613*C*T*2*NS*2|chr8*104898359*104898359*COSM125867*C*T*3*NS,upper_aerodigestive_tract*2,1	NA	NA	NA	NA	NA	D_lc	PrD	NA	MODERATE	NA	NA	161	160	0	303	240	62	20.46
Non-user	IOB_21	chr8	124238883	G	A	C8orf76	NCT	ENSG00000189376:[ENST00000519791(n.334-11C>T)]	NA	NA	NA	8.40E-06	0.00E+00	NA	NA	NA	NA	NA	MODIFIER	NA	NA	130	129	0	119	99	19	15.97
Non-user	IOB_21	chr8	124238883	G	A	ZHX1-C8ORF76	INTRONIC	ENSG00000259305:[ENST00000357082(c.720-11C>T)]	NA	NA	NA	8.40E-06	0.00E+00	NA	NA	NA	NA	NA	MODIFIER	NA	NA	130	129	0	119	99	19	15.97
Non-user	IOB_21	chr8	145140692	C	A	GPAA1	EXONIC-NC	ENSG00000197858:[ENST00000534072(n.695C>A)]	NA	NA	NA	NA	NA	NA	NA	NA	NA	NA	MODIFIER	NA	NA	403	402	0	338	277	60	17.75
Non-user	IOB_21	chr9	14746326	T	G	FREM1	INTRONIC	ENSG00000164946:[ENST00000380894(c.1862+25A>C)]	NA	NA	NA	NA	NA	NA	NA	NA	NA	NA	MODIFIER	NA	NA	50	50	0	67	62	5	7.46
Non-user	IOB_21	chr9	15444637	C	A	SNAPC3	MISSENSE	ENSG00000164975:[ENST00000380821(c.515C>A,gCa>gAa):ENSP00000370200(p.Ala172Glu)]	NA	NA	NA	NA	NA	NA	NA	T	BN	NA	MODERATE	NA	NA	125	125	0	188	183	5	2.66
Non-user	IOB_21	chr9	27047411	C	T	IFT74	NCT	ENSG00000096872:[ENST00000482986(n.449+42C>T)]	NA	NA	NA	NA	NA	NA	NA	NA	NA	NA	MODIFIER	NA	NA	119	119	0	189	183	6	3.17
Non-user	IOB_21	chr9	33338560	G	A	NFX1	SILENT	ENSG00000086102:[ENST00000318524(c.2088G>A,cgG>cgA):ENSP00000317695(p.Arg696(=))]	NA	NA	NA	NA	NA	NA	NA	NA	NA	NA	LOW	NA	NA	292	290	0	366	357	7	1.91
Non-user	IOB_21	chr9	35792958	G	A	NPR2	MISSENSE	ENSG00000159899:[ENST00000342694(c.553G>A,Gag>Aag):ENSP00000341083(p.Glu185Lys)]	NA	chr9*35792958*35792958*COSM1259400*G*A*1*oesophagus*1|chr9*35792958*35792958*COSM1259401*G*A*1*oesophagus*1	NA	NA	NA	NA	NA	T	PoD	NA	MODERATE	D	1	239	238	0	355	348	7	1.97
Non-user	IOB_21	chr9	68415285	A	G	NA	INTERGENIC	NA	NA	NA	NA	2.46E-02	2.63E-02	NA	NA	NA	NA	NA	MODIFIER	NA	NA	335	332	2	334	324	10	2.99
Non-user	IOB_21	chr9	73240193	C	T	TRPM3	MISSENSE	ENSG00000083067:[ENST00000423814(c.1768G>A,Gag>Aag):ENSP00000389542(p.Glu590Lys)]	NA	NA	chr9*73240193*73240193*C*G*rs758428614*NA*NA*NA	NA	NA	NA	NA	D	PoD	NA	MODERATE	D	1	90	90	0	145	114	31	21.38
Non-user	IOB_21	chr9	95277340	T	C	CENPP	INTRONIC	401541:[NM_001286969.1(c.229-96256T>C)]	NA	NA	chr9*95277340*95277340*T*G*rs577270956*0*0.9998*0.0001997	NA	NA	NA	NA	NA	NA	NA	MODIFIER	NA	NA	82	82	0	148	143	5	3.38
Non-user	IOB_21	chr9	95277340	T	C	ECM2	SILENT	ENSG00000106823:[ENST00000444490(c.561A>G,gaA>gaG):ENSP00000393971(p.Glu187(=))]	NA	NA	chr9*95277340*95277340*T*G*rs577270956*0*0.9998*0.0001997	NA	NA	NA	NA	NA	NA	NA	MODIFIER	NA	NA	82	82	0	148	143	5	3.38
Non-user	IOB_21	chr9	100889002	C	A	CORO2A	SILENT	ENSG00000106789:[ENST00000343933(c.1275G>T,cgG>cgT):ENSP00000343746(p.Arg425(=))]	NA	NA	chr9*100889002*100889002*C*T*rs760096970*NA*NA*NA	NA	NA	NA	NA	NA	NA	NA	LOW	NA	NA	192	191	1	222	216	5	2.25
Non-user	IOB_21	chr9	107599206	A	T	ABCA1	INTRONIC	19:[XM_005251775.1(c.1248+55T>A)]	NA	NA	NA	NA	NA	NA	NA	NA	NA	NA	MODIFIER	NA	NA	44	44	0	55	42	13	23.64
Non-user	IOB_21	chr9	111662154	C	T	IKBKAP	MISSENSE	ENSG00000070061:[ENST00000374647(c.2152G>A,Gtt>Att):ENSP00000363779(p.Val718Ile)]	NA	NA	chr9*111662154*111662154*C*A,T*rs773965863*NA*NA*NA	8.27E-06	0.00E+00	NA	NA	D	BN	NA	MODERATE	D	1	156	156	0	192	151	41	21.35
Non-user	IOB_21	chr9	130569414	C	T	FPGS	INTRONIC	2356:[XM_005251865.1(c.-19+48C>T)]	NA	NA	NA	8.26E-06	0.00E+00	NA	NA	NA	NA	NA	MODIFIER	NA	NA	260	257	0	318	222	93	29.25
Non-user	IOB_21	chr9	135779233	G	C	TSC1	INTRONIC	7248:[NM_001162426.1(c.2039-29C>G)]	NA	NA	NA	NA	NA	NA	NA	NA	NA	NA	MODIFIER	NA	NA	44	44	0	46	32	14	30.43
Non-user	IOB_21	chr9	139402690	G	A	NOTCH1	NONSENSE	ENSG00000148400:[ENST00000277541(c.3319C>T,Cga>Tga):ENSP00000277541(p.Arg1107Ter)]	NA	NA	NA	NA	NA	NA	NA	NA	NA	NA	HIGH	NA	0.962	104	104	0	133	99	33	24.81

Patel *et al.* , 2021. Whole-exome sequencing analysis of oral squamous cell carcinoma delineated by tobacco usage habits
Supplementary Table 4. List of somatic single nucleotide variants identified using whole exome sequencing of OSCC patients

Cohort	Sample	Chromosome	Genomic position	Reference allele	Alternate allele	Genesymbol	Variant type	Variant description	OncoMD	COSMIC	dbSNP	ExAC	ExAC_SAS	1000G	1000G_SAS	SIFT	Polyphen2	CONDEL	VEP	LRT	PhastCon7Way	Total reads in Normal	Reference read support in Normal	Alternative read support in Normal	Total reads in Tumor	Reference read support in Tumor	Alternate read support in Tumor	Alternate allele frequency in Tumor
Non-user	IOB_21	chr9	139412594	G	T	NOTCH1	NONSENSE	ENSG00000148400:[ENST00000277541(c.1250C>A,tCg>tAg):ENSP00000277541(p.Ser417Ter)]	NA	NA	chr9*139412594*139412594*G*A,C*rs757631575*NA*NA*NA	NA	NA	NA	NA	NA	NA	NA	HIGH	NA	1	134	134	0	171	101	70	40.94
Non-user	IOB_21	chr9	139636502	G	C	LCN10	EXONIC-NC	ENSG00000187922:[ENST00000406721(n.1307C>G)]	NA	NA	NA	NA	NA	NA	NA	NA	NA	NA	MODIFIER	NA	NA	316	315	0	464	343	121	26.08
Non-user	IOB_21	chr9	139636502	G	C	LCN6	NCT	ENSG00000204003:[ENST00000480584(n.481-92C>G)]	NA	NA	NA	NA	NA	NA	NA	NA	NA	NA	MODIFIER	NA	NA	316	315	0	464	343	121	26.08
Non-user	IOB_21	chr9	140080750	G	A	ANAPC2	NONSENSE	ENSG00000176248:[ENST00000323927(c.799C>T,Cag>Tag):ENSP00000314004(p.Gln267Ter)]	NA	NA	NA	NA	NA	NA	NA	NA	NA	NA	MODIFIER	NA	NA	94	94	0	108	68	38	35.19
Non-user	IOB_21	chrX	46696483	C	G	RP2	5UTR	ENSG00000102218:[ENST00000218340(c.-53C>G)]	NA	NA	NA	NA	NA	NA	NA	NA	NA	NA	MODIFIER	NA	NA	147	147	0	166	109	56	33.73
Non-user	IOB_21	chrX	47662836	G	A	WASF4P	EXONIC-NC	ENSG00000188459:[ENST00000338709(n.526G>A)]	NA	NA	2.65E-04	1.14E-05	0.00E+00	2.65E-04	0.00E+00	NA	NA	NA	MODIFIER	NA	NA	61	61	0	36	24	12	33.33
Non-user	IOB_21	chrX	129299570	G	T	AIFM1	SILENT	ENSG00000156709:[ENST00000287295(c.61C>A,Cgg>Agg):ENSP00000287295(p.Arg21(=))]	NA	NA	chrX*129299570*129299570*G*A*rs780367991*NA*NA*NA	NA	NA	NA	NA	NA	NA	NA	MODIFIER	NA	NA	75	75	0	91	86	5	5.49
Non-user	IOB_21	chrX	142718041	G	C	SLITRK4	MISSENSE	ENSG00000179542:[ENST00000338017(c.884C>G,tCt>tGt):ENSP00000336627(p.Ser295Cys)]	NA	NA	NA	NA	NA	NA	NA	D	PoD	NA	MODERATE	D	1	172	172	0	171	120	50	29.24
Non-user	IOB_21	chrX	153459080	T	A	OPN1MW	MISSENSE	ENSG00000147380:[ENST00000369935(c.926T>A,tTc>tAc):ENSP00000358951(p.Phe309Tyr)]	NA	NA	NA	1.97E-04	1.33E-04	NA	NA	NA	NA	NA	MODIFIER	NA	NA	81	80	0	121	101	20	16.53
Non-user	IOB_22	chr1	19244887	C	T	IFFO2	MISSENSE	ENSG00000169991:[ENST00000455833(c.932G>A,cGg>cAg):ENSP00000387941(p.Arg311Gln)]	NA	NA	NA	NA	NA	NA	NA	D	PrD	NA	MODERATE	D	1	408	407	0	242	234	6	2.48
Non-user	IOB_22	chr1	19244887	C	T	RP13-279N23.2	MISSENSE	ENSG00000255275:[ENST00000494072(c.267G>A,cGg>cAg):ENSP00000455952(p.Arg90Gln)]	NA	NA	NA	NA	NA	NA	NA	D	PrD	NA	MODERATE	D	1	408	407	0	242	234	6	2.48
Non-user	IOB_22	chr1	32646901	C	T	TXLNA	SILENT	ENSG00000084652:[ENST00000373609(c.228C>T,agC>agT):ENSP00000362711(p.Ser76(=))]	NA	NA	NA	NA	NA	NA	NA	NA	NA	NA	MODIFIER	NA	NA	256	253	0	116	111	5	4.31
Non-user	IOB_22	chr1	114696304	C	A	SYT6	MISSENSE	ENSG00000134207:[ENST00000393296(c.91G>T,Ggg>Tgg):ENSP00000376974(p.Gly31Trp)]	NA	NA	NA	NA	NA	NA	NA	NA	NA	NA	MODIFIER	NA	NA	243	243	0	192	185	6	3.12
Non-user	IOB_22	chr1	145527607	C	A	ITGA10	MISSENSE	8515:[XM_005272978.1(c.13C>A,Cca>Aca):XP_005273035.1(p.Pro5Thr)]	NA	NA	chr1*145527607*145527607*C*G*rs587672026*0*0.9998*0.0001997	NA	NA	NA	NA	NA	NA	NA	LOW	NA	NA	346	345	0	191	184	6	3.14
Non-user	IOB_22	chr10	99531296	G	A	SFRP5	MISSENSE	ENSG00000120057:[ENST00000266066(c.295C>T,Cgc>Tgc):ENSP00000266066(p.Arg99Cys)]	NA	NA	NA	NA	NA	NA	NA	D	BN	NA	MODERATE	D	1	386	385	1	195	190	5	2.56
Non-user	IOB_22	chr11	33612942	G	A	KIAA1549L	MISSENSE	ENSG00000110427:[ENST00000389726(c.3853G>A,Gcc>Acc):ENSP00000374376(p.Ala1285Thr)]	NA	NA	NA	1.67E-05	6.18E-05	NA	NA	NA	NA	NA	MODIFIER	NA	NA	208	206	0	151	146	5	3.31
Non-user	IOB_22	chr11	35198224	A	G	CD44	MISSENSE	ENSG00000026508:[ENST00000442151(c.168A>G,aAt>aGt):ENSP00000398099(p.Asn57Ser)]	NA	NA	NA	NA	NA	NA	NA	D	BN	NA	MODERATE	NA	NA	299	295	0	199	189	10	5.03
Non-user	IOB_22	chr11	47823333	G	T	NUP160	INTRONIC	ENSG00000030066:[ENST00000528071(c.2553+30C>A)]	NA	NA	NA	NA	NA	NA	NA	NA	NA	NA	MODIFIER	NA	NA	176	176	0	128	122	5	3.91
Non-user	IOB_22	chr11	55339962	G	A	OR4C16	MISSENSE	ENSG00000181935:[ENST00000314634(c.359G>A,cGc>cAc):ENSP00000324913(p.Arg120His)]	NA	chr11*55339962*55339962*COSM1321641*G*A*1*ovary*1	5.99E-04	8.57E-04	2.61E-03	5.99E-04	1.00E-03	T	BN	NA	MODERATE	NA	0.746	630	626	2	404	385	18	4.46
Non-user	IOB_22	chr11	66999175	G	A	KDM2A	MISSENSE	ENSG00000173120:[ENST00000398645(c.1223G>A,gGc>gAc):ENSP00000381640(p.Gly408Asp)]	NA	NA	NA	NA	NA	NA	NA	T	BN	NA	MODERATE	N	1	218	218	0	176	171	5	2.84
Non-user	IOB_22	chr12	4554487	G	T	FGF6	SILENT	ENSG00000111241:[ENST00000228837(c.250C>A,Cgg>Agg):ENSP00000228837(p.Arg84(=))]	NA	NA	chr12*4554487*4554487*G*A*rs373061794*NA*NA*NA	NA	NA	NA	NA	NA	NA	NA	LOW	NA	1	867	862	1	627	599	28	4.47
Non-user	IOB_22	chr12	40940355	C	T	MUC19	NCT	ENSG00000205592:[ENST00000541039(n.359-50C>T)]	NA	NA	7.99E-04	6.05E-04	0.00E+00	7.99E-04	0.00E+00	NA	NA	NA	MODIFIER	NA	NA	268	267	1	197	187	10	5.08
Non-user	IOB_22	chr12	48919470	C	T	OR8S1	MISSENSE	ENSG00000197376:[ENST00000310194(c.56C>T,gCc>gTc):ENSP00000310632(p.Ala19Val)]	NA	chr12*48919470*48919470*COSM3398749*C*T*1*central_nervous_system*1	NA	NA	NA	NA	NA	NA	NA	NA	MODIFIER	NA	NA	263	262	1	193	185	5	2.59
Non-user	IOB_22	chr12	89865391	C	A	POC1B	MISSENSE-SS-PRX	ENSG00000139323:[ENST00000552563(n.477-3929G>T)]	NA	NA	NA	NA	NA	NA	NA	D	PrD	NA	MODERATE	D	1	114	114	0	73	68	5	6.85
Non-user	IOB_22	chr12	94975601	C	T	TMCC3	SILENT	ENSG00000057704:[ENST00000551457(c.699G>A,tcG>tcA):ENSP00000449888(p.Ser233(=))]	NA	NA	chr12*94975601*94975601*C*A,T*rs749913386*NA*NA*NA	4.95E-05	1.82E-04	NA	NA	NA	NA	NA	LOW	NA	NA	478	476	2	253	247	6	2.37
Non-user	IOB_22	chr12	111078218	C	T	HVCN1	INTRONIC	ENSG00000122986:[ENST00000548312(c.756+9755G>A)]	NA	NA	NA	NA	NA	NA	NA	NA	NA	NA	MODIFIER	NA	NA	192	192	0	104	97	6	5.77
Non-user	IOB_22	chr12	111078218	C	T	TCTN1	NONSENSE	ENSG00000204852:[ENST00000397655(c.832C>T,Cag>Tag):ENSP00000380775(p.Gln278Ter)]	NA	NA	NA	NA	NA	NA	NA	NA	NA	NA	MODIFIER	NA	NA	192	192	0	104	97	6	5.77
Non-user	IOB_22	chr14	75264858	C	A	YLPM1	MISSENSE	ENSG00000119596:[ENST00000549293(c.1517C>A,cCt>cAt):ENSP00000449860(p.Pro506His)]	NA	NA	NA	NA	NA	NA	NA	NA	PoD	NA	MODERATE	N	1	424	423	0	285	277	5	1.75
Non-user	IOB_22	chr14	92537379	T	C	ATXN3	MISSENSE	4287:[NM_001164778.1(c.406A>G,Aca>Gca):NP_001158250.1(p.Thr136Ala)]	NA	chr14*92537379*92537379*COSM433493*T*C*1*breast*1	NA	3.34E-01	2.84E-01	NA	NA	NA	NA	NA	MODIFIER	NA	NA	78	77	0	35	21	12	34.29
Non-user	IOB_22	chr14	102691132	C	A	MOK	INTRONIC-SS-ACR	ENSG00000080823:[ENST00000559512(n.454-1G>T)]	NA	NA	NA	NA	NA	NA	NA	NA	NA	NA	HIGH	NA	NA	171	170	1	86	80	6	6.98
Non-user	IOB_22	chr14	102691132	C	A	WDR20	3UTR	ENSG00000140153:[ENST00000559478(c.*153C>A)]	NA	NA	NA	NA	NA	NA	NA	NA	NA	NA	HIGH	NA	NA	171	170	1	86	80	6	6.98
Non-user	IOB_22	chr15	64056408	C	T	HERC1	NCT	ENSG00000103657:[ENST00000559886(n.1048-18G>A)]	NA	NA	2.00E-04	5.80E-05	0.00E+00	2.00E-04	0.00E+00	NA	NA	NA	MODIFIER	NA	NA	258	258	0	168	161	6	3.57
Non-user	IOB_22	chr16	2024990	G	A	TBL3	MISSENSE	ENSG00000183751:[ENST00000568546(c.526G>A,Gcc>Acc):ENSP00000454836(p.Ala176Thr)]	NA	NA	NA	8.50E-06	0.00E+00	NA	NA	T	BN	NA	MODERATE	NA	NA	283	282	1	127	122	5	3.94
Non-user	IOB_22	chr17	1183438	C	A	TUSC5	NONSENSE	ENSG00000184811:[ENST00000333813(c.143C>A,tCg>tAg):ENSP00000329548(p.Ser48Ter)]	NA	NA	chr17*1183438*1183438*C*T*rs527632397*0*0.9998*0.0001997	NA	NA	NA	NA	NA	NA	NA	HIGH	NA	0.005	335	334	0	228	223	5	2.19
Non-user	IOB_22	chr17	20459506	A	G	RP11-434D2.11	EXONIC-NC	ENSG00000263946:[ENST00000579603(n.90T>C)]	NA	NA	NA	NA	NA	NA	NA	NA	NA	NA	MODIFIER	NA	NA	123	123	0	77	69	8	10.39
Non-user	IOB_22	chr17	34955412	C	T	DHRS11	MISSENSE	ENSG00000108272:[ENST00000251312(c.515C>T,gCc>gTc):ENSP00000251312(p.Ala172Val)]	NA	chr17*34955412*34955412*COSM560623*C*T*1*lung*1	NA	NA	NA	NA	NA	NA	NA	NA	MODERATE	NA	1	553	548	1	329	320	5	1.52
Non-user	IOB_22	chr17	48153837	G	T	ITGA3	NONSENSE-SS-PRX	ENSG00000005884:[ENST00000505306(n.2194G>T)]	NA	NA	NA	NA	NA	NA	NA	NA	NA	NA	LOW	NA	NA	397	397	0	244	236	8	3.28
Non-user	IOB_22	chr18	19034516	G	A	GREB1L	MISSENSE	ENSG00000141449:[ENST00000400483(c.2174G>A,cGa>cAa):ENSP00000383331(p.Arg725Gln)]	NA	NA	NA	9.22E-05	2.53E-04	NA	NA	T	PrD	NA	MODERATE	NA	NA	212	212	0	130	125	5	3.85
Non-user	IOB_22	chr19	1489849	G	C	PCSK4	SILENT	ENSG00000115257:[ENST00000591201(c.226C>G,gtC>gtG):ENSP00000465102(p.Val76(=))]	NA	NA	NA	NA	NA	NA	NA	NA	NA	NA	MODIFIER	NA	NA	183	181	0	88	82	6	6.82
Non-user	IOB_22	chr19	3637888	G	A	PIP5K1C	SILENT	ENSG00000186111:[ENST00000537021(c.2037C>T,tgC>tgT):ENSP00000444779(p.Cys679(=))]	NA	NA	NA	NA	NA	NA	NA	NA	NA	NA	MODIFIER	NA	NA	176	176	0	144	139	5	3.47
Non-user	IOB_22	chr19	7976023	C	T	MAP2K7	SILENT	ENSG00000076984:[ENST00000545011(c.960C>T,gcC>gcT):ENSP00000443946(p.Ala320(=))]	NA	NA	NA	6.24E-05	1.23E-04	NA	NA	NA	NA	NA	MODIFIER	NA	NA	150	150	0	101	95	5	4.95
Non-user	IOB_22	chr19	14074343	C	T	RFX1	INTRONIC-SS-DNR-PRX	ENSG00000132005:[ENST00000254325(c.2570+7G>A)]	NA	NA	NA	NA	NA	NA	NA	NA	NA	NA	LOW	NA	NA	397	397	0	195	188	5	2.56
Non-user	IOB_22	chr19	15234194	C	T	ILVBL	MISSENSE	ENSG00000105135:[ENST00000263383(c.329G>A,cGc>cAc):ENSP00000263383(p.Arg110His)]	NA	chr19*15234194*15234194*COSM3422484*C*T*1*large_intestine*1	NA	3.35E-05	0.00E+00	NA	NA	NA	NA	NA	MODIFIER	NA	NA	545	540	1	322	315	7	2.17
Non-user	IOB_22	chr19	17837286	C	A	MAP1S	MISSENSE	ENSG00000130479:[ENST00000544059(c.1015C>A,Ctg>Atg):ENSP00000439243(p.Leu339Met)]	NA	NA	NA	NA	NA	NA	NA	D	PrD	NA	MODERATE	N	0.932	242	242	0	149	143	6	4.03
Non-user	IOB_22	chr19	19823742	C	T	ZNF14	SILENT	ENSG00000105708:[ENST00000344099(c.348G>A,tcG>tcA):ENSP00000340514(p.Ser116(=))]	NA	NA	NA	3.30E-05	1.21E-04	NA	NA	NA	NA	NA	LOW	NA	NA	423	422	0	241	226	15	6.22
Non-user	IOB_22	chr19	49517799	C	A	RUVBL2	MISSENSE	ENSG00000183207:[ENST00000413176(c.925C>A,Ctg>Atg):ENSP00000413890(p.Leu309Met)]	NA	NA	NA	NA	NA	NA	NA	NA	NA	NA	MODIFIER	NA	NA	214	214	0	129	123	5	3.88
Non-user	IOB_22	chr2	44031859	A	G	DYNC2LI1	MISSENSE	ENSG00000138036:[ENST00000443170(c.503A>G,gAa>gGa):ENSP00000388941(p.Glu168Gly)]	NA	NA	NA	NA	NA	NA	NA	T	BN	NA	MODERATE	D	1	136	136	0	83	78	5	6.02
Non-user	IOB_22	chr2	67631589	C	A	ETAA1	MISSENSE	ENSG00000143971:[ENST00000272342(c.1775C>A,gCa>gAa):ENSP00000272342(p.Ala592Glu)]	NA	NA	NA	NA	NA	NA	NA	NA	NA	NA	MODIFIER	NA	NA	219	217	2	159	153	6	3.77
Non-user	IOB_22	chr2	106694362	G	A	C2orf40	MISSENSE	ENSG00000119147:[ENST00000409944(c.319G>A,Gtc>Atc):ENSP00000386421(p.Val107Ile)]	NA	NA	2.00E-04	2.50E-05	6.13E-05	2.00E-04	0.00E+00	D	PrD	NA	MODERATE	NA	NA	207	205	1	142	137	5	3.52
Non-user	IOB_22	chr2	152500989	G	A	NEB	MISSENSE	ENSG00000183091:[ENST00000172853(c.7637C>T,gCc>gTc):ENSP00000172853(p.Ala2546Val)]	NA	NA	NA	NA	NA	NA	NA	T	BN	NA	MODERATE	D	1	633	632	0	399	390	7	1.75
Non-user	IOB_22	chr2	162738976	G	A	SLC4A10	INTRONIC	57282:[XM_005246696.1(c.1401+22G>A)]	NA	NA	NA	9.49E-05	0.00E+00	NA	NA	NA	NA	NA	MODIFIER	NA	NA	200	200	0	174	167	5	2.87
Non-user	IOB_22	chr2	168102942	G	C	XIRP2	MISSENSE	ENSG00000163092:[ENST00000409273(c.4374G>C,caG>caC):ENSP00000387255(p.Gln1458His)]	NA	NA	NA	NA	NA	NA	NA	D	PrD	NA	MODERATE	D	0.996	175	175	0	103	97	6	5.83
Non-user	IOB_22	chr2	179633577	C	A	TTN	MISSENSE	ENSG00000155657:[ENST00000342175(c.8848G>T,Ggc>Tgc):ENSP00000340554(p.Gly2950Cys)]	NA	NA	NA	NA	NA	NA	NA	NA	NA	NA	MODERATE	NA	NA	333	331	0	226	221	5	2.21
Non-user	IOB_22	chr2	179633577	C	A	TTN-AS1	NCT	ENSG00000237298:[ENST00000585451(n.457-4425C>A)]	NA	NA	NA	NA	NA	NA	NA	NA	NA	NA	MODERATE	NA	NA	333	331	0	226	221	5	2.21
Non-user	IOB_22	chr2	228109627	T	G	AC097662.2	NCT	ENSG00000236432:[ENST00000606119(n.22-6737A>C)]	NA	NA	9.59E-03	7.22E-02	6.68E-03	9.58E-03	2.00E-03	NA	NA	NA	MODIFIER	NA	NA	133	132	1	75	68	6	8
Non-user	IOB_22	chr2	228109627	T	G	COL4A3	NCT	1285:[XR_241280.1(n.423-40T>G)]	NA	NA	9.59E-03	7.22E-02	6.68E-03	9.58E-03	2.00E-03	NA	NA	NA	MODIFIER	NA	NA	133	132	1	75	68	6	8
Non-user	IOB_22	chr2	239038956	C	T	ESPNL	MISSENSE	ENSG00000144488:[ENST00000409169(c.1469C>T,gCg>gTg):ENSP00000386577(p.Ala490Val)]	NA	NA	NA	4.53E-05	0.00E+00	NA	NA	T	BN	NA	MODERATE	D	0.042	270	268	0	176	169	6	3.41
Non-user	IOB_22	chr20	20349140	C	T	INSM1	NONSENSE	ENSG00000173404:[ENST00000310227(c.229C>T,Cag>Tag):ENSP00000312631(p.Gln77Ter)]	NA	NA	NA	NA	NA	NA	NA	NA	NA	NA	HIGH	NA	0	226	225	0	142	137	5	3.52
Non-user	IOB_22	chr20	34807733	C	A	EPB41L1	SILENT	ENSG00000088367:[ENST00000454226(c.489C>A,acC>acA):ENSP00000388281(p.Thr163(=))]	NA	NA	NA	NA	NA	NA	NA	NA	NA	NA	LOW	NA	NA	143	142	0	82	77	5	6.1
Non-user	IOB_22	chr20	36393629	C	A	CTNNBL1	MISSENSE	ENSG00000132792:[ENST00000361383(c.595C>A,Cag>Aag):ENSP00000355050(p.Gln199Lys)]	NA	NA	NA	NA	NA	NA	NA	D	PrD	NA	MODERATE	D	1	369	366	0	251	241	8	3.19
Non-user	IOB_22	chr20	62560715	C	T	DNAJC5	MISSENSE	ENSG00000101152:[ENST00000360864(c.158C>T,gCc>gTc):ENSP00000354111(p.Ala53Val)]	NA	NA	NA	NA	NA	NA	NA	D	PoD	NA	MODERATE	NA	NA	538	534	0	307	298	5	1.63
Non-user	IOB_22	chr21	41165425	C	T	IGSF5	NCT	ENSG00000183067:[ENST00000459922(n.471-36C>T)]	NA	NA	NA	NA	NA	NA	NA	NA	NA	NA	MODIFIER	NA	NA	384	382	0	258	249	7	2.71
Non-user	IOB_22	chr21	43413213	C	A	ZBTB21	MISSENSE	ENSG00000173276:[ENST00000310826(c.992G>T,aGg>aTg):ENSP00000308759(p.Arg331Met)]	NA	NA	NA	NA	NA	NA	NA	D	PoD	NA	MODERATE	D	0.883	314	312	1	179	174	5	2.79
Non-user	IOB_22	chr22	32617022	C	A	RP1-90G24.10	NCT	ENSG00000242082:[ENST00000434942(n.274+13759C>A)]	NA	NA	NA	NA	NA	NA	NA	NA	NA	NA	MODIFIER	NA	NA	297	297	0	235	228	5	2.13
Non-user	IOB_22	chr22	32617022	C	A	SLC5A4	INTRONIC	ENSG00000100191:[ENST00000266086(c.1666-13G>T)]	NA	NA	NA	NA	NA	NA	NA	NA	NA	NA	MODIFIER	NA	NA	297	297	0	235	228	5	2.13
Non-user	IOB_22	chr22	36122306	C	G	APOL5	NONSENSE	ENSG00000128313:[ENST00000249044(c.191C>G,tCa>tGa):ENSP00000249044(p.Ser64Ter)]	NA	NA	NA	NA	NA	NA	NA	NA	NA	NA	HIGH	NA	0	383	380	1	205	199	5	2.44
Non-user	IOB_22	chr3	50255690	A	C	SLC38A3	NCT	ENSG00000188338:[ENST00000445096(n.700-12A>C)]	NA	NA	NA	NA	NA	NA	NA	NA	NA	NA	MODIFIER	NA	NA	266	256	6	119	102	11	9.24
Non-user	IOB_22	chr3	123420219	G	C	MYLK	INTRONIC	4638:[XM_005247493.1(c.2492+66C>G)]	NA	NA	NA	NA	NA	NA	NA	NA	NA	NA	MODIFIER	NA	NA	98	98	0	60	55	5	8.33
Non-user	IOB_22	chr3	123420219	G	C	NA	NCT	101927028:[XR_241557.1(n.66-326G>C)]	NA	NA	NA	NA	NA	NA	NA	NA	NA	NA	MODIFIER	NA	NA	98	98	0	60	55	5	8.33
Non-user	IOB_22	chr4	76415821	C	A	RCHY1	MISSENSE	ENSG00000163743:[ENST00000513257(c.600G>T,atG>atT):ENSP00000421084(p.Met200Ile)]	NA	NA	NA	NA	NA	NA	NA	NA	NA	NA	MODIFIER	NA	NA	460	459	0	226	216	7	3.1
Non-user	IOB_22	chr5	140073688	G	T	HARS2	INTRONIC	ENSG00000112855:[ENST00000503873(c.321+49G>T)]	NA	NA	NA	NA	NA	NA	NA	NA	NA	NA	MODIFIER	NA	NA	249	249	0	127	120	5	3.94
Non-user	IOB_22	chr5	140858111	C	A	PCDHGA1	INTRONIC	ENSG00000204956:[ENST00000517417(c.2422-16263C>A)]	NA	NA	NA	NA	NA	NA	NA	NA	NA	NA	MODIFIER	NA	NA	352	352	0	182	177	5	2.75
Non-user	IOB_22	chr5	140858111	C	A	PCDHGA10	INTRONIC	ENSG00000253846:[ENST00000398610(c.2437-16263C>A)]	NA	NA	NA	NA	NA	NA	NA	NA	NA	NA	MODIFIER	NA	NA	352	352	0	182	177	5	2.75
Non-user	IOB_22	chr5	140858111	C	A	PCDHGA11	INTRONIC	ENSG00000253873:[ENST00000398587(c.2434-16263C>A)]	NA	NA	NA	NA	NA	NA	NA	NA	NA	NA	MODIFIER	NA	NA	352	352	0	182	177	5	2.75

Patel *et al.* , 2021. Whole-exome sequencing analysis of oral squamous cell carcinoma delineated by tobacco usage habits
Supplementary Table 4. List of somatic single nucleotide variants identified using whole exome sequencing of OSCC patients

Cohort	Sample	Chromosome	Genomic position	Reference allele	Alternate allele	Genesymbol	Variant type	Variant description	OncoMD	COSMIC	dbSNP	ExAC	ExAC_SAS	1000G	1000G_SAS	SIFT	Polyphen2	CONDEL	VEP	LRT	PhastCon7Way	Total reads in Normal	Reference read support in Normal	Alternative read support in Normal	Total reads in Tumor	Reference read support in Tumor	Alternate read support in Tumor	Alternate allele frequency in Tumor
Non-user	IOB_22	chr5	140858111	C	A	PCDHGA12	INTRONIC	ENSG00000253159:[ENST00000252085(c.2425-16263C>A)]	NA	NA	NA	NA	NA	NA	NA	NA	NA	NA	MODIFIER	NA	NA	352	352	0	182	177	5	2.75
Non-user	IOB_22	chr5	140858111	C	A	PCDHGA2	INTRONIC	ENSG00000081853:[ENST00000394576(c.2425-16263C>A)]	NA	NA	NA	NA	NA	NA	NA	NA	NA	NA	MODIFIER	NA	NA	352	352	0	182	177	5	2.75
Non-user	IOB_22	chr5	140858111	C	A	PCDHGA3	INTRONIC	ENSG00000254245:[ENST00000253812(c.2425-16263C>A)]	NA	NA	NA	NA	NA	NA	NA	NA	NA	NA	MODIFIER	NA	NA	352	352	0	182	177	5	2.75
Non-user	IOB_22	chr5	140858111	C	A	PCDHGA4	INTRONIC	56111:[NM_018917.3(c.2515-16263C>A)]	NA	NA	NA	NA	NA	NA	NA	NA	NA	NA	MODIFIER	NA	NA	352	352	0	182	177	5	2.75
Non-user	IOB_22	chr5	140858111	C	A	PCDHGA5	INTRONIC	ENSG00000253485:[ENST00000518069(c.2422-16263C>A)]	NA	NA	NA	NA	NA	NA	NA	NA	NA	NA	MODIFIER	NA	NA	352	352	0	182	177	5	2.75
Non-user	IOB_22	chr5	140858111	C	A	PCDHGA6	INTRONIC	56109:[XM_005268482.1(c.4936-16263C>A)]	NA	NA	NA	NA	NA	NA	NA	NA	NA	NA	MODIFIER	NA	NA	352	352	0	182	177	5	2.75
Non-user	IOB_22	chr5	140858111	C	A	PCDHGA7	INTRONIC	ENSG00000253537:[ENST00000518325(c.2425-16263C>A)]	NA	NA	NA	NA	NA	NA	NA	NA	NA	NA	MODIFIER	NA	NA	352	352	0	182	177	5	2.75
Non-user	IOB_22	chr5	140858111	C	A	PCDHGA8	INTRONIC	ENSG00000253767:[ENST00000398604(c.2425-16263C>A)]	NA	NA	NA	NA	NA	NA	NA	NA	NA	NA	MODIFIER	NA	NA	352	352	0	182	177	5	2.75
Non-user	IOB_22	chr5	140858111	C	A	PCDHGA9	INTRONIC	ENSG00000261934:[ENST00000573521(c.2425-16263C>A)]	NA	NA	NA	NA	NA	NA	NA	NA	NA	NA	MODIFIER	NA	NA	352	352	0	182	177	5	2.75
Non-user	IOB_22	chr5	140858111	C	A	PCDHGB1	INTRONIC	ENSG00000254221:[ENST00000523390(c.2410-16263C>A)]	NA	NA	NA	NA	NA	NA	NA	NA	NA	NA	MODIFIER	NA	NA	352	352	0	182	177	5	2.75
Non-user	IOB_22	chr5	140858111	C	A	PCDHGB2	INTRONIC	ENSG00000253910:[ENST00000522605(c.2422-16263C>A)]	NA	NA	NA	NA	NA	NA	NA	NA	NA	NA	MODIFIER	NA	NA	352	352	0	182	177	5	2.75
Non-user	IOB_22	chr5	140858111	C	A	PCDHGB3	INTRONIC	ENSG00000262209:[ENST00000576222(c.2416-16263C>A)]	NA	NA	NA	NA	NA	NA	NA	NA	NA	NA	MODIFIER	NA	NA	352	352	0	182	177	5	2.75
Non-user	IOB_22	chr5	140858111	C	A	PCDHGB4	INTRONIC	ENSG00000253953:[ENST00000519479(c.2398-16263C>A)]	NA	NA	NA	NA	NA	NA	NA	NA	NA	NA	MODIFIER	NA	NA	352	352	0	182	177	5	2.75
Non-user	IOB_22	chr5	140858111	C	A	PCDHGB6	INTRONIC	ENSG00000253305:[ENST00000520790(c.2419-16263C>A)]	NA	NA	NA	NA	NA	NA	NA	NA	NA	NA	MODIFIER	NA	NA	352	352	0	182	177	5	2.75
Non-user	IOB_22	chr5	140858111	C	A	PCDHGB7	INTRONIC	ENSG00000254122:[ENST00000398594(c.2416-16263C>A)]	NA	NA	NA	NA	NA	NA	NA	NA	NA	NA	MODIFIER	NA	NA	352	352	0	182	177	5	2.75
Non-user	IOB_22	chr5	140858111	C	A	PCDHGC3	MISSENSE-SS-PRX	5098:[NM_032403.2(c.30+2398C>A)]	NA	NA	NA	NA	NA	NA	NA	NA	NA	NA	MODIFIER	NA	NA	352	352	0	182	177	5	2.75
Non-user	IOB_22	chr5	176916729	A	G	PDLIM7	NCT	9260:[NR_103804.1(n.922+43T>C)]	NA	NA	NA	NA	NA	NA	NA	NA	NA	NA	MODIFIER	NA	NA	156	155	0	79	73	5	6.33
Non-user	IOB_22	chr5	179201464	G	A	MAML1	SILENT	ENSG00000161021:[ENST00000292599(c.2637G>A,ccG>ccA):ENSP00000292599(p.Pro879(=))]	NA	NA	4.39E-03	1.07E-02	6.61E-03	4.39E-03	3.10E-03	NA	NA	NA	LOW	NA	NA	387	386	0	208	202	6	2.88
Non-user	IOB_22	chr6	133005566	C	A	VNN1	NONSENSE	ENSG00000112299:[ENST00000367928(c.1267G>T,Gaa>Taa):ENSP00000356905(p.Glu423Ter)]	NA	NA	NA	NA	NA	NA	NA	NA	NA	NA	HIGH	NA	1	249	248	1	179	171	5	2.79
Non-user	IOB_22	chr7	2977696	G	A	CARD11	INTRONIC	ENSG00000198286:[ENST00000396946(c.1018-30C>T)]	NA	NA	NA	2.47E-05	1.21E-04	NA	NA	NA	NA	NA	MODIFIER	NA	NA	312	309	1	189	184	5	2.65
Non-user	IOB_22	chr7	5428719	G	A	TNRC18	MISSENSE	ENSG00000182095:[ENST00000399537(c.736C>T,Cgc>Tgc):ENSP00000382452(p.Arg246Cys)]	NA	NA	NA	NA	NA	NA	NA	D	PoD	NA	MODERATE	NA	NA	169	169	0	83	78	5	6.02
Non-user	IOB_22	chr7	142499789	A	T	TRBC2	SILENT-SS-PRX	ENSG00000211772:[ENST00000466254(c.406A>T,gAg>gTg):ENSP00000417300(p.Glu136Val)]	NA	NA	NA	1.69E-05	0.00E+00	NA	NA	NA	NA	NA	LOW	NA	NA	151	136	15	110	75	35	31.82
Non-user	IOB_22	chr7	142499793	T	C	TRBC2	SILENT	ENSG00000211772:[ENST00000466254(c.410T>C,tcT>tcC):ENSP00000417300(p.Ser137(=))]	NA	NA	NA	1.69E-05	0.00E+00	NA	NA	NA	NA	NA	LOW	NA	NA	151	136	15	111	76	35	31.53
Non-user	IOB_22	chr9	14806786	G	A	FREM1	SILENT	ENSG00000164946:[ENST00000380875(c.3147C>T,tcC>tcT):ENSP00000370257(p.Ser1049(=))]	NA	NA	7.99E-04	1.27E-03	0.00E+00	7.99E-04	0.00E+00	NA	NA	NA	LOW	NA	NA	234	232	1	134	129	5	3.73
Non-user	IOB_22	chr9	128002963	G	A	HSPA5	MISSENSE	ENSG00000044574:[ENST00000324460(c.346C>T,Ccg>Tcg):ENSP00000324173(p.Pro116Ser)]	NA	NA	NA	NA	NA	NA	NA	T_lc	PrD	NA	MODERATE	D	1	399	395	1	190	182	7	3.68
Non-user	IOB_22	chr9	133346199	C	A	ASS1	NCT	ENSG00000130707:[ENST00000467695(n.205-22C>A)]	NA	NA	NA	NA	NA	NA	NA	NA	NA	NA	MODIFIER	NA	NA	240	239	0	182	176	5	2.75
Non-user	IOB_22	chr9	140772670	A	T	CACNA1B	INTRONIC-SS-DNR	ENSG00000148408:[ENST00000277549(c.-2141+1A>T)]	NA	NA	chr9*140772669*140772672*CATA*C*rs750056326*NA*NA*NA|chr9*140772670*140772670*A*C,T*rs200491212*NA*NA*NA	NA	NA	NA	NA	NA	NA	NA	HIGH	NA	NA	117	114	1	64	57	6	9.38
Non-user	IOB_22	chr9	140772670	A	T	RP11-188C12.3	NCT	ENSG00000203987:[ENST00000371390(n.2692-2538T>A)]	NA	NA	chr9*140772669*140772672*CATA*C*rs750056326*NA*NA*NA|chr9*140772670*140772670*A*C,T*rs200491212*NA*NA*NA	NA	NA	NA	NA	NA	NA	NA	HIGH	NA	NA	117	114	1	64	57	6	9.38
Non-user	IOB_22	chrX	117576289	C	A	WDR44	MISSENSE	ENSG00000131725:[ENST00000371848(c.1947C>A,cCt>cAt):ENSP00000360914(p.Pro650His)]	NA	chrX*117576289*117576289*COSM1113643*C*A*1*endometrium*1|chrX*117576289*117576289*COSM1113642*C*A*1*endometrium*1	NA	NA	NA	NA	NA	D	PrD	NA	MODERATE	D	1	259	259	0	117	112	5	4.27
Non-user	IOB_23	chr1	2121260	G	A	AL590822.2	5UTR	ENSG00000269554:[ENST00000597060(c.-230G>A)]	NA	NA	NA	NA	NA	NA	NA	NA	NA	NA	MODIFIER	NA	NA	127	127	0	82	75	6	7.32
Non-user	IOB_23	chr1	2121260	G	A	C1orf86	INTRONIC	ENSG00000162585:[ENST00000400919(c.-476-40C>T)]	NA	NA	NA	NA	NA	NA	NA	NA	NA	NA	MODIFIER	NA	NA	127	127	0	82	75	6	7.32
Non-user	IOB_23	chr1	2121260	G	A	FAAP20	INTRONIC	199990:[NM_001256945.1(c.334+3818C>T)]	NA	NA	NA	NA	NA	NA	NA	NA	NA	NA	MODIFIER	NA	NA	127	127	0	82	75	6	7.32
Non-user	IOB_23	chr1	2519976	C	A	FAM213B	MISSENSE	ENSG00000157870:[ENST00000465233(c.445C>A,cCc>cAc):ENSP00000462185(p.Pro149His)]	NA	NA	NA	NA	NA	NA	NA	NA	NA	NA	MODIFIER	NA	NA	216	216	0	166	161	5	3.01
Non-user	IOB_23	chr1	2541281	C	A	MMEL1	NCT	ENSG00000142606:[ENST00000509374(n.122-11G>T)]	NA	NA	NA	NA	NA	NA	NA	NA	NA	NA	MODIFIER	NA	NA	113	113	0	107	102	5	4.67
Non-user	IOB_23	chr1	13839770	C	T	LRRC38	MISSENSE	ENSG00000162494:[ENST00000376085(c.319G>A,Gtg>Atg):ENSP00000365253(p.Val107Met)]	NA	NA	2.60E-03	2.94E-03	3.52E-03	2.60E-03	9.20E-03	D	PoD	NA	MODERATE	N	1	299	297	1	254	248	5	1.97
Non-user	IOB_23	chr1	13839770	C	T	RP4-597A16.2	EXONIC-NC	ENSG00000259961:[ENST00000563570(n.56C>T)]	NA	NA	2.60E-03	2.94E-03	3.52E-03	2.60E-03	9.20E-03	D	PoD	NA	MODERATE	N	1	299	297	1	254	248	5	1.97
Non-user	IOB_23	chr1	19597040	C	T	AKR7L	MISSENSE	ENSG00000211454:[ENST00000429712(c.412G>A,Gtg>Atg):ENSP00000411913(p.Val138Met)]	NA	NA	NA	4.46E-04	3.04E-04	NA	NA	NA	NA	NA	MODIFIER	NA	NA	75	75	0	68	62	6	8.82
Non-user	IOB_23	chr1	144874928	G	A	PDE4DIP	INTRONIC	ENSG00000178104:[ENST00000369359(c.5112-24C>T)]	NA	NA	NA	3.36E-02	2.94E-02	NA	NA	NA	NA	NA	MODIFIER	NA	NA	62	56	6	39	29	9	23.08
Non-user	IOB_23	chr1	157776914	T	C	FCRL1	INTRONIC-SS-ACR	ENSG00000163534:[ENST00000368175(n.67-2A>G)]	NA	NA	NA	NA	NA	NA	NA	NA	NA	NA	HIGH	NA	NA	58	58	0	39	33	6	15.38
Non-user	IOB_23	chr1	237777610	C	A	RYR2	MISSENSE	ENSG00000198626:[ENST00000542537(c.5134C>A,Ccc>Acc):ENSP00000443798(p.Pro1712Thr)]	NA	NA	NA	NA	NA	NA	NA	NA	PrD	NA	MODERATE	D	1	192	190	0	148	142	6	4.05
Non-user	IOB_23	chr10	46970751	G	C	SYT15	MISSENSE	ENSG00000204176:[ENST00000374323(c.62C>G,gCg>gGg):ENSP00000363443(p.Ala21Gly)]	NA	NA	1.60E-03	1.13E-02	1.44E-02	1.60E-03	4.10E-03	D_lc	BN	NA	MODERATE	NA	NA	23	22	1	25	19	6	24
Non-user	IOB_23	chr11	64029956	C	T	PLCB3	MISSENSE	ENSG00000149782:[ENST00000279230(c.2116C>T,Cgg>Tgg):ENSP00000279230(p.Arg706Trp)]	NA	chr11*64029956*64029956*COSM1676138*C*T*1*haematopoietic_and_lymphoid_tissue*1	NA	1.65E-05	0.00E+00	NA	NA	D	PrD	NA	MODERATE	D	1	234	233	1	166	159	5	3.01
Non-user	IOB_23	chr11	76427908	T	C	GUCY2EP	EXONIC-NC	390226:[NR_024042.1(n.579A>G)]	NA	NA	NA	NA	NA	NA	NA	NA	NA	NA	MODIFIER	NA	NA	212	211	0	158	153	5	3.16
Non-user	IOB_23	chr11	76427908	T	C	RP11-672A2.1	NCT	ENSG00000254761:[ENST00000525328(n.41+2704A>G)]	NA	NA	NA	NA	NA	NA	NA	NA	NA	NA	MODIFIER	NA	NA	212	211	0	158	153	5	3.16
Non-user	IOB_23	chr11	93430721	G	A	KIAA1731	SILENT	ENSG00000166004:[ENST00000325212(c.2643G>A,caG>caA):ENSP00000316681(p.Gln881(=))]	NA	NA	NA	NA	NA	NA	NA	NA	NA	NA	LOW	NA	0.297	171	170	1	155	149	5	3.23
Non-user	IOB_23	chr11	126316700	C	T	KIRREL3	MISSENSE	ENSG00000149571:[ENST00000525144(c.1079G>A,gGc>gAc):ENSP00000435466(p.Gly360Asp)]	NA	NA	NA	NA	NA	NA	NA	D	PrD	NA	MODERATE	D	1	149	147	1	113	106	6	5.31
Non-user	IOB_23	chr12	27627810	C	A	SMCO2	MISSENSE	ENSG00000165935:[ENST00000298876(c.326C>A,aCa>aAa):ENSP00000298876(p.Thr109Lys)]	NA	NA	NA	NA	NA	NA	NA	D	PoD	NA	MODERATE	N	0.003	143	143	0	106	101	5	4.72
Non-user	IOB_23	chr13	38154026	C	A	POSTN	MISSENSE	ENSG00000133110:[ENST00000379742(c.1632G>T,atG>atT):ENSP00000369066(p.Met544Ile)]	NA	NA	chr13*38154026*38154026*C*T*rs766008659*NA*NA*NA	NA	NA	NA	NA	D	BN	NA	MODERATE	D	1	226	226	0	165	159	5	3.03
Non-user	IOB_23	chr16	11541755	C	A	CTD-3088G3.8	INTRONIC	ENSG00000188897:[ENST00000344649(c.1361+35G>T)]	NA	NA	NA	NA	NA	NA	NA	NA	NA	NA	MODIFIER	NA	NA	86	86	0	70	64	6	8.57
Non-user	IOB_23	chr16	11541755	C	A	LOC400499	INTRONIC	400499:[XM_005255733.1(c.1454+35G>T)]	NA	NA	NA	NA	NA	NA	NA	NA	NA	NA	MODIFIER	NA	NA	86	86	0	70	64	6	8.57
Non-user	IOB_23	chr16	23490086	C	A	GGA2	NCT	ENSG00000103365:[ENST00000569182(n.344+118G>T)]	NA	NA	NA	NA	NA	NA	NA	NA	NA	NA	MODIFIER	NA	NA	128	127	0	116	111	5	4.31
Non-user	IOB_23	chr16	30035313	C	A	C16orf92	INTRONIC-SS-ACR-PRX	ENSG00000167194:[ENST00000300575(c.290-7C>A)]	NA	NA	NA	NA	NA	NA	NA	NA	NA	NA	LOW	NA	NA	218	218	0	174	169	5	2.87
Non-user	IOB_23	chr16	31121622	C	A	BCKDK	MISSENSE	ENSG00000103507:[ENST00000484226(c.493C>A,Cgt>Agt):ENSP00000457226(p.Arg165Ser)]	NA	NA	NA	NA	NA	NA	NA	NA	NA	NA	MODIFIER	NA	NA	109	106	1	89	83	5	5.62
Non-user	IOB_23	chr16	84695283	C	A	KLHL36	NONSENSE	ENSG00000135686:[ENST00000258157(c.1206C>A,tgC>tgA):ENSP00000258157(p.Cys402Ter)]	NA	NA	NA	NA	NA	NA	NA	NA	NA	NA	HIGH	NA	1	239	238	1	205	197	5	2.44
Non-user	IOB_23	chr17	5328662	T	C	CTC-524C5.2	EXONIC-NC	ENSG00000263272:[ENST00000575890(n.1735A>G)]	NA	NA	NA	7.68E-02	9.46E-02	NA	NA	NA	NA	NA	MODIFIER	NA	NA	59	57	2	39	33	6	15.38
Non-user	IOB_23	chr17	5328662	T	C	RPAIN	NCT	ENSG00000129197:[ENST00000570883(n.340-629T>C)]	NA	NA	NA	7.68E-02	9.46E-02	NA	NA	NA	NA	NA	MODIFIER	NA	NA	59	57	2	39	33	6	15.38
Non-user	IOB_23	chr17	10411335	G	T	CTC-297N7.11	NCT	ENSG00000272975:[ENST00000587182(n.156-59519G>T)]	NA	NA	NA	NA	NA	NA	NA	NA	NA	NA	MODIFIER	NA	NA	87	82	3	80	64	13	16.25
Non-user	IOB_23	chr17	10411335	G	T	MYH1	INTRONIC	ENSG00000109061:[ENST00000226207(c.1898-62C>A)]	NA	NA	NA	NA	NA	NA	NA	NA	NA	NA	MODIFIER	NA	NA	87	82	3	80	64	13	16.25
Non-user	IOB_23	chr17	10411335	G	T	MYHAS	NCT	100128560:[NR_125367.1(n.168-59519G>T)]	NA	NA	NA	NA	NA	NA	NA	NA	NA	NA	MODIFIER	NA	NA	87	82	3	80	64	13	16.25
Non-user	IOB_23	chr17	10411335	G	T	RP11-799N11.1	NCT	ENSG00000272736:[ENST00000581304(n.144-25306G>T)]	NA	NA	NA	NA	NA	NA	NA	NA	NA	NA	MODIFIER	NA	NA	87	82	3	80	64	13	16.25
Non-user	IOB_23	chr17	19451273	C	A	SLC47A1	INTRONIC	ENSG00000142494:[ENST00000436810(c.238-25C>A)]	NA	NA	chr17*19451273*19451273*C*T*rs895208029*NA*NA*NA	NA	NA	NA	NA	NA	NA	NA	MODIFIER	NA	NA	88	87	1	58	53	5	8.62
Non-user	IOB_23	chr17	61907778	C	A	PSMC5	MISSENSE	ENSG00000087191:[ENST00000584880(c.33C>A,Cag>Aag):ENSP00000464347(p.Gln12Lys)]	NA	NA	NA	NA	NA	NA	NA	D	PrD	NA	MODERATE	NA	NA	201	201	0	170	164	6	3.53
Non-user	IOB_23	chr18	2738482	C	T	RP11-703M24.5	NCT	ENSG00000266049:[ENST00000583546(n.371-46604G>A)]	NA	NA	NA	NA	NA	NA	NA	NA	PrD	NA	MODERATE	NA	NA	258	257	0	220	213	5	2.27
Non-user	IOB_23	chr18	2738482	C	T	SMCHD1	MISSENSE	ENSG00000101596:[ENST00000261598(c.3364C>T,Cgc>Tgc):ENSP00000261598(p.Arg1122Cys)]	NA	NA	NA	NA	NA	NA	NA	D	PrD	NA	MODERATE	NA	NA	258	257	0	220	213	5	2.27
Non-user	IOB_23	chr18	34387905	C	A	TPGS2	INTRONIC-SS-ACR-PRX	ENSG00000134779:[ENST00000587207(c.*115-8G>T)]	NA	NA	NA	8.25E-06	0.00E+00	NA	NA	NA	NA	NA	LOW	NA	NA	223	223	0	200	195	5	2.5
Non-user	IOB_23	chr19	19946164	C	T	NA	INTERGENIC	NA	NA	NA	4.19E-03	NA	NA	4.19E-03	0.00E+00	NA	NA	NA	MODIFIER	NA	NA	155	151	2	110	100	8	7.27
Non-user	IOB_23	chr19	55869544	C	A	CTD-2105E13.6	NONSENSE	ENSG00000267706:[ENST00000591954(c.1341G>T,Gag>Tag):ENSP00000467419(p.Glu448Ter)]	NA	NA	NA	NA	NA	NA	NA	NA	NA	NA	HIGH	NA	NA	146	146	0	125	119	6	4.8
Non-user	IOB_23	chr19	55869544	C	A	FAM71E2	NONSENSE	ENSG00000180043:[ENST00000424985(c.2692G>T,Gag>Tag):ENSP00000398617(p.Glu898Ter)]	NA	NA	NA	NA	NA	NA	NA	NA	NA	NA	HIGH	NA	NA	146	146	0	125	119	6	4.8
Non-user	IOB_23	chr2	72958340	C	A	EXOC6B	MISSENSE	ENSG00000144036:[ENST00000272427(c.362G>T,cGa>cTa):ENSP00000272427(p.Arg121Leu)]	NA	NA	chr2*72958340*72958340*C*T*rs567934007*0*1*0	NA	NA	NA	NA	NA	NA	NA	MODERATE	NA	1	295	293	0	235	225	6	2.55
Non-user	IOB_23	chr2	74599986	C	A	DCTN1	INTRONIC	ENSG00000204843:[ENST00000409868(c.402+69G>T)]	NA	NA	NA	NA	NA	NA	NA	NA	NA	NA	MODIFIER	NA	NA	123	123	0	87	80	5	5.75
Non-user	IOB_23	chr2	88874329	C	A	AC104134.2	EXONIC-NC	ENSG00000225420:[ENST00000413234(n.948C>A)]	NA	NA	NA	NA	NA	NA	NA	NA	NA	NA	MODERATE	NA	1	260	259	0	247	240	5	2.02
Non-user	IOB_23	chr2	88874329	C	A	EIF2AK3	MISSENSE	ENSG00000172071:[ENST00000415570(c.2309G>T,tGc>tTc):ENSP00000412076(p.Cys770Phe)]	NA	NA	NA	NA	NA	NA	NA	NA	NA	NA	MODERATE	NA	1	260	259	0	247	240	5	2.02
Non-user	IOB_23	chr2	152371326	C	A	NEB	INTRONIC	4703:[XM_005246618.1(c.13110+6G>T)]	NA	NA	NA	NA	NA	NA	NA	NA	NA	NA	LOW	NA	NA	159	159	0	105	99	5	4.76
Non-user	IOB_23	chr2	225659727	C	T	DOCK10	MISSENSE	ENSG00000135905:[ENST00000258390(c.5023G>A,Gag>Aag):ENSP00000258390(p.Glu1675Lys)]	NA	NA	2.00E-04	8.28E-06	6.06E-05	2.00E-04	1.00E-03	D	BN	NA	MODERATE	D	1	111	109	0	100	94	6	6
Non-user	IOB_23	chr2	228109627	T	G	AC097662.2	NCT	ENSG00000236432:[ENST00000606119(n.22-6737A>C)]	NA	NA	9.59E-03	7.22E-02	6.68E-03	9.58E-03	2.00E-03	NA	NA	NA	MODIFIER	NA	NA	82	80	0	57	52	5	8.77
Non-user	IOB_23	chr2	228109627	T	G	COL4A3	NCT	1285:[XR_241280.1(n.423-40T>G)]	NA	NA	9.59E-03	7.22E-02	6.68E-03	9.58E-03	2.00E-03	NA	NA	NA	MODIFIER	NA	NA	82	80	0	57	52	5	8.77
Non-user	IOB_23	chr20	57485867	C	A	GNAS	MISSENSE	ENSG00000087460:[ENST00000371102(c.3055C>A,Cag>Aag):ENSP00000360143(p.Gln1019Lys)]	NA	NA	NA	NA	NA	NA	NA	D	PrD	NA	MODERATE	D	1	174	174	0	116	111	5	4.31
Non-user	IOB_23	chr3	88104840	G	T	CGGBP1	MISSENSE	ENSG00000163320:[ENST00000309534(c.287C>A,gCg>gAg):ENSP00000381428(p.Ala96Glu)]	NA	NA	chr3*88104840*88104840*G*A*rs764743017*NA*NA*NA	NA	NA	NA	NA	T	BN	NA	MODERATE	U	1	199	199	0	175	170	5	2.86
Non-user	IOB_23	chr3	114070599	C	T	ZBTB20	MISSENSE	ENSG00000181722:[ENST00000474710(c.326G>A,cGc>cAc):ENSP00000419153(p.Arg109His)]	NA	NA	NA	NA	NA	NA	NA	T	PrD	NA	MODERATE	D	1	407	406	0	283	277	5	1.77
Non-user	IOB_23	chr3	119456261	C	A	MAATS1	MISSENSE	ENSG00000183833:[ENST00000273390(c.1393C>A,Cgc>Agc):ENSP00000273390(p.Arg465Ser)]	NA	NA	chr3*119456261*119456261*C*T*rs199600362*0*0.9998*0.0001997	NA	NA	NA	NA	NA	NA	NA	MODERATE	NA	0.21	290	289	0	251	244	6	2.39

Patel *et al.* , 2021. Whole-exome sequencing analysis of oral squamous cell carcinoma delineated by tobacco usage habits
Supplementary Table 4. List of somatic single nucleotide variants identified using whole exome sequencing of OSCC patients

Cohort	Sample	Chromosome	Genomic position	Reference allele	Alternate allele	Genesymbol	Variant type	Variant description	OncoMD	COSMIC	dbSNP	ExAC	ExAC_SAS	1000G	1000G_SAS	SIFT	Polyphen2	CONDEL	VEP	LRT	PhastCon7Way	Total reads in Normal	Reference read support in Normal	Alternative read support in Normal	Total reads in Tumor	Reference read support in Tumor	Alternate read support in Tumor	Alternate allele frequency in Tumor
Non-user	IOB_23	chr3	128721019	G	A	EFCC1	MISSENSE	ENSG00000114654:[ENST00000480450(c.548G>A,cGc>cAc):ENSP00000420075(p.Arg183His)]	NA	NA	NA	NA	NA	NA	NA	NA	NA	NA	MODIFIER	NA	NA	180	180	0	161	156	5	3.11
Non-user	IOB_23	chr3	128721019	G	A	KIAA1257	NCT	ENSG00000114656:[ENST00000510149(n.117+398C>T)]	NA	NA	NA	NA	NA	NA	NA	NA	NA	NA	MODIFIER	NA	NA	180	180	0	161	156	5	3.11
Non-user	IOB_23	chr4	76407668	C	T	RCHY1	3UTR	ENSG00000163743:[ENST00000324439(c.*80G>A)]	NA	NA	5.99E-04	NA	NA	5.99E-04	2.00E-03	NA	NA	NA	MODIFIER	NA	NA	187	187	0	183	177	5	2.73
Non-user	IOB_23	chr4	103682000	C	A	MANBA	MISSENSE	ENSG00000109323:[ENST00000226578(c.52G>T,Gcc>Tcc):ENSP00000226578(p.Ala18Ser)]	NA	NA	NA	NA	NA	NA	NA	NA	NA	NA	MODIFIER	NA	NA	102	102	0	92	87	5	5.43
Non-user	IOB_23	chr4	126239421	C	A	FAT4	MISSENSE	ENSG00000196159:[ENST00000394329(c.1855C>A,Cgc>Agc):ENSP00000377862(p.Arg619Ser)]	NA	chr4*126239421*126239421*COSM1050816*C*T*1*endometrium*1|chr4*126239421*126239421*COSM1050817*C*T*1*endometrium*1	chr4*126239421*126239421*C*T*rs202125547*0*0.9998*0.0001997	NA	NA	NA	NA	T	PoD	NA	MODERATE	U	1	217	216	0	155	150	5	3.23
Non-user	IOB_23	chr5	159478218	C	A	TTC1	NCT	ENSG00000113312:[ENST00000520274(n.527+28C>A)]	NA	NA	NA	NA	NA	NA	NA	NA	NA	NA	MODIFIER	NA	NA	107	106	0	111	104	5	4.5
Non-user	IOB_23	chr6	6251194	C	A	F13A1	NCT	ENSG00000124491:[ENST00000479211(n.557-32G>T)]	NA	NA	NA	NA	NA	NA	NA	NA	NA	NA	MODIFIER	NA	NA	220	220	0	183	178	5	2.73
Non-user	IOB_23	chr6	38758142	C	A	DNAH8	SILENT	ENSG00000124721:[ENST00000359357(c.2091C>A,gtC>gtA):ENSP00000352312(p.Val697(=))]	NA	chr6*38758142*38758142*COSM173820*C*T*1*large_intestine*1|chr6*38758142*38758142*COSM173821*C*T*1*large_intestine*1	chr6*38758142*38758142*C*T*rs748253698*NA*NA*NA	NA	NA	NA	NA	NA	NA	NA	LOW	NA	NA	84	84	0	102	97	5	4.9
Non-user	IOB_23	chr7	73151667	C	A	ABHD11	MISSENSE	ENSG00000106077:[ENST00000437891(c.334G>T,caG>caT):ENSP00000410536(p.Gln112His)]	NA	NA	NA	NA	NA	NA	NA	NA	NA	NA	MODIFIER	NA	NA	165	165	0	143	138	5	3.5
Non-user	IOB_23	chr7	158824570	G	T	VIPR2	NCT	7434:[NR_130758.1(n.1288-10C>A)]	NA	NA	NA	NA	NA	NA	NA	NA	NA	NA	MODIFIER	NA	NA	154	154	0	139	133	5	3.6
Non-user	IOB_23	chr9	36087915	G	A	RECK	MISSENSE	ENSG00000122707:[ENST00000377966(c.862G>A,Gct>Act):ENSP00000367202(p.Ala288Thr)]	NA	NA	chr9*36087915*36087915*G*T*rs763312722*NA*NA*NA	NA	NA	NA	NA	NA	NA	NA	MODERATE	NA	1	168	168	0	128	123	5	3.91
Non-user	IOB_23	chr9	68415317	C	T	MIR4477A	EXONIC-NC	ENSG00000266017:[ENST00000581659(n.10C>T)]	NA	NA	NA	5.55E-03	8.93E-03	NA	NA	NA	NA	NA	MODIFIER	NA	NA	279	278	1	266	253	11	4.14
Non-user	IOB_23	chr9	113141703	A	G	SVEP1	SILENT	ENSG00000165124:[ENST00000374469(c.10263T>C,agT>agC):ENSP00000363593(p.Ser3421(=))]	NA	NA	NA	NA	NA	NA	NA	NA	NA	NA	LOW	NA	1	223	222	0	169	163	5	2.96
Non-user	IOB_23	chrX	71427424	C	T	ERCC6L	MISSENSE	ENSG00000186871:[ENST00000334463(c.1193G>A,cGc>cAc):ENSP00000334675(p.Arg398His)]	NA	chrX*71427424*71427424*COSM195473*C*T*1*large_intestine*1	NA	1.94E-04	0.00E+00	NA	NA	D	BN	NA	MODERATE	NA	NA	207	207	0	164	159	5	3.05
Non-user	IOB_23	chrX	71427424	C	T	PIN4	INTRONIC	ENSG00000102309:[ENST00000439980(c.258+10670C>T)]	NA	chrX*71427424*71427424*COSM195473*C*T*1*large_intestine*1	NA	1.94E-04	0.00E+00	NA	NA	D	BN	NA	MODERATE	NA	NA	207	207	0	164	159	5	3.05
Non-user	IOB_23	chrX	127185519	C	A	ACTRT1	MISSENSE	ENSG00000123165:[ENST00000371124(c.667G>T,Gcc>Tcc):ENSP00000360165(p.Ala223Ser)]	NA	chrX*127185519*127185519*COSM1331265*C*T*1*ovary*1	chrX*127185519*127185519*C*T*rs148603135*1*0.9992*0.0007947	NA	NA	NA	NA	T	BN	NA	MODERATE	N	0.806	214	214	0	154	149	5	3.25
Non-user	IOB_24	chr1	898539	C	A	KLHL17	MISSENSE	ENSG00000187961:[ENST00000338591(c.1093C>A,Cgc>Agc):ENSP00000343930(p.Arg365Ser)]	NA	NA	chr1*898539*898539*C*T*rs748711259*NA*NA*NA	NA	NA	NA	NA	NA	NA	NA	MODERATE	NA	0.958	271	271	0	223	117	106	47.53
Non-user	IOB_24	chr1	986161	G	A	AGRN	MISSENSE	ENSG00000188157:[ENST00000379370(c.5197G>A,Ggc>Agc):ENSP00000368678(p.Gly1733Ser)]	NA	NA	NA	1.12E-04	0.00E+00	NA	NA	T	PrD	NA	MODERATE	NA	NA	35	34	1	22	13	9	40.91
Non-user	IOB_24	chr1	1139394	C	T	TNFRSF18	SILENT	8784:[XM_005244814.1(c.621G>A,gcG>gcA):XP_005244871.1(p.Ala207(=))]	NA	NA	NA	1.05E-05	0.00E+00	NA	NA	NA	NA	NA	MODIFIER	NA	NA	173	173	0	140	57	82	58.57
Non-user	IOB_24	chr1	1221409	G	A	SCNN1D	MISSENSE	ENSG00000162572:[ENST00000338555(c.170G>A,cGg>cAg):ENSP00000339504(p.Arg57Gln)]	NA	NA	NA	2.88E-03	0.00E+00	NA	NA	T	BN	NA	MODERATE	NA	NA	357	355	0	337	177	159	47.18
Non-user	IOB_24	chr1	1229776	G	A	ACAP3	INTRONIC	116983:[XM_005244716.1(c.2013+6C>T)]	NA	NA	NA	NA	NA	NA	NA	NA	NA	NA	LOW	NA	NA	466	465	1	326	179	145	44.48
Non-user	IOB_24	chr1	1254503	C	G	CPSF3L	MISSENSE	ENSG00000127054:[ENST00000434694(c.602G>C,cGg>cCg):ENSP00000411233(p.Arg201Pro)]	NA	NA	1.06E-02	1.34E-02	5.61E-02	1.06E-02	5.42E-02	NA	NA	NA	MODIFIER	NA	NA	261	261	0	160	93	67	41.88
Non-user	IOB_24	chr1	1254503	C	G	INTS11	INTRONIC	54973:[NM_001256462.1(c.140-3509G>C)]	NA	NA	1.06E-02	1.34E-02	5.61E-02	1.06E-02	5.42E-02	NA	NA	NA	MODIFIER	NA	NA	261	261	0	160	93	67	41.88
Non-user	IOB_24	chr1	1271696	G	C	DVL1	SILENT	ENSG00000107404:[ENST00000378891(c.1839C>G,gcC>gcG):ENSP00000368169(p.Ala613(=))]	NA	NA	1.40E-03	2.01E-04	9.27E-04	1.40E-03	7.20E-03	NA	NA	NA	LOW	NA	NA	238	238	0	213	97	114	53.52
Non-user	IOB_24	chr1	1275200	G	A	DVL1	INTRONIC-SS-ACR-PRX	ENSG00000107404:[ENST00000378888(c.910-8C>T)]	NA	NA	1.06E-02	6.57E-03	4.63E-02	1.06E-02	5.42E-02	NA	NA	NA	MODIFIER	NA	NA	342	340	0	274	147	125	45.62
Non-user	IOB_24	chr1	1575836	C	G	CDK11B	INTRONIC	984:[NM_033487.1(c.308-23G>C)]	NA	NA	NA	4.01E-01	3.99E-01	NA	NA	NA	NA	NA	MODIFIER	NA	NA	136	136	0	145	93	52	35.86
Non-user	IOB_24	chr1	1599940	C	T	SLC35E2B	INTRONIC	728661:[XM_005244793.1(c.587-29G>A)]	NA	NA	chr1*1599940*1599940*C*G,T*rs61776767*NA*NA*NA	2.18E-01	1.86E-01	NA	NA	NA	NA	NA	MODIFIER	NA	NA	106	104	2	125	101	22	17.6
Non-user	IOB_24	chr1	2267964	G	A	MORN1	SILENT	ENSG00000116151:[ENST00000378531(c.1194C>T,ggC>ggT):ENSP00000367792(p.Gly398(=))]	NA	NA	2.60E-03	1.72E-03	1.21E-02	2.60E-03	1.33E-02	NA	NA	NA	MODIFIER	NA	NA	64	64	0	58	31	26	44.83
Non-user	IOB_24	chr1	2491306	G	A	TNFRSF14	MISSENSE	ENSG00000157873:[ENST00000355716(c.349G>A,Gcc>Acc):ENSP00000347948(p.Ala117Thr)]	NA	NA	2.12E-02	1.59E-02	1.77E-02	2.12E-02	1.64E-02	T	BN	NA	MODERATE	NA	NA	389	388	0	323	175	146	45.2
Non-user	IOB_24	chr1	2576959	C	T	TTC34	MISSENSE	ENSG00000215912:[ENST00000401095(c.731G>A,gGg>gAg):ENSP00000383873(p.Gly244Glu)]	NA	NA	2.82E-02	1.69E-02	1.83E-02	2.82E-02	1.53E-02	T	BN	NA	MODERATE	N	0	29	29	0	32	19	12	37.5
Non-user	IOB_24	chr1	3388291	C	A	ARHGEF16	MISSENSE	ENSG00000130762:[ENST00000413250(c.70C>A,Cag>Aag):ENSP00000408887(p.Gln24Lys)]	NA	NA	.	2.82E-03	6.29E-03	NA	NA	NA	NA	NA	MODIFIER	NA	NA	181	179	0	145	86	59	40.69
Non-user	IOB_24	chr1	3544187	C	T	TPRG1L	SILENT	ENSG00000158109:[ENST00000344579(c.417C>T,ggC>ggT):ENSP00000339714(p.Gly139(=))]	NA	NA	1.42E-02	2.44E-02	1.96E-02	1.42E-02	2.04E-02	NA	NA	NA	LOW	NA	NA	125	124	0	85	40	45	52.94
Non-user	IOB_24	chr1	3731959	C	T	CEP104	3UTR	ENSG00000116198:[ENST00000378230(c.*7G>A)]	NA	NA	3.02E-02	1.03E-01	5.51E-02	3.02E-02	2.45E-02	NA	NA	NA	MODIFIER	NA	NA	209	209	0	172	95	75	43.6
Non-user	IOB_24	chr1	3732729	A	G	CEP104	INTRONIC	ENSG00000116198:[ENST00000378230(c.2662+115T>C)]	NA	NA	8.59E-03	NA	NA	8.59E-03	1.12E-02	NA	NA	NA	MODIFIER	NA	NA	52	50	1	25	15	10	40
Non-user	IOB_24	chr1	3732904	C	T	CEP104	MISSENSE	ENSG00000116198:[ENST00000438539(c.492G>A,Ggc>Agc):ENSP00000395283(p.Gly165Ser)]	NA	NA	NA	NA	NA	NA	NA	D	PrD	NA	MODERATE	D	1	97	96	0	75	42	33	44
Non-user	IOB_24	chr1	3784549	C	T	DFFB	NONSENSE	ENSG00000169598:[ENST00000338895(c.442C>T,Cga>Tga):ENSP00000339524(p.Arg148Ter)]	NA	NA	3.99E-04	3.05E-04	2.18E-03	3.99E-04	2.00E-03	NA	NA	NA	MODIFIER	NA	NA	178	177	0	139	72	66	47.48
Non-user	IOB_24	chr1	6202423	C	A	CHD5	INTRONIC	ENSG00000116254:[ENST00000378021(c.-1194-35G>T)]	NA	NA	4.35E-02	5.54E-02	1.24E-02	4.35E-02	2.00E-03	NA	NA	NA	MODIFIER	NA	NA	292	289	1	205	124	81	39.51
Non-user	IOB_24	chr1	6273206	C	T	RNF207	MISSENSE	ENSG00000158286:[ENST00000377939(c.1615C>T,Cgc>Tgc):ENSP00000367173(p.Arg539Cys)]	NA	NA	9.39E-03	1.54E-02	1.06E-02	9.38E-03	1.33E-02	D	BN	NA	MODERATE	D	1	263	262	0	220	123	96	43.64
Non-user	IOB_24	chr1	6355066	A	G	ACOT7	INTRONIC	ENSG00000097021:[ENST00000545482(c.398-26T>C)]	NA	NA	2.44E-02	3.82E-02	4.01E-02	2.44E-02	4.70E-02	NA	NA	NA	MODIFIER	NA	NA	149	149	0	95	52	41	43.16
Non-user	IOB_24	chr1	6409979	A	G	ACOT7	INTRONIC	ENSG00000097021:[ENST00000473466(c.-4-53T>C)]	NA	NA	4.71E-02	NA	NA	4.71E-02	5.62E-02	NA	NA	NA	MODIFIER	NA	NA	98	98	0	73	34	39	53.42
Non-user	IOB_24	chr1	6637026	G	A	TAS1R1	MISSENSE	ENSG00000173662:[ENST00000415267(c.505G>A,tGt>tAt):ENSP00000408448(p.Cys169Tyr)]	NA	NA	NA	NA	NA	NA	NA	NA	NA	NA	MODIFIER	NA	NA	220	220	0	160	96	63	39.38
Non-user	IOB_24	chr1	6696319	A	G	DNAJC11	INTRONIC	ENSG00000007923:[ENST00000377573(c.1255-13T>C)]	NA	NA	5.39E-03	2.37E-03	1.76E-02	5.39E-03	2.76E-02	NA	NA	NA	MODIFIER	NA	NA	123	120	0	128	64	64	50
Non-user	IOB_24	chr1	9118334	G	A	SLC2A5	INTRONIC	6518:[NM_001328620.1(c.-61-25C>T)]	NA	NA	2.00E-04	1.70E-04	6.18E-04	2.00E-04	0.00E+00	NA	NA	NA	MODIFIER	NA	NA	150	150	0	102	52	50	49.02
Non-user	IOB_24	chr1	9188774	A	G	GPR157	MISSENSE	ENSG00000180758:[ENST00000377411(c.313T>C,Tac>Cac):ENSP00000366628(p.Tyr105His)]	NA	NA	NA	1.77E-05	1.23E-04	NA	NA	D	PoD	NA	MODERATE	D	1	656	656	0	554	295	257	46.39
Non-user	IOB_24	chr1	11155886	T	G	EXOSC10	MISSENSE	ENSG00000171824:[ENST00000304457(c.301A>C,Agt>Cgt):ENSP00000307307(p.Ser101Arg)]	NA	NA	1.80E-03	1.97E-03	1.42E-02	1.80E-03	9.20E-03	NA	NA	NA	MODERATE	NA	1	287	286	1	238	122	115	48.32
Non-user	IOB_24	chr1	11796240	C	T	AGTRAP	EXONIC-NC	ENSG00000177674:[ENST00000491346(n.42C>T)]	NA	NA	1.16E-02	3.45E-02	1.16E-02	1.16E-02	9.20E-03	NA	NA	NA	MODIFIER	NA	NA	50	50	0	34	21	12	35.29
Non-user	IOB_24	chr1	12321247	G	T	VPS13D	INTRONIC	ENSG00000048707:[ENST00000356315(c.1414+41G>T)]	NA	NA	2.44E-02	3.90E-02	4.59E-02	2.44E-02	4.50E-02	NA	NA	NA	MODIFIER	NA	NA	33	33	0	39	21	18	46.15
Non-user	IOB_24	chr1	12779563	C	T	AADACL3	SILENT	ENSG00000188984:[ENST00000359318(c.84C>T,ccC>ccT):ENSP00000352268(p.Pro28(=))]	NA	NA	2.80E-02	2.28E-02	5.55E-02	2.80E-02	5.73E-02	NA	NA	NA	MODIFIER	NA	NA	205	202	0	144	83	61	42.36
Non-user	IOB_24	chr1	12785295	C	T	AADACL3	MISSENSE	ENSG00000188984:[ENST00000359318(c.385C>T,Cgg>Tgg):ENSP00000352268(p.Arg129Trp)]	NA	NA	1.38E-02	1.05E-02	1.14E-02	1.38E-02	5.10E-03	D	PrD	NA	MODERATE	N	0	177	175	0	123	61	61	49.59
Non-user	IOB_24	chr1	12835168	C	A	PRAMEF12	MISSENSE	ENSG00000116726:[ENST00000357726(c.158C>A,aCa>aAa):ENSP00000350358(p.Thr53Lys)]	NA	NA	3.30E-02	3.35E-02	5.54E-02	3.29E-02	6.13E-02	T	BN	NA	MODERATE	N	0	149	149	0	131	74	57	43.51
Non-user	IOB_24	chr1	12837619	A	G	PRAMEF12	SILENT	ENSG00000116726:[ENST00000357726(c.1329A>G,ttA>ttG):ENSP00000350358(p.Leu443(=))]	NA	NA	NA	2.98E-02	5.15E-02	NA	NA	NA	NA	NA	LOW	NA	0	101	100	0	88	74	13	14.77
Non-user	IOB_24	chr1	12837664	C	T	PRAMEF12	SILENT	ENSG00000116726:[ENST00000357726(c.1374C>T,cgC>cgT):ENSP00000350358(p.Arg458(=))]	NA	NA	NA	2.58E-02	4.58E-02	NA	NA	NA	NA	NA	LOW	NA	NA	93	92	0	100	85	15	15
Non-user	IOB_24	chr1	12837669	G	T	PRAMEF12	MISSENSE	ENSG00000116726:[ENST00000357726(c.1379G>T,gGc>gTc):ENSP00000350358(p.Gly460Val)]	NA	NA	NA	2.55E-02	4.48E-02	NA	NA	D	PrD	NA	MODERATE	N	0	88	88	0	100	85	15	15
Non-user	IOB_24	chr1	12837720	G	A	PRAMEF12	MISSENSE	ENSG00000116726:[ENST00000357726(c.1430G>A,tGc>tAc):ENSP00000350358(p.Cys477Tyr)]	NA	NA	NA	2.80E-02	4.80E-02	NA	NA	D_lc	BN	NA	MODERATE	NA	NA	75	75	0	80	68	9	11.25
Non-user	IOB_24	chr1	12837728	G	A	PRAMEF12	MISSENSE	ENSG00000116726:[ENST00000357726(c.1438G>A,Ggt>Agt):ENSP00000350358(p.Gly480Ser)]	NA	NA	NA	2.84E-02	4.89E-02	NA	NA	T_lc	BN	NA	MODERATE	NA	NA	76	76	0	80	68	10	12.5
Non-user	IOB_24	chr1	12919022	C	T	PRAMEF2	MISSENSE	ENSG00000120952:[ENST00000240189(c.158C>T,aCg>aTg):ENSP00000240189(p.Thr53Met)]	NA	NA	NA	2.55E-02	4.26E-02	NA	NA	T	BN	NA	MODERATE	N	0	51	51	0	27	22	5	18.52
Non-user	IOB_24	chr1	12919041	G	A	PRAMEF2	NONSENSE	ENSG00000120952:[ENST00000240189(c.177G>A,tgG>tgA):ENSP00000240189(p.Trp59Ter)]	NA	NA	NA	2.57E-02	4.47E-02	NA	NA	NA	NA	NA	HIGH	NA	0.979	56	56	0	32	26	6	18.75
Non-user	IOB_24	chr1	12919068	G	A	PRAMEF2	SILENT	ENSG00000120952:[ENST00000240189(c.204G>A,tcG>tcA):ENSP00000240189(p.Ser68(=))]	NA	NA	2.04E-02	3.06E-02	4.97E-02	2.04E-02	3.89E-02	NA	NA	NA	LOW	NA	NA	63	62	0	31	25	6	19.35
Non-user	IOB_24	chr1	12919095	A	G	PRAMEF2	SILENT	ENSG00000120952:[ENST00000240189(c.231A>G,ccA>ccG):ENSP00000240189(p.Pro77(=))]	NA	NA	3.20E-02	3.33E-02	5.48E-02	3.19E-02	5.93E-02	NA	NA	NA	LOW	NA	NA	55	55	0	27	21	6	22.22
Non-user	IOB_24	chr1	13183472	G	A	HNRNPCL2	MISSENSE	440563:[NM_001136561.3(c.401C>T,cCt>cTt):NP_001130033.3(p.Pro134Leu)]	NA	NA	NA	3.90E-02	4.65E-02	NA	NA	D	PoD	NA	MODERATE	NA	NA	243	242	0	209	177	31	14.83
Non-user	IOB_24	chr1	13183472	G	A	HNRNPCP5	EXONIC-NC	ENSG00000179412:[ENST00000449855(n.644C>T)]	NA	NA	NA	3.90E-02	4.65E-02	NA	NA	D	PoD	NA	MODERATE	NA	NA	243	242	0	209	177	31	14.83
Non-user	IOB_24	chr1	14049383	G	T	PRDM2	INTRONIC	7799:[XM_005245996.1(c.-213-8112G>T)]	NA	NA	3.87E-02	3.36E-02	1.24E-01	3.87E-02	1.51E-01	NA	NA	NA	MODIFIER	NA	NA	64	64	0	59	37	22	37.29
Non-user	IOB_24	chr1	14057478	C	T	PRDM2	INTRONIC	7799:[XM_005245995.1(c.40-17C>T)]	NA	NA	3.87E-02	3.22E-02	1.25E-01	3.87E-02	1.51E-01	NA	NA	NA	MODIFIER	NA	NA	97	91	0	77	38	39	50.65
Non-user	IOB_24	chr1	14105049	C	A	PRDM2	MISSENSE	ENSG00000116731:[ENST00000343137(c.156C>A,gaC>gaA):ENSP00000341621(p.Asp52Glu)]	NA	NA	3.20E-02	2.52E-02	8.89E-02	3.19E-02	1.22E-01	NA	NA	NA	MODIFIER	NA	NA	336	333	2	255	150	104	40.78
Non-user	IOB_24	chr1	14105382	T	C	PRDM2	SILENT	ENSG00000116731:[ENST00000343137(c.489T>C,caT>caC):ENSP00000341621(p.His163(=))]	NA	NA	3.20E-02	2.51E-02	8.88E-02	3.19E-02	1.22E-01	NA	NA	NA	LOW	NA	0.895	371	369	0	301	163	137	45.51
Non-user	IOB_24	chr1	14105639	G	A	PRDM2	MISSENSE	ENSG00000116731:[ENST00000343137(c.746G>A,aGt>aAt):ENSP00000341621(p.Ser249Asn)]	NA	NA	3.95E-02	3.02E-02	1.23E-01	3.95E-02	1.51E-01	NA	NA	NA	MODERATE	NA	0	134	134	0	99	50	49	49.49
Non-user	IOB_24	chr1	14105856	A	G	PRDM2	SILENT	ENSG00000116731:[ENST00000343137(c.963A>G,gaA>gaG):ENSP00000341621(p.Glu321(=))]	NA	NA	3.20E-02	2.52E-02	8.89E-02	3.19E-02	1.22E-01	NA	NA	NA	MODIFIER	NA	NA	356	353	1	240	121	117	48.75
Non-user	IOB_24	chr1	14105946	T	C	PRDM2	SILENT	ENSG00000116731:[ENST00000235372(c.1656T>C,gaT>gaC):ENSP00000235372(p.Asp552(=))]	NA	NA	3.77E-02	2.99E-02	1.23E-01	3.77E-02	1.51E-01	NA	NA	NA	LOW	NA	0	209	209	0	139	67	70	50.36
Non-user	IOB_24	chr1	14106330	C	T	PRDM2	SILENT	ENSG00000116731:[ENST00000235372(c.2040C>T,caC>caT):ENSP00000235372(p.His680(=))]	NA	NA	3.77E-02	2.98E-02	1.23E-01	3.77E-02	1.51E-01	NA	NA	NA	LOW	NA	0.177	189	188	0	159	92	67	42.14
Non-user	IOB_24	chr1	14107135	C	G	PRDM2	MISSENSE	ENSG00000116731:[ENST00000343137(c.2242C>G,Cct>Gct):ENSP00000341621(p.Pro748Ala)]	NA	NA	3.20E-02	2.52E-02	9.13E-02	3.19E-02	1.22E-01	T	BN	NA	MODERATE	D	0.135	244	244	0	217	126	88	40.55
Non-user	IOB_24	chr1	14107164	C	T	PRDM2	SILENT	ENSG00000116731:[ENST00000343137(c.2271C>T,tcC>tcT):ENSP00000341621(p.Ser757(=))]	NA	NA	3.20E-02	2.53E-02	9.60E-02	3.19E-02	1.22E-01	NA	NA	NA	MODIFIER	NA	NA	231	231	0	195	115	79	40.51

Patel *et al.* , 2021. Whole-exome sequencing analysis of oral squamous cell carcinoma delineated by tobacco usage habits
Supplementary Table 4. List of somatic single nucleotide variants identified using whole exome sequencing of OSCC patients

Cohort	Sample	Chromosome	Genomic position	Reference allele	Alternate allele	Genesymbol	Variant type	Variant description	OncoMD	COSMIC	dbSNP	ExAC	ExAC_SAS	1000G	1000G_SAS	SIFT	Polyphen2	CONDEL	VEP	LRT	PhastCon7Way	Total reads in Normal	Reference read support in Normal	Alternative read support in Normal	Total reads in Tumor	Reference read support in Tumor	Alternate read support in Tumor	Alternate allele frequency in Tumor
Non-user	IOB_24	chr1	14107764	A	G	PRDM2	SILENT	ENSG00000116731:[ENST00000235372(c.3474A>G,gaA>gaG):ENSP00000235372(p.Glu1158(=))]	NA	NA	1.92E-02	7.53E-03	4.76E-02	1.92E-02	7.57E-02	NA	NA	NA	LOW	NA	0.981	245	244	0	176	89	87	49.43
Non-user	IOB_24	chr1	14108988	A	G	PRDM2	SILENT	ENSG00000116731:[ENST00000343137(c.4095A>G,aaA>aaG):ENSP00000341621(p.Lys1365(=))]	NA	NA	3.20E-02	2.53E-02	8.99E-02	3.19E-02	1.22E-01	NA	NA	NA	LOW	NA	0.994	256	256	0	193	95	97	50.26
Non-user	IOB_24	chr1	14109667	T	C	PRDM2	INTRONIC	ENSG00000116731:[ENST00000343137(c.4433+341T>C)]	NA	NA	3.77E-02	3.92E-02	1.37E-01	3.77E-02	1.51E-01	NA	NA	NA	MODIFIER	NA	NA	77	76	0	38	18	20	52.63
Non-user	IOB_24	chr1	14109735	A	G	PRDM2	INTRONIC	7799:[XM_005245994.1(c.5033+409A>G)]	NA	NA	NA	1.64E-05	9.69E-05	NA	NA	NA	NA	NA	MODIFIER	NA	NA	40	40	0	24	11	13	54.17
Non-user	IOB_24	chr1	14113132	G	A	PRDM2	INTRONIC	7799:[XM_005245996.1(c.4814+3806G>A)]	NA	NA	3.20E-02	NA	NA	3.19E-02	1.22E-01	NA	NA	NA	MODIFIER	NA	NA	151	151	0	163	83	80	49.08
Non-user	IOB_24	chr1	15287168	C	T	KAZN	INTRONIC	ENSG00000189337:[ENST00000361144(c.209-12C>T)]	NA	NA	4.61E-02	5.30E-02	5.34E-02	4.61E-02	4.81E-02	NA	NA	NA	MODIFIER	NA	NA	105	105	0	58	24	33	56.9
Non-user	IOB_24	chr1	16051930	A	G	PLEKHM2	SILENT	ENSG00000116786:[ENST00000375799(c.831A>G,gcA>gcG):ENSP00000364956(p.Ala277(=))]	NA	NA	6.39E-03	8.54E-03	2.36E-02	6.39E-03	3.27E-02	NA	NA	NA	LOW	NA	NA	122	122	0	79	40	39	49.37
Non-user	IOB_24	chr1	16051930	A	G	RP11-288I21.1	NCT	ENSG00000237938:[ENST00000453804(n.212-2148T>C)]	NA	NA	6.39E-03	8.54E-03	2.36E-02	6.39E-03	3.27E-02	NA	NA	NA	LOW	NA	NA	122	122	0	79	40	39	49.37
Non-user	IOB_24	chr1	16862638	A	G	NA	INTERGENIC	NA	NA	NA	NA	2.21E-01	2.56E-01	NA	NA	NA	NA	NA	MODIFIER	NA	NA	414	413	0	342	286	56	16.37
Non-user	IOB_24	chr1	17026004	C	G	ESPNP	EXONIC-NC	ENSG00000268869:[ENST00000270691(n.1445G>C)]	NA	NA	NA	4.43E-01	4.33E-01	NA	NA	NA	NA	NA	MODIFIER	NA	NA	61	61	0	64	47	17	26.56
Non-user	IOB_24	chr1	17030590	G	A	ESPNP	EXONIC-NC	ENSG00000268869:[ENST00000270691(n.746C>T)]	NA	NA	NA	3.52E-01	3.07E-01	NA	NA	NA	NA	NA	MODIFIER	NA	NA	197	195	1	221	163	57	25.79
Non-user	IOB_24	chr1	17046549	G	A	ESPNP	EXONIC-NC	ENSG00000268869:[ENST00000270691(n.104C>T)]	NA	NA	3.20E-03	4.50E-01	4.30E-01	3.19E-03	0.00E+00	NA	NA	NA	MODIFIER	NA	NA	133	133	0	142	101	40	28.17
Non-user	IOB_24	chr1	17085908	C	G	CROCC	NCT	ENSG00000058453:[ENST00000466256(n.125+19016C>G)]	NA	NA	NA	2.78E-01	2.38E-01	NA	NA	NA	NA	NA	MODIFIER	NA	NA	66	64	1	61	49	12	19.67
Non-user	IOB_24	chr1	17085908	C	G	LOC102724562	NCT	102724562:[NR_135824.1(n.338-14G>C)]	NA	NA	NA	2.78E-01	2.38E-01	NA	NA	NA	NA	NA	MODIFIER	NA	NA	66	64	1	61	49	12	19.67
Non-user	IOB_24	chr1	17085908	C	G	MST1L	INTRONIC	11223:[NM_001271733.1(c.927-14G>C)]	NA	NA	NA	2.78E-01	2.38E-01	NA	NA	NA	NA	NA	MODIFIER	NA	NA	66	64	1	61	49	12	19.67
Non-user	IOB_24	chr1	17256850	C	T	CROCC	NCT-SS-PRX	ENSG00000058453:[ENST00000467938(n.364+8C>T)]	NA	NA	1.54E-02	2.39E-02	5.13E-02	1.54E-02	3.68E-02	NA	NA	NA	LOW	NA	NA	20	19	0	13	6	7	53.85
Non-user	IOB_24	chr1	19062281	C	A	PAX7	SILENT	ENSG00000009709:[ENST00000375375(c.1311C>A,ccC>ccA):ENSP00000364524(p.Pro437(=))]	NA	NA	chr1*19062281*19062281*C*T*rs764412438*NA*NA*NA	NA	NA	NA	NA	NA	NA	NA	LOW	NA	NA	198	198	0	134	63	70	52.24
Non-user	IOB_24	chr1	19235138	C	T	IFFO2	MISSENSE	ENSG00000169991:[ENST00000455833(c.1471G>A,Gtg>Atg):ENSP00000387941(p.Val491Met)]	NA	NA	1.92E-02	1.74E-02	3.63E-02	1.92E-02	5.52E-02	T_lc	BN	NA	MODERATE	NA	NA	126	126	0	83	41	42	50.6
Non-user	IOB_24	chr1	19235138	C	T	RP13-279N23.2	MISSENSE	ENSG00000255275:[ENST00000494072(c.806G>A,Gtg>Atg):ENSP00000455952(p.Val270Met)]	NA	NA	1.92E-02	1.74E-02	3.63E-02	1.92E-02	5.52E-02	T_lc	BN	NA	MODERATE	NA	NA	126	126	0	83	41	42	50.6
Non-user	IOB_24	chr1	19237015	A	G	IFFO2	INTRONIC	ENSG00000169991:[ENST00000455833(c.1318-49T>C)]	NA	NA	2.54E-02	1.90E-02	4.22E-02	2.54E-02	5.73E-02	NA	NA	NA	MODIFIER	NA	NA	115	115	0	98	51	45	45.92
Non-user	IOB_24	chr1	19237015	A	G	RP13-279N23.2	INTRONIC	ENSG00000255275:[ENST00000494072(c.653-49T>C)]	NA	NA	2.54E-02	1.90E-02	4.22E-02	2.54E-02	5.73E-02	NA	NA	NA	MODIFIER	NA	NA	115	115	0	98	51	45	45.92
Non-user	IOB_24	chr1	19481906	T	C	UBR4	INTRONIC	ENSG00000127481:[ENST00000375217(c.6300+29A>G)]	NA	NA	2.34E-02	3.70E-02	1.71E-02	2.34E-02	1.23E-02	NA	NA	NA	MODIFIER	NA	NA	68	67	0	56	30	25	44.64
Non-user	IOB_24	chr1	19488276	T	C	UBR4	INTRONIC	ENSG00000127481:[ENST00000417040(c.1157+45A>G)]	NA	NA	chr1*19488276*19488276*T*A,C*rs776831651*NA*NA*NA	1.08E-04	7.91E-04	NA	NA	NA	NA	NA	MODIFIER	NA	NA	282	280	0	258	145	111	43.02
Non-user	IOB_24	chr1	21167565	T	C	EIF4G3	INTRONIC	ENSG00000075151:[ENST00000374935(c.2881-44A>G)]	NA	NA	1.14E-02	4.85E-03	1.57E-02	1.14E-02	1.94E-02	NA	NA	NA	MODIFIER	NA	NA	117	116	0	80	40	40	50
Non-user	IOB_24	chr1	21268143	T	C	EIF4G3	MISSENSE	ENSG00000075151:[ENST00000264211(c.1336A>G,Atc>Gtc):ENSP00000264211(p.Ile446Val)]	NA	NA	3.20E-03	2.55E-03	1.42E-02	3.19E-03	1.64E-02	T_lc	BN	NA	MODERATE	N	0.998	146	146	0	114	64	50	43.86
Non-user	IOB_24	chr1	21798208	G	A	NBPF3	MISSENSE	ENSG00000142794:[ENST00000454000(c.383G>A,cGg>cAg):ENSP00000415711(p.Arg128Gln)]	NA	NA	2.96E-02	1.08E-02	1.39E-02	2.96E-02	1.12E-02	T	BN	NA	MODERATE	NA	NA	36	36	0	36	22	13	36.11
Non-user	IOB_24	chr1	22050606	G	A	USP48	MISSENSE	ENSG00000090686:[ENST00000374732(c.50C>T,cCg>cTg):ENSP00000363864(p.Pro17Leu)]	NA	NA	2.00E-04	3.97E-05	0.00E+00	2.00E-04	0.00E+00	NA	NA	NA	MODIFIER	NA	NA	212	211	1	195	87	106	54.36
Non-user	IOB_24	chr1	22183660	C	T	HSPG2	MISSENSE	ENSG00000142798:[ENST00000374695(c.5423G>A,cGc>cAc):ENSP00000363827(p.Arg1808His)]	NA	NA	5.99E-04	3.14E-04	1.76E-03	5.99E-04	1.00E-03	NA	NA	NA	MODERATE	NA	1	239	236	0	186	83	101	54.3
Non-user	IOB_24	chr1	22915424	A	T	EPHA8	MISSENSE	ENSG00000070886:[ENST00000166244(c.1040A>T,gAg>gTg):ENSP00000166244(p.Glu347Val)]	NA	NA	2.00E-04	5.88E-05	4.42E-04	2.00E-04	1.00E-03	D	PoD	NA	MODERATE	D	1	263	261	0	173	106	67	38.73
Non-user	IOB_24	chr1	23759743	G	T	ASAP3	MISSENSE	ENSG00000088280:[ENST00000437606(c.2123C>A,gCc>gAc):ENSP00000408826(p.Ala708Asp)]	NA	NA	2.60E-03	4.60E-03	1.12E-03	2.60E-03	1.00E-03	NA	NA	NA	MODERATE	NA	0.939	238	238	0	185	112	73	39.46
Non-user	IOB_24	chr1	23885415	G	A	ID3	NCT	ENSG00000117318:[ENST00000463312(n.141+11C>T)]	NA	NA	1.18E-02	8.55E-03	5.82E-02	1.18E-02	6.03E-02	NA	NA	NA	MODIFIER	NA	NA	52	52	0	41	25	16	39.02
Non-user	IOB_24	chr1	24394558	G	C	MYOM3	NCT	ENSG00000142661:[ENST00000448831(n.188-11742C>G)]	NA	NA	1.56E-02	8.52E-03	6.12E-02	1.56E-02	7.98E-02	NA	NA	NA	MODIFIER	NA	NA	110	108	0	65	25	40	61.54
Non-user	IOB_24	chr1	24394558	G	C	RP11-293P20.2	NCT	ENSG00000225315:[ENST00000439239(n.404+3795G>C)]	NA	NA	1.56E-02	8.52E-03	6.12E-02	1.56E-02	7.98E-02	NA	NA	NA	MODIFIER	NA	NA	110	108	0	65	25	40	61.54
Non-user	IOB_24	chr1	24394558	G	C	RP11-293P20.4	NCT	ENSG00000230703:[ENST00000429191(n.69+1226G>C)]	NA	NA	1.56E-02	8.52E-03	6.12E-02	1.56E-02	7.98E-02	NA	NA	NA	MODIFIER	NA	NA	110	108	0	65	25	40	61.54
Non-user	IOB_24	chr1	24432539	T	A	MYOM3	MISSENSE	ENSG00000142661:[ENST00000330966(c.434A>T,gAg>gTg):ENSP00000332670(p.Glu145Val)]	NA	NA	9.98E-03	6.32E-03	3.33E-02	9.98E-03	3.17E-02	D	PoD	NA	MODERATE	D	1	94	94	0	89	41	46	51.69
Non-user	IOB_24	chr1	24432540	C	A	MYOM3	NONSENSE	ENSG00000142661:[ENST00000329601(c.430G>T,Gag>Tag):ENSP00000328415(p.Glu144Ter)]	NA	NA	9.98E-03	6.32E-03	3.33E-02	9.98E-03	3.17E-02	NA	NA	NA	HIGH	NA	1	95	95	0	89	41	46	51.69
Non-user	IOB_24	chr1	26349472	G	A	EXTL1	MISSENSE	ENSG00000158008:[ENST00000374280(c.335G>A,cGc>cAc):ENSP00000363398(p.Arg112His)]	NA	NA	NA	7.42E-05	5.48E-04	NA	NA	NA	NA	NA	MODIFIER	NA	NA	291	290	0	239	114	125	52.3
Non-user	IOB_24	chr1	26349472	G	A	NA	NCT	101928216:[XR_241228.1(n.262+2092C>T)]	NA	NA	NA	7.42E-05	5.48E-04	NA	NA	NA	NA	NA	MODIFIER	NA	NA	291	290	0	239	114	125	52.3
Non-user	IOB_24	chr1	26510940	C	A	CNKSR1	MISSENSE	ENSG00000142675:[ENST00000374253(c.1070C>A,cCa>cAa):ENSP00000363371(p.Pro357Gln)]	NA	NA	3.40E-02	1.57E-02	7.21E-02	3.39E-02	7.98E-02	NA	NA	NA	MODIFIER	NA	NA	166	166	0	110	49	60	54.55
Non-user	IOB_24	chr1	26515261	A	G	CNKSR1	INTRONIC-SS-ACR	ENSG00000142675:[ENST00000361530(c.1691-2A>G)]	NA	NA	4.39E-03	2.89E-03	1.80E-02	4.39E-03	2.25E-02	NA	NA	NA	HIGH	NA	NA	147	147	0	121	75	46	38.02
Non-user	IOB_24	chr1	26608896	C	G	UBXN11	MISSENSE	ENSG00000158062:[ENST00000374221(c.1457G>C,tGt>tCt):ENSP00000363338(p.Cys486Ser)]	NA	chr1*26608896*26608896*COSM425990*C*G*2*breast, large_intestine*1,1	NA	6.53E-03	2.13E-04	NA	NA	NA	NA	NA	MODIFIER	NA	NA	104	100	2	88	35	50	56.82
Non-user	IOB_24	chr1	26672506	G	A	AIM1L	MISSENSE	ENSG00000176092:[ENST00000475866(c.643C>T,Cgc>Tgc):ENSP00000428746(p.Arg215Cys)]	NA	NA	3.14E-02	1.67E-02	3.39E-03	3.13E-02	6.10E-03	D	PrD	NA	MODERATE	NA	NA	366	365	0	249	131	117	46.99
Non-user	IOB_24	chr1	27320730	G	T	TRNP1	SILENT	ENSG00000253368:[ENST00000522111(c.453G>T,ctG>ctT):ENSP00000429216(p.Leu151(=))]	NA	NA	2.66E-02	1.15E-01	1.05E-01	2.66E-02	2.04E-02	NA	NA	NA	LOW	NA	NA	19	19	0	14	9	5	35.71
Non-user	IOB_24	chr1	28586376	C	G	SESN2	SILENT	ENSG00000130766:[ENST00000253063(c.18C>G,tcC>tcG):ENSP00000253063(p.Ser6(=))]	NA	NA	3.42E-02	9.35E-02	1.48E-01	3.41E-02	2.45E-02	NA	NA	NA	LOW	NA	NA	108	107	0	62	40	22	35.48
Non-user	IOB_24	chr1	31349983	C	T	SDC3	MISSENSE	ENSG00000162512:[ENST00000336798(c.112G>A,Gcc>Acc):ENSP00000338346(p.Ala38Thr)]	NA	NA	5.99E-03	9.72E-03	1.03E-02	5.99E-03	1.53E-02	D	PoD	NA	MODERATE	N	0.984	141	140	0	102	57	45	44.12
Non-user	IOB_24	chr1	31791179	T	C	ZCCHC17	INTRONIC	ENSG00000121766:[ENST00000546109(c.42+96T>C)]	NA	NA	NA	NA	NA	NA	NA	NA	NA	NA	MODIFIER	NA	NA	77	74	0	49	27	21	42.86
Non-user	IOB_24	chr1	32050468	C	T	TINAGL1	NCT	ENSG00000142910:[ENST00000468635(n.441-19C>T)]	NA	NA	9.39E-03	7.62E-03	4.20E-02	9.38E-03	4.70E-02	NA	NA	NA	MODIFIER	NA	NA	312	311	0	276	136	139	50.36
Non-user	IOB_24	chr1	32110574	G	A	PEF1	EXONIC-NC	553115:[NR_033686.1(n.265C>T)]	NA	NA	3.49E-02	NA	NA	3.49E-02	6.85E-02	NA	NA	NA	MODIFIER	NA	NA	169	169	0	166	84	82	49.4
Non-user	IOB_24	chr1	32110574	G	A	RP11-73M7.6	NCT	ENSG00000235790:[ENST00000608332(n.87+127G>A)]	NA	NA	3.49E-02	NA	NA	3.49E-02	6.85E-02	NA	NA	NA	MODIFIER	NA	NA	169	169	0	166	84	82	49.4
Non-user	IOB_24	chr1	32110574	G	A	RP11-73M7.9	NCT	ENSG00000264078:[ENST00000581333(n.153+127G>A)]	NA	NA	3.49E-02	NA	NA	3.49E-02	6.85E-02	NA	NA	NA	MODIFIER	NA	NA	169	169	0	166	84	82	49.4
Non-user	IOB_24	chr1	32128140	T	C	COL16A1	INTRONIC	ENSG00000084636:[ENST00000271069(c.3627+48A>G)]	NA	NA	3.45E-02	6.09E-02	6.78E-02	3.45E-02	6.85E-02	NA	NA	NA	MODIFIER	NA	NA	63	58	1	49	31	15	30.61
Non-user	IOB_24	chr1	32145374	G	A	COL16A1	INTRONIC	1307:[XM_005270482.1(c.2541+31C>T)]	NA	NA	5.99E-04	2.36E-03	5.68E-03	5.99E-04	2.00E-03	NA	NA	NA	MODIFIER	NA	NA	71	69	1	56	31	25	44.64
Non-user	IOB_24	chr1	32210341	C	T	BAI2	INTRONIC-SS-ACR-PRX	ENSG00000121753:[ENST00000257070(c.839-9G>A)]	NA	NA	NA	1.50E-04	9.19E-04	NA	NA	NA	NA	NA	MODIFIER	NA	NA	125	125	0	100	51	48	48
Non-user	IOB_24	chr1	32259792	G	A	SPOCD1	SILENT	ENSG00000134668:[ENST00000257100(c.795C>T,atC>atT):ENSP00000257100(p.Ile265(=))]	NA	NA	1.20E-03	9.02E-04	6.26E-03	1.20E-03	6.10E-03	NA	NA	NA	LOW	NA	NA	112	112	0	110	59	50	45.45
Non-user	IOB_24	chr1	33248032	C	T	YARS	MISSENSE	ENSG00000134684:[ENST00000373477(c.1015G>A,Gct>Act):ENSP00000362576(p.Ala339Thr)]	NA	NA	3.59E-03	3.02E-03	1.70E-02	3.59E-03	1.43E-02	T	BN	NA	MODERATE	N	0.153	107	106	1	89	43	46	51.69
Non-user	IOB_24	chr1	33944993	A	G	ZSCAN20	MISSENSE	ENSG00000121903:[ENST00000361328(c.104A>G,aAa>aGa):ENSP00000355053(p.Lys35Arg)]	NA	NA	1.92E-02	3.84E-02	1.70E-02	1.92E-02	1.74E-02	T	BN	NA	MODERATE	N	0	127	126	0	78	39	39	50
Non-user	IOB_24	chr1	33954805	C	T	ZSCAN20	INTRONIC	ENSG00000121903:[ENST00000373413(c.604+554C>T)]	NA	NA	3.87E-02	5.80E-02	5.36E-02	3.87E-02	4.09E-02	NA	NA	NA	MODIFIER	NA	NA	55	55	0	37	19	18	48.65
Non-user	IOB_24	chr1	36636634	C	T	MAP7D1	MISSENSE	ENSG00000116871:[ENST00000316156(c.109C>T,Cca>Tca):ENSP00000320228(p.Pro37Ser)]	NA	NA	3.99E-04	1.10E-04	8.79E-04	3.99E-04	2.00E-03	NA	NA	NA	MODIFIER	NA	NA	168	157	0	130	51	72	55.38
Non-user	IOB_24	chr1	36636854	G	A	MAP7D1	MISSENSE	ENSG00000116871:[ENST00000429533(c.212G>A,cGa>cAa):ENSP00000390091(p.Arg71Gln)]	NA	NA	1.16E-02	2.79E-02	1.85E-02	1.16E-02	1.23E-02	T	BN	NA	MODERATE	N	0.163	132	132	0	72	46	26	36.11
Non-user	IOB_24	chr1	36809496	G	A	STK40	SILENT	ENSG00000196182:[ENST00000373130(c.984C>T,gaC>gaT):ENSP00000362222(p.Asp328(=))]	NA	NA	2.00E-04	7.78E-05	5.51E-04	2.00E-04	1.00E-03	NA	NA	NA	LOW	NA	0.006	217	217	0	164	81	82	50
Non-user	IOB_24	chr1	38039990	G	A	GNL2	MISSENSE	ENSG00000134697:[ENST00000373062(c.1370C>T,cCt>cTt):ENSP00000362153(p.Pro457Leu)]	NA	NA	NA	3.38E-05	2.50E-04	NA	NA	D	PrD	NA	MODERATE	D	1	117	117	0	74	35	38	51.35
Non-user	IOB_24	chr1	39802168	A	C	MACF1	MISSENSE	ENSG00000127603:[ENST00000567887(c.10019A>C,aAg>aCg):ENSP00000455823(p.Lys3340Thr)]	NA	NA	1.20E-02	1.59E-02	2.93E-02	1.20E-02	3.99E-02	NA	NA	NA	MODIFIER	NA	NA	70	70	0	45	21	23	51.11
Non-user	IOB_24	chr1	39988799	G	A	BMP8A	INTRONIC-SS-DNR-PRX	ENSG00000183682:[ENST00000331593(c.1059+10G>A)]	NA	NA	2.56E-02	4.32E-02	5.27E-02	2.56E-02	5.52E-02	NA	NA	NA	MODIFIER	NA	NA	188	186	1	159	90	69	43.4
Non-user	IOB_24	chr1	39988799	G	A	PPIEL	NCT	728448:[NR_144357.1(n.2386+431C>T)]	NA	NA	2.56E-02	4.32E-02	5.27E-02	2.56E-02	5.52E-02	NA	NA	NA	MODIFIER	NA	NA	188	186	1	159	90	69	43.4
Non-user	IOB_24	chr1	39988799	G	A	RP11-69E11.4	NCT	ENSG00000182109:[ENST00000440190(n.456-622C>T)]	NA	NA	2.56E-02	4.32E-02	5.27E-02	2.56E-02	5.52E-02	NA	NA	NA	MODIFIER	NA	NA	188	186	1	159	90	69	43.4
Non-user	IOB_24	chr1	40627110	G	C	RLF	SILENT	ENSG00000117000:[ENST00000372771(c.39G>C,ggG>ggC):ENSP00000361857(p.Gly13(=))]	NA	NA	NA	2.59E-04	8.95E-04	NA	NA	NA	NA	NA	LOW	NA	NA	63	63	0	89	40	49	55.06
Non-user	IOB_24	chr1	41848060	C	T	FOXO6	MISSENSE	ENSG00000204060:[ENST00000372591(c.1187C>T,gCc>gTc):ENSP00000361672(p.Ala396Val)]	NA	NA	NA	6.90E-01	6.82E-01	NA	NA	D_lc	UN	NA	MODERATE	U	0.002	65	64	1	49	11	38	77.55
Non-user	IOB_24	chr1	41976665	G	A	HIVEP3	SILENT	ENSG00000127124:[ENST00000372584(c.6675C>T,tcC>tcT):ENSP00000361665(p.Ser2225(=))]	NA	NA	1.06E-02	4.64E-03	7.92E-03	1.06E-02	1.23E-02	NA	NA	NA	LOW	NA	NA	187	185	1	168	73	93	55.36
Non-user	IOB_24	chr1	41979162	C	T	HIVEP3	SILENT	ENSG00000127124:[ENST00000247584(c.5730G>A,acG>acA):ENSP00000247584(p.Thr1910(=))]	NA	NA	1.32E-02	7.01E-03	1.59E-02	1.32E-02	2.56E-02	NA	NA	NA	MODIFIER	NA	NA	91	91	0	68	38	30	44.12
Non-user	IOB_24	chr1	42049278	G	A	HIVEP3	SILENT	ENSG00000127124:[ENST00000247584(c.1191C>T,tcC>tcT):ENSP00000247584(p.Ser397(=))]	NA	NA	5.59E-03	1.98E-03	1.39E-02	5.59E-03	2.76E-02	NA	NA	NA	LOW	NA	NA	324	321	1	261	131	129	49.43
Non-user	IOB_24	chr1	42896441	C	A	ZMYND12	MISSENSE	ENSG00000066185:[ENST00000433602(c.734G>T,aGt>aTt):ENSP00000398340(p.Ser245Ile)]	NA	NA	8.39E-03	2.30E-02	6.24E-03	8.39E-03	5.10E-03	T	BN	NA	MODERATE	N	0	249	249	0	221	111	109	49.32
Non-user	IOB_24	chr1	43220563	T	C	LEPRE1	MISSENSE	ENSG00000117385:[ENST00000431412(c.144A>G,gAt>gGt):ENSP00000408056(p.Asp49Gly)]	NA	NA	1.20E-03	2.56E-03	1.33E-03	1.20E-03	1.00E-03	NA	NA	NA	MODIFIER	NA	NA	201	201	0	153	76	76	49.67
Non-user	IOB_24	chr1	43228206	C	G	LEPRE1	INTRONIC	ENSG00000117385:[ENST00000372526(c.466-189G>C)]	NA	NA	1.80E-03	NA	NA	1.80E-03	1.00E-03	NA	NA	NA	MODIFIER	NA	NA	54	54	0	42	18	24	57.14
Non-user	IOB_24	chr1	44084432	C	T	PTPRF	INTRONIC	ENSG00000142949:[ENST00000372413(c.4464+12C>T)]	NA	NA	2.80E-03	6.82E-03	2.39E-03	2.80E-03	0.00E+00	NA	NA	NA	MODIFIER	NA	NA	145	143	0	106	56	50	47.17
Non-user	IOB_24	chr1	45524311	C	T	ZSWIM5	MISSENSE	ENSG00000162415:[ENST00000359600(c.1222G>A,Gac>Aac):ENSP00000352614(p.Asp408Asn)]	NA	NA	2.60E-03	2.50E-03	1.05E-02	2.60E-03	1.12E-02	D	PoD	NA	MODERATE	D	1	128	128	0	95	55	39	41.05
Non-user	IOB_24	chr1	46499526	A	G	MAST2	MISSENSE	ENSG00000086015:[ENST00000361297(c.3590A>G,aAa>aGa):ENSP00000354671(p.Lys1197Arg)]	NA	NA	2.44E-02	5.38E-02	1.46E-02	2.44E-02	4.10E-03	T_lc	BN	NA	MODERATE	D	1	141	141	0	122	57	63	51.64
Non-user	IOB_24	chr1	46500251	G	A	MAST2	MISSENSE	ENSG00000086015:[ENST00000361297(c.3910G>A,Gtg>Atg):ENSP00000354671(p.Val1304Met)]	NA	NA	2.44E-02	5.53E-02	1.45E-02	2.44E-02	4.10E-03	NA	NA	NA	MODERATE	NA	1	232	231	0	171	93	76	44.44
Non-user	IOB_24	chr1	46827485	C	T	NSUN4	SILENT	ENSG00000117481:[ENST00000474844(c.1122C>T,ccC>ccT):ENSP00000419740(p.Pro374(=))]	NA	NA	NA	2.19E-01	1.07E-01	NA	NA	NA	NA	NA	LOW	NA	NA	105	105	0	81	28	53	65.43
Non-user	IOB_24	chr1	46977812	T	C	DMBX1	SILENT	ENSG00000197587:[ENST00000360032(c.780T>C,cgT>cgC):ENSP00000353132(p.Arg260(=))]	NA	NA	3.87E-02	3.00E-02	4.65E-02	3.87E-02	5.21E-02	NA	NA	NA	LOW	NA	NA	344	342	1	241	132	108	44.81
Non-user	IOB_24	chr1	47399810	A	G	CYP4A11	NCT	ENSG00000187048:[ENST00000496519(n.71+38T>C)]	NA	NA	2.40E-02	4.19E-02	5.92E-02	2.40E-02	5.52E-02	NA	NA	NA	MODIFIER	NA	NA	126	123	1	99	46	53	53.54
Non-user	IOB_24	chr1	47610293	C	T	CYP4A22	SILENT	ENSG00000162365:[ENST00000294337(c.969C>T,caC>caT):ENSP00000294337(p.His323(=))]	NA	NA	NA	2.34E-01	1.90E-01	NA	NA	NA	NA	NA	MODIFIER	NA	NA	357	355	0	267	230	37	13.86
Non-user	IOB_24	chr1	47610293	C	T	CYP4A22-AS1	NCT	ENSG00000225506:[ENST00000444042(n.396+32414G>A)]	NA	NA	NA	2.34E-01	1.90E-01	NA	NA	NA	NA	NA	MODIFIER	NA	NA	357	355	0	267	230	37	13.86
Non-user	IOB_24	chr1	49113384	C	T	AGBL4	INTRONIC	ENSG00000186094:[ENST00000416121(c.376+5625G>A)]	NA	NA	2.46E-02	NA	NA	2.46E-02	4.70E-02	NA	NA	NA	MODIFIER	NA	NA	88	88	0	82	37	45	54.88
Non-user	IOB_24	chr1	54502268	A	T	TMEM59	INTRONIC	9528:[NM_001305043.1(c.819+15T>A)]	NA	NA	NA	6.74E-05	0.00E+00	NA	NA	NA	NA	NA	MODIFIER	NA	NA	120	120	0	99	59	39	39.39

Patel *et al.*, 2021. Whole-exome sequencing analysis of oral squamous cell carcinoma delineated by tobacco usage habits
Supplementary Table 4. List of somatic single nucleotide variants identified using whole exome sequencing of OSCC patients

Cohort	Sample	Chromosome	Genomic position	Reference allele	Alternate allele	Genesymbol	Variant type	Variant description	OncoMD	COSMIC	dbSNP	ExAC	ExAC_SAS	1000G	1000G_SAS	SIFT	Polyphen2	CONDEL	VEP	LRT	PhastCon7Way	Total reads in Normal	Reference read support in Normal	Alternative read support in Normal	Total reads in Tumor	Reference read support in Tumor	Alternate read support in Tumor	Alternate allele frequency in Tumor
Non-user	IOB_24	chr1	55062960	G	A	ACOT11	MISSENSE	ENSG00000162390:[ENST00000343744(c.636G>A,atG>atA):ENSP00000340260(p.Met212Ile)]	NA	NA	3.04E-02	1.73E-02	5.29E-03	3.04E-02	4.10E-03	NA	NA	NA	MODIFIER	NA	NA	164	162	0	166	92	74	44.58
Non-user	IOB_24	chr1	55470706	C	T	BSND	SILENT	ENSG00000162399:[ENST00000371265(c.189C>T,gtC>gtT):ENSP00000360312(p.Val63(=))]	NA	NA	3.20E-03	2.50E-03	7.40E-03	3.19E-03	1.23E-02	NA	NA	NA	LOW	NA	NA	285	283	0	185	107	74	40
Non-user	IOB_24	chr1	57209794	C	A	C1orf168	NCT	ENSG00000187889:[ENST00000484327(n.1744+31G>T)]	NA	NA	chr1*57209794*57209794*C*T*rs998721287*NA*NA*NA	NA	NA	NA	NA	NA	NA	NA	MODIFIER	NA	NA	128	126	1	130	61	69	53.08
Non-user	IOB_24	chr1	57411525	G	A	C8B	SILENT	ENSG00000021852:[ENST00000371237(c.1074C>T,ctC>ctT):ENSP00000360281(p.Leu358(=))]	NA	NA	1.80E-03	3.43E-03	1.72E-02	1.80E-03	7.20E-03	NA	NA	NA	LOW	NA	NA	50	50	0	54	24	30	55.56
Non-user	IOB_24	chr1	57415310	G	A	C8B	MISSENSE	ENSG00000021852:[ENST00000535057(c.596C>T,cCt>cTt):ENSP00000440113(p.Pro199Leu)]	NA	NA	4.21E-02	4.98E-02	6.22E-02	4.21E-02	5.52E-02	T	BN	NA	MODERATE	N	0.093	122	122	0	74	34	40	54.05
Non-user	IOB_24	chr1	57422511	C	T	C8B	MISSENSE	ENSG00000021852:[ENST00000543257(c.166G>A,Gaa>Aaa):ENSP00000442548(p.Glu56Lys)]	NA	chr1*57422511*57422511*COSM192665*C*T*1*large_intestine*1	4.15E-02	4.98E-02	6.23E-02	4.15E-02	5.52E-02	D	BN	NA	MODERATE	D	0.995	230	230	0	221	120	98	44.34
Non-user	IOB_24	chr1	62676636	A	G	L1TD1	SILENT	ENSG00000240563:[ENST00000498273(c.2190A>G,gcA>gcG):ENSP00000419901(p.Ala730(=))]	NA	NA	NA	NA	NA	NA	NA	NA	NA	NA	LOW	NA	NA	112	112	0	78	45	33	42.31
Non-user	IOB_24	chr1	65303659	C	T	JAK1	SILENT	ENSG00000162434:[ENST00000342505(c.3096G>A,aaG>aaA):ENSP00000343204(p.Lys1032(=))]	NA	chr1*65303658*65303661*COSM1343903*CCTT*C*1*large_intestine*1	3.85E-02	7.57E-02	1.53E-02	3.85E-02	1.02E-02	NA	NA	NA	LOW	NA	NA	175	175	0	150	72	78	52
Non-user	IOB_24	chr1	65335062	A	G	JAK1	SILENT	ENSG00000162434:[ENST00000342505(c.579T>C,gcT>gcC):ENSP00000343204(p.Ala193(=))]	NA	NA	3.91E-02	7.64E-02	1.54E-02	3.91E-02	1.02E-02	NA	NA	NA	LOW	NA	NA	172	172	0	156	76	78	50
Non-user	IOB_24	chr1	67401629	A	G	MIER1	INTRONIC	57708:[NM_001146112.1(c.-72-4082A>G)]	NA	NA	3.85E-02	NA	NA	3.85E-02	6.95E-02	NA	NA	NA	MODIFIER	NA	NA	87	87	0	78	50	26	33.33
Non-user	IOB_24	chr1	67401630	A	C	MIER1	INTRONIC	57708:[NM_001146112.1(c.-72-4081A>C)]	NA	NA	3.85E-02	NA	NA	3.85E-02	6.95E-02	NA	NA	NA	MODIFIER	NA	NA	87	87	0	78	50	26	33.33
Non-user	IOB_24	chr1	67685301	A	G	C1orf141	INTRONIC	ENSG00000203963:[ENST00000371007(c.-104+12228T>C)]	NA	NA	2.80E-03	2.01E-03	1.46E-02	2.80E-03	1.43E-02	NA	NA	NA	LOW	NA	0.919	187	187	0	148	84	64	43.24
Non-user	IOB_24	chr1	67685301	A	G	IL23R	SILENT	ENSG00000162594:[ENST00000395227(c.78A>G,gaA>gaG):ENSP00000378652(p.Glu26(=))]	NA	NA	2.80E-03	2.01E-03	1.46E-02	2.80E-03	1.43E-02	NA	NA	NA	LOW	NA	0.919	187	187	0	148	84	64	43.24
Non-user	IOB_24	chr1	76190439	T	C	ACADM	EXONIC-NC	ENSG00000117054:[ENST00000534146(n.46T>C)]	NA	NA	4.03E-02	2.92E-02	8.60E-03	4.03E-02	5.10E-03	NA	NA	NA	MODIFIER	NA	NA	194	193	0	121	60	60	49.59
Non-user	IOB_24	chr1	76199277	A	C	ACADM	SILENT	ENSG00000117054:[ENST00000370841(c.351A>C,acA>acC):ENSP00000359878(p.Thr117(=))]	NA	NA	4.01E-02	2.79E-02	7.45E-03	4.01E-02	5.10E-03	NA	NA	NA	MODIFIER	NA	NA	320	318	1	242	149	91	37.6
Non-user	IOB_24	chr1	76355088	A	T	MSH4	INTRONIC	ENSG00000057468:[ENST00000263187(c.2226+34A>T)]	NA	NA	5.39E-03	2.34E-03	1.87E-02	5.39E-03	2.76E-02	NA	NA	NA	MODIFIER	NA	NA	48	48	0	38	22	16	42.11
Non-user	IOB_24	chr1	78098403	T	C	ZZZ3	MISSENSE	ENSG00000036549:[ENST00000370801(c.637A>G,Ata>Gta):ENSP00000359837(p.Ile213Val)]	NA	NA	2.54E-02	2.76E-02	4.86E-02	2.54E-02	4.50E-02	NA	NA	NA	MODIFIER	NA	NA	478	477	0	330	176	152	46.06
Non-user	IOB_24	chr1	78511960	C	T	GIPC2	MISSENSE	ENSG00000137960:[ENST00000370759(c.182C>T,tCc>tTc):ENSP00000359795(p.Ser61Phe)]	NA	NA	3.75E-02	1.91E-02	7.91E-02	3.75E-02	9.30E-02	NA	NA	NA	MODIFIER	NA	NA	137	137	0	117	55	61	52.14
Non-user	IOB_24	chr1	79093910	G	A	IFI44L	MISSENSE	ENSG00000137959:[ENST00000450498(c.241G>A,Gca>Aca):ENSP00000400784(p.Ala81Thr)]	NA	chr1*79093910*79093910*COSM146547*G*A*1*stomach*1	3.83E-02	3.05E-02	4.63E-02	3.83E-02	4.91E-02	NA	NA	NA	MODIFIER	NA	NA	87	87	0	63	37	26	41.27
Non-user	IOB_24	chr1	79129355	G	A	IFI44	NCT	10561:[XR_246226.1(n.1273-95G>A)]	NA	NA	2.00E-04	NA	NA	2.00E-04	1.00E-03	NA	NA	NA	MODIFIER	NA	NA	160	159	0	140	79	61	43.57
Non-user	IOB_24	chr1	86315095	A	G	COL24A1	INTRONIC-SS-ACR-PRX	ENSG00000171502:[ENST00000426639(c.*751-6T>C)]	NA	NA	1.44E-02	8.68E-03	5.88E-02	1.44E-02	6.75E-02	NA	NA	NA	LOW	NA	NA	62	62	0	65	25	40	61.54
Non-user	IOB_24	chr1	92109076	A	T	HSP90B3P	EXONIC-NC	ENSG00000203914:[ENST00000415545(n.1004A>T)]	NA	NA	7.39E-03	5.36E-03	2.17E-02	7.39E-03	3.07E-02	NA	NA	NA	MODIFIER	NA	NA	227	227	0	170	77	93	54.71
Non-user	IOB_24	chr1	92647636	T	G	KIAA1107	MISSENSE	ENSG00000069712:[ENST00000409154(c.3247T>G,Ttt>Gtt):ENSP00000386957(p.Phe1083Val)]	NA	NA	3.16E-02	4.76E-02	5.38E-02	3.15E-02	5.42E-02	D	BN	NA	MODERATE	N	0	124	123	0	111	57	54	48.65
Non-user	IOB_24	chr1	94484123	T	C	ABCA4	INTRONIC-SS-DNR-PRX	ENSG00000198691:[ENST00000536513(c.6+7A>G)]	NA	NA	4.19E-02	7.26E-02	6.73E-02	4.19E-02	6.03E-02	NA	NA	NA	MODIFIER	NA	NA	99	99	0	81	46	35	43.21
Non-user	IOB_24	chr1	98165799	G	A	DPYD	SILENT	ENSG00000188641:[ENST00000423006(c.432C>T,aaC>aaT):ENSP00000398884(p.Asn144(=))]	NA	NA	NA	NA	NA	NA	NA	NA	NA	NA	LOW	NA	NA	86	85	1	59	28	30	50.85
Non-user	IOB_24	chr1	108771721	T	C	NBPF4	MISSENSE	ENSG00000196427:[ENST00000415641(c.1481A>G,cAg>cGg):ENSP00000389237(p.Gln494Arg)]	NA	NA	NA	1.57E-01	1.13E-01	NA	NA	NA	NA	NA	MODERATE	NA	NA	101	99	0	111	76	34	30.63
Non-user	IOB_24	chr1	110151344	C	T	GNAT2	MISSENSE	ENSG00000134183:[ENST00000351050(c.370G>A,Gtg>Atg):ENSP00000251337(p.Val124Met)]	NA	NA	2.60E-03	5.44E-03	6.12E-03	2.60E-03	4.10E-03	T	PoD	NA	MODERATE	N	0.897	226	226	0	178	88	90	50.56
Non-user	IOB_24	chr1	110882103	A	G	RBM15	MISSENSE	ENSG00000162775:[ENST00000369784(c.76A>G,Agc>Ggc):ENSP00000358799(p.Ser26Gly)]	NA	NA	1.08E-02	1.47E-02	2.98E-02	1.08E-02	2.04E-02	T_lc	PoD	NA	MODERATE	D	1	142	142	0	134	67	65	48.51
Non-user	IOB_24	chr1	110925550	T	G	LAMTOR5-AS1	EXONIC-NC	ENSG00000224699:[ENST00000590413(n.110T>G)]	NA	NA	2.36E-02	5.24E-02	1.66E-02	2.36E-02	7.20E-03	NA	NA	NA	MODIFIER	NA	NA	315	313	0	241	129	112	46.47
Non-user	IOB_24	chr1	110925550	T	G	SLC16A4	SILENT	ENSG00000168679:[ENST00000369779(c.126A>C,gcA>gcC):ENSP00000358794(p.Ala42(=))]	NA	NA	2.36E-02	5.24E-02	1.66E-02	2.36E-02	7.20E-03	NA	NA	NA	MODIFIER	NA	NA	315	313	0	241	129	112	46.47
Non-user	IOB_24	chr1	111731318	A	T	DENND2D	INTRONIC	79961:[XM_005271225.1(c.559+6T>A)]	NA	NA	1.80E-02	4.14E-02	9.12E-03	1.80E-02	9.20E-03	NA	NA	NA	LOW	NA	NA	94	94	0	86	44	42	48.84
Non-user	IOB_24	chr1	111992516	C	T	ATP5F1	INTRONIC	ENSG00000116459:[ENST00000369722(c.77+21C>T)]	NA	NA	2.28E-02	2.24E-02	3.63E-03	2.28E-02	2.00E-03	NA	NA	NA	MODIFIER	NA	NA	119	119	0	102	48	53	51.96
Non-user	IOB_24	chr1	112028515	T	C	ADORA3	INTRONIC	ENSG00000121933:[ENST00000442484(c.311-53A>G)]	NA	NA	2.80E-02	NA	NA	2.80E-02	2.15E-02	NA	NA	NA	MODIFIER	NA	NA	130	130	0	138	50	88	63.77
Non-user	IOB_24	chr1	112028515	T	C	TMIGD3	INTRONIC	57413:[NM_001302680.1(c.366-53A>G)]	NA	NA	2.80E-02	NA	NA	2.80E-02	2.15E-02	NA	NA	NA	MODIFIER	NA	NA	130	130	0	138	50	88	63.77
Non-user	IOB_24	chr1	117142583	T	A	IGSF3	MISSENSE	ENSG00000143061:[ENST00000318837(c.2069A>T,gAg>gTg):ENSP00000321184(p.Glu690Val)]	NA	chr1*117142583*117142583*COSM311948*T*A*1*lung*1|chr1*117142583*117142583*COSM311949*T*A*1*lung*1	NA	6.70E-05	6.35E-05	NA	NA	T	PrD	NA	MODERATE	D	1	104	100	4	58	41	17	29.31
Non-user	IOB_24	chr1	117146545	C	A	IGSF3	MISSENSE	ENSG00000143061:[ENST00000318837(c.1385G>T,gGt>gTt):ENSP00000321184(p.Gly462Val)]	NA	NA	NA	NA	NA	NA	NA	D	BN	NA	MODERATE	D	0.972	158	158	0	154	133	21	13.64
Non-user	IOB_24	chr1	117659276	G	A	TRIM45	SILENT	ENSG00000134253:[ENST00000369464(c.1260C>T,aaC>aaT):ENSP00000358476(p.Asn420(=))]	NA	NA	NA	9.89E-05	6.06E-04	NA	NA	NA	NA	NA	LOW	NA	0.431	72	72	0	55	26	29	52.73
Non-user	IOB_24	chr1	117659292	C	T	TRIM45	MISSENSE	ENSG00000134253:[ENST00000256649(c.1298G>A,gGc>gAc):ENSP00000256649(p.Gly433Asp)]	NA	NA	4.69E-02	3.78E-02	8.79E-02	4.69E-02	9.30E-02	T	BN	NA	MODERATE	N	1	80	80	0	57	28	29	50.88
Non-user	IOB_24	chr1	120269549	G	A	PHGDH	NCT	ENSG00000092621:[ENST00000493622(n.600+21G>A)]	NA	NA	8.59E-03	1.94E-02	7.03E-03	8.59E-03	2.00E-03	NA	NA	NA	MODIFIER	NA	NA	180	178	2	140	1	138	98.57
Non-user	IOB_24	chr1	121115981	C	T	RP11-343N15.1	NCT	ENSG00000230806:[ENST00000437515(n.330-11232G>A)]	NA	NA	NA	9.23E-02	9.25E-02	NA	NA	NA	NA	NA	MODIFIER	NA	NA	76	76	0	9	4	5	55.56
Non-user	IOB_24	chr1	121115981	C	T	SRGAP2C	MISSENSE	653464:[NM_001329984.1(c.213C>T,Cgt>Tgt):NP_001316913.1(p.Arg446Cys)]	NA	NA	NA	9.23E-02	9.25E-02	NA	NA	NA	NA	NA	MODIFIER	NA	NA	76	76	0	9	4	5	55.56
Non-user	IOB_24	chr1	144875979	G	C	PDE4DIP	SILENT-SS-PRX	ENSG00000178104:[ENST00000479369(n.45C>G)]	NA	NA	NA	1.95E-01	1.92E-01	NA	NA	NA	NA	NA	LOW	NA	NA	133	131	0	101	79	22	21.78
Non-user	IOB_24	chr1	144875979	G	C	RP4-791M13.5	NCT	ENSG00000254913:[ENST00000531288(n.43+992G>C)]	NA	NA	NA	1.95E-01	1.92E-01	NA	NA	NA	NA	NA	LOW	NA	NA	133	131	0	101	79	22	21.78
Non-user	IOB_24	chr1	144916676	C	T	PDE4DIP	NONSENSE	ENSG00000178104:[ENST00000369359(c.2090G>A,tGg>tAg):ENSP00000358366(p.Trp697Ter)]	NA	NA	NA	2.95E-01	2.17E-01	NA	NA	NA	NA	NA	MODIFIER	NA	NA	281	281	0	220	174	46	20.91
Non-user	IOB_24	chr1	144921924	G	A	PDE4DIP	SILENT	ENSG00000178104:[ENST00000313431(c.1594C>T,Ctg>Ttg):ENSP00000316434(p.Leu532(=))]	NA	NA	NA	1.95E-01	1.93E-01	NA	NA	NA	NA	NA	MODIFIER	NA	NA	383	380	0	329	235	92	27.96
Non-user	IOB_24	chr1	144922523	C	T	PDE4DIP	MISSENSE	ENSG00000178104:[ENST00000313382(c.1082G>A,cGt>cAt):ENSP00000327209(p.Arg361His)]	NA	NA	NA	2.91E-01	2.18E-01	NA	NA	D	BN	NA	MODERATE	NA	NA	592	588	0	438	353	84	19.18
Non-user	IOB_24	chr1	144930816	A	G	PDE4DIP	MISSENSE	ENSG00000178104:[ENST00000313431(c.893T>C,cTt>cCt):ENSP00000316434(p.Leu298Pro)]	NA	NA	3.99E-04	6.42E-04	8.48E-04	3.99E-04	0.00E+00	NA	NA	NA	MODIFIER	NA	NA	339	336	1	287	216	70	24.39
Non-user	IOB_24	chr1	144994715	C	T	PDE4DIP	MISSENSE	ENSG00000178104:[ENST00000369347(c.17G>A,cGc>cAc):ENSP00000358353(p.Arg6His)]	NA	NA	chr1*144994715*144994715*C*G,T*rs781917759*NA*NA*NA	4.94E-05	1.21E-04	NA	NA	NA	NA	NA	MODIFIER	NA	NA	722	721	1	569	510	56	9.84
Non-user	IOB_24	chr1	145014210	G	A	PDE4DIP	MISSENSE	ENSG00000178104:[ENST00000493130(c.332C>T,cCt>cTt):ENSP00000432251(p.Pro111Leu)]	NA	NA	NA	4.03E-01	3.85E-01	NA	NA	NA	NA	NA	MODIFIER	NA	NA	417	413	1	338	291	43	12.72
Non-user	IOB_24	chr1	145014218	G	A	PDE4DIP	SILENT	ENSG00000178104:[ENST00000493130(c.324C>T,ccC>ccT):ENSP00000432251(p.Pro108(=))]	NA	NA	NA	2.89E-01	2.70E-01	NA	NA	NA	NA	NA	MODIFIER	NA	NA	476	472	1	381	329	42	11.02
Non-user	IOB_24	chr1	145015877	G	T	PDE4DIP	MISSENSE	ENSG00000178104:[ENST00000531369(c.214C>A,Ctt>Att):ENSP00000435616(p.Leu72Ile)]	NA	chr1*145015877*145015877*COSM1579193*G*T*1*breast*1|chr1*145015877*145015877*COSM1579194*G*T*1*breast*1	NA	2.68E-01	2.56E-01	NA	NA	NA	NA	NA	MODIFIER	NA	NA	562	560	0	434	364	69	15.9
Non-user	IOB_24	chr1	145039594	T	C	PDE4DIP	MISSENSE	ENSG00000178104:[ENST00000313382(c.16A>G,Acg>Gcg):ENSP00000327209(p.Thr6Ala)]	NA	chr1*145039594*145039594*COSM1333549*T*C*1*large_intestine*1	NA	3.43E-02	1.53E-02	NA	NA	NA	NA	NA	MODIFIER	NA	NA	395	394	0	266	237	25	9.4
Non-user	IOB_24	chr1	145115815	A	C	NA	EXONIC-NC	100996517:[XR_159107.2(n.733A>C)]	NA	NA	NA	7.59E-02	5.27E-02	NA	NA	NA	NA	NA	MODIFIER	NA	NA	1018	1015	0	893	793	98	10.97
Non-user	IOB_24	chr1	145115815	A	C	SEC22B	EXONIC-NC	ENSG00000223380:[ENST00000453618(n.901A>C)]	NA	NA	NA	7.59E-02	5.27E-02	NA	NA	NA	NA	NA	MODIFIER	NA	NA	1018	1015	0	893	793	98	10.97
Non-user	IOB_24	chr1	145281656	A	T	NOTCH2NL	MISSENSE	ENSG00000213240:[ENST00000344859(c.586A>T,Acc>Tcc):ENSP00000344557(p.Thr196Ser)]	NA	chr1*145281656*145281656*COSM1179144*A*T*2*central_nervous_system,prostate*1,1|chr1*145281656*145281656*COSM1179143*A*T*2*central_nervous_system,prostate*1,1	NA	3.05E-01	2.74E-01	NA	NA	D	PrD	NA	MODERATE	NA	NA	502	499	0	348	301	46	13.22
Non-user	IOB_24	chr1	145281656	A	T	RP11-458D21.5	MISSENSE	ENSG00000255168:[ENST00000468030(c.586A>T,Acc>Tcc):ENSP00000457682(p.Thr196Ser)]	NA	chr1*145281656*145281656*COSM1179144*A*T*2*central_nervous_system,prostate*1,1|chr1*145281656*145281656*COSM1179143*A*T*2*central_nervous_system,prostate*1,1	NA	3.05E-01	2.74E-01	NA	NA	D	PrD	NA	MODERATE	NA	NA	502	499	0	348	301	46	13.22
Non-user	IOB_24	chr1	145439574	C	A	TXNIP	SILENT	ENSG00000117289:[ENST00000425134(c.67C>A,Cga>Aga):ENSP00000396322(p.Arg23(=))]	NA	NA	NA	NA	NA	NA	NA	NA	NA	NA	MODIFIER	NA	NA	366	364	0	291	284	6	2.06
Non-user	IOB_24	chr1	145594220	C	T	POLR3C	INTRONIC	10623:[XM_005272928.1(c.1023-32G>A)]	NA	NA	9.98E-03	2.51E-02	1.00E-02	9.98E-03	1.00E-03	NA	NA	NA	MODIFIER	NA	NA	139	139	0	145	77	68	46.9
Non-user	IOB_24	chr1	145601728	C	A	POLR3C	INTRONIC	10623:[XM_005272928.1(c.432+20G>T)]	NA	NA	NA	NA	NA	NA	NA	NA	NA	NA	MODIFIER	NA	NA	191	191	0	155	149	5	3.23
Non-user	IOB_24	chr1	146759428	G	A	CHD1L	INTRONIC	9557:[NM_001256336.1(c.2020+16G>A)]	NA	NA	4.79E-03	9.09E-03	6.45E-03	4.79E-03	5.10E-03	NA	NA	NA	MODIFIER	NA	NA	109	109	0	67	32	35	52.24
Non-user	IOB_24	chr1	147091005	C	T	BCL9	SILENT	ENSG00000116128:[ENST00000234739(c.1044C>T,ccC>ccT):ENSP00000234739(p.Pro348(=))]	NA	NA	7.99E-04	1.63E-03	6.83E-03	7.99E-04	3.10E-03	NA	NA	NA	LOW	NA	NA	113	113	0	90	57	33	36.67
Non-user	IOB_24	chr1	147091689	G	A	BCL9	SILENT	ENSG00000116128:[ENST00000234739(c.1728G>A,ccG>ccA):ENSP00000234739(p.Pro576(=))]	NA	NA	1.50E-02	2.85E-02	1.69E-02	1.50E-02	1.23E-02	NA	NA	NA	LOW	NA	NA	229	229	0	162	87	75	46.3
Non-user	IOB_24	chr1	147122105	A	T	ACP6	NCT	51205:[NR_136636.1(n.1652-64T>A)]	NA	NA	NA	NA	NA	NA	NA	NA	NA	NA	MODIFIER	NA	NA	52	50	0	55	24	30	54.55
Non-user	IOB_24	chr1	149379659	T	A	HIST2H2BB	NCT	ENSG00000240929:[ENST00000609585(n.425-9799A>T)]	NA	NA	NA	NA	NA	NA	NA	NA	NA	NA	MODERATE	NA	NA	14	14	0	7	0	7	100
Non-user	IOB_24	chr1	149379659	T	A	LOC100996713	MISSENSE	100996713:[XM_005245707.1(c.326A>T,cAg>cTg):XP_005245764.1(p.Gln109Leu)]	NA	NA	NA	NA	NA	NA	NA	NA	NA	NA	MODERATE	NA	NA	14	14	0	7	0	7	100
Non-user	IOB_24	chr1	150195616	T	C	ANP32E	MISSENSE	ENSG00000143401:[ENST00000534437(c.128A>G,gAa>gGa):ENSP00000431434(p.Glu43Gly)]	NA	NA	NA	2.67E-04	7.70E-04	NA	NA	NA	NA	NA	MODIFIER	NA	NA	54	54	0	48	22	26	54.17
Non-user	IOB_24	chr1	150526270	C	T	ADAMTSL4	MISSENSE	ENSG00000143382:[ENST00000271643(c.803C>T,tCc>tTc):ENSP00000271643(p.Ser268Phe)]	NA	NA	3.40E-03	8.50E-03	3.94E-03	3.39E-03	1.00E-03	NA	NA	NA	MODIFIER	NA	NA	164	163	0	125	58	66	52.8

Patel *et al.* , 2021. Whole-exome sequencing analysis of oral squamous cell carcinoma delineated by tobacco usage habits
Supplementary Table 4. List of somatic single nucleotide variants identified using whole exome sequencing of OSCC patients

Cohort	Sample	Chromosome	Genomic position	Reference allele	Alternate allele	Genesymbol	Variant type	Variant description	OncoMD	COSMIC	dbSNP	ExAC	ExAC_SAS	1000G	1000G_SAS	SIFT	Polyphen2	CONDEL	VEP	LRT	PhastCon7Way	Total reads in Normal	Reference read support in Normal	Alternative read support in Normal	Total reads in Tumor	Reference read support in Tumor	Alternate read support in Tumor	Alternate allele frequency in Tumor
Non-user	IOB_24	chr1	150526270	C	T	NA	NCT	100289061:[XR_241118.1(n.485+741G>A)]	NA	NA	3.40E-03	8.50E-03	3.94E-03	3.39E-03	1.00E-03	NA	NA	NA	MODIFIER	NA	NA	164	163	0	125	58	66	52.8
Non-user	IOB_24	chr1	150526270	C	T	RP11-54A4.2	NCT	ENSG00000237781:[ENST00000442435(n.476+741G>A)]	NA	NA	3.40E-03	8.50E-03	3.94E-03	3.39E-03	1.00E-03	NA	NA	NA	MODIFIER	NA	NA	164	163	0	125	58	66	52.8
Non-user	IOB_24	chr1	150551295	T	C	MCL1	INTRONIC	4170:[NM_001197320.1(c.229+24A>G)]	NA	NA	2.34E-02	3.92E-02	7.33E-02	2.34E-02	3.99E-02	NA	NA	NA	MODIFIER	NA	NA	131	130	0	76	47	28	36.84
Non-user	IOB_24	chr1	150601569	G	A	ENSA	MISSENSE	ENSG00000143420:[ENST00000361532(c.13C>T,Ctt>Ttt):ENSP00000354835(p.Leu5Phe)]	NA	NA	NA	4.94E-05	3.63E-04	NA	NA	NA	NA	NA	MODIFIER	NA	NA	172	172	0	167	87	80	47.9
Non-user	IOB_24	chr1	150620852	T	C	GOLPH3L	MISSENSE	ENSG00000143457:[ENST00000540514(c.671A>G,aAg>aGg):ENSP00000446070(p.Lys224Arg)]	NA	NA	1.40E-03	2.66E-03	1.30E-03	1.40E-03	0.00E+00	T	PoD	NA	MODERATE	D	1	95	95	0	91	47	44	48.35
Non-user	IOB_24	chr1	150666614	C	T	GOLPH3L	SILENT	ENSG00000143457:[ENST00000427665(c.219G>A,acG>acA):ENSP00000410476(p.Thr73(=))]	NA	NA	3.79E-03	9.19E-03	4.23E-03	3.79E-03	1.00E-03	NA	NA	NA	LOW	NA	NA	21	21	0	9	3	6	66.67
Non-user	IOB_24	chr1	151537016	C	T	TUFT1	NONSENSE	ENSG00000143367:[ENST00000353024(c.163C>T,Cag>Tag):ENSP00000343781(p.Gln55Ter)]	NA	NA	NA	8.41E-06	6.22E-05	NA	NA	NA	NA	NA	MODIFIER	NA	NA	80	80	0	98	56	42	42.86
Non-user	IOB_24	chr1	152129115	T	C	RPTN	MISSENSE	ENSG00000215853:[ENST00000316073(c.460A>G,Aga>Gga):ENSP00000317895(p.Arg154Gly)]	NA	chr1*152129115*152129115*COSM1498488*T*C*2*lung*2	NA	2.49E-05	0.00E+00	NA	NA	D	BN	NA	MODERATE	U	0	560	552	0	342	278	54	15.79
Non-user	IOB_24	chr1	152770533	G	A	LCE1D	MISSENSE	ENSG00000172155:[ENST00000326233(c.263G>A,aGc>aAc):ENSP00000316737(p.Ser88Asn)]	NA	NA	NA	1.67E-01	2.34E-01	NA	NA	NA	BN	NA	MODERATE	N	0.168	12	12	0	30	1	29	96.67
Non-user	IOB_24	chr1	152770589	A	G	LCE1D	MISSENSE	ENSG00000172155:[ENST00000326233(c.319A>G,Agc>Ggc):ENSP00000316737(p.Ser107Gly)]	NA	NA	.	2.82E-01	3.90E-01	NA	NA	NA	BN	NA	MODERATE	N	0	5	5	0	25	0	25	100
Non-user	IOB_24	chr1	153276449	T	C	PGLYRP3	MISSENSE	ENSG00000159527:[ENST00000290722(c.413A>G,gAg>gGg):ENSP00000290722(p.Glu138Gly)]	NA	chr1*153276449*153276449*COSM146630*T*C*1*stomach*1	4.05E-02	2.06E-02	6.54E-03	4.05E-02	1.33E-02	D	BN	NA	MODERATE	NA	NA	63	63	0	46	24	21	45.65
Non-user	IOB_24	chr1	153279598	C	T	PGLYRP3	SILENT	ENSG00000159527:[ENST00000290722(c.201G>A,ctG>ctA):ENSP00000290722(p.Leu67(=))]	NA	NA	2.88E-02	1.87E-02	7.09E-03	2.88E-02	1.23E-02	NA	NA	NA	LOW	NA	NA	140	140	0	145	71	73	50.34
Non-user	IOB_24	chr1	153312850	C	T	PGLYRP4	INTRONIC-SS-DNR-PRX	ENSG00000163218:[ENST00000359650(c.824+7G>A)]	NA	chr1*153312850*153312850*COSM146631*C*T*1*stomach*1	2.26E-02	1.22E-02	6.19E-03	2.26E-02	1.23E-02	NA	NA	NA	LOW	NA	NA	106	106	0	74	44	30	40.54
Non-user	IOB_24	chr1	153320343	A	G	PGLYRP4	NCT	ENSG00000163218:[ENST00000490266(n.95+17T>C)]	NA	NA	2.40E-02	1.26E-02	5.97E-03	2.40E-02	1.23E-02	NA	NA	NA	MODIFIER	NA	NA	58	58	0	46	34	12	26.09
Non-user	IOB_24	chr1	154995920	G	A	DCST2	SILENT	ENSG00000163354:[ENST00000368424(c.1884C>T,ctC>ctT):ENSP00000357409(p.Leu628(=))]	NA	NA	9.98E-03	1.68E-02	2.93E-02	9.98E-03	2.35E-02	NA	NA	NA	LOW	NA	NA	102	101	0	75	33	42	56
Non-user	IOB_24	chr1	155028450	C	A	ADAM15	MISSENSE	ENSG00000143537:[ENST00000447332(c.670C>A,Ctg>Atg):ENSP00000476000(p.Leu224Met)]	NA	NA	1.10E-02	4.94E-03	1.39E-03	1.10E-02	2.00E-03	NA	NA	NA	MODIFIER	NA	NA	214	212	0	150	67	82	54.67
Non-user	IOB_24	chr1	155028450	C	A	DCST1-AS1	NCT	100505666:[NR_040773.1(n.236-6613G>T)]	NA	NA	1.10E-02	4.94E-03	1.39E-03	1.10E-02	2.00E-03	NA	NA	NA	MODIFIER	NA	NA	214	212	0	150	67	82	54.67
Non-user	IOB_24	chr1	155160942	G	A	MUC1	SILENT	ENSG00000185499:[ENST00000368395(c.585C>T,aaC>aaT):ENSP00000357380(p.Asn195(=))]	NA	NA	3.99E-04	1.27E-03	3.07E-03	3.99E-04	2.00E-03	NA	NA	NA	MODIFIER	NA	NA	142	142	0	107	46	61	57.01
Non-user	IOB_24	chr1	155178993	G	C	MTX1	MISSENSE	ENSG00000173171:[ENST00000316721(c.398G>C,gGg>gCg):ENSP00000317106(p.Gly133Ala)]	NA	NA	1.40E-03	1.71E-03	1.53E-03	1.40E-03	6.10E-03	T_lc	BN	NA	MODERATE	N	0	49	49	0	48	26	22	45.83
Non-user	IOB_24	chr1	155217643	C	T	FAM189B	MISSENSE	ENSG00000160767:[ENST00000361361(c.1937G>A,cGc>cAc):ENSP00000354958(p.Arg646His)]	NA	NA	9.59E-03	1.68E-02	2.73E-03	9.58E-03	2.00E-03	D_lc	PrD	NA	MODERATE	D	0.731	84	84	0	63	38	25	39.68
Non-user	IOB_24	chr1	155240656	C	G	CLK2	MISSENSE	ENSG00000176444:[ENST00000355560(c.113G>C,aGt>aCt):ENSP00000347759(p.Ser38Thr)]	NA	NA	1.40E-03	4.78E-04	9.09E-04	1.40E-03	6.10E-03	T	PoD	NA	MODERATE	N	1	121	121	0	106	60	46	43.4
Non-user	IOB_24	chr1	155657845	C	A	DAP3	EXONIC-NC	ENSG00000132676:[ENST00000461479(n.95C>A)]	NA	NA	2.20E-03	1.20E-03	0.00E+00	2.20E-03	1.00E-03	NA	NA	NA	MODIFIER	NA	NA	70	70	0	39	25	14	35.9
Non-user	IOB_24	chr1	155657845	C	A	MSTO1	INTRONIC	ENSG00000125459:[ENST00000538143(c.291-58742C>A)]	NA	NA	2.20E-03	1.20E-03	0.00E+00	2.20E-03	1.00E-03	NA	NA	NA	MODIFIER	NA	NA	70	70	0	39	25	14	35.9
Non-user	IOB_24	chr1	155657845	C	A	MSTO2P	NCT	ENSG00000203761:[ENST00000538914(n.267-59447C>A)]	NA	NA	2.20E-03	1.20E-03	0.00E+00	2.20E-03	1.00E-03	NA	NA	NA	MODIFIER	NA	NA	70	70	0	39	25	14	35.9
Non-user	IOB_24	chr1	155657845	C	A	YY1AP1	INTRONIC	55249:[XM_005245309.1(c.-725+17G>T)]	NA	NA	2.20E-03	1.20E-03	0.00E+00	2.20E-03	1.00E-03	NA	NA	NA	MODIFIER	NA	NA	70	70	0	39	25	14	35.9
Non-user	IOB_24	chr1	155896514	C	T	KIAA0907	MISSENSE	ENSG00000132680:[ENST00000368319(c.634G>A,Gct>Act):ENSP00000357302(p.Ala212Thr)]	NA	NA	2.00E-03	8.07E-04	0.00E+00	2.00E-03	1.00E-03	NA	NA	NA	MODIFIER	NA	NA	114	113	0	93	57	35	37.63
Non-user	IOB_24	chr1	156021079	G	A	UBQLN4	SILENT	ENSG00000160803:[ENST00000368309(c.300C>T,tcC>tcT):ENSP00000357292(p.Ser100(=))]	NA	NA	1.36E-02	3.59E-02	1.36E-02	1.36E-02	0.00E+00	NA	NA	NA	MODIFIER	NA	NA	110	110	0	63	37	26	41.27
Non-user	IOB_24	chr1	156640581	C	G	NES	MISSENSE	ENSG00000132688:[ENST00000368223(c.3399G>C,agG>agC):ENSP00000357206(p.Arg1133Ser)]	NA	NA	3.04E-02	1.65E-02	4.92E-02	3.04E-02	6.75E-02	T	BN	NA	MODERATE	N	0	168	166	0	116	65	50	43.1
Non-user	IOB_24	chr1	158637704	C	T	SPTA1	MISSENSE	ENSG00000163554:[ENST00000368147(c.1982G>A,cGt>cAt):ENSP00000357129(p.Arg661His)]	NA	NA	1.40E-03	1.63E-03	4.78E-03	1.40E-03	6.10E-03	D	BN	NA	MODERATE	N	1	250	249	0	203	92	108	53.2
Non-user	IOB_24	chr1	159272741	C	A	FCER1A	INTRONIC	ENSG00000179639:[ENST00000368114(c.76+77C>A)]	NA	NA	2.40E-03	NA	NA	2.40E-03	1.00E-03	NA	NA	NA	MODIFIER	NA	NA	181	179	0	124	80	44	35.48
Non-user	IOB_24	chr1	159804940	G	A	C1orf204	3UTR	ENSG00000188004:[ENST00000368102(c.*1007C>T)]	NA	NA	1.32E-02	3.10E-02	3.15E-02	1.32E-02	1.94E-02	NA	NA	NA	MODIFIER	NA	NA	47	47	0	51	26	25	49.02
Non-user	IOB_24	chr1	159804940	G	A	SLAMF8	INTRONIC	ENSG00000158714:[ENST00000368104(c.455-34G>A)]	NA	NA	1.32E-02	3.10E-02	3.15E-02	1.32E-02	1.94E-02	NA	NA	NA	MODIFIER	NA	NA	47	47	0	51	26	25	49.02
Non-user	IOB_24	chr1	160265668	G	C	COPA	INTRONIC	ENSG00000122218:[ENST00000241704(c.2353-19C>G)]	NA	NA	1.86E-02	3.68E-02	3.37E-02	1.86E-02	3.07E-02	NA	NA	NA	MODIFIER	NA	NA	158	158	0	158	78	79	50
Non-user	IOB_24	chr1	161049509	G	T	PVRL4	MISSENSE	ENSG00000143217:[ENST00000368012(c.310C>A,Cca>Aca):ENSP00000356991(p.Pro104Thr)]	NA	NA	3.85E-02	7.81E-02	3.10E-02	3.85E-02	2.04E-02	T	PrD	NA	MODERATE	D	0.999	171	171	0	141	78	63	44.68
Non-user	IOB_24	chr1	161132777	G	A	USP21	MISSENSE	ENSG00000143258:[ENST00000289865(c.962G>A,gGc>gAc):ENSP00000289865(p.Gly321Asp)]	NA	NA	2.14E-02	4.42E-02	2.40E-02	2.14E-02	1.53E-02	D	PrD	NA	MODERATE	U	1	86	85	0	79	55	24	30.38
Non-user	IOB_24	chr1	161168147	T	C	ADAMTS4	MISSENSE	ENSG00000158859:[ENST00000367995(c.271A>G,Acg>Gcg):ENSP00000356974(p.Thr91Ala)]	NA	NA	2.80E-03	4.33E-03	3.07E-03	2.80E-03	0.00E+00	NA	NA	NA	MODIFIER	NA	NA	243	241	2	204	86	116	56.86
Non-user	IOB_24	chr1	161168147	T	C	NDUFS2	NCT	ENSG00000158864:[ENST00000479948(n.53+1201T>C)]	NA	NA	2.80E-03	4.33E-03	3.07E-03	2.80E-03	0.00E+00	NA	NA	NA	MODIFIER	NA	NA	243	241	2	204	86	116	56.86
Non-user	IOB_24	chr1	161518214	T	C	FCGR3A	MISSENSE	ENSG00000203747:[ENST00000442336(c.313A>G,Atc>Gtc):ENSP00000396567(p.Ile105Val)]	NA	NA	NA	2.99E-01	3.20E-01	NA	NA	T	BN	NA	MODERATE	N	0	128	128	0	192	142	49	25.52
Non-user	IOB_24	chr1	161518214	T	C	RP11-25K21.6	NCT	ENSG00000273112:[ENST00000537821(n.271+30287T>C)]	NA	NA	NA	2.99E-01	3.20E-01	NA	NA	T	BN	NA	MODERATE	N	0	128	128	0	192	142	49	25.52
Non-user	IOB_24	chr1	161518336	C	T	FCGR3A	MISSENSE	ENSG00000203747:[ENST00000367967(c.194G>A,aGc>aAc):ENSP00000356944(p.Ser65Asn)]	NA	NA	NA	1.02E-01	1.26E-01	NA	NA	T	BN	NA	MODERATE	N	0	249	249	0	343	254	89	25.95
Non-user	IOB_24	chr1	161518336	C	T	RP11-25K21.6	NCT	ENSG00000273112:[ENST00000537821(n.271+30409C>T)]	NA	NA	NA	1.02E-01	1.26E-01	NA	NA	T	BN	NA	MODERATE	N	0	249	249	0	343	254	89	25.95
Non-user	IOB_24	chr1	161692459	C	G	FCRLB	5UTR	ENSG00000162746:[ENST00000336830(c.-4C>G)]	NA	NA	2.00E-04	2.56E-04	1.83E-03	2.00E-04	1.00E-03	NA	NA	NA	MODIFIER	NA	NA	168	167	0	158	92	65	41.14
Non-user	IOB_24	chr1	162742117	C	T	DDR2	INTRONIC	4921:[XM_005245223.1(c.1656+80C>T)]	NA	NA	3.57E-02	NA	NA	3.57E-02	5.21E-02	NA	NA	NA	MODIFIER	NA	NA	74	74	0	50	26	24	48
Non-user	IOB_24	chr1	162754212	G	A	DDR2	3UTR	ENSG00000162733:[ENST00000367922(c.*4176G>A)]	NA	NA	3.65E-02	NA	NA	3.65E-02	5.21E-02	NA	NA	NA	MODIFIER	NA	NA	42	42	0	35	21	14	40
Non-user	IOB_24	chr1	162787358	C	T	NA	INTERGENIC	NA	NA	NA	3.28E-02	NA	NA	3.27E-02	1.84E-02	NA	NA	NA	MODIFIER	NA	NA	263	262	0	212	116	95	44.81
Non-user	IOB_24	chr1	167086657	G	A	DUSP27	MISSENSE	ENSG00000198842:[ENST00000271385(c.298G>A,Gtc>Atc):ENSP00000271385(p.Val100Ile)]	NA	NA	1.60E-03	1.35E-03	9.84E-03	1.60E-03	8.20E-03	NA	NA	NA	MODERATE	NA	1	110	110	0	100	58	41	41
Non-user	IOB_24	chr1	170633322	C	A	PRRX1	NCT	ENSG00000116132:[ENST00000553786(n.198-125C>A)]	NA	NA	chr1*170633322*170633322*C*T*rs77911930*1*0.9776*0.02236	NA	NA	NA	NA	NA	NA	NA	MODIFIER	NA	NA	90	81	0	60	30	30	50
Non-user	IOB_24	chr1	171070412	G	C	FMO3	NCT	ENSG00000007933:[ENST00000478457(n.445+65G>C)]	NA	NA	2.00E-03	NA	NA	2.00E-03	6.10E-03	NA	NA	NA	MODIFIER	NA	NA	59	59	0	41	19	22	53.66
Non-user	IOB_24	chr1	173876721	C	A	SERPINC1	INTRONIC	462:[XM_005245198.1(c.1010-69G>T)]	NA	NA	5.19E-03	NA	NA	5.19E-03	2.25E-02	NA	NA	NA	MODIFIER	NA	NA	133	133	0	106	53	52	49.06
Non-user	IOB_24	chr1	173910475	C	T	RC3H1	MISSENSE	ENSG00000135870:[ENST00000258349(c.3179G>A,aGt>aAt):ENSP00000258349(p.Ser1060Asn)]	NA	NA	7.99E-04	6.18E-04	4.54E-03	7.99E-04	4.10E-03	NA	NA	NA	MODIFIER	NA	NA	89	88	0	52	22	30	57.69
Non-user	IOB_24	chr1	175129946	C	T	KIAA0040	SILENT	ENSG00000235750:[ENST00000423313(c.204G>A,aaG>aaA):ENSP00000462172(p.Lys68(=))]	NA	NA	chr1*175129941*175129949*TTCTTCTTC*T*rs779364339*NA*NA*NA|chr1*175129941*175129955*TTCTTCTTCTTCTTG*T*rs759713799*NA*NA*NA|chr1*175129944*175129946*TTC*T*rs764422998*NA*NA*NA|chr1*175129944*175129952*TTCTTCTTC*T*rs542219168*NA*NA*NA|chr1*175129944*175129955*TTCTTCTTCTTG*T*rs756639306*NA*NA*NA|chr1*175129945*175129946*TC*T*rs754204844*NA*NA*NA|chr1*175129945*175129949*TCTTC*T*rs758082810*NA*NA*NA|chr1*175129945*175129955*TCTTCTTCTTG*T*rs796763013*NA*NA*NA|chr1*175129945*175129955*TCTTCTTCTTG*TT*rs386636937*NA*NA*NA|chr1*175129946*175129946*C*CTTCTTT,CTTT*rs779709912*NA*NA*NA|chr1*175129946*175129946*C*G,T*rs2072035*NA*NA*NA|chr1*175129946*175129955*CTTCTTCTT	5.91E-01	5.68E-01	NA	NA	NA	NA	NA	LOW	NA	NA	30	30	0	23	0	22	95.65
Non-user	IOB_24	chr1	175325625	G	T	TNR	INTRONIC	ENSG00000116147:[ENST00000263525(c.2975-27C>A)]	NA	NA	NA	NA	NA	NA	NA	NA	NA	NA	MODIFIER	NA	NA	61	61	0	36	17	19	52.78
Non-user	IOB_24	chr1	176679311	T	C	PAPPA2	MISSENSE-SS-PRX	ENSG00000116183:[ENST00000367662(c.3650T>C,cTa>cCa):ENSP00000356634(p.Leu1217Pro)]	NA	NA	3.59E-03	4.88E-03	2.62E-02	3.59E-03	1.64E-02	T	BN	NA	MODERATE	D	0.993	64	64	0	70	43	27	38.57
Non-user	IOB_24	chr1	176681005	A	G	PAPPA2	MISSENSE	ENSG00000116183:[ENST00000367662(c.3686A>G,cAc>cGc):ENSP00000356634(p.His1229Arg)]	NA	NA	1.60E-03	7.21E-04	5.21E-03	1.60E-03	8.20E-03	T	BN	NA	MODERATE	N	0.006	232	232	0	194	112	81	41.75
Non-user	IOB_24	chr1	177225257	C	A	BRINP2	INTRONIC	ENSG00000198797:[ENST00000361539(c.460+12C>A)]	NA	NA	chr1*177225257*177225257*C*T*rs753376799*NA*NA*NA	NA	NA	NA	NA	NA	NA	NA	MODIFIER	NA	NA	249	249	0	196	189	5	2.55
Non-user	IOB_24	chr1	178380859	G	A	RASAL2	INTRONIC	ENSG00000075391:[ENST00000448150(c.511-8787G>A)]	NA	NA	3.85E-02	NA	NA	3.85E-02	9.92E-02	NA	NA	NA	MODIFIER	NA	NA	110	110	0	75	43	32	42.67
Non-user	IOB_24	chr1	178380859	G	A	RP4-765C7.2	EXONIC-NC	ENSG00000213058:[ENST00000455621(n.109G>A)]	NA	NA	3.85E-02	NA	NA	3.85E-02	9.92E-02	NA	NA	NA	MODIFIER	NA	NA	110	110	0	75	43	32	42.67
Non-user	IOB_24	chr1	178876044	G	A	RALGPS2	INTRONIC	ENSG00000116191:[ENST00000367634(c.1644+42G>A)]	NA	NA	NA	1.78E-05	1.29E-04	NA	NA	NA	NA	NA	MODIFIER	NA	NA	83	83	0	59	29	30	50.85
Non-user	IOB_24	chr1	179051362	C	T	TOR3A	SILENT	ENSG00000186283:[ENST00000352445(c.99C>T,acC>acT):ENSP00000335351(p.Thr33(=))]	NA	NA	1.80E-03	4.60E-04	4.00E-04	1.80E-03	0.00E+00	NA	NA	NA	LOW	NA	NA	124	123	0	92	49	43	46.74
Non-user	IOB_24	chr1	183871646	T	C	RGL1	INTRONIC	ENSG00000143344:[ENST00000539189(c.1231-56T>C)]	NA	NA	2.16E-02	NA	NA	2.16E-02	4.09E-02	NA	NA	NA	MODIFIER	NA	NA	53	53	0	36	18	18	50
Non-user	IOB_24	chr1	186275564	C	T	PRG4	MISSENSE	ENSG00000116690:[ENST00000367485(c.434C>T,aCg>aTg):ENSP00000356455(p.Thr145Met)]	NA	NA	2.18E-02	3.81E-02	4.61E-02	2.18E-02	4.29E-02	T	BN	NA	MODERATE	NA	NA	205	204	1	207	85	122	58.94
Non-user	IOB_24	chr1	186281400	C	T	PRG4	MISSENSE	ENSG00000116690:[ENST00000367486(c.3758C>T,aCg>aTg):ENSP00000356456(p.Thr1253Met)]	NA	NA	3.06E-02	4.04E-02	4.62E-02	3.06E-02	4.40E-02	NA	PoD	NA	MODERATE	N	0	136	136	0	88	34	52	59.09
Non-user	IOB_24	chr1	186281400	C	T	TPR	3UTR	ENSG00000047410:[ENST00000367478(c.*1703G>A)]	NA	NA	3.06E-02	4.04E-02	4.62E-02	3.06E-02	4.40E-02	NA	PoD	NA	MODERATE	N	0	136	136	0	88	34	52	59.09

Patel *et al.* , 2021. Whole-exome sequencing analysis of oral squamous cell carcinoma delineated by tobacco usage habits
Supplementary Table 4. List of somatic single nucleotide variants identified using whole exome sequencing of OSCC patients

Cohort	Sample	Chromosome	Genomic position	Reference allele	Alternate allele	Genesymbol	Variant type	Variant description	OncoMD	COSMIC	dbSNP	ExAC	ExAC_SAS	1000G	1000G_SAS	SIFT	Polyphen2	CONDEL	VEP	LRT	PhastCon7Way	Total reads in Normal	Reference read support in Normal	Alternative read support in Normal	Total reads in Tumor	Reference read support in Tumor	Alternate read support in Tumor	Alternate allele frequency in Tumor
Non-user	IOB_24	chr1	186291512	T	C	TPR	SILENT	ENSG00000047410:[ENST00000367478(c.6399A>G,acA>acG):ENSP00000356448(p.Thr2133(=))]	NA	NA	3.02E-02	4.11E-02	4.71E-02	3.02E-02	4.40E-02	NA	NA	NA	MODIFIER	NA	NA	92	91	0	50	25	25	50
Non-user	IOB_24	chr1	186301447	T	C	TPR	SILENT	ENSG00000047410:[ENST00000367478(c.5484A>G,ccA>ccG):ENSP00000356448(p.Pro1828(=))]	NA	NA	3.02E-02	4.04E-02	4.63E-02	3.02E-02	4.40E-02	NA	NA	NA	LOW	NA	NA	95	95	0	82	47	34	41.46
Non-user	IOB_24	chr1	192335157	A	G	RGS21	MISSENSE	ENSG00000253148:[ENST00000417209(c.362A>G,aAg>aGg):ENSP00000428343(p.Lys121Arg)]	NA	NA	2.00E-04	2.66E-04	1.94E-03	2.00E-04	1.00E-03	T	BN	NA	MODERATE	U	1	143	141	1	116	57	59	50.86
Non-user	IOB_24	chr1	192627431	G	A	RGS13	SILENT	ENSG00000127074:[ENST00000391995(c.228G>A,cgG>cgA):ENSP00000375853(p.Arg76(=))]	NA	chr1*192627431*192627431*COSM901345*G*C*1*endometrium*1	2.12E-02	2.35E-02	2.72E-02	2.12E-02	2.56E-02	NA	NA	NA	LOW	NA	NA	142	140	0	151	68	82	54.3
Non-user	IOB_24	chr1	192778206	A	G	RGS2	MISSENSE	ENSG00000116741:[ENST00000235382(c.5A>G,cAa>cGa):ENSP00000235382(p.Gln2Arg)]	NA	NA	2.00E-04	5.36E-04	6.06E-05	2.00E-04	0.00E+00	NA	NA	NA	MODIFIER	NA	NA	95	95	0	69	31	38	55.07
Non-user	IOB_24	chr1	193038283	A	G	TROVE2	SILENT	ENSG00000116747:[ENST00000367441(c.99A>G,ctA>ctG):ENSP00000356411(p.Leu33(=))]	NA	NA	2.00E-04	4.97E-05	2.42E-04	2.00E-04	1.00E-03	NA	NA	NA	LOW	NA	NA	451	450	0	333	193	140	42.04
Non-user	IOB_24	chr1	193090877	C	G	NA	INTERGENIC	NA	NA	NA	5.99E-04	1.25E-03	2.00E-03	5.99E-04	3.10E-03	NA	NA	NA	MODIFIER	NA	NA	219	219	0	155	96	59	38.06
Non-user	IOB_24	chr1	196227832	A	C	KCNT2	INTRONIC	ENSG00000162687:[ENST00000609185(c.2710-208T>G)]	NA	NA	7.59E-03	1.73E-02	1.30E-02	7.59E-03	9.20E-03	NA	NA	NA	MODIFIER	NA	NA	28	28	0	20	4	16	80
Non-user	IOB_24	chr1	196712577	T	C	CFH	NCT-SS-PRX	ENSG00000000971:[ENST00000466229(n.6232-5T>C)]	NA	NA	chr1*196712577*196712577*T*A,C*rs513699*NA*NA*NA	2.59E-01	2.24E-01	NA	NA	NA	NA	NA	LOW	NA	NA	258	258	0	342	215	125	36.55
Non-user	IOB_24	chr1	196712586	C	T	CFH	SILENT	ENSG00000000971:[ENST00000367429(c.3138C>T,acC>acT):ENSP00000356399(p.Thr1046(=))]	NA	NA	NA	2.66E-01	2.29E-01	NA	NA	NA	NA	NA	LOW	NA	NA	273	273	0	378	227	148	39.15
Non-user	IOB_24	chr1	196797238	C	T	CFHR1	MISSENSE	ENSG00000244414:[ENST00000320493(c.469C>T,Cat>Tat):ENSP00000314299(p.His157Tyr)]	NA	NA	NA	2.61E-01	2.74E-01	NA	NA	NA	NA	NA	MODIFIER	NA	NA	290	290	0	94	0	94	100
Non-user	IOB_24	chr1	196797238	C	T	CFHR2	INTRONIC	ENSG00000080910:[ENST00000367421(c.58+8206C>T)]	NA	NA	NA	2.61E-01	2.74E-01	NA	NA	NA	NA	NA	MODIFIER	NA	NA	290	290	0	94	0	94	100
Non-user	IOB_24	chr1	196797244	C	G	CFHR1	MISSENSE	ENSG00000244414:[ENST00000320493(c.475C>G,Ctg>Gtg):ENSP00000314299(p.Leu159Val)]	NA	NA	NA	2.10E-01	2.17E-01	NA	NA	NA	NA	NA	MODIFIER	NA	NA	297	297	0	86	2	84	97.67
Non-user	IOB_24	chr1	196797244	C	G	CFHR2	INTRONIC	ENSG00000080910:[ENST00000367421(c.58+8212C>G)]	NA	NA	NA	2.10E-01	2.17E-01	NA	NA	NA	NA	NA	MODIFIER	NA	NA	297	297	0	86	2	84	97.67
Non-user	IOB_24	chr1	196797292	G	C	CFHR1	MISSENSE	ENSG00000244414:[ENST00000320493(c.523G>C,Gaa>Caa):ENSP00000314299(p.Glu175Gln)]	NA	NA	NA	7.99E-02	1.51E-01	NA	NA	NA	NA	NA	MODIFIER	NA	NA	284	283	0	38	0	38	100
Non-user	IOB_24	chr1	196797292	G	C	CFHR2	INTRONIC	ENSG00000080910:[ENST00000367421(c.58+8260G>C)]	NA	NA	NA	7.99E-02	1.51E-01	NA	NA	NA	NA	NA	MODIFIER	NA	NA	284	283	0	38	0	38	100
Non-user	IOB_24	chr1	196797357	A	G	CFHR1	SILENT	ENSG00000244414:[ENST00000320493(c.588A>G,acA>acG):ENSP00000314299(p.Thr196(=))]	NA	chr1*196797357*196797357*COSM1626718*A*G*1*liver*1	NA	1.40E-01	2.13E-01	NA	NA	NA	NA	NA	LOW	NA	NA	204	203	1	10	0	10	100
Non-user	IOB_24	chr1	196797357	A	G	CFHR2	INTRONIC	ENSG00000080910:[ENST00000367421(c.58+8325A>G)]	NA	chr1*196797357*196797357*COSM1626718*A*G*1*liver*1	NA	1.40E-01	2.13E-01	NA	NA	NA	NA	NA	LOW	NA	NA	204	203	1	10	0	10	100
Non-user	IOB_24	chr1	196884258	A	T	CFHR2	INTRONIC	ENSG00000080910:[ENST00000367421(c.59-34327A>T)]	NA	NA	NA	1.93E-01	1.42E-01	NA	NA	NA	NA	NA	LOW	NA	NA	132	132	0	227	166	59	25.99
Non-user	IOB_24	chr1	196884258	A	T	CFHR4	SILENT	ENSG00000134365:[ENST00000608469(c.399A>T,ccA>ccT):ENSP00000477162(p.Pro133(=))]	NA	NA	NA	1.93E-01	1.42E-01	NA	NA	NA	NA	NA	LOW	NA	NA	132	132	0	227	166	59	25.99
Non-user	IOB_24	chr1	196884290	T	A	CFHR2	INTRONIC	ENSG00000080910:[ENST00000367421(c.59-34295T>A)]	NA	NA	NA	2.03E-01	1.49E-01	NA	NA	NA	NA	NA	MODIFIER	NA	NA	77	77	0	139	104	34	24.46
Non-user	IOB_24	chr1	196884290	T	A	CFHR4	INTRONIC	10877:[NM_001201550.2(c.1540+22T>A)]	NA	NA	NA	2.03E-01	1.49E-01	NA	NA	NA	NA	NA	MODIFIER	NA	NA	77	77	0	139	104	34	24.46
Non-user	IOB_24	chr1	196965057	A	G	CFHR5	INTRONIC	ENSG00000134389:[ENST00000256785(c.790+28A>G)]	NA	NA	1.60E-03	1.80E-03	1.02E-02	1.60E-03	8.20E-03	NA	NA	NA	MODIFIER	NA	NA	122	122	0	89	48	41	46.07
Non-user	IOB_24	chr1	197060124	A	G	ASPM	SILENT	ENSG00000066279:[ENST00000294732(c.4737T>C,taT>taC):ENSP00000294732(p.Tyr1579(=))]	NA	NA	2.20E-03	2.32E-03	1.36E-02	2.20E-03	1.12E-02	NA	NA	NA	LOW	NA	0	149	149	0	85	40	45	52.94
Non-user	IOB_24	chr1	197070521	C	G	ASPM	MISSENSE	ENSG00000066279:[ENST00000367409(c.7860G>C,caG>caC):ENSP00000356379(p.Gln2620His)]	NA	NA	2.88E-02	5.11E-02	3.09E-02	2.88E-02	2.76E-02	T	BN	NA	MODERATE	N	0	87	86	0	61	36	25	40.98
Non-user	IOB_24	chr1	197509099	C	T	DENND1B	MISSENSE	ENSG00000213047:[ENST00000391979(c.430G>A,Gct>Act):ENSP00000375839(p.Ala144Thr)]	NA	NA	9.98E-04	6.39E-04	3.84E-03	9.98E-04	5.10E-03	NA	NA	NA	MODIFIER	NA	NA	43	43	0	59	31	28	47.46
Non-user	IOB_24	chr1	200587533	A	G	KIF14	SILENT	ENSG00000118193:[ENST00000367350(c.319T>C,Ttg>Ctg):ENSP00000356319(p.Leu107(=))]	NA	NA	NA	3.29E-05	2.42E-04	NA	NA	NA	NA	NA	MODIFIER	NA	NA	297	296	0	228	119	109	47.81
Non-user	IOB_24	chr1	201164930	A	G	IGFN1	INTRONIC	ENSG00000163395:[ENST00000335211(c.128-37A>G)]	NA	NA	4.63E-02	8.59E-02	1.23E-01	4.63E-02	8.69E-02	NA	NA	NA	MODIFIER	NA	NA	96	95	0	57	27	30	52.63
Non-user	IOB_24	chr1	202152763	C	T	PTPRVP	EXONIC-NC	ENSG00000243323:[ENST00000490225(n.3560C>T)]	NA	NA	3.99E-04	1.57E-03	2.41E-03	3.99E-04	2.00E-03	NA	NA	NA	MODIFIER	NA	NA	131	131	0	97	53	44	45.36
Non-user	IOB_24	chr1	203028323	G	A	PPFIA4	MISSENSE	ENSG00000143847:[ENST00000447715(c.2359G>A,Gaa>Aaa):ENSP00000402576(p.Glu787Lys)]	NA	NA	NA	4.16E-04	1.25E-03	NA	NA	D	PrD	NA	MODERATE	U	1	177	176	1	141	69	72	51.06
Non-user	IOB_24	chr1	203452409	G	A	PRELP	MISSENSE	ENSG00000188783:[ENST00000343110(c.97G>A,Ggg>Agg):ENSP00000343924(p.Gly33Arg)]	NA	NA	1.06E-02	1.36E-02	3.11E-02	1.06E-02	2.15E-02	T_lc	BN	NA	MODERATE	N	0	246	242	0	183	98	84	45.9
Non-user	IOB_24	chr1	203452720	C	T	PRELP	SILENT	ENSG00000188783:[ENST00000343110(c.408C>T,aaC>aaT):ENSP00000343924(p.Asn136(=))]	NA	NA	1.90E-02	1.72E-02	4.49E-02	1.90E-02	4.19E-02	NA	NA	NA	LOW	NA	0.999	273	273	0	203	113	88	43.35
Non-user	IOB_24	chr1	203471963	T	C	OPTC	INTRONIC	ENSG00000188770:[ENST00000367222(c.733-79T>C)]	NA	NA	1.80E-02	NA	NA	1.80E-02	3.89E-02	NA	NA	NA	MODIFIER	NA	NA	153	153	0	118	51	66	55.93
Non-user	IOB_24	chr1	203472119	G	A	OPTC	SILENT	ENSG00000188770:[ENST00000367222(c.810G>A,ctG>ctA):ENSP00000356191(p.Leu270(=))]	NA	NA	1.80E-02	1.68E-02	4.23E-02	1.80E-02	3.89E-02	NA	NA	NA	LOW	NA	NA	195	193	0	145	83	62	42.76
Non-user	IOB_24	chr1	203800919	T	G	ZC3H11A	INTRONIC	ENSG00000058673:[ENST00000332127(c.811+20T>G)]	NA	NA	3.99E-04	2.38E-04	1.52E-03	3.99E-04	2.00E-03	NA	NA	NA	MODIFIER	NA	NA	77	77	0	78	44	34	43.59
Non-user	IOB_24	chr1	204228851	G	A	PLEKHA6	MISSENSE	ENSG00000143850:[ENST00000414478(c.602C>T,tCg>tTg):ENSP00000402046(p.Ser201Leu)]	NA	NA	NA	8.54E-05	2.72E-04	NA	NA	T	PrD	NA	MODERATE	N	1	50	48	0	49	24	25	51.02
Non-user	IOB_24	chr1	204435989	G	A	PIK3C2B	SILENT	ENSG00000133056:[ENST00000367184(c.177C>T,tgC>tgT):ENSP00000356152(p.Cys59(=))]	NA	NA	2.40E-03	3.03E-03	1.53E-02	2.40E-03	1.23E-02	NA	NA	NA	MODIFIER	NA	NA	117	117	0	75	44	31	41.33
Non-user	IOB_24	chr1	205549933	G	A	MFSD4	MISSENSE	ENSG00000174514:[ENST00000367147(c.574G>A,Ggc>Agc):ENSP00000356115(p.Gly192Ser)]	NA	NA	2.12E-02	1.18E-02	2.47E-02	2.12E-02	2.86E-02	NA	NA	NA	MODIFIER	NA	NA	301	300	0	212	126	85	40.09
Non-user	IOB_24	chr1	205549998	C	T	MFSD4	SILENT	ENSG00000174514:[ENST00000367147(c.639C>T,gaC>gaT):ENSP00000356115(p.Asp213(=))]	NA	NA	2.16E-02	1.19E-02	2.49E-02	2.16E-02	2.86E-02	NA	NA	NA	LOW	NA	0	189	187	2	143	86	57	39.86
Non-user	IOB_24	chr1	205812879	A	G	PM20D1	MISSENSE	ENSG00000162877:[ENST00000367136(c.743T>C,aTg>aCg):ENSP00000356104(p.Met248Thr)]	NA	NA	1.60E-03	2.80E-03	2.19E-03	1.60E-03	3.10E-03	NA	NA	NA	MODIFIER	NA	NA	92	92	0	89	45	44	49.44
Non-user	IOB_24	chr1	206566195	C	T	SRGAP2	MISSENSE	23380:[NM_015326.3(c.379C>T,aCg>aTg):NP_056141.2(p.Thr127Met)]	NA	chr1*206566195*206566195*COSM1127171*C*T*1*prostate*1	NA	3.00E-01	2.43E-01	NA	NA	NA	NA	NA	LOW	NA	NA	143	143	0	170	129	41	24.12
Non-user	IOB_24	chr1	206767073	G	A	EIF2D	NONSENSE	ENSG00000143486:[ENST00000271764(c.1579C>T,Cga>Tga):ENSP00000271764(p.Arg527Ter)]	NA	NA	NA	3.31E-05	6.08E-05	NA	NA	NA	NA	NA	HIGH	NA	1	64	63	1	65	33	32	49.23
Non-user	IOB_24	chr1	207653364	A	G	CR2	MISSENSE	ENSG00000117322:[ENST00000367059(c.2791A>G,Att>Gtt):ENSP00000356026(p.Ile931Val)]	NA	NA	4.91E-02	6.40E-02	6.54E-02	4.91E-02	4.91E-02	T_lc	BN	NA	MODERATE	NA	NA	346	346	0	287	153	133	46.34
Non-user	IOB_24	chr1	207791434	A	G	CR1	MISSENSE	ENSG00000203710:[ENST00000367049(c.6908A>G,aAg>aGg):ENSP00000356016(p.Lys2303Arg)]	NA	NA	6.79E-03	1.53E-02	4.33E-03	6.79E-03	2.00E-03	NA	NA	NA	MODERATE	NA	NA	94	94	0	88	29	56	63.64
Non-user	IOB_24	chr1	207857222	G	A	CR1L	MISSENSE	ENSG00000197721:[ENST00000508064(c.383G>A,cGa>cAa):ENSP00000421736(p.Arg128Gln)]	NA	chr1*207857222*207857222*COSM1338451*G*A*1*large_intestine*1|chr1*207857222*207857222*COSM1338452*G*A*1*large_intestine*1	6.59E-03	1.54E-02	4.12E-03	6.59E-03	2.00E-03	NA	NA	NA	MODIFIER	NA	NA	601	598	0	436	207	226	51.83
Non-user	IOB_24	chr1	212209301	A	G	DTL	NCT	ENSG00000143476:[ENST00000475419(n.97+17A>G)]	NA	NA	1.60E-03	6.22E-04	1.52E-03	1.60E-03	1.00E-03	NA	NA	NA	MODIFIER	NA	NA	139	138	0	107	64	42	39.25
Non-user	IOB_24	chr1	212799139	A	G	FAM71A	MISSENSE	ENSG00000162771:[ENST00000294829(c.920A>G,cAg>cGg):ENSP00000294829(p.Gln307Arg)]	NA	NA	4.79E-03	1.27E-02	9.64E-03	4.79E-03	2.00E-03	T	BN	NA	MODERATE	NA	NA	185	184	1	156	76	80	51.28
Non-user	IOB_24	chr1	212799139	A	G	RP11-338C15.5	EXONIC-NC	ENSG00000235862:[ENST00000427949(n.975T>C)]	NA	NA	4.79E-03	1.27E-02	9.64E-03	4.79E-03	2.00E-03	T	BN	NA	MODERATE	NA	NA	185	184	1	156	76	80	51.28
Non-user	IOB_24	chr1	214813782	A	G	CENPF	MISSENSE	ENSG00000117724:[ENST00000366955(c.2101A>G,Atg>Gtg):ENSP00000355922(p.Met701Val)]	NA	NA	chr1*214813782*214813782*A*C,G*rs3795524*NA*NA*NA	8.23E-02	1.45E-01	NA	NA	T	BN	NA	MODERATE	N	0.01	152	152	0	125	51	74	59.2
Non-user	IOB_24	chr1	214814059	T	G	CENPF	MISSENSE	ENSG00000117724:[ENST00000366955(c.2378T>G,aTg>aGg):ENSP00000355922(p.Met793Arg)]	NA	NA	3.49E-02	4.43E-02	5.17E-02	3.49E-02	4.91E-02	T	BN	NA	MODERATE	N	0.038	182	181	0	125	65	60	48
Non-user	IOB_24	chr1	214821220	A	G	CENPF	INTRONIC	ENSG00000117724:[ENST00000366955(c.7830+477A>G)]	NA	NA	3.79E-02	NA	NA	3.79E-02	5.52E-02	NA	NA	NA	MODIFIER	NA	NA	183	182	1	146	67	79	54.11
Non-user	IOB_24	chr1	215752352	G	A	KCTD3	MISSENSE	ENSG00000136636:[ENST00000259154(c.407G>A,aGt>aAt):ENSP00000259154(p.Ser136Asn)]	NA	NA	NA	NA	NA	NA	NA	T	BN	NA	MODERATE	NA	NA	113	111	0	87	52	35	40.23
Non-user	IOB_24	chr1	217856622	A	G	SPATA17	MISSENSE	ENSG00000162814:[ENST00000366933(c.314A>G,tAt>tGt):ENSP00000355900(p.Tyr105Cys)]	NA	NA	2.58E-02	1.64E-02	3.26E-02	2.58E-02	5.11E-02	D	PrD	NA	MODERATE	D	1	44	44	0	33	20	13	39.39
Non-user	IOB_24	chr1	222705418	T	G	HHIPL2	MISSENSE	ENSG00000143512:[ENST00000343410(c.1613A>C,aAg>aCg):ENSP00000342118(p.Lys538Thr)]	NA	NA	1.60E-03	3.37E-03	4.40E-03	1.60E-03	2.00E-03	D	BN	NA	MODERATE	N	0.001	101	101	0	95	39	56	58.95
Non-user	IOB_24	chr1	222732051	C	T	TAF1A	MISSENSE	ENSG00000143498:[ENST00000366890(c.962G>A,cGg>cAg):ENSP00000355856(p.Arg321Gln)]	NA	chr1*222732051*222732051*COSM904280*C*T*1*endometrium*1	3.99E-03	6.26E-03	5.67E-03	3.99E-03	3.10E-03	NA	NA	NA	MODERATE	NA	0.039	44	44	0	36	25	11	30.56
Non-user	IOB_24	chr1	223933061	C	T	CAPN2	SILENT	ENSG00000162909:[ENST00000295006(c.480C>T,acC>acT):ENSP00000295006(p.Thr160(=))]	NA	NA	4.03E-02	6.52E-02	4.71E-02	4.03E-02	3.89E-02	NA	NA	NA	LOW	NA	NA	120	120	0	152	81	70	46.05
Non-user	IOB_24	chr1	223991119	G	T	TP53BP2	MISSENSE	ENSG00000143514:[ENST00000391878(c.298C>A,Caa>Aaa):ENSP00000375750(p.Gln100Lys)]	NA	NA	3.53E-02	6.64E-02	5.28E-02	3.53E-02	4.09E-02	D	PrD	NA	MODERATE	D	1	137	137	0	92	69	23	25
Non-user	IOB_24	chr1	225685473	T	C	ENAH	EXONIC-NC	ENSG00000154380:[ENST00000483952(n.441A>G)]	NA	NA	3.83E-02	5.73E-02	1.01E-01	3.83E-02	1.04E-01	NA	NA	NA	MODIFIER	NA	NA	19	19	0	26	17	9	34.62
Non-user	IOB_24	chr1	225706943	C	T	ENAH	SILENT	ENSG00000154380:[ENST00000366843(c.759G>A,agG>agA):ENSP00000355808(p.Arg253(=))]	NA	NA	3.83E-02	5.72E-02	1.01E-01	3.83E-02	1.04E-01	NA	NA	NA	LOW	NA	0.388	530	528	0	389	223	165	42.42
Non-user	IOB_24	chr1	225742588	A	G	ENAH	INTRONIC	ENSG00000154380:[ENST00000284563(c.349+20T>C)]	NA	NA	3.87E-02	5.81E-02	1.13E-01	3.87E-02	1.03E-01	NA	NA	NA	MODIFIER	NA	NA	178	178	0	119	69	49	41.18
Non-user	IOB_24	chr1	226127629	C	T	LEFTY2	SILENT	ENSG00000143768:[ENST00000366820(c.324G>A,ccG>ccA):ENSP00000355785(p.Pro108(=))]	NA	NA	9.98E-04	6.75E-03	1.76E-03	9.98E-04	0.00E+00	NA	NA	NA	MODIFIER	NA	NA	27	27	0	13	2	11	84.62
Non-user	IOB_24	chr1	226496968	C	G	LIN9	INTRONIC	286826:[NM_001270410.1(c.-126+359G>C)]	NA	NA	9.19E-03	1.69E-02	3.67E-02	9.19E-03	4.50E-02	NA	NA	NA	MODIFIER	NA	NA	67	66	0	70	39	31	44.29
Non-user	IOB_24	chr1	226555348	G	A	PARP1	INTRONIC	ENSG00000143799:[ENST00000366794(c.2278-39C>T)]	NA	NA	2.66E-02	4.59E-02	4.41E-02	2.66E-02	3.68E-02	NA	NA	NA	MODIFIER	NA	NA	110	110	0	89	49	40	44.94
Non-user	IOB_24	chr1	226567241	C	T	PARP1	INTRONIC	ENSG00000143799:[ENST00000366794(c.1612+33G>A)]	NA	NA	1.62E-02	1.96E-02	3.45E-02	1.62E-02	4.29E-02	NA	NA	NA	MODIFIER	NA	NA	89	88	0	64	39	25	39.06
Non-user	IOB_24	chr1	227920186	G	A	JMJD4	SILENT	ENSG00000081692:[ENST00000366758(c.1299C>T,gaC>gaT):ENSP00000355720(p.Asp433(=))]	NA	NA	1.96E-02	4.29E-02	1.90E-02	1.96E-02	1.53E-02	NA	NA	NA	MODIFIER	NA	NA	206	202	0	156	83	73	46.79
Non-user	IOB_24	chr1	227920186	G	A	LOC100130093	EXONIC-NC	100130093:[NR_024486.1(n.345G>A)]	NA	NA	1.96E-02	4.29E-02	1.90E-02	1.96E-02	1.53E-02	NA	NA	NA	MODIFIER	NA	NA	206	202	0	156	83	73	46.79
Non-user	IOB_24	chr1	227920186	G	A	SNAP47	NCT	ENSG00000143740:[ENST00000491439(n.243+738G>A)]	NA	NA	1.96E-02	4.29E-02	1.90E-02	1.96E-02	1.53E-02	NA	NA	NA	MODIFIER	NA	NA	206	202	0	156	83	73	46.79
Non-user	IOB_24	chr1	228362386	C	A	IBA57	INTRONIC-SS-ACR-PRX	ENSG00000181873:[ENST00000546123(c.-238-7C>A)]	NA	NA	4.39E-03	4.32E-03	4.44E-02	4.39E-03	2.25E-02	NA	NA	NA	LOW	NA	NA	136	136	0	93	43	49	52.69
Non-user	IOB_24	chr1	228444563	G	A	OBSCN	SILENT	ENSG00000154358:[ENST00000284548(c.4521G>A,gcG>gcA):ENSP00000284548(p.Ala1507(=))]	NA	NA	4.59E-03	2.17E-03	1.52E-02	4.59E-03	2.35E-02	NA	NA	NA	MODIFIER	NA	NA	133	132	0	117	53	62	52.99
Non-user	IOB_24	chr1	228504507	G	T	OBSCN	SILENT	ENSG00000154358:[ENST00000284548(c.13383G>T,gtG>gtT):ENSP00000284548(p.Val4461(=))]	NA	NA	4.99E-03	1.71E-02	2.09E-03	4.99E-03	0.00E+00	NA	NA	NA	LOW	NA	NA	162	162	0	131	66	65	49.62
Non-user	IOB_24	chr1	228509677	C	T	OBSCN	SILENT	ENSG00000154358:[ENST00000284548(c.15135C>T,ccC>ccT):ENSP00000284548(p.Pro5045(=))]	NA	NA	1.20E-03	9.79E-04	6.18E-03	1.20E-03	6.10E-03	NA	NA	NA	LOW	NA	NA	224	222	0	165	84	80	48.48
Non-user	IOB_24	chr1	228565329	G	A	OBSCN	MISSENSE	ENSG00000154358:[ENST00000422127(c.23419G>A,Gtc>Atc):ENSP00000409493(p.Val7807Ile)]	NA	NA	4.99E-03	1.49E-02	1.76E-03	4.99E-03	0.00E+00	NA	NA	NA	MODERATE	NA	NA	138	138	0	88	49	38	43.18

Patel *et al.* , 2021. Whole-exome sequencing analysis of oral squamous cell carcinoma delineated by tobacco usage habits
Supplementary Table 4. List of somatic single nucleotide variants identified using whole exome sequencing of OSCC patients

Cohort	Sample	Chromosome	Genomic position	Reference allele	Alternate allele	Genesymbol	Variant type	Variant description	OncoMD	COSMIC	dbSNP	ExAC	ExAC_SAS	1000G	1000G_SAS	SIFT	Polyphen2	CONDEL	VEP	LRT	PhastCon7Way	Total reads in Normal	Reference read support in Normal	Alternative read support in Normal	Total reads in Tumor	Reference read support in Tumor	Alternate read support in Tumor	Alternate allele frequency in Tumor
Non-user	IOB_24	chr1	228588713	G	A	TRIM11	SILENT	ENSG00000154370:[ENST00000493030(c.312C>T,atC>atT):ENSP00000473360(p.Ile104(=))]	NA	NA	3.20E-03	1.25E-02	1.21E-03	3.19E-03	0.00E+00	NA	NA	NA	LOW	NA	0.043	97	97	0	76	42	34	44.74
Non-user	IOB_24	chr1	228675570	G	C	RNF187	SILENT	ENSG00000168159:[ENST00000305943(c.54G>C,ctG>ctC):ENSP00000306396(p.Leu18(=))]	NA	NA	4.53E-02	2.21E-01	1.97E-01	4.53E-02	5.42E-02	NA	NA	NA	LOW	NA	NA	9	9	0	11	6	5	45.45
Non-user	IOB_24	chr1	228698311	C	A	BTNL10	MISSENSE	100129094:[NM_001287262.1(c.376G>T,Gca>Tca):NP_001274191.1(p.Ala126Ser)]	NA	NA	2.98E-02	4.71E-02	5.62E-02	2.98E-02	4.60E-02	NA	NA	NA	MODIFIER	NA	NA	95	94	1	110	57	53	48.18
Non-user	IOB_24	chr1	230461229	C	G	PGBD5	INTRONIC	ENSG00000177614:[ENST00000525115(c.1067-68G>C)]	NA	NA	1.20E-02	NA	NA	1.20E-02	8.20E-03	NA	NA	NA	MODIFIER	NA	NA	106	106	0	87	38	49	56.32
Non-user	IOB_24	chr1	231954145	G	A	DISC1	SILENT	ENSG00000162946:[ENST00000366633(c.1863G>A,ctG>ctA):ENSP00000355593(p.Leu621(=))]	NA	NA	3.24E-02	5.12E-02	4.37E-02	3.23E-02	3.48E-02	NA	NA	NA	MODIFIER	NA	NA	68	66	0	50	32	17	34
Non-user	IOB_24	chr1	231954145	G	A	DISC2	EXONIC-NC	27184:[NR_002227.2(n.119C>T)]	NA	NA	3.24E-02	5.12E-02	4.37E-02	3.23E-02	3.48E-02	NA	NA	NA	MODIFIER	NA	NA	68	66	0	50	32	17	34
Non-user	IOB_24	chr1	231954145	G	A	TSNAX-DISC1	EXONIC-NC	100303453:[NR_028398.1(n.2203G>A)]	NA	NA	3.24E-02	5.12E-02	4.37E-02	3.23E-02	3.48E-02	NA	NA	NA	MODIFIER	NA	NA	68	66	0	50	32	17	34
Non-user	IOB_24	chr1	232002332	C	G	DISC1	INTRONIC	ENSG00000162946:[ENST00000366637(c.-24+48069C>G)]	NA	NA	4.21E-02	5.04E-02	6.28E-02	4.21E-02	5.93E-02	NA	NA	NA	MODIFIER	NA	NA	83	83	0	56	26	29	51.79
Non-user	IOB_24	chr1	232002332	C	G	TSNAX-DISC1	NCT	100303453:[NR_028393.1(n.2647+48069C>G)]	NA	NA	4.21E-02	5.04E-02	6.28E-02	4.21E-02	5.93E-02	NA	NA	NA	MODIFIER	NA	NA	83	83	0	56	26	29	51.79
Non-user	IOB_24	chr1	232596728	G	A	SIPA1L2	SILENT	ENSG00000116991:[ENST00000308942(c.222C>T,gcC>gcT):ENSP00000309102(p.Ala74(=))]	NA	NA	2.74E-02	5.39E-02	2.76E-02	2.74E-02	1.84E-02	NA	NA	NA	LOW	NA	NA	221	219	0	163	78	84	51.53
Non-user	IOB_24	chr1	233112095	A	G	NTPCR	MISSENSE	ENSG00000135778:[ENST00000366627(c.512A>G,tAc>tGc):ENSP00000355586(p.Tyr171Cys)]	NA	NA	4.73E-02	9.64E-02	1.20E-01	4.73E-02	7.26E-02	NA	NA	NA	MODIFIER	NA	NA	57	57	0	57	39	18	31.58
Non-user	IOB_24	chr1	233136261	G	A	PCNXL2	SILENT	ENSG00000135749:[ENST00000344698(c.1074C>T,gaC>gaT):ENSP00000340759(p.Asp358(=))]	NA	NA	1.80E-03	1.93E-03	7.95E-03	1.80E-03	4.10E-03	NA	NA	NA	LOW	NA	0.016	131	130	0	119	64	54	45.38
Non-user	IOB_24	chr1	234455926	G	A	SLC35F3	SILENT	ENSG00000183780:[ENST00000366617(c.1023G>A,gtG>gtA):ENSP00000355576(p.Val341(=))]	NA	NA	2.00E-04	3.30E-04	3.03E-04	2.00E-04	0.00E+00	NA	NA	NA	LOW	NA	NA	213	212	0	189	102	86	45.5
Non-user	IOB_24	chr1	234744480	G	A	IRF2BP2	MISSENSE	ENSG00000168264:[ENST00000366609(c.761C>T,gCg>gTg):ENSP00000355568(p.Ala254Val)]	NA	NA	3.63E-02	1.45E-01	6.65E-02	3.63E-02	2.25E-02	NA	NA	NA	MODIFIER	NA	NA	44	44	0	42	18	24	57.14
Non-user	IOB_24	chr1	234744480	G	A	RP4-781K5.2	NCT	ENSG00000228830:[ENST00000436039(n.631-687G>A)]	NA	NA	3.63E-02	1.45E-01	6.65E-02	3.63E-02	2.25E-02	NA	NA	NA	MODIFIER	NA	NA	44	44	0	42	18	24	57.14
Non-user	IOB_24	chr1	235972981	C	A	LYST	MISSENSE	ENSG00000143669:[ENST00000389793(c.1137G>T,aaG>aaT):ENSP00000374443(p.Lys379Asn)]	NA	NA	NA	NA	NA	NA	NA	NA	NA	NA	MODIFIER	NA	NA	272	272	0	196	190	5	2.55
Non-user	IOB_24	chr1	236142318	G	A	NID1	MISSENSE	ENSG00000116962:[ENST00000366595(c.3200C>T,aCg>aTg):ENSP00000355554(p.Thr1067Met)]	NA	chr1*236142318*236142318*COSM905941*G*A*1*endometrium*1	1.80E-03	1.33E-03	9.21E-03	1.80E-03	9.20E-03	D	PoD	NA	MODERATE	D	1	169	168	0	154	78	76	49.35
Non-user	IOB_24	chr1	236716833	T	C	HEATR1	INTRONIC	ENSG00000119285:[ENST00000366581(c.5994+48A>G)]	NA	NA	3.83E-02	3.87E-02	3.94E-02	3.83E-02	3.48E-02	NA	NA	NA	MODIFIER	NA	NA	113	113	0	89	47	42	47.19
Non-user	IOB_24	chr1	236716994	T	C	HEATR1	MISSENSE	ENSG00000119285:[ENST00000366582(c.6124A>G,Aca>Gca):ENSP00000355541(p.Thr2042Ala)]	NA	chr1*236716994*236716994*COSM906028*T*C*1*endometrium*1	9.98E-04	5.36E-04	3.94E-03	9.98E-04	5.10E-03	T	BN	NA	MODERATE	N	1	108	107	0	85	37	47	55.29
Non-user	IOB_24	chr1	236740135	T	C	HEATR1	MISSENSE	ENSG00000119285:[ENST00000366581(c.2870A>G,gAt>gGt):ENSP00000355540(p.Asp957Gly)]	NA	NA	3.77E-02	3.80E-02	3.64E-02	3.77E-02	3.37E-02	T	BN	NA	MODERATE	N	1	100	99	0	80	38	42	52.5
Non-user	IOB_24	chr1	236918327	C	T	ACTN2	SILENT	ENSG00000077522:[ENST00000366578(c.1983C>T,gcC>gcT):ENSP00000355537(p.Ala661(=))]	NA	NA	NA	1.73E-04	6.06E-04	NA	NA	NA	NA	NA	LOW	NA	NA	70	70	0	53	30	21	39.62
Non-user	IOB_24	chr1	237655173	A	T	RYR2	SILENT	ENSG00000198626:[ENST00000542537(c.1728A>T,ggA>ggT):ENSP00000443798(p.Gly576(=))]	NA	NA	2.16E-02	2.87E-02	7.99E-03	2.16E-02	2.00E-03	NA	NA	NA	LOW	NA	NA	20	20	0	20	12	8	40
Non-user	IOB_24	chr1	237798342	G	A	RYR2	INTRONIC	ENSG00000198626:[ENST00000366574(c.6792+50G>A)]	NA	NA	2.94E-02	7.77E-02	1.51E-02	2.94E-02	7.20E-03	NA	NA	NA	MODIFIER	NA	NA	126	126	0	82	44	38	46.34
Non-user	IOB_24	chr1	237823256	A	C	RYR2	INTRONIC	ENSG00000198626:[ENST00000366574(c.8209-29A>C)]	NA	NA	4.07E-02	6.05E-02	8.14E-03	4.07E-02	3.10E-03	NA	NA	NA	MODIFIER	NA	NA	61	61	0	50	26	24	48
Non-user	IOB_24	chr1	240655560	C	A	GREM2	3UTR	ENSG00000180875:[ENST00000318160(c.*709G>T)]	NA	NA	4.11E-02	6.98E-02	7.71E-02	4.11E-02	4.50E-02	NA	NA	NA	MODIFIER	NA	NA	88	88	0	79	46	33	41.77
Non-user	IOB_24	chr1	242035444	G	C	EXO1	MISSENSE	ENSG00000174371:[ENST00000348581(c.1378G>C,Gtg>Ctg):ENSP00000311873(p.Val460Leu)]	NA	NA	9.19E-03	1.15E-02	3.34E-02	9.19E-03	3.27E-02	NA	NA	NA	MODERATE	NA	0	316	315	0	223	107	115	51.57
Non-user	IOB_24	chr1	245849342	G	A	KIF26B	SILENT	ENSG00000162849:[ENST00000366518(c.1914G>A,ccG>ccA):ENSP00000355475(p.Pro638(=))]	NA	NA	1.68E-02	6.28E-02	1.93E-02	1.68E-02	1.12E-02	NA	NA	NA	MODIFIER	NA	NA	187	185	0	130	55	75	57.69
Non-user	IOB_24	chr1	247024352	C	T	AHCTF1	SILENT	ENSG00000153207:[ENST00000326225(c.4008G>A,ccG>ccA):ENSP00000355465(p.Pro1336(=))]	NA	NA	NA	1.48E-04	3.63E-04	NA	NA	NA	NA	NA	MODIFIER	NA	NA	91	91	0	56	27	29	51.79
Non-user	IOB_24	chr1	247094263	G	A	AHCTF1	INTRONIC	ENSG00000153207:[ENST00000478568(c.-8+617C>T)]	NA	NA	3.69E-02	NA	NA	3.69E-02	1.53E-02	NA	NA	NA	MODIFIER	NA	NA	93	93	0	63	33	30	47.62
Non-user	IOB_24	chr1	247094302	A	G	AHCTF1	INTRONIC	ENSG00000153207:[ENST00000478568(c.-8+578T>C)]	NA	NA	3.69E-02	NA	NA	3.69E-02	1.53E-02	NA	NA	NA	MODIFIER	NA	NA	116	116	0	83	44	39	46.99
Non-user	IOB_24	chr1	247130709	A	G	ZNF670	INTRONIC	ENSG00000135747:[ENST00000465049(c.*207+288T>C)]	NA	NA	4.49E-02	1.81E-02	2.13E-02	4.49E-02	1.74E-02	NA	NA	NA	MODIFIER	NA	NA	86	85	1	54	33	21	38.89
Non-user	IOB_24	chr1	247130709	A	G	ZNF670-ZNF695	NCT	100533111:[NR_037894.1(n.838+288T>C)]	NA	NA	4.49E-02	1.81E-02	2.13E-02	4.49E-02	1.74E-02	NA	NA	NA	MODIFIER	NA	NA	86	85	1	54	33	21	38.89
Non-user	IOB_24	chr1	247130709	A	G	ZNF695	INTRONIC	ENSG00000197472:[ENST00000487338(c.488+288T>C)]	NA	NA	4.49E-02	1.81E-02	2.13E-02	4.49E-02	1.74E-02	NA	NA	NA	MODIFIER	NA	NA	86	85	1	54	33	21	38.89
Non-user	IOB_24	chr1	247275218	C	G	C1orf229	SILENT	ENSG00000221953:[ENST00000408893(c.309G>C,gcG>gcC):ENSP00000386203(p.Ala103(=))]	NA	NA	3.59E-02	1.12E-01	7.61E-02	3.59E-02	5.42E-02	NA	NA	NA	LOW	NA	NA	49	49	0	32	13	19	59.38
Non-user	IOB_24	chr1	247588858	C	A	NLRP3	MISSENSE	ENSG00000162711:[ENST00000336119(c.2113C>A,Cag>Aag):ENSP00000337383(p.Gln705Lys)]	NA	NA	2.24E-02	4.09E-02	3.64E-02	2.24E-02	3.68E-02	NA	NA	NA	MODERATE	NA	0	62	62	0	52	22	30	57.69
Non-user	IOB_24	chr1	247592842	T	C	NLRP3	INTRONIC	ENSG00000162711:[ENST00000348069(c.2156+3941T>C)]	NA	NA	NA	1.93E-04	1.33E-03	NA	NA	NA	NA	NA	MODIFIER	NA	NA	98	98	0	77	36	41	53.25
Non-user	IOB_24	chr1	247769217	T	G	OR2G3	SILENT	ENSG00000177476:[ENST00000320002(c.330T>G,acT>acG):ENSP00000326301(p.Thr110(=))]	NA	NA	3.67E-02	7.46E-02	3.82E-02	3.67E-02	2.97E-02	NA	NA	NA	MODIFIER	NA	NA	413	410	0	284	135	149	52.46
Non-user	IOB_24	chr1	247769217	T	G	RP11-978I15.10	NCT	ENSG00000236817:[ENST00000435333(n.225+29940A>C)]	NA	NA	3.67E-02	7.46E-02	3.82E-02	3.67E-02	2.97E-02	NA	NA	NA	MODIFIER	NA	NA	413	410	0	284	135	149	52.46
Non-user	IOB_24	chr1	248031225	A	T	TRIM58	INTRONIC	25893:[XM_005273092.1(c.339-40A>T)]	NA	NA	1.80E-03	8.84E-04	4.39E-03	1.80E-03	9.20E-03	NA	NA	NA	MODIFIER	NA	NA	51	51	0	43	24	18	41.86
Non-user	IOB_24	chr1	248059251	C	T	OR2W3	SILENT	ENSG00000238243:[ENST00000360358(c.363C>T,gaC>gaT):ENSP00000353516(p.Asp121(=))]	NA	NA	NA	2.47E-05	1.82E-04	NA	NA	NA	NA	NA	LOW	NA	0.171	249	247	0	163	87	76	46.63
Non-user	IOB_24	chr1	248637381	G	A	OR2T3	MISSENSE	ENSG00000196539:[ENST00000359594(c.730G>A,Gcc>Acc):ENSP00000352604(p.Ala244Thr)]	NA	chr1*248637381*248637381*COSM1473785*G*A*3*breast*3	NA	1.23E-02	1.31E-02	NA	NA	T	BN	NA	MODERATE	NA	NA	368	367	0	194	157	36	18.56
Non-user	IOB_24	chr1	248737329	C	T	OR2T34	MISSENSE	ENSG00000183310:[ENST00000328782(c.730G>A,Gcc>Acc):ENSP00000330904(p.Ala244Thr)]	NA	NA	NA	1.41E-01	2.15E-01	NA	NA	T	BN	NA	MODERATE	NA	NA	335	330	0	306	224	80	26.14
Non-user	IOB_24	chr1	248737454	A	G	OR2T34	MISSENSE	ENSG00000183310:[ENST00000328782(c.605T>C,aTg>aCg):ENSP00000330904(p.Met202Thr)]	NA	NA	NA	1.37E-01	2.18E-01	NA	NA	T	BN	NA	MODERATE	NA	NA	754	747	0	701	569	131	18.69
Non-user	IOB_24	chr10	1094906	C	T	IDI1	MISSENSE	ENSG00000067064:[ENST00000381344(c.38G>A,tGc>tAc):ENSP00000370748(p.Cys13Tyr)]	NA	NA	3.08E-02	9.61E-02	1.22E-01	3.08E-02	9.20E-02	NA	NA	NA	MODIFIER	NA	NA	128	128	0	115	54	61	53.04
Non-user	IOB_24	chr10	1313160	G	A	ADARB2	SILENT	ENSG00000185736:[ENST00000381312(c.1182C>T,gtC>gtT):ENSP00000370713(p.Val394(=))]	NA	NA	2.00E-03	6.80E-04	4.93E-03	2.00E-03	1.02E-02	NA	NA	NA	LOW	NA	NA	142	142	0	153	81	72	47.06
Non-user	IOB_24	chr10	3177855	C	A	PFKP	INTRONIC	5214:[NM_001323067.1(c.2042-73C>A)]	NA	NA	6.99E-03	NA	NA	6.99E-03	3.58E-02	NA	NA	NA	MODIFIER	NA	NA	74	74	0	44	23	21	47.73
Non-user	IOB_24	chr10	3823759	G	A	KLF6	SILENT	ENSG00000067082:[ENST00000469435(c.750C>T,gcC>gcT):ENSP00000419079(p.Ala250(=))]	NA	NA	1.20E-03	1.91E-03	5.10E-03	1.20E-03	4.10E-03	NA	NA	NA	MODIFIER	NA	NA	181	180	0	167	61	105	62.87
Non-user	IOB_24	chr10	4867293	C	G	AKR1E2	NCT	ENSG00000165568:[ENST00000462718(n.53-5574C>G)]	NA	NA	3.99E-04	NA	NA	3.99E-04	2.00E-03	NA	NA	NA	MODIFIER	NA	NA	50	50	0	34	17	17	50
Non-user	IOB_24	chr10	5037914	G	C	AKR1C2	NCT	ENSG00000151632:[ENST00000460124(n.2140+34C>G)]	NA	NA	3.44E-02	6.56E-02	4.43E-02	3.43E-02	2.97E-02	NA	NA	NA	MODIFIER	NA	NA	50	50	0	90	39	50	55.56
Non-user	IOB_24	chr10	5037914	G	C	RP11-499O7.7	NCT	ENSG00000224251:[ENST00000451575(n.180+55G>C)]	NA	NA	3.44E-02	6.56E-02	4.43E-02	3.43E-02	2.97E-02	NA	NA	NA	MODIFIER	NA	NA	50	50	0	90	39	50	55.56
Non-user	IOB_24	chr10	5041398	T	C	AKR1C2	SILENT	ENSG00000151632:[ENST00000380753(c.441A>G,acA>acG):ENSP00000370129(p.Thr147(=))]	NA	NA	NA	3.44E-01	2.87E-01	NA	NA	NA	NA	NA	MODIFIER	NA	NA	105	104	0	84	48	36	42.86
Non-user	IOB_24	chr10	5202902	C	T	AKR1CL1	INTRONIC	ENSG00000196326:[ENST00000488756(c.379-50G>A)]	NA	NA	3.00E-03	5.96E-03	1.18E-02	3.00E-03	1.53E-02	NA	NA	NA	MODIFIER	NA	NA	114	114	0	105	61	44	41.9
Non-user	IOB_24	chr10	5803475	G	T	FAM208B	INTRONIC	ENSG00000108021:[ENST00000328090(c.7174+41G>T)]	NA	NA	NA	1.71E-05	1.41E-04	NA	NA	NA	NA	NA	MODIFIER	NA	NA	191	188	0	119	58	61	51.26
Non-user	IOB_24	chr10	6061479	A	C	IL2RA	INTRONIC	3559:[NM_001308243.1(c.368-17T>G)]	NA	NA	4.59E-02	9.25E-02	9.30E-02	4.59E-02	7.57E-02	NA	NA	NA	MODIFIER	NA	NA	99	99	0	77	38	39	50.65
Non-user	IOB_24	chr10	6061499	C	T	IL2RA	INTRONIC	ENSG00000134460:[ENST00000256876(c.629-37G>A)]	NA	NA	4.55E-02	9.25E-02	9.28E-02	4.55E-02	7.36E-02	NA	NA	NA	MODIFIER	NA	NA	87	87	0	64	33	29	45.31
Non-user	IOB_24	chr10	12139884	C	T	DHTKD1	SILENT	ENSG00000181192:[ENST00000448829(c.214C>T,acC>acT):ENSP00000398482(p.Thr72(=))]	NA	NA	1.38E-02	2.63E-02	1.34E-02	1.38E-02	6.10E-03	NA	NA	NA	LOW	NA	NA	189	188	0	158	79	77	48.73
Non-user	IOB_24	chr10	12199893	G	A	SEC61A2	INTRONIC	ENSG00000065665:[ENST00000441368(c.514-14G>A)]	NA	NA	1.34E-02	2.49E-02	1.36E-02	1.34E-02	6.10E-03	NA	NA	NA	MODIFIER	NA	NA	172	172	0	90	56	34	37.78
Non-user	IOB_24	chr10	12620763	T	G	CAMK1D	NCT	ENSG00000183049:[ENST00000487696(n.260-87972T>G)]	NA	NA	4.77E-02	7.71E-03	NA	4.77E-02	0.00E+00	NA	NA	NA	MODIFIER	NA	NA	48	47	1	33	15	17	51.52
Non-user	IOB_24	chr10	12620763	T	G	MIR4480	EXONIC-NC	ENSG00000263584:[ENST00000582492(n.12T>G)]	NA	NA	4.77E-02	7.71E-03	NA	4.77E-02	0.00E+00	NA	NA	NA	MODIFIER	NA	NA	48	47	1	33	15	17	51.52
Non-user	IOB_24	chr10	13350486	C	T	NA	INTERGENIC	NA	NA	NA	2.20E-03	1.63E-03	1.36E-02	2.20E-03	1.12E-02	NA	NA	NA	MODIFIER	NA	NA	125	125	0	78	40	38	48.72
Non-user	IOB_24	chr10	13633930	T	G	PRPF18	INTRONIC	ENSG00000165630:[ENST00000378572(c.66+4778T>G)]	NA	NA	NA	9.50E-02	8.15E-02	NA	NA	NA	NA	NA	MODIFIER	NA	NA	75	74	0	55	45	8	14.55
Non-user	IOB_24	chr10	13633930	T	G	RP11-295P9.2	EXONIC-NC	ENSG00000227474:[ENST00000445338(n.394A>C)]	NA	NA	NA	9.50E-02	8.15E-02	NA	NA	NA	NA	NA	MODIFIER	NA	NA	75	74	0	55	45	8	14.55
Non-user	IOB_24	chr10	14920929	A	C	SUV39H2	5UTR	ENSG00000152455:[ENST00000420416(c.-34A>C)]	NA	NA	1.28E-02	2.94E-02	4.99E-02	1.28E-02	6.03E-02	NA	NA	NA	MODIFIER	NA	NA	222	221	0	210	108	101	48.1
Non-user	IOB_24	chr10	15121165	A	G	ACBD7	INTRONIC	ENSG00000176244:[ENST00000356189(c.13-126T>C)]	NA	NA	3.47E-02	NA	NA	3.47E-02	4.29E-02	NA	NA	NA	MODIFIER	NA	NA	113	113	0	81	41	40	49.38
Non-user	IOB_24	chr10	15121165	A	G	DCLRE1CP1	NCT	ENSG00000237470:[ENST00000378211(n.61-126T>C)]	NA	NA	3.47E-02	NA	NA	3.47E-02	4.29E-02	NA	NA	NA	MODIFIER	NA	NA	113	113	0	81	41	40	49.38
Non-user	IOB_24	chr10	15130794	C	G	NA	INTERGENIC	NA	NA	NA	3.91E-02	NA	NA	3.91E-02	4.81E-02	NA	NA	NA	MODIFIER	NA	NA	152	152	0	144	78	65	45.14
Non-user	IOB_24	chr10	15146061	C	G	NMT2	NCT	ENSG00000152465:[ENST00000466201(n.128-379G>C)]	NA	NA	4.51E-02	5.54E-02	5.35E-02	4.51E-02	4.70E-02	NA	NA	NA	MODIFIER	NA	NA	132	132	0	145	60	83	57.24
Non-user	IOB_24	chr10	15146061	C	G	RPP38	MISSENSE	ENSG00000152464:[ENST00000378197(c.748C>G,Cgg>Ggg):ENSP00000367439(p.Arg250Gly)]	NA	NA	4.51E-02	5.54E-02	5.35E-02	4.51E-02	4.70E-02	NA	NA	NA	MODIFIER	NA	NA	132	132	0	145	60	83	57.24
Non-user	IOB_24	chr10	16946125	T	C	CUBN	INTRONIC	ENSG00000107611:[ENST00000377833(c.7913-11A>G)]	NA	NA	2.80E-02	5.78E-02	6.94E-02	2.80E-02	5.11E-02	NA	NA	NA	MODIFIER	NA	NA	191	190	0	142	68	73	51.41
Non-user	IOB_24	chr10	16989272	G	A	CUBN	SILENT	ENSG00000107611:[ENST00000377833(c.5304C>T,atC>atT):ENSP00000367064(p.Ile1768(=))]	NA	NA	2.92E-02	6.09E-02	4.91E-02	2.92E-02	2.97E-02	NA	NA	NA	LOW	NA	0.035	80	80	0	65	24	41	63.08
Non-user	IOB_24	chr10	17164569	G	A	CUBN	SILENT	ENSG00000107611:[ENST00000433666(c.270C>T,aaC>aaT):ENSP00000415970(p.Asn90(=))]	NA	NA	5.99E-03	1.21E-02	1.12E-02	5.99E-03	1.02E-02	NA	NA	NA	LOW	NA	NA	177	177	0	137	71	66	48.18
Non-user	IOB_24	chr10	18122716	A	G	MRC1	SILENT	ENSG00000120586:[ENST00000239761(c.726A>G,caA>caG):ENSP00000239761(p.Gln242(=))]	NA	NA	NA	2.22E-01	2.48E-01	NA	NA	NA	NA	NA	LOW	NA	NA	100	100	0	32	0	31	96.88
Non-user	IOB_24	chr10	19568935	A	G	MALRD1	INTRONIC	ENSG00000204740:[ENST00000377266(c.1007-41A>G)]	NA	NA	4.39E-03	7.45E-03	1.55E-02	4.39E-03	1.33E-02	NA	NA	NA	MODIFIER	NA	NA	226	224	0	159	80	79	49.69
Non-user	IOB_24	chr10	19569100	G	T	MALRD1	MISSENSE	ENSG00000204740:[ENST00000454679(c.1092G>T,caG>caT):ENSP00000412763(p.Gln364His)]	NA	NA	4.39E-03	1.24E-02	1.49E-02	4.39E-03	1.33E-02	D	PoD	NA	MODERATE	NA	NA	179	176	0	152	67	84	55.26
Non-user	IOB_24	chr10	21805292	A	G	SKIDA1	MISSENSE	ENSG00000180592:[ENST00000449193(c.1460T>C,cTg>cCg):ENSP00000410041(p.Leu487Pro)]	NA	NA	NA	8.49E-06	0.00E+00	NA	NA	T_lc	BN	NA	MODERATE	N	1	166	166	0	103	43	59	57.28
Non-user	IOB_24	chr10	22024199	G	C	MLLT10	INTRONIC	8028:[XM_005252611.1(c.2268+35G>C)]	NA	NA	NA	NA	NA	NA	NA	NA	NA	NA	MODIFIER	NA	NA	92	91	0	52	22	30	57.69
Non-user	IOB_24	chr10	23409809	G	A	MSRB2	3UTR	ENSG00000148450:[ENST00000472663(c.*176G>A)]	NA	NA	9.59E-03	1.30E-02	1.20E-02	9.58E-03	1.12E-02	NA	NA	NA	MODIFIER	NA	NA	228	228	0	234	122	110	47.01
Non-user	IOB_24	chr10	23728990	C	T	OTUD1	SILENT	ENSG00000165312:[ENST00000376495(c.604C>T,Ctg>Ttg):ENSP00000365678(p.Leu202(=))]	NA	NA	2.60E-03	1.84E-02	1.97E-02	2.60E-03	7.20E-03	NA	NA	NA	LOW	NA	0.216	80	80	0	59	27	31	52.54
Non-user	IOB_24	chr10	25312895	C	A	ENKUR	INTRONIC	219670:[NM_001270383.1(c.38-28097G>T)]	NA	NA	2.68E-02	4.12E-02	7.47E-02	2.68E-02	6.44E-02	NA	NA	NA	MODIFIER	NA	NA	315	315	0	269	144	124	46.1
Non-user	IOB_24	chr10	25312895	C	A	THNSL1	MISSENSE	ENSG00000185875:[ENST00000376356(c.743C>A,gCa>gAa):ENSP00000365534(p.Ala248Glu)]	NA	NA	2.68E-02	4.12E-02	7.47E-02	2.68E-02	6.44E-02	NA	NA	NA	MODIFIER	NA	NA	315	315	0	269	144	124	46.1
Non-user	IOB_24	chr10	27462061	G	A	MASTL	SILENT	ENSG00000120539:[ENST00000342386(c.2136G>A,caG>caA):ENSP00000343446(p.Gln712(=))]	NA	NA	9.98E-03	1.61E-02	3.64E-02	9.98E-03	2.76E-02	NA	NA	NA	MODIFIER	NA	NA	103	103	0	55	33	22	40
Non-user	IOB_24	chr10	27577669	A	T	ARMC4P1	EXONIC-NC	ENSG00000238021:[ENST00000438905(n.623A>T)]	NA	NA	1.22E-02	NA	NA	1.22E-02	3.48E-02	NA	NA	NA	MODIFIER	NA	NA	95	95	0	54	29	25	46.3
Non-user	IOB_24	chr10	27692307	A	G	PTCHD3	INTRONIC	374308:[XM_005252449.1(c.1090-21T>C)]	NA	NA	2.62E-02	4.73E-02	5.26E-02	2.62E-02	4.60E-02	NA	NA	NA	MODIFIER	NA	NA	124	124	0	90	56	34	37.78
Non-user	IOB_24	chr10	29783885	A	G	SVIL	SILENT	ENSG00000197321:[ENST00000538146(c.175T>C,Ttg>Ctg):ENSP00000440343(p.Leu59(=))]	NA	NA	NA	4.04E-01	3.57E-01	NA	NA	NA	NA	NA	LOW	NA	1	151	151	0	96	18	77	80.21
Non-user	IOB_24	chr10	30336682	T	C	KIAA1462	SILENT	ENSG00000165757:[ENST00000375377(c.60A>G,ccA>ccG):ENSP00000364526(p.Pro20(=))]	NA	chr10*30336682*30336682*COSM1474581*T*TG*1*breast*1	1.72E-02	1.44E-02	5.87E-02	1.72E-02	6.65E-02	NA	NA	NA	MODIFIER	NA	NA	315	315	0	247	122	125	50.61

Patel *et al.* , 2021. Whole-exome sequencing analysis of oral squamous cell carcinoma delineated by tobacco usage habits
Supplementary Table 4. List of somatic single nucleotide variants identified using whole exome sequencing of OSCC patients

Cohort	Sample	Chromosome	Genomic position	Reference allele	Alternate allele	Genesymbol	Variant type	Variant description	OncoMD	COSMIC	dbSNP	ExAC	ExAC_SAS	1000G	1000G_SAS	SIFT	Polyphen2	CONDEL	VEP	LRT	PhastCon7Way	Total reads in Normal	Reference read support in Normal	Alternative read support in Normal	Total reads in Tumor	Reference read support in Tumor	Alternate read support in Tumor	Alternate allele frequency in Tumor
Non-user	IOB_24	chr10	30653746	C	A	GOLGA2P6	EXONIC-NC	729668:[NR_120609.1(n.2480G>T)]	NA	NA	3.99E-03	4.39E-03	2.47E-02	3.99E-03	2.04E-02	NA	NA	NA	MODIFIER	NA	NA	34	34	0	17	10	7	41.18
Non-user	IOB_24	chr10	30653746	C	A	MTPAP	MISSENSE	ENSG00000107951:[ENST00000358107(c.436G>T,Gat>Tat):ENSP00000350820(p.Asp146Tyr)]	NA	NA	3.99E-03	4.39E-03	2.47E-02	3.99E-03	2.04E-02	NA	NA	NA	MODIFIER	NA	NA	34	34	0	17	10	7	41.18
Non-user	IOB_24	chr10	30915048	T	C	LYZL2	MISSENSE	ENSG00000151033:[ENST00000375318(c.422A>G,cAc>cGc):ENSP00000364467(p.His141Arg)]	NA	chr10*30915048*30915048*COSM684518*T*A*1*lung*1	2.80E-03	1.79E-03	1.04E-02	2.80E-03	1.43E-02	T	BN	NA	MODERATE	N	0.785	140	140	0	111	51	59	53.15
Non-user	IOB_24	chr10	33200923	G	A	ITGB1	SILENT	ENSG00000150093:[ENST00000302278(c.1599C>T,tgC>tgT):ENSP00000303351(p.Cys533(=))]	NA	NA	NA	1.15E-04	4.24E-04	NA	NA	NA	NA	NA	LOW	NA	NA	403	402	0	337	171	166	49.26
Non-user	IOB_24	chr10	33491722	C	T	NRP1	MISSENSE	ENSG00000099250:[ENST00000374823(c.1961G>A,gGc>gAc):ENSP00000363956(p.Gly654Asp)]	NA	NA	7.99E-04	3.16E-03	3.11E-03	7.99E-04	0.00E+00	NA	NA	NA	MODIFIER	NA	NA	259	258	1	205	107	97	47.32
Non-user	IOB_24	chr10	35897387	C	T	GJD4	NONSENSE	ENSG00000177291:[ENST00000321660(c.946C>T,Cga>Tga):ENSP00000315070(p.Arg316Ter)]	NA	NA	3.40E-03	9.98E-03	1.77E-02	3.39E-03	1.74E-02	NA	NA	NA	HIGH	NA	0	77	76	0	60	34	25	41.67
Non-user	IOB_24	chr10	38639017	G	A	NA	INTERGENIC	NA	NA	NA	NA	NA	NA	NA	NA	NA	NA	NA	MODIFIER	NA	NA	214	213	0	122	102	20	16.39
Non-user	IOB_24	chr10	43232492	C	T	RSU1P1	EXONIC-NC-SS-PRX	ENSG00000230425:[ENST00000453416(n.326C>T)]	NA	NA	3.14E-02	NA	NA	3.13E-02	7.98E-02	NA	NA	NA	LOW	NA	NA	241	240	0	166	103	63	37.95
Non-user	IOB_24	chr10	44053471	C	T	ZNF239	SILENT	ENSG00000196793:[ENST00000306006(c.57G>A,gtG>gtA):ENSP00000307774(p.Val19(=))]	NA	NA	3.79E-03	3.01E-03	2.16E-02	3.79E-03	1.94E-02	NA	NA	NA	LOW	NA	NA	226	226	0	169	91	77	45.56
Non-user	IOB_24	chr10	45430234	C	T	TMEM72	SILENT	ENSG00000187783:[ENST00000389583(c.480C>T,acC>acT):ENSP00000374234(p.Thr160(=))]	NA	NA	2.00E-04	1.28E-03	1.22E-04	2.00E-04	1.00E-03	NA	NA	NA	LOW	NA	NA	207	207	0	131	72	59	45.04
Non-user	IOB_24	chr10	45430234	C	T	TMEM72-AS1	NCT	220980:[XR_246521.1(n.712+2506G>A)]	NA	NA	2.00E-04	1.28E-03	1.22E-04	2.00E-04	1.00E-03	NA	NA	NA	LOW	NA	NA	207	207	0	131	72	59	45.04
Non-user	IOB_24	chr10	46952758	G	T	RP11-38L15.3	NCT	ENSG00000231187:[ENST00000506914(n.312+975G>T)]	NA	NA	NA	7.78E-02	2.04E-02	NA	NA	NA	NA	NA	MODIFIER	NA	NA	95	83	12	21	0	21	100
Non-user	IOB_24	chr10	46952802	T	C	RP11-38L15.3	NCT	ENSG00000231187:[ENST00000430188(n.232-485T>C)]	NA	NA	NA	3.56E-02	1.23E-02	NA	NA	NA	NA	NA	MODIFIER	NA	NA	130	115	14	25	0	25	100
Non-user	IOB_24	chr10	47086915	C	T	LINC00842	NCT	ENSG00000223477:[ENST00000503031(n.254-13936G>A)]	NA	NA	NA	1.72E-01	1.49E-01	NA	NA	NA	NA	NA	LOW	NA	NA	414	412	0	321	235	84	26.17
Non-user	IOB_24	chr10	47086915	C	T	NPY4R	SILENT	ENSG00000204174:[ENST00000374312(c.132C>T,atC>atT):ENSP00000363431(p.Ile44(=))]	NA	NA	NA	1.72E-01	1.49E-01	NA	NA	NA	NA	NA	LOW	NA	NA	414	412	0	321	235	84	26.17
Non-user	IOB_24	chr10	47087501	C	T	LINC00842	NCT	ENSG00000223477:[ENST00000503031(n.254-14522G>A)]	NA	NA	NA	3.07E-01	3.54E-01	NA	NA	NA	NA	NA	MODIFIER	NA	NA	611	609	0	496	393	101	20.36
Non-user	IOB_24	chr10	47087501	C	T	NPY4R	MISSENSE	ENSG00000204174:[ENST00000374312(c.718C>T,Cgc>Tgc):ENSP00000363431(p.Arg240Cys)]	NA	NA	NA	3.07E-01	3.54E-01	NA	NA	NA	NA	NA	MODIFIER	NA	NA	611	609	0	496	393	101	20.36
Non-user	IOB_24	chr10	47416860	G	C	FAM35DP	INTRONIC-SS-ACR	ENSG00000189014:[ENST00000540459(n.653-1G>C)]	NA	NA	chr10*47416860*47416860*G*A,C*rs28377449*NA*NA*NA	5.48E-02	5.45E-02	NA	NA	NA	NA	NA	HIGH	NA	NA	882	875	3	609	369	236	38.75
Non-user	IOB_24	chr10	49951198	G	A	WDFY4	SILENT	ENSG00000128815:[ENST00000325239(c.2064G>A,gtG>gtA):ENSP00000320563(p.Val688(=))]	NA	NA	4.99E-03	8.59E-03	1.42E-02	4.99E-03	1.53E-02	NA	NA	NA	LOW	NA	NA	328	326	0	233	123	109	46.78
Non-user	IOB_24	chr10	50185055	G	A	RP11-523O18.5	EXONIC-NC	ENSG00000233665:[ENST00000428825(n.1154C>T)]	NA	NA	2.76E-02	2.32E-02	1.22E-01	2.76E-02	8.59E-02	NA	NA	NA	MODIFIER	NA	NA	112	111	0	99	46	53	53.54
Non-user	IOB_24	chr10	50185055	G	A	WDFY4	INTRONIC	57705:[XM_005270007.1(c.4584+31G>A)]	NA	NA	2.76E-02	2.32E-02	1.22E-01	2.76E-02	8.59E-02	NA	NA	NA	MODIFIER	NA	NA	112	111	0	99	46	53	53.54
Non-user	IOB_24	chr10	50256548	G	A	VSTM4	SILENT	ENSG00000165633:[ENST00000332853(c.750C>T,gaC>gaT):ENSP00000331062(p.Asp250(=))]	NA	NA	3.34E-02	2.24E-02	1.34E-01	3.33E-02	1.47E-01	NA	NA	NA	LOW	NA	0	42	42	0	28	17	11	39.29
Non-user	IOB_24	chr10	50311775	T	A	VSTM4	INTRONIC	ENSG00000165633:[ENST00000332853(c.457+3864A>T)]	NA	NA	3.99E-04	3.99E-04	2.09E-03	3.99E-04	0.00E+00	NA	NA	NA	MODIFIER	NA	NA	89	89	0	37	20	17	45.95
Non-user	IOB_24	chr10	51623096	G	C	TIMM23	INTRONIC	ENSG00000138297:[ENST00000260867(c.106+13C>G)]	NA	NA	NA	3.22E-03	2.15E-02	NA	NA	NA	NA	NA	MODIFIER	NA	NA	177	176	0	149	120	26	17.45
Non-user	IOB_24	chr10	51623096	G	C	TIMM23B	NCT	100652748:[NR_110767.1(n.801-109676G>C)]	NA	NA	NA	3.22E-03	2.15E-02	NA	NA	NA	NA	NA	MODIFIER	NA	NA	177	176	0	149	120	26	17.45
Non-user	IOB_24	chr10	55568971	C	G	PCDH15	MISSENSE	ENSG00000150275:[ENST00000395440(c.1641G>C,gaG>gaC):ENSP00000378827(p.Glu547Asp)]	NA	NA	3.00E-03	2.11E-03	1.48E-02	3.00E-03	1.53E-02	D_lc	BN	NA	MODERATE	NA	NA	167	166	0	96	54	40	41.67
Non-user	IOB_24	chr10	60147934	T	C	TFAM	INTRONIC	ENSG00000108064:[ENST00000395377(c.165-16T>C)]	NA	NA	3.16E-02	3.77E-02	7.36E-02	3.15E-02	6.54E-02	NA	NA	NA	MODIFIER	NA	NA	74	74	0	46	25	21	45.65
Non-user	IOB_24	chr10	61828954	G	A	ANK3	SILENT	ENSG00000151150:[ENST00000280772(c.11685C>T,tcC>tcT):ENSP00000280772(p.Ser3895(=))]	NA	NA	9.78E-03	2.27E-02	7.15E-03	9.78E-03	6.10E-03	NA	NA	NA	MODIFIER	NA	NA	483	482	0	354	189	164	46.33
Non-user	IOB_24	chr10	61831290	T	C	ANK3	MISSENSE	ENSG00000151150:[ENST00000280772(c.9349A>G,Atc>Gtc):ENSP00000280772(p.Ile3117Val)]	NA	chr10*61831289*61831290*COSM1348429*AT*A*2*large_intestine*2	3.16E-02	7.44E-02	2.07E-02	3.15E-02	1.23E-02	NA	NA	NA	MODERATE	NA	1	249	248	0	147	80	67	45.58
Non-user	IOB_24	chr10	61831984	G	T	ANK3	MISSENSE	ENSG00000151150:[ENST00000280772(c.8655C>A,caC>caA):ENSP00000280772(p.His2885Gln)]	NA	NA	3.14E-02	7.44E-02	2.06E-02	3.13E-02	1.12E-02	NA	NA	NA	MODIFIER	NA	NA	291	287	1	221	112	106	47.96
Non-user	IOB_24	chr10	61834573	G	A	ANK3	SILENT	ENSG00000151150:[ENST00000280772(c.6066C>T,gcC>gcT):ENSP00000280772(p.Ala2022(=))]	NA	NA	3.16E-02	7.42E-02	2.07E-02	3.15E-02	1.23E-02	NA	NA	NA	LOW	NA	NA	209	209	0	186	100	86	46.24
Non-user	IOB_24	chr10	65358903	A	T	REEP3	INTRONIC	ENSG00000165476:[ENST00000373758(c.304-27A>T)]	NA	NA	chr10*65358903*65358903*A*G,T*rs777758272*NA*NA*NA	3.78E-05	2.67E-04	NA	NA	NA	NA	NA	MODIFIER	NA	NA	245	244	0	167	81	86	51.5
Non-user	IOB_24	chr10	65380751	T	G	REEP3	3UTR	ENSG00000165476:[ENST00000298249(c.*122T>G)]	NA	NA	1.94E-02	NA	NA	1.94E-02	2.04E-02	NA	NA	NA	MODIFIER	NA	NA	31	31	0	34	13	21	61.76
Non-user	IOB_24	chr10	68687179	C	T	CTNNA3	INTRONIC	ENSG00000183230:[ENST00000373744(c.1048-151897G>A)]	NA	NA	3.69E-02	1.47E-02	9.17E-03	3.69E-02	1.23E-02	NA	NA	NA	MODIFIER	NA	NA	192	191	0	182	92	88	48.35
Non-user	IOB_24	chr10	68687179	C	T	LOC101928961	NCT	101928961:[NR_111911.1(n.2718-16928G>A)]	NA	NA	3.69E-02	1.47E-02	9.17E-03	3.69E-02	1.23E-02	NA	NA	NA	MODIFIER	NA	NA	192	191	0	182	92	88	48.35
Non-user	IOB_24	chr10	68687179	C	T	LRRTM3	SILENT	ENSG00000198739:[ENST00000361320(c.505C>T,Ctg>Ttg):ENSP00000355187(p.Leu169(=))]	NA	NA	3.69E-02	1.47E-02	9.17E-03	3.69E-02	1.23E-02	NA	NA	NA	MODIFIER	NA	NA	192	191	0	182	92	88	48.35
Non-user	IOB_24	chr10	69793819	T	G	HERC4	SILENT	ENSG00000148634:[ENST00000427635(c.577A>C,gcA>gcC):ENSP00000406171(p.Ala193(=))]	NA	NA	3.20E-03	4.03E-03	1.90E-02	3.19E-03	1.23E-02	NA	NA	NA	LOW	NA	NA	220	218	1	181	92	89	49.17
Non-user	IOB_24	chr10	70946243	A	T	SUPV3L1	MISSENSE	ENSG00000156502:[ENST00000359655(c.394A>T,Atg>Ttg):ENSP00000352678(p.Met132Leu)]	NA	NA	NA	8.24E-06	6.06E-05	NA	NA	NA	NA	NA	MODIFIER	NA	NA	214	213	0	130	66	64	49.23
Non-user	IOB_24	chr10	71685532	G	C	COL13A1	INTRONIC	ENSG00000197467:[ENST00000398968(c.1240-28G>C)]	NA	NA	chr10*71685532*71685532*G*A,C*rs1227778*NA*NA*NA	6.40E-01	5.76E-01	NA	NA	NA	NA	NA	MODIFIER	NA	NA	68	68	0	39	18	21	53.85
Non-user	IOB_24	chr10	71689738	C	T	COL13A1	INTRONIC	ENSG00000197467:[ENST00000479733(c.1480-61C>T)]	NA	NA	9.78E-03	NA	NA	9.78E-03	4.19E-02	NA	NA	NA	MODIFIER	NA	NA	115	115	0	81	43	38	46.91
Non-user	IOB_24	chr10	71696385	A	G	COL13A1	INTRONIC	ENSG00000197467:[ENST00000517713(c.1627-127A>G)]	NA	NA	9.98E-03	NA	NA	9.98E-03	4.09E-02	NA	NA	NA	MODIFIER	NA	NA	51	51	0	37	11	26	70.27
Non-user	IOB_24	chr10	71697388	C	T	COL13A1	INTRONIC-SS-ACR-PRX	ENSG00000197467:[ENST00000479733(c.*140-3C>T)]	NA	NA	9.98E-03	6.27E-03	3.48E-02	9.98E-03	4.09E-02	NA	NA	NA	LOW	NA	NA	46	46	0	52	24	27	51.92
Non-user	IOB_24	chr10	71917472	A	G	SAR1A	INTRONIC	ENSG00000079332:[ENST00000452767(c.60+48T>C)]	NA	NA	4.39E-03	5.21E-03	2.74E-02	4.39E-03	2.04E-02	NA	NA	NA	MODIFIER	NA	NA	14	14	0	19	14	5	26.32
Non-user	IOB_24	chr10	72503810	C	A	ADAMTS14	INTRONIC	ENSG00000138316:[ENST00000373208(c.2068-12C>A)]	NA	NA	4.03E-02	1.01E-01	4.68E-02	4.03E-02	3.78E-02	NA	NA	NA	MODIFIER	NA	NA	113	112	0	126	59	65	51.59
Non-user	IOB_24	chr10	73269891	G	A	CDH23	SILENT	ENSG00000107736:[ENST00000299366(c.333G>A,gtG>gtA):ENSP00000299366(p.Val111(=))]	NA	NA	3.20E-03	4.90E-03	2.51E-02	3.19E-03	1.43E-02	NA	NA	NA	LOW	NA	NA	314	314	0	234	123	110	47.01
Non-user	IOB_24	chr10	73269891	G	A	CDH23-AS1	NCT	102723377:[NR_120672.1(n.144-124C>T)]	NA	NA	3.20E-03	4.90E-03	2.51E-02	3.19E-03	1.43E-02	NA	NA	NA	LOW	NA	NA	314	314	0	234	123	110	47.01
Non-user	IOB_24	chr10	73442207	A	G	CDH23	MISSENSE	ENSG00000107736:[ENST00000442677(c.639A>G,Agc>Ggc):ENSP00000388894(p.Ser214Gly)]	NA	NA	NA	NA	NA	NA	NA	D	BN	NA	MODERATE	NA	NA	118	118	0	122	73	48	39.34
Non-user	IOB_24	chr10	73537449	G	A	CDH23	MISSENSE	ENSG00000107736:[ENST00000224721(c.4873G>A,Gtg>Atg):ENSP00000224721(p.Val1625Met)]	NA	NA	1.24E-02	2.27E-02	5.98E-02	1.24E-02	4.50E-02	NA	NA	NA	MODERATE	NA	1	68	68	0	49	22	27	55.1
Non-user	IOB_24	chr10	73581601	C	A	PSAP	INTRONIC	5660:[NM_001042465.2(c.918+32G>T)]	NA	NA	4.43E-02	7.11E-02	9.18E-02	4.43E-02	7.57E-02	NA	NA	NA	MODIFIER	NA	NA	160	160	0	123	63	60	48.78
Non-user	IOB_24	chr10	74235586	A	T	MICU1	INTRONIC	10367:[XM_005269383.1(c.754-531T>A)]	NA	NA	7.99E-03	1.22E-02	1.17E-02	7.99E-03	1.02E-02	NA	NA	NA	MODIFIER	NA	NA	238	237	1	194	110	84	43.3
Non-user	IOB_24	chr10	74235587	G	C	MICU1	INTRONIC	10367:[NM_006077.3(c.742-532C>G)]	NA	NA	7.99E-03	1.22E-02	1.17E-02	7.99E-03	1.02E-02	NA	NA	NA	MODIFIER	NA	NA	239	238	0	195	111	83	42.56
Non-user	IOB_24	chr10	75609141	A	G	CAMK2G	INTRONIC	818:[XM_005270196.1(c.318-50T>C)]	NA	NA	2.00E-04	6.88E-05	3.84E-04	2.00E-04	1.00E-03	NA	NA	NA	MODIFIER	NA	NA	192	191	1	122	55	67	54.92
Non-user	IOB_24	chr10	75671875	G	A	C10orf55	INTRONIC	ENSG00000222047:[ENST00000409178(c.46-22C>T)]	NA	NA	3.30E-02	6.37E-02	9.01E-02	3.29E-02	5.32E-02	NA	NA	NA	MODIFIER	NA	NA	203	202	0	156	78	76	48.72
Non-user	IOB_24	chr10	75671875	G	A	PLAU	INTRONIC	ENSG00000122861:[ENST00000372762(c.85+49G>A)]	NA	NA	3.30E-02	6.37E-02	9.01E-02	3.29E-02	5.32E-02	NA	NA	NA	MODIFIER	NA	NA	203	202	0	156	78	76	48.72
Non-user	IOB_24	chr10	78755456	A	G	KCNMA1	SILENT	ENSG00000156113:[ENST00000450795(c.580T>C,tcT>tcC):ENSP00000388370(p.Ser194(=))]	NA	NA	NA	1.22E-04	2.61E-04	NA	NA	NA	NA	NA	MODIFIER	NA	NA	40	40	0	15	8	7	46.67
Non-user	IOB_24	chr10	79581510	C	T	DLG5	MISSENSE	ENSG00000151208:[ENST00000372391(c.2732G>A,cGt>cAt):ENSP00000361467(p.Arg911His)]	NA	NA	5.99E-04	5.07E-04	1.23E-03	5.99E-04	2.00E-03	T	BN	NA	MODERATE	N	0.16	247	246	0	197	105	92	46.7
Non-user	IOB_24	chr10	79588605	C	T	DLG5	INTRONIC	9231:[XM_005270276.1(c.2277+35G>A)]	NA	NA	2.00E-04	7.68E-05	3.77E-04	2.00E-04	0.00E+00	NA	NA	NA	MODIFIER	NA	NA	161	160	0	108	55	53	49.07
Non-user	IOB_24	chr10	81050665	C	G	ZMIZ1	NCT	ENSG00000108175:[ENST00000472035(n.331-51C>G)]	NA	NA	1.60E-03	2.35E-03	1.99E-03	1.60E-03	2.00E-03	NA	NA	NA	MODIFIER	NA	NA	264	264	0	243	111	129	53.09
Non-user	IOB_24	chr10	81060752	G	A	ZMIZ1	INTRONIC	57178:[XM_005269991.1(c.2037+53G>A)]	NA	NA	4.27E-02	NA	NA	4.27E-02	9.20E-03	NA	NA	NA	MODIFIER	NA	NA	74	74	0	39	20	19	48.72
Non-user	IOB_24	chr10	81109535	G	C	PPIF	INTRONIC	ENSG00000108179:[ENST00000225174(c.315+26G>C)]	NA	NA	9.39E-03	2.07E-02	4.11E-03	9.38E-03	4.10E-03	NA	NA	NA	MODIFIER	NA	NA	41	41	0	38	17	20	52.63
Non-user	IOB_24	chr10	81373542	C	T	SFTPA1	SILENT	ENSG00000122852:[ENST00000372308(c.420C>T,tcC>tcT):ENSP00000361382(p.Ser140(=))]	NA	NA	NA	2.09E-01	2.37E-01	NA	NA	NA	NA	NA	LOW	NA	NA	121	120	0	121	84	37	30.58
Non-user	IOB_24	chr10	81926802	C	T	ANXA11	INTRONIC	311:[XM_005269741.1(c.950-62G>A)]	NA	NA	1.20E-03	NA	NA	1.20E-03	0.00E+00	NA	NA	NA	MODIFIER	NA	NA	100	100	0	57	18	39	68.42
Non-user	IOB_24	chr10	81995007	A	G	RP11-40F6.1	NCT	ENSG00000234382:[ENST00000423182(n.121-28A>G)]	NA	NA	NA	NA	NA	NA	NA	NA	NA	NA	MODIFIER	NA	NA	83	83	0	122	85	37	30.33
Non-user	IOB_24	chr10	82013047	T	C	AL359195.1	MISSENSE	ENSG00000204038:[ENST00000356374(c.565T>C,Tgg>Cgg):ENSP00000348738(p.Trp189Arg)]	NA	NA	1.24E-02	2.49E-02	1.18E-02	1.24E-02	9.20E-03	D_lc	PoD	NA	MODERATE	NA	NA	205	205	0	168	75	91	54.17
Non-user	IOB_24	chr10	82013047	T	C	LOC100130698	EXONIC-NC	100130698:[XR_110427.2(n.3576T>C)]	NA	NA	1.24E-02	2.49E-02	1.18E-02	1.24E-02	9.20E-03	D_lc	PoD	NA	MODERATE	NA	NA	205	205	0	168	75	91	54.17
Non-user	IOB_24	chr10	88447027	T	C	LDB3	SILENT-SS-PRX	ENSG00000122367:[ENST00000372056(c.891T>C,agT>agC):ENSP00000361126(p.Ser297(=))]	NA	NA	3.40E-03	5.49E-03	1.24E-02	3.39E-03	9.20E-03	NA	NA	NA	MODIFIER	NA	NA	152	152	0	165	78	86	52.12
Non-user	IOB_24	chr10	91099831	C	T	IFIT3	SILENT	ENSG00000119917:[ENST00000371811(c.1419C>T,ggC>ggT):ENSP00000360876(p.Gly473(=))]	NA	NA	NA	3.37E-05	0.00E+00	NA	NA	NA	NA	NA	LOW	NA	NA	104	104	0	90	44	45	50
Non-user	IOB_24	chr10	91099831	C	T	LIPA	NCT	ENSG00000107798:[ENST00000463623(n.50+2487G>A)]	NA	NA	NA	3.37E-05	0.00E+00	NA	NA	NA	NA	NA	LOW	NA	NA	104	104	0	90	44	45	50
Non-user	IOB_24	chr10	92631778	G	A	RPP30	MISSENSE	ENSG00000148688:[ENST00000277882(c.35G>A,gGt>gAt):ENSP00000277882(p.Gly12Asp)]	NA	NA	1.12E-02	1.95E-02	1.64E-02	1.12E-02	1.12E-02	T	BN	NA	MODERATE	N	0.004	337	336	0	291	168	123	42.27
Non-user	IOB_24	chr10	93390149	C	T	PPP1R3C	SILENT	ENSG00000119938:[ENST00000238994(c.489G>A,tcG>tcA):ENSP00000238994(p.Ser163(=))]	NA	NA	1.60E-02	3.59E-02	1.60E-02	1.60E-02	6.10E-03	NA	NA	NA	LOW	NA	NA	283	281	0	257	116	139	54.09
Non-user	IOB_24	chr10	93392776	A	G	PPP1R3C	5UTR	ENSG00000119938:[ENST00000238994(c.-50T>C)]	NA	NA	1.88E-02	2.94E-02	2.92E-02	1.88E-02	2.76E-02	NA	NA	NA	MODIFIER	NA	NA	70	70	0	63	31	31	49.21
Non-user	IOB_24	chr10	93776169	C	T	BTAF1	MISSENSE	ENSG00000095564:[ENST00000544642(c.1141C>T,Ctt>Ttt):ENSP00000439924(p.Leu381Phe)]	NA	NA	NA	3.30E-05	2.42E-04	NA	NA	T	BN	NA	MODERATE	N	1	127	127	0	93	48	44	47.31
Non-user	IOB_24	chr10	95433538	T	C	FRA10AC1	SILENT	ENSG00000148690:[ENST00000394100(c.855A>G,gaA>gaG):ENSP00000377660(p.Glu285(=))]	NA	NA	1.10E-02	2.21E-02	1.26E-02	1.10E-02	8.20E-03	NA	NA	NA	LOW	NA	NA	90	89	0	57	31	26	45.61
Non-user	IOB_24	chr10	95849097	G	A	PLCE1	SILENT	ENSG00000138193:[ENST00000371375(c.246G>A,aaG>aaA):ENSP00000360426(p.Lys82(=))]	NA	NA	7.39E-03	7.02E-03	6.88E-03	7.39E-03	7.20E-03	NA	NA	NA	MODIFIER	NA	NA	172	172	0	111	60	51	45.95
Non-user	IOB_24	chr10	95849097	G	A	PLCE1-AS2	NCT	101927049:[NR_120616.1(n.637-1139C>T)]	NA	NA	7.39E-03	7.02E-03	6.88E-03	7.39E-03	7.20E-03	NA	NA	NA	MODIFIER	NA	NA	172	172	0	111	60	51	45.95
Non-user	IOB_24	chr10	95849097	G	A	RP11-429H9.4	NCT	ENSG00000232913:[ENST00000438899(n.623-1139C>T)]	NA	NA	7.39E-03	7.02E-03	6.88E-03	7.39E-03	7.20E-03	NA	NA	NA	MODIFIER	NA	NA	172	172	0	111	60	51	45.95
Non-user	IOB_24	chr10	97386497	G	T	ALDH18A1	MISSENSE	ENSG00000059573:[ENST00000371224(c.1115C>A,tCt>tAt):ENSP00000360268(p.Ser372Tyr)]	NA	NA	2.04E-02	1.72E-02	1.56E-02	2.04E-02	2.35E-02	NA	NA	NA	MODERATE	NA	0.98	80	80	0	57	32	25	43.86
Non-user	IOB_24	chr10	97445357	C	A	TCTN3	MISSENSE	ENSG00000119977:[ENST00000265993(c.979G>T,Gct>Tct):ENSP00000265993(p.Ala327Ser)]	NA	chr10*97445357*97445357*COSM1585175*C*T*1*endometrium*1|chr10*97445357*97445357*COSM921835*C*T*1*endometrium*1	2.04E-02	1.78E-02	1.64E-02	2.04E-02	2.35E-02	NA	NA	NA	MODIFIER	NA	NA	109	109	0	83	45	37	44.58
Non-user	IOB_24	chr10	97784683	C	T	CC2D2B	INTRONIC	ENSG00000188649:[ENST00000371198(c.394-2286C>T)]	NA	NA	4.63E-02	3.51E-02	3.27E-02	4.63E-02	4.29E-02	NA	NA	NA	MODIFIER	NA	NA	63	63	0	47	32	15	31.91
Non-user	IOB_24	chr10	97784683	C	T	ENTPD1-AS1	NCT	ENSG00000226688:[ENST00000451364(n.216+64340G>A)]	NA	NA	4.63E-02	3.51E-02	3.27E-02	4.63E-02	4.29E-02	NA	NA	NA	MODIFIER	NA	NA	63	63	0	47	32	15	31.91
Non-user	IOB_24	chr10	97784683	C	T	RP11-690P14.4	INTRONIC	ENSG00000269920:[ENST00000475252(c.*915-2286C>T)]	NA	NA	4.63E-02	3.51E-02	3.27E-02	4.63E-02	4.29E-02	NA	NA	NA	MODIFIER	NA	NA	63	63	0	47	32	15	31.91
Non-user	IOB_24	chr10	98007802	A	C	BLNK	INTRONIC	ENSG00000095585:[ENST00000224337(c.48-997T>G)]	NA	NA	NA	NA	NA	NA	NA	NA	NA	NA	MODIFIER	NA	NA	74	73	1	60	24	35	58.33
Non-user	IOB_24	chr10	98115056	T	C	OPALIN	INTRONIC-SS-ACR-PRX	ENSG00000197430:[ENST00000419479(c.-66-9A>G)]	NA	NA	1.02E-02	1.42E-02	3.23E-02	1.02E-02	2.35E-02	NA	NA	NA	MODIFIER	NA	NA	32	32	0	26	14	11	42.31
Non-user	IOB_24	chr10	98803252	G	A	ARHGAP19-SLIT1	3UTR	ENSG00000269891:[ENST00000453547(c.*1595C>T)]	NA	NA	4.67E-02	1.01E-01	4.78E-02	4.67E-02	5.01E-02	NA	NA	NA	MODIFIER	NA	NA	89	89	0	49	29	19	38.78

Supplementary Table 4. List of somatic single nucleotide variants identified using whole exome sequencing of OSCC patients

Cohort	Sample	Chromosome	Genomic position	Reference allele	Alternate allele	Genesymbol	Variant type	Variant description	OncoMD	COSMIC	dbSNP	ExAC	ExAC_SAS	1000G	1000G_SAS	SIFT	Polyphen2	CONDEL	VEP	LRT	PhastCon7Way	Total reads in Normal	Reference read support in Normal	Alternative read support in Normal	Total reads in Tumor	Reference read support in Tumor	Alternate read support in Tumor	Alternate allele frequency in Tumor
Non-user	IOB_24	chr10	98803252	G	A	SLIT1	SILENT	ENSG00000187122:[ENST00000266058(c.1872C>T,cgC>cgT):ENSP00000266058(p.Arg624(=))]	NA	NA	4.67E-02	1.01E-01	4.78E-02	4.67E-02	5.01E-02	NA	NA	NA	MODIFIER	NA	NA	89	89	0	49	29	19	38.78
Non-user	IOB_24	chr10	99422724	G	A	PI4K2A	MISSENSE-SS-PRX	ENSG00000249967:[ENST00000370649(c.893G>A,cGg>cAg):ENSP00000359683(p.Arg298Gln)]	NA	chr10*99422724*99422724*COSM1262143*G*A*1*oesophagus*1|chr10*99422724*99422724*COSM1262142*G*A*1*oesophagus*1	5.39E-03	6.80E-03	1.71E-02	5.39E-03	2.15E-02	T	BN	NA	MODERATE	NA	1	277	272	0	229	129	99	43.23
Non-user	IOB_24	chr10	99439556	A	C	AVPI1	MISSENSE	ENSG00000119986:[ENST00000370626(c.107T>G,cTg>cGg):ENSP00000359660(p.Leu36Arg)]	NA	chr10*99439556*99439556*COSM1162384*A*C*1*pancreas*1	4.77E-02	6.78E-02	8.04E-02	4.77E-02	8.38E-02	D	PrD	NA	MODERATE	NA	NA	215	215	0	195	95	99	50.77
Non-user	IOB_24	chr10	100202987	A	G	HPS1	MISSENSE	ENSG00000107521:[ENST00000325103(c.11T>C,gTc>gCc):ENSP00000326649(p.Val4Ala)]	NA	NA	4.07E-02	3.33E-02	5.05E-02	4.07E-02	6.44E-02	NA	NA	NA	MODIFIER	NA	NA	197	197	0	128	67	60	46.88
Non-user	IOB_24	chr10	101716112	G	C	DNMBP	MISSENSE	ENSG00000107554:[ENST00000324109(c.1119C>G,aaC>aaG):ENSP00000315659(p.Asn373Lys)]	NA	NA	4.95E-02	4.52E-02	2.88E-02	4.95E-02	1.84E-02	NA	NA	NA	MODIFIER	NA	NA	120	120	0	88	56	32	36.36
Non-user	IOB_24	chr10	101716112	G	C	DNMBP-AS1	NCT	ENSG00000227695:[ENST00000434409(n.332-127G>C)]	NA	NA	4.95E-02	4.52E-02	2.88E-02	4.95E-02	1.84E-02	NA	NA	NA	MODIFIER	NA	NA	120	120	0	88	56	32	36.36
Non-user	IOB_24	chr10	102295658	T	G	HIF1AN	5UTR	ENSG00000166135:[ENST00000299163(c.-58T>G)]	NA	NA	NA	1.94E-01	2.01E-01	NA	NA	NA	NA	NA	MODIFIER	NA	NA	188	179	0	124	71	52	41.94
Non-user	IOB_24	chr10	102826162	T	C	KAZALD1	EXONIC-NC	ENSG00000107821:[ENST00000608812(n.11T>C)]	NA	NA	NA	NA	NA	NA	NA	NA	NA	NA	MODIFIER	NA	NA	220	220	0	180	80	100	55.56
Non-user	IOB_24	chr10	104135277	C	T	GBF1	SILENT	ENSG00000107862:[ENST00000369983(c.3819C>T,atC>atT):ENSP00000359000(p.Ile1273(=))]	NA	NA	7.99E-04	4.21E-04	3.09E-03	7.99E-04	4.10E-03	NA	NA	NA	LOW	NA	0.814	203	203	0	179	96	83	46.37
Non-user	IOB_24	chr10	104160959	C	T	NFKB2	SILENT	ENSG00000077150:[ENST00000189444(c.2094C>T,aaC>aaT):ENSP00000189444(p.Asn698(=))]	NA	NA	3.18E-02	4.19E-02	4.56E-02	3.17E-02	3.27E-02	NA	NA	NA	LOW	NA	0	258	256	1	226	129	96	42.48
Non-user	IOB_24	chr10	104851400	G	A	NT5C2	INTRONIC	22978:[XM_005269646.1(c.587-28C>T)]	NA	NA	4.37E-02	3.17E-02	1.18E-01	4.37E-02	1.47E-01	NA	NA	NA	MODIFIER	NA	NA	30	29	0	29	13	16	55.17
Non-user	IOB_24	chr10	105037385	C	T	INA	SILENT	ENSG00000148798:[ENST00000369849(c.417C>T,gtC>gtT):ENSP00000358865(p.Val139(=))]	NA	NA	2.88E-02	4.40E-02	1.14E-01	2.88E-02	9.51E-02	NA	NA	NA	LOW	NA	NA	262	262	0	205	102	103	50.24
Non-user	IOB_24	chr10	105037853	C	T	INA	SILENT	ENSG00000148798:[ENST00000369849(c.885C>T,aaC>aaT):ENSP00000358865(p.Asn295(=))]	NA	NA	3.00E-02	1.95E-02	7.32E-02	3.00E-02	9.41E-02	NA	NA	NA	LOW	NA	0.226	252	252	0	224	148	74	33.04
Non-user	IOB_24	chr10	105765504	G	C	SLK	INTRONIC	ENSG00000065613:[ENST00000335753(c.2480+55G>C)]	NA	NA	2.60E-03	NA	NA	2.60E-03	1.33E-02	NA	NA	NA	MODIFIER	NA	NA	41	41	0	30	11	19	63.33
Non-user	IOB_24	chr10	106027059	G	A	GSTO1	MISSENSE	ENSG00000148834:[ENST00000369713(c.622G>A,Gaa>Aaa):ENSP00000358727(p.Glu208Lys)]	NA	NA	4.15E-02	3.78E-02	1.13E-01	4.15E-02	1.22E-01	T	BN	NA	MODERATE	N	0	89	89	0	99	41	58	58.59
Non-user	IOB_24	chr10	108371697	C	T	SORCS1	MISSENSE	ENSG00000108018:[ENST00000263054(c.3005G>A,gGt>gAt):ENSP00000263054(p.Gly1002Asp)]	NA	NA	NA	1.98E-04	1.45E-03	NA	NA	D	PoD	NA	MODERATE	NA	NA	68	68	0	45	27	18	40
Non-user	IOB_24	chr10	108437088	G	A	SORCS1	SILENT	ENSG00000108018:[ENST00000369698(c.420C>T,ctC>ctT):ENSP00000358712(p.Leu140(=))]	NA	NA	NA	7.43E-05	4.85E-04	NA	NA	NA	NA	NA	LOW	NA	NA	209	209	0	164	87	77	46.95
Non-user	IOB_24	chr10	108437181	T	C	SORCS1	INTRONIC	ENSG00000108018:[ENST00000263054(c.1741-19A>G)]	NA	NA	3.02E-02	2.02E-02	5.97E-02	3.02E-02	5.52E-02	NA	NA	NA	MODIFIER	NA	NA	259	258	0	216	101	113	52.31
Non-user	IOB_24	chr10	112058426	T	C	SMNDC1	SILENT	ENSG00000119953:[ENST00000369592(c.243A>G,gcA>gcG):ENSP00000358605(p.Ala81(=))]	NA	NA	4.17E-02	6.30E-02	1.08E-01	4.17E-02	1.05E-01	NA	NA	NA	MODIFIER	NA	NA	128	128	0	147	75	71	48.3
Non-user	IOB_24	chr10	112063216	C	G	SMNDC1	NCT	ENSG00000119953:[ENST00000471297(n.311+10G>C)]	NA	NA	4.17E-02	6.33E-02	1.11E-01	4.17E-02	1.05E-01	NA	NA	NA	MODIFIER	NA	NA	31	31	0	40	24	16	40
Non-user	IOB_24	chr10	112579949	A	G	RBM20	INTRONIC	ENSG00000203867:[ENST00000369519(c.2655+15A>G)]	NA	NA	1.60E-03	3.03E-03	2.19E-03	1.60E-03	2.00E-03	NA	NA	NA	MODIFIER	NA	NA	40	40	0	42	22	19	45.24
Non-user	IOB_24	chr10	114059303	C	T	TECTB	SILENT	ENSG00000119913:[ENST00000369422(c.888C>T,gtC>gtT):ENSP00000358430(p.Val296(=))]	NA	NA	1.92E-02	2.25E-02	3.73E-02	1.92E-02	5.83E-02	NA	NA	NA	LOW	NA	NA	206	206	0	206	110	96	46.6
Non-user	IOB_24	chr10	115962812	G	A	TDRD1	INTRONIC	ENSG00000095627:[ENST00000251864(c.697-19G>A)]	NA	NA	2.66E-02	4.62E-02	7.98E-02	2.66E-02	7.16E-02	NA	NA	NA	MODIFIER	NA	NA	103	103	0	95	44	50	52.63
Non-user	IOB_24	chr10	116199017	A	G	ABLIM1	INTRONIC	3983:[NM_001322900.1(c.796-8T>C)]	NA	NA	NA	7.44E-05	1.22E-04	NA	NA	NA	NA	NA	LOW	NA	NA	164	164	0	142	70	72	50.7
Non-user	IOB_24	chr10	118443263	G	T	HSPA12A	MISSENSE	ENSG00000165868:[ENST00000480802(c.328C>A,Ctt>Att):ENSP00000466041(p.Leu110Ile)]	NA	NA	3.06E-02	2.04E-02	5.42E-02	3.06E-02	5.11E-02	NA	NA	NA	MODIFIER	NA	NA	71	71	0	74	48	25	33.78
Non-user	IOB_24	chr10	118897406	C	G	VAX1	SILENT	ENSG00000148704:[ENST00000277905(c.162G>C,tcG>tcC):ENSP00000277905(p.Ser54(=))]	NA	NA	1.80E-03	1.54E-03	1.07E-02	1.80E-03	9.20E-03	NA	NA	NA	LOW	NA	NA	89	89	0	59	27	32	54.24
Non-user	IOB_24	chr10	120907358	C	T	SFXN4	SILENT	ENSG00000183605:[ENST00000355697(c.747G>A,acG>acA):ENSP00000347924(p.Thr249(=))]	NA	NA	5.39E-03	4.18E-03	2.21E-02	5.39E-03	2.04E-02	NA	NA	NA	MODIFIER	NA	NA	100	99	0	76	29	47	61.84
Non-user	IOB_24	chr10	122273374	T	C	PLPP4	NCT	196051:[NR_134516.1(n.309+9936T>C)]	NA	NA	2.00E-04	3.06E-04	0.00E+00	2.00E-04	0.00E+00	NA	NA	NA	MODIFIER	NA	NA	366	364	0	270	136	133	49.26
Non-user	IOB_24	chr10	122273374	T	C	PPAPDC1A	INTRONIC	ENSG00000203805:[ENST00000398250(c.166-49T>C)]	NA	NA	2.00E-04	3.06E-04	0.00E+00	2.00E-04	0.00E+00	NA	NA	NA	MODIFIER	NA	NA	366	364	0	270	136	133	49.26
Non-user	IOB_24	chr10	122661777	A	G	WDR11	MISSENSE	ENSG00000120008:[ENST00000263461(c.2696A>G,aAg>aGg):ENSP00000263461(p.Lys899Arg)]	NA	NA	NA	NA	NA	NA	NA	NA	NA	NA	MODIFIER	NA	NA	122	121	0	108	56	52	48.15
Non-user	IOB_24	chr10	123997018	G	A	TACC2	MISSENSE	ENSG00000138162:[ENST00000360561(c.2380G>A,Gac>Aac):ENSP00000353763(p.Asp794Asn)]	NA	chr10*123997018*123997018*COSM147072*G*A*1*stomach*1	2.12E-02	1.60E-02	3.64E-02	2.12E-02	3.37E-02	D_lc	BN	NA	MODERATE	NA	NA	87	87	0	68	36	32	47.06
Non-user	IOB_24	chr10	124043544	A	G	BTBD16	INTRONIC	118663:[XM_005269495.1(c.-119-5880A>G)]	NA	NA	1.24E-02	NA	NA	1.24E-02	4.10E-03	NA	NA	NA	MODIFIER	NA	NA	172	172	0	107	45	62	57.94
Non-user	IOB_24	chr10	124325400	C	T	DMBT1	INTRONIC	ENSG00000187908:[ENST00000330163(c.62-83C>T)]	NA	NA	1.48E-02	NA	NA	1.48E-02	6.10E-03	NA	NA	NA	MODIFIER	NA	NA	134	134	0	103	61	42	40.78
Non-user	IOB_24	chr10	124806770	A	G	ACADSB	MISSENSE	ENSG00000196177:[ENST00000368869(c.640A>G,Att>Gtt):ENSP00000357862(p.Ile214Val)]	NA	NA	3.89E-02	6.04E-02	2.70E-02	3.89E-02	1.84E-02	T	BN	NA	MODERATE	N	1	74	74	0	68	40	28	41.18
Non-user	IOB_24	chr10	126094203	C	T	OAT	EXONIC-NC	ENSG00000065154:[ENST00000483711(n.296G>A)]	NA	NA	9.98E-04	NA	NA	9.98E-04	4.10E-03	NA	NA	NA	MODIFIER	NA	NA	64	64	0	54	37	17	31.48
Non-user	IOB_24	chr10	126138345	C	T	NKX1-2	SILENT	ENSG00000229544:[ENST00000440536(c.168G>A,ggG>ggA):ENSP00000450924(p.Gly56(=))]	NA	NA	4.89E-02	8.04E-02	7.35E-02	4.89E-02	6.65E-02	NA	NA	NA	MODIFIER	NA	NA	114	112	1	95	41	54	56.84
Non-user	IOB_24	chr10	126138345	C	T	RP13-238F13.3	NCT	ENSG00000271434:[ENST00000604581(n.197-44C>T)]	NA	NA	4.89E-02	8.04E-02	7.35E-02	4.89E-02	6.65E-02	NA	NA	NA	MODIFIER	NA	NA	114	112	1	95	41	54	56.84
Non-user	IOB_24	chr10	127585154	G	A	FANK1	5UTR	ENSG00000203780:[ENST00000368695(c.-76G>A)]	NA	NA	NA	NA	NA	NA	NA	NA	NA	NA	MODIFIER	NA	NA	665	648	0	378	353	13	3.44
Non-user	IOB_24	chr10	127585186	C	A	FANK1	5UTR	92565:[XM_005270280.1(c.-292C>A)]	NA	NA	NA	2.33E-02	3.43E-02	NA	NA	NA	NA	NA	MODIFIER	NA	NA	450	419	0	245	215	16	6.53
Non-user	IOB_24	chr10	127585233	G	A	FANK1	INTRONIC	92565:[XM_005270277.1(c.-31+9G>A)]	NA	NA	NA	1.55E-04	2.12E-03	NA	NA	NA	NA	NA	MODIFIER	NA	NA	237	236	0	124	118	6	4.84
Non-user	IOB_24	chr10	127787012	C	T	ADAM12	SILENT	ENSG00000148848:[ENST00000368676(c.978G>A,acG>acA):ENSP00000357665(p.Thr326(=))]	NA	NA	3.49E-02	1.77E-02	2.82E-02	3.49E-02	3.17E-02	NA	NA	NA	LOW	NA	NA	91	91	0	69	32	37	53.62
Non-user	IOB_24	chr10	128851051	A	G	DOCK1	MISSENSE	ENSG00000150760:[ENST00000280333(c.2245A>G,Atc>Gtc):ENSP00000280333(p.Ile749Val)]	NA	NA	3.00E-03	1.05E-03	7.60E-03	3.00E-03	1.53E-02	NA	NA	NA	MODERATE	NA	1	97	97	0	61	30	30	49.18
Non-user	IOB_24	chr10	129137361	G	T	DOCK1	INTRONIC	ENSG00000150760:[ENST00000280333(c.3060+13G>T)]	NA	NA	1.80E-02	1.50E-02	1.71E-02	1.80E-02	1.12E-02	NA	NA	NA	MODIFIER	NA	NA	213	213	0	186	92	94	50.54
Non-user	IOB_24	chr10	131755493	G	C	EBF3	INTRONIC	ENSG00000108001:[ENST00000355311(c.554+29C>G)]	NA	NA	1.68E-02	2.53E-02	2.48E-02	1.68E-02	2.76E-02	NA	NA	NA	MODIFIER	NA	NA	179	178	0	163	78	85	52.15
Non-user	IOB_24	chr10	131906394	C	T	CTAGE7P	EXONIC-NC	ENSG00000233122:[ENST00000435712(n.2085C>T)]	NA	NA	3.59E-03	1.81E-03	1.23E-02	3.59E-03	1.74E-02	NA	NA	NA	MODIFIER	NA	NA	155	154	0	115	68	46	40
Non-user	IOB_24	chr10	131906394	C	T	LINC00959	NCT	ENSG00000237489:[ENST00000456581(n.114+2574G>A)]	NA	NA	3.59E-03	1.81E-03	1.23E-02	3.59E-03	1.74E-02	NA	NA	NA	MODIFIER	NA	NA	155	154	0	115	68	46	40
Non-user	IOB_24	chr10	131906396	T	C	CTAGE7P	EXONIC-NC	ENSG00000233122:[ENST00000417351(n.2124T>C)]	NA	NA	3.59E-03	1.82E-03	1.23E-02	3.59E-03	1.74E-02	NA	NA	NA	MODIFIER	NA	NA	154	153	0	115	69	45	39.13
Non-user	IOB_24	chr10	131906396	T	C	LINC00959	NCT	ENSG00000237489:[ENST00000456581(n.114+2572A>G)]	NA	NA	3.59E-03	1.82E-03	1.23E-02	3.59E-03	1.74E-02	NA	NA	NA	MODIFIER	NA	NA	154	153	0	115	69	45	39.13
Non-user	IOB_24	chr10	134649681	A	G	TTC40	MISSENSE	ENSG00000171811:[ENST00000368586(c.6578T>C,aTt>aCt):ENSP00000357575(p.Ile2193Thr)]	NA	NA	NA	1.68E-05	1.22E-04	NA	NA	T	BN	NA	MODERATE	N	0	150	147	0	137	70	65	47.45
Non-user	IOB_24	chr10	134723471	C	T	TTC40	SILENT	ENSG00000171811:[ENST00000368582(c.2601G>A,aaG>aaA):ENSP00000357571(p.Lys867(=))]	NA	NA	9.98E-04	3.85E-03	7.59E-03	9.98E-04	4.10E-03	NA	NA	NA	MODIFIER	NA	NA	118	118	0	100	48	52	52
Non-user	IOB_24	chr10	135015114	G	A	KNDC1	SILENT	ENSG00000171798:[ENST00000368571(c.2904G>A,ggG>ggA):ENSP00000357560(p.Gly968(=))]	NA	NA	NA	NA	NA	NA	NA	NA	NA	NA	LOW	NA	NA	104	104	0	65	36	29	44.62
Non-user	IOB_24	chr10	135051678	G	A	VENTX	INTRONIC	ENSG00000151650:[ENST00000325980(c.241+19G>A)]	NA	NA	3.49E-02	1.20E-02	1.45E-02	3.49E-02	1.23E-02	NA	NA	NA	MODIFIER	NA	NA	43	43	0	41	12	29	70.73
Non-user	IOB_24	chr10	135061108	C	T	MIR202HG	EXONIC-NC	ENSG00000166917:[ENST00000300167(n.193G>A)]	NA	NA	2.00E-04	3.47E-04	7.59E-04	2.00E-04	0.00E+00	NA	NA	NA	MODIFIER	NA	NA	99	99	0	82	44	38	46.34
Non-user	IOB_24	chr10	135236793	G	C	SPRN	SILENT	ENSG00000203772:[ENST00000414069(c.393C>G,cgC>cgG):ENSP00000433712(p.Arg131(=))]	NA	NA	NA	9.82E-05	1.43E-04	NA	NA	NA	NA	NA	LOW	NA	NA	106	106	0	97	51	46	47.42
Non-user	IOB_24	chr11	375700	G	A	B4GALNT4	SILENT	ENSG00000182272:[ENST00000329962(c.912G>A,gtG>gtA):ENSP00000328277(p.Val304(=))]	NA	NA	5.79E-03	3.10E-03	2.26E-02	5.79E-03	2.86E-02	NA	NA	NA	LOW	NA	NA	345	345	0	266	122	142	53.38
Non-user	IOB_24	chr11	377100	C	G	B4GALNT4	SILENT	ENSG00000182272:[ENST00000329962(c.1977C>G,gcC>gcG):ENSP00000328277(p.Ala659(=))]	NA	NA	NA	5.60E-04	1.03E-03	NA	NA	NA	NA	NA	LOW	NA	NA	95	95	0	98	41	57	58.16
Non-user	IOB_24	chr11	430339	C	G	ANO9	MISSENSE	ENSG00000185101:[ENST00000332826(c.604G>C,Gtg>Ctg):ENSP00000332788(p.Val202Leu)]	NA	NA	1.88E-02	3.51E-02	6.04E-03	1.88E-02	7.20E-03	NA	NA	NA	MODIFIER	NA	NA	121	121	0	93	40	53	56.99
Non-user	IOB_24	chr11	558165	T	G	C11orf35	MISSENSE	ENSG00000185522:[ENST00000329451(c.395A>C,cAg>cCg):ENSP00000331167(p.Gln132Pro)]	NA	NA	4.61E-02	6.17E-02	3.67E-02	4.61E-02	3.99E-02	NA	NA	NA	MODERATE	NA	0.919	219	218	0	186	83	103	55.38
Non-user	IOB_24	chr11	558165	T	G	RP11-496I9.1	EXONIC-NC	ENSG00000254815:[ENST00000527620(n.571T>G)]	NA	NA	4.61E-02	6.17E-02	3.67E-02	4.61E-02	3.99E-02	NA	NA	NA	MODERATE	NA	0.919	219	218	0	186	83	103	55.38
Non-user	IOB_24	chr11	581503	A	G	PHRF1	5UTR	ENSG00000070047:[ENST00000264555(c.-10A>G)]	NA	NA	NA	4.22E-05	1.83E-04	NA	NA	NA	NA	NA	MODIFIER	NA	NA	416	411	1	290	145	143	49.31
Non-user	IOB_24	chr11	608210	G	T	PHRF1	MISSENSE	ENSG00000070047:[ENST00000533464(c.2742G>T,gaG>gaT):ENSP00000431870(p.Glu914Asp)]	NA	NA	NA	NA	NA	NA	NA	T	PoD	NA	MODERATE	NA	NA	94	94	0	98	47	51	52.04
Non-user	IOB_24	chr11	621368	T	C	CDHR5	MISSENSE	ENSG00000099834:[ENST00000526077(c.502A>G,Atg>Gtg):ENSP00000435082(p.Met168Val)]	NA	NA	NA	9.15E-05	6.68E-04	NA	NA	D	PrD	NA	MODERATE	N	0	157	156	0	139	73	66	47.48
Non-user	IOB_24	chr11	627517	C	T	NA	INTERGENIC	NA	NA	NA	3.75E-02	NA	NA	3.75E-02	3.68E-02	NA	NA	NA	MODIFIER	NA	NA	70	70	0	87	40	46	52.87
Non-user	IOB_24	chr11	698799	G	T	DEAF1	NCT	ENSG00000177030:[ENST00000525626(n.145-7201C>A)]	NA	NA	NA	NA	NA	NA	NA	NA	NA	NA	MODIFIER	NA	NA	102	101	0	90	46	44	48.89
Non-user	IOB_24	chr11	698799	G	T	EPS8L2	INTRONIC	ENSG00000177106:[ENST00000524763(c.-227+4324G>T)]	NA	NA	NA	NA	NA	NA	NA	NA	NA	NA	MODIFIER	NA	NA	102	101	0	90	46	44	48.89
Non-user	IOB_24	chr11	698799	G	T	NA	NCT	101927373:[XR_242839.1(n.97-800C>A)]	NA	NA	NA	NA	NA	NA	NA	NA	NA	NA	MODIFIER	NA	NA	102	101	0	90	46	44	48.89
Non-user	IOB_24	chr11	698799	G	T	TMEM80	INTRONIC	ENSG00000177042:[ENST00000526170(c.52-70G>T)]	NA	NA	NA	NA	NA	NA	NA	NA	NA	NA	MODIFIER	NA	NA	102	101	0	90	46	44	48.89
Non-user	IOB_24	chr11	792428	C	T	SLC25A22	SILENT	ENSG00000177542:[ENST00000481290(c.693G>A,ccG>ccA):ENSP00000431829(p.Pro231(=))]	NA	NA	1.50E-02	8.08E-03	4.42E-02	1.50E-02	4.70E-02	NA	NA	NA	LOW	NA	NA	253	250	0	202	93	107	52.97
Non-user	IOB_24	chr11	800414	G	A	PIDD	SILENT	ENSG00000177595:[ENST00000347755(c.2079C>T,gtC>gtT):ENSP00000337797(p.Val693(=))]	NA	NA	3.00E-02	1.40E-02	3.74E-02	3.00E-02	3.89E-02	NA	NA	NA	MODIFIER	NA	NA	219	216	0	155	95	58	37.42
Non-user	IOB_24	chr11	800432	C	T	PIDD	SILENT	ENSG00000177595:[ENST00000347755(c.2061G>A,gaG>gaA):ENSP00000337797(p.Glu687(=))]	NA	NA	1.40E-02	7.77E-03	3.93E-02	1.40E-02	3.89E-02	NA	NA	NA	LOW	NA	0	221	219	0	155	96	56	36.13
Non-user	IOB_24	chr11	821686	C	A	PNPLA2	SILENT	ENSG00000177666:[ENST00000336615(c.246C>A,ggC>ggA):ENSP00000337701(p.Gly82(=))]	NA	NA	5.59E-03	3.51E-03	2.53E-02	5.59E-03	2.86E-02	NA	NA	NA	LOW	NA	NA	160	160	0	134	77	57	42.54
Non-user	IOB_24	chr11	821686	C	A	RPLP2	INTRONIC	6181:[XM_005253056.1(c.272-5809C>A)]	NA	NA	5.59E-03	3.51E-03	2.53E-02	5.59E-03	2.86E-02	NA	NA	NA	LOW	NA	NA	160	160	0	134	77	57	42.54
Non-user	IOB_24	chr11	1021268	T	C	MUC6	MISSENSE	ENSG00000184956:[ENST00000421673(c.3536A>G,aAc>aGc):ENSP00000406861(p.Asn1179Ser)]	NA	NA	2.58E-02	5.24E-02	9.20E-02	2.58E-02	6.85E-02	D	PrD	NA	MODERATE	NA	NA	395	391	0	352	170	178	50.57
Non-user	IOB_24	chr11	1029135	C	T	MUC6	MISSENSE	ENSG00000184956:[ENST00000421673(c.1291G>A,Gag>Aag):ENSP00000406861(p.Glu431Lys)]	NA	NA	2.78E-02	4.96E-02	6.29E-02	2.78E-02	4.81E-02	T	BN	NA	MODERATE	NA	NA	188	187	0	179	105	72	40.22
Non-user	IOB_24	chr11	1090881	C	T	MUC2	MISSENSE	ENSG00000198788:[ENST00000441003(c.3776C>T,aCg>aTg):ENSP00000415183(p.Thr1259Met)]	NA	NA	2.00E-04	1.34E-04	1.22E-04	2.00E-04	0.00E+00	NA	PoD	NA	MODERATE	NA	NA	180	179	0	147	85	62	42.18
Non-user	IOB_24	chr11	1213480	C	T	LOC101927530	MISSENSE	101927530:[XM_005253273.1(c.694C>T,Cgg>Tgg):XP_005253330.1(p.Arg232Trp)]	NA	NA	NA	1.76E-05	0.00E+00	NA	NA	NA	NA	NA	MODIFIER	NA	NA	1143	1137	0	844	800	41	4.86
Non-user	IOB_24	chr11	1213480	C	T	MUC5AC	MISSENSE	4586:[NM_001304359.1(c.2596C>T,Cgg>Tgg):NP_001291288.1(p.Arg866Trp)]	NA	NA	NA	1.76E-05	0.00E+00	NA	NA	NA	NA	NA	MODIFIER	NA	NA	1143	1137	0	844	800	41	4.86
Non-user	IOB_24	chr11	1279576	G	A	MUC5B	SILENT	ENSG00000117983:[ENST00000526859(c.207G>A,tcG>tcA):ENSP00000434539(p.Ser69(=))]	NA	NA	2.20E-03	1.57E-03	1.12E-02	2.20E-03	1.12E-02	NA	NA	NA	LOW	NA	NA	226	222	1	114	57	56	49.12
Non-user	IOB_24	chr11	2154502	C	A	IGF2	INTRONIC	ENSG00000167244:[ENST00000434045(c.475-49G>T)]	NA	NA	4.99E-03	1.22E-03	1.46E-02	4.99E-03	2.56E-02	NA	NA	NA	MODIFIER	NA	NA	117	117	0	90	56	34	37.78
Non-user	IOB_24	chr11	2154502	C	A	INS-IGF2	INTRONIC	ENSG00000129965:[ENST00000356578(c.*359-49G>T)]	NA	NA	4.99E-03	1.22E-03	1.46E-02	4.99E-03	2.56E-02	NA	NA	NA	MODIFIER	NA	NA	117	117	0	90	56	34	37.78
Non-user	IOB_24	chr11	2169055	G	C	IGF2	INTRONIC	3481:[XM_005252901.1(c.-252-18C>G)]	NA	NA	NA	5.38E-04	0.00E+00	NA	NA	NA	NA	NA	MODIFIER	NA	NA	24	24	0	14	5	9	64.29
Non-user	IOB_24	chr11	2169055	G	C	IGF2-AS	EXONIC-NC	51214:[NR_028044.1(n.2001G>C)]	NA	NA	NA	5.38E-04	0.00E+00	NA	NA	NA	NA	NA	MODIFIER	NA	NA	24	24	0	14	5	9	64.29

Patel *et al.* , 2021. Whole-exome sequencing analysis of oral squamous cell carcinoma delineated by tobacco usage habits
Supplementary Table 4. List of somatic single nucleotide variants identified using whole exome sequencing of OSCC patients

Cohort	Sample	Chromosome	Genomic position	Reference allele	Alternate allele	Genesymbol	Variant type	Variant description	OncoMD	COSMIC	dbSNP	ExAC	ExAC_SAS	1000G	1000G_SAS	SIFT	Polyphen2	CONDEL	VEP	LRT	PhastCon7Way	Total reads in Normal	Reference read support in Normal	Alternative read support in Normal	Total reads in Tumor	Reference read support in Tumor	Alternate read support in Tumor	Alternate allele frequency in Tumor
Non-user	IOB_24	chr11	2169055	G	C	INS-IGF2	INTRONIC	ENSG00000129965:[ENST00000356578(c.408-18C>G)]	NA	NA	NA	5.38E-04	0.00E+00	NA	NA	NA	NA	NA	MODIFIER	NA	NA	24	24	0	14	5	9	64.29
Non-user	IOB_24	chr11	3059360	C	A	CARS	MISSENSE	ENSG00000110619:[ENST00000401769(c.511G>T,Gca>Tca):ENSP00000384069(p.Ala171Ser)]	NA	chr11*3059360*3059360*COSM542256*C*T*1*lung*1|chr11*3059360*3059360*COSM542257*C*T*1*lung*1	7.39E-03	9.85E-03	2.28E-02	7.39E-03	2.15E-02	T	BN	NA	MODERATE	N	0.003	193	193	0	151	73	78	51.66
Non-user	IOB_24	chr11	3059360	C	A	CARS-AS1	NCT	ENSG00000247473:[ENST00000499962(n.2184-2650C>A)]	NA	chr11*3059360*3059360*COSM542256*C*T*1*lung*1|chr11*3059360*3059360*COSM542257*C*T*1*lung*1	7.39E-03	9.85E-03	2.28E-02	7.39E-03	2.15E-02	T	BN	NA	MODERATE	N	0.003	193	193	0	151	73	78	51.66
Non-user	IOB_24	chr11	3661543	T	C	ART5	MISSENSE	ENSG00000167311:[ENST00000425767(c.53A>G,tAt>tGt):ENSP00000413852(p.Tyr18Cys)]	NA	NA	7.99E-04	1.47E-03	1.22E-03	7.99E-04	2.00E-03	NA	NA	NA	MODIFIER	NA	NA	312	311	0	275	159	114	41.45
Non-user	IOB_24	chr11	4105991	C	T	STIM1	SILENT	ENSG00000167323:[ENST00000526596(c.755C>T,taC>taT):ENSP00000433266(p.Tyr252(=))]	NA	NA	NA	NA	NA	NA	NA	NA	NA	NA	LOW	NA	NA	60	59	0	35	12	23	65.71
Non-user	IOB_24	chr11	4407557	T	C	TRIM21	INTRONIC	ENSG00000132109:[ENST00000254436(c.759-70A>G)]	NA	NA	1.40E-02	4.27E-02	2.32E-02	1.40E-02	1.64E-02	NA	NA	NA	LOW	NA	NA	34	34	0	37	16	21	56.76
Non-user	IOB_24	chr11	4566923	G	A	OR52M1	MISSENSE	ENSG00000197790:[ENST00000360213(c.503G>A,cGg>cAg):ENSP00000353343(p.Arg168Gln)]	NA	chr11*4566923*4566923*COSM218320*G*A*4*pancreas*4	2.00E-04	9.98E-05	2.54E-04	2.00E-04	0.00E+00	T	BN	NA	MODERATE	N	0	181	180	0	143	67	76	53.15
Non-user	IOB_24	chr11	4967651	A	G	MMP26	NCT	ENSG00000167346:[ENST00000477339(n.192-41849A>G)]	NA	NA	NA	2.15E-01	4.02E-01	NA	NA	NA	NA	NA	MODIFIER	NA	NA	423	421	0	211	160	49	23.22
Non-user	IOB_24	chr11	4967651	A	G	OR51A4	MISSENSE	ENSG00000205497:[ENST00000380373(c.680T>C,cTg>cCg):ENSP00000369731(p.Leu227Pro)]	NA	NA	NA	2.15E-01	4.02E-01	NA	NA	NA	NA	NA	MODIFIER	NA	NA	423	421	0	211	160	49	23.22
Non-user	IOB_24	chr11	4976959	C	T	MMP26	NCT	ENSG00000167346:[ENST00000477339(n.192-32541C>T)]	NA	NA	6.79E-03	2.05E-02	3.14E-03	6.79E-03	5.10E-03	NA	NA	NA	MODIFIER	NA	NA	171	171	0	102	45	57	55.88
Non-user	IOB_24	chr11	5173149	C	A	OR52A1	MISSENSE	ENSG00000182070:[ENST00000328942(c.451G>T,Gta>Tta):ENSP00000333684(p.Val151Leu)]	NA	NA	3.79E-02	4.64E-02	2.69E-02	3.79E-02	2.25E-02	T	BN	NA	MODERATE	N	0	170	167	0	142	65	77	54.23
Non-user	IOB_24	chr11	5410937	T	G	AC104389.28	NCT	ENSG00000167355:[ENST00000420726(n.303-42797A>C)]	NA	NA	3.20E-03	1.32E-03	9.45E-03	3.19E-03	1.64E-02	NA	NA	NA	MODIFIER	NA	NA	344	342	1	252	129	119	47.22
Non-user	IOB_24	chr11	5410937	T	G	HBE1	INTRONIC	ENSG00000213931:[ENST00000396895(c.-267+115862A>C)]	NA	NA	3.20E-03	1.32E-03	9.45E-03	3.19E-03	1.64E-02	NA	NA	NA	MODIFIER	NA	NA	344	342	1	252	129	119	47.22
Non-user	IOB_24	chr11	5410937	T	G	HBG2	INTRONIC	ENSG00000196565:[ENST00000380259(c.-564-43808A>C)]	NA	NA	3.20E-03	1.32E-03	9.45E-03	3.19E-03	1.64E-02	NA	NA	NA	MODIFIER	NA	NA	344	342	1	252	129	119	47.22
Non-user	IOB_24	chr11	5410937	T	G	OR51B5	INTRONIC	282763:[NM_001005567.2(c.-363-42797A>C)]	NA	NA	3.20E-03	1.32E-03	9.45E-03	3.19E-03	1.64E-02	NA	NA	NA	MODIFIER	NA	NA	344	342	1	252	129	119	47.22
Non-user	IOB_24	chr11	5410937	T	G	OR51M1	MISSENSE	ENSG00000184698:[ENST00000328611(c.309T>G,agT>agG):ENSP00000333196(p.Ser103Arg)]	NA	NA	3.20E-03	1.32E-03	9.45E-03	3.19E-03	1.64E-02	NA	NA	NA	MODIFIER	NA	NA	344	342	1	252	129	119	47.22
Non-user	IOB_24	chr11	5631465	A	C	AC015691.13	NCT	ENSG00000239920:[ENST00000394793(n.71+14591T>G)]	NA	NA	2.00E-04	2.55E-04	1.82E-03	2.00E-04	1.00E-03	NA	NA	NA	LOW	NA	NA	119	118	0	64	39	25	39.06
Non-user	IOB_24	chr11	5631465	A	C	HBG2	INTRONIC	ENSG00000196565:[ENST00000380259(c.-882-14885T>G)]	NA	NA	2.00E-04	2.55E-04	1.82E-03	2.00E-04	1.00E-03	NA	NA	NA	LOW	NA	NA	119	118	0	64	39	25	39.06
Non-user	IOB_24	chr11	5631465	A	C	NA	NCT	101927800:[XR_242846.1(n.240+10452T>G)]	NA	NA	2.00E-04	2.55E-04	1.82E-03	2.00E-04	1.00E-03	NA	NA	NA	LOW	NA	NA	119	118	0	64	39	25	39.06
Non-user	IOB_24	chr11	5631465	A	C	TRIM6	SILENT	ENSG00000121236:[ENST00000380097(c.948A>C,cgA>cgC):ENSP00000369440(p.Arg316(=))]	NA	NA	2.00E-04	2.55E-04	1.82E-03	2.00E-04	1.00E-03	NA	NA	NA	LOW	NA	NA	119	118	0	64	39	25	39.06
Non-user	IOB_24	chr11	5631465	A	C	TRIM6-TRIM34	SILENT	ENSG00000258588:[ENST00000354852(c.948A>C,cgA>cgC):ENSP00000346916(p.Arg316(=))]	NA	NA	2.00E-04	2.55E-04	1.82E-03	2.00E-04	1.00E-03	NA	NA	NA	LOW	NA	NA	119	118	0	64	39	25	39.06
Non-user	IOB_24	chr11	5799876	A	G	OR52N5	5UTR	ENSG00000181009:[ENST00000317093(c.-12T>C)]	NA	NA	1.46E-02	3.59E-02	1.48E-02	1.46E-02	8.20E-03	NA	NA	NA	MODIFIER	NA	NA	18	18	0	25	11	14	56
Non-user	IOB_24	chr11	5799876	A	G	TRIM5	INTRONIC	ENSG00000132256:[ENST00000380027(c.-441+77106T>C)]	NA	NA	1.46E-02	3.59E-02	1.48E-02	1.46E-02	8.20E-03	NA	NA	NA	MODIFIER	NA	NA	18	18	0	25	11	14	56
Non-user	IOB_24	chr11	6423941	G	A	APBB1	SILENT	ENSG00000166313:[ENST00000609331(c.414C>T,cgC>cgT):ENSP00000477069(p.Arg138(=))]	NA	NA	2.00E-04	5.11E-04	1.21E-04	2.00E-04	0.00E+00	NA	NA	NA	MODIFIER	NA	NA	196	196	0	167	84	82	49.1
Non-user	IOB_24	chr11	7570729	C	T	PPFIBP2	MISSENSE	ENSG00000166387:[ENST00000299492(c.23C>T,gCg>gTg):ENSP00000299492(p.Ala8Val)]	NA	chr11*7570729*7570729*COSM3416240*C*T*1*large intestine*1	NA	1.23E-04	1.26E-04	NA	NA	T	BN	NA	MODERATE	NA	NA	145	144	1	152	144	6	3.95
Non-user	IOB_24	chr11	7949707	A	G	OR10A6	MISSENSE	ENSG00000175393:[ENST00000309838(c.503T>C,tTt>tCt):ENSP00000312470(p.Phe168Ser)]	NA	NA	1.84E-02	3.43E-02	1.70E-02	1.84E-02	1.94E-02	D	PoD	NA	MODERATE	NA	0.625	104	103	0	66	33	33	50
Non-user	IOB_24	chr11	8149883	G	A	RIC3	NCT	79608:[NR_144485.1(n.676-1532C>T)]	NA	NA	3.14E-02	NA	NA	3.13E-02	1.23E-02	NA	NA	NA	MODIFIER	NA	NA	61	60	0	47	30	17	36.17
Non-user	IOB_24	chr11	8414229	G	T	STK33	MISSENSE	ENSG00000130413:[ENST00000358872(c.812C>A,gCa>gAa):ENSP00000351743(p.Ala271Glu)]	NA	NA	1.86E-02	2.37E-02	7.44E-02	1.86E-02	7.16E-02	T	BN	NA	MODERATE	NA	NA	196	193	0	163	83	80	49.08
Non-user	IOB_24	chr11	8641037	T	C	TRIM66	MISSENSE	ENSG00000166436:[ENST00000299550(c.3265A>G,Atc>Gtc):ENSP00000299550(p.Ile1089Val)]	NA	NA	1.02E-02	2.56E-02	5.27E-02	1.02E-02	3.37E-02	NA	NA	NA	MODIFIER	NA	NA	101	100	0	73	38	35	47.95
Non-user	IOB_24	chr11	8739491	C	G	ST5	INTRONIC	6764:[XM_005253081.1(c.1568-52G>C)]	NA	NA	1.50E-02	2.02E-02	5.92E-02	1.50E-02	6.13E-02	NA	NA	NA	MODIFIER	NA	NA	46	46	0	50	32	18	36
Non-user	IOB_24	chr11	9456659	A	G	IPO7	INTRONIC	ENSG00000205339:[ENST00000379719(c.2172+81A>G)]	NA	NA	4.39E-03	NA	NA	4.39E-03	2.25E-02	NA	NA	NA	MODIFIER	NA	NA	77	77	0	52	36	16	30.77
Non-user	IOB_24	chr11	10022590	C	T	SBF2	NCT	ENSG00000133812:[ENST00000527019(n.700-21G>A)]	NA	NA	3.20E-03	1.57E-03	1.06E-02	3.19E-03	1.64E-02	NA	NA	NA	MODIFIER	NA	NA	36	36	0	23	14	9	39.13
Non-user	IOB_24	chr11	10829923	G	T	EIF4G2	MISSENSE	1982:[XM_005252817.1(c.95C>A,gCg>gAg):XP_005252874.1(p.Ala32Glu)]	NA	NA	5.19E-03	3.43E-02	4.35E-02	5.19E-03	2.45E-02	NA	NA	NA	MODERATE	NA	NA	102	102	0	76	24	52	68.42
Non-user	IOB_24	chr11	11348794	C	T	GALNT18	INTRONIC	ENSG00000110328:[ENST00000227756(c.1417-66G>A)]	NA	NA	4.37E-02	NA	NA	4.37E-02	6.54E-02	NA	NA	NA	MODIFIER	NA	NA	43	42	0	80	35	45	56.25
Non-user	IOB_24	chr11	14900931	G	A	CYP2R1	SILENT	ENSG00000186104:[ENST00000334636(c.1059C>T,gaC>gaT):ENSP00000334592(p.Asp353(=))]	NA	NA	1.16E-02	1.86E-02	2.55E-02	1.16E-02	2.45E-02	NA	NA	NA	LOW	NA	0.037	119	119	0	100	60	40	40
Non-user	IOB_24	chr11	17118730	A	G	PIK3C2A	SILENT	ENSG00000011405:[ENST00000265970(c.4200T>C,ggT>ggC):ENSP00000265970(p.Gly1400(=))]	NA	NA	5.39E-03	1.09E-02	2.14E-02	5.39E-03	1.53E-02	NA	NA	NA	MODIFIER	NA	NA	202	202	0	164	97	67	40.85
Non-user	IOB_24	chr11	17526256	A	G	USH1C	INTRONIC	ENSG00000006611:[ENST00000526313(c.1211-2729T>C)]	NA	NA	2.62E-02	1.34E-01	1.42E-01	2.62E-02	4.09E-02	NA	NA	NA	MODIFIER	NA	NA	136	136	0	135	74	59	43.7
Non-user	IOB_24	chr11	17667267	G	T	OTOG	INTRONIC	ENSG00000188162:[ENST00000399397(c.8359-24G>T)]	NA	NA	5.39E-03	9.76E-03	8.87E-03	5.39E-03	1.53E-02	NA	NA	NA	MODIFIER	NA	NA	353	351	0	296	139	155	52.36
Non-user	IOB_24	chr11	18022379	T	C	RP1-59M18.2	NCT	ENSG00000255448:[ENST00000525523(n.673-275A>G)]	NA	NA	5.99E-03	9.62E-03	8.93E-03	5.99E-03	8.20E-03	NA	NA	NA	MODIFIER	NA	NA	114	113	0	72	32	40	55.56
Non-user	IOB_24	chr11	18022379	T	C	SERGEF	INTRONIC	ENSG00000129158:[ENST00000525920(c.58-275A>G)]	NA	NA	5.99E-03	9.62E-03	8.93E-03	5.99E-03	8.20E-03	NA	NA	NA	MODIFIER	NA	NA	114	113	0	72	32	40	55.56
Non-user	IOB_24	chr11	18102014	G	A	SAAL1	MISSENSE	ENSG00000166788:[ENST00000529318(c.1363C>T,Cgt>Tgt):ENSP00000432216(p.Arg455Cys)]	NA	NA	9.98E-03	1.45E-02	1.54E-02	9.98E-03	2.04E-02	NA	NA	NA	MODIFIER	NA	NA	48	46	0	59	26	33	55.93
Non-user	IOB_24	chr11	18267463	A	G	SAA2	MISSENSE	ENSG00000134339:[ENST00000256733(c.224T>C,gTg>gCg):ENSP00000256733(p.Val75Ala)]	NA	NA	NA	3.85E-01	3.72E-01	NA	NA	D	BN	NA	MODERATE	NA	0.986	243	241	0	168	131	37	22.02
Non-user	IOB_24	chr11	18267463	A	G	SAA2-SAA4	EXONIC-NC	ENSG00000255071:[ENST00000524555(n.261T>C)]	NA	NA	NA	3.85E-01	3.72E-01	NA	NA	D	BN	NA	MODERATE	NA	0.986	243	241	0	168	131	37	22.02
Non-user	IOB_24	chr11	18290874	T	C	SAA1	MISSENSE	ENSG00000173432:[ENST00000356524(c.224T>C,gTg>gCg):ENSP00000348918(p.Val75Ala)]	NA	NA	NA	5.41E-01	4.87E-01	NA	NA	D	BN	NA	MODERATE	N	1	56	55	0	23	10	9	39.13
Non-user	IOB_24	chr11	18305355	C	T	HPS5	MISSENSE	ENSG00000110756:[ENST00000349215(c.3045G>A,atG>atA):ENSP00000265967(p.Met1015Ile)]	NA	NA	2.20E-03	5.33E-03	1.88E-03	2.20E-03	1.00E-03	NA	NA	NA	MODIFIER	NA	NA	199	198	0	178	80	98	55.06
Non-user	IOB_24	chr11	18305355	C	T	SAA1	INTRONIC	6288:[XM_005253061.1(c.231-1027C>T)]	NA	NA	2.20E-03	5.33E-03	1.88E-03	2.20E-03	1.00E-03	NA	NA	NA	MODIFIER	NA	NA	199	198	0	178	80	98	55.06
Non-user	IOB_24	chr11	18723454	G	A	TMEM86A	SILENT	ENSG00000151117:[ENST00000280734(c.621G>A,gcG>gcA):ENSP00000280734(p.Ala207(=))]	NA	NA	5.59E-03	5.85E-03	1.84E-02	5.59E-03	2.35E-02	NA	NA	NA	LOW	NA	NA	264	262	0	183	99	84	45.9
Non-user	IOB_24	chr11	18727381	C	T	IGSF22	MISSENSE	ENSG00000179057:[ENST00000513874(c.3893G>A,tGc>tAc):ENSP00000421191(p.Cys1298Tyr)]	NA	NA	3.00E-03	7.61E-03	1.76E-02	3.00E-03	1.53E-02	NA	NA	NA	MODIFIER	NA	NA	82	82	0	69	36	33	47.83
Non-user	IOB_24	chr11	18735677	G	A	IGSF22	MISSENSE	ENSG00000179057:[ENST00000319338(c.1817C>T,cCg>cTg):ENSP00000322422(p.Pro606Leu)]	NA	NA	1.06E-02	1.61E-02	2.56E-02	1.06E-02	3.17E-02	NA	NA	NA	MODIFIER	NA	NA	228	226	0	150	71	79	52.67
Non-user	IOB_24	chr11	18735677	G	A	RP11-1081L13.4	NCT	ENSG00000254966:[ENST00000527285(n.729+6737G>A)]	NA	NA	1.06E-02	1.61E-02	2.56E-02	1.06E-02	3.17E-02	NA	NA	NA	MODIFIER	NA	NA	228	226	0	150	71	79	52.67
Non-user	IOB_24	chr11	20078214	C	G	NAV2	INTRONIC	89797:[XM_005253215.1(c.1828+711C>G)]	NA	NA	NA	7.68E-04	6.95E-04	NA	NA	NA	NA	NA	MODIFIER	NA	NA	140	140	0	98	45	53	54.08
Non-user	IOB_24	chr11	20113812	G	A	NAV2	MISSENSE	ENSG00000166833:[ENST00000396085(c.5722G>A,Ggc>Agc):ENSP00000379394(p.Gly1908Ser)]	NA	NA	NA	NA	NA	NA	NA	T	BN	NA	MODERATE	D	0.997	104	104	0	87	36	51	58.62
Non-user	IOB_24	chr11	20939655	T	C	NELL1	NCT	ENSG00000165973:[ENST00000529595(n.492-73T>C)]	NA	NA	4.41E-02	NA	NA	4.41E-02	3.37E-02	NA	NA	NA	MODIFIER	NA	NA	144	144	0	102	0	100	98.04
Non-user	IOB_24	chr11	20959394	C	T	NELL1	MISSENSE	ENSG00000165973:[ENST00000357134(c.1060C>T,Cgg>Tgg):ENSP00000349654(p.Arg354Trp)]	NA	NA	3.81E-02	4.56E-02	3.15E-02	3.81E-02	3.07E-02	T	BN	NA	MODERATE	D	1	198	197	1	147	0	147	100
Non-user	IOB_24	chr11	26584776	A	C	ANO3	EXONIC-NC	ENSG00000134343:[ENST00000529242(n.607A>C)]	NA	NA	NA	NA	NA	NA	NA	NA	NA	NA	HIGH	NA	1	152	152	0	143	71	72	50.35
Non-user	IOB_24	chr11	26584776	A	C	MUC15	NONSENSE	ENSG00000169550:[ENST00000436318(c.812T>G,tTa>tGa):ENSP00000416753(p.Leu271Ter)]	NA	NA	NA	NA	NA	NA	NA	NA	NA	NA	HIGH	NA	1	152	152	0	143	71	72	50.35
Non-user	IOB_24	chr11	30034238	C	T	KCNA4	5UTR	ENSG00000182255:[ENST00000328224(c.-13G>A)]	NA	NA	NA	9.23E-06	1.02E-04	NA	NA	NA	NA	NA	MODIFIER	NA	NA	94	94	0	73	33	40	54.79
Non-user	IOB_24	chr11	34673237	G	T	EHF	INTRONIC	ENSG00000135373:[ENST00000450654(c.475+80G>T)]	NA	NA	chr11*34673237*34673237*G*A*rs767930242*NA*NA*NA	NA	NA	NA	NA	NA	NA	NA	MODIFIER	NA	NA	115	115	0	109	55	54	49.54
Non-user	IOB_24	chr11	36057669	C	A	LDLRAD3	SILENT	ENSG00000179241:[ENST00000315571(c.63C>A,ccC>ccA):ENSP00000318607(p.Pro21(=))]	NA	NA	3.99E-04	8.83E-04	2.43E-03	3.99E-04	0.00E+00	NA	NA	NA	MODIFIER	NA	NA	314	313	0	232	129	103	44.4
Non-user	IOB_24	chr11	36680765	T	C	C11orf74	3UTR	ENSG00000166352:[ENST00000334307(c.*29T>C)]	NA	NA	1.44E-02	3.10E-02	9.86E-03	1.44E-02	5.10E-03	NA	NA	NA	MODIFIER	NA	NA	123	123	0	101	48	53	52.48
Non-user	IOB_24	chr11	45245778	G	T	CTD-2560E9.3	NCT	ENSG00000254664:[ENST00000527450(n.179-8024C>A)]	NA	chr11*45245778*45245778*COSM1353848*G*T*2*haematopoietic_and_lymphoid_tissue,large_intestine*1,1	4.55E-02	8.53E-02	7.82E-02	4.55E-02	4.29E-02	NA	NA	NA	MODIFIER	NA	NA	59	59	0	42	23	18	42.86
Non-user	IOB_24	chr11	45245778	G	T	PRDM11	MISSENSE	ENSG00000019485:[ENST00000263765(c.855G>T,agG>agT):ENSP00000263765(p.Arg285Ser)]	NA	chr11*45245778*45245778*COSM1353848*G*T*2*haematopoietic_and_lymphoid_tissue,large_intestine*1,1	4.55E-02	8.53E-02	7.82E-02	4.55E-02	4.29E-02	NA	NA	NA	MODIFIER	NA	NA	59	59	0	42	23	18	42.86
Non-user	IOB_24	chr11	47652048	A	G	MTCH2	INTRONIC	ENSG00000109919:[ENST00000542981(c.95+59T>C)]	NA	NA	chr11*47652048*47652048*A*C*rs771171263*NA*NA*NA	NA	NA	NA	NA	NA	NA	NA	MODIFIER	NA	NA	140	140	0	103	47	56	54.37
Non-user	IOB_24	chr11	48347082	A	G	OR4C3	MISSENSE	ENSG00000176547:[ENST00000319856(c.590A>G,aAt>aGt):ENSP00000321419(p.Asn197Ser)]	NA	NA	NA	3.02E-02	2.88E-02	NA	NA	D	BN	NA	MODERATE	D	0.005	467	457	0	325	257	63	19.38
Non-user	IOB_24	chr11	49075621	G	A	TRIM64C	MISSENSE	ENSG00000214891:[ENST00000530230(c.989C>T,gCg>gTg):ENSP00000431987(p.Ala330Val)]	NA	NA	2.14E-02	3.93E-02	3.59E-02	2.14E-02	3.07E-02	T	BN	NA	MODERATE	NA	NA	87	86	0	64	25	39	60.94
Non-user	IOB_24	chr11	49138871	T	G	RP11-107P7.5	EXONIC-NC	ENSG00000254993:[ENST00000524985(n.819A>C)]	NA	NA	2.14E-02	NA	NA	2.14E-02	3.07E-02	NA	NA	NA	MODIFIER	NA	NA	241	239	0	167	82	84	50.3
Non-user	IOB_24	chr11	49194890	T	A	FOLH1	INTRONIC	ENSG00000086205:[ENST00000343844(c.301+19A>T)]	NA	NA	2.04E-02	3.76E-02	3.59E-02	2.04E-02	3.07E-02	NA	NA	NA	MODIFIER	NA	NA	61	60	0	48	25	23	47.92
Non-user	IOB_24	chr11	49455021	C	A	NA	INTERGENIC	NA	NA	NA	2.44E-02	NA	NA	2.44E-02	3.27E-02	NA	NA	NA	MODIFIER	NA	NA	63	58	0	57	31	22	38.6
Non-user	IOB_24	chr11	49598257	G	T	LOC440040	EXONIC-NC	440040:[NR_027044.1(n.848G>T)]	NA	NA	2.42E-02	4.78E-02	3.59E-02	2.42E-02	3.27E-02	NA	NA	NA	MODIFIER	NA	NA	300	300	0	217	119	98	45.16
Non-user	IOB_24	chr11	49598257	G	T	RP11-707M1.1	EXONIC-NC	ENSG00000205035:[ENST00000534201(n.830G>T)]	NA	NA	2.42E-02	4.78E-02	3.59E-02	2.42E-02	3.27E-02	NA	NA	NA	MODIFIER	NA	NA	300	300	0	217	119	98	45.16
Non-user	IOB_24	chr11	49829184	C	T	LOC440040	NCT	440040:[NR_027044.1(n.1385-23C>T)]	NA	NA	2.32E-02	4.21E-02	3.68E-02	2.32E-02	3.17E-02	NA	NA	NA	MODIFIER	NA	NA	202	201	0	135	67	67	49.63
Non-user	IOB_24	chr11	49829184	C	T	RP11-707M1.1	NCT	ENSG00000205035:[ENST00000527477(n.1398-23C>T)]	NA	NA	2.32E-02	4.21E-02	3.68E-02	2.32E-02	3.17E-02	NA	NA	NA	MODIFIER	NA	NA	202	201	0	135	67	67	49.63
Non-user	IOB_24	chr11	51411914	T	C	OR4A5	MISSENSE	ENSG00000221840:[ENST00000319760(c.482A>G,tAc>tGc):ENSP00000367664(p.Tyr161Cys)]	NA	NA	4.29E-02	5.01E-02	3.60E-02	4.29E-02	3.27E-02	D_lc	BN	NA	MODERATE	NA	NA	189	188	0	134	64	69	51.49
Non-user	IOB_24	chr11	51515546	G	A	OR4C46	MISSENSE	ENSG00000185926:[ENST00000328188(c.265G>A,Gcc>Acc):ENSP00000329056(p.Ala89Thr)]	NA	NA	4.31E-02	5.01E-02	3.60E-02	4.31E-02	3.27E-02	T_lc	BN	NA	MODERATE	NA	NA	433	433	0	297	169	125	42.09
Non-user	IOB_24	chr11	51515596	C	T	OR4C46	SILENT	ENSG00000185926:[ENST00000328188(c.315C>T,ttC>ttT):ENSP00000329056(p.Phe105(=))]	NA	NA	2.34E-02	4.60E-02	3.60E-02	2.34E-02	3.27E-02	NA	NA	NA	LOW	NA	NA	395	393	1	278	151	126	45.32
Non-user	IOB_24	chr11	51515830	C	G	OR4C46	SILENT	ENSG00000185926:[ENST00000328188(c.549C>G,ctC>ctG):ENSP00000329056(p.Leu183(=))]	NA	NA	4.19E-02	4.98E-02	3.60E-02	4.19E-02	3.27E-02	NA	NA	NA	LOW	NA	NA	418	417	0	254	145	106	41.73
Non-user	IOB_24	chr11	51515834	C	A	OR4C46	MISSENSE	ENSG00000185926:[ENST00000328188(c.553C>A,Ctc>Atc):ENSP00000329056(p.Leu185Ile)]	NA	NA	2.34E-02	4.59E-02	3.59E-02	2.34E-02	3.27E-02	D_lc	PrD	NA	MODERATE	NA	NA	431	429	0	255	144	108	42.35
Non-user	IOB_24	chr11	51527695	T	C	OR4C50P	EXONIC-NC	ENSG00000237610:[ENST00000330155(n.106A>G)]	NA	NA	4.19E-02	NA	NA	4.19E-02	3.27E-02	NA	NA	NA	MODIFIER	NA	NA	75	75	0	50	20	30	60

Patel *et al.* , 2021. Whole-exome sequencing analysis of oral squamous cell carcinoma delineated by tobacco usage habits
Supplementary Table 4. List of somatic single nucleotide variants identified using whole exome sequencing of OSCC patients

Cohort	Sample	Chromosome	Genomic position	Reference allele	Alternate allele	Genesymbol	Variant type	Variant description	OncoMD	COSMIC	dbSNP	ExAC	ExAC_SAS	1000G	1000G_SAS	SIFT	Polyphen2	CONDEL	VEP	LRT	PhastCon7Way	Total reads in Normal	Reference read support in Normal	Alternative read support in Normal	Total reads in Tumor	Reference read support in Tumor	Alternate read support in Tumor	Alternate allele frequency in Tumor
Non-user	IOB_24	chr11	55110648	T	A	OR4A16	5UTR	ENSG00000181961:[ENST00000314721(c.-29T>A)]	NA	NA	NA	NA	NA	NA	NA	NA	NA	NA	MODIFIER	NA	NA	272	272	0	230	187	39	16.96
Non-user	IOB_24	chr11	55110690	G	A	OR4A16	MISSENSE	ENSG00000181961:[ENST00000314721(c.14G>A,aGc>aAc):ENSP00000325128(p.Ser5Asn)]	NA	NA	NA	2.00E-04	3.45E-04	NA	NA	T_lc	BN	NA	MODERATE	NA	NA	296	293	1	262	213	41	15.65
Non-user	IOB_24	chr11	55110715	G	A	OR4A16	SILENT	ENSG00000181961:[ENST00000314721(c.39G>A,ctG>ctA):ENSP00000325128(p.Leu13(=))]	NA	chr11*55110715*55110715*COSM330237*G*A*1*haematopoietic_and_lymphoid_tissue*1	NA	1.36E-04	4.63E-04	NA	NA	NA	NA	NA	LOW	NA	NA	278	275	0	262	209	40	15.27
Non-user	IOB_24	chr11	55110843	T	C	OR4A16	MISSENSE	ENSG00000181961:[ENST00000314721(c.167T>C,cTa>cCa):ENSP00000325128(p.Leu56Pro)]	NA	NA	NA	1.84E-03	2.13E-03	NA	NA	T_lc	BN	NA	MODERATE	NA	NA	349	346	0	338	282	42	12.43
Non-user	IOB_24	chr11	55110861	C	A	OR4A16	MISSENSE	ENSG00000181961:[ENST00000314721(c.185C>A,gCc>gAc):ENSP00000325128(p.Ala62Asp)]	NA	NA	NA	1.75E-03	2.07E-03	NA	NA	D_lc	BN	NA	MODERATE	NA	NA	380	377	0	389	324	15	3.86
Non-user	IOB_24	chr11	55110865	C	T	OR4A16	SILENT	ENSG00000181961:[ENST00000314721(c.189C>T,taC>taT):ENSP00000325128(p.Tyr63(=))]	NA	NA	NA	1.81E-03	2.13E-03	NA	NA	NA	NA	NA	LOW	NA	NA	400	397	1	408	336	21	5.15
Non-user	IOB_24	chr11	55110877	G	A	OR4A16	MISSENSE	ENSG00000181961:[ENST00000314721(c.201G>A,atG>atA):ENSP00000325128(p.Met67Ile)]	NA	NA	4.99E-03	2.22E-03	2.44E-03	4.99E-03	2.00E-03	T_lc	BN	NA	MODERATE	NA	NA	427	424	0	444	360	80	18.02
Non-user	IOB_24	chr11	55110903	C	A	OR4A16	NONSENSE	ENSG00000181961:[ENST00000314721(c.227C>A,tCa>tAa):ENSP00000325128(p.Ser76Ter)]	NA	NA	6.99E-03	2.41E-02	1.91E-02	6.99E-03	8.20E-03	NA	NA	NA	HIGH	NA	NA	455	451	0	482	381	93	19.29
Non-user	IOB_24	chr11	55110918	T	C	OR4A16	MISSENSE	ENSG00000181961:[ENST00000314721(c.242T>C,aTa>aCa):ENSP00000325128(p.Ile81Thr)]	NA	NA	8.79E-03	2.89E-02	2.59E-02	8.79E-03	1.12E-02	T_lc	BN	NA	MODERATE	NA	NA	465	465	0	490	390	8	1.63
Non-user	IOB_24	chr11	55110941	G	A	OR4A16	MISSENSE	ENSG00000181961:[ENST00000314721(c.265G>A,Get>Act):ENSP00000325128(p.Ala89Thr)]	NA	NA	NA	2.92E-02	2.58E-02	NA	NA	T_lc	BN	NA	MODERATE	NA	NA	451	450	0	490	383	11	2.24
Non-user	IOB_24	chr11	55111115	A	G	OR4A16	MISSENSE	ENSG00000181961:[ENST00000314721(c.439A>G,Atg>Gtg):ENSP00000325128(p.Met147Val)]	NA	NA	1.90E-02	4.09E-02	2.96E-02	1.90E-02	1.23E-02	T_lc	BN	NA	MODERATE	NA	NA	154	152	0	124	87	37	29.84
Non-user	IOB_24	chr11	55111118	A	G	OR4A16	MISSENSE	ENSG00000181961:[ENST00000314721(c.442A>G,Att>Gtt):ENSP00000325128(p.Ile148Val)]	NA	NA	1.90E-02	4.08E-02	2.92E-02	1.90E-02	1.23E-02	T_lc	BN	NA	MODERATE	NA	NA	149	149	0	122	86	36	29.51
Non-user	IOB_24	chr11	55111157	C	G	OR4A16	MISSENSE	ENSG00000181961:[ENST00000314721(c.481C>G,Ctg>Gtg):ENSP00000325128(p.Leu161Val)]	NA	NA	NA	6.93E-03	1.20E-02	NA	NA	T_lc	BN	NA	MODERATE	NA	NA	140	139	0	114	74	40	35.09
Non-user	IOB_24	chr11	55111172	A	T	OR4A16	MISSENSE	ENSG00000181961:[ENST00000314721(c.496A>T,Atc>Ttc):ENSP00000325128(p.Ile166Phe)]	NA	NA	chr11*55111172*55111172*A*G,T*rs80108485*NA*NA*NA	5.48E-03	9.35E-03	NA	NA	T_lc	BN	NA	MODERATE	NA	NA	138	138	0	113	75	37	32.74
Non-user	IOB_24	chr11	55111189	T	C	OR4A16	SILENT	ENSG00000181961:[ENST00000314721(c.513T>C,gtT>gtC):ENSP00000325128(p.Val171(=))]	NA	NA	NA	4.68E-03	8.08E-03	NA	NA	NA	NA	NA	LOW	NA	NA	122	121	0	107	69	36	33.64
Non-user	IOB_24	chr11	55111200	C	T	OR4A16	MISSENSE	ENSG00000181961:[ENST00000314721(c.524C>T,tCt>tTt):ENSP00000325128(p.Ser175Phe)]	NA	NA	NA	3.44E-03	5.58E-03	NA	NA	T_lc	BN	NA	MODERATE	NA	NA	118	117	0	100	62	34	34
Non-user	IOB_24	chr11	55111438	T	C	OR4A16	SILENT	ENSG00000181961:[ENST00000314721(c.762T>C,ttT>ttC):ENSP00000325128(p.Phe254(=))]	NA	NA	NA	3.98E-04	0.00E+00	NA	NA	NA	NA	NA	LOW	NA	NA	65	65	0	47	40	7	14.89
Non-user	IOB_24	chr11	55111453	C	T	OR4A16	SILENT	ENSG00000181961:[ENST00000314721(c.777C>T,ccC>ccT):ENSP00000325128(p.Pro259(=))]	NA	NA	2.60E-03	7.45E-05	6.06E-05	2.60E-03	3.10E-03	NA	NA	NA	LOW	NA	NA	65	65	0	48	42	6	12.5
Non-user	IOB_24	chr11	55111480	A	C	OR4A16	MISSENSE	ENSG00000181961:[ENST00000314721(c.804A>C,ttA>ttC):ENSP00000325128(p.Leu268Phe)]	NA	NA	NA	1.40E-03	1.21E-04	NA	NA	T_lc	BN	NA	MODERATE	NA	NA	67	67	0	55	49	6	10.91
Non-user	IOB_24	chr11	55111496	T	A	OR4A16	MISSENSE	ENSG00000181961:[ENST00000314721(c.820T>A,Tca>Aca):ENSP00000325128(p.Ser274Thr)]	NA	NA	9.78E-03	1.00E-03	6.06E-05	9.78E-03	2.00E-03	T_lc	BN	NA	MODERATE	NA	NA	69	69	0	55	49	5	9.09
Non-user	IOB_24	chr11	55111509	T	C	OR4A16	MISSENSE	ENSG00000181961:[ENST00000314721(c.833T>C,cTc>cCc):ENSP00000325128(p.Leu278Pro)]	NA	NA	NA	4.57E-04	6.06E-05	NA	NA	T_lc	BN	NA	MODERATE	NA	NA	68	68	0	60	53	5	8.33
Non-user	IOB_24	chr11	55136219	C	T	OR4A15	MISSENSE	ENSG00000181958:[ENST00000314706(c.860C>T,gCa>gTa):ENSP00000325065(p.Ala287Val)]	NA	NA	2.40E-02	4.55E-02	3.81E-02	2.40E-02	3.89E-02	T_lc	BN	NA	MODERATE	N	0	382	382	0	279	145	134	48.03
Non-user	IOB_24	chr11	55321823	T	A	OR4C15	MISSENSE	ENSG00000181939:[ENST00000314644(c.41T>A,cTt>cAt):ENSP00000324958(p.Leu14His)]	NA	NA	2.40E-02	4.63E-02	4.14E-02	2.40E-02	3.89E-02	D_lc	BN	NA	MODERATE	NA	NA	151	151	0	114	60	53	46.49
Non-user	IOB_24	chr11	55322099	C	A	OR4C15	MISSENSE	ENSG00000181939:[ENST00000314644(c.317C>A,gCt>gAt):ENSP00000324958(p.Ala106Asp)]	NA	NA	2.40E-02	4.56E-02	3.80E-02	2.40E-02	3.89E-02	D_lc	PoD	NA	MODERATE	NA	NA	287	286	1	199	112	86	43.22
Non-user	IOB_24	chr11	55322422	C	A	OR4C15	MISSENSE	ENSG00000181939:[ENST00000314644(c.640C>A,Ctt>Att):ENSP00000324958(p.Leu214Ile)]	NA	NA	2.40E-02	4.56E-02	3.80E-02	2.40E-02	3.89E-02	T_lc	BN	NA	MODERATE	NA	NA	348	348	0	236	133	103	43.64
Non-user	IOB_24	chr11	55322464	A	C	OR4C15	MISSENSE	ENSG00000181939:[ENST00000314644(c.682A>C,Aat>Cat):ENSP00000324958(p.Asn228His)]	NA	NA	2.40E-02	4.55E-02	3.81E-02	2.40E-02	3.89E-02	D_lc	PoD	NA	MODERATE	NA	NA	417	415	0	297	170	126	42.42
Non-user	IOB_24	chr11	55322490	G	A	OR4C15	SILENT	ENSG00000181939:[ENST00000314644(c.708G>A,ccG>ccA):ENSP00000324958(p.Pro236(=))]	NA	NA	2.42E-02	4.58E-02	3.90E-02	2.42E-02	3.89E-02	NA	NA	NA	LOW	NA	NA	432	428	1	306	172	131	42.81
Non-user	IOB_24	chr11	55322606	T	C	OR4C15	MISSENSE	ENSG00000181939:[ENST00000314644(c.824T>C,tTg>tCg):ENSP00000324958(p.Leu275Ser)]	NA	NA	2.40E-02	4.56E-02	3.80E-02	2.40E-02	3.89E-02	D_lc	PrD	NA	MODERATE	NA	NA	312	312	0	199	104	95	47.74
Non-user	IOB_24	chr11	55322902	C	A	NA	INTERGENIC	NA	NA	NA	2.40E-02	5.63E-02	5.15E-02	2.40E-02	3.89E-02	NA	NA	NA	MODIFIER	NA	NA	52	52	0	28	11	17	60.71
Non-user	IOB_24	chr11	55322921	G	A	NA	INTERGENIC	NA	NA	NA	2.40E-02	5.53E-02	4.90E-02	2.40E-02	3.89E-02	NA	NA	NA	MODIFIER	NA	NA	41	41	0	21	9	12	57.14
Non-user	IOB_24	chr11	55339704	T	G	OR4C16	MISSENSE	ENSG00000181935:[ENST00000314634(c.101T>G,tTg>tGg):ENSP00000324913(p.Leu34Trp)]	NA	NA	2.40E-02	4.54E-02	3.79E-02	2.40E-02	3.89E-02	D	PrD	NA	MODERATE	NA	0.002	356	354	0	290	143	145	50
Non-user	IOB_24	chr11	55339712	C	T	OR4C16	SILENT	ENSG00000181935:[ENST00000314634(c.109C>T,Ctg>Ttg):ENSP00000324913(p.Leu37(=))]	NA	NA	2.40E-02	4.56E-02	3.79E-02	2.40E-02	3.89E-02	NA	NA	NA	LOW	NA	0	358	354	1	295	148	146	49.49
Non-user	IOB_24	chr11	55339740	G	C	OR4C16	MISSENSE	ENSG00000181935:[ENST00000314634(c.137G>C,aGt>aCt):ENSP00000324913(p.Ser46Thr)]	NA	NA	2.40E-02	4.55E-02	3.80E-02	2.40E-02	3.89E-02	T	BN	NA	MODERATE	NA	0	399	394	1	334	162	172	51.5
Non-user	IOB_24	chr11	55340170	A	C	OR4C16	MISSENSE	ENSG00000181935:[ENST00000314634(c.567A>C,gaA>gaC):ENSP00000324913(p.Glu189Asp)]	NA	NA	2.40E-02	4.56E-02	3.82E-02	2.40E-02	3.89E-02	T	BN	NA	MODERATE	NA	0	433	430	1	297	150	146	49.16
Non-user	IOB_24	chr11	55340466	A	G	OR4C16	MISSENSE	ENSG00000181935:[ENST00000314634(c.863A>G,aAg>aGg):ENSP00000324913(p.Lys288Arg)]	NA	NA	2.40E-02	4.76E-02	4.43E-02	2.40E-02	3.89E-02	T	BN	NA	MODERATE	NA	0.855	218	218	0	128	61	67	52.34
Non-user	IOB_24	chr11	55371132	A	T	OR4C11	MISSENSE	ENSG00000172188:[ENST00000302231(c.718T>A,Tct>Act):ENSP00000306651(p.Ser240Thr)]	NA	NA	2.96E-02	6.31E-02	5.02E-02	2.96E-02	4.60E-02	D	BN	NA	MODERATE	U	0.695	154	153	0	114	0	113	99.12
Non-user	IOB_24	chr11	55406524	C	T	OR4P4	MISSENSE	ENSG00000181927:[ENST00000314612(c.691C>T,Cgc>Tgc):ENSP00000324831(p.Arg231Cys)]	NA	NA	2.98E-02	6.27E-02	5.08E-02	2.98E-02	4.60E-02	T	BN	NA	MODERATE	N	0	198	198	0	129	3	125	96.9
Non-user	IOB_24	chr11	55418963	T	G	OR4S2	MISSENSE	ENSG00000174982:[ENST00000312422(c.584T>G,gTt>gGt):ENSP00000310337(p.Val195Gly)]	NA	NA	2.96E-02	6.35E-02	5.04E-02	2.96E-02	4.60E-02	T	BN	NA	MODERATE	N	0.016	218	212	1	163	1	162	99.39
Non-user	IOB_24	chr11	55541620	G	T	OR5D13	MISSENSE	ENSG00000198877:[ENST00000361760(c.707G>T,cGc>cTc):ENSP00000354800(p.Arg236Leu)]	NA	chr11*55541620*55541620*COSM218331*G*A*3*pancreas,lung*2,1	2.46E-02	4.95E-02	3.79E-02	2.46E-02	3.89E-02	T	BN	NA	MODERATE	NA	0	261	259	1	196	107	88	44.9
Non-user	IOB_24	chr11	55563761	G	A	OR5D14	MISSENSE	ENSG00000186113:[ENST00000335605(c.730G>A,Gcc>Acc):ENSP00000334456(p.Ala244Thr)]	NA	NA	2.46E-02	4.94E-02	3.79E-02	2.46E-02	3.89E-02	T	BN	NA	MODERATE	N	0	263	260	0	187	106	80	42.78
Non-user	IOB_24	chr11	55563846	C	A	OR5D14	MISSENSE	ENSG00000186113:[ENST00000335605(c.815C>A,aCa>aAa):ENSP00000334456(p.Thr272Lys)]	NA	NA	2.46E-02	4.95E-02	3.79E-02	2.46E-02	3.89E-02	T	BN	NA	MODERATE	N	0	261	261	0	208	120	87	41.83
Non-user	IOB_24	chr11	55587212	A	G	OR5D18	MISSENSE	ENSG00000186119:[ENST00000333976(c.107A>G,tAc>tGc):ENSP00000335025(p.Tyr36Cys)]	NA	NA	2.46E-02	4.95E-02	3.79E-02	2.46E-02	3.89E-02	D	PrD	NA	MODERATE	N	0.025	279	279	0	200	89	109	54.5
Non-user	IOB_24	chr11	55595116	G	A	OR5L2	MISSENSE	ENSG00000205030:[ENST00000378397(c.422G>A,cGt>cAt):ENSP00000367650(p.Arg141His)]	NA	NA	4.79E-03	5.32E-03	3.03E-04	4.79E-03	0.00E+00	D	BN	NA	MODERATE	N	0.034	369	368	0	210	102	106	50.48
Non-user	IOB_24	chr11	55681149	G	C	OR5W2	MISSENSE	ENSG00000187612:[ENST00000344514(c.910C>G,Ctg>Gtg):ENSP00000342448(p.Leu304Val)]	NA	NA	3.22E-02	6.74E-02	4.22E-02	3.21E-02	4.19E-02	T	BN	NA	MODERATE	N	0	58	58	0	33	13	20	60.61
Non-user	IOB_24	chr11	55681375	T	G	OR5W2	SILENT	ENSG00000187612:[ENST00000344514(c.684A>C,atA>atC):ENSP00000342448(p.Ile228(=))]	NA	NA	3.22E-02	6.28E-02	3.95E-02	3.21E-02	4.19E-02	NA	NA	NA	LOW	NA	0	140	139	0	75	43	32	42.67
Non-user	IOB_24	chr11	55681416	A	G	OR5W2	MISSENSE	ENSG00000187612:[ENST00000344514(c.643T>C,Ttc>Ctc):ENSP00000342448(p.Phe215Leu)]	NA	NA	3.30E-02	6.28E-02	3.95E-02	3.29E-02	4.19E-02	T	BN	NA	MODERATE	N	0	129	129	0	70	44	26	37.14
Non-user	IOB_24	chr11	55681580	A	G	OR5W2	MISSENSE	ENSG00000187612:[ENST00000344514(c.479T>C,aTg>aCg):ENSP00000342448(p.Met160Thr)]	NA	NA	3.22E-02	6.29E-02	3.95E-02	3.21E-02	4.19E-02	T	BN	NA	MODERATE	N	0	175	175	0	132	63	69	52.27
Non-user	IOB_24	chr11	55681942	G	T	OR5W2	MISSENSE	ENSG00000187612:[ENST00000344514(c.117C>A,ttC>ttA):ENSP00000342448(p.Phe39Leu)]	NA	NA	3.22E-02	6.27E-02	3.94E-02	3.21E-02	4.19E-02	T	BN	NA	MODERATE	N	0	128	128	0	99	41	58	58.59
Non-user	IOB_24	chr11	55702961	C	T	OR5I1	MISSENSE	ENSG00000167825:[ENST00000301532(c.916G>A,Gtt>Att):ENSP00000301532(p.Val306Ile)]	NA	NA	3.42E-02	6.57E-02	5.22E-02	3.41E-02	4.19E-02	T	BN	NA	MODERATE	N	0	27	27	0	26	14	12	46.15
Non-user	IOB_24	chr11	55703010	C	A	OR5I1	SILENT	ENSG00000167825:[ENST00000301532(c.867G>T,ccG>ccT):ENSP00000301532(p.Pro289(=))]	NA	chr11*55703010*55703010*COSM928165*C*T*1*endometrium*1	3.20E-02	6.25E-02	4.00E-02	3.19E-02	4.19E-02	NA	NA	NA	LOW	NA	NA	50	50	0	38	20	18	47.37
Non-user	IOB_24	chr11	55703650	A	G	OR5I1	MISSENSE	ENSG00000167825:[ENST00000301532(c.227T>C,tTc>tCc):ENSP00000301532(p.Phe76Ser)]	NA	NA	3.49E-02	6.38E-02	3.96E-02	3.49E-02	4.19E-02	T	BN	NA	MODERATE	N	0	337	336	0	245	125	119	48.57
Non-user	IOB_24	chr11	55703766	A	G	OR5I1	SILENT	ENSG00000167825:[ENST00000301532(c.111T>C,taT>taC):ENSP00000301532(p.Tyr37(=))]	NA	NA	3.49E-02	6.38E-02	3.97E-02	3.49E-02	4.19E-02	NA	NA	NA	LOW	NA	0	355	354	0	239	131	108	45.19
Non-user	IOB_24	chr11	55703861	T	C	OR5I1	MISSENSE	ENSG00000167825:[ENST00000301532(c.16A>G,Aga>Gga):ENSP00000301532(p.Arg6Gly)]	NA	chr11*55703861*55703861*COSM298753*T*G*1*large_intestine*1	3.55E-02	7.10E-02	4.87E-02	3.55E-02	4.50E-02	T	BN	NA	MODERATE	N	0	207	206	0	147	78	69	46.94
Non-user	IOB_24	chr11	55735106	A	G	OR10AG1	SILENT	ENSG00000174970:[ENST00000312345(c.834T>C,ccT>ccC):ENSP00000311477(p.Pro278(=))]	NA	NA	3.81E-02	6.56E-02	5.05E-02	3.81E-02	5.32E-02	NA	NA	NA	LOW	NA	NA	104	104	0	85	35	50	58.82
Non-user	IOB_24	chr11	55735280	T	A	OR10AG1	SILENT	ENSG00000174970:[ENST00000312345(c.660A>T,tcA>tcT):ENSP00000311477(p.Ser220(=))]	NA	NA	3.81E-02	6.54E-02	5.04E-02	3.81E-02	5.32E-02	NA	NA	NA	LOW	NA	NA	307	306	1	192	97	95	49.48
Non-user	IOB_24	chr11	55735518	G	A	OR10AG1	MISSENSE	ENSG00000174970:[ENST00000312345(c.422C>T,aCc>aTc):ENSP00000311477(p.Thr141Ile)]	NA	NA	3.81E-02	6.54E-02	5.04E-02	3.81E-02	5.32E-02	T	BN	NA	MODERATE	N	0	364	364	0	301	163	137	45.51
Non-user	IOB_24	chr11	55735579	A	G	OR10AG1	SILENT	ENSG00000174970:[ENST00000312345(c.361T>C,Ttg>Ctg):ENSP00000311477(p.Leu121(=))]	NA	NA	3.87E-02	6.55E-02	5.07E-02	3.87E-02	5.62E-02	NA	NA	NA	LOW	NA	0.002	310	310	0	250	140	110	44
Non-user	IOB_24	chr11	55735649	C	A	OR10AG1	MISSENSE	ENSG00000174970:[ENST00000312345(c.291G>T,atG>atT):ENSP00000311477(p.Met97Ile)]	NA	NA	3.81E-02	6.55E-02	5.04E-02	3.81E-02	5.32E-02	T	BN	NA	MODERATE	N	0	232	232	0	186	99	86	46.24
Non-user	IOB_24	chr11	55735808	G	A	OR10AG1	SILENT	ENSG00000174970:[ENST00000312345(c.132C>T,ccC>ccT):ENSP00000311477(p.Pro44(=))]	NA	NA	3.83E-02	6.60E-02	5.07E-02	3.83E-02	5.32E-02	NA	NA	NA	LOW	NA	NA	65	64	0	55	19	36	65.45
Non-user	IOB_24	chr11	55735813	G	C	OR10AG1	MISSENSE	ENSG00000174970:[ENST00000312345(c.127C>G,Cac>Gac):ENSP00000311477(p.His43Asp)]	NA	NA	3.81E-02	6.58E-02	5.04E-02	3.81E-02	5.32E-02	T	BN	NA	MODERATE	N	0	67	66	0	46	15	31	67.39
Non-user	IOB_24	chr11	55735848	A	G	OR10AG1	MISSENSE	ENSG00000174970:[ENST00000312345(c.92T>C,aTg>aCg):ENSP00000311477(p.Met31Thr)]	NA	NA	3.83E-02	6.60E-02	5.04E-02	3.83E-02	5.32E-02	T	BN	NA	MODERATE	N	0	51	50	0	30	7	23	76.67
Non-user	IOB_24	chr11	55735857	A	G	OR10AG1	MISSENSE	ENSG00000174970:[ENST00000312345(c.83T>C,aTg>aCg):ENSP00000311477(p.Met28Thr)]	NA	NA	3.81E-02	6.61E-02	5.05E-02	3.81E-02	5.32E-02	T	BN	NA	MODERATE	N	0	49	48	0	29	6	23	79.31
Non-user	IOB_24	chr11	55747013	C	T	OR7E5P	EXONIC-NC	ENSG00000214880:[ENST00000529916(n.567G>A)]	NA	NA	3.79E-02	6.47E-02	4.99E-02	3.79E-02	5.32E-02	NA	NA	NA	MODIFIER	NA	NA	122	120	2	93	42	51	54.84
Non-user	IOB_24	chr11	55747136	G	C	OR7E5P	EXONIC-NC	ENSG00000214880:[ENST00000529916(n.444C>G)]	NA	NA	3.81E-02	1.22E-01	5.93E-02	3.81E-02	5.32E-02	NA	NA	NA	MODIFIER	NA	NA	115	114	0	105	53	51	48.57
Non-user	IOB_24	chr11	55761256	A	G	OR5F1	SILENT	ENSG00000149133:[ENST00000278409(c.846T>C,atT>atC):ENSP00000278409(p.Ile282(=))]	NA	NA	3.81E-02	6.55E-02	5.03E-02	3.81E-02	5.32E-02	NA	NA	NA	LOW	NA	NA	67	66	0	65	34	31	47.69

Patel *et al.* , 2021. Whole-exome sequencing analysis of oral squamous cell carcinoma delineated by tobacco usage habits
Supplementary Table 4. List of somatic single nucleotide variants identified using whole exome sequencing of OSCC patients

Cohort	Sample	Chromosome	Genomic position	Reference allele	Alternate allele	Genesymbol	Variant type	Variant description	OncoMD	COSMIC	dbSNP	ExAC	ExAC_SAS	1000G	1000G_SAS	SIFT	Polyphen2	CONDEL	VEP	LRT	PhastCon7Way	Total reads in Normal	Reference read support in Normal	Alternative read support in Normal	Total reads in Tumor	Reference read support in Tumor	Alternate read support in Tumor	Alternate allele frequency in Tumor
Non-user	IOB_24	chr11	55761281	G	A	OR5F1	MISSENSE	ENSG00000149133:[ENST00000278409(c.821C>T,gCt>gTt):ENSP00000278409(p.Ala274Val)]	NA	NA	3.81E-02	6.55E-02	5.05E-02	3.81E-02	5.32E-02	T	BN	NA	MODERATE	NA	NA	75	75	0	68	37	31	45.59
Non-user	IOB_24	chr11	55798237	C	T	OR5AS1	SILENT	ENSG00000181785:[ENST00000313555(c.343C>T,Ctg>Ttg):ENSP00000324111(p.Leu115(=))]	NA	NA	3.83E-02	6.56E-02	5.04E-02	3.83E-02	5.32E-02	NA	NA	NA	LOW	NA	0.005	374	370	0	297	150	147	49.49
Non-user	IOB_24	chr11	55798533	A	C	OR5AS1	SILENT	ENSG00000181785:[ENST00000313555(c.639A>C,gtA>gtC):ENSP00000324111(p.Val213(=))]	NA	chr11*55798533*55798533*COSM928222*A*G*1*endometrium*1	3.83E-02	6.56E-02	5.03E-02	3.83E-02	5.32E-02	NA	NA	NA	LOW	NA	NA	347	346	0	256	118	138	53.91
Non-user	IOB_24	chr11	55798761	T	C	OR5AS1	SILENT	ENSG00000181785:[ENST00000313555(c.867T>C,atT>atC):ENSP00000324111(p.Ile289(=))]	NA	chr11*55798761*55798761*COSM928224*T*G*1*endometrium*1	3.83E-02	6.59E-02	5.14E-02	3.83E-02	5.32E-02	NA	NA	NA	LOW	NA	0	65	65	0	53	30	23	43.4
Non-user	IOB_24	chr11	55798764	T	C	OR5AS1	SILENT	ENSG00000181785:[ENST00000313555(c.870T>C,taT>taC):ENSP00000324111(p.Tyr290(=))]	NA	NA	3.83E-02	6.59E-02	5.17E-02	3.83E-02	5.32E-02	NA	NA	NA	LOW	NA	0.002	64	62	1	52	30	22	42.31
Non-user	IOB_24	chr11	55798823	A	G	OR5AS1	MISSENSE	ENSG00000181785:[ENST00000313555(c.929A>G,tAt>tGt):ENSP00000324111(p.Tyr310Cys)]	NA	chr11*55798823*55798823*COSM1162955*A*G*1*pancreas*1	3.83E-02	6.82E-02	5.75E-02	3.83E-02	5.32E-02	T	BN	NA	MODERATE	NA	NA	30	30	0	23	14	9	39.13
Non-user	IOB_24	chr11	55861153	A	G	OR8I2	MISSENSE	ENSG00000172154:[ENST00000302124(c.370A>G,Ata>Gta):ENSP00000303864(p.Ile124Val)]	NA	NA	3.89E-02	6.54E-02	5.02E-02	3.89E-02	5.32E-02	T	BN	NA	MODERATE	N	0	515	514	0	338	173	165	48.82
Non-user	IOB_24	chr11	55861297	A	G	OR8I2	MISSENSE	ENSG00000172154:[ENST00000302124(c.514A>G,Agc>Ggc):ENSP00000303864(p.Ser172Gly)]	NA	NA	3.63E-02	6.43E-02	5.01E-02	3.63E-02	5.32E-02	D	BN	NA	MODERATE	U	0	518	517	0	327	178	143	43.73
Non-user	IOB_24	chr11	55861484	A	C	OR8I2	MISSENSE	ENSG00000172154:[ENST00000302124(c.701A>C,cAg>cCg):ENSP00000303864(p.Gln234Pro)]	NA	NA	3.89E-02	6.55E-02	5.02E-02	3.89E-02	5.32E-02	D	BN	NA	MODERATE	U	0	186	186	0	112	53	58	51.79
Non-user	IOB_24	chr11	55872832	C	T	OR8H2	MISSENSE	ENSG00000181767:[ENST00000313503(c.314C>T,gCc>gTc):ENSP00000323982(p.Ala105Val)]	NA	NA	3.89E-02	6.54E-02	5.02E-02	3.89E-02	5.32E-02	T	BN	NA	MODERATE	N	0	458	457	1	331	184	146	44.11
Non-user	IOB_24	chr11	55872980	A	G	OR8H2	MISSENSE	ENSG00000181767:[ENST00000313503(c.462A>G,atA>atG):ENSP00000323982(p.Ile154Met)]	NA	NA	3.91E-02	6.54E-02	5.02E-02	3.91E-02	5.32E-02	T	BN	NA	MODERATE	N	0	548	548	0	409	245	160	39.12
Non-user	IOB_24	chr11	55872990	G	C	OR8H2	MISSENSE	ENSG00000181767:[ENST00000313503(c.472G>C,Gtc>Ctc):ENSP00000323982(p.Val158Leu)]	NA	NA	3.89E-02	6.54E-02	5.02E-02	3.89E-02	5.32E-02	T	BN	NA	MODERATE	N	0	582	580	2	415	249	164	39.52
Non-user	IOB_24	chr11	55873026	G	T	OR8H2	MISSENSE	ENSG00000181767:[ENST00000313503(c.508G>T,Gac>Tac):ENSP00000323982(p.Asp170Tyr)]	NA	NA	3.89E-02	6.54E-02	5.02E-02	3.89E-02	5.32E-02	D	BN	NA	MODERATE	N	0	626	626	0	440	266	173	39.32
Non-user	IOB_24	chr11	55873293	T	C	OR8H2	MISSENSE	ENSG00000181767:[ENST00000313503(c.775T>C,Tat>Cat):ENSP00000323982(p.Tyr259His)]	NA	NA	3.89E-02	6.55E-02	5.02E-02	3.89E-02	5.32E-02	D	BN	NA	MODERATE	N	1	187	186	0	121	55	66	54.55
Non-user	IOB_24	chr11	55890156	G	T	OR8H3	MISSENSE	ENSG00000181761:[ENST00000313472(c.308G>T,tGt>tTt):ENSP00000323928(p.Cys103Phe)]	NA	NA	3.91E-02	6.54E-02	5.01E-02	3.91E-02	5.32E-02	T	BN	NA	MODERATE	N	0.021	485	484	0	334	167	164	49.1
Non-user	IOB_24	chr11	55890320	G	C	OR8H3	MISSENSE	ENSG00000181761:[ENST00000313472(c.472G>C,Gtc>Ctc):ENSP00000323928(p.Val158Leu)]	NA	NA	3.89E-02	6.54E-02	5.02E-02	3.89E-02	5.32E-02	T	BN	NA	MODERATE	N	0	672	669	0	464	274	189	40.73
Non-user	IOB_24	chr11	55890355	T	C	OR8H3	SILENT	ENSG00000181761:[ENST00000313472(c.507T>C,tgT>tgC):ENSP00000323928(p.Cys169(=))]	NA	NA	3.63E-02	6.44E-02	5.02E-02	3.63E-02	5.32E-02	NA	NA	NA	LOW	NA	0	712	706	0	512	291	221	43.16
Non-user	IOB_24	chr11	55890356	G	T	OR8H3	MISSENSE	ENSG00000181761:[ENST00000313472(c.508G>T,Gac>Tac):ENSP00000323928(p.Asp170Tyr)]	NA	NA	3.63E-02	6.44E-02	5.02E-02	3.63E-02	5.32E-02	D	BN	NA	MODERATE	N	0	717	713	1	510	293	216	42.35
Non-user	IOB_24	chr11	55890571	C	T	OR8H3	SILENT	ENSG00000181761:[ENST00000313472(c.723C>T,tgC>tgT):ENSP00000323928(p.Cys241(=))]	NA	NA	3.81E-02	6.52E-02	5.02E-02	3.81E-02	5.32E-02	NA	NA	NA	LOW	NA	0	207	206	0	141	71	70	49.65
Non-user	IOB_24	chr11	55890609	C	T	OR8H3	MISSENSE	ENSG00000181761:[ENST00000313472(c.761C>T,aCt>aTt):ENSP00000323928(p.Thr254Ile)]	NA	NA	3.81E-02	6.52E-02	5.02E-02	3.81E-02	5.32E-02	D	PrD	NA	MODERATE	D	0	213	212	0	126	66	60	47.62
Non-user	IOB_24	chr11	55890726	G	T	OR8H3	MISSENSE	ENSG00000181761:[ENST00000313472(c.878G>T,aGa>aTa):ENSP00000323928(p.Arg293Ile)]	NA	NA	3.81E-02	6.58E-02	5.16E-02	3.81E-02	5.32E-02	D	PrD	NA	MODERATE	D	0.18	170	169	0	123	70	53	43.09
Non-user	IOB_24	chr11	55927442	T	C	OR8K5	MISSENSE	ENSG00000181752:[ENST00000313447(c.352A>G,Atg>Gtg):ENSP00000323853(p.Met118Val)]	NA	NA	3.95E-02	6.54E-02	5.03E-02	3.95E-02	5.62E-02	D_lc	PrD	NA	MODERATE	D	1	214	212	1	194	110	84	43.3
Non-user	IOB_24	chr11	55999897	G	A	OR5T2	SILENT	ENSG00000181718:[ENST00000313264(c.765C>T,atC>atT):ENSP00000323688(p.Ile255(=))]	NA	chr11*55999897*55999897*COSM928278*G*T*1*endometrium*1	3.69E-02	6.42E-02	4.66E-02	3.69E-02	5.62E-02	NA	NA	NA	LOW	NA	0	297	297	0	253	144	108	42.69
Non-user	IOB_24	chr11	56000288	G	A	OR5T2	MISSENSE	ENSG00000181718:[ENST00000313264(c.374C>T,aCg>aTg):ENSP00000323688(p.Thr125Met)]	NA	NA	3.69E-02	6.49E-02	5.10E-02	3.69E-02	5.62E-02	T	BN	NA	MODERATE	N	0.008	164	163	0	97	52	45	46.39
Non-user	IOB_24	chr11	56000581	G	A	OR5T2	SILENT	ENSG00000181718:[ENST00000313264(c.81C>T,aaC>aaT):ENSP00000323688(p.Asn27(=))]	NA	NA	3.87E-02	6.66E-02	5.55E-02	3.87E-02	5.62E-02	NA	NA	NA	LOW	NA	NA	241	240	0	171	91	80	46.78
Non-user	IOB_24	chr11	56058474	T	A	OR8H1	MISSENSE	ENSG00000181693:[ENST00000313022(c.65A>T,gAg>gTg):ENSP00000323595(p.Glu22Val)]	NA	NA	3.89E-02	6.49E-02	5.06E-02	3.89E-02	5.52E-02	D	PoD	NA	MODERATE	N	0	232	230	1	174	81	91	52.3
Non-user	IOB_24	chr11	56113764	G	A	OR8K1	MISSENSE	ENSG00000150261:[ENST00000279783(c.250G>A,Gcc>Acc):ENSP00000279783(p.Ala84Thr)]	NA	NA	3.91E-02	6.50E-02	5.12E-02	3.91E-02	5.73E-02	D_lc	BN	NA	MODERATE	N	0	450	445	2	326	185	141	43.25
Non-user	IOB_24	chr11	56127829	G	T	OR8J1	MISSENSE	ENSG00000172487:[ENST00000303039(c.107G>T,gGg>gTg):ENSP00000304060(p.Gly36Val)]	NA	NA	3.89E-02	6.50E-02	5.12E-02	3.89E-02	5.73E-02	T	BN	NA	MODERATE	N	0	321	317	3	228	100	127	55.7
Non-user	IOB_24	chr11	56128406	A	G	OR8J1	MISSENSE	ENSG00000172487:[ENST00000303039(c.684A>G,atA>atG):ENSP00000304060(p.Ile228Met)]	NA	NA	3.71E-02	6.57E-02	5.19E-02	3.71E-02	5.73E-02	T	PoD	NA	MODERATE	N	0	223	223	0	160	75	84	52.5
Non-user	IOB_24	chr11	56185347	T	C	OR5R1	MISSENSE	ENSG00000174942:[ENST00000312253(c.362A>G,gAt>gGt):ENSP00000308595(p.Asp121Gly)]	NA	NA	3.71E-02	6.55E-02	5.12E-02	3.71E-02	5.73E-02	D	PrD	NA	MODERATE	NA	1	177	175	0	130	64	66	50.77
Non-user	IOB_24	chr11	56185401	C	T	OR5R1	MISSENSE	ENSG00000174942:[ENST00000312253(c.308G>A,tGt>tAt):ENSP00000308595(p.Cys103Tyr)]	NA	NA	3.71E-02	6.55E-02	5.12E-02	3.71E-02	5.73E-02	T	PoD	NA	MODERATE	NA	0	195	195	0	151	77	74	49.01
Non-user	IOB_24	chr11	56185738	G	C	NA	INTERGENIC	NA	NA	NA	3.71E-02	6.85E-02	5.46E-02	3.71E-02	5.73E-02	NA	NA	NA	MODIFIER	NA	NA	99	99	0	59	24	35	59.32
Non-user	IOB_24	chr11	56230069	T	C	OR5M9	MISSENSE	ENSG00000150269:[ENST00000279791(c.809A>G,aAa>aGa):ENSP00000279791(p.Lys270Arg)]	NA	NA	3.63E-02	6.54E-02	5.08E-02	3.63E-02	5.42E-02	D	PrD	NA	MODERATE	D	0.173	105	104	0	69	37	32	46.38
Non-user	IOB_24	chr11	56230611	G	T	OR5M9	SILENT	ENSG00000150269:[ENST00000279791(c.267C>A,acC>acA):ENSP00000279791(p.Thr89(=))]	NA	NA	3.63E-02	6.55E-02	5.08E-02	3.63E-02	5.42E-02	NA	NA	NA	LOW	NA	NA	196	196	0	125	60	65	52
Non-user	IOB_24	chr11	56237966	T	C	OR5M3	MISSENSE	ENSG00000174937:[ENST00000312240(c.8A>G,aAt>aGt):ENSP00000312208(p.Asn3Ser)]	NA	NA	3.63E-02	6.88E-02	6.02E-02	3.63E-02	5.42E-02	D	PrD	NA	MODERATE	D	0.999	30	30	0	26	8	18	69.23
Non-user	IOB_24	chr11	56257877	G	A	OR5M8	3UTR	ENSG00000181371:[ENST00000327216(c.*34C>T)]	NA	NA	3.63E-02	7.72E-02	6.30E-02	3.63E-02	5.42E-02	NA	NA	NA	MODIFIER	NA	NA	29	29	0	20	10	10	50
Non-user	IOB_24	chr11	56309896	C	G	OR5M11	MISSENSE	ENSG00000255223:[ENST00000528616(c.838G>C,Gtg>Ctg):ENSP00000432417(p.Val280Leu)]	NA	NA	4.27E-02	6.93E-02	5.78E-02	4.27E-02	6.24E-02	T	BN	NA	MODERATE	NA	NA	124	123	0	108	53	54	50
Non-user	IOB_24	chr11	56310356	A	T	OR5M11	NONSENSE	ENSG00000255223:[ENST00000528616(c.378T>A,taT>taA):ENSP00000432417(p.Tyr126Ter)]	NA	NA	4.27E-02	6.89E-02	5.70E-02	4.27E-02	6.24E-02	NA	NA	NA	HIGH	NA	NA	188	188	0	139	68	71	51.08
Non-user	IOB_24	chr11	56380971	G	A	OR5M1	MISSENSE	ENSG00000255012:[ENST00000526538(c.8C>T,tCc>tTc):ENSP00000435416(p.Ser3Phe)]	NA	NA	1.52E-02	2.02E-02	3.58E-02	1.52E-02	3.58E-02	D	BN	NA	MODERATE	N	0	406	406	0	263	124	139	52.85
Non-user	IOB_24	chr11	57244296	C	T	RP11-624G17.3	NCT	ENSG00000255301:[ENST00000528885(n.538+174G>A)]	NA	chr11*57244296*57244296*COSM928617*C*T*1*endometrium*1	4.79E-03	8.61E-03	1.27E-02	4.79E-03	8.20E-03	T	BN	NA	MODERATE	NA	NA	171	170	1	136	65	69	50.74
Non-user	IOB_24	chr11	57244296	C	T	RTN4RL2	MISSENSE	ENSG00000186907:[ENST00000335099(c.1175C>T,gCg>gTg):ENSP00000335397(p.Ala392Val)]	NA	chr11*57244296*57244296*COSM928617*C*T*1*endometrium*1	4.79E-03	8.61E-03	1.27E-02	4.79E-03	8.20E-03	T	BN	NA	MODERATE	NA	NA	171	170	1	136	65	69	50.74
Non-user	IOB_24	chr11	58206714	C	T	OR5B12	MISSENSE	ENSG00000172362:[ENST00000302572(c.911G>A,gGg>gAg):ENSP00000306657(p.Gly304Glu)]	NA	NA	6.39E-03	1.54E-02	6.18E-03	6.39E-03	3.10E-03	T_lc	BN	NA	MODERATE	N	0	157	157	0	82	39	43	52.44
Non-user	IOB_24	chr11	59190120	A	G	OR5A2	MISSENSE	ENSG00000172324:[ENST00000302040(c.307T>C,Ttt>Ctt):ENSP00000303834(p.Phe103Leu)]	NA	NA	4.49E-02	4.47E-02	6.29E-02	4.49E-02	6.95E-02	D	PrD	NA	MODERATE	U	0.644	120	119	0	90	40	50	55.56
Non-user	IOB_24	chr11	59224765	A	C	OR4D6	MISSENSE	ENSG00000166884:[ENST00000300127(c.332A>C,gAt>gCt):ENSP00000300127(p.Asp111Ala)]	NA	NA	2.48E-02	4.30E-02	7.96E-02	2.48E-02	6.24E-02	D	BN	NA	MODERATE	D	1	321	319	0	216	99	115	53.24
Non-user	IOB_24	chr11	59368057	C	G	OSBP	MISSENSE	ENSG00000110048:[ENST00000263847(c.1223G>C,aGa>aCa):ENSP00000263847(p.Arg408Thr)]	NA	NA	3.99E-04	3.63E-04	4.24E-04	3.99E-04	0.00E+00	NA	NA	NA	MODIFIER	NA	NA	216	215	0	175	101	73	41.71
Non-user	IOB_24	chr11	59426945	A	G	PATL1	INTRONIC-SS-ACR-PRX	ENSG00000166889:[ENST00000300146(c.128-3T>C)]	NA	NA	NA	5.10E-05	0.00E+00	NA	NA	NA	NA	NA	LOW	NA	NA	213	210	0	164	84	80	48.78
Non-user	IOB_24	chr11	59516411	G	T	OR10V2P	EXONIC-NC	ENSG00000255538:[ENST00000525296(n.128G>T)]	NA	NA	3.91E-02	1.47E-01	1.53E-01	3.91E-02	7.57E-02	NA	NA	NA	MODIFIER	NA	NA	161	160	0	109	69	38	34.86
Non-user	IOB_24	chr11	59516411	G	T	STX3	INTRONIC	ENSG00000166900:[ENST00000300150(c.-64+20248G>T)]	NA	NA	3.91E-02	1.47E-01	1.53E-01	3.91E-02	7.57E-02	NA	NA	NA	MODIFIER	NA	NA	161	160	0	109	69	38	34.86
Non-user	IOB_24	chr11	60231260	G	A	MS4A1	EXONIC-NC	ENSG00000156738:[ENST00000534503(n.408G>A)]	NA	NA	3.22E-02	6.46E-02	7.69E-02	3.21E-02	6.65E-02	NA	NA	NA	MODIFIER	NA	NA	225	224	0	184	72	112	60.87
Non-user	IOB_24	chr11	61183554	C	G	CPSF7	EXONIC-NC	ENSG00000149532:[ENST00000537641(n.609G>C)]	NA	NA	2.06E-02	4.58E-02	1.80E-02	2.06E-02	1.23E-02	NA	NA	NA	MODIFIER	NA	NA	65	65	0	39	23	16	41.03
Non-user	IOB_24	chr11	61493401	G	A	DAGLA	INTRONIC	ENSG00000134780:[ENST00000540717(c.*22-66G>A)]	NA	NA	3.00E-03	NA	NA	3.00E-03	1.53E-02	NA	NA	NA	MODIFIER	NA	NA	91	91	0	76	26	50	65.79
Non-user	IOB_24	chr11	61672193	C	T	RAB3IL1	INTRONIC	ENSG00000167994:[ENST00000301773(c.708+33G>A)]	NA	NA	2.60E-03	2.38E-03	1.10E-02	2.60E-03	1.33E-02	NA	NA	NA	MODIFIER	NA	NA	124	118	0	116	56	58	50
Non-user	IOB_24	chr11	61730439	C	A	BEST1	MISSENSE	ENSG00000167995:[ENST00000449131(c.1633C>A,Ccc>Acc):ENSP00000399709(p.Pro545Thr)]	NA	NA	2.60E-03	2.39E-03	1.55E-02	2.60E-03	1.33E-02	D_lc	BN	NA	MODERATE	NA	NA	352	351	0	234	109	125	53.42
Non-user	IOB_24	chr11	61730439	C	A	FTH1	INTRONIC	ENSG00000167996:[ENST00000529191(c.115-3040G>T)]	NA	NA	2.60E-03	2.39E-03	1.55E-02	2.60E-03	1.33E-02	D_lc	BN	NA	MODERATE	NA	NA	352	351	0	234	109	125	53.42
Non-user	IOB_24	chr11	62621105	G	A	SNHG1	NCT	ENSG00000255717:[ENST00000545688(n.286-43C>T)]	NA	NA	9.98E-04	7.54E-04	4.69E-03	9.98E-04	5.10E-03	NA	NA	NA	MODIFIER	NA	NA	162	160	0	111	54	57	51.35
Non-user	IOB_24	chr11	62931425	G	A	SLC22A10	NCT	ENSG00000184999:[ENST00000525620(n.58-2912G>A)]	NA	NA	NA	NA	NA	NA	NA	NA	NA	NA	MODIFIER	NA	NA	100	100	0	89	47	41	46.07
Non-user	IOB_24	chr11	62931425	G	A	SLC22A25	SILENT	ENSG00000196600:[ENST00000306494(c.1515C>T,gcC>gcT):ENSP00000307443(p.Ala505(=))]	NA	NA	NA	NA	NA	NA	NA	NA	NA	NA	MODIFIER	NA	NA	100	100	0	89	47	41	46.07
Non-user	IOB_24	chr11	64111794	G	A	CCDC88B	MISSENSE	ENSG00000168071:[ENST00000356786(c.1781G>A,cGt>cAt):ENSP00000349238(p.Arg594His)]	NA	NA	7.99E-03	1.07E-02	3.59E-02	7.99E-03	2.97E-02	T_lc	BN	NA	MODERATE	NA	NA	128	127	0	89	48	41	46.07
Non-user	IOB_24	chr11	64119681	C	T	CCDC88B	MISSENSE	ENSG00000168071:[ENST00000359902(c.635C>T,gCa>gTa):ENSP00000352974(p.Ala212Val)]	NA	NA	7.99E-03	1.13E-02	3.96E-02	7.99E-03	2.97E-02	T	PoD	NA	MODERATE	NA	NA	72	72	0	65	30	33	50.77
Non-user	IOB_24	chr11	64507316	G	A	RASGRP2	INTRONIC	10235:[XM_005273707.1(c.838-35C>T)]	NA	NA	1.16E-02	2.43E-02	3.57E-02	1.16E-02	2.66E-02	NA	NA	NA	MODIFIER	NA	NA	92	91	0	88	52	35	39.77
Non-user	IOB_24	chr11	65347830	G	A	EHBP1L1	MISSENSE	ENSG00000173442:[ENST00000309295(c.514G>A,Gca>Aca):ENSP00000312671(p.Ala172Thr)]	NA	NA	3.99E-04	1.09E-03	5.58E-03	3.99E-04	1.00E-03	D	PrD	NA	MODERATE	NA	NA	167	167	0	124	77	46	37.1
Non-user	IOB_24	chr11	65422486	C	G	RELA	INTRONIC	5970:[NM_001243984.1(c.1034-222G>C)]	NA	NA	2.00E-04	2.39E-03	7.47E-04	2.00E-04	0.00E+00	NA	NA	NA	MODIFIER	NA	NA	141	141	0	106	73	33	31.13
Non-user	IOB_24	chr11	65650173	C	T	CTSW	INTRONIC	ENSG00000172543:[ENST00000526034(c.536-77C>T)]	NA	NA	NA	4.95E-05	3.63E-04	NA	NA	NA	NA	NA	MODIFIER	NA	NA	291	291	0	222	111	111	50
Non-user	IOB_24	chr11	65655470	C	A	FIBP	MISSENSE	9158:[XM_005274392.1(c.8G>T,cGc>cTc):XP_005274449.1(p.Arg3Leu)]	NA	NA	9.98E-04	3.34E-03	4.74E-03	9.98E-04	2.00E-03	NA	NA	NA	MODERATE	NA	NA	163	161	0	129	49	80	62.02
Non-user	IOB_24	chr11	65779733	A	G	CST6	MISSENSE	ENSG00000175315:[ENST00000312134(c.218A>G,cAc>cGc):ENSP00000311313(p.His73Arg)]	NA	NA	NA	NA	NA	NA	NA	T	BN	NA	MODERATE	N	0	154	151	0	148	75	73	49.32
Non-user	IOB_24	chr11	65835675	G	A	RP11-1167A19.2	INTRONIC	ENSG00000255038:[ENST00000529036(c.107-665C>T)]	NA	NA	5.99E-04	2.34E-03	7.42E-03	5.99E-04	3.10E-03	NA	NA	NA	LOW	NA	NA	161	161	0	148	76	71	47.97
Non-user	IOB_24	chr11	65835675	G	A	SF3B2	SILENT	ENSG00000087365:[ENST00000530981(c.746G>A,gcG>gcA):ENSP00000436757(p.Ala249(=))]	NA	NA	5.99E-04	2.34E-03	7.42E-03	5.99E-04	3.10E-03	NA	NA	NA	LOW	NA	NA	161	161	0	148	76	71	47.97
Non-user	IOB_24	chr11	66302683	T	A	ZDHHC24	INTRONIC	ENSG00000174165:[ENST00000526986(c.560-5724A>T)]	NA	NA	1.16E-02	NA	NA	1.16E-02	2.66E-02	NA	NA	NA	MODIFIER	NA	NA	17	17	0	19	12	7	36.84
Non-user	IOB_24	chr11	66627483	C	T	LRFN4	SILENT	ENSG00000173621:[ENST00000309602(c.1725C>T,agC>agT):ENSP00000312535(p.Ser575(=))]	NA	NA	3.16E-02	4.42E-02	6.45E-02	3.15E-02	4.50E-02	NA	NA	NA	MODIFIER	NA	NA	80	80	0	72	34	37	51.39
Non-user	IOB_24	chr11	66627483	C	T	PC	NCT	ENSG00000173599:[ENST00000530187(n.213+251G>A)]	NA	NA	3.16E-02	4.42E-02	6.45E-02	3.15E-02	4.50E-02	NA	NA	NA	MODIFIER	NA	NA	80	80	0	72	34	37	51.39
Non-user	IOB_24	chr11	67226097	C	T	CABP4	INTRONIC-SS-ACR-PRX	ENSG00000175544:[ENST00000438189(c.485-5C>T)]	NA	NA	3.26E-02	6.14E-02	5.20E-02	3.25E-02	4.60E-02	NA	NA	NA	LOW	NA	NA	171	168	2	127	0	126	99.21

Patel *et al.* , 2021. Whole-exome sequencing analysis of oral squamous cell carcinoma delineated by tobacco usage habits
Supplementary Table 4. List of somatic single nucleotide variants identified using whole exome sequencing of OSCC patients

Cohort	Sample	Chromosome	Genomic position	Reference allele	Alternate allele	Genesymbol	Variant type	Variant description	OncoMD	COSMIC	dbSNP	ExAC	ExAC_SAS	1000G	1000G_SAS	SIFT	Polyphen2	CONDEL	VEP	LRT	PhastCon7Way	Total reads in Normal	Reference read support in Normal	Alternative read support in Normal	Total reads in Tumor	Reference read support in Tumor	Alternate read support in Tumor	Alternate allele frequency in Tumor
Non-user	IOB_24	chr11	67287483	A	G	CABP2	INTRONIC	ENSG00000167791:[ENST00000353903(c.318+39T>C)]	NA	NA	chr11*67287483*67287483*A*T*rs778047665*NA*NA*NA	NA	NA	NA	NA	NA	NA	NA	MODIFIER	NA	NA	223	221	0	203	88	114	56.16
Non-user	IOB_24	chr11	68562328	C	T	CPT1A	MISSENSE	ENSG00000110090:[ENST00000538994(c.79G>A,Gcc>Acc):ENSP00000454332(p.Ala27Thr)]	NA	NA	2.38E-02	6.47E-02	2.43E-02	2.38E-02	1.33E-02	NA	NA	NA	MODERATE	NA	0.938	147	146	0	115	73	41	35.65
Non-user	IOB_24	chr11	68673672	C	T	IGHMBP2	SILENT	ENSG00000132740:[ENST00000255078(c.222C>T,tcC>tcT):ENSP00000255078(p.Ser74(=))]	NA	NA	chr11*68673672*68673672*C*A,T*rs139237340*NA*NA*NA	NA	NA	NA	NA	NA	NA	NA	MODIFIER	NA	NA	115	115	0	88	52	35	39.77
Non-user	IOB_24	chr11	68707196	G	A	IGHMBP2	SILENT	ENSG00000132740:[ENST00000255078(c.2979G>A,acG>acA):ENSP00000255078(p.Thr993(=))]	NA	NA	4.59E-03	4.43E-03	2.74E-02	4.59E-03	2.15E-02	NA	NA	NA	LOW	NA	NA	60	60	0	63	25	38	60.32
Non-user	IOB_24	chr11	69589556	G	A	FGF4	SILENT	ENSG00000075388:[ENST00000168712(c.297C>T,ctC>ctT):ENSP00000168712(p.Leu99(=))]	NA	NA	2.90E-02	1.30E-01	3.05E-02	2.90E-02	1.94E-02	NA	NA	NA	LOW	NA	NA	113	113	0	88	42	45	51.14
Non-user	IOB_24	chr11	69631046	G	A	FGF3	INTRONIC	ENSG00000186895:[ENST00000334134(c.324+42C>T)]	NA	NA	6.19E-03	6.60E-03	1.77E-02	6.19E-03	1.94E-02	NA	NA	NA	MODIFIER	NA	NA	238	238	0	194	79	113	58.25
Non-user	IOB_24	chr11	69924476	T	G	ANO1	5UTR	ENSG00000131620:[ENST00000355303(c.-237T>G)]	NA	NA	NA	NA	NA	NA	NA	NA	NA	NA	MODIFIER	NA	NA	34	34	0	13	3	10	76.92
Non-user	IOB_24	chr11	70002083	G	A	ANO1	MISSENSE	ENSG00000131620:[ENST00000355303(c.1492G>A,Ggg>Agg):ENSP00000347454(p.Gly498Arg)]	NA	NA	NA	NA	NA	NA	NA	NA	NA	NA	MODIFIER	NA	NA	99	98	0	68	44	23	33.82
Non-user	IOB_24	chr11	71259841	C	T	KRTAP5-9	SILENT	ENSG00000254997:[ENST00000528743(c.138C>T,tgC>tgT):ENSP00000431443(p.Cys46(=))]	NA	NA	3.20E-03	7.69E-03	6.66E-03	3.19E-03	3.10E-03	NA	NA	NA	LOW	NA	NA	76	76	0	47	21	26	55.32
Non-user	IOB_24	chr11	71277083	C	T	KRTAP5-10	SILENT	ENSG00000204572:[ENST00000376536(c.306C>T,tgC>tgT):ENSP00000365719(p.Cys102(=))]	NA	NA	4.61E-02	4.17E-02	7.92E-02	4.61E-02	7.57E-02	NA	NA	NA	LOW	NA	NA	120	120	0	86	46	40	46.51
Non-user	IOB_24	chr11	71627159	C	A	LOC100133315	EXONIC-NC	100133315:[NR_029192.1(n.860G>T)]	NA	NA	4.71E-02	4.67E-02	5.13E-02	4.71E-02	5.11E-02	NA	NA	NA	LOW	NA	NA	31	31	0	23	12	11	47.83
Non-user	IOB_24	chr11	71627159	C	A	RP11-849H4.2	SILENT	ENSG00000254469:[ENST00000532852(c.369G>T,gtG>gtT):ENSP00000436725(p.Val123(=))]	NA	NA	4.71E-02	4.67E-02	5.13E-02	4.71E-02	5.11E-02	NA	NA	NA	LOW	NA	NA	31	31	0	23	12	11	47.83
Non-user	IOB_24	chr11	71701763	G	A	RNF121	SILENT-SS-PRX	55298:[NR_024148.1(n.1067G>A)]	NA	NA	4.03E-02	3.36E-02	4.50E-02	4.03E-02	4.60E-02	NA	NA	NA	LOW	NA	NA	94	94	0	70	38	32	45.71
Non-user	IOB_24	chr11	71706544	A	G	RNF121	MISSENSE	ENSG00000137522:[ENST00000393713(c.590A>G,aAa>aGa):ENSP00000377316(p.Lys197Arg)]	NA	NA	4.89E-02	6.72E-02	6.09E-02	4.89E-02	5.01E-02	NA	NA	NA	MODIFIER	NA	NA	125	123	2	105	56	49	46.67
Non-user	IOB_24	chr11	71726168	G	C	NUMA1	MISSENSE	ENSG00000137497:[ENST00000358965(c.2381C>G,gCt>gGt):ENSP00000351851(p.Ala794Gly)]	NA	NA	4.39E-02	3.43E-02	4.47E-02	4.39E-02	4.60E-02	D	BN	NA	MODERATE	NA	NA	311	311	0	205	111	93	45.37
Non-user	IOB_24	chr11	71726168	G	C	RP11-849H4.4	EXONIC-NC	ENSG00000251143:[ENST00000502284(n.832G>C)]	NA	NA	4.39E-02	3.43E-02	4.47E-02	4.39E-02	4.60E-02	D	BN	NA	MODERATE	NA	NA	311	311	0	205	111	93	45.37
Non-user	IOB_24	chr11	71729383	T	C	NUMA1	NCT	ENSG00000137497:[ENST00000536119(n.1021-37A>G)]	NA	NA	4.39E-02	3.42E-02	4.47E-02	4.39E-02	4.60E-02	NA	NA	NA	MODIFIER	NA	NA	124	120	1	110	55	54	49.09
Non-user	IOB_24	chr11	71729383	T	C	RP11-849H4.4	NCT	ENSG00000251143:[ENST00000502284(n.3533+43T>C)]	NA	NA	4.39E-02	3.42E-02	4.47E-02	4.39E-02	4.60E-02	NA	NA	NA	MODIFIER	NA	NA	124	120	1	110	55	54	49.09
Non-user	IOB_24	chr11	71730071	C	G	NUMA1	NCT	ENSG00000137497:[ENST00000534987(n.966-45G>C)]	NA	NA	4.39E-02	3.43E-02	4.53E-02	4.39E-02	4.60E-02	NA	NA	NA	MODIFIER	NA	NA	52	51	0	39	15	23	58.97
Non-user	IOB_24	chr11	71730071	C	G	RP11-849H4.4	NCT	ENSG00000251143:[ENST00000502284(n.3648+16C>G)]	NA	NA	4.39E-02	3.43E-02	4.53E-02	4.39E-02	4.60E-02	NA	NA	NA	MODIFIER	NA	NA	52	51	0	39	15	23	58.97
Non-user	IOB_24	chr11	72794694	C	T	FCHSD2	INTRONIC	ENSG00000137478:[ENST00000422375(c.102+46G>A)]	NA	NA	8.39E-03	1.45E-02	1.33E-02	8.39E-03	1.23E-02	NA	NA	NA	MODIFIER	NA	NA	111	110	1	81	51	30	37.04
Non-user	IOB_24	chr11	73020846	G	T	ARHGEF17	MISSENSE	ENSG00000110237:[ENST00000263674(c.1163G>T,cGc>cTc):ENSP00000263674(p.Arg388Leu)]	NA	NA	4.89E-02	8.29E-02	6.97E-02	4.89E-02	7.67E-02	D_lc	BN	NA	MODERATE	N	0.839	297	296	1	185	110	75	40.54
Non-user	IOB_24	chr11	73073580	C	T	ARHGEF17	SILENT	ENSG00000110237:[ENST00000263674(c.4797C>T,ctC>ctT):ENSP00000263674(p.Leu1599(=))]	NA	NA	4.77E-02	8.59E-02	7.22E-02	4.77E-02	7.67E-02	NA	NA	NA	LOW	NA	NA	182	180	1	108	60	47	43.52
Non-user	IOB_24	chr11	73745596	G	A	C2CD3	SILENT	ENSG00000168014:[ENST00000414160(c.2322C>T,aaC>aaT):ENSP00000388750(p.Asn774(=))]	NA	NA	3.99E-04	4.03E-04	2.66E-04	3.99E-04	0.00E+00	NA	NA	NA	MODIFIER	NA	NA	114	114	0	95	59	36	37.89
Non-user	IOB_24	chr11	73881848	A	G	C2CD3	INTRONIC	ENSG00000168014:[ENST00000535954(c.181+227T>C)]	NA	NA	1.14E-02	2.01E-02	2.65E-02	1.14E-02	2.35E-02	NA	NA	NA	MODIFIER	NA	NA	104	104	0	94	45	49	52.13
Non-user	IOB_24	chr11	84822808	T	G	DLG2	INTRONIC	ENSG00000150672:[ENST00000527088(c.31-29A>C)]	NA	NA	2.50E-02	2.62E-02	2.07E-02	2.50E-02	2.15E-02	NA	NA	NA	MODIFIER	NA	NA	36	35	0	34	17	17	50
Non-user	IOB_24	chr11	85448679	G	A	SYTL2	MISSENSE	ENSG00000137501:[ENST00000527523(c.266C>T,gCt>gTt):ENSP00000434010(p.Ala89Val)]	NA	NA	2.06E-02	2.81E-02	8.18E-03	2.06E-02	1.02E-02	T	BN	NA	MODERATE	NA	NA	242	241	0	246	130	115	46.75
Non-user	IOB_24	chr11	85975337	A	T	EED	NCT	8726:[XR_247215.1(n.1412+32A>T)]	NA	NA	3.57E-02	3.87E-02	3.69E-02	3.57E-02	4.40E-02	NA	NA	NA	MODIFIER	NA	NA	111	111	0	83	41	42	50.6
Non-user	IOB_24	chr11	86123665	T	C	CCDC81	INTRONIC	ENSG00000149201:[ENST00000278487(c.596+64T>C)]	NA	NA	2.80E-03	NA	NA	2.80E-03	1.43E-02	NA	NA	NA	MODIFIER	NA	NA	90	90	0	61	36	25	40.98
Non-user	IOB_24	chr11	89608153	G	C	TRIM64B	MISSENSE	ENSG00000189253:[ENST00000329862(c.497C>G,tCg>tGg):ENSP00000332969(p.Ser166Trp)]	NA	NA	2.24E-02	4.05E-02	3.87E-02	2.24E-02	2.76E-02	T	PrD	NA	MODERATE	NA	NA	139	139	0	169	93	75	44.38
Non-user	IOB_24	chr11	89880530	C	T	NAALAD2	MISSENSE	ENSG00000077616:[ENST00000321955(c.227C>T,aCa>aTa):ENSP00000320083(p.Thr76Ile)]	NA	NA	3.00E-03	1.26E-03	8.99E-03	3.00E-03	1.53E-02	D	PrD	NA	MODERATE	NA	NA	272	271	0	204	109	95	46.57
Non-user	IOB_24	chr11	89896640	A	C	NAALAD2	INTRONIC	ENSG00000077616:[ENST00000527493(c.*249+43A>C)]	NA	NA	2.70E-02	4.47E-02	4.43E-02	2.70E-02	3.89E-02	NA	NA	NA	MODIFIER	NA	NA	181	180	0	130	59	71	54.62
Non-user	IOB_24	chr11	94731905	C	G	KDM4D	MISSENSE	ENSG00000186280:[ENST00000335080(c.1369C>G,Cct>Gct):ENSP00000334181(p.Pro457Ala)]	NA	NA	9.98E-04	3.34E-03	3.20E-03	9.98E-04	2.00E-03	T_lc	BN	NA	MODERATE	U	0.016	301	300	0	202	116	83	41.09
Non-user	IOB_24	chr11	100807089	G	A	ARHGAP42	INTRONIC	ENSG00000165895:[ENST00000524892(c.730+26G>A)]	NA	NA	1.20E-03	1.85E-03	3.42E-03	1.20E-03	5.10E-03	NA	NA	NA	MODIFIER	NA	NA	231	231	0	176	93	82	46.59
Non-user	IOB_24	chr11	102576493	A	C	MMP27	5UTR	ENSG00000137675:[ENST00000260229(c.-48T>G)]	NA	NA	3.83E-02	5.43E-02	4.32E-02	3.83E-02	3.58E-02	NA	NA	NA	MODIFIER	NA	NA	143	143	0	118	64	53	44.92
Non-user	IOB_24	chr11	102668686	T	A	MMP1	INTRONIC	4312:[NM_001145938.1(c.-53+144A>T)]	NA	NA	3.08E-02	6.48E-02	4.60E-02	3.08E-02	2.97E-02	NA	NA	NA	MODIFIER	NA	NA	61	60	0	33	14	19	57.58
Non-user	IOB_24	chr11	102668686	T	A	WTAPP1	NCT	ENSG00000255282:[ENST00000544704(n.345-69T>A)]	NA	NA	3.08E-02	6.48E-02	4.60E-02	3.08E-02	2.97E-02	NA	NA	NA	MODIFIER	NA	NA	61	60	0	33	14	19	57.58
Non-user	IOB_24	chr11	102668687	T	G	MMP1	INTRONIC	4312:[NM_001145938.1(c.-53+143A>C)]	NA	NA	3.08E-02	6.48E-02	4.59E-02	3.08E-02	2.97E-02	NA	NA	NA	MODIFIER	NA	NA	61	60	0	33	14	19	57.58
Non-user	IOB_24	chr11	102668687	T	G	WTAPP1	NCT	ENSG00000255282:[ENST00000525739(n.584-68T>G)]	NA	NA	3.08E-02	6.48E-02	4.59E-02	3.08E-02	2.97E-02	NA	NA	NA	MODIFIER	NA	NA	61	60	0	33	14	19	57.58
Non-user	IOB_24	chr11	103907828	G	C	DDI1	MISSENSE	ENSG00000170967:[ENST00000302259(c.278G>C,cGt>cCt):ENSP00000302805(p.Arg93Pro)]	NA	NA	NA	2.48E-05	0.00E+00	NA	NA	NA	NA	NA	MODIFIER	NA	NA	344	341	2	303	154	149	49.17
Non-user	IOB_24	chr11	103907828	G	C	PDGFD	INTRONIC	ENSG00000170962:[ENST00000393158(c.125-36845C>G)]	NA	NA	NA	2.48E-05	0.00E+00	NA	NA	NA	NA	NA	MODIFIER	NA	NA	344	341	2	303	154	149	49.17
Non-user	IOB_24	chr11	111404569	G	A	C11orf88	INTRONIC	ENSG00000183644:[ENST00000529661(c.*219+39G>A)]	NA	NA	2.30E-02	3.87E-02	4.49E-02	2.30E-02	2.45E-02	NA	NA	NA	MODIFIER	NA	NA	149	149	0	140	74	65	46.43
Non-user	IOB_24	chr11	111608258	C	G	PPP2R1B	MISSENSE	ENSG00000137713:[ENST00000311129(c.1825G>C,Gtg>Ctg):ENSP00000311344(p.Val609Leu)]	NA	NA	2.00E-02	3.15E-02	3.86E-02	2.00E-02	2.35E-02	D_lc	BN	NA	MODERATE	N	0.983	237	234	0	145	92	53	36.55
Non-user	IOB_24	chr11	114121337	T	C	RP11-64D24.2	NCT	ENSG00000256947:[ENST00000544925(n.56+18869A>G)]	NA	NA	1.04E-02	NA	NA	1.04E-02	1.53E-02	NA	NA	NA	MODIFIER	NA	NA	51	49	0	49	20	27	55.1
Non-user	IOB_24	chr11	114121337	T	C	ZBTB16	3UTR	ENSG00000109906:[ENST00000335953(c.*60T>C)]	NA	NA	1.04E-02	NA	NA	1.04E-02	1.53E-02	NA	NA	NA	MODIFIER	NA	NA	51	49	0	49	20	27	55.1
Non-user	IOB_24	chr11	114421973	A	C	NXPE1	INTRONIC	ENSG00000095110:[ENST00000534921(c.-10-40T>G)]	NA	NA	1.22E-02	2.37E-02	2.44E-02	1.22E-02	1.33E-02	NA	NA	NA	MODIFIER	NA	NA	245	242	1	196	157	38	19.39
Non-user	IOB_24	chr11	116629905	G	A	BUD13	INTRONIC-SS-ACR-PRX	ENSG00000137656:[ENST00000260210(c.1255-10C>T)]	NA	NA	4.95E-02	6.21E-02	4.38E-02	4.95E-02	4.40E-02	NA	NA	NA	MODIFIER	NA	NA	256	255	0	223	101	120	53.81
Non-user	IOB_24	chr11	116633947	G	A	BUD13	MISSENSE	ENSG00000137656:[ENST00000260210(c.358C>T,Cgt>Tgt):ENSP00000260210(p.Arg120Cys)]	NA	NA	4.91E-02	6.41E-02	4.42E-02	4.91E-02	4.40E-02	T	BN	NA	MODERATE	N	0.001	166	164	1	130	68	60	46.15
Non-user	IOB_24	chr11	116653762	C	T	ZNF259	INTRONIC	ENSG00000109917:[ENST00000444935(c.981-17G>A)]	NA	NA	4.37E-02	6.03E-02	4.38E-02	4.37E-02	4.29E-02	NA	NA	NA	MODIFIER	NA	NA	84	84	0	67	37	30	44.78
Non-user	IOB_24	chr11	116653762	C	T	ZPR1	INTRONIC	8882:[NM_001317086.1(c.820-17G>A)]	NA	NA	4.37E-02	6.03E-02	4.38E-02	4.37E-02	4.29E-02	NA	NA	NA	MODIFIER	NA	NA	84	84	0	67	37	30	44.78
Non-user	IOB_24	chr11	116661392	C	A	APOA5	MISSENSE	ENSG00000110243:[ENST00000227665(c.553G>T,Ggc>Tgc):ENSP00000227665(p.Gly185Cys)]	NA	chr11*116661392*116661392*COSM686256*C*T*1*lung*1	1.14E-02	6.37E-03	8.33E-03	1.14E-02	9.20E-03	D	PrD	NA	MODERATE	N	0	265	264	0	212	116	95	44.81
Non-user	IOB_24	chr11	116692552	A	G	APOA4	SILENT	ENSG00000110244:[ENST00000357780(c.222T>C,ggT>ggC):ENSP00000350425(p.Gly74(=))]	NA	NA	2.94E-02	3.17E-02	4.30E-02	2.94E-02	4.19E-02	NA	NA	NA	LOW	NA	NA	220	219	0	187	98	89	47.59
Non-user	IOB_24	chr11	117152890	G	A	RNF214	MISSENSE	ENSG00000167257:[ENST00000530849(c.1151G>A,gGt>gAt):ENSP00000432903(p.Gly384Asp)]	NA	NA	NA	8.32E-06	0.00E+00	NA	NA	NA	NA	NA	MODIFIER	NA	NA	163	162	0	132	79	51	38.64
Non-user	IOB_24	chr11	117988105	C	T	TMPRSS4	MISSENSE	ENSG00000137648:[ENST00000522307(c.797C>T,cCg>cTg):ENSP00000428814(p.Pro266Leu)]	NA	NA	3.59E-02	2.67E-02	4.09E-02	3.59E-02	4.29E-02	NA	NA	NA	MODIFIER	NA	NA	172	172	0	128	64	64	50
Non-user	IOB_24	chr11	117988665	T	C	TMPRSS4	EXONIC-NC	56649:[XR_246454.1(n.1457T>C)]	NA	NA	NA	NA	NA	NA	NA	NA	NA	NA	MODIFIER	NA	NA	34	34	0	26	15	10	38.46
Non-user	IOB_24	chr11	118896430	G	A	SLC37A4	MISSENSE	ENSG00000137700:[ENST00000330775(c.1019C>T,gCg>gTg):ENSP00000476242(p.Ala340Val)]	NA	NA	2.80E-03	6.06E-03	1.43E-02	2.80E-03	1.23E-02	NA	NA	NA	MODIFIER	NA	NA	26	26	0	41	19	22	53.66
Non-user	IOB_24	chr11	118972336	C	T	DPAGT1	SILENT	ENSG00000172269:[ENST00000354202(c.30G>A,ccG>ccA):ENSP00000346142(p.Pro10(=))]	NA	NA	NA	3.30E-05	2.42E-04	NA	NA	NA	NA	NA	MODIFIER	NA	NA	171	171	0	141	65	75	53.19
Non-user	IOB_24	chr11	119187816	C	A	MCAM	INTRONIC	4162:[XM_005271552.1(c.-636+607G>T)]	NA	NA	NA	NA	NA	NA	NA	NA	NA	NA	MODIFIER	NA	NA	88	85	0	60	54	5	8.33
Non-user	IOB_24	chr11	121061475	C	T	TECTA	MISSENSE	ENSG00000109927:[ENST00000264037(c.6428C>T,tCa>tTa):ENSP00000264037(p.Ser2143Leu)]	NA	NA	7.99E-03	3.40E-03	2.50E-02	7.99E-03	4.09E-02	NA	NA	NA	MODERATE	NA	0.991	217	214	0	211	121	90	42.65
Non-user	IOB_24	chr11	121323277	G	A	RP11-730K11.1	NCT	ENSG00000246790:[ENST00000501964(n.339+107C>T)]	NA	NA	2.00E-02	4.35E-02	2.04E-02	2.00E-02	1.02E-02	NA	NA	NA	MODIFIER	NA	NA	109	109	0	88	43	45	51.14
Non-user	IOB_24	chr11	121323277	G	A	SORL1	SILENT	ENSG00000137642:[ENST00000260197(c.237G>A,agG>agA):ENSP00000260197(p.Arg79(=))]	NA	NA	2.00E-02	4.35E-02	2.04E-02	2.00E-02	1.02E-02	NA	NA	NA	MODIFIER	NA	NA	109	109	0	88	43	45	51.14
Non-user	IOB_24	chr11	124005574	A	G	VWA5A	INTRONIC	ENSG00000110002:[ENST00000392748(c.1245-53A>G)]	NA	NA	5.99E-04	NA	NA	5.99E-04	1.00E-03	NA	NA	NA	MODIFIER	NA	NA	92	91	0	86	40	46	53.49
Non-user	IOB_24	chr11	124180359	A	C	OR8D1	MISSENSE	ENSG00000196341:[ENST00000357821(c.304T>G,Ttt>Gtt):ENSP00000350474(p.Phe102Val)]	NA	NA	1.06E-02	1.70E-02	2.32E-02	1.06E-02	1.43E-02	D	PoD	NA	MODERATE	U	0.862	150	150	0	102	53	48	47.06
Non-user	IOB_24	chr11	124953662	A	G	SLC37A2	INTRONIC	ENSG00000134955:[ENST00000298280(c.977-49A>G)]	NA	NA	4.99E-03	1.05E-02	5.03E-03	4.99E-03	2.00E-03	NA	NA	NA	MODIFIER	NA	NA	80	80	0	79	45	34	43.04
Non-user	IOB_24	chr11	126144811	G	A	FOXRED1	NCT	ENSG00000110074:[ENST00000534011(n.593-15G>A)]	NA	NA	3.99E-04	4.29E-04	2.97E-03	3.99E-04	2.00E-03	NA	NA	NA	MODIFIER	NA	NA	158	158	0	127	62	64	50.39
Non-user	IOB_24	chr11	126162647	C	T	TIRAP	MISSENSE	ENSG00000150455:[ENST00000392678(c.343C>T,Cgc>Tgc):ENSP00000376445(p.Arg115Cys)]	NA	chr11*126162647*126162647*COSM1267864*C*T*1*oesophagus*1|chr11*126162647*126162647*COSM1267865*C*T*1*oesophagus*1	NA	1.66E-04	8.81E-04	NA	NA	D	PrD	NA	MODERATE	NA	NA	163	162	0	124	70	53	42.74
Non-user	IOB_24	chr11	126873714	G	A	AP002833.1	EXONIC-NC	ENSG00000256527:[ENST00000404882(n.417G>A)]	NA	NA	2.98E-02	NA	NA	2.98E-02	2.56E-02	NA	NA	NA	MODIFIER	NA	NA	116	116	0	103	51	50	48.54
Non-user	IOB_24	chr11	126873714	G	A	KIRREL3-AS3	EXONIC-NC	ENSG00000218109:[ENST00000525678(n.910G>A)]	NA	NA	2.98E-02	NA	NA	2.98E-02	2.56E-02	NA	NA	NA	MODIFIER	NA	NA	116	116	0	103	51	50	48.54
Non-user	IOB_24	chr11	130010466	C	T	APLP2	INTRONIC	ENSG00000084234:[ENST00000533616(c.*1373+88C>T)]	NA	NA	2.00E-04	NA	NA	2.00E-04	1.00E-03	NA	NA	NA	MODIFIER	NA	NA	137	136	0	123	58	65	52.85
Non-user	IOB_24	chr11	130059528	A	G	ST14	INTRONIC	ENSG00000149418:[ENST00000278742(c.441-106A>G)]	NA	NA	4.01E-02	6.10E-02	4.17E-02	4.01E-02	1.94E-02	NA	NA	NA	MODIFIER	NA	NA	148	148	0	95	48	47	49.47
Non-user	IOB_24	chr11	130270855	T	C	LOC100996646	NCT	100996646:[XR_246502.1(n.333-50T>C)]	NA	NA	NA	6.86E-01	6.70E-01	NA	NA	NA	NA	NA	MODIFIER	NA	NA	57	57	0	44	0	43	97.73
Non-user	IOB_24	chr11	133791225	C	T	IGSF9B	MISSENSE	ENSG00000080854:[ENST00000321016(c.2395G>A,Gac>Aac):ENSP00000317980(p.Asp799Asn)]	NA	NA	NA	9.43E-06	0.00E+00	NA	NA	T	PrD	NA	MODERATE	D	1	223	222	0	158	89	68	43.04
Non-user	IOB_24	chr12	347225	C	G	SLC6A13	INTRONIC	6540:[XM_005253750.1(c.416-49G>C)]	NA	NA	3.02E-02	1.18E-02	3.09E-04	3.02E-02	0.00E+00	NA	NA	NA	MODIFIER	NA	NA	29	29	0	30	16	14	46.67
Non-user	IOB_24	chr12	432938	T	A	KDM5A	MISSENSE	ENSG00000073614:[ENST00000382815(c.1978A>T,Atg>Ttg):ENSP00000372265(p.Met660Leu)]	NA	NA	NA	4.17E-05	3.04E-04	NA	NA	NA	NA	NA	MODIFIER	NA	NA	75	75	0	55	32	23	41.82
Non-user	IOB_24	chr12	666721	G	A	B4GALNT3	INTRONIC	283358:[XM_005253682.1(c.2384-53G>A)]	NA	NA	4.39E-02	NA	NA	4.39E-02	8.49E-02	NA	NA	NA	MODIFIER	NA	NA	26	26	0	36	19	17	47.22
Non-user	IOB_24	chr12	2794920	C	T	CACNA1C	SILENT	ENSG00000151067:[ENST00000327702(c.5697C>T,gaC>gaT):ENSP00000329877(p.Asp1899(=))]	NA	NA	NA	1.66E-05	1.21E-04	NA	NA	NA	NA	NA	LOW	NA	0.83	90	90	0	44	27	17	38.64
Non-user	IOB_24	chr12	2794920	C	T	CACNA1C-AS1	NCT	100652846:[NR_045725.1(n.333+4386G>A)]	NA	NA	NA	1.66E-05	1.21E-04	NA	NA	NA	NA	NA	LOW	NA	0.83	90	90	0	44	27	17	38.64
Non-user	IOB_24	chr12	5153694	C	T	KCNA5	SILENT	ENSG00000130037:[ENST00000252321(c.381C>T,tcC>tcT):ENSP00000252321(p.Ser127(=))]	NA	NA	1.48E-02	2.67E-02	1.28E-02	1.48E-02	1.02E-02	NA	NA	NA	LOW	NA	NA	323	323	0	268	137	131	48.88
Non-user	IOB_24	chr12	6061559	C	T	VWF	MISSENSE-SS-PRX	ENSG00000110799:[ENST00000261405(c.8113G>A,Gga>Aga):ENSP00000261405(p.Gly2705Arg)]	NA	NA	4.47E-02	5.93E-02	1.24E-01	4.47E-02	1.21E-01	D	PrD	NA	MODERATE	N	1	74	73	0	59	27	32	54.24
Non-user	IOB_24	chr12	6344472	G	A	CD9	SILENT	ENSG00000010278:[ENST00000382515(c.219G>A,acG>acA):ENSP00000371955(p.Thr73(=))]	NA	NA	4.59E-03	2.04E-03	1.48E-02	4.59E-03	2.35E-02	NA	NA	NA	MODIFIER	NA	NA	138	137	1	120	57	63	52.5
Non-user	IOB_24	chr12	6579796	T	C	VAMP1	5UTR	ENSG00000139190:[ENST00000361716(c.-99A>G)]	NA	NA	6.79E-03	NA	NA	6.59E-03	1.94E-02	NA	NA	NA	MODIFIER	NA	NA	136	135	0	103	42	60	58.25
Non-user	IOB_24	chr12	6626232	C	T	NCAPD2	INTRONIC	ENSG00000010292:[ENST00000315579(c.1185+41C>T)]	NA	NA	1.14E-02	5.73E-03	3.80E-02	1.14E-02	5.42E-02	NA	NA	NA	MODIFIER	NA	NA	67	67	0	47	24	22	46.81
Non-user	IOB_24	chr12	6634692	A	C	NCAPD2	NCT	ENSG00000010292:[ENST00000542492(n.148-57A>C)]	NA	NA	1.14E-02	NA	NA	1.14E-02	5.42E-02	NA	NA	NA	MODIFIER	NA	NA	160	159	0	139	72	67	48.2
Non-user	IOB_24	chr12	6637070	G	A	NCAPD2	INTRONIC	ENSG00000010292:[ENST00000545962(c.2884+16G>A)]	NA	NA	NA	NA	NA	NA	NA	NA	NA	NA	MODIFIER	NA	NA	223	223	0	206	200	5	2.43
Non-user	IOB_24	chr12	6660096	G	A	IFFO1	INTRONIC	ENSG00000010295:[ENST00000336604(c.834+11C>T)]	NA	NA	9.98E-04	3.13E-04	2.12E-03	9.98E-04	4.10E-03	NA	NA	NA	MODIFIER	NA	NA	104	104	0	71	38	33	46.48
Non-user	IOB_24	chr12	6679876	G	A	CHD4	INTRONIC	ENSG00000111642:[ENST00000544040(c.5701-17C>T)]	NA	NA	2.62E-02	9.12E-03	3.12E-02	2.62E-02	3.58E-02	NA	NA	NA	MODIFIER	NA	NA	57	56	0	61	41	20	32.79

Patel *et al.* , 2021. Whole-exome sequencing analysis of oral squamous cell carcinoma delineated by tobacco usage habits
Supplementary Table 4. List of somatic single nucleotide variants identified using whole exome sequencing of OSCC patients

Cohort	Sample	Chromosome	Genomic position	Reference allele	Alternate allele	Genesymbol	Variant type	Variant description	OncoMD	COSMIC	dbSNP	ExAC	ExAC_SAS	1000G	1000G_SAS	SIFT	Polyphen2	CONDEL	VEP	LRT	PhastCon7Way	Total reads in Normal	Reference read support in Normal	Alternative read support in Normal	Total reads in Tumor	Reference read support in Tumor	Alternate read support in Tumor	Alternate allele frequency in Tumor
Non-user	IOB_24	chr12	6729460	T	C	LPAR5	MISSENSE	ENSG00000184574:[ENST00000329858(c.955A>G,Acc>Gcc):ENSP00000327875(p.Thr319Ala)]	NA	NA	1.76E-02	9.02E-03	3.52E-02	1.76E-02	3.68E-02	T	BN	NA	MODERATE	N	0	285	285	0	177	92	84	47.46
Non-user	IOB_24	chr12	6883722	T	C	LAG3	INTRONIC	ENSG00000089692:[ENST00000203629(c.512-39T>C)]	NA	NA	4.59E-03	5.28E-03	2.42E-02	4.59E-03	1.84E-02	NA	NA	NA	MODIFIER	NA	NA	98	98	0	87	39	48	55.17
Non-user	IOB_24	chr12	6933589	C	T	GPR162	SILENT	ENSG00000250510:[ENST00000311268(c.525C>T,atC>atT):ENSP00000311528(p.Ile175(=))]	NA	NA	2.40E-02	2.45E-02	7.19E-02	2.40E-02	8.18E-02	NA	NA	NA	MODIFIER	NA	NA	192	191	0	162	76	86	53.09
Non-user	IOB_24	chr12	6942823	A	G	LEPREL2	EXONIC-NC	ENSG00000110811:[ENST00000545596(n.45A>G)]	NA	NA	NA	6.66E-01	7.38E-01	8.16E-01	7.49E-01	NA	NA	NA	MODIFIER	NA	NA	186	185	0	143	3	140	97.9
Non-user	IOB_24	chr12	7045531	A	G	ATN1	SILENT	ENSG00000111676:[ENST00000356654(c.1101A>G,ccA>ccG):ENSP00000349076(p.Pro367(=))]	NA	NA	4.99E-03	4.23E-03	1.40E-02	4.99E-03	1.53E-02	NA	NA	NA	LOW	NA	NA	162	162	0	149	71	77	51.68
Non-user	IOB_24	chr12	8192590	G	C	FOXJ2	SILENT	ENSG00000065970:[ENST00000162391(c.162G>C,ctG>ctC):ENSP00000162391(p.Leu54(=))]	NA	NA	4.09E-02	1.56E-02	2.00E-02	4.09E-02	1.94E-02	NA	NA	NA	LOW	NA	NA	296	295	0	177	89	88	49.72
Non-user	IOB_24	chr12	8195173	T	C	FOXJ2	INTRONIC	ENSG00000065970:[ENST00000162391(c.334-81T>C)]	NA	NA	4.39E-02	NA	NA	4.39E-02	1.94E-02	NA	NA	NA	MODIFIER	NA	NA	110	110	0	104	52	52	50
Non-user	IOB_24	chr12	8666296	G	A	CLEC4D	5UTR	ENSG00000166527:[ENST00000299665(c.-33G>A)]	NA	NA	3.49E-02	2.09E-02	1.20E-01	3.49E-02	1.32E-01	NA	NA	NA	MODIFIER	NA	NA	72	72	0	41	29	12	29.27
Non-user	IOB_24	chr12	9230098	G	T	A2M	INTRONIC	2:[NM_001347424.1(c.2977-82C>A)]	NA	NA	1.46E-02	NA	NA	1.46E-02	7.46E-02	NA	NA	NA	MODIFIER	NA	NA	58	58	0	39	24	15	38.46
Non-user	IOB_24	chr12	9254241	G	A	A2M	SILENT	ENSG00000175899:[ENST00000318602(c.1296C>T,taC>taT):ENSP00000323929(p.Tyr432(=))]	NA	NA	3.49E-02	5.10E-02	8.11E-02	3.49E-02	8.49E-02	NA	NA	NA	LOW	NA	0.001	57	56	1	45	18	27	60
Non-user	IOB_24	chr12	9451378	C	A	LOC642846	EXONIC-NC-SS-PRX	642846:[NR_024374.1(n.1039C>A)]	NA	NA	NA	3.20E-04	2.07E-03	NA	NA	NA	NA	NA	LOW	NA	NA	179	177	0	121	97	23	19.01
Non-user	IOB_24	chr12	9451378	C	A	RP11-22B23.1	EXONIC-NC-SS-PRX	ENSG00000111788:[ENST00000341155(n.1045C>A)]	NA	NA	NA	3.20E-04	2.07E-03	NA	NA	NA	NA	NA	LOW	NA	NA	179	177	0	121	97	23	19.01
Non-user	IOB_24	chr12	10587984	G	C	KLRC2	SILENT	ENSG00000205809:[ENST00000535069(c.132C>G,ctC>ctG):ENSP00000438983(p.Leu44(=))]	NA	NA	NA	1.65E-05	1.21E-04	NA	NA	NA	NA	NA	LOW	NA	NA	206	206	0	188	138	49	26.06
Non-user	IOB_24	chr12	10587984	G	C	NKG2-E	SILENT	ENSG00000255641:[ENST00000539033(c.213C>G,ctC>ctG):ENSP00000437563(p.Leu71(=))]	NA	NA	NA	1.65E-05	1.21E-04	NA	NA	NA	NA	NA	LOW	NA	NA	206	206	0	188	138	49	26.06
Non-user	IOB_24	chr12	10659217	T	C	EIF2S3L	MISSENSE	ENSG00000180574:[ENST00000322446(c.716T>C,aTa>aCa):ENSP00000323063(p.Ile239Thr)]	NA	NA	3.20E-03	2.47E-03	1.79E-02	3.19E-03	1.64E-02	D	BN	NA	MODERATE	D	1	282	278	0	194	96	97	50
Non-user	IOB_24	chr12	11149688	G	C	PRH1	NCT	5554:[NR_133575.1(n.372-23368C>G)]	NA	NA	1.20E-03	5.77E-04	4.18E-03	1.20E-03	6.10E-03	NA	NA	NA	MODIFIER	NA	NA	217	217	0	194	87	105	54.12
Non-user	IOB_24	chr12	11149688	G	C	PRH1-PRR4	NCT	100533464:[NR_037918.2(n.478-23368C>G)]	NA	NA	1.20E-03	5.77E-04	4.18E-03	1.20E-03	6.10E-03	NA	NA	NA	MODIFIER	NA	NA	217	217	0	194	87	105	54.12
Non-user	IOB_24	chr12	11149688	G	C	PRH1-TAS2R14	INTRONIC	106707243:[NM_001316893.1(c.141-23368C>G)]	NA	NA	1.20E-03	5.77E-04	4.18E-03	1.20E-03	6.10E-03	NA	NA	NA	MODIFIER	NA	NA	217	217	0	194	87	105	54.12
Non-user	IOB_24	chr12	11149688	G	C	PRR4	NCT	ENSG00000111215:[ENST00000536086(n.23-23368C>G)]	NA	NA	1.20E-03	5.77E-04	4.18E-03	1.20E-03	6.10E-03	NA	NA	NA	MODIFIER	NA	NA	217	217	0	194	87	105	54.12
Non-user	IOB_24	chr12	11149688	G	C	TAS2R14	NCT	ENSG00000212127:[ENST00000381852(n.426-23368C>G)]	NA	NA	1.20E-03	5.77E-04	4.18E-03	1.20E-03	6.10E-03	NA	NA	NA	MODIFIER	NA	NA	217	217	0	194	87	105	54.12
Non-user	IOB_24	chr12	11149688	G	C	TAS2R20	MISSENSE	ENSG00000255837:[ENST00000538986(c.787C>G,Ctt>Gtt):ENSP00000441624(p.Leu263Val)]	NA	NA	1.20E-03	5.77E-04	4.18E-03	1.20E-03	6.10E-03	NA	NA	NA	MODIFIER	NA	NA	217	217	0	194	87	105	54.12
Non-user	IOB_24	chr12	11214886	G	T	PRH1	NCT	5554:[NR_133575.1(n.203-15099C>A)]	NA	NA	4.39E-03	2.65E-03	1.99E-02	4.39E-03	2.25E-02	NA	NA	NA	MODIFIER	NA	NA	213	212	0	177	84	91	51.41
Non-user	IOB_24	chr12	11214886	G	T	PRH1-PRR4	NCT	100533464:[NR_037918.2(n.205-15099C>A)]	NA	NA	4.39E-03	2.65E-03	1.99E-02	4.39E-03	2.25E-02	NA	NA	NA	MODIFIER	NA	NA	213	212	0	177	84	91	51.41
Non-user	IOB_24	chr12	11214886	G	T	PRH1-TAS2R14	INTRONIC	106707243:[NM_001316893.1(c.-133-15099C>A)]	NA	NA	4.39E-03	2.65E-03	1.99E-02	4.39E-03	2.25E-02	NA	NA	NA	MODIFIER	NA	NA	213	212	0	177	84	91	51.41
Non-user	IOB_24	chr12	11214886	G	T	PRR4	NCT	ENSG00000111215:[ENST00000536668(n.178-15099C>A)]	NA	NA	4.39E-03	2.65E-03	1.99E-02	4.39E-03	2.25E-02	NA	NA	NA	MODIFIER	NA	NA	213	212	0	177	84	91	51.41
Non-user	IOB_24	chr12	11214886	G	T	TAS2R14	NCT	ENSG00000212127:[ENST00000381852(n.153-15099C>A)]	NA	NA	4.39E-03	2.65E-03	1.99E-02	4.39E-03	2.25E-02	NA	NA	NA	MODIFIER	NA	NA	213	212	0	177	84	91	51.41
Non-user	IOB_24	chr12	11214886	G	T	TAS2R46	MISSENSE	ENSG00000226761:[ENST00000533467(c.8C>A,aCt>aAt):ENSP00000436450(p.Thr3Asn)]	NA	NA	4.39E-03	2.65E-03	1.99E-02	4.39E-03	2.25E-02	NA	NA	NA	MODIFIER	NA	NA	213	212	0	177	84	91	51.41
Non-user	IOB_24	chr12	11461519	C	T	PRB4	MISSENSE	ENSG00000230657:[ENST00000279575(c.398G>A,cGt>cAt):ENSP00000279575(p.Arg133His)]	NA	NA	2.60E-03	4.35E-04	2.21E-03	2.60E-03	7.20E-03	T_lc	BN	NA	MODERATE	NA	NA	49	39	0	40	26	10	25
Non-user	IOB_24	chr12	12967053	C	G	APOLD1	INTRONIC	ENSG00000178878:[ENST00000534843(c.*479-12C>G)]	NA	NA	3.87E-02	1.83E-02	2.41E-02	3.87E-02	1.84E-02	NA	NA	NA	MODIFIER	NA	NA	303	302	0	278	135	141	50.72
Non-user	IOB_24	chr12	12967053	C	G	DDX47	NCT	ENSG00000213782:[ENST00000542123(n.98-12C>G)]	NA	NA	3.87E-02	1.83E-02	2.41E-02	3.87E-02	1.84E-02	NA	NA	NA	MODIFIER	NA	NA	303	302	0	278	135	141	50.72
Non-user	IOB_24	chr12	13233605	G	T	KIAA1467	EXONIC-NC	ENSG00000084444:[ENST00000541950(n.945G>T)]	NA	NA	1.02E-02	2.25E-02	1.57E-02	1.02E-02	1.12E-02	NA	NA	NA	MODIFIER	NA	NA	70	70	0	43	27	16	37.21
Non-user	IOB_24	chr12	14613631	A	G	ATF7IP	SILENT	ENSG00000171681:[ENST00000536444(c.2358A>G,gtA>gtG):ENSP00000445955(p.Val786(=))]	NA	NA	5.99E-04	1.63E-03	2.42E-04	5.99E-04	0.00E+00	NA	NA	NA	LOW	NA	NA	258	258	0	183	92	91	49.73
Non-user	IOB_24	chr12	16347327	G	A	SLC15A5	MISSENSE	ENSG00000188991:[ENST00000344941(c.1544C>T,gCa>gTa):ENSP00000340402(p.Ala515Val)]	NA	NA	9.98E-03	5.80E-03	1.28E-02	9.98E-03	1.53E-02	D	PoD	NA	MODERATE	N	1	238	236	0	163	81	82	50.31
Non-user	IOB_24	chr12	16377347	C	T	SLC15A5	INTRONIC-SS-DNR	ENSG00000188991:[ENST00000344941(c.1351+1G>A)]	NA	NA	9.59E-03	6.35E-03	1.33E-02	9.58E-03	1.43E-02	NA	NA	NA	HIGH	NA	NA	113	111	0	80	45	35	43.75
Non-user	IOB_24	chr12	18242254	A	G	RERGL	INTRONIC	ENSG00000111404:[ENST00000536890(c.53-364T>C)]	NA	NA	1.40E-03	5.10E-04	3.16E-03	1.40E-03	7.20E-03	NA	NA	NA	MODIFIER	NA	NA	39	39	0	32	12	20	62.5
Non-user	IOB_24	chr12	19427764	T	C	PLEKHA5	MISSENSE	ENSG00000052126:[ENST00000539256(c.416T>C,gTt>gCt):ENSP00000440611(p.Val139Ala)]	NA	NA	2.70E-02	1.60E-02	3.38E-02	2.70E-02	3.58E-02	T	BN	NA	MODERATE	N	0.429	139	139	0	114	58	55	48.25
Non-user	IOB_24	chr12	19427766	A	G	PLEKHA5	MISSENSE	ENSG00000052126:[ENST00000299275(c.1144A>G,Agc>Ggc):ENSP00000299275(p.Ser382Gly)]	NA	NA	2.70E-02	1.60E-02	3.38E-02	2.70E-02	3.58E-02	D	BN	NA	MODERATE	N	0.987	139	139	0	113	58	54	47.79
Non-user	IOB_24	chr12	21054393	A	T	LST3	SILENT	ENSG00000257046:[ENST00000540229(c.1857A>T,gtA>gtT):ENSP00000441269(p.Val619(=))]	NA	chr12*21054393*21054393*COSM303243*A*AT*1*central_nervous_system*1|chr12*21054393*21054394*COSM1360649*AT*A*1*large_intestine*1	3.20E-03	2.20E-03	1.15E-02	3.19E-03	1.64E-02	NA	NA	NA	LOW	NA	NA	29	29	0	20	10	10	50
Non-user	IOB_24	chr12	21054393	A	T	SLCO1B3	SILENT	ENSG00000111700:[ENST00000261196(c.1857A>T,gtA>gtT):ENSP00000261196(p.Val619(=))]	NA	chr12*21054393*21054393*COSM303243*A*AT*1*central_nervous_system*1|chr12*21054393*21054394*COSM1360649*AT*A*1*large_intestine*1	3.20E-03	2.20E-03	1.15E-02	3.19E-03	1.64E-02	NA	NA	NA	LOW	NA	NA	29	29	0	20	10	10	50
Non-user	IOB_24	chr12	21054393	A	T	SLCO1B7	INTRONIC	ENSG00000205754:[ENST00000554957(c.359+42888A>T)]	NA	chr12*21054393*21054393*COSM303243*A*AT*1*central_nervous_system*1|chr12*21054393*21054394*COSM1360649*AT*A*1*large_intestine*1	3.20E-03	2.20E-03	1.15E-02	3.19E-03	1.64E-02	NA	NA	NA	LOW	NA	NA	29	29	0	20	10	10	50
Non-user	IOB_24	chr12	22655821	G	A	C2CD5	INTRONIC	ENSG00000111731:[ENST00000333957(c.1147+3824C>T)]	NA	NA	4.27E-02	NA	NA	4.27E-02	1.01E-01	NA	NA	NA	MODIFIER	NA	NA	49	49	0	33	22	11	33.33
Non-user	IOB_24	chr12	26755486	T	C	ITPR2	INTRONIC	ENSG00000123104:[ENST00000381340(c.3553-58A>G)]	NA	NA	4.61E-02	NA	NA	4.61E-02	1.01E-01	NA	NA	NA	MODIFIER	NA	NA	107	104	0	87	44	42	48.28
Non-user	IOB_24	chr12	29596365	T	C	OVCH1	MISSENSE	ENSG00000187950:[ENST00000318184(c.3086A>G,aAg>aGg):ENSP00000326708(p.Lys1029Arg)]	NA	NA	4.13E-02	6.89E-02	5.52E-02	4.13E-02	5.42E-02	NA	NA	NA	MODIFIER	NA	NA	185	184	0	143	87	56	39.16
Non-user	IOB_24	chr12	29596365	T	C	OVCH1-AS1	INTRONIC	ENSG00000257599:[ENST00000551108(c.349-43454T>C)]	NA	NA	4.13E-02	6.89E-02	5.52E-02	4.13E-02	5.42E-02	NA	NA	NA	MODIFIER	NA	NA	185	184	0	143	87	56	39.16
Non-user	IOB_24	chr12	29618027	A	C	OVCH1	INTRONIC	ENSG00000187950:[ENST00000318184(c.1929+53T>G)]	NA	NA	4.79E-02	NA	NA	4.79E-02	5.42E-02	NA	NA	NA	MODIFIER	NA	NA	95	94	0	90	38	51	56.67
Non-user	IOB_24	chr12	29618027	A	C	OVCH1-AS1	NCT	101055625:[NR_073172.1(n.561-21792A>C)]	NA	NA	4.79E-02	NA	NA	4.79E-02	5.42E-02	NA	NA	NA	MODIFIER	NA	NA	95	94	0	90	38	51	56.67
Non-user	IOB_24	chr12	29689157	C	T	TMTC1	SILENT	ENSG00000133687:[ENST00000551659(c.1956G>A,tcG>tcA):ENSP00000448112(p.Ser652(=))]	NA	NA	1.70E-02	1.55E-02	3.61E-02	1.70E-02	3.27E-02	NA	NA	NA	LOW	NA	NA	170	170	0	134	66	68	50.75
Non-user	IOB_24	chr12	29689264	T	C	TMTC1	INTRONIC	83857:[XM_005253504.1(c.624-14A>G)]	NA	NA	1.70E-02	1.55E-02	3.61E-02	1.70E-02	3.27E-02	NA	NA	NA	MODIFIER	NA	NA	181	180	0	153	82	71	46.41
Non-user	IOB_24	chr12	31249708	C	T	DDX11	INTRONIC	ENSG00000013573:[ENST00000251758(c.*951+29C>T)]	NA	NA	3.97E-02	7.06E-02	4.45E-02	3.97E-02	3.17E-02	NA	NA	NA	MODIFIER	NA	NA	170	170	0	176	94	82	46.59
Non-user	IOB_24	chr12	31256517	C	T	DDX11	MISSENSE	ENSG00000013573:[ENST00000545115(c.198C>T,Cca>Tca):ENSP00000443750(p.Pro67Ser)]	NA	chr12*31256517*31256517*COSM1318020*C*T*1*haematopoietic_and_lymphoid_tissue*1|chr12*31256517*31256517*COSM1318019*C*T*1*haematopoietic_and_lymphoid_tissue*1	4.23E-02	7.25E-02	4.37E-02	4.23E-02	3.17E-02	NA	NA	NA	LOW	NA	1	89	88	0	60	24	36	60
Non-user	IOB_24	chr12	32480327	G	A	BICD1	NCT	636:[XR_242898.1(n.1559-68G>A)]	NA	NA	1.52E-02	NA	NA	1.52E-02	1.23E-02	NA	NA	NA	MODIFIER	NA	NA	92	91	0	60	32	28	46.67
Non-user	IOB_24	chr12	32886672	A	G	DNM1L	SILENT	ENSG00000087470:[ENST00000266481(c.1470A>G,gaA>gaG):ENSP00000266481(p.Glu490(=))]	NA	NA	5.39E-03	7.74E-03	4.06E-03	5.39E-03	4.10E-03	NA	NA	NA	MODIFIER	NA	NA	94	93	0	67	39	28	41.79
Non-user	IOB_24	chr12	32886672	A	G	YARS2	NCT	51067:[XR_242892.1(n.1650-8373T>C)]	NA	NA	5.39E-03	7.74E-03	4.06E-03	5.39E-03	4.10E-03	NA	NA	NA	MODIFIER	NA	NA	94	93	0	67	39	28	41.79
Non-user	IOB_24	chr12	40114816	C	A	C12orf40	SILENT	ENSG00000180116:[ENST00000324616(c.1722C>A,gcC>gcA):ENSP00000317671(p.Ala574(=))]	NA	NA	NA	NA	NA	NA	NA	NA	NA	NA	MODIFIER	NA	NA	220	220	0	173	166	6	3.47
Non-user	IOB_24	chr12	40837165	C	T	MUC19	MISSENSE	ENSG00000205592:[ENST00000454784(c.2363C>T,aCt>aTt):ENSP00000476404(p.Thr788Ile)]	NA	NA	1.34E-02	2.94E-02	2.20E-02	1.34E-02	1.94E-02	NA	PrD	NA	MODERATE	NA	NA	90	90	0	70	37	33	47.14
Non-user	IOB_24	chr12	40837165	C	T	RP11-115F18.1	NCT	ENSG00000258167:[ENST00000552757(n.25+460G>A)]	NA	NA	1.34E-02	2.94E-02	2.20E-02	1.34E-02	1.94E-02	NA	PrD	NA	MODERATE	NA	NA	90	90	0	70	37	33	47.14
Non-user	IOB_24	chr12	40839382	C	A	MUC19	SILENT	ENSG00000205592:[ENST00000454784(c.2919C>A,ggC>ggA):ENSP00000476404(p.Gly973(=))]	NA	NA	1.06E-02	2.79E-02	2.06E-02	1.06E-02	1.94E-02	NA	NA	NA	LOW	NA	NA	221	221	0	166	82	84	50.6
Non-user	IOB_24	chr12	40894832	A	C	MUC19	INTRONIC	283463:[NM_173600.2(c.18734-123A>C)]	NA	NA	7.99E-03	NA	NA	7.99E-03	1.23E-02	NA	NA	NA	MODIFIER	NA	NA	144	142	0	95	50	44	46.32
Non-user	IOB_24	chr12	40917506	C	T	MUC19	INTRONIC	ENSG00000205592:[ENST00000454784(c.*5783-18858C>T)]	NA	NA	1.08E-02	NA	NA	1.08E-02	1.94E-02	NA	NA	NA	MODIFIER	NA	NA	95	95	0	76	32	43	56.58
Non-user	IOB_24	chr12	40925021	T	G	MUC19	MISSENSE	283463:[XM_003846356.2(c.21442T>G,Ttt>Gtt):XP_003846404.1(p.Phe7148Val)]	NA	NA	1.08E-02	2.96E-02	2.07E-02	1.08E-02	1.94E-02	NA	NA	NA	MODIFIER	NA	NA	113	113	0	98	54	44	44.9
Non-user	IOB_24	chr12	40939131	C	T	MUC19	NCT-SS-PRX	ENSG00000205592:[ENST00000460785(n.354-7C>T)]	NA	NA	7.79E-03	2.13E-02	1.61E-02	7.79E-03	1.23E-02	NA	NA	NA	LOW	NA	NA	181	180	1	107	55	52	48.6
Non-user	IOB_24	chr12	40939267	C	G	MUC19	INTRONIC	ENSG00000205592:[ENST00000454784(c.*6196+67C>G)]	NA	NA	1.46E-02	NA	NA	1.46E-02	1.94E-02	NA	NA	NA	MODIFIER	NA	NA	248	248	0	166	93	65	39.16
Non-user	IOB_24	chr12	40939691	G	T	MUC19	INTRONIC	283463:[NM_173600.2(c.22871-67G>T)]	NA	NA	2.32E-02	NA	NA	2.32E-02	1.94E-02	NA	NA	NA	MODIFIER	NA	NA	29	29	0	39	27	11	28.21
Non-user	IOB_24	chr12	40942460	T	C	MUC19	INTRONIC	283463:[XM_003846356.2(c.23186+27T>C)]	NA	NA	1.46E-02	3.22E-02	2.33E-02	1.46E-02	1.94E-02	NA	NA	NA	MODIFIER	NA	NA	149	144	0	110	62	46	41.82
Non-user	IOB_24	chr12	40951866	T	C	MUC19	NCT	ENSG00000205592:[ENST00000460785(n.1192-29T>C)]	NA	NA	1.76E-02	2.97E-02	2.07E-02	1.76E-02	1.94E-02	NA	NA	NA	MODIFIER	NA	NA	220	220	0	111	52	57	51.35
Non-user	IOB_24	chr12	40951890	G	A	MUC19	NCT-SS-PRX	ENSG00000205592:[ENST00000484665(n.30-5G>A)]	NA	NA	1.46E-02	2.89E-02	2.06E-02	1.46E-02	1.94E-02	NA	NA	NA	LOW	NA	NA	267	267	0	148	77	70	47.3
Non-user	IOB_24	chr12	40957375	C	T	MUC19	INTRONIC	ENSG00000205592:[ENST00000380816(c.1430+13C>T)]	NA	NA	1.06E-02	2.66E-02	2.08E-02	1.06E-02	1.94E-02	NA	NA	NA	MODIFIER	NA	NA	366	364	1	306	178	127	41.5
Non-user	IOB_24	chr12	41303735	T	C	CNTN1	INTRONIC	ENSG00000018236:[ENST00000348761(c.61+1440T>C)]	NA	NA	NA	NA	NA	NA	NA	NA	NA	NA	MODIFIER	NA	NA	52	52	0	32	23	9	28.12
Non-user	IOB_24	chr12	41587983	T	A	PDZRN4	MISSENSE	ENSG00000165966:[ENST00000402685(c.836T>A,aTc>aAc):ENSP00000384197(p.Ile279Asn)]	NA	NA	2.74E-02	1.75E-02	5.08E-03	2.74E-02	1.02E-02	D	PoD	NA	MODERATE	NA	NA	40	40	0	50	26	24	48
Non-user	IOB_24	chr12	43846199	G	T	ADAMTS20	MISSENSE	ENSG00000173157:[ENST00000389420(c.1957C>A,Cgt>Agt):ENSP00000374071(p.Arg653Ser)]	NA	chr12*43846199*43846199*COSM142263*G*A*1*skin*1|chr12*43846199*43846199*COSM142264*G*A*1*skin*1	3.20E-03	1.65E-03	7.90E-04	3.19E-03	2.00E-03	D	PrD	NA	MODERATE	D	1	118	117	0	108	56	51	47.22
Non-user	IOB_24	chr12	44148773	A	C	PUS7L	MISSENSE	ENSG00000129317:[ENST00000344862(c.276T>G,atT>atG):ENSP00000343081(p.Ile92Met)]	NA	NA	1.58E-02	2.96E-02	3.85E-02	1.58E-02	3.58E-02	NA	NA	NA	MODIFIER	NA	NA	126	126	0	105	48	57	54.29
Non-user	IOB_24	chr12	44196311	T	C	TWF1	INTRONIC	ENSG00000151239:[ENST00000325127(c.206-44A>G)]	NA	NA	NA	2.69E-04	0.00E+00	NA	NA	NA	NA	NA	MODIFIER	NA	NA	28	28	0	44	30	14	31.82
Non-user	IOB_24	chr12	46328096	A	G	SCAF11	INTRONIC	9169:[XM_005269229.1(c.745-28T>C)]	NA	NA	4.79E-03	3.91E-03	1.91E-02	4.79E-03	2.35E-02	NA	NA	NA	MODIFIER	NA	NA	35	35	0	27	15	12	44.44
Non-user	IOB_24	chr12	48105506	T	C	ENDOU	MISSENSE	ENSG00000111405:[ENST00000422538(c.1025A>G,tAt>tGt):ENSP00000397679(p.Tyr342Cys)]	NA	NA	3.99E-04	6.61E-04	4.38E-03	3.99E-04	2.00E-03	D	PrD	NA	MODERATE	D	1	75	75	0	60	31	29	48.33
Non-user	IOB_24	chr12	48105506	T	C	RP1-197B17.3	NCT	ENSG00000257433:[ENST00000547799(n.79+5560T>C)]	NA	NA	3.99E-04	6.61E-04	4.38E-03	3.99E-04	2.00E-03	D	PrD	NA	MODERATE	D	1	75	75	0	60	31	29	48.33
Non-user	IOB_24	chr12	48131794	C	T	RAPGEF3	NCT	ENSG00000079337:[ENST00000479866(n.3487+11G>A)]	NA	NA	6.79E-03	4.59E-03	3.27E-02	6.79E-03	3.27E-02	NA	NA	NA	MODIFIER	NA	NA	45	42	0	45	16	27	60
Non-user	IOB_24	chr12	48131794	C	T	RP1-197B17.3	NCT	ENSG00000257433:[ENST00000547799(n.451-904C>T)]	NA	NA	6.79E-03	4.59E-03	3.27E-02	6.79E-03	3.27E-02	NA	NA	NA	MODIFIER	NA	NA	45	42	0	45	16	27	60
Non-user	IOB_24	chr12	48134664	G	A	RAPGEF3	NCT	ENSG00000079337:[ENST00000473777(n.520+34C>T)]	NA	NA	0.00E+00	1.85E-04	1.03E-03	0.00E+00	0.00E+00	NA	NA	NA	MODIFIER	NA	NA	300	299	1	304	138	163	53.62
Non-user	IOB_24	chr12	48134664	G	A	RP1-197B17.3	EXONIC-NC	ENSG00000257433:[ENST00000547799(n.1886G>A)]	NA	NA	0.00E+00	1.85E-04	1.03E-03	0.00E+00	0.00E+00	NA	NA	NA	MODIFIER	NA	NA	300	299	1	304	138	163	53.62
Non-user	IOB_24	chr12	48174222	C	T	SLC48A1	3UTR	ENSG00000211584:[ENST00000551301(c.*436C>T)]	NA	NA	NA	3.30E-05	6.06E-05	NA	NA	NA	NA	NA	MODIFIER	NA	NA	143	143	0	94	41	52	55.32

Patel *et al.* , 2021. Whole-exome sequencing analysis of oral squamous cell carcinoma delineated by tobacco usage habits
Supplementary Table 4. List of somatic single nucleotide variants identified using whole exome sequencing of OSCC patients

Cohort	Sample	Chromosome	Genomic position	Reference allele	Alternate allele	Genesymbol	Variant type	Variant description	OncoMD	COSMIC	dbSNP	ExAC	ExAC_SAS	1000G	1000G_SAS	SIFT	Polyphen2	CONDEL	VEP	LRT	PhastCon7Way	Total reads in Normal	Reference read support in Normal	Alternative read support in Normal	Total reads in Tumor	Reference read support in Tumor	Alternate read support in Tumor	Alternate allele frequency in Tumor
Non-user	IOB_24	chr12	48367186	G	A	COL2A1	3UTR	ENSG00000139219:[ENST00000380518(c.*4C>T)]	NA	NA	2.12E-02	1.02E-02	2.97E-03	2.12E-02	0.00E+00	NA	NA	NA	MODIFIER	NA	NA	101	101	0	96	46	50	52.08
Non-user	IOB_24	chr12	48388302	C	T	COL2A1	INTRONIC	ENSG00000139219:[ENST00000380518(c.763-42G>A)]	NA	NA	1.40E-03	3.95E-03	2.61E-03	1.40E-03	0.00E+00	NA	NA	NA	MODIFIER	NA	NA	63	62	0	45	20	25	55.56
Non-user	IOB_24	chr12	48391769	C	T	COL2A1	NCT	ENSG00000139219:[ENST00000474996(n.613+46G>A)]	NA	NA	1.86E-02	8.84E-03	2.85E-03	1.86E-02	0.00E+00	NA	NA	NA	MODIFIER	NA	NA	142	142	0	107	52	55	51.4
Non-user	IOB_24	chr12	48535080	G	T	PFKM	INTRONIC	ENSG00000152556:[ENST00000340802(c.1555-14G>T)]	NA	NA	2.12E-02	2.64E-02	5.87E-02	2.12E-02	5.83E-02	NA	NA	NA	MODIFIER	NA	NA	52	52	0	42	22	20	47.62
Non-user	IOB_24	chr12	48597049	C	T	OR10AD1	SILENT	ENSG00000172640:[ENST00000310248(c.27G>A,acG>acA):ENSP00000308689(p.Thr9(=))]	NA	NA	2.88E-02	1.35E-02	5.00E-03	2.88E-02	0.00E+00	NA	NA	NA	LOW	NA	NA	86	86	0	44	24	20	45.45
Non-user	IOB_24	chr12	49314917	G	C	CCDC65	INTRONIC	ENSG00000139537:[ENST00000266984(c.1180-34G>C)]	NA	NA	2.74E-02	4.86E-02	9.04E-02	2.74E-02	7.16E-02	NA	NA	NA	MODIFIER	NA	NA	50	50	0	48	26	22	45.83
Non-user	IOB_24	chr12	49314917	G	C	RP11-302B13.5	INTRONIC	ENSG00000272822:[ENST00000398092(c.385-17226C>G)]	NA	NA	2.74E-02	4.86E-02	9.04E-02	2.74E-02	7.16E-02	NA	NA	NA	MODIFIER	NA	NA	50	50	0	48	26	22	45.83
Non-user	IOB_24	chr12	49438283	G	A	KMT2D	SILENT	ENSG00000167548:[ENST00000301067(c.4986C>T,tgC>tgT):ENSP00000301067(p.Cys1662(=))]	NA	chr12*49438283*49438283*COSM1362047*G*A*1*large_intestine*1|chr12*49438283*49438283*COSM1362046*G*A*1*large_intestine*1	7.99E-04	1.82E-04	9.73E-04	7.99E-04	1.00E-03	NA	NA	NA	LOW	NA	0.988	282	281	1	241	126	114	47.3
Non-user	IOB_24	chr12	49720518	C	T	TROAP	INTRONIC	ENSG00000135451:[ENST00000547923(c.-108+15C>T)]	NA	NA	7.99E-04	4.55E-03	9.42E-03	7.99E-04	4.10E-03	NA	NA	NA	MODIFIER	NA	NA	89	88	0	57	28	29	50.88
Non-user	IOB_24	chr12	49730056	G	T	C1QL4	SILENT	ENSG00000186897:[ENST00000334221(c.205C>A,Cgg>Agg):ENSP00000335285(p.Arg69(=))]	NA	NA	4.39E-03	4.04E-02	3.13E-02	4.39E-03	4.10E-03	NA	NA	NA	LOW	NA	0.999	41	41	0	30	13	16	53.33
Non-user	IOB_24	chr12	49950251	C	G	KCNH3	MISSENSE	ENSG00000135519:[ENST00000257981(c.2567C>G,cCt>cGt):ENSP00000257981(p.Pro856Arg)]	NA	NA	3.24E-02	6.42E-02	6.84E-02	3.23E-02	6.75E-02	NA	NA	NA	MODIFIER	NA	NA	233	233	0	209	108	101	48.33
Non-user	IOB_24	chr12	49958202	C	T	MCRS1	INTRONIC	ENSG00000187778:[ENST00000546244(c.-17+62G>A)]	NA	NA	NA	NA	NA	NA	NA	NA	NA	NA	MODIFIER	NA	NA	123	122	0	129	71	58	44.96
Non-user	IOB_24	chr12	50146263	C	T	TMBIM6	MISSENSE	ENSG00000139644:[ENST00000423828(c.161C>T,aCg>aTg):ENSP00000389277(p.Thr54Met)]	NA	NA	3.99E-04	2.97E-04	1.76E-03	3.99E-04	1.00E-03	NA	NA	NA	MODIFIER	NA	NA	181	176	1	137	69	65	47.45
Non-user	IOB_24	chr12	50187579	C	A	NCKAP5L	MISSENSE	ENSG00000167566:[ENST00000433948(c.2339G>T,Gcc>Tcc):ENSP00000402619(p.Ala781Ser)]	NA	NA	3.00E-03	1.18E-02	8.98E-03	3.00E-03	1.00E-03	T	BN	NA	MODERATE	N	0.618	133	133	0	99	49	49	49.49
Non-user	IOB_24	chr12	50370755	G	A	AQP6	EXONIC-NC	363:[XR_245925.1(n.5065G>A)]	NA	NA	1.20E-03	3.63E-03	3.73E-03	1.20E-03	0.00E+00	NA	NA	NA	MODIFIER	NA	NA	108	108	0	104	65	39	37.5
Non-user	IOB_24	chr12	50370755	G	A	RACGAP1	MISSENSE	ENSG00000161800:[ENST00000548961(c.332C>T,tCt>tTt):ENSP00000446889(p.Ser111Phe)]	NA	NA	1.20E-03	3.63E-03	3.73E-03	1.20E-03	0.00E+00	NA	NA	NA	MODIFIER	NA	NA	108	108	0	104	65	39	37.5
Non-user	IOB_24	chr12	51442164	G	C	LETMD1	MISSENSE	ENSG00000050426:[ENST00000262055(c.25G>C,Gct>Cct):ENSP00000262055(p.Ala9Pro)]	NA	NA	NA	NA	NA	NA	NA	NA	NA	NA	MODIFIER	NA	NA	315	315	0	268	143	125	46.64
Non-user	IOB_24	chr12	52215106	C	G	FIGNL2	SILENT	401720:[NM_001013690.4(c.1092G>C,ggG>ggC):NP_001013712.4(p.Gly364(=))]	NA	NA	1.40E-03	3.80E-03	5.69E-03	1.40E-03	7.20E-03	NA	NA	NA	MODIFIER	NA	NA	280	279	0	225	93	132	58.67
Non-user	IOB_24	chr12	52215106	C	G	RP11-923I11.7	EXONIC-NC	ENSG00000261308:[ENST00000564840(n.1094G>C)]	NA	NA	1.40E-03	3.80E-03	5.69E-03	1.40E-03	7.20E-03	NA	NA	NA	MODIFIER	NA	NA	280	279	0	225	93	132	58.67
Non-user	IOB_24	chr12	52285090	G	A	ANKRD33	MISSENSE	ENSG00000167612:[ENST00000538991(c.578G>A,gGg>gAg):ENSP00000443722(p.Gly193Glu)]	NA	NA	7.79E-03	1.99E-02	8.71E-03	7.79E-03	5.10E-03	NA	NA	NA	MODIFIER	NA	NA	161	161	0	117	75	42	35.9
Non-user	IOB_24	chr12	52711715	G	A	KRT83	SILENT	ENSG00000170523:[ENST00000293670(c.633C>T,aaC>aaT):ENSP00000293670(p.Asn211(=))]	NA	NA	3.00E-03	2.03E-03	1.17E-02	3.00E-03	1.53E-02	NA	NA	NA	LOW	NA	0	78	78	0	106	53	53	50
Non-user	IOB_24	chr12	52843232	G	A	KRT6B	INTRONIC	ENSG00000185479:[ENST00000252252(c.1077+21C>T)]	NA	NA	1.18E-02	1.60E-02	6.00E-02	1.18E-02	4.81E-02	NA	NA	NA	MODIFIER	NA	NA	185	185	0	154	66	88	57.14
Non-user	IOB_24	chr12	52843932	T	A	KRT6B	INTRONIC	ENSG00000185479:[ENST00000252252(c.756-76A>T)]	NA	NA	9.98E-04	NA	NA	9.98E-04	4.10E-03	NA	NA	NA	MODIFIER	NA	NA	303	302	0	264	219	45	17.05
Non-user	IOB_24	chr12	52865535	A	G	KRT6C	INTRONIC	ENSG00000170465:[ENST00000252250(c.756-19T>C)]	NA	NA	3.99E-04	2.13E-03	4.85E-04	3.99E-04	1.00E-03	NA	NA	NA	MODIFIER	NA	NA	240	240	0	161	137	23	14.29
Non-user	IOB_24	chr12	52865544	A	T	KRT6C	INTRONIC	ENSG00000170465:[ENST00000252250(c.756-28T>A)]	NA	NA	5.99E-04	2.12E-03	7.87E-04	5.99E-04	2.00E-03	NA	NA	NA	MODIFIER	NA	NA	245	245	0	168	138	29	17.26
Non-user	IOB_24	chr12	52913668	C	T	KRT5	MISSENSE	ENSG00000186081:[ENST00000551275(c.308G>A,gGa>gAa):ENSP00000448041(p.Gly103Glu)]	NA	NA	2.66E-02	5.82E-02	5.09E-02	2.66E-02	4.81E-02	D	PoD	NA	MODERATE	D	0.994	139	139	0	89	40	49	55.06
Non-user	IOB_24	chr12	52942421	C	G	KRT71	INTRONIC	ENSG00000139648:[ENST00000267119(c.813+64G>C)]	NA	NA	7.99E-04	NA	NA	7.99E-04	1.00E-03	NA	NA	NA	MODIFIER	NA	NA	89	89	0	76	39	37	48.68
Non-user	IOB_24	chr12	52966465	C	G	KRT74	INTRONIC	ENSG00000170484:[ENST00000305620(c.472-14G>C)]	NA	NA	5.99E-04	8.55E-04	4.61E-03	5.99E-04	3.10E-03	NA	NA	NA	MODIFIER	NA	NA	133	133	0	100	56	44	44
Non-user	IOB_24	chr12	53074153	G	A	KRT1	5UTR	ENSG00000167768:[ENST00000252244(c.-21C>T)]	NA	NA	1.20E-03	1.14E-03	2.85E-03	1.20E-03	4.10E-03	NA	NA	NA	MODIFIER	NA	NA	131	131	0	93	38	55	59.14
Non-user	IOB_24	chr12	53170960	G	C	KRT76	MISSENSE	ENSG00000185069:[ENST00000332411(c.116C>G,gCt>gGt):ENSP00000330101(p.Ala39Gly)]	NA	NA	1.80E-03	5.13E-03	1.27E-03	1.80E-03	1.00E-03	T	BN	NA	MODERATE	N	0	130	129	0	105	60	44	41.9
Non-user	IOB_24	chr12	53298775	A	T	KRT8	SILENT	ENSG00000170421:[ENST00000552150(c.75T>A,ccT>ccA):ENSP00000449404(p.Pro25(=))]	NA	NA	4.21E-02	1.20E-01	5.49E-02	4.21E-02	2.45E-02	NA	NA	NA	MODIFIER	NA	NA	48	48	0	26	17	9	34.62
Non-user	IOB_24	chr12	53427597	C	G	EIF4B	SILENT	ENSG00000063046:[ENST00000416762(c.870C>G,ccC>ccG):ENSP00000412530(p.Pro290(=))]	NA	NA	2.14E-02	2.84E-02	1.08E-02	2.14E-02	8.20E-03	NA	NA	NA	LOW	NA	NA	87	87	0	45	29	16	35.56
Non-user	IOB_24	chr12	53427597	C	G	RP11-983P16.4	NCT	ENSG00000257337:[ENST00000552905(n.208+12148G>C)]	NA	NA	2.14E-02	2.84E-02	1.08E-02	2.14E-02	8.20E-03	NA	NA	NA	LOW	NA	NA	87	87	0	45	29	16	35.56
Non-user	IOB_24	chr12	53455109	C	T	TENC1	INTRONIC	ENSG00000111077:[ENST00000549700(c.3164-55C>T)]	NA	NA	2.14E-02	NA	NA	2.14E-02	1.23E-02	NA	NA	NA	MODIFIER	NA	NA	308	305	2	249	109	139	55.82
Non-user	IOB_24	chr12	53468819	T	C	SPRYD3	INTRONIC	ENSG00000167778:[ENST00000301463(c.371+60A>G)]	NA	NA	3.02E-02	NA	NA	3.02E-02	1.23E-02	NA	NA	NA	MODIFIER	NA	NA	50	49	0	53	30	23	43.4
Non-user	IOB_24	chr12	53566174	A	T	CSAD	MISSENSE	ENSG00000139631:[ENST00000379850(c.376T>A,caT>caA):ENSP00000369179(p.His126Gln)]	NA	NA	1.40E-03	3.19E-03	1.64E-03	1.40E-03	0.00E+00	NA	NA	NA	MODIFIER	NA	NA	119	119	0	80	46	33	41.25
Non-user	IOB_24	chr12	54936297	A	G	NCKAP1L	INTRONIC	ENSG00000123338:[ENST00000293373(c.3274-62A>G)]	NA	NA	NA	NA	NA	NA	NA	NA	NA	NA	MODIFIER	NA	NA	104	104	0	79	36	43	54.43
Non-user	IOB_24	chr12	55794531	A	G	OR6C65	SILENT	ENSG00000205328:[ENST00000379665(c.219A>G,acA>acG):ENSP00000368986(p.Thr73(=))]	NA	NA	2.80E-03	3.13E-03	1.52E-02	2.80E-03	1.33E-02	NA	NA	NA	LOW	NA	NA	116	115	0	86	52	34	39.53
Non-user	IOB_24	chr12	56090761	G	C	ITGA7	SILENT	ENSG00000135424:[ENST00000257879(c.1659C>G,ccC>ccG):ENSP00000257879(p.Pro553(=))]	NA	NA	2.20E-03	1.42E-03	4.56E-03	2.20E-03	7.20E-03	NA	NA	NA	LOW	NA	NA	159	159	0	129	69	60	46.51
Non-user	IOB_24	chr12	56435920	C	G	RPS26	5UTR	ENSG00000197728:[ENST00000356464(c.-31C>G)]	NA	NA	4.77E-02	6.70E-02	5.38E-02	4.77E-02	5.01E-02	NA	NA	NA	MODIFIER	NA	NA	193	193	0	187	88	99	52.94
Non-user	IOB_24	chr12	56487201	T	C	ERBB3	SILENT	ENSG00000065361:[ENST00000267101(c.1347T>C,atT>atC):ENSP00000267101(p.Ile449(=))]	NA	NA	4.95E-02	6.55E-02	5.20E-02	4.95E-02	4.81E-02	NA	NA	NA	LOW	NA	0.001	122	121	0	98	40	56	57.14
Non-user	IOB_24	chr12	56601565	T	G	RNF41	INTRONIC	ENSG00000181852:[ENST00000345093(c.499-32A>C)]	NA	NA	2.00E-04	3.38E-04	2.00E-03	2.00E-04	1.00E-03	NA	NA	NA	MODIFIER	NA	NA	103	102	0	95	47	48	50.53
Non-user	IOB_24	chr12	56618292	G	A	NABP2	SILENT	79035:[XM_005269147.1(c.12G>A,ccG>ccA):XP_005269204.1(p.Pro4(=))]	NA	NA	3.30E-02	NA	NA	3.29E-02	2.35E-02	NA	NA	NA	MODIFIER	NA	NA	86	86	0	72	43	29	40.28
Non-user	IOB_24	chr12	56826757	A	G	TIMELESS	NCT	8914:[NR_138471.1(n.748+53T>C)]	NA	NA	4.39E-03	NA	NA	4.39E-03	5.10E-03	NA	NA	NA	MODIFIER	NA	NA	43	39	0	36	14	21	58.33
Non-user	IOB_24	chr12	57498402	G	A	STAT6	NCT	ENSG00000166888:[ENST00000555641(n.1686-33C>T)]	NA	NA	1.80E-03	1.17E-03	4.69E-03	1.80E-03	6.10E-03	NA	NA	NA	MODIFIER	NA	NA	139	138	0	112	64	48	42.86
Non-user	IOB_24	chr12	57637532	A	G	STAC3	3UTR	ENSG00000185482:[ENST00000332782(c.*63T>C)]	NA	NA	9.98E-04	4.30E-03	7.24E-03	9.98E-04	4.10E-03	NA	NA	NA	MODIFIER	NA	NA	155	155	0	164	87	76	46.34
Non-user	IOB_24	chr12	57643420	C	T	R3HDM2	3UTR-SS-PRX	ENSG00000179912:[ENST00000413953(c.*182G>A)]	NA	NA	9.98E-04	1.72E-03	4.62E-03	9.98E-04	4.10E-03	NA	NA	NA	MODIFIER	NA	NA	64	64	0	50	28	22	44
Non-user	IOB_24	chr12	57643420	C	T	RP11-123K3.4	3UTR-SS-PRX	ENSG00000258830:[ENST00000548184(c.*1884G>A)]	NA	NA	9.98E-04	1.72E-03	4.62E-03	9.98E-04	4.10E-03	NA	NA	NA	MODIFIER	NA	NA	64	64	0	50	28	22	44
Non-user	IOB_24	chr12	57643420	C	T	STAC3	NCT	246329:[NR_104422.1(n.201+1356G>A)]	NA	NA	9.98E-04	1.72E-03	4.62E-03	9.98E-04	4.10E-03	NA	NA	NA	MODIFIER	NA	NA	64	64	0	50	28	22	44
Non-user	IOB_24	chr12	57861785	T	C	GLI1	SILENT	ENSG00000111087:[ENST00000528467(c.963T>C,taT>taC):ENSP00000434408(p.Tyr321(=))]	NA	NA	1.52E-02	2.66E-02	3.16E-02	1.52E-02	3.89E-02	NA	NA	NA	LOW	NA	0	158	158	0	136	67	68	50
Non-user	IOB_24	chr12	57976829	T	C	KIF5A	INTRONIC	ENSG00000155980:[ENST00000286452(c.2754-55T>C)]	NA	NA	4.19E-03	NA	NA	4.19E-03	1.02E-02	NA	NA	NA	MODIFIER	NA	NA	81	81	0	72	41	31	43.06
Non-user	IOB_24	chr12	58165375	T	C	METTL1	3UTR	ENSG00000037897:[ENST00000553125(c.*13A>G)]	NA	NA	2.00E-03	6.08E-03	6.46E-03	2.00E-03	7.20E-03	NA	NA	NA	MODIFIER	NA	NA	70	70	0	48	29	19	39.58
Non-user	IOB_24	chr12	58165375	T	C	METTL21B	SILENT	ENSG00000123427:[ENST00000548256(c.18T>C,acT>acC):ENSP00000447718(p.Thr6(=))]	NA	NA	2.00E-03	6.08E-03	6.46E-03	2.00E-03	7.20E-03	NA	NA	NA	MODIFIER	NA	NA	70	70	0	48	29	19	39.58
Non-user	IOB_24	chr12	64238726	T	C	SRGAP1	NCT	57522:[XR_245947.1(n.388+63T>C)]	NA	NA	1.78E-02	NA	NA	1.78E-02	8.20E-03	NA	NA	NA	MODIFIER	NA	NA	50	50	0	51	18	33	64.71
Non-user	IOB_24	chr12	66698658	A	G	HELB	MISSENSE	ENSG00000127311:[ENST00000247815(c.335A>G,tAc>tGc):ENSP00000247815(p.Tyr112Cys)]	NA	NA	1.40E-03	5.11E-04	3.63E-03	1.40E-03	7.20E-03	D	PrD	NA	MODERATE	NA	NA	198	197	0	193	116	76	39.38
Non-user	IOB_24	chr12	66800163	A	G	GRIP1	SILENT	ENSG00000155974:[ENST00000540433(c.1560T>C,aaT>aaC):ENSP00000446024(p.Asn520(=))]	NA	NA	NA	2.48E-05	1.82E-04	NA	NA	NA	NA	NA	LOW	NA	NA	391	389	0	307	178	129	42.02
Non-user	IOB_24	chr12	72026160	T	C	ZFC3H1	SILENT	ENSG00000133858:[ENST00000378743(c.2952A>G,aaA>aaG):ENSP00000368017(p.Lys984(=))]	NA	NA	4.39E-03	8.47E-03	6.36E-03	4.39E-03	5.10E-03	NA	NA	NA	LOW	NA	NA	238	238	0	188	83	105	55.85
Non-user	IOB_24	chr12	72097823	C	A	TMEM19	MISSENSE	ENSG00000139291:[ENST00000546795(c.493C>A,Cct>Act):ENSP00000450089(p.Pro165Thr)]	NA	NA	5.99E-04	2.80E-04	1.34E-04	5.99E-04	2.00E-03	NA	NA	NA	MODIFIER	NA	NA	64	63	0	59	32	27	45.76
Non-user	IOB_24	chr12	72314462	T	C	TBC1D15	NCT	64786:[NR_027449.1(n.1464-49T>C)]	NA	NA	1.82E-02	4.00E-02	4.56E-02	1.82E-02	2.76E-02	NA	NA	NA	MODIFIER	NA	NA	99	98	0	71	31	40	56.34
Non-user	IOB_24	chr12	75601443	G	A	KCNC2	SILENT	ENSG00000166006:[ENST00000298972(c.321C>T,caC>caT):ENSP00000298972(p.His107(=))]	NA	NA	4.67E-02	8.12E-02	4.50E-02	4.67E-02	3.17E-02	NA	NA	NA	LOW	NA	1	23	23	0	40	24	15	37.5
Non-user	IOB_24	chr12	76740149	G	A	BBS10	MISSENSE	ENSG00000179941:[ENST00000393262(c.1616C>T,cCa>cTa):ENSP00000376946(p.Pro539Leu)]	NA	NA	3.36E-02	6.47E-02	9.18E-02	3.35E-02	6.54E-02	T	BN	NA	MODERATE	N	0	257	256	0	210	123	87	41.43
Non-user	IOB_24	chr12	76804425	T	C	OSBPL8	INTRONIC	ENSG00000091039:[ENST00000550628(c.43-7879A>G)]	NA	NA	9.98E-04	3.66E-03	1.11E-03	9.98E-04	0.00E+00	NA	NA	NA	MODIFIER	NA	NA	48	47	0	30	10	19	63.33
Non-user	IOB_24	chr12	77158623	T	C	ZDHHC17	INTRONIC	ENSG00000186908:[ENST00000549682(c.24+31T>C)]	NA	NA	NA	1.86E-04	2.66E-04	NA	NA	NA	NA	NA	MODIFIER	NA	NA	188	186	0	143	70	70	48.95
Non-user	IOB_24	chr12	78538725	A	G	NAV3	INTRONIC	ENSG00000067798:[ENST00000552895(c.1367-1424A>G)]	NA	NA	NA	NA	NA	NA	NA	NA	NA	NA	MODIFIER	NA	NA	8	8	0	11	3	8	72.73
Non-user	IOB_24	chr12	78538728	G	A	NAV3	INTRONIC	ENSG00000067798:[ENST00000228327(c.4707+1531G>A)]	NA	NA	NA	NA	NA	NA	NA	NA	NA	NA	MODIFIER	NA	NA	9	9	0	13	3	10	76.92
Non-user	IOB_24	chr12	78582622	G	A	NAV3	NCT	ENSG00000067798:[ENST00000552300(n.363+499G>A)]	NA	NA	NA	NA	NA	NA	NA	NA	NA	NA	MODIFIER	NA	NA	24	22	0	26	13	13	50
Non-user	IOB_24	chr12	80613584	T	C	OTOGL	INTRONIC-SS-ACR-PRX	ENSG00000165899:[ENST00000458043(c.209-10T>C)]	NA	NA	NA	2.39E-03	1.01E-02	NA	NA	NA	NA	NA	MODIFIER	NA	NA	140	139	0	138	73	57	41.3
Non-user	IOB_24	chr12	86198594	A	G	RASSF9	SILENT	ENSG00000198774:[ENST00000361228(c.1194T>C,acT>acC):ENSP00000354884(p.Thr398(=))]	NA	NA	4.33E-02	6.05E-02	1.12E-02	4.33E-02	5.10E-03	NA	NA	NA	LOW	NA	NA	301	299	0	187	94	92	49.2
Non-user	IOB_24	chr12	86198935	C	T	RASSF9	MISSENSE	ENSG00000198774:[ENST00000361228(c.853G>A,Gct>Act):ENSP00000354884(p.Ala285Thr)]	NA	NA	4.33E-02	6.05E-02	1.12E-02	4.33E-02	5.10E-03	T	BN	NA	MODERATE	U	0.001	391	390	0	256	126	130	50.78
Non-user	IOB_24	chr12	94692478	T	A	PLXNC1	MISSENSE	ENSG00000136040:[ENST00000547057(c.1286T>A,tTt>tAt):ENSP00000446720(p.Phe429Tyr)]	NA	NA	1.80E-03	6.30E-03	6.12E-03	1.80E-03	5.10E-03	T	PoD	NA	MODERATE	N	0.996	80	80	0	78	33	45	57.69
Non-user	IOB_24	chr12	96077486	C	T	NTN4	SILENT-SS-PRX	ENSG00000074527:[ENST00000343702(c.1182G>A,ccG>ccA):ENSP00000340998(p.Pro394(=))]	NA	chr12*96077486*96077486*COSM163010*C*T*1*breast*1	1.20E-03	2.15E-03	9.08E-05	1.20E-03	0.00E+00	NA	NA	NA	LOW	NA	NA	47	47	0	33	20	13	39.39
Non-user	IOB_24	chr12	100552712	C	A	GOLGA2P5	NCT	ENSG00000238105:[ENST00000304813(n.1753+24G>T)]	NA	NA	4.11E-02	NA	NA	4.11E-02	8.69E-02	NA	NA	NA	MODIFIER	NA	NA	135	134	1	108	44	63	58.33
Non-user	IOB_24	chr12	101573812	A	G	SLC5A8	SILENT	ENSG00000256870:[ENST00000536262(c.1228T>C,Ttg>Ctg):ENSP00000445340(p.Leu410(=))]	NA	NA	1.46E-02	2.72E-02	4.54E-02	1.46E-02	3.78E-02	NA	NA	NA	LOW	NA	0.982	70	69	0	45	23	22	48.89
Non-user	IOB_24	chr12	102147142	G	T	GNPTAB	INTRONIC-SS-DNR-PRX	ENSG00000111670:[ENST00000549738(c.*209+8C>A)]	NA	NA	9.39E-03	3.40E-02	4.97E-03	9.38E-03	3.10E-03	NA	NA	NA	LOW	NA	NA	110	110	0	109	54	55	50.46
Non-user	IOB_24	chr12	103232934	C	A	PAH	3UTR	ENSG00000171759:[ENST00000307000(c.*19G>T)]	NA	NA	2.60E-03	2.16E-03	7.83E-03	2.60E-03	1.02E-02	NA	NA	NA	MODIFIER	NA	NA	261	261	0	206	121	84	40.78
Non-user	IOB_24	chr12	103558242	A	G	LOC101929058	NCT	101929058:[NR_126333.1(n.582+27A>G)]	NA	NA	NA	NA	NA	NA	NA	NA	NA	NA	MODIFIER	NA	NA	171	171	0	150	143	7	4.67
Non-user	IOB_24	chr12	103558242	A	G	RP11-328J6.1	NCT	ENSG00000257703:[ENST00000548594(n.167+13665T>C)]	NA	NA	NA	NA	NA	NA	NA	NA	NA	NA	MODIFIER	NA	NA	171	171	0	150	143	7	4.67
Non-user	IOB_24	chr12	103558242	A	G	RP11-552I14.1	NCT	ENSG00000257860:[ENST00000550664(n.75+27A>G)]	NA	NA	NA	NA	NA	NA	NA	NA	NA	NA	MODIFIER	NA	NA	171	171	0	150	143	7	4.67
Non-user	IOB_24	chr12	104054133	G	A	STAB2	MISSENSE	ENSG00000136011:[ENST00000388887(c.1759G>A,Gtc>Atc):ENSP00000373539(p.Val587Ile)]	NA	NA	2.00E-04	4.04E-04	2.67E-03	2.00E-04	0.00E+00	T	BN	NA	MODERATE	N	0.03	432	431	1	344	168	174	50.58
Non-user	IOB_24	chr12	105550565	C	T	KIAA1033	SILENT	ENSG00000136051:[ENST00000332180(c.2820C>T,gcC>gcT):ENSP00000328062(p.Ala940(=))]	NA	NA	4.33E-02	7.27E-02	7.93E-02	4.33E-02	6.75E-02	NA	NA	NA	LOW	NA	NA	69	67	0	67	37	29	43.28
Non-user	IOB_24	chr12	105550565	C	T	WASHC4	SILENT	23325:[NM_001293640.1(c.2823C>T,gcC>gcT):NP_001280569.1(p.Ala941(=))]	NA	NA	4.33E-02	7.27E-02	7.93E-02	4.33E-02	6.75E-02	NA	NA	NA	LOW	NA	NA	69	67	0	67	37	29	43.28
Non-user	IOB_24	chr12	105570796	T	G	APPL2	INTRONIC-SS-ACR	ENSG00000136044:[ENST00000547790(n.231-2A>C)]	NA	NA	NA	1.66E-05	0.00E+00	NA	NA	NA	NA	NA	HIGH	NA	NA	170	170	0	162	65	96	59.26
Non-user	IOB_24	chr12	106460763	G	A	NUAK1	SILENT	ENSG00000074590:[ENST00000261402(c.1803C>T,tgC>tgT):ENSP00000261402(p.Cys601(=))]	NA	NA	3.99E-04	4.88E-04	2.30E-03	3.99E-04	2.00E-03	NA	NA	NA	LOW	NA	0.008	184	183	0	126	70	54	42.86
Non-user	IOB_24	chr12	106723132	C	A	TCP11L2	NONSENSE	ENSG00000166046:[ENST00000547153(c.797C>A,tCa>tAa):ENSP00000448952(p.Ser266Ter)]	NA	NA	NA	NA	NA	NA	NA	NA	NA	NA	MODIFIER	NA	NA	55	55	0	59	53	6	10.17
Non-user	IOB_24	chr12	108910962	C	T	FICD	SILENT	ENSG00000198855:[ENST00000361549(c.213C>T,gcC>gcT):ENSP00000354421(p.Ala71(=))]	NA	NA	3.97E-02	4.02E-02	7.63E-02	3.97E-02	8.69E-02	NA	NA	NA	LOW	NA	NA	189	188	1	152	82	70	46.05
Non-user	IOB_24	chr12	108941700	C	G	SART3	SILENT	ENSG00000075856:[ENST00000228284(c.507G>C,gtG>gtC):ENSP00000228284(p.Val169(=))]	NA	NA	4.99E-02	4.20E-02	6.93E-02	4.99E-02	8.59E-02	NA	NA	NA	MODIFIER	NA	NA	103	101	0	61	34	27	44.26

Patel *et al.* , 2021. Whole-exome sequencing analysis of oral squamous cell carcinoma delineated by tobacco usage habits
Supplementary Table 4. List of somatic single nucleotide variants identified using whole exome sequencing of OSCC patients

Cohort	Sample	Chromosome	Genomic position	Reference allele	Alternate allele	Genesymbol	Variant type	Variant description	OncoMD	COSMIC	dbSNP	ExAC	ExAC_SAS	1000G	1000G_SAS	SIFT	Polyphen2	CONDEL	VEP	LRT	PhastCon7Way	Total reads in Normal	Reference read support in Normal	Alternative read support in Normal	Total reads in Tumor	Reference read support in Tumor	Alternate read support in Tumor	Alternate allele frequency in Tumor
Non-user	IOB_24	chr12	109338984	C	A	SVOP	INTRONIC	ENSG00000166111:[ENST00000550436(c.*79-42G>T)]	NA	NA	NA	NA	NA	NA	NA	NA	NA	NA	MODIFIER	NA	NA	106	106	0	98	43	55	56.12
Non-user	IOB_24	chr12	109635024	A	T	ACACB	NCT	32:[XR_243001.1(n.2924+66A>T)]	NA	NA	NA	NA	NA	NA	NA	NA	NA	NA	MODIFIER	NA	NA	91	91	0	92	63	29	31.52
Non-user	IOB_24	chr12	109696150	A	T	ACACB	MISSENSE	ENSG00000076555:[ENST00000538526(c.2340A>T,aAc>aTc):ENSP00000443281(p.Asn781Ile)]	NA	NA	chr12*109696150*109696150*A*G*rs754757968*NA*NA*NA	NA	NA	NA	NA	D	PrD	NA	MODERATE	D	1	28	28	0	11	1	10	90.91
Non-user	IOB_24	chr12	109838853	T	C	MYO1H	INTRONIC	ENSG00000174527:[ENST00000310903(c.523-45T>C)]	NA	NA	9.98E-04	4.26E-04	3.74E-03	9.98E-04	5.10E-03	NA	NA	NA	MODIFIER	NA	NA	157	154	0	124	59	65	52.42
Non-user	IOB_24	chr12	110206760	C	A	FAM222A	SILENT	ENSG00000139438:[ENST00000358906(c.1026C>A,ggC>ggA):ENSP00000351783(p.Gly342(=))]	NA	NA	5.79E-03	1.30E-02	1.28E-02	5.79E-03	9.20E-03	NA	NA	NA	MODIFIER	NA	NA	207	206	0	155	75	80	51.61
Non-user	IOB_24	chr12	110206760	C	A	FAM222A-AS1	NCT	ENSG00000255650:[ENST00000541460(n.188+4342G>T)]	NA	NA	5.79E-03	1.30E-02	1.28E-02	5.79E-03	9.20E-03	NA	NA	NA	MODIFIER	NA	NA	207	206	0	155	75	80	51.61
Non-user	IOB_24	chr12	110224551	C	A	TRPV4	MISSENSE	ENSG00000111199:[ENST00000392719(c.2159G>T,aGc>aTc):ENSP00000376480(p.Ser720Ile)]	NA	NA	NA	NA	NA	NA	NA	D	BN	NA	MODERATE	D	0.999	132	132	0	111	105	5	4.5
Non-user	IOB_24	chr12	110252547	G	A	TRPV4	MISSENSE	ENSG00000111199:[ENST00000261740(c.55C>T,Ccc>Tcc):ENSP00000261740(p.Pro19Ser)]	NA	NA	4.35E-02	5.21E-02	6.55E-02	4.35E-02	5.01E-02	T_lc	BN	NA	MODERATE	U	0.967	74	74	0	66	37	29	43.94
Non-user	IOB_24	chr12	111729274	C	T	CUX2	SILENT	ENSG00000111249:[ENST00000261726(c.354C>T,ccC>ccT):ENSP00000261726(p.Pro118(=))]	NA	NA	NA	8.31E-04	3.97E-03	NA	NA	NA	NA	NA	LOW	NA	NA	108	108	0	57	35	22	38.6
Non-user	IOB_24	chr12	111733292	G	A	CUX2	INTRONIC	ENSG00000111249:[ENST00000261726(c.637+79G>A)]	NA	NA	4.31E-02	NA	NA	4.31E-02	6.85E-02	NA	NA	NA	MODIFIER	NA	NA	143	143	0	112	57	55	49.11
Non-user	IOB_24	chr12	111746300	C	A	CUX2	MISSENSE	ENSG00000111249:[ENST00000261726(c.1228C>A,Ctg>Atg):ENSP00000261726(p.Leu410Met)]	NA	NA	NA	NA	NA	NA	NA	T	PoD	NA	MODERATE	N	0.543	173	173	0	127	55	70	55.12
Non-user	IOB_24	chr12	111958691	C	G	ATXN2	SILENT	ENSG00000204842:[ENST00000608853(c.783G>C,tcG>tcC):ENSP00000476504(p.Ser261(=))]	NA	NA	1.20E-03	5.75E-04	3.70E-03	1.20E-03	6.10E-03	NA	NA	NA	LOW	NA	NA	62	62	0	53	29	24	45.28
Non-user	IOB_24	chr12	112213500	G	A	ALDH2	MISSENSE	ENSG00000111275:[ENST00000548536(c.206G>A,cGc>cAc):ENSP00000448179(p.Arg69His)]	NA	NA	NA	NA	NA	NA	NA	NA	NA	NA	MODIFIER	NA	NA	77	77	0	41	22	19	46.34
Non-user	IOB_24	chr12	112213500	G	A	RP11-162P23.2	INTRONIC	ENSG00000257767:[ENST00000546840(c.105-6222G>A)]	NA	NA	NA	NA	NA	NA	NA	NA	NA	NA	MODIFIER	NA	NA	77	77	0	41	22	19	46.34
Non-user	IOB_24	chr12	112506806	G	A	NAA25	NCT	80018:[XR_243023.1(n.1286-10C>T)]	NA	NA	NA	3.68E-04	1.65E-03	NA	NA	NA	NA	NA	MODIFIER	NA	NA	96	96	0	76	42	33	43.42
Non-user	IOB_24	chr12	113724956	C	T	TPCN1	NCT	ENSG00000186815:[ENST00000551127(n.608+57C>T)]	NA	NA	2.00E-03	NA	NA	2.00E-03	1.02E-02	NA	NA	NA	MODIFIER	NA	NA	105	102	0	73	43	30	41.1
Non-user	IOB_24	chr12	116972636	C	T	LINC00173	NCT	ENSG00000196668:[ENST00000470091(n.182+1215C>T)]	NA	NA	2.20E-03	1.10E-03	7.93E-03	2.20E-03	1.12E-02	NA	NA	NA	MODIFIER	NA	NA	166	165	0	108	45	62	57.41
Non-user	IOB_24	chr12	117964908	A	G	KSR2	SILENT	ENSG00000171435:[ENST00000302438(c.910T>C,Tta>Cta):ENSP00000305466(p.Leu304(=))]	NA	NA	NA	NA	NA	NA	NA	NA	NA	NA	LOW	NA	1	210	209	0	153	90	61	39.87
Non-user	IOB_24	chr12	119968695	T	G	CCDC60	MISSENSE	ENSG00000183273:[ENST00000327554(c.1378T>G,Tcc>Gcc):ENSP00000333374(p.Ser460Ala)]	NA	NA	4.39E-03	2.61E-03	1.96E-02	4.39E-03	2.15E-02	NA	NA	NA	MODIFIER	NA	NA	52	52	0	59	27	32	54.24
Non-user	IOB_24	chr12	119968695	T	G	RP11-768F21.1	NCT	ENSG00000248636:[ENST00000537366(n.235+22658A>C)]	NA	NA	4.39E-03	2.61E-03	1.96E-02	4.39E-03	2.15E-02	NA	NA	NA	MODIFIER	NA	NA	52	52	0	59	27	32	54.24
Non-user	IOB_24	chr12	120146164	G	A	CIT	NCT	ENSG00000122966:[ENST00000543324(n.264-41C>T)]	NA	NA	2.66E-02	2.14E-02	1.34E-02	2.66E-02	1.43E-02	NA	NA	NA	MODIFIER	NA	NA	86	85	0	76	37	39	51.32
Non-user	IOB_24	chr12	120148363	C	T	CIT	SILENT	ENSG00000122966:[ENST00000392520(c.3629G>A,acG>acA):ENSP00000376305(p.Thr1210(=))]	NA	NA	2.70E-02	2.06E-02	1.26E-02	2.70E-02	1.43E-02	NA	NA	NA	LOW	NA	NA	76	76	0	71	35	35	49.3
Non-user	IOB_24	chr12	120156001	C	T	CIT	INTRONIC	11113:[XM_005253828.1(c.2725+49G>A)]	NA	NA	1.18E-02	1.67E-02	1.23E-02	1.18E-02	1.23E-02	NA	NA	NA	MODIFIER	NA	NA	117	117	0	57	27	30	52.63
Non-user	IOB_24	chr12	120901765	G	A	SRSF9	SILENT	ENSG00000111786:[ENST00000229390(c.510C>T,ttC>ttT):ENSP00000229390(p.Phe170(=))]	NA	NA	2.80E-03	1.05E-02	4.41E-03	2.80E-03	2.00E-03	NA	NA	NA	LOW	NA	0.985	222	220	0	167	85	82	49.1
Non-user	IOB_24	chr12	121678298	G	A	CAMKK2	SILENT	ENSG00000110931:[ENST00000392474(c.1644C>T,gcC>gcT):ENSP00000376266(p.Ala548(=))]	NA	NA	8.79E-03	2.77E-02	2.78E-02	8.79E-03	1.43E-02	NA	NA	NA	MODIFIER	NA	NA	98	96	0	82	42	39	47.56
Non-user	IOB_24	chr12	124110980	A	G	EIF2B1	SILENT	ENSG00000111361:[ENST00000539951(c.504T>C,gcT>gcC):ENSP00000438060(p.Ala168(=))]	NA	NA	NA	1.57E-04	1.09E-03	NA	NA	NA	NA	NA	LOW	NA	NA	149	148	0	99	46	53	53.54
Non-user	IOB_24	chr12	124815504	C	T	NCOR2	INTRONIC	9612:[XM_005253653.1(c.5162-60G>A)]	NA	NA	4.15E-02	NA	NA	4.15E-02	9.10E-02	NA	NA	NA	MODIFIER	NA	NA	106	105	0	105	51	53	50.48
Non-user	IOB_24	chr12	124825026	C	T	NCOR2	INTRONIC	9612:[XM_005253653.1(c.3482-37G>A)]	NA	NA	2.00E-04	5.52E-04	4.00E-03	2.00E-04	1.00E-03	NA	NA	NA	MODIFIER	NA	NA	169	169	0	122	63	57	46.72
Non-user	IOB_24	chr12	125451838	G	A	DHX37	INTRONIC	ENSG00000150990:[ENST00000544745(c.770-74C>T)]	NA	NA	2.28E-02	NA	NA	2.28E-02	2.56E-02	NA	NA	NA	MODIFIER	NA	NA	102	101	0	98	53	45	45.92
Non-user	IOB_24	chr12	125457038	C	G	DHX37	NCT	ENSG00000150990:[ENST00000539298(n.1177+11G>C)]	NA	NA	3.20E-03	4.60E-03	1.02E-02	3.19E-03	8.20E-03	NA	NA	NA	MODIFIER	NA	NA	254	253	0	167	89	78	46.71
Non-user	IOB_24	chr12	129154069	G	A	TMEM132C	SILENT	ENSG00000181234:[ENST00000315208(c.261G>A,tcG>tcA):ENSP00000324458(p.Ser87(=))]	NA	NA	5.79E-03	8.77E-03	1.37E-02	5.79E-03	7.20E-03	NA	NA	NA	LOW	NA	NA	107	106	1	74	46	28	37.84
Non-user	IOB_24	chr12	130927184	C	T	RIMBP2	MISSENSE	ENSG00000060709:[ENST00000261655(c.662G>A,aGg>aAg):ENSP00000261655(p.Arg221Lys)]	NA	chr12*130927184*130927184*COSM1720262*C*T*2*NS*2	3.99E-04	1.93E-04	1.42E-03	3.99E-04	2.00E-03	NA	NA	NA	MODERATE	NA	1	196	195	0	220	93	124	56.36
Non-user	IOB_24	chr12	131555415	C	T	GPR133	MISSENSE	ENSG00000111452:[ENST00000335486(c.18C>T,Cgg>Tgg):ENSP00000334127(p.Arg7Trp)]	NA	NA	9.19E-03	4.07E-03	1.66E-02	9.19E-03	2.25E-02	NA	NA	NA	MODIFIER	NA	NA	124	124	0	90	44	46	51.11
Non-user	IOB_24	chr12	133102403	C	T	FBRSL1	SILENT	ENSG00000112787:[ENST00000434748(c.573C>T,ctC>ctT):ENSP00000396160(p.Leu191(=))]	NA	NA	3.47E-02	2.38E-02	2.46E-02	3.47E-02	2.04E-02	NA	NA	NA	LOW	NA	NA	136	136	0	130	65	64	49.23
Non-user	IOB_24	chr12	133127964	G	A	FBRSL1	INTRONIC	57666:[XM_005266179.1(c.348+3346G>A)]	NA	NA	3.16E-02	1.99E-02	2.36E-02	3.15E-02	2.56E-02	NA	NA	NA	MODIFIER	NA	NA	135	135	0	92	46	46	50
Non-user	IOB_24	chr12	133128069	G	A	FBRSL1	INTRONIC	57666:[XM_005266179.1(c.348+3451G>A)]	NA	NA	3.12E-02	1.85E-02	2.19E-02	3.12E-02	2.45E-02	NA	NA	NA	MODIFIER	NA	NA	302	302	0	247	122	124	50.2
Non-user	IOB_24	chr12	133144164	G	A	FBRSL1	NCT	ENSG00000112787:[ENST00000543360(n.124+52G>A)]	NA	NA	4.03E-02	NA	NA	4.03E-02	4.40E-02	NA	NA	NA	MODIFIER	NA	NA	175	172	0	180	83	97	53.89
Non-user	IOB_24	chr12	133146701	C	T	FBRSL1	MISSENSE	ENSG00000112787:[ENST00000261673(c.662C>T,cCg>cTg):ENSP00000261673(p.Pro221Leu)]	NA	NA	3.71E-02	2.75E-02	3.04E-02	3.71E-02	2.76E-02	NA	NA	NA	MODERATE	NA	NA	153	153	0	163	81	79	48.47
Non-user	IOB_24	chr12	133146732	G	A	FBRSL1	SILENT	ENSG00000112787:[ENST00000261673(c.693G>A,ccG>ccA):ENSP00000261673(p.Pro231(=))]	NA	NA	3.71E-02	2.87E-02	3.19E-02	3.71E-02	2.76E-02	NA	NA	NA	LOW	NA	NA	139	138	0	148	68	78	52.7
Non-user	IOB_24	chr12	133149056	G	A	FBRSL1	INTRONIC	57666:[XM_005266173.1(c.1552-57G>A)]	NA	NA	1.36E-02	NA	NA	1.36E-02	1.02E-02	NA	NA	NA	MODIFIER	NA	NA	338	336	0	222	93	128	57.66
Non-user	IOB_24	chr12	133149168	C	T	FBRSL1	MISSENSE	ENSG00000112787:[ENST00000261673(c.1325C>T,cCc>cTc):ENSP00000261673(p.Pro442Leu)]	NA	chr12*133149168*133149168*COSM147638*C*T*1*stomach*1	2.00E-02	1.12E-02	9.75E-03	2.00E-02	6.10E-03	NA	NA	NA	MODERATE	NA	1	243	243	0	188	96	91	48.4
Non-user	IOB_24	chr12	133158097	T	C	FBRSL1	MISSENSE	ENSG00000112787:[ENST00000261673(c.1817T>C,cTg>cCg):ENSP00000261673(p.Leu606Pro)]	NA	NA	3.75E-02	2.02E-02	2.59E-02	3.75E-02	2.76E-02	NA	NA	NA	MODERATE	NA	1	94	94	0	75	36	39	52
Non-user	IOB_24	chr12	133428181	C	T	CHFR	NCT	55743:[XR_245348.1(n.1593+23G>A)]	NA	NA	2.12E-02	3.06E-02	3.07E-02	2.12E-02	2.45E-02	NA	NA	NA	MODIFIER	NA	NA	301	300	0	218	134	84	38.53
Non-user	IOB_24	chr12	133617991	G	A	ZNF84	EXONIC-NC	ENSG00000198040:[ENST00000539098(n.171G>A)]	NA	NA	NA	NA	NA	NA	NA	NA	NA	NA	MODIFIER	NA	NA	115	114	0	84	36	47	55.95
Non-user	IOB_24	chr12	133618022	G	A	ZNF84	EXONIC-NC	ENSG00000198040:[ENST00000441040(n.159G>A)]	NA	NA	NA	NA	NA	NA	NA	NA	NA	NA	MODIFIER	NA	NA	140	139	0	102	44	56	54.9
Non-user	IOB_24	chr12	133625276	A	T	ZNF84	INTRONIC	ENSG00000198040:[ENST00000327668(c.143-63A>T)]	NA	NA	chr12*133625276*133625276*A*G,T*rs7314140*NA*NA*NA	NA	NA	NA	NA	NA	NA	NA	MODIFIER	NA	NA	76	76	0	74	40	34	45.95
Non-user	IOB_24	chr12	133625292	A	T	ZNF84	EXONIC-NC	ENSG00000198040:[ENST00000543124(n.1086A>T)]	NA	NA	NA	NA	NA	NA	NA	NA	NA	NA	MODIFIER	NA	NA	89	89	0	82	44	38	46.34
Non-user	IOB_24	chr12	133677542	T	C	ZNF140	INTRONIC	7699:[NM_001300778.1(c.-144-31T>C)]	NA	NA	NA	NA	NA	NA	NA	NA	NA	NA	MODIFIER	NA	NA	24	24	0	37	15	22	59.46
Non-user	IOB_24	chr13	19775784	C	T	PSPC1P1	EXONIC-NC	ENSG00000227879:[ENST00000442352(n.658G>A)]	NA	NA	4.49E-02	NA	NA	4.49E-02	8.59E-02	NA	NA	NA	MODIFIER	NA	NA	117	115	0	93	47	45	48.39
Non-user	IOB_24	chr13	19778601	G	A	PSPC1P1	EXONIC-NC	ENSG00000227879:[ENST00000442352(n.358C>T)]	NA	NA	NA	NA	NA	NA	NA	NA	NA	NA	MODIFIER	NA	NA	122	109	11	10	0	10	100
Non-user	IOB_24	chr13	20233980	G	A	MPHOSPH8	NCT	54737:[XR_245390.1(n.1893+551G>A)]	NA	NA	1.38E-02	NA	NA	1.38E-02	6.44E-02	NA	NA	NA	MODIFIER	NA	NA	431	425	1	321	145	175	54.52
Non-user	IOB_24	chr13	21199902	G	T	IFT88	MISSENSE	ENSG00000032742:[ENST00000319980(c.1240G>T,Gtg>Ttg):ENSP00000323580(p.Val414Leu)]	NA	NA	3.00E-03	2.48E-03	1.74E-02	3.00E-03	1.43E-02	NA	NA	NA	MODERATE	NA	1	40	40	0	34	21	13	38.24
Non-user	IOB_24	chr13	23253392	G	A	NA	INTERGENIC	NA	NA	NA	2.56E-02	NA	NA	2.56E-02	3.89E-02	NA	NA	NA	MODIFIER	NA	NA	189	188	0	170	94	74	43.53
Non-user	IOB_24	chr13	24868919	T	C	RP11-307N16.6	SILENT	ENSG00000273167:[ENST00000382141(c.2757T>C,aaT>aaC):ENSP00000371576(p.Asn919(=))]	NA	NA	2.90E-02	3.03E-02	1.44E-02	2.90E-02	8.20E-03	NA	NA	NA	LOW	NA	0.001	167	167	0	134	70	64	47.76
Non-user	IOB_24	chr13	24868919	T	C	SPATA13	SILENT	ENSG00000182957:[ENST00000434675(c.942T>C,aaT>aaC):ENSP00000401605(p.Asn314(=))]	NA	NA	2.90E-02	3.03E-02	1.44E-02	2.90E-02	8.20E-03	NA	NA	NA	LOW	NA	0.001	167	167	0	134	70	64	47.76
Non-user	IOB_24	chr13	25078009	A	G	PARP4	INTRONIC	ENSG00000102699:[ENST00000381989(c.-1-94T>C)]	NA	NA	NA	NA	NA	NA	NA	NA	NA	NA	MODIFIER	NA	NA	101	101	0	105	48	57	54.29
Non-user	IOB_24	chr13	25078009	A	G	TPTE2P6	NCT	ENSG00000205822:[ENST00000445572(n.234-77790A>G)]	NA	NA	NA	NA	NA	NA	NA	NA	NA	NA	MODIFIER	NA	NA	101	101	0	105	48	57	54.29
Non-user	IOB_24	chr13	27259809	C	T	WASF3	INTRONIC	ENSG00000132970:[ENST00000361042(c.1343-16C>T)]	NA	NA	2.06E-02	1.37E-02	5.15E-02	2.06E-02	7.06E-02	NA	NA	NA	MODIFIER	NA	NA	110	110	0	88	50	38	43.18
Non-user	IOB_24	chr13	28009247	A	C	GTF3A	INTRONIC	ENSG00000122034:[ENST00000381140(c.874-23A>C)]	NA	NA	1.38E-02	2.27E-02	3.75E-02	1.38E-02	2.76E-02	NA	NA	NA	MODIFIER	NA	NA	70	68	1	53	19	34	64.15
Non-user	IOB_24	chr13	28498656	G	A	PDX1	MISSENSE	ENSG00000139515:[ENST00000381033(c.670G>A,Gag>Aag):ENSP00000370421(p.Glu224Lys)]	NA	NA	3.20E-03	2.11E-03	1.28E-02	3.19E-03	1.64E-02	T	BN	NA	MODERATE	D	1	54	54	0	50	24	26	52
Non-user	IOB_24	chr13	28896979	C	T	FLT1	SILENT	ENSG00000102755:[ENST00000282397(c.2901G>A,gcG>gcA):ENSP00000282397(p.Ala967(=))]	NA	NA	4.47E-02	4.99E-02	6.94E-02	4.47E-02	6.24E-02	NA	NA	NA	LOW	NA	NA	43	43	0	43	25	18	41.86
Non-user	IOB_24	chr13	29933533	G	A	MTUS2	MISSENSE	ENSG00000132938:[ENST00000431530(c.3070G>A,Gtt>Att):ENSP00000392057(p.Val1024Ile)]	NA	NA	4.59E-03	2.03E-03	1.43E-02	4.59E-03	2.25E-02	T	BN	NA	MODERATE	N	0	98	98	0	79	41	38	48.1
Non-user	IOB_24	chr13	30054280	C	T	MTUS2	INTRONIC-SS-ACR-PRX	ENSG00000132938:[ENST00000380808(c.122-7C>T)]	NA	NA	3.00E-03	1.09E-02	1.20E-02	3.00E-03	4.10E-03	NA	NA	NA	MODIFIER	NA	NA	72	72	0	77	37	40	51.95
Non-user	IOB_24	chr13	30054280	C	T	MTUS2-AS1	NCT	100874107:[NR_046378.1(n.690-3204G>A)]	NA	NA	3.00E-03	1.09E-02	1.20E-02	3.00E-03	4.10E-03	NA	NA	NA	MODIFIER	NA	NA	72	72	0	77	37	40	51.95
Non-user	IOB_24	chr13	30072695	G	A	MTUS2	INTRONIC	ENSG00000132938:[ENST00000542829(c.465+21G>A)]	NA	NA	2.24E-02	3.80E-02	3.50E-02	2.24E-02	2.35E-02	NA	NA	NA	MODIFIER	NA	NA	105	105	0	79	43	36	45.57
Non-user	IOB_24	chr13	31732902	C	A	HSPH1	INTRONIC	ENSG00000120694:[ENST00000445273(c.171+41G>T)]	NA	NA	2.60E-03	1.54E-03	1.12E-02	2.60E-03	1.33E-02	NA	NA	NA	MODIFIER	NA	NA	112	112	0	70	47	23	32.86
Non-user	IOB_24	chr13	32723920	C	T	FRY	SILENT	ENSG00000073910:[ENST00000380250(c.1428C>T,ttC>ttT):ENSP00000369600(p.Phe476(=))]	NA	NA	NA	NA	NA	NA	NA	NA	NA	NA	LOW	NA	1	291	290	0	212	107	105	49.53
Non-user	IOB_24	chr13	33713029	G	C	STARD13	INTRONIC	ENSG00000133121:[ENST00000336934(c.387+3418C>G)]	NA	NA	1.58E-02	3.50E-02	8.21E-02	1.58E-02	6.44E-02	NA	NA	NA	MODIFIER	NA	NA	113	112	0	78	28	50	64.1
Non-user	IOB_24	chr13	34404864	C	T	RFC3	SILENT	ENSG00000133119:[ENST00000380071(c.582C>T,caC>caT):ENSP00000369411(p.His194(=))]	NA	NA	NA	1.42E-04	1.03E-03	NA	NA	NA	NA	NA	LOW	NA	0.469	47	46	1	47	23	24	51.06
Non-user	IOB_24	chr13	37016799	A	G	CCNA1	SILENT	ENSG00000133101:[ENST00000255465(c.1395A>G,caA>caG):ENSP00000255465(p.Gln465(=))]	NA	NA	2.00E-04	2.49E-04	1.60E-03	2.00E-04	1.00E-03	NA	NA	NA	LOW	NA	0	180	179	0	122	68	53	43.44
Non-user	IOB_24	chr13	39263422	C	T	FREM2	SILENT	ENSG00000150893:[ENST00000280481(c.1941C>T,gaC>gaT):ENSP00000280481(p.Asp647(=))]	NA	NA	1.22E-02	2.63E-02	1.65E-02	1.22E-02	7.20E-03	NA	NA	NA	LOW	NA	1	130	129	0	118	59	59	50
Non-user	IOB_24	chr13	39265558	T	A	FREM2	SILENT	ENSG00000150893:[ENST00000280481(c.4077T>A,ggT>ggA):ENSP00000280481(p.Gly1359(=))]	NA	NA	1.18E-02	2.56E-02	1.18E-02	1.18E-02	5.10E-03	NA	NA	NA	LOW	NA	NA	263	263	0	202	109	93	46.04
Non-user	IOB_24	chr13	40238257	T	C	COG6	INTRONIC	ENSG00000133103:[ENST00000543804(c.*131-976T>C)]	NA	NA	1.12E-02	5.85E-03	1.14E-02	1.12E-02	1.43E-02	NA	NA	NA	MODIFIER	NA	NA	71	71	0	63	33	30	47.62
Non-user	IOB_24	chr13	40238257	T	C	MIR4305	MATURE-MIRNA	ENSG00000264171:[ENST00000583252(n.16A>G)]	NA	NA	1.12E-02	5.85E-03	1.14E-02	1.12E-02	1.43E-02	NA	NA	NA	MODIFIER	NA	NA	71	71	0	63	33	30	47.62
Non-user	IOB_24	chr13	42265469	T	C	VWA8	MISSENSE	ENSG00000102763:[ENST00000379310(c.3853A>G,Agc>Ggc):ENSP00000368612(p.Ser1285Gly)]	NA	NA	9.98E-04	6.72E-04	4.80E-03	9.98E-04	5.10E-03	NA	NA	NA	MODIFIER	NA	NA	196	196	0	172	83	89	51.74
Non-user	IOB_24	chr13	44971503	A	C	SERP2	EXONIC-NC	387923:[NR_144535.1(n.659A>C)]	NA	NA	1.58E-02	NA	NA	1.58E-02	1.02E-02	NA	NA	NA	MODIFIER	NA	NA	70	70	0	60	32	27	45
Non-user	IOB_24	chr13	46539550	T	C	ZC3H13	MISSENSE	ENSG00000123200:[ENST00000378921(c.1204A>G,Aag>Gag):ENSP00000368201(p.Lys402Glu)]	NA	NA	NA	NA	NA	NA	NA	D_lc	PrD	NA	MODERATE	D	1	166	165	0	140	74	66	47.14
Non-user	IOB_24	chr13	46705020	T	C	LCP1	SILENT	ENSG00000136167:[ENST00000435666(c.387A>G,caA>caG):ENSP00000405134(p.Gln129(=))]	NA	NA	NA	8.24E-06	6.06E-05	NA	NA	NA	NA	NA	LOW	NA	0.917	160	159	0	117	71	45	38.46
Non-user	IOB_24	chr13	49096022	T	G	RCBTB2	INTRONIC	ENSG00000136161:[ENST00000544492(c.-418-6145A>C)]	NA	NA	.	7.43E-05	5.46E-04	NA	NA	NA	NA	NA	MODIFIER	NA	NA	184	184	0	86	41	43	50
Non-user	IOB_24	chr13	51519581	G	A	RNASEH2B	MISSENSE	ENSG00000136104:[ENST00000336617(c.529G>A,Gca>Aca):ENSP00000337623(p.Ala177Thr)]	NA	NA	2.00E-04	1.31E-03	1.27E-03	2.00E-04	1.00E-03	T	PoD	NA	MODERATE	D	0.992	320	320	0	250	125	123	49.2
Non-user	IOB_24	chr13	52330572	C	T	WDFY2	SILENT	ENSG00000139668:[ENST00000298125(c.1038C>T,agC>agT):ENSP00000298125(p.Ser346(=))]	NA	NA	NA	NA	NA	NA	NA	NA	NA	NA	LOW	NA	1	109	109	0	97	39	58	59.79
Non-user	IOB_24	chr13	52439603	C	T	CCDC70	MISSENSE	ENSG00000123171:[ENST00000242819(c.89C>T,gCg>gTg):ENSP00000242819(p.Ala30Val)]	NA	NA	chr13*52439603*52439603*C*A,T*rs146304026*NA*NA*NA	2.32E-04	9.73E-04	NA	NA	T	BN	NA	MODERATE	N	0	151	150	0	118	53	64	54.24
Non-user	IOB_24	chr13	54615413	G	A	NA	INTERGENIC	NA	NA	NA	2.40E-03	6.75E-03	1.30E-02	2.40E-03	9.20E-03	NA	NA	NA	MODIFIER	NA	NA	180	180	0	118	57	61	51.69
Non-user	IOB_24	chr13	60240829	C	T	DIAPH3	SILENT	ENSG00000139734:[ENST00000400324(c.3471G>A,gcG>gcA):ENSP00000383178(p.Ala1157(=))]	NA	NA	4.17E-02	4.80E-02	3.93E-02	4.17E-02	4.70E-02	NA	NA	NA	LOW	NA	NA	234	231	0	169	84	84	49.7

Patel *et al.* , 2021. Whole-exome sequencing analysis of oral squamous cell carcinoma delineated by tobacco usage habits
Supplementary Table 4. List of somatic single nucleotide variants identified using whole exome sequencing of OSCC patients

Cohort	Sample	Chromosome	Genomic position	Reference allele	Alternate allele	Genesymbol	Variant type	Variant description	OncoMD	COSMIC	dbSNP	ExAC	ExAC_SAS	1000G	1000G_SAS	SIFT	Polyphen2	CONDEL	VEP	LRT	PhastCon7Way	Total reads in Normal	Reference read support in Normal	Alternative read support in Normal	Total reads in Tumor	Reference read support in Tumor	Alternate read support in Tumor	Alternate allele frequency in Tumor
Non-user	IOB_24	chr13	76383408	A	T	LMO7	INTRONIC	4008:[XM_005266382.1(c.2387+89A>T)]	NA	NA	1.68E-02	NA	NA	1.68E-02	7.06E-02	NA	NA	NA	MODIFIER	NA	NA	176	175	1	165	70	94	56.97
Non-user	IOB_24	chr13	77619471	T	C	MYCBP2	3UTR	ENSG00000005810:[ENST00000357337(c.*42A>G)]	NA	NA	6.59E-03	6.39E-03	3.84E-02	6.59E-03	3.07E-02	NA	NA	NA	MODIFIER	NA	NA	52	52	0	55	25	30	54.55
Non-user	IOB_24	chr13	93879810	G	A	GPC6	MISSENSE	ENSG00000183098:[ENST00000377047(c.101G>A,cGc>cAc):ENSP00000366246(p.Arg34His)]	NA	NA	NA	8.37E-06	6.08E-05	NA	NA	D	PoD	NA	MODERATE	N	1	136	134	1	124	119	5	4.03
Non-user	IOB_24	chr13	95735520	C	A	ABCC4	MISSENSE	ENSG00000125257:[ENST00000412704(c.2419G>T,Gtc>Ttc):ENSP00000388657(p.Val807Phe)]	NA	NA	7.59E-03	7.89E-03	2.94E-02	7.59E-03	2.76E-02	NA	NA	NA	MODERATE	NA	0.999	123	123	0	70	32	38	54.29
Non-user	IOB_24	chr13	95840628	C	T	ABCC4	INTRONIC	ENSG00000125257:[ENST00000538287(c.*1544+79G>A)]	NA	NA	3.99E-04	NA	NA	3.99E-04	2.00E-03	NA	NA	NA	MODIFIER	NA	NA	44	44	0	40	25	13	32.5
Non-user	IOB_24	chr13	99908040	A	G	GPR18	SILENT	ENSG00000125245:[ENST00000340807(c.87T>C,taT>taC):ENSP00000343428(p.Tyr29(=))]	NA	NA	1.22E-02	5.74E-03	4.28E-02	1.22E-02	6.24E-02	NA	NA	NA	MODIFIER	NA	NA	202	201	0	184	93	91	49.46
Non-user	IOB_24	chr13	99908040	A	G	UBAC2	NCT	ENSG00000134882:[ENST00000494576(n.110+11162A>G)]	NA	NA	1.22E-02	5.74E-03	4.28E-02	1.22E-02	6.24E-02	NA	NA	NA	MODIFIER	NA	NA	202	201	0	184	93	91	49.46
Non-user	IOB_24	chr13	100008374	T	G	UBAC2	INTRONIC	ENSG00000134882:[ENST00000376440(c.703-11667T>G)]	NA	NA	1.22E-02	5.78E-03	4.17E-02	1.22E-02	6.24E-02	NA	NA	NA	MODIFIER	NA	NA	118	118	0	101	54	47	46.53
Non-user	IOB_24	chr13	100623870	A	G	ZIC5	SILENT	ENSG00000139800:[ENST00000267294(c.60T>C,gaT>gaC):ENSP00000267294(p.Asp20(=))]	NA	NA	2.00E-04	3.26E-03	3.13E-03	2.00E-04	1.00E-03	NA	NA	NA	LOW	NA	NA	170	170	0	140	67	71	50.71
Non-user	IOB_24	chr13	101316442	G	C	TMTC4	INTRONIC	ENSG00000125247:[ENST00000376234(c.278+33C>G)]	NA	NA	chr13*101316442*101316442*G*A,C*rs753155227*NA*NA*NA	1.76E-05	2.23E-04	NA	NA	NA	NA	NA	MODIFIER	NA	NA	84	84	0	62	33	29	46.77
Non-user	IOB_24	chr13	101735365	T	G	NALCN	INTRONIC	ENSG00000102452:[ENST00000251127(c.3690+78A>C)]	NA	NA	1.20E-03	NA	NA	1.20E-03	6.10E-03	NA	NA	NA	MODIFIER	NA	NA	233	232	1	176	95	80	45.45
Non-user	IOB_24	chr13	103492092	G	A	BIVM	SILENT	ENSG00000134897:[ENST00000257336(c.1389G>A,ggG>ggA):ENSP00000257336(p.Gly463(=))]	NA	NA	5.39E-03	8.82E-03	2.63E-02	5.39E-03	1.53E-02	NA	NA	NA	LOW	NA	NA	173	173	0	135	63	70	51.85
Non-user	IOB_24	chr13	103492092	G	A	BIVM-ERCC5	SILENT	ENSG00000270181:[ENST00000602836(c.1303G>A,ggG>ggA):ENSP00000473384(p.Gly435(=))]	NA	NA	5.39E-03	8.82E-03	2.63E-02	5.39E-03	1.53E-02	NA	NA	NA	LOW	NA	NA	173	173	0	135	63	70	51.85
Non-user	IOB_24	chr13	109793177	G	A	MYO16	SILENT	ENSG00000041515:[ENST00000356711(c.4551G>A,aaG>aaA):ENSP00000349145(p.Lys1517(=))]	NA	NA	2.18E-02	3.09E-02	4.07E-02	2.18E-02	3.68E-02	NA	NA	NA	LOW	NA	0.793	91	88	1	74	39	34	45.95
Non-user	IOB_24	chr13	109793747	C	A	MYO16	INTRONIC	23026:[NM_001198950.1(c.5164+23C>A)]	NA	NA	2.00E-03	1.12E-02	3.77E-03	2.00E-03	0.00E+00	NA	NA	NA	MODIFIER	NA	NA	141	140	0	106	50	54	50.94
Non-user	IOB_24	chr13	110434418	T	C	IRS2	MISSENSE	ENSG00000185950:[ENST00000375856(c.3983A>G,cAc>cGc):ENSP00000365016(p.His1328Arg)]	NA	NA	9.98E-04	4.77E-04	1.67E-03	9.98E-04	4.10E-03	D_lc	BN	NA	MODERATE	U	1	144	144	0	113	62	51	45.13
Non-user	IOB_24	chr13	110435728	C	G	IRS2	SILENT	ENSG00000185950:[ENST00000375856(c.2673G>C,acG>acC):ENSP00000365016(p.Thr891(=))]	NA	NA	2.70E-02	6.56E-02	3.66E-02	2.70E-02	2.15E-02	NA	NA	NA	LOW	NA	NA	100	100	0	107	51	55	51.4
Non-user	IOB_24	chr13	111083164	A	G	COL4A2	INTRONIC	ENSG00000134871:[ENST00000360467(c.648+210A>G)]	NA	NA	1.40E-03	1.43E-03	9.53E-03	1.40E-03	7.20E-03	NA	NA	NA	MODIFIER	NA	NA	189	188	0	154	81	72	46.75
Non-user	IOB_24	chr13	111294820	C	T	CARS2	MISSENSE	ENSG00000134905:[ENST00000257347(c.1465G>A,Gag>Aag):ENSP00000257347(p.Glu489Lys)]	NA	NA	NA	3.65E-05	0.00E+00	NA	NA	NA	NA	NA	MODIFIER	NA	NA	338	336	0	520	170	346	66.54
Non-user	IOB_24	chr13	111335406	C	T	CARS2	MISSENSE	ENSG00000134905:[ENST00000257347(c.647G>A,gGa>gAa):ENSP00000257347(p.Gly216Glu)]	NA	NA	1.24E-02	1.86E-02	1.75E-02	1.24E-02	1.12E-02	NA	NA	NA	MODIFIER	NA	NA	45	44	0	29	17	12	41.38
Non-user	IOB_24	chr13	113623825	G	A	MCF2L	5UTR	23263:[XM_005268312.1(c.-6G>A)]	NA	NA	NA	NA	NA	NA	NA	NA	NA	NA	MODIFIER	NA	NA	175	172	0	169	162	5	2.96
Non-user	IOB_24	chr13	113899447	C	A	CUL4A	INTRONIC	8451:[NM_001278514.1(c.1169-19C>A)]	NA	NA	1.42E-02	2.61E-02	4.34E-02	1.42E-02	3.37E-02	NA	NA	NA	MODIFIER	NA	NA	273	270	0	195	109	86	44.1
Non-user	IOB_24	chr13	114112389	T	C	DCUN1D2	SILENT	ENSG00000150401:[ENST00000478244(c.735A>G,gaA>gaG):ENSP00000417706(p.Glu245(=))]	NA	chr13*114112389*114112389*COSM147723*T*C*1*stomach*1	3.71E-02	3.46E-02	2.49E-02	3.71E-02	2.35E-02	NA	NA	NA	LOW	NA	0.702	111	111	0	80	45	35	43.75
Non-user	IOB_24	chr13	114113580	C	T	DCUN1D2	INTRONIC	55208:[XM_005268321.1(c.655+47G>A)]	NA	NA	3.65E-02	3.36E-02	2.30E-02	3.65E-02	2.25E-02	NA	NA	NA	MODIFIER	NA	NA	129	129	0	117	55	61	52.14
Non-user	IOB_24	chr13	114144928	G	C	DCUN1D2	INTRONIC	55208:[XM_005268321.1(c.-183+54C>G)]	NA	NA	3.59E-02	2.41E-01	1.67E-01	3.59E-02	2.66E-02	NA	NA	NA	MODIFIER	NA	NA	66	66	0	55	25	30	54.55
Non-user	IOB_24	chr13	114453590	C	T	LINC00552	EXONIC-NC	100130386:[NR_028064.1(n.473G>A)]	NA	NA	1.48E-02	2.88E-02	2.58E-02	1.48E-02	2.04E-02	NA	NA	NA	MODIFIER	NA	NA	297	296	0	217	114	99	45.62
Non-user	IOB_24	chr13	114623821	G	A	LINC00452	MISSENSE	643365:[NM_001278674.1(c.659G>A,tGc>tAc):NP_001265603.1(p.Cys220Tyr)]	NA	NA	4.83E-02	5.87E-02	3.11E-02	4.83E-02	3.07E-02	NA	NA	NA	MODERATE	NA	NA	455	454	0	393	192	196	49.87
Non-user	IOB_24	chr14	20248676	T	C	OR4M1	SILENT	ENSG00000176299:[ENST00000315957(c.195T>C,aaT>aaC):ENSP00000319654(p.Asn65(=))]	NA	NA	2.20E-03	8.32E-04	6.06E-03	2.20E-03	1.12E-02	NA	NA	NA	LOW	NA	0	803	802	0	788	530	255	32.36
Non-user	IOB_24	chr14	20248676	T	C	OR4N2	INTRONIC	ENSG00000176294:[ENST00000557414(c.-208+18534T>C)]	NA	NA	2.20E-03	8.32E-04	6.06E-03	2.20E-03	1.12E-02	NA	NA	NA	LOW	NA	0	803	802	0	788	530	255	32.36
Non-user	IOB_24	chr14	20296278	G	A	OR4N2	MISSENSE	ENSG00000176294:[ENST00000315947(c.671G>A,cGc>cAc):ENSP00000319601(p.Arg224His)]	NA	NA	4.21E-02	3.01E-02	5.29E-02	4.21E-02	6.44E-02	T	BN	NA	MODERATE	N	0	403	403	0	422	291	126	29.86
Non-user	IOB_24	chr14	21502110	G	A	NDRG2	INTRONIC	ENSG00000165795:[ENST00000555026(c.-6-10630C>T)]	NA	NA	1.66E-02	1.81E-02	2.94E-02	1.66E-02	2.56E-02	NA	NA	NA	MODIFIER	NA	NA	178	176	0	125	59	66	52.8
Non-user	IOB_24	chr14	21502110	G	A	RNASE13	MISSENSE	ENSG00000206150:[ENST00000382951(c.338C>T,tCc>tTc):ENSP00000372410(p.Ser113Phe)]	NA	NA	1.66E-02	1.81E-02	2.94E-02	1.66E-02	2.56E-02	NA	NA	NA	MODIFIER	NA	NA	178	176	0	125	59	66	52.8
Non-user	IOB_24	chr14	21502110	G	A	TPPP2	INTRONIC	ENSG00000179636:[ENST00000495665(c.303-1843G>A)]	NA	NA	1.66E-02	1.81E-02	2.94E-02	1.66E-02	2.56E-02	NA	NA	NA	MODIFIER	NA	NA	178	176	0	125	59	66	52.8
Non-user	IOB_24	chr14	21555068	A	G	ARHGEF40	INTRONIC	55701:[XM_005267845.1(c.2549-92A>G)]	NA	NA	NA	NA	NA	NA	NA	NA	NA	NA	MODIFIER	NA	NA	48	44	0	40	20	18	45
Non-user	IOB_24	chr14	21789402	G	A	RPGRIP1	INTRONIC	ENSG00000092200:[ENST00000382933(c.394-16G>A)]	NA	NA	2.00E-04	4.42E-05	3.24E-04	2.00E-04	1.00E-03	NA	NA	NA	MODIFIER	NA	NA	103	103	0	104	61	42	40.38
Non-user	IOB_24	chr14	21831419	C	T	SUPT16H	SILENT	ENSG00000092201:[ENST00000216297(c.1368G>A,cgG>cgA):ENSP00000216297(p.Arg456(=))]	NA	NA	4.53E-02	8.62E-02	9.61E-02	4.53E-02	7.06E-02	NA	NA	NA	LOW	NA	NA	150	150	0	113	53	60	53.1
Non-user	IOB_24	chr14	21899776	G	A	CHD8	SILENT	ENSG00000100888:[ENST00000399982(c.27C>T,ttC>ttT):ENSP00000382863(p.Phe9(=))]	NA	NA	3.00E-03	6.86E-03	7.81E-03	3.00E-03	6.10E-03	NA	NA	NA	LOW	NA	NA	115	114	0	94	55	39	41.49
Non-user	IOB_24	chr14	21936855	T	G	RAB2B	SILENT	ENSG00000129472:[ENST00000397762(c.243A>C,ggA>ggC):ENSP00000380869(p.Gly81(=))]	NA	NA	3.40E-03	1.85E-03	1.55E-02	3.39E-03	1.74E-02	NA	NA	NA	LOW	NA	NA	63	62	1	42	28	14	33.33
Non-user	IOB_24	chr14	22636737	G	A	NA	NCT	101929755:[XR_245743.1(n.35-62619C>T)]	NA	NA	2.94E-02	4.53E-02	1.14E-02	2.94E-02	1.12E-02	T	BN	NA	MODERATE	NA	NA	113	108	1	86	46	39	45.35
Non-user	IOB_24	chr14	22636737	G	A	TRAV30	MISSENSE	ENSG00000259092:[ENST00000557168(c.190G>A,Gtc>Atc):ENSP00000451308(p.Val64Ile)]	NA	NA	2.94E-02	4.53E-02	1.14E-02	2.94E-02	1.12E-02	T	BN	NA	MODERATE	NA	NA	113	108	1	86	46	39	45.35
Non-user	IOB_24	chr14	22671125	G	A	NA	NCT	101929755:[XR_245743.1(n.34+60597C>T)]	NA	NA	4.39E-03	1.89E-03	1.31E-02	4.39E-03	2.25E-02	T	BN	NA	MODERATE	NA	NA	125	124	0	103	51	51	49.51
Non-user	IOB_24	chr14	22671125	G	A	TRAV26-2	MISSENSE	ENSG00000211812:[ENST00000390460(c.190G>A,Gtg>Atg):ENSP00000450970(p.Val64Met)]	NA	NA	4.39E-03	1.89E-03	1.31E-02	4.39E-03	2.25E-02	T	BN	NA	MODERATE	NA	NA	125	124	0	103	51	51	49.51
Non-user	IOB_24	chr14	23282525	G	A	SLC7A7	MISSENSE	ENSG00000155465:[ENST00000285850(c.83C>T,cCg>cTg):ENSP00000285850(p.Pro28Leu)]	NA	NA	2.00E-04	1.68E-05	6.07E-05	2.00E-04	1.00E-03	T	BN	NA	MODERATE	NA	NA	162	162	0	100	66	33	33
Non-user	IOB_24	chr14	23397356	C	G	PRMT5	MISSENSE	ENSG00000100462:[ENST00000557415(c.175G>C,Gat>Cat):ENSP00000450633(p.Asp59His)]	NA	NA	NA	NA	NA	NA	NA	NA	NA	NA	MODIFIER	NA	NA	279	278	1	200	98	100	50
Non-user	IOB_24	chr14	23442664	G	A	AJUBA	SILENT	ENSG00000129474:[ENST00000397388(c.354C>T,gcC>gcT):ENSP00000380543(p.Ala118(=))]	NA	NA	NA	NA	NA	NA	NA	NA	NA	NA	LOW	NA	NA	118	118	0	122	63	59	48.36
Non-user	IOB_24	chr14	23442664	G	A	RP11-298I13.5	INTRONIC	ENSG00000259132:[ENST00000555074(c.50+8754C>T)]	NA	NA	NA	NA	NA	NA	NA	NA	NA	NA	LOW	NA	NA	118	118	0	122	63	59	48.36
Non-user	IOB_24	chr14	23652038	G	A	SLC7A8	MISSENSE	ENSG00000092068:[ENST00000316902(c.86C>T,tCc>tTc):ENSP00000320378(p.Ser29Phe)]	NA	NA	8.19E-03	8.04E-03	4.00E-02	8.19E-03	3.89E-02	D	BN	NA	MODERATE	NA	NA	417	416	0	332	191	141	42.47
Non-user	IOB_24	chr14	23842570	C	T	IL25	SILENT	ENSG00000166090:[ENST00000329715(c.243C>T,ccC>ccT):ENSP00000328111(p.Pro81(=))]	NA	NA	2.00E-03	9.45E-04	5.61E-03	2.00E-03	8.20E-03	NA	NA	NA	LOW	NA	NA	64	64	0	43	22	21	48.84
Non-user	IOB_24	chr14	23866925	G	C	MYH6	INTRONIC	ENSG00000197616:[ENST00000356287(c.1892-103C>G)]	NA	NA	4.99E-02	NA	NA	4.99E-02	2.66E-02	NA	NA	NA	MODIFIER	NA	NA	62	62	0	51	33	17	33.33
Non-user	IOB_24	chr14	23871909	G	A	MYH6	SILENT	ENSG00000197616:[ENST00000356287(c.999C>T,acC>acT):ENSP00000348634(p.Thr333(=))]	NA	NA	4.23E-02	2.03E-02	2.62E-02	4.23E-02	2.66E-02	NA	NA	NA	LOW	NA	NA	113	113	0	113	47	66	58.41
Non-user	IOB_24	chr14	23896116	G	A	MYH7	NCT	4625:[XR_245686.1(n.2035-43C>T)]	NA	NA	NA	2.28E-04	1.65E-03	NA	NA	NA	NA	NA	MODIFIER	NA	NA	77	77	0	43	24	18	41.86
Non-user	IOB_24	chr14	23944744	C	A	NGDN	EXONIC-NC	ENSG00000129460:[ENST00000556022(n.353C>A)]	NA	NA	1.80E-03	8.12E-04	5.36E-03	1.80E-03	9.20E-03	NA	NA	NA	MODIFIER	NA	NA	46	46	0	43	26	16	37.21
Non-user	IOB_24	chr14	24045424	C	A	JPH4	SILENT	ENSG00000092051:[ENST00000356300(c.621G>T,tcG>tcT):ENSP00000348648(p.Ser207(=))]	NA	NA	9.98E-04	3.70E-02	5.00E-02	9.98E-04	3.10E-03	NA	NA	NA	LOW	NA	NA	45	45	0	42	30	11	26.19
Non-user	IOB_24	chr14	24533381	G	A	LRRC16B	INTRONIC	ENSG00000186648:[ENST00000342740(c.2980-74G>A)]	NA	NA	3.99E-04	NA	NA	3.99E-04	2.00E-03	NA	NA	NA	MODIFIER	NA	NA	57	56	0	53	27	26	49.06
Non-user	IOB_24	chr14	24546566	T	C	CPNE6	SILENT	ENSG00000100884:[ENST00000537691(c.1668T>C,atT>atC):ENSP00000440077(p.Ile556(=))]	NA	NA	1.72E-02	2.08E-02	5.57E-02	1.72E-02	5.73E-02	NA	NA	NA	LOW	NA	0.08	579	577	0	451	218	230	51
Non-user	IOB_24	chr14	24780751	A	G	LTB4R	5UTR	ENSG00000213903:[ENST00000345363(c.-275A>G)]	NA	NA	2.20E-03	8.89E-04	3.34E-03	2.20E-03	1.02E-02	T	BN	NA	MODERATE	U	0	294	292	0	226	130	96	42.48
Non-user	IOB_24	chr14	24780751	A	G	LTB4R2	MISSENSE	ENSG00000272658:[ENST00000528054(c.974A>G,gAt>gGt):ENSP00000432146(p.Asp325Gly)]	NA	NA	2.20E-03	8.89E-04	3.34E-03	2.20E-03	1.02E-02	T	BN	NA	MODERATE	U	0	294	292	0	226	130	96	42.48
Non-user	IOB_24	chr14	24910076	A	T	KHNYN	3UTR	ENSG00000100441:[ENST00000251343(c.*3585A>T)]	NA	NA	4.79E-03	1.15E-02	3.69E-03	4.79E-03	5.10E-03	NA	NA	NA	MODIFIER	NA	NA	96	96	0	89	34	54	60.67
Non-user	IOB_24	chr14	24910076	A	T	SDR39U1	MISSENSE	ENSG00000100445:[ENST00000553343(c.67T>A,Ttt>Att):ENSP00000452076(p.Phe23Ile)]	NA	NA	4.79E-03	1.15E-02	3.69E-03	4.79E-03	5.10E-03	NA	NA	NA	MODIFIER	NA	NA	96	96	0	89	34	54	60.67
Non-user	IOB_24	chr14	30046511	T	C	PRKD1	MISSENSE	ENSG00000184304:[ENST00000415220(c.2696A>G,cAc>cGc):ENSP00000390535(p.His899Arg)]	NA	NA	5.19E-03	1.12E-02	3.63E-03	5.19E-03	3.10E-03	T_lc	BN	NA	MODERATE	N	1	117	117	0	77	41	35	45.45
Non-user	IOB_24	chr14	36003618	C	T	INSM2	MISSENSE	ENSG00000168348:[ENST00000307169(c.160C>T,Ccc>Tcc):ENSP00000306523(p.Pro54Ser)]	NA	NA	9.78E-03	2.58E-02	2.10E-02	9.78E-03	1.12E-02	T	BN	NA	MODERATE	NA	NA	104	104	0	78	44	33	42.31
Non-user	IOB_24	chr14	36946282	C	T	RP11-896J10.3	EXONIC-NC	ENSG00000257520:[ENST00000521945(n.335G>A)]	NA	NA	1.84E-02	3.06E-02	5.98E-02	1.84E-02	4.50E-02	NA	NA	NA	MODIFIER	NA	NA	158	157	0	97	57	39	40.21
Non-user	IOB_24	chr14	36946282	C	T	SFTA3	MISSENSE	ENSG00000229415:[ENST00000518002(c.155G>A,cGg>cAg):ENSP00000428247(p.Arg52Gln)]	NA	NA	1.84E-02	3.06E-02	5.98E-02	1.84E-02	4.50E-02	NA	NA	NA	MODIFIER	NA	NA	158	157	0	97	57	39	40.21
Non-user	IOB_24	chr14	38678753	G	A	SSTR1	SILENT	ENSG00000139874:[ENST00000267377(c.159G>A,gaG>gaA):ENSP00000267377(p.Glu53(=))]	NA	NA	1.20E-03	4.21E-04	4.06E-03	1.20E-03	6.10E-03	NA	NA	NA	LOW	NA	1	214	214	0	144	68	75	52.08
Non-user	IOB_24	chr14	39783929	T	C	CTAGE5	NCT	ENSG00000150527:[ENST00000556593(n.42-14T>C)]	NA	NA	3.99E-04	2.74E-04	1.36E-03	3.99E-04	2.00E-03	NA	NA	NA	MODIFIER	NA	NA	33	32	0	35	24	11	31.43
Non-user	IOB_24	chr14	39783929	T	C	MIA2	INTRONIC	4253:[XM_005267646.1(c.1293-14T>C)]	NA	NA	3.99E-04	2.74E-04	1.36E-03	3.99E-04	2.00E-03	NA	NA	NA	MODIFIER	NA	NA	33	32	0	35	24	11	31.43
Non-user	IOB_24	chr14	39783929	T	C	RP11-407N17.3	INTRONIC	ENSG00000258941:[ENST00000603904(c.1209-14T>C)]	NA	NA	3.99E-04	2.74E-04	1.36E-03	3.99E-04	2.00E-03	NA	NA	NA	MODIFIER	NA	NA	33	32	0	35	24	11	31.43
Non-user	IOB_24	chr14	39901377	G	A	FBXO33	5UTR	ENSG00000165355:[ENST00000298097(c.-11C>T)]	NA	NA	2.00E-03	1.89E-02	1.41E-02	2.00E-03	3.10E-03	NA	NA	NA	MODIFIER	NA	NA	320	317	0	217	123	93	42.86
Non-user	IOB_24	chr14	45465079	C	G	FAM179B	MISSENSE	ENSG00000198718:[ENST00000555874(c.134C>G,cCa>cGa):ENSP00000451141(p.Pro45Arg)]	NA	NA	7.99E-04	1.48E-03	1.10E-02	7.99E-04	4.10E-03	T	BN	NA	MODERATE	NA	NA	90	90	0	80	36	44	55
Non-user	IOB_24	chr14	45465079	C	G	KLHL28	INTRONIC	ENSG00000179454:[ENST00000556239(c.-101-17578G>C)]	NA	NA	7.99E-04	1.48E-03	1.10E-02	7.99E-04	4.10E-03	T	BN	NA	MODERATE	NA	NA	90	90	0	80	36	44	55
Non-user	IOB_24	chr14	45654398	T	G	FANCM	INTRONIC	ENSG00000187790:[ENST00000267430(c.4516-22T>G)]	NA	NA	5.99E-04	9.15E-04	5.61E-03	5.99E-04	3.10E-03	NA	NA	NA	MODIFIER	NA	NA	35	35	0	31	18	13	41.94
Non-user	IOB_24	chr14	45673260	A	C	MIS18BP1	EXONIC-NC	ENSG00000129534:[ENST00000470424(n.573T>G)]	NA	NA	1.56E-02	NA	NA	1.56E-02	2.66E-02	NA	NA	NA	MODIFIER	NA	NA	36	36	0	44	23	21	47.73
Non-user	IOB_24	chr14	47566145	G	A	MDGA2	SILENT	ENSG00000139915:[ENST00000554762(c.223C>T,atC>atT):ENSP00000450827(p.Ile75(=))]	NA	NA	3.99E-04	2.74E-03	1.76E-03	3.99E-04	0.00E+00	NA	NA	NA	LOW	NA	1	236	236	0	239	114	125	52.3
Non-user	IOB_24	chr14	50092386	G	A	DNAAF2	SILENT	ENSG00000165506:[ENST00000298292(c.2388C>T,caC>caT):ENSP00000298292(p.His796(=))]	NA	NA	2.00E-03	8.25E-04	6.00E-03	2.00E-03	1.02E-02	NA	NA	NA	LOW	NA	0.078	139	139	0	121	65	55	45.45
Non-user	IOB_24	chr14	50585012	A	T	SOS2	3UTR	ENSG00000100485:[ENST00000216373(c.*50T>A)]	NA	NA	3.59E-03	4.10E-03	1.42E-02	3.59E-03	9.20E-03	NA	NA	NA	MODIFIER	NA	NA	48	48	0	31	12	19	61.29
Non-user	IOB_24	chr14	50910675	C	G	MAP4K5	MISSENSE	ENSG00000012983:[ENST00000013125(c.1419G>C,aaG>aaC):ENSP00000013125(p.Lys473Asn)]	NA	NA	5.39E-03	1.40E-02	1.92E-02	5.39E-03	8.20E-03	D	PrD	NA	MODERATE	D	1	130	130	0	143	59	83	58.04
Non-user	IOB_24	chr14	51225097	T	C	NIN	MISSENSE	ENSG00000100503:[ENST00000389869(c.1122A>G,aAa>aGa):ENSP00000374519(p.Lys375Arg)]	NA	NA	8.99E-03	1.61E-02	1.79E-02	8.99E-03	9.20E-03	NA	NA	NA	MODIFIER	NA	NA	155	155	0	85	40	44	51.76
Non-user	IOB_24	chr14	51324794	T	C	PYGL	3UTR	ENSG00000100504:[ENST00000532462(c.*13A>G)]	NA	NA	3.73E-02	2.23E-02	2.69E-02	3.73E-02	3.07E-02	NA	NA	NA	MODIFIER	NA	NA	283	282	0	217	126	91	41.94
Non-user	IOB_24	chr14	51324794	T	C	RP11-218E20.2	NCT	ENSG00000258687:[ENST00000557152(n.29-7438T>C)]	NA	NA	3.73E-02	2.23E-02	2.69E-02	3.73E-02	3.07E-02	NA	NA	NA	MODIFIER	NA	NA	283	282	0	217	126	91	41.94
Non-user	IOB_24	chr14	51378611	G	A	PYGL	INTRONIC	ENSG00000100504:[ENST00000544180(c.1726-22C>T)]	NA	NA	2.00E-04	8.24E-05	3.63E-04	2.00E-04	0.00E+00	NA	NA	NA	MODIFIER	NA	NA	239	238	0	192	97	95	49.48
Non-user	IOB_24	chr14	52906044	A	G	TXNDC16	MISSENSE	ENSG00000087301:[ENST00000281741(c.2072T>C,cTt>cCt):ENSP00000281741(p.Leu691Pro)]	NA	NA	NA	NA	NA	NA	NA	NA	NA	NA	MODERATE	NA	1	138	138	0	102	50	52	50.98
Non-user	IOB_24	chr14	54955665	G	T	GMFB	EXONIC-NC	ENSG00000197045:[ENST00000554682(n.34C>A)]	NA	NA	3.99E-04	3.35E-04	1.48E-03	3.99E-04	2.00E-03	NA	NA	NA	MODIFIER	NA	NA	208	207	0	106	48	57	53.77
Non-user	IOB_24	chr14	55510110	G	A	SOCS4	SILENT	ENSG00000180008:[ENST00000339298(c.351G>A,ccG>ccA):ENSP00000341327(p.Pro117(=))]	NA	NA	3.28E-02	7.17E-02	5.02E-02	3.27E-02	3.68E-02	NA	NA	NA	LOW	NA	NA	279	278	0	176	97	78	44.32
Non-user	IOB_24	chr14	55604768	T	C	LGALS3	SILENT	ENSG00000131981:[ENST00000254301(c.24T>C,caT>caC):ENSP00000254301(p.His8(=))]	NA	NA	9.98E-04	1.50E-03	9.98E-04	9.98E-04	2.00E-03	NA	NA	NA	MODIFIER	NA	NA	152	151	0	97	58	38	39.18

Patel *et al.* , 2021. Whole-exome sequencing analysis of oral squamous cell carcinoma delineated by tobacco usage habits
Supplementary Table 4. List of somatic single nucleotide variants identified using whole exome sequencing of OSCC patients

Cohort	Sample	Chromosome	Genomic position	Reference allele	Alternate allele	Genesymbol	Variant type	Variant description	OncoMD	COSMIC	dbSNP	ExAC	ExAC_SAS	1000G	1000G_SAS	SIFT	Polyphen2	CONDEL	VEP	LRT	PhastCon7Way	Total reads in Normal	Reference read support in Normal	Alternative read support in Normal	Total reads in Tumor	Reference read support in Tumor	Alternate read support in Tumor	Alternate allele frequency in Tumor
Non-user	IOB_24	chr14	60616886	C	T	DHRS7	MISSENSE	ENSG00000100612:[ENST00000557751(c.256G>A,cGa>cAa):ENSP00000452026(p.Arg86Gln)]	NA	NA	9.78E-03	1.16E-02	3.62E-02	9.78E-03	4.09E-02	T	BN	NA	MODERATE	NA	NA	199	178	0	135	69	53	39.26
Non-user	IOB_24	chr14	60616886	C	T	PCNXL4	INTRONIC	ENSG00000126773:[ENST00000406949(c.2566-18524C>T)]	NA	NA	9.78E-03	1.16E-02	3.62E-02	9.78E-03	4.09E-02	T	BN	NA	MODERATE	NA	NA	199	178	0	135	69	53	39.26
Non-user	IOB_24	chr14	64749369	G	A	ESR2	MISSENSE	ENSG00000140009:[ENST00000267525(c.335C>T,tCg>tTg):ENSP00000267525(p.Ser112Leu)]	NA	chr14*64749369*64749369*COSM3401403*G*A*1*central_nervous_system*1|chr14*64749369*64749369*COSM3401404*G*A*1*central_nervous_system*1|chr14*64749369*64749369*COSM3401405*G*A*1*central_nervous_system*1|chr14*64749369*64749369*COSM3401402*G*A*1*central_nervous_system*1	1.60E-03	7.94E-04	6.18E-03	1.60E-03	8.20E-03	NA	NA	NA	MODIFIER	NA	NA	119	119	0	127	75	52	40.94
Non-user	IOB_24	chr14	64892639	C	A	CTD-2555O16.2	NCT	ENSG00000258824:[ENST00000556640(n.506-2942G>T)]	NA	NA	1.52E-02	NA	NA	1.52E-02	2.15E-02	NA	NA	NA	MODIFIER	NA	NA	75	74	1	55	27	28	50.91
Non-user	IOB_24	chr14	64892639	C	A	MTHFD1	INTRONIC	ENSG00000100714:[ENST00000545908(c.1121+94C>A)]	NA	NA	1.52E-02	NA	NA	1.52E-02	2.15E-02	NA	NA	NA	MODIFIER	NA	NA	75	74	1	55	27	28	50.91
Non-user	IOB_24	chr14	65208952	G	A	PLEKHG3	MISSENSE	ENSG00000126822:[ENST00000471182(c.1316G>A,cGc>cAc):ENSP00000450945(p.Arg439His)]	NA	chr14*65208952*65208952*COSM1194380*G*T*1*lung*1|chr14*65208952*65208952*COSM1370592*G*A*1*large_intestine*1	7.99E-04	2.35E-04	1.70E-03	7.99E-04	4.10E-03	T	BN	NA	MODERATE	N	0.002	157	157	0	113	51	61	53.98
Non-user	IOB_24	chr14	65209825	G	A	PLEKHG3	MISSENSE	ENSG00000126822:[ENST00000471182(c.1663G>A,Gtc>Atc):ENSP00000450945(p.Val555Ile)]	NA	NA	NA	3.33E-05	2.43E-04	NA	NA	T	BN	NA	MODERATE	N	0	476	475	0	358	204	152	42.46
Non-user	IOB_24	chr14	68050780	G	A	PIGH	INTRONIC	5283:[XM_005267764.1(c.475-1789C>T)]	NA	NA	NA	NA	NA	NA	NA	NA	NA	NA	MODIFIER	NA	NA	88	88	0	68	33	33	48.53
Non-user	IOB_24	chr14	68050780	G	A	PLEKHH1	MISSENSE	ENSG00000054690:[ENST00000417684(c.233G>A,cGc>cAc):ENSP00000397712(p.Arg78His)]	NA	NA	NA	NA	NA	NA	NA	NA	NA	NA	MODIFIER	NA	NA	88	88	0	68	33	33	48.53
Non-user	IOB_24	chr14	69356932	G	T	ACTN1	SILENT	ENSG00000072110:[ENST00000376839(c.963C>A,atC>atA):ENSP00000366035(p.Ile321(=))]	NA	NA	1.22E-02	1.96E-02	2.19E-02	1.22E-02	2.35E-02	NA	NA	NA	LOW	NA	1	221	221	0	183	85	97	53.01
Non-user	IOB_24	chr14	69376165	C	T	ACTN1	NCT	ENSG00000072110:[ENST00000554508(n.209-52G>A)]	NA	NA	2.56E-02	2.71E-02	6.71E-02	2.56E-02	6.13E-02	NA	NA	NA	MODIFIER	NA	NA	98	97	0	88	53	34	38.64
Non-user	IOB_24	chr14	69806313	G	A	GALNT16	SILENT	ENSG00000100626:[ENST00000337827(c.1164G>A,tcG>tcA):ENSP00000336729(p.Ser388(=))]	NA	NA	5.99E-04	4.00E-04	2.25E-03	5.99E-04	1.00E-03	NA	NA	NA	MODIFIER	NA	NA	178	177	0	134	67	67	50
Non-user	IOB_24	chr14	69988928	T	C	PLEKHD1	INTRONIC	ENSG00000175985:[ENST00000322564(c.556-72T>C)]	NA	NA	9.98E-04	NA	NA	9.98E-04	5.10E-03	NA	NA	NA	MODIFIER	NA	NA	129	129	0	120	68	52	43.33
Non-user	IOB_24	chr14	70926338	C	T	ADAM21	NONSENSE	ENSG00000139985:[ENST00000267499(c.2122C>T,Cga>Tga):ENSP00000267499(p.Arg708Ter)]	NA	chr14*70926338*70926338*COSM3386657*C*T*1*pancreas*1|chr14*70926338*70926338*COSM3386658*C*T*1*pancreas*1	NA	1.24E-04	7.88E-04	NA	NA	NA	NA	NA	MODIFIER	NA	NA	161	160	0	128	70	57	44.53
Non-user	IOB_24	chr14	70926338	C	T	RP11-486O13.4	NCT	ENSG00000257759:[ENST00000556646(n.184-6112G>A)]	NA	chr14*70926338*70926338*COSM3386657*C*T*1*pancreas*1|chr14*70926338*70926338*COSM3386658*C*T*1*pancreas*1	NA	1.24E-04	7.88E-04	NA	NA	NA	NA	NA	MODIFIER	NA	NA	161	160	0	128	70	57	44.53
Non-user	IOB_24	chr14	73198629	G	C	DPF3	SILENT	ENSG00000205683:[ENST00000366353(c.480C>G,ccC>ccG):ENSP00000381791(p.Pro160(=))]	NA	NA	2.60E-03	6.53E-03	9.59E-04	2.60E-03	0.00E+00	NA	NA	NA	LOW	NA	NA	45	45	0	49	23	24	48.98
Non-user	IOB_24	chr14	73718514	G	A	PAPLN	SILENT	ENSG00000100767:[ENST00000216658(c.813G>A,cgG>cgA):ENSP00000216658(p.Arg271(=))]	NA	NA	1.14E-02	1.72E-02	3.64E-02	1.14E-02	2.76E-02	NA	NA	NA	LOW	NA	NA	273	273	0	203	91	112	55.17
Non-user	IOB_24	chr14	73730661	G	A	PAPLN	MISSENSE	ENSG00000100767:[ENST00000216658(c.2395G>A,Ggc>Agc):ENSP00000216658(p.Gly799Ser)]	NA	NA	NA	1.01E-03	1.25E-03	NA	NA	NA	NA	NA	MODIFIER	NA	NA	77	75	0	38	26	11	28.95
Non-user	IOB_24	chr14	73989145	G	A	HEATR4	MISSENSE	ENSG00000187105:[ENST00000560393(c.571C>T,Cgc>Tgc):ENSP00000453597(p.Arg191Cys)]	NA	NA	3.04E-02	3.85E-02	2.96E-02	3.04E-02	1.64E-02	D	PoD	NA	MODERATE	N	0.005	286	283	2	224	112	111	49.55
Non-user	IOB_24	chr14	73989816	C	T	HEATR4	MISSENSE	ENSG00000187105:[ENST00000334988(c.41G>A,tGc>tAc):ENSP00000335447(p.Cys14Tyr)]	NA	NA	3.36E-02	4.14E-02	3.11E-02	3.35E-02	1.53E-02	NA	NA	NA	MODIFIER	NA	NA	115	115	0	77	35	42	54.55
Non-user	IOB_24	chr14	73989816	C	T	RP3-414A15.2	NCT	ENSG00000258695:[ENST00000555972(n.60+175C>T)]	NA	NA	3.36E-02	4.14E-02	3.11E-02	3.35E-02	1.53E-02	NA	NA	NA	MODIFIER	NA	NA	115	115	0	77	35	42	54.55
Non-user	IOB_24	chr14	74041748	A	G	ACOT2	MISSENSE	ENSG00000119673:[ENST00000538782(c.392A>G,cAc>cGc):ENSP00000440961(p.His131Arg)]	NA	NA	NA	6.95E-01	6.86E-01	NA	NA	NA	NA	NA	MODIFIER	NA	NA	159	139	18	35	0	35	100
Non-user	IOB_24	chr14	74402693	C	T	FAM161B	SILENT	ENSG00000156050:[ENST00000534936(c.1764G>A,cgG>cgA):ENSP00000445326(p.Arg588(=))]	NA	NA	4.37E-02	8.13E-02	5.32E-02	4.37E-02	5.11E-02	NA	NA	NA	LOW	NA	NA	72	71	0	46	18	28	60.87
Non-user	IOB_24	chr14	74402693	C	T	RP5-1021I20.5	EXONIC-NC	ENSG00000258891:[ENST00000555916(n.366G>A)]	NA	NA	4.37E-02	8.13E-02	5.32E-02	4.37E-02	5.11E-02	NA	NA	NA	LOW	NA	NA	72	71	0	46	18	28	60.87
Non-user	IOB_24	chr14	75580158	G	A	NEK9	INTRONIC	91754:[NM_001329238.1(c.520-52C>T)]	NA	NA	7.99E-04	4.07E-04	1.23E-04	7.99E-04	0.00E+00	NA	NA	NA	MODIFIER	NA	NA	56	55	0	45	19	25	55.56
Non-user	IOB_24	chr14	76368521	G	A	IFT43	EXONIC-NC	ENSG00000119650:[ENST00000555677(n.43G>A)]	NA	NA	3.59E-03	2.77E-03	2.02E-02	3.59E-03	1.84E-02	NA	NA	NA	MODIFIER	NA	NA	145	145	0	86	35	51	59.3
Non-user	IOB_24	chr14	76368521	G	A	TTLL5	SILENT	ENSG00000119685:[ENST00000298832(c.3777G>A,caG>caA):ENSP00000298832(p.Gln1259(=))]	NA	NA	3.59E-03	2.77E-03	2.02E-02	3.59E-03	1.84E-02	NA	NA	NA	MODIFIER	NA	NA	145	145	0	86	35	51	59.3
Non-user	IOB_24	chr14	76966347	C	T	ESRRB	MISSENSE	ENSG00000119715:[ENST00000380887(c.1438C>T,Cca>Tca):ENSP00000370270(p.Pro480Ser)]	NA	NA	5.99E-04	1.00E-03	6.88E-04	5.99E-04	1.00E-03	D_lc	BN	NA	MODERATE	NA	NA	53	53	0	55	19	35	63.64
Non-user	IOB_24	chr14	76966347	C	T	RP11-187O7.3	NCT	ENSG00000259124:[ENST00000554926(n.414+2153G>A)]	NA	NA	5.99E-04	1.00E-03	6.88E-04	5.99E-04	1.00E-03	D_lc	BN	NA	MODERATE	NA	NA	53	53	0	55	19	35	63.64
Non-user	IOB_24	chr14	77743842	T	C	POMT2	INTRONIC	ENSG00000009830:[ENST00000555710(c.*281-18A>G)]	NA	NA	2.60E-03	4.45E-03	7.33E-03	2.60E-03	7.20E-03	NA	NA	NA	MODIFIER	NA	NA	142	140	0	132	67	65	49.24
Non-user	IOB_24	chr14	79423716	T	C	NRXN3	INTRONIC	9369:[XM_005268218.1(c.2431+12T>C)]	NA	NA	NA	1.31E-03	4.20E-03	NA	NA	NA	NA	NA	MODIFIER	NA	NA	101	100	1	63	36	27	42.86
Non-user	IOB_24	chr14	81676282	T	C	GTF2A1	INTRONIC-SS-ACR	ENSG00000165417:[ENST00000298173(c.133-2A>G)]	NA	NA	4.55E-02	8.96E-02	6.87E-02	4.55E-02	6.75E-02	NA	NA	NA	HIGH	NA	NA	211	211	0	155	84	71	45.81
Non-user	IOB_24	chr14	90752754	G	A	NRDE2	MISSENSE	ENSG00000119720:[ENST00000357904(c.2548C>T,Cgc>Tgc):ENSP00000350579(p.Arg850Cys)]	NA	NA	1.24E-02	3.41E-02	1.24E-02	1.24E-02	1.02E-02	D	BN	NA	MODERATE	N	0.977	162	159	2	129	64	65	50.39
Non-user	IOB_24	chr14	90755497	G	A	NRDE2	INTRONIC-SS-ACR-PRX	ENSG00000119720:[ENST00000553409(c.*1755-8C>T)]	NA	NA	1.30E-02	3.53E-02	1.54E-02	1.30E-02	1.33E-02	NA	NA	NA	LOW	NA	NA	127	127	0	105	61	44	41.9
Non-user	IOB_24	chr14	91739046	C	G	CCDC88C	MISSENSE	ENSG00000015133:[ENST00000331194(c.1582G>C,Gtc>Ctc):ENSP00000330332(p.Val528Leu)]	NA	NA	chr14*91739046*91739046*C*A,G,T*rs370102360*NA*NA*NA	2.00E-04	1.18E-03	NA	NA	NA	NA	NA	MODIFIER	NA	NA	87	87	0	60	41	19	31.67
Non-user	IOB_24	chr14	92361279	C	G	FBLN5	INTRONIC	ENSG00000140092:[ENST00000342058(c.502+15G>C)]	NA	NA	4.59E-03	5.12E-03	1.15E-02	4.59E-03	1.43E-02	NA	NA	NA	MODIFIER	NA	NA	166	164	0	121	82	37	30.58
Non-user	IOB_24	chr14	94408637	T	C	ASB2	INTRONIC	ENSG00000100628:[ENST00000315988(c.909-2619A>G)]	NA	NA	NA	NA	NA	NA	NA	NA	NA	NA	MODIFIER	NA	NA	66	66	0	56	31	25	44.64
Non-user	IOB_24	chr14	94408637	T	C	NA	NCT	101929033:[XR_245810.1(n.201+32T>C)]	NA	NA	NA	NA	NA	NA	NA	NA	NA	NA	MODIFIER	NA	NA	66	66	0	56	31	25	44.64
Non-user	IOB_24	chr14	94408637	T	C	RP11-131H24.4	INTRONIC	ENSG00000258987:[ENST00000557646(c.125+32T>C)]	NA	NA	NA	NA	NA	NA	NA	NA	NA	NA	MODIFIER	NA	NA	66	66	0	56	31	25	44.64
Non-user	IOB_24	chr14	94423327	T	A	ASB2	EXONIC-NC	ENSG00000100628:[ENST00000556793(n.69A>T)]	NA	NA	3.81E-02	5.95E-02	5.63E-02	3.81E-02	6.13E-02	NA	NA	NA	MODIFIER	NA	NA	100	99	0	81	44	37	45.68
Non-user	IOB_24	chr14	95081081	A	G	RP11-986E7.7	3UTR	ENSG00000273259:[ENST00000553947(c.*1129A>G)]	NA	NA	4.47E-02	8.19E-02	8.07E-02	4.47E-02	6.03E-02	NA	NA	NA	MODIFIER	NA	NA	186	185	0	172	72	100	58.14
Non-user	IOB_24	chr14	95081081	A	G	SERPINA3	SILENT	ENSG00000196136:[ENST00000393078(c.303A>G,aaA>aaG):ENSP00000376793(p.Lys101(=))]	NA	NA	4.47E-02	8.19E-02	8.07E-02	4.47E-02	6.03E-02	NA	NA	NA	MODIFIER	NA	NA	186	185	0	172	72	100	58.14
Non-user	IOB_24	chr14	100192468	G	T	CYP46A1	INTRONIC	ENSG00000036530:[ENST00000554176(c.777-59G>T)]	NA	NA	8.99E-03	NA	NA	8.99E-03	6.10E-03	NA	NA	NA	MODIFIER	NA	NA	139	139	0	72	33	39	54.17
Non-user	IOB_24	chr14	102348621	G	A	PPP2R5C	INTRONIC	5527:[XM_005267827.1(c.180+13G>A)]	NA	NA	NA	NA	NA	NA	NA	NA	NA	NA	MODIFIER	NA	NA	198	197	0	188	96	91	48.4
Non-user	IOB_24	chr14	102505913	C	T	DYNC1H1	INTRONIC	ENSG00000197102:[ENST00000360184(c.11595+30C>T)]	NA	NA	NA	2.47E-05	0.00E+00	NA	NA	NA	NA	NA	MODIFIER	NA	NA	222	220	1	164	78	86	52.44
Non-user	IOB_24	chr14	102505913	C	T	RP11-1017G21.4	NCT	ENSG00000258959:[ENST00000557551(n.112-2807G>A)]	NA	NA	NA	2.47E-05	0.00E+00	NA	NA	NA	NA	NA	MODIFIER	NA	NA	222	220	1	164	78	86	52.44
Non-user	IOB_24	chr14	102548482	A	T	HSP90AA1	SILENT	ENSG00000080824:[ENST00000216281(c.2055T>A,gcT>gcA):ENSP00000216281(p.Ala685(=))]	NA	NA	NA	NA	NA	NA	NA	NA	NA	NA	LOW	NA	NA	158	157	1	121	60	61	50.41
Non-user	IOB_24	chr14	104518299	C	T	TDRD9	NCT	ENSG00000156414:[ENST00000481318(n.330-19C>T)]	NA	NA	1.40E-02	4.56E-02	5.36E-02	1.40E-02	3.78E-02	NA	NA	NA	MODIFIER	NA	NA	308	308	0	187	105	81	43.32
Non-user	IOB_24	chr14	104633227	A	C	KIF26A	SILENT	ENSG00000066735:[ENST00000423312(c.955A>C,Agg>Cgg):ENSP00000388241(p.Arg319(=))]	NA	NA	2.28E-02	9.74E-02	3.96E-02	2.28E-02	2.76E-02	NA	NA	NA	LOW	NA	NA	63	62	0	55	36	18	32.73
Non-user	IOB_24	chr14	104639519	C	G	KIF26A	SILENT	ENSG00000066735:[ENST00000423312(c.1626C>G,ctC>ctG):ENSP00000388241(p.Leu542(=))]	NA	NA	2.22E-02	4.60E-02	2.81E-02	2.22E-02	2.35E-02	NA	NA	NA	LOW	NA	NA	154	153	1	191	105	84	43.98
Non-user	IOB_24	chr14	104642524	C	T	KIF26A	SILENT	ENSG00000066735:[ENST00000315264(c.2982C>T,ttC>ttT):ENSP00000325452(p.Phe994(=))]	NA	NA	2.10E-02	7.06E-02	2.95E-02	2.10E-02	1.74E-02	NA	NA	NA	LOW	NA	NA	136	136	0	91	46	44	48.35
Non-user	IOB_24	chr14	105052846	G	A	C14orf180	MISSENSE	ENSG00000184601:[ENST00000331952(c.79G>A,Gcg>Acg):ENSP00000333041(p.Ala27Thr)]	NA	NA	9.98E-04	2.46E-03	4.34E-03	9.98E-04	3.10E-03	NA	NA	NA	MODIFIER	NA	NA	56	56	0	38	16	22	57.89
Non-user	IOB_24	chr14	105052846	G	A	TMEM179	INTRONIC	ENSG00000258986:[ENST00000415614(c.522+8656C>T)]	NA	NA	9.98E-04	2.46E-03	4.34E-03	9.98E-04	3.10E-03	NA	NA	NA	MODIFIER	NA	NA	56	56	0	38	16	22	57.89
Non-user	IOB_24	chr14	105054196	C	A	C14orf180	SILENT	ENSG00000184601:[ENST00000331952(c.222C>A,ccC>ccA):ENSP00000333041(p.Pro74(=))]	NA	NA	2.60E-03	1.26E-03	1.47E-03	2.60E-03	8.20E-03	NA	NA	NA	LOW	NA	NA	230	229	0	226	107	119	52.65
Non-user	IOB_24	chr14	105054196	C	A	TMEM179	INTRONIC	ENSG00000258986:[ENST00000415614(c.522+7306G>T)]	NA	NA	2.60E-03	1.26E-03	1.47E-03	2.60E-03	8.20E-03	NA	NA	NA	LOW	NA	NA	230	229	0	226	107	119	52.65
Non-user	IOB_24	chr14	105167868	C	T	INF2	MISSENSE	ENSG00000203485:[ENST00000330634(c.166C>T,Cgc>Tgc):ENSP00000376406(p.Arg56Cys)]	NA	NA	NA	NA	NA	NA	NA	NA	NA	NA	MODERATE	NA	1	154	153	1	112	66	45	40.18
Non-user	IOB_24	chr14	105258868	C	T	AKT1	INTRONIC	ENSG00000142208:[ENST00000349310(c.46+67G>A)]	NA	NA	NA	NA	NA	NA	NA	NA	NA	NA	MODIFIER	NA	NA	223	220	0	223	102	116	52.02
Non-user	IOB_24	chr14	105353011	C	T	CEP170B	MISSENSE	ENSG00000099814:[ENST00000453495(c.2438C>T,cCg>cTg):ENSP00000407238(p.Pro813Leu)]	NA	NA	6.79E-03	3.44E-03	3.28E-02	6.79E-03	2.76E-02	T	BN	NA	MODERATE	N	0.005	298	296	2	245	131	114	46.53
Non-user	IOB_24	chr14	106368455	A	G	KIAA0125	NCT	9834:[XR_245734.1(n.71+12400A>G)]	NA	NA	NA	NA	NA	NA	NA	NA	NA	NA	MODIFIER	NA	NA	25	25	0	25	19	5	20
Non-user	IOB_24	chr14	106379020	G	A	KIAA0125	NCT	9834:[XR_245734.1(n.72-7902G>A)]	NA	NA	NA	NA	NA	NA	NA	NA	NA	NA	MODIFIER	NA	NA	165	165	0	132	64	68	51.52
Non-user	IOB_24	chr14	106380171	C	T	KIAA0125	NCT	9834:[XR_245734.1(n.72-6751C>T)]	NA	NA	chr14*106380171*106380171*C*A,T*rs3794478*NA*NA*NA	1.33E-01	7.94E-02	NA	NA	NA	NA	NA	MODIFIER	NA	NA	33	33	0	27	15	11	40.74
Non-user	IOB_24	chr14	106758124	C	T	NA	INTERGENIC	NA	NA	NA	NA	8.32E-06	6.09E-05	NA	NA	NA	NA	NA	MODIFIER	NA	NA	57	57	0	87	37	50	57.47
Non-user	IOB_24	chr14	107035142	G	A	IGHV5-51	SILENT	ENSG00000211966:[ENST00000390626(c.21C>T,ctC>ctT):ENSP00000375035(p.Leu7(=))]	NA	NA	2.14E-02	1.00E-01	1.21E-01	2.14E-02	3.48E-02	NA	NA	NA	LOW	NA	NA	260	259	0	117	61	56	47.86
Non-user	IOB_24	chr14	107178941	T	A	IGHV2-70	MISSENSE	ENSG00000211974:[ENST00000390634(c.236A>T,tAc>tTc):ENSP00000375043(p.Tyr79Phe)]	NA	NA	NA	3.08E-01	2.28E-01	NA	NA	T	BN	NA	MODERATE	NA	NA	229	225	0	168	91	75	44.64
Non-user	IOB_24	chr14	107259510	G	A	IGHV5-78	MISSENSE	28387:[NC_000014.8:IGHV5-78:u_t_1(c.169C>T,Cgc>Tgc):NC_000014.8:IGHV5-78:u_t_1(p.Arg57Cys)]	NA	NA	1.60E-03	4.39E-03	2.23E-02	1.60E-03	8.20E-03	NA	NA	NA	MODERATE	NA	NA	176	176	0	159	80	78	49.06
Non-user	IOB_24	chr15	20450376	G	A	RP11-492D6.3	NCT	ENSG00000258628:[ENST00000557586(n.1685-30C>T)]	NA	NA	NA	NA	NA	NA	NA	NA	NA	NA	MODIFIER	NA	NA	363	363	0	238	195	42	17.65
Non-user	IOB_24	chr15	20462603	G	T	RP11-492D6.3	EXONIC-NC	ENSG00000258628:[ENST00000557586(n.1149C>A)]	NA	chr15*20462603*20462603*COSM959759*G*A*1*endometrium*1	chr15*20462603*20462603*G*A,T*rs2880572*NA*NA*NA	NA	NA	NA	NA	NA	NA	NA	MODIFIER	NA	NA	130	114	13	119	86	33	27.73
Non-user	IOB_24	chr15	20482094	C	T	RP11-492D6.3	EXONIC-NC	ENSG00000258628:[ENST00000557586(n.663G>A)]	NA	NA	NA	NA	NA	NA	NA	NA	NA	NA	MODIFIER	NA	NA	388	384	0	222	142	80	36.04
Non-user	IOB_24	chr15	20588705	C	A	HERC2P3	EXONIC-NC	ENSG00000180229:[ENST00000426501(n.2236G>T)]	NA	chr15*20588705*20588705*COSM147862*C*A*1*stomach*1	NA	2.79E-01	2.40E-01	NA	NA	NA	NA	NA	MODIFIER	NA	NA	373	372	0	277	209	66	23.83
Non-user	IOB_24	chr15	22319166	G	T	LOC727924	NCT	727924:[NR_015416.1(n.433+195G>T)]	NA	NA	NA	NA	NA	NA	NA	NA	NA	NA	MODIFIER	NA	NA	501	497	0	389	293	95	24.42
Non-user	IOB_24	chr15	22319166	G	T	OR11K1P	EXONIC-NC	ENSG00000259324:[ENST00000558299(n.620C>A)]	NA	NA	NA	NA	NA	NA	NA	NA	NA	NA	MODIFIER	NA	NA	501	497	0	389	293	95	24.42
Non-user	IOB_24	chr15	22319166	G	T	RP11-69H14.6	NCT	ENSG00000259176:[ENST00000560134(n.694+195G>T)]	NA	NA	NA	NA	NA	NA	NA	NA	NA	NA	MODIFIER	NA	NA	501	497	0	389	293	95	24.42

Patel *et al.*, 2021. Whole-exome sequencing analysis of oral squamous cell carcinoma delineated by tobacco usage habits
Supplementary Table 4. List of somatic single nucleotide variants identified using whole exome sequencing of OSCC patients

Cohort	Sample	Chromosome	Genomic position	Reference allele	Alternate allele	Genesymbol	Variant type	Variant description	OncoMD	COSMIC	dbSNP	ExAC	ExAC_SAS	1000G	1000G_SAS	SIFT	Polyphen2	CONDEL	VEP	LRT	PhastCon7Way	Total reads in Normal	Reference read support in Normal	Alternative read support in Normal	Total reads in Tumor	Reference read support in Tumor	Alternate read support in Tumor	Alternate allele frequency in Tumor
Non-user	IOB_24	chr15	22319295	T	C	LOC727924	NCT	727924:[NR_015416.1(n.433+324T>C)]	NA	NA	NA	NA	NA	NA	NA	NA	NA	NA	MODIFIER	NA	NA	458	457	0	328	231	96	29.27
Non-user	IOB_24	chr15	22319295	T	C	OR11K1P	EXONIC-NC	ENSG00000259324:[ENST00000558299(n.491A>G)]	NA	NA	NA	NA	NA	NA	NA	NA	NA	NA	MODIFIER	NA	NA	458	457	0	328	231	96	29.27
Non-user	IOB_24	chr15	22319295	T	C	RP11-69H14.6	NCT	ENSG00000259176:[ENST00000559392(n.195+324T>C)]	NA	NA	NA	NA	NA	NA	NA	NA	NA	NA	MODIFIER	NA	NA	458	457	0	328	231	96	29.27
Non-user	IOB_24	chr15	22332859	G	T	LOC727924	NCT	727924:[NR_015416.1(n.822+83G>T)]	NA	NA	NA	NA	NA	NA	NA	NA	NA	NA	MODIFIER	NA	NA	513	511	1	357	279	77	21.57
Non-user	IOB_24	chr15	22332859	G	T	OR4Q1P	EXONIC-NC	ENSG00000258652:[ENST00000557009(n.45G>T)]	NA	NA	NA	NA	NA	NA	NA	NA	NA	NA	MODIFIER	NA	NA	513	511	1	357	279	77	21.57
Non-user	IOB_24	chr15	22332859	G	T	RP11-69H14.6	NCT	ENSG00000259176:[ENST00000557855(n.111-13813G>T)]	NA	NA	NA	NA	NA	NA	NA	NA	NA	NA	MODIFIER	NA	NA	513	511	1	357	279	77	21.57
Non-user	IOB_24	chr15	22345097	C	T	LOC727924	NCT	727924:[NR_015416.1(n.823-1575C>T)]	NA	NA	NA	NA	NA	NA	NA	NA	NA	NA	MODIFIER	NA	NA	536	534	0	510	377	129	25.29
Non-user	IOB_24	chr15	22345097	C	T	OR4H6P	EXONIC-NC	ENSG00000261277:[ENST00000563771(n.438C>T)]	NA	NA	NA	NA	NA	NA	NA	NA	NA	NA	MODIFIER	NA	NA	536	534	0	510	377	129	25.29
Non-user	IOB_24	chr15	22345097	C	T	RP11-69H14.6	NCT	ENSG00000259176:[ENST00000560193(n.435-1575C>T)]	NA	NA	NA	NA	NA	NA	NA	NA	NA	NA	MODIFIER	NA	NA	536	534	0	510	377	129	25.29
Non-user	IOB_24	chr15	22382821	G	C	LOC101927079	NCT	101927079:[NR_110480.1(n.1102+79G>C)]	NA	NA	NA	2.41E-01	1.75E-01	NA	NA	NA	NA	NA	MODIFIER	NA	NA	390	390	0	327	244	82	25.08
Non-user	IOB_24	chr15	22382821	G	C	OR4N4	MISSENSE	ENSG00000183706:[ENST00000328795(c.349G>C,Gtg>Ctg):ENSP00000332500(p.Val117Leu)]	NA	NA	NA	2.41E-01	1.75E-01	NA	NA	NA	NA	NA	MODIFIER	NA	NA	390	390	0	327	244	82	25.08
Non-user	IOB_24	chr15	22382821	G	C	RP11-69H14.6	NCT	ENSG00000259176:[ENST00000558798(n.556-25446G>C)]	NA	NA	NA	2.41E-01	1.75E-01	NA	NA	NA	NA	NA	MODIFIER	NA	NA	390	390	0	327	244	82	25.08
Non-user	IOB_24	chr15	22382828	C	A	LOC101927079	NCT	101927079:[NR_110480.1(n.1102+86C>A)]	NA	NA	NA	2.05E-01	1.71E-01	NA	NA	NA	NA	NA	MODIFIER	NA	NA	402	402	0	331	248	82	24.77
Non-user	IOB_24	chr15	22382828	C	A	OR4N4	MISSENSE	ENSG00000183706:[ENST00000328795(c.356C>A,gCc>gAc):ENSP00000332500(p.Ala119Asp)]	NA	NA	NA	2.05E-01	1.71E-01	NA	NA	NA	NA	NA	MODIFIER	NA	NA	402	402	0	331	248	82	24.77
Non-user	IOB_24	chr15	22382828	C	A	RP11-69H14.6	NCT	ENSG00000259176:[ENST00000558798(n.556-25439C>A)]	NA	NA	NA	2.05E-01	1.71E-01	NA	NA	NA	NA	NA	MODIFIER	NA	NA	402	402	0	331	248	82	24.77
Non-user	IOB_24	chr15	22466159	G	T	IGHV1OR15-3	EXONIC-NC	ENSG00000271288:[ENST00000564926(n.250C>A)]	NA	NA	1.04E-02	6.70E-03	4.80E-02	1.04E-02	5.32E-02	NA	NA	NA	MODIFIER	NA	NA	915	906	5	699	541	157	22.46
Non-user	IOB_24	chr15	22928094	C	T	CYFIP1	NCT	ENSG00000068793:[ENST00000559393(n.280-35C>T)]	NA	NA	3.99E-04	4.46E-04	2.56E-03	3.99E-04	2.00E-03	NA	NA	NA	MODIFIER	NA	NA	256	256	0	208	90	118	56.73
Non-user	IOB_24	chr15	23181922	T	C	NA	INTERGENIC	NA	NA	NA	NA	NA	NA	NA	NA	NA	NA	NA	MODIFIER	NA	NA	150	150	0	143	121	21	14.69
Non-user	IOB_24	chr15	23811904	T	C	MKRN3	SILENT	ENSG00000179455:[ENST00000314520(c.975T>C,aaT>aaC):ENSP00000313881(p.Asn325(=))]	NA	NA	chr15*23811904*23811904*T*A,C,G*rs776038669*NA*NA*NA	8.24E-06	6.06E-05	NA	NA	NA	NA	NA	LOW	NA	0.976	193	191	0	144	72	72	50
Non-user	IOB_24	chr15	25331737	A	G	SNHG14	NCT	ENSG00000224078:[ENST00000549804(n.1894-877A>G)]	NA	NA	1.60E-03	2.72E-03	3.14E-03	1.60E-03	1.00E-03	NA	NA	NA	MODIFIER	NA	NA	328	326	0	273	161	111	40.66
Non-user	IOB_24	chr15	25331737	A	G	SNORD116-19	EXONIC-NC	727708:[NR_001290.2(n.65A>G)]	NA	NA	1.60E-03	2.72E-03	3.14E-03	1.60E-03	1.00E-03	NA	NA	NA	MODIFIER	NA	NA	328	326	0	273	161	111	40.66
Non-user	IOB_24	chr15	25470457	G	A	SNHG14	NCT	ENSG00000224078:[ENST00000453082(n.1061+164G>A)]	NA	NA	1.68E-02	2.84E-02	3.19E-02	1.68E-02	3.17E-02	NA	NA	NA	MODIFIER	NA	NA	91	91	0	99	64	35	35.35
Non-user	IOB_24	chr15	26147627	C	T	RP11-108I9.1	EXONIC-NC	ENSG00000206187:[ENST00000557171(n.48C>T)]	NA	NA	1.40E-03	NA	NA	1.40E-03	7.20E-03	NA	NA	NA	MODIFIER	NA	NA	58	58	0	37	20	17	45.95
Non-user	IOB_24	chr15	28375699	G	A	HERC2	SILENT	ENSG00000128731:[ENST00000261609(c.12612C>T,tgC>tgT):ENSP00000261609(p.Cys4204(=))]	NA	chr15*28375699*28375699*COSM1493407*G*A*1*kidney*1	4.79E-03	5.53E-03	2.40E-02	4.79E-03	1.94E-02	NA	NA	NA	LOW	NA	0.995	146	146	0	123	51	72	58.54
Non-user	IOB_24	chr15	28517492	C	A	HERC2	MISSENSE	ENSG00000128731:[ENST00000261609(c.952G>T,Gac>Tac):ENSP00000261609(p.Asp318Tyr)]	NA	NA	NA	4.08E-02	7.57E-03	NA	NA	D	BN	NA	MODERATE	D	1	65	64	0	66	55	11	16.67
Non-user	IOB_24	chr15	29008851	C	T	RP11-578F21.9	EXONIC-NC	ENSG00000260844:[ENST00000562515(n.447G>A)]	NA	NA	NA	NA	NA	NA	NA	NA	NA	NA	MODIFIER	NA	NA	196	195	0	105	85	18	17.14
Non-user	IOB_24	chr15	29561203	C	T	FAM189A1	INTRONIC	23359:[XM_005254261.1(c.160-16521G>A)]	NA	NA	NA	NA	NA	NA	NA	T	PoD	NA	MODERATE	N	0.992	211	208	0	179	99	77	43.02
Non-user	IOB_24	chr15	29561203	C	T	NDNL2	MISSENSE	ENSG00000185115:[ENST00000332303(c.707G>A,cGg>cAg):ENSP00000330694(p.Arg236Gln)]	NA	NA	NA	NA	NA	NA	NA	T	PoD	NA	MODERATE	N	0.992	211	208	0	179	99	77	43.02
Non-user	IOB_24	chr15	30054502	A	G	TJP1	INTRONIC	7082:[NM_001301025.1(c.894+36T>C)]	NA	NA	2.80E-03	1.86E-03	1.41E-02	2.80E-03	1.43E-02	NA	NA	NA	MODIFIER	NA	NA	47	47	0	37	20	17	45.95
Non-user	IOB_24	chr15	31197144	C	T	FAN1	MISSENSE	ENSG00000198690:[ENST00000362065(c.278C>T,aCc>aTc):ENSP00000354497(p.Thr93Ile)]	NA	NA	2.00E-04	2.39E-04	1.76E-03	2.00E-04	1.00E-03	T	BN	NA	MODERATE	N	0	262	262	0	212	102	110	51.89
Non-user	IOB_24	chr15	31320528	C	T	RP11-348B17.1	NCT	ENSG00000259720:[ENST00000561299(n.19-7428C>T)]	NA	NA	3.79E-03	1.09E-03	7.87E-03	3.79E-03	1.94E-02	NA	NA	NA	LOW	NA	NA	35	35	0	33	17	16	48.48
Non-user	IOB_24	chr15	31320528	C	T	TRPM1	INTRONIC-SS-DNR-PRX	ENSG00000134160:[ENST00000558768(c.2999+7G>A)]	NA	NA	3.79E-03	1.09E-03	7.87E-03	3.79E-03	1.94E-02	NA	NA	NA	LOW	NA	NA	35	35	0	33	17	16	48.48
Non-user	IOB_24	chr15	31341564	T	C	TRPM1	INTRONIC	ENSG00000134160:[ENST00000256552(c.1572+14A>G)]	NA	NA	2.32E-02	3.77E-02	4.69E-02	2.32E-02	3.48E-02	NA	NA	NA	MODIFIER	NA	NA	98	98	0	78	36	42	53.85
Non-user	IOB_24	chr15	31342673	C	A	TRPM1	MISSENSE	ENSG00000134160:[ENST00000256552(c.1376G>T,gGg>gTg):ENSP00000256552(p.Gly459Val)]	NA	NA	3.00E-03	1.00E-03	7.27E-03	3.00E-03	1.53E-02	T	BN	NA	MODERATE	NA	NA	217	214	0	132	71	61	46.21
Non-user	IOB_24	chr15	32404404	G	A	CHRNA7	INTRONIC	ENSG00000175344:[ENST00000306901(c.350+304G>A)]	NA	NA	1.42E-02	3.11E-02	3.61E-02	1.42E-02	3.48E-02	NA	NA	NA	MODIFIER	NA	NA	117	117	0	107	57	49	45.79
Non-user	IOB_24	chr15	33010483	T	C	GREM1	INTRONIC	26585:[XM_005254303.1(c.167+121T>C)]	NA	NA	3.18E-02	3.98E-02	2.45E-02	3.17E-02	1.12E-02	NA	NA	NA	MODIFIER	NA	NA	274	272	0	193	94	98	50.78
Non-user	IOB_24	chr15	33010483	T	C	LOC100131315	EXONIC-NC	100131315:[NR_109767.1(n.404A>G)]	NA	NA	3.18E-02	3.98E-02	2.45E-02	3.17E-02	1.12E-02	NA	NA	NA	MODIFIER	NA	NA	274	272	0	193	94	98	50.78
Non-user	IOB_24	chr15	33010483	T	C	LOC101926927	5UTR	101926927:[XM_005254829.1(c.-307A>G)]	NA	NA	3.18E-02	3.98E-02	2.45E-02	3.17E-02	1.12E-02	NA	NA	NA	MODIFIER	NA	NA	274	272	0	193	94	98	50.78
Non-user	IOB_24	chr15	33010483	T	C	RP11-758N13.1	EXONIC-NC	ENSG00000259721:[ENST00000558441(n.726A>G)]	NA	NA	3.18E-02	3.98E-02	2.45E-02	3.17E-02	1.12E-02	NA	NA	NA	MODIFIER	NA	NA	274	272	0	193	94	98	50.78
Non-user	IOB_24	chr15	33202823	A	G	FMN1	INTRONIC	ENSG00000248905:[ENST00000561249(c.2933-87T>C)]	NA	NA	1.14E-02	NA	NA	1.14E-02	1.23E-02	NA	NA	NA	MODIFIER	NA	NA	171	169	1	141	76	65	46.1
Non-user	IOB_24	chr15	33359093	T	C	FMN1	SILENT	ENSG00000248905:[ENST00000334528(c.993A>G,aaA>aaG):ENSP00000333950(p.Lys331(=))]	NA	NA	5.79E-03	7.47E-03	5.94E-03	5.79E-03	5.10E-03	NA	NA	NA	MODIFIER	NA	NA	156	156	0	129	73	56	43.41
Non-user	IOB_24	chr15	33381040	C	G	FMN1	MISSENSE	ENSG00000248905:[ENST00000559047(c.2003G>C,aGg>aCg):ENSP00000454047(p.Arg668Thr)]	NA	NA	1.06E-02	1.46E-02	1.25E-02	1.06E-02	1.23E-02	D_lc	UN	NA	MODERATE	NA	NA	230	230	0	193	104	88	45.6
Non-user	IOB_24	chr15	34157430	C	G	RYR3	3UTR	ENSG00000198838:[ENST00000389232(c.*3C>G)]	NA	NA	3.73E-02	3.47E-02	1.00E-02	3.73E-02	9.20E-03	NA	NA	NA	MODIFIER	NA	NA	261	260	1	208	102	105	50.48
Non-user	IOB_24	chr15	34168130	T	A	AVEN	MISSENSE	ENSG00000169857:[ENST00000560649(c.66A>T,aAa>aTa):ENSP00000452906(p.Lys23Ile)]	NA	NA	8.39E-03	4.05E-03	1.28E-02	8.39E-03	1.84E-02	NA	NA	NA	MODIFIER	NA	NA	291	289	0	215	131	82	38.14
Non-user	IOB_24	chr15	34434665	A	C	KATNBL1	NCT	ENSG00000134152:[ENST00000558681(n.459-93T>G)]	NA	NA	5.19E-03	NA	NA	5.19E-03	6.10E-03	NA	NA	NA	MODIFIER	NA	NA	133	132	0	120	75	45	37.5
Non-user	IOB_24	chr15	38857450	G	T	RASGRP1	INTRONIC	ENSG00000172575:[ENST00000561180(c.188+53C>A)]	NA	NA	chr15*38857450*38857450*G*A*rs925251828*NA*NA*NA	NA	NA	NA	NA	NA	NA	NA	MODIFIER	NA	NA	96	94	1	69	42	26	37.68
Non-user	IOB_24	chr15	40030417	A	C	FSIP1	INTRONIC	161835:[XM_005254210.1(c.88-15T>G)]	NA	NA	1.16E-02	2.09E-02	1.40E-02	1.16E-02	6.10E-03	NA	NA	NA	MODIFIER	NA	NA	124	122	0	94	47	47	50
Non-user	IOB_24	chr15	40587107	G	A	PLCB2	INTRONIC	ENSG00000137841:[ENST00000260402(c.1906+30C>T)]	NA	NA	9.98E-04	4.53E-04	3.10E-03	9.98E-04	4.10E-03	NA	NA	NA	MODIFIER	NA	NA	124	124	0	82	57	25	30.49
Non-user	IOB_24	chr15	40914831	C	G	CASC5	MISSENSE	ENSG00000137812:[ENST00000346991(c.2447C>G,aCt>aGt):ENSP00000335463(p.Thr816Ser)]	NA	NA	7.19E-03	1.88E-02	2.31E-02	7.19E-03	8.20E-03	NA	NA	NA	MODIFIER	NA	NA	182	181	1	94	46	48	51.06
Non-user	IOB_24	chr15	41060084	A	T	DNAJC17	EXONIC-NC	ENSG00000104129:[ENST00000561110(n.516T>A)]	NA	NA	3.18E-02	NA	NA	3.17E-02	8.28E-02	NA	NA	NA	MODIFIER	NA	NA	46	45	0	34	14	20	58.82
Non-user	IOB_24	chr15	41689043	G	C	NDUFAF1	MISSENSE	ENSG00000137806:[ENST00000260361(c.215C>G,aCt>aGt):ENSP00000260361(p.Thr72Ser)]	NA	NA	4.19E-03	3.25E-03	1.85E-02	4.19E-03	1.94E-02	NA	NA	NA	MODIFIER	NA	NA	109	109	0	82	44	37	45.12
Non-user	IOB_24	chr15	41803754	G	A	LTK	MISSENSE	ENSG00000062524:[ENST00000263800(c.680C>T,cCg>cTg):ENSP00000263800(p.Pro227Leu)]	NA	NA	3.20E-02	8.13E-02	9.24E-02	3.19E-02	4.40E-02	NA	NA	NA	MODIFIER	NA	NA	128	126	2	87	50	37	42.53
Non-user	IOB_24	chr15	41816259	C	T	RPAP1	INTRONIC	ENSG00000103932:[ENST00000565035(c.407+35G>A)]	NA	NA	2.02E-02	3.52E-02	5.81E-02	2.02E-02	4.19E-02	NA	NA	NA	MODIFIER	NA	NA	80	80	0	70	37	32	45.71
Non-user	IOB_24	chr15	41822163	C	T	RPAP1	MISSENSE	ENSG00000103932:[ENST00000304330(c.958G>A,Gtg>Atg):ENSP00000306123(p.Val320Met)]	NA	NA	3.30E-02	5.41E-02	5.73E-02	3.29E-02	4.60E-02	NA	NA	NA	MODIFIER	NA	NA	136	136	0	132	83	49	37.12
Non-user	IOB_24	chr15	42137324	G	A	JMJD7-PLA2G4B	NCT	ENSG00000168970:[ENST00000490848(n.5816+56G>A)]	NA	NA	1.20E-03	NA	NA	1.20E-03	6.10E-03	NA	NA	NA	MODIFIER	NA	NA	208	208	0	166	87	78	46.99
Non-user	IOB_24	chr15	42137324	G	A	PLA2G4B	NCT	ENSG00000243708:[ENST00000483748(n.1648+56G>A)]	NA	NA	1.20E-03	NA	NA	1.20E-03	6.10E-03	NA	NA	NA	MODIFIER	NA	NA	208	208	0	166	87	78	46.99
Non-user	IOB_24	chr15	42148830	G	A	SPTBN5	INTRONIC	ENSG00000137877:[ENST00000320955(c.8809-34C>T)]	NA	NA	1.98E-02	3.70E-02	4.41E-02	1.98E-02	5.62E-02	NA	NA	NA	MODIFIER	NA	NA	137	137	0	81	54	27	33.33
Non-user	IOB_24	chr15	42162467	G	A	SPTBN5	NONSENSE	ENSG00000137877:[ENST00000320955(c.5647C>T,Cga>Tga):ENSP00000317790(p.Arg1883Ter)]	NA	NA	1.08E-02	3.56E-02	2.14E-02	1.08E-02	1.12E-02	NA	NA	NA	HIGH	NA	0.005	96	96	0	78	46	32	41.03
Non-user	IOB_24	chr15	42162713	G	A	SPTBN5	SILENT	ENSG00000137877:[ENST00000320955(c.5493C>T,caC>caT):ENSP00000317790(p.His1831(=))]	NA	NA	3.02E-02	4.55E-02	5.21E-02	3.02E-02	6.34E-02	NA	NA	NA	LOW	NA	0	199	199	0	184	90	93	50.54
Non-user	IOB_24	chr15	42167165	T	C	SPTBN5	SILENT	ENSG00000137877:[ENST00000320955(c.4377A>G,aaA>aaG):ENSP00000317790(p.Lys1459(=))]	NA	NA	2.60E-03	5.90E-03	8.77E-03	2.60E-03	6.10E-03	NA	NA	NA	LOW	NA	0.779	99	98	0	77	37	40	51.95
Non-user	IOB_24	chr15	42167268	A	G	SPTBN5	INTRONIC	ENSG00000137877:[ENST00000320955(c.4288-14T>C)]	NA	NA	4.83E-02	7.03E-02	6.18E-02	4.83E-02	5.93E-02	NA	NA	NA	MODIFIER	NA	NA	98	98	0	90	37	53	58.89
Non-user	IOB_24	chr15	42169129	G	A	SPTBN5	SILENT	ENSG00000137877:[ENST00000320955(c.3729C>T,gcC>gcT):ENSP00000317790(p.Ala1243(=))]	NA	NA	1.72E-02	4.01E-02	2.64E-02	1.72E-02	1.33E-02	NA	NA	NA	LOW	NA	NA	27	27	0	19	9	10	52.63
Non-user	IOB_24	chr15	42171654	G	A	SPTBN5	SILENT	ENSG00000137877:[ENST00000320955(c.2986C>T,Ctg>Ttg):ENSP00000317790(p.Leu996(=))]	NA	NA	3.08E-02	6.88E-02	6.56E-02	3.08E-02	6.95E-02	NA	NA	NA	LOW	NA	1	76	76	0	68	32	36	52.94
Non-user	IOB_24	chr15	42171655	C	A	SPTBN5	MISSENSE	ENSG00000137877:[ENST00000320955(c.2985G>T,caG>caT):ENSP00000317790(p.Gln995His)]	NA	NA	3.08E-02	6.99E-02	6.63E-02	3.08E-02	6.95E-02	T	BN	NA	MODERATE	N	0.996	76	76	0	67	31	36	53.73
Non-user	IOB_24	chr15	42174413	C	A	SPTBN5	INTRONIC	ENSG00000137877:[ENST00000320955(c.2247+54G>T)]	NA	NA	3.10E-02	NA	NA	3.10E-02	6.95E-02	NA	NA	NA	MODIFIER	NA	NA	10	10	0	11	3	8	72.73
Non-user	IOB_24	chr15	42192885	G	A	EHD4	SILENT	ENSG00000103966:[ENST00000220325(c.1584C>T,ctC>ctT):ENSP00000220325(p.Leu528(=))]	NA	NA	4.09E-02	6.31E-02	5.57E-02	4.09E-02	6.65E-02	NA	NA	NA	LOW	NA	NA	118	116	0	92	51	40	43.48
Non-user	IOB_24	chr15	42433976	C	G	PLA2G4F	3UTR	ENSG00000168907:[ENST00000382396(c.*206G>C)]	NA	NA	5.79E-03	NA	NA	5.79E-03	9.20E-03	NA	NA	NA	MODIFIER	NA	NA	143	142	0	146	74	72	49.32
Non-user	IOB_24	chr15	42981021	C	T	STARD9	SILENT	ENSG00000159433:[ENST00000290607(c.7245C>T,gcC>gcT):ENSP00000290607(p.Ala2415(=))]	NA	NA	NA	NA	NA	NA	NA	NA	NA	NA	LOW	NA	NA	238	237	1	170	109	60	35.29
Non-user	IOB_24	chr15	43256191	T	C	UBR1	MISSENSE	ENSG00000159459:[ENST00000290650(c.4642A>G,Aca>Gca):ENSP00000290650(p.Thr1548Ala)]	NA	NA	3.32E-02	6.38E-02	1.04E-01	3.31E-02	7.16E-02	T	BN	NA	MODERATE	D	1	89	89	0	42	0	41	97.62
Non-user	IOB_24	chr15	43427757	C	T	EPB42	NCT	ENSG00000166947:[ENST00000570199(n.95-4417G>A)]	NA	NA	3.99E-04	2.48E-04	1.53E-03	3.99E-04	2.00E-03	NA	NA	NA	MODIFIER	NA	NA	67	67	0	76	42	34	44.74
Non-user	IOB_24	chr15	43427757	C	T	TMEM62	MISSENSE	ENSG00000137842:[ENST00000564494(c.135C>T,Cat>Tat):ENSP00000458098(p.His46Tyr)]	NA	NA	3.99E-04	2.48E-04	1.53E-03	3.99E-04	2.00E-03	NA	NA	NA	MODIFIER	NA	NA	67	67	0	76	42	34	44.74
Non-user	IOB_24	chr15	43569105	A	G	TGM7	MISSENSE	ENSG00000159495:[ENST00000452443(c.1928T>C,aTg>aCg):ENSP00000389466(p.Met643Thr)]	NA	NA	NA	NA	NA	NA	NA	NA	NA	NA	MODIFIER	NA	NA	145	144	0	114	59	54	47.37
Non-user	IOB_24	chr15	43821392	G	A	MAP1A	MISSENSE	ENSG00000166963:[ENST00000382031(c.8435G>A,cGc>cAc):ENSP00000371462(p.Arg2812His)]	NA	chr15*43821392*43821392*COSM3690407*G*A*1*large_intestine*1	7.99E-04	2.58E-04	1.62E-03	7.99E-04	3.10E-03	NA	PrD	NA	MODERATE	NA	NA	116	107	0	70	32	32	45.71
Non-user	IOB_24	chr15	43973324	T	C	AC011330.5	EXONIC-NC	ENSG00000249839:[ENST00000510070(n.875A>G)]	NA	NA	NA	6.66E-05	0.00E+00	NA	NA	NA	NA	NA	MODIFIER	NA	NA	287	263	24	41	0	41	100
Non-user	IOB_24	chr15	43973324	T	C	STRC	INTRONIC	161497:[XM_005254193.1(c.-49+28658A>G)]	NA	NA	NA	6.66E-05	0.00E+00	NA	NA	NA	NA	NA	MODIFIER	NA	NA	287	263	24	41	0	41	100
Non-user	IOB_24	chr15	44037323	C	T	CATSPER2P1	EXONIC-NC	ENSG00000205771:[ENST00000429276(n.607G>A)]	NA	NA	9.39E-03	NA	NA	9.38E-03	2.86E-02	NA	NA	NA	MODIFIER	NA	NA	74	73	0	51	29	21	41.18
Non-user	IOB_24	chr15	45428712	C	T	DUOX1	INTRONIC	ENSG00000137857:[ENST00000321429(c.926-15C>T)]	NA	NA	4.59E-02	8.37E-02	1.08E-01	4.59E-02	7.46E-02	NA	NA	NA	MODIFIER	NA	NA	104	102	0	84	34	48	57.14
Non-user	IOB_24	chr15	45491136	C	G	CTD-2651B20.7	EXONIC-NC	ENSG00000259932:[ENST00000568314(n.202G>C)]	NA	NA	3.95E-02	7.94E-02	5.83E-02	3.95E-02	3.58E-02	D_lc	BN	NA	MODERATE	N	0	199	198	0	151	77	73	48.34
Non-user	IOB_24	chr15	45491136	C	G	RP11-519G16.2	NCT	ENSG00000259519:[ENST00000560034(n.192-920C>G)]	NA	NA	3.95E-02	7.94E-02	5.83E-02	3.95E-02	3.58E-02	D_lc	BN	NA	MODERATE	N	0	199	198	0	151	77	73	48.34
Non-user	IOB_24	chr15	45491136	C	G	SHF	MISSENSE	ENSG00000138606:[ENST00000318390(c.308G>C,tGg>tCg):ENSP00000315978(p.Trp103Ser)]	NA	NA	3.95E-02	7.94E-02	5.83E-02	3.95E-02	3.58E-02	D_lc	BN	NA	MODERATE	N	0	199	198	0	151	77	73	48.34
Non-user	IOB_24	chr15	49420853	C	T	COPS2	INTRONIC	ENSG00000166200:[ENST00000299259(c.1208+46G>A)]	NA	NA	4.63E-02	2.12E-02	6.25E-02	4.63E-02	8.69E-02	NA	NA	NA	MODIFIER	NA	NA	45	45	0	39	19	20	51.28
Non-user	IOB_24	chr15	49461177	T	G	GALK2	INTRONIC	ENSG00000156958:[ENST00000560654(c.-20+10012T>G)]	NA	NA	4.53E-02	NA	NA	4.53E-02	8.69E-02	NA	NA	NA	MODIFIER	NA	NA	70	69	0	46	31	15	32.61
Non-user	IOB_24	chr15	49627607	T	C	FAM227B	INTRONIC	196951:[XM_005254217.1(c.1100+9A>G)]	NA	NA	4.39E-02	2.07E-02	6.10E-02	4.39E-02	8.59E-02	NA	NA	NA	MODIFIER	NA	NA	50	49	0	38	19	19	50
Non-user	IOB_24	chr15	49627607	T	C	GALK2	INTRONIC	2585:[XM_005254280.1(c.1315+7280T>C)]	NA	NA	4.39E-02	2.07E-02	6.10E-02	4.39E-02	8.59E-02	NA	NA	NA	MODIFIER	NA	NA	50	49	0	38	19	19	50
Non-user	IOB_24	chr15	50899514	T	C	TRPM7	SILENT	ENSG00000092439:[ENST00000313478(c.2592A>G,ttA>ttG):ENSP00000320239(p.Leu864(=))]	NA	NA	NA	1.53E-04	1.15E-03	NA	NA	NA	NA	NA	LOW	NA	NA	33	33	0	30	9	21	70
Non-user	IOB_24	chr15	50902090	G	A	TRPM7	SILENT	ENSG00000092439:[ENST00000313478(c.2349C>T,tcC>tcT):ENSP00000320239(p.Ser783(=))]	NA	NA	NA	NA	NA	NA	NA	NA	NA	NA	LOW	NA	NA	376	372	0	264	149	112	42.42
Non-user	IOB_24	chr15	51503104	T	C	CYP19A1	MISSENSE	ENSG00000137869:[ENST00000260433(c.1413A>G,atA>atG):ENSP00000260433(p.Ile471Met)]	NA	NA	NA	NA	NA	NA	NA	NA	NA	NA	MODIFIER	NA	NA	243	243	0	196	109	85	43.37
Non-user	IOB_24	chr15	51503104	T	C	RP11-108K3.1	NCT	ENSG00000259240:[ENST00000559909(n.195-67076T>C)]	NA	NA	NA	NA	NA	NA	NA	NA	NA	NA	MODIFIER	NA	NA	243	243	0	196	109	85	43.37
Non-user	IOB_24	chr15	51503112	C	T	CYP19A1	MISSENSE	ENSG00000137869:[ENST00000260433(c.1405G>A,Gag>Aag):ENSP00000260433(p.Glu469Lys)]	NA	NA	NA	8.25E-06	0.00E+00	NA	NA	NA	NA	NA	MODIFIER	NA	NA	239	238	0	199	112	86	43.22
Non-user	IOB_24	chr15	51503112	C	T	RP11-108K3.1	NCT	ENSG00000259240:[ENST00000559909(n.195-67068C>T)]	NA	NA	NA	8.25E-06	0.00E+00	NA	NA	NA	NA	NA	MODIFIER	NA	NA	239	238	0	199	112	86	43.22
Non-user	IOB_24	chr15	51975608	G	A	SCG3	MISSENSE	ENSG00000104112:[ENST00000220478(c.374G>A,aGt>aAt):ENSP00000220478(p.Ser125Asn)]	NA	NA	4.59E-02	4.97E-02	7.92E-02	4.59E-02	9.51E-02	T_lc	BN	NA	MODERATE	D	1	51	51	0	38	18	20	52.63

Patel *et al.* , 2021. Whole-exome sequencing analysis of oral squamous cell carcinoma delineated by tobacco usage habits
Supplementary Table 4. List of somatic single nucleotide variants identified using whole exome sequencing of OSCC patients

Cohort	Sample	Chromosome	Genomic position	Reference allele	Alternate allele	Genesymbol	Variant type	Variant description	OncoMD	COSMIC	dbSNP	ExAC	ExAC_SAS	1000G	1000G_SAS	SIFT	Polyphen2	CONDEL	VEP	LRT	PhastCon7Way	Total reads in Normal	Reference read support in Normal	Alternative read support in Normal	Total reads in Tumor	Reference read support in Tumor	Alternate read support in Tumor	Alternate allele frequency in Tumor
Non-user	IOB_24	chr15	54306716	G	A	UNC13C	MISSENSE	ENSG00000137766:[ENST00000260323(c.1616G>A,aGt>aAt):ENSP00000260323(p.Ser539Asn)]	NA	NA	3.22E-02	1.69E-02	1.21E-01	3.21E-02	1.63E-01	T_lc	BN	NA	MODERATE	NA	NA	359	358	0	311	168	142	45.66
Non-user	IOB_24	chr15	54542392	G	A	UNC13C	INTRONIC	ENSG00000137766:[ENST00000260323(c.3229-31G>A)]	NA	NA	1.30E-02	1.55E-02	4.18E-02	1.30E-02	4.81E-02	NA	NA	NA	MODIFIER	NA	NA	205	204	0	154	78	76	49.35
Non-user	IOB_24	chr15	58837933	C	A	LIPC	INTRONIC	3990:[XM_005254374.1(c.512-8C>A)]	NA	NA	1.86E-02	2.80E-02	3.28E-02	1.86E-02	3.48E-02	NA	NA	NA	LOW	NA	NA	88	88	0	74	42	32	43.24
Non-user	IOB_24	chr15	58968396	C	T	ADAM10	NCT	ENSG00000137845:[ENST00000497846(n.601+2927G>A)]	NA	NA	5.99E-03	NA	NA	5.99E-03	1.74E-02	NA	NA	NA	MODIFIER	NA	NA	150	150	0	89	41	48	53.93
Non-user	IOB_24	chr15	59564608	T	C	MYO1E	MISSENSE	ENSG00000157483:[ENST00000288235(c.44A>G,aAt>aGt):ENSP00000288235(p.Asn15Ser)]	NA	NA	2.00E-04	2.47E-05	0.00E+00	2.00E-04	0.00E+00	T	BN	NA	MODERATE	NA	NA	140	140	0	137	68	68	49.64
Non-user	IOB_24	chr15	60649547	T	C	ANXA2	INTRONIC	ENSG00000182718:[ENST00000396024(c.358-112A>G)]	NA	NA	9.39E-03	NA	NA	9.38E-03	4.81E-02	NA	NA	NA	MODIFIER	NA	NA	79	78	1	74	42	32	43.24
Non-user	IOB_24	chr15	60724193	A	G	NARG2	INTRONIC-SS-ACR-PRX	ENSG00000128915:[ENST00000558181(c.*2129-10T>C)]	NA	NA	7.99E-04	7.04E-04	5.01E-03	7.99E-04	4.10E-03	NA	NA	NA	MODIFIER	NA	NA	13	13	0	10	5	5	50
Non-user	IOB_24	chr15	63351873	T	C	TPM1	SILENT	ENSG00000140416:[ENST00000559831(c.258T>C,taT>taC):ENSP00000452977(p.Tyr86(=))]	NA	NA	2.42E-02	5.33E-02	1.72E-02	2.42E-02	2.04E-02	NA	NA	NA	LOW	NA	1	67	66	1	26	15	11	42.31
Non-user	IOB_24	chr15	63932590	C	A	HERC1	MISSENSE	ENSG00000103657:[ENST00000443617(c.11662G>T,Gct>Tct):ENSP00000390158(p.Ala3888Ser)]	NA	NA	NA	NA	NA	NA	NA	NA	NA	NA	MODERATE	NA	1	124	123	0	78	72	5	6.41
Non-user	IOB_24	chr15	63948598	G	A	HERC1	INTRONIC	8925:[XM_005254747.1(c.9405-41C>T)]	NA	NA	4.19E-03	1.11E-02	3.77E-03	4.19E-03	2.00E-03	NA	NA	NA	MODIFIER	NA	NA	43	43	0	41	18	23	56.1
Non-user	IOB_24	chr15	66257486	C	T	MEGF11	NCT	ENSG00000157890:[ENST00000484618(n.165-28G>A)]	NA	NA	2.60E-03	2.31E-03	8.20E-03	2.60E-03	1.02E-02	NA	NA	NA	MODIFIER	NA	NA	28	28	0	24	18	6	25
Non-user	IOB_24	chr15	66850151	C	T	LCTL	SILENT	ENSG00000188501:[ENST00000341509(c.831G>A,gaG>gaA):ENSP00000343490(p.Glu277(=))]	NA	NA	2.60E-03	1.39E-03	8.18E-03	2.60E-03	1.12E-02	NA	NA	NA	LOW	NA	0.994	70	69	0	40	20	20	50
Non-user	IOB_24	chr15	68473533	A	T	PIAS1	INTRONIC	8554:[XM_005254734.1(c.1461-17A>T)]	NA	NA	5.99E-04	2.02E-04	1.36E-03	5.99E-04	3.10E-03	NA	NA	NA	MODIFIER	NA	NA	197	196	0	177	98	78	44.07
Non-user	IOB_24	chr15	72338297	T	C	MYO9A	MISSENSE	ENSG00000066933:[ENST00000568438(c.27A>G,tAt>tGt):ENSP00000457235(p.Tyr10Cys)]	NA	NA	NA	2.23E-04	1.33E-03	NA	NA	NA	NA	NA	MODIFIER	NA	NA	274	270	0	254	136	118	46.46
Non-user	IOB_24	chr15	72743465	G	T	LOC105370888	NCT	105370888:[NR_135678.1(n.365+22751C>A)]	NA	NA	2.00E-04	NA	NA	2.00E-04	1.00E-03	NA	NA	NA	MODIFIER	NA	NA	100	99	0	57	30	27	47.37
Non-user	IOB_24	chr15	72743465	G	T	RP11-1007O24.3	NCT	ENSG00000261423:[ENST00000562573(n.335+22751C>A)]	NA	NA	2.00E-04	NA	NA	2.00E-04	1.00E-03	NA	NA	NA	MODIFIER	NA	NA	100	99	0	57	30	27	47.37
Non-user	IOB_24	chr15	72743465	G	T	RP11-1007O24.4	EXONIC-NC	ENSG00000271072:[ENST00000603694(n.468G>T)]	NA	NA	2.00E-04	NA	NA	2.00E-04	1.00E-03	NA	NA	NA	MODIFIER	NA	NA	100	99	0	57	30	27	47.37
Non-user	IOB_24	chr15	73545837	C	T	NEO1	INTRONIC	4756:[XM_005254415.1(c.2004+47C>T)]	NA	NA	NA	2.48E-05	1.22E-04	NA	NA	NA	NA	NA	MODIFIER	NA	NA	95	92	1	91	46	44	48.35
Non-user	IOB_24	chr15	73616417	C	T	HCN4	INTRONIC	ENSG00000138622:[ENST00000261917(c.2143+13G>A)]	NA	NA	3.99E-04	5.15E-05	3.66E-04	3.99E-04	2.00E-03	NA	NA	NA	MODIFIER	NA	NA	55	55	0	54	28	26	48.15
Non-user	IOB_24	chr15	74000747	A	G	CD276	SILENT	ENSG00000103855:[ENST00000561176(c.430A>G,gcA>gcG):ENSP00000453014(p.Ala144(=))]	NA	NA	4.59E-03	2.71E-03	1.71E-02	4.59E-03	2.15E-02	NA	NA	NA	LOW	NA	NA	80	80	0	61	39	22	36.07
Non-user	IOB_24	chr15	74474737	C	G	STRA6	MISSENSE	ENSG00000137868:[ENST00000563965(c.1482G>C,atG>atC):ENSP00000456609(p.Met494Ile)]	NA	NA	NA	3.93E-04	2.08E-03	NA	NA	T	BN	NA	MODERATE	D	0.586	75	74	0	51	26	25	49.02
Non-user	IOB_24	chr15	74704072	G	A	SEMA7A	INTRONIC	ENSG00000138623:[ENST00000542748(c.928-21C>T)]	NA	NA	1.20E-03	2.20E-03	2.62E-03	1.20E-03	1.00E-03	NA	NA	NA	MODIFIER	NA	NA	114	114	0	113	58	54	47.79
Non-user	IOB_24	chr15	75188506	C	T	MPI	SILENT	ENSG00000178802:[ENST00000323744(c.501C>T,aaC>aaT):ENSP00000318192(p.Asn167(=))]	NA	NA	2.34E-02	2.68E-02	6.61E-02	2.34E-02	6.24E-02	NA	NA	NA	LOW	NA	NA	82	81	0	59	37	21	35.59
Non-user	IOB_24	chr15	75981972	A	G	CSPG4	SILENT	ENSG00000173546:[ENST00000308508(c.1434T>C,caT>caC):ENSP00000312506(p.His478(=))]	NA	NA	2.26E-02	1.70E-02	1.09E-02	2.26E-02	1.02E-02	NA	NA	NA	LOW	NA	0	115	114	0	96	46	50	52.08
Non-user	IOB_24	chr15	75982063	T	C	CSPG4	MISSENSE	ENSG00000173546:[ENST00000308508(c.1343A>G,gAg>gGg):ENSP00000312506(p.Glu448Gly)]	NA	NA	NA	NA	NA	NA	NA	D	PoD	NA	MODERATE	D	1	90	89	0	98	62	35	35.71
Non-user	IOB_24	chr15	75982227	T	G	CSPG4	SILENT	ENSG00000173546:[ENST00000308508(c.1179A>C,ggA>ggC):ENSP00000312506(p.Gly393(=))]	NA	NA	NA	1.76E-01	2.17E-01	NA	NA	NA	NA	NA	LOW	NA	NA	83	83	0	67	45	22	32.84
Non-user	IOB_24	chr15	76019751	C	T	ODF3L1	MISSENSE	ENSG00000182950:[ENST00000332145(c.695C>T,aCg>aTg):ENSP00000329584(p.Thr232Met)]	NA	NA	2.00E-04	5.85E-04	2.42E-03	2.00E-04	0.00E+00	D	BN	NA	MODERATE	N	0.004	239	239	0	204	114	89	43.63
Non-user	IOB_24	chr15	78825917	A	G	HYKK	MISSENSE	ENSG00000188266:[ENST00000388988(c.1027A>G,Aaa>Gaa):ENSP00000373640(p.Lys343Glu)]	NA	NA	4.27E-02	7.53E-02	6.19E-02	4.27E-02	4.09E-02	NA	NA	NA	MODIFIER	NA	NA	200	200	0	162	71	90	55.56
Non-user	IOB_24	chr15	79083598	G	C	ADAMTS7	NCT	ENSG00000136378:[ENST00000565793(n.717-33C>G)]	NA	NA	chr15*79083598*79083598*G*A*rs377140248*NA*NA*NA	NA	NA	NA	NA	NA	NA	NA	MODIFIER	NA	NA	35	35	0	21	13	7	33.33
Non-user	IOB_24	chr15	79277257	C	T	RASGRF1	INTRONIC	ENSG00000058335:[ENST00000394745(c.1110+92G>A)]	NA	NA	NA	6.30E-05	1.94E-04	NA	NA	NA	NA	NA	MODIFIER	NA	NA	207	205	0	136	81	54	39.71
Non-user	IOB_24	chr15	79277257	C	T	RP11-16K12.1	EXONIC-NC	ENSG00000177699:[ENST00000316148(n.889C>T)]	NA	NA	NA	6.30E-05	1.94E-04	NA	NA	NA	NA	NA	MODIFIER	NA	NA	207	205	0	136	81	54	39.71
Non-user	IOB_24	chr15	79755573	C	T	KIAA1024	SILENT	ENSG00000169330:[ENST00000305428(c.2463C>T,gcC>gcT):ENSP00000307461(p.Ala821(=))]	NA	NA	6.39E-03	1.26E-02	1.37E-02	6.39E-03	1.64E-02	NA	NA	NA	LOW	NA	NA	255	253	0	214	112	101	47.2
Non-user	IOB_24	chr15	82422927	T	C	EFL1	NCT	79631:[NR_136410.1(n.3358-25A>G)]	NA	NA	1.60E-03	9.13E-04	6.56E-03	1.60E-03	8.20E-03	NA	NA	NA	MODIFIER	NA	NA	149	147	0	116	57	58	50
Non-user	IOB_24	chr15	82422927	T	C	EFTUD1	INTRONIC	ENSG00000140598:[ENST00000359445(c.3022-25A>G)]	NA	NA	1.60E-03	9.13E-04	6.56E-03	1.60E-03	8.20E-03	NA	NA	NA	MODIFIER	NA	NA	149	147	0	116	57	58	50
Non-user	IOB_24	chr15	85186837	T	C	WDR73	MISSENSE	ENSG00000177082:[ENST00000434634(c.1001A>G,gAt>gGt):ENSP00000387982(p.Asp334Gly)]	NA	NA	4.43E-02	7.55E-02	6.25E-02	4.43E-02	5.42E-02	NA	NA	NA	MODIFIER	NA	NA	152	151	0	141	65	75	53.19
Non-user	IOB_24	chr15	86312273	T	A	KLHL25	MISSENSE	ENSG00000183655:[ENST00000337975(c.769A>T,Atg>Ttg):ENSP00000336800(p.Met257Leu)]	NA	NA	9.59E-03	1.47E-02	8.39E-03	9.58E-03	1.02E-02	T	BN	NA	MODERATE	N	0.999	210	209	0	155	85	70	45.16
Non-user	IOB_24	chr15	89155121	G	A	MIR3529	EXONIC-NC	100616238:[NR_039867.1(n.35C>T)]	NA	NA	1.10E-02	7.43E-03	4.41E-02	1.10E-02	4.70E-02	NA	NA	NA	MODIFIER	NA	NA	39	39	0	38	22	16	42.11
Non-user	IOB_24	chr15	89155121	G	A	MIR7-2	EXONIC-NC	ENSG00000207703:[ENST00000384970(n.66G>A)]	NA	NA	1.10E-02	7.43E-03	4.41E-02	1.10E-02	4.70E-02	NA	NA	NA	MODIFIER	NA	NA	39	39	0	38	22	16	42.11
Non-user	IOB_24	chr15	89155163	G	A	MIR7-2	EXONIC-NC	ENSG00000207703:[ENST00000384970(n.108G>A)]	NA	NA	3.59E-03	3.18E-03	1.75E-02	3.59E-03	1.84E-02	NA	NA	NA	MODIFIER	NA	NA	40	40	0	40	20	20	50
Non-user	IOB_24	chr15	89173292	G	A	AEN	MISSENSE	ENSG00000181026:[ENST00000332810(c.745G>A,Ggc>Agc):ENSP00000331944(p.Gly249Ser)]	NA	NA	9.59E-03	4.98E-03	3.49E-02	9.58E-03	4.70E-02	NA	NA	NA	MODIFIER	NA	NA	196	192	0	130	62	68	52.31
Non-user	IOB_24	chr15	89173480	C	T	AEN	SILENT	ENSG00000181026:[ENST00000332810(c.933C>T,caC>caT):ENSP00000331944(p.His311(=))]	NA	NA	9.39E-03	9.29E-03	4.56E-02	9.38E-03	4.60E-02	NA	NA	NA	LOW	NA	NA	278	277	0	197	96	101	51.27
Non-user	IOB_24	chr15	89174287	C	T	AEN	EXONIC-NC	ENSG00000181026:[ENST00000557927(n.3814C>T)]	NA	NA	2.04E-02	1.73E-02	2.89E-02	2.04E-02	4.60E-02	NA	NA	NA	MODIFIER	NA	NA	132	132	0	97	53	44	45.36
Non-user	IOB_24	chr15	89390513	C	T	ACAN	MISSENSE	ENSG00000157766:[ENST00000352105(c.1469C>T,tCg>tTg):ENSP00000341615(p.Ser490Leu)]	NA	NA	7.19E-03	2.71E-02	4.34E-02	7.19E-03	2.76E-02	D	PoD	NA	MODERATE	NA	NA	91	91	0	78	46	31	39.74
Non-user	IOB_24	chr15	90758151	C	T	SEMA4B	INTRONIC	10509:[NM_001324030.1(c.-311-2520C>T)]	NA	NA	3.93E-02	9.45E-02	1.04E-01	3.93E-02	7.77E-02	NA	NA	NA	MODIFIER	NA	NA	195	194	0	122	67	55	45.08
Non-user	IOB_24	chr15	90903618	G	A	ZNF774	SILENT	ENSG00000196391:[ENST00000354377(c.555G>A,ggG>ggA):ENSP00000346348(p.Gly185(=))]	NA	NA	3.99E-03	1.25E-02	8.48E-04	3.99E-03	0.00E+00	NA	NA	NA	MODIFIER	NA	NA	117	117	0	85	47	38	44.71
Non-user	IOB_24	chr15	90982666	A	C	IQGAP1	INTRONIC	ENSG00000140575:[ENST00000560738(c.107-26613A>C)]	NA	NA	7.19E-03	4.78E-03	3.23E-02	7.19E-03	3.48E-02	NA	NA	NA	MODIFIER	NA	NA	117	117	0	70	33	36	51.43
Non-user	IOB_24	chr15	91420904	G	A	FURIN	INTRONIC	ENSG00000140564:[ENST00000268171(c.667+59G>A)]	NA	NA	NA	NA	NA	NA	NA	NA	NA	NA	MODIFIER	NA	NA	60	60	0	58	27	30	51.72
Non-user	IOB_24	chr15	91795173	T	A	SV2B	SILENT	ENSG00000185518:[ENST00000545111(c.123T>A,tcT>tcA):ENSP00000443243(p.Ser41(=))]	NA	NA	2.16E-02	3.25E-02	5.40E-02	2.16E-02	5.11E-02	NA	NA	NA	MODIFIER	NA	NA	109	109	0	87	33	54	62.07
Non-user	IOB_24	chr15	93470538	C	T	CHD2	MISSENSE	ENSG00000173575:[ENST00000394196(c.359C>T,tCg>tTg):ENSP00000377747(p.Ser120Leu)]	NA	NA	NA	NA	NA	NA	NA	NA	NA	NA	MODIFIER	NA	NA	287	283	0	264	135	127	48.11
Non-user	IOB_24	chr15	99434744	G	A	IGF1R	SILENT	ENSG00000140443:[ENST00000268035(c.831G>A,gtG>gtA):ENSP00000268035(p.Val277(=))]	NA	NA	NA	4.96E-05	3.64E-04	NA	NA	NA	NA	NA	MODIFIER	NA	NA	347	346	0	268	136	132	49.25
Non-user	IOB_24	chr15	99434744	G	A	RP11-654A16.1	NCT	ENSG00000259621:[ENST00000558736(n.69+1952C>T)]	NA	NA	NA	4.96E-05	3.64E-04	NA	NA	NA	NA	NA	MODIFIER	NA	NA	347	346	0	268	136	132	49.25
Non-user	IOB_24	chr15	99670660	T	A	RP11-6O2.4	NCT	ENSG00000261054:[ENST00000566974(n.1005-66A>T)]	NA	NA	NA	NA	NA	NA	NA	NA	NA	NA	MODIFIER	NA	NA	247	247	0	205	110	94	45.85
Non-user	IOB_24	chr15	99670660	T	A	SYNM	MISSENSE	ENSG00000182253:[ENST00000328642(c.2092T>A,Tcc>Acc):ENSP00000330469(p.Ser698Thr)]	NA	NA	NA	NA	NA	NA	NA	NA	NA	NA	MODIFIER	NA	NA	247	247	0	205	110	94	45.85
Non-user	IOB_24	chr15	100332799	A	C	CTD-2054N24.1	EXONIC-NC	ENSG00000259655:[ENST00000557959(n.572A>C)]	NA	NA	NA	5.13E-01	5.04E-01	NA	NA	NA	NA	NA	MODIFIER	NA	NA	135	133	2	98	81	17	17.35
Non-user	IOB_24	chr15	100332799	A	C	DNM1P46	EXONIC-NC	ENSG00000182397:[ENST00000559110(n.1323T>G)]	NA	NA	NA	5.13E-01	5.04E-01	NA	NA	NA	NA	NA	MODIFIER	NA	NA	135	133	2	98	81	17	17.35
Non-user	IOB_24	chr15	100332807	C	T	CTD-2054N24.1	EXONIC-NC	ENSG00000259655:[ENST00000557959(n.580C>T)]	NA	NA	chr15*100332807*100332807*C*G,T*rs11633999*NA*NA*NA	5.11E-01	5.03E-01	NA	NA	NA	NA	NA	MODIFIER	NA	NA	131	130	0	102	85	17	16.67
Non-user	IOB_24	chr15	100332807	C	T	DNM1P46	EXONIC-NC	ENSG00000182397:[ENST00000559110(n.1315G>A)]	NA	NA	chr15*100332807*100332807*C*G,T*rs11633999*NA*NA*NA	5.11E-01	5.03E-01	NA	NA	NA	NA	NA	MODIFIER	NA	NA	131	130	0	102	85	17	16.67
Non-user	IOB_24	chr15	101718239	C	G	CHSY1	MISSENSE	ENSG00000131873:[ENST00000254190(c.1763G>C,aGa>aCa):ENSP00000254190(p.Arg588Thr)]	NA	chr15*101718235*101718239*COSM146267*ATCTC*A*2*haematopoietic_and_lymphoid_tissue*2	4.77E-02	6.37E-02	1.55E-01	4.77E-02	1.52E-01	NA	NA	NA	MODIFIER	NA	NA	158	158	0	110	65	45	40.91
Non-user	IOB_24	chr15	101922312	G	A	PCSK6	MISSENSE	ENSG00000140479:[ENST00000344273(c.1514C>T,tCg>tTg):ENSP00000344410(p.Ser505Leu)]	NA	NA	3.20E-03	2.19E-03	9.25E-03	3.19E-03	1.53E-02	NA	NA	NA	LOW	NA	0.003	89	89	0	68	24	44	64.71
Non-user	IOB_24	chr15	102312008	C	T	CTD-2611K5.5	EXONIC-NC	ENSG00000259236:[ENST00000559009(n.1751G>A)]	NA	NA	NA	NA	NA	NA	NA	NA	NA	NA	MODIFIER	NA	NA	64	64	0	73	53	20	27.4
Non-user	IOB_24	chr15	102312008	C	T	DNM1P47	EXONIC-NC	ENSG00000259660:[ENST00000561463(n.13376C>T)]	NA	NA	NA	NA	NA	NA	NA	NA	NA	NA	MODIFIER	NA	NA	64	64	0	73	53	20	27.4
Non-user	IOB_24	chr15	102312008	C	T	LOC101927594	3UTR	101927594:[XM_005255005.1(c.*386C>T)]	NA	NA	NA	NA	NA	NA	NA	NA	NA	NA	MODIFIER	NA	NA	64	64	0	73	53	20	27.4
Non-user	IOB_24	chr15	102358451	G	A	OR4F15	MISSENSE	ENSG00000182854:[ENST00000332238(c.62G>A,cGg>cAg):ENSP00000333184(p.Arg21Gln)]	NA	NA	2.20E-03	3.36E-03	1.10E-03	2.20E-03	2.00E-03	D_lc	BN	NA	MODERATE	N	0	345	343	0	259	125	133	51.35
Non-user	IOB_24	chr16	109183	C	A	RHBDF1	INTRONIC	64285:[XM_005255496.1(c.1872-58G>T)]	NA	NA	NA	NA	NA	NA	NA	NA	NA	NA	MODIFIER	NA	NA	125	125	0	75	42	32	42.67
Non-user	IOB_24	chr16	133280	G	C	MPG	INTRONIC	ENSG00000103152:[ENST00000219431(c.520+25G>C)]	NA	NA	5.39E-03	1.44E-02	1.60E-02	5.39E-03	5.10E-03	NA	NA	NA	MODIFIER	NA	NA	177	177	0	165	82	83	50.3
Non-user	IOB_24	chr16	318822	G	T	ARHGDIG	5UTR	ENSG00000242173:[ENST00000412541(c.-14G>T)]	NA	NA	2.28E-02	2.31E-02	2.00E-02	2.28E-02	1.02E-02	NA	NA	NA	LOW	NA	NA	234	234	0	139	62	76	54.68
Non-user	IOB_24	chr16	318822	G	T	FAM234A	EXONIC-NC	83986:[NR_104317.1(n.1946G>T)]	NA	NA	2.28E-02	2.31E-02	2.00E-02	2.28E-02	1.02E-02	NA	NA	NA	LOW	NA	NA	234	234	0	139	62	76	54.68
Non-user	IOB_24	chr16	318822	G	T	ITFG3	MISSENSE	ENSG00000167930:[ENST00000442458(c.1495G>T,Gcg>Tcg):ENSP00000397477(p.Ala499Ser)]	NA	NA	2.28E-02	2.31E-02	2.00E-02	2.28E-02	1.02E-02	NA	NA	NA	LOW	NA	NA	234	234	0	139	62	76	54.68
Non-user	IOB_24	chr16	318822	G	T	RGS11	EXONIC-NC	ENSG00000076344:[ENST00000477143(n.3790C>A)]	NA	NA	2.28E-02	2.31E-02	2.00E-02	2.28E-02	1.02E-02	NA	NA	NA	LOW	NA	NA	234	234	0	139	62	76	54.68
Non-user	IOB_24	chr16	335381	G	A	PDIA2	MISSENSE	ENSG00000185615:[ENST00000404312(c.856G>A,Gcg>Acg):ENSP00000384410(p.Ala286Thr)]	NA	NA	NA	1.70E-05	1.22E-04	NA	NA	NA	NA	NA	MODIFIER	NA	NA	151	148	0	122	55	64	52.46
Non-user	IOB_24	chr16	427699	C	G	TMEM8A	MISSENSE	ENSG00000129925:[ENST00000431232(c.271G>C,Gct>Cct):ENSP00000401338(p.Ala91Pro)]	NA	NA	3.79E-03	1.54E-02	1.41E-02	3.79E-03	7.20E-03	NA	NA	NA	MODIFIER	NA	NA	109	109	0	78	33	44	56.41
Non-user	IOB_24	chr16	601651	G	A	CAPN15	MISSENSE	ENSG00000103326:[ENST00000219611(c.2332G>A,Ggc>Agc):ENSP00000219611(p.Gly778Ser)]	NA	NA	9.98E-04	5.01E-04	2.95E-03	9.98E-04	5.10E-03	NA	NA	NA	MODERATE	NA	0.987	191	189	0	188	91	97	51.6
Non-user	IOB_24	chr16	601651	G	A	LA16c-366D1.3	NCT	ENSG00000261691:[ENST00000565879(n.287+1910C>T)]	NA	NA	9.98E-04	5.01E-04	2.95E-03	9.98E-04	5.10E-03	NA	NA	NA	MODERATE	NA	0.987	191	189	0	188	91	97	51.6
Non-user	IOB_24	chr16	716651	G	A	WDR90	INTRONIC	ENSG00000161996:[ENST00000547944(c.682-22G>A)]	NA	NA	5.99E-04	1.18E-04	8.03E-04	5.99E-04	2.00E-03	NA	NA	NA	MODIFIER	NA	NA	367	365	0	276	148	126	45.65
Non-user	IOB_24	chr16	718296	G	A	RHOT2	EXONIC-NC	ENSG00000140983:[ENST00000569706(n.165G>A)]	NA	NA	5.19E-03	NA	NA	5.19E-03	4.10E-03	NA	NA	NA	MODIFIER	NA	NA	43	41	0	37	14	23	62.16
Non-user	IOB_24	chr16	782535	T	A	HAGHL	NCT	ENSG00000103253:[ENST00000563156(n.194-934T>A)]	NA	NA	4.99E-03	1.03E-02	1.70E-02	4.99E-03	2.56E-02	NA	NA	NA	MODIFIER	NA	NA	191	190	1	209	113	96	45.93
Non-user	IOB_24	chr16	782535	T	A	NARFL	EXONIC-NC	ENSG00000103245:[ENST00000563051(n.366A>T)]	NA	NA	4.99E-03	1.03E-02	1.70E-02	4.99E-03	2.56E-02	NA	NA	NA	MODIFIER	NA	NA	191	190	1	209	113	96	45.93
Non-user	IOB_24	chr16	814091	G	A	MSLN	MISSENSE	ENSG00000102854:[ENST00000382862(c.248G>A,cGg>cAg):ENSP00000372313(p.Arg83Gln)]	NA	NA	7.99E-04	1.46E-04	2.47E-04	7.99E-04	0.00E+00	NA	NA	NA	MODERATE	NA	0	154	152	0	104	58	46	44.23
Non-user	IOB_24	chr16	1004500	T	C	LMF1	SILENT	ENSG00000103227:[ENST00000262301(c.360A>G,tcA>tcG):ENSP00000262301(p.Ser120(=))]	NA	NA	NA	4.40E-05	0.00E+00	NA	NA	NA	NA	NA	MODIFIER	NA	NA	221	219	0	159	87	72	45.28
Non-user	IOB_24	chr16	1400242	G	A	TSR3	INTRONIC-SS-ACR-PRX	ENSG00000007520:[ENST00000566296(c.290-7C>T)]	NA	NA	NA	1.79E-05	1.40E-04	NA	NA	NA	NA	NA	LOW	NA	NA	59	58	0	33	12	21	63.64
Non-user	IOB_24	chr16	1784916	C	T	MAPK8IP3	INTRONIC	ENSG00000138834:[ENST00000250894(c.602+5337C>T)]	NA	NA	7.39E-03	NA	NA	7.39E-03	2.00E-03	NA	NA	NA	MODIFIER	NA	NA	155	153	0	95	59	36	37.89
Non-user	IOB_24	chr16	2028156	G	A	TBL3	MISSENSE	ENSG00000183751:[ENST00000568546(c.2069G>A,cGg>cAg):ENSP00000454836(p.Arg690Gln)]	NA	NA	2.20E-03	3.82E-03	2.94E-03	2.20E-03	5.10E-03	T	BN	NA	MODERATE	NA	NA	223	223	0	177	79	97	54.8
Non-user	IOB_24	chr16	2159189	T	C	PKD1	SILENT	ENSG00000008710:[ENST00000262304(c.5979A>G,acA>acG):ENSP00000262304(p.Thr1993(=))]	NA	NA	NA	3.10E-04	2.08E-03	NA	NA	NA	NA	NA	MODIFIER	NA	NA	68	67	0	53	32	21	39.62
Non-user	IOB_24	chr16	2287026	C	T	DNASE1L2	INTRONIC	ENSG00000167968:[ENST00000320700(c.145-13C>T)]	NA	NA	3.69E-02	8.41E-02	5.50E-02	3.69E-02	4.19E-02	NA	NA	NA	MODIFIER	NA	NA	214	213	0	172	76	95	55.23
Non-user	IOB_24	chr16	2814640	A	G	SRRM2	MISSENSE	ENSG00000167978:[ENST00000301740(c.4111A>G,Atg>Gtg):ENSP00000301740(p.Met1371Val)]	NA	NA	8.99E-03	5.28E-03	3.77E-02	8.99E-03	4.60E-02	T_lc	BN	NA	MODERATE	N	0	160	160	0	166	84	82	49.4

Patel *et al.* , 2021. Whole-exome sequencing analysis of oral squamous cell carcinoma delineated by tobacco usage habits
Supplementary Table 4. List of somatic single nucleotide variants identified using whole exome sequencing of OSCC patients

Cohort	Sample	Chromosome	Genomic position	Reference allele	Alternate allele	Genesymbol	Variant type	Variant description	OncoMD	COSMIC	dbSNP	ExAC	ExAC_SAS	1000G	1000G_SAS	SIFT	Polyphen2	CONDEL	VEP	LRT	PhastCon7Way	Total reads in Normal	Reference read support in Normal	Alternative read support in Normal	Total reads in Tumor	Reference read support in Tumor	Alternate read support in Tumor	Alternate allele frequency in Tumor
Non-user	IOB_24	chr16	2871451	C	T	PRSS21	MISSENSE	ENSG00000007038:[ENST00000455114(c.784C>T,Cgg>Tgg):ENSP00000400632(p.Arg262Trp)]	NA	chr16*2871451*2871451*COSM969506*C*T*1*endometrium*1	2.00E-03	1.25E-03	9.09E-03	2.00E-03	1.02E-02	NA	NA	NA	MODIFIER	NA	NA	135	134	0	128	70	57	44.53
Non-user	IOB_24	chr16	3445163	T	C	NAA60	INTRONIC	ENSG00000262621:[ENST00000575785(c.-12-9263T>C)]	NA	NA	5.19E-03	NA	NA	5.19E-03	1.12E-02	NA	NA	NA	MODIFIER	NA	NA	104	104	0	84	59	25	29.76
Non-user	IOB_24	chr16	3445163	T	C	ZSCAN32	INTRONIC	54925:[XM_005255404.1(c.26+2029A>G)]	NA	NA	5.19E-03	NA	NA	5.19E-03	1.12E-02	NA	NA	NA	MODIFIER	NA	NA	104	104	0	84	59	25	29.76
Non-user	IOB_24	chr16	3446340	T	A	NAA60	INTRONIC	ENSG00000262621:[ENST00000575785(c.-12-8086T>A)]	NA	NA	3.06E-02	NA	NA	3.06E-02	3.89E-02	NA	NA	NA	MODIFIER	NA	NA	265	264	0	221	103	116	52.49
Non-user	IOB_24	chr16	3446340	T	A	ZSCAN32	INTRONIC	ENSG00000140987:[ENST00000422427(c.-225-2526A>T)]	NA	NA	3.06E-02	NA	NA	3.06E-02	3.89E-02	NA	NA	NA	MODIFIER	NA	NA	265	264	0	221	103	116	52.49
Non-user	IOB_24	chr16	3487201	G	A	NAA60	INTRONIC	ENSG00000262621:[ENST00000575785(c.212-11270G>A)]	NA	NA	3.08E-02	4.63E-02	3.40E-02	3.08E-02	3.37E-02	NA	NA	NA	LOW	NA	0	280	280	0	193	95	97	50.26
Non-user	IOB_24	chr16	3487201	G	A	ZNF597	SILENT	ENSG00000167981:[ENST00000301744(c.498C>T,agC>agT):ENSP00000301744(p.Ser166(=))]	NA	NA	3.08E-02	4.63E-02	3.40E-02	3.08E-02	3.37E-02	NA	NA	NA	LOW	NA	0	280	280	0	193	95	97	50.26
Non-user	IOB_24	chr16	3490879	T	A	NAA60	INTRONIC	ENSG00000262621:[ENST00000575785(c.212-7592T>A)]	NA	NA	3.08E-02	4.66E-02	3.43E-02	3.08E-02	3.68E-02	T	BN	NA	MODERATE	N	0	72	72	0	32	15	16	50
Non-user	IOB_24	chr16	3490879	T	A	ZNF597	MISSENSE	ENSG00000167981:[ENST00000301744(c.88A>T,Act>Tct):ENSP00000301744(p.Thr30Ser)]	NA	NA	3.08E-02	4.66E-02	3.43E-02	3.08E-02	3.68E-02	T	BN	NA	MODERATE	N	0	72	72	0	32	15	16	50
Non-user	IOB_24	chr16	3493878	G	C	NAA60	INTRONIC	ENSG00000122390:[ENST00000573580(c.-86+51G>C)]	NA	NA	3.08E-02	NA	NA	3.08E-02	3.68E-02	NA	NA	NA	MODIFIER	NA	NA	44	44	0	54	29	24	44.44
Non-user	IOB_24	chr16	3498499	G	A	NAA60	SILENT	ENSG00000262621:[ENST00000575785(c.240G>A,agG>agA):ENSP00000477472(p.Arg80(=))]	NA	NA	3.08E-02	4.11E-02	3.07E-02	3.08E-02	3.68E-02	NA	NA	NA	MODIFIER	NA	NA	53	53	0	51	30	20	39.22
Non-user	IOB_24	chr16	3534831	C	G	LA16c-306E5.3	NCT	ENSG00000263212:[ENST00000574423(n.112-21500C>G)]	NA	NA	5.19E-03	1.40E-02	1.33E-02	5.19E-03	1.12E-02	D_lc	BN	NA	MODERATE	NA	NA	352	351	0	225	108	116	51.56
Non-user	IOB_24	chr16	3534831	C	G	NAA60	MISSENSE	ENSG00000122390:[ENST00000570819(c.407C>G,gCa>gGa):ENSP00000460763(p.Ala136Gly)]	NA	NA	5.19E-03	1.40E-02	1.33E-02	5.19E-03	1.12E-02	D_lc	BN	NA	MODERATE	NA	NA	352	351	0	225	108	116	51.56
Non-user	IOB_24	chr16	3544512	G	C	C16orf90	INTRONIC	ENSG00000215131:[ENST00000399645(c.425+12C>G)]	NA	NA	3.59E-02	2.02E-02	4.68E-02	3.59E-02	3.37E-02	NA	NA	NA	MODIFIER	NA	NA	145	144	0	142	75	67	47.18
Non-user	IOB_24	chr16	3544512	G	C	LA16c-306E5.3	NCT	ENSG00000263212:[ENST00000574423(n.112-11819G>C)]	NA	NA	3.59E-02	2.02E-02	4.68E-02	3.59E-02	3.37E-02	NA	NA	NA	MODIFIER	NA	NA	145	144	0	142	75	67	47.18
Non-user	IOB_24	chr16	3544586	C	T	C16orf90	MISSENSE	ENSG00000215131:[ENST00000437192(c.338G>A,cGt>cAt):ENSP00000401335(p.Arg113His)]	NA	NA	3.61E-02	1.88E-02	4.27E-02	3.61E-02	3.37E-02	D_lc	BN	NA	MODERATE	NA	NA	177	176	0	152	83	68	44.74
Non-user	IOB_24	chr16	3544586	C	T	LA16c-306E5.3	NCT	ENSG00000263212:[ENST00000574423(n.112-11745C>T)]	NA	NA	3.61E-02	1.88E-02	4.27E-02	3.61E-02	3.37E-02	D_lc	BN	NA	MODERATE	NA	NA	177	176	0	152	83	68	44.74
Non-user	IOB_24	chr16	3598725	C	A	NA	NCT	101929732:[XR_243342.1(n.501+2727C>A)]	NA	NA	NA	1.71E-05	1.09E-04	NA	NA	NA	NA	NA	MODIFIER	NA	NA	114	114	0	97	52	43	44.33
Non-user	IOB_24	chr16	3598725	C	A	NLRC3	MISSENSE	ENSG00000167984:[ENST00000448023(c.2770G>T,Ggt>Tgt):ENSP00000414415(p.Gly924Cys)]	NA	NA	NA	1.71E-05	1.09E-04	NA	NA	NA	NA	NA	MODIFIER	NA	NA	114	114	0	97	52	43	44.33
Non-user	IOB_24	chr16	3604305	C	T	NA	NCT	101929732:[XR_243342.1(n.502-3095C>T)]	NA	NA	2.20E-03	2.04E-03	8.83E-03	2.20E-03	8.20E-03	NA	NA	NA	MODIFIER	NA	NA	94	94	0	81	47	34	41.98
Non-user	IOB_24	chr16	3604305	C	T	NLRC3	SILENT	ENSG00000167984:[ENST00000301749(c.2205G>A,agG>agA):ENSP00000301749(p.Arg735(=))]	NA	NA	2.20E-03	2.04E-03	8.83E-03	2.20E-03	8.20E-03	NA	NA	NA	MODIFIER	NA	NA	94	94	0	81	47	34	41.98
Non-user	IOB_24	chr16	3706688	G	A	DNASE1	MISSENSE	ENSG00000213918:[ENST00000572237(c.72G>A,Gag>Aag):ENSP00000461527(p.Glu25Lys)]	NA	NA	NA	5.77E-05	7.12E-05	NA	NA	NA	NA	NA	MODIFIER	NA	NA	141	140	0	121	49	71	58.68
Non-user	IOB_24	chr16	3706688	G	A	TRAP1	INTRONIC	ENSG00000126602:[ENST00000575707(c.*76+1366C>T)]	NA	NA	NA	5.77E-05	7.12E-05	NA	NA	NA	NA	NA	MODIFIER	NA	NA	141	140	0	121	49	71	58.68
Non-user	IOB_24	chr16	3807282	C	G	CREBBP	INTRONIC	1387:[XM_005255124.1(c.3653+7G>C)]	NA	NA	.	5.61E-05	4.03E-04	NA	NA	NA	NA	NA	LOW	NA	NA	88	87	1	60	33	26	43.33
Non-user	IOB_24	chr16	4257549	C	T	SRL	SILENT	ENSG00000185739:[ENST00000572111(c.618G>A,aaG>aaA):ENSP00000461179(p.Lys206(=))]	NA	NA	2.32E-02	3.53E-03	5.95E-04	2.32E-02	1.00E-03	NA	NA	NA	LOW	NA	NA	176	175	0	160	81	79	49.38
Non-user	IOB_24	chr16	4431373	G	C	CORO7	NCT	ENSG00000262246:[ENST00000575714(n.314+6614C>G)]	NA	chr16*4431372*4431373*COSM1683212*CG*C*1*large_intestine*1	1.32E-02	4.07E-02	2.28E-02	1.32E-02	1.53E-02	NA	NA	NA	MODIFIER	NA	NA	311	311	0	222	97	125	56.31
Non-user	IOB_24	chr16	4431373	G	C	CORO7-PAM16	INTRONIC	ENSG00000103426:[ENST00000572467(c.785+6614C>G)]	NA	chr16*4431372*4431373*COSM1683212*CG*C*1*large_intestine*1	1.32E-02	4.07E-02	2.28E-02	1.32E-02	1.53E-02	NA	NA	NA	MODIFIER	NA	NA	311	311	0	222	97	125	56.31
Non-user	IOB_24	chr16	4431373	G	C	VASN	SILENT	ENSG00000168140:[ENST00000304735(c.495G>C,ccG>ccC):ENSP00000306864(p.Pro165(=))]	NA	chr16*4431372*4431373*COSM1683212*CG*C*1*large_intestine*1	1.32E-02	4.07E-02	2.28E-02	1.32E-02	1.53E-02	NA	NA	NA	MODIFIER	NA	NA	311	311	0	222	97	125	56.31
Non-user	IOB_24	chr16	4557696	C	T	HMOX2	INTRONIC	3163:[NM_001286267.1(c.367-18C>T)]	NA	NA	3.99E-04	1.62E-04	1.39E-03	3.99E-04	2.00E-03	NA	NA	NA	MODIFIER	NA	NA	200	199	0	181	106	74	40.88
Non-user	IOB_24	chr16	4733311	C	T	MGRN1	NCT	23295:[XR_243269.1(n.1492+41C>T)]	NA	NA	3.20E-03	6.79E-03	1.71E-03	3.19E-03	2.00E-03	NA	NA	NA	MODIFIER	NA	NA	151	151	0	118	66	51	43.22
Non-user	IOB_24	chr16	4764117	A	C	ANKS3	MISSENSE	ENSG00000168096:[ENST00000450067(c.26T>G,aTg>aGg):ENSP00000388270(p.Met9Arg)]	NA	NA	1.46E-02	3.16E-02	2.47E-02	1.46E-02	1.94E-02	T	BN	NA	MODERATE	NA	NA	189	188	0	132	68	64	48.48
Non-user	IOB_24	chr16	4802576	C	T	ZNF500	MISSENSE	ENSG00000103199:[ENST00000588942(c.56G>A,Gga>Aga):ENSP00000465073(p.Gly20Arg)]	NA	NA	NA	NA	NA	NA	NA	D	PrD	NA	MODERATE	N	0	216	215	0	107	64	42	39.25
Non-user	IOB_24	chr16	10788550	C	T	TEKT5	MISSENSE	ENSG00000153060:[ENST00000283025(c.181G>A,Gtc>Atc):ENSP00000283025(p.Val61Ile)]	NA	NA	1.60E-03	7.12E-03	1.98E-03	1.60E-03	0.00E+00	T	BN	NA	MODERATE	N	0	145	142	0	109	60	48	44.04
Non-user	IOB_24	chr16	12297186	C	T	SNX29	INTRONIC	ENSG00000048471:[ENST00000564791(c.145+3646C>T)]	NA	NA	chr16*12297186*12297186*C*G,T*rs7187644*NA*NA*NA	NA	NA	NA	NA	NA	NA	NA	MODIFIER	NA	NA	608	582	10	621	408	183	29.47
Non-user	IOB_24	chr16	12297195	G	A	SNX29	INTRONIC	ENSG00000048471:[ENST00000566228(c.1678+3655G>A)]	NA	NA	NA	NA	NA	NA	NA	NA	NA	NA	MODIFIER	NA	NA	609	596	2	643	453	162	25.19
Non-user	IOB_24	chr16	12297201	C	G	SNX29	INTRONIC	ENSG00000048471:[ENST00000306030(c.523+3661C>G)]	NA	NA	NA	NA	NA	NA	NA	NA	NA	NA	MODIFIER	NA	NA	609	590	3	673	429	215	31.95
Non-user	IOB_24	chr16	12297234	A	G	SNX29	INTRONIC	ENSG00000048471:[ENST00000566228(c.1678+3694A>G)]	NA	NA	NA	NA	NA	NA	NA	NA	NA	NA	MODIFIER	NA	NA	619	560	24	768	395	338	44.01
Non-user	IOB_24	chr16	12297247	A	G	SNX29	INTRONIC	ENSG00000048471:[ENST00000564791(c.145+3707A>G)]	NA	NA	NA	NA	NA	NA	NA	NA	NA	NA	MODIFIER	NA	NA	619	565	29	795	389	372	46.79
Non-user	IOB_24	chr16	13297348	C	T	AC009134.1	INTRONIC-SS-ACR	ENSG00000262116:[ENST00000571939(n.104-1G>A)]	NA	NA	1.60E-02	3.89E-02	5.01E-02	1.60E-02	3.37E-02	NA	NA	NA	LOW	NA	NA	145	143	0	106	69	37	34.91
Non-user	IOB_24	chr16	13297348	C	T	SHISA9	SILENT	ENSG00000237515:[ENST00000424107(c.789C>T,ccC>ccT):ENSP00000407958(p.Pro263(=))]	NA	NA	1.60E-02	3.89E-02	5.01E-02	1.60E-02	3.37E-02	NA	NA	NA	LOW	NA	NA	145	143	0	106	69	37	34.91
Non-user	IOB_24	chr16	14042077	A	G	ERCC4	MISSENSE	ENSG00000175595:[ENST00000311895(c.2624A>G,gAa>gGa):ENSP00000310520(p.Glu875Gly)]	NA	NA	5.79E-03	1.31E-02	2.60E-03	5.79E-03	5.10E-03	NA	NA	NA	MODIFIER	NA	NA	140	140	0	108	51	57	52.78
Non-user	IOB_24	chr16	14721044	C	T	PARN	SILENT-SS-PRX	ENSG00000140694:[ENST00000341484(c.63G>A,aaG>aaA):ENSP00000345456(p.Lys21(=))]	NA	NA	NA	NA	NA	NA	NA	NA	NA	NA	MODIFIER	NA	NA	223	223	0	181	91	87	48.07
Non-user	IOB_24	chr16	15082865	C	T	PDXDC1	INTRONIC	ENSG00000179889:[ENST00000325823(c.-24-8745C>T)]	NA	NA	NA	NA	NA	NA	NA	NA	NA	NA	MODIFIER	NA	NA	139	138	0	196	149	47	23.98
Non-user	IOB_24	chr16	15082865	C	T	RP11-680G24.4	EXONIC-NC	ENSG00000261819:[ENST00000567634(n.1153G>A)]	NA	NA	NA	NA	NA	NA	NA	NA	NA	NA	MODIFIER	NA	NA	139	138	0	196	149	47	23.98
Non-user	IOB_24	chr16	15098047	C	G	PDXDC1	MISSENSE	ENSG00000179889:[ENST00000450288(c.162C>G,atC>atG):ENSP00000391147(p.Ile54Met)]	NA	NA	NA	3.04E-01	3.47E-01	NA	NA	T	BN	NA	MODERATE	N	1	72	72	0	156	108	46	29.49
Non-user	IOB_24	chr16	15116521	C	T	PDXDC1	INTRONIC	23042:[XM_005255178.1(c.1033-40C>T)]	NA	NA	NA	5.24E-01	5.33E-01	NA	NA	NA	NA	NA	MODIFIER	NA	NA	158	157	1	208	65	140	67.31
Non-user	IOB_24	chr16	15116625	G	T	PDXDC1	INTRONIC	ENSG00000179889:[ENST00000455313(c.1071+14G>T)]	NA	NA	NA	5.30E-01	5.38E-01	NA	NA	NA	NA	NA	MODIFIER	NA	NA	83	79	1	95	36	57	60
Non-user	IOB_24	chr16	15126890	C	T	PDXDC1	INTRONIC	23042:[NM_001324021.1(c.1318+2977C>T)]	NA	NA	4.13E-02	NA	NA	4.13E-02	1.53E-02	NA	NA	NA	MODIFIER	NA	NA	180	179	0	129	67	62	48.06
Non-user	IOB_24	chr16	15178499	G	A	PDXDC1	INTRONIC	ENSG00000179889:[ENST00000535621(c.1400-54237G>A)]	NA	NA	NA	3.04E-01	3.70E-01	NA	NA	NA	NA	NA	MODIFIER	NA	NA	195	194	0	254	169	84	33.07
Non-user	IOB_24	chr16	15178499	G	A	RRN3	MISSENSE-SS-PRX	ENSG00000085721:[ENST00000540462(c.149C>T,tCg>tTg):ENSP00000437963(p.Ser50Leu)]	NA	NA	NA	3.04E-01	3.70E-01	NA	NA	NA	NA	NA	MODIFIER	NA	NA	195	194	0	254	169	84	33.07
Non-user	IOB_24	chr16	15178612	C	T	PDXDC1	INTRONIC	23042:[NM_001324021.1(c.1319-54124C>T)]	NA	NA	chr16*15178612*15178612*C*A,T*rs373009083*NA*NA*NA	2.36E-01	3.06E-01	NA	NA	NA	NA	NA	MODIFIER	NA	NA	185	185	0	163	135	27	16.56
Non-user	IOB_24	chr16	15178612	C	T	RRN3	INTRONIC	ENSG00000085721:[ENST00000540462(c.86-50G>A)]	NA	NA	chr16*15178612*15178612*C*A,T*rs373009083*NA*NA*NA	2.36E-01	3.06E-01	NA	NA	NA	NA	NA	MODIFIER	NA	NA	185	185	0	163	135	27	16.56
Non-user	IOB_24	chr16	15711335	G	A	C16orf45	INTRONIC	ENSG00000166780:[ENST00000565857(c.*13-7412G>A)]	NA	NA	3.53E-02	4.96E-02	4.67E-02	3.53E-02	2.97E-02	NA	NA	NA	MODIFIER	NA	NA	34	34	0	21	11	10	47.62
Non-user	IOB_24	chr16	15711335	G	A	KIAA0430	SILENT	ENSG00000166783:[ENST00000344181(c.1704C>T,atC>atT):ENSP00000341939(p.Ile568(=))]	NA	NA	3.53E-02	4.96E-02	4.67E-02	3.53E-02	2.97E-02	NA	NA	NA	MODIFIER	NA	NA	34	34	0	21	11	10	47.62
Non-user	IOB_24	chr16	15737177	G	A	MIR484	MATURE-MIRNA	ENSG00000272213:[ENST00000606601(n.27G>A)]	NA	NA	2.00E-04	5.43E-03	5.50E-03	2.00E-04	0.00E+00	NA	NA	NA	MODIFIER	NA	NA	87	84	0	52	26	26	50
Non-user	IOB_24	chr16	15737177	G	A	NDE1	5UTR	ENSG00000072864:[ENST00000396353(c.-773G>A)]	NA	NA	2.00E-04	5.43E-03	5.50E-03	2.00E-04	0.00E+00	NA	NA	NA	MODIFIER	NA	NA	87	84	0	52	26	26	50
Non-user	IOB_24	chr16	15818842	A	G	MYH11	MISSENSE	ENSG00000133392:[ENST00000396324(c.3887T>C,gTt>gCt):ENSP00000379616(p.Val1296Ala)]	NA	NA	2.02E-02	3.98E-02	2.50E-02	2.02E-02	1.94E-02	T	BN	NA	MODERATE	N	1	140	139	1	111	51	59	53.15
Non-user	IOB_24	chr16	15818842	A	G	NDE1	EXONIC-NC	ENSG00000072864:[ENST00000571896(n.3349A>G)]	NA	NA	2.02E-02	3.98E-02	2.50E-02	2.02E-02	1.94E-02	T	BN	NA	MODERATE	N	1	140	139	1	111	51	59	53.15
Non-user	IOB_24	chr16	18844369	G	A	SMG1	SILENT	ENSG00000157106:[ENST00000389467(c.8685C>T,acC>acT):ENSP00000374118(p.Thr2895(=))]	NA	NA	3.99E-04	1.82E-04	8.48E-04	3.99E-04	2.00E-03	NA	NA	NA	LOW	NA	NA	172	171	0	131	75	56	42.75
Non-user	IOB_24	chr16	19126470	C	T	CTD-2349B8.1	INTRONIC	ENSG00000261427:[ENST00000568526(c.211+363C>T)]	NA	NA	1.10E-02	2.27E-02	2.57E-02	1.10E-02	1.84E-02	NA	NA	NA	MODIFIER	NA	NA	225	224	0	196	97	99	50.51
Non-user	IOB_24	chr16	19126470	C	T	ITPRIPL2	SILENT	ENSG00000205730:[ENST00000381440(c.687C>T,tgC>tgT):ENSP00000370849(p.Cys229(=))]	NA	NA	1.10E-02	2.27E-02	2.57E-02	1.10E-02	1.84E-02	NA	NA	NA	MODIFIER	NA	NA	225	224	0	196	97	99	50.51
Non-user	IOB_24	chr16	19195253	C	G	SYT17	SILENT	ENSG00000103528:[ENST00000565183(c.28C>G,acC>acG):ENSP00000456879(p.Thr10(=))]	NA	chr16*19195253*19195255*COSM1708807*CGG*CAA*1*skin*1	chr16*19195253*19195253*C*T*rs371509222*NA*NA*NA|chr16*19195253*19195254*CG*C*rs747107874*NA*NA*NA	8.24E-06	6.06E-05	NA	NA	NA	NA	NA	LOW	NA	NA	317	317	0	248	155	91	36.69
Non-user	IOB_24	chr16	20974740	C	T	DNAH3	MISSENSE	ENSG00000158486:[ENST00000261383(c.10466G>A,cGg>cAg):ENSP00000261383(p.Arg3489Gln)]	NA	NA	7.99E-03	2.01E-02	3.45E-03	7.99E-03	3.10E-03	NA	NA	NA	MODERATE	NA	0.06	91	91	0	87	49	38	43.68
Non-user	IOB_24	chr16	20998720	G	A	DNAH3	SILENT	ENSG00000158486:[ENST00000261383(c.6933C>T,ttC>ttT):ENSP00000261383(p.Phe2311(=))]	NA	NA	8.79E-03	2.11E-02	5.15E-03	8.79E-03	5.10E-03	NA	NA	NA	LOW	NA	1	213	213	0	170	83	87	51.18
Non-user	IOB_24	chr16	21531111	T	C	CTD-2547E10.2	NCT	ENSG00000180747:[ENST00000522841(n.617+38A>G)]	NA	NA	NA	2.53E-01	2.57E-01	NA	NA	NA	NA	NA	MODIFIER	NA	NA	118	118	0	71	55	15	21.13
Non-user	IOB_24	chr16	21531111	T	C	SLC7A5P2	EXONIC-NC	387254:[NR_002594.1(n.655A>G)]	NA	NA	NA	2.53E-01	2.57E-01	NA	NA	NA	NA	NA	MODIFIER	NA	NA	118	118	0	71	55	15	21.13
Non-user	IOB_24	chr16	22120976	G	A	VWA3A	INTRONIC	ENSG00000175267:[ENST00000389398(c.582+75G>A)]	NA	NA	2.20E-03	NA	NA	2.20E-03	2.00E-03	NA	NA	NA	MODIFIER	NA	NA	143	143	0	93	56	37	39.78
Non-user	IOB_24	chr16	22260058	G	A	EEF2K	INTRONIC	ENSG00000103319:[ENST00000263026(c.348-18G>A)]	NA	NA	1.62E-02	3.47E-02	8.91E-03	1.62E-02	6.10E-03	NA	NA	NA	MODIFIER	NA	NA	93	93	0	81	41	40	49.38
Non-user	IOB_24	chr16	22580611	T	G	RP11-368J21.3	NCT	ENSG00000257838:[ENST00000550753(n.2211+28T>G)]	NA	NA	NA	2.40E-01	2.94E-01	NA	NA	NA	NA	NA	MODIFIER	NA	NA	64	63	1	51	39	11	21.57
Non-user	IOB_24	chr16	23079501	G	A	USP31	MISSENSE	ENSG00000103404:[ENST00000567975(c.1804C>T,Cgc>Tgc):ENSP00000461621(p.Arg602Cys)]	NA	NA	3.20E-02	6.21E-02	6.81E-02	3.19E-02	7.06E-02	NA	NA	NA	MODERATE	NA	1	162	162	0	104	63	41	39.42
Non-user	IOB_24	chr16	23093708	T	C	USP31	NCT	ENSG00000103404:[ENST00000563525(n.367+51A>G)]	NA	NA	chr16*23093708*23093708*T*A*rs753165971*NA*NA*NA	NA	NA	NA	NA	NA	NA	NA	MODIFIER	NA	NA	93	93	0	91	43	48	52.75
Non-user	IOB_24	chr16	29908310	G	A	SEZ6L2	MISSENSE	ENSG00000174938:[ENST00000308713(c.344C>T,gCa>gTa):ENSP00000312550(p.Ala115Val)]	NA	chr16*29908310*29908311*COSM1377279*GC*G*1*large_intestine*1	NA	1.86E-04	8.80E-04	NA	NA	D_lc	BN	NA	MODERATE	NA	NA	29	29	0	29	14	15	51.72
Non-user	IOB_24	chr16	29979525	G	A	TMEM219	MISSENSE	ENSG00000149932:[ENST00000279396(c.535G>A,Gtt>Att):ENSP00000279396(p.Val179Ile)]	NA	NA	7.99E-04	5.80E-04	4.24E-03	7.99E-04	4.10E-03	T_lc	BN	NA	MODERATE	NA	NA	73	73	0	70	32	38	54.29
Non-user	IOB_24	chr16	30106103	G	A	YPEL3	MISSENSE	ENSG00000090238:[ENST00000562641(c.391C>T,Cac>Tac):ENSP00000456825(p.His131Tyr)]	NA	NA	NA	1.66E-05	1.21E-04	NA	NA	NA	NA	NA	LOW	NA	NA	161	161	0	103	52	51	49.51
Non-user	IOB_24	chr16	30208618	C	T	SLX1A	SILENT	ENSG00000132207:[ENST00000345535(c.396C>T,gaC>gaT):ENSP00000333945(p.Asp132(=))]	NA	NA	NA	4.25E-03	1.33E-02	NA	NA	NA	NA	NA	MODIFIER	NA	NA	60	60	0	51	43	8	15.69
Non-user	IOB_24	chr16	30208618	C	T	SLX1A-SULT1A3	EXONIC-NC	ENSG00000213599:[ENST00000569959(n.681C>T)]	NA	NA	NA	4.25E-03	1.33E-02	NA	NA	NA	NA	NA	MODIFIER	NA	NA	60	60	0	51	43	8	15.69
Non-user	IOB_24	chr16	30208618	C	T	SULT1A3	5UTR	ENSG00000261052:[ENST00000354723(c.-65C>T)]	NA	NA	NA	4.25E-03	1.33E-02	NA	NA	NA	NA	NA	MODIFIER	NA	NA	60	60	0	51	43	8	15.69
Non-user	IOB_24	chr16	30666803	C	T	PRR14	NCT	ENSG00000156858:[ENST00000571654(n.248-20C>T)]	NA	NA	1.18E-02	1.87E-02	3.85E-02	1.18E-02	4.29E-02	NA	NA	NA	MODIFIER	NA	NA	212	210	0	158	86	68	43.04
Non-user	IOB_24	chr16	30680766	C	T	FBRS	MISSENSE	ENSG00000156860:[ENST00000356166(c.2743C>T,Ctc>Ttc):ENSP00000348489(p.Leu915Phe)]	NA	NA	NA	NA	NA	NA	NA	D	PoD	NA	MODERATE	N	1	426	425	0	366	172	192	52.46
Non-user	IOB_24	chr16	30745976	C	T	SRCAP	INTRONIC	ENSG00000080603:[ENST00000380361(c.6198+40C>T)]	NA	NA	4.11E-02	9.73E-02	5.05E-02	4.11E-02	5.01E-02	NA	NA	NA	MODIFIER	NA	NA	69	68	0	45	22	22	48.89

Patel *et al.* , 2021. Whole-exome sequencing analysis of oral squamous cell carcinoma delineated by tobacco usage habits
Supplementary Table 4. List of somatic single nucleotide variants identified using whole exome sequencing of OSCC patients

Cohort	Sample	Chromosome	Genomic position	Reference allele	Alternate allele	Genesymbol	Variant type	Variant description	OncoMD	COSMIC	dbSNP	ExAC	ExAC_SAS	1000G	1000G_SAS	SIFT	Polyphen2	CONDEL	VEP	LRT	PhastCon7Way	Total reads in Normal	Reference read support in Normal	Alternative read support in Normal	Total reads in Tumor	Reference read support in Tumor	Alternate read support in Tumor	Alternate allele frequency in Tumor
Non-user	IOB_24	chr16	31373204	G	T	ITGAX	NONSENSE	ENSG00000140678:[ENST00000268296(c.1060G>T,Gag>Tag):ENSP00000268296(p.Glu354Ter)]	NA	NA	NA	NA	NA	NA	NA	NA	NA	NA	MODIFIER	NA	NA	233	233	0	186	180	5	2.69
Non-user	IOB_24	chr16	31471331	C	T	ARMC5	SILENT	ENSG00000140691:[ENST00000564514(c.17C>T,tcC>tcT):ENSP00000454430(p.Ser6(=))]	NA	NA	4.99E-03	4.83E-03	2.66E-02	4.99E-03	2.15E-02	NA	NA	NA	LOW	NA	NA	133	133	0	127	58	69	54.33
Non-user	IOB_24	chr16	31476186	C	G	ARMC5	SILENT	ENSG00000140691:[ENST00000408912(c.2127C>G,ctC>ctG):ENSP00000386125(p.Leu709(=))]	NA	NA	3.30E-02	7.15E-02	4.54E-02	3.29E-02	2.97E-02	NA	NA	NA	LOW	NA	NA	343	343	0	239	125	114	47.7
Non-user	IOB_24	chr16	32487846	G	A	ABCD1P3	NCT	ENSG00000261233:[ENST00000562927(n.161-725C>T)]	NA	NA	2.18E-02	NA	NA	2.18E-02	1.12E-01	NA	NA	NA	MODIFIER	NA	NA	22	22	0	27	18	9	33.33
Non-user	IOB_24	chr16	33784439	C	G	NA	NCT	653562:[XR_132610.2(n.1500-33C>G)]	NA	NA	1.12E-02	5.60E-03	3.78E-02	1.12E-02	5.73E-02	NA	NA	NA	MODIFIER	NA	NA	167	167	0	192	129	63	32.81
Non-user	IOB_24	chr16	33784439	C	G	RP11-598D12.4	NCT	ENSG00000198555:[ENST00000568600(n.1391-33C>G)]	NA	NA	1.12E-02	5.60E-03	3.78E-02	1.12E-02	5.73E-02	NA	NA	NA	MODIFIER	NA	NA	167	167	0	192	129	63	32.81
Non-user	IOB_24	chr16	34257067	C	T	CTD-2144E22.5	MISSENSE	ENSG00000179755:[ENST00000319817(c.311C>T,cCg>cTg):ENSP00000318279(p.Pro104Leu)]	NA	NA	3.40E-03	1.04E-02	1.32E-02	3.39E-03	1.53E-02	D_lc	PoD	NA	MODERATE	NA	NA	161	161	0	97	53	44	45.36
Non-user	IOB_24	chr16	34257067	C	T	NA	EXONIC-NC	162137:[XR_110135.4(n.690C>T)]	NA	NA	3.40E-03	1.04E-02	1.32E-02	3.39E-03	1.53E-02	D_lc	PoD	NA	MODERATE	NA	NA	161	161	0	97	53	44	45.36
Non-user	IOB_24	chr16	46934745	A	G	GPT2	INTRONIC	ENSG00000166123:[ENST00000340124(c.442+43A>G)]	NA	NA	2.00E-04	1.76E-04	1.25E-03	2.00E-04	1.00E-03	NA	NA	NA	MODIFIER	NA	NA	105	105	0	81	34	46	56.79
Non-user	IOB_24	chr16	48221137	T	C	ABCC11	MISSENSE	ENSG00000121270:[ENST00000353782(c.2908A>G,Atg>Gtg):ENSP00000311326(p.Met970Val)]	NA	NA	3.59E-03	7.43E-03	1.32E-02	3.59E-03	1.12E-02	T	BN	NA	MODERATE	N	0	191	186	0	118	56	61	51.69
Non-user	IOB_24	chr16	50332895	C	T	ADCY7	SILENT	ENSG00000121281:[ENST00000254235(c.1029C>T,caC>caT):ENSP00000254235(p.His343(=))]	NA	NA	3.20E-03	2.21E-03	5.84E-03	3.19E-03	2.00E-03	NA	NA	NA	MODIFIER	NA	NA	253	252	0	209	97	110	52.63
Non-user	IOB_24	chr16	53404580	C	T	LOC643802	INTRONIC	643802:[NM_001347693.1(c.270+52G>A)]	NA	NA	1.44E-02	NA	NA	1.44E-02	3.27E-02	NA	NA	NA	MODIFIER	NA	NA	58	58	0	40	16	24	60
Non-user	IOB_24	chr16	53404580	C	T	RP11-44F14.1	EXONIC-NC	ENSG00000260078:[ENST00000565598(n.728G>A)]	NA	NA	1.44E-02	NA	NA	1.44E-02	3.27E-02	NA	NA	NA	MODIFIER	NA	NA	58	58	0	40	16	24	60
Non-user	IOB_24	chr16	53499374	G	C	RBL2	MISSENSE	ENSG00000103479:[ENST00000544545(c.1075G>C,Gaa>Caa):ENSP00000444685(p.Glu359Gln)]	NA	NA	1.02E-02	1.63E-02	1.90E-02	1.02E-02	1.74E-02	NA	NA	NA	MODIFIER	NA	NA	126	126	0	95	57	38	40
Non-user	IOB_24	chr16	57077523	G	A	NLRC5	INTRONIC-SS-DNR-PRX	ENSG00000140853:[ENST00000540182(c.295+7G>A)]	NA	NA	4.47E-02	6.10E-02	4.80E-02	4.47E-02	5.42E-02	NA	NA	NA	LOW	NA	NA	199	197	0	159	88	71	44.65
Non-user	IOB_24	chr16	58768129	C	A	GOT2	MISSENSE	ENSG00000125166:[ENST00000245206(c.4G>T,Gcc>Tcc):ENSP00000245206(p.Ala2Ser)]	NA	chr16*58768129*58768129*COSM435472*C*A*1*breast*1	4.33E-02	1.22E-01	8.81E-02	4.33E-02	6.13E-02	NA	NA	NA	MODIFIER	NA	NA	23	23	0	20	6	14	70
Non-user	IOB_24	chr16	58768129	C	A	RP11-410D17.2	EXONIC-NC	ENSG00000245768:[ENST00000568134(n.144C>A)]	NA	chr16*58768129*58768129*COSM435472*C*A*1*breast*1	4.33E-02	1.22E-01	8.81E-02	4.33E-02	6.13E-02	NA	NA	NA	MODIFIER	NA	NA	23	23	0	20	6	14	70
Non-user	IOB_24	chr16	66432381	T	C	CDH5	MISSENSE	ENSG00000179776:[ENST00000341529(c.1508T>C,aTa>aCa):ENSP00000344115(p.Ile503Thr)]	NA	NA	4.35E-02	8.56E-02	8.70E-02	4.35E-02	7.26E-02	T	BN	NA	MODERATE	NA	NA	99	98	0	100	50	49	49
Non-user	IOB_24	chr16	66597096	C	T	CKLF	SILENT	ENSG00000217555:[ENST00000351137(c.150C>T,acC>acT):ENSP00000264003(p.Thr50(=))]	NA	NA	3.40E-03	1.78E-03	1.27E-02	3.39E-03	1.74E-02	NA	NA	NA	MODIFIER	NA	NA	269	268	0	204	117	85	41.67
Non-user	IOB_24	chr16	66597096	C	T	CKLF-CMTM1	SILENT	ENSG00000254788:[ENST00000532838(c.150C>T,acC>acT):ENSP00000433857(p.Thr50(=))]	NA	NA	3.40E-03	1.78E-03	1.27E-02	3.39E-03	1.74E-02	NA	NA	NA	MODIFIER	NA	NA	269	268	0	204	117	85	41.67
Non-user	IOB_24	chr16	67034779	G	A	CES4A	MISSENSE	ENSG00000172824:[ENST00000326686(c.422G>A,gGa>gAa):ENSP00000314145(p.Gly141Glu)]	NA	NA	2.20E-03	2.73E-03	1.30E-02	2.20E-03	1.12E-02	NA	NA	NA	MODIFIER	NA	NA	237	233	1	165	78	86	52.12
Non-user	IOB_24	chr16	67304654	G	A	SLC9A5	MISSENSE	6553:[NM_001323971.1(c.1856G>A,tGt>tAt):NP_001310900.1(p.Cys619Tyr)]	NA	NA	6.39E-03	1.15E-02	4.24E-02	6.39E-03	1.94E-02	NA	NA	NA	MODIFIER	NA	NA	102	102	0	77	37	40	51.95
Non-user	IOB_24	chr16	67338357	A	T	KCTD19	MISSENSE	ENSG00000168676:[ENST00000304372(c.418T>A,Tca>Aca):ENSP00000305702(p.Ser140Thr)]	NA	NA	6.59E-03	8.72E-03	2.70E-02	6.59E-03	1.94E-02	NA	NA	NA	MODIFIER	NA	NA	86	85	0	74	36	38	51.35
Non-user	IOB_24	chr16	67360778	T	A	LRRC36	MISSENSE	ENSG00000159708:[ENST00000329956(c.13T>A,Tgg>Agg):ENSP00000329943(p.Trp5Arg)]	NA	NA	5.99E-04	5.26E-04	1.18E-03	5.99E-04	3.10E-03	NA	NA	NA	MODIFIER	NA	NA	103	100	0	102	43	58	56.86
Non-user	IOB_24	chr16	67577275	C	T	FAM65A	MISSENSE	ENSG00000039523:[ENST00000042381(c.2503C>T,Cgg>Tgg):ENSP00000042381(p.Arg835Trp)]	NA	NA	5.99E-04	2.50E-04	1.39E-03	5.99E-04	3.10E-03	D	PrD	NA	MODERATE	D	1	130	130	0	114	61	52	45.61
Non-user	IOB_24	chr16	67979423	G	T	SLC12A4	SILENT	ENSG00000124067:[ENST00000422611(c.2887C>A,Cgg>Agg):ENSP00000395983(p.Arg963(=))]	NA	NA	3.02E-02	5.62E-02	7.43E-02	3.02E-02	6.95E-02	NA	NA	NA	LOW	NA	1	216	216	0	190	100	90	47.37
Non-user	IOB_24	chr16	68024784	C	T	DPEP2	SILENT	ENSG00000167261:[ENST00000575510(c.225G>A,tcG>tcA):ENSP00000462652(p.Ser75(=))]	NA	NA	NA	2.06E-04	0.00E+00	NA	NA	NA	NA	NA	LOW	NA	NA	168	168	0	127	56	71	55.91
Non-user	IOB_24	chr16	68024784	C	T	DUS2	INTRONIC	ENSG00000167264:[ENST00000571081(c.-99+2928C>T)]	NA	NA	NA	2.06E-04	0.00E+00	NA	NA	NA	NA	NA	LOW	NA	NA	168	168	0	127	56	71	55.91
Non-user	IOB_24	chr16	68397776	G	A	SMPD3	INTRONIC-SS-ACR-PRX	ENSG00000103056:[ENST00000219334(c.1556-7C>T)]	NA	NA	7.79E-03	1.58E-02	2.93E-02	7.79E-03	1.94E-02	NA	NA	NA	LOW	NA	NA	120	120	0	92	48	44	47.83
Non-user	IOB_24	chr16	68597330	G	A	ZFP90	MISSENSE	ENSG00000184939:[ENST00000398253(c.640G>A,Gat>Aat):ENSP00000381304(p.Asp214Asn)]	NA	NA	2.80E-03	3.48E-03	1.76E-02	2.80E-03	8.20E-03	NA	NA	NA	MODIFIER	NA	NA	91	91	0	95	52	43	45.26
Non-user	IOB_24	chr16	69377499	T	C	RP11-343C2.7	INTRONIC	ENSG00000259900:[ENST00000570293(c.316+5929A>G)]	NA	NA	3.16E-02	6.43E-02	2.48E-02	3.15E-02	1.84E-02	NA	NA	NA	MODIFIER	NA	NA	92	92	0	72	34	38	52.78
Non-user	IOB_24	chr16	69377499	T	C	RP11-343C2.9	INTRONIC	ENSG00000260371:[ENST00000563634(c.3-6884A>G)]	NA	NA	3.16E-02	6.43E-02	2.48E-02	3.15E-02	1.84E-02	NA	NA	NA	MODIFIER	NA	NA	92	92	0	72	34	38	52.78
Non-user	IOB_24	chr16	69377499	T	C	TMED6	SILENT	ENSG00000157315:[ENST00000288025(c.534A>G,cgA>cgG):ENSP00000288025(p.Arg178(=))]	NA	NA	3.16E-02	6.43E-02	2.48E-02	3.15E-02	1.84E-02	NA	NA	NA	MODIFIER	NA	NA	92	92	0	72	34	38	52.78
Non-user	IOB_24	chr16	69748869	G	A	NQO1	MISSENSE-SS-PRX	ENSG00000181019:[ENST00000564043(c.352C>T,Cgg>Tgg):ENSP00000455020(p.Arg118Trp)]	NA	NA	2.14E-02	3.28E-02	6.16E-02	2.14E-02	5.01E-02	NA	NA	NA	MODIFIER	NA	NA	118	118	0	103	57	44	42.72
Non-user	IOB_24	chr16	70163691	C	T	PDPR	INTRONIC	55066:[XM_005256018.1(c.607+666C>T)]	NA	NA	NA	NA	NA	NA	NA	NA	NA	NA	MODIFIER	NA	NA	416	415	0	520	232	285	54.81
Non-user	IOB_24	chr16	70164334	A	G	PDPR	MISSENSE	ENSG00000090857:[ENST00000288050(c.616A>G,Atc>Gtc):ENSP00000288050(p.Ile206Val)]	NA	chr16*70164334*70164334*COSM148098*A*G*1*stomach*1	NA	4.01E-01	3.61E-01	NA	NA	T	BN	NA	MODERATE	D	1	208	206	2	223	93	129	57.85
Non-user	IOB_24	chr16	70165746	C	T	PDPR	INTRONIC	55066:[XM_005256019.1(c.32-308C>T)]	NA	NA	NA	NA	NA	NA	NA	NA	NA	NA	MODIFIER	NA	NA	347	345	0	528	497	30	5.68
Non-user	IOB_24	chr16	70165749	T	C	PDPR	NCT	55066:[XR_243415.1(n.1059-305T>C)]	NA	NA	NA	NA	NA	NA	NA	NA	NA	NA	MODIFIER	NA	NA	359	356	0	539	502	33	6.12
Non-user	IOB_24	chr16	70172890	C	T	PDPR	MISSENSE	ENSG00000090857:[ENST00000398122(c.979C>T,Cgc>Tgc):ENSP00000381190(p.Arg327Cys)]	NA	chr16*70172890*70172890*COSM148100*C*T*1*stomach*1	NA	1.81E-01	1.63E-01	NA	NA	D	PoD	NA	MODERATE	D	1	133	132	0	155	98	56	36.13
Non-user	IOB_24	chr16	70176451	C	T	PDPR	INTRONIC	55066:[NM_001322119.1(c.266-5C>T)]	NA	NA	NA	7.23E-02	8.16E-02	NA	NA	NA	NA	NA	LOW	NA	NA	278	278	0	301	243	55	18.27
Non-user	IOB_24	chr16	70182375	T	C	PDPR	SILENT	ENSG00000090857:[ENST00000542659(c.6T>C,agT>agC):ENSP00000441690(p.Ser2(=))]	NA	NA	NA	2.76E-01	2.70E-01	NA	NA	NA	NA	NA	MODIFIER	NA	NA	193	193	0	176	105	71	40.34
Non-user	IOB_24	chr16	70182390	T	G	PDPR	MISSENSE	ENSG00000090857:[ENST00000563930(c.60T>G,aaT>aaG):ENSP00000457961(p.Asn20Lys)]	NA	NA	chr16*70182390*70182390*T*C,G*rs62050978*NA*NA*NA	3.38E-01	3.22E-01	NA	NA	NA	NA	NA	MODERATE	NA	0.013	190	189	1	186	107	78	41.94
Non-user	IOB_24	chr16	70187270	T	G	PDPR	INTRONIC	ENSG00000090857:[ENST00000569042(c.202-24T>G)]	NA	NA	chr16*70187270*70187270*T*C,G*rs2287980*NA*NA*NA	3.78E-01	3.66E-01	NA	NA	NA	NA	NA	MODIFIER	NA	NA	359	359	0	445	273	171	38.43
Non-user	IOB_24	chr16	70190401	C	T	LOC400541	EXONIC-NC	400541:[NR_126007.1(n.413G>A)]	NA	NA	NA	3.90E-01	3.50E-01	NA	NA	NA	NA	NA	LOW	NA	0.026	254	252	0	257	112	142	55.25
Non-user	IOB_24	chr16	70190401	C	T	PDPR	SILENT	ENSG00000090857:[ENST00000288050(c.2259C>T,gaC>gaT):ENSP00000288050(p.Asp753(=))]	NA	NA	NA	3.90E-01	3.50E-01	NA	NA	NA	NA	NA	LOW	NA	0.026	254	252	0	257	112	142	55.25
Non-user	IOB_24	chr16	70190401	C	T	RP11-296I10.3	EXONIC-NC	ENSG00000247228:[ENST00000566989(n.384G>A)]	NA	NA	NA	3.90E-01	3.50E-01	NA	NA	NA	NA	NA	LOW	NA	0.026	254	252	0	257	112	142	55.25
Non-user	IOB_24	chr16	70211218	G	A	CLEC18C	SILENT	ENSG00000157335:[ENST00000314151(c.291G>A,gcG>gcA):ENSP00000326538(p.Ala97(=))]	NA	chr16*70211218*70211218*COSM1479048*G*A*1*breast*1	NA	2.33E-01	1.47E-01	NA	NA	NA	NA	NA	MODIFIER	NA	NA	93	93	0	152	114	38	25
Non-user	IOB_24	chr16	70219777	A	C	CLEC18C	INTRONIC	ENSG00000157335:[ENST00000314151(c.1212-11A>C)]	NA	NA	NA	2.53E-01	3.74E-01	NA	NA	NA	NA	NA	MODIFIER	NA	NA	305	290	13	17	0	17	100
Non-user	IOB_24	chr16	70866856	C	T	HYDIN	SILENT	ENSG00000157423:[ENST00000393567(c.13794G>A,gtG>gtA):ENSP00000377197(p.Val4598(=))]	NA	NA	NA	1.03E-04	2.19E-04	NA	NA	NA	NA	NA	LOW	NA	NA	89	89	0	53	30	22	41.51
Non-user	IOB_24	chr16	70883749	C	T	HYDIN	SILENT	ENSG00000157423:[ENST00000393567(c.12753G>A,ctG>ctA):ENSP00000377197(p.Leu4251(=))]	NA	NA	NA	6.44E-04	5.00E-03	NA	NA	NA	NA	NA	LOW	NA	NA	337	336	0	251	205	43	17.13
Non-user	IOB_24	chr16	70913579	C	A	HYDIN	SILENT	ENSG00000157423:[ENST00000393567(c.10296G>T,acG>acT):ENSP00000377197(p.Thr3432(=))]	NA	chr16*70913579*70913579*COSM194924*C*A*1*large_intestine*1|chr16*70913579*70913579*COSM194925*C*A*1*large_intestine*1	NA	1.35E-05	1.00E-04	NA	NA	NA	NA	NA	LOW	NA	NA	316	315	0	195	151	44	22.56
Non-user	IOB_24	chr16	71094434	G	A	HYDIN	SILENT	ENSG00000157423:[ENST00000393552(c.1356C>T,ttC>ttT):ENSP00000463767(p.Phe452(=))]	NA	NA	NA	3.57E-03	0.00E+00	NA	NA	NA	NA	NA	MODIFIER	NA	NA	188	185	0	194	137	55	28.35
Non-user	IOB_24	chr16	71318570	C	T	CMTR2	NONSENSE	ENSG00000180917:[ENST00000338099(c.1254G>A,tgG>tgA):ENSP00000337512(p.Trp418Ter)]	NA	chr16*71318570*71318570*COSM1379598*C*T*1*large_intestine*1	NA	NA	NA	NA	NA	NA	NA	NA	HIGH	NA	1	137	137	0	98	60	38	38.78
Non-user	IOB_24	chr16	71710428	G	A	PHLPP2	SILENT	ENSG00000040199:[ENST00000567016(c.1498C>T,Ctg>Ttg):ENSP00000454519(p.Leu500(=))]	NA	NA	4.23E-02	5.78E-02	7.29E-02	4.23E-02	7.98E-02	NA	NA	NA	LOW	NA	1	134	132	0	108	47	60	55.56
Non-user	IOB_24	chr16	71718333	G	A	PHLPP2	NCT	ENSG00000040199:[ENST00000538126(n.785+46C>T)]	NA	NA	3.99E-04	4.46E-04	3.30E-03	3.99E-04	2.00E-03	NA	NA	NA	MODIFIER	NA	NA	45	45	0	36	11	25	69.44
Non-user	IOB_24	chr16	71885433	C	A	ATXN1L	MISSENSE	ENSG00000224470:[ENST00000427980(c.1790C>A,cCc>cAc):ENSP00000415822(p.Pro597His)]	NA	NA	1.42E-02	3.00E-02	6.72E-02	1.42E-02	6.65E-02	NA	NA	NA	MODIFIER	NA	NA	227	222	2	183	96	87	47.54
Non-user	IOB_24	chr16	71885433	C	A	IST1	INTRONIC	ENSG00000182149:[ENST00000568581(c.-16+3459C>A)]	NA	NA	1.42E-02	3.00E-02	6.72E-02	1.42E-02	6.65E-02	NA	NA	NA	MODIFIER	NA	NA	227	222	2	183	96	87	47.54
Non-user	IOB_24	chr16	72001121	C	T	PKD1L3	MISSENSE	342372:[NM_181536.1(c.2380G>A,Gtc>Atc):NP_853514.1(p.Val794Ile)]	NA	NA	9.98E-04	1.38E-03	3.79E-03	9.98E-04	4.10E-03	NA	NA	NA	MODIFIER	NA	NA	86	86	0	60	42	18	30
Non-user	IOB_24	chr16	72088467	A	G	TXNL4B	INTRONIC	ENSG00000140830:[ENST00000562153(c.285-10211T>C)]	NA	NA	NA	3.49E-01	3.74E-01	NA	NA	NA	NA	NA	MODIFIER	NA	NA	71	71	0	53	13	39	73.58
Non-user	IOB_24	chr16	72110948	C	G	HPR	MISSENSE	ENSG00000261701:[ENST00000356967(c.1015C>G,Cac>Gac):ENSP00000349451(p.His339Asp)]	NA	NA	NA	3.63E-01	4.59E-01	NA	NA	T	BN	NA	MODERATE	N	1	197	196	0	117	0	117	100
Non-user	IOB_24	chr16	72110948	C	G	TXNL4B	INTRONIC	ENSG00000140830:[ENST00000562153(c.284+11938G>C)]	NA	NA	NA	3.63E-01	4.59E-01	NA	NA	T	BN	NA	MODERATE	N	1	197	196	0	117	0	117	100
Non-user	IOB_24	chr16	72188359	C	T	PMFBP1	INTRONIC-SS-ACR	ENSG00000118557:[ENST00000355636(c.-270-1G>A)]	NA	NA	.	1.65E-05	1.22E-04	NA	NA	NA	NA	NA	HIGH	NA	NA	59	59	0	37	16	21	56.76
Non-user	IOB_24	chr16	73126970	C	A	HCCAT5	NCT	ENSG00000260880:[ENST00000569990(n.638-10C>A)]	NA	NA	NA	3.38E-05	0.00E+00	NA	NA	NA	NA	NA	MODIFIER	NA	NA	319	317	1	244	127	116	47.54
Non-user	IOB_24	chr16	74366531	A	C	AC009120.6	NCT	ENSG00000259972:[ENST00000561921(n.455+2259T>G)]	NA	NA	NA	4.23E-01	3.87E-01	NA	NA	NA	NA	NA	MODIFIER	NA	NA	68	66	1	128	66	62	48.44
Non-user	IOB_24	chr16	74366531	A	C	LOC283922	EXONIC-NC	283922:[NR_026950.1(n.2090T>G)]	NA	NA	NA	4.23E-01	3.87E-01	NA	NA	NA	NA	NA	MODIFIER	NA	NA	68	66	1	128	66	62	48.44
Non-user	IOB_24	chr16	74366531	A	C	RP11-252A24.2	EXONIC-NC	ENSG00000214331:[ENST00000566802(n.2089T>G)]	NA	NA	NA	4.23E-01	3.87E-01	NA	NA	NA	NA	NA	MODIFIER	NA	NA	68	66	1	128	66	62	48.44
Non-user	IOB_24	chr16	74372302	C	T	LOC283922	NCT	283922:[NR_026950.1(n.1709+41G>A)]	NA	NA	NA	1.58E-01	1.47E-01	NA	NA	NA	NA	NA	MODIFIER	NA	NA	149	148	0	136	99	37	27.21
Non-user	IOB_24	chr16	74372302	C	T	RP11-252A24.2	NCT	ENSG00000214331:[ENST00000563377(n.1604+41G>A)]	NA	NA	NA	1.58E-01	1.47E-01	NA	NA	NA	NA	NA	MODIFIER	NA	NA	149	148	0	136	99	37	27.21
Non-user	IOB_24	chr16	74394745	A	G	LOC283922	EXONIC-NC	283922:[NR_026950.1(n.236T>C)]	NA	NA	chr16*74394745*74394745*A*C,G*rs71391080*NA*NA*NA	4.14E-01	3.92E-01	NA	NA	NA	NA	NA	MODIFIER	NA	NA	130	130	0	171	112	59	34.5
Non-user	IOB_24	chr16	74394745	A	G	RP11-252A24.2	EXONIC-NC	ENSG00000214331:[ENST00000563377(n.13T>C)]	NA	NA	chr16*74394745*74394745*A*C,G*rs71391080*NA*NA*NA	4.14E-01	3.92E-01	NA	NA	NA	NA	NA	MODIFIER	NA	NA	130	130	0	171	112	59	34.5
Non-user	IOB_24	chr16	74443542	G	C	CLEC18B	INTRONIC-SS-ACR-PRX	ENSG00000140839:[ENST00000339953(c.1239-3C>G)]	NA	NA	NA	1.35E-01	1.33E-01	NA	NA	NA	NA	NA	LOW	NA	NA	199	197	2	406	293	113	27.83
Non-user	IOB_24	chr16	75241139	C	G	NA	INTERGENIC	NA	NA	NA	2.94E-02	NA	NA	2.94E-02	9.30E-02	NA	NA	NA	MODIFIER	NA	NA	210	210	0	140	68	72	51.43
Non-user	IOB_24	chr16	75269003	G	A	BCAR1	SILENT	ENSG00000050820:[ENST00000162330(c.1794C>T,caC>caT):ENSP00000162330(p.His598(=))]	NA	NA	3.89E-02	3.58E-02	9.27E-02	3.89E-02	9.41E-02	NA	NA	NA	LOW	NA	0.026	356	356	0	243	140	102	41.98
Non-user	IOB_24	chr16	75269325	C	T	BCAR1	MISSENSE	ENSG00000050820:[ENST00000535626(c.1028G>A,cGt>cAt):ENSP00000440370(p.Arg343His)]	NA	NA	2.96E-02	3.83E-02	9.85E-02	2.96E-02	9.41E-02	NA	NA	NA	MODERATE	NA	0.976	271	269	1	196	107	89	45.41
Non-user	IOB_24	chr16	75272029	G	A	BCAR1	EXONIC-NC	ENSG00000050820:[ENST00000566982(n.530C>T)]	NA	NA	3.95E-02	6.77E-02	9.95E-02	3.95E-02	9.71E-02	NA	NA	NA	MODIFIER	NA	NA	164	164	0	108	52	56	51.85
Non-user	IOB_24	chr16	75282024	A	T	BCAR1	INTRONIC	ENSG00000050820:[ENST00000393422(c.67-5036T>A)]	NA	NA	3.87E-02	NA	NA	3.87E-02	9.41E-02	NA	NA	NA	MODIFIER	NA	NA	96	96	0	66	23	43	65.15

Patel *et al.* , 2021. Whole-exome sequencing analysis of oral squamous cell carcinoma delineated by tobacco usage habits
Supplementary Table 4. List of somatic single nucleotide variants identified using whole exome sequencing of OSCC patients

Cohort	Sample	Chromosome	Genomic position	Reference allele	Alternate allele	Genesymbol	Variant type	Variant description	OncoMD	COSMIC	dbSNP	ExAC	ExAC_SAS	1000G	1000G_SAS	SIFT	Polyphen2	CONDEL	VEP	LRT	PhastCon7Way	Total reads in Normal	Reference read support in Normal	Alternative read support in Normal	Total reads in Tumor	Reference read support in Tumor	Alternate read support in Tumor	Alternate allele frequency in Tumor
Non-user	IOB_24	chr16	75285239	A	G	BCAR1	INTRONIC	ENSG00000050820:[ENST00000162330(c.12+130T>C)]	NA	NA	3.87E-02	NA	NA	3.87E-02	9.41E-02	NA	NA	NA	MODIFIER	NA	NA	97	97	0	82	47	35	42.68
Non-user	IOB_24	chr16	75301759	G	C	BCAR1	INTRONIC	ENSG00000050820:[ENST00000393422(c.66+54C>G)]	NA	NA	3.79E-02	NA	NA	3.79E-02	9.00E-02	NA	NA	NA	MODIFIER	NA	NA	51	51	0	36	23	13	36.11
Non-user	IOB_24	chr16	75301838	G	C	BCAR1	MISSENSE	ENSG00000050820:[ENST00000393422(c.41C>G,cCc>cGc):ENSP00000377074(p.Pro14Arg)]	NA	NA	3.75E-02	5.10E-02	1.12E-01	3.75E-02	8.79E-02	T_lc	BN	NA	MODERATE	NA	NA	60	60	0	53	33	20	37.74
Non-user	IOB_24	chr16	77228147	A	G	MON1B	EXONIC-NC	ENSG00000103111:[ENST00000566455(n.283A>G)]	NA	NA	1.46E-02	7.06E-03	5.30E-02	1.46E-02	7.46E-02	NA	NA	NA	MODIFIER	NA	NA	301	299	0	237	114	123	51.9
Non-user	IOB_24	chr16	78064555	G	A	CLEC3A	SILENT	ENSG00000166509:[ENST00000299642(c.438G>A,acG>acA):ENSP00000299642(p.Thr146(=))]	NA	NA	7.19E-03	3.61E-03	2.51E-02	7.19E-03	3.07E-02	NA	NA	NA	MODIFIER	NA	NA	163	162	0	116	56	60	51.72
Non-user	IOB_24	chr16	78064555	G	A	RP11-281J9.2	NCT	ENSG00000261540:[ENST00000563114(n.42-15529C>T)]	NA	NA	7.19E-03	3.61E-03	2.51E-02	7.19E-03	3.07E-02	NA	NA	NA	MODIFIER	NA	NA	163	162	0	116	56	60	51.72
Non-user	IOB_24	chr16	80696691	T	C	CDYL2	INTRONIC	ENSG00000166446:[ENST00000570137(c.616+21744A>G)]	NA	NA	3.12E-02	2.03E-02	2.41E-02	3.12E-02	3.68E-02	NA	NA	NA	MODIFIER	NA	NA	123	122	0	121	64	57	47.11
Non-user	IOB_24	chr16	81124354	T	C	GCSH	NCT	2653:[NR_033249.1(n.246-69A>G)]	NA	NA	7.99E-04	NA	NA	7.99E-04	1.00E-03	NA	NA	NA	MODIFIER	NA	NA	111	110	1	98	59	39	39.8
Non-user	IOB_24	chr16	81164095	G	T	PKD1L2	SILENT	ENSG00000166473:[ENST00000533478(c.3957C>A,acC>acA):ENSP00000434644(p.Thr1319(=))]	NA	NA	5.99E-04	8.31E-04	6.11E-04	5.99E-04	0.00E+00	NA	NA	NA	LOW	NA	NA	60	59	0	72	37	34	47.22
Non-user	IOB_24	chr16	81211511	T	C	PKD1L2	MISSENSE	ENSG00000166473:[ENST00000526632(c.920A>G,Att>Gtt):ENSP00000436389(p.Ile308Val)]	NA	NA	NA	1.17E-04	8.88E-04	NA	NA	NA	NA	NA	MODIFIER	NA	NA	81	81	0	50	23	27	54
Non-user	IOB_24	chr16	81250029	C	T	PKD1L2	NCT	114780:[NR_126532.1(n.302-18G>A)]	NA	NA	3.99E-03	6.34E-03	1.30E-02	3.99E-03	9.20E-03	NA	NA	NA	MODIFIER	NA	NA	145	143	0	68	38	30	44.12
Non-user	IOB_24	chr16	84213439	G	A	TAF1C	MISSENSE	ENSG00000103168:[ENST00000568265(c.429C>T,cCg>cTg):ENSP00000456101(p.Pro144Leu)]	NA	NA	1.40E-03	7.67E-03	5.23E-03	1.40E-03	2.00E-03	D	BN	NA	MODERATE	N	0	221	215	0	171	99	69	40.35
Non-user	IOB_24	chr16	84494168	A	T	ATP2C2	NCT	ENSG00000064270:[ENST00000420010(n.2007-92A>T)]	NA	NA	3.53E-02	5.60E-02	3.41E-02	3.53E-02	3.27E-02	NA	NA	NA	MODIFIER	NA	NA	203	203	0	147	78	68	46.26
Non-user	IOB_24	chr16	84494168	A	T	RP11-517C16.2	EXONIC-NC	ENSG00000261286:[ENST00000565700(n.1695T>A)]	NA	NA	3.53E-02	5.60E-02	3.41E-02	3.53E-02	3.27E-02	NA	NA	NA	MODIFIER	NA	NA	203	203	0	147	78	68	46.26
Non-user	IOB_24	chr16	85699706	G	A	GSE1	SILENT	ENSG00000131149:[ENST00000405402(c.2571G>A,tcG>tcA):ENSP00000384839(p.Ser857(=))]	NA	NA	5.99E-03	2.85E-03	2.02E-02	5.99E-03	3.07E-02	NA	NA	NA	LOW	NA	NA	165	165	0	137	68	69	50.36
Non-user	IOB_24	chr16	85948098	G	A	IRF8	SILENT	ENSG00000140968:[ENST00000268638(c.573G>A,ggG>ggA):ENSP00000268638(p.Gly191(=))]	NA	NA	4.65E-02	5.49E-02	8.14E-02	4.65E-02	5.93E-02	NA	NA	NA	LOW	NA	NA	251	250	0	164	87	77	46.95
Non-user	IOB_24	chr16	85948140	C	T	IRF8	INTRONIC	ENSG00000140968:[ENST00000566369(c.275+2876C>T)]	NA	NA	chr16*85948140*85948140*C*G,T*rs373421618*NA*NA*NA	1.08E-04	0.00E+00	NA	NA	NA	NA	NA	MODIFIER	NA	NA	215	213	0	135	75	60	44.44
Non-user	IOB_24	chr16	86565666	C	T	MTHFSD	MISSENSE	ENSG00000103248:[ENST00000546093(c.614G>A,gGc>gAc):ENSP00000438761(p.Gly205Asp)]	NA	NA	NA	NA	NA	NA	NA	NA	NA	NA	MODIFIER	NA	NA	72	72	0	70	35	34	48.57
Non-user	IOB_24	chr16	86588649	T	A	MTHFSD	INTRONIC	ENSG00000103248:[ENST00000546093(c.-548+130A>T)]	NA	NA	2.00E-03	NA	NA	2.00E-03	8.20E-03	NA	NA	NA	MODIFIER	NA	NA	128	126	1	143	55	87	60.84
Non-user	IOB_24	chr16	87678576	C	T	JPH3	SILENT	ENSG00000154118:[ENST00000284262(c.1095C>T,cgC>cgT):ENSP00000284262(p.Arg365(=))]	NA	chr16*87678576*87678576*COSM435891*C*T*1*breast*1	1.38E-02	2.03E-02	1.16E-02	1.38E-02	7.20E-03	NA	NA	NA	LOW	NA	NA	144	143	1	119	58	58	48.74
Non-user	IOB_24	chr16	87743109	G	A	KLHDC4	SILENT	ENSG00000104731:[ENST00000562261(c.939C>T,acC>acT):ENSP00000455780(p.Thr313(=))]	NA	NA	4.39E-03	2.19E-03	3.29E-03	4.39E-03	4.10E-03	NA	NA	NA	MODIFIER	NA	NA	328	326	0	235	128	106	45.11
Non-user	IOB_24	chr16	88071516	C	T	BANP	INTRONIC	ENSG00000172530:[ENST00000286122(c.1240-63C>T)]	NA	NA	3.79E-03	NA	NA	3.79E-03	1.64E-02	NA	NA	NA	MODIFIER	NA	NA	118	117	0	92	46	46	50
Non-user	IOB_24	chr16	88494280	C	T	ZNF469	SILENT	ENSG00000225614:[ENST00000437464(c.402C>T,gaC>gaT):ENSP00000402343(p.Asp134(=))]	NA	NA	2.80E-03	3.87E-03	6.24E-03	2.80E-03	1.43E-02	NA	NA	NA	LOW	NA	NA	322	321	0	209	107	100	47.85
Non-user	IOB_24	chr16	88520070	C	T	ZFPM1	SILENT	ENSG00000179588:[ENST00000319555(c.24C>T,aaC>aaT):ENSP00000326630(p.Asn8(=))]	NA	NA	4.65E-02	7.61E-02	8.66E-02	4.65E-02	7.06E-02	NA	NA	NA	MODIFIER	NA	NA	93	91	0	93	54	39	41.94
Non-user	IOB_24	chr16	88698188	C	T	ZC3H18	3UTR	ENSG00000158545:[ENST00000301011(c.*481C>T)]	NA	NA	8.99E-03	NA	NA	8.99E-03	4.10E-03	NA	NA	NA	MODIFIER	NA	NA	52	51	0	25	16	8	32
Non-user	IOB_24	chr16	88781272	G	T	CTU2	MISSENSE	ENSG00000174177:[ENST00000567949(c.1570G>T,Gac>Tac):ENSP00000456908(p.Asp524Tyr)]	NA	NA	1.20E-03	3.28E-04	2.37E-03	1.20E-03	6.10E-03	D_lc	PrD	NA	MODERATE	NA	NA	148	146	0	136	71	64	47.06
Non-user	IOB_24	chr16	88783418	C	A	PIEZO1	INTRONIC	ENSG00000103335:[ENST00000301015(c.6660+13G>T)]	NA	NA	1.12E-02	1.33E-02	3.37E-02	1.12E-02	5.52E-02	NA	NA	NA	MODIFIER	NA	NA	253	251	0	268	131	137	51.12
Non-user	IOB_24	chr16	88872535	C	T	CDT1	INTRONIC-SS-DNR-PRX	ENSG00000167513:[ENST00000301019(c.933+6C>T)]	NA	NA	NA	NA	NA	NA	NA	NA	NA	NA	MODIFIER	NA	NA	245	245	0	185	180	5	2.7
Non-user	IOB_24	chr16	88902678	G	A	GALNS	NCT-SS-PRX	ENSG00000141012:[ENST00000566563(n.269-3C>T)]	NA	NA	3.99E-04	8.93E-04	6.03E-03	3.99E-04	2.00E-03	NA	NA	NA	LOW	NA	NA	227	226	0	176	85	89	50.57
Non-user	IOB_24	chr16	89167668	G	A	ACSF3	SILENT	ENSG00000176715:[ENST00000317447(c.579G>A,agG>agA):ENSP00000320646(p.Arg193(=))]	NA	NA	3.99E-04	7.82E-04	4.07E-03	3.99E-04	2.00E-03	NA	NA	NA	MODIFIER	NA	NA	301	298	0	256	149	103	40.23
Non-user	IOB_24	chr16	89287481	C	A	ZNF778	5UTR	ENSG00000170100:[ENST00000433976(c.-55C>A)]	NA	NA	4.01E-02	NA	NA	4.01E-02	7.46E-02	NA	NA	NA	MODIFIER	NA	NA	43	43	0	57	36	21	36.84
Non-user	IOB_24	chr16	89293291	G	A	RP11-46C24.6	EXONIC-NC	ENSG00000260659:[ENST00000563182(n.327C>T)]	NA	NA	4.13E-02	4.22E-02	9.42E-02	4.13E-02	8.18E-02	T	BN	NA	MODERATE	NA	NA	271	270	0	182	91	91	50
Non-user	IOB_24	chr16	89293291	G	A	ZNF778	MISSENSE	ENSG00000170100:[ENST00000306502(c.385G>A,Gtg>Atg):ENSP00000305203(p.Val129Met)]	NA	NA	4.13E-02	4.22E-02	9.42E-02	4.13E-02	8.18E-02	T	BN	NA	MODERATE	NA	NA	271	270	0	182	91	91	50
Non-user	IOB_24	chr16	89294798	A	G	RP11-46C24.6	NCT	ENSG00000260659:[ENST00000563182(n.235+68T>C)]	NA	NA	4.13E-02	4.27E-02	9.50E-02	4.13E-02	8.18E-02	D	PoD	NA	MODERATE	NA	NA	97	95	0	98	49	49	50
Non-user	IOB_24	chr16	89294798	A	G	ZNF778	MISSENSE	ENSG00000170100:[ENST00000433976(c.2018A>G,tAt>tGt):ENSP00000405289(p.Tyr673Cys)]	NA	NA	4.13E-02	4.27E-02	9.50E-02	4.13E-02	8.18E-02	D	PoD	NA	MODERATE	NA	NA	97	95	0	98	49	49	50
Non-user	IOB_24	chr16	89337312	G	A	AC137932.1	3UTR	ENSG00000268218:[ENST00000602042(c.*1114G>A)]	NA	NA	2.00E-04	9.28E-05	3.09E-04	2.00E-04	0.00E+00	NA	NA	NA	LOW	NA	0.14	163	162	0	135	64	69	51.11
Non-user	IOB_24	chr16	89337312	G	A	ANKRD11	SILENT	ENSG00000167522:[ENST00000562194(c.400C>T,gaC>gaT):ENSP00000458043(p.Asp134(=))]	NA	NA	2.00E-04	9.28E-05	3.09E-04	2.00E-04	0.00E+00	NA	NA	NA	LOW	NA	0.14	163	162	0	135	64	69	51.11
Non-user	IOB_24	chr16	89337312	G	A	NA	EXONIC-NC	101927838:[XR_243512.1(n.1520G>A)]	NA	NA	2.00E-04	9.28E-05	3.09E-04	2.00E-04	0.00E+00	NA	NA	NA	LOW	NA	0.14	163	162	0	135	64	69	51.11
Non-user	IOB_24	chr16	89346614	G	A	ANKRD11	SILENT	ENSG00000167522:[ENST00000301030(c.6336C>T,ctC>ctT):ENSP00000301030(p.Leu2112(=))]	NA	NA	7.99E-04	4.80E-04	3.04E-03	7.99E-04	4.10E-03	NA	NA	NA	MODIFIER	NA	NA	143	143	0	126	66	60	47.62
Non-user	IOB_24	chr16	89749244	C	G	CDK10	INTRONIC	ENSG00000185324:[ENST00000331006(c.19+340C>G)]	NA	NA	2.56E-02	5.43E-02	6.67E-02	2.56E-02	6.65E-02	NA	NA	NA	MODIFIER	NA	NA	55	53	1	29	18	11	37.93
Non-user	IOB_24	chr16	89749244	C	G	RP11-368I7.4	MISSENSE	ENSG00000260259:[ENST00000567544(c.343G>C,Gca>Cca):ENSP00000457429(p.Ala115Pro)]	NA	NA	2.56E-02	5.43E-02	6.67E-02	2.56E-02	6.65E-02	NA	NA	NA	MODIFIER	NA	NA	55	53	1	29	18	11	37.93
Non-user	IOB_24	chr16	89858962	G	C	FANCA	EXONIC-NC	ENSG00000187741:[ENST00000566133(n.172C>G)]	NA	NA	2.00E-04	1.51E-04	1.10E-03	2.00E-04	1.00E-03	NA	NA	NA	LOW	NA	NA	62	61	0	40	24	15	37.5
Non-user	IOB_24	chr16	89877398	C	A	FANCA	MISSENSE	ENSG00000187741:[ENST00000565582(c.278G>T,gGa>gTa):ENSP00000456722(p.Gly93Val)]	NA	NA	chr16*89877398*89877398*C*T*rs914493816*NA*NA*NA	NA	NA	NA	NA	T	BN	NA	MODERATE	N	0	262	261	0	173	167	5	2.89
Non-user	IOB_24	chr16	89882574	C	T	FANCA	SILENT	ENSG00000187741:[ENST00000567943(c.450G>A,cgG>cgA):ENSP00000455941(p.Arg150(=))]	NA	NA	2.00E-04	4.42E-04	6.57E-04	2.00E-04	1.00E-03	NA	NA	NA	MODIFIER	NA	NA	112	112	0	90	52	38	42.22
Non-user	IOB_24	chr16	90086470	C	T	DBNDD1	SILENT	ENSG00000003249:[ENST00000568838(c.57G>A,agG>agA):ENSP00000457625(p.Arg19(=))]	NA	NA	chr16*90086470*90086470*C*G*rs762194695*NA*NA*NA	NA	NA	NA	NA	NA	NA	NA	MODIFIER	NA	NA	122	122	0	118	62	56	47.46
Non-user	IOB_24	chr16	90086470	C	T	GAS8	INTRONIC	ENSG00000141013:[ENST00000561675(c.-269+294C>T)]	NA	NA	chr16*90086470*90086470*C*G*rs762194695*NA*NA*NA	NA	NA	NA	NA	NA	NA	NA	MODIFIER	NA	NA	122	122	0	118	62	56	47.46
Non-user	IOB_24	chr16	90110678	G	A	GAS8	3UTR	ENSG00000141013:[ENST00000268699(c.*925G>A)]	NA	NA	NA	9.34E-05	1.35E-04	NA	NA	NA	NA	NA	MODIFIER	NA	NA	158	156	0	93	58	34	36.56
Non-user	IOB_24	chr16	90110678	G	A	URAHP	NCT	ENSG00000222019:[ENST00000610227(n.322+232C>T)]	NA	NA	NA	9.34E-05	1.35E-04	NA	NA	NA	NA	NA	MODIFIER	NA	NA	158	156	0	93	58	34	36.56
Non-user	IOB_24	chr17	455184	G	A	VPS53	MISSENSE	ENSG00000141252:[ENST00000389040(c.1597C>T,Ctc>Ttc):ENSP00000373692(p.Leu533Phe)]	NA	NA	1.60E-03	1.08E-03	6.22E-03	1.60E-03	8.20E-03	D	PrD	NA	MODERATE	NA	NA	140	140	0	77	33	44	57.14
Non-user	IOB_24	chr17	505105	T	C	VPS53	MISSENSE	ENSG00000141252:[ENST00000401468(c.317A>G,aAt>aGt):ENSP00000384294(p.Asn106Ser)]	NA	NA	1.38E-02	5.86E-03	4.21E-03	1.38E-02	2.00E-03	T	BN	NA	MODERATE	D	1	133	130	1	100	53	45	45
Non-user	IOB_24	chr17	644531	T	A	FAM57A	INTRONIC-SS-ACR-PRX	ENSG00000167695:[ENST00000572018(c.207-10T>A)]	NA	NA	7.99E-04	2.31E-04	1.70E-03	7.99E-04	4.10E-03	NA	NA	NA	MODIFIER	NA	NA	127	127	0	71	44	27	38.03
Non-user	IOB_24	chr17	914042	G	C	ABR	SILENT	ENSG00000159842:[ENST00000302538(c.2163C>G,ctC>ctG):ENSP00000303909(p.Leu721(=))]	NA	NA	NA	6.61E-05	4.24E-04	NA	NA	NA	NA	NA	LOW	NA	NA	111	111	0	60	27	32	53.33
Non-user	IOB_24	chr17	1438878	G	C	PITPNA	INTRONIC	ENSG00000174238:[ENST00000313486(c.457-66C>G)]	NA	NA	NA	NA	NA	NA	NA	NA	NA	NA	MODIFIER	NA	NA	60	59	0	37	18	18	48.65
Non-user	IOB_24	chr17	1761332	T	C	RPA1	INTRONIC	ENSG00000132383:[ENST00000254719(c.361+4849T>C)]	NA	NA	7.39E-03	NA	NA	7.39E-03	3.78E-02	NA	NA	NA	MODIFIER	NA	NA	108	97	1	76	30	35	46.05
Non-user	IOB_24	chr17	1795205	G	A	RPA1	MISSENSE	ENSG00000132383:[ENST00000254719(c.1630G>A,Gct>Act):ENSP00000254719(p.Ala544Thr)]	NA	NA	7.99E-04	2.31E-04	4.85E-04	7.99E-04	0.00E+00	NA	NA	NA	MODIFIER	NA	NA	51	51	0	23	9	13	56.52
Non-user	IOB_24	chr17	1961223	C	T	HIC1	SILENT	ENSG00000177374:[ENST00000322941(c.1296C>T,ttC>ttT):ENSP00000314080(p.Phe432(=))]	NA	NA	7.99E-04	5.46E-04	1.46E-03	7.99E-04	0.00E+00	NA	NA	NA	LOW	NA	NA	244	244	0	250	135	113	45.2
Non-user	IOB_24	chr17	2276765	C	T	SGSM2	SILENT	ENSG00000141258:[ENST00000426855(c.1923C>T,caC>caT):ENSP00000415107(p.His641(=))]	NA	NA	7.99E-04	6.08E-04	1.78E-03	7.99E-04	1.00E-03	NA	NA	NA	LOW	NA	0.157	170	168	0	131	74	57	43.51
Non-user	IOB_24	chr17	3427705	C	G	TRPV3	INTRONIC	ENSG00000167723:[ENST00000571139(c.*1570-48G>C)]	NA	NA	2.04E-02	2.63E-02	1.50E-02	2.04E-02	6.10E-03	NA	NA	NA	MODIFIER	NA	NA	141	139	0	134	71	62	46.27
Non-user	IOB_24	chr17	3552244	C	T	CTNS	NCT	ENSG00000040531:[ENST00000488623(n.446+19C>T)]	NA	NA	2.00E-04	9.89E-05	5.45E-04	2.00E-04	1.00E-03	NA	NA	NA	MODIFIER	NA	NA	112	112	0	101	50	50	49.5
Non-user	IOB_24	chr17	3626902	G	C	ITGAE	NCT	ENSG00000083457:[ENST00000572179(n.150+80C>G)]	NA	NA	1.84E-02	NA	NA	1.84E-02	5.62E-02	NA	NA	NA	MODIFIER	NA	NA	89	89	0	57	29	28	49.12
Non-user	IOB_24	chr17	4488035	A	G	SMTNL2	SILENT	ENSG00000188176:[ENST00000389313(c.135A>G,cgA>cgG):ENSP00000373964(p.Arg45(=))]	NA	NA	NA	NA	NA	NA	NA	NA	NA	NA	MODIFIER	NA	NA	54	54	0	38	18	20	52.63
Non-user	IOB_24	chr17	4784249	A	C	MINK1	INTRONIC	50488:[NM_001321236.1(c.-42-30A>C)]	NA	NA	4.95E-02	8.18E-02	3.95E-02	4.95E-02	2.56E-02	NA	NA	NA	MODIFIER	NA	NA	142	142	0	142	81	61	42.96
Non-user	IOB_24	chr17	4805389	G	A	C17orf107	3UTR	ENSG00000205710:[ENST00000381365(c.*1561G>A)]	NA	NA	4.97E-02	7.47E-02	2.91E-02	4.97E-02	2.56E-02	NA	NA	NA	MODIFIER	NA	NA	249	245	0	204	105	98	48.04
Non-user	IOB_24	chr17	4805389	G	A	CHRNE	NCT-SS-PRX	ENSG00000108556:[ENST00000575637(n.166-7C>T)]	NA	NA	4.97E-02	7.47E-02	2.91E-02	4.97E-02	2.56E-02	NA	NA	NA	MODIFIER	NA	NA	249	245	0	204	105	98	48.04
Non-user	IOB_24	chr17	4904564	T	C	KIF1C	SILENT	ENSG00000129250:[ENST00000320785(c.231T>C,atT>atC):ENSP00000320821(p.Ile77(=))]	NA	NA	4.71E-02	8.38E-02	3.36E-02	4.71E-02	2.45E-02	NA	NA	NA	LOW	NA	NA	155	155	0	99	57	42	42.42
Non-user	IOB_24	chr17	4907958	A	G	KIF1C	INTRONIC	ENSG00000129250:[ENST00000320785(c.1019+18A>G)]	NA	NA	4.87E-02	8.58E-02	3.28E-02	4.87E-02	2.25E-02	NA	NA	NA	MODIFIER	NA	NA	159	158	0	151	91	60	39.74
Non-user	IOB_24	chr17	5044749	G	A	USP6	MISSENSE	ENSG00000129204:[ENST00000304328(c.577G>A,Gag>Aag):ENSP00000305473(p.Glu193Lys)]	NA	NA	6.79E-03	5.18E-03	3.77E-02	6.79E-03	3.48E-02	D	BN	NA	MODERATE	D	1	109	109	0	80	44	36	45
Non-user	IOB_24	chr17	5291126	T	C	NUP88	MISSENSE	ENSG00000108559:[ENST00000573584(c.1742A>G,gAc>gGc):ENSP00000458954(p.Asp581Gly)]	NA	NA	9.98E-04	2.71E-03	2.01E-03	9.98E-04	1.00E-03	NA	NA	NA	MODIFIER	NA	NA	182	182	0	156	66	89	57.05
Non-user	IOB_24	chr17	5292262	C	T	NUP88	SILENT	ENSG00000108559:[ENST00000573584(c.1503G>A,gcG>gcA):ENSP00000458954(p.Ala501(=))]	NA	NA	7.99E-03	1.43E-02	7.05E-03	7.99E-03	5.10E-03	NA	NA	NA	LOW	NA	NA	58	58	0	42	25	17	40.48
Non-user	IOB_24	chr17	5486122	C	T	NLRP1	MISSENSE	ENSG00000091592:[ENST00000262467(c.316G>A,Ggg>Agg):ENSP00000262467(p.Gly106Arg)]	NA	NA	9.98E-04	2.97E-03	2.18E-03	9.98E-04	1.00E-03	NA	NA	NA	MODIFIER	NA	NA	108	108	0	85	45	40	47.06
Non-user	IOB_24	chr17	7095198	A	C	DLG4	INTRONIC	ENSG00000132535:[ENST00000399510(c.2105+14T>G)]	NA	NA	4.71E-02	2.70E-02	2.44E-02	4.71E-02	3.48E-02	NA	NA	NA	MODIFIER	NA	NA	242	241	0	202	110	91	45.05
Non-user	IOB_24	chr17	7164011	G	C	CLDN7	MISSENSE	ENSG00000181885:[ENST00000571881(c.397C>G,Cca>Gca):ENSP00000465963(p.Pro133Ala)]	NA	NA	NA	8.24E-06	6.06E-05	NA	NA	NA	NA	NA	LOW	NA	NA	217	217	0	173	79	93	53.76
Non-user	IOB_24	chr17	7164011	G	C	RP1-4G17.5	INTRONIC	ENSG00000262302:[ENST00000577138(c.223+1129C>G)]	NA	NA	NA	8.24E-06	6.06E-05	NA	NA	NA	NA	NA	LOW	NA	NA	217	217	0	173	79	93	53.76
Non-user	IOB_24	chr17	7259883	C	T	TMEM95	INTRONIC	ENSG00000182896:[ENST00000576060(c.498-35C>T)]	NA	NA	3.20E-03	2.49E-03	1.72E-02	3.19E-03	1.53E-02	NA	NA	NA	MODIFIER	NA	NA	133	133	0	97	47	46	47.42
Non-user	IOB_24	chr17	7290312	C	T	TNK1	SILENT	ENSG00000174292:[ENST00000576812(c.1251C>T,ccC>ccT):ENSP00000459799(p.Pro417(=))]	NA	NA	3.99E-04	1.03E-03	4.32E-03	3.99E-04	2.00E-03	NA	NA	NA	LOW	NA	NA	275	275	0	144	83	61	42.36
Non-user	IOB_24	chr17	7339419	T	C	RP11-104H15.7	NCT	ENSG00000262880:[ENST00000575310(n.273-5371T>C)]	NA	NA	NA	NA	NA	NA	NA	NA	NA	NA	MODIFIER	NA	NA	259	254	1	203	97	104	51.23
Non-user	IOB_24	chr17	7339419	T	C	TMEM102	INTRONIC	ENSG00000181284:[ENST00000323206(c.214+15T>C)]	NA	NA	NA	NA	NA	NA	NA	NA	NA	NA	MODIFIER	NA	NA	259	254	1	203	97	104	51.23
Non-user	IOB_24	chr17	7483483	C	T	CD68	SILENT	ENSG00000129226:[ENST00000250092(c.405C>T,acC>acT):ENSP00000250092(p.Thr135(=))]	NA	NA	5.99E-04	2.89E-04	2.06E-03	5.99E-04	3.10E-03	NA	NA	NA	LOW	NA	NA	184	183	0	160	75	83	51.88
Non-user	IOB_24	chr17	7483483	C	T	SNORA67	EXONIC-NC	ENSG00000264772:[ENST00000581621(n.3381C>T)]	NA	NA	5.99E-04	2.89E-04	2.06E-03	5.99E-04	3.10E-03	NA	NA	NA	LOW	NA	NA	184	183	0	160	75	83	51.88
Non-user	IOB_24	chr17	7662434	G	A	DNAH2	MISSENSE	ENSG00000183914:[ENST00000389173(c.2440G>A,Ggt>Agt):ENSP00000373825(p.Gly814Ser)]	NA	NA	3.20E-03	2.91E-03	2.04E-02	3.19E-03	1.64E-02	T	BN	NA	MODERATE	N	1	55	55	0	34	15	19	55.88
Non-user	IOB_24	chr17	7721189	G	T	DNAH2	INTRONIC	ENSG00000183914:[ENST00000575105(c.1083+15G>T)]	NA	NA	.	7.43E-05	4.85E-04	NA	NA	NA	NA	NA	MODIFIER	NA	NA	162	159	0	133	65	67	50.38
Non-user	IOB_24	chr17	7721196	T	C	DNAH2	INTRONIC	ENSG00000183914:[ENST00000575105(c.1083+22T>C)]	NA	NA	2.62E-02	4.27E-02	4.79E-02	2.62E-02	3.68E-02	NA	NA	NA	MODIFIER	NA	NA	166	163	0	137	70	67	48.91
Non-user	IOB_24	chr17	7722643	C	T	DNAH2	SILENT	ENSG00000183914:[ENST00000575105(c.1779C>T,ctC>ctT):ENSP00000461726(p.Leu593(=))]	NA	NA	4.41E-02	3.08E-02	1.56E-01	4.41E-02	1.70E-01	NA	NA	NA	LOW	NA	NA	133	133	0	99	52	47	47.47
Non-user	IOB_24	chr17	7755980	C	T	KDM6B	INTRONIC	ENSG00000132510:[ENST00000254846(c.4610+26C>T)]	NA	NA	3.44E-02	2.73E-02	1.41E-01	3.43E-02	1.53E-01	NA	NA	NA	MODIFIER	NA	NA	134	133	0	107	58	48	44.86

Patel *et al.* , 2021. Whole-exome sequencing analysis of oral squamous cell carcinoma delineated by tobacco usage habits
Supplementary Table 4. List of somatic single nucleotide variants identified using whole exome sequencing of OSCC patients

Cohort	Sample	Chromosome	Genomic position	Reference allele	Alternate allele	Genesymbol	Variant type	Variant description	OncoMD	COSMIC	dbSNP	ExAC	ExAC_SAS	1000G	1000G_SAS	SIFT	Polyphen2	CONDEL	VEP	LRT	PhastCon7Way	Total reads in Normal	Reference read support in Normal	Alternative read support in Normal	Total reads in Tumor	Reference read support in Tumor	Alternate read support in Tumor	Alternate allele frequency in Tumor
Non-user	IOB_24	chr17	7810596	C	T	CHD3	INTRONIC	ENSG00000170004:[ENST00000470531(c.*1159+31C>T)]	NA	NA	2.44E-02	3.74E-02	1.29E-02	2.44E-02	6.10E-03	NA	NA	NA	MODIFIER	NA	NA	93	93	0	73	36	36	49.32
Non-user	IOB_24	chr17	7814099	T	C	CHD3	INTRONIC	1107:[XM_005256429.1(c.5935-66T>C)]	NA	NA	2.62E-02	NA	NA	2.62E-02	6.10E-03	NA	NA	NA	MODIFIER	NA	NA	231	227	2	184	94	90	48.91
Non-user	IOB_24	chr17	7911333	A	T	GUCY2D	MISSENSE	ENSG00000132518:[ENST00000254854(c.1651A>T,Aac>Tac):ENSP00000254854(p.Asn551Tyr)]	NA	NA	NA	NA	NA	NA	NA	D	PrD	NA	MODERATE	D	1	131	128	0	98	59	39	39.8
Non-user	IOB_24	chr17	8021946	C	T	ALOXE3	NCT	59344:[XR_243563.1(n.352+68G>A)]	NA	NA	4.49E-02	NA	NA	4.49E-02	6.10E-03	NA	NA	NA	MODIFIER	NA	NA	363	357	1	243	116	127	52.26
Non-user	IOB_24	chr17	8135146	A	C	CTC1	NCT	80169:[NR_046431.1(n.2340-13T>G)]	NA	NA	4.31E-02	2.81E-02	1.28E-02	4.31E-02	8.20E-03	NA	NA	NA	MODIFIER	NA	NA	181	181	0	208	110	97	46.63
Non-user	IOB_24	chr17	8135184	G	A	CTC1	NCT	80169:[NR_046431.1(n.2339+37C>T)]	NA	NA	4.29E-02	2.80E-02	1.28E-02	4.29E-02	8.20E-03	NA	NA	NA	MODIFIER	NA	NA	160	160	0	177	97	80	45.2
Non-user	IOB_24	chr17	8135446	G	A	CTC1	SILENT	ENSG00000178971:[ENST00000578537(c.56C>T,acC>acT):ENSP00000463230(p.Thr19(=))]	NA	NA	4.33E-02	3.10E-02	1.23E-02	4.33E-02	8.20E-03	NA	NA	NA	LOW	NA	NA	108	108	0	107	59	48	44.86
Non-user	IOB_24	chr17	8784042	G	A	PIK3R5	MISSENSE	ENSG00000141506:[ENST00000584803(c.2554C>T,Cct>Tct):ENSP00000462680(p.Pro852Ser)]	NA	NA	1.80E-03	2.83E-03	1.61E-03	1.80E-03	3.10E-03	NA	NA	NA	MODIFIER	NA	NA	169	169	0	135	65	70	51.85
Non-user	IOB_24	chr17	8794009	T	A	PIK3R5	INTRONIC	ENSG00000141506:[ENST00000269300(c.483-566A>T)]	NA	NA	4.29E-02	8.59E-02	5.85E-02	4.29E-02	6.03E-02	NA	NA	NA	MODIFIER	NA	NA	44	43	0	48	23	24	50
Non-user	IOB_24	chr17	9604623	G	T	USP43	MISSENSE	ENSG00000154914:[ENST00000573955(c.192G>T,tgG>tgT):ENSP00000477318(p.Trp64Cys)]	NA	NA	4.07E-02	2.33E-02	2.41E-02	4.07E-02	1.84E-02	NA	NA	NA	MODIFIER	NA	NA	253	252	0	201	103	98	48.76
Non-user	IOB_24	chr17	9808426	C	G	RCVRN	SILENT	ENSG00000109047:[ENST00000226193(c.72G>C,tcG>tcC):ENSP00000226193(p.Ser24(=))]	NA	chr17*9808426*9808426*COSM1640920*C*T*1*stomach*1	3.40E-03	1.02E-02	3.71E-03	3.39E-03	1.00E-03	NA	NA	NA	LOW	NA	NA	213	213	0	152	68	84	55.26
Non-user	IOB_24	chr17	10243471	A	G	MYH13	SILENT	ENSG00000006788:[ENST00000252172(c.2052T>C,acT>acC):ENSP00000252172(p.Thr684(=))]	NA	NA	7.99E-04	7.90E-04	2.04E-03	7.99E-04	2.00E-03	NA	NA	NA	MODIFIER	NA	NA	160	158	1	130	65	65	50
Non-user	IOB_24	chr17	10243471	A	G	RP11-401O9.3	NCT	ENSG00000264067:[ENST00000577743(n.247-910A>G)]	NA	NA	7.99E-04	7.90E-04	2.04E-03	7.99E-04	2.00E-03	NA	NA	NA	MODIFIER	NA	NA	160	158	1	130	65	65	50
Non-user	IOB_24	chr17	10258155	T	C	MYH13	INTRONIC	ENSG00000006788:[ENST00000252172(c.902-55A>G)]	NA	NA	4.39E-03	NA	NA	4.39E-03	1.00E-03	NA	NA	NA	MODIFIER	NA	NA	127	127	0	99	1	96	96.97
Non-user	IOB_24	chr17	10427227	A	T	CTC-297N7.11	NCT	ENSG00000272975:[ENST00000587182(n.156-43627A>T)]	NA	NA	2.50E-02	4.32E-02	3.62E-02	2.50E-02	2.56E-02	NA	NA	NA	MODIFIER	NA	NA	39	39	0	35	19	16	45.71
Non-user	IOB_24	chr17	10427227	A	T	MYH2	INTRONIC	ENSG00000125414:[ENST00000245503(c.5176-26T>A)]	NA	NA	2.50E-02	4.32E-02	3.62E-02	2.50E-02	2.56E-02	NA	NA	NA	MODIFIER	NA	NA	39	39	0	35	19	16	45.71
Non-user	IOB_24	chr17	10427227	A	T	MYHAS	NCT	100128560:[NR_125367.1(n.168-43627A>T)]	NA	NA	2.50E-02	4.32E-02	3.62E-02	2.50E-02	2.56E-02	NA	NA	NA	MODIFIER	NA	NA	39	39	0	35	19	16	45.71
Non-user	IOB_24	chr17	10427227	A	T	RP11-799N11.1	NCT	ENSG00000272736:[ENST00000581304(n.144-9414A>T)]	NA	NA	2.50E-02	4.32E-02	3.62E-02	2.50E-02	2.56E-02	NA	NA	NA	MODIFIER	NA	NA	39	39	0	35	19	16	45.71
Non-user	IOB_24	chr17	11502123	T	C	DNAH9	MISSENSE	ENSG00000007174:[ENST00000262442(c.308T>C,tTc>tCc):ENSP00000262442(p.Phe103Ser)]	NA	NA	.	1.28E-03	7.06E-04	NA	NA	NA	NA	NA	MODIFIER	NA	NA	144	142	0	100	47	49	49
Non-user	IOB_24	chr17	11790306	G	C	DNAH9	NCT	ENSG00000007174:[ENST00000581682(n.393+24G>C)]	NA	NA	2.00E-04	4.20E-05	6.76E-05	2.00E-04	0.00E+00	NA	NA	NA	MODIFIER	NA	NA	209	209	0	176	89	87	49.43
Non-user	IOB_24	chr17	12859941	C	G	ARHGAP44	NCT	9912:[NR_135569.1(n.1578-31C>G)]	NA	NA	3.99E-04	9.44E-04	3.96E-03	3.99E-04	1.00E-03	NA	NA	NA	MODIFIER	NA	NA	128	128	0	95	52	43	45.26
Non-user	IOB_24	chr17	12901688	G	A	ELAC2	NCT	ENSG00000006744:[ENST00000492559(n.327+41C>T)]	NA	NA	4.95E-02	9.75E-02	1.03E-01	4.95E-02	6.75E-02	NA	NA	NA	MODIFIER	NA	NA	222	221	0	196	94	102	52.04
Non-user	IOB_24	chr17	12916606	A	G	ELAC2	INTRONIC	60528:[XM_005256748.1(c.-501-14T>C)]	NA	NA	4.95E-02	1.02E-01	1.07E-01	4.95E-02	6.75E-02	NA	NA	NA	MODIFIER	NA	NA	109	109	0	92	44	47	51.09
Non-user	IOB_24	chr17	13928093	G	A	CDRT15P1	INTRONIC-SS-DNR	ENSG00000141028:[ENST00000577632(n.262+1G>A)]	NA	NA	NA	NA	NA	NA	NA	NA	NA	NA	MODIFIER	NA	NA	43	43	0	62	39	23	37.1
Non-user	IOB_24	chr17	13928093	G	A	COX10-AS1	NCT	ENSG00000236088:[ENST00000602743(n.224+44496C>T)]	NA	NA	NA	NA	NA	NA	NA	NA	NA	NA	MODIFIER	NA	NA	43	43	0	62	39	23	37.1
Non-user	IOB_24	chr17	14095550	G	A	COX10	INTRONIC	ENSG00000006695:[ENST00000537334(c.277+12G>A)]	NA	NA	NA	1.87E-01	2.29E-01	NA	NA	NA	NA	NA	MODIFIER	NA	NA	404	402	0	344	201	143	41.57
Non-user	IOB_24	chr17	14095566	T	C	COX10	INTRONIC	ENSG00000006695:[ENST00000536205(c.352+28T>C)]	NA	NA	NA	2.68E-01	2.60E-01	NA	NA	NA	NA	NA	MODIFIER	NA	NA	367	363	4	298	125	173	58.05
Non-user	IOB_24	chr17	15532104	G	A	RP11-385D13.1	INTRONIC	ENSG00000251537:[ENST00000455584(c.1283+237C>T)]	NA	NA	2.00E-03	1.50E-03	6.91E-03	2.00E-03	8.20E-03	NA	NA	NA	MODIFIER	NA	NA	144	144	0	140	84	55	39.29
Non-user	IOB_24	chr17	15532104	G	A	TRIM16	MISSENSE	ENSG00000221926:[ENST00000577886(c.872C>T,tCc>tTc):ENSP00000462903(p.Ser291Phe)]	NA	NA	2.00E-03	1.50E-03	6.91E-03	2.00E-03	8.20E-03	NA	NA	NA	MODIFIER	NA	NA	144	144	0	140	84	55	39.29
Non-user	IOB_24	chr17	15532147	G	A	RP11-385D13.1	INTRONIC	ENSG00000251537:[ENST00000455584(c.1283+194C>T)]	NA	NA	1.96E-02	3.86E-02	2.50E-02	1.96E-02	3.07E-02	NA	NA	NA	MODIFIER	NA	NA	149	148	1	154	91	63	40.91
Non-user	IOB_24	chr17	15532147	G	A	TRIM16	MISSENSE	ENSG00000221926:[ENST00000577886(c.829C>T,Cgg>Tgg):ENSP00000462903(p.Arg277Trp)]	NA	NA	1.96E-02	3.86E-02	2.50E-02	1.96E-02	3.07E-02	NA	NA	NA	MODIFIER	NA	NA	149	148	1	154	91	63	40.91
Non-user	IOB_24	chr17	16246129	G	A	CENPV	EXONIC-NC	ENSG00000166582:[ENST00000472570(n.273C>T)]	NA	NA	1.40E-03	8.40E-04	5.88E-03	1.40E-03	7.20E-03	NA	NA	NA	MODIFIER	NA	NA	93	91	0	81	42	39	48.15
Non-user	IOB_24	chr17	16246129	G	A	PIGL	NCT	ENSG00000108474:[ENST00000414755(n.210+95G>A)]	NA	NA	1.40E-03	8.40E-04	5.88E-03	1.40E-03	7.20E-03	NA	NA	NA	MODIFIER	NA	NA	93	91	0	81	42	39	48.15
Non-user	IOB_24	chr17	17071068	G	T	MPRIP	MISSENSE	ENSG00000133030:[ENST00000313485(c.4269G>T,Gcc>Tcc):ENSP00000317786(p.Ala1424Ser)]	NA	NA	1.24E-02	3.49E-03	2.35E-03	1.24E-02	1.00E-03	NA	NA	NA	MODIFIER	NA	NA	56	56	0	51	19	32	62.75
Non-user	IOB_24	chr17	17699165	C	T	RAI1	MISSENSE	ENSG00000108557:[ENST00000261641(c.2903C>T,tCg>tTg):ENSP00000261641(p.Ser968Leu)]	NA	NA	NA	8.40E-06	6.07E-05	NA	NA	D	BN	NA	MODERATE	N	0	187	184	0	187	94	93	49.73
Non-user	IOB_24	chr17	18145968	G	T	LLGL1	INTRONIC	3996:[XM_005256644.1(c.3110+26G>T)]	NA	NA	3.99E-04	2.92E-03	1.93E-03	3.99E-04	1.00E-03	NA	NA	NA	MODIFIER	NA	NA	83	83	0	64	41	23	35.94
Non-user	IOB_24	chr17	18542432	T	C	TBC1D28	INTRONIC	ENSG00000189375:[ENST00000345096(c.198+56A>G)]	NA	NA	NA	NA	NA	NA	NA	NA	NA	NA	MODIFIER	NA	NA	47	47	0	10	0	10	100
Non-user	IOB_24	chr17	18542519	T	G	TBC1D28	MISSENSE	ENSG00000189375:[ENST00000345096(c.167A>C,gAg>gCg):ENSP00000339973(p.Glu56Ala)]	NA	NA	NA	4.41E-01	4.63E-01	NA	NA	NA	NA	NA	MODIFIER	NA	NA	50	49	0	28	0	28	100
Non-user	IOB_24	chr17	18544551	C	G	TBC1D28	NCT	ENSG00000189375:[ENST00000570881(n.38-134G>C)]	NA	NA	2.80E-03	3.54E-03	2.31E-03	2.80E-03	1.00E-03	NA	NA	NA	MODIFIER	NA	NA	59	59	0	34	15	17	50
Non-user	IOB_24	chr17	18682280	C	G	FBXW10	MISSENSE	ENSG00000171931:[ENST00000395667(c.2825C>G,aCc>aGc):ENSP00000379026(p.Thr942Ser)]	NA	NA	chr17*18682280*18682280*C*A,G,T*rs776045350*NA*NA*NA	2.04E-05	6.69E-05	NA	NA	NA	NA	NA	MODIFIER	NA	NA	147	146	1	188	97	91	48.4
Non-user	IOB_24	chr17	18864053	A	G	SLC5A10	INTRONIC	ENSG00000154025:[ENST00000317977(c.285+88A>G)]	NA	NA	NA	NA	NA	NA	NA	NA	NA	NA	MODIFIER	NA	NA	186	185	0	147	80	66	44.9
Non-user	IOB_24	chr17	19282231	G	A	MAPK7	SILENT	ENSG00000166484:[ENST00000308406(c.18G>A,aaG>aaA):ENSP00000311005(p.Lys6(=))]	NA	NA	NA	4.13E-05	0.00E+00	NA	NA	NA	NA	NA	MODIFIER	NA	NA	158	158	0	117	70	47	40.17
Non-user	IOB_24	chr17	19680949	C	T	ULK2	SILENT	ENSG00000083290:[ENST00000361658(c.2997G>A,ttG>ttA):ENSP00000354877(p.Leu999(=))]	NA	NA	NA	NA	NA	NA	NA	NA	NA	NA	LOW	NA	1	146	146	0	141	79	61	43.26
Non-user	IOB_24	chr17	20361493	T	C	LGALS9B	INTRONIC-SS-DNR-PRX	ENSG00000170298:[ENST00000324290(c.333+3A>G)]	NA	NA	NA	2.17E-01	2.86E-01	NA	NA	NA	NA	NA	LOW	NA	NA	98	98	0	93	67	26	27.96
Non-user	IOB_24	chr17	21204290	G	C	MAP2K3	SILENT	ENSG00000034152:[ENST00000342679(c.384G>C,ggG>ggC):ENSP00000345083(p.Gly128(=))]	NA	chr17*21204290*21204290*COSM330336*G*C*2*haematopoietic_and_lymphoid_tissue*2	NA	2.23E-01	2.57E-01	NA	NA	NA	NA	NA	LOW	NA	NA	467	395	52	364	224	122	33.52
Non-user	IOB_24	chr17	21536128	A	C	NCOR1P2	NCT	ENSG00000265019:[ENST00000584728(n.104+11A>C)]	NA	NA	NA	NA	NA	NA	NA	NA	NA	NA	MODIFIER	NA	NA	269	269	0	231	146	83	35.93
Non-user	IOB_24	chr17	25877543	A	G	KSR1	INTRONIC	ENSG00000141068:[ENST00000583370(c.-322-51A>G)]	NA	NA	3.59E-03	NA	NA	3.59E-03	1.84E-02	NA	NA	NA	MODIFIER	NA	NA	78	78	0	59	33	26	44.07
Non-user	IOB_24	chr17	28088161	G	C	RP11-82O19.1	EXONIC-NC	ENSG00000263657:[ENST00000577846(n.41G>C)]	NA	NA	5.59E-03	1.74E-02	2.03E-02	5.59E-03	1.33E-02	NA	NA	NA	MODIFIER	NA	NA	306	303	0	211	109	102	48.34
Non-user	IOB_24	chr17	28088161	G	C	SSH2	NCT	ENSG00000141298:[ENST00000577483(n.214+32751C>G)]	NA	NA	5.59E-03	1.74E-02	2.03E-02	5.59E-03	1.33E-02	NA	NA	NA	MODIFIER	NA	NA	306	303	0	211	109	102	48.34
Non-user	IOB_24	chr17	31322405	T	C	SPACA3	NCT	ENSG00000141316:[ENST00000394637(n.156-22T>C)]	NA	NA	4.33E-02	4.11E-02	9.57E-02	4.33E-02	7.16E-02	NA	NA	NA	MODIFIER	NA	NA	89	87	1	65	0	65	100
Non-user	IOB_24	chr17	32687511	G	C	CCL1	3UTR	ENSG00000108702:[ENST00000225842(c.*67C>G)]	NA	NA	3.36E-02	NA	NA	3.35E-02	3.99E-02	NA	NA	NA	MODIFIER	NA	NA	44	44	0	34	21	13	38.24
Non-user	IOB_24	chr17	32687689	A	G	CCL1	INTRONIC-SS-ACR-PRX	ENSG00000108702:[ENST00000225842(c.189-9T>C)]	NA	NA	3.36E-02	2.65E-02	4.62E-02	3.35E-02	3.99E-02	NA	NA	NA	MODIFIER	NA	NA	16	16	0	33	19	14	42.42
Non-user	IOB_24	chr17	33460453	G	A	NLE1	MISSENSE	ENSG00000073536:[ENST00000586869(c.403C>T,Cgg>Tgg):ENSP00000466588(p.Arg135Trp)]	NA	NA	NA	8.41E-06	0.00E+00	NA	NA	NA	NA	NA	MODERATE	NA	1	134	133	0	148	78	70	47.3
Non-user	IOB_24	chr17	33463486	C	T	NLE1	MISSENSE	ENSG00000073536:[ENST00000588019(c.198G>A,Ggc>Agc):ENSP00000466764(p.Gly67Ser)]	NA	chr17*33463486*33463486*COSM977829*C*T*1*endometrium*1	NA	5.78E-05	0.00E+00	NA	NA	NA	NA	NA	MODERATE	NA	1	35	35	0	46	25	20	43.48
Non-user	IOB_24	chr17	33464975	C	A	NLE1	INTRONIC	ENSG00000073536:[ENST00000588642(c.243-66G>T)]	NA	NA	1.40E-02	NA	NA	1.40E-02	9.20E-03	NA	NA	NA	MODIFIER	NA	NA	37	37	0	20	11	9	45
Non-user	IOB_24	chr17	33592194	C	G	SLFN5	MISSENSE	ENSG00000166750:[ENST00000299977(c.1963C>G,Cgt>Ggt):ENSP00000299977(p.Arg655Gly)]	NA	NA	2.52E-02	1.72E-02	4.28E-02	2.52E-02	4.40E-02	NA	NA	NA	MODIFIER	NA	NA	217	217	0	157	71	86	54.78
Non-user	IOB_24	chr17	33680783	T	C	SLFN11	SILENT	ENSG00000172716:[ENST00000308377(c.1494A>G,ctA>ctG):ENSP00000312402(p.Leu498(=))]	NA	NA	7.79E-03	2.54E-02	1.46E-02	7.79E-03	6.10E-03	NA	NA	NA	LOW	NA	NA	26	26	0	18	8	10	55.56
Non-user	IOB_24	chr17	33768937	C	T	SLFN13	MISSENSE	ENSG00000154760:[ENST00000360502(c.613G>A,Gca>Aca):ENSP00000353692(p.Ala205Thr)]	NA	NA	NA	2.65E-01	3.90E-01	NA	NA	T	BN	NA	MODERATE	N	0	58	56	2	38	26	12	31.58
Non-user	IOB_24	chr17	33769010	T	C	SLFN13	SILENT	ENSG00000154760:[ENST00000360502(c.540A>G,ctA>ctG):ENSP00000353692(p.Leu180(=))]	NA	NA	chr17*33769010*33769010*T*A,C*rs112473055*NA*NA*NA	1.07E-02	6.82E-03	NA	NA	NA	NA	NA	MODIFIER	NA	NA	111	111	0	79	65	13	16.46
Non-user	IOB_24	chr17	34072480	C	T	GAS2L2	MISSENSE	ENSG00000132139:[ENST00000587565(c.1988G>A,gGc>gAc):ENSP00000464935(p.Gly663Asp)]	NA	NA	NA	8.89E-06	9.43E-05	NA	NA	T	BN	NA	MODERATE	NA	0	257	256	0	243	109	130	53.5
Non-user	IOB_24	chr17	34183918	G	C	C17orf66	NCT	256957:[XR_243776.1(n.1154-109C>G)]	NA	NA	7.99E-04	NA	NA	7.99E-04	0.00E+00	NA	NA	NA	MODIFIER	NA	NA	80	80	0	53	28	25	47.17
Non-user	IOB_24	chr17	34183918	G	C	HEATR9	NCT	256957:[NR_135630.1(n.1346-109C>G)]	NA	NA	7.99E-04	NA	NA	7.99E-04	0.00E+00	NA	NA	NA	MODIFIER	NA	NA	80	80	0	53	28	25	47.17
Non-user	IOB_24	chr17	34183918	G	C	TAF15	INTRONIC	ENSG00000172660:[ENST00000586593(c.105-7150G>C)]	NA	NA	7.99E-04	NA	NA	7.99E-04	0.00E+00	NA	NA	NA	MODIFIER	NA	NA	80	80	0	53	28	25	47.17
Non-user	IOB_24	chr17	34340834	G	A	CCL23	SILENT	ENSG00000167236:[ENST00000591423(c.201C>T,ctC>ctT):ENSP00000465954(p.Leu67(=))]	NA	NA	1.38E-02	3.09E-02	8.85E-03	1.38E-02	1.00E-03	NA	NA	NA	LOW	NA	NA	110	107	0	64	38	26	40.62
Non-user	IOB_24	chr17	35734785	G	C	ACACA	INTRONIC	ENSG00000132142:[ENST00000416895(c.-74+31567C>G)]	NA	NA	7.19E-03	4.38E-03	3.12E-02	7.19E-03	3.58E-02	NA	NA	NA	MODIFIER	NA	NA	73	68	5	33	18	14	42.42
Non-user	IOB_24	chr17	35734785	G	C	C17orf78	INTRONIC	ENSG00000167230:[ENST00000300618(c.59-32G>C)]	NA	NA	7.19E-03	4.38E-03	3.12E-02	7.19E-03	3.58E-02	NA	NA	NA	MODIFIER	NA	NA	73	68	5	33	18	14	42.42
Non-user	IOB_24	chr17	35748727	C	A	ACACA	INTRONIC	ENSG00000132142:[ENST00000451642(c.-748+17625G>T)]	NA	NA	NA	NA	NA	NA	NA	NA	NA	NA	MODIFIER	NA	NA	223	223	0	186	111	75	40.32
Non-user	IOB_24	chr17	35748727	C	A	C17orf78	3UTR	ENSG00000167230:[ENST00000300618(c.*49C>A)]	NA	NA	NA	NA	NA	NA	NA	NA	NA	NA	MODIFIER	NA	NA	223	223	0	186	111	75	40.32
Non-user	IOB_24	chr17	36064897	A	G	HNF1B	INTRONIC	ENSG00000108753:[ENST00000225893(c.1339+27T>C)]	NA	NA	4.67E-02	9.28E-02	7.82E-02	4.67E-02	5.93E-02	NA	NA	NA	MODIFIER	NA	NA	229	228	0	168	95	72	42.86
Non-user	IOB_24	chr17	36352489	A	C	LOC440434	EXONIC-NC	440434:[NR_036750.2(n.1384T>G)]	NA	chr17*36352489*36352489*COSM1493915*A*C*1*kidney*1|chr17*36352489*36352489*COSM1493914*A*C*1*kidney*1	chr17*36352489*36352491*AAT*A*rs750865779*NA*NA*NA	2.90E-01	2.31E-01	NA	NA	D	PrD	NA	MODERATE	NA	NA	279	275	1	275	216	58	21.09
Non-user	IOB_24	chr17	36352489	A	C	RP11-1407O15.2	MISSENSE	ENSG00000174093:[ENST00000523258(c.360T>G,tTt>tGt):ENSP00000429970(p.Phe121Cys)]	NA	chr17*36352489*36352489*COSM1493915*A*C*1*kidney*1|chr17*36352489*36352489*COSM1493914*A*C*1*kidney*1	chr17*36352489*36352491*AAT*A*rs750865779*NA*NA*NA	2.90E-01	2.31E-01	NA	NA	D	PrD	NA	MODERATE	NA	NA	279	275	1	275	216	58	21.09
Non-user	IOB_24	chr17	36352489	A	C	TBC1D3	5UTR	ENSG00000197681:[ENST00000537432(c.-130T>G)]	NA	chr17*36352489*36352489*COSM1493915*A*C*1*kidney*1|chr17*36352489*36352489*COSM1493914*A*C*1*kidney*1	chr17*36352489*36352491*AAT*A*rs750865779*NA*NA*NA	2.90E-01	2.31E-01	NA	NA	D	PrD	NA	MODERATE	NA	NA	279	275	1	275	216	58	21.09
Non-user	IOB_24	chr17	36361992	C	T	LOC440434	NCT	440434:[NR_036750.2(n.582-188G>A)]	NA	NA	NA	NA	NA	NA	NA	NA	NA	NA	MODIFIER	NA	NA	129	128	1	88	54	34	38.64
Non-user	IOB_24	chr17	36361992	C	T	RP11-1407O15.2	INTRONIC	ENSG00000174093:[ENST00000544906(c.76-188G>A)]	NA	NA	NA	NA	NA	NA	NA	NA	NA	NA	MODIFIER	NA	NA	129	128	1	88	54	34	38.64
Non-user	IOB_24	chr17	36384868	C	G	LOC440434	NCT	440434:[NR_036750.2(n.438-9691G>C)]	NA	NA	NA	NA	NA	NA	NA	NA	NA	NA	MODIFIER	NA	NA	163	162	0	102	80	21	20.59
Non-user	IOB_24	chr17	36384868	C	G	RP11-1407O15.2	INTRONIC	ENSG00000174093:[ENST00000523089(c.315-9691G>C)]	NA	NA	NA	NA	NA	NA	NA	NA	NA	NA	MODIFIER	NA	NA	163	162	0	102	80	21	20.59
Non-user	IOB_24	chr17	36484916	T	C	GPR179	SILENT	ENSG00000188888:[ENST00000342292(c.4536A>G,ggA>ggG):ENSP00000345060(p.Gly1512(=))]	NA	NA	NA	NA	NA	NA	NA	NA	NA	NA	LOW	NA	NA	328	327	0	283	149	133	47
Non-user	IOB_24	chr17	36492957	G	A	GPR179	SILENT	ENSG00000188888:[ENST00000342292(c.1131C>T,gcC>gcT):ENSP00000345060(p.Ala377(=))]	NA	NA	NA	NA	NA	NA	NA	NA	NA	NA	LOW	NA	NA	117	117	0	106	55	51	48.11
Non-user	IOB_24	chr17	36718301	G	A	SRCIN1	SILENT	ENSG00000017373:[ENST00000578925(c.1200C>T,agC>agT):ENSP00000462070(p.Ser400(=))]	NA	NA	9.59E-03	3.62E-02	4.36E-02	9.58E-03	2.66E-02	NA	NA	NA	MODIFIER	NA	NA	145	145	0	145	71	73	50.34
Non-user	IOB_24	chr17	37815024	C	T	STARD3	MISSENSE	ENSG00000131748:[ENST00000578577(c.530C>T,cCg>cTg):ENSP00000464472(p.Pro177Leu)]	NA	NA	4.79E-03	6.76E-03	1.88E-02	4.79E-03	1.12E-02	NA	NA	NA	MODIFIER	NA	NA	129	129	0	110	65	44	40
Non-user	IOB_24	chr17	38130148	G	A	GSDMA	INTRONIC	ENSG00000167914:[ENST00000301659(c.751+65G>A)]	NA	NA	3.40E-03	NA	NA	3.39E-03	1.43E-02	NA	NA	NA	MODIFIER	NA	NA	60	58	0	55	29	25	45.45
Non-user	IOB_24	chr17	38172797	C	A	CSF3	SILENT	ENSG00000108342:[ENST00000583218(c.255C>A,gcC>gcA):ENSP00000464421(p.Ala85(=))]	NA	NA	chr17*38172797*38172797*C*T*rs769044135*NA*NA*NA	NA	NA	NA	NA	NA	NA	NA	MODIFIER	NA	NA	273	272	0	205	120	83	40.49

Supplementary Table 4. List of somatic single nucleotide variants identified using whole exome sequencing of OSCC patients

Cohort	Sample	Chromosome	Genomic position	Reference allele	Alternate allele	Genesymbol	Variant type	Variant description	OncoMD	COSMIC	dbSNP	ExAC	ExAC_SAS	1000G	1000G_SAS	SIFT	Polyphen2	CONDEL	VEP	LRT	PhastCon7Way	Total reads in Normal	Reference read support in Normal	Alternative read support in Normal	Total reads in Tumor	Reference read support in Tumor	Alternate read support in Tumor	Alternate allele frequency in Tumor
Non-user	IOB_24	chr17	39020028	T	C	KRT12	MISSENSE	ENSG00000187242:[ENST00000251643(c.896A>G,aAt>aGt):ENSP00000251643(p.Asn299Ser)]	NA	NA	5.99E-04	3.23E-04	1.76E-03	5.99E-04	3.10E-03	T	BN	NA	MODERATE	N	0.894	34	34	0	36	18	17	47.22
Non-user	IOB_24	chr17	39134475	A	G	KRT40	MISSENSE	ENSG00000204889:[ENST00000377755(c.1270T>C,Tgc>Cgc):ENSP00000366984(p.Cys424Arg)]	NA	NA	4.19E-03	2.29E-03	1.67E-02	4.19E-03	2.15E-02	NA	NA	NA	MODERATE	NA	NA	108	106	0	66	37	29	43.94
Non-user	IOB_24	chr17	39406419	T	C	KRTAP9-4	SILENT	ENSG00000241595:[ENST00000334109(c.447T>C,tgT>tgC):ENSP00000334922(p.Cys149(=))]	NA	NA	NA	1.15E-01	6.87E-02	NA	NA	NA	NA	NA	LOW	NA	NA	132	132	0	156	104	50	32.05
Non-user	IOB_24	chr17	39406427	T	C	KRTAP9-4	MISSENSE	ENSG00000241595:[ENST00000334109(c.455T>C,tTt>tCt):ENSP00000334922(p.Phe152Ser)]	NA	NA	NA	1.15E-01	6.87E-02	NA	NA	T	BN	NA	MODERATE	NA	NA	130	129	0	154	100	51	33.12
Non-user	IOB_24	chr17	39502448	T	C	KRT33A	MISSENSE	ENSG00000006059:[ENST00000007735(c.1138A>G,Aag>Gag):ENSP00000007735(p.Lys380Glu)]	NA	NA	chr17*39502448*39502448*T*A*rs756630645*NA*NA*NA	NA	NA	NA	NA	T	BN	NA	MODERATE	N	0.017	165	163	0	130	67	61	46.92
Non-user	IOB_24	chr17	40175902	G	A	NKIRAS2	MISSENSE	28511:[XM_005257252.1(c.448G>A,Gga>Aga):XP_005257309.1(p.Gly150Arg)]	NA	NA	1.06E-02	1.71E-02	2.80E-02	1.06E-02	2.25E-02	NA	NA	NA	MODIFIER	NA	NA	193	192	0	147	84	63	42.86
Non-user	IOB_24	chr17	40253855	C	T	DHX58	MISSENSE	ENSG00000108771:[ENST00000251642(c.1961G>A,cGc>cAc):ENSP00000251642(p.Arg654His)]	NA	NA	2.60E-03	3.95E-03	2.42E-03	2.60E-03	3.10E-03	NA	NA	NA	MODIFIER	NA	NA	139	139	0	95	46	49	51.58
Non-user	IOB_24	chr17	40263400	C	T	DHX58	MISSENSE	ENSG00000108771:[ENST00000251642(c.284G>A,cGg>cAg):ENSP00000251642(p.Arg95Gln)]	NA	NA	2.88E-02	4.36E-02	5.41E-02	2.88E-02	5.01E-02	NA	NA	NA	MODIFIER	NA	NA	139	139	0	131	66	65	49.62
Non-user	IOB_24	chr17	40312349	G	A	KCNH4	SILENT	ENSG00000089558:[ENST00000264661(c.2763C>T,tcC>tcT):ENSP00000264661(p.Ser921(=))]	NA	NA	2.20E-03	4.36E-03	2.97E-03	2.20E-03	3.10E-03	NA	NA	NA	LOW	NA	NA	250	250	0	160	72	88	55
Non-user	IOB_24	chr17	40841070	C	T	CNTNAP1	INTRONIC	8506:[XM_005257748.1(c.1400+5C>T)]	NA	NA	1.56E-02	3.32E-02	7.48E-03	1.56E-02	3.10E-03	NA	NA	NA	LOW	NA	NA	126	126	0	86	47	39	45.35
Non-user	IOB_24	chr17	40841070	C	T	CTD-3193K9.3	NCT	ENSG00000267765:[ENST00000592440(n.364-5510G>A)]	NA	NA	1.56E-02	3.32E-02	7.48E-03	1.56E-02	3.10E-03	NA	NA	NA	LOW	NA	NA	126	126	0	86	47	39	45.35
Non-user	IOB_24	chr17	42837103	A	G	ADAM11	SILENT	ENSG00000073670:[ENST00000200557(c.75A>G,caA>caG):ENSP00000200557(p.Gln25(=))]	NA	NA	3.40E-03	1.68E-03	9.00E-03	3.39E-03	1.53E-02	NA	NA	NA	MODIFIER	NA	NA	91	91	0	84	32	52	61.9
Non-user	IOB_24	chr17	42925539	T	C	HIGD1B	MISSENSE	ENSG00000131097:[ENST00000253410(c.19T>C,Tgg>Cgg):ENSP00000253410(p.Trp7Arg)]	NA	NA	3.20E-03	2.23E-03	1.50E-02	3.19E-03	1.64E-02	NA	NA	NA	MODIFIER	NA	NA	38	38	0	23	17	6	26.09
Non-user	IOB_24	chr17	43227079	G	A	HEXIM1	SILENT	ENSG00000186834:[ENST00000332499(c.522G>A,gaG>gaA):ENSP00000328773(p.Glu174(=))]	NA	NA	1.80E-03	1.31E-03	8.87E-03	1.80E-03	9.20E-03	NA	NA	NA	LOW	NA	1	184	182	0	106	39	67	63.21
Non-user	IOB_24	chr17	44360744	T	C	ARL17B	INTRONIC	ENSG00000228696:[ENST00000570618(c.*22-7960A>G)]	NA	NA	NA	NA	NA	NA	NA	NA	NA	NA	MODIFIER	NA	NA	160	159	0	214	174	40	18.69
Non-user	IOB_24	chr17	44360744	T	C	LRRC37A	INTRONIC	9884:[XM_005257887.1(c.27-16416T>C)]	NA	NA	NA	NA	NA	NA	NA	NA	NA	NA	MODIFIER	NA	NA	160	159	0	214	174	40	18.69
Non-user	IOB_24	chr17	45481886	G	A	EFCAB13	INTRONIC	ENSG00000178852:[ENST00000331493(c.2162-42G>A)]	NA	NA	5.99E-04	9.78E-04	6.87E-03	5.99E-04	3.10E-03	NA	NA	NA	MODIFIER	NA	NA	144	143	0	109	48	60	55.05
Non-user	IOB_24	chr17	45776072	C	G	TBKBP1	MISSENSE	ENSG00000198933:[ENST00000361722(c.565C>G,Ccc>Gcc):ENSP00000354777(p.Pro189Ala)]	NA	NA	9.78E-03	1.38E-02	5.83E-02	9.78E-03	3.78E-02	T_lc	BN	NA	MODERATE	NA	NA	134	134	0	149	71	77	51.68
Non-user	IOB_24	chr17	46002410	C	A	AC003665.1	NCT	ENSG00000234494:[ENST00000451140(n.76-6473G>T)]	NA	NA	NA	NA	NA	NA	NA	NA	NA	NA	LOW	NA	1	263	263	0	184	177	5	2.72
Non-user	IOB_24	chr17	46002410	C	A	LOC100506325	NCT	100506325:[XR_243614.1(n.119-6473G>T)]	NA	NA	NA	NA	NA	NA	NA	NA	NA	NA	LOW	NA	1	263	263	0	184	177	5	2.72
Non-user	IOB_24	chr17	46002410	C	A	SP2	SILENT	ENSG00000167182:[ENST00000376741(c.1498C>A,Cgg>Agg):ENSP00000365931(p.Arg500(=))]	NA	NA	NA	NA	NA	NA	NA	NA	NA	NA	LOW	NA	1	263	263	0	184	177	5	2.72
Non-user	IOB_24	chr17	46002410	C	A	SP2-AS1	NCT	100506325:[NR_103856.1(n.70-6473G>T)]	NA	NA	NA	NA	NA	NA	NA	NA	NA	NA	LOW	NA	1	263	263	0	184	177	5	2.72
Non-user	IOB_24	chr17	46022065	G	A	PNPO	MISSENSE	ENSG00000108439:[ENST00000534893(c.62G>A,cGa>cAa):ENSP00000437480(p.Arg21Gln)]	NA	NA	2.98E-02	5.58E-02	6.00E-02	2.98E-02	4.60E-02	D	BN	NA	MODERATE	D	1	61	61	0	33	23	10	30.3
Non-user	IOB_24	chr17	46023694	G	A	PNPO	SILENT	ENSG00000108439:[ENST00000225573(c.552G>A,ctG>ctA):ENSP00000225573(p.Leu184(=))]	NA	NA	4.21E-02	6.97E-02	9.53E-02	4.21E-02	6.85E-02	NA	NA	NA	LOW	NA	NA	71	71	0	48	18	29	60.42
Non-user	IOB_24	chr17	46023694	G	A	RP11-6N17.9	EXONIC-NC	ENSG00000263798:[ENST00000582262(n.81G>A)]	NA	NA	4.21E-02	6.97E-02	9.53E-02	4.21E-02	6.85E-02	NA	NA	NA	LOW	NA	NA	71	71	0	48	18	29	60.42
Non-user	IOB_24	chr17	46023954	A	G	PNPO	INTRONIC	55163:[XM_005257500.1(c.378-26A>G)]	NA	NA	4.21E-02	6.97E-02	9.52E-02	4.21E-02	6.85E-02	NA	NA	NA	MODIFIER	NA	NA	112	112	0	69	35	33	47.83
Non-user	IOB_24	chr17	46023954	A	G	RP11-6N17.9	NCT	ENSG00000263798:[ENST00000582262(n.146+195A>G)]	NA	NA	4.21E-02	6.97E-02	9.52E-02	4.21E-02	6.85E-02	NA	NA	NA	MODIFIER	NA	NA	112	112	0	69	35	33	47.83
Non-user	IOB_24	chr17	46115079	C	T	COPZ2	MISSENSE	ENSG00000005243:[ENST00000006101(c.59G>A,cGg>cAg):ENSP00000006101(p.Arg20Gln)]	NA	NA	NA	6.25E-01	NA	NA	NA	NA	NA	NA	MODIFIER	NA	NA	39	39	0	24	9	15	62.5
Non-user	IOB_24	chr17	46441132	G	A	SKAP1	INTRONIC	ENSG00000141293:[ENST00000336915(c.178+19C>T)]	NA	NA	2.32E-02	3.53E-02	3.05E-02	2.32E-02	3.37E-02	NA	NA	NA	MODIFIER	NA	NA	178	177	0	110	54	55	50
Non-user	IOB_24	chr17	46939658	C	G	CALCOCO2	MISSENSE	ENSG00000136436:[ENST00000509507(c.1228C>G,Ccc>Gcc):ENSP00000424352(p.Pro410Ala)]	NA	NA	chr17*46939658*46939658*C*A,G*rs10278*NA*NA*NA	2.99E-01	2.47E-01	NA	NA	T	BN	NA	MODERATE	N	0	238	237	0	172	98	73	42.44
Non-user	IOB_24	chr17	47284787	T	A	GNGT2	5UTR	ENSG00000167083:[ENST00000300406(c.-3A>T)]	NA	NA	4.19E-03	7.15E-03	8.48E-03	4.19E-03	1.33E-02	NA	NA	NA	MODIFIER	NA	NA	94	94	0	79	44	35	44.3
Non-user	IOB_24	chr17	47329902	G	C	RP1-62O9.3	EXONIC-NC	ENSG00000177369:[ENST00000511008(n.355G>C)]	NA	NA	2.00E-03	4.12E-03	8.14E-03	2.00E-03	1.02E-02	NA	NA	NA	MODIFIER	NA	NA	228	227	0	142	76	65	45.77
Non-user	IOB_24	chr17	48226786	C	G	PPP1R9B	MISSENSE	ENSG00000108819:[ENST00000316878(c.1087G>C,Ggg>Cgg):ENSP00000475417(p.Gly363Arg)]	NA	NA	3.40E-03	3.49E-03	1.18E-02	3.39E-03	1.53E-02	NA	NA	NA	MODIFIER	NA	NA	206	203	0	170	89	79	46.47
Non-user	IOB_24	chr17	48458239	G	A	EME1	MISSENSE	ENSG00000154920:[ENST00000393271(c.1691G>A,aGg>aAg):ENSP00000376952(p.Arg564Lys)]	NA	NA	2.00E-03	8.57E-04	6.30E-03	2.00E-03	1.02E-02	T	BN	NA	MODERATE	D	1	127	127	0	114	58	55	48.25
Non-user	IOB_24	chr17	48458239	G	A	LRRC59	INTRONIC	ENSG00000108829:[ENST00000503118(c.17+4240C>T)]	NA	NA	2.00E-03	8.57E-04	6.30E-03	2.00E-03	1.02E-02	T	BN	NA	MODERATE	D	1	127	127	0	114	58	55	48.25
Non-user	IOB_24	chr17	48601096	C	T	MYCBPAP	MISSENSE	ENSG00000136449:[ENST00000488432(c.16C>T,gCg>gTg):ENSP00000464992(p.Ala6Val)]	NA	NA	1.14E-02	1.13E-02	1.96E-02	1.14E-02	1.74E-02	D	PrD	NA	MODERATE	N	0.999	131	131	0	113	45	67	59.29
Non-user	IOB_24	chr17	48703955	G	A	CACNA1G	MISSENSE	ENSG00000006283:[ENST00000510366(c.6542G>A,aGc>aAc):ENSP00000426814(p.Ser2181Asn)]	NA	NA	3.99E-04	6.70E-04	4.64E-03	3.99E-04	2.00E-03	T	BN	NA	MODERATE	N	1	188	179	1	169	74	86	50.89
Non-user	IOB_24	chr17	53027479	C	G	TOM1L1	SILENT	ENSG00000141198:[ENST00000575882(c.1362C>G,gtC>gtG):ENSP00000460823(p.Val454(=))]	NA	NA	9.59E-03	5.94E-03	4.32E-02	9.58E-03	4.81E-02	NA	NA	NA	LOW	NA	NA	180	180	0	176	77	97	55.11
Non-user	IOB_24	chr17	53042010	G	A	COX11	NCT	1353:[NR_027942.1(n.579+48C>T)]	NA	NA	9.59E-03	6.35E-03	4.59E-02	9.58E-03	4.81E-02	NA	NA	NA	MODIFIER	NA	NA	38	38	0	22	15	7	31.82
Non-user	IOB_24	chr17	55752389	G	A	MSI2	MISSENSE	ENSG00000153944:[ENST00000416426(c.835G>A,Gcc>Acc):ENSP00000414671(p.Ala279Thr)]	NA	NA	3.00E-03	9.94E-03	3.10E-03	3.00E-03	1.00E-03	NA	NA	NA	MODIFIER	NA	NA	172	172	0	168	74	92	54.76
Non-user	IOB_24	chr17	55944707	T	C	CUEDC1	NCT	404093:[NR_073495.1(n.1812+11A>G)]	NA	NA	4.59E-03	6.23E-03	1.62E-02	4.59E-03	2.25E-02	NA	NA	NA	MODIFIER	NA	NA	119	117	0	95	33	60	63.16
Non-user	IOB_24	chr17	55946562	G	T	CUEDC1	NCT-SS-PRX	404093:[NR_073495.1(n.1588-8C>A)]	NA	NA	2.60E-02	6.83E-02	1.94E-02	2.60E-02	1.33E-02	NA	NA	NA	LOW	NA	NA	147	146	1	100	53	47	47
Non-user	IOB_24	chr17	56356626	G	T	MPO	INTRONIC	ENSG00000005381:[ENST00000225275(c.678+32C>A)]	NA	NA	5.59E-03	1.02E-02	2.90E-02	5.59E-03	2.86E-02	NA	NA	NA	MODIFIER	NA	NA	141	140	0	112	63	49	43.75
Non-user	IOB_24	chr17	56400299	C	T	BZRAP1	INTRONIC	ENSG00000005379:[ENST00000268893(c.1014+12G>A)]	NA	NA	4.99E-03	3.36E-03	2.06E-02	4.99E-03	2.15E-02	NA	NA	NA	MODIFIER	NA	NA	211	211	0	176	94	80	45.45
Non-user	IOB_24	chr17	58009086	G	A	RPS6KB1	NCT-SS-PRX	ENSG00000108443:[ENST00000587622(n.403+3G>A)]	NA	NA	NA	8.55E-06	6.27E-05	NA	NA	NA	NA	NA	LOW	NA	NA	80	79	1	74	39	35	47.3
Non-user	IOB_24	chr17	58740529	C	T	PPM1D	SILENT	ENSG00000170836:[ENST00000305921(c.1434C>T,tgC>tgT):ENSP00000306682(p.Cys478(=))]	NA	chr17*58740529*58740529*COSM246745*C*T*1*prostate*1|chr17*58740529*58740529*COSM98068*C*A*2*upper_aerodigestive_tract,breast*1,1	3.99E-03	3.12E-03	2.22E-02	3.99E-03	1.94E-02	NA	NA	NA	LOW	NA	0.981	290	289	0	215	109	104	48.37
Non-user	IOB_24	chr17	60042534	T	G	MED13	SILENT	ENSG00000108510:[ENST00000397786(c.4677A>C,ccA>ccC):ENSP00000380888(p.Pro1559(=))]	NA	NA	1.40E-03	4.22E-04	3.03E-03	1.40E-03	7.20E-03	NA	NA	NA	LOW	NA	NA	356	353	1	302	151	147	48.68
Non-user	IOB_24	chr17	60505123	G	A	METTL2A	NCT	ENSG00000087995:[ENST00000333483(n.793-26G>A)]	NA	NA	3.28E-02	4.14E-02	9.40E-02	3.27E-02	1.02E-01	NA	NA	NA	MODIFIER	NA	NA	172	171	0	133	59	74	55.64
Non-user	IOB_24	chr17	61466842	G	A	AC015923.1	NCT	ENSG00000226797:[ENST00000431604(n.160-1393C>T)]	NA	NA	5.99E-04	3.60E-04	2.39E-03	5.99E-04	3.10E-03	NA	NA	NA	MODIFIER	NA	NA	105	105	0	101	57	43	42.57
Non-user	IOB_24	chr17	61466842	G	A	TANC2	SILENT	ENSG00000170921:[ENST00000389520(c.2766G>A,ctG>ctA):ENSP00000374171(p.Leu922(=))]	NA	NA	5.99E-04	3.60E-04	2.39E-03	5.99E-04	3.10E-03	NA	NA	NA	MODIFIER	NA	NA	105	105	0	101	57	43	42.57
Non-user	IOB_24	chr17	61512586	A	G	CYB561	MISSENSE	ENSG00000008283:[ENST00000582997(c.445T>C,Ttc>Ctc):ENSP00000464215(p.Phe149Leu)]	NA	NA	5.99E-04	2.75E-04	1.84E-03	5.99E-04	3.10E-03	T	BN	NA	MODERATE	NA	NA	111	111	0	98	55	43	43.88
Non-user	IOB_24	chr17	61512592	A	G	CYB561	MISSENSE	ENSG00000008283:[ENST00000360793(c.418T>C,Ttc>Ctc):ENSP00000354028(p.Phe140Leu)]	NA	NA	5.99E-04	2.70E-04	1.86E-03	5.99E-04	3.10E-03	NA	NA	NA	MODIFIER	NA	NA	106	104	1	94	49	44	46.81
Non-user	IOB_24	chr17	61568621	C	T	ACE	SILENT	ENSG00000159640:[ENST00000582761(c.559C>T,Ctg>Ttg):ENSP00000462909(p.Leu187(=))]	NA	NA	1.54E-02	7.43E-03	5.37E-02	1.54E-02	7.87E-02	NA	NA	NA	MODIFIER	NA	NA	122	122	0	97	33	63	64.95
Non-user	IOB_24	chr17	61744268	T	C	MAP3K3	INTRONIC	ENSG00000198909:[ENST00000577597(c.*207-32T>C)]	NA	NA	2.00E-04	1.65E-05	1.21E-04	2.00E-04	1.00E-03	NA	NA	NA	MODIFIER	NA	NA	53	53	0	39	19	19	48.72
Non-user	IOB_24	chr17	62290699	G	A	TEX2	SILENT	ENSG00000136478:[ENST00000258991(c.879C>T,cgC>cgT):ENSP00000258991(p.Arg293(=))]	NA	NA	4.71E-02	3.18E-02	9.30E-02	4.71E-02	1.00E-01	NA	NA	NA	LOW	NA	NA	238	237	0	188	100	88	46.81
Non-user	IOB_24	chr17	62525542	A	T	CEP95	MISSENSE	90799:[XM_005257783.1(c.1433A>T,cAc>cTc):XP_005257840.1(p.His478Leu)]	NA	NA	NA	NA	NA	NA	NA	NA	NA	NA	MODIFIER	NA	NA	95	94	0	81	49	31	38.27
Non-user	IOB_24	chr17	64222164	C	T	APOH	MISSENSE	ENSG00000091583:[ENST00000581797(c.140G>A,aGt>aAt):ENSP00000463553(p.Ser47Asn)]	NA	NA	2.90E-02	4.06E-02	5.60E-02	2.90E-02	4.70E-02	T	BN	NA	MODERATE	NA	NA	184	184	0	167	103	64	38.32
Non-user	IOB_24	chr17	65199608	T	A	HELZ	INTRONIC	ENSG00000198265:[ENST00000358691(c.248-49A>T)]	NA	NA	NA	NA	NA	NA	NA	NA	NA	NA	MODIFIER	NA	NA	136	136	0	105	63	42	40
Non-user	IOB_24	chr17	72245471	G	A	TTYH2	INTRONIC	ENSG00000141540:[ENST00000269346(c.875-12G>A)]	NA	NA	1.04E-02	1.21E-02	4.28E-02	1.04E-02	3.27E-02	NA	NA	NA	MODIFIER	NA	NA	255	254	0	226	126	99	43.81
Non-user	IOB_24	chr17	72733265	C	T	RAB37	INTRONIC	ENSG00000172794:[ENST00000528438(c.12+395C>T)]	NA	NA	NA	8.45E-05	4.99E-04	NA	NA	NA	NA	NA	MODIFIER	NA	NA	127	127	0	131	71	60	45.8
Non-user	IOB_24	chr17	72816013	G	A	TMEM104	INTRONIC	ENSG00000109066:[ENST00000335464(c.730+31G>A)]	NA	NA	3.51E-02	1.49E-02	3.43E-02	3.51E-02	4.50E-02	NA	NA	NA	MODIFIER	NA	NA	98	98	0	98	52	46	46.94
Non-user	IOB_24	chr17	72846368	C	T	GRIN2C	MISSENSE	ENSG00000161509:[ENST00000293190(c.1468G>A,Gta>Ata):ENSP00000293190(p.Val490Ile)]	NA	NA	6.79E-03	4.45E-03	2.14E-02	6.79E-03	2.86E-02	T	BN	NA	MODERATE	D	0.965	292	291	0	280	162	117	41.79
Non-user	IOB_24	chr17	72926387	C	T	OTOP2	SILENT	ENSG00000183034:[ENST00000331427(c.657C>T,aaC>aaT):ENSP00000332528(p.Asn219(=))]	NA	NA	2.00E-04	3.27E-04	1.29E-04	2.00E-04	0.00E+00	NA	NA	NA	LOW	NA	0.007	161	161	0	128	69	59	46.09
Non-user	IOB_24	chr17	72943418	G	A	OTOP3	MISSENSE	ENSG00000182938:[ENST00000328801(c.1468G>A,Gag>Aag):ENSP00000328090(p.Glu490Lys)]	NA	NA	7.59E-03	1.01E-02	1.06E-02	7.59E-03	4.10E-03	NA	NA	NA	MODERATE	NA	1	258	258	0	211	106	105	49.76
Non-user	IOB_24	chr17	73235478	C	G	GGA3	SILENT	ENSG00000125447:[ENST00000580646(c.142G>C,gtG>gtC):ENSP00000464574(p.Val48(=))]	NA	NA	5.59E-03	1.34E-02	8.98E-03	5.59E-03	3.10E-03	NA	NA	NA	LOW	NA	NA	193	193	0	150	89	61	40.67
Non-user	IOB_24	chr17	73484845	C	T	KIAA0195	SILENT	ENSG00000177728:[ENST00000314256(c.618C>T,gaC>gaT):ENSP00000313885(p.Asp206(=))]	NA	NA	2.40E-03	3.25E-03	1.42E-03	2.40E-03	2.00E-03	NA	NA	NA	MODIFIER	NA	NA	112	112	0	89	46	40	44.94
Non-user	IOB_24	chr17	73484845	C	T	TMEM94	INTRONIC	9772:[XM_005257866.1(c.-13-502C>T)]	NA	NA	2.40E-03	3.25E-03	1.42E-03	2.40E-03	2.00E-03	NA	NA	NA	MODIFIER	NA	NA	112	112	0	89	46	40	44.94
Non-user	IOB_24	chr17	73567208	G	T	LLGL2	INTRONIC	ENSG00000073350:[ENST00000578638(c.*908+27G>T)]	NA	NA	3.06E-02	4.78E-02	9.93E-02	3.06E-02	9.10E-02	NA	NA	NA	MODIFIER	NA	NA	208	206	0	134	64	68	50.75
Non-user	IOB_24	chr17	73702135	G	A	SAP30BP	SILENT	ENSG00000161526:[ENST00000583536(c.757G>A,acG>acA):ENSP00000463758(p.Thr253(=))]	NA	NA	2.36E-02	4.18E-02	7.46E-02	2.36E-02	5.42E-02	NA	NA	NA	MODIFIER	NA	NA	235	235	0	182	77	105	57.69
Non-user	IOB_24	chr17	73738809	C	T	ITGB4	MISSENSE	ENSG00000132470:[ENST00000200181(c.2929C>T,Cgc>Tgc):ENSP00000200181(p.Arg977Cys)]	NA	NA	1.40E-03	2.85E-03	6.47E-03	1.40E-03	2.00E-03	D	PrD	NA	MODERATE	D	1	255	254	0	209	111	97	46.41
Non-user	IOB_24	chr17	73870919	C	T	TRIM47	MISSENSE	ENSG00000132481:[ENST00000587339(c.848G>A,tGc>tAc):ENSP00000465010(p.Cys283Tyr)]	NA	NA	NA	5.79E-05	6.06E-05	NA	NA	D_lc	PrD	NA	MODERATE	NA	NA	435	435	0	376	187	188	50
Non-user	IOB_24	chr17	74075587	T	C	ZACN	SILENT	ENSG00000186919:[ENST00000334586(c.165T>C,aaT>aaC):ENSP00000334854(p.Asn55(=))]	NA	NA	3.00E-03	1.81E-03	1.31E-02	3.00E-03	1.43E-02	NA	NA	NA	MODIFIER	NA	NA	279	277	1	222	107	114	51.35
Non-user	IOB_24	chr17	74163247	C	T	RNF157	INTRONIC	ENSG00000141576:[ENST00000269391(c.444-40G>A)]	NA	NA	4.95E-02	6.72E-02	1.03E-01	4.95E-02	1.05E-01	NA	NA	NA	MODIFIER	NA	NA	124	124	0	122	55	67	54.92
Non-user	IOB_24	chr17	74287141	C	T	QRICH2	MISSENSE	ENSG00000129646:[ENST00000447564(c.193G>A,Ggc>Agc):ENSP00000394461(p.Gly65Ser)]	NA	NA	4.39E-03	2.46E-03	1.81E-02	4.39E-03	2.25E-02	D	PrD	NA	MODERATE	NA	NA	86	86	0	85	53	32	37.65
Non-user	IOB_24	chr17	74289648	G	A	QRICH2	MISSENSE	ENSG00000129646:[ENST00000262765(c.662C>T,cCa>cTa):ENSP00000262765(p.Pro221Leu)]	NA	NA	6.19E-03	1.45E-02	2.42E-03	6.19E-03	1.00E-03	NA	NA	NA	MODIFIER	NA	NA	377	376	0	271	137	133	49.08
Non-user	IOB_24	chr17	74290008	G	T	QRICH2	MISSENSE	ENSG00000129646:[ENST00000262765(c.302C>A,cCa>cAa):ENSP00000262765(p.Pro101Gln)]	NA	NA	7.39E-03	1.60E-02	2.85E-03	7.39E-03	2.00E-03	D	PoD	NA	MODERATE	NA	NA	194	194	0	179	90	89	49.72
Non-user	IOB_24	chr17	74300931	G	A	QRICH2	INTRONIC	ENSG00000129646:[ENST00000262765(c.96+32C>T)]	NA	NA	6.99E-03	1.59E-02	2.79E-03	6.99E-03	2.00E-03	NA	NA	NA	MODIFIER	NA	NA	54	54	0	49	19	30	61.22
Non-user	IOB_24	chr17	74468126	C	A	RHBDF2	SILENT	ENSG00000129667:[ENST00000389760(c.2073G>T,ccG>ccT):ENSP00000374410(p.Pro691(=))]	NA	NA	chr17*74468126*74468126*C*T*rs374520556*NA*NA*NA	NA	NA	NA	NA	NA	NA	NA	MODIFIER	NA	NA	60	60	0	57	52	5	8.77

Patel *et al.* , 2021. Whole-exome sequencing analysis of oral squamous cell carcinoma delineated by tobacco usage habits
Supplementary Table 4. List of somatic single nucleotide variants identified using whole exome sequencing of OSCC patients

Cohort	Sample	Chromosome	Genomic position	Reference allele	Alternate allele	Genesymbol	Variant type	Variant description	OncoMD	COSMIC	dbSNP	ExAC	ExAC_SAS	1000G	1000G_SAS	SIFT	Polyphen2	CONDEL	VEP	LRT	PhastCon7Way	Total reads in Normal	Reference read support in Normal	Alternative read support in Normal	Total reads in Tumor	Reference read support in Tumor	Alternate read support in Tumor	Alternate allele frequency in Tumor
Non-user	IOB_24	chr17	74470194	C	A	RHBDF2	MISSENSE	ENSG00000129667:[ENST00000313080(c.1582G>T,Gat>Tat):ENSP00000322775(p.Asp528Tyr)]	NA	NA	1.40E-03	3.80E-03	3.75E-03	1.40E-03	0.00E+00	D	BN	NA	MODERATE	N	0.05	152	152	0	125	58	67	53.6
Non-user	IOB_24	chr17	74524706	T	C	CYGB	INTRONIC	ENSG00000161544:[ENST00000589145(c.345-13A>G)]	NA	NA	2.00E-03	9.05E-03	1.51E-03	2.00E-03	0.00E+00	NA	NA	NA	MODIFIER	NA	NA	60	59	0	43	16	27	62.79
Non-user	IOB_24	chr17	74524706	T	C	PRCD	NCT	ENSG00000214140:[ENST00000592432(n.248+791T>C)]	NA	NA	2.00E-03	9.05E-03	1.51E-03	2.00E-03	0.00E+00	NA	NA	NA	MODIFIER	NA	NA	60	59	0	43	16	27	62.79
Non-user	IOB_24	chr17	74673774	C	T	MXRA7	MISSENSE	ENSG00000182534:[ENST00000355797(c.511G>A,Gaa>Aaa):ENSP00000348050(p.Glu171Lys)]	NA	NA	2.00E-04	2.97E-04	6.66E-04	2.00E-04	0.00E+00	NA	NA	NA	MODERATE	NA	NA	174	174	0	161	85	76	47.2
Non-user	IOB_24	chr17	75196787	C	A	SEC14L1	INTRONIC	ENSG00000129657:[ENST00000392476(c.1009+32C>A)]	NA	NA	NA	8.54E-06	7.39E-05	NA	NA	NA	NA	NA	MODIFIER	NA	NA	49	49	0	58	33	25	43.1
Non-user	IOB_24	chr17	76117800	C	T	TMC6	NCT-SS-PRX	ENSG00000141524:[ENST00000593044(n.1808-8G>A)]	NA	NA	6.79E-03	1.27E-02	1.27E-03	6.79E-03	0.00E+00	NA	NA	NA	LOW	NA	NA	589	589	0	470	239	230	48.94
Non-user	IOB_24	chr17	76235355	G	A	TMEM235	INTRONIC-SS-DNR	ENSG00000204278:[ENST00000551068(c.659+1G>A)]	NA	NA	NA	6.70E-04	1.24E-03	NA	NA	NA	NA	NA	HIGH	NA	NA	66	65	0	53	27	25	47.17
Non-user	IOB_24	chr17	76446872	A	G	DNAH17	SILENT	ENSG00000187775:[ENST00000585328(c.10776T>C,gaT>gaC):ENSP00000465516(p.Asp3592(=))]	NA	NA	2.80E-02	3.86E-02	7.48E-02	2.80E-02	7.36E-02	NA	NA	NA	LOW	NA	0.79	87	87	0	82	44	38	46.34
Non-user	IOB_24	chr17	76563187	C	T	DNAH17	MISSENSE	ENSG00000187775:[ENST00000389840(c.1346G>A,cGt>cAt):ENSP00000374490(p.Arg449His)]	NA	NA	chr17*76563187*76563187*C*A,T*rs748524638*NA*NA*NA	NA	NA	NA	NA	D	BN	NA	MODERATE	N	0.01	129	129	0	96	45	51	53.12
Non-user	IOB_24	chr17	77111735	C	T	RBFOX3	SILENT	ENSG00000167281:[ENST00000415831(c.63G>A,gaG>gaA):ENSP00000408395(p.Glu21(=))]	NA	NA	1.40E-03	8.96E-04	2.07E-03	1.40E-03	3.10E-03	NA	NA	NA	LOW	NA	0.999	49	49	0	61	32	29	47.54
Non-user	IOB_24	chr17	78063467	G	A	CCDC40	NCT	ENSG00000141519:[ENST00000575431(n.356-96G>A)]	NA	NA	3.20E-02	NA	NA	3.19E-02	3.58E-02	NA	NA	NA	MODIFIER	NA	NA	82	82	0	93	51	41	44.09
Non-user	IOB_24	chr17	78169374	C	T	CARD14	MISSENSE	ENSG00000141527:[ENST00000392434(c.806C>T,cCg>cTg):ENSP00000376229(p.Pro269Leu)]	NA	chr17*78169374*78169374*COSM1324755*C*T*1*ovary*1|chr17*78169374*78169374*COSM1324754*C*T*1*ovary*1	1.56E-02	1.65E-02	5.49E-02	1.56E-02	6.03E-02	NA	NA	NA	LOW	NA	NA	239	237	1	242	118	123	50.83
Non-user	IOB_24	chr17	78298926	G	A	RNF213	MISSENSE	ENSG00000173821:[ENST00000456466(c.3121G>A,Gcg>Acg):ENSP00000392123(p.Ala1041Thr)]	NA	NA	3.75E-02	4.83E-02	4.22E-02	3.75E-02	3.27E-02	NA	BN	NA	MODERATE	NA	NA	144	144	0	110	52	58	52.73
Non-user	IOB_24	chr17	78335610	G	A	CTD-2047H16.4	NCT	ENSG00000263069:[ENST00000575034(n.191-6522C>T)]	NA	NA	4.05E-02	4.96E-02	6.38E-02	4.05E-02	4.81E-02	NA	NA	NA	MODIFIER	NA	NA	105	105	0	107	46	61	57.01
Non-user	IOB_24	chr17	78335610	G	A	LOC100294362	NCT	100294362:[NR_029376.1(n.241-6522C>T)]	NA	NA	4.05E-02	4.96E-02	6.38E-02	4.05E-02	4.81E-02	NA	NA	NA	MODIFIER	NA	NA	105	105	0	107	46	61	57.01
Non-user	IOB_24	chr17	78335610	G	A	RNF213	SILENT	ENSG00000173821:[ENST00000582970(c.11277G>A,ccG>ccA):ENSP00000464087(p.Pro3759(=))]	NA	NA	4.05E-02	4.96E-02	6.38E-02	4.05E-02	4.81E-02	NA	NA	NA	MODIFIER	NA	NA	105	105	0	107	46	61	57.01
Non-user	IOB_24	chr17	78934088	G	A	RPTOR	INTRONIC	57521:[XM_005257541.1(c.2777+83G>A)]	NA	NA	3.44E-02	NA	NA	3.43E-02	4.50E-02	NA	NA	NA	MODIFIER	NA	NA	96	96	0	67	36	31	46.27
Non-user	IOB_24	chr17	78938134	C	T	RPTOR	3UTR	ENSG00000141564:[ENST00000306801(c.*4C>T)]	NA	NA	1.56E-02	2.24E-02	3.85E-02	1.56E-02	2.76E-02	NA	NA	NA	MODIFIER	NA	NA	419	418	0	334	181	150	44.91
Non-user	IOB_24	chr17	79006900	G	A	BAIAP2-AS1	EXONIC-NC	440465:[NR_026857.1(n.454C>T)]	NA	NA	6.99E-03	1.05E-02	1.13E-02	6.99E-03	1.53E-02	NA	NA	NA	MODIFIER	NA	NA	266	266	0	173	85	88	50.87
Non-user	IOB_24	chr17	79031612	C	T	BAIAP2	INTRONIC	ENSG00000175866:[ENST00000572329(c.230-69C>T)]	NA	NA	3.18E-02	NA	NA	3.17E-02	5.73E-02	NA	NA	NA	MODIFIER	NA	NA	109	109	0	72	30	41	56.94
Non-user	IOB_24	chr17	79164649	C	T	AZI1	INTRONIC	ENSG00000141577:[ENST00000575907(c.2845-47G>A)]	NA	NA	NA	1.81E-05	0.00E+00	NA	NA	NA	NA	NA	MODIFIER	NA	NA	145	144	1	109	53	55	50.46
Non-user	IOB_24	chr17	79169670	C	T	AZI1	MISSENSE	ENSG00000141577:[ENST00000573053(c.298G>A,Gag>Aag):ENSP00000460006(p.Glu100Lys)]	NA	NA	1.76E-02	2.34E-02	5.29E-02	1.76E-02	5.11E-02	T	BN	NA	MODERATE	N	0.994	72	72	0	72	0	72	100
Non-user	IOB_24	chr17	79497692	G	A	FSCN2	INTRONIC	ENSG00000186765:[ENST00000334850(c.826+1309G>A)]	NA	NA	2.00E-04	2.77E-04	1.93E-03	2.00E-04	1.00E-03	NA	NA	NA	MODIFIER	NA	NA	75	75	0	50	26	24	48
Non-user	IOB_24	chr17	79612433	G	C	NPLOC4	NCT	ENSG00000182446:[ENST00000570300(n.36+3027C>G)]	NA	NA	2.60E-03	2.38E-03	1.65E-02	2.60E-03	1.33E-02	NA	NA	NA	MODIFIER	NA	NA	230	228	1	146	76	67	45.89
Non-user	IOB_24	chr17	79612433	G	C	TSPAN10	MISSENSE	ENSG00000182612:[ENST00000328585(c.452G>C,cGt>cCt):ENSP00000331620(p.Arg151Pro)]	NA	NA	2.60E-03	2.38E-03	1.65E-02	2.60E-03	1.33E-02	NA	NA	NA	MODIFIER	NA	NA	230	228	1	146	76	67	45.89
Non-user	IOB_24	chr17	79801827	G	A	P4HB	EXONIC-NC	ENSG00000185624:[ENST00000473021(n.455C>T)]	NA	NA	1.94E-02	NA	NA	1.94E-02	3.78E-02	NA	NA	NA	MODIFIER	NA	NA	56	56	0	45	21	24	53.33
Non-user	IOB_24	chr17	79801827	G	A	RP11-498C9.2	EXONIC-NC	ENSG00000262831:[ENST00000576784(n.522G>A)]	NA	NA	1.94E-02	NA	NA	1.94E-02	3.78E-02	NA	NA	NA	MODIFIER	NA	NA	56	56	0	45	21	24	53.33
Non-user	IOB_24	chr17	79980743	C	T	STRA13	INTRONIC	ENSG00000169689:[ENST00000579520(c.43+1198G>A)]	NA	NA	2.80E-03	7.21E-03	4.18E-03	2.80E-03	3.10E-03	NA	NA	NA	MODIFIER	NA	NA	74	73	0	69	34	34	49.28
Non-user	IOB_24	chr17	79986156	C	T	LRRC45	MISSENSE	ENSG00000169683:[ENST00000306688(c.1094C>T,gCc>gTc):ENSP00000306760(p.Ala365Val)]	NA	chr17*79986156*79986156*COSM437811*C*T*1*breast*1	3.75E-02	1.11E-01	2.61E-02	3.75E-02	2.35E-02	NA	NA	NA	MODIFIER	NA	NA	107	107	0	81	37	44	54.32
Non-user	IOB_24	chr17	79990728	C	T	RAC3	INTRONIC	ENSG00000169750:[ENST00000306897(c.225+24C>T)]	NA	NA	2.72E-02	6.85E-02	2.09E-02	2.72E-02	2.35E-02	NA	NA	NA	MODIFIER	NA	NA	216	216	0	187	94	92	49.2
Non-user	IOB_24	chr17	79994649	G	A	DCXR	NCT	ENSG00000169738:[ENST00000580750(n.321-10C>T)]	NA	NA	3.20E-03	2.17E-03	1.15E-02	3.19E-03	1.33E-02	NA	NA	NA	MODIFIER	NA	NA	203	202	0	175	89	85	48.57
Non-user	IOB_24	chr17	80040186	G	A	FASN	NCT	ENSG00000169710:[ENST00000579758(n.542+13C>T)]	NA	NA	6.59E-03	4.58E-03	1.89E-02	6.59E-03	2.56E-02	NA	NA	NA	MODIFIER	NA	NA	78	78	0	47	21	25	53.19
Non-user	IOB_24	chr17	80543937	G	A	FOXK2	SILENT	ENSG00000141568:[ENST00000335255(c.1437G>A,gcG>gcA):ENSP00000335677(p.Ala479(=))]	NA	NA	2.00E-04	6.38E-04	6.67E-04	2.00E-04	1.00E-03	NA	NA	NA	LOW	NA	NA	348	344	1	213	113	100	46.95
Non-user	IOB_24	chr17	80828203	C	T	TBCD	SILENT	ENSG00000141556:[ENST00000355528(c.1422C>T,ttC>ttT):ENSP00000347719(p.Phe474(=))]	NA	NA	1.06E-02	2.81E-02	6.75E-03	1.06E-02	6.10E-03	NA	NA	NA	MODIFIER	NA	NA	380	378	2	296	152	142	47.97
Non-user	IOB_24	chr18	264012	G	A	THOC1	NCT	ENSG00000079134:[ENST00000579232(n.256+14C>T)]	NA	NA	NA	1.98E-01	2.22E-01	NA	NA	NA	NA	NA	MODIFIER	NA	NA	71	71	0	54	33	21	38.89
Non-user	IOB_24	chr18	658035	A	G	C18orf56	SILENT	ENSG00000176912:[ENST00000323813(c.213T>C,ggT>ggC):ENSP00000316465(p.Gly71(=))]	NA	NA	2.86E-02	2.46E-02	4.50E-02	2.86E-02	3.48E-02	NA	NA	NA	MODIFIER	NA	NA	336	334	1	307	136	171	55.7
Non-user	IOB_24	chr18	658035	A	G	TYMS	NCT	ENSG00000176890:[ENST00000579128(n.283+88A>G)]	NA	NA	2.86E-02	2.46E-02	4.50E-02	2.86E-02	3.48E-02	NA	NA	NA	MODIFIER	NA	NA	336	334	1	307	136	171	55.7
Non-user	IOB_24	chr18	3126888	T	C	MYOM1	SILENT	ENSG00000101605:[ENST00000356443(c.2802A>G,ccA>ccG):ENSP00000348821(p.Pro934(=))]	NA	NA	1.54E-02	6.92E-03	2.81E-03	1.54E-02	2.00E-03	NA	NA	NA	MODIFIER	NA	NA	297	294	0	219	118	101	46.12
Non-user	IOB_24	chr18	6928459	G	A	LINC00668	EXONIC-NC	ENSG00000265933:[ENST00000580197(n.113C>T)]	NA	NA	NA	NA	NA	NA	NA	NA	NA	NA	MODIFIER	NA	NA	68	68	0	47	28	19	40.43
Non-user	IOB_24	chr18	7035973	C	T	LAMA1	INTRONIC	ENSG00000101680:[ENST00000389658(c.1839+13G>A)]	NA	NA	1.32E-02	1.72E-02	6.23E-02	1.32E-02	5.11E-02	NA	NA	NA	MODIFIER	NA	NA	127	127	0	106	66	37	34.91
Non-user	IOB_24	chr18	7231287	G	T	LRRC30	MISSENSE	ENSG00000206422:[ENST00000383467(c.151G>T,Gtc>Ttc):ENSP00000372959(p.Val51Phe)]	NA	chr18*7231287*7231287*COSM365109*G*T*1*lung*1	NA	4.14E-05	1.82E-04	NA	NA	T	BN	NA	MODERATE	N	0.841	231	231	0	157	81	75	47.77
Non-user	IOB_24	chr18	8806824	C	G	SOGA2	NCT	ENSG00000168502:[ENST00000522592(n.337-67C>G)]	NA	NA	NA	NA	NA	NA	NA	NA	NA	NA	MODIFIER	NA	NA	62	62	0	51	19	31	60.78
Non-user	IOB_24	chr18	9122611	T	C	NDUFV2	MISSENSE	ENSG00000178127:[ENST00000318388(c.401T>C,gTc>gCc):ENSP00000327268(p.Val134Ala)]	NA	NA	NA	7.64E-02	6.08E-02	NA	NA	NA	NA	NA	MODIFIER	NA	NA	271	270	0	225	217	7	3.11
Non-user	IOB_24	chr18	9122611	T	C	NDUFV2-AS1	NCT	101927275:[XR_243829.1(n.473-1157A>G)]	NA	NA	NA	7.64E-02	6.08E-02	NA	NA	NA	NA	NA	MODIFIER	NA	NA	271	270	0	225	217	7	3.11
Non-user	IOB_24	chr18	9122611	T	C	RP11-143J12.2	NCT	ENSG00000266053:[ENST00000583081(n.220-1157A>G)]	NA	NA	NA	7.64E-02	6.08E-02	NA	NA	NA	NA	NA	MODIFIER	NA	NA	271	270	0	225	217	7	3.11
Non-user	IOB_24	chr18	9122611	T	C	RP11-21J18.1	NCT	ENSG00000265257:[ENST00000578850(n.62+19816T>C)]	NA	NA	NA	7.64E-02	6.08E-02	NA	NA	NA	NA	NA	MODIFIER	NA	NA	271	270	0	225	217	7	3.11
Non-user	IOB_24	chr18	9122638	G	C	NDUFV2	MISSENSE	ENSG00000178127:[ENST00000318388(c.428G>C,cGa>cCa):ENSP00000327268(p.Arg143Pro)]	NA	chr18*9122638*9122638*COSM990227*G*A*1*endometrium*1|chr18*9122638*9122638*COSM148385*G*C*1*stomach*1	chr18*9122638*9122638*G*A,C*rs148158107*NA*NA*NA	1.01E-02	8.12E-03	NA	NA	NA	NA	NA	MODIFIER	NA	NA	283	283	0	252	244	8	3.17
Non-user	IOB_24	chr18	9122638	G	C	NDUFV2-AS1	NCT	101927275:[NR_110772.1(n.478-1184C>G)]	NA	chr18*9122638*9122638*COSM990227*G*A*1*endometrium*1|chr18*9122638*9122638*COSM148385*G*C*1*stomach*1	chr18*9122638*9122638*G*A,C*rs148158107*NA*NA*NA	1.01E-02	8.12E-03	NA	NA	NA	NA	NA	MODIFIER	NA	NA	283	283	0	252	244	8	3.17
Non-user	IOB_24	chr18	9122638	G	C	RP11-143J12.2	NCT	ENSG00000266053:[ENST00000582375(n.579-1184C>G)]	NA	chr18*9122638*9122638*COSM990227*G*A*1*endometrium*1|chr18*9122638*9122638*COSM148385*G*C*1*stomach*1	chr18*9122638*9122638*G*A,C*rs148158107*NA*NA*NA	1.01E-02	8.12E-03	NA	NA	NA	NA	NA	MODIFIER	NA	NA	283	283	0	252	244	8	3.17
Non-user	IOB_24	chr18	9122638	G	C	RP11-21J18.1	EXONIC-NC	ENSG00000265257:[ENST00000579126(n.436G>C)]	NA	chr18*9122638*9122638*COSM990227*G*A*1*endometrium*1|chr18*9122638*9122638*COSM148385*G*C*1*stomach*1	chr18*9122638*9122638*G*A,C*rs148158107*NA*NA*NA	1.01E-02	8.12E-03	NA	NA	NA	NA	NA	MODIFIER	NA	NA	283	283	0	252	244	8	3.17
Non-user	IOB_24	chr18	9204535	A	G	ANKRD12	SILENT	ENSG00000101745:[ENST00000262126(c.297A>G,acA>acG):ENSP00000262126(p.Thr99(=))]	NA	NA	4.39E-03	6.16E-03	1.81E-02	4.39E-03	1.33E-02	NA	NA	NA	MODIFIER	NA	NA	187	186	0	127	73	54	42.52
Non-user	IOB_24	chr18	9204535	A	G	RP11-21J18.1	NCT	ENSG00000265257:[ENST00000578850(n.63-49674A>G)]	NA	NA	4.39E-03	6.16E-03	1.81E-02	4.39E-03	1.33E-02	NA	NA	NA	MODIFIER	NA	NA	187	186	0	127	73	54	42.52
Non-user	IOB_24	chr18	9859341	C	T	RAB31	EXONIC-NC	ENSG00000168461:[ENST00000583921(n.555C>T)]	NA	NA	1.64E-02	1.42E-02	5.82E-02	1.64E-02	7.26E-02	NA	NA	NA	MODIFIER	NA	NA	97	96	0	69	31	36	52.17
Non-user	IOB_24	chr18	9886567	T	G	TXNDC2	INTRONIC	ENSG00000168454:[ENST00000357775(c.-92-19T>G)]	NA	NA	NA	4.78E-01	4.01E-01	NA	NA	NA	NA	NA	MODIFIER	NA	NA	36	36	0	21	12	9	42.86
Non-user	IOB_24	chr18	10485824	G	A	APCDD1	INTRONIC	ENSG00000154856:[ENST00000579685(c.*234+44G>A)]	NA	NA	1.20E-03	2.42E-03	9.32E-04	1.20E-03	0.00E+00	NA	NA	NA	MODIFIER	NA	NA	110	109	0	78	39	39	50
Non-user	IOB_24	chr18	10857097	C	A	PIEZO2	MISSENSE	ENSG00000154864:[ENST00000302079(c.605G>T,cGc>cTc):ENSP00000303316(p.Arg202Leu)]	NA	NA	NA	9.89E-05	0.00E+00	NA	NA	T	PrD	NA	MODERATE	NA	NA	105	104	0	86	41	45	52.33
Non-user	IOB_24	chr18	11876613	T	G	GNAL	NCT-SS-PRX	2774:[XR_243798.1(n.1498-7T>G)]	NA	NA	4.39E-02	6.28E-02	5.26E-02	4.39E-02	4.70E-02	NA	NA	NA	LOW	NA	NA	170	170	0	175	103	72	41.14
Non-user	IOB_24	chr18	12720647	G	A	PSMG2	SILENT	ENSG00000128789:[ENST00000585331(c.453G>A,ccG>ccA):ENSP00000476763(p.Pro151(=))]	NA	NA	5.99E-04	1.96E-04	3.77E-04	5.99E-04	1.00E-03	NA	NA	NA	LOW	NA	NA	186	186	0	150	82	68	45.33
Non-user	IOB_24	chr18	19154158	C	A	ESCO1	MISSENSE	ENSG00000141446:[ENST00000269214(c.647G>T,tGt>tTt):ENSP00000269214(p.Cys216Phe)]	NA	NA	5.19E-03	2.70E-03	1.97E-02	5.19E-03	2.66E-02	T	PoD	NA	MODERATE	NA	NA	427	427	0	291	159	130	44.67
Non-user	IOB_24	chr18	21355912	G	A	LAMA3	INTRONIC	ENSG00000053747:[ENST00000313654(c.1405+25G>A)]	NA	NA	1.74E-02	1.51E-02	6.45E-02	1.74E-02	7.06E-02	NA	NA	NA	MODIFIER	NA	NA	188	188	0	183	92	88	48.09
Non-user	IOB_24	chr18	21394384	G	A	LAMA3	SILENT	ENSG00000053747:[ENST00000313654(c.1806G>A,aaG>aaA):ENSP00000324532(p.Lys602(=))]	NA	NA	1.72E-02	1.52E-02	6.51E-02	1.72E-02	6.95E-02	NA	NA	NA	LOW	NA	NA	204	203	0	182	91	91	50
Non-user	IOB_24	chr18	21447771	A	G	LAMA3	INTRONIC	ENSG00000053747:[ENST00000313654(c.4684-27A>G)]	NA	NA	1.76E-02	1.58E-02	6.80E-02	1.76E-02	7.16E-02	NA	NA	NA	MODIFIER	NA	NA	135	135	0	146	77	68	46.58
Non-user	IOB_24	chr18	24441215	C	T	AQP4	SILENT	ENSG00000171885:[ENST00000440832(c.426G>A,ttG>ttA):ENSP00000393121(p.Leu142(=))]	NA	NA	4.11E-02	3.00E-02	1.00E-01	4.11E-02	1.13E-01	NA	NA	NA	MODIFIER	NA	NA	247	245	0	153	56	96	62.75
Non-user	IOB_24	chr18	24441215	C	T	AQP4-AS1	NCT	ENSG00000260372:[ENST00000582605(n.306-63509C>T)]	NA	NA	4.11E-02	3.00E-02	1.00E-01	4.11E-02	1.13E-01	NA	NA	NA	MODIFIER	NA	NA	247	245	0	153	56	96	62.75
Non-user	IOB_24	chr18	24442227	C	T	AQP4	SILENT	ENSG00000171885:[ENST00000383168(c.366G>A,caG>caA):ENSP00000372654(p.Gln122(=))]	NA	NA	3.16E-02	2.72E-02	9.63E-02	3.15E-02	1.07E-01	NA	NA	NA	LOW	NA	NA	119	119	0	98	49	48	48.98
Non-user	IOB_24	chr18	24442227	C	T	AQP4-AS1	NCT	ENSG00000260372:[ENST00000579964(n.93-62497C>T)]	NA	NA	3.16E-02	2.72E-02	9.63E-02	3.15E-02	1.07E-01	NA	NA	NA	LOW	NA	NA	119	119	0	98	49	48	48.98
Non-user	IOB_24	chr18	28980821	C	T	DSG4	INTRONIC	ENSG00000175065:[ENST00000308128(c.1278-23C>T)]	NA	NA	1.24E-02	5.55E-03	2.25E-02	1.24E-02	2.76E-02	NA	NA	NA	MODIFIER	NA	NA	21	21	0	23	11	12	52.17
Non-user	IOB_24	chr18	28980821	C	T	RP11-534N16.1	NCT	ENSG00000266729:[ENST00000581856(n.95+25975G>A)]	NA	NA	1.24E-02	5.55E-03	2.25E-02	1.24E-02	2.76E-02	NA	NA	NA	MODIFIER	NA	NA	21	21	0	23	11	12	52.17
Non-user	IOB_24	chr18	28989574	C	T	DSG4	INTRONIC	ENSG00000175065:[ENST00000308128(c.2073+20C>T)]	NA	NA	9.98E-04	8.65E-04	6.30E-03	9.98E-04	5.10E-03	NA	NA	NA	MODIFIER	NA	NA	98	98	0	64	29	35	54.69
Non-user	IOB_24	chr18	28989574	C	T	RP11-534N16.1	NCT	ENSG00000266729:[ENST00000578477(n.156+17222G>A)]	NA	NA	9.98E-04	8.65E-04	6.30E-03	9.98E-04	5.10E-03	NA	NA	NA	MODIFIER	NA	NA	98	98	0	64	29	35	54.69
Non-user	IOB_24	chr18	29046606	G	A	DSG3	MISSENSE	ENSG00000134757:[ENST00000257189(c.1525G>A,Gtg>Atg):ENSP00000257189(p.Val509Met)]	NA	NA	4.89E-02	2.72E-02	9.87E-02	4.89E-02	1.22E-01	D	PrD	NA	MODERATE	N	0.706	356	355	0	329	144	183	55.62
Non-user	IOB_24	chr18	29426649	A	G	TRAPPC8	INTRONIC	22878:[XM_005258234.1(c.3840+30T>C)]	NA	NA	4.73E-02	5.81E-02	5.12E-02	4.73E-02	4.40E-02	NA	NA	NA	MODIFIER	NA	NA	46	46	0	38	16	22	57.89
Non-user	IOB_24	chr18	29706835	C	G	GAREM	INTRONIC	ENSG00000141441:[ENST00000583696(c.*49-1648G>C)]	NA	NA	4.59E-03	NA	NA	4.59E-03	2.35E-02	NA	NA	NA	MODIFIER	NA	NA	11	11	0	8	3	5	62.5
Non-user	IOB_24	chr18	29706835	C	G	RNF138	INTRONIC	51444:[XM_005258285.1(c.531+72C>G)]	NA	NA	4.59E-03	NA	NA	4.59E-03	2.35E-02	NA	NA	NA	MODIFIER	NA	NA	11	11	0	8	3	5	62.5
Non-user	IOB_24	chr18	30926284	G	A	CCDC178	SILENT	ENSG00000166960:[ENST00000300227(c.549C>T,gaC>gaT):ENSP00000300227(p.Asp183(=))]	NA	chr18*30926284*30926284*COSM1388485*G*A*1*large_intestine*1|chr18*30926284*30926284*COSM1388484*G*A*1*large_intestine*1	7.99E-03	6.11E-03	7.64E-03	7.99E-03	4.10E-03	NA	NA	NA	LOW	NA	NA	190	189	0	168	94	74	44.05
Non-user	IOB_24	chr18	32833263	A	C	ZNF397	INTRONIC	ENSG00000186812:[ENST00000261333(c.647-933A>C)]	NA	NA	3.10E-02	NA	NA	3.10E-02	7.87E-02	NA	NA	NA	MODIFIER	NA	NA	107	107	0	73	46	27	36.99
Non-user	IOB_24	chr18	32833263	A	C	ZSCAN30	EXONIC-NC	ENSG00000186814:[ENST00000590777(n.1323T>G)]	NA	NA	3.10E-02	NA	NA	3.10E-02	7.87E-02	NA	NA	NA	MODIFIER	NA	NA	107	107	0	73	46	27	36.99
Non-user	IOB_24	chr18	33691094	A	G	SLC39A6	SILENT	ENSG00000141424:[ENST00000269187(c.2043T>C,taT>taC):ENSP00000269187(p.Tyr681(=))]	NA	NA	9.98E-04	1.11E-03	7.88E-03	9.98E-04	5.10E-03	NA	NA	NA	LOW	NA	NA	125	125	0	126	64	60	47.62
Non-user	IOB_24	chr18	43204609	G	A	SLC14A2	5UTR	ENSG00000132874:[ENST00000255226(c.-21G>A)]	NA	NA	7.99E-04	3.32E-04	2.60E-03	7.99E-04	4.10E-03	NA	NA	NA	MODIFIER	NA	NA	73	73	0	63	40	23	36.51

Patel *et al.* , 2021. Whole-exome sequencing analysis of oral squamous cell carcinoma delineated by tobacco usage habits
Supplementary Table 4. List of somatic single nucleotide variants identified using whole exome sequencing of OSCC patients

Cohort	Sample	Chromosome	Genomic position	Reference allele	Alternate allele	Genesymbol	Variant type	Variant description	OncoMD	COSMIC	dbSNP	ExAC	ExAC_SAS	1000G	1000G_SAS	SIFT	Polyphen2	CONDEL	VEP	LRT	PhastCon7Way	Total reads in Normal	Reference read support in Normal	Alternative read support in Normal	Total reads in Tumor	Reference read support in Tumor	Alternate read support in Tumor	Alternate allele frequency in Tumor
Non-user	IOB_24	chr18	43534944	C	T	EPG5	MISSENSE	ENSG00000152223:[ENST00000282041(c.424G>A,Gag>Aag):ENSP00000282041(p.Glu142Lys)]	NA	NA	2.00E-04	2.32E-04	4.25E-04	2.00E-04	0.00E+00	NA	NA	NA	MODIFIER	NA	NA	221	220	0	157	68	88	56.05
Non-user	IOB_24	chr18	44104633	T	G	LOXHD1	INTRONIC	ENSG00000167210:[ENST00000398722(c.3906+38A>C)]	NA	NA	7.99E-04	1.46E-03	7.59E-04	7.99E-04	0.00E+00	NA	NA	NA	MODIFIER	NA	NA	153	151	2	74	41	33	44.59
Non-user	IOB_24	chr18	44137400	C	T	LOXHD1	MISSENSE	ENSG00000167210:[ENST00000398722(c.2435G>A,cGg>cAg):ENSP00000381707(p.Arg812Gln)]	NA	NA	7.19E-03	9.59E-03	2.02E-03	7.19E-03	0.00E+00	NA	NA	NA	MODIFIER	NA	NA	143	139	0	115	65	48	41.74
Non-user	IOB_24	chr18	44400944	C	T	PIAS2	MISSENSE	ENSG00000078043:[ENST00000324794(c.1600G>A,Gta>Ata):ENSP00000317163(p.Val534Ile)]	NA	NA	5.99E-04	4.29E-04	3.09E-03	5.99E-04	3.10E-03	NA	NA	NA	MODIFIER	NA	NA	263	262	0	206	109	96	46.6
Non-user	IOB_24	chr18	46147966	G	A	CTIF	EXONIC-NC	ENSG00000134030:[ENST00000589585(n.152G>A)]	NA	NA	2.32E-02	7.13E-02	8.68E-02	2.32E-02	4.81E-02	NA	NA	NA	MODIFIER	NA	NA	67	66	0	57	37	19	33.33
Non-user	IOB_24	chr18	46570644	A	G	DYM	INTRONIC	54808:[XM_005258289.1(c.1843-70T>C)]	NA	NA	3.89E-02	NA	NA	3.89E-02	6.34E-02	NA	NA	NA	MODIFIER	NA	NA	36	36	0	33	18	15	45.45
Non-user	IOB_24	chr18	46570644	A	G	RP11-15F12.1	NCT	ENSG00000264269:[ENST00000584252(n.313-3854A>G)]	NA	NA	3.89E-02	NA	NA	3.89E-02	6.34E-02	NA	NA	NA	MODIFIER	NA	NA	36	36	0	33	18	15	45.45
Non-user	IOB_24	chr18	46812815	T	C	DYM	MISSENSE	ENSG00000141627:[ENST00000442713(c.365A>G,aAg>aGg):ENSP00000395942(p.Lys122Arg)]	NA	NA	NA	6.59E-05	4.85E-04	NA	NA	T	BN	NA	MODERATE	N	1	101	101	0	87	45	42	48.28
Non-user	IOB_24	chr18	47379930	C	T	MYO5B	SILENT	ENSG00000167306:[ENST00000324581(c.1455G>A,gaG>gaA):ENSP00000315531(p.Glu485(=))]	NA	NA	2.00E-04	9.11E-05	6.67E-04	2.00E-04	1.00E-03	NA	NA	NA	LOW	NA	0.005	164	162	0	167	87	80	47.9
Non-user	IOB_24	chr18	47511113	C	A	MYO5B	MISSENSE	ENSG00000167306:[ENST00000285039(c.921G>T,aaG>aaT):ENSP00000285039(p.Lys307Asn)]	NA	NA	3.16E-02	5.11E-02	3.49E-02	3.15E-02	2.66E-02	T	BN	NA	MODERATE	D	1	43	43	0	42	18	24	57.14
Non-user	IOB_24	chr18	55336637	A	G	ATP8B1	SILENT	ENSG00000081923:[ENST00000283684(c.2010T>C,aaT>aaC):ENSP00000283684(p.Asn670(=))]	NA	NA	7.99E-04	1.66E-04	1.22E-03	7.99E-04	4.10E-03	NA	NA	NA	LOW	NA	1	123	123	0	97	50	47	48.45
Non-user	IOB_24	chr18	55336637	A	G	RP11-35G9.3	NCT	ENSG00000267040:[ENST00000599199(n.570+27353A>G)]	NA	NA	7.99E-04	1.66E-04	1.22E-03	7.99E-04	4.10E-03	NA	NA	NA	LOW	NA	1	123	123	0	97	50	47	48.45
Non-user	IOB_24	chr18	55336637	A	G	RP11-35G9.5	NCT	ENSG00000267787:[ENST00000588925(n.570+27353A>G)]	NA	NA	7.99E-04	1.66E-04	1.22E-03	7.99E-04	4.10E-03	NA	NA	NA	LOW	NA	1	123	123	0	97	50	47	48.45
Non-user	IOB_24	chr18	56401523	C	T	MALT1	EXONIC-NC	ENSG00000172175:[ENST00000589873(n.782C>T)]	NA	NA	3.59E-03	4.96E-03	3.71E-03	3.59E-03	5.10E-03	NA	NA	NA	MODIFIER	NA	NA	323	322	0	245	115	126	51.43
Non-user	IOB_24	chr18	56401523	C	T	RP11-126O1.4	NCT	ENSG00000267476:[ENST00000588835(n.56+18384G>A)]	NA	NA	3.59E-03	4.96E-03	3.71E-03	3.59E-03	5.10E-03	NA	NA	NA	MODIFIER	NA	NA	323	322	0	245	115	126	51.43
Non-user	IOB_24	chr18	56963852	T	G	CPLX4	INTRONIC	ENSG00000166569:[ENST00000587244(c.255+16065A>C)]	NA	NA	4.59E-02	NA	NA	4.59E-02	4.29E-02	NA	NA	NA	MODIFIER	NA	NA	99	99	0	99	46	53	53.54
Non-user	IOB_24	chr18	56998268	C	T	LMAN1	EXONIC-NC	ENSG00000074695:[ENST00000592562(n.316G>A)]	NA	NA	1.40E-03	NA	NA	1.40E-03	2.00E-03	NA	NA	NA	MODIFIER	NA	NA	27	26	0	24	13	11	45.83
Non-user	IOB_24	chr18	58298010	C	T	RP11-325K19.1	EXONIC-NC	ENSG00000267098:[ENST00000591869(n.362G>A)]	NA	NA	NA	NA	NA	NA	NA	NA	NA	NA	MODIFIER	NA	NA	287	287	0	264	144	120	45.45
Non-user	IOB_24	chr18	58298010	C	T	RP11-325K19.2	EXONIC-NC	ENSG00000267382:[ENST00000592477(n.1060C>T)]	NA	NA	NA	NA	NA	NA	NA	NA	NA	NA	MODIFIER	NA	NA	287	287	0	264	144	120	45.45
Non-user	IOB_24	chr18	59770171	A	C	PIGN	INTRONIC	23556:[XM_005266676.1(c.1800-36T>G)]	NA	NA	1.40E-03	2.63E-03	2.00E-03	1.40E-03	2.00E-03	NA	NA	NA	MODIFIER	NA	NA	102	102	0	74	32	42	56.76
Non-user	IOB_24	chr18	60625955	C	T	PHLPP1	MISSENSE	ENSG00000081913:[ENST00000400316(c.1882C>T,Cgc>Tgc):ENSP00000383170(p.Arg628Cys)]	NA	NA	1.20E-03	2.07E-04	9.69E-04	1.20E-03	4.10E-03	D	PrD	NA	MODERATE	NA	NA	159	158	1	142	72	70	49.3
Non-user	IOB_24	chr18	60645818	C	T	PHLPP1	SILENT	ENSG00000081913:[ENST00000262719(c.4308C>T,gcC>gcT):ENSP00000262719(p.Ala1436(=))]	NA	chr18*60645818*60645818*COSM438300*C*T*1*breast*1|chr18*60645818*60645818*COSM1480418*C*T*1*breast*1|chr18*60645818*60645818*COSM1480419*C*T*1*breast*1	1.06E-02	2.24E-02	1.11E-02	1.06E-02	1.33E-02	NA	NA	NA	LOW	NA	NA	275	273	0	207	110	97	46.86
Non-user	IOB_24	chr18	61264445	G	A	SERPINB13	MISSENSE	ENSG00000197641:[ENST00000269489(c.868G>A,Ggc>Agc):ENSP00000269489(p.Gly290Ser)]	NA	NA	NA	3.30E-05	1.21E-04	NA	NA	NA	NA	NA	MODIFIER	NA	NA	291	290	0	202	94	108	53.47
Non-user	IOB_24	chr18	63477206	G	A	CDH7	SILENT	ENSG00000081138:[ENST00000323011(c.477G>A,acG>acA):ENSP00000319166(p.Thr159(=))]	NA	NA	1.66E-02	2.51E-02	9.28E-03	1.66E-02	8.20E-03	NA	NA	NA	LOW	NA	NA	164	163	0	134	62	71	52.99
Non-user	IOB_24	chr18	63530140	C	T	CDH7	SILENT	ENSG00000081138:[ENST00000323011(c.1851C>T,gtC>gtT):ENSP00000319166(p.Val617(=))]	NA	NA	2.66E-02	4.61E-02	1.66E-02	2.66E-02	1.02E-02	NA	NA	NA	LOW	NA	NA	97	97	0	69	20	49	71.01
Non-user	IOB_24	chr18	70461544	T	C	NETO1	NCT	ENSG00000166342:[ENST00000579730(n.303-10403A>G)]	NA	NA	NA	NA	NA	NA	NA	NA	NA	NA	MODIFIER	NA	NA	69	69	0	56	35	20	35.71
Non-user	IOB_24	chr18	71740743	A	G	FBXO15	MISSENSE	ENSG00000141665:[ENST00000269500(c.1258T>C,Tat>Cat):ENSP00000269500(p.Tyr420His)]	NA	chr18*71740743*71740743*COSM1579615*A*G*1*haematopoietic_and_lymphoid_tissue*1|chr18*71740743*71740743*COSM1579616*A*G*1*haematopoietic_and_lymphoid_tissue*1	2.88E-02	6.19E-02	5.09E-02	2.88E-02	4.09E-02	NA	NA	NA	MODIFIER	NA	NA	364	364	0	272	112	160	58.82
Non-user	IOB_24	chr18	74639433	G	A	ZNF236	SILENT	ENSG00000130856:[ENST00000320610(c.4374G>A,ccG>ccA):ENSP00000322361(p.Pro1458(=))]	NA	NA	7.19E-03	1.26E-02	1.57E-02	7.19E-03	1.94E-02	NA	NA	NA	LOW	NA	NA	153	152	0	123	50	72	58.54
Non-user	IOB_24	chr18	74691951	T	C	MBP	MISSENSE	ENSG00000197971:[ENST00000527041(c.271A>G,Act>Gct):ENSP00000435243(p.Thr91Ala)]	NA	NA	2.20E-02	4.56E-02	5.26E-02	2.20E-02	4.09E-02	NA	NA	NA	MODIFIER	NA	NA	52	51	0	25	16	9	36
Non-user	IOB_24	chr18	74691951	T	C	RP11-862L9.3	NCT	ENSG00000266844:[ENST00000582763(n.232+798T>C)]	NA	NA	2.20E-02	4.56E-02	5.26E-02	2.20E-02	4.09E-02	NA	NA	NA	MODIFIER	NA	NA	52	51	0	25	16	9	36
Non-user	IOB_24	chr18	74728823	C	T	MBP	MISSENSE	ENSG00000197971:[ENST00000354542(c.142G>A,Ggt>Agt):ENSP00000346545(p.Gly48Ser)]	NA	NA	2.68E-02	2.62E-02	7.04E-02	2.68E-02	6.54E-02	NA	NA	NA	MODIFIER	NA	NA	166	166	0	106	48	58	54.72
Non-user	IOB_24	chr18	77067183	C	T	ATP9B	SILENT	ENSG00000166377:[ENST00000589326(c.72C>T,gaC>gaT):ENSP00000464761(p.Asp24(=))]	NA	NA	8.39E-03	1.61E-02	9.63E-03	8.39E-03	9.20E-03	NA	NA	NA	LOW	NA	0.979	107	105	0	105	50	55	52.38
Non-user	IOB_24	chr18	77221350	G	A	NA	NCT	101927897:[XR_245492.1(n.390-3345C>T)]	NA	chr18*77221350*77221350*COSM304241*G*A*1*large_intestine*1	NA	4.14E-05	1.21E-04	NA	NA	T	BN	NA	MODERATE	N	1	118	118	0	101	57	42	41.58
Non-user	IOB_24	chr18	77221350	G	A	NFATC1	MISSENSE	ENSG00000131196:[ENST00000587635(c.1858G>A,Ggg>Agg):ENSP00000468111(p.Gly620Arg)]	NA	chr18*77221350*77221350*COSM304241*G*A*1*large_intestine*1	NA	4.14E-05	1.21E-04	NA	NA	T	BN	NA	MODERATE	N	1	118	118	0	101	57	42	41.58
Non-user	IOB_24	chr18	77420680	G	A	LOC284241	EXONIC-NC	284241:[XR_159017.1(n.471C>T)]	NA	NA	3.20E-03	8.79E-04	1.12E-03	3.19E-03	0.00E+00	NA	NA	NA	MODIFIER	NA	NA	57	56	0	49	33	16	32.65
Non-user	IOB_24	chr18	77420680	G	A	RP11-567M16.3	EXONIC-NC	ENSG00000178412:[ENST00000317008(n.477C>T)]	NA	NA	3.20E-03	8.79E-04	1.12E-03	3.19E-03	0.00E+00	NA	NA	NA	MODIFIER	NA	NA	57	56	0	49	33	16	32.65
Non-user	IOB_24	chr18	77659591	C	T	KCNG2	SILENT	ENSG00000178342:[ENST00000316249(c.1176C>T,agC>agT):ENSP00000315654(p.Ser392(=))]	NA	NA	5.99E-04	8.64E-04	8.39E-04	5.99E-04	1.00E-03	NA	NA	NA	MODIFIER	NA	NA	631	629	0	398	387	6	1.51
Non-user	IOB_24	chr19	288062	G	A	PPAP2C	SILENT	ENSG00000141934:[ENST00000269812(c.162C>T,caC>caT):ENSP00000269812(p.His54(=))]	NA	chr19*288062*288062*COSM439154*G*A*1*breast*1|chr19*288062*288062*COSM1480890*G*A*1*breast*1	3.89E-02	6.83E-02	6.26E-02	3.89E-02	4.91E-02	NA	NA	NA	LOW	NA	0	60	60	0	71	40	30	42.25
Non-user	IOB_24	chr19	544212	G	A	GZMM	INTRONIC	3004:[NM_001258351.1(c.-59+86G>A)]	NA	NA	1.48E-02	NA	NA	1.48E-02	4.19E-02	NA	NA	NA	MODIFIER	NA	NA	83	83	0	51	20	30	58.82
Non-user	IOB_24	chr19	549080	A	G	GZMM	SILENT	ENSG00000197540:[ENST00000264553(c.507A>G,caA>caG):ENSP00000264553(p.Gln169(=))]	NA	NA	3.67E-02	8.05E-02	6.55E-02	3.67E-02	3.99E-02	NA	NA	NA	LOW	NA	NA	161	160	0	157	85	72	45.86
Non-user	IOB_24	chr19	549620	G	A	GZMM	INTRONIC	3004:[NM_001258351.1(c.496-10G>A)]	NA	NA	3.99E-04	3.43E-04	1.92E-03	3.99E-04	2.00E-03	NA	NA	NA	MODIFIER	NA	NA	92	91	0	94	66	28	29.79
Non-user	IOB_24	chr19	652785	C	T	RNF126	NCT	ENSG00000070423:[ENST00000591394(n.197+41G>A)]	NA	NA	5.99E-04	2.85E-04	1.77E-03	5.99E-04	3.10E-03	NA	NA	NA	MODIFIER	NA	NA	143	140	1	175	92	82	46.86
Non-user	IOB_24	chr19	685833	G	T	PRSS57	SILENT	ENSG00000185198:[ENST00000329267(c.735C>A,ccC>ccA):ENSP00000327386(p.Pro245(=))]	NA	NA	2.30E-02	4.81E-02	4.83E-02	2.30E-02	5.01E-02	NA	NA	NA	LOW	NA	NA	148	145	0	94	50	43	45.74
Non-user	IOB_24	chr19	731161	G	A	PALM	SILENT	ENSG00000099864:[ENST00000264560(c.336G>A,gcG>gcA):ENSP00000264560(p.Ala112(=))]	NA	NA	NA	7.06E-05	7.10E-05	NA	NA	NA	NA	NA	LOW	NA	NA	135	135	0	93	47	46	49.46
Non-user	IOB_24	chr19	808415	C	T	PTBP1	SILENT	ENSG00000011304:[ENST00000356948(c.1209C>T,gcC>gcT):ENSP00000349428(p.Ala403(=))]	NA	NA	7.99E-04	1.58E-03	9.11E-03	7.99E-04	4.10E-03	NA	NA	NA	LOW	NA	NA	35	35	0	44	15	29	65.91
Non-user	IOB_24	chr19	855778	A	C	ELANE	MISSENSE	ENSG00000197561:[ENST00000263621(c.581A>C,cAg>cCg):ENSP00000263621(p.Gln194Pro)]	NA	NA	2.00E-04	5.16E-05	3.65E-04	2.00E-04	1.00E-03	D	BN	NA	MODERATE	U	0	42	42	0	16	9	7	43.75
Non-user	IOB_24	chr19	855779	G	T	ELANE	MISSENSE	ENSG00000197561:[ENST00000263621(c.582G>T,caG>caT):ENSP00000263621(p.Gln194His)]	NA	chr19*855779*855779*COSM158718*G*T*3*haematopoietic_and_lymphoid_tissue*3	2.00E-04	5.16E-05	3.65E-04	2.00E-04	1.00E-03	D	BN	NA	MODERATE	U	0.028	42	42	0	16	9	7	43.75
Non-user	IOB_24	chr19	863220	C	G	CFD	MISSENSE	ENSG00000197766:[ENST00000592860(c.765C>G,atC>atG):ENSP00000468253(p.Ile255Met)]	NA	NA	3.91E-02	2.91E-02	3.20E-02	3.91E-02	5.32E-02	D	PrD	NA	MODERATE	NA	NA	327	327	0	265	134	130	49.06
Non-user	IOB_24	chr19	966584	C	T	ARID3A	MISSENSE	ENSG00000116017:[ENST00000263620(c.1211C>T,gCc>gTc):ENSP00000263620(p.Ala404Val)]	NA	chr19*966584*966584*COSM1318211*C*T*1*haematopoietic_and_lymphoid_tissue*1	5.79E-03	1.30E-02	4.40E-03	5.79E-03	3.10E-03	NA	NA	NA	MODIFIER	NA	NA	203	202	0	186	96	90	48.39
Non-user	IOB_24	chr19	1004872	G	A	GRIN3B	MISSENSE	ENSG00000116032:[ENST00000234389(c.1372G>A,Gca>Aca):ENSP00000234389(p.Ala458Thr)]	NA	NA	3.79E-02	1.98E-02	3.29E-02	3.79E-02	2.86E-02	NA	NA	NA	MODIFIER	NA	NA	406	404	0	388	174	212	54.64
Non-user	IOB_24	chr19	1005334	A	G	GRIN3B	MISSENSE	ENSG00000116032:[ENST00000234389(c.1834A>G,Acc>Gcc):ENSP00000234389(p.Thr612Ala)]	NA	NA	3.81E-02	2.00E-02	3.32E-02	3.81E-02	2.97E-02	D	PoD	NA	MODERATE	D	1	628	623	1	454	233	216	47.58
Non-user	IOB_24	chr19	1011894	G	A	TMEM259	SILENT	ENSG00000182087:[ENST00000333175(c.939C>T,atC>atT):ENSP00000331423(p.Ile313(=))]	NA	NA	4.43E-02	3.14E-02	4.74E-02	4.43E-02	4.81E-02	NA	NA	NA	LOW	NA	1	554	546	1	450	240	207	46
Non-user	IOB_24	chr19	1065044	C	T	ABCA7	SILENT	ENSG00000064687:[ENST00000435683(c.5745C>T,ggC>ggT):ENSP00000465322(p.Gly1915(=))]	NA	NA	NA	4.05E-01	2.95E-01	NA	NA	NA	NA	NA	LOW	NA	NA	122	120	0	111	63	48	43.24
Non-user	IOB_24	chr19	1080925	G	A	HMHA1	SILENT	ENSG00000180448:[ENST00000313093(c.2052G>A,gtG>gtA):ENSP00000316772(p.Val684(=))]	NA	NA	9.19E-03	1.99E-02	2.35E-02	9.19E-03	1.53E-02	NA	NA	NA	LOW	NA	NA	277	276	0	281	165	114	40.57
Non-user	IOB_24	chr19	1268398	G	A	CIRBP	INTRONIC	ENSG00000099622:[ENST00000585913(c.-7+956G>A)]	NA	NA	3.85E-02	6.22E-02	7.14E-02	3.85E-02	4.09E-02	NA	NA	NA	MODIFIER	NA	NA	41	41	0	30	10	20	66.67
Non-user	IOB_24	chr19	1268398	G	A	CIRBP-AS1	NCT	ENSG00000267493:[ENST00000585832(n.418-9C>T)]	NA	NA	3.85E-02	6.22E-02	7.14E-02	3.85E-02	4.09E-02	NA	NA	NA	MODIFIER	NA	NA	41	41	0	30	10	20	66.67
Non-user	IOB_24	chr19	1397443	G	A	GAMT	MISSENSE	ENSG00000130005:[ENST00000252288(c.626C>T,aCg>aTg):ENSP00000252288(p.Thr209Met)]	NA	NA	3.65E-02	7.55E-02	7.01E-02	3.65E-02	5.21E-02	T	PoD	NA	MODERATE	D	0.999	155	155	0	124	59	64	51.61
Non-user	IOB_24	chr19	1491078	G	A	REEP6	5UTR	92840:[NM_138393.2(c.-191G>A)]	NA	NA	NA	NA	NA	NA	NA	NA	NA	NA	MODIFIER	NA	NA	18	17	0	15	6	8	53.33
Non-user	IOB_24	chr19	1790737	C	T	ATP8B3	NCT	148229:[NR_047593.2(n.2874+19G>A)]	NA	NA	2.80E-03	1.18E-02	1.19E-02	2.80E-03	5.10E-03	NA	NA	NA	MODIFIER	NA	NA	97	94	0	95	52	42	44.21
Non-user	IOB_24	chr19	2213895	G	A	AC004490.1	EXONIC-NC	ENSG00000267122:[ENST00000585593(n.871C>T)]	NA	NA	5.79E-03	2.72E-03	1.89E-02	5.79E-03	2.86E-02	NA	NA	NA	LOW	NA	1	256	255	0	231	134	95	41.13
Non-user	IOB_24	chr19	2213895	G	A	DOT1L	SILENT	ENSG00000104885:[ENST00000586024(c.99G>A,aaG>aaA):ENSP00000476469(p.Lys33(=))]	NA	NA	5.79E-03	2.72E-03	1.89E-02	5.79E-03	2.86E-02	NA	NA	NA	LOW	NA	1	256	255	0	231	134	95	41.13
Non-user	IOB_24	chr19	2328730	G	T	SPPL2B	MISSENSE	56928:[NM_152988.2(c.22G>T,Gcg>Tcg):NP_694533.1(p.Ala8Ser)]	NA	NA	2.30E-02	1.20E-01	1.46E-01	2.30E-02	5.42E-02	NA	NA	NA	MODIFIER	NA	NA	139	138	0	108	50	58	53.7
Non-user	IOB_24	chr19	2421875	C	G	TMPRSS9	SILENT	ENSG00000178297:[ENST00000332578(c.2076C>G,gcC>gcG):ENSP00000330264(p.Ala692(=))]	NA	NA	4.59E-03	1.72E-03	1.45E-02	4.59E-03	2.25E-02	NA	NA	NA	LOW	NA	NA	142	141	0	129	70	59	45.74
Non-user	IOB_24	chr19	3546183	G	A	AC005786.7	NCT	ENSG00000267436:[ENST00000589360(n.456+1531G>A)]	NA	NA	8.19E-03	8.31E-03	2.63E-02	8.19E-03	2.86E-02	NA	NA	NA	MODIFIER	NA	NA	173	169	1	148	68	80	54.05
Non-user	IOB_24	chr19	3546183	G	A	MFSD12	INTRONIC	ENSG00000161091:[ENST00000589995(c.577-17C>T)]	NA	NA	8.19E-03	8.31E-03	2.63E-02	8.19E-03	2.86E-02	NA	NA	NA	MODIFIER	NA	NA	173	169	1	148	68	80	54.05
Non-user	IOB_24	chr19	3667330	G	T	PIP5K1C	MISSENSE	ENSG00000186111:[ENST00000335312(c.116C>A,gCc>gAc):ENSP00000335333(p.Ala39Asp)]	NA	NA	NA	3.43E-05	2.48E-04	NA	NA	NA	NA	NA	MODIFIER	NA	NA	135	135	0	114	56	58	50.88
Non-user	IOB_24	chr19	4298179	G	A	TMIGD2	SILENT	ENSG00000167664:[ENST00000301272(c.210C>T,taC>taT):ENSP00000301272(p.Tyr70(=))]	NA	NA	NA	NA	NA	NA	NA	NA	NA	NA	MODIFIER	NA	NA	162	161	1	129	68	61	47.29
Non-user	IOB_24	chr19	4308069	T	C	FSD1	INTRONIC	ENSG00000105255:[ENST00000597480(c.275+89T>C)]	NA	NA	4.13E-02	NA	NA	4.13E-02	4.50E-02	NA	NA	NA	MODIFIER	NA	NA	66	66	0	59	36	22	37.29
Non-user	IOB_24	chr19	4310217	G	A	FSD1	NCT	ENSG00000105255:[ENST00000598010(n.46-53G>A)]	NA	NA	4.35E-02	NA	NA	4.35E-02	6.10E-03	NA	NA	NA	MODIFIER	NA	NA	26	26	0	23	15	8	34.78
Non-user	IOB_24	chr19	4322990	G	A	FSD1	SILENT	ENSG00000105255:[ENST00000221856(c.1047G>A,acG>acA):ENSP00000221856(p.Thr349(=))]	NA	NA	4.97E-02	7.23E-02	3.84E-02	4.97E-02	2.15E-02	NA	NA	NA	MODIFIER	NA	NA	80	79	0	65	36	29	44.62
Non-user	IOB_24	chr19	4556916	G	A	SEMA6B	INTRONIC	ENSG00000167680:[ENST00000301293(c.369+47C>T)]	NA	NA	1.62E-02	3.83E-02	1.66E-02	1.62E-02	1.53E-02	NA	NA	NA	MODIFIER	NA	NA	101	99	0	77	44	33	42.86

Patel *et al.* , 2021. Whole-exome sequencing analysis of oral squamous cell carcinoma delineated by tobacco usage habits
Supplementary Table 4. List of somatic single nucleotide variants identified using whole exome sequencing of OSCC patients

Cohort	Sample	Chromosome	Genomic position	Reference allele	Alternate allele	Genesymbol	Variant type	Variant description	OncoMD	COSMIC	dbSNP	ExAC	ExAC_SAS	1000G	1000G_SAS	SIFT	Polyphen2	CONDEL	VEP	LRT	PhastCon7Way	Total reads in Normal	Reference read support in Normal	Alternative read support in Normal	Total reads in Tumor	Reference read support in Tumor	Alternate read support in Tumor	Alternate allele frequency in Tumor
Non-user	IOB_24	chr19	5131148	C	T	KDM4B	SILENT	ENSG00000127663:[ENST00000159111(c.1377C>T,ttC>ttT):ENSP00000159111(p.Phe459(=))]	NA	NA	5.99E-04	4.26E-03	1.64E-03	5.99E-04	1.00E-03	NA	NA	NA	LOW	NA	0	145	145	0	114	57	57	50
Non-user	IOB_24	chr19	5137941	C	T	KDM4B	NCT	ENSG00000127663:[ENST00000589104(n.2208-32C>T)]	NA	NA	7.79E-03	1.96E-02	9.24E-03	7.79E-03	8.20E-03	NA	NA	NA	MODIFIER	NA	NA	158	157	1	114	53	61	53.51
Non-user	IOB_24	chr19	5651122	C	A	SAFB	NCT	ENSG00000160633:[ENST00000592396(n.387+39C>A)]	NA	NA	7.99E-04	1.13E-03	8.62E-03	7.99E-04	4.10E-03	NA	NA	NA	MODIFIER	NA	NA	154	154	0	128	74	54	42.19
Non-user	IOB_24	chr19	5892943	C	T	AC024592.12	INTRONIC	ENSG00000267740:[ENST00000586349(c.383+3521G>A)]	NA	NA	1.80E-03	6.32E-03	0.00E+00	1.80E-03	1.00E-03	NA	NA	NA	LOW	NA	NA	112	111	0	97	43	54	55.67
Non-user	IOB_24	chr19	5892943	C	T	FUT5	INTRONIC	ENSG00000130383:[ENST00000252675(c.-172+3521G>A)]	NA	NA	1.80E-03	6.32E-03	0.00E+00	1.80E-03	1.00E-03	NA	NA	NA	LOW	NA	NA	112	111	0	97	43	54	55.67
Non-user	IOB_24	chr19	5892943	C	T	NDUFA11	SILENT	ENSG00000174886:[ENST00000418389(c.672G>A,tcG>tcA):ENSP00000389160(p.Ser224(=))]	NA	NA	1.80E-03	6.32E-03	0.00E+00	1.80E-03	1.00E-03	NA	NA	NA	LOW	NA	NA	112	111	0	97	43	54	55.67
Non-user	IOB_24	chr19	6147434	C	T	ACSBG2	INTRONIC	ENSG00000130377:[ENST00000588485(c.-373-23C>T)]	NA	NA	NA	8.34E-06	6.08E-05	NA	NA	NA	NA	NA	MODIFIER	NA	NA	68	68	0	57	31	25	43.86
Non-user	IOB_24	chr19	6147434	C	T	RFX2	INTRONIC	ENSG00000087903:[ENST00000592883(c.-9+808G>A)]	NA	NA	NA	8.34E-06	6.08E-05	NA	NA	NA	NA	NA	MODIFIER	NA	NA	68	68	0	57	31	25	43.86
Non-user	IOB_24	chr19	6418123	G	A	KHSRP	INTRONIC	ENSG00000088247:[ENST00000595548(c.448-33C>T)]	NA	NA	NA	9.04E-05	5.23E-04	NA	NA	NA	NA	NA	MODIFIER	NA	NA	316	310	1	229	107	121	52.84
Non-user	IOB_24	chr19	6421373	C	T	KHSRP	INTRONIC	ENSG00000088247:[ENST00000595258(c.-47-45G>A)]	NA	NA	1.04E-02	2.24E-02	3.32E-02	1.04E-02	3.58E-02	NA	NA	NA	MODIFIER	NA	NA	111	110	0	93	59	33	35.48
Non-user	IOB_24	chr19	7591772	G	A	MCOLN1	SILENT	ENSG00000090674:[ENST00000264079(c.531G>A,ccG>ccA):ENSP00000264079(p.Pro177(=))]	NA	NA	2.00E-04	1.07E-04	5.45E-04	2.00E-04	0.00E+00	NA	NA	NA	MODIFIER	NA	NA	184	180	0	162	73	87	53.7
Non-user	IOB_24	chr19	7687308	C	T	XAB2	MISSENSE	ENSG00000076924:[ENST00000534844(c.1517G>A,cGc>cAc):ENSP00000438225(p.Arg506His)]	NA	NA	NA	1.65E-05	0.00E+00	NA	NA	D	PoD	NA	MODERATE	D	1	329	325	0	260	253	6	2.31
Non-user	IOB_24	chr19	7712585	T	G	STXBP2	INTRONIC	ENSG00000076944:[ENST00000414284(c.1688-26T>G)]	NA	NA	chr19*7712585*7712585*T*A,G*rs794073*NA*NA*NA	6.34E-01	4.92E-01	NA	NA	NA	NA	NA	MODIFIER	NA	NA	38	37	0	36	24	11	30.56
Non-user	IOB_24	chr19	7935948	A	G	CTD-3193O13.11	EXONIC-NC	ENSG00000268120:[ENST00000597156(n.433A>G)]	NA	NA	3.99E-04	NA	NA	3.99E-04	0.00E+00	NA	NA	NA	MODIFIER	NA	NA	31	30	0	23	13	10	43.48
Non-user	IOB_24	chr19	7935948	A	G	CTD-3193O13.9	MISSENSE	ENSG00000183248:[ENST00000599142(c.426T>C,Tcc>Ccc):ENSP00000469043(p.Ser143Pro)]	NA	NA	3.99E-04	NA	NA	3.99E-04	0.00E+00	NA	NA	NA	MODIFIER	NA	NA	31	30	0	23	13	10	43.48
Non-user	IOB_24	chr19	7935948	A	G	FLJ22184	MISSENSE	80164:[NM_001190467.1(c.2181T>C,Tcc>Ccc):NP_001177396.1(p.Ser728Pro)]	NA	NA	3.99E-04	NA	NA	3.99E-04	0.00E+00	NA	NA	NA	MODIFIER	NA	NA	31	30	0	23	13	10	43.48
Non-user	IOB_24	chr19	7935948	A	G	PRR36	MISSENSE	80164:[NM_001190467.1(c.2179T>C,Tcc>Ccc):NP_001177396.1(p.Ser727Pro)]	NA	NA	3.99E-04	NA	NA	3.99E-04	0.00E+00	NA	NA	NA	MODIFIER	NA	NA	31	30	0	23	13	10	43.48
Non-user	IOB_24	chr19	8430919	C	T	ANGPTL4	MISSENSE	ENSG00000167772:[ENST00000594875(c.324C>T,Cac>Tac):ENSP00000472869(p.His109Tyr)]	NA	NA	NA	NA	NA	NA	NA	NA	NA	NA	MODIFIER	NA	NA	185	183	1	148	84	63	42.57
Non-user	IOB_24	chr19	9015846	A	C	MUC16	INTRONIC	94025:[XM_005260162.1(c.13820-101T>G)]	NA	NA	NA	NA	NA	NA	NA	NA	NA	NA	MODIFIER	NA	NA	28	28	0	27	13	14	51.85
Non-user	IOB_24	chr19	9049982	C	A	MUC16	MISSENSE	ENSG00000181143:[ENST00000397910(c.31649G>T,aGc>aTc):ENSP00000381008(p.Ser10550Ile)]	NA	NA	chr19*9049982*9049982*C*T*rs778732831*NA*NA*NA	NA	NA	NA	NA	NA	PrD	NA	MODERATE	NA	NA	165	164	0	150	143	5	3.33
Non-user	IOB_24	chr19	9646944	A	G	ZNF426	5UTR	79088:[XM_005260077.1(c.-679T>C)]	NA	NA	1.60E-03	1.20E-03	8.20E-03	1.60E-03	8.20E-03	NA	NA	NA	MODIFIER	NA	NA	217	216	0	162	79	83	51.23
Non-user	IOB_24	chr19	10129338	G	C	RDH8	NCT	ENSG00000080511:[ENST00000589570(n.50+1447G>C)]	NA	NA	9.98E-03	NA	NA	9.98E-03	5.01E-02	NA	NA	NA	MODIFIER	NA	NA	74	74	0	48	23	25	52.08
Non-user	IOB_24	chr19	10662902	C	T	ATG4D	MISSENSE	ENSG00000130734:[ENST00000588972(c.253C>T,Cgc>Tgc):ENSP00000466759(p.Arg85Cys)]	NA	chr19*10662902*10662902*COSM1390149*C*T*1*large_intestine*1	7.99E-04	2.23E-04	1.33E-03	7.99E-04	3.10E-03	NA	NA	NA	MODIFIER	NA	NA	282	280	0	199	97	102	51.26
Non-user	IOB_24	chr19	10823901	C	A	QTRT1	SILENT	ENSG00000213339:[ENST00000250237(c.1167C>A,acC>acA):ENSP00000250237(p.Thr389(=))]	NA	NA	chr19*10823901*10823901*C*G*rs748357640*NA*NA*NA	NA	NA	NA	NA	NA	NA	NA	LOW	NA	NA	151	151	0	91	85	5	5.49
Non-user	IOB_24	chr19	11149020	C	T	SMARCA4	NCT	ENSG00000127616:[ENST00000592604(n.1985-2954C>T)]	NA	NA	NA	NA	NA	NA	NA	NA	NA	NA	MODIFIER	NA	NA	78	77	0	44	23	21	47.73
Non-user	IOB_24	chr19	11169069	A	G	SMARCA4	INTRONIC	6597:[XM_005260026.1(c.4638+30A>G)]	NA	NA	5.39E-03	5.90E-03	3.10E-02	5.39E-03	2.76E-02	NA	NA	NA	MODIFIER	NA	NA	239	239	0	223	120	102	45.74
Non-user	IOB_24	chr19	11559696	G	A	PRKCSH	INTRONIC	ENSG00000130175:[ENST00000592741(c.1287-33G>A)]	NA	NA	3.69E-02	3.95E-02	5.00E-02	3.69E-02	5.11E-02	NA	NA	NA	MODIFIER	NA	NA	192	192	0	204	102	98	48.04
Non-user	IOB_24	chr19	11624708	C	T	ECSIT	MISSENSE	ENSG00000130159:[ENST00000593231(c.78G>A,cGg>cAg):ENSP00000464783(p.Arg27Gln)]	NA	NA	5.19E-03	2.84E-03	2.08E-02	5.19E-03	2.66E-02	T	BN	NA	MODERATE	D	1	214	212	1	194	112	81	41.75
Non-user	IOB_24	chr19	11943687	A	T	ZNF440	MISSENSE	ENSG00000171295:[ENST00000304060(c.1696A>T,Atg>Ttg):ENSP00000305373(p.Met566Leu)]	NA	NA	1.40E-03	8.36E-04	5.94E-03	1.40E-03	7.20E-03	NA	NA	NA	MODERATE	NA	NA	169	169	0	137	62	73	53.28
Non-user	IOB_24	chr19	12358083	T	G	ZNF44	INTRONIC	ENSG00000197857:[ENST00000600003(c.336-2595A>C)]	NA	NA	NA	2.08E-04	1.03E-03	NA	NA	NA	NA	NA	MODIFIER	NA	NA	15	15	0	15	8	7	46.67
Non-user	IOB_24	chr19	12383704	C	T	ZNF44	MISSENSE	ENSG00000197857:[ENST00000356109(c.1510G>A,Gct>Act):ENSP00000348419(p.Ala504Thr)]	NA	NA	7.99E-04	2.24E-04	1.64E-03	7.99E-04	4.10E-03	NA	NA	NA	MODERATE	NA	NA	133	133	0	92	57	35	38.04
Non-user	IOB_24	chr19	12575309	T	C	CTD-3105H18.18	INTRONIC	ENSG00000269755:[ENST00000598753(c.188+1967A>G)]	NA	NA	NA	NA	NA	NA	NA	NA	NA	NA	MODIFIER	NA	NA	225	221	1	163	88	75	46.01
Non-user	IOB_24	chr19	12575309	T	C	ZNF709	MISSENSE	ENSG00000242852:[ENST00000397732(c.1427A>G,tAt>tGt):ENSP00000380840(p.Tyr476Cys)]	NA	NA	NA	NA	NA	NA	NA	NA	NA	NA	MODIFIER	NA	NA	225	221	1	163	88	75	46.01
Non-user	IOB_24	chr19	12787991	G	A	DHPS	SILENT	ENSG00000095059:[ENST00000598246(c.205C>T,gcC>gcT):ENSP00000471466(p.Ala69(=))]	NA	NA	NA	8.24E-06	6.06E-05	NA	NA	NA	NA	NA	MODIFIER	NA	NA	122	122	0	75	40	34	45.33
Non-user	IOB_24	chr19	13033592	C	T	FARSA	SILENT	ENSG00000179115:[ENST00000423140(c.1404G>A,ccG>ccA):ENSP00000396548(p.Pro468(=))]	NA	NA	NA	8.24E-06	6.06E-05	NA	NA	NA	NA	NA	LOW	NA	NA	95	92	0	69	35	34	49.28
Non-user	IOB_24	chr19	13317506	T	A	CACNA1A	3UTR	ENSG00000141837:[ENST00000360228(c.*621A>T)]	NA	NA	4.99E-03	NA	NA	4.99E-03	8.20E-03	NA	NA	NA	MODIFIER	NA	NA	39	39	0	41	13	28	68.29
Non-user	IOB_24	chr19	13879386	T	C	MRI1	MISSENSE	ENSG00000037757:[ENST00000040663(c.559T>C,Tca>Cca):ENSP00000040663(p.Ser187Pro)]	NA	NA	NA	1.37E-05	8.61E-05	NA	NA	NA	NA	NA	MODIFIER	NA	NA	191	191	0	158	83	75	47.47
Non-user	IOB_24	chr19	14165204	G	C	PALM3	MISSENSE	ENSG00000187867:[ENST00000340790(c.1235C>G,aCa>aGa):ENSP00000344996(p.Thr412Arg)]	NA	NA	3.38E-02	6.47E-02	2.01E-02	3.37E-02	1.12E-02	T_lc	PoD	NA	MODERATE	NA	NA	253	251	0	195	101	94	48.21
Non-user	IOB_24	chr19	14499537	A	G	CD97	MISSENSE	ENSG00000123146:[ENST00000242786(c.97A>G,Aac>Gac):ENSP00000242786(p.Asn33Asp)]	NA	NA	NA	3.28E-03	1.59E-02	NA	NA	NA	NA	NA	MODERATE	NA	NA	140	138	1	91	48	42	46.15
Non-user	IOB_24	chr19	14584153	G	A	PTGER1	INTRONIC	ENSG00000160951:[ENST00000292513(c.942+38C>T)]	NA	NA	chr19*14584153*14584153*G*C*rs758964471*NA*NA*NA	NA	NA	NA	NA	NA	NA	NA	MODIFIER	NA	NA	125	124	0	123	65	57	46.34
Non-user	IOB_24	chr19	14674972	T	C	TECR	NCT	ENSG00000099797:[ENST00000601461(n.468-22T>C)]	NA	NA	NA	8.25E-06	6.06E-05	NA	NA	NA	NA	NA	MODIFIER	NA	NA	210	210	0	163	93	67	41.1
Non-user	IOB_24	chr19	14674973	G	T	TECR	NCT	ENSG00000099797:[ENST00000597607(n.424-21G>T)]	NA	NA	NA	8.25E-06	6.06E-05	NA	NA	NA	NA	NA	MODIFIER	NA	NA	211	211	0	163	95	66	40.49
Non-user	IOB_24	chr19	14674974	A	C	TECR	NCT	9524:[NR_038104.1(n.579-20A>C)]	NA	NA	NA	8.25E-06	6.06E-05	NA	NA	NA	NA	NA	MODIFIER	NA	NA	210	207	0	164	93	68	41.46
Non-user	IOB_24	chr19	14703150	A	G	CLEC17A	MISSENSE	ENSG00000187912:[ENST00000339847(c.257A>G,aAg>aGg):ENSP00000341620(p.Lys86Arg)]	NA	NA	2.70E-02	4.82E-02	7.64E-02	2.70E-02	6.75E-02	T	PoD	NA	MODERATE	NA	NA	136	133	0	126	65	59	46.83
Non-user	IOB_24	chr19	14707785	G	A	CLEC17A	SILENT	ENSG00000187912:[ENST00000397439(c.492G>A,ctG>ctA):ENSP00000380581(p.Leu164(=))]	NA	NA	1.60E-03	1.47E-03	1.06E-02	1.60E-03	8.20E-03	NA	NA	NA	LOW	NA	NA	82	82	0	59	32	27	45.76
Non-user	IOB_24	chr19	14854373	T	C	EMR2	INTRONIC	30817:[XM_005259884.1(c.2560-31A>G)]	NA	NA	1.40E-03	1.16E-03	8.48E-03	1.40E-03	7.20E-03	NA	NA	NA	MODIFIER	NA	NA	185	180	4	121	67	52	42.98
Non-user	IOB_24	chr19	15133803	G	A	CCDC105	MISSENSE	ENSG00000160994:[ENST00000292574(c.1372G>A,Ggc>Agc):ENSP00000292574(p.Gly458Ser)]	NA	NA	3.32E-02	2.07E-02	3.34E-02	3.31E-02	5.42E-02	T	BN	NA	MODERATE	N	0	497	496	0	421	222	197	46.79
Non-user	IOB_24	chr19	15350185	A	G	BRD4	INTRONIC	ENSG00000141867:[ENST00000263377(c.3576+18T>C)]	NA	NA	4.99E-03	2.04E-03	1.48E-02	4.99E-03	2.56E-02	NA	NA	NA	MODIFIER	NA	NA	179	179	0	159	102	56	35.22
Non-user	IOB_24	chr19	15651301	G	A	CYP4F22	MISSENSE	ENSG00000171954:[ENST00000269703(c.712G>A,Gct>Act):ENSP00000269703(p.Ala238Thr)]	NA	NA	9.98E-04	8.76E-04	6.30E-03	9.98E-04	5.10E-03	T	BN	NA	MODERATE	N	0	206	206	0	146	76	67	45.89
Non-user	IOB_24	chr19	15733914	G	C	CYP4F8	INTRONIC-SS-ACR	11283:[NM_007253.3(c.645-1G>C)]	NA	NA	4.39E-03	2.72E-03	1.99E-02	4.39E-03	2.15E-02	NA	NA	NA	HIGH	NA	NA	111	111	0	87	47	39	44.83
Non-user	IOB_24	chr19	15784519	T	C	CYP4F12	SILENT	ENSG00000186204:[ENST00000324632(c.180T>C,ttT>ttC):ENSP00000321821(p.Phe60(=))]	NA	NA	NA	1.50E-04	1.09E-03	NA	NA	NA	NA	NA	MODIFIER	NA	NA	257	256	0	203	99	102	50.25
Non-user	IOB_24	chr19	15807212	T	G	CYP4F12	INTRONIC	66002:[XM_005260038.1(c.745-28T>G)]	NA	NA	NA	2.45E-01	2.37E-01	NA	NA	NA	NA	NA	MODIFIER	NA	NA	83	83	0	68	40	28	41.18
Non-user	IOB_24	chr19	15989730	T	C	CYP4F2	MISSENSE	ENSG00000186115:[ENST00000221700(c.1414A>G,Acg>Gcg):ENSP00000221700(p.Thr472Ala)]	NA	chr19*15989730*15989730*COSM230368*T*C*1*NS*1	NA	3.69E-01	2.84E-01	NA	NA	NA	NA	NA	MODIFIER	NA	NA	55	55	0	58	51	7	12.07
Non-user	IOB_24	chr19	16513313	T	A	EPS15L1	INTRONIC	ENSG00000127527:[ENST00000248070(c.1627-17A>T)]	NA	NA	5.39E-03	1.30E-02	3.69E-03	5.39E-03	4.10E-03	NA	NA	NA	MODIFIER	NA	NA	98	96	0	82	50	32	39.02
Non-user	IOB_24	chr19	16910747	C	T	NWD1	SILENT	ENSG00000188039:[ENST00000523826(c.2892C>T,gtC>gtT):ENSP00000428955(p.Val964(=))]	NA	NA	3.40E-03	1.11E-02	4.40E-03	3.39E-03	5.10E-03	NA	NA	NA	MODIFIER	NA	NA	183	182	0	133	72	61	45.86
Non-user	IOB_24	chr19	17100550	A	T	CPAMD8	MISSENSE	ENSG00000160111:[ENST00000443236(c.1439T>A,gTg>gAg):ENSP00000402505(p.Val480Glu)]	NA	NA	1.02E-02	1.73E-02	2.28E-02	1.02E-02	2.66E-02	NA	NA	NA	MODIFIER	NA	NA	195	190	1	164	77	86	52.44
Non-user	IOB_24	chr19	17306074	G	A	MYO9B	MISSENSE	ENSG00000099331:[ENST00000397274(c.3838G>A,Ggc>Agc):ENSP00000380444(p.Gly1280Ser)]	NA	chr19*17306074*17306074*COSM1717744*G*A*1*adrenal_gland*1|chr19*17306074*17306074*COSM1717745*G*A*1*adrenal_gland*1	3.79E-03	1.26E-02	5.11E-03	3.79E-03	3.10E-03	T	BN	NA	MODERATE	NA	NA	287	282	0	258	134	118	45.74
Non-user	IOB_24	chr19	17397497	G	T	ANKLE1	SILENT	ENSG00000160117:[ENST00000598347(c.1767G>T,gtG>gtT):ENSP00000470895(p.Val589(=))]	NA	NA	NA	1.78E-03	0.00E+00	NA	NA	NA	NA	NA	MODIFIER	NA	NA	76	73	0	21	12	8	38.1
Non-user	IOB_24	chr19	17631821	G	A	PGLS	SILENT	ENSG00000130313:[ENST00000252603(c.708G>A,ctG>ctA):ENSP00000252603(p.Leu236(=))]	NA	NA	1.52E-02	5.91E-02	2.32E-02	1.52E-02	1.64E-02	NA	NA	NA	LOW	NA	NA	182	182	0	138	68	70	50.72
Non-user	IOB_24	chr19	17750217	G	C	UNC13A	INTRONIC	ENSG00000130477:[ENST00000252773(c.2946+28C>G)]	NA	NA	NA	NA	NA	NA	NA	NA	NA	NA	MODIFIER	NA	NA	52	51	0	46	23	23	50
Non-user	IOB_24	chr19	17937516	C	T	JAK3	3UTR	ENSG00000105639:[ENST00000458235(c.*36G>A)]	NA	NA	2.28E-02	1.16E-02	2.22E-02	2.28E-02	2.56E-02	NA	NA	NA	MODIFIER	NA	NA	110	108	0	96	62	34	35.42
Non-user	IOB_24	chr19	17952209	G	A	JAK3	SILENT	ENSG00000105639:[ENST00000458235(c.1131C>T,caC>caT):ENSP00000391676(p.His377(=))]	NA	NA	2.00E-03	7.95E-03	1.66E-02	2.00E-03	9.20E-03	NA	NA	NA	MODIFIER	NA	NA	142	142	0	108	62	45	41.67
Non-user	IOB_24	chr19	17994760	A	G	SLC5A5	SILENT	ENSG00000105641:[ENST00000222248(c.1431A>G,ccA>ccG):ENSP00000222248(p.Pro477(=))]	NA	NA	NA	5.91E-04	1.46E-03	NA	NA	NA	NA	NA	LOW	NA	NA	84	82	0	55	27	28	50.91
Non-user	IOB_24	chr19	18047392	C	T	CCDC124	INTRONIC-SS-DNR-PRX	ENSG00000007080:[ENST00000445755(c.159+4C>T)]	NA	NA	NA	1.10E-04	0.00E+00	NA	NA	NA	NA	NA	MODIFIER	NA	NA	273	271	1	290	155	134	46.21
Non-user	IOB_24	chr19	18545786	G	T	ISYNA1	SILENT	ENSG00000105655:[ENST00000457269(c.1452C>A,acC>acA):ENSP00000415458(p.Thr484(=))]	NA	NA	NA	2.12E-01	2.17E-01	NA	NA	NA	NA	NA	LOW	NA	NA	91	89	0	84	52	32	38.1
Non-user	IOB_24	chr19	18546369	G	T	ISYNA1	NCT-SS-PRX	ENSG00000105655:[ENST00000583816(n.770+4C>A)]	NA	NA	1.40E-03	2.56E-03	5.07E-04	1.40E-03	1.00E-03	NA	NA	NA	LOW	NA	NA	268	266	0	207	108	98	47.34
Non-user	IOB_24	chr19	18853713	C	T	CRTC1	INTRONIC-SS-ACR-PRX	ENSG00000105662:[ENST00000321949(c.127-7C>T)]	NA	NA	2.00E-04	9.59E-04	5.47E-04	2.00E-04	1.00E-03	NA	NA	NA	LOW	NA	NA	103	103	0	87	50	37	42.53
Non-user	IOB_24	chr19	18854816	T	G	CRTC1	INTRONIC	ENSG00000105662:[ENST00000594658(c.120+980T>G)]	NA	NA	9.98E-04	NA	NA	9.98E-04	5.10E-03	NA	NA	NA	MODIFIER	NA	NA	190	189	1	168	79	88	52.38
Non-user	IOB_24	chr19	19168557	T	A	ARMC6	3UTR	ENSG00000105676:[ENST00000535795(c.*253T>A)]	NA	NA	9.98E-04	4.36E-04	2.97E-03	9.98E-04	4.10E-03	NA	NA	NA	MODIFIER	NA	NA	184	148	0	103	36	44	42.72
Non-user	IOB_24	chr19	19230816	T	C	TMEM161A	MISSENSE	ENSG00000064545:[ENST00000587583(c.1288A>G,Atc>Gtc):ENSP00000467300(p.Ile430Val)]	NA	NA	4.85E-02	1.09E-01	1.11E-01	4.85E-02	6.95E-02	T	BN	NA	MODERATE	D	1	31	30	0	21	14	6	28.57
Non-user	IOB_24	chr19	21712442	G	T	ZNF429	MISSENSE	353088:[XM_005259892.1(c.49G>T,Ggt>Tgt):XP_005259949.1(p.Gly17Cys)]	NA	NA	1.14E-02	2.83E-02	7.59E-03	1.14E-02	0.00E+00	NA	NA	NA	MODIFIER	NA	NA	210	210	0	151	72	79	52.32
Non-user	IOB_24	chr19	23545516	G	C	ZNF91	MISSENSE	ENSG00000167232:[ENST00000300619(c.265C>G,Cat>Gat):ENSP00000300619(p.His89Asp)]	NA	NA	1.92E-02	3.82E-02	2.22E-02	1.92E-02	2.66E-02	NA	NA	NA	MODIFIER	NA	NA	97	97	0	67	33	34	50.75
Non-user	IOB_24	chr19	24309080	A	G	ZNF254	MISSENSE	ENSG00000213096:[ENST00000357002(c.278A>G,gAc>gGc):ENSP00000349494(p.Asp93Gly)]	NA	NA	9.98E-03	1.93E-02	2.29E-02	9.98E-03	1.74E-02	D	PoD	NA	MODERATE	NA	NA	81	80	1	68	37	31	45.59
Non-user	IOB_24	chr19	33444479	C	T	CEP89	INTRONIC	ENSG00000121289:[ENST00000593276(c.406+42G>A)]	NA	NA	NA	4.15E-01	3.23E-01	NA	NA	NA	NA	NA	MODIFIER	NA	NA	346	345	0	340	286	52	15.29
Non-user	IOB_24	chr19	33444542	G	A	CEP89	SILENT	ENSG00000121289:[ENST00000305768(c.471C>T,taC>taT):ENSP00000306105(p.Tyr157(=))]	NA	NA	NA	1.19E-03	1.43E-03	NA	NA	NA	NA	NA	LOW	NA	NA	337	335	1	368	337	31	8.42
Non-user	IOB_24	chr19	33444590	C	T	CEP89	SILENT	ENSG00000121289:[ENST00000593276(c.337G>A,ggG>ggA):ENSP00000467839(p.Gly113(=))]	NA	NA	chr19*33444590*33444590*C*A,T*rs78403716*NA*NA*NA	2.99E-02	9.08E-03	NA	NA	NA	NA	NA	LOW	NA	NA	273	270	0	279	242	29	10.39
Non-user	IOB_24	chr19	34583729	A	G	RPS4XP21	EXONIC-NC	ENSG00000186008:[ENST00000469064(n.487T>C)]	NA	NA	1.86E-02	5.20E-02	3.38E-02	1.86E-02	2.25E-02	NA	NA	NA	MODIFIER	NA	NA	83	83	0	73	34	39	53.42
Non-user	IOB_24	chr19	34857872	A	G	GPI	INTRONIC	ENSG00000105220:[ENST00000588991(c.327+116A>G)]	NA	NA	3.59E-03	3.47E-03	1.51E-02	3.59E-03	1.84E-02	NA	NA	NA	MODIFIER	NA	NA	146	145	0	102	46	56	54.9
Non-user	IOB_24	chr19	35448919	C	T	ZNF792	MISSENSE	ENSG00000180884:[ENST00000605484(c.1639G>A,Gct>Act):ENSP00000474130(p.Ala547Thr)]	NA	NA	1.98E-02	1.13E-02	7.85E-02	1.98E-02	9.20E-02	T	BN	NA	MODERATE	NA	NA	66	66	0	64	30	34	53.12

Patel *et al.* , 2021. Whole-exome sequencing analysis of oral squamous cell carcinoma delineated by tobacco usage habits
Supplementary Table 4. List of somatic single nucleotide variants identified using whole exome sequencing of OSCC patients

Cohort	Sample	Chromosome	Genomic position	Reference allele	Alternate allele	Genesymbol	Variant type	Variant description	OncoMD	COSMIC	dbSNP	ExAC	ExAC_SAS	1000G	1000G_SAS	SIFT	Polyphen2	CONDEL	VEP	LRT	PhastCon7Way	Total reads in Normal	Reference read support in Normal	Alternative read support in Normal	Total reads in Tumor	Reference read support in Tumor	Alternate read support in Tumor	Alternate allele frequency in Tumor
Non-user	IOB_24	chr19	35448945	C	G	ZNF792	MISSENSE	ENSG00000180884:[ENST00000605484(c.1613G>C,aGc>aCc):ENSP00000474130(p.Ser538Thr)]	NA	NA	1.98E-02	1.12E-02	7.72E-02	1.98E-02	9.20E-02	T	BN	NA	MODERATE	NA	NA	89	89	0	70	36	34	48.57
Non-user	IOB_24	chr19	35451090	A	G	ZNF792	INTRONIC	ENSG00000180884:[ENST00000605484(c.82+49T>C)]	NA	NA	1.96E-02	1.06E-02	7.20E-02	1.96E-02	9.10E-02	NA	NA	NA	MODIFIER	NA	NA	52	52	0	57	29	27	47.37
Non-user	IOB_24	chr19	35451839	G	A	ZNF792	SILENT	ENSG00000180884:[ENST00000404801(c.93C>T,ctC>ctT):ENSP00000385099(p.Leu31(=))]	NA	chr19*35451839*35451839*COSM712031*G*T*1*lung*1	1.78E-02	9.58E-03	6.48E-02	1.78E-02	8.28E-02	NA	NA	NA	LOW	NA	NA	187	186	1	174	79	95	54.6
Non-user	IOB_24	chr19	35741314	C	T	LSR	MISSENSE	ENSG00000105699:[ENST00000605618(c.227C>T,tCg>tTg):ENSP00000474797(p.Ser76Leu)]	NA	NA	1.40E-03	5.98E-04	4.33E-03	1.40E-03	7.20E-03	D	BN	NA	MODERATE	N	0	369	367	2	309	143	163	52.75
Non-user	IOB_24	chr19	35757389	C	T	LSR	SILENT	ENSG00000105699:[ENST00000361790(c.1050C>T,aaC>aaT):ENSP00000354575(p.Asn350(=))]	NA	NA	1.40E-03	5.82E-04	4.52E-03	1.40E-03	7.20E-03	NA	NA	NA	LOW	NA	0.002	127	124	0	100	46	52	52
Non-user	IOB_24	chr19	35828773	C	T	CD22	SILENT	ENSG00000012124:[ENST00000419549(c.318C>T,tcC>tcT):ENSP00000403822(p.Ser106(=))]	NA	NA	2.92E-02	1.37E-02	6.66E-02	2.92E-02	9.30E-02	NA	NA	NA	LOW	NA	NA	224	223	0	207	109	96	46.38
Non-user	IOB_24	chr19	36207510	G	A	ZBTB32	SILENT	ENSG00000011590:[ENST00000262630(c.1320G>A,ctG>ctA):ENSP00000262630(p.Leu440(=))]	NA	NA	4.17E-02	1.01E-01	4.15E-02	4.17E-02	3.17E-02	NA	NA	NA	MODIFIER	NA	NA	378	377	0	228	122	104	45.61
Non-user	IOB_24	chr19	36339247	C	T	NPHS1	MISSENSE	ENSG00000161270:[ENST00000353632(c.1223G>A,cGg>cAg):ENSP00000343634(p.Arg408Gln)]	NA	NA	2.06E-02	4.73E-02	2.57E-02	2.06E-02	1.64E-02	D	PrD	NA	MODERATE	D	0.331	216	213	0	201	99	101	50.25
Non-user	IOB_24	chr19	36355661	G	A	KIRREL2	INTRONIC	84063:[XM_005259308.1(c.1731+46G>A)]	NA	NA	9.19E-03	9.78E-03	2.55E-03	9.19E-03	2.00E-03	NA	NA	NA	MODIFIER	NA	NA	69	68	0	45	29	16	35.56
Non-user	IOB_24	chr19	36355661	G	A	NPHS1	NCT	ENSG00000161270:[ENST00000591817(n.559+1728C>T)]	NA	NA	9.19E-03	9.78E-03	2.55E-03	9.19E-03	2.00E-03	NA	NA	NA	MODIFIER	NA	NA	69	68	0	45	29	16	35.56
Non-user	IOB_24	chr19	36592287	G	A	WDR62	3UTR	ENSG00000075702:[ENST00000587391(c.*1729G>A)]	NA	NA	2.60E-03	NA	NA	2.60E-03	2.00E-03	NA	NA	NA	MODIFIER	NA	NA	149	149	0	133	77	56	42.11
Non-user	IOB_24	chr19	38014368	A	T	ZNF793	INTRONIC	ENSG00000188227:[ENST00000445217(c.15+12A>T)]	NA	NA	5.99E-04	9.54E-04	3.22E-03	5.99E-04	0.00E+00	NA	NA	NA	MODIFIER	NA	NA	223	222	0	141	66	73	51.77
Non-user	IOB_24	chr19	38229926	G	A	ZNF573	MISSENSE	ENSG00000189144:[ENST00000339503(c.1291C>T,Cgg>Tgg):ENSP00000340171(p.Arg431Trp)]	NA	NA	2.96E-02	4.76E-02	1.13E-01	2.96E-02	8.79E-02	NA	NA	NA	MODIFIER	NA	NA	78	75	0	43	26	16	37.21
Non-user	IOB_24	chr19	38384636	A	T	WDR87	SILENT	ENSG00000171804:[ENST00000447313(c.1707T>A,tcT>tcA):ENSP00000405012(p.Ser569(=))]	NA	NA	5.99E-04	1.92E-03	2.15E-03	5.99E-04	1.00E-03	NA	NA	NA	LOW	NA	NA	131	131	0	92	40	52	56.52
Non-user	IOB_24	chr19	38573347	T	C	SIPA1L3	MISSENSE	ENSG00000105738:[ENST00000222345(c.1142T>C,aTg>aCg):ENSP00000222345(p.Met381Thr)]	NA	NA	1.50E-02	2.04E-02	3.63E-02	1.50E-02	3.37E-02	T	BN	NA	MODERATE	N	0.001	450	448	1	371	198	170	45.82
Non-user	IOB_24	chr19	38609940	C	T	SIPA1L3	NCT-SS-PRX	ENSG00000105738:[ENST00000594553(n.187-6C>T)]	NA	NA	7.99E-04	1.28E-03	2.38E-03	7.99E-04	1.00E-03	NA	NA	NA	LOW	NA	NA	321	319	1	241	141	100	41.49
Non-user	IOB_24	chr19	38706886	G	A	DPF1	SILENT	ENSG00000011332:[ENST00000420980(c.741C>T,ccC>ccT):ENSP00000397354(p.Pro247(=))]	NA	NA	1.98E-02	2.21E-02	5.14E-02	1.98E-02	4.70E-02	NA	NA	NA	MODIFIER	NA	NA	95	95	0	107	43	64	59.81
Non-user	IOB_24	chr19	38910706	G	A	RASGRP4	INTRONIC	ENSG00000171777:[ENST00000293062(c.510-53C>T)]	NA	NA	1.58E-02	NA	NA	1.58E-02	2.04E-02	NA	NA	NA	MODIFIER	NA	NA	126	126	0	101	46	55	54.46
Non-user	IOB_24	chr19	38985308	G	A	RYR1	INTRONIC	ENSG00000196218:[ENST00000355481(c.6548+43G>A)]	NA	NA	3.16E-02	3.91E-02	2.15E-02	3.15E-02	2.15E-02	NA	NA	NA	MODIFIER	NA	NA	40	39	0	46	28	18	39.13
Non-user	IOB_24	chr19	39292925	G	T	LGALS4	NCT	ENSG00000171747:[ENST00000595342(n.219+31C>A)]	NA	NA	2.00E-04	7.75E-04	2.97E-03	2.00E-04	1.00E-03	NA	NA	NA	MODIFIER	NA	NA	65	64	0	51	33	18	35.29
Non-user	IOB_24	chr19	39440729	C	T	FBXO17	SILENT	ENSG00000269190:[ENST00000292852(c.231G>A,gtG>gtA):ENSP00000292852(p.Val77(=))]	NA	NA	NA	2.24E-04	5.05E-04	NA	NA	NA	NA	NA	LOW	NA	NA	605	601	1	471	248	223	47.35
Non-user	IOB_24	chr19	39663668	G	A	PAK4	SILENT	ENSG00000130669:[ENST00000321944(c.315G>A,ccG>ccA):ENSP00000326864(p.Pro105(=))]	NA	NA	NA	4.16E-04	5.10E-04	NA	NA	NA	NA	NA	MODIFIER	NA	NA	107	106	0	78	32	45	57.69
Non-user	IOB_24	chr19	39735548	T	A	IFNL3	SILENT	ENSG00000197110:[ENST00000413851(c.60A>T,ggA>ggT):ENSP00000409000(p.Gly20(=))]	NA	NA	8.39E-03	1.26E-02	1.41E-02	8.39E-03	1.94E-02	NA	NA	NA	LOW	NA	NA	63	61	0	42	18	24	57.14
Non-user	IOB_24	chr19	39759370	G	A	IFNL2	MISSENSE	ENSG00000183709:[ENST00000331982(c.64G>A,Gtg>Atg):ENSP00000333639(p.Val22Met)]	NA	NA	NA	1.07E-04	4.85E-04	NA	NA	T	BN	NA	MODERATE	N	0	55	55	0	49	27	22	44.9
Non-user	IOB_24	chr19	39879705	C	T	PAF1	INTRONIC	ENSG00000006712:[ENST00000416728(c.408+28G>A)]	NA	NA	4.99E-03	2.16E-03	1.58E-02	4.99E-03	2.56E-02	NA	NA	NA	MODIFIER	NA	NA	165	165	0	137	71	66	48.18
Non-user	IOB_24	chr19	39914456	C	T	PLEKHG2	MISSENSE	ENSG00000090924:[ENST00000205135(c.2286C>T,Cct>Tct):ENSP00000205135(p.Pro763Ser)]	NA	NA	2.20E-03	1.24E-03	8.72E-03	2.20E-03	1.12E-02	NA	NA	NA	MODIFIER	NA	NA	190	188	0	152	83	68	44.74
Non-user	IOB_24	chr19	39930394	C	T	AC011500.1	SILENT	ENSG00000213922:[ENST00000396843(c.183C>T,aaC>aaT):ENSP00000380055(p.Asn61(=))]	NA	NA	2.20E-03	4.36E-03	7.52E-03	2.20E-03	1.12E-02	NA	NA	NA	MODIFIER	NA	NA	250	250	0	209	121	87	41.63
Non-user	IOB_24	chr19	39930394	C	T	NA	EXONIC-NC	723805:[XR_158938.2(n.188C>T)]	NA	NA	2.20E-03	4.36E-03	7.52E-03	2.20E-03	1.12E-02	NA	NA	NA	MODIFIER	NA	NA	250	250	0	209	121	87	41.63
Non-user	IOB_24	chr19	39930394	C	T	SUPT5H	INTRONIC	ENSG00000196235:[ENST00000594990(c.-374-43C>T)]	NA	NA	2.20E-03	4.36E-03	7.52E-03	2.20E-03	1.12E-02	NA	NA	NA	MODIFIER	NA	NA	250	250	0	209	121	87	41.63
Non-user	IOB_24	chr19	39930487	C	T	AC011500.1	INTRONIC-SS-DNR-PRX	ENSG00000213922:[ENST00000396843(c.270+6C>T)]	NA	NA	2.20E-03	4.16E-03	7.27E-03	2.20E-03	1.12E-02	NA	NA	NA	LOW	NA	NA	216	215	1	182	101	75	41.21
Non-user	IOB_24	chr19	39930487	C	T	NA	NCT-SS-PRX	723805:[XR_158938.2(n.275+6C>T)]	NA	NA	2.20E-03	4.16E-03	7.27E-03	2.20E-03	1.12E-02	NA	NA	NA	LOW	NA	NA	216	215	1	182	101	75	41.21
Non-user	IOB_24	chr19	39930487	C	T	SUPT5H	INTRONIC-SS-DNR-PRX	ENSG00000196235:[ENST00000594990(c.-330+6C>T)]	NA	NA	2.20E-03	4.16E-03	7.27E-03	2.20E-03	1.12E-02	NA	NA	NA	LOW	NA	NA	216	215	1	182	101	75	41.21
Non-user	IOB_24	chr19	39948308	G	A	SUPT5H	NCT-SS-PRX	ENSG00000196235:[ENST00000601978(n.281-7G>A)]	NA	NA	2.40E-03	1.52E-03	1.05E-02	2.40E-03	1.12E-02	NA	NA	NA	LOW	NA	NA	166	165	0	105	54	51	48.57
Non-user	IOB_24	chr19	39949845	G	A	SUPT5H	SILENT	ENSG00000196235:[ENST00000432763(c.480G>A,gaG>gaA):ENSP00000404029(p.Glu160(=))]	NA	NA	2.60E-03	3.96E-03	1.57E-02	2.60E-03	1.33E-02	NA	NA	NA	LOW	NA	NA	83	81	0	73	41	28	38.36
Non-user	IOB_24	chr19	40478362	T	C	PSMC4	SILENT	ENSG00000013275:[ENST00000157812(c.222T>C,caT>caC):ENSP00000157812(p.His74(=))]	NA	NA	1.86E-02	2.66E-02	5.78E-02	1.86E-02	6.03E-02	NA	NA	NA	MODIFIER	NA	NA	156	156	0	96	44	52	54.17
Non-user	IOB_24	chr19	40554705	T	C	AC005614.5	INTRONIC-SS-ACR	ENSG00000269749:[ENST00000595508(n.422-2A>G)]	NA	NA	4.59E-03	8.56E-03	4.43E-03	4.59E-03	3.10E-03	NA	NA	NA	HIGH	NA	NA	391	390	0	308	155	150	48.7
Non-user	IOB_24	chr19	40554705	T	C	ZNF780B	INTRONIC-SS-ACR	ENSG00000128000:[ENST00000434248(c.10-2A>G)]	NA	NA	4.59E-03	8.56E-03	4.43E-03	4.59E-03	3.10E-03	NA	NA	NA	HIGH	NA	NA	391	390	0	308	155	150	48.7
Non-user	IOB_24	chr19	40964325	G	A	BLVRB	SILENT	ENSG00000090013:[ENST00000263368(c.207C>T,gaC>gaT):ENSP00000263368(p.Asp69(=))]	NA	chr19*40964325*40964325*COSM1393829*G*A*1*large_intestine*1	1.20E-03	3.19E-03	2.65E-03	1.20E-03	3.10E-03	NA	NA	NA	MODIFIER	NA	NA	99	98	1	66	36	30	45.45
Non-user	IOB_24	chr19	41327297	G	A	CTC-490E21.12	INTRONIC	ENSG00000268797:[ENST00000601627(c.119+19977G>A)]	NA	NA	1.04E-02	NA	NA	1.04E-02	2.56E-02	NA	NA	NA	MODIFIER	NA	NA	163	163	0	15	8	7	46.67
Non-user	IOB_24	chr19	41327297	G	A	CYP2F2P	NCT	ENSG00000237118:[ENST00000437853(n.873+90C>T)]	NA	NA	1.04E-02	NA	NA	1.04E-02	2.56E-02	NA	NA	NA	MODIFIER	NA	NA	163	163	0	15	8	7	46.67
Non-user	IOB_24	chr19	42092088	G	C	CEACAM21	INTRONIC	90273:[NM_001290113.1(c.464-105G>C)]	NA	NA	NA	NA	NA	NA	NA	NA	NA	NA	MODIFIER	NA	NA	61	61	0	77	47	29	37.66
Non-user	IOB_24	chr19	42126833	C	T	CEACAM4	INTRONIC	ENSG00000105352:[ENST00000221954(c.595+115G>A)]	NA	NA	1.04E-02	NA	NA	1.04E-02	1.43E-02	NA	NA	NA	MODIFIER	NA	NA	70	69	0	40	31	9	22.5
Non-user	IOB_24	chr19	42736706	C	T	GSK3A	SILENT	ENSG00000105723:[ENST00000398249(c.981G>A,ctG>ctA):ENSP00000381301(p.Leu327(=))]	NA	NA	3.99E-04	2.89E-04	2.07E-03	3.99E-04	2.00E-03	NA	NA	NA	MODIFIER	NA	NA	113	113	0	83	32	50	60.24
Non-user	IOB_24	chr19	42788967	G	A	CIC	INTRONIC	ENSG00000079432:[ENST00000160740(c.67+44G>A)]	NA	NA	4.79E-03	7.47E-03	1.35E-02	4.79E-03	2.45E-02	NA	NA	NA	MODIFIER	NA	NA	72	71	0	74	44	30	40.54
Non-user	IOB_24	chr19	42930888	C	T	LIPE	SILENT	ENSG00000079435:[ENST00000244289(c.414G>A,caG>caA):ENSP00000244289(p.Gln138(=))]	NA	NA	NA	NA	NA	NA	NA	NA	NA	NA	MODIFIER	NA	NA	163	163	0	133	76	56	42.11
Non-user	IOB_24	chr19	42930888	C	T	LIPE-AS1	NCT	ENSG00000213904:[ENST00000457234(n.936+1532C>T)]	NA	NA	NA	NA	NA	NA	NA	NA	NA	NA	MODIFIER	NA	NA	163	163	0	133	76	56	42.11
Non-user	IOB_24	chr19	43999432	A	T	PHLDB3	SILENT	ENSG00000176531:[ENST00000292140(c.1011T>A,tcT>tcA):ENSP00000292140(p.Ser337(=))]	NA	NA	2.80E-02	1.19E-02	2.61E-02	2.80E-02	3.17E-02	NA	NA	NA	LOW	NA	NA	95	95	0	69	33	36	52.17
Non-user	IOB_24	chr19	44251759	C	T	SMG9	INTRONIC	ENSG00000105771:[ENST00000597598(c.102+26G>A)]	NA	NA	4.45E-02	5.13E-02	3.26E-02	4.45E-02	2.35E-02	NA	NA	NA	MODIFIER	NA	NA	114	114	0	102	48	53	51.96
Non-user	IOB_24	chr19	44585249	T	C	ZNF223	3UTR	ENSG00000267022:[ENST00000591793(c.*143T>C)]	NA	chr19*44585249*44585249*COSM3692841*T*C*1*large_intestine*1	3.00E-03	4.01E-03	1.44E-02	3.00E-03	1.53E-02	D	PrD	NA	MODERATE	NA	NA	225	224	0	157	76	81	51.59
Non-user	IOB_24	chr19	44585249	T	C	ZNF284	MISSENSE	ENSG00000186026:[ENST00000421176(c.97T>C,Tat>Cat):ENSP00000411032(p.Tyr33His)]	NA	chr19*44585249*44585249*COSM3692841*T*C*1*large_intestine*1	3.00E-03	4.01E-03	1.44E-02	3.00E-03	1.53E-02	D	PrD	NA	MODERATE	NA	NA	225	224	0	157	76	81	51.59
Non-user	IOB_24	chr19	44832737	C	T	ZFP112	MISSENSE	7771:[XM_005259238.1(c.1390G>A,Ggc>Agc):XP_005259295.1(p.Gly464Ser)]	NA	NA	7.99E-04	5.27E-04	3.45E-03	7.99E-04	3.10E-03	NA	NA	NA	MODERATE	NA	0	326	325	0	206	106	99	48.06
Non-user	IOB_24	chr19	44832737	C	T	ZNF112	MISSENSE	ENSG00000062370:[ENST00000354340(c.1573G>A,Ggc>Agc):ENSP00000346305(p.Gly525Ser)]	NA	NA	7.99E-04	5.27E-04	3.45E-03	7.99E-04	3.10E-03	NA	NA	NA	MODERATE	NA	0	326	325	0	206	106	99	48.06
Non-user	IOB_24	chr19	45176028	C	T	CEACAM19	SILENT	ENSG00000186567:[ENST00000358777(c.216C>T,taC>taT):ENSP00000351627(p.Tyr72(=))]	NA	NA	chr19*45176028*45176028*C*A,T*rs35695969*NA*NA*NA	5.22E-02	6.08E-02	NA	NA	NA	NA	NA	LOW	NA	0	218	216	0	156	90	64	41.03
Non-user	IOB_24	chr19	45176028	C	T	CTB-171A8.1	NCT	ENSG00000266903:[ENST00000590796(n.408+26326G>A)]	NA	NA	chr19*45176028*45176028*C*A,T*rs35695969*NA*NA*NA	5.22E-02	6.08E-02	NA	NA	NA	NA	NA	LOW	NA	0	218	216	0	156	90	64	41.03
Non-user	IOB_24	chr19	45296894	G	A	CBLC	INTRONIC	ENSG00000142273:[ENST00000341505(c.1146+17G>A)]	NA	NA	1.70E-02	4.30E-02	5.47E-02	1.70E-02	3.99E-02	NA	NA	NA	MODIFIER	NA	NA	52	52	0	26	13	11	42.31
Non-user	IOB_24	chr19	45297479	C	T	CBLC	MISSENSE	ENSG00000142273:[ENST00000270279(c.1303C>T,Cca>Tca):ENSP00000270279(p.Pro435Ser)]	NA	NA	3.89E-02	3.66E-02	5.87E-02	3.89E-02	4.81E-02	T	BN	NA	MODERATE	NA	NA	149	148	0	87	59	27	31.03
Non-user	IOB_24	chr19	45321905	G	A	BCAM	INTRONIC	ENSG00000187244:[ENST00000270233(c.1194+11G>A)]	NA	NA	3.87E-02	2.65E-02	4.05E-02	3.87E-02	3.48E-02	NA	NA	NA	MODIFIER	NA	NA	143	143	0	92	46	45	48.91
Non-user	IOB_24	chr19	45731339	G	C	EXOC3L2	INTRONIC-SS-ACR-PRX	ENSG00000130201:[ENST00000252482(c.193-7C>G)]	NA	NA	2.32E-02	1.81E-02	2.74E-02	2.32E-02	2.25E-02	NA	NA	NA	LOW	NA	NA	105	105	0	49	0	48	97.96
Non-user	IOB_24	chr19	45731339	G	C	MARK4	INTRONIC	ENSG00000007047:[ENST00000587566(c.-276-30908G>C)]	NA	NA	2.32E-02	1.81E-02	2.74E-02	2.32E-02	2.25E-02	NA	NA	NA	LOW	NA	NA	105	105	0	49	0	48	97.96
Non-user	IOB_24	chr19	45731348	C	T	EXOC3L2	INTRONIC	ENSG00000130201:[ENST00000252482(c.193-16G>A)]	NA	NA	2.34E-02	1.82E-02	2.74E-02	2.34E-02	2.25E-02	NA	NA	NA	MODIFIER	NA	NA	107	107	0	50	0	49	98
Non-user	IOB_24	chr19	45731348	C	T	MARK4	INTRONIC	ENSG00000007047:[ENST00000587566(c.-276-30899C>T)]	NA	NA	2.34E-02	1.82E-02	2.74E-02	2.34E-02	2.25E-02	NA	NA	NA	MODIFIER	NA	NA	107	107	0	50	0	49	98
Non-user	IOB_24	chr19	45731515	G	T	EXOC3L2	SILENT	ENSG00000130201:[ENST00000252482(c.100C>A,Cgg>Agg):ENSP00000252482(p.Arg34(=))]	NA	NA	2.32E-02	1.82E-02	2.73E-02	2.32E-02	2.25E-02	NA	NA	NA	LOW	NA	0.124	92	92	0	66	1	65	98.48
Non-user	IOB_24	chr19	45731515	G	T	MARK4	INTRONIC	ENSG00000007047:[ENST00000587566(c.-276-30732G>T)]	NA	NA	2.32E-02	1.82E-02	2.73E-02	2.32E-02	2.25E-02	NA	NA	NA	LOW	NA	0.124	92	92	0	66	1	65	98.48
Non-user	IOB_24	chr19	45856100	G	A	ERCC2	NCT	ENSG00000104884:[ENST00000588652(n.1920-26C>T)]	NA	NA	2.70E-02	4.98E-02	5.05E-02	2.70E-02	4.09E-02	NA	NA	NA	MODIFIER	NA	NA	117	116	0	91	46	44	48.35
Non-user	IOB_24	chr19	45867799	G	A	ERCC2	MISSENSE	ENSG00000104884:[ENST00000391944(c.367C>T,Cat>Tat):ENSP00000375808(p.His123Tyr)]	NA	NA	5.99E-04	6.44E-04	3.23E-03	5.99E-04	2.00E-03	T	BN	NA	MODERATE	NA	NA	39	39	0	42	20	22	52.38
Non-user	IOB_24	chr19	46145661	C	T	C19orf83	MISSENSE	ENSG00000267757:[ENST00000593161(c.202C>T,Cca>Tca):ENSP00000464956(p.Pro68Ser)]	NA	NA	2.92E-02	8.44E-02	1.06E-01	2.92E-02	7.26E-02	NA	NA	NA	MODIFIER	NA	NA	185	185	0	145	72	72	49.66
Non-user	IOB_24	chr19	46145661	C	T	EML2	NCT	ENSG00000125746:[ENST00000585386(n.78-51G>A)]	NA	NA	2.92E-02	8.44E-02	1.06E-01	2.92E-02	7.26E-02	NA	NA	NA	MODIFIER	NA	NA	185	185	0	145	72	72	49.66
Non-user	IOB_24	chr19	46145661	C	T	EML2-AS1	EXONIC-NC	100287177:[NR_130728.1(n.646C>T)]	NA	NA	2.92E-02	8.44E-02	1.06E-01	2.92E-02	7.26E-02	NA	NA	NA	MODIFIER	NA	NA	185	185	0	145	72	72	49.66
Non-user	IOB_24	chr19	46258024	A	G	AC074212.3	INTRONIC	ENSG00000237452:[ENST00000457052(c.802+42A>G)]	NA	NA	chr19*46258024*46258024*A*C,G,T*rs918164*NA*NA*NA	5.27E-01	5.86E-01	NA	NA	NA	NA	NA	MODIFIER	NA	NA	47	47	0	37	19	18	48.65
Non-user	IOB_24	chr19	46258024	A	G	BHMG1	NCT	388553:[XR_243931.1(n.1212+42A>G)]	NA	NA	chr19*46258024*46258024*A*C,G,T*rs918164*NA*NA*NA	5.27E-01	5.86E-01	NA	NA	NA	NA	NA	MODIFIER	NA	NA	47	47	0	37	19	18	48.65
Non-user	IOB_24	chr19	46280917	G	A	DMPK	NCT-SS-PRX	1760:[XR_243910.1(n.1020+8C>T)]	NA	NA	5.99E-03	5.91E-03	1.77E-02	5.99E-03	1.12E-02	NA	NA	NA	LOW	NA	NA	271	271	0	264	122	139	52.65
Non-user	IOB_24	chr19	48219893	C	T	CTD-2571L23.8	NCT	ENSG00000268746:[ENST00000599924(n.87-15429C>T)]	NA	NA	3.44E-02	1.56E-01	9.46E-02	3.43E-02	3.37E-02	NA	NA	NA	MODIFIER	NA	NA	141	139	1	145	80	65	44.83
Non-user	IOB_24	chr19	48219893	C	T	EHD2	SILENT	ENSG00000024422:[ENST00000263277(c.24C>T,ggC>ggT):ENSP00000263277(p.Gly8(=))]	NA	NA	3.44E-02	1.56E-01	9.46E-02	3.43E-02	3.37E-02	NA	NA	NA	MODIFIER	NA	NA	141	139	1	145	80	65	44.83
Non-user	IOB_24	chr19	48591981	T	C	PLA2G4C	INTRONIC	ENSG00000105499:[ENST00000354276(c.764-12A>G)]	NA	NA	1.40E-03	8.33E-04	5.47E-04	1.40E-03	0.00E+00	NA	NA	NA	MODIFIER	NA	NA	117	116	0	81	40	40	49.38
Non-user	IOB_24	chr19	48640231	C	A	LIG1	NCT	3978:[NR_135499.1(n.1661+34G>T)]	NA	NA	2.20E-03	1.38E-03	8.28E-04	2.20E-03	0.00E+00	NA	NA	NA	MODIFIER	NA	NA	106	106	0	105	60	44	41.9
Non-user	IOB_24	chr19	49106935	G	A	FAM83E	MISSENSE	ENSG00000105523:[ENST00000263266(c.992C>T,cCg>cTg):ENSP00000263266(p.Pro331Leu)]	NA	NA	3.55E-02	8.04E-02	2.20E-01	3.55E-02	1.31E-01	T	BN	NA	MODERATE	N	0	31	31	0	32	10	22	68.75
Non-user	IOB_24	chr19	49129020	C	T	SPHK2	MISSENSE	ENSG00000063176:[ENST00000443164(c.98C>T,tCt>tTt):ENSP00000413369(p.Ser33Phe)]	NA	NA	7.79E-03	2.19E-02	3.64E-02	7.79E-03	3.89E-02	NA	NA	NA	MODIFIER	NA	NA	297	295	0	212	106	104	49.06
Non-user	IOB_24	chr19	49206548	C	T	FUT2	MISSENSE	ENSG00000176920:[ENST00000391876(c.335C>T,cCg>cTg):ENSP00000375748(p.Pro112Leu)]	NA	NA	3.73E-02	1.91E-02	1.35E-01	3.73E-02	1.83E-01	D	PoD	NA	MODERATE	NA	NA	95	95	0	92	42	50	54.35
Non-user	IOB_24	chr19	49206548	C	T	LOC105447645	EXONIC-NC	105447645:[NR_131188.1(n.558G>A)]	NA	NA	3.73E-02	1.91E-02	1.35E-01	3.73E-02	1.83E-01	D	PoD	NA	MODERATE	NA	NA	95	95	0	92	42	50	54.35
Non-user	IOB_24	chr19	49835004	C	A	CTC-301O7.4	EXONIC-NC	ENSG00000197813:[ENST00000602554(n.381G>T)]	NA	NA	9.78E-03	1.97E-02	2.11E-02	9.78E-03	2.35E-02	NA	NA	NA	MODIFIER	NA	NA	121	121	0	69	39	30	43.48
Non-user	IOB_24	chr19	49933899	G	A	SLC17A7	SILENT	ENSG00000104888:[ENST00000221485(c.1560C>T,gaC>gaT):ENSP00000221485(p.Asp520(=))]	NA	NA	1.32E-02	1.26E-02	6.09E-02	1.32E-02	6.44E-02	NA	NA	NA	LOW	NA	0	160	160	0	125	70	54	43.2
Non-user	IOB_24	chr19	50123590	C	T	PRR12	INTRONIC	ENSG00000126464:[ENST00000418929(c.5498-19C>T)]	NA	NA	3.34E-02	3.87E-02	1.19E-01	3.33E-02	1.23E-01	NA	NA	NA	MODIFIER	NA	NA	198	196	2	144	89	55	38.19

Patel *et al.* , 2021. Whole-exome sequencing analysis of oral squamous cell carcinoma delineated by tobacco usage habits
Supplementary Table 4. List of somatic single nucleotide variants identified using whole exome sequencing of OSCC patients

Cohort	Sample	Chromosome	Genomic position	Reference allele	Alternate allele	Genesymbol	Variant type	Variant description	OncoMD	COSMIC	dbSNP	ExAC	ExAC_SAS	1000G	1000G_SAS	SIFT	Polyphen2	CONDEL	VEP	LRT	PhastCon7Way	Total reads in Normal	Reference read support in Normal	Alternative read support in Normal	Total reads in Tumor	Reference read support in Tumor	Alternate read support in Tumor	Alternate allele frequency in Tumor
Non-user	IOB_24	chr19	50128905	C	T	PRR12	3UTR	ENSG00000126464:[ENST00000418929(c.*41C>T)]	NA	NA	3.34E-02	3.26E-02	1.11E-01	3.33E-02	1.23E-01	NA	NA	NA	MODIFIER	NA	NA	214	213	0	135	67	68	50.37
Non-user	IOB_24	chr19	50367493	C	T	PNKP	SILENT-SS-PRX	ENSG00000039650:[ENST00000322344(c.579G>A,agG>agA):ENSP00000323511(p.Arg193(=))]	NA	NA	3.20E-03	4.12E-03	1.18E-02	3.19E-03	1.33E-02	NA	NA	NA	LOW	NA	NA	281	278	0	221	123	97	43.89
Non-user	IOB_24	chr19	50906394	G	A	POLD1	MISSENSE	ENSG00000062822:[ENST00000440232(c.1055G>A,cGc>cAc):ENSP00000406046(p.Arg352His)]	NA	NA	3.99E-04	3.14E-04	6.22E-04	3.99E-04	2.00E-03	D	PrD	NA	MODERATE	NA	NA	123	123	0	105	65	38	36.19
Non-user	IOB_24	chr19	51132746	G	A	SYT3	SILENT	ENSG00000213023:[ENST00000338916(c.1086C>T,ccC>ccT):ENSP00000340914(p.Pro362(=))]	NA	NA	1.48E-02	2.75E-02	1.54E-02	1.48E-02	8.20E-03	NA	NA	NA	LOW	NA	NA	76	75	0	99	51	45	45.45
Non-user	IOB_24	chr19	51537763	G	A	KLK12	MISSENSE	ENSG00000186474:[ENST00000526824(c.115C>T,Ccc>Tcc):ENSP00000434604(p.Pro39Ser)]	NA	NA	5.99E-04	1.84E-03	6.42E-03	5.99E-04	3.10E-03	NA	NA	NA	MODIFIER	NA	NA	23	23	0	35	19	16	45.71
Non-user	IOB_24	chr19	52000042	G	A	SIGLEC12	INTRONIC	ENSG00000254521:[ENST00000598614(c.1245+92C>T)]	NA	NA	3.40E-03	NA	NA	3.39E-03	4.10E-03	NA	NA	NA	MODIFIER	NA	NA	85	85	0	71	38	32	45.07
Non-user	IOB_24	chr19	52000797	C	T	SIGLEC12	INTRONIC	ENSG00000254521:[ENST00000598614(c.1052-98G>A)]	NA	NA	3.12E-02	NA	NA	3.12E-02	7.36E-02	NA	NA	NA	MODIFIER	NA	NA	91	91	0	74	44	30	40.54
Non-user	IOB_24	chr19	52001282	G	A	SIGLEC12	SILENT	ENSG00000254521:[ENST00000598614(c.1041C>T,aaC>aaT):ENSP00000472873(p.Asn347(=))]	NA	NA	2.80E-02	3.38E-02	7.25E-02	2.80E-02	6.03E-02	NA	NA	NA	LOW	NA	NA	81	80	0	54	27	27	50
Non-user	IOB_24	chr19	52709171	C	T	PPP2R1A	INTRONIC	ENSG00000105568:[ENST00000444322(c.105+3884C>T)]	NA	NA	1.02E-02	1.32E-02	2.70E-02	1.02E-02	2.25E-02	NA	NA	NA	MODIFIER	NA	NA	146	143	0	112	63	47	41.96
Non-user	IOB_24	chr19	52716143	G	A	PPP2R1A	NCT	5518:[NR_033500.1(n.845+57G>A)]	NA	NA	2.00E-02	NA	NA	2.00E-02	1.33E-02	NA	NA	NA	MODIFIER	NA	NA	342	341	0	234	128	106	45.3
Non-user	IOB_24	chr19	53513790	G	A	CTD-2620I22.3	EXONIC-NC	ENSG00000267943:[ENST00000596769(n.1417G>A)]	NA	NA	2.32E-02	5.25E-02	5.28E-02	2.32E-02	4.19E-02	NA	NA	NA	MODIFIER	NA	NA	104	104	0	68	38	30	44.12
Non-user	IOB_24	chr19	53513790	G	A	ZNF702P	NCT	ENSG00000242779:[ENST00000600425(n.130-23691C>T)]	NA	NA	2.32E-02	5.25E-02	5.28E-02	2.32E-02	4.19E-02	NA	NA	NA	MODIFIER	NA	NA	104	104	0	68	38	30	44.12
Non-user	IOB_24	chr19	53579027	C	T	ZNF160	INTRONIC	ENSG00000170949:[ENST00000355147(c.16-591G>A)]	NA	NA	NA	8.87E-06	6.17E-05	NA	NA	NA	NA	NA	MODIFIER	NA	NA	123	123	0	62	26	36	58.06
Non-user	IOB_24	chr19	53802405	G	A	FAM90A28P	EXONIC-NC	ENSG00000269118:[ENST00000597191(n.1273C>T)]	NA	NA	NA	NA	NA	NA	NA	NA	NA	NA	MODIFIER	NA	NA	32	32	0	38	14	24	63.16
Non-user	IOB_24	chr19	53856808	T	G	ZNF845	SILENT	ENSG00000213799:[ENST00000458035(c.2880T>G,cgT>cgG):ENSP00000388311(p.Arg960(=))]	NA	NA	3.79E-03	4.20E-03	2.71E-02	3.79E-03	1.94E-02	NA	NA	NA	LOW	NA	NA	95	94	0	58	35	23	39.66
Non-user	IOB_24	chr19	54261549	C	G	MIR1283-2	EXONIC-NC	ENSG00000221548:[ENST00000408621(n.64C>G)]	NA	NA	9.98E-03	1.85E-02	2.93E-02	9.98E-03	2.15E-02	NA	NA	NA	MODIFIER	NA	NA	79	78	0	60	33	27	45
Non-user	IOB_24	chr19	54653517	A	G	CNOT3	NCT	4849:[XR_243936.1(n.1929+24A>G)]	NA	NA	2.00E-04	2.24E-04	1.26E-03	2.00E-04	1.00E-03	NA	NA	NA	MODIFIER	NA	NA	150	148	0	129	59	68	52.71
Non-user	IOB_24	chr19	54705205	T	A	RPS9	NCT	ENSG00000170889:[ENST00000495002(n.137+56T>A)]	NA	NA	1.60E-03	NA	NA	1.60E-03	8.20E-03	NA	NA	NA	MODIFIER	NA	NA	161	160	0	175	80	94	53.71
Non-user	IOB_24	chr19	54726299	A	C	LILRA6	INTRONIC	ENSG00000244482:[ENST00000391735(c.*642-3185T>G)]	NA	NA	NA	4.82E-01	4.44E-01	NA	NA	NA	NA	NA	MODIFIER	NA	NA	164	164	0	39	20	19	48.72
Non-user	IOB_24	chr19	54726299	A	C	LILRB3	MISSENSE	ENSG00000204577:[ENST00000245620(c.206T>G,tTg>tGg):ENSP00000245620(p.Leu69Trp)]	NA	NA	NA	4.82E-01	4.44E-01	NA	NA	NA	NA	NA	MODIFIER	NA	NA	164	164	0	39	20	19	48.72
Non-user	IOB_24	chr19	54726299	A	C	RPS9	INTRONIC	ENSG00000170889:[ENST00000448962(c.221-9318A>C)]	NA	NA	NA	4.82E-01	4.44E-01	NA	NA	NA	NA	NA	MODIFIER	NA	NA	164	164	0	39	20	19	48.72
Non-user	IOB_24	chr19	54726324	G	C	LILRA6	INTRONIC	ENSG00000244482:[ENST00000270464(c.659-1073C>G)]	NA	NA	NA	8.67E-01	8.16E-01	NA	NA	NA	NA	NA	MODIFIER	NA	NA	153	153	0	26	0	25	96.15
Non-user	IOB_24	chr19	54726324	G	C	LILRB3	MISSENSE	ENSG00000204577:[ENST00000245620(c.181C>G,Cat>Gat):ENSP00000245620(p.His61Asp)]	NA	NA	NA	8.67E-01	8.16E-01	NA	NA	NA	NA	NA	MODIFIER	NA	NA	153	153	0	26	0	25	96.15
Non-user	IOB_24	chr19	54726324	G	C	RPS9	INTRONIC	ENSG00000170889:[ENST00000448962(c.221-9293G>C)]	NA	NA	NA	8.67E-01	8.16E-01	NA	NA	NA	NA	NA	MODIFIER	NA	NA	153	153	0	26	0	25	96.15
Non-user	IOB_24	chr19	54726711	G	T	LILRA6	INTRONIC	ENSG00000244482:[ENST00000440558(c.956-2011C>A)]	NA	NA	NA	NA	NA	NA	NA	NA	NA	NA	MODIFIER	NA	NA	33	33	0	27	0	27	100
Non-user	IOB_24	chr19	54726711	G	T	LILRB3	NCT	11025:[NR_135493.1(n.1421-3597C>A)]	NA	NA	NA	NA	NA	NA	NA	NA	NA	NA	MODIFIER	NA	NA	33	33	0	27	0	27	100
Non-user	IOB_24	chr19	54726711	G	T	RPS9	INTRONIC	ENSG00000170889:[ENST00000448962(c.221-8906G>T)]	NA	NA	NA	NA	NA	NA	NA	NA	NA	NA	MODIFIER	NA	NA	33	33	0	27	0	27	100
Non-user	IOB_24	chr19	54726816	A	T	LILRA6	INTRONIC	ENSG00000244482:[ENST00000419410(c.1310-3702T>A)]	NA	chr19*54726816*54726816*COSM1285923*A*T*1*autonomic_ganglia*1	chr19*54726816*54726816*A*C,T*rs80332440*NA*NA*NA	1.96E-01	8.83E-02	NA	NA	NA	NA	NA	LOW	NA	NA	100	100	0	50	29	21	42
Non-user	IOB_24	chr19	54726816	A	T	LILRB3	SILENT-SS-PRX	11025:[NR_135494.1(n.1335-3702T>A)]	NA	chr19*54726816*54726816*COSM1285923*A*T*1*autonomic_ganglia*1	chr19*54726816*54726816*A*C,T*rs80332440*NA*NA*NA	1.96E-01	8.83E-02	NA	NA	NA	NA	NA	LOW	NA	NA	100	100	0	50	29	21	42
Non-user	IOB_24	chr19	54726816	A	T	RPS9	INTRONIC	ENSG00000170889:[ENST00000448962(c.221-8801A>T)]	NA	chr19*54726816*54726816*COSM1285923*A*T*1*autonomic_ganglia*1	chr19*54726816*54726816*A*C,T*rs80332440*NA*NA*NA	1.96E-01	8.83E-02	NA	NA	NA	NA	NA	LOW	NA	NA	100	100	0	50	29	21	42
Non-user	IOB_24	chr19	54726861	C	T	LILRA6	INTRONIC	ENSG00000244482:[ENST00000440558(c.956-2161G>A)]	NA	NA	NA	6.37E-02	1.65E-02	NA	NA	NA	NA	NA	MODIFIER	NA	NA	110	109	0	66	48	16	24.24
Non-user	IOB_24	chr19	54726861	C	T	LILRB3	NCT	11025:[NR_135494.1(n.1335-3747G>A)]	NA	NA	NA	6.37E-02	1.65E-02	NA	NA	NA	NA	NA	MODIFIER	NA	NA	110	109	0	66	48	16	24.24
Non-user	IOB_24	chr19	54726861	C	T	RPS9	INTRONIC	ENSG00000170889:[ENST00000448962(c.221-8756C>T)]	NA	NA	NA	6.37E-02	1.65E-02	NA	NA	NA	NA	NA	MODIFIER	NA	NA	110	109	0	66	48	16	24.24
Non-user	IOB_24	chr19	54726872	C	G	LILRA6	INTRONIC	ENSG00000244482:[ENST00000440558(c.956-2172G>C)]	NA	NA	chr19*54726872*54726872*C*A,G,T*rs140228691*NA*NA*NA	1.03E-01	3.33E-02	NA	NA	NA	NA	NA	MODIFIER	NA	NA	107	107	0	64	44	20	31.25
Non-user	IOB_24	chr19	54726872	C	G	LILRB3	NCT	11025:[NR_135494.1(n.1335-3758G>C)]	NA	NA	chr19*54726872*54726872*C*A,G,T*rs140228691*NA*NA*NA	1.03E-01	3.33E-02	NA	NA	NA	NA	NA	MODIFIER	NA	NA	107	107	0	64	44	20	31.25
Non-user	IOB_24	chr19	54726872	C	G	RPS9	INTRONIC	ENSG00000170889:[ENST00000448962(c.221-8745C>G)]	NA	NA	chr19*54726872*54726872*C*A,G,T*rs140228691*NA*NA*NA	1.03E-01	3.33E-02	NA	NA	NA	NA	NA	MODIFIER	NA	NA	107	107	0	64	44	20	31.25
Non-user	IOB_24	chr19	54743758	C	A	LILRA6	INTRONIC	ENSG00000244482:[ENST00000391735(c.*641+19G>T)]	NA	NA	NA	3.21E-01	2.60E-01	NA	NA	NA	NA	NA	MODIFIER	NA	NA	125	125	0	81	52	28	34.57
Non-user	IOB_24	chr19	54743758	C	A	LILRB3	INTRONIC	ENSG00000204577:[ENST00000407860(c.355+2144G>T)]	NA	NA	NA	3.21E-01	2.60E-01	NA	NA	NA	NA	NA	MODIFIER	NA	NA	125	125	0	81	52	28	34.57
Non-user	IOB_24	chr19	54743758	C	A	RPS9	INTRONIC	ENSG00000170889:[ENST00000448962(c.*183-7193C>A)]	NA	NA	NA	3.21E-01	2.60E-01	NA	NA	NA	NA	NA	MODIFIER	NA	NA	125	125	0	81	52	28	34.57
Non-user	IOB_24	chr19	54745550	G	T	LILRA6	MISSENSE	ENSG00000244482:[ENST00000245621(c.560C>A,aCc>aAc):ENSP00000245621(p.Thr187Asn)]	NA	NA	NA	9.23E-02	1.64E-01	NA	NA	NA	NA	NA	MODIFIER	NA	NA	85	85	0	33	28	5	15.15
Non-user	IOB_24	chr19	54745550	G	T	LILRB3	EXONIC-NC	11025:[NR_135496.1(n.671C>A)]	NA	NA	NA	9.23E-02	1.64E-01	NA	NA	NA	NA	NA	MODIFIER	NA	NA	85	85	0	33	28	5	15.15
Non-user	IOB_24	chr19	54745550	G	T	RPS9	INTRONIC	ENSG00000170889:[ENST00000448962(c.*183-5401G>T)]	NA	NA	NA	9.23E-02	1.64E-01	NA	NA	NA	NA	NA	MODIFIER	NA	NA	85	85	0	33	28	5	15.15
Non-user	IOB_24	chr19	54745978	T	C	LILRA6	SILENT	ENSG00000244482:[ENST00000245621(c.279A>G,gcA>gcG):ENSP00000245621(p.Ala93(=))]	NA	NA	chr19*54745978*54745978*T*A,C*rs1052964*NA*NA*NA	1.26E-01	1.12E-01	NA	NA	NA	NA	NA	MODIFIER	NA	NA	426	425	0	189	155	33	17.46
Non-user	IOB_24	chr19	54745978	T	C	LILRB3	SILENT	ENSG00000204577:[ENST00000407860(c.279A>G,gcA>gcG):ENSP00000384274(p.Ala93(=))]	NA	NA	chr19*54745978*54745978*T*A,C*rs1052964*NA*NA*NA	1.26E-01	1.12E-01	NA	NA	NA	NA	NA	MODIFIER	NA	NA	426	425	0	189	155	33	17.46
Non-user	IOB_24	chr19	54745978	T	C	RPS9	INTRONIC	ENSG00000170889:[ENST00000448962(c.*183-4973T>C)]	NA	NA	chr19*54745978*54745978*T*A,C*rs1052964*NA*NA*NA	1.26E-01	1.12E-01	NA	NA	NA	NA	NA	MODIFIER	NA	NA	426	425	0	189	155	33	17.46
Non-user	IOB_24	chr19	54746081	T	C	LILRA6	MISSENSE	ENSG00000244482:[ENST00000245621(c.176A>G,cAa>cGa):ENSP00000245621(p.Gln59Arg)]	NA	NA	NA	4.38E-01	3.97E-01	NA	NA	NA	NA	NA	MODIFIER	NA	NA	465	462	1	304	192	108	35.53
Non-user	IOB_24	chr19	54746081	T	C	LILRB3	MISSENSE	ENSG00000204577:[ENST00000407860(c.176A>G,cAa>cGa):ENSP00000384274(p.Gln59Arg)]	NA	NA	NA	4.38E-01	3.97E-01	NA	NA	NA	NA	NA	MODIFIER	NA	NA	465	462	1	304	192	108	35.53
Non-user	IOB_24	chr19	54746081	T	C	RPS9	INTRONIC	ENSG00000170889:[ENST00000448962(c.*183-4870T>C)]	NA	NA	NA	4.38E-01	3.97E-01	NA	NA	NA	NA	NA	MODIFIER	NA	NA	465	462	1	304	192	108	35.53
Non-user	IOB_24	chr19	54746528	C	T	LILRA6	EXONIC-NC	ENSG00000244482:[ENST00000474697(n.94G>A)]	NA	NA	NA	3.34E-01	3.06E-01	NA	NA	NA	NA	NA	MODIFIER	NA	NA	178	177	0	98	68	30	30.61
Non-user	IOB_24	chr19	54746528	C	T	LILRB3	INTRONIC	ENSG00000204577:[ENST00000407860(c.34+39G>A)]	NA	NA	NA	3.34E-01	3.06E-01	NA	NA	NA	NA	NA	MODIFIER	NA	NA	178	177	0	98	68	30	30.61
Non-user	IOB_24	chr19	54746528	C	T	RPS9	INTRONIC	ENSG00000170889:[ENST00000448962(c.*183-4423C>T)]	NA	NA	NA	3.34E-01	3.06E-01	NA	NA	NA	NA	NA	MODIFIER	NA	NA	178	177	0	98	68	30	30.61
Non-user	IOB_24	chr19	54930273	C	T	TTYH1	NCT	ENSG00000167614:[ENST00000478036(n.325-29C>T)]	NA	NA	7.99E-04	1.50E-03	8.37E-03	7.99E-04	2.00E-03	NA	NA	NA	MODIFIER	NA	NA	164	164	0	125	44	80	64
Non-user	IOB_24	chr19	54969039	G	A	LENG8	INTRONIC	114823:[XM_005258483.1(c.1779+12G>A)]	NA	NA	1.12E-02	2.50E-02	2.83E-02	1.12E-02	1.43E-02	NA	NA	NA	MODIFIER	NA	NA	197	196	0	165	79	86	52.12
Non-user	IOB_24	chr19	55044236	C	T	KIR3DX1	EXONIC-NC	ENSG00000104970:[ENST00000465702(n.328C>T)]	NA	NA	NA	3.75E-03	2.72E-02	NA	NA	NA	NA	NA	MODIFIER	NA	NA	91	82	3	87	71	14	16.09
Non-user	IOB_24	chr19	55044259	T	C	KIR3DX1	INTRONIC-SS-ACR-PRX	ENSG00000104970:[ENST00000221567(c.35-3T>C)]	NA	NA	7.59E-03	3.78E-03	2.71E-02	7.59E-03	3.78E-02	NA	NA	NA	LOW	NA	NA	87	82	1	78	52	22	28.21
Non-user	IOB_24	chr19	55045095	A	G	KIR3DX1	MISSENSE	ENSG00000104970:[ENST00000221567(c.215A>G,cAc>cGc):ENSP00000221567(p.His72Arg)]	NA	NA	7.39E-03	3.81E-03	2.74E-02	7.39E-03	3.78E-02	NA	NA	NA	MODIFIER	NA	NA	170	170	0	128	81	47	36.72
Non-user	IOB_24	chr19	55047037	A	G	KIR3DX1	SILENT	ENSG00000104970:[ENST00000221567(c.582A>G,agA>agG):ENSP00000221567(p.Arg194(=))]	NA	NA	7.39E-03	3.79E-03	2.73E-02	7.39E-03	3.78E-02	NA	NA	NA	MODIFIER	NA	NA	109	108	0	106	55	51	48.11
Non-user	IOB_24	chr19	55048205	C	A	KIR3DX1	MISSENSE	ENSG00000104970:[ENST00000221567(c.772C>A,Cac>Aac):ENSP00000221567(p.His258Asn)]	NA	NA	7.39E-03	3.89E-03	2.80E-02	7.39E-03	3.78E-02	NA	NA	NA	MODIFIER	NA	NA	164	164	0	104	53	50	48.08
Non-user	IOB_24	chr19	55048207	C	T	KIR3DX1	SILENT	ENSG00000104970:[ENST00000221567(c.774C>T,caC>caT):ENSP00000221567(p.His258(=))]	NA	chr19*55048207*55048209*COSM143487*CGG*CAA*1*skin*1	7.59E-03	4.00E-03	2.80E-02	7.59E-03	3.78E-02	NA	NA	NA	MODIFIER	NA	NA	166	166	0	107	54	51	47.66
Non-user	IOB_24	chr19	55048318	C	T	KIR3DX1	SILENT	ENSG00000104970:[ENST00000221567(c.885C>T,tgC>tgT):ENSP00000221567(p.Cys295(=))]	NA	NA	7.39E-03	3.93E-03	2.82E-02	7.39E-03	3.78E-02	NA	NA	NA	MODIFIER	NA	NA	163	163	0	123	66	54	43.9
Non-user	IOB_24	chr19	55048346	A	C	KIR3DX1	MISSENSE	ENSG00000104970:[ENST00000221567(c.913A>C,Aag>Cag):ENSP00000221567(p.Lys305Gln)]	NA	NA	.	NA	NA	NA	NA	NA	NA	NA	MODIFIER	NA	NA	130	130	0	103	56	5	4.85
Non-user	IOB_24	chr19	55147508	G	A	AC009892.10	MISSENSE	ENSG00000224730:[ENST00000456337(c.65C>T,gCg>gTg):ENSP00000401812(p.Ala22Val)]	NA	NA	NA	4.45E-02	2.80E-02	NA	NA	NA	NA	NA	MODIFIER	NA	NA	61	61	0	39	29	10	25.64
Non-user	IOB_24	chr19	55147508	G	A	LILRB1	INTRONIC	ENSG00000104972:[ENST00000396321(c.1651-440G>A)]	NA	NA	NA	4.45E-02	2.80E-02	NA	NA	NA	NA	NA	MODIFIER	NA	NA	61	61	0	39	29	10	25.64
Non-user	IOB_24	chr19	55488180	C	T	NLRP2	NCT	ENSG00000022556:[ENST00000540597(n.436-940C>T)]	NA	NA	NA	3.10E-01	2.89E-01	NA	NA	NA	NA	NA	MODIFIER	NA	NA	120	115	0	78	41	35	44.87
Non-user	IOB_24	chr19	55603066	C	A	PPP1R12C	MISSENSE-SS-PRX	ENSG00000125503:[ENST00000376393(c.2025G>T,gaG>gaT):ENSP00000365573(p.Glu675Asp)]	NA	NA	NA	NA	NA	NA	NA	D	BN	NA	MODERATE	NA	NA	108	105	1	74	35	38	51.35
Non-user	IOB_24	chr19	55660427	C	G	TNNT1	INTRONIC	ENSG00000105048:[ENST00000291901(c.-12+102G>C)]	NA	NA	5.59E-03	NA	NA	5.59E-03	1.12E-02	NA	NA	NA	MODIFIER	NA	NA	161	160	0	113	68	45	39.82
Non-user	IOB_24	chr19	55697236	G	A	PTPRH	SILENT	ENSG00000080031:[ENST00000376350(c.2895C>T,ctC>ctT):ENSP00000365528(p.Leu965(=))]	NA	NA	3.63E-02	6.16E-02	1.06E-01	3.63E-02	9.00E-02	NA	NA	NA	LOW	NA	NA	97	96	1	94	48	46	48.94
Non-user	IOB_24	chr19	55718153	C	A	PTPRH	MISSENSE	ENSG00000080031:[ENST00000263434(c.256G>T,Gtg>Ttg):ENSP00000263434(p.Val86Leu)]	NA	NA	chr19*55718153*55718153*C*T*rs373999553*NA*NA*NA	NA	NA	NA	NA	NA	NA	NA	MODIFIER	NA	NA	154	154	0	99	40	59	59.6
Non-user	IOB_24	chr19	55739833	G	A	AC010327.2	3UTR	ENSG00000269808:[ENST00000598855(c.*379G>A)]	NA	NA	9.98E-04	1.55E-03	3.25E-03	9.98E-04	1.00E-03	NA	NA	NA	MODIFIER	NA	NA	125	125	0	110	61	49	44.55
Non-user	IOB_24	chr19	55739833	G	A	TMEM86B	EXONIC-NC	ENSG00000180089:[ENST00000589190(n.309C>T)]	NA	NA	9.98E-04	1.55E-03	3.25E-03	9.98E-04	1.00E-03	NA	NA	NA	MODIFIER	NA	NA	125	125	0	110	61	49	44.55
Non-user	IOB_24	chr19	55751238	A	C	PPP6R1	MISSENSE	ENSG00000105063:[ENST00000412770(c.1519T>G,Tcg>Gcg):ENSP00000414202(p.Ser507Ala)]	NA	NA	3.16E-02	1.62E-02	1.95E-02	3.15E-02	1.23E-02	T	BN	NA	MODERATE	N	0	84	84	0	60	32	28	46.67
Non-user	IOB_24	chr19	55751549	C	T	PPP6R1	NCT	ENSG00000105063:[ENST00000591323(n.719+55G>A)]	NA	NA	2.80E-02	NA	NA	2.80E-02	1.23E-02	NA	NA	NA	MODIFIER	NA	NA	321	320	1	189	86	99	52.38
Non-user	IOB_24	chr19	55751556	T	C	PPP6R1	NCT	ENSG00000105063:[ENST00000591323(n.719+48A>G)]	NA	NA	3.36E-02	2.02E-02	2.07E-02	3.35E-02	1.43E-02	NA	NA	NA	MODIFIER	NA	NA	337	332	2	195	88	105	53.85
Non-user	IOB_24	chr19	55753536	C	A	PPP6R1	SILENT-SS-PRX	ENSG00000105063:[ENST00000412770(c.843G>T,ccG>ccT):ENSP00000414202(p.Pro281(=))]	NA	NA	2.66E-02	1.46E-02	1.95E-02	2.66E-02	1.23E-02	NA	NA	NA	LOW	NA	NA	109	108	0	69	40	29	42.03
Non-user	IOB_24	chr19	55758535	C	G	PPP6R1	5UTR	ENSG00000105063:[ENST00000587283(c.-64G>C)]	NA	NA	3.00E-02	1.59E-02	2.29E-02	3.00E-02	1.43E-02	NA	NA	NA	MODIFIER	NA	NA	177	176	1	150	96	54	36
Non-user	IOB_24	chr19	55790709	G	C	HSPBP1	INTRONIC	23640:[NM_001297600.1(c.348+58C>G)]	NA	NA	1.18E-02	NA	NA	1.18E-02	6.10E-03	NA	NA	NA	MODIFIER	NA	NA	76	74	1	80	42	38	47.5
Non-user	IOB_24	chr19	55798555	C	T	BRSK1	INTRONIC	84446:[XM_005259327.1(c.-43-27C>T)]	NA	NA	5.59E-03	1.01E-02	6.73E-03	5.59E-03	4.10E-03	NA	NA	NA	MODIFIER	NA	NA	255	253	1	191	93	96	50.26
Non-user	IOB_24	chr19	55871070	C	G	FAM71E2	MISSENSE	ENSG00000180043:[ENST00000424985(c.1166G>C,gGc>gCc):ENSP00000398617(p.Gly389Ala)]	NA	chr19*55871070*55871070*COSM222393*C*T*1*skin*1	7.99E-04	3.14E-03	7.61E-03	7.99E-04	4.10E-03	T	BN	NA	MODERATE	NA	NA	165	165	0	140	76	64	45.71
Non-user	IOB_24	chr19	56135983	T	C	NA	INTERGENIC	NA	NA	NA	4.21E-02	NA	NA	4.21E-02	6.75E-02	NA	NA	NA	MODIFIER	NA	NA	79	77	1	43	26	17	39.53
Non-user	IOB_24	chr19	56284090	G	A	RFPL4AL1	MISSENSE	ENSG00000229292:[ENST00000341750(c.409G>A,Gcc>Acc):ENSP00000345151(p.Ala137Thr)]	NA	chr19*56284090*56284090*COSM148755*G*A*1*stomach*1	NA	4.13E-01	3.40E-01	NA	NA	T	BN	NA	MODERATE	NA	NA	141	140	1	98	62	34	34.69
Non-user	IOB_24	chr19	56370309	C	T	NLRP4	MISSENSE	ENSG00000160505:[ENST00000589437(c.247C>T,gCg>gTg):ENSP00000468754(p.Ala83Val)]	NA	chr19*56370309*56370309*COSM440391*C*T*1*breast*1	chr19*56370309*56370309*C*G,T*rs200576295*NA*NA*NA	8.24E-06	0.00E+00	NA	NA	T	BN	NA	MODERATE	NA	NA	235	233	0	140	79	61	43.57

Patel *et al.*, 2021. Whole-exome sequencing analysis of oral squamous cell carcinoma delineated by tobacco usage habits
Supplementary Table 4. List of somatic single nucleotide variants identified using whole exome sequencing of OSCC patients

Cohort	Sample	Chromosome	Genomic position	Reference allele	Alternate allele	Genesymbol	Variant type	Variant description	OncoMD	COSMIC	dbSNP	ExAC	ExAC_SAS	1000G	1000G_SAS	SIFT	Polyphen2	CONDEL	VEP	LRT	PhastCon7Way	Total reads in Normal	Reference read support in Normal	Alternative read support in Normal	Total reads in Tumor	Reference read support in Tumor	Alternate read support in Tumor	Alternate allele frequency in Tumor
Non-user	IOB_24	chr19	56538832	C	T	NLRP5	SILENT	ENSG00000171487:[ENST00000390649(c.1233C>T,gaC>gaT):ENSP00000375063(p.Asp411(=))]	NA	chr19*56538832*56538832*COSM1631109*C*T*1*liver*1	4.19E-02	4.80E-02	1.13E-02	4.19E-02	5.10E-03	NA	NA	NA	LOW	NA	0	207	206	0	129	64	65	50.39
Non-user	IOB_24	chr19	56539013	G	A	NLRP5	MISSENSE	ENSG00000171487:[ENST00000390649(c.1414G>A,Gcc>Acc):ENSP00000375063(p.Ala472Thr)]	NA	chr19*56539013*56539013*COSM255299*G*A*1*central_nervous_system*1	6.59E-03	5.28E-03	2.13E-02	6.59E-03	1.84E-02	T	BN	NA	MODERATE	N	0	286	286	0	224	120	103	45.98
Non-user	IOB_24	chr19	56543982	G	T	NLRP5	MISSENSE	ENSG00000171487:[ENST00000390649(c.2282G>T,cGg>cTg):ENSP00000375063(p.Arg761Leu)]	NA	NA	1.34E-02	2.81E-02	7.61E-03	1.34E-02	4.10E-03	T	BN	NA	MODERATE	N	0	53	52	0	43	25	17	39.53
Non-user	IOB_24	chr19	57157140	C	T	SMIM17	SILENT	ENSG00000268182:[ENST00000598409(c.105C>T,ccC>ccT):ENSP00000471126(p.Pro35(=))]	NA	NA	2.68E-02	8.91E-03	4.24E-03	2.68E-02	9.20E-03	NA	NA	NA	MODIFIER	NA	NA	85	85	0	76	39	36	47.37
Non-user	IOB_24	chr19	57328300	G	A	PEG3	MISSENSE	ENSG00000198300:[ENST00000326441(c.1510C>T,Cgt>Tgt):ENSP00000326581(p.Arg504Cys)]	NA	NA	3.99E-04	2.22E-04	1.57E-03	3.99E-04	1.00E-03	NA	NA	NA	MODIFIER	NA	NA	485	484	0	340	153	185	54.41
Non-user	IOB_24	chr19	57328300	G	A	ZIM2	INTRONIC	ENSG00000269699:[ENST00000221722(c.397+1668C>T)]	NA	NA	3.99E-04	2.22E-04	1.57E-03	3.99E-04	1.00E-03	NA	NA	NA	MODIFIER	NA	NA	485	484	0	340	153	185	54.41
Non-user	IOB_24	chr19	58118418	C	T	ZNF530	MISSENSE	ENSG00000183647:[ENST00000332854(c.1525C>T,Cac>Tac):ENSP00000332861(p.His509Tyr)]	NA	NA	9.19E-03	2.75E-02	1.42E-02	9.19E-03	1.12E-02	D	PrD	NA	MODERATE	NA	NA	293	289	0	237	126	103	43.46
Non-user	IOB_24	chr19	58491223	T	C	ZNF606	SILENT	ENSG00000166704:[ENST00000536132(c.555A>G,caA>caG):ENSP00000445624(p.Gln185(=))]	NA	NA	3.59E-03	2.33E-03	1.69E-02	3.59E-03	1.84E-02	NA	NA	NA	MODIFIER	NA	NA	135	134	1	106	61	45	42.45
Non-user	IOB_24	chr19	58512098	G	A	ZNF606	MISSENSE	ENSG00000166704:[ENST00000341164(c.41C>T,aCg>aTg):ENSP00000343617(p.Thr14Met)]	NA	NA	3.79E-03	2.54E-03	1.89E-02	3.79E-03	1.84E-02	NA	NA	NA	MODIFIER	NA	NA	194	192	0	139	69	69	49.64
Non-user	IOB_24	chr19	58907700	G	A	AC012313.1	MISSENSE	ENSG00000269855:[ENST00000601382(c.244G>A,Gac>Aac):ENSP00000470441(p.Asp82Asn)]	NA	NA	2.22E-02	4.67E-02	4.34E-02	2.22E-02	4.09E-02	T	PrD	NA	MODERATE	NA	NA	211	211	0	165	91	73	44.24
Non-user	IOB_24	chr19	58946091	G	A	ZNF132	SILENT	ENSG00000131849:[ENST00000254166(c.720C>T,aaC>aaT):ENSP00000254166(p.Asn240(=))]	NA	NA	3.99E-04	3.71E-04	2.73E-03	3.99E-04	2.00E-03	NA	NA	NA	MODIFIER	NA	NA	148	147	1	149	83	66	44.3
Non-user	IOB_24	chr19	59059177	C	T	TRIM28	SILENT	ENSG00000130726:[ENST00000253024(c.858C>T,gaC>gaT):ENSP00000253024(p.Asp286(=))]	NA	NA	3.59E-03	2.52E-03	1.79E-02	3.59E-03	1.84E-02	NA	NA	NA	MODIFIER	NA	NA	291	289	0	213	97	115	53.99
Non-user	IOB_24	chr2	1251088	C	T	SNTG2	INTRONIC	ENSG00000172554:[ENST00000308624(c.889-11C>T)]	NA	NA	chr2*1251087*1251088*AC*AAAGGTTT*rs375675419*NA*NA*NA|chr2*1251088*1251088*C*A,T*rs55953201*NA*NA*NA	3.01E-01	4.00E-01	NA	NA	NA	NA	NA	MODIFIER	NA	NA	77	76	0	50	32	17	34
Non-user	IOB_24	chr2	1748117	C	G	PXDN	SILENT	ENSG00000130508:[ENST00000433670(c.98G>C,ccG>ccC):ENSP00000402738(p.Pro33(=))]	NA	NA	7.99E-03	2.34E-02	2.93E-02	7.99E-03	2.04E-02	NA	NA	NA	LOW	NA	NA	90	90	0	84	47	37	44.05
Non-user	IOB_24	chr2	3200870	G	T	TSSC1	NCT	ENSG00000032389:[ENST00000470625(n.305-82C>A)]	NA	NA	chr2*3200870*3200870*G*A*rs935052959*NA*NA*NA	NA	NA	NA	NA	NA	NA	NA	MODIFIER	NA	NA	50	50	0	43	21	22	51.16
Non-user	IOB_24	chr2	3381422	G	A	TSSC1	SILENT	ENSG00000032389:[ENST00000382125(c.39C>T,ttC>ttT):ENSP00000371559(p.Phe13(=))]	NA	NA	NA	1.76E-04	5.97E-04	NA	NA	NA	NA	NA	LOW	NA	NA	134	133	0	105	53	52	49.52
Non-user	IOB_24	chr2	8998753	T	C	MBOAT2	EXONIC-NC	129642:[NR_135601.1(n.1450A>G)]	NA	NA	NA	NA	NA	NA	NA	NA	NA	NA	MODIFIER	NA	NA	111	110	0	77	35	42	54.55
Non-user	IOB_24	chr2	10044999	C	G	TAF1B	MISSENSE	ENSG00000115750:[ENST00000492648(c.133C>G,gaC>gaG):ENSP00000475608(p.Asp45Glu)]	NA	NA	7.99E-03	1.41E-02	6.31E-03	7.99E-03	6.10E-03	T	BN	NA	MODERATE	N	0.002	217	215	0	161	83	77	47.83
Non-user	IOB_24	chr2	11696911	G	A	GREB1	INTRONIC	ENSG00000196208:[ENST00000234142(c.157+14G>A)]	NA	NA	3.00E-03	3.34E-03	1.88E-02	3.00E-03	1.43E-02	NA	NA	NA	MODIFIER	NA	NA	49	49	0	20	3	17	85
Non-user	IOB_24	chr2	17955694	G	A	GEN1	EXONIC-NC	ENSG00000178295:[ENST00000534451(n.412G>A)]	NA	NA	2.88E-02	3.14E-02	2.05E-02	2.88E-02	2.35E-02	NA	NA	NA	MODIFIER	NA	NA	28	28	0	29	13	16	55.17
Non-user	IOB_24	chr2	17955694	G	A	SMC6	INTRONIC	ENSG00000163029:[ENST00000402989(c.-6+13388C>T)]	NA	NA	2.88E-02	3.14E-02	2.05E-02	2.88E-02	2.35E-02	NA	NA	NA	MODIFIER	NA	NA	28	28	0	29	13	16	55.17
Non-user	IOB_24	chr2	20205541	C	T	MATN3	MISSENSE	ENSG00000132031:[ENST00000407540(c.754G>A,Gag>Aag):ENSP00000383894(p.Glu252Lys)]	NA	NA	1.44E-02	3.09E-02	5.66E-02	1.44E-02	3.48E-02	D	PrD	NA	MODERATE	D	0.996	149	149	0	138	83	54	39.13
Non-user	IOB_24	chr2	20647412	C	T	RHOB	SILENT	ENSG00000143878:[ENST00000272233(c.186C>T,ggC>ggT):ENSP00000272233(p.Gly62(=))]	NA	NA	5.99E-04	7.44E-05	6.06E-05	5.99E-04	0.00E+00	NA	NA	NA	LOW	NA	NA	276	272	0	212	111	98	46.23
Non-user	IOB_24	chr2	20840823	G	A	HS1BP3	MISSENSE	ENSG00000118960:[ENST00000304031(c.316C>T,Cgg>Tgg):ENSP00000305193(p.Arg106Trp)]	NA	NA	3.40E-03	6.18E-03	1.25E-02	3.39E-03	7.20E-03	NA	NA	NA	MODIFIER	NA	NA	239	238	0	229	114	115	50.22
Non-user	IOB_24	chr2	25141251	C	T	ADCY3	SILENT	ENSG00000138031:[ENST00000260600(c.606G>A,acG>acA):ENSP00000260600(p.Thr202(=))]	NA	NA	3.79E-03	2.19E-03	1.56E-02	3.79E-03	1.84E-02	NA	NA	NA	LOW	NA	NA	59	58	1	46	29	17	36.96
Non-user	IOB_24	chr2	25973122	C	T	ASXL2	MISSENSE	ENSG00000143970:[ENST00000272341(c.523G>A,Gca>Aca):ENSP00000272341(p.Ala175Thr)]	NA	NA	NA	3.31E-05	2.42E-04	NA	NA	T	BN	NA	MODERATE	N	0.007	277	276	1	205	113	91	44.39
Non-user	IOB_24	chr2	26534244	C	T	ADGRF3	SILENT	165082:[NM_001321971.1(c.2148G>A,gcG>gcA):NP_001308900.1(p.Ala716(=))]	NA	NA	2.46E-02	3.90E-02	4.62E-02	2.46E-02	4.81E-02	NA	NA	NA	MODIFIER	NA	NA	252	251	0	184	110	74	40.22
Non-user	IOB_24	chr2	26534244	C	T	EPT1	INTRONIC	ENSG00000138018:[ENST00000442141(c.-40+2719C>T)]	NA	NA	2.46E-02	3.90E-02	4.62E-02	2.46E-02	4.81E-02	NA	NA	NA	MODIFIER	NA	NA	252	251	0	184	110	74	40.22
Non-user	IOB_24	chr2	26534244	C	T	GPR113	SILENT	ENSG00000173567:[ENST00000333478(c.1755G>A,gcG>gcA):ENSP00000327396(p.Ala585(=))]	NA	NA	2.46E-02	3.90E-02	4.62E-02	2.46E-02	4.81E-02	NA	NA	NA	MODIFIER	NA	NA	252	251	0	184	110	74	40.22
Non-user	IOB_24	chr2	27258902	C	G	TMEM214	SILENT	ENSG00000119777:[ENST00000404032(c.567C>G,gcC>gcG):ENSP00000384417(p.Ala189(=))]	NA	NA	1.40E-03	1.36E-03	8.30E-03	1.40E-03	6.10E-03	NA	NA	NA	LOW	NA	NA	99	99	0	80	32	48	60
Non-user	IOB_24	chr2	27293041	C	T	AGBL5	SILENT	ENSG00000084693:[ENST00000360131(c.2571C>T,tcC>tcT):ENSP00000353249(p.Ser857(=))]	NA	NA	1.40E-02	1.89E-02	5.17E-02	1.40E-02	4.29E-02	NA	NA	NA	MODIFIER	NA	NA	128	127	0	114	57	55	48.25
Non-user	IOB_24	chr2	27327369	T	C	CGREF1	MISSENSE	ENSG00000138028:[ENST00000404694(c.232A>G,Agt>Ggt):ENSP00000385574(p.Ser78Gly)]	NA	NA	2.50E-02	4.29E-02	6.28E-02	2.50E-02	5.62E-02	NA	NA	NA	MODIFIER	NA	NA	130	130	0	120	58	61	50.83
Non-user	IOB_24	chr2	27480349	T	C	SLC30A3	INTRONIC	7781:[XM_005264547.1(c.425-129A>G)]	NA	NA	4.39E-03	8.75E-03	1.67E-02	4.39E-03	2.15E-02	NA	NA	NA	MODIFIER	NA	NA	112	112	0	95	55	40	42.11
Non-user	IOB_24	chr2	27803279	C	T	C2orf16	SILENT	ENSG00000221843:[ENST00000408964(c.3840C>T,acC>acT):ENSP00000386190(p.Thr1280(=))]	NA	NA	1.14E-02	2.14E-02	4.05E-02	1.14E-02	2.86E-02	NA	NA	NA	LOW	NA	NA	103	103	0	98	53	43	43.88
Non-user	IOB_24	chr2	28752348	G	A	PLB1	INTRONIC	ENSG00000163803:[ENST00000404858(c.412+74G>A)]	NA	NA	1.98E-02	NA	NA	1.98E-02	1.74E-02	NA	NA	NA	MODIFIER	NA	NA	42	42	0	51	29	21	41.18
Non-user	IOB_24	chr2	29294253	C	T	C2orf71	MISSENSE	ENSG00000179270:[ENST00000331664(c.2875G>A,Gcc>Acc):ENSP00000332809(p.Ala959Thr)]	NA	NA	2.00E-03	3.68E-03	1.14E-02	2.00E-03	9.20E-03	T	BN	NA	MODERATE	NA	NA	126	124	0	131	77	53	40.46
Non-user	IOB_24	chr2	31189144	C	T	GALNT14	SILENT	ENSG00000158089:[ENST00000349752(c.324G>A,acG>acA):ENSP00000288988(p.Thr108(=))]	NA	NA	3.51E-02	2.38E-02	1.82E-02	3.51E-02	3.07E-02	NA	NA	NA	MODIFIER	NA	NA	134	131	0	119	62	57	47.9
Non-user	IOB_24	chr2	32800247	C	T	BIRC6	MISSENSE	ENSG00000115760:[ENST00000421745(c.13169C>T,cCa>cTa):ENSP00000393596(p.Pro4390Leu)]	NA	NA	NA	NA	NA	NA	NA	NA	NA	NA	MODERATE	NA	1	167	166	1	162	86	74	45.68
Non-user	IOB_24	chr2	33780571	C	T	AC020594.5	NCT	ENSG00000237133:[ENST00000437680(n.71-190G>A)]	NA	NA	NA	3.00E-05	1.87E-04	NA	NA	NA	NA	NA	MODIFIER	NA	NA	228	227	0	163	91	72	44.17
Non-user	IOB_24	chr2	33780571	C	T	RASGRP3	INTRONIC	ENSG00000152689:[ENST00000402538(c.1543-27C>T)]	NA	NA	NA	3.00E-05	1.87E-04	NA	NA	NA	NA	NA	MODIFIER	NA	NA	228	227	0	163	91	72	44.17
Non-user	IOB_24	chr2	33952770	G	T	AC009499.1	NCT	ENSG00000203386:[ENST00000366209(n.68+20750G>T)]	NA	NA	4.01E-02	2.21E-02	7.95E-03	4.01E-02	1.02E-02	NA	NA	NA	MODIFIER	NA	NA	329	329	0	276	143	131	47.46
Non-user	IOB_24	chr2	33952770	G	T	MYADML	EXONIC-NC	ENSG00000239649:[ENST00000491596(n.286C>A)]	NA	NA	4.01E-02	2.21E-02	7.95E-03	4.01E-02	1.02E-02	NA	NA	NA	MODIFIER	NA	NA	329	329	0	276	143	131	47.46
Non-user	IOB_24	chr2	36623698	G	T	CRIM1	INTRONIC	ENSG00000150938:[ENST00000426856(c.181+39932G>T)]	NA	NA	chr2*36623698*36623698*G*A*rs1054897691*NA*NA*NA	NA	NA	NA	NA	NA	NA	NA	MODIFIER	NA	NA	174	173	0	133	65	68	51.13
Non-user	IOB_24	chr2	37287683	T	A	HEATR5B	INTRONIC	ENSG00000008869:[ENST00000233099(c.1849+41A>T)]	NA	NA	4.11E-02	7.09E-02	8.74E-02	4.11E-02	7.67E-02	NA	NA	NA	MODIFIER	NA	NA	84	84	0	76	39	37	48.68
Non-user	IOB_24	chr2	37336403	G	C	EIF2AK2	SILENT	ENSG00000055332:[ENST00000233057(c.1413C>G,ctC>ctG):ENSP00000233057(p.Leu471(=))]	NA	NA	2.20E-02	2.91E-02	3.99E-02	2.20E-02	2.66E-02	NA	NA	NA	LOW	NA	NA	172	172	0	133	61	72	54.14
Non-user	IOB_24	chr2	39184129	C	T	ARHGEF33	SILENT	ENSG00000214694:[ENST00000398800(c.1311C>T,taC>taT):ENSP00000381780(p.Tyr437(=))]	NA	NA	NA	3.97E-04	1.01E-03	NA	NA	NA	NA	NA	LOW	NA	0.998	287	287	0	240	119	119	49.58
Non-user	IOB_24	chr2	42530331	C	A	EML4	SILENT	ENSG00000143924:[ENST00000402711(c.1555C>A,Cga>Aga):ENSP00000385059(p.Arg519(=))]	NA	NA	9.39E-03	1.80E-02	1.57E-02	9.38E-03	1.64E-02	NA	NA	NA	LOW	NA	1	112	111	0	90	52	37	41.11
Non-user	IOB_24	chr2	44457222	T	C	PPM1B	NCT	ENSG00000138032:[ENST00000487286(n.379+11546T>C)]	NA	NA	2.80E-03	NA	NA	2.80E-03	4.10E-03	NA	NA	NA	MODIFIER	NA	NA	81	81	0	50	15	35	70
Non-user	IOB_24	chr2	45704229	T	A	SRBD1	INTRONIC	ENSG00000068784:[ENST00000263736(c.1967-15A>T)]	NA	NA	1.40E-03	2.44E-03	3.01E-03	1.40E-03	4.10E-03	NA	NA	NA	MODIFIER	NA	NA	47	47	0	48	30	18	37.5
Non-user	IOB_24	chr2	46237530	G	A	PRKCE	SILENT	ENSG00000171132:[ENST00000306156(c.1311G>A,aaG>aaA):ENSP00000306124(p.Lys437(=))]	NA	NA	2.20E-03	4.19E-03	1.83E-04	2.20E-03	0.00E+00	NA	NA	NA	LOW	NA	0.986	172	172	0	140	70	68	48.57
Non-user	IOB_24	chr2	47136381	G	C	MCFD2	NCT	ENSG00000180398:[ENST00000477791(n.70-65C>G)]	NA	NA	1.14E-02	NA	NA	1.14E-02	2.25E-02	NA	NA	NA	MODIFIER	NA	NA	55	54	0	54	26	28	51.85
Non-user	IOB_24	chr2	48046081	C	T	FBXO11	INTRONIC	ENSG00000138081:[ENST00000403359(c.1920+14G>A)]	NA	NA	NA	8.25E-06	6.08E-05	NA	NA	NA	NA	NA	MODIFIER	NA	NA	114	114	0	143	76	64	44.76
Non-user	IOB_24	chr2	48602186	A	G	FOXN2	SILENT	ENSG00000170802:[ENST00000340553(c.900A>G,gaA>gaG):ENSP00000343633(p.Glu300(=))]	NA	NA	3.00E-02	6.53E-02	5.63E-02	3.00E-02	5.11E-02	NA	NA	NA	LOW	NA	1	273	273	0	204	115	89	43.63
Non-user	IOB_24	chr2	48681918	G	A	PPP1R21	NCT	129285:[NR_024188.2(n.497+38G>A)]	NA	NA	4.99E-03	6.10E-03	2.59E-03	4.99E-03	2.00E-03	NA	NA	NA	MODIFIER	NA	NA	144	142	0	83	42	41	49.4
Non-user	IOB_24	chr2	48873925	A	G	GTF2A1L	MISSENSE	ENSG00000242441:[ENST00000437125(c.749A>G,tAt>tGt):ENSP00000396702(p.Tyr250Cys)]	NA	NA	1.40E-02	4.16E-02	1.34E-02	1.40E-02	3.10E-03	T	BN	NA	MODERATE	NA	0.001	264	264	0	200	96	103	51.5
Non-user	IOB_24	chr2	48873925	A	G	LHCGR	NCT	ENSG00000138039:[ENST00000420913(n.188-172T>C)]	NA	NA	1.40E-02	4.16E-02	1.34E-02	1.40E-02	3.10E-03	T	BN	NA	MODERATE	NA	0.001	264	264	0	200	96	103	51.5
Non-user	IOB_24	chr2	48873925	A	G	STON1-GTF2A1L	MISSENSE	ENSG00000068781:[ENST00000309827(c.2834A>G,tAt>tGt):ENSP00000311493(p.Tyr945Cys)]	NA	NA	1.40E-02	4.16E-02	1.34E-02	1.40E-02	3.10E-03	T	BN	NA	MODERATE	NA	0.001	264	264	0	200	96	103	51.5
Non-user	IOB_24	chr2	48921438	T	C	GTF2A1L	INTRONIC	11036:[XM_005264116.1(c.1329+22619T>C)]	NA	NA	1.20E-02	5.19E-02	1.66E-02	1.20E-02	3.10E-03	T	PrD	NA	MODERATE	D	1	66	66	0	68	37	30	44.12
Non-user	IOB_24	chr2	48921438	T	C	LHCGR	MISSENSE	ENSG00000138039:[ENST00000294954(c.872A>G,aAt>aGt):ENSP00000294954(p.Asn291Ser)]	NA	NA	1.20E-02	5.19E-02	1.66E-02	1.20E-02	3.10E-03	T	PrD	NA	MODERATE	D	1	66	66	0	68	37	30	44.12
Non-user	IOB_24	chr2	48921438	T	C	STON1-GTF2A1L	INTRONIC	ENSG00000068781:[ENST00000402114(c.3441+22619T>C)]	NA	NA	1.20E-02	5.19E-02	1.66E-02	1.20E-02	3.10E-03	T	PrD	NA	MODERATE	D	1	66	66	0	68	37	30	44.12
Non-user	IOB_24	chr2	54124103	T	C	PSME4	INTRONIC	ENSG00000068878:[ENST00000421748(c.1039-81A>G)]	NA	NA	4.19E-03	NA	NA	4.19E-03	1.23E-02	NA	NA	NA	MODIFIER	NA	NA	55	55	0	37	21	16	43.24
Non-user	IOB_24	chr2	54161689	G	C	PSME4	INTRONIC	ENSG00000068878:[ENST00000389993(c.957+53C>G)]	NA	NA	NA	NA	NA	NA	NA	NA	NA	NA	MODIFIER	NA	NA	102	102	0	62	31	31	50
Non-user	IOB_24	chr2	55121996	G	C	EML6	INTRONIC	ENSG00000214595:[ENST00000356458(c.2743-56G>C)]	NA	NA	4.21E-02	NA	NA	4.21E-02	2.86E-02	NA	NA	NA	MODIFIER	NA	NA	117	116	0	104	55	49	47.12
Non-user	IOB_24	chr2	60688204	C	A	BCL11A	MISSENSE	ENSG00000119866:[ENST00000537768(c.850G>T,Ggg>Tgg):ENSP00000443712(p.Gly284Trp)]	NA	NA	NA	NA	NA	NA	NA	NA	NA	NA	MODIFIER	NA	NA	181	181	0	159	154	5	3.14
Non-user	IOB_24	chr2	60769160	T	C	BCL11A	INTRONIC	ENSG00000119866:[ENST00000537768(c.-291+3946A>G)]	NA	NA	3.79E-03	NA	NA	3.79E-03	2.00E-03	NA	NA	NA	MODIFIER	NA	NA	25	25	0	14	6	8	57.14
Non-user	IOB_24	chr2	63720089	A	T	WDPCP	INTRONIC	ENSG00000143951:[ENST00000418148(c.71-15T>A)]	NA	NA	8.19E-03	4.15E-03	2.85E-02	8.19E-03	4.19E-02	NA	NA	NA	MODIFIER	NA	NA	85	85	0	75	37	38	50.67
Non-user	IOB_24	chr2	68444171	C	G	PPP3R1	INTRONIC	ENSG00000221823:[ENST00000409752(c.100+53G>C)]	NA	NA	NA	NA	NA	NA	NA	NA	NA	NA	MODIFIER	NA	NA	46	44	2	33	18	14	42.42
Non-user	IOB_24	chr2	68444171	C	G	RP11-474G23.1	INTRONIC	ENSG00000273398:[ENST00000406334(c.13+53G>C)]	NA	NA	NA	NA	NA	NA	NA	NA	NA	NA	MODIFIER	NA	NA	46	44	2	33	18	14	42.42
Non-user	IOB_24	chr2	69043288	C	T	ARHGAP25	SILENT-SS-PRX	ENSG00000163219:[ENST00000473986(c.*631-133C>T)]	NA	NA	NA	3.18E-05	1.08E-04	NA	NA	NA	NA	NA	MODIFIER	NA	NA	200	196	0	141	78	59	41.84
Non-user	IOB_24	chr2	69302829	G	A	ANTXR1	NCT	ENSG00000169604:[ENST00000463335(n.704+39G>A)]	NA	NA	9.98E-04	1.67E-03	1.51E-03	9.98E-04	0.00E+00	NA	NA	NA	MODIFIER	NA	NA	239	239	0	172	94	78	45.35
Non-user	IOB_24	chr2	73455730	C	T	PRADC1	NCT	84279:[NR_144358.1(n.549-28G>A)]	NA	NA	3.00E-03	2.34E-03	1.56E-02	3.00E-03	1.33E-02	NA	NA	NA	MODIFIER	NA	NA	257	256	1	159	83	76	47.8
Non-user	IOB_24	chr2	73477491	C	T	CCT7	SILENT	ENSG00000135624:[ENST00000258091(c.1128C>T,ggC>ggT):ENSP00000258091(p.Gly376(=))]	NA	NA	2.00E-04	4.97E-05	3.03E-04	2.00E-04	0.00E+00	NA	NA	NA	LOW	NA	NA	83	83	0	68	32	36	52.94
Non-user	IOB_24	chr2	74076551	C	T	STAMBP	MISSENSE	ENSG00000124356:[ENST00000536064(c.623C>T,tCc>tTc):ENSP00000443502(p.Ser208Phe)]	NA	NA	2.80E-03	1.33E-03	9.39E-03	2.80E-03	1.33E-02	NA	NA	NA	LOW	NA	NA	62	62	0	40	23	16	40
Non-user	IOB_24	chr2	84896590	G	A	DNAH6	MISSENSE	ENSG00000115423:[ENST00000237449(c.6262G>A,Gtc>Atc):ENSP00000237449(p.Val2088Ile)]	NA	NA	NA	NA	NA	NA	NA	T	BN	NA	MODERATE	NA	NA	268	267	1	236	115	121	51.27
Non-user	IOB_24	chr2	86272309	G	A	POLR1A	INTRONIC	ENSG00000068654:[ENST00000263857(c.2969+92C>T)]	NA	NA	2.92E-02	NA	NA	2.92E-02	3.99E-02	NA	NA	NA	MODIFIER	NA	NA	70	70	0	78	44	34	43.59
Non-user	IOB_24	chr2	86302409	A	G	POLR1A	INTRONIC	ENSG00000068654:[ENST00000263857(c.1381-26T>C)]	NA	NA	2.92E-02	2.69E-02	3.22E-02	2.92E-02	3.99E-02	NA	NA	NA	MODIFIER	NA	NA	64	64	0	54	31	22	40.74
Non-user	IOB_24	chr2	86400791	T	C	IMMT	MISSENSE	ENSG00000132305:[ENST00000419070(c.95A>G,Act>Gct):ENSP00000415762(p.Thr33Ala)]	NA	NA	2.88E-02	1.63E-02	3.36E-02	2.88E-02	3.99E-02	T_lc	BN	NA	MODERATE	N	0.072	39	39	0	28	17	11	39.29
Non-user	IOB_24	chr2	86420201	G	C	IMMT	NCT	ENSG00000132305:[ENST00000486633(n.113+2260C>G)]	NA	NA	2.02E-02	2.00E+00	NA	2.02E-02	8.20E-03	NA	NA	NA	MODIFIER	NA	NA	105	105	0	57	29	28	49.12
Non-user	IOB_24	chr2	86420201	G	C	MIR4779	EXONIC-NC	ENSG00000265420:[ENST00000579234(n.31C>G)]	NA	NA	2.02E-02	2.00E+00	NA	2.02E-02	8.20E-03	NA	NA	NA	MODIFIER	NA	NA	105	105	0	57	29	28	49.12

Patel *et al.*, 2021. Whole-exome sequencing analysis of oral squamous cell carcinoma delineated by tobacco usage habits
Supplementary Table 4. List of somatic single nucleotide variants identified using whole exome sequencing of OSCC patients

Cohort	Sample	Chromosome	Genomic position	Reference allele	Alternate allele	Genesymbol	Variant type	Variant description	OncoMD	COSMIC	dbSNP	ExAC	ExAC_SAS	1000G	1000G_SAS	SIFT	Polyphen2	CONDEL	VEP	LRT	PhastCon7Way	Total reads in Normal	Reference read support in Normal	Alternative read support in Normal	Total reads in Tumor	Reference read support in Tumor	Alternate read support in Tumor	Alternate allele frequency in Tumor
Non-user	IOB_24	chr2	86702091	T	G	KDM3A	SILENT	ENSG00000115548:[ENST00000312912(c.1917T>G,gcT>gcG):ENSP00000323659(p.Ala639(=))]	NA	NA	4.19E-03	1.67E-03	6.09E-04	4.19E-03	2.00E-03	NA	NA	NA	LOW	NA	NA	420	417	0	350	172	178	50.86
Non-user	IOB_24	chr2	88383970	G	A	SMYD1	SILENT	ENSG00000115593:[ENST00000419482(c.273G>A,tcG>tcA):ENSP00000393453(p.Ser91(=))]	NA	NA	2.20E-02	4.78E-02	5.27E-03	2.20E-02	5.10E-03	NA	NA	NA	MODIFIER	NA	NA	85	85	0	62	37	25	40.32
Non-user	IOB_24	chr2	91767379	C	T	NA	NCT	101926969:[XR_246529.1(n.685-11C>T)]	NA	NA	NA	NA	NA	NA	NA	NA	NA	NA	MODIFIER	NA	NA	305	304	0	314	267	46	14.65
Non-user	IOB_24	chr2	91940484	G	A	AC027612.4	EXONIC-NC	ENSG00000223703:[ENST00000445234(n.1508G>A)]	NA	NA	2.00E-04	NA	NA	2.00E-04	1.00E-03	NA	NA	NA	MODIFIER	NA	NA	215	213	1	229	192	36	15.72
Non-user	IOB_24	chr2	91955375	G	A	AC027612.4	EXONIC-NC	ENSG00000223703:[ENST00000445234(n.2305G>A)]	NA	NA	NA	NA	NA	NA	NA	NA	NA	NA	MODIFIER	NA	NA	136	136	0	131	106	24	18.32
Non-user	IOB_24	chr2	95537568	C	T	AC097374.2	EXONIC-NC	ENSG00000259848:[ENST00000597308(n.2169G>A)]	NA	chr2*95537568*95537568*COSM3695637*C*A*1*large_intestine*1|chr2*95537568*95537570*COSM97010*CGC*CCG*1*lung*1	1.06E-02	2.03E-02	4.14E-02	1.06E-02	3.07E-02	NA	NA	NA	MODIFIER	NA	NA	157	157	0	149	76	71	47.65
Non-user	IOB_24	chr2	95537568	C	T	LOC442028	NCT	442028:[NR_037597.1(n.1318-1126G>A)]	NA	chr2*95537568*95537568*COSM3695637*C*A*1*large_intestine*1|chr2*95537568*95537570*COSM97010*CGC*CCG*1*lung*1	1.06E-02	2.03E-02	4.14E-02	1.06E-02	3.07E-02	NA	NA	NA	MODIFIER	NA	NA	157	157	0	149	76	71	47.65
Non-user	IOB_24	chr2	95537568	C	T	TEKT4	MISSENSE	ENSG00000163060:[ENST00000295201(c.244C>T,Cgc>Tgc):ENSP00000295201(p.Arg82Cys)]	NA	chr2*95537568*95537568*COSM3695637*C*A*1*large_intestine*1|chr2*95537568*95537570*COSM97010*CGC*CCG*1*lung*1	1.06E-02	2.03E-02	4.14E-02	1.06E-02	3.07E-02	NA	NA	NA	MODIFIER	NA	NA	157	157	0	149	76	71	47.65
Non-user	IOB_24	chr2	96525687	C	G	ANKRD36C	MISSENSE	ENSG00000174501:[ENST00000456556(c.3818G>C,aGa>aCa):ENSP00000403302(p.Arg1273Thr)]	NA	NA	NA	NA	NA	NA	NA	T	PrD	NA	MODERATE	NA	NA	104	104	0	68	30	38	55.88
Non-user	IOB_24	chr2	96604606	C	A	ANKRD36C	MISSENSE	ENSG00000174501:[ENST00000456556(c.1604G>T,gGa>gTa):ENSP00000403302(p.Gly535Val)]	NA	NA	NA	2.26E-01	2.34E-01	NA	NA	NA	NA	NA	MODIFIER	NA	NA	912	906	2	604	506	95	15.73
Non-user	IOB_24	chr2	96619773	A	C	ANKRD36C	INTRONIC	ENSG00000174501:[ENST00000456556(c.1137-20T>G)]	NA	NA	NA	4.38E-02	3.31E-02	NA	NA	NA	NA	NA	MODIFIER	NA	NA	212	212	0	154	121	32	20.78
Non-user	IOB_24	chr2	96798305	C	T	ASTL	MISSENSE	ENSG00000188886:[ENST00000342380(c.611G>A,cGt>cAt):ENSP00000343674(p.Arg204His)]	NA	NA	2.88E-02	3.00E-02	2.83E-02	2.88E-02	2.35E-02	D	BN	NA	MODERATE	N	0	99	99	0	80	42	38	47.5
Non-user	IOB_24	chr2	96798441	G	A	ASTL	MISSENSE	ENSG00000188886:[ENST00000342380(c.475C>T,Cgc>Tgc):ENSP00000343674(p.Arg159Cys)]	NA	chr2*96798441*96798441*COSM2825270*G*A*1*central_nervous_system*1	NA	6.69E-05	2.49E-04	NA	NA	D	BN	NA	MODERATE	D	0.989	94	94	0	75	41	34	45.33
Non-user	IOB_24	chr2	96804107	G	A	ASTL	INTRONIC	ENSG00000188886:[ENST00000342380(c.55+14C>T)]	NA	NA	2.94E-02	4.45E-02	3.29E-02	2.94E-02	2.35E-02	NA	NA	NA	MODIFIER	NA	NA	155	153	1	86	56	29	33.72
Non-user	IOB_24	chr2	96874626	C	A	STARD7-AS1	EXONIC-NC	ENSG00000204685:[ENST00000446816(n.473C>A)]	NA	NA	2.88E-02	NA	NA	2.88E-02	2.15E-02	NA	NA	NA	MODIFIER	NA	NA	404	404	0	360	183	175	48.61
Non-user	IOB_24	chr2	96942978	C	G	SNRNP200	MISSENSE-SS-PRX	ENSG00000144028:[ENST00000429650(c.1514G>C,gGa>gCa):ENSP00000387870(p.Gly505Ala)]	NA	NA	4.99E-03	6.76E-03	5.40E-03	4.99E-03	4.10E-03	T	BN	NA	MODERATE	D	1	140	139	0	95	54	41	43.16
Non-user	IOB_24	chr2	96944480	C	G	SNRNP200	INTRONIC	ENSG00000144028:[ENST00000429650(c.905-31G>C)]	NA	NA	2.12E-02	1.62E-02	2.14E-02	2.12E-02	2.04E-02	NA	NA	NA	MODIFIER	NA	NA	215	213	2	166	92	74	44.58
Non-user	IOB_24	chr2	97526964	G	A	SEMA4C	MISSENSE	ENSG00000168758:[ENST00000305476(c.1901C>T,gCg>gTg):ENSP00000306844(p.Ala634Val)]	NA	NA	2.00E-04	6.11E-05	2.44E-04	2.00E-04	1.00E-03	NA	NA	NA	MODIFIER	NA	NA	325	325	0	304	161	141	46.38
Non-user	IOB_24	chr2	97827830	T	A	ANKRD36	INTRONIC	375248:[XM_005263937.1(c.133-6T>A)]	NA	NA	NA	2.89E-01	2.90E-01	NA	NA	NA	NA	NA	LOW	NA	NA	27	25	0	24	18	5	20.83
Non-user	IOB_24	chr2	97877478	G	A	ANKRD36	MISSENSE-SS-PRX	ENSG00000135976:[ENST00000420699(c.3469G>A,Gtg>Atg):ENSP00000391950(p.Val1157Met)]	NA	chr2*97877478*97877478*COSM1024079*G*A*1*endometrium*1|chr2*97877478*97877478*COSM1024078*G*A*1*endometrium*1	NA	3.95E-01	2.20E-01	NA	NA	NA	NA	NA	MODERATE	NA	NA	173	173	0	252	155	96	38.1
Non-user	IOB_24	chr2	98375414	G	A	TMEM131	MISSENSE	ENSG00000075568:[ENST00000186436(c.5309C>T,gCg>gTg):ENSP00000186436(p.Ala1770Val)]	NA	chr2*98375414*98375414*COSM1024117*G*A*1*endometrium*1|chr2*98375414*98375414*COSM1024118*G*A*1*endometrium*1	2.00E-04	5.87E-05	6.11E-05	2.00E-04	0.00E+00	NA	NA	NA	MODERATE	NA	0.793	178	178	0	111	54	56	50.45
Non-user	IOB_24	chr2	99172243	C	T	INPP4A	SILENT	ENSG00000040933:[ENST00000074304(c.1809C>T,tcC>tcT):ENSP00000074304(p.Ser603(=))]	NA	NA	2.54E-02	8.03E-02	2.67E-02	2.54E-02	2.86E-02	NA	NA	NA	MODIFIER	NA	NA	149	149	0	138	71	66	47.83
Non-user	IOB_24	chr2	99179971	C	G	INPP4A	SILENT	ENSG00000040933:[ENST00000409016(c.1797C>G,acC>acG):ENSP00000386704(p.Thr599(=))]	NA	NA	4.19E-03	1.13E-02	8.38E-03	4.19E-03	5.10E-03	NA	NA	NA	MODIFIER	NA	NA	159	159	0	116	64	52	44.83
Non-user	IOB_24	chr2	103124521	C	T	SLC9A4	INTRONIC	ENSG00000180251:[ENST00000295269(c.1199-17C>T)]	NA	NA	4.79E-03	2.28E-03	1.48E-02	4.79E-03	2.25E-02	NA	NA	NA	MODIFIER	NA	NA	182	182	0	169	87	82	48.52
Non-user	IOB_24	chr2	106987501	C	T	AC114755.7	EXONIC-NC	ENSG00000235486:[ENST00000447760(n.253C>T)]	NA	NA	1.50E-02	NA	NA	1.50E-02	1.12E-02	NA	NA	NA	MODIFIER	NA	NA	60	60	0	67	36	31	46.27
Non-user	IOB_24	chr2	108863728	C	T	SULT1C3	SILENT	ENSG00000196228:[ENST00000329106(c.78C>T,gtC>gtT):ENSP00000333310(p.Val26(=))]	NA	NA	6.59E-03	1.24E-02	4.20E-03	6.59E-03	3.10E-03	NA	NA	NA	LOW	NA	NA	200	200	0	136	71	64	47.06
Non-user	IOB_24	chr2	111556523	T	C	ACOXL	INTRONIC	ENSG00000153093:[ENST00000340561(c.461-68T>C)]	NA	NA	1.52E-02	NA	NA	1.52E-02	1.64E-02	NA	NA	NA	MODIFIER	NA	NA	169	166	0	131	69	61	46.56
Non-user	IOB_24	chr2	111881470	G	C	BCL2L11	MISSENSE	ENSG00000153094:[ENST00000357757(c.148G>C,Ggt>Cgt):ENSP00000350398(p.Gly50Arg)]	NA	NA	chr2*111881470*111881470*G*A,C*rs772949984*NA*NA*NA	7.68E-05	6.56E-04	NA	NA	NA	NA	NA	MODIFIER	NA	NA	166	165	0	162	82	78	48.15
Non-user	IOB_24	chr2	111902124	G	A	BCL2L11	NCT	10018:[XR_244809.1(n.503-5497G>A)]	NA	NA	5.79E-03	1.47E-02	1.16E-02	5.79E-03	4.10E-03	NA	NA	NA	MODIFIER	NA	NA	135	133	0	107	59	48	44.86
Non-user	IOB_24	chr2	113513814	C	T	CKAP2L	SILENT	ENSG00000169607:[ENST00000302450(c.1134G>A,agG>agA):ENSP00000305204(p.Arg378(=))]	NA	NA	6.39E-03	3.03E-03	2.17E-02	6.39E-03	3.27E-02	NA	NA	NA	LOW	NA	0.006	352	349	1	296	163	132	44.59
Non-user	IOB_24	chr2	113820167	C	T	IL36RN	SILENT	ENSG00000136695:[ENST00000514072(c.71C>T,gcC>gcT):ENSP00000475308(p.Ala24(=))]	NA	NA	NA	7.43E-05	4.24E-04	NA	NA	NA	NA	NA	LOW	NA	NA	149	149	0	103	59	44	42.72
Non-user	IOB_24	chr2	113940207	T	A	PSD4	SILENT	ENSG00000125637:[ENST00000245796(c.174T>A,ccT>ccA):ENSP00000245796(p.Pro58(=))]	NA	NA	4.31E-02	3.55E-02	9.91E-02	4.31E-02	9.82E-02	NA	NA	NA	MODIFIER	NA	NA	373	368	0	283	152	130	45.94
Non-user	IOB_24	chr2	113955443	C	A	PSD4	SILENT	ENSG00000125637:[ENST00000245796(c.2577C>A,gtC>gtA):ENSP00000245796(p.Val859(=))]	NA	NA	1.56E-02	3.46E-02	3.70E-02	1.56E-02	2.97E-02	NA	NA	NA	LOW	NA	NA	41	41	0	39	21	18	46.15
Non-user	IOB_24	chr2	118582274	G	T	DDX18	MISSENSE	ENSG00000088205:[ENST00000263239(c.1196G>T,gGt>gTt):ENSP00000263239(p.Gly399Val)]	NA	NA	NA	NA	NA	NA	NA	NA	NA	NA	MODIFIER	NA	NA	155	153	1	95	53	42	44.21
Non-user	IOB_24	chr2	127656861	C	T	AC114783.1	NONSENSE	ENSG00000237524:[ENST00000454927(c.268C>T,Cga>Tga):ENSP00000454243(p.Arg90Ter)]	NA	NA	4.93E-02	1.05E-01	9.78E-02	4.93E-02	8.69E-02	NA	NA	NA	HIGH	NA	NA	138	138	0	110	67	42	38.18
Non-user	IOB_24	chr2	127656861	C	T	LOC101929926	EXONIC-NC	101929926:[NR_136239.1(n.247C>T)]	NA	NA	4.93E-02	1.05E-01	9.78E-02	4.93E-02	8.69E-02	NA	NA	NA	HIGH	NA	NA	138	138	0	110	67	42	38.18
Non-user	IOB_24	chr2	128528508	C	T	WDR33	SILENT	ENSG00000136709:[ENST00000322313(c.48G>A,agG>agA):ENSP00000325377(p.Arg16(=))]	NA	NA	1.32E-02	3.95E-02	4.36E-03	1.32E-02	1.00E-03	NA	NA	NA	LOW	NA	0.988	94	94	0	105	53	52	49.52
Non-user	IOB_24	chr2	130750446	C	A	AC079776.6	EXONIC-NC	ENSG00000250207:[ENST00000511039(n.233G>T)]	NA	NA	NA	NA	NA	NA	NA	NA	NA	NA	MODIFIER	NA	NA	35	35	0	61	40	21	34.43
Non-user	IOB_24	chr2	130750446	C	A	FAR2P1	NCT	ENSG00000180178:[ENST00000440931(n.105-2335G>T)]	NA	NA	NA	NA	NA	NA	NA	NA	NA	NA	MODIFIER	NA	NA	35	35	0	61	40	21	34.43
Non-user	IOB_24	chr2	131704175	C	G	ARHGEF4	MISSENSE	ENSG00000136002:[ENST00000438985(c.934C>G,Cac>Gac):ENSP00000389661(p.His312Asp)]	NA	NA	NA	NA	NA	NA	NA	T	BN	NA	MODERATE	U	0.001	109	107	1	115	55	60	52.17
Non-user	IOB_24	chr2	133540061	A	C	NCKAP5	MISSENSE	ENSG00000176771:[ENST00000317721(c.4323T>G,agT>agG):ENSP00000380603(p.Ser1441Arg)]	NA	NA	7.99E-04	1.81E-03	2.69E-03	7.99E-04	2.00E-03	NA	NA	NA	MODIFIER	NA	NA	160	159	0	137	74	61	44.53
Non-user	IOB_24	chr2	133540776	G	A	NCKAP5	MISSENSE	ENSG00000176771:[ENST00000317721(c.3608C>T,gCg>gTg):ENSP00000380603(p.Ala1203Val)]	NA	NA	NA	3.48E-04	5.47E-04	NA	NA	NA	NA	NA	MODIFIER	NA	NA	215	215	0	132	59	72	54.55
Non-user	IOB_24	chr2	133712259	T	C	NCKAP5	INTRONIC	ENSG00000176771:[ENST00000317721(c.579+9034A>G)]	NA	NA	2.00E-03	7.21E-03	7.75E-03	2.00E-03	5.10E-03	NA	NA	NA	MODIFIER	NA	NA	61	61	0	34	18	16	47.06
Non-user	IOB_24	chr2	135922290	C	A	RAB3GAP1	INTRONIC	ENSG00000115839:[ENST00000264158(c.2709+24C>A)]	NA	NA	4.57E-02	4.85E-02	5.94E-02	4.57E-02	4.29E-02	NA	NA	NA	MODIFIER	NA	NA	40	40	0	38	24	14	36.84
Non-user	IOB_24	chr2	135922290	C	A	ZRANB3	NCT	ENSG00000121988:[ENST00000412849(n.1793+423G>T)]	NA	NA	4.57E-02	4.85E-02	5.94E-02	4.57E-02	4.29E-02	NA	NA	NA	MODIFIER	NA	NA	40	40	0	38	24	14	36.84
Non-user	IOB_24	chr2	138771373	A	G	HNMT	SILENT	ENSG00000150540:[ENST00000280097(c.552A>G,aaA>aaG):ENSP00000280097(p.Lys184(=))]	NA	NA	NA	NA	NA	NA	NA	NA	NA	NA	MODIFIER	NA	NA	206	203	0	130	57	72	55.38
Non-user	IOB_24	chr2	138771373	A	G	NA	NCT	101928299:[XR_244863.1(n.579-6007T>C)]	NA	NA	NA	NA	NA	NA	NA	NA	NA	NA	MODIFIER	NA	NA	206	203	0	130	57	72	55.38
Non-user	IOB_24	chr2	139045818	G	A	YWHAEP5	EXONIC-NC	ENSG00000234645:[ENST00000431545(n.546C>T)]	NA	chr2*139045818*139045818*COSM1006957*G*A*1*endometrium*1	1.60E-03	NA	NA	1.60E-03	7.20E-03	NA	NA	NA	MODIFIER	NA	NA	280	279	0	241	134	107	44.4
Non-user	IOB_24	chr2	139308191	C	A	SPOPL	INTRONIC	ENSG00000144228:[ENST00000280098(c.200+17C>A)]	NA	NA	1.58E-02	2.98E-02	5.29E-02	1.58E-02	3.78E-02	NA	NA	NA	MODIFIER	NA	NA	62	62	0	50	22	28	56
Non-user	IOB_24	chr2	142238097	C	T	LRP1B	MISSENSE	ENSG00000168702:[ENST00000389484(c.211G>A,Gag>Aag):ENSP00000374135(p.Glu71Lys)]	NA	NA	chr2*142238097*142238097*C*G,T*rs145250710*NA*NA*NA	1.16E-04	0.00E+00	NA	NA	NA	NA	NA	MODIFIER	NA	NA	184	183	0	138	65	73	52.9
Non-user	IOB_24	chr2	148730367	G	C	ORC4	MISSENSE	ENSG00000115947:[ENST00000264169(c.166C>G,Ctc>Gtc):ENSP00000264169(p.Leu56Val)]	NA	NA	1.72E-02	3.81E-02	2.75E-02	1.72E-02	1.53E-02	NA	NA	NA	MODIFIER	NA	NA	107	107	0	88	52	36	40.91
Non-user	IOB_24	chr2	152276851	A	G	RIF1	SILENT	ENSG00000080345:[ENST00000414861(c.625A>G,aaA>aaG):ENSP00000390486(p.Lys209(=))]	NA	NA	3.99E-04	2.40E-04	1.52E-03	3.99E-04	2.00E-03	NA	NA	NA	MODIFIER	NA	NA	198	197	0	130	82	47	36.15
Non-user	IOB_24	chr2	152279645	A	T	RIF1	INTRONIC	ENSG00000080345:[ENST00000414861(c.760+89A>T)]	NA	NA	5.99E-03	NA	NA	5.99E-03	4.10E-03	NA	NA	NA	MODIFIER	NA	NA	42	42	0	34	22	12	35.29
Non-user	IOB_24	chr2	152529122	T	C	NEB	MISSENSE	ENSG00000183091:[ENST00000172853(c.4060A>G,Aag>Gag):ENSP00000172853(p.Lys1354Glu)]	NA	NA	NA	3.15E-04	2.18E-03	NA	NA	T	PrD	NA	MODERATE	D	1	168	167	1	115	50	65	56.52
Non-user	IOB_24	chr2	158485075	G	T	ACVR1C	INTRONIC-SS-DNR-PRX	ENSG00000123612:[ENST00000243349(c.73+9C>A)]	NA	NA	2.08E-02	5.03E-02	3.00E-02	2.08E-02	1.53E-02	NA	NA	NA	MODIFIER	NA	NA	266	264	0	198	117	78	39.39
Non-user	IOB_24	chr2	160636492	A	G	CD302	INTRONIC	9936:[XM_005246982.1(c.412+24T>C)]	NA	NA	1.20E-02	2.88E-02	7.18E-03	1.20E-02	8.20E-03	NA	NA	NA	MODIFIER	NA	NA	305	303	1	213	99	114	53.52
Non-user	IOB_24	chr2	160636492	A	G	LY75	INTRONIC	ENSG00000054219:[ENST00000554112(c.5392+24T>C)]	NA	NA	1.20E-02	2.88E-02	7.18E-03	1.20E-02	8.20E-03	NA	NA	NA	MODIFIER	NA	NA	305	303	1	213	99	114	53.52
Non-user	IOB_24	chr2	160636492	A	G	LY75-CD302	INTRONIC	ENSG00000248672:[ENST00000504764(c.5392+24T>C)]	NA	NA	1.20E-02	2.88E-02	7.18E-03	1.20E-02	8.20E-03	NA	NA	NA	MODIFIER	NA	NA	305	303	1	213	99	114	53.52
Non-user	IOB_24	chr2	165424847	G	A	GRB14	MISSENSE	ENSG00000115290:[ENST00000543549(c.41C>T,aCg>aTg):ENSP00000443699(p.Thr14Met)]	NA	NA	NA	NA	NA	NA	NA	NA	NA	NA	MODIFIER	NA	NA	169	167	1	147	73	72	48.98
Non-user	IOB_24	chr2	166179779	T	C	SCN2A	SILENT	ENSG00000136531:[ENST00000283256(c.1785T>C,gaT>gaC):ENSP00000283256(p.Asp595(=))]	NA	NA	5.19E-03	9.24E-03	2.06E-03	5.19E-03	1.00E-03	NA	NA	NA	LOW	NA	1	342	342	0	248	127	119	47.98
Non-user	IOB_24	chr2	167277777	G	A	SCN7A	MISSENSE	ENSG00000136546:[ENST00000409855(c.3058C>T,Cgg>Tgg):ENSP00000386796(p.Arg1020Trp)]	NA	NA	2.00E-04	6.93E-04	1.78E-03	2.00E-04	1.00E-03	NA	NA	NA	MODIFIER	NA	NA	16	16	0	9	3	6	66.67
Non-user	IOB_24	chr2	169417697	C	T	CERS6	INTRONIC-SS-ACR-PRX	ENSG00000172292:[ENST00000305747(c.277-5C>T)]	NA	NA	3.40E-03	1.58E-03	1.16E-02	3.39E-03	1.64E-02	NA	NA	NA	LOW	NA	NA	149	149	0	91	41	50	54.95
Non-user	IOB_24	chr2	170038761	C	T	LRP2	MISSENSE	ENSG00000081479:[ENST00000263816(c.9914G>A,cGc>cAc):ENSP00000263816(p.Arg3305His)]	NA	NA	3.20E-03	3.24E-03	3.03E-04	3.19E-03	0.00E+00	NA	NA	NA	MODIFIER	NA	NA	94	94	0	80	53	27	33.75
Non-user	IOB_24	chr2	170218871	G	A	LRP2	SILENT	ENSG00000081479:[ENST00000263816(c.39C>T,ctC>ctT):ENSP00000263816(p.Leu13(=))]	NA	NA	1.20E-03	2.65E-03	7.90E-03	1.20E-03	6.10E-03	NA	NA	NA	LOW	NA	NA	177	177	0	131	57	74	56.49
Non-user	IOB_24	chr2	173337618	G	A	ITGA6	MISSENSE-SS-PRX	ENSG00000091409:[ENST00000264107(c.776-1165G>A)]	NA	NA	1.38E-02	1.05E-02	4.33E-02	1.38E-02	3.17E-02	D	PrD	NA	MODERATE	NA	NA	196	196	0	158	76	82	51.9
Non-user	IOB_24	chr2	173337618	G	A	NA	NCT	101926931:[XR_241350.1(n.462-6869C>T)]	NA	NA	1.38E-02	1.05E-02	4.33E-02	1.38E-02	3.17E-02	D	PrD	NA	MODERATE	NA	NA	196	196	0	158	76	82	51.9
Non-user	IOB_24	chr2	175006700	G	A	OLA1	SILENT	ENSG00000138430:[ENST00000409546(c.462C>T,caC>caT):ENSP00000386350(p.His154(=))]	NA	NA	3.79E-03	6.44E-03	7.21E-03	3.79E-03	1.12E-02	NA	NA	NA	MODIFIER	NA	NA	54	54	0	31	16	12	38.71
Non-user	IOB_24	chr2	175742880	T	A	CHN1	NCT	ENSG00000128656:[ENST00000469597(n.366-24A>T)]	NA	NA	6.19E-03	7.10E-03	5.81E-03	6.19E-03	1.02E-02	NA	NA	NA	MODIFIER	NA	NA	156	153	0	111	60	51	45.95
Non-user	IOB_24	chr2	176995559	G	A	HOXD8	SILENT	ENSG00000175879:[ENST00000548663(c.153G>A,gaG>gaA):ENSP00000448196(p.Glu51(=))]	NA	NA	3.99E-04	6.42E-04	3.10E-03	3.99E-04	2.00E-03	NA	NA	NA	MODIFIER	NA	NA	179	179	0	156	69	84	53.85
Non-user	IOB_24	chr2	176995559	G	A	HOXD-AS2	NCT	ENSG00000237380:[ENST00000440016(n.497+4488C>T)]	NA	NA	3.99E-04	6.42E-04	3.10E-03	3.99E-04	2.00E-03	NA	NA	NA	MODIFIER	NA	NA	179	179	0	156	69	84	53.85
Non-user	IOB_24	chr2	178257724	A	G	AGPS	SILENT	ENSG00000018510:[ENST00000264167(c.207A>G,gcA>gcG):ENSP00000264167(p.Ala69(=))]	NA	NA	3.36E-02	3.84E-02	5.04E-02	3.35E-02	6.03E-02	NA	NA	NA	LOW	NA	NA	255	254	0	202	97	105	51.98

Patel *et al.* , 2021. Whole-exome sequencing analysis of oral squamous cell carcinoma delineated by tobacco usage habits
Supplementary Table 4. List of somatic single nucleotide variants identified using whole exome sequencing of OSCC patients

Cohort	Sample	Chromosome	Genomic position	Reference allele	Alternate allele	Genesymbol	Variant type	Variant description	OncoMD	COSMIC	dbSNP	ExAC	ExAC_SAS	1000G	1000G_SAS	SIFT	Polyphen2	CONDEL	VEP	LRT	PhastCon7Way	Total reads in Normal	Reference read support in Normal	Alternative read support in Normal	Total reads in Tumor	Reference read support in Tumor	Alternate read support in Tumor	Alternate allele frequency in Tumor
Non-user	IOB_24	chr2	178364491	G	C	AGPS	INTRONIC	8540:[XM_005246917.1(c.1205+33G>C)]	NA	NA	2.26E-02	1.53E-02	5.44E-02	2.26E-02	6.03E-02	NA	NA	NA	MODIFIER	NA	NA	61	61	0	51	25	26	50.98
Non-user	IOB_24	chr2	178482269	A	G	AC073834.3	NCT	ENSG00000237655:[ENST00000357045(n.305+21A>G)]	NA	NA	4.59E-02	2.35E-02	4.46E-02	4.59E-02	4.60E-02	NA	NA	NA	MODIFIER	NA	NA	268	267	0	200	106	93	46.5
Non-user	IOB_24	chr2	178482269	A	G	TTC30A	SILENT	ENSG00000197557:[ENST00000355689(c.1161T>C,acT>acC):ENSP00000347915(p.Thr387(=))]	NA	NA	4.59E-02	2.35E-02	4.46E-02	4.59E-02	4.60E-02	NA	NA	NA	MODIFIER	NA	NA	268	267	0	200	106	93	46.5
Non-user	IOB_24	chr2	178879078	G	A	PDE11A	MISSENSE	ENSG00000128655:[ENST00000358450(c.272C>T,gCg>gTg):ENSP00000351232(p.Ala91Val)]	NA	chr2*178879078*178879078*COSM75888*G*A*2*ovary,large_intestine*1,1|chr2*178879078*178879078*COSM1401511*G*A*1*large_intestine*1	NA	NA	NA	NA	NA	T	BN	NA	MODERATE	D	1	464	463	1	387	380	7	1.81
Non-user	IOB_24	chr2	179400360	C	T	TTN	MISSENSE	ENSG00000155657:[ENST00000460472(c.73787G>A,aGa>aAa):ENSP00000434586(p.Arg24596Lys)]	NA	chr2*179400360*179400360*COSM418455*C*G*1*urinary_tract*1|chr2*179400360*179400360*COSM418456*C*G*1*urinary_tract*1|chr2*179400360*179400360*COSM418457*C*G*1*urinary_tract*1|chr2*179400360*179400360*COSM1133584*C*G*1*urinary_tract*1|chr2*179400360*179400360*COSM418458*C*G*1*urinary_tr	7.99E-04	4.14E-04	3.03E-03	7.99E-04	4.10E-03	NA	PrD	NA	MODERATE	NA	NA	178	178	0	141	72	68	48.23
Non-user	IOB_24	chr2	179400360	C	T	TTN-AS1	NCT	ENSG00000237298:[ENST00000588244(n.340-99C>T)]	NA	chr2*179400360*179400360*COSM418455*C*G*1*urinary_tract*1|chr2*179400360*179400360*COSM418456*C*G*1*urinary_tract*1|chr2*179400360*179400360*COSM418457*C*G*1*urinary_tract*1|chr2*179400360*179400360*COSM1133584*C*G*1*urinary_tract*1|chr2*179400360*179400360*COSM418458*C*G*1*urinary_tr	7.99E-04	4.14E-04	3.03E-03	7.99E-04	4.10E-03	NA	PrD	NA	MODERATE	NA	NA	178	178	0	141	72	68	48.23
Non-user	IOB_24	chr2	179441479	C	T	TTN	SILENT	ENSG00000155657:[ENST00000589042(c.69492G>A,gtG>gtA):ENSP00000467141(p.Val23164(=))]	NA	NA	NA	4.97E-05	3.63E-04	NA	NA	NA	NA	NA	MODIFIER	NA	NA	295	294	0	264	129	131	49.62
Non-user	IOB_24	chr2	179441479	C	T	TTN-AS1	NCT	ENSG00000237298:[ENST00000592630(n.434-20844C>T)]	NA	NA	NA	4.97E-05	3.63E-04	NA	NA	NA	NA	NA	MODIFIER	NA	NA	295	294	0	264	129	131	49.62
Non-user	IOB_24	chr2	179585723	C	A	TTN	MISSENSE	ENSG00000155657:[ENST00000591111(c.22072G>T,Gac>Tac):ENSP00000465570(p.Asp7358Tyr)]	NA	NA	2.20E-03	1.36E-03	9.94E-03	2.20E-03	1.12E-02	NA	NA	NA	MODERATE	NA	NA	268	268	0	232	109	123	53.02
Non-user	IOB_24	chr2	179585723	C	A	TTN-AS1	NCT	ENSG00000237298:[ENST00000585451(n.199-39944C>A)]	NA	NA	2.20E-03	1.36E-03	9.94E-03	2.20E-03	1.12E-02	NA	NA	NA	MODERATE	NA	NA	268	268	0	232	109	123	53.02
Non-user	IOB_24	chr2	179634392	A	T	TTN	INTRONIC	ENSG00000155657:[ENST00000342992(c.8902+14T>A)]	NA	NA	1.80E-03	1.78E-03	3.34E-03	1.80E-03	0.00E+00	NA	NA	NA	MODIFIER	NA	NA	59	59	0	49	0	48	97.96
Non-user	IOB_24	chr2	179634392	A	T	TTN-AS1	NCT	ENSG00000237298:[ENST00000610005(n.562-3610A>T)]	NA	NA	1.80E-03	1.78E-03	3.34E-03	1.80E-03	0.00E+00	NA	NA	NA	MODIFIER	NA	NA	59	59	0	49	0	48	97.96
Non-user	IOB_24	chr2	179654792	T	G	TTN	SILENT	ENSG00000155657:[ENST00000342992(c.1851A>C,acA>acC):ENSP00000343764(p.Thr617(=))]	NA	NA	NA	1.65E-05	0.00E+00	NA	NA	NA	NA	NA	LOW	NA	NA	95	95	0	104	35	68	65.38
Non-user	IOB_24	chr2	186657774	T	C	AC008174.3	NCT	ENSG00000231646:[ENST00000436557(n.72-4104A>G)]	NA	NA	NA	NA	NA	NA	NA	NA	BN	NA	MODERATE	NA	NA	267	266	0	228	112	116	50.88
Non-user	IOB_24	chr2	186657774	T	C	FSIP2	MISSENSE	ENSG00000188738:[ENST00000343098(c.6178T>C,Tat>Cat):ENSP00000344403(p.Tyr2060His)]	NA	NA	NA	NA	NA	NA	NA	NA	BN	NA	MODERATE	NA	NA	267	266	0	228	112	116	50.88
Non-user	IOB_24	chr2	189931477	T	A	COL5A2	MISSENSE	ENSG00000204262:[ENST00000374866(c.1433A>T,gAa>gTa):ENSP00000364000(p.Glu478Val)]	NA	NA	3.99E-04	2.48E-04	1.82E-03	3.99E-04	2.00E-03	D	PoD	NA	MODERATE	D	1	224	221	1	177	88	88	49.72
Non-user	IOB_24	chr2	196722384	T	C	DNAH7	MISSENSE	ENSG00000118997:[ENST00000312428(c.8131A>G,Ata>Gta):ENSP00000311273(p.Ile2711Val)]	NA	NA	9.98E-04	1.03E-03	6.93E-03	9.98E-04	5.10E-03	T	BN	NA	MODERATE	D	0.977	155	155	0	107	57	49	45.79
Non-user	IOB_24	chr2	196822004	A	G	DNAH7	MISSENSE	ENSG00000118997:[ENST00000312428(c.3059T>C,aTa>aCa):ENSP00000311273(p.Ile1020Thr)]	NA	NA	4.77E-02	9.08E-02	1.01E-01	4.77E-02	6.85E-02	T	BN	NA	MODERATE	N	0.116	45	45	0	36	22	14	38.89
Non-user	IOB_24	chr2	197184552	G	A	HECW2	SILENT	ENSG00000138411:[ENST00000260983(c.1062C>T,gaC>gaT):ENSP00000260983(p.Asp354(=))]	NA	chr2*197184552*197184552*COSM81256*G*A*1*ovary*1	3.99E-04	3.39E-04	1.76E-03	3.99E-04	0.00E+00	NA	NA	NA	LOW	NA	0.805	218	216	0	203	101	101	49.75
Non-user	IOB_24	chr2	201437221	C	T	SGOL2	MISSENSE	ENSG00000163535:[ENST00000357799(c.2152C>T,Cgg>Tgg):ENSP00000350447(p.Arg718Trp)]	NA	chr2*201437221*201437221*COSM1014709*C*T*2*large_intestine,endometrium*1,1	NA	8.37E-06	6.09E-05	NA	NA	D	PrD	NA	MODERATE	N	0.649	179	179	0	104	46	57	54.81
Non-user	IOB_24	chr2	201857136	C	T	AC005037.3	NCT	ENSG00000183308:[ENST00000413848(n.369-16573C>T)]	NA	NA	3.57E-02	1.98E-02	1.01E-01	3.57E-02	1.46E-01	NA	NA	NA	MODIFIER	NA	NA	148	148	0	85	0	83	97.65
Non-user	IOB_24	chr2	201857136	C	T	FAM126B	INTRONIC	ENSG00000155744:[ENST00000286181(c.*497-45G>A)]	NA	NA	3.57E-02	1.98E-02	1.01E-01	3.57E-02	1.46E-01	NA	NA	NA	MODIFIER	NA	NA	148	148	0	85	0	83	97.65
Non-user	IOB_24	chr2	203825974	A	G	CARF	SILENT	ENSG00000138380:[ENST00000428585(c.429A>G,tcA>tcG):ENSP00000396221(p.Ser143(=))]	NA	NA	NA	2.50E-05	1.88E-04	NA	NA	NA	NA	NA	MODIFIER	NA	NA	203	203	0	127	73	53	41.73
Non-user	IOB_24	chr2	203825974	A	G	WDR12	NCT	ENSG00000138442:[ENST00000477723(n.243-35783T>C)]	NA	NA	NA	2.50E-05	1.88E-04	NA	NA	NA	NA	NA	MODIFIER	NA	NA	203	203	0	127	73	53	41.73
Non-user	IOB_24	chr2	204103705	G	T	CYP20A1	5UTR	57404:[XM_005246699.1(c.-170G>T)]	NA	NA	chr2*204103705*204103705*G*C*rs868431215*NA*NA*NA	NA	NA	NA	NA	NA	NA	NA	MODIFIER	NA	NA	139	138	0	110	54	56	50.91
Non-user	IOB_24	chr2	207023705	T	C	NDUFS1	INTRONIC	ENSG00000023228:[ENST00000233190(c.-5+360A>G)]	NA	NA	2.00E-04	NA	NA	2.00E-04	1.00E-03	NA	NA	NA	MODIFIER	NA	NA	46	46	0	36	21	15	41.67
Non-user	IOB_24	chr2	207564581	G	A	DYTN	SILENT	ENSG00000232125:[ENST00000452335(c.589C>T,Ctg>Ttg):ENSP00000396593(p.Leu197(=))]	NA	NA	NA	NA	NA	NA	NA	NA	NA	NA	MODIFIER	NA	NA	272	270	0	213	103	109	51.17
Non-user	IOB_24	chr2	210642294	A	T	UNC80	NCT	ENSG00000144406:[ENST00000478701(n.680+11A>T)]	NA	NA	7.59E-03	1.06E-02	1.11E-02	7.59E-03	1.02E-02	NA	NA	NA	MODIFIER	NA	NA	55	53	1	32	18	14	43.75
Non-user	IOB_24	chr2	210682485	A	G	UNC80	INTRONIC	ENSG00000144406:[ENST00000272845(c.1553-51A>G)]	NA	NA	2.26E-02	NA	NA	2.26E-02	2.35E-02	NA	NA	NA	MODIFIER	NA	NA	57	57	0	47	22	25	53.19
Non-user	IOB_24	chr2	211085491	G	A	AC006994.2	NCT	ENSG00000231294:[ENST00000412065(n.405+2203G>A)]	NA	NA	1.56E-02	2.83E-02	2.82E-02	1.56E-02	2.04E-02	NA	NA	NA	MODIFIER	NA	NA	28	28	0	15	9	6	40
Non-user	IOB_24	chr2	211085491	G	A	ACADL	MISSENSE	ENSG00000115361:[ENST00000233710(c.113C>T,aCt>aTt):ENSP00000233710(p.Thr38Ile)]	NA	NA	1.56E-02	2.83E-02	2.82E-02	1.56E-02	2.04E-02	NA	NA	NA	MODIFIER	NA	NA	28	28	0	15	9	6	40
Non-user	IOB_24	chr2	216176824	C	G	ATIC	EXONIC-NC	ENSG00000138363:[ENST00000488712(n.110C>G)]	NA	NA	4.97E-02	3.96E-02	4.99E-02	4.97E-02	4.29E-02	NA	NA	NA	MODIFIER	NA	NA	22	22	0	38	20	18	47.37
Non-user	IOB_24	chr2	216990772	A	G	XRCC5	NCT	ENSG00000079246:[ENST00000493706(n.109+18A>G)]	NA	NA	2.46E-02	4.03E-02	6.29E-02	2.46E-02	6.03E-02	NA	NA	NA	MODIFIER	NA	NA	150	150	0	125	52	72	57.6
Non-user	IOB_24	chr2	217315664	C	T	SMARCAL1	SILENT	ENSG00000138375:[ENST00000357276(c.1947C>T,gaC>gaT):ENSP00000349823(p.Asp649(=))]	NA	NA	1.20E-02	2.35E-02	2.83E-02	1.20E-02	2.66E-02	NA	NA	NA	LOW	NA	NA	98	98	0	49	29	20	40.82
Non-user	IOB_24	chr2	218568732	C	G	DIRC3	MISSENSE	ENSG00000231672:[ENST00000423123(c.340G>C,Gta>Cta):ENSP00000389455(p.Val114Leu)]	NA	NA	3.99E-03	1.38E-02	1.50E-02	3.99E-03	4.10E-03	NA	BN	NA	MODERATE	NA	NA	59	59	0	26	10	16	61.54
Non-user	IOB_24	chr2	218713469	G	A	TNS1	MISSENSE	ENSG00000079308:[ENST00000171887(c.1396C>T,Cgc>Tgc):ENSP00000171887(p.Arg466Cys)]	NA	NA	1.54E-02	2.00E-02	3.98E-02	1.54E-02	2.56E-02	T	BN	NA	MODERATE	NA	NA	223	221	1	166	90	75	45.18
Non-user	IOB_24	chr2	219000460	C	T	CXCR2	SILENT	ENSG00000180871:[ENST00000318507(c.936C>T,ctC>ctT):ENSP00000319635(p.Leu312(=))]	NA	NA	1.12E-02	1.40E-02	3.54E-02	1.12E-02	4.40E-02	NA	NA	NA	LOW	NA	NA	463	459	0	381	178	198	51.97
Non-user	IOB_24	chr2	219134762	C	T	AAMP	SILENT	ENSG00000127837:[ENST00000248450(c.48G>A,ctG>ctA):ENSP00000248450(p.Leu16(=))]	NA	NA	4.99E-03	2.18E-03	1.58E-02	4.99E-03	2.56E-02	NA	NA	NA	LOW	NA	NA	271	270	0	248	123	122	49.19
Non-user	IOB_24	chr2	219563651	C	T	STK36	SILENT	ENSG00000163482:[ENST00000295709(c.3384C>T,caC>caT):ENSP00000295709(p.His1128(=))]	NA	NA	NA	NA	NA	NA	NA	NA	NA	NA	MODIFIER	NA	NA	244	243	0	204	197	5	2.45
Non-user	IOB_24	chr2	219678877	C	T	CYP27A1	MISSENSE	ENSG00000135929:[ENST00000258415(c.1151C>T,cCg>cTg):ENSP00000258415(p.Pro384Leu)]	NA	NA	8.59E-03	1.88E-02	3.05E-02	8.59E-03	1.43E-02	D	PrD	NA	MODERATE	D	1	208	206	1	143	83	60	41.96
Non-user	IOB_24	chr2	219866336	G	C	NA	INTERGENIC	NA	NA	NA	4.65E-02	6.15E-02	6.57E-02	4.65E-02	4.70E-02	NA	NA	NA	MODIFIER	NA	NA	107	106	0	85	51	34	40
Non-user	IOB_24	chr2	220011537	A	T	NHEJ1	INTRONIC	ENSG00000187736:[ENST00000356853(c.530-77T>A)]	NA	NA	1.04E-02	NA	NA	1.04E-02	3.99E-02	NA	NA	NA	MODIFIER	NA	NA	55	55	0	44	26	18	40.91
Non-user	IOB_24	chr2	220011537	A	T	SLC23A3	NCT	ENSG00000213901:[ENST00000498327(n.2718-77T>A)]	NA	NA	1.04E-02	NA	NA	1.04E-02	3.99E-02	NA	NA	NA	MODIFIER	NA	NA	55	55	0	44	26	18	40.91
Non-user	IOB_24	chr2	220045054	C	T	FAM134A	INTRONIC	ENSG00000144567:[ENST00000430297(c.555+87C>T)]	NA	NA	2.00E-03	NA	NA	2.00E-03	1.02E-02	NA	NA	NA	MODIFIER	NA	NA	22	22	0	25	14	11	44
Non-user	IOB_24	chr2	220078017	C	T	ABCB6	MISSENSE	ENSG00000115657:[ENST00000439002(c.1613G>A,cGc>cAc):ENSP00000394333(p.Arg538His)]	NA	chr2*220078017*220078017*COSM3044586*C*T*1*large_intestine*1	NA	2.47E-05	0.00E+00	NA	NA	T	BN	NA	MODERATE	NA	NA	222	219	0	178	86	92	51.69
Non-user	IOB_24	chr2	220078017	C	T	ATG9A	3UTR	ENSG00000198925:[ENST00000446716(c.*3710G>A)]	NA	chr2*220078017*220078017*COSM3044586*C*T*1*large_intestine*1	NA	2.47E-05	0.00E+00	NA	NA	T	BN	NA	MODERATE	NA	NA	222	219	0	178	86	92	51.69
Non-user	IOB_24	chr2	220251685	T	G	DNPEP	MISSENSE	ENSG00000123992:[ENST00000429013(c.106A>C,Aac>Cac):ENSP00000400944(p.Asn36His)]	NA	NA	1.22E-02	2.22E-02	2.78E-02	1.22E-02	2.25E-02	NA	NA	NA	MODIFIER	NA	NA	76	76	0	60	29	31	51.67
Non-user	IOB_24	chr2	220420716	G	A	OBSL1	3UTR	ENSG00000124006:[ENST00000265317(c.*3C>T)]	NA	NA	9.78E-03	6.82E-03	5.59E-02	9.78E-03	5.01E-02	NA	NA	NA	MODIFIER	NA	NA	160	158	0	106	66	40	37.74
Non-user	IOB_24	chr2	220431802	G	A	OBSL1	SILENT	ENSG00000124006:[ENST00000289656(c.645C>T,taC>taT):ENSP00000289656(p.Tyr215(=))]	NA	NA	1.48E-02	2.33E-02	5.60E-02	1.48E-02	5.01E-02	NA	NA	NA	LOW	NA	NA	191	190	0	139	70	69	49.64
Non-user	IOB_24	chr2	220439916	G	A	INHA	MISSENSE	ENSG00000123999:[ENST00000243786(c.769G>A,Gct>Act):ENSP00000243786(p.Ala257Thr)]	NA	NA	1.50E-02	2.36E-02	5.56E-02	1.50E-02	5.11E-02	T	BN	NA	MODERATE	N	0	350	350	0	250	119	131	52.4
Non-user	IOB_24	chr2	222365938	T	C	EPHA4	INTRONIC	ENSG00000116106:[ENST00000443796(c.92-18528A>G)]	NA	NA	4.41E-02	2.95E-02	7.16E-02	4.41E-02	7.87E-02	NA	NA	NA	MODIFIER	NA	NA	45	45	0	37	24	13	35.14
Non-user	IOB_24	chr2	223509314	G	A	FARSB	NCT	10056:[NR_130154.1(n.593-1590C>T)]	NA	NA	9.98E-04	3.91E-03	5.17E-03	9.98E-04	3.10E-03	NA	NA	NA	MODIFIER	NA	NA	116	116	0	76	38	38	50
Non-user	IOB_24	chr2	225670122	T	A	DOCK10	INTRONIC	55619:[XM_005246673.1(c.3975+3A>T)]	NA	NA	NA	NA	NA	NA	NA	NA	NA	NA	LOW	NA	NA	188	188	0	136	79	56	41.18
Non-user	IOB_24	chr2	228217260	C	A	MFF	SILENT	ENSG00000168958:[ENST00000304593(c.630C>A,gcC>gcA):ENSP00000304898(p.Ala210(=))]	NA	NA	1.10E-02	1.22E-02	4.37E-02	1.10E-02	3.89E-02	NA	NA	NA	MODIFIER	NA	NA	221	220	1	151	77	74	49.01
Non-user	IOB_24	chr2	228336873	C	T	AGFG1	5UTR	ENSG00000173744:[ENST00000409979(c.-265C>T)]	NA	NA	NA	NA	NA	NA	NA	NA	NA	NA	MODIFIER	NA	NA	88	88	0	97	53	44	45.36
Non-user	IOB_24	chr2	228336873	C	T	MIR5703	EXONIC-NC	ENSG00000266382:[ENST00000582757(n.26C>T)]	NA	NA	NA	NA	NA	NA	NA	NA	NA	NA	MODIFIER	NA	NA	88	88	0	97	53	44	45.36
Non-user	IOB_24	chr2	230377585	T	C	DNER	MISSENSE	ENSG00000187957:[ENST00000341772(c.1061A>G,cAg>cGg):ENSP00000345229(p.Gln354Arg)]	NA	chr2*230377583*230377585*COSM394261*TCT*TAA*1*lung*1	chr2*230377585*230377585*T*G*rs751461005*NA*NA*NA	NA	NA	NA	NA	T	BN	NA	MODERATE	D	1	247	246	0	209	111	97	46.41
Non-user	IOB_24	chr2	232325349	C	A	NCL	NCT	ENSG00000115053:[ENST00000494618(n.57+31G>T)]	NA	NA	3.65E-02	4.66E-02	4.03E-02	3.65E-02	4.19E-02	NA	NA	NA	MODIFIER	NA	NA	335	335	0	275	141	132	48
Non-user	IOB_24	chr2	232327273	G	A	NCL	INTRONIC	ENSG00000115053:[ENST00000417652(c.88-545C>T)]	NA	NA	5.59E-03	1.37E-02	5.26E-03	5.59E-03	3.10E-03	NA	NA	NA	MODIFIER	NA	NA	74	74	0	66	42	24	36.36
Non-user	IOB_24	chr2	232457880	T	C	C2orf57	MISSENSE	ENSG00000177673:[ENST00000313965(c.218T>C,gTt>gCt):ENSP00000315557(p.Val73Ala)]	NA	NA	3.26E-02	5.68E-02	4.16E-02	3.25E-02	4.81E-02	T	BN	NA	MODERATE	NA	NA	171	171	0	101	48	52	51.49
Non-user	IOB_24	chr2	232574908	C	T	PTMA	MISSENSE	ENSG00000187514:[ENST00000410064(c.20C>T,gCc>gTc):ENSP00000387102(p.Ala7Val)]	NA	NA	6.99E-03	4.42E-02	2.85E-02	6.99E-03	4.10E-03	NA	NA	NA	MODIFIER	NA	NA	48	48	0	36	22	12	33.33
Non-user	IOB_24	chr2	232575280	G	A	PTMA	MISSENSE	ENSG00000187514:[ENST00000412128(c.144G>A,Gga>Aga):ENSP00000406903(p.Gly49Arg)]	NA	NA	6.99E-03	8.61E-03	4.10E-03	6.99E-03	4.10E-03	NA	NA	NA	MODIFIER	NA	NA	111	111	0	114	57	57	50
Non-user	IOB_24	chr2	232602682	C	T	PDE6D	INTRONIC	ENSG00000156973:[ENST00000428104(c.208+41G>A)]	NA	NA	6.59E-03	1.35E-02	5.17E-03	6.59E-03	4.10E-03	NA	NA	NA	MODIFIER	NA	NA	302	301	0	257	118	139	54.09
Non-user	IOB_24	chr2	233245182	C	G	AC068134.8	NCT	ENSG00000224516:[ENST00000439072(n.157+409G>C)]	NA	chr2*233245182*233245182*COSM1530948*C*G*1*lung*1	NA	2.47E-05	1.82E-04	NA	NA	NA	NA	NA	MODIFIER	NA	NA	157	157	0	108	68	39	36.11

Patel *et al.* , 2021. Whole-exome sequencing analysis of oral squamous cell carcinoma delineated by tobacco usage habits
Supplementary Table 4. List of somatic single nucleotide variants identified using whole exome sequencing of OSCC patients

Cohort	Sample	Chromosome	Genomic position	Reference allele	Alternate allele	Genesymbol	Variant type	Variant description	OncoMD	COSMIC	dbSNP	ExAC	ExAC_SAS	1000G	1000G_SAS	SIFT	Polyphen2	CONDEL	VEP	LRT	PhastCon7Way	Total reads in Normal	Reference read support in Normal	Alternative read support in Normal	Total reads in Tumor	Reference read support in Tumor	Alternate read support in Tumor	Alternate allele frequency in Tumor
Non-user	IOB_24	chr2	233245182	C	G	ALPP	MISSENSE	ENSG00000163283:[ENST00000392027(c.845C>G,cCg>cGg):ENSP00000375881(p.Pro282Arg)]	NA	chr2*233245182*233245182*COSM1530948*C*G*1*lung*1	NA	2.47E-05	1.82E-04	NA	NA	NA	NA	NA	MODIFIER	NA	NA	157	157	0	108	68	39	36.11
Non-user	IOB_24	chr2	233245210	C	T	AC068134.8	NCT	ENSG00000224516:[ENST00000439072(n.157+381G>A)]	NA	NA	NA	2.06E-01	1.79E-01	NA	NA	NA	NA	NA	MODIFIER	NA	NA	162	161	0	134	86	48	35.82
Non-user	IOB_24	chr2	233245210	C	T	ALPP	INTRONIC-SS-DNR-PRX	ENSG00000163283:[ENST00000392027(c.865+8C>T)]	NA	NA	NA	2.06E-01	1.79E-01	NA	NA	NA	NA	NA	MODIFIER	NA	NA	162	161	0	134	86	48	35.82
Non-user	IOB_24	chr2	233245532	C	A	AC068134.8	NCT	ENSG00000224516:[ENST00000441266(n.357+59G>T)]	NA	NA	NA	1.07E-01	1.06E-01	NA	NA	NA	NA	NA	MODIFIER	NA	NA	46	46	0	42	32	10	23.81
Non-user	IOB_24	chr2	233245532	C	A	ALPP	INTRONIC	250:[XM_005246439.1(c.1046-18C>A)]	NA	NA	NA	1.07E-01	1.06E-01	NA	NA	NA	NA	NA	MODIFIER	NA	NA	46	46	0	42	32	10	23.81
Non-user	IOB_24	chr2	233273107	A	G	ALPPL2	MISSENSE	ENSG00000163286:[ENST00000295453(c.779A>G,cAc>cGc):ENSP00000295453(p.His260Arg)]	NA	NA	chr2*233273100*233273116*GCGAAGCACCAGGTGAT*G*rs772357135*NA*NA*NA|chr2*233273102*233273126*GAAGCACCAGGTGATGGGGGCTGGT*G*rs747520874*NA*NA*NA|chr2*233273107*233273107*A*C,G*rs1130346*NA*NA*NA	1.66E-01	1.32E-01	NA	NA	T	BN	NA	MODERATE	N	0	119	118	0	49	35	14	28.57
Non-user	IOB_24	chr2	233346188	G	A	ECEL1	INTRONIC	ENSG00000171551:[ENST00000411860(c.234+28C>T)]	NA	NA	NA	5.95E-04	2.38E-03	NA	NA	NA	NA	NA	MODIFIER	NA	NA	174	174	0	125	73	52	41.6
Non-user	IOB_24	chr2	233386152	C	A	PRSS56	INTRONIC	ENSG00000237412:[ENST00000449534(c.205+14C>A)]	NA	NA	1.90E-02	2.66E-02	3.66E-02	1.90E-02	3.27E-02	NA	NA	NA	MODIFIER	NA	NA	180	178	0	140	92	47	33.57
Non-user	IOB_24	chr2	233390807	C	T	CHRND	5UTR	ENSG00000135902:[ENST00000441621(c.-119C>T)]	NA	NA	3.93E-02	NA	NA	3.93E-02	3.07E-02	NA	NA	NA	MODIFIER	NA	NA	55	55	0	43	20	23	53.49
Non-user	IOB_24	chr2	233406178	G	A	CHRNG	MISSENSE	ENSG00000196811:[ENST00000389494(c.445G>A,Gcc>Acc):ENSP00000374145(p.Ala149Thr)]	NA	NA	4.65E-02	3.20E-02	2.81E-02	4.65E-02	3.07E-02	NA	NA	NA	MODIFIER	NA	NA	186	186	0	137	63	73	53.28
Non-user	IOB_24	chr2	234102542	C	A	INPP5D	MISSENSE	ENSG00000168918:[ENST00000415617(c.1394C>A,aCa>aAa):ENSP00000397421(p.Thr465Lys)]	NA	NA	NA	NA	NA	NA	NA	D	PoD	NA	MODERATE	D	0.622	161	161	0	98	92	6	6.12
Non-user	IOB_24	chr2	234255145	A	C	SAG	NCT	ENSG00000130561:[ENST00000471884(n.3143+87A>C)]	NA	NA	NA	NA	NA	NA	NA	NA	NA	NA	MODIFIER	NA	NA	97	97	0	78	37	40	51.28
Non-user	IOB_24	chr2	234713681	G	T	MROH2A	MISSENSE	ENSG00000185038:[ENST00000389758(c.1976G>T,gGg>gTg):ENSP00000374408(p.Gly659Val)]	NA	NA	1.74E-02	2.55E-02	1.28E-02	1.74E-02	1.02E-02	D	PoD	NA	MODERATE	NA	1	63	63	0	67	35	30	44.78
Non-user	IOB_24	chr2	234716027	T	A	MROH2A	INTRONIC	339766:[NM_001287395.1(c.2062-42T>A)]	NA	NA	2.48E-02	3.58E-02	1.74E-02	2.48E-02	1.53E-02	NA	NA	NA	MODIFIER	NA	NA	47	47	0	52	19	33	63.46
Non-user	IOB_24	chr2	234717790	C	T	MROH2A	MISSENSE	ENSG00000185038:[ENST00000389758(c.2314C>T,Cgg>Tgg):ENSP00000374408(p.Arg772Trp)]	NA	NA	3.99E-03	7.79E-03	1.59E-02	3.99E-03	1.53E-02	D	BN	NA	MODERATE	D	0.999	199	197	1	201	114	87	43.28
Non-user	IOB_24	chr2	234758539	T	C	HJURP	INTRONIC	ENSG00000123485:[ENST00000411486(c.241-34A>G)]	NA	NA	4.61E-02	7.71E-02	7.89E-02	4.61E-02	7.98E-02	NA	NA	NA	MODIFIER	NA	NA	86	84	0	56	17	39	69.64
Non-user	IOB_24	chr2	234854540	G	C	TRPM8	MISSENSE	ENSG00000144481:[ENST00000324695(c.740G>C,aGa>aCa):ENSP00000323926(p.Arg247Thr)]	NA	NA	4.63E-02	4.70E-02	4.19E-02	4.63E-02	3.89E-02	NA	NA	NA	MODIFIER	NA	NA	115	114	0	85	49	34	40
Non-user	IOB_24	chr2	234854552	A	G	TRPM8	MISSENSE	ENSG00000144481:[ENST00000324695(c.752A>G,tAt>tGt):ENSP00000323926(p.Tyr251Cys)]	NA	NA	4.81E-02	4.78E-02	4.19E-02	4.81E-02	3.89E-02	NA	NA	NA	MODIFIER	NA	NA	121	120	0	86	49	35	40.7
Non-user	IOB_24	chr2	234855943	C	G	AC005538.5	NCT	ENSG00000237581:[ENST00000455991(n.401-960G>C)]	NA	NA	NA	NA	NA	NA	NA	NA	NA	NA	MODIFIER	NA	NA	338	337	0	270	135	134	49.63
Non-user	IOB_24	chr2	234855943	C	G	TRPM8	INTRONIC	ENSG00000144481:[ENST00000444298(c.943-27C>G)]	NA	NA	NA	NA	NA	NA	NA	NA	NA	NA	MODIFIER	NA	NA	338	337	0	270	135	134	49.63
Non-user	IOB_24	chr2	236685678	G	C	AC064874.1	MISSENSE	ENSG00000222007:[ENST00000440101(c.82C>G,Cct>Gct):ENSP00000392481(p.Pro28Ala)]	NA	NA	2.52E-02	4.94E-02	4.97E-02	2.52E-02	4.81E-02	NA	NA	NA	MODIFIER	NA	NA	120	120	0	76	47	29	38.16
Non-user	IOB_24	chr2	236685678	G	C	AGAP1	INTRONIC	116987:[XM_005246064.1(c.374-20725G>C)]	NA	NA	2.52E-02	4.94E-02	4.97E-02	2.52E-02	4.81E-02	NA	NA	NA	MODIFIER	NA	NA	120	120	0	76	47	29	38.16
Non-user	IOB_24	chr2	238277379	C	T	COL6A3	MISSENSE	ENSG00000163359:[ENST00000472056(c.2906G>A,cGg>cAg):ENSP00000418285(p.Arg969Gln)]	NA	NA	3.79E-03	1.03E-02	5.39E-03	3.79E-03	5.10E-03	NA	BN	NA	MODERATE	N	0.013	488	487	0	331	183	146	44.11
Non-user	IOB_24	chr2	238742701	A	G	RBM44	MISSENSE	ENSG00000177483:[ENST00000316997(c.2951A>G,cAa>cGa):ENSP00000321179(p.Gln984Arg)]	NA	NA	NA	3.95E-04	9.15E-04	NA	NA	T	BN	NA	MODERATE	NA	NA	57	57	0	72	38	34	47.22
Non-user	IOB_24	chr2	238820363	G	A	RAMP1	MISSENSE	ENSG00000132329:[ENST00000254661(c.385G>A,Gtg>Atg):ENSP00000254661(p.Val129Met)]	NA	NA	2.00E-04	5.99E-04	3.50E-03	2.00E-04	1.00E-03	T	PoD	NA	MODERATE	N	1	276	276	0	166	95	70	42.17
Non-user	IOB_24	chr2	239229440	A	G	TRAF3IP1	MISSENSE	ENSG00000204104:[ENST00000409739(c.137A>G,gAc>gGc):ENSP00000386648(p.Asp46Gly)]	NA	NA	4.19E-02	5.11E-02	4.61E-02	4.19E-02	3.99E-02	NA	NA	NA	MODIFIER	NA	NA	94	89	1	77	45	31	40.26
Non-user	IOB_24	chr2	239253266	C	A	TRAF3IP1	INTRONIC	26146:[XM_005246070.1(c.992-2830C>A)]	NA	NA	4.01E-02	6.93E-02	4.59E-02	4.01E-02	3.99E-02	NA	NA	NA	MODIFIER	NA	NA	44	44	0	42	21	18	42.86
Non-user	IOB_24	chr2	239344553	C	T	ASB1	SILENT	ENSG00000065802:[ENST00000264607(c.393C>T,ctC>ctT):ENSP00000264607(p.Leu131(=))]	NA	NA	2.00E-04	2.07E-04	1.82E-04	2.00E-04	1.00E-03	NA	NA	NA	MODIFIER	NA	NA	328	326	0	248	119	129	52.02
Non-user	IOB_24	chr2	240834669	G	A	NA	EXONIC-NC	101927147:[XR_241441.1(n.236C>T)]	NA	NA	3.81E-02	4.17E-02	3.26E-02	3.81E-02	3.58E-02	NA	NA	NA	LOW	NA	NA	251	249	0	157	87	70	44.59
Non-user	IOB_24	chr2	240834669	G	A	NDUFA10	SILENT	ENSG00000130414:[ENST00000419408(c.357C>T,ttC>ttT):ENSP00000408055(p.Phe119(=))]	NA	NA	3.81E-02	4.17E-02	3.26E-02	3.81E-02	3.58E-02	NA	NA	NA	LOW	NA	NA	251	249	0	157	87	70	44.59
Non-user	IOB_24	chr2	240834768	A	G	NA	NCT	101927147:[XR_241441.1(n.174-37T>C)]	NA	NA	2.40E-02	3.28E-02	2.88E-02	2.40E-02	3.37E-02	NA	NA	NA	MODIFIER	NA	NA	138	138	0	90	52	38	42.22
Non-user	IOB_24	chr2	240834768	A	G	NDUFA10	NCT	4705:[NR_136158.1(n.4410-37T>C)]	NA	NA	2.40E-02	3.28E-02	2.88E-02	2.40E-02	3.37E-02	NA	NA	NA	MODIFIER	NA	NA	138	138	0	90	52	38	42.22
Non-user	IOB_24	chr2	240950984	G	A	NDUFA10	INTRONIC	ENSG00000130414:[ENST00000448880(c.38+50C>T)]	NA	NA	NA	4.15E-05	1.83E-04	NA	NA	NA	NA	NA	MODIFIER	NA	NA	86	86	0	63	27	35	55.56
Non-user	IOB_24	chr2	240985347	G	A	OR6B3	MISSENSE	ENSG00000178586:[ENST00000319423(c.143C>T,aCc>aTc):ENSP00000322435(p.Thr48Ile)]	NA	NA	NA	4.44E-01	4.70E-01	NA	NA	T	PoD	NA	MODERATE	U	0.444	198	197	1	99	72	26	26.26
Non-user	IOB_24	chr2	241451471	G	A	ANKMY1	INTRONIC	51281:[XM_005247020.1(c.1390-53C>T)]	NA	NA	5.59E-03	NA	NA	5.59E-03	1.53E-02	NA	NA	NA	MODIFIER	NA	NA	128	127	0	98	64	34	34.69
Non-user	IOB_24	chr2	241511812	G	A	RNPEPL1	INTRONIC	57140:[NM_018226.4(c.529-28G>A)]	NA	NA	1.80E-03	5.87E-03	9.60E-03	1.80E-03	9.20E-03	NA	NA	NA	MODIFIER	NA	NA	181	178	0	165	81	84	50.91
Non-user	IOB_24	chr2	241700676	G	A	KIF1A	SILENT	ENSG00000130294:[ENST00000320389(c.2208C>T,gcC>gcT):ENSP00000322791(p.Ala736(=))]	NA	NA	1.90E-02	3.81E-02	1.55E-02	1.90E-02	9.20E-03	NA	NA	NA	LOW	NA	NA	120	120	0	79	36	43	54.43
Non-user	IOB_24	chr2	241722031	A	G	KIF1A	INTRONIC-SS-ACR-PRX	ENSG00000130294:[ENST00000428768(c.287-7T>C)]	NA	NA	NA	NA	NA	NA	NA	NA	NA	NA	LOW	NA	NA	110	109	1	82	45	37	45.12
Non-user	IOB_24	chr2	241903943	G	A	AC104809.3	SILENT	ENSG00000226321:[ENST00000442811(c.343G>A,gcG>gcA):ENSP00000397844(p.Ala115(=))]	NA	NA	1.68E-02	1.88E-02	7.70E-03	1.68E-02	8.20E-03	NA	NA	NA	MODIFIER	NA	NA	41	41	0	30	17	12	40
Non-user	IOB_24	chr2	241903943	G	A	AC104809.4	NCT	ENSG00000233392:[ENST00000418218(n.126-350C>T)]	NA	NA	1.68E-02	1.88E-02	7.70E-03	1.68E-02	8.20E-03	NA	NA	NA	MODIFIER	NA	NA	41	41	0	30	17	12	40
Non-user	IOB_24	chr2	241903943	G	A	LOC728763	SILENT	728763:[XM_003118518.2(c.2547G>A,gcG>gcA):XP_003118566.2(p.Ala849(=))]	NA	NA	1.68E-02	1.88E-02	7.70E-03	1.68E-02	8.20E-03	NA	NA	NA	MODIFIER	NA	NA	41	41	0	30	17	12	40
Non-user	IOB_24	chr2	241907552	G	A	AC104809.3	MISSENSE	ENSG00000226321:[ENST00000443866(c.41G>A,cGc>cAc):ENSP00000397968(p.Arg14His)]	NA	NA	1.60E-03	2.45E-02	3.34E-02	1.60E-03	7.20E-03	NA	NA	NA	MODERATE	NA	NA	105	104	0	79	51	28	35.44
Non-user	IOB_24	chr2	241907552	G	A	LOC101927470	MISSENSE	101927470:[XM_005247054.1(c.56G>A,cGc>cAc):XP_005247111.1(p.Arg19His)]	NA	NA	1.60E-03	2.45E-02	3.34E-02	1.60E-03	7.20E-03	NA	NA	NA	MODERATE	NA	NA	105	104	0	79	51	28	35.44
Non-user	IOB_24	chr2	242021011	A	G	MTERF4	NCT	130916:[NR_138463.1(n.3170-5914T>C)]	NA	NA	NA	NA	NA	NA	NA	NA	NA	NA	MODIFIER	NA	NA	178	177	0	142	61	77	54.23
Non-user	IOB_24	chr2	242021011	A	G	MTERFD2	NCT	ENSG00000122085:[ENST00000496878(n.1641-5914T>C)]	NA	NA	NA	NA	NA	NA	NA	NA	NA	NA	MODIFIER	NA	NA	178	177	0	142	61	77	54.23
Non-user	IOB_24	chr2	242021011	A	G	SNED1	INTRONIC	ENSG00000162804:[ENST00000342631(c.3818-81A>G)]	NA	NA	NA	NA	NA	NA	NA	NA	NA	NA	MODIFIER	NA	NA	178	177	0	142	61	77	54.23
Non-user	IOB_24	chr2	242716380	A	T	GAL3ST2	MISSENSE	ENSG00000154252:[ENST00000192314(c.10A>T,Atg>Ttg):ENSP00000192314(p.Met4Leu)]	NA	NA	NA	3.46E-01	3.44E-01	3.70E-01	2.71E-01	T_lc	BN	NA	MODERATE	U	0	278	275	1	247	133	113	45.75
Non-user	IOB_24	chr2	242758179	C	T	NEU4	SILENT	ENSG00000204099:[ENST00000325935(c.1299C>T,tcC>tcT):ENSP00000320318(p.Ser433(=))]	NA	NA	NA	1.71E-01	1.60E-01	2.16E-01	1.66E-01	NA	NA	NA	MODIFIER	NA	NA	179	179	0	198	88	108	54.55
Non-user	IOB_24	chr2	242758203	T	C	NEU4	SILENT	ENSG00000204099:[ENST00000325935(c.1323T>C,gcT>gcC):ENSP00000320318(p.Ala441(=))]	NA	chr2*242758203*242758203*COSM442650*T*C*1*breast*1	NA	4.13E-01	5.13E-01	4.45E-01	5.46E-01	NA	NA	NA	LOW	NA	NA	198	196	1	200	90	107	53.5
Non-user	IOB_24	chr2	242758326	G	A	NEU4	SILENT	ENSG00000204099:[ENST00000404257(c.1443G>A,aaG>aaA):ENSP00000385149(p.Lys481(=))]	NA	chr2*242758326*242758326*COSM1565630*G*A*1*large_intestine*1	NA	4.27E-01	5.34E-01	4.44E-01	5.41E-01	NA	NA	NA	LOW	NA	NA	146	144	1	108	59	47	43.52
Non-user	IOB_24	chr20	138165	C	T	DEFB127	5UTR	ENSG00000088782:[ENST00000382388(c.-21C>T)]	NA	NA	4.19E-03	8.72E-03	9.97E-03	4.19E-03	8.20E-03	NA	NA	NA	MODIFIER	NA	NA	58	57	0	42	23	19	45.24
Non-user	IOB_24	chr20	333970	T	C	NRSN2	SILENT	ENSG00000125841:[ENST00000382285(c.306T>C,gaT>gaC):ENSP00000371722(p.Asp102(=))]	NA	NA	5.99E-04	6.76E-04	4.97E-03	5.99E-04	3.10E-03	NA	NA	NA	MODIFIER	NA	NA	176	174	1	123	69	54	43.9
Non-user	IOB_24	chr20	2779096	T	C	CPXM1	MISSENSE	ENSG00000088882:[ENST00000380605(c.428A>G,cAc>cGc):ENSP00000369979(p.His143Arg)]	NA	NA	NA	NA	NA	NA	NA	D	PrD	NA	MODERATE	D	1	211	211	0	155	81	74	47.74
Non-user	IOB_24	chr20	3128403	T	C	FASTKD5	SILENT	ENSG00000215251:[ENST00000380266(c.1314A>G,ggA>ggG):ENSP00000369618(p.Gly438(=))]	NA	NA	2.42E-02	1.43E-02	5.77E-02	2.42E-02	6.34E-02	NA	NA	NA	MODIFIER	NA	NA	303	302	0	223	116	106	47.53
Non-user	IOB_24	chr20	3128403	T	C	UBOX5	INTRONIC	ENSG00000185019:[ENST00000449731(c.-42+8927A>G)]	NA	NA	2.42E-02	1.43E-02	5.77E-02	2.42E-02	6.34E-02	NA	NA	NA	MODIFIER	NA	NA	303	302	0	223	116	106	47.53
Non-user	IOB_24	chr20	3128403	T	C	UBOX5-AS1	NCT	ENSG00000235958:[ENST00000446537(n.1463-2781T>C)]	NA	NA	2.42E-02	1.43E-02	5.77E-02	2.42E-02	6.34E-02	NA	NA	NA	MODIFIER	NA	NA	303	302	0	223	116	106	47.53
Non-user	IOB_24	chr20	3210301	G	A	SLC4A11	MISSENSE	ENSG00000088836:[ENST00000474451(c.1484C>T,aCg>aTg):ENSP00000476859(p.Thr495Met)]	NA	NA	4.85E-02	9.16E-02	2.91E-02	4.85E-02	1.84E-02	NA	NA	NA	MODIFIER	NA	NA	104	104	0	74	28	45	60.81
Non-user	IOB_24	chr20	3211235	C	T	SLC4A11	SILENT	ENSG00000088836:[ENST00000474451(c.1284G>A,acG>acA):ENSP00000476859(p.Thr428(=))]	NA	NA	4.51E-02	8.51E-02	2.27E-02	4.51E-02	1.43E-02	NA	NA	NA	LOW	NA	NA	102	101	1	86	47	39	45.35
Non-user	IOB_24	chr20	3211304	G	A	SLC4A11	INTRONIC	83959:[XM_005260856.1(c.1652-11C>T)]	NA	NA	4.79E-02	9.66E-02	3.06E-02	4.79E-02	1.84E-02	NA	NA	NA	MODIFIER	NA	NA	149	149	0	124	66	58	46.77
Non-user	IOB_24	chr20	4837878	G	A	SLC23A2	INTRONIC	ENSG00000089057:[ENST00000424750(c.1379-28C>T)]	NA	NA	NA	NA	NA	NA	NA	NA	NA	NA	MODIFIER	NA	NA	132	129	1	134	121	9	6.72
Non-user	IOB_24	chr20	10036202	G	A	ANKEF1	MISSENSE	ENSG00000132623:[ENST00000378380(c.2225G>A,cGg>cAg):ENSP00000367631(p.Arg742Gln)]	NA	NA	4.91E-02	9.02E-02	4.21E-02	4.91E-02	3.58E-02	T	BN	NA	MODERATE	N	0.043	205	205	0	138	73	63	45.65
Non-user	IOB_24	chr20	10036202	G	A	SNAP25-AS1	NCT	100131208:[NR_040710.1(n.500-28906C>T)]	NA	NA	4.91E-02	9.02E-02	4.21E-02	4.91E-02	3.58E-02	T	BN	NA	MODERATE	N	0.043	205	205	0	138	73	63	45.65
Non-user	IOB_24	chr20	16025372	G	A	MACROD2	INTRONIC	ENSG00000172264:[ENST00000407045(c.253+88G>A)]	NA	NA	7.99E-04	NA	NA	7.99E-04	4.10E-03	NA	NA	NA	MODIFIER	NA	NA	212	211	0	134	66	68	50.75
Non-user	IOB_24	chr20	17968871	C	T	MGME1	MISSENSE	ENSG00000125871:[ENST00000377710(c.794C>T,aCa>aTa):ENSP00000366939(p.Thr265Ile)]	NA	NA	4.39E-03	7.88E-03	1.22E-03	4.39E-03	2.00E-03	NA	NA	NA	MODIFIER	NA	NA	286	285	0	160	95	64	40
Non-user	IOB_24	chr20	17968871	C	T	OVOL2	NCT	ENSG00000125850:[ENST00000486776(n.491+8334G>A)]	NA	NA	4.39E-03	7.88E-03	1.22E-03	4.39E-03	2.00E-03	NA	NA	NA	MODIFIER	NA	NA	286	285	0	160	95	64	40
Non-user	IOB_24	chr20	18568697	C	G	DTD1	EXONIC-NC	ENSG00000125821:[ENST00000494921(n.90C>G)]	NA	NA	9.98E-03	2.43E-02	2.76E-02	9.98E-03	2.56E-02	NA	NA	NA	MODIFIER	NA	NA	45	45	0	31	19	12	38.71
Non-user	IOB_24	chr20	19665973	A	G	SLC24A3	MISSENSE	ENSG00000185052:[ENST00000328041(c.1292A>G,gAt>gGt):ENSP00000333519(p.Asp431Gly)]	NA	NA	NA	NA	NA	NA	NA	T	BN	NA	MODERATE	N	0.909	34	34	0	39	24	15	38.46
Non-user	IOB_24	chr20	20504966	T	C	RALGAPA2	INTRONIC	57186:[XM_005260769.1(c.3414+84A>G)]	NA	NA	4.23E-02	NA	NA	4.23E-02	5.73E-02	NA	NA	NA	MODIFIER	NA	NA	39	39	0	41	18	23	56.1
Non-user	IOB_24	chr20	25273133	C	T	PYGB	SILENT	ENSG00000100994:[ENST00000428458(c.317C>T,gcC>gcT):ENSP00000395451(p.Ala106(=))]	NA	NA	3.99E-04	1.49E-04	1.09E-03	3.99E-04	2.00E-03	NA	NA	NA	LOW	NA	NA	293	291	1	254	131	119	46.85
Non-user	IOB_24	chr20	26061845	T	A	FAM182A	EXONIC-NC	ENSG00000125804:[ENST00000415411(n.464T>A)]	NA	NA	NA	1.27E-02	1.38E-03	NA	NA	NA	NA	NA	MODIFIER	NA	NA	106	94	8	37	24	12	32.43
Non-user	IOB_24	chr20	30102474	G	A	HM13	SILENT	ENSG00000101294:[ENST00000335574(c.120G>A,ctG>ctA):ENSP00000335294(p.Leu40(=))]	NA	NA	NA	6.32E-04	1.48E-03	NA	NA	NA	NA	NA	MODIFIER	NA	NA	290	290	0	257	131	126	49.03
Non-user	IOB_24	chr20	30460793	C	T	TTLL9	INTRONIC	164395:[XM_005260311.1(c.-164+1795C>T)]	NA	NA	1.38E-02	2.94E-02	2.57E-02	1.38E-02	1.43E-02	NA	NA	NA	MODIFIER	NA	NA	72	71	0	55	25	29	52.73
Non-user	IOB_24	chr20	31383299	A	G	DNMT3B	MISSENSE	ENSG00000088305:[ENST00000456297(c.923A>G,tAt>tGt):ENSP00000412305(p.Tyr308Cys)]	NA	NA	1.60E-02	8.89E-03	6.34E-02	1.60E-02	8.18E-02	D_lc	UN	NA	MODERATE	NA	0.052	115	115	0	79	35	43	54.43
Non-user	IOB_24	chr20	31496030	C	A	LOC101928239	INTRONIC	101928239:[XM_005260630.1(c.220-51C>A)]	NA	NA	NA	NA	NA	NA	NA	NA	NA	NA	MODIFIER	NA	NA	130	130	0	96	52	44	45.83
Non-user	IOB_24	chr20	31643355	T	C	BPIFB3	INTRONIC-SS-DNR	ENSG00000186190:[ENST00000375494(c.124+2T>C)]	NA	NA	7.99E-04	1.09E-03	5.45E-04	7.99E-04	1.00E-03	NA	NA	NA	HIGH	NA	NA	213	212	1	166	88	78	46.99
Non-user	IOB_24	chr20	31652409	C	T	BPIFB3	INTRONIC	ENSG00000186190:[ENST00000375494(c.744+55C>T)]	NA	NA	1.14E-02	NA	NA	1.14E-02	8.20E-03	NA	NA	NA	MODIFIER	NA	NA	174	174	0	160	81	77	48.12
Non-user	IOB_24	chr20	31671213	A	C	BPIFB4	SILENT	ENSG00000186191:[ENST00000375483(c.210A>C,ggA>ggC):ENSP00000364632(p.Gly70(=))]	NA	chr20*31671213*31671213*COSM112099*A*AC*7*ovary,large_intestine,breast*1,5,1|chr20*31671213*31671214*COSM1411168*AC*A*8*pancreas,large_intestine*2,6	NA	8.28E-06	6.12E-05	NA	NA	NA	NA	NA	LOW	NA	NA	129	125	0	103	39	63	61.17
Non-user	IOB_24	chr20	31889153	G	A	BPIFB1	MISSENSE	ENSG00000125999:[ENST00000253354(c.862G>A,Gtg>Atg):ENSP00000253354(p.Val288Met)]	NA	NA	3.99E-04	1.24E-04	8.48E-04	3.99E-04	2.00E-03	D	PoD	NA	MODERATE	N	0.285	68	68	0	29	19	10	34.48

Patel *et al.* , 2021. Whole-exome sequencing analysis of oral squamous cell carcinoma delineated by tobacco usage habits
Supplementary Table 4. List of somatic single nucleotide variants identified using whole exome sequencing of OSCC patients

Cohort	Sample	Chromosome	Genomic position	Reference allele	Alternate allele	Genesymbol	Variant type	Variant description	OncoMD	COSMIC	dbSNP	ExAC	ExAC_SAS	1000G	1000G_SAS	SIFT	Polyphen2	CONDEL	VEP	LRT	PhastCon7Way	Total reads in Normal	Reference read support in Normal	Alternative read support in Normal	Total reads in Tumor	Reference read support in Tumor	Alternate read support in Tumor	Alternate allele frequency in Tumor
Non-user	IOB_24	chr20	33114201	A	G	DYNLRB1	INTRONIC	ENSG00000125971:[ENST00000300469(c.79+53A>G)]	NA	NA	3.57E-02	NA	NA	3.57E-02	1.02E-02	NA	NA	NA	MODIFIER	NA	NA	111	111	0	48	28	20	41.67
Non-user	IOB_24	chr20	33326680	T	C	NCOA6	INTRONIC	23054:[NM_001242539.2(c.2915-2118A>G)]	NA	NA	1.36E-02	8.46E-03	4.80E-03	1.36E-02	1.00E-03	NA	NA	NA	MODIFIER	NA	NA	120	117	0	92	38	54	58.7
Non-user	IOB_24	chr20	33568502	C	T	MYH7B	MISSENSE	ENSG00000078814:[ENST00000262873(c.590C>T,gCg>gTg):ENSP00000262873(p.Ala197Val)]	NA	NA	NA	NA	NA	NA	NA	NA	NA	NA	MODERATE	NA	1	131	131	0	131	126	5	3.82
Non-user	IOB_24	chr20	33576021	G	A	MYH7B	MISSENSE	ENSG00000078814:[ENST00000262873(c.1669G>A,Gac>Aac):ENSP00000262873(p.Asp557Asn)]	NA	NA	7.99E-04	3.55E-04	1.89E-03	7.99E-04	1.00E-03	NA	NA	NA	MODERATE	NA	1	135	135	0	103	45	58	56.31
Non-user	IOB_24	chr20	33581009	A	G	MYH7B	MISSENSE	ENSG00000078814:[ENST00000262873(c.2414A>G,tAc>tGc):ENSP00000262873(p.Tyr805Cys)]	NA	NA	NA	NA	NA	NA	NA	NA	NA	NA	MODERATE	NA	1	152	152	0	107	58	49	45.79
Non-user	IOB_24	chr20	33587565	A	C	MYH7B	INTRONIC	ENSG00000078814:[ENST00000262873(c.4792-29A>C)]	NA	NA	NA	1.25E-01	2.48E-01	NA	NA	NA	NA	NA	MODIFIER	NA	NA	45	38	1	34	9	9	26.47
Non-user	IOB_24	chr20	33645273	G	A	TRPC4AP	INTRONIC	ENSG00000100991:[ENST00000432634(c.355+44C>T)]	NA	NA	1.30E-02	3.16E-02	3.30E-03	1.30E-02	1.00E-03	NA	NA	NA	MODIFIER	NA	NA	47	47	0	49	26	23	46.94
Non-user	IOB_24	chr20	33962010	G	C	UQCC	INTRONIC	55245:[XM_005260443.1(c.5-7593C>G)]	NA	NA	NA	NA	NA	NA	NA	NA	NA	NA	MODIFIER	NA	NA	105	105	0	66	44	22	33.33
Non-user	IOB_24	chr20	33962010	G	C	UQCC1	MISSENSE	ENSG00000101019:[ENST00000374384(c.383C>G,aCt>aGt):ENSP00000363505(p.Thr128Ser)]	NA	NA	NA	NA	NA	NA	NA	NA	NA	NA	MODIFIER	NA	NA	105	105	0	66	44	22	33.33
Non-user	IOB_24	chr20	34205901	G	A	SPAG4	INTRONIC	6676:[XM_005260521.1(c.409+739G>A)]	NA	NA	4.15E-02	NA	NA	4.15E-02	5.62E-02	NA	NA	NA	MODIFIER	NA	NA	136	136	0	78	48	30	38.46
Non-user	IOB_24	chr20	37146142	T	A	RALGAPB	NCT-SS-PRX	ENSG00000170471:[ENST00000461423(n.304-7T>A)]	NA	NA	NA	8.64E-06	7.61E-05	NA	NA	NA	NA	NA	LOW	NA	NA	89	89	0	57	35	21	36.84
Non-user	IOB_24	chr20	37257590	C	G	ARHGAP40	SILENT	ENSG00000124143:[ENST00000243967(c.241C>G,ggC>ggG):ENSP00000243967(p.Gly81(=))]	NA	NA	NA	3.09E-01	2.85E-01	NA	NA	NA	NA	NA	LOW	NA	NA	89	87	0	47	0	47	100
Non-user	IOB_24	chr20	37536611	G	C	PPP1R16B	INTRONIC	ENSG00000101445:[ENST00000373331(c.902+33G>C)]	NA	NA	3.53E-02	2.55E-02	4.09E-02	3.53E-02	5.93E-02	NA	NA	NA	MODIFIER	NA	NA	184	184	0	133	77	56	42.11
Non-user	IOB_24	chr20	39657625	C	A	TOP1	5UTR	ENSG00000198900:[ENST00000361337(c.-83C>A)]	NA	NA	4.19E-02	NA	NA	4.19E-02	1.06E-01	NA	NA	NA	MODIFIER	NA	NA	116	116	0	105	50	55	52.38
Non-user	IOB_24	chr20	42343821	C	G	MYBL2	MISSENSE	ENSG00000101057:[ENST00000217026(c.1872C>G,atC>atG):ENSP00000217026(p.Ile624Met)]	NA	NA	2.32E-02	4.28E-02	3.38E-02	2.32E-02	3.37E-02	T	PoD	NA	MODERATE	N	0.05	151	151	0	112	58	53	47.32
Non-user	IOB_24	chr20	42743603	G	A	JPH2	INTRONIC	ENSG00000149596:[ENST00000372980(c.2011-87C>T)]	NA	NA	9.98E-04	NA	NA	9.98E-04	5.10E-03	NA	NA	NA	MODIFIER	NA	NA	149	149	0	142	71	71	50
Non-user	IOB_24	chr20	43042287	T	C	HNF4A	INTRONIC	ENSG00000101076:[ENST00000316673(c.320-47T>C)]	NA	NA	1.22E-02	4.20E-03	1.52E-02	1.22E-02	2.56E-02	NA	NA	NA	MODIFIER	NA	NA	102	95	2	75	32	36	48
Non-user	IOB_24	chr20	43042300	T	C	HNF4A	INTRONIC	ENSG00000101076:[ENST00000316673(c.320-34T>C)]	NA	NA	1.22E-02	4.59E-03	1.66E-02	1.22E-02	2.56E-02	NA	NA	NA	MODIFIER	NA	NA	123	117	0	88	41	39	44.32
Non-user	IOB_24	chr20	43042407	T	C	HNF4A	SILENT	ENSG00000101076:[ENST00000316673(c.393T>C,aaT>aaC):ENSP00000315180(p.Asn131(=))]	NA	NA	1.22E-02	4.24E-03	1.69E-02	1.22E-02	2.56E-02	NA	NA	NA	LOW	NA	0	116	116	0	94	45	49	52.13
Non-user	IOB_24	chr20	43043118	C	T	HNF4A	INTRONIC	ENSG00000101076:[ENST00000372920(c.*260-29C>T)]	NA	NA	5.99E-03	2.27E-03	1.54E-02	5.99E-03	2.56E-02	NA	NA	NA	MODIFIER	NA	NA	132	131	0	90	51	35	38.89
Non-user	IOB_24	chr20	43043127	C	T	HNF4A	INTRONIC	ENSG00000101076:[ENST00000372920(c.*260-20C>T)]	NA	NA	5.99E-03	2.27E-03	1.54E-02	5.99E-03	2.56E-02	NA	NA	NA	MODIFIER	NA	NA	140	139	0	104	60	41	39.42
Non-user	IOB_24	chr20	43043143	G	A	HNF4A	INTRONIC	3172:[XM_005260407.1(c.610-4G>A)]	NA	NA	6.39E-03	2.34E-03	1.54E-02	6.39E-03	2.56E-02	NA	NA	NA	LOW	NA	NA	186	185	0	123	69	50	40.65
Non-user	IOB_24	chr20	43043159	G	A	HNF4A	MISSENSE	ENSG00000101076:[ENST00000316099(c.505G>A,Gtc>Atc):ENSP00000312987(p.Val169Ile)]	NA	NA	5.99E-03	2.34E-03	1.53E-02	5.99E-03	2.56E-02	T	BN	NA	MODERATE	N	0.857	221	220	0	143	79	60	41.96
Non-user	IOB_24	chr20	43561763	A	T	PABPC1L	MISSENSE	ENSG00000101104:[ENST00000217073(c.1381A>T,Atc>Ttc):ENSP00000217073(p.Ile461Phe)]	NA	NA	NA	NA	NA	NA	NA	T	PoD	NA	MODERATE	NA	NA	172	170	0	159	89	69	43.4
Non-user	IOB_24	chr20	43964539	C	T	SDC4	MISSENSE	ENSG00000124145:[ENST00000372733(c.82G>A,Gac>Aac):ENSP00000361818(p.Asp28Asn)]	NA	NA	2.00E-03	8.55E-04	5.48E-03	2.00E-03	8.20E-03	T	BN	NA	MODERATE	N	0.481	63	63	0	72	35	36	50
Non-user	IOB_24	chr20	44354312	C	T	SPINT4	3UTR	ENSG00000149651:[ENST00000279058(c.*38C>T)]	NA	NA	4.75E-02	3.25E-02	6.25E-02	4.75E-02	5.11E-02	NA	NA	NA	MODIFIER	NA	NA	122	122	0	72	45	27	37.5
Non-user	IOB_24	chr20	44507108	G	A	ZSWIM3	SILENT	ENSG00000132801:[ENST00000454862(c.1893G>A,gaG>gaA):ENSP00000406313(p.Glu631(=))]	NA	NA	2.60E-03	1.35E-03	9.63E-03	2.60E-03	1.33E-02	NA	NA	NA	LOW	NA	1	183	182	1	194	93	101	52.06
Non-user	IOB_24	chr20	44534909	G	A	PLTP	MISSENSE-SS-PRX	ENSG00000100979:[ENST00000372420(c.439C>T,Cgg>Tgg):ENSP00000361497(p.Arg147Trp)]	NA	NA	NA	NA	NA	NA	NA	D	PrD	NA	MODERATE	D	1	102	102	0	69	39	30	43.48
Non-user	IOB_24	chr20	47244417	C	T	PREX1	SILENT	ENSG00000124126:[ENST00000371941(c.4851G>A,acG>acA):ENSP00000361009(p.Thr1617(=))]	NA	NA	1.24E-02	5.15E-03	1.25E-02	1.24E-02	2.15E-02	NA	NA	NA	LOW	NA	NA	82	82	0	68	35	33	48.53
Non-user	IOB_24	chr20	47256335	G	A	PREX1	SILENT	ENSG00000124126:[ENST00000371941(c.3873C>T,acC>acT):ENSP00000361009(p.Thr1291(=))]	NA	chr20*47256335*47256335*COSM149189*G*A*1*stomach*1	2.26E-02	1.02E-02	2.26E-02	2.26E-02	2.86E-02	NA	NA	NA	LOW	NA	NA	79	79	0	61	25	36	59.02
Non-user	IOB_24	chr20	47258761	G	A	PREX1	SILENT	ENSG00000124126:[ENST00000371941(c.3720C>T,gtC>gtT):ENSP00000361009(p.Val1240(=))]	NA	chr20*47258761*47258761*COSM149190*G*A*1*stomach*1	2.42E-02	1.08E-02	2.30E-02	2.42E-02	2.86E-02	NA	NA	NA	LOW	NA	NA	281	281	0	285	149	135	47.37
Non-user	IOB_24	chr20	47268030	G	A	PREX1	SILENT	ENSG00000124126:[ENST00000482556(c.524C>T,caC>caT):ENSP00000434632(p.His175(=))]	NA	NA	8.79E-03	2.94E-03	1.18E-02	8.79E-03	2.25E-02	NA	NA	NA	LOW	NA	0	114	113	0	97	55	42	43.3
Non-user	IOB_24	chr20	47317471	C	T	PREX1	INTRONIC	ENSG00000124126:[ENST00000371941(c.784-47G>A)]	NA	NA	1.80E-03	7.25E-04	5.27E-03	1.80E-03	9.20E-03	NA	NA	NA	MODIFIER	NA	NA	33	33	0	29	11	18	62.07
Non-user	IOB_24	chr20	48807975	C	A	CEBPB	SILENT	ENSG00000172216:[ENST00000303004(c.405C>A,gcC>gcA):ENSP00000305422(p.Ala135(=))]	NA	NA	9.98E-04	1.37E-03	3.52E-03	9.98E-04	5.10E-03	NA	NA	NA	MODIFIER	NA	NA	457	457	0	374	208	165	44.12
Non-user	IOB_24	chr20	48807975	C	A	CEBPB-AS1	NCT	101927559:[NR_125739.1(n.407-16G>T)]	NA	NA	9.98E-04	1.37E-03	3.52E-03	9.98E-04	5.10E-03	NA	NA	NA	MODIFIER	NA	NA	457	457	0	374	208	165	44.12
Non-user	IOB_24	chr20	50713883	C	G	ZFP64	NCT	ENSG00000020256:[ENST00000477786(n.801+29G>C)]	NA	NA	3.00E-03	1.64E-03	1.21E-02	3.00E-03	1.53E-02	NA	NA	NA	MODIFIER	NA	NA	186	184	0	136	65	71	52.21
Non-user	IOB_24	chr20	55071953	A	G	GCNT7	SILENT	ENSG00000124091:[ENST00000243913(c.346T>C,Ttg>Ctg):ENSP00000243913(p.Leu116(=))]	NA	NA	4.59E-03	4.50E-03	1.23E-02	4.59E-03	2.15E-02	NA	NA	NA	MODIFIER	NA	NA	175	174	0	139	75	63	45.32
Non-user	IOB_24	chr20	55071953	A	G	RTFDC1	INTRONIC	ENSG00000022277:[ENST00000357348(c.567+12708A>G)]	NA	NA	4.59E-03	4.50E-03	1.23E-02	4.59E-03	2.15E-02	NA	NA	NA	MODIFIER	NA	NA	175	174	0	139	75	63	45.32
Non-user	IOB_24	chr20	56826020	A	G	PPP4R1L	NCT	ENSG00000124224:[ENST00000495058(n.309-12T>C)]	NA	NA	4.29E-02	7.93E-02	8.64E-02	4.29E-02	1.00E-01	NA	NA	NA	MODIFIER	NA	NA	73	73	0	49	29	20	40.82
Non-user	IOB_24	chr20	57292763	A	T	NPEPL1	EXONIC-NC	ENSG00000215440:[ENST00000532531(n.344A>T)]	NA	NA	2.60E-03	NA	NA	2.60E-03	1.00E-03	NA	NA	NA	MODIFIER	NA	NA	121	121	0	78	43	35	44.87
Non-user	IOB_24	chr20	57766296	C	T	ZNF831	SILENT	ENSG00000124203:[ENST00000371030(c.222C>T,cgC>cgT):ENSP00000360069(p.Arg74(=))]	NA	chr20*57766296*57766296*COSM478344*C*T*2*endometrium,kidney*1,1	NA	4.08E-05	0.00E+00	NA	NA	NA	NA	NA	LOW	NA	NA	312	311	0	259	136	122	47.1
Non-user	IOB_24	chr20	58452566	C	T	SYCP2	SILENT	ENSG00000196074:[ENST00000357552(c.3024G>A,ccG>ccA):ENSP00000350162(p.Pro1008(=))]	NA	NA	4.59E-02	1.92E-02	3.00E-02	4.59E-02	1.74E-02	NA	NA	NA	LOW	NA	NA	48	48	0	40	24	16	40
Non-user	IOB_24	chr20	58455447	A	G	SYCP2	MISSENSE	ENSG00000196074:[ENST00000357552(c.2852T>C,aTt>aCt):ENSP00000350162(p.Ile951Thr)]	NA	NA	2.80E-03	7.47E-03	2.42E-02	2.80E-03	1.12E-02	NA	NA	NA	MODERATE	NA	0.986	50	50	0	28	10	18	64.29
Non-user	IOB_24	chr20	58488979	T	C	SYCP2	NCT	ENSG00000196074:[ENST00000471331(n.456+51A>G)]	NA	NA	3.40E-03	4.73E-03	1.91E-02	3.39E-03	1.12E-02	NA	NA	NA	MODIFIER	NA	NA	20	20	0	19	12	7	36.84
Non-user	IOB_24	chr20	58533713	C	T	CDH26	5UTR	ENSG00000124215:[ENST00000244047(c.-69C>T)]	NA	NA	1.28E-02	NA	NA	1.28E-02	8.20E-03	NA	NA	NA	MODIFIER	NA	NA	89	89	0	68	44	24	35.29
Non-user	IOB_24	chr20	58569563	T	C	CDH26	NCT	ENSG00000124215:[ENST00000497614(n.282+19T>C)]	NA	NA	8.59E-03	1.76E-02	2.49E-02	8.59E-03	1.74E-02	NA	NA	NA	MODIFIER	NA	NA	126	122	1	108	38	68	62.96
Non-user	IOB_24	chr20	58571864	A	G	CDH26	INTRONIC	ENSG00000124215:[ENST00000370991(c.794+48A>G)]	NA	NA	8.59E-03	1.73E-02	2.46E-02	8.59E-03	1.74E-02	NA	NA	NA	MODIFIER	NA	NA	177	177	0	111	55	54	48.65
Non-user	IOB_24	chr20	58576432	A	G	CDH26	MISSENSE	ENSG00000124215:[ENST00000348616(c.2132A>G,cAg>cGg):ENSP00000339390(p.Gln711Arg)]	NA	NA	8.59E-03	1.73E-02	2.45E-02	8.59E-03	1.74E-02	NA	NA	NA	MODIFIER	NA	NA	110	110	0	56	31	25	44.64
Non-user	IOB_24	chr20	58577957	T	C	CDH26	NCT	60437:[XR_244144.1(n.2652+36T>C)]	NA	NA	8.79E-03	1.78E-02	2.47E-02	8.79E-03	1.74E-02	NA	NA	NA	MODIFIER	NA	NA	47	47	0	29	15	14	48.28
Non-user	IOB_24	chr20	60318875	G	T	CDH4	INTRONIC	1002:[NM_001252338.2(c.285+30G>T)]	NA	NA	1.40E-03	3.43E-03	1.01E-02	1.40E-03	7.20E-03	NA	NA	NA	MODIFIER	NA	NA	131	131	0	97	64	33	34.02
Non-user	IOB_24	chr20	60318875	G	T	RP11-429E11.2	NCT	ENSG00000225106:[ENST00000442888(n.35-2932C>A)]	NA	NA	1.40E-03	3.43E-03	1.01E-02	1.40E-03	7.20E-03	NA	NA	NA	MODIFIER	NA	NA	131	131	0	97	64	33	34.02
Non-user	IOB_24	chr20	60738620	C	T	SS18L1	SILENT	ENSG00000184402:[ENST00000492466(c.107C>T,agC>agT):ENSP00000434451(p.Ser36(=))]	NA	NA	1.26E-02	3.05E-02	7.78E-03	1.26E-02	5.10E-03	NA	NA	NA	LOW	NA	0.981	100	97	0	87	40	47	54.02
Non-user	IOB_24	chr20	60866735	C	G	OSBPL2	INTRONIC	9885:[XM_005260625.1(c.611-24C>G)]	NA	NA	9.39E-03	2.44E-02	8.12E-03	9.38E-03	7.20E-03	NA	NA	NA	MODIFIER	NA	NA	88	88	0	95	52	43	45.26
Non-user	IOB_24	chr20	60988492	G	A	RBBP8NL	MISSENSE	ENSG00000130701:[ENST00000252998(c.1640C>T,cCc>cTc):ENSP00000252998(p.Pro547Leu)]	NA	NA	1.80E-02	1.59E-02	4.42E-02	1.80E-02	3.58E-02	D	BN	NA	MODERATE	U	0	30	30	0	37	19	17	45.95
Non-user	IOB_24	chr20	60989187	G	T	RBBP8NL	MISSENSE	ENSG00000130701:[ENST00000252998(c.1220C>A,gCc>gAc):ENSP00000252998(p.Ala407Asp)]	NA	NA	2.60E-03	1.78E-02	1.47E-03	2.60E-03	0.00E+00	T	BN	NA	MODERATE	N	0	176	176	0	119	51	67	56.3
Non-user	IOB_24	chr20	61303264	C	T	SLCO4A1	NCT	ENSG00000101187:[ENST00000495889(n.392-544C>T)]	NA	NA	2.74E-02	5.23E-02	5.19E-02	2.74E-02	4.40E-02	NA	NA	NA	MODIFIER	NA	NA	564	562	0	406	234	172	42.36
Non-user	IOB_24	chr20	61459319	C	T	COL9A3	SILENT	ENSG00000092758:[ENST00000343916(c.891C>T,agC>agT):ENSP00000341640(p.Ser297(=))]	NA	NA	1.20E-03	1.53E-03	3.21E-03	1.20E-03	4.10E-03	NA	NA	NA	MODIFIER	NA	NA	198	198	0	145	66	78	53.79
Non-user	IOB_24	chr20	61870656	C	T	BIRC7	INTRONIC	ENSG00000101197:[ENST00000217169(c.650-54C>T)]	NA	NA	1.20E-03	2.32E-03	9.13E-03	1.20E-03	5.10E-03	NA	NA	NA	MODIFIER	NA	NA	103	102	0	71	35	36	50.7
Non-user	IOB_24	chr20	61917442	A	G	ARFGAP1	SILENT	ENSG00000101199:[ENST00000370275(c.945A>G,gaA>gaG):ENSP00000359298(p.Glu315(=))]	NA	NA	2.20E-03	5.14E-03	8.63E-03	2.20E-03	5.10E-03	NA	NA	NA	MODIFIER	NA	NA	335	332	1	246	111	133	54.07
Non-user	IOB_24	chr20	62051100	C	T	KCNQ2	INTRONIC	ENSG00000075043:[ENST00000359125(c.1248-75G>A)]	NA	NA	2.60E-03	NA	NA	2.60E-03	1.33E-02	NA	NA	NA	MODIFIER	NA	NA	83	82	0	67	31	36	53.73
Non-user	IOB_24	chr20	62073861	G	A	KCNQ2	SILENT	ENSG00000075043:[ENST00000344425(c.714C>T,atC>atT):ENSP00000345523(p.Ile238(=))]	NA	chr20*62073861*62073861*COSM1483841*G*T*1*breast*1|chr20*62073861*62073861*COSM444215*G*T*1*breast*1	4.13E-02	2.10E-02	1.51E-01	4.13E-02	2.09E-01	NA	NA	NA	LOW	NA	0.336	161	159	0	144	85	59	40.97
Non-user	IOB_24	chr20	62195923	G	A	HELZ2	MISSENSE	ENSG00000130589:[ENST00000467148(c.4252C>T,Ctc>Ttc):ENSP00000417401(p.Leu1418Phe)]	NA	NA	2.00E-03	6.15E-03	1.61E-02	2.00E-03	1.02E-02	D	PrD	NA	MODERATE	D	1	140	138	0	121	58	63	52.07
Non-user	IOB_24	chr20	62197004	T	C	HELZ2	SILENT	ENSG00000130589:[ENST00000467148(c.3171A>G,gcA>gcG):ENSP00000417401(p.Ala1057(=))]	NA	NA	2.00E-03	2.11E-03	1.47E-02	2.00E-03	1.02E-02	NA	NA	NA	LOW	NA	NA	430	429	0	315	171	143	45.4
Non-user	IOB_24	chr21	9825790	C	T	NA	INTERGENIC	NA	NA	NA	chr21*9825790*9825790*C*G*rs770213425*NA*NA*NA	NA	NA	NA	NA	NA	NA	NA	MODIFIER	NA	NA	453	445	0	347	314	24	6.92
Non-user	IOB_24	chr21	9825793	C	T	NA	INTERGENIC	NA	NA	NA	NA	1.28E-03	0.00E+00	NA	NA	NA	NA	NA	MODIFIER	NA	NA	462	454	0	351	323	20	5.7
Non-user	IOB_24	chr21	9826040	G	A	NA	INTERGENIC	NA	NA	NA	NA	NA	NA	NA	NA	NA	NA	NA	MODIFIER	NA	NA	12627	12603	4	10890	9835	1030	9.46
Non-user	IOB_24	chr21	9826061	C	T	NA	INTERGENIC	NA	NA	NA	NA	NA	NA	NA	NA	NA	NA	NA	MODIFIER	NA	NA	12360	12325	6	10728	10164	537	5.01
Non-user	IOB_24	chr21	9826251	C	T	MIR3687	MATURE-MIRNA	ENSG00000264063:[ENST00000577708(n.49C>T)]	NA	NA	NA	3.78E-03	8.37E-03	NA	NA	NA	NA	NA	MODIFIER	NA	NA	8950	8930	1	8350	7776	540	6.47
Non-user	IOB_24	chr21	9907419	A	G	TEKT4P2	EXONIC-NC	ENSG00000188681:[ENST00000416067(n.1374T>C)]	NA	NA	NA	1.30E-03	2.42E-03	NA	NA	NA	NA	NA	MODIFIER	NA	NA	662	558	0	624	448	163	26.12
Non-user	IOB_24	chr21	9907422	G	A	TEKT4P2	EXONIC-NC	100132288:[NR_038327.1(n.1379C>T)]	NA	NA	NA	4.83E-03	2.57E-03	NA	NA	NA	NA	NA	MODIFIER	NA	NA	646	548	0	616	437	165	26.79
Non-user	IOB_24	chr21	9908380	G	A	TEKT4P2	EXONIC-NC	ENSG00000188681:[ENST00000559466(n.994C>T)]	NA	NA	NA	NA	NA	NA	NA	NA	NA	NA	MODIFIER	NA	NA	900	897	0	812	727	81	9.98
Non-user	IOB_24	chr21	9909079	C	T	TEKT4P2	EXONIC-NC	100132288:[NR_038328.2(n.337G>A)]	NA	NA	NA	NA	NA	NA	NA	NA	NA	NA	MODIFIER	NA	NA	171	170	0	126	103	19	15.08
Non-user	IOB_24	chr21	9909080	G	A	TEKT4P2	EXONIC-NC	ENSG00000188681:[ENST00000416067(n.327C>T)]	NA	NA	NA	NA	NA	NA	NA	NA	NA	NA	MODIFIER	NA	NA	175	175	0	127	105	19	14.96
Non-user	IOB_24	chr21	9909089	T	C	TEKT4P2	EXONIC-NC	100132288:[NR_038328.1(n.326A>G)]	NA	NA	NA	NA	NA	NA	NA	NA	NA	NA	MODIFIER	NA	NA	172	172	0	132	109	19	14.39
Non-user	IOB_24	chr21	9909122	G	A	TEKT4P2	EXONIC-NC	ENSG00000188681:[ENST00000400754(n.248C>T)]	NA	NA	NA	NA	NA	NA	NA	NA	NA	NA	MODIFIER	NA	NA	203	202	0	173	155	17	9.83
Non-user	IOB_24	chr21	9909149	G	A	TEKT4P2	EXONIC-NC	ENSG00000188681:[ENST00000416067(n.258C>T)]	NA	NA	NA	NA	NA	NA	NA	NA	NA	NA	MODIFIER	NA	NA	210	209	0	184	163	20	10.87
Non-user	IOB_24	chr21	9909281	G	A	TEKT4P2	NCT-SS-PRX	ENSG00000188681:[ENST00000416067(n.130-4C>T)]	NA	NA	NA	NA	NA	NA	NA	NA	NA	NA	LOW	NA	NA	96	96	0	94	83	11	11.7
Non-user	IOB_24	chr21	10862598	C	T	NA	INTERGENIC	NA	NA	NA	3.28E-02	6.27E-02	1.34E-01	3.27E-02	6.03E-02	NA	NA	NA	MODIFIER	NA	NA	389	386	0	240	199	41	17.08
Non-user	IOB_24	chr21	11038924	T	G	BAGE2	EXONIC-NC	ENSG00000187172:[ENST00000496773(n.974A>C)]	NA	NA	NA	NA	NA	NA	NA	NA	NA	NA	MODIFIER	NA	NA	895	890	0	551	470	78	14.16
Non-user	IOB_24	chr21	11038924	T	G	LOC101926954	SILENT	101926954:[XM_005269267.1(c.513A>C,gcA>gcC):XP_005269324.1(p.Ala171(=))]	NA	NA	NA	NA	NA	NA	NA	NA	NA	NA	MODIFIER	NA	NA	895	890	0	551	470	78	14.16
Non-user	IOB_24	chr21	17250749	C	T	USP25	SILENT	ENSG00000155313:[ENST00000400183(c.3333C>T,gcC>gcT):ENSP00000383044(p.Ala1111(=))]	NA	NA	7.99E-04	1.64E-03	1.94E-03	7.99E-04	2.00E-03	NA	NA	NA	LOW	NA	NA	433	431	1	299	154	144	48.16
Non-user	IOB_24	chr21	28338039	C	T	ADAMTS5	SILENT	ENSG00000154736:[ENST00000284987(c.672G>A,gcG>gcA):ENSP00000284987(p.Ala224(=))]	NA	NA	1.60E-03	1.81E-03	8.43E-03	1.60E-03	8.20E-03	NA	NA	NA	LOW	NA	NA	150	150	0	132	52	80	60.61
Non-user	IOB_24	chr21	30391523	G	A	RWDD2B	INTRONIC	10069:[NM_001320724.1(c.-119+9C>T)]	NA	NA	1.72E-02	2.09E-02	3.99E-04	1.72E-02	2.00E-03	NA	NA	NA	MODIFIER	NA	NA	25	25	0	17	7	10	58.82
Non-user	IOB_24	chr21	31986097	G	A	KRTAP6-1	MISSENSE	ENSG00000184724:[ENST00000329122(c.127C>T,Cgc>Tgc):ENSP00000332690(p.Arg43Cys)]	NA	NA	4.03E-02	4.86E-02	1.05E-01	4.03E-02	1.06E-01	NA	PoD	NA	MODERATE	NA	NA	216	216	0	151	67	84	55.63
Non-user	IOB_24	chr21	32931595	G	A	AP000251.3	EXONIC-NC	ENSG00000237594:[ENST00000433071(n.38G>A)]	NA	NA	1.14E-02	8.67E+00	NA	1.14E-02	3.48E-02	NA	NA	NA	MODERATE	NA	NA	106	106	0	83	32	51	61.45
Non-user	IOB_24	chr21	32931595	G	A	LOC150051	MISSENSE	150051:[NM_001321023.1(c.22G>A,Gcg>Acg):NP_001307952.1(p.Ala8Thr)]	NA	NA	1.14E-02	8.67E+00	NA	1.14E-02	3.48E-02	NA	NA	NA	MODERATE	NA	NA	106	106	0	83	32	51	61.45
Non-user	IOB_24	chr21	32931595	G	A	TIAM1	NCT	ENSG00000156299:[ENST00000469412(n.59+637C>T)]	NA	NA	1.14E-02	8.67E+00	NA	1.14E-02	3.48E-02	NA	NA	NA	MODERATE	NA	NA	106	106	0	83	32	51	61.45

Patel *et al.*, 2021. Whole-exome sequencing analysis of oral squamous cell carcinoma delineated by tobacco usage habits
Supplementary Table 4. List of somatic single nucleotide variants identified using whole exome sequencing of OSCC patients

Cohort	Sample	Chromosome	Genomic position	Reference allele	Alternate allele	Genesymbol	Variant type	Variant description	OncoMD	COSMIC	dbSNP	ExAC	ExAC_SAS	1000G	1000G_SAS	SIFT	Polyphen2	CONDEL	VEP	LRT	PhastCon7Way	Total reads in Normal	Reference read support in Normal	Alternative read support in Normal	Total reads in Tumor	Reference read support in Tumor	Alternate read support in Tumor	Alternate allele frequency in Tumor
Non-user	IOB_24	chr21	33840166	T	G	EVA1C	NCT	59271:[XR_244287.1(n.1762+10T>G)]	NA	NA	5.59E-03	1.45E-02	4.59E-03	5.59E-03	1.00E-03	NA	NA	NA	MODIFIER	NA	NA	77	77	0	57	34	22	38.6
Non-user	IOB_24	chr21	33949156	C	T	TCP10L	SILENT	ENSG00000242220:[ENST00000472557(c.318G>A,agG>agA):ENSP00000476481(p.Arg106(=))]	NA	NA	NA	2.47E-05	1.82E-04	NA	NA	NA	NA	NA	LOW	NA	NA	110	110	0	61	35	26	42.62
Non-user	IOB_24	chr21	34717726	C	T	IFNAR1	INTRONIC	ENSG00000142166:[ENST00000270139(c.788+60C>T)]	NA	NA	1.14E-02	NA	NA	1.14E-02	2.15E-02	NA	NA	NA	MODIFIER	NA	NA	256	255	0	143	67	76	53.15
Non-user	IOB_24	chr21	35757915	A	G	SMIM11	MISSENSE	ENSG00000205670:[ENST00000399292(c.152A>G,aAg>aGg):ENSP00000382231(p.Lys51Arg)]	NA	NA	1.62E-02	2.39E-02	3.76E-02	1.62E-02	3.58E-02	NA	NA	NA	MODIFIER	NA	NA	63	62	0	31	19	11	35.48
Non-user	IOB_24	chr21	37510203	G	A	CBR3	MISSENSE	ENSG00000159231:[ENST00000290354(c.370G>A,Gag>Aag):ENSP00000290354(p.Glu124Lys)]	NA	chr21*37510203*37510203*COSM1030479*G*A*1*endometrium*1	3.59E-03	2.52E-03	1.81E-02	3.59E-03	1.64E-02	NA	NA	NA	MODIFIER	NA	NA	311	309	0	217	115	102	47
Non-user	IOB_24	chr21	37510203	G	A	CBR3-AS1	NCT	100506428:[NR_038892.1(n.193-4144C>T)]	NA	chr21*37510203*37510203*COSM1030479*G*A*1*endometrium*1	3.59E-03	2.52E-03	1.81E-02	3.59E-03	1.64E-02	NA	NA	NA	MODIFIER	NA	NA	311	309	0	217	115	102	47
Non-user	IOB_24	chr21	38095355	T	C	SIM2	MISSENSE	ENSG00000159263:[ENST00000431229(c.279T>C,aTa>aCa):ENSP00000392003(p.Ile94Thr)]	NA	NA	5.99E-04	2.72E-04	1.94E-03	5.99E-04	3.10E-03	T	BN	NA	MODERATE	D	1	137	137	0	120	73	46	38.33
Non-user	IOB_24	chr21	38131420	G	A	HLCS	INTRONIC	3141:[XM_005260954.1(c.2236+608C>T)]	NA	NA	5.99E-04	7.87E-04	1.70E-03	5.99E-04	3.10E-03	NA	NA	NA	MODIFIER	NA	NA	92	91	0	84	44	40	47.62
Non-user	IOB_24	chr21	38494273	C	G	TTC3	MISSENSE	ENSG00000182670:[ENST00000540756(c.127C>G,Cta>Gta):ENSP00000442875(p.Leu43Val)]	NA	NA	2.78E-02	5.01E-02	8.50E-02	2.78E-02	5.83E-02	T	BN	NA	MODERATE	NA	NA	41	40	0	21	12	9	42.86
Non-user	IOB_24	chr21	40551786	C	T	PSMG1	INTRONIC	ENSG00000183527:[ENST00000380900(c.393+425G>A)]	NA	NA	3.20E-03	NA	NA	3.19E-03	1.53E-02	NA	NA	NA	MODIFIER	NA	NA	188	188	0	131	73	58	44.27
Non-user	IOB_24	chr21	40582823	C	T	BRWD1	SILENT	ENSG00000185658:[ENST00000333229(c.3933G>A,acG>acA):ENSP00000330753(p.Thr1311(=))]	NA	NA	1.78E-02	3.10E-02	1.04E-02	1.78E-02	4.10E-03	NA	NA	NA	LOW	NA	NA	68	67	0	52	31	21	40.38
Non-user	IOB_24	chr21	40685284	A	G	BRWD1	INTRONIC	ENSG00000185658:[ENST00000333229(c.50-59T>C)]	NA	NA	.	NA	NA	NA	NA	NA	NA	NA	MODIFIER	NA	NA	20	19	0	12	0	8	66.67
Non-user	IOB_24	chr21	41160134	C	G	IGSF5	INTRONIC	ENSG00000183067:[ENST00000380588(c.956+19C>G)]	NA	NA	2.00E-03	5.61E-03	3.39E-03	2.00E-03	3.10E-03	NA	NA	NA	MODIFIER	NA	NA	56	56	0	45	18	27	60
Non-user	IOB_24	chr21	41725475	C	T	DSCAM	MISSENSE	ENSG00000171587:[ENST00000400454(c.851G>A,cGc>cAc):ENSP00000383303(p.Arg284His)]	NA	NA	3.99E-04	1.33E-04	4.24E-04	3.99E-04	0.00E+00	D	PoD	NA	MODERATE	NA	NA	119	119	0	108	54	54	50
Non-user	IOB_24	chr21	42860520	T	C	TMPRSS2	NCT	ENSG00000184012:[ENST00000463138(n.193-80A>G)]	NA	NA	1.16E-02	NA	NA	1.16E-02	5.93E-02	NA	NA	NA	MODIFIER	NA	NA	55	55	0	24	13	10	41.67
Non-user	IOB_24	chr21	43133659	C	T	LINC00479	NCT	ENSG00000236384:[ENST00000586552(n.177-528G>A)]	NA	NA	2.92E-02	NA	NA	2.92E-02	3.89E-02	NA	NA	NA	MODIFIER	NA	NA	25	25	0	13	7	6	46.15
Non-user	IOB_24	chr21	43291618	G	A	AP001619.3	NCT	ENSG00000236545:[ENST00000458654(n.268-226G>A)]	NA	NA	1.32E-02	9.46E-03	2.75E-03	1.32E-02	1.00E-03	NA	NA	NA	MODIFIER	NA	NA	50	50	0	23	9	14	60.87
Non-user	IOB_24	chr21	43291618	G	A	PRDM15	MISSENSE	ENSG00000141956:[ENST00000269844(c.526C>T,Cct>Tct):ENSP00000269844(p.Pro176Ser)]	NA	NA	1.32E-02	9.46E-03	2.75E-03	1.32E-02	1.00E-03	NA	NA	NA	MODIFIER	NA	NA	50	50	0	23	9	14	60.87
Non-user	IOB_24	chr21	44841091	G	A	SIK1	INTRONIC	ENSG00000142178:[ENST00000270162(c.624+32C>T)]	NA	NA	6.99E-03	9.70E-03	7.35E-03	6.99E-03	7.20E-03	NA	NA	NA	MODIFIER	NA	NA	171	171	0	115	57	58	50.43
Non-user	IOB_24	chr21	45210771	C	T	RRP1	NCT	ENSG00000160214:[ENST00000475534(n.184-460C>T)]	NA	NA	1.60E-03	NA	NA	1.60E-03	7.20E-03	NA	NA	NA	MODIFIER	NA	NA	33	33	0	29	17	12	41.38
Non-user	IOB_24	chr21	45741693	G	A	PFKL	MISSENSE	ENSG00000141959:[ENST00000349048(c.1273G>A,Ggc>Agc):ENSP00000269848(p.Gly425Ser)]	NA	NA	NA	6.04E-05	2.97E-04	NA	NA	T	PoD	NA	MODERATE	D	1	313	312	0	286	128	157	54.9
Non-user	IOB_24	chr21	47542421	C	G	COL6A2	SILENT	ENSG00000142173:[ENST00000413758(c.207C>G,ggC>ggG):ENSP00000395751(p.Gly69(=))]	NA	NA	chr21*47542421*47542421*C*A,T*rs566854019*0*0.9998*0.0001997..	NA	NA	NA	NA	NA	NA	NA	LOW	NA	NA	142	141	0	85	54	31	36.47
Non-user	IOB_24	chr21	47552103	G	T	COL6A2	SILENT	ENSG00000142173:[ENST00000300527(c.2697G>T,acG>acT):ENSP00000300527(p.Thr899(=))]	NA	NA	1.70E-02	6.05E-02	6.27E-02	1.70E-02	2.97E-02	NA	NA	NA	LOW	NA	NA	288	287	0	235	109	126	53.62
Non-user	IOB_24	chr21	47566287	C	T	FTCD	NCT	ENSG00000160282:[ENST00000498355(n.976-46G>A)]	NA	NA	6.79E-03	1.17E-02	2.44E-02	6.79E-03	2.25E-02	NA	NA	NA	MODIFIER	NA	NA	103	102	0	65	28	37	56.92
Non-user	IOB_24	chr21	47611903	A	G	LOC101060037	3UTR	101060037:[XM_005261212.1(c.*459T>C)]	NA	NA	NA	3.13E-05	2.18E-04	NA	NA	NA	NA	NA	MODIFIER	NA	NA	199	197	0	162	77	83	51.23
Non-user	IOB_24	chr21	47611903	A	G	LSS	INTRONIC	ENSG00000160285:[ENST00000457828(c.1749-30T>C)]	NA	NA	NA	3.13E-05	2.18E-04	NA	NA	NA	NA	NA	MODIFIER	NA	NA	199	197	0	162	77	83	51.23
Non-user	IOB_24	chr21	47850033	G	A	PCNT	SILENT	ENSG00000160299:[ENST00000359568(c.7800G>A,gcG>gcA):ENSP00000352572(p.Ala2600(=))]	NA	NA	5.79E-03	1.37E-02	3.94E-03	5.79E-03	1.00E-03	NA	NA	NA	LOW	NA	NA	188	187	0	210	116	94	44.76
Non-user	IOB_24	chr21	47855812	A	C	PCNT	NCT-SS-PRX	ENSG00000160299:[ENST00000480896(n.8784-5A>C)]	NA	NA	5.39E-03	7.84E-03	1.82E-02	5.39E-03	1.33E-02	NA	NA	NA	LOW	NA	NA	183	182	0	169	78	91	53.85
Non-user	IOB_24	chr21	48084319	C	T	PRMT2	EXONIC-NC	ENSG00000160310:[ENST00000486520(n.3487C>T)]	NA	NA	NA	2.02E-04	1.23E-04	NA	NA	NA	NA	NA	MODIFIER	NA	NA	141	141	0	123	53	70	56.91
Non-user	IOB_24	chr22	17265194	G	A	XKR3	MISSENSE	ENSG00000172967:[ENST00000331428(c.695C>T,cCg>cTg):ENSP00000331704(p.Pro232Leu)]	NA	NA	NA	NA	NA	NA	NA	T	BN	NA	MODERATE	N	0.051	154	153	0	156	0	156	100
Non-user	IOB_24	chr22	18291801	C	T	MICAL3	INTRONIC	ENSG00000243156:[ENST00000584751(c.35-98G>A)]	NA	NA	7.99E-04	NA	NA	7.99E-04	0.00E+00	NA	NA	NA	MODIFIER	NA	NA	108	106	0	63	39	24	38.1
Non-user	IOB_24	chr22	18291801	C	T	XXbac-B461K10.4	NCT	ENSG00000093100:[ENST00000476405(n.796-98G>A)]	NA	NA	7.99E-04	NA	NA	7.99E-04	0.00E+00	NA	NA	NA	MODIFIER	NA	NA	108	106	0	63	39	24	38.1
Non-user	IOB_24	chr22	18905882	G	T	PRODH	SILENT	ENSG00000100033:[ENST00000334029(c.1050C>A,ggC>ggA):ENSP00000334726(p.Gly350(=))]	NA	NA	3.36E-02	2.61E-02	3.95E-02	3.35E-02	4.81E-02	NA	NA	NA	LOW	NA	NA	43	43	0	36	22	12	33.33
Non-user	IOB_24	chr22	18909954	C	T	PRODH	INTRONIC	5625:[XM_005261255.1(c.277-37G>A)]	NA	NA	7.99E-04	2.05E-03	0.00E+00	7.99E-04	0.00E+00	NA	NA	NA	MODIFIER	NA	NA	59	59	0	74	39	35	47.3
Non-user	IOB_24	chr22	19028643	C	T	DGCR2	MISSENSE	ENSG00000070413:[ENST00000263196(c.1324G>A,Ggc>Agc):ENSP00000263196(p.Gly442Ser)]	NA	NA	4.03E-02	2.92E-02	4.50E-02	4.03E-02	7.57E-02	NA	NA	NA	MODIFIER	NA	NA	289	287	1	283	136	145	51.24
Non-user	IOB_24	chr22	19109597	C	A	AC004471.9	NCT	ENSG00000223461:[ENST00000456035(n.424+132C>A)]	NA	NA	3.99E-02	2.89E-02	4.65E-02	3.99E-02	7.57E-02	NA	NA	NA	MODIFIER	NA	NA	65	65	0	69	31	36	52.17
Non-user	IOB_24	chr22	19109597	C	A	DGCR2	INTRONIC	ENSG00000070413:[ENST00000389262(c.-975+44G>T)]	NA	NA	3.99E-02	2.89E-02	4.65E-02	3.99E-02	7.57E-02	NA	NA	NA	MODIFIER	NA	NA	65	65	0	69	31	36	52.17
Non-user	IOB_24	chr22	19119955	T	G	DGCR14	3UTR	ENSG00000100056:[ENST00000252137(c.*1754A>C)]	NA	NA	3.99E-04	3.57E-04	2.70E-03	3.99E-04	2.00E-03	NA	NA	NA	MODIFIER	NA	NA	315	313	0	264	144	118	44.7
Non-user	IOB_24	chr22	19119955	T	G	TSSK2	MISSENSE	ENSG00000206203:[ENST00000399635(c.1043T>G,aTc>aGc):ENSP00000382544(p.Ile348Ser)]	NA	NA	3.99E-04	3.57E-04	2.70E-03	3.99E-04	2.00E-03	NA	NA	NA	MODIFIER	NA	NA	315	313	0	264	144	118	44.7
Non-user	IOB_24	chr22	19121721	C	T	DGCR14	SILENT	ENSG00000100056:[ENST00000252137(c.1419G>A,tcG>tcA):ENSP00000252137(p.Ser473(=))]	NA	NA	4.05E-02	2.99E-02	5.19E-02	4.05E-02	7.57E-02	NA	NA	NA	LOW	NA	NA	53	53	0	50	30	20	40
Non-user	IOB_24	chr22	19124866	G	A	DGCR14	SILENT	ENSG00000100056:[ENST00000252137(c.1005C>T,taC>taT):ENSP00000252137(p.Tyr335(=))]	NA	NA	4.07E-02	2.95E-02	4.52E-02	4.07E-02	7.57E-02	NA	NA	NA	MODIFIER	NA	NA	70	69	0	40	15	25	62.5
Non-user	IOB_24	chr22	19125015	T	C	DGCR14	INTRONIC	8220:[XM_005261284.1(c.230-70A>G)]	NA	NA	4.05E-02	NA	NA	4.05E-02	7.57E-02	NA	NA	NA	MODIFIER	NA	NA	78	78	0	85	43	42	49.41
Non-user	IOB_24	chr22	19751843	C	T	TBX1	SILENT	ENSG00000184058:[ENST00000329705(c.678C>T,aaC>aaT):ENSP00000331176(p.Asn226(=))]	NA	NA	2.00E-04	3.44E-04	1.15E-03	2.00E-04	0.00E+00	NA	NA	NA	LOW	NA	0.003	399	398	0	292	136	153	52.4
Non-user	IOB_24	chr22	19890449	G	A	TXNRD2	INTRONIC	ENSG00000184470:[ENST00000334363(c.663-3858C>T)]	NA	NA	7.39E-03	NA	NA	7.39E-03	3.17E-02	NA	NA	NA	MODIFIER	NA	NA	97	97	0	96	53	42	43.75
Non-user	IOB_24	chr22	20104123	T	C	RANBP1	INTRONIC	ENSG00000099901:[ENST00000432879(c.246+170T>C)]	NA	NA	NA	NA	NA	NA	NA	NA	NA	NA	MODIFIER	NA	NA	92	90	0	74	36	35	47.3
Non-user	IOB_24	chr22	20104123	T	C	TRMT2A	MISSENSE	ENSG00000099899:[ENST00000252136(c.37A>G,Atg>Gtg):ENSP00000252136(p.Met13Val)]	NA	NA	NA	NA	NA	NA	NA	NA	NA	NA	MODIFIER	NA	NA	92	90	0	74	36	35	47.3
Non-user	IOB_24	chr22	20724386	A	C	USP41	INTRONIC	373856:[XM_036729.11(c.258+24T>G)]	NA	NA	2.26E-02	1.76E-02	5.43E-02	2.26E-02	5.93E-02	NA	NA	NA	MODIFIER	NA	NA	238	237	1	169	80	89	52.66
Non-user	IOB_24	chr22	20729941	A	G	USP41	INTRONIC	ENSG00000161133:[ENST00000454608(c.255-16T>C)]	NA	NA	1.04E-02	5.50E-03	3.81E-02	1.04E-02	4.60E-02	NA	NA	NA	MODIFIER	NA	NA	43	43	0	19	5	14	73.68
Non-user	IOB_24	chr22	20781725	G	A	SCARF2	SILENT	ENSG00000244486:[ENST00000405555(c.1653C>T,gaC>gaT):ENSP00000385589(p.Asp551(=))]	NA	NA	7.99E-03	4.17E-03	1.63E-02	7.99E-03	2.04E-02	NA	NA	NA	MODIFIER	NA	NA	72	72	0	82	48	34	41.46
Non-user	IOB_24	chr22	20940822	C	T	MED15	INTRONIC	ENSG00000099917:[ENST00000382974(c.1898-33C>T)]	NA	NA	1.20E-02	2.06E-02	3.59E-02	1.20E-02	2.76E-02	NA	NA	NA	MODIFIER	NA	NA	503	502	0	445	235	209	46.97
Non-user	IOB_24	chr22	21066789	A	G	PI4KA	SILENT	ENSG00000241973:[ENST00000572273(c.5613T>C,acT>acC):ENSP00000458238(p.Thr1871(=))]	NA	chr22*21066789*21066789*COSM149255*A*G*1*stomach*1	NA	4.09E-01	3.48E-01	NA	NA	NA	NA	NA	MODIFIER	NA	NA	33	33	0	34	26	8	23.53
Non-user	IOB_24	chr22	22312292	A	G	TOP3B	INTRONIC	ENSG00000100038:[ENST00000457179(c.*1944-325T>C)]	NA	NA	NA	3.27E-04	0.00E+00	NA	NA	NA	NA	NA	MODIFIER	NA	NA	201	201	0	80	66	13	16.25
Non-user	IOB_24	chr22	22707493	T	C	IGLV5-48	SILENT	ENSG00000211647:[ENST00000390293(c.81T>C,acT>acC):ENSP00000374828(p.Thr27(=))]	NA	NA	NA	3.21E-01	2.67E-01	NA	NA	NA	NA	NA	LOW	NA	NA	358	357	0	308	261	47	15.26
Non-user	IOB_24	chr22	22707527	C	T	IGLV5-48	MISSENSE	ENSG00000211647:[ENST00000390293(c.115C>T,Ctc>Ttc):ENSP00000374828(p.Leu39Phe)]	NA	NA	chr22*22707527*22707527*C*G,T*rs61739192*NA*NA*NA	3.11E-01	2.32E-01	NA	NA	D	PoD	NA	MODERATE	NA	NA	346	343	0	314	268	46	14.65
Non-user	IOB_24	chr22	22707564	G	C	IGLV5-48	MISSENSE	ENSG00000211647:[ENST00000390293(c.152G>C,aGc>aCc):ENSP00000374828(p.Ser51Thr)]	NA	NA	2.00E-04	7.11E-02	3.40E-02	2.00E-04	0.00E+00	T	BN	NA	MODERATE	NA	NA	279	277	0	233	207	24	10.3
Non-user	IOB_24	chr22	22730619	T	A	IGLV5-45	MISSENSE	ENSG00000211650:[ENST00000390296(c.114T>A,agT>agA):ENSP00000374831(p.Ser38Arg)]	NA	NA	NA	3.21E-01	2.84E-01	NA	NA	T	BN	NA	MODERATE	NA	NA	344	342	0	238	192	41	17.23
Non-user	IOB_24	chr22	22730620	C	T	IGLV5-45	MISSENSE	ENSG00000211650:[ENST00000390296(c.115C>T,Ctc>Ttc):ENSP00000374831(p.Leu39Phe)]	NA	NA	NA	2.90E-01	2.59E-01	NA	NA	D	PoD	NA	MODERATE	NA	NA	345	344	0	239	193	44	18.41
Non-user	IOB_24	chr22	22730696	C	T	IGLV5-45	MISSENSE	ENSG00000211650:[ENST00000390296(c.191C>T,cCt>cTt):ENSP00000374831(p.Pro64Leu)]	NA	NA	NA	3.53E-01	3.09E-01	NA	NA	D	BN	NA	MODERATE	NA	NA	158	157	0	149	105	43	28.86
Non-user	IOB_24	chr22	22730702	A	G	IGLV5-45	MISSENSE	ENSG00000211650:[ENST00000390296(c.197A>G,cAg>cGg):ENSP00000374831(p.Gln66Arg)]	NA	NA	chr22*22730695*22730706*CCTCCCCAGTAT*C*rs762514940*NA*NA*NA|chr22*22730702*22730702*A*C,G*rs61731373*NA*NA*NA	3.74E-01	3.29E-01	NA	NA	T	BN	NA	MODERATE	NA	NA	150	149	0	139	97	41	29.5
Non-user	IOB_24	chr22	22730829	G	A	IGLV5-45	SILENT	ENSG00000211650:[ENST00000390296(c.324G>A,gaG>gaA):ENSP00000374831(p.Glu108(=))]	NA	NA	3.99E-04	5.78E-02	1.59E-01	3.99E-04	0.00E+00	NA	NA	NA	LOW	NA	NA	208	206	0	210	171	31	14.76
Non-user	IOB_24	chr22	22730891	G	C	NA	INTERGENIC	NA	NA	NA	NA	5.93E-02	1.43E-01	NA	NA	NA	NA	NA	MODIFIER	NA	NA	237	236	0	240	193	46	19.17
Non-user	IOB_24	chr22	23237860	A	G	IGLC1	MISSENSE	ENSG00000211675:[ENST00000390321(c.306A>G,Aca>Gca):ENSP00000374855(p.Thr103Ala)]	NA	chr22*23237860*23237860*COSM3693964*A*G*1*large_intestine*1	3.47E-02	2.41E-02	3.44E-02	3.47E-02	3.07E-02	T_lc	BN	NA	MODERATE	NA	NA	57	57	0	52	32	20	38.46
Non-user	IOB_24	chr22	23237860	A	G	IGLL5	MISSENSE	ENSG00000254709:[ENST00000532223(c.634A>G,Aca>Gca):ENSP00000436353(p.Thr212Ala)]	NA	chr22*23237860*23237860*COSM3693964*A*G*1*large_intestine*1	3.47E-02	2.41E-02	3.44E-02	3.47E-02	3.07E-02	T_lc	BN	NA	MODERATE	NA	NA	57	57	0	52	32	20	38.46
Non-user	IOB_24	chr22	23248837	C	A	IGLC3	3UTR	ENSG00000211679:[ENST00000390325(c.*6C>A)]	NA	NA	NA	2.31E-01	2.69E-01	NA	NA	NA	NA	NA	MODIFIER	NA	NA	25	25	0	34	21	12	35.29
Non-user	IOB_24	chr22	23615346	G	A	BCR	INTRONIC	ENSG00000186716:[ENST00000305877(c.1974+26G>A)]	NA	NA	3.63E-02	4.75E-02	3.12E-02	3.63E-02	3.17E-02	NA	NA	NA	MODIFIER	NA	NA	110	108	1	84	59	25	29.76
Non-user	IOB_24	chr22	23968295	G	A	C22orf43	INTRONIC	ENSG00000189269:[ENST00000317749(c.209-60C>T)]	NA	NA	2.00E-04	NA	NA	2.00E-04	1.00E-03	NA	NA	NA	MODIFIER	NA	NA	89	89	0	67	42	25	37.31
Non-user	IOB_24	chr22	24086348	C	T	ZNF70	MISSENSE	ENSG00000187792:[ENST00000341976(c.980G>A,cGc>cAc):ENSP00000339314(p.Arg327His)]	NA	chr22*24086348*24086348*COSM1234499*C*A*1*large_intestine*1	5.99E-04	1.81E-04	0.00E+00	5.99E-04	0.00E+00	D	PoD	NA	MODERATE	NA	NA	231	228	2	164	157	7	4.27
Non-user	IOB_24	chr22	24122750	C	T	MMP11	NCT	4320:[XR_244377.1(n.457-19C>T)]	NA	NA	2.54E-02	1.52E-02	9.11E-02	2.54E-02	9.30E-02	NA	NA	NA	MODIFIER	NA	NA	322	321	0	248	115	130	52.42
Non-user	IOB_24	chr22	24179176	C	G	DERL3	SILENT	91319:[XM_005261821.1(c.777G>C,ctG>ctC):XP_005261878.1(p.Leu259(=))]	NA	NA	2.48E-02	1.60E-02	9.19E-02	2.48E-02	8.90E-02	NA	NA	NA	MODIFIER	NA	NA	67	66	0	56	35	20	35.71
Non-user	IOB_24	chr22	25251500	C	T	SGSM1	NCT	ENSG00000167037:[ENST00000480523(n.763-16C>T)]	NA	NA	2.64E-02	4.95E-02	5.78E-02	2.64E-02	5.83E-02	NA	NA	NA	MODIFIER	NA	NA	191	191	0	130	76	54	41.54
Non-user	IOB_24	chr22	25424579	C	A	KIAA1671	MISSENSE	ENSG00000197077:[ENST00000358431(c.613C>A,Cca>Aca):ENSP00000351207(p.Pro205Thr)]	NA	NA	NA	NA	NA	NA	NA	T	BN	NA	MODERATE	N	0	161	152	1	90	53	34	37.78
Non-user	IOB_24	chr22	25424868	T	G	KIAA1671	MISSENSE	ENSG00000197077:[ENST00000358431(c.902T>G,gTg>gGg):ENSP00000351207(p.Val301Gly)]	NA	NA	NA	NA	NA	NA	NA	T	BN	NA	MODERATE	N	0.001	207	203	2	160	83	76	47.5
Non-user	IOB_24	chr22	25425282	A	G	KIAA1671	MISSENSE	ENSG00000197077:[ENST00000358431(c.1316A>G,aAg>aGg):ENSP00000351207(p.Lys439Arg)]	NA	NA	NA	NA	NA	NA	NA	D	PoD	NA	MODERATE	N	0.983	252	250	0	200	0	200	100
Non-user	IOB_24	chr22	25435028	G	T	KIAA1671	MISSENSE	ENSG00000197077:[ENST00000358431(c.1931G>T,cGt>cTt):ENSP00000351207(p.Arg644Leu)]	NA	NA	NA	NA	NA	NA	NA	T	BN	NA	MODERATE	N	0	119	119	0	52	20	32	61.54
Non-user	IOB_24	chr22	25586744	T	G	KIAA1671	SILENT	ENSG00000197077:[ENST00000358431(c.5418T>G,gtT>gtG):ENSP00000351207(p.Val1806(=))]	NA	NA	3.83E-02	6.74E-02	6.71E-02	3.83E-02	8.90E-02	NA	NA	NA	LOW	NA	NA	101	101	0	85	34	51	60
Non-user	IOB_24	chr22	25756024	C	T	LRP5L	SILENT	ENSG00000100068:[ENST00000402785(c.36G>A,gtG>gtA):ENSP00000384562(p.Val12(=))]	NA	NA	2.42E-02	1.79E-02	6.83E-02	2.42E-02	8.59E-02	NA	NA	NA	MODIFIER	NA	NA	150	150	0	89	0	88	98.88
Non-user	IOB_24	chr22	26118285	A	G	ADRBK2	SILENT	ENSG00000100077:[ENST00000324198(c.1935A>G,aaA>aaG):ENSP00000317578(p.Lys645(=))]	NA	NA	1.60E-03	1.45E-03	1.04E-02	1.60E-03	8.20E-03	NA	NA	NA	LOW	NA	0.012	197	196	0	141	70	71	50.35
Non-user	IOB_24	chr22	26286738	T	A	CTA-125H2.2	NCT	ENSG00000231933:[ENST00000453457(n.959+1456A>T)]	NA	NA	4.11E-02	2.63E-02	3.49E-02	4.11E-02	3.27E-02	T	BN	NA	MODERATE	N	0.005	243	243	0	159	74	84	52.83

Patel *et al.*, 2021. Whole-exome sequencing analysis of oral squamous cell carcinoma delineated by tobacco usage habits
Supplementary Table 4. List of somatic single nucleotide variants identified using whole exome sequencing of OSCC patients

Cohort	Sample	Chromosome	Genomic position	Reference allele	Alternate allele	Genesymbol	Variant type	Variant description	OncoMD	COSMIC	dbSNP	ExAC	ExAC_SAS	1000G	1000G_SAS	SIFT	Polyphen2	CONDEL	VEP	LRT	PhastCon7Way	Total reads in Normal	Reference read support in Normal	Alternative read support in Normal	Total reads in Tumor	Reference read support in Tumor	Alternate read support in Tumor	Alternate allele frequency in Tumor
Non-user	IOB_24	chr22	26286738	T	A	MYO18B	MISSENSE	ENSG00000133454:[ENST00000335473(c.4330T>A,Tct>Act):ENSP00000334563(p.Ser1444Thr)]	NA	NA	4.11E-02	2.63E-02	3.49E-02	4.11E-02	3.27E-02	T	BN	NA	MODERATE	N	0.005	243	243	0	159	74	84	52.83
Non-user	IOB_24	chr22	26859917	G	A	HPS4	MISSENSE	ENSG00000100099:[ENST00000402105(c.1664C>T,cCg>cTg):ENSP00000384185(p.Pro555Leu)]	NA	NA	2.00E-04	4.25E-04	2.37E-03	2.00E-04	1.00E-03	NA	NA	NA	MODIFIER	NA	NA	101	100	0	67	35	31	46.27
Non-user	IOB_24	chr22	26879984	G	A	SRRD	MISSENSE	ENSG00000100104:[ENST00000215917(c.128G>A,gGg>gAg):ENSP00000215917(p.Gly43Glu)]	NA	NA	1.60E-03	NA	NA	1.60E-03	8.20E-03	T	BN	NA	MODERATE	NA	NA	20	20	0	10	3	7	70
Non-user	IOB_24	chr22	26888060	G	A	SRRD	3UTR	ENSG00000100104:[ENST00000215917(c.*422G>A)]	NA	NA	1.80E-02	3.14E-02	2.36E-02	1.80E-02	1.84E-02	NA	NA	NA	MODIFIER	NA	NA	229	229	0	178	93	82	46.07
Non-user	IOB_24	chr22	26888060	G	A	TFIP11	SILENT	ENSG00000100109:[ENST00000405938(c.2433C>T,atC>atT):ENSP00000384297(p.Ile811(=))]	NA	NA	1.80E-02	3.14E-02	2.36E-02	1.80E-02	1.84E-02	NA	NA	NA	MODIFIER	NA	NA	229	229	0	178	93	82	46.07
Non-user	IOB_24	chr22	26894974	G	A	TFIP11	INTRONIC	24144:[NM_001346862.1(c.1237-33C>T)]	NA	NA	3.30E-02	3.60E-02	2.14E-02	3.29E-02	1.94E-02	NA	NA	NA	MODIFIER	NA	NA	108	107	0	138	67	71	51.45
Non-user	IOB_24	chr22	27026286	G	A	CRYBA4	INTRONIC	ENSG00000196431:[ENST00000354760(c.444-18G>A)]	NA	NA	4.81E-02	4.39E-02	5.93E-02	4.81E-02	7.46E-02	NA	NA	NA	MODIFIER	NA	NA	81	81	0	54	37	17	31.48
Non-user	IOB_24	chr22	29602136	C	T	EMID1	INTRONIC-SS-DNR-PRX	ENSG00000186998:[ENST00000334018(c.101+8C>T)]	NA	NA	4.07E-02	1.72E-01	0.00E+00	4.07E-02	4.19E-02	NA	NA	NA	LOW	NA	NA	42	42	0	40	17	22	55
Non-user	IOB_24	chr22	29656251	G	T	RHBDD3	INTRONIC	25807:[XM_005261504.1(c.993-25C>A)]	NA	NA	1.80E-03	7.75E-03	5.88E-03	1.80E-03	2.00E-03	NA	NA	NA	MODIFIER	NA	NA	210	210	0	179	105	74	41.34
Non-user	IOB_24	chr22	29755741	G	A	AP1B1	INTRONIC	ENSG00000100280:[ENST00000317368(c.279+72C>T)]	NA	NA	1.80E-03	NA	NA	1.80E-03	9.20E-03	NA	NA	NA	MODIFIER	NA	NA	61	61	0	63	28	32	50.79
Non-user	IOB_24	chr22	30928620	G	C	SEC14L6	MISSENSE	ENSG00000214491:[ENST00000402034(c.315C>G,agC>agG):ENSP00000385695(p.Ser105Arg)]	NA	NA	4.79E-03	4.99E-03	5.08E-03	4.79E-03	1.12E-02	T	BN	NA	MODERATE	NA	NA	241	241	0	180	105	74	41.11
Non-user	IOB_24	chr22	30977348	G	A	PES1	SILENT	ENSG00000100029:[ENST00000402284(c.744C>T,taC>taT):ENSP00000384252(p.Tyr248(=))]	NA	NA	NA	2.49E-05	0.00E+00	NA	NA	NA	NA	NA	MODIFIER	NA	NA	80	80	0	66	35	31	46.97
Non-user	IOB_24	chr22	31006180	A	G	TCN2	INTRONIC	ENSG00000185339:[ENST00000450638(c.65-42A>G)]	NA	NA	2.00E-03	4.46E-03	8.36E-03	2.00E-03	1.02E-02	NA	NA	NA	MODIFIER	NA	NA	262	260	1	214	115	95	44.39
Non-user	IOB_24	chr22	31090266	T	C	OSBP2	SILENT	ENSG00000184792:[ENST00000407373(c.60T>C,ggT>ggC):ENSP00000385237(p.Gly20(=))]	NA	NA	4.79E-03	4.67E-03	8.90E-03	4.79E-03	2.45E-02	NA	NA	NA	LOW	NA	NA	268	265	1	190	103	86	45.26
Non-user	IOB_24	chr22	31345821	A	C	MORC2	SILENT	ENSG00000133422:[ENST00000397641(c.234T>G,gcT>gcG):ENSP00000380763(p.Ala78(=))]	NA	NA	4.99E-03	8.57E-03	5.45E-04	4.99E-03	0.00E+00	NA	NA	NA	LOW	NA	NA	151	150	0	131	66	65	49.62
Non-user	IOB_24	chr22	31671244	G	A	LIMK2	SILENT	ENSG00000182541:[ENST00000406516(c.1488G>A,ccG>ccA):ENSP00000384602(p.Pro496(=))]	NA	NA	5.99E-04	5.77E-04	3.51E-03	5.99E-04	3.10E-03	NA	NA	NA	LOW	NA	NA	229	229	0	195	97	97	49.74
Non-user	IOB_24	chr22	31844270	G	A	DRG1	INTRONIC	ENSG00000185721:[ENST00000548143(c.165+21102G>A)]	NA	NA	1.82E-02	4.06E-02	3.36E-02	1.82E-02	1.94E-02	NA	NA	NA	MODIFIER	NA	NA	251	250	0	179	88	89	49.72
Non-user	IOB_24	chr22	31844270	G	A	EIF4ENIF1	NCT	ENSG00000184708:[ENST00000487671(n.144-52C>T)]	NA	NA	1.82E-02	4.06E-02	3.36E-02	1.82E-02	1.94E-02	NA	NA	NA	MODIFIER	NA	NA	251	250	0	179	88	89	49.72
Non-user	IOB_24	chr22	32275689	C	A	DEPDC5	MISSENSE	ENSG00000100150:[ENST00000433147(c.2110C>A,agC>agA):ENSP00000410544(p.Ser704Arg)]	NA	NA	chr22*32275689*32275689*C*T*rs769335336*NA*NA*NA	NA	NA	NA	NA	T	PrD	NA	MODERATE	D	1	101	101	0	98	93	5	5.1
Non-user	IOB_24	chr22	32909866	A	G	SYN3	INTRONIC	8224:[NM_133633.2(c.1319-55T>C)]	NA	NA	1.82E-02	NA	NA	1.82E-02	2.76E-02	NA	NA	NA	MODIFIER	NA	NA	97	97	0	59	20	39	66.1
Non-user	IOB_24	chr22	36054944	C	T	APOL6	SILENT	ENSG00000221963:[ENST00000409652(c.333C>T,acC>acT):ENSP00000386280(p.Thr111(=))]	NA	NA	2.12E-02	2.01E-02	2.59E-02	2.12E-02	2.45E-02	NA	NA	NA	LOW	NA	NA	117	116	0	97	46	51	52.58
Non-user	IOB_24	chr22	36055130	T	A	APOL6	MISSENSE	ENSG00000221963:[ENST00000409652(c.519T>A,aaT>aaA):ENSP00000386280(p.Asn173Lys)]	NA	NA	2.40E-02	2.13E-02	2.59E-02	2.40E-02	2.45E-02	T	BN	NA	MODERATE	N	0	157	156	0	123	69	53	43.09
Non-user	IOB_24	chr22	36055406	A	C	APOL6	SILENT	ENSG00000221963:[ENST00000409652(c.795A>C,acA>acC):ENSP00000386280(p.Thr265(=))]	NA	NA	2.20E-02	3.96E-02	4.75E-02	2.20E-02	3.99E-02	NA	NA	NA	LOW	NA	NA	112	111	0	109	47	62	56.88
Non-user	IOB_24	chr22	36634725	A	G	APOL2	INTRONIC	23780:[XM_005261475.1(c.-902+762T>C)]	NA	NA	4.99E-02	NA	NA	4.99E-02	5.93E-02	NA	NA	NA	MODIFIER	NA	NA	170	169	0	136	0	135	99.26
Non-user	IOB_24	chr22	36900812	C	T	FOXRED2	MISSENSE-SS-PRX	ENSG00000100350:[ENST00000216187(c.529G>A,Gtc>Atc):ENSP00000216187(p.Val177Ile)]	NA	NA	1.70E-02	1.56E-02	8.15E-03	1.70E-02	3.10E-03	T	PoD	NA	MODERATE	D	0.937	103	103	0	60	35	25	41.67
Non-user	IOB_24	chr22	37414528	G	A	TST	SILENT	ENSG00000128311:[ENST00000249042(c.246C>T,gcC>gcT):ENSP00000249042(p.Ala82(=))]	NA	NA	2.74E-02	1.59E-02	8.33E-02	2.74E-02	9.71E-02	NA	NA	NA	LOW	NA	NA	105	102	0	98	44	53	54.08
Non-user	IOB_24	chr22	37466992	G	A	TMPRSS6	MISSENSE	ENSG00000187045:[ENST00000346753(c.1663C>T,Ccc>Tcc):ENSP00000334962(p.Pro555Ser)]	NA	NA	2.78E-02	1.40E-02	8.85E-02	2.78E-02	1.15E-01	T	BN	NA	MODERATE	N	0	123	122	1	104	63	41	39.42
Non-user	IOB_24	chr22	38121227	A	G	NOL12	3UTR	ENSG00000100101:[ENST00000455236(c.*3000A>G)]	NA	NA	4.19E-03	1.04E-02	3.64E-03	4.19E-03	2.00E-03	NA	NA	NA	MODIFIER	NA	NA	330	329	1	214	100	113	52.8
Non-user	IOB_24	chr22	38121227	A	G	TRIOBP	SILENT	ENSG00000100106:[ENST00000406386(c.2664A>G,caA>caG):ENSP00000384312(p.Gln888(=))]	NA	NA	4.19E-03	1.04E-02	3.64E-03	4.19E-03	2.00E-03	NA	NA	NA	MODIFIER	NA	NA	330	329	1	214	100	113	52.8
Non-user	IOB_24	chr22	38321806	C	T	MICALL1	SILENT	ENSG00000100139:[ENST00000215957(c.1221C>T,acC>acT):ENSP00000215957(p.Thr407(=))]	NA	NA	5.19E-03	1.62E-02	6.96E-03	5.19E-03	4.10E-03	NA	NA	NA	LOW	NA	NA	49	49	0	35	21	14	40
Non-user	IOB_24	chr22	38328691	G	A	MICALL1	INTRONIC	85377:[XM_005261792.1(c.2137+5G>A)]	NA	NA	3.20E-03	1.41E-03	9.87E-03	3.19E-03	1.64E-02	NA	NA	NA	LOW	NA	NA	107	107	0	95	41	54	56.84
Non-user	IOB_24	chr22	38694062	G	A	CSNK1E	NCT	ENSG00000213923:[ENST00000498529(n.320+729C>T)]	NA	NA	NA	NA	NA	NA	NA	NA	NA	NA	MODIFIER	NA	NA	125	125	0	133	127	6	4.51
Non-user	IOB_24	chr22	39069274	G	A	CBY1	3UTR	ENSG00000100211:[ENST00000216029(c.*33G>A)]	NA	NA	2.00E-04	4.23E-04	2.91E-03	2.00E-04	1.00E-03	NA	NA	NA	MODIFIER	NA	NA	82	82	0	54	30	24	44.44
Non-user	IOB_24	chr22	39069274	G	A	RP3-508I15.9	NCT	ENSG00000228274:[ENST00000431924(n.46-4944C>T)]	NA	NA	2.00E-04	4.23E-04	2.91E-03	2.00E-04	1.00E-03	NA	NA	NA	MODIFIER	NA	NA	82	82	0	54	30	24	44.44
Non-user	IOB_24	chr22	39120291	C	T	GTPBP1	NCT	9567:[XR_244397.1(n.1187-11C>T)]	NA	NA	1.16E-02	2.43E-02	5.26E-02	1.16E-02	3.58E-02	NA	NA	NA	MODIFIER	NA	NA	110	110	0	72	36	36	50
Non-user	IOB_24	chr22	39222771	G	C	NPTXR	INTRONIC	ENSG00000221890:[ENST00000333039(c.851-19C>G)]	NA	NA	1.26E-02	2.36E-02	5.58E-02	1.26E-02	4.29E-02	NA	NA	NA	MODIFIER	NA	NA	115	114	1	73	36	35	47.95
Non-user	IOB_24	chr22	39636859	C	G	PDGFB	INTRONIC-SS-DNR	ENSG00000100311:[ENST00000455790(c.-31+2072G>C)]	NA	NA		6.64E-05	4.86E-04	NA	NA	NA	NA	NA	HIGH	NA	NA	68	68	0	52	26	24	46.15
Non-user	IOB_24	chr22	39967082	G	A	CACNA1I	INTRONIC	ENSG00000100346:[ENST00000336649(c.226-28G>A)]	NA	NA	5.59E-03	1.87E-02	2.92E-02	5.59E-03	1.53E-02	NA	NA	NA	MODIFIER	NA	NA	148	148	0	132	76	56	42.42
Non-user	IOB_24	chr22	40804094	C	T	SGSM3	SILENT	ENSG00000100359:[ENST00000248929(c.1599C>T,ttC>ttT):ENSP00000248929(p.Phe533(=))]	NA	NA	NA	2.07E-04	1.54E-03	NA	NA	NA	NA	NA	LOW	NA	0.071	186	180	0	150	75	69	46
Non-user	IOB_24	chr22	42089919	C	T	C22orf46	SILENT	ENSG00000184208:[ENST00000402966(c.669C>T,acC>acT):ENSP00000385467(p.Thr223(=))]	NA	NA	3.00E-03	4.89E-03	1.12E-02	3.00E-03	1.53E-02	NA	NA	NA	LOW	NA	NA	180	179	0	123	78	45	36.59
Non-user	IOB_24	chr22	42538489	G	A	CYP2D7	EXONIC-NC	1564:[NR_002570.3(n.769C>T)]	NA	NA	1.38E-02	5.06E-02	2.14E-02	1.38E-02	9.20E-03	NA	NA	NA	MODIFIER	NA	NA	13	13	0	27	3	24	88.89
Non-user	IOB_24	chr22	42538489	G	A	CYP2D7P	MISSENSE	ENSG00000205702:[ENST00000358097(c.658C>T,Cct>Tct):ENSP00000445124(p.Pro220Ser)]	NA	NA	1.38E-02	5.06E-02	2.14E-02	1.38E-02	9.20E-03	NA	NA	NA	MODIFIER	NA	NA	13	13	0	27	3	24	88.89
Non-user	IOB_24	chr22	42538489	G	A	CYP2D7P1	EXONIC-NC	1564:[NR_002570.2(n.768C>T)]	NA	NA	1.38E-02	5.06E-02	2.14E-02	1.38E-02	9.20E-03	NA	NA	NA	MODIFIER	NA	NA	13	13	0	27	3	24	88.89
Non-user	IOB_24	chr22	43213724	G	C	ARFGAP3	INTRONIC	ENSG00000242247:[ENST00000437119(c.809+11C>G)]	NA	NA	1.50E-02	2.02E-02	1.80E-02	1.50E-02	2.35E-02	NA	NA	NA	MODIFIER	NA	NA	199	198	1	172	94	78	45.35
Non-user	IOB_24	chr22	43950853	T	G	EFCAB6	MISSENSE	ENSG00000186976:[ENST00000396231(c.3088A>C,Acc>Ccc):ENSP00000379533(p.Thr1030Pro)]	NA	NA	chr22*43950853*43950853*COSM3363688*T*C*1*kidney*1 3.06E-02	4.42E-02	7.19E-02	3.06E-02	7.77E-02	NA	NA	NA	MODERATE	NA	0.22	203	202	0	163	79	84	51.53
Non-user	IOB_24	chr22	44328832	C	T	PNPLA3	SILENT	ENSG00000100344:[ENST00000423180(c.549C>T,ttC>ttT):ENSP00000397987(p.Phe183(=))]	NA	NA	3.49E-02	6.64E-02	5.51E-02	3.49E-02	3.78E-02	NA	NA	NA	MODIFIER	NA	NA	97	97	0	77	32	45	58.44
Non-user	IOB_24	chr22	44360399	T	C	SAMM50	MISSENSE	ENSG00000100347:[ENST00000350028(c.200T>C,aTt>aCt):ENSP00000345445(p.Ile67Thr)]	NA	NA	NA	2.48E-05	6.10E-05	NA	NA	NA	NA	NA	MODIFIER	NA	NA	146	145	0	122	58	64	52.46
Non-user	IOB_24	chr22	44585006	C	T	PARVG	MISSENSE	ENSG00000138964:[ENST00000356909(c.260C>T,gCg>gTg):ENSP00000349378(p.Ala87Val)]	NA	NA	2.60E-03	2.68E-03	1.67E-02	2.60E-03	1.33E-02	NA	NA	NA	MODIFIER	NA	NA	145	144	0	114	64	50	43.86
Non-user	IOB_24	chr22	44967225	C	T	LINC00207	NCT	ENSG00000187012:[ENST00000603530(n.216-31C>T)]	NA	NA	NA	1.81E-04	8.12E-04	NA	NA	NA	NA	NA	MODIFIER	NA	NA	193	193	0	180	98	81	45
Non-user	IOB_24	chr22	44967815	A	T	LINC00207	NCT	388910:[NR_028410.1(n.345-104A>T)]	NA	NA	NA	NA	NA	NA	NA	NA	NA	NA	MODIFIER	NA	NA	88	87	0	80	47	33	41.25
Non-user	IOB_24	chr22	45258284	G	C	ARHGAP8	MISSENSE	ENSG00000241484:[ENST00000389774(c.1204G>C,Gaa>Caa):ENSP00000374424(p.Glu402Gln)]	NA	NA	3.20E-03	2.74E-03	9.22E-03	3.19E-03	1.12E-02	NA	NA	NA	MODIFIER	NA	NA	244	243	0	221	128	93	42.08
Non-user	IOB_24	chr22	45258284	G	C	PRR5-ARHGAP8	MISSENSE	ENSG00000248405:[ENST00000361473(c.1504G>C,Gaa>Caa):ENSP00000354732(p.Glu502Gln)]	NA	NA	3.20E-03	2.74E-03	9.22E-03	3.19E-03	1.12E-02	NA	NA	NA	MODIFIER	NA	NA	244	243	0	221	128	93	42.08
Non-user	IOB_24	chr22	45683248	G	A	UPK3A	MISSENSE	ENSG00000100373:[ENST00000216211(c.404G>A,aGg>aAg):ENSP00000216211(p.Arg135Lys)]	NA	NA	2.00E-04	4.06E-04	3.77E-04	2.00E-04	0.00E+00	D	BN	NA	MODERATE	N	1	144	144	0	93	53	40	43.01
Non-user	IOB_24	chr22	45810273	A	G	RIBC2	MISSENSE	ENSG00000128408:[ENST00000538017(c.185A>G,cAt>cGt):ENSP00000444196(p.His62Arg)]	NA	NA	3.99E-03	5.03E-01	5.05E-01	3.99E-03	1.33E-02	NA	NA	NA	MODIFIER	NA	NA	162	160	0	105	59	45	42.86
Non-user	IOB_24	chr22	46762970	G	C	CELSR1	MISSENSE	ENSG00000075275:[ENST00000262738(c.8125C>G,Cgg>Ggg):ENSP00000262738(p.Arg2709Gly)]	NA	NA	9.98E-04	9.18E-04	1.50E-03	9.98E-04	3.10E-03	D	PoD	NA	MODERATE	U	0.999	78	78	0	50	28	22	44
Non-user	IOB_24	chr22	46768811	G	A	CELSR1	SILENT	ENSG00000075275:[ENST00000262738(c.7728C>T,gtC>gtT):ENSP00000262738(p.Val2576(=))]	NA	NA	2.64E-02	4.27E-02	3.15E-02	2.64E-02	3.07E-02	NA	NA	NA	LOW	NA	NA	123	123	0	93	0	92	98.92
Non-user	IOB_24	chr22	46930570	G	A	CELSR1	MISSENSE	ENSG00000075275:[ENST00000454637(c.622C>T,cCg>cTg):ENSP00000414689(p.Pro208Leu)]	NA	NA	NA	NA	NA	NA	NA	D	PrD	NA	MODERATE	U	1	351	350	0	252	135	116	46.03
Non-user	IOB_24	chr22	46931402	G	C	CELSR1	MISSENSE	ENSG00000075275:[ENST00000262738(c.1666C>G,Ctg>Gtg):ENSP00000262738(p.Leu556Val)]	NA	NA	2.20E-02	2.87E-02	5.90E-02	2.20E-02	5.73E-02	T	BN	NA	MODERATE	U	0.805	360	359	0	366	186	179	48.91
Non-user	IOB_24	chr22	47091143	A	C	CERK	MISSENSE	ENSG00000100422:[ENST00000541677(c.419T>G,gTg>gGg):ENSP00000438659(p.Val140Gly)]	NA	NA	7.99E-04	2.77E-03	3.35E-03	7.99E-04	2.00E-03	T	BN	NA	MODERATE	N	0.981	103	101	1	85	43	42	49.41
Non-user	IOB_24	chr22	47091231	G	A	CERK	INTRONIC	ENSG00000100422:[ENST00000443629(c.*322-19C>T)]	NA	NA	1.06E-02	1.55E-02	3.02E-02	1.06E-02	3.37E-02	NA	NA	NA	MODIFIER	NA	NA	55	55	0	28	11	17	60.71
Non-user	IOB_24	chr22	50170674	C	T	BRD1	INTRONIC-SS-DNR-PRX	ENSG00000100425:[ENST00000404760(c.3121+8G>A)]	NA	NA	1.30E-02	1.31E-02	4.23E-02	1.30E-02	5.21E-02	NA	NA	NA	LOW	NA	NA	237	236	0	183	93	89	48.63
Non-user	IOB_24	chr22	50277494	G	A	ZBED4	MISSENSE	ENSG00000100426:[ENST00000216268(c.184G>A,Ggt>Agt):ENSP00000216268(p.Gly62Ser)]	NA	NA	6.99E-03	6.11E-03	2.60E-02	6.99E-03	2.97E-02	T_lc	BN	NA	MODERATE	N	0	227	222	0	197	105	92	46.7
Non-user	IOB_24	chr22	50303575	G	A	ALG12	MISSENSE	ENSG00000182858:[ENST00000492791(c.162C>T,Cgc>Tgc):ENSP00000417387(p.Arg55Cys)]	NA	NA	1.66E-02	9.48E-03	3.12E-02	1.66E-02	3.58E-02	NA	BN	NA	MODERATE	N	0.704	177	176	0	98	51	47	47.96
Non-user	IOB_24	chr22	50484261	G	A	TTLL8	INTRONIC	ENSG00000138892:[ENST00000433387(c.616+24C>T)]	NA	NA	3.59E-03	5.65E-03	2.05E-02	3.59E-03	1.74E-02	NA	NA	NA	MODIFIER	NA	NA	233	228	0	189	106	80	42.33
Non-user	IOB_24	chr22	50553706	G	A	MOV10L1	INTRONIC	ENSG00000073146:[ENST00000540615(c.1199+31G>A)]	NA	NA	NA	5.30E-05	3.52E-04	NA	NA	NA	NA	NA	MODIFIER	NA	NA	35	35	0	27	14	13	48.15
Non-user	IOB_24	chr22	50599328	C	T	MOV10L1	INTRONIC	54456:[XM_005261923.1(c.3403-65C>T)]	NA	NA	2.30E-02	NA	NA	2.30E-02	4.29E-02	NA	NA	NA	MODIFIER	NA	NA	173	172	0	128	66	61	47.66
Non-user	IOB_24	chr22	50631945	C	T	TRABD	NCT	ENSG00000170638:[ENST00000463233(n.152-60C>T)]	NA	NA	2.82E-02	NA	NA	2.82E-02	4.70E-02	NA	NA	NA	MODIFIER	NA	NA	245	242	2	224	100	119	53.12
Non-user	IOB_24	chr22	50656072	C	A	NA	INTERGENIC	NA	NA	NA	3.95E-02	NA	NA	3.95E-02	4.91E-02	NA	NA	NA	MODIFIER	NA	NA	222	222	0	174	78	96	55.17
Non-user	IOB_24	chr22	50656108	G	A	NA	INTERGENIC	NA	NA	NA	7.99E-04	NA	NA	7.99E-04	4.10E-03	NA	NA	NA	MODIFIER	NA	NA	225	223	0	184	102	81	44.02
Non-user	IOB_24	chr22	50656647	G	A	TUBGCP6	MISSENSE	ENSG00000128159:[ENST00000425018(c.1165C>T,Cgc>Tgc):ENSP00000405979(p.Arg389Cys)]	NA	NA	7.99E-04	2.06E-03	1.95E-03	7.99E-04	2.00E-03	NA	NA	NA	MODIFIER	NA	NA	290	287	2	215	113	102	47.44
Non-user	IOB_24	chr22	50658966	C	T	TUBGCP6	SILENT	ENSG00000128159:[ENST00000248846(c.3822G>A,ccG>ccA):ENSP00000248846(p.Pro1274(=))]	NA	NA	7.99E-04	1.11E-03	6.25E-03	7.99E-04	3.10E-03	NA	NA	NA	MODIFIER	NA	NA	212	211	1	172	91	80	46.51
Non-user	IOB_24	chr22	50687201	G	T	HDAC10	INTRONIC	ENSG00000100429:[ENST00000415993(c.*445-21C>A)]	NA	NA	NA	1.13E-04	5.12E-04	NA	NA	NA	NA	NA	MODIFIER	NA	NA	288	287	0	225	118	105	46.67
Non-user	IOB_24	chr22	50687201	G	T	MAPK12	NCT	ENSG00000188130:[ENST00000497036(n.5089-21C>A)]	NA	NA	NA	1.13E-04	5.12E-04	NA	NA	NA	NA	NA	MODIFIER	NA	NA	288	287	0	225	118	105	46.67
Non-user	IOB_24	chr22	50689334	G	A	HDAC10	SILENT-SS-PRX	83933:[XM_005261950.1(c.-697C>T)]	NA	NA	NA	9.90E-05	6.42E-04	NA	NA	NA	NA	NA	LOW	NA	NA	93	93	0	76	37	39	51.32
Non-user	IOB_24	chr22	50689334	G	A	MAPK12	EXONIC-NC	ENSG00000188130:[ENST00000497036(n.3660C>T)]	NA	NA	NA	9.90E-05	6.42E-04	NA	NA	NA	NA	NA	LOW	NA	NA	93	93	0	76	37	39	51.32
Non-user	IOB_24	chr22	50716473	G	A	PLXNB2	INTRONIC	ENSG00000196576:[ENST00000411680(c.544-31C>T)]	NA	NA	7.99E-04	1.01E-03	6.73E-03	7.99E-04	4.10E-03	NA	NA	NA	MODIFIER	NA	NA	179	172	0	149	71	67	44.97
Non-user	IOB_24	chr22	50720580	C	T	PLXNB2	INTRONIC	ENSG00000196576:[ENST00000411680(c.203-3455G>A)]	NA	NA	1.40E-03	9.97E-04	7.21E-03	1.40E-03	5.10E-03	NA	NA	NA	MODIFIER	NA	NA	391	390	0	303	162	141	46.53
Non-user	IOB_24	chr22	50724566	C	T	PLXNB2	INTRONIC	23654:[XM_005261908.1(c.2123+36G>A)]	NA	NA	3.99E-03	2.49E-03	1.76E-02	3.99E-03	2.04E-02	NA	NA	NA	MODIFIER	NA	NA	228	227	0	180	89	90	50
Non-user	IOB_24	chr22	50753042	C	T	DENND6B	SILENT	ENSG00000205593:[ENST00000413817(c.969G>A,acG>acA):ENSP00000391524(p.Thr323(=))]	NA	NA	5.99E-04	1.95E-03	5.59E-03	5.99E-04	2.00E-03	NA	NA	NA	LOW	NA	NA	257	257	0	185	92	93	50.27
Non-user	IOB_24	chr22	50970391	C	T	ODF3B	INTRONIC	440836:[XM_005261922.1(c.-46+7G>A)]	NA	NA	7.99E-04	6.75E-04	8.47E-03	7.99E-04	4.10E-03	NA	NA	NA	LOW	NA	NA	108	107	0	83	39	43	51.81
Non-user	IOB_24	chr22	51008121	G	A	CHKB-CPT1B	NCT	ENSG00000254413:[ENST00000492556(n.4346-24C>T)]	NA	NA	3.93E-02	5.44E-02	5.29E-02	3.93E-02	3.89E-02	NA	NA	NA	MODIFIER	NA	NA	171	169	0	119	67	52	43.7
Non-user	IOB_24	chr22	51008121	G	A	CPT1B	INTRONIC	ENSG00000205560:[ENST00000457250(c.2041-24C>T)]	NA	NA	3.93E-02	5.44E-02	5.29E-02	3.93E-02	3.89E-02	NA	NA	NA	MODIFIER	NA	NA	171	169	0	119	67	52	43.7
Non-user	IOB_24	chr22	51011348	G	A	CHKB-CPT1B	3UTR	ENSG00000254413:[ENST00000453634(c.*1539C>T)]	NA	NA	3.38E-02	3.14E-02	7.12E-02	3.37E-02	7.98E-02	NA	NA	NA	LOW	NA	NA	114	114	0	99	47	52	52.53
Non-user	IOB_24	chr22	51011348	G	A	CPT1B	SILENT	ENSG00000205560:[ENST00000434492(c.699C>T,ctC>ctT):ENSP00000410966(p.Leu233(=))]	NA	NA	3.38E-02	3.14E-02	7.12E-02	3.37E-02	7.98E-02	NA	NA	NA	LOW	NA	NA	114	114	0	99	47	52	52.53
Non-user	IOB_24	chr22	51012854	G	A	CHKB-CPT1B	NCT-SS-PRX	386593:[NR_027928.2(n.2456-6C>T)]	NA	NA	2.44E-02	2.89E-02	7.13E-02	2.44E-02	7.98E-02	NA	NA	NA	LOW	NA	NA	252	250	1	176	80	95	53.98
Non-user	IOB_24	chr22	51012854	G	A	CPT1B	INTRONIC-SS-ACR-PRX	ENSG00000205560:[ENST00000423069(c.139-6C>T)]	NA	NA	2.44E-02	2.89E-02	7.13E-02	2.44E-02	7.98E-02	NA	NA	NA	LOW	NA	NA	252	250	1	176	80	95	53.98
Non-user	IOB_24	chr22	51041663	C	A	MAPK8IP2	SILENT	ENSG00000008735:[ENST00000329492(c.183C>A,tcC>tcA):ENSP00000330572(p.Ser61(=))]	NA	NA	4.97E-02	8.59E-02	8.67E-02	4.97E-02	5.93E-02	NA	NA	NA	LOW	NA	NA	183	182	0	134	88	45	33.58

Patel *et al.* , 2021. Whole-exome sequencing analysis of oral squamous cell carcinoma delineated by tobacco usage habits
Supplementary Table 4. List of somatic single nucleotide variants identified using whole exome sequencing of OSCC patients

Cohort	Sample	Chromosome	Genomic position	Reference allele	Alternate allele	Genesymbol	Variant type	Variant description	OncoMD	COSMIC	dbSNP	ExAC	ExAC_SAS	1000G	1000G_SAS	SIFT	Polyphen2	CONDEL	VEP	LRT	PhastCon7Way	Total reads in Normal	Reference read support in Normal	Alternative read support in Normal	Total reads in Tumor	Reference read support in Tumor	Alternate read support in Tumor	Alternate allele frequency in Tumor
Non-user	IOB_24	chr22	51153371	G	A	SHANK3	MISSENSE	ENSG00000251322:[ENST00000262795(c.2209G>A,Gcc>Acc):ENSP00000442518(p.Ala737Thr)]	NA	NA	2.54E-02	6.45E-02	9.36E-02	2.54E-02	7.98E-02	NA	NA	NA	MODERATE	NA	NA	80	80	0	72	45	27	37.5
Non-user	IOB_24	chr3	3080651	C	T	CNTN4	SILENT	ENSG00000144619:[ENST00000397459(c.1143C>T,ggC>ggT):ENSP00000380600(p.Gly381(=))]	NA	chr3*3080651*3080651*COSM173708*C*T*1*large_intestine*1|chr3*3080651*3080651*COSM173709*C*T*1*large_intestine*1	5.99E-04	5.44E-04	4.00E-03	5.99E-04	3.10E-03	NA	NA	NA	LOW	NA	NA	107	107	0	93	41	50	53.76
Non-user	IOB_24	chr3	4669478	C	G	ITPR1	SILENT	ENSG00000150995:[ENST00000302640(c.195C>G,cgC>cgG):ENSP00000306253(p.Arg65(=))]	NA	NA	4.19E-03	1.95E-03	1.41E-02	4.19E-03	2.15E-02	NA	NA	NA	MODIFIER	NA	NA	277	274	0	225	102	119	52.89
Non-user	IOB_24	chr3	4704734	G	A	ITPR1	INTRONIC	ENSG00000150995:[ENST00000354582(c.1413-15G>A)]	NA	NA	2.00E-04	2.50E-05	0.00E+00	2.00E-04	0.00E+00	NA	NA	NA	MODIFIER	NA	NA	310	309	0	268	148	119	44.4
Non-user	IOB_24	chr3	9547805	C	T	LHFPL4	SILENT	ENSG00000156959:[ENST00000287585(c.489G>A,acG>acA):ENSP00000287585(p.Thr163(=))]	NA	chr3*9547805*9547805*COSM118787*C*T*1*ovary*1		2.72E-04	1.57E-03	NA	NA	NA	NA	NA	LOW	NA	NA	236	236	0	187	112	73	39.04
Non-user	IOB_24	chr3	9988175	C	T	PRRT3	SILENT	ENSG00000163704:[ENST00000295984(c.2682G>A,ggG>ggA):ENSP00000295984(p.Gly894(=))]	NA	NA	2.38E-02	7.24E-02	1.04E-01	2.38E-02	6.34E-02	NA	NA	NA	MODIFIER	NA	NA	185	185	0	153	68	85	55.56
Non-user	IOB_24	chr3	10094014	A	G	FANCD2	INTRONIC	ENSG00000144554:[ENST00000421731(c.45-57A>G)]	NA	NA	NA	NA	NA	NA	NA	NA	NA	NA	MODIFIER	NA	NA	90	90	0	54	28	26	48.15
Non-user	IOB_24	chr3	12851445	T	C	CAND2	SILENT	ENSG00000144712:[ENST00000446928(c.241T>C,Ttg>Ctg):ENSP00000390996(p.Leu81(=))]	NA	NA	4.79E-02	2.69E-02	1.19E-01	4.79E-02	6.13E-02	NA	NA	NA	LOW	NA	NA	165	164	0	130	57	73	56.15
Non-user	IOB_24	chr3	13395539	G	A	NUP210	SILENT	ENSG00000132182:[ENST00000254508(c.2397C>T,ttC>ttT):ENSP00000254508(p.Phe799(=))]	NA	NA	2.40E-03	1.75E-03	8.49E-03	2.40E-03	8.20E-03	NA	NA	NA	LOW	NA	0.967	156	156	0	176	69	107	60.8
Non-user	IOB_24	chr3	14180706	C	T	RP11-434D12.1	SILENT	ENSG00000268279:[ENST00000608606(c.145C>T,agC>agT):ENSP00000476275(p.Ser49(=))]	NA	NA	2.14E-02	2.00E-02	3.17E-02	2.14E-02	2.45E-02	NA	NA	NA	LOW	NA	NA	173	173	0	125	67	58	46.4
Non-user	IOB_24	chr3	14180706	C	T	TMEM43	SILENT	ENSG00000170876:[ENST00000306077(c.909C>T,agC>agT):ENSP00000303992(p.Ser303(=))]	NA	NA	2.14E-02	2.00E-02	3.17E-02	2.14E-02	2.45E-02	NA	NA	NA	LOW	NA	NA	173	173	0	125	67	58	46.4
Non-user	IOB_24	chr3	14567382	C	T	GRIP2	MISSENSE	80852:[NM_001080423.2(c.388G>A,Gcg>Acg):NP_001073892.2(p.Ala130Thr)]	NA	NA	3.06E-02	3.24E-02	3.16E-02	3.06E-02	3.68E-02	NA	NA	NA	MODIFIER	NA	NA	41	41	0	26	8	18	69.23
Non-user	IOB_24	chr3	15711942	G	A	ANKRD28	SILENT	ENSG00000206560:[ENST00000399451(c.2997C>T,gcC>gcT):ENSP00000382379(p.Ala999(=))]	NA	NA	NA	NA	NA	NA	NA	NA	NA	NA	LOW	NA	NA	306	304	0	259	148	109	42.08
Non-user	IOB_24	chr3	21478582	T	C	ZNF385D	MISSENSE	ENSG00000151789:[ENST00000281523(c.553A>G,Act>Gct):ENSP00000281523(p.Thr185Ala)]	NA	NA	5.99E-04	3.63E-04	2.66E-03	5.99E-04	3.10E-03	NA	NA	NA	MODIFIER	NA	NA	207	205	0	168	76	91	54.17
Non-user	IOB_24	chr3	21553208	T	C	ZNF385D	3UTR	ENSG00000151789:[ENST00000446749(c.*6A>G)]	NA	NA	3.55E-02	4.59E-02	6.88E-02	3.55E-02	9.00E-02	NA	NA	NA	MODIFIER	NA	NA	307	307	0	298	141	156	52.35
Non-user	IOB_24	chr3	21553248	A	T	ZNF385D	INTRONIC	79750:[XM_005265486.1(c.580-733T>A)]	NA	NA	3.87E-02	4.64E-02	6.88E-02	3.87E-02	9.00E-02	NA	NA	NA	MODIFIER	NA	NA	247	246	0	223	116	105	47.09
Non-user	IOB_24	chr3	25770784	C	G	NGLY1	MISSENSE	ENSG00000151092:[ENST00000417874(c.1325G>C,tGt>tCt):ENSP00000389888(p.Cys442Ser)]	NA	NA	2.40E-03	1.15E-03	9.16E-03	2.40E-03	1.23E-02	NA	NA	NA	MODIFIER	NA	NA	67	64	0	43	21	22	51.16
Non-user	IOB_24	chr3	25833178	A	T	OXSM	MISSENSE	ENSG00000151093:[ENST00000280701(c.667A>T,Ata>Tta):ENSP00000280701(p.Ile223Leu)]	NA	NA	2.80E-03	1.19E-03	8.36E-03	2.80E-03	1.33E-02	D	PoD	NA	MODERATE	NA	NA	108	108	0	108	59	49	45.37
Non-user	IOB_24	chr3	25900139	T	A	LINC00692	EXONIC-NC	285326:[XR_245123.1(n.725A>T)]	NA	NA	3.77E-02	1.91E-02	1.77E-02	3.77E-02	1.74E-02	NA	NA	NA	MODIFIER	NA	NA	464	462	0	376	181	193	51.33
Non-user	IOB_24	chr3	32201038	T	C	GPD1L	INTRONIC	23171:[XM_005264983.1(c.619-22T>C)]	NA	NA	1.40E-03	1.11E-03	7.34E-03	1.40E-03	7.20E-03	NA	NA	NA	MODIFIER	NA	NA	34	34	0	11	4	7	63.64
Non-user	IOB_24	chr3	32491084	T	C	CMTM7	INTRONIC	ENSG00000153551:[ENST00000334983(c.432+40T>C)]	NA	NA	4.87E-02	5.74E-02	4.44E-02	4.87E-02	3.68E-02	NA	NA	NA	MODIFIER	NA	NA	59	59	0	44	22	22	50
Non-user	IOB_24	chr3	33255543	G	A	SUSD5	MISSENSE	ENSG00000173705:[ENST00000309558(c.167C>T,gCt>gTt):ENSP00000308727(p.Ala56Val)]	NA	NA	NA	NA	NA	NA	NA	T	BN	NA	MODERATE	NA	NA	91	91	0	92	44	47	51.09
Non-user	IOB_24	chr3	36779929	C	T	DCLK3	SILENT	ENSG00000163673:[ENST00000416516(c.222G>A,agG>agA):ENSP00000394484(p.Arg74(=))]	NA	NA	8.79E-03	3.90E-03	4.65E-02	8.79E-03	4.40E-02	NA	NA	NA	LOW	NA	0	318	315	0	173	85	86	49.71
Non-user	IOB_24	chr3	36896824	C	T	TRANK1	SILENT	ENSG00000168016:[ENST00000301807(c.2607G>A,acG>acA):ENSP00000301807(p.Thr869(=))]	NA	NA	9.98E-04	1.46E-03	9.87E-03	9.98E-04	5.10E-03	NA	NA	NA	LOW	NA	NA	284	283	1	220	113	106	48.18
Non-user	IOB_24	chr3	37096013	G	A	LRRFIP2	INTRONIC	9209:[XM_005265541.1(c.1843-7C>T)]	NA	NA	8.99E-03	1.60E-02	7.51E-03	8.99E-03	6.10E-03	NA	NA	NA	LOW	NA	NA	294	292	0	230	113	116	50.43
Non-user	IOB_24	chr3	37096013	G	A	MLH1	INTRONIC	ENSG00000076242:[ENST00000536378(c.1380+5505G>A)]	NA	NA	8.99E-03	1.60E-02	7.51E-03	8.99E-03	6.10E-03	NA	NA	NA	LOW	NA	NA	294	292	0	230	113	116	50.43
Non-user	IOB_24	chr3	38347785	G	A	SLC22A14	MISSENSE	ENSG00000144671:[ENST00000273173(c.268G>A,Gtg>Atg):ENSP00000273173(p.Val90Met)]	NA	chr3*38347785*38347785*COSM1537516*G*C*1*lung*1	2.00E-03	1.56E-03	7.57E-03	2.00E-03	8.20E-03	T	BN	NA	MODERATE	N	0	281	279	0	190	86	102	53.68
Non-user	IOB_24	chr3	39152386	C	T	TTC21A	MISSENSE	ENSG00000168026:[ENST00000301819(c.313C>T,Cgc>Tgc):ENSP00000301819(p.Arg105Cys)]	NA	NA	1.80E-03	7.97E-04	5.76E-03	1.80E-03	9.20E-03	D	PoD	NA	MODERATE	D	0.998	79	79	0	59	40	19	32.2
Non-user	IOB_24	chr3	39178437	G	T	TTC21A	MISSENSE	ENSG00000168026:[ENST00000440121(c.3020G>T,cGc>cTc):ENSP00000410882(p.Arg1007Leu)]	NA	NA	2.00E-02	4.83E-02	2.85E-02	2.00E-02	2.45E-02	D	PrD	NA	MODERATE	N	1	71	71	0	63	34	28	44.44
Non-user	IOB_24	chr3	40503582	G	A	RPL14	SILENT	ENSG00000188846:[ENST00000338970(c.507G>A,gcG>gcA):ENSP00000345156(p.Ala169(=))]	NA	chr3*40503582*40503582*COSM1422978*G*A*1*large_intestine*1	NA	3.94E-05	1.95E-04	NA	NA	NA	NA	NA	LOW	NA	NA	96	96	0	59	37	20	33.9
Non-user	IOB_24	chr3	40573556	A	G	ZNF621	MISSENSE	ENSG00000172888:[ENST00000310898(c.295A>G,Ata>Gta):ENSP00000312144(p.Ile99Val)]	NA	NA	1.12E-02	4.71E-03	1.15E-02	1.12E-02	1.02E-02	NA	NA	NA	MODERATE	NA	0	117	117	0	88	37	51	57.95
Non-user	IOB_24	chr3	42265641	C	T	TRAK1	3UTR	ENSG00000182606:[ENST00000327628(c.*412C>T)]	NA	NA	1.04E-02	3.61E-02	4.09E-02	1.04E-02	3.68E-02	NA	NA	NA	MODIFIER	NA	NA	97	96	0	71	36	35	49.3
Non-user	IOB_24	chr3	42727907	C	G	KLHL40	MISSENSE	ENSG00000157119:[ENST00000287777(c.797C>G,aCc>aGc):ENSP00000287777(p.Thr266Ser)]	NA	chr3*42727907*42727907*COSM1662354*C*G*1*kidney*1	NA	NA	NA	NA	NA	D	BN	NA	MODERATE	D	0.999	103	103	0	93	59	34	36.56
Non-user	IOB_24	chr3	42733341	T	C	KLHL40	INTRONIC	131377:[XM_005264866.1(c.1647-33T>C)]	NA	NA	4.91E-02	4.24E-02	4.30E-02	4.91E-02	4.60E-02	NA	NA	NA	MODIFIER	NA	NA	166	166	0	126	67	59	46.83
Non-user	IOB_24	chr3	42954793	G	A	KRBOX1	INTRONIC	ENSG00000240747:[ENST00000426937(c.-47+4392G>A)]	NA	NA	1.04E-02	3.30E-03	4.18E-03	1.04E-02	1.02E-02	NA	NA	NA	MODIFIER	NA	NA	88	88	0	59	34	25	42.37
Non-user	IOB_24	chr3	42954793	G	A	ZNF662	SILENT-SS-PRX	ENSG00000182983:[ENST00000422021(c.152-4148G>A)]	NA	NA	1.04E-02	3.30E-03	4.18E-03	1.04E-02	1.02E-02	NA	NA	NA	MODIFIER	NA	NA	88	88	0	59	34	25	42.37
Non-user	IOB_24	chr3	42956869	G	C	KRBOX1	NCT	ENSG00000273291:[ENST00000451200(n.169-23473G>C)]	NA	NA	1.04E-02	3.11E-03	5.65E-03	1.04E-02	1.02E-02	NA	NA	NA	MODIFIER	NA	NA	118	118	0	92	42	50	54.35
Non-user	IOB_24	chr3	42956869	G	C	ZNF662	INTRONIC	ENSG00000182983:[ENST00000422021(c.152-2072G>C)]	NA	NA	1.04E-02	3.11E-03	5.65E-03	1.04E-02	1.02E-02	NA	NA	NA	MODIFIER	NA	NA	118	118	0	92	42	50	54.35
Non-user	IOB_24	chr3	44489880	T	C	ZNF445	MISSENSE	ENSG00000185219:[ENST00000396077(c.1283A>G,tAt>tGt):ENSP00000379387(p.Tyr428Cys)]	NA	NA	4.85E-02	1.13E-01	2.35E-02	4.85E-02	1.02E-02	NA	NA	NA	MODERATE	NA	0	253	252	0	217	112	105	48.39
Non-user	IOB_24	chr3	44762905	T	C	ZNF502	MISSENSE	ENSG00000196653:[ENST00000296091(c.596T>C,gTt>gCt):ENSP00000296091(p.Val199Ala)]	NA	NA	3.99E-04	1.48E-03	5.03E-03	3.99E-04	1.00E-03	T	BN	NA	MODERATE	NA	NA	133	133	0	95	53	42	44.21
Non-user	IOB_24	chr3	44905764	C	T	KIF15	INTRONIC	ENSG00000163808:[ENST00000422209(c.*60-9057C>T)]	NA	chr3*44905764*44905764*COSM1045109*C*T*1*endometrium*1	3.99E-04	1.07E-04	4.24E-04	3.99E-04	2.00E-03	NA	NA	NA	MODIFIER	NA	NA	94	94	0	76	45	31	40.79
Non-user	IOB_24	chr3	44905764	C	T	TMEM42	MISSENSE	ENSG00000169964:[ENST00000302392(c.268C>T,Cgg>Tgg):ENSP00000306564(p.Arg90Trp)]	NA	chr3*44905764*44905764*COSM1045109*C*T*1*endometrium*1	3.99E-04	1.07E-04	4.24E-04	3.99E-04	2.00E-03	NA	NA	NA	MODIFIER	NA	NA	94	94	0	76	45	31	40.79
Non-user	IOB_24	chr3	44999984	C	T	ZDHHC3	INTRONIC	ENSG00000163812:[ENST00000455235(c.-150-13200G>A)]	NA	NA	NA	NA	NA	NA	NA	NA	NA	NA	MODIFIER	NA	NA	100	100	0	83	32	51	61.45
Non-user	IOB_24	chr3	45152343	G	A	CDCP1	INTRONIC	64866:[XM_005265404.1(c.458-10C>T)]	NA	NA	2.78E-02	5.73E-02	4.95E-02	2.78E-02	3.27E-02	NA	NA	NA	MODIFIER	NA	NA	74	74	0	43	28	15	34.88
Non-user	IOB_24	chr3	45637440	C	T	LIMD1	SILENT	ENSG00000144791:[ENST00000273317(c.1069C>T,Ctg>Ttg):ENSP00000273317(p.Leu357(=))]	NA	NA	7.99E-04	6.43E-04	4.84E-04	7.99E-04	0.00E+00	NA	NA	NA	LOW	NA	0	164	164	0	133	79	53	39.85
Non-user	IOB_24	chr3	46488936	C	T	LTF	INTRONIC	ENSG00000012223:[ENST00000443496(c.1174-47G>A)]	NA	NA	3.10E-02	5.81E-02	5.04E-02	3.10E-02	4.60E-02	NA	NA	NA	MODIFIER	NA	NA	120	120	0	77	44	31	40.26
Non-user	IOB_24	chr3	47044740	A	C	NBEAL2	SILENT	ENSG00000160796:[ENST00000292309(c.5109A>C,ccA>ccC):ENSP00000292309(p.Pro1703(=))]	NA	NA	7.99E-03	1.51E-02	2.29E-02	7.99E-03	1.33E-02	NA	NA	NA	LOW	NA	NA	142	141	1	151	75	75	49.67
Non-user	IOB_24	chr3	48228212	G	A	CDC25A	SILENT	ENSG00000164045:[ENST00000302506(c.228C>T,tcC>tcT):ENSP00000303706(p.Ser76(=))]	NA	NA	5.39E-03	1.99E-02	1.59E-02	5.39E-03	1.02E-02	NA	NA	NA	MODIFIER	NA	NA	166	166	0	130	71	59	45.38
Non-user	IOB_24	chr3	48414274	C	T	FBXW12	MISSENSE	ENSG00000164049:[ENST00000296438(c.17C>T,cCt>cTt):ENSP00000296438(p.Pro6Leu)]	NA	NA	5.99E-03	1.36E-02	5.15E-03	5.99E-03	2.00E-03	D	PoD	NA	MODERATE	D	0.087	106	106	0	69	43	26	37.68
Non-user	IOB_24	chr3	48464167	G	T	PLXNB1	NCT-SS-PRX	ENSG00000164050:[ENST00000473683(n.253+7C>A)]	NA	NA	3.99E-04	3.67E-04	2.98E-03	3.99E-04	2.00E-03	NA	NA	NA	LOW	NA	NA	141	141	0	143	72	70	48.95
Non-user	IOB_24	chr3	48506409	G	A	ATRIP	SILENT	ENSG00000164053:[ENST00000320211(c.2235G>A,ctG>ctA):ENSP00000323099(p.Leu745(=))]	NA	NA	7.99E-04	5.40E-04	3.41E-03	7.99E-04	4.10E-03	NA	NA	NA	LOW	NA	NA	274	274	0	229	101	128	55.9
Non-user	IOB_24	chr3	48604016	C	G	COL7A1	INTRONIC	ENSG00000114270:[ENST00000454817(c.8209-20G>C)]	NA	NA	2.58E-02	5.37E-02	1.74E-02	2.58E-02	9.20E-03	NA	NA	NA	MODIFIER	NA	NA	154	153	0	123	71	52	42.28
Non-user	IOB_24	chr3	48604059	G	A	COL7A1	NCT	ENSG00000114270:[ENST00000487017(n.4943+34C>T)]	NA	NA	2.58E-02	5.34E-02	1.75E-02	2.58E-02	9.20E-03	NA	NA	NA	MODIFIER	NA	NA	136	135	1	125	72	53	42.4
Non-user	IOB_24	chr3	48725707	C	T	IP6K2	SILENT	ENSG00000068745:[ENST00000328631(c.1280G>A,tGa>tAa):ENSP00000331103(p.Ter427Ter)]	NA	NA	3.00E-02	6.29E-02	1.82E-02	3.00E-02	1.02E-02	NA	NA	NA	LOW	NA	NA	95	95	0	74	31	43	58.11
Non-user	IOB_24	chr3	49321437	G	A	USP4	SILENT	ENSG00000114316:[ENST00000351842(c.2382C>T,gtC>gtT):ENSP00000341028(p.Val794(=))]	NA	NA	3.99E-04	3.63E-04	1.57E-03	3.99E-04	2.00E-03	NA	NA	NA	LOW	NA	NA	239	239	0	181	87	94	51.93
Non-user	IOB_24	chr3	49719361	C	A	APEH	MISSENSE	ENSG00000164062:[ENST00000296456(c.1564C>A,Cca>Aca):ENSP00000296456(p.Pro522Thr)]	NA	NA	NA	NA	NA	NA	NA	NA	NA	NA	MODIFIER	NA	NA	224	224	0	183	178	5	2.73
Non-user	IOB_24	chr3	49830558	C	T	CDHR4	INTRONIC	ENSG00000187492:[ENST00000460430(c.112+36G>A)]	NA	NA	1.60E-03	5.63E-03	6.23E-03	1.60E-03	3.10E-03	NA	NA	NA	MODIFIER	NA	NA	330	329	0	269	151	114	42.38
Non-user	IOB_24	chr3	49842064	G	A	FAM212A	MISSENSE	ENSG00000185614:[ENST00000333323(c.508G>A,Gag>Aag):ENSP00000329735(p.Glu170Lys)]	NA	NA	5.99E-04	6.93E-04	3.88E-03	5.99E-04	3.10E-03	D	BN	NA	MODERATE	N	0.992	201	201	0	161	84	75	46.58
Non-user	IOB_24	chr3	49846934	G	A	UBA7	INTRONIC	ENSG00000182179:[ENST00000333486(c.2089-37C>T)]	NA	NA	1.60E-03	2.74E-03	5.03E-03	1.60E-03	3.10E-03	NA	NA	NA	MODIFIER	NA	NA	281	280	0	173	77	96	55.49
Non-user	IOB_24	chr3	49847737	C	T	UBA7	MISSENSE	ENSG00000182179:[ENST00000333486(c.1592G>A,cGt>cAt):ENSP00000333266(p.Arg531His)]	NA	NA	2.00E-03	2.80E-03	5.03E-03	2.00E-03	3.10E-03	NA	NA	NA	MODIFIER	NA	NA	219	219	0	161	89	72	44.72
Non-user	IOB_24	chr3	49967128	G	T	MON1A	SILENT	ENSG00000164077:[ENST00000296473(c.192C>A,ccC>ccA):ENSP00000296473(p.Pro64(=))]	NA	NA	NA	NA	NA	NA	NA	NA	NA	NA	MODIFIER	NA	NA	147	147	0	143	138	5	3.5
Non-user	IOB_24	chr3	50396144	G	A	CYB561D2	NCT	11068:[NR_111912.1(n.442+7153G>A)]	NA	NA	NA	NA	NA	NA	NA	NA	NA	NA	MODIFIER	NA	NA	280	279	0	230	135	93	40.43
Non-user	IOB_24	chr3	50396144	G	A	TMEM115	SILENT	ENSG00000126062:[ENST00000266025(c.351C>T,taC>taT):ENSP00000266025(p.Tyr117(=))]	NA	NA	NA	NA	NA	NA	NA	NA	NA	NA	MODIFIER	NA	NA	280	279	0	230	135	93	40.43
Non-user	IOB_24	chr3	50396144	G	A	XXcos-LUCA11.5	INTRONIC	ENSG00000272104:[ENST00000606589(c.127+7153G>A)]	NA	NA	NA	NA	NA	NA	NA	NA	NA	NA	MODIFIER	NA	NA	280	279	0	230	135	93	40.43
Non-user	IOB_24	chr3	51967633	C	T	RRP9	INTRONIC	ENSG00000114767:[ENST00000232888(c.1335-18G>A)]	NA	NA	5.19E-03	2.32E-03	1.64E-02	5.19E-03	2.56E-02	NA	NA	NA	MODIFIER	NA	NA	240	238	1	177	90	84	47.46
Non-user	IOB_24	chr3	52582237	C	T	PBRM1	MISSENSE	ENSG00000163939:[ENST00000409057(c.4747G>A,Gtc>Atc):ENSP00000386593(p.Val1583Ile)]	NA	NA	NA	2.47E-05	6.07E-05	NA	NA	NA	NA	NA	MODERATE	NA	0.597	45	45	0	31	20	11	35.48
Non-user	IOB_24	chr3	52582237	C	T	SMIM4	INTRONIC	ENSG00000168273:[ENST00000476842(c.151+11315C>T)]	NA	NA	NA	2.47E-05	6.07E-05	NA	NA	NA	NA	NA	MODERATE	NA	0.597	45	45	0	31	20	11	35.48
Non-user	IOB_24	chr3	52816229	C	T	ITIH1	SILENT	ENSG00000055957:[ENST00000540715(c.450C>T,aaC>aaT):ENSP00000443973(p.Asn150(=))]	NA	NA	1.80E-03	4.01E-03	9.57E-03	1.80E-03	5.10E-03	NA	NA	NA	LOW	NA	0.237	205	205	0	126	64	61	48.41
Non-user	IOB_24	chr3	52867696	G	A	MUSTN1	NONSENSE	ENSG00000272573:[ENST00000486659(c.94C>T,Cga>Tga):ENSP00000419877(p.Arg32Ter)]	NA	NA	2.00E-04	2.29E-05	0.00E+00	2.00E-04	0.00E+00	NA	NA	NA	HIGH	NA	NA	147	147	0	140	135	5	3.57
Non-user	IOB_24	chr3	52867696	G	A	RP5-966M1.6	NONSENSE	ENSG00000243696:[ENST00000468472(c.79C>T,Cga>Tga):ENSP00000422253(p.Arg27Ter)]	NA	NA	2.00E-04	2.29E-05	0.00E+00	2.00E-04	0.00E+00	NA	NA	NA	HIGH	NA	NA	147	147	0	140	135	5	3.57
Non-user	IOB_24	chr3	52867696	G	A	TMEM110-MUSTN1	MISSENSE	ENSG00000248592:[ENST00000514466(c.382C>T,cCg>cTg):ENSP00000422189(p.Pro128Leu)]	NA	NA	2.00E-04	2.29E-05	0.00E+00	2.00E-04	0.00E+00	NA	NA	NA	HIGH	NA	NA	147	147	0	140	135	5	3.57
Non-user	IOB_24	chr3	53260926	G	A	TKT	INTRONIC	ENSG00000163931:[ENST00000423516(c.1598-32C>T)]	NA	NA	3.00E-03	1.34E-03	1.49E-02	3.00E-03	1.53E-02	NA	NA	NA	MODIFIER	NA	NA	107	106	0	70	40	30	42.86
Non-user	IOB_24	chr3	53787662	G	T	CACNA1D	MISSENSE	ENSG00000157388:[ENST00000350061(c.3739G>T,Gtg>Ttg):ENSP00000288133(p.Val1247Leu)]	NA	NA	NA	1.24E-04	9.08E-04	NA	NA	T_lc	BN	NA	MODERATE	D	1	172	171	1	140	73	66	47.14
Non-user	IOB_24	chr3	53855738	G	C	CHDH	SILENT	ENSG00000016391:[ENST00000315251(c.921C>G,ctC>ctG):ENSP00000319851(p.Leu307(=))]	NA	NA	2.46E-02	4.43E-02	6.62E-02	2.46E-02	5.32E-02	NA	NA	NA	LOW	NA	NA	147	147	0	102	53	48	47.06
Non-user	IOB_24	chr3	53889439	T	C	IL17RB	NCT	55540:[XR_245147.1(n.568+71T>C)]	NA	NA	2.26E-02	NA	NA	2.26E-02	4.91E-02	NA	NA	NA	MODIFIER	NA	NA	72	72	0	62	34	28	45.16
Non-user	IOB_24	chr3	53909952	A	G	ACTR8	INTRONIC	ENSG00000113812:[ENST00000231909(c.161+23T>C)]	NA	NA	2.54E-02	4.10E-02	6.95E-02	2.54E-02	4.91E-02	NA	NA	NA	MODIFIER	NA	NA	226	226	0	215	98	115	53.49
Non-user	IOB_24	chr3	53910633	G	C	ACTR8	INTRONIC	ENSG00000113812:[ENST00000486794(c.174+14C>G)]	NA	NA	2.54E-02	4.32E-02	7.20E-02	2.54E-02	4.91E-02	NA	NA	NA	MODIFIER	NA	NA	45	44	0	41	25	16	39.02

Patel *et al.*, 2021. Whole-exome sequencing analysis of oral squamous cell carcinoma delineated by tobacco usage habits
Supplementary Table 4. List of somatic single nucleotide variants identified using whole exome sequencing of OSCC patients

Cohort	Sample	Chromosome	Genomic position	Reference allele	Alternate allele	Genesymbol	Variant type	Variant description	OncoMD	COSMIC	dbSNP	ExAC	ExAC_SAS	1000G	1000G_SAS	SIFT	Polyphen2	CONDEL	VEP	LRT	PhastCon7Way	Total reads in Normal	Reference read support in Normal	Alternative read support in Normal	Total reads in Tumor	Reference read support in Tumor	Alternate read support in Tumor	Alternate allele frequency in Tumor
Non-user	IOB_24	chr3	53912380	G	A	ACTR8	INTRONIC-SS-DNR-PRX	ENSG00000113812:[ENST00000482349(c.72+4C>T)]	NA	NA	2.68E-02	4.11E-02	6.94E-02	2.68E-02	4.91E-02	NA	NA	NA	LOW	NA	NA	208	208	0	177	86	89	50.28
Non-user	IOB_24	chr3	53919720	G	T	SELK	INTRONIC	ENSG00000113811:[ENST00000495461(c.282-132C>A)]	NA	NA	2.54E-02	NA	NA	2.54E-02	4.91E-02	NA	NA	NA	MODIFIER	NA	NA	42	42	0	31	15	16	51.61
Non-user	IOB_24	chr3	54615803	T	C	CACNA2D3	INTRONIC	ENSG00000157445:[ENST00000468658(c.*303-27T>C)]	NA	NA	NA	4.59E-04	2.92E-03	NA	NA	NA	NA	NA	MODIFIER	NA	NA	32	32	0	12	5	7	58.33
Non-user	IOB_24	chr3	56600676	T	C	CCDC66	MISSENSE	ENSG00000180376:[ENST00000326595(c.497T>C,aTt>aCt):ENSP00000326050(p.Ile166Thr)]	NA	NA	1.20E-02	6.90E-03	4.95E-02	1.20E-02	5.93E-02	NA	NA	NA	MODIFIER	NA	NA	55	55	0	26	13	12	46.15
Non-user	IOB_24	chr3	56649309	C	T	CCDC66	INTRONIC-SS-DNR-PRX	ENSG00000180376:[ENST00000341455(c.*999+9C>T)]	NA	NA	2.72E-02	1.12E-02	5.01E-02	2.72E-02	5.93E-02	NA	NA	NA	MODIFIER	NA	NA	146	146	0	116	67	49	42.24
Non-user	IOB_24	chr3	57432311	C	T	DNAH12	MISSENSE	ENSG00000174844:[ENST00000351747(c.3823G>A,Gtg>Atg):ENSP00000295937(p.Val1275Met)]	NA	NA	1.94E-02	2.21E-02	4.43E-02	1.94E-02	6.85E-02	D	PrD	NA	MODERATE	NA	NA	219	219	0	175	79	96	54.86
Non-user	IOB_24	chr3	57542456	T	C	PDE12	MISSENSE	ENSG00000174840:[ENST00000311180(c.350T>C,gTg>gCg):ENSP00000309142(p.Val117Ala)]	NA	NA	1.58E-02	1.42E-02	5.37E-02	1.58E-02	6.44E-02	T	BN	NA	MODERATE	N	0	247	247	0	198	121	77	38.89
Non-user	IOB_24	chr3	58097556	T	G	FLNB	SILENT	ENSG00000136068:[ENST00000419752(c.2016T>G,ccT>ccG):ENSP00000414532(p.Pro672(=))]	NA	NA	4.19E-03	3.16E-03	2.28E-02	4.19E-03	2.15E-02	NA	NA	NA	LOW	NA	NA	170	170	0	144	79	65	45.14
Non-user	IOB_24	chr3	58395842	A	T	PXK	MISSENSE	ENSG00000168297:[ENST00000356151(c.1421A>T,aAt>aTt):ENSP00000348472(p.Asn474Ile)]	NA	NA	9.39E-03	9.99E-03	3.96E-02	9.38E-03	3.68E-02	NA	NA	NA	MODIFIER	NA	NA	274	272	0	223	105	118	52.91
Non-user	IOB_24	chr3	58520774	G	A	ACOX2	SILENT	ENSG00000168306:[ENST00000302819(c.60C>T,ccC>ccT):ENSP00000307697(p.Pro20(=))]	NA	chr3*58520774*58520774*COSM2782810*G*A*1*central_nervous_system*1	NA	8.24E-06	0.00E+00	NA	NA	NA	NA	NA	MODIFIER	NA	NA	128	128	0	100	43	56	56
Non-user	IOB_24	chr3	62063860	A	G	PTPRG	SILENT	ENSG00000144724:[ENST00000295874(c.543A>G,ccA>ccG):ENSP00000295874(p.Pro181(=))]	NA	NA	NA	6.41E-04	7.93E-04	NA	NA	NA	NA	NA	LOW	NA	NA	62	62	0	71	34	37	52.11
Non-user	IOB_24	chr3	66428234	C	A	SLC25A26	EXONIC-NC	115286:[NR_028475.1_dupl9(n.1109C>A)]	NA	NA	9.78E-03	2.32E-02	1.58E-02	9.78E-03	5.10E-03	NA	NA	NA	MODIFIER	NA	NA	132	132	0	97	53	43	44.33
Non-user	IOB_24	chr3	66430870	G	A	LRIG1	SILENT	ENSG00000144749:[ENST00000273261(c.3099C>T,tcC>tcT):ENSP00000273261(p.Ser1033(=))]	NA	NA	4.45E-02	5.93E-02	7.72E-02	4.45E-02	7.67E-02	NA	NA	NA	MODIFIER	NA	NA	196	194	0	127	59	68	53.54
Non-user	IOB_24	chr3	66430870	G	A	SLC25A26	3UTR	ENSG00000144741:[ENST00000536651(c.*1342G>A)]	NA	NA	4.45E-02	5.93E-02	7.72E-02	4.45E-02	7.67E-02	NA	NA	NA	MODIFIER	NA	NA	196	194	0	127	59	68	53.54
Non-user	IOB_24	chr3	69985838	G	A	MITF	INTRONIC	ENSG00000187098:[ENST00000314589(c.307-1135G>A)]	NA	NA	1.98E-02	1.51E-02	3.73E-02	1.98E-02	3.27E-02	NA	NA	NA	MODIFIER	NA	NA	89	88	0	68	34	33	48.53
Non-user	IOB_24	chr3	69987480	C	T	MITF	INTRONIC	ENSG00000187098:[ENST00000314589(c.534+280C>T)]	NA	NA	1.98E-02	2.17E-02	3.92E-02	1.98E-02	3.27E-02	NA	NA	NA	MODIFIER	NA	NA	50	50	0	48	30	18	37.5
Non-user	IOB_24	chr3	74350557	T	C	CNTN3	MISSENSE	ENSG00000113805:[ENST00000263665(c.2087A>G,gAa>gGa):ENSP00000263665(p.Glu696Gly)]	NA	NA	NA	2.48E-05	1.83E-04	NA	NA	D	BN	NA	MODERATE	D	1	130	130	0	85	44	41	48.24
Non-user	IOB_24	chr3	75713948	C	T	FRG2C	INTRONIC	ENSG00000172969:[ENST00000464571(c.179-25C>T)]	NA	NA	NA	1.00E+00	NA	NA	NA	NA	NA	NA	MODIFIER	NA	NA	88	75	6	70	29	36	51.43
Non-user	IOB_24	chr3	75714379	T	G	FRG2C	INTRONIC	ENSG00000172969:[ENST00000308062(c.334+25T>G)]	NA	NA	NA	NA	NA	NA	NA	NA	NA	NA	MODIFIER	NA	NA	852	749	0	694	404	93	13.4
Non-user	IOB_24	chr3	75781152	A	G	ZNF717	INTRONIC	ENSG00000227124:[ENST00000477374(c.339+59T>C)]	NA	NA	NA	NA	NA	NA	NA	NA	NA	NA	MODIFIER	NA	NA	187	160	6	131	99	24	18.32
Non-user	IOB_24	chr3	75781300	T	C	ZNF717	INTRONIC	100131827:[NM_001324027.1(c.2167-28A>G)]	NA	NA	NA	NA	NA	NA	NA	NA	NA	NA	MODIFIER	NA	NA	208	197	11	135	104	31	22.96
Non-user	IOB_24	chr3	75786483	C	A	ZNF717	MISSENSE	ENSG00000227124:[ENST00000400845(c.2270G>T,gGg>gTg):ENSP00000383643(p.Gly757Val)]	NA	NA	NA	NA	NA	NA	NA	D	PrD	NA	MODERATE	NA	NA	625	454	51	377	201	83	22.02
Non-user	IOB_24	chr3	75788414	G	A	ZNF717	SILENT	ENSG00000227124:[ENST00000478296(c.210C>T,agC>agT):ENSP00000419377(p.Ser70(=))]	NA	NA	NA	NA	NA	NA	NA	NA	NA	NA	LOW	NA	NA	315	312	0	160	132	28	17.5
Non-user	IOB_24	chr3	75790417	A	T	ZNF717	NCT	ENSG00000227124:[ENST00000491507(n.544+10T>A)]	NA	NA	NA	NA	NA	NA	NA	NA	NA	NA	MODIFIER	NA	NA	273	188	0	182	123	6	3.3
Non-user	IOB_24	chr3	75790478	G	T	ZNF717	MISSENSE	ENSG00000227124:[ENST00000471541(c.76C>A,Caa>Aaa):ENSP00000420241(p.Gln26Lys)]	NA	NA	NA	3.14E-02	4.84E-02	NA	NA	T	BN	NA	MODERATE	NA	NA	481	475	0	324	300	24	7.41
Non-user	IOB_24	chr3	93813141	A	G	NSUN3	INTRONIC-SS-DNR-PRX	ENSG00000178694:[ENST00000483378(c.*139+3A>G)]	NA	NA	1.20E-03	2.57E-03	1.32E-04	1.20E-03	0.00E+00	NA	NA	NA	LOW	NA	NA	296	295	1	217	104	113	52.07
Non-user	IOB_24	chr3	97591396	C	G	CRYBG3	MISSENSE	ENSG00000233280:[ENST00000419587(c.1358C>G,aCa>aGa):ENSP00000391551(p.Thr453Arg)]	NA	NA	5.99E-04	1.47E-03	2.37E-03	5.99E-04	3.10E-03	NA	NA	NA	MODIFIER	NA	NA	266	265	0	200	103	97	48.5
Non-user	IOB_24	chr3	98503792	A	G	ST3GAL6	MISSENSE	ENSG00000064225:[ENST00000394162(c.339A>G,atA>atG):ENSP00000377717(p.Ile113Met)]	NA	NA	3.02E-02	4.04E-02	4.73E-02	3.02E-02	5.73E-02	T	BN	NA	MODERATE	NA	0.011	225	225	0	133	66	67	50.38
Non-user	IOB_24	chr3	98512540	G	A	ST3GAL6	MISSENSE	ENSG00000064225:[ENST00000265261(c.577G>A,Gca>Aca):ENSP00000265261(p.Ala193Thr)]	NA	NA	4.63E-02	4.46E-02	4.40E-02	4.63E-02	4.81E-02	T	PoD	NA	MODERATE	D	1	91	91	0	67	34	32	47.76
Non-user	IOB_24	chr3	108298108	T	C	KIAA1524	NCT	ENSG00000163507:[ENST00000487834(n.1087+20A>G)]	NA	NA	3.36E-02	6.27E-02	6.00E-02	3.35E-02	4.70E-02	NA	NA	NA	MODIFIER	NA	NA	54	54	0	51	28	23	45.1
Non-user	IOB_24	chr3	111999618	C	T	SLC9C1	MISSENSE	ENSG00000172139:[ENST00000305815(c.101G>A,cGg>cAg):ENSP00000306627(p.Arg34Gln)]	NA	NA	NA	9.16E-05	6.12E-04	NA	NA	T	BN	NA	MODERATE	N	0.251	32	32	0	21	7	14	66.67
Non-user	IOB_24	chr3	113557820	G	C	GRAMD1C	NCT	ENSG00000178075:[ENST00000463760(n.37+37G>C)]	NA	NA	6.59E-03	3.13E-03	4.53E-02	6.59E-03	3.37E-02	NA	NA	NA	MODIFIER	NA	NA	294	294	0	244	126	118	48.36
Non-user	IOB_24	chr3	118913242	T	G	UPK1B	MISSENSE	ENSG00000114638:[ENST00000497685(c.405T>G,aaT>aaG):ENSP00000418972(p.Asn135Lys)]	NA	NA	NA	9.14E-04	3.10E-04	NA	NA	T	BN	NA	MODERATE	N	0.001	124	123	0	105	59	45	42.86
Non-user	IOB_24	chr3	120169616	A	C	FSTL1	NCT	ENSG00000163430:[ENST00000472536(n.97-23T>G)]	NA	NA	NA	2.50E-05	1.57E-04	NA	NA	NA	NA	NA	MODIFIER	NA	NA	326	323	0	258	146	111	43.02
Non-user	IOB_24	chr3	121151784	G	A	POLQ	MISSENSE	ENSG00000051341:[ENST00000264233(c.7640C>T,gCa>gTa):ENSP00000264233(p.Ala2547Val)]	NA	NA	3.38E-02	2.59E-02	5.95E-02	3.37E-02	7.26E-02	D	PrD	NA	MODERATE	D	1	218	217	0	160	90	69	43.12
Non-user	IOB_24	chr3	124369671	C	T	KALRN	MISSENSE	ENSG00000160145:[ENST00000354186(c.5584C>T,tCa>tTa):ENSP00000346122(p.Ser1862Leu)]	NA	NA	4.39E-03	1.03E-02	5.45E-03	4.39E-03	0.00E+00	T	BN	NA	MODERATE	N	0.998	88	88	0	63	39	23	36.51
Non-user	IOB_24	chr3	126255146	C	T	C3orf22	NCT	152065:[NR_130715.1(n.666-6931G>A)]	NA	NA	6.39E-03	3.46E-03	2.49E-02	6.39E-03	3.27E-02	T	BN	NA	MODERATE	N	0.468	128	127	0	131	75	54	41.22
Non-user	IOB_24	chr3	126255146	C	T	CHST13	MISSENSE	ENSG00000180767:[ENST00000319340(c.130C>T,Ctt>Ttt):ENSP00000317404(p.Leu44Phe)]	NA	NA	6.39E-03	3.46E-03	2.49E-02	6.39E-03	3.27E-02	T	BN	NA	MODERATE	N	0.468	128	127	0	131	75	54	41.22
Non-user	IOB_24	chr3	128618283	T	C	ACAD9	SILENT	ENSG00000177646:[ENST00000308982(c.787T>C,Tta>Cta):ENSP00000312618(p.Leu263(=))]	NA	NA	4.83E-02	2.95E-02	7.49E-02	4.83E-02	9.10E-02	NA	NA	NA	MODIFIER	NA	NA	136	136	0	130	70	59	45.38
Non-user	IOB_24	chr3	130141634	G	A	COL6A5	NCT-SS-PRX	256076:[NR_022012.2(n.5594+8G>A)]	NA	NA	NA	1.55E-04	2.62E-04	NA	NA	NA	NA	NA	LOW	NA	NA	87	87	0	59	34	25	42.37
Non-user	IOB_24	chr3	132211285	C	T	DNAJC13	SILENT	ENSG00000138246:[ENST00000260818(c.3651C>T,gcC>gcT):ENSP00000260818(p.Ala1217(=))]	NA	NA	1.32E-02	1.91E-02	3.62E-02	1.32E-02	2.45E-02	NA	NA	NA	LOW	NA	NA	216	214	0	178	102	75	42.13
Non-user	IOB_24	chr3	133538434	A	G	SRPRB	MISSENSE	ENSG00000144867:[ENST00000466636(c.306A>G,Aca>Gca):ENSP00000417316(p.Thr103Ala)]	NA	NA	NA	NA	NA	NA	NA	NA	NA	NA	MODIFIER	NA	NA	191	191	0	152	69	83	54.61
Non-user	IOB_24	chr3	137989003	C	T	ARMC8	INTRONIC	ENSG00000114098:[ENST00000491704(c.1353+29C>T)]	NA	NA	9.98E-04	4.90E-03	1.73E-03	9.98E-04	2.00E-03	NA	NA	NA	MODIFIER	NA	NA	53	53	0	36	23	13	36.11
Non-user	IOB_24	chr3	137989003	C	T	NME9	INTRONIC	ENSG00000181322:[ENST00000317876(c.746-7575G>A)]	NA	NA	9.98E-04	4.90E-03	1.73E-03	9.98E-04	2.00E-03	NA	NA	NA	MODIFIER	NA	NA	53	53	0	36	23	13	36.11
Non-user	IOB_24	chr3	138724858	T	C	MRPS22	5UTR	ENSG00000175110:[ENST00000495075(c.-222T>C)]	NA	NA	2.48E-02	3.47E-02	1.10E-01	2.48E-02	8.69E-02	T	BN	NA	MODERATE	N	0	101	100	0	87	51	36	41.38
Non-user	IOB_24	chr3	138724858	T	C	PRR23A	MISSENSE	ENSG00000206260:[ENST00000383163(c.253A>G,Acg>Gcg):ENSP00000372649(p.Thr85Ala)]	NA	NA	2.48E-02	3.47E-02	1.10E-01	2.48E-02	8.69E-02	T	BN	NA	MODERATE	N	0	101	100	0	87	51	36	41.38
Non-user	IOB_24	chr3	141724328	T	C	TFDP2	SILENT	ENSG00000114126:[ENST00000467634(c.141A>G,ttA>ttG):ENSP00000419540(p.Leu47(=))]	NA	chr3*141724328*141724328*COSM1039278*T*C*1*endometrium*1	4.13E-02	5.56E-02	8.19E-02	4.13E-02	7.16E-02	NA	NA	NA	MODIFIER	NA	NA	94	92	0	44	18	26	59.09
Non-user	IOB_24	chr3	142116277	T	A	XRN1	MISSENSE	ENSG00000114127:[ENST00000264951(c.2233A>T,Aca>Tca):ENSP00000264951(p.Thr745Ser)]	NA	NA	1.20E-03	5.13E-04	3.66E-03	1.20E-03	6.10E-03	T	BN	NA	MODERATE	NA	NA	80	80	0	47	22	25	53.19
Non-user	IOB_24	chr3	142259898	C	G	ATR	NCT	545:[XR_241498.1(n.3540-22G>C)]	NA	NA	8.39E-03	9.57E-03	2.42E-02	8.39E-03	2.86E-02	NA	NA	NA	MODIFIER	NA	NA	136	135	0	97	54	42	43.3
Non-user	IOB_24	chr3	142281600	C	T	ATR	MISSENSE	ENSG00000175054:[ENST00000350721(c.644G>A,cGt>cAt):ENSP00000343741(p.Arg215His)]	NA	chr3*142281600*142281600*COSM3427168*C*T*1*large_intestine*1	NA	1.24E-04	6.09E-04	NA	NA	NA	NA	NA	MODIFIER	NA	NA	167	166	0	108	45	62	57.41
Non-user	IOB_24	chr3	146254478	C	G	PLSCR1	EXONIC-NC	ENSG00000188313:[ENST00000490745(n.129G>C)]	NA	NA	4.37E-02	NA	NA	4.37E-02	5.83E-02	NA	NA	NA	MODIFIER	NA	NA	195	195	0	149	74	74	49.66
Non-user	IOB_24	chr3	147123199	C	T	ZIC1	INTRONIC	ENSG00000152977:[ENST00000488404(c.49-7106C>T)]	NA	NA	5.19E-03	6.01E-03	1.34E-02	5.19E-03	2.66E-02	NA	NA	NA	MODIFIER	NA	NA	96	96	0	54	27	26	48.15
Non-user	IOB_24	chr3	147123199	C	T	ZIC4	SILENT	ENSG00000174963:[ENST00000425731(c.60G>A,tcG>tcA):ENSP00000397695(p.Ser20(=))]	NA	NA	5.19E-03	6.01E-03	1.34E-02	5.19E-03	2.66E-02	NA	NA	NA	MODIFIER	NA	NA	96	96	0	54	27	26	48.15
Non-user	IOB_24	chr3	148868554	A	G	HPS3	NCT	84343:[XR_241515.1(n.1360+87A>G)]	NA	NA	4.59E-03	NA	NA	4.59E-03	7.20E-03	NA	NA	NA	MODIFIER	NA	NA	43	43	0	36	23	13	36.11
Non-user	IOB_24	chr3	148904282	G	T	CP	NCT	1356:[NR_046371.1(n.2118-1049C>A)]	NA	NA	1.80E-03	1.24E-03	8.91E-03	1.80E-03	9.20E-03	NA	NA	NA	MODIFIER	NA	NA	182	182	0	110	51	58	52.73
Non-user	IOB_24	chr3	148904434	T	G	CP	SILENT	ENSG00000047457:[ENST00000494544(c.1299A>C,ggA>ggC):ENSP00000420545(p.Gly433(=))]	NA	NA	1.96E-02	4.39E-02	3.33E-02	1.96E-02	2.56E-02	NA	NA	NA	MODIFIER	NA	NA	85	85	0	66	40	26	39.39
Non-user	IOB_24	chr3	150332261	A	C	SELT	INTRONIC	ENSG00000198843:[ENST00000477889(c.-257-4235A>C)]	NA	NA	3.24E-02	NA	NA	3.23E-02	3.27E-02	NA	NA	NA	MODIFIER	NA	NA	38	38	0	44	29	14	31.82
Non-user	IOB_24	chr3	151163972	G	C	IGSF10	MISSENSE	ENSG00000152580:[ENST00000282466(c.3797C>G,aCc>aGc):ENSP00000282466(p.Thr1266Ser)]	NA	NA	9.98E-04	1.67E-03	8.48E-04	9.98E-04	0.00E+00	T	BN	NA	MODERATE	N	0	270	268	0	211	100	111	52.61
Non-user	IOB_24	chr3	151986427	C	T	MBNL1	INTRONIC	4154:[XM_005247475.1(c.-103+24198C>T)]	NA	NA	3.59E-03	3.61E-02	4.67E-02	3.59E-03	1.74E-02	NA	NA	NA	MODIFIER	NA	NA	300	297	0	233	137	95	40.77
Non-user	IOB_24	chr3	151986427	C	T	MBNL1-AS1	EXONIC-NC	401093:[NR_027038.1(n.634G>A)]	NA	NA	3.59E-03	3.61E-02	4.67E-02	3.59E-03	1.74E-02	NA	NA	NA	MODIFIER	NA	NA	300	297	0	233	137	95	40.77
Non-user	IOB_24	chr3	155200133	T	C	PLCH1	MISSENSE	ENSG00000114805:[ENST00000340059(c.3706A>G,Atg>Gtg):ENSP00000345988(p.Met1236Val)]	NA	NA	1.92E-02	2.86E-02	5.32E-02	1.92E-02	5.52E-02	NA	NA	NA	MODIFIER	NA	NA	181	180	1	117	48	68	58.12
Non-user	IOB_24	chr3	156139276	G	A	KCNAB1	NCT	ENSG00000169282:[ENST00000478609(n.175-129G>A)]	NA	NA	3.34E-02	NA	NA	3.33E-02	1.29E-01	NA	NA	NA	MODIFIER	NA	NA	168	166	1	131	60	71	54.2
Non-user	IOB_24	chr3	156544303	T	G	LEKR1	INTRONIC	ENSG00000197980:[ENST00000470811(c.-1266+138T>G)]	NA	NA	7.19E-03	NA	NA	7.19E-03	2.04E-02	NA	NA	NA	MODIFIER	NA	NA	56	54	1	48	27	21	43.75
Non-user	IOB_24	chr3	159614478	A	G	IQCJ-SCHIP1	INTRONIC	ENSG00000250588:[ENST00000485419(c.1641-34A>G)]	NA	NA	3.00E-02	4.99E-02	6.16E-02	3.00E-02	4.70E-02	NA	NA	NA	MODIFIER	NA	NA	31	31	0	23	11	12	52.17
Non-user	IOB_24	chr3	159614478	A	G	SCHIP1	INTRONIC	ENSG00000151967:[ENST00000482804(c.732-34A>G)]	NA	NA	3.00E-02	4.99E-02	6.16E-02	3.00E-02	4.70E-02	NA	NA	NA	MODIFIER	NA	NA	31	31	0	23	11	12	52.17
Non-user	IOB_24	chr3	160939777	A	T	NMD3	NCT-SS-PRX	ENSG00000169251:[ENST00000473909(n.45-5A>T)]	NA	NA	2.00E-02	NA	NA	2.00E-02	2.35E-02	NA	NA	NA	MODIFIER	NA	NA	76	66	0	15	9	5	33.33
Non-user	IOB_24	chr3	167508198	G	A	SERPINI1	MISSENSE	ENSG00000163536:[ENST00000295777(c.289G>A,Gta>Ata):ENSP00000295777(p.Val97Ile)]	NA	NA	2.20E-03	4.65E-03	6.97E-03	2.20E-03	3.10E-03	T	BN	NA	MODERATE	NA	NA	105	104	1	73	42	31	42.47
Non-user	IOB_24	chr3	167728054	C	T	GOLIM4	3UTR	ENSG00000173905:[ENST00000309027(c.*3G>A)]	NA	NA	3.40E-02	5.01E-02	2.69E-02	3.39E-02	2.45E-02	NA	NA	NA	MODIFIER	NA	NA	129	129	0	92	42	49	53.26
Non-user	IOB_24	chr3	167742248	G	A	GOLIM4	INTRONIC	ENSG00000173905:[ENST00000309027(c.1776+66C>T)]	NA	NA	2.06E-02	NA	NA	2.06E-02	2.45E-02	NA	NA	NA	MODIFIER	NA	NA	107	106	0	69	33	36	52.17
Non-user	IOB_24	chr3	167747022	C	T	GOLIM4	MISSENSE	ENSG00000173905:[ENST00000309027(c.1418G>A,gGa>gAa):ENSP00000309893(p.Gly473Glu)]	NA	NA	2.68E-02	4.87E-02	3.62E-02	2.68E-02	3.27E-02	T	BN	NA	MODERATE	N	1	217	214	0	181	98	83	45.86
Non-user	IOB_24	chr3	170137144	A	G	CLDN11	INTRONIC	ENSG00000013297:[ENST00000064724(c.226+64A>G)]	NA	NA	1.48E-02	NA	NA	1.48E-02	0.00E+00	NA	NA	NA	MODIFIER	NA	NA	173	172	1	187	90	95	50.8
Non-user	IOB_24	chr3	171455700	C	T	PLD1	MISSENSE	ENSG00000075651:[ENST00000340989(c.142G>A,Gat>Aat):ENSP00000340326(p.Asp48Asn)]	NA	NA	NA	1.65E-05	6.13E-05	NA	NA	NA	NA	NA	MODIFIER	NA	NA	91	91	0	53	30	23	43.4
Non-user	IOB_24	chr3	171510522	A	C	PLD1	INTRONIC	ENSG00000075651:[ENST00000340989(c.-32+17667T>G)]	NA	NA	NA	2.83E-04	5.27E-04	NA	NA	NA	BN	NA	MODERATE	NA	NA	43	43	0	26	13	13	50
Non-user	IOB_24	chr3	171510522	A	C	PP13439	MISSENSE	ENSG00000183657:[ENST00000331659(c.77T>G,aTg>aGg):ENSP00000328422(p.Met26Arg)]	NA	NA	NA	2.83E-04	5.27E-04	NA	NA	NA	BN	NA	MODERATE	NA	NA	43	43	0	26	13	13	50
Non-user	IOB_24	chr3	184157199	T	C	EIF2B5	INTRONIC	ENSG00000145191:[ENST00000444495(c.2107-245075T>C)]	NA	NA	3.99E-04	3.02E-04	5.76E-04	3.99E-04	2.00E-03	NA	NA	NA	MODIFIER	NA	NA	87	87	0	71	47	24	33.8
Non-user	IOB_24	chr3	184157199	T	C	RP11-433C9.2	3UTR	ENSG00000224153:[ENST00000419960(c.*19T>C)]	NA	NA	3.99E-04	3.02E-04	5.76E-04	3.99E-04	2.00E-03	NA	NA	NA	MODIFIER	NA	NA	87	87	0	71	47	24	33.8
Non-user	IOB_24	chr3	185798898	G	A	ETV5	MISSENSE	ENSG00000244405:[ENST00000306376(c.299C>T,tCg>tTg):ENSP00000306894(p.Ser100Leu)]	NA	NA	NA	1.65E-04	1.15E-03	NA	NA	NA	NA	NA	MODERATE	NA	1	104	104	0	91	38	53	58.24
Non-user	IOB_24	chr3	185969613	C	T	DGKG	MISSENSE	ENSG00000058866:[ENST00000265022(c.1696G>A,Gag>Aag):ENSP00000265022(p.Glu566Lys)]	NA	NA	5.99E-04	2.73E-04	2.05E-03	5.99E-04	3.10E-03	NA	NA	NA	MODERATE	NA	0.998	102	102	0	102	54	46	45.1
Non-user	IOB_24	chr3	186503885	A	G	EIF4A2	NCT	ENSG00000156976:[ENST00000468362(n.90+45A>G)]	NA	NA	5.59E-03	6.39E-03	2.26E-02	5.59E-03	2.35E-02	NA	NA	NA	MODIFIER	NA	NA	83	83	0	37	26	11	29.73
Non-user	IOB_24	chr3	186969554	A	C	MASP1	INTRONIC	ENSG00000127241:[ENST00000392472(c.554-14T>G)]	NA	NA	5.59E-03	4.07E-03	1.47E-02	5.59E-03	2.45E-02	NA	NA	NA	MODIFIER	NA	NA	71	71	0	58	31	27	46.55
Non-user	IOB_24	chr3	189033298	C	T	TPRG1	MISSENSE	ENSG00000188001:[ENST00000425670(c.432C>T,gCt>gTt):ENSP00000400171(p.Ala145Val)]	NA	NA	3.18E-02	4.50E-02	2.05E-02	3.17E-02	1.02E-02	NA	NA	NA	MODIFIER	NA	NA	245	242	0	174	92	78	44.83
Non-user	IOB_24	chr3	189604237	A	G	TP63	SILENT	ENSG00000073282:[ENST00000449992(c.867A>G,aaA>aaG):ENSP00000387839(p.Lys289(=))]	NA	NA	NA	1.65E-05	1.21E-04	NA	NA	NA	NA	NA	LOW	NA	1	174	174	0	138	70	68	49.28
Non-user	IOB_24	chr3	190374359	C	T	IL1RAP	MISSENSE	ENSG00000196083:[ENST00000317757(c.2027C>T,aCg>aTg):ENSP00000314807(p.Thr676Met)]	NA	NA	.	NA	NA	NA	NA	D_lc	PrD	NA	MODERATE	NA	NA	143	143	0	98	56	42	42.86
Non-user	IOB_24	chr3	190994615	T	C	UTS2B	MISSENSE	ENSG00000188958:[ENST00000340524(c.221A>G,aAa>aGa):ENSP00000340526(p.Lys74Arg)]	NA	NA	NA	2.50E-05	1.83E-04	NA	NA	NA	NA	NA	MODIFIER	NA	NA	92	92	0	62	23	37	59.68
Non-user	IOB_24	chr3	193128740	A	G	ATP13A4	INTRONIC	ENSG00000127249:[ENST00000428352(c.*458+31T>C)]	NA	NA	NA	6.66E-05	4.88E-04	NA	NA	NA	NA	NA	MODIFIER	NA	NA	52	51	0	53	26	27	50.94

Patel *et al.*, 2021. Whole-exome sequencing analysis of oral squamous cell carcinoma delineated by tobacco usage habits
Supplementary Table 4. List of somatic single nucleotide variants identified using whole exome sequencing of OSCC patients

Cohort	Sample	Chromosome	Genomic position	Reference allele	Alternate allele	Genesymbol	Variant type	Variant description	OncoMD	COSMIC	dbSNP	ExAC	ExAC_SAS	1000G	1000G_SAS	SIFT	Polyphen2	CONDEL	VEP	LRT	PhastCon7Way	Total reads in Normal	Reference read support in Normal	Alternative read support in Normal	Total reads in Tumor	Reference read support in Tumor	Alternate read support in Tumor	Alternate allele frequency in Tumor
Non-user	IOB_24	chr3	194080824	G	A	LRRC15	MISSENSE	ENSG00000172061:[ENST00000347624(c.949C>T,Cgc>Tgc):ENSP00000306276(p.Arg317Cys)]	NA	NA	9.98E-04	3.71E-04	0.00E+00	9.98E-04	0.00E+00	D	BN	NA	MODERATE	N	0.059	213	213	0	179	99	80	44.69
Non-user	IOB_24	chr3	195345758	A	C	AC069213.1	EXONIC-NC	ENSG00000224769:[ENST00000457750(n.334A>C)]	NA	NA	NA	8.75E-03	1.21E-02	NA	NA	NA	NA	NA	MODIFIER	NA	NA	198	198	0	421	344	74	17.58
Non-user	IOB_24	chr3	195345789	G	A	AC069213.1	EXONIC-NC	ENSG00000224769:[ENST00000457750(n.365G>A)]	NA	NA	NA	1.39E-02	2.20E-02	NA	NA	NA	NA	NA	MODIFIER	NA	NA	268	268	0	622	512	109	17.52
Non-user	IOB_24	chr3	195346477	C	T	AC069213.1	EXONIC-NC	ENSG00000224769:[ENST00000457750(n.1053C>T)]	NA	NA	chr3*195346477*195346477*C*G,T*rs7631117*NA*NA*NA	4.36E-01	4.28E-01	NA	NA	NA	NA	NA	MODIFIER	NA	NA	93	93	0	235	151	83	35.32
Non-user	IOB_24	chr3	195395515	A	C	LINC00969	NCT-SS-PRX	ENSG00000242086:[ENST00000539717(n.699+3A>C)]	NA	NA	NA	7.44E-02	1.27E-01	NA	NA	NA	NA	NA	LOW	NA	NA	180	179	1	339	295	43	12.68
Non-user	IOB_24	chr3	195395515	A	C	SDHAP2	NCT-SS-PRX	ENSG00000215837:[ENST00000455183(n.900+3A>C)]	NA	NA	NA	7.44E-02	1.27E-01	NA	NA	NA	NA	NA	LOW	NA	NA	180	179	1	339	295	43	12.68
Non-user	IOB_24	chr3	195395564	C	T	LINC00969	NCT	ENSG00000242086:[ENST00000453324(n.779+52C>T)]	NA	NA	NA	NA	NA	NA	NA	NA	NA	NA	MODIFIER	NA	NA	138	138	0	270	237	30	11.11
Non-user	IOB_24	chr3	195395564	C	T	SDHAP2	NCT	727956:[NR_003265.3(n.957+52C>T)]	NA	NA	NA	NA	NA	NA	NA	NA	NA	NA	MODIFIER	NA	NA	138	138	0	270	237	30	11.11
Non-user	IOB_24	chr3	195398105	T	C	LINC00969	EXONIC-NC	ENSG00000242086:[ENST00000429897(n.890T>C)]	NA	NA	NA	3.88E-01	3.85E-01	NA	NA	NA	NA	NA	MODIFIER	NA	NA	123	123	0	196	164	29	14.8
Non-user	IOB_24	chr3	195398105	T	C	SDHAP2	EXONIC-NC	ENSG00000215837:[ENST00000455183(n.1003T>C)]	NA	NA	NA	3.88E-01	3.85E-01	NA	NA	NA	NA	NA	MODIFIER	NA	NA	123	123	0	196	164	29	14.8
Non-user	IOB_24	chr3	195400822	G	A	LINC00969	EXONIC-NC	ENSG00000242086:[ENST00000429897(n.1286G>A)]	NA	NA	NA	7.33E-02	1.38E-01	NA	NA	NA	NA	NA	MODIFIER	NA	NA	129	129	0	134	119	13	9.7
Non-user	IOB_24	chr3	195400822	G	A	SDHAP2	EXONIC-NC	ENSG00000215837:[ENST00000455183(n.1399G>A)]	NA	NA	NA	7.33E-02	1.38E-01	NA	NA	NA	NA	NA	MODIFIER	NA	NA	129	129	0	134	119	13	9.7
Non-user	IOB_24	chr3	195400841	C	A	LINC00969	EXONIC-NC	ENSG00000242086:[ENST00000446521(n.226C>A)]	NA	NA	NA	1.21E-01	2.32E-01	NA	NA	NA	NA	NA	MODIFIER	NA	NA	117	117	0	122	106	12	9.84
Non-user	IOB_24	chr3	195400841	C	A	SDHAP2	EXONIC-NC	ENSG00000215837:[ENST00000455183(n.1418C>A)]	NA	NA	NA	1.21E-01	2.32E-01	NA	NA	NA	NA	NA	MODIFIER	NA	NA	117	117	0	122	106	12	9.84
Non-user	IOB_24	chr3	195400848	G	A	LINC00969	EXONIC-NC	ENSG00000242086:[ENST00000429897(n.1312G>A)]	NA	NA	NA	1.31E-01	2.49E-01	NA	NA	NA	NA	NA	MODIFIER	NA	NA	110	110	0	114	97	14	12.28
Non-user	IOB_24	chr3	195400848	G	A	SDHAP2	EXONIC-NC	727956:[NR_003265.3(n.1482G>A)]	NA	NA	NA	1.31E-01	2.49E-01	NA	NA	NA	NA	NA	MODIFIER	NA	NA	110	110	0	114	97	14	12.28
Non-user	IOB_24	chr3	195412695	G	C	LINC00969	EXONIC-NC	ENSG00000242086:[ENST00000608699(n.96G>C)]	NA	NA	NA	NA	NA	NA	NA	NA	NA	NA	MODIFIER	NA	NA	241	239	0	175	143	30	17.14
Non-user	IOB_24	chr3	195412695	G	C	SDHAP2	NCT	727956:[NR_003265.3(n.1971+1959G>C)]	NA	NA	NA	NA	NA	NA	NA	NA	NA	NA	MODIFIER	NA	NA	241	239	0	175	143	30	17.14
Non-user	IOB_24	chr3	195412715	T	C	LINC00969	EXONIC-NC	ENSG00000242086:[ENST00000608699(n.116T>C)]	NA	NA	NA	NA	NA	NA	NA	NA	NA	NA	MODIFIER	NA	NA	235	232	3	173	141	29	16.76
Non-user	IOB_24	chr3	195412715	T	C	SDHAP2	NCT	727956:[NR_003265.3(n.1971+1979T>C)]	NA	NA	NA	NA	NA	NA	NA	NA	NA	NA	MODIFIER	NA	NA	235	232	3	173	141	29	16.76
Non-user	IOB_24	chr3	195447886	G	C	LINC00969	NCT	ENSG00000242086:[ENST00000595086(n.211-8633G>C)]	NA	NA	NA	4.86E-01	4.77E-01	NA	NA	NA	NA	NA	MODIFIER	NA	NA	13	13	0	29	13	16	55.17
Non-user	IOB_24	chr3	195447886	G	C	MUC20	MISSENSE	ENSG00000176945:[ENST00000320736(c.8G>C,tGt>tCt):ENSP00000325431(p.Cys3Ser)]	NA	NA	NA	4.86E-01	4.77E-01	NA	NA	NA	NA	NA	MODIFIER	NA	NA	13	13	0	29	13	16	55.17
Non-user	IOB_24	chr3	195452619	C	T	LINC00969	NCT	ENSG00000242086:[ENST00000600197(n.689-7402C>T)]	NA	chr3*195452619*195452619*COSM479880*C*T*1*kidney*1	NA	1.46E-01	4.21E-02	NA	NA	NA	NA	NA	MODIFIER	NA	NA	100	99	0	104	87	17	16.35
Non-user	IOB_24	chr3	195452619	C	T	MUC20	MISSENSE	ENSG00000176945:[ENST00000320736(c.632C>T,gCc>gTc):ENSP00000325431(p.Ala211Val)]	NA	chr3*195452619*195452619*COSM479880*C*T*1*kidney*1	NA	1.46E-01	4.21E-02	NA	NA	NA	NA	NA	MODIFIER	NA	NA	100	99	0	104	87	17	16.35
Non-user	IOB_24	chr3	195452645	G	A	LINC00969	NCT	ENSG00000242086:[ENST00000600197(n.689-7376G>A)]	NA	chr3*195452645*195452645*COSM479881*G*A*2*kidney,breast*1,1	NA	1.11E-01	2.41E-02	NA	NA	NA	NA	NA	MODIFIER	NA	NA	123	121	0	120	98	19	15.83
Non-user	IOB_24	chr3	195452645	G	A	MUC20	MISSENSE	ENSG00000176945:[ENST00000445522(c.1066G>A,Ggc>Agc):ENSP00000405629(p.Gly356Ser)]	NA	chr3*195452645*195452645*COSM479881*G*A*2*kidney,breast*1,1	NA	1.11E-01	2.41E-02	NA	NA	NA	NA	NA	MODIFIER	NA	NA	123	121	0	120	98	19	15.83
Non-user	IOB_24	chr3	195452649	C	T	LINC00969	NCT	ENSG00000242086:[ENST00000600197(n.689-7372C>T)]	NA	chr3*195452649*195452649*COSM1484986*C*T*4*breast*4	NA	5.62E-03	2.08E-03	NA	NA	NA	NA	NA	MODIFIER	NA	NA	126	122	2	121	87	30	24.79
Non-user	IOB_24	chr3	195452649	C	T	MUC20	MISSENSE	ENSG00000176945:[ENST00000320736(c.662C>T,cCc>cTc):ENSP00000325431(p.Pro221Leu)]	NA	chr3*195452649*195452649*COSM1484986*C*T*4*breast*4	NA	5.62E-03	2.08E-03	NA	NA	NA	NA	NA	MODIFIER	NA	NA	126	122	2	121	87	30	24.79
Non-user	IOB_24	chr3	195452951	G	C	LINC00969	NCT	ENSG00000242086:[ENST00000597662(n.378-3263G>C)]	NA	chr3*195452951*195452951*COSM446212*G*C*1*breast*1	NA	6.09E-01	6.14E-01	NA	NA	T	PoD	NA	MODERATE	N	0.001	175	175	0	273	80	190	69.6
Non-user	IOB_24	chr3	195452951	G	C	MUC20	MISSENSE	ENSG00000176945:[ENST00000436408(c.1477G>C,Gat>Cat):ENSP00000396774(p.Asp493His)]	NA	chr3*195452951*195452951*COSM446212*G*C*1*breast*1	NA	6.09E-01	6.14E-01	NA	NA	T	PoD	NA	MODERATE	N	0.001	175	175	0	273	80	190	69.6
Non-user	IOB_24	chr3	195452987	G	A	LINC00969	NCT	ENSG00000242086:[ENST00000597662(n.378-3227G>A)]	NA	NA	NA	2.08E-01	2.92E-01	NA	NA	T	BN	NA	MODERATE	NA	0	189	185	4	348	253	89	25.57
Non-user	IOB_24	chr3	195452987	G	A	MUC20	MISSENSE	ENSG00000176945:[ENST00000320736(c.1000G>A,Gcc>Acc):ENSP00000325431(p.Ala334Thr)]	NA	NA	NA	2.08E-01	2.92E-01	NA	NA	T	BN	NA	MODERATE	NA	0	189	185	4	348	253	89	25.57
Non-user	IOB_24	chr3	195452991	C	T	LINC00969	NCT	ENSG00000242086:[ENST00000597662(n.378-3223C>T)]	NA	NA	chr3*195452991*195452991*C*A,T*rs3828411*NA*NA*NA	2.05E-01	2.79E-01	NA	NA	NA	NA	NA	MODIFIER	NA	NA	190	187	2	346	250	90	26.01
Non-user	IOB_24	chr3	195452991	C	T	MUC20	MISSENSE	ENSG00000176945:[ENST00000445522(c.1412C>T,aCa>aTa):ENSP00000405629(p.Thr471Ile)]	NA	NA	chr3*195452991*195452991*C*A,T*rs3828411*NA*NA*NA	2.05E-01	2.79E-01	NA	NA	NA	NA	NA	MODIFIER	NA	NA	190	187	2	346	250	90	26.01
Non-user	IOB_24	chr3	195453064	T	G	LINC00969	NCT	ENSG00000242086:[ENST00000597662(n.378-3150T>G)]	NA	NA	NA	8.21E-01	7.16E-01	NA	NA	NA	NA	NA	MODIFIER	NA	NA	149	142	6	278	98	179	64.39
Non-user	IOB_24	chr3	195453064	T	G	MUC20	SILENT	ENSG00000176945:[ENST00000320736(c.1077T>G,ctT>ctG):ENSP00000325431(p.Leu359(=))]	NA	NA	NA	8.21E-01	7.16E-01	NA	NA	NA	NA	NA	MODIFIER	NA	NA	149	142	6	278	98	179	64.39
Non-user	IOB_24	chr3	195453205	G	A	LINC00969	NCT	ENSG00000242086:[ENST00000600197(n.689-6816G>A)]	NA	NA	NA	1.14E-01	2.07E-01	NA	NA	NA	NA	NA	LOW	NA	NA	178	177	0	146	92	54	36.99
Non-user	IOB_24	chr3	195453205	G	A	MUC20	SILENT	ENSG00000176945:[ENST00000320736(c.1218G>A,tcG>tcA):ENSP00000325431(p.Ser406(=))]	NA	NA	NA	1.14E-01	2.07E-01	NA	NA	NA	NA	NA	LOW	NA	NA	178	177	0	146	92	54	36.99
Non-user	IOB_24	chr3	195453257	G	A	LINC00969	NCT	ENSG00000242086:[ENST00000600197(n.689-6764G>A)]	NA	NA	NA	3.71E-01	4.11E-01	NA	NA	NA	NA	NA	MODIFIER	NA	NA	215	210	2	199	69	128	64.32
Non-user	IOB_24	chr3	195453257	G	A	MUC20	MISSENSE	ENSG00000176945:[ENST00000320736(c.1270G>A,Gca>Aca):ENSP00000325431(p.Ala424Thr)]	NA	NA	NA	3.71E-01	4.11E-01	NA	NA	NA	NA	NA	MODIFIER	NA	NA	215	210	2	199	69	128	64.32
Non-user	IOB_24	chr3	195453406	G	C	LINC00969	NCT	ENSG00000242086:[ENST00000594446(n.211-2808G>C)]	NA	chr3*195453406*195453406*COSM1633067*G*C*1*liver*1|chr3*195453406*195453406*COSM1633066*G*C*1*liver*1	chr3*195453406*195453406*G*A,C*rs3210209*NA*NA*NA	1.60E-01	2.79E-01	NA	NA	NA	NA	NA	LOW	NA	NA	201	200	1	198	142	55	27.78
Non-user	IOB_24	chr3	195453406	G	C	MUC20	SILENT	ENSG00000176945:[ENST00000423938(c.166G>C,acG>acC):ENSP00000397774(p.Thr56(=))]	NA	chr3*195453406*195453406*COSM1633067*G*C*1*liver*1|chr3*195453406*195453406*COSM1633066*G*C*1*liver*1	chr3*195453406*195453406*G*A,C*rs3210209*NA*NA*NA	1.60E-01	2.79E-01	NA	NA	NA	NA	NA	LOW	NA	NA	201	200	1	198	142	55	27.78
Non-user	IOB_24	chr3	195453412	C	T	LINC00969	NCT	ENSG00000242086:[ENST00000594446(n.211-2802C>T)]	NA	chr3*195453412*195453412*COSM149550*C*T*1*stomach*1	NA	1.57E-01	2.78E-01	NA	NA	NA	NA	NA	LOW	NA	NA	199	198	1	195	138	56	28.72
Non-user	IOB_24	chr3	195453412	C	T	MUC20	SILENT	ENSG00000176945:[ENST00000320736(c.1425C>T,gcC>gcT):ENSP00000325431(p.Ala475(=))]	NA	chr3*195453412*195453412*COSM149550*C*T*1*stomach*1	NA	1.57E-01	2.78E-01	NA	NA	NA	NA	NA	LOW	NA	NA	199	198	1	195	138	56	28.72
Non-user	IOB_24	chr3	195456561	C	G	LINC00969	EXONIC-NC	ENSG00000242086:[ENST00000594446(n.462C>G)]	NA	NA	chr3*195456561*195456561*C*A,G,T*rs3762739*NA*NA*NA	3.06E-01	3.46E-01	NA	NA	D	PrD	NA	MODERATE	NA	NA	70	70	0	88	59	29	32.95
Non-user	IOB_24	chr3	195456561	C	G	MUC20	MISSENSE	ENSG00000176945:[ENST00000320736(c.1499C>G,tCc>tGc):ENSP00000325431(p.Ser500Cys)]	NA	NA	chr3*195456561*195456561*C*A,G,T*rs3762739*NA*NA*NA	3.06E-01	3.46E-01	NA	NA	D	PrD	NA	MODERATE	NA	NA	70	70	0	88	59	29	32.95
Non-user	IOB_24	chr3	195475989	G	A	MUC4	INTRONIC	ENSG00000145113:[ENST00000463781(c.15872-54C>T)]	NA	NA	NA	NA	NA	NA	NA	NA	NA	NA	MODIFIER	NA	NA	54	54	0	102	59	41	40.2
Non-user	IOB_24	chr3	195597083	G	A	TNK2	EXONIC-NC	ENSG00000061938:[ENST00000468680(n.698C>T)]	NA	NA	2.00E-04	2.72E-04	4.27E-04	2.00E-04	0.00E+00	NA	NA	NA	LOW	NA	NA	157	153	0	141	58	81	57.45
Non-user	IOB_24	chr3	195686891	T	C	SDHAP1	EXONIC-NC	ENSG00000185485:[ENST00000427841(n.2392A>G)]	NA	NA	NA	NA	NA	NA	NA	NA	NA	NA	MODIFIER	NA	NA	25	25	0	33	20	12	36.36
Non-user	IOB_24	chr3	195686899	C	T	SDHAP1	EXONIC-NC	ENSG00000185485:[ENST00000427149(n.426G>A)]	NA	NA	NA	NA	NA	NA	NA	NA	NA	NA	MODIFIER	NA	NA	24	24	0	32	24	6	18.75
Non-user	IOB_24	chr3	195690216	T	C	SDHAP1	EXONIC-NC	ENSG00000185485:[ENST00000440850(n.1990A>G)]	NA	NA	NA	2.64E-02	1.52E-02	NA	NA	NA	NA	NA	MODIFIER	NA	NA	175	175	0	210	152	58	27.62
Non-user	IOB_24	chr3	195690233	A	G	SDHAP1	EXONIC-NC	ENSG00000185485:[ENST00000440850(n.1973T>C)]	NA	NA	NA	2.46E-03	6.27E-04	NA	NA	NA	NA	NA	MODIFIER	NA	NA	201	199	1	230	197	32	13.91
Non-user	IOB_24	chr3	195690251	A	G	SDHAP1	EXONIC-NC	ENSG00000185485:[ENST00000354559(n.339T>C)]	NA	NA	NA	4.03E-02	4.75E-02	NA	NA	NA	NA	NA	MODIFIER	NA	NA	216	214	0	254	184	70	27.56
Non-user	IOB_24	chr3	195690286	C	G	SDHAP1	EXONIC-NC	ENSG00000185485:[ENST00000427149(n.128G>C)]	NA	NA	NA	2.50E-01	1.91E-01	NA	NA	NA	NA	NA	MODIFIER	NA	NA	208	207	0	252	219	32	12.7
Non-user	IOB_24	chr3	195692419	C	G	SDHAP1	NCT-SS-PRX	ENSG00000185485:[ENST00000440850(n.1800-6G>C)]	NA	NA	NA	3.56E-01	3.81E-01	NA	NA	NA	NA	NA	LOW	NA	NA	53	53	0	53	40	13	24.53
Non-user	IOB_24	chr3	195701278	C	T	SDHAP1	EXONIC-NC	ENSG00000185485:[ENST00000440850(n.1424G>A)]	NA	NA	NA	1.32E-01	1.52E-01	NA	NA	NA	NA	NA	MODIFIER	NA	NA	169	166	2	233	184	49	21.03
Non-user	IOB_24	chr3	195701285	G	T	SDHAP1	EXONIC-NC	ENSG00000185485:[ENST00000354937(n.1474C>A)]	NA	NA	NA	1.14E-01	1.34E-01	NA	NA	NA	NA	NA	MODIFIER	NA	NA	184	183	0	238	189	48	20.17
Non-user	IOB_24	chr3	195701304	C	T	SDHAP1	EXONIC-NC-SS-PRX	ENSG00000185485:[ENST00000354937(n.1455G>A)]	NA	NA	NA	9.86E-02	1.18E-01	NA	NA	NA	NA	NA	MODIFIER	NA	NA	196	196	0	267	208	54	20.22
Non-user	IOB_24	chr3	195701331	G	A	SDHAP1	EXONIC-NC	ENSG00000185485:[ENST00000354937(n.1429C>T)]	NA	NA	NA	2.61E-01	3.10E-01	NA	NA	NA	NA	NA	MODIFIER	NA	NA	197	197	0	279	212	60	21.51
Non-user	IOB_24	chr3	195701338	C	T	SDHAP1	EXONIC-NC	ENSG00000185485:[ENST00000354937(n.1422G>A)]	NA	NA	.	1.66E-02	3.74E-02	NA	NA	NA	NA	NA	MODIFIER	NA	NA	190	189	1	276	243	27	9.78
Non-user	IOB_24	chr3	195702639	C	T	SDHAP1	EXONIC-NC	ENSG00000185485:[ENST00000538515(n.841G>A)]	NA	NA	chr3*195702639*195702639*C*A,T*rs201406351*NA*NA*NA	6.98E-02	7.00E-02	NA	NA	NA	NA	NA	MODIFIER	NA	NA	104	104	0	160	122	36	22.5
Non-user	IOB_24	chr3	195702738	C	T	SDHAP1	EXONIC-NC	ENSG00000185485:[ENST00000545003(n.902G>A)]	NA	NA	NA	1.37E-01	2.18E-01	NA	NA	NA	NA	NA	MODIFIER	NA	NA	100	98	1	156	119	35	22.44
Non-user	IOB_24	chr3	195704020	A	G	SDHAP1	EXONIC-NC	ENSG00000185485:[ENST00000538515(n.642T>C)]	NA	NA	NA	2.18E-01	1.40E-01	NA	NA	NA	NA	NA	MODIFIER	NA	NA	118	117	0	105	62	41	39.05
Non-user	IOB_24	chr3	195710948	G	A	SDHAP1	NCT	ENSG00000185485:[ENST00000427841(n.750+35C>T)]	NA	NA	NA	NA	NA	NA	NA	NA	NA	NA	MODIFIER	NA	NA	68	68	0	92	78	14	15.22
Non-user	IOB_24	chr3	195710971	G	T	SDHAP1	NCT	ENSG00000185485:[ENST00000413474(n.500+12C>A)]	NA	NA	NA	NA	NA	NA	NA	NA	NA	NA	MODIFIER	NA	NA	81	81	0	110	75	35	31.82
Non-user	IOB_24	chr3	196096271	G	A	UBXN7	INTRONIC	ENSG00000163960:[ENST00000429160(c.*330+21C>T)]	NA	NA	4.57E-02	6.81E-02	1.30E-01	4.57E-02	1.29E-01	NA	NA	NA	MODIFIER	NA	NA	67	67	0	52	25	27	51.92
Non-user	IOB_24	chr3	196235107	A	G	SMCO1	MISSENSE	ENSG00000214097:[ENST00000397537(c.296T>C,gTg>gCg):ENSP00000380671(p.Val99Ala)]	NA	NA	2.48E-02	1.37E-02	9.34E-02	2.48E-02	1.07E-01	NA	NA	NA	MODERATE	NA	NA	187	186	0	114	59	54	47.37
Non-user	IOB_24	chr3	196236453	A	G	SMCO1	SILENT	ENSG00000214097:[ENST00000397537(c.138T>C,caT>caC):ENSP00000380671(p.His46(=))]	NA	NA	NA	NA	NA	NA	NA	NA	NA	NA	LOW	NA	NA	201	201	0	162	82	80	49.38
Non-user	IOB_24	chr3	196381434	C	T	NRROS	SILENT	ENSG00000174004:[ENST00000328557(c.24C>T,ctC>ctT):ENSP00000328625(p.Leu8(=))]	NA	NA	1.22E-02	9.27E-03	5.34E-02	1.22E-02	5.93E-02	NA	NA	NA	LOW	NA	NA	104	103	0	89	53	36	40.45
Non-user	IOB_24	chr3	196381434	C	T	PIGX	5UTR	ENSG00000163964:[ENST00000426755(c.-96C>T)]	NA	NA	1.22E-02	9.27E-03	5.34E-02	1.22E-02	5.93E-02	NA	NA	NA	LOW	NA	NA	104	103	0	89	53	36	40.45
Non-user	IOB_24	chr3	196438776	C	G	CEP19	INTRONIC	ENSG00000174007:[ENST00000409690(c.-59+24G>C)]	NA	NA	NA	NA	NA	NA	NA	NA	NA	NA	MODIFIER	NA	NA	147	147	0	62	43	19	30.65
Non-user	IOB_24	chr3	196438776	C	G	PIGX	INTRONIC	ENSG00000163964:[ENST00000426755(c.-11-4953C>G)]	NA	NA	NA	NA	NA	NA	NA	NA	NA	NA	MODIFIER	NA	NA	147	147	0	62	43	19	30.65
Non-user	IOB_24	chr3	196663781	A	C	NCBP2	NCT	ENSG00000114503:[ENST00000467803(n.676+105T>G)]	NA	NA	3.00E-03	NA	NA	3.00E-03	9.20E-03	NA	NA	NA	MODIFIER	NA	NA	80	79	0	70	40	30	42.86
Non-user	IOB_24	chr4	59469	A	G	ZNF595	NCT	ENSG00000197701:[ENST00000339368(n.334+20A>G)]	NA	NA	NA	9.49E-02	1.37E-01	NA	NA	NA	NA	NA	MODIFIER	NA	NA	881	870	1	742	614	113	15.23
Non-user	IOB_24	chr4	60371	G	A	ZNF595	INTRONIC	152687:[NM_001286054.1(c.-425+325G>A)]	NA	NA	NA	NA	NA	NA	NA	NA	NA	NA	MODIFIER	NA	NA	281	279	0	175	170	5	2.86
Non-user	IOB_24	chr4	338129	G	A	ZNF141	MISSENSE	ENSG00000131127:[ENST00000240499(c.136G>A,Gct>Act):ENSP00000240499(p.Ala46Thr)]	NA	NA	NA	1.75E-02	1.50E-02	NA	NA	NA	NA	NA	MODIFIER	NA	NA	78	72	0	74	57	15	20.27
Non-user	IOB_24	chr4	509709	A	G	PIGG	INTRONIC	ENSG00000174227:[ENST00000310340(c.902-53A>G)]	NA	NA	5.99E-04	NA	NA	5.99E-04	2.00E-03	NA	NA	NA	MODIFIER	NA	NA	123	123	0	74	33	41	55.41
Non-user	IOB_24	chr4	1348987	A	G	UVSSA	MISSENSE	ENSG00000163945:[ENST00000389851(c.1130A>G,aAa>aGa):ENSP00000374501(p.Lys377Arg)]	NA	NA	3.99E-04	9.73E-04	5.90E-03	3.99E-04	1.00E-03	NA	NA	NA	MODIFIER	NA	NA	221	217	0	218	110	105	48.17

Patel *et al.* , 2021. Whole-exome sequencing analysis of oral squamous cell carcinoma delineated by tobacco usage habits
Supplementary Table 4. List of somatic single nucleotide variants identified using whole exome sequencing of OSCC patients

Cohort	Sample	Chromosome	Genomic position	Reference allele	Alternate allele	Genesymbol	Variant type	Variant description	OncoMD	COSMIC	dbSNP	ExAC	ExAC_SAS	1000G	1000G_SAS	SIFT	Polyphen2	CONDEL	VEP	LRT	PhastCon7Way	Total reads in Normal	Reference read support in Normal	Alternative read support in Normal	Total reads in Tumor	Reference read support in Tumor	Alternate read support in Tumor	Alternate allele frequency in Tumor
Non-user	IOB_24	chr4	2967133	G	A	GRK4	MISSENSE	ENSG00000125388:[ENST00000442472(c.304G>A,Ggt>Agt):ENSP00000392297(p.Gly102Ser)]	NA	NA	4.79E-03	8.68E-03	8.27E-03	4.79E-03	7.20E-03	NA	NA	NA	MODIFIER	NA	NA	279	278	0	207	104	102	49.28
Non-user	IOB_24	chr4	2988863	A	G	GRK4	INTRONIC	ENSG00000125388:[ENST00000398052(c.149-1591A>G)]	NA	NA	9.98E-04	8.57E-04	1.76E-03	9.98E-04	3.10E-03	NA	NA	NA	MODIFIER	NA	NA	227	224	1	172	106	65	37.79
Non-user	IOB_24	chr4	4436517	A	T	STX18	MISSENSE	ENSG00000168818:[ENST00000507908(c.439T>A,Tcc>Acc):ENSP00000422376(p.Ser147Thr)]	NA	NA	4.51E-02	4.57E-02	4.78E-02	4.51E-02	2.97E-02	NA	NA	NA	MODIFIER	NA	NA	21	21	0	28	12	16	57.14
Non-user	IOB_24	chr4	4440183	T	G	STX18	MISSENSE	ENSG00000168818:[ENST00000306200(c.551A>C,gAg>gCg):ENSP00000305810(p.Glu184Ala)]	NA	NA	3.83E-02	4.29E-02	4.67E-02	3.83E-02	2.97E-02	NA	NA	NA	MODIFIER	NA	NA	73	73	0	49	23	25	51.02
Non-user	IOB_24	chr4	5016972	T	C	CYTL1	INTRONIC	ENSG00000170891:[ENST00000509419(c.195-11A>G)]	NA	NA	2.34E-02	3.07E-02	5.07E-02	2.34E-02	5.73E-02	NA	NA	NA	MODIFIER	NA	NA	84	84	0	68	35	33	48.53
Non-user	IOB_24	chr4	5142012	A	G	STK32B	3UTR	ENSG00000152953:[ENST00000512018(c.*41A>G)]	NA	NA	3.20E-03	1.68E-02	2.34E-02	3.19E-03	1.33E-02	NA	NA	NA	MODIFIER	NA	NA	93	93	0	58	37	21	36.21
Non-user	IOB_24	chr4	5448297	A	C	STK32B	INTRONIC	55351:[NM_001345969.1(c.473-103A>C)]	NA	NA	2.40E-03	NA	NA	2.40E-03	4.10E-03	NA	NA	NA	MODIFIER	NA	NA	98	92	0	81	38	38	46.91
Non-user	IOB_24	chr4	5991429	C	T	C4orf50	MISSENSE	ENSG00000181215:[ENST00000531445(c.70G>A,Gaa>Aaa):ENSP00000437121(p.Glu24Lys)]	NA	NA	3.34E-02	7.18E-02	8.97E-02	3.33E-02	7.26E-02	T	PoD	NA	MODERATE	NA	NA	295	292	1	216	95	119	55.09
Non-user	IOB_24	chr4	6050572	G	A	JAKMIP1	INTRONIC	ENSG00000152969:[ENST00000409371(c.1473+12C>T)]	NA	NA	3.99E-04	1.43E-03	7.92E-04	3.99E-04	0.00E+00	NA	NA	NA	MODIFIER	NA	NA	119	119	0	108	54	54	50
Non-user	IOB_24	chr4	6374263	G	A	PPP2R2C	SILENT	ENSG00000074211:[ENST00000335585(c.612C>T,acC>acT):ENSP00000335083(p.Thr204(=))]	NA	NA	3.06E-02	8.80E-03	7.21E-03	3.06E-02	7.20E-03	NA	NA	NA	MODIFIER	NA	NA	193	193	0	159	83	76	47.8
Non-user	IOB_24	chr4	6610889	G	A	MAN2B2	MISSENSE	ENSG00000013288:[ENST00000285599(c.1870G>A,Ggg>Agg):ENSP00000285599(p.Gly624Arg)]	NA	NA	3.61E-02	6.68E-02	4.57E-02	3.61E-02	5.73E-02	NA	NA	NA	MODIFIER	NA	NA	205	203	0	179	98	81	45.25
Non-user	IOB_24	chr4	7044189	C	T	CCDC96	SILENT	ENSG00000173013:[ENST00000310085(c.477G>A,gaG>gaA):ENSP00000309285(p.Glu159(=))]	NA	NA	1.20E-03	6.78E-04	4.76E-03	1.20E-03	6.10E-03	NA	NA	NA	LOW	NA	0	239	238	0	231	113	118	51.08
Non-user	IOB_24	chr4	7044189	C	T	RP11-367J11.2	NCT	ENSG00000245748:[ENST00000500031(n.696+2518G>A)]	NA	NA	1.20E-03	6.78E-04	4.76E-03	1.20E-03	6.10E-03	NA	NA	NA	LOW	NA	0	239	238	0	231	113	118	51.08
Non-user	IOB_24	chr4	7044189	C	T	TADA2B	INTRONIC	ENSG00000173011:[ENST00000506692(c.-7+376C>T)]	NA	NA	1.20E-03	6.78E-04	4.76E-03	1.20E-03	6.10E-03	NA	NA	NA	LOW	NA	0	239	238	0	231	113	118	51.08
Non-user	IOB_24	chr4	9370809	G	C	NA	INTERGENIC	NA	NA	NA	2.00E-04	1.22E-03	5.34E-03	2.00E-04	1.00E-03	NA	NA	NA	MODIFIER	NA	NA	48	48	0	38	19	18	47.37
Non-user	IOB_24	chr4	9909922	C	T	SLC2A9	SILENT	ENSG00000109667:[ENST00000264784(c.1050G>A,ccG>ccA):ENSP00000264784(p.Pro350(=))]	NA	NA	3.20E-03	1.29E-03	9.21E-03	3.19E-03	1.64E-02	NA	NA	NA	MODIFIER	NA	NA	99	98	0	85	38	47	55.29
Non-user	IOB_24	chr4	10560112	T	C	CLNK	INTRONIC	ENSG00000109684:[ENST00000442825(c.274-36A>G)]	NA	NA	1.20E-03	6.54E-04	4.73E-03	1.20E-03	6.10E-03	NA	NA	NA	MODIFIER	NA	NA	528	528	0	376	167	206	54.79
Non-user	IOB_24	chr4	13604027	G	A	BOD1L1	SILENT	ENSG00000038219:[ENST00000040738(c.4497C>T,aaC>aaT):ENSP00000040738(p.Asn1499(=))]	NA	NA	5.79E-03	1.15E-02	7.66E-03	5.79E-03	5.10E-03	NA	NA	NA	LOW	NA	0	124	123	0	103	54	48	46.6
Non-user	IOB_24	chr4	13605040	C	T	BOD1L1	MISSENSE	ENSG00000038219:[ENST00000040738(c.3484G>A,Gtt>Att):ENSP00000040738(p.Val1162Ile)]	NA	NA	7.99E-03	1.48E-02	8.54E-03	7.99E-03	6.10E-03	T	BN	NA	MODERATE	N	0	263	262	0	204	96	107	52.45
Non-user	IOB_24	chr4	15981651	T	C	PROM1	INTRONIC	ENSG00000007062:[ENST00000503884(c.158-124A>G)]	NA	NA	8.59E-03	NA	NA	8.59E-03	1.02E-02	NA	NA	NA	MODIFIER	NA	NA	11	11	0	14	6	8	57.14
Non-user	IOB_24	chr4	17488905	T	A	QDPR	INTRONIC	ENSG00000151552:[ENST00000428702(c.537-46A>T)]	NA	NA	3.36E-02	4.22E-02	4.39E-02	3.35E-02	3.78E-02	NA	NA	NA	MODIFIER	NA	NA	94	90	0	75	33	36	48
Non-user	IOB_24	chr4	17506042	G	A	QDPR	SILENT	ENSG00000151552:[ENST00000281243(c.255C>T,tgC>tgT):ENSP00000281243(p.Cys85(=))]	NA	NA	3.30E-02	4.05E-02	3.97E-02	3.29E-02	3.48E-02	NA	NA	NA	LOW	NA	NA	196	195	0	144	82	62	43.06
Non-user	IOB_24	chr4	17690072	T	C	FAM184B	MISSENSE	ENSG00000047662:[ENST00000265018(c.1571A>G,cAg>cGg):ENSP00000265018(p.Gln524Arg)]	NA	NA	1.62E-02	3.77E-02	5.88E-02	1.62E-02	4.60E-02	T	PoD	NA	MODERATE	N	0	53	53	0	27	16	11	40.74
Non-user	IOB_24	chr4	25915826	G	A	SMIM20	MISSENSE	389203:[XM_005248162.1(c.197G>A,aGc>aAc):XP_005248219.1(p.Ser66Asn)]	NA	NA	4.49E-02	1.27E-01	1.64E-01	4.49E-02	6.65E-02	NA	NA	NA	MODIFIER	NA	NA	158	157	0	131	62	65	49.62
Non-user	IOB_24	chr4	37592562	G	A	C4orf19	SILENT	ENSG00000154274:[ENST00000284437(c.885G>A,gcG>gcA):ENSP00000284437(p.Ala295(=))]	NA	NA	7.19E-03	2.59E-03	2.92E-03	7.19E-03	2.00E-03	NA	NA	NA	MODIFIER	NA	NA	93	92	1	83	43	40	48.19
Non-user	IOB_24	chr4	37592562	G	A	RELL1	3UTR	ENSG00000181826:[ENST00000314117(c.*281C>T)]	NA	NA	7.19E-03	2.59E-03	2.92E-03	7.19E-03	2.00E-03	NA	NA	NA	MODIFIER	NA	NA	93	92	1	83	43	40	48.19
Non-user	IOB_24	chr4	38022167	C	T	TBC1D1	INTRONIC	ENSG00000065882:[ENST00000510573(c.301-131C>T)]	NA	NA	3.57E-02	2.47E-02	5.81E-02	3.57E-02	6.75E-02	NA	NA	NA	MODIFIER	NA	NA	150	150	0	118	54	63	53.39
Non-user	IOB_24	chr4	38933869	C	T	FAM114A1	MISSENSE	ENSG00000197712:[ENST00000358869(c.1337C>T,tCg>tTg):ENSP00000351740(p.Ser446Leu)]	NA	NA	2.92E-02	1.34E-02	3.75E-02	2.92E-02	3.89E-02	T	BN	NA	MODERATE	N	1	240	240	0	153	88	63	41.18
Non-user	IOB_24	chr4	39205390	T	A	WDR19	INTRONIC	57728:[XM_005262658.1(c.537+48T>A)]	NA	NA	3.20E-03	2.94E-03	1.11E-02	3.19E-03	1.64E-02	NA	NA	NA	MODIFIER	NA	NA	202	201	0	188	95	92	48.94
Non-user	IOB_24	chr4	42658902	C	A	ATP8A1	5UTR	ENSG00000124406:[ENST00000264449(c.-12G>T)]	NA	NA	3.38E-02	4.47E-02	5.65E-02	3.37E-02	4.19E-02	NA	NA	NA	MODIFIER	NA	NA	228	227	0	190	103	87	45.79
Non-user	IOB_24	chr4	47033580	G	T	GABRB1	5UTR	ENSG00000163288:[ENST00000295454(c.-89G>T)]	NA	NA	5.59E-03	NA	NA	5.59E-03	1.74E-02	NA	NA	NA	MODIFIER	NA	NA	205	204	1	155	82	73	47.1
Non-user	IOB_24	chr4	47538515	T	G	ATP10D	MISSENSE	ENSG00000145246:[ENST00000503288(c.19T>G,gaT>gaG):ENSP00000421536(p.Asp7Glu)]	NA	NA	1.60E-03	6.26E-04	4.42E-03	1.60E-03	8.20E-03	T	BN	NA	MODERATE	D	1	323	322	0	219	111	107	48.86
Non-user	IOB_24	chr4	47945417	T	C	CNGA1	SILENT	ENSG00000198515:[ENST00000402813(c.519A>G,gaA>gaG):ENSP00000384264(p.Glu173(=))]	NA	NA	8.39E-03	1.64E-02	1.04E-02	8.39E-03	1.64E-02	NA	NA	NA	LOW	NA	0.986	25	25	0	14	7	7	50
Non-user	IOB_24	chr4	47945417	T	C	LOC101927157	NCT	101927157:[XR_241671.1(n.477-15624T>C)]	NA	NA	8.39E-03	1.64E-02	1.04E-02	8.39E-03	1.64E-02	NA	NA	NA	LOW	NA	0.986	25	25	0	14	7	7	50
Non-user	IOB_24	chr4	47945417	T	C	NIPAL1	NCT	ENSG00000163293:[ENST00000500571(n.479-15624T>C)]	NA	NA	8.39E-03	1.64E-02	1.04E-02	8.39E-03	1.64E-02	NA	NA	NA	LOW	NA	0.986	25	25	0	14	7	7	50
Non-user	IOB_24	chr4	56301675	T	G	CLOCK	SILENT	ENSG00000134852:[ENST00000309964(c.2448A>C,tcA>tcC):ENSP00000308741(p.Ser816(=))]	NA	NA	1.94E-02	3.49E-02	2.67E-02	1.94E-02	2.97E-02	NA	NA	NA	LOW	NA	NA	137	136	1	116	57	59	50.86
Non-user	IOB_24	chr4	56301675	T	G	TMEM165	INTRONIC	ENSG00000134851:[ENST00000608091(c.408+10865T>G)]	NA	NA	1.94E-02	3.49E-02	2.67E-02	1.94E-02	2.97E-02	NA	NA	NA	LOW	NA	NA	137	136	1	116	57	59	50.86
Non-user	IOB_24	chr4	56325365	G	C	CLOCK	MISSENSE	ENSG00000134852:[ENST00000309964(c.623C>G,tCt>tGt):ENSP00000308741(p.Ser208Cys)]	NA	NA	1.94E-02	3.51E-02	2.67E-02	1.94E-02	2.97E-02	D	BN	NA	MODERATE	D	1	60	60	0	49	29	20	40.82
Non-user	IOB_24	chr4	56466653	G	A	NMU	INTRONIC	ENSG00000109255:[ENST00000264218(c.489+36C>T)]	NA	NA	3.69E-02	8.02E-02	3.55E-02	3.69E-02	1.53E-02	NA	NA	NA	MODIFIER	NA	NA	37	37	0	26	18	8	30.77
Non-user	IOB_24	chr4	56475431	A	G	NMU	NCT	ENSG00000109255:[ENST00000515325(n.263-85T>C)]	NA	NA	3.24E-02	NA	NA	3.23E-02	4.29E-02	NA	NA	NA	MODIFIER	NA	NA	146	145	0	127	69	56	44.09
Non-user	IOB_24	chr4	65155584	C	A	TECRL	INTRONIC	ENSG00000205678:[ENST00000381210(c.775-99G>T)]	NA	NA	1.36E-02	NA	NA	1.36E-02	4.40E-02	NA	NA	NA	MODIFIER	NA	NA	25	25	0	8	1	7	87.5
Non-user	IOB_24	chr4	69811110	A	C	UGT2A3	MISSENSE	ENSG00000135220:[ENST00000251566(c.778T>G,Tgg>Ggg):ENSP00000251566(p.Trp260Gly)]	NA	chr4*69811110*69811110*COSM481440*A*C*1*kidney*1	6.99E-03	7.33E-03	3.72E-02	6.99E-03	3.27E-02	NA	NA	NA	MODIFIER	NA	NA	157	157	0	145	68	77	53.1
Non-user	IOB_24	chr4	70824747	T	C	CSN2	INTRONIC	ENSG00000135222:[ENST00000353151(c.99+20A>G)]	NA	NA	6.79E-03	1.14E-02	4.21E-02	6.79E-03	2.97E-02	NA	NA	NA	MODIFIER	NA	NA	21	21	0	14	6	8	57.14
Non-user	IOB_24	chr4	71020520	C	A	C4orf40	INTRONIC	ENSG00000187533:[ENST00000344526(c.51+377C>A)]	NA	NA	1.88E-02	1.83E-02	2.34E-02	1.88E-02	2.25E-02	NA	NA	NA	MODIFIER	NA	NA	178	177	0	155	91	64	41.29
Non-user	IOB_24	chr4	71697236	T	C	GRSF1	INTRONIC	2926:[XM_005265681.1(c.1193+20A>G)]	NA	NA	1.80E-03	1.68E-03	9.53E-03	1.80E-03	9.20E-03	NA	NA	NA	MODIFIER	NA	NA	212	210	0	209	95	114	54.55
Non-user	IOB_24	chr4	73156678	T	C	ADAMTS3	MISSENSE	ENSG00000156140:[ENST00000286657(c.2825A>G,aAc>aGc):ENSP00000286657(p.Asn942Ser)]	NA	NA	7.99E-04	2.45E-03	8.48E-04	7.99E-04	0.00E+00	T	BN	NA	MODERATE	N	1	131	131	0	111	51	60	54.05
Non-user	IOB_24	chr4	73169737	G	A	ADAMTS3	MISSENSE	ENSG00000156140:[ENST00000286657(c.2321C>T,tCg>tTg):ENSP00000286657(p.Ser774Leu)]	NA	chr4*73169737*73169737*COSM481479*G*T*1*kidney*1	NA	9.06E-05	6.06E-04	NA	NA	D	PrD	NA	MODERATE	D	1	216	215	0	193	85	106	54.92
Non-user	IOB_24	chr4	74313220	G	A	AFP	SILENT	ENSG00000081051:[ENST00000226359(c.885G>A,ctG>ctA):ENSP00000226359(p.Leu295(=))]	NA	NA	9.98E-04	8.69E-04	5.75E-03	9.98E-04	4.10E-03	NA	NA	NA	LOW	NA	NA	27	27	0	19	10	9	47.37
Non-user	IOB_24	chr4	78034438	G	A	RP11-1E1.2	EXONIC-NC	ENSG00000271676:[ENST00000603335(n.174C>T)]	NA	NA	5.99E-04	NA	NA	5.99E-04	0.00E+00	NA	NA	NA	MODIFIER	NA	NA	137	137	0	89	43	46	51.69
Non-user	IOB_24	chr4	78830434	G	C	MRPL1	MISSENSE	ENSG00000169288:[ENST00000502384(c.545G>C,Gac>Cac):ENSP00000421616(p.Asp183His)]	NA	NA	3.71E-02	3.56E-02	4.21E-02	3.71E-02	3.58E-02	NA	NA	NA	MODIFIER	NA	NA	50	50	0	57	21	36	63.16
Non-user	IOB_24	chr4	79285187	G	A	FRAS1	MISSENSE	ENSG00000138759:[ENST00000264895(c.2701G>A,Gat>Aat):ENSP00000264895(p.Asp901Asn)]	NA	NA	NA	1.77E-05	9.87E-05	NA	NA	T	BN	NA	MODERATE	D	1	294	293	0	297	166	130	43.77
Non-user	IOB_24	chr4	87674183	G	A	PTPN13	MISSENSE	ENSG00000163629:[ENST00000411767(c.3188G>A,gGa>gAa):ENSP00000407249(p.Gly1063Glu)]	NA	NA	5.99E-03	7.17E-03	2.57E-02	5.99E-03	2.04E-02	T	PoD	NA	MODERATE	U	1	132	132	0	83	54	28	33.73
Non-user	IOB_24	chr4	88761269	C	A	MEPE	INTRONIC	ENSG00000152595:[ENST00000560249(c.-232+85C>A)]	NA	NA	1.10E-02	NA	NA	1.10E-02	6.10E-03	NA	NA	NA	MODIFIER	NA	NA	90	90	0	78	33	45	57.69
Non-user	IOB_24	chr4	89421086	A	G	HERC5	SILENT	ENSG00000138646:[ENST00000264350(c.2454A>G,caA>caG):ENSP00000264350(p.Gln818(=))]	NA	NA	3.47E-02	7.63E-02	6.26E-02	3.47E-02	5.73E-02	NA	NA	NA	LOW	NA	0	55	55	0	51	22	29	56.86
Non-user	IOB_24	chr4	90816581	A	G	MMRN1	SILENT	ENSG00000138722:[ENST00000394981(c.357A>G,gcA>gcG):ENSP00000378432(p.Ala119(=))]	NA	NA	1.96E-02	1.81E-02	1.60E-02	1.96E-02	1.23E-02	NA	NA	NA	LOW	NA	NA	190	189	0	162	82	79	48.77
Non-user	IOB_24	chr4	90856091	C	T	MMRN1	SILENT	ENSG00000138722:[ENST00000508372(c.486C>T,ctC>ctT):ENSP00000426461(p.Leu162(=))]	NA	chr4*90856091*90856091*COSM1431735*C*T*1*large intestine*1	1.06E-02	9.47E-03	1.51E-02	1.06E-02	1.12E-02	NA	NA	NA	MODIFIER	NA	NA	209	206	0	121	52	69	57.02
Non-user	IOB_24	chr4	90857129	C	T	MMRN1	SILENT	ENSG00000138722:[ENST00000264790(c.2298C>T,acC>acT):ENSP00000264790(p.Thr766(=))]	NA	NA	1.06E-02	9.43E-03	1.51E-02	1.06E-02	1.12E-02	NA	NA	NA	MODIFIER	NA	NA	161	158	1	123	62	61	49.59
Non-user	IOB_24	chr4	93511396	C	T	GRID2	MISSENSE	ENSG00000152208:[ENST00000282020(c.203C>T,aCg>aTg):ENSP00000282020(p.Thr68Met)]	NA	chr4*93511396*93511396*COSM1130995*C*T*1*prostate*1	2.28E-02	3.51E-02	2.08E-02	2.28E-02	1.43E-02	T	BN	NA	MODERATE	D	1	185	185	0	134	79	55	41.04
Non-user	IOB_24	chr4	95198144	G	A	SMARCAD1	NCT	56916:[NR_045644.1(n.2271-29G>A)]	NA	NA	2.48E-02	1.24E-02	7.39E-02	2.48E-02	9.71E-02	NA	NA	NA	MODIFIER	NA	NA	215	214	1	151	88	63	41.72
Non-user	IOB_24	chr4	95507643	G	A	PDLIM5	INTRONIC	ENSG00000163110:[ENST00000513341(c.496+48G>A)]	NA	NA	2.08E-02	2.95E-02	6.11E-02	2.08E-02	6.13E-02	NA	NA	NA	MODIFIER	NA	NA	73	73	0	44	19	25	56.82
Non-user	IOB_24	chr4	96052416	C	G	BMPR1B	MISSENSE	ENSG00000138696:[ENST00000264568(c.829C>G,Cta>Gta):ENSP00000264568(p.Leu277Val)]	NA	NA	NA	1.65E-05	0.00E+00	NA	NA	D	PrD	NA	MODERATE	D	1	318	318	0	231	111	118	51.08
Non-user	IOB_24	chr4	99064218	T	C	STPG2	SILENT	ENSG00000163116:[ENST00000295268(c.84A>G,gtA>gtG):ENSP00000295268(p.Val28(=))]	NA	NA	5.99E-04	1.08E-03	7.64E-03	5.99E-04	3.10E-03	NA	NA	NA	LOW	NA	NA	84	84	0	73	37	36	49.32
Non-user	IOB_24	chr4	100349669	C	G	ADH7	MISSENSE	ENSG00000196344:[ENST00000476959(c.299G>C,gGa>gCa):ENSP00000420269(p.Gly100Ala)]	NA	NA	3.79E-02	8.40E-02	6.41E-02	NA	NA	D	PrD	NA	MODERATE	N	0.842	306	305	1	297	159	137	46.13
Non-user	IOB_24	chr4	100503272	A	G	MTTP	INTRONIC	ENSG00000138823:[ENST00000506883(c.279+23A>G)]	NA	NA	7.59E-03	2.71E-02	3.29E-02	7.59E-03	1.02E-02	NA	NA	NA	MODIFIER	NA	NA	43	43	0	39	14	24	61.54
Non-user	IOB_24	chr4	100512919	C	G	MTTP	MISSENSE	ENSG00000138823:[ENST00000511045(c.811C>G,Caa>Gaa):ENSP00000427679(p.Gln271Glu)]	NA	NA	2.06E-02	3.33E-02	3.42E-02	2.06E-02	1.02E-02	T	BN	NA	MODERATE	N	0.024	178	178	0	127	81	46	36.22
Non-user	IOB_24	chr4	100570601	C	T	LOC285556	SILENT	285556:[XM_373030.7(c.4773G>A,ccG>ccA):XP_373030.4(p.Pro1591(=))]	NA	NA	1.80E-03	2.20E-02	2.05E-02	1.80E-03	5.10E-03	NA	NA	NA	LOW	NA	NA	106	106	0	76	36	40	52.63
Non-user	IOB_24	chr4	100570601	C	T	RP11-766F14.2	SILENT	ENSG00000248713:[ENST00000511828(c.5205G>A,ccG>ccA):ENSP00000427555(p.Pro1735(=))]	NA	NA	1.80E-03	2.20E-02	2.05E-02	1.80E-03	5.10E-03	NA	NA	NA	LOW	NA	NA	106	106	0	76	36	40	52.63
Non-user	IOB_24	chr4	100571761	C	T	LOC285556	MISSENSE	285556:[XM_373030.7(c.3613G>A,Gct>Act):XP_373030.4(p.Ala1205Thr)]	NA	NA	1.80E-03	2.19E-02	2.05E-02	1.80E-03	5.10E-03	T	BN	NA	MODERATE	NA	NA	281	281	0	255	126	127	49.8
Non-user	IOB_24	chr4	100571761	C	T	RP11-766F14.2	MISSENSE	ENSG00000248713:[ENST00000511828(c.4045G>A,Gct>Act):ENSP00000427555(p.Ala1349Thr)]	NA	NA	1.80E-03	2.19E-02	2.05E-02	1.80E-03	5.10E-03	T	BN	NA	MODERATE	NA	NA	281	281	0	255	126	127	49.8
Non-user	IOB_24	chr4	103970037	A	T	SLC9B2	INTRONIC	ENSG00000164038:[ENST00000339611(c.713+13T>A)]	NA	NA	4.59E-03	3.16E-03	2.31E-02	4.59E-03	2.35E-02	NA	NA	NA	MODIFIER	NA	NA	101	101	0	74	35	38	51.35
Non-user	IOB_24	chr4	108855012	G	A	CYP2U1	NCT	ENSG00000155016:[ENST00000513302(n.549+1723G>A)]	NA	NA	1.40E-03	NA	NA	1.40E-03	6.10E-03	NA	NA	NA	MODIFIER	NA	NA	55	55	0	61	31	30	49.18
Non-user	IOB_24	chr4	108855012	G	A	RP11-286E11.1	NCT	ENSG00000245293:[ENST00000513071(n.81-23307C>T)]	NA	NA	1.40E-03	NA	NA	1.40E-03	6.10E-03	NA	NA	NA	MODIFIER	NA	NA	55	55	0	61	31	30	49.18
Non-user	IOB_24	chr4	109766351	A	G	COL25A1	MISSENSE	ENSG00000188517:[ENST00000494183(c.1312T>C,Tcc>Ccc):ENSP00000437131(p.Ser438Pro)]	NA	NA	NA	8.28E-06	6.06E-05	NA	NA	T_lc	UN	NA	MODERATE	NA	NA	170	170	0	75	46	29	38.67
Non-user	IOB_24	chr4	110370754	G	T	SEC24B	INTRONIC	ENSG00000138802:[ENST00000504968(c.226+89G>T)]	NA	NA	1.40E-03	NA	NA	1.40E-03	5.10E-03	NA	NA	NA	MODIFIER	NA	NA	72	71	0	51	28	23	45.1
Non-user	IOB_24	chr4	110370755	A	T	SEC24B	INTRONIC	ENSG00000138802:[ENST00000504968(c.226+90A>T)]	NA	NA	1.40E-03	NA	NA	1.40E-03	5.10E-03	NA	NA	NA	MODIFIER	NA	NA	71	69	0	51	28	23	45.1
Non-user	IOB_24	chr4	113351822	G	A	ALPK1	SILENT	ENSG00000073331:[ENST00000177648(c.1119G>A,ggG>ggA):ENSP00000177648(p.Gly373(=))]	NA	NA	3.83E-02	6.22E-02	8.07E-02	3.83E-02	6.95E-02	NA	NA	NA	LOW	NA	NA	128	126	0	99	54	45	45.45
Non-user	IOB_24	chr4	114253233	G	A	ANK2	INTRONIC	287:[XM_005262941.1(c.3320+7G>A)]	NA	NA	7.99E-04	4.37E-04	2.85E-03	7.99E-04	4.10E-03	NA	NA	NA	MODIFIER	NA	NA	58	58	0	42	16	25	59.52
Non-user	IOB_24	chr4	120255513	T	C	KLHL2P1	EXONIC-NC	ENSG00000250412:[ENST00000508691(n.30T>C)]	NA	NA	NA	NA	NA	NA	NA	NA	NA	NA	MODIFIER	NA	NA	123	122	0	15	0	15	100
Non-user	IOB_24	chr4	120265322	A	G	KLHL2P1	EXONIC-NC	ENSG00000250412:[ENST00000508691(n.135A>G)]	NA	NA	NA	NA	NA	NA	NA	NA	NA	NA	MODIFIER	NA	NA	59	59	0	7	0	7	100
Non-user	IOB_24	chr4	120549862	T	C	PDE5A	5UTR	ENSG00000138735:[ENST00000354960(c.-36A>G)]	NA	NA	1.88E-02	2.14E-02	1.20E-03	1.88E-02	1.00E-03	NA	NA	NA	MODIFIER	NA	NA	231	230	0	155	91	63	40.65
Non-user	IOB_24	chr4	122148502	G	A	TNIP3	INTRONIC	ENSG00000050730:[ENST00000509841(c.3+38C>T)]	NA	NA	5.99E-04	1.63E-04	1.31E-04	5.99E-04	0.00E+00	NA	NA	NA	MODIFIER	NA	NA	183	180	0	149	82	66	44.3
Non-user	IOB_24	chr4	123249429	G	A	KIAA1109	SILENT	ENSG00000138688:[ENST00000306802(c.398G>A,ttG>ttA):ENSP00000304374(p.Leu133(=))]	NA	NA	9.98E-03	2.24E-02	1.51E-02	9.98E-03	9.20E-03	NA	NA	NA	LOW	NA	0.875	88	85	1	73	40	33	45.21
Non-user	IOB_24	chr4	128608927	G	A	INTU	MISSENSE	ENSG00000164066:[ENST00000335251(c.1354G>A,Gcc>Acc):ENSP00000334003(p.Ala452Thr)]	NA	NA	2.20E-03	2.99E-03	9.94E-03	2.20E-03	7.20E-03	NA	NA	NA	MODIFIER	NA	NA	149	148	0	118	59	59	50

Patel *et al.* , 2021. Whole-exome sequencing analysis of oral squamous cell carcinoma delineated by tobacco usage habits
Supplementary Table 4. List of somatic single nucleotide variants identified using whole exome sequencing of OSCC patients

Cohort	Sample	Chromosome	Genomic position	Reference allele	Alternate allele	Genesymbol	Variant type	Variant description	OncoMD	COSMIC	dbSNP	ExAC	ExAC_SAS	1000G	1000G_SAS	SIFT	Polyphen2	CONDEL	VEP	LRT	PhastCon7Way	Total reads in Normal	Reference read support in Normal	Alternative read support in Normal	Total reads in Tumor	Reference read support in Tumor	Alternate read support in Tumor	Alternate allele frequency in Tumor
Non-user	IOB_24	chr4	129100683	A	G	LARP1B	NCT	ENSG00000138709:[ENST00000506199(n.199+16A>G)]	NA	NA	2.00E-04	5.20E-04	3.28E-03	2.00E-04	1.00E-03	NA	NA	NA	MODIFIER	NA	NA	145	144	0	141	72	69	48.94
Non-user	IOB_24	chr4	141330963	A	G	CLGN	INTRONIC	ENSG00000153132:[ENST00000325617(c.277+28T>C)]	NA	NA	2.76E-02	3.78E-02	7.92E-02	2.76E-02	7.16E-02	NA	NA	NA	MODIFIER	NA	NA	46	46	0	37	26	11	29.73
Non-user	IOB_24	chr4	145041750	A	C	GYPA	INTRONIC-SS-ACR-PRX	ENSG00000170180:[ENST00000360771(c.38-9T>G)]	NA	NA	NA	3.48E-01	4.13E-01	NA	NA	NA	NA	NA	MODIFIER	NA	NA	125	107	17	15	0	15	100
Non-user	IOB_24	chr4	145041750	A	C	GYPB	INTRONIC	ENSG00000250361:[ENST00000283126(c.37+20002T>G)]	NA	NA	NA	3.48E-01	4.13E-01	NA	NA	NA	NA	NA	MODIFIER	NA	NA	125	107	17	15	0	15	100
Non-user	IOB_24	chr4	145041750	A	C	RP11-673E1.4	NCT	ENSG00000248828:[ENST00000506982(n.396-5326A>C)]	NA	NA	NA	3.48E-01	4.13E-01	NA	NA	NA	NA	NA	MODIFIER	NA	NA	125	107	17	15	0	15	100
Non-user	IOB_24	chr4	148984321	A	G	ARHGAP10	MISSENSE	ENSG00000071205:[ENST00000336498(c.2050A>G,Atg>Gtg):ENSP00000336923(p.Met684Val)]	NA	NA	3.95E-02	5.89E-02	3.06E-02	3.95E-02	2.66E-02	NA	NA	NA	MODIFIER	NA	NA	304	302	1	216	104	112	51.85
Non-user	IOB_24	chr4	153881704	A	G	FHDC1	INTRONIC	ENSG00000137460:[ENST00000260008(c.664-13A>G)]	NA	NA	5.99E-04	2.72E-04	1.76E-03	5.99E-04	3.10E-03	NA	NA	NA	MODIFIER	NA	NA	250	245	2	172	80	91	52.91
Non-user	IOB_24	chr4	154543019	G	A	KIAA0922	INTRONIC	23240:[XM_005262871.1(c.1924+18G>A)]	NA	NA	NA	1.71E-03	3.75E-04	NA	NA	NA	NA	NA	MODIFIER	NA	NA	74	74	0	73	36	37	50.68
Non-user	IOB_24	chr4	154649420	C	T	RNF175	MISSENSE	ENSG00000145428:[ENST00000347063(c.340G>A,Gtt>Att):ENSP00000340979(p.Val114Ile)]	NA	chr4*154649420*154649420*COSM287497*C*T*1*large_intestine*1|chr4*154649420*154649420*COSM241439*C*T*2*large_intestine,prostate*1,1	NA	6.63E-05	1.82E-04	NA	NA	NA	NA	NA	MODIFIER	NA	NA	76	74	0	58	31	27	46.55
Non-user	IOB_24	chr4	154709492	C	T	SFRP2	MISSENSE	ENSG00000145423:[ENST00000274063(c.496G>A,Gag>Aag):ENSP00000274063(p.Glu166Lys)]	NA	NA	NA	8.30E-06	6.15E-05	NA	NA	T	BN	NA	MODERATE	N	0.987	173	172	0	146	138	6	4.11
Non-user	IOB_24	chr4	166150086	A	G	KLHL2	INTRONIC	ENSG00000109466:[ENST00000509704(c.145+21A>G)]	NA	NA	NA	3.25E-01	2.63E-01	NA	NA	NA	NA	NA	MODIFIER	NA	NA	61	61	0	82	54	28	34.15
Non-user	IOB_24	chr4	169341537	T	A	DDX60L	INTRONIC-SS-ACR-PRX	ENSG00000181381:[ENST00000505863(c.1579-6A>T)]	NA	NA	3.02E-02	5.46E-02	2.28E-02	3.02E-02	7.20E-03	NA	NA	NA	LOW	NA	NA	166	160	0	135	80	52	38.52
Non-user	IOB_24	chr4	173873256	G	A	GALNTL6	SILENT	ENSG00000174473:[ENST00000508122(c.1167G>A,gaG>gaA):ENSP00000423827(p.Glu389(=))]	NA	NA	2.20E-03	8.21E-03	1.05E-02	2.20E-03	5.10E-03	NA	NA	NA	LOW	NA	1	101	101	0	96	39	57	59.38
Non-user	IOB_24	chr4	183837910	C	T	DCTD	MISSENSE	ENSG00000129187:[ENST00000507543(c.104G>A,cGc>cAc):ENSP00000422386(p.Arg35His)]	NA	NA	7.79E-03	7.12E-02	8.42E-02	7.79E-03	1.84E-02	NA	NA	NA	MODIFIER	NA	NA	69	69	0	65	30	33	50.77
Non-user	IOB_24	chr4	184587424	G	C	TRAPPC11	MISSENSE	ENSG00000168538:[ENST00000334690(c.219G>C,gaG>gaC):ENSP00000335371(p.Glu73Asp)]	NA	NA	5.19E-03	4.25E-03	2.68E-02	5.19E-03	2.56E-02	NA	NA	NA	MODIFIER	NA	NA	55	54	0	44	25	18	40.91
Non-user	IOB_24	chr4	184614244	T	A	TRAPPC11	SILENT	ENSG00000168538:[ENST00000334690(c.2181T>A,ccT>ccA):ENSP00000335371(p.Pro727(=))]	NA	NA	NA	NA	NA	NA	NA	NA	NA	NA	MODIFIER	NA	NA	157	157	0	127	58	69	54.33
Non-user	IOB_24	chr4	185709891	A	G	ACSL1	INTRONIC-SS-ACR-PRX	ENSG00000151726:[ENST00000454703(c.-318-6T>C)]	NA	NA	2.96E-02	5.59E-02	4.40E-02	2.96E-02	2.56E-02	NA	NA	NA	LOW	NA	NA	78	77	0	65	0	65	100
Non-user	IOB_24	chr4	186112208	G	T	KIAA1430	MISSENSE	ENSG00000164323:[ENST00000296775(c.143C>A,aCa>aAa):ENSP00000296775(p.Thr48Lys)]	NA	NA	1.06E-02	2.26E-02	1.07E-02	1.06E-02	8.20E-03	T	BN	NA	MODERATE	N	0	127	127	0	75	40	35	46.67
Non-user	IOB_24	chr4	186326898	C	T	UFSP2	NCT	55325:[NR_144317.1(n.1418+36G>A)]	NA	NA	NA	8.27E-06	6.07E-05	NA	NA	NA	NA	NA	MODIFIER	NA	NA	192	192	0	160	84	75	46.88
Non-user	IOB_24	chr4	187077234	G	A	FAM149A	MISSENSE	ENSG00000109794:[ENST00000356371(c.1337G>A,cGt>cAt):ENSP00000348732(p.Arg446His)]	NA	NA	5.99E-04	2.12E-03	1.21E-04	5.99E-04	0.00E+00	NA	NA	NA	MODERATE	NA	0.757	209	209	0	134	67	66	49.25
Non-user	IOB_24	chr4	187093215	A	G	FAM149A	3UTR	ENSG00000109794:[ENST00000502894(c.*15A>G)]	NA	NA	3.87E-02	5.28E-02	6.05E-02	3.87E-02	3.58E-02	NA	NA	NA	MODIFIER	NA	NA	197	195	0	130	80	50	38.46
Non-user	IOB_24	chr4	187525048	C	T	FAT1	SILENT	ENSG00000083857:[ENST00000441802(c.10632G>A,gcG>gcA):ENSP00000406229(p.Ala3544(=))]	NA	NA	3.79E-03	5.46E-03	8.06E-03	3.79E-03	1.33E-02	NA	NA	NA	LOW	NA	NA	133	133	0	100	56	42	42
Non-user	IOB_24	chr4	189018533	G	C	TRIML2	INTRONIC	205860:[NM_173553.2(c.622-33C>G)]	NA	NA	9.19E-03	1.48E-02	2.92E-02	9.19E-03	2.56E-02	NA	NA	NA	MODIFIER	NA	NA	66	65	0	33	14	18	54.55
Non-user	IOB_24	chr5	140636	G	A	PLEKHG4B	MISSENSE	ENSG00000153404:[ENST00000283426(c.214G>A,Gct>Act):ENSP00000283426(p.Ala72Thr)]	NA	NA	3.26E-02	1.08E-01	4.00E-02	3.25E-02	2.66E-02	T	BN	NA	MODERATE	NA	NA	160	160	0	162	90	72	44.44
Non-user	IOB_24	chr5	824228	G	A	ZDHHC11	NCT	ENSG00000188818:[ENST00000503758(n.2087-304C>T)]	NA	NA	1.52E-02	1.45E-02	7.87E-03	1.52E-02	4.10E-03	NA	NA	NA	MODIFIER	NA	NA	78	78	0	74	38	35	47.3
Non-user	IOB_24	chr5	870740	C	T	BRD9	INTRONIC	ENSG00000028310:[ENST00000489816(c.*1115-50G>A)]	NA	NA	4.69E-02	1.15E-01	3.09E-02	4.69E-02	2.15E-02	NA	NA	NA	MODIFIER	NA	NA	46	46	0	37	26	11	29.73
Non-user	IOB_24	chr5	1074803	C	T	SLC12A7	MISSENSE	ENSG00000113504:[ENST00000513223(c.23G>A,Gac>Aac):ENSP00000428854(p.Asp9Asn)]	NA	NA	9.78E-03	1.38E-02	2.97E-02	9.78E-03	2.97E-02	T_lc	UN	NA	MODERATE	NA	NA	137	137	0	97	58	39	40.21
Non-user	IOB_24	chr5	1208964	T	C	SLC6A19	SILENT	ENSG00000174358:[ENST00000304460(c.306T>C,ggT>ggC):ENSP00000305302(p.Gly102(=))]	NA	NA	NA	3.70E-01	3.63E-01	NA	NA	NA	NA	NA	LOW	NA	NA	69	69	0	60	33	27	45
Non-user	IOB_24	chr5	1214263	C	T	SLC6A19	INTRONIC	ENSG00000174358:[ENST00000304460(c.887+83C>T)]	NA	NA	6.19E-03	NA	NA	6.19E-03	1.43E-02	NA	NA	NA	MODIFIER	NA	NA	138	135	0	82	51	31	37.8
Non-user	IOB_24	chr5	1225656	G	A	SLC6A18	MISSENSE	ENSG00000164363:[ENST00000296821(c.64G>A,Gac>Aac):ENSP00000296821(p.Asp22Asn)]	NA	NA	1.20E-03	2.40E-04	1.70E-03	1.20E-03	6.10E-03	D	PrD	NA	MODERATE	D	1	91	91	0	47	22	23	48.94
Non-user	IOB_24	chr5	1321850	C	T	CLPTM1L	INTRONIC	ENSG00000049656:[ENST00000320927(c.1263+29G>A)]	NA	NA	5.59E-03	2.99E-03	2.14E-02	5.59E-03	2.86E-02	NA	NA	NA	MODIFIER	NA	NA	298	298	0	245	122	121	49.39
Non-user	IOB_24	chr5	1466919	G	A	LPCAT1	SILENT	ENSG00000153395:[ENST00000283415(c.1365C>T,acC>acT):ENSP00000283415(p.Thr455(=))]	NA	chr5*1466919*1466919*COSM1130846*G*A*1*prostate*1	NA	2.82E-01	3.86E-01	NA	NA	NA	NA	NA	MODIFIER	NA	NA	342	340	2	319	155	162	50.78
Non-user	IOB_24	chr5	5318471	G	T	ADAMTS16	INTRONIC	ENSG00000145536:[ENST00000274181(c.3559+77G>T)]	NA	NA	6.19E-03	NA	NA	6.19E-03	1.94E-02	NA	NA	NA	MODIFIER	NA	NA	81	81	0	58	24	34	58.62
Non-user	IOB_24	chr5	5423145	G	T	KIAA0947	NCT	ENSG00000164151:[ENST00000512608(n.29+2340G>T)]	NA	NA	NA	2.13E-03	2.66E-03	NA	NA	NA	NA	NA	MODIFIER	NA	NA	19	19	0	13	6	7	53.85
Non-user	IOB_24	chr5	7897277	G	A	MTRR	SILENT	ENSG00000124275:[ENST00000264668(c.1950G>A,aaG>aaA):ENSP00000264668(p.Lys650(=))]	NA	NA	NA	1.65E-05	1.21E-04	NA	NA	NA	NA	NA	MODIFIER	NA	NA	148	148	0	103	32	70	67.96
Non-user	IOB_24	chr5	9122926	C	T	SEMA5A	SILENT	ENSG00000112902:[ENST00000382496(c.1623G>A,ggG>ggA):ENSP00000371936(p.Gly541(=))]	NA	NA	NA	1.28E-01	1.61E-01	NA	NA	NA	NA	NA	LOW	NA	NA	239	236	0	211	86	124	58.77
Non-user	IOB_24	chr5	10988317	G	C	CTNND2	SILENT	ENSG00000169862:[ENST00000503622(c.2238C>G,ctC>ctG):ENSP00000426887(p.Leu746(=))]	NA	NA	NA	8.24E-06	6.06E-05	NA	NA	NA	NA	NA	LOW	NA	NA	170	169	1	138	64	74	53.62
Non-user	IOB_24	chr5	14316642	G	A	TRIO	SILENT	ENSG00000038382:[ENST00000512070(c.1344G>A,tcG>tcA):ENSP00000421555(p.Ser448(=))]	NA	NA	3.63E-02	4.18E-02	3.60E-02	3.63E-02	2.66E-02	NA	NA	NA	MODIFIER	NA	NA	80	80	0	51	26	25	49.02
Non-user	IOB_24	chr5	14330940	T	C	TRIO	SILENT	ENSG00000038382:[ENST00000512070(c.1608T>C,ggT>ggC):ENSP00000421555(p.Gly536(=))]	NA	NA	3.99E-02	4.29E-02	3.62E-02	3.99E-02	2.86E-02	NA	NA	NA	LOW	NA	NA	219	218	0	177	92	84	47.46
Non-user	IOB_24	chr5	14610309	C	G	FAM105A	MISSENSE	ENSG00000145569:[ENST00000274217(c.957C>G,ttC>ttG):ENSP00000274217(p.Phe319Leu)]	NA	NA	2.18E-02	5.58E-02	1.24E-02	2.18E-02	1.23E-02	T	BN	NA	MODERATE	N	1	61	61	0	65	36	28	43.08
Non-user	IOB_24	chr5	17275756	A	G	BASP1	MISSENSE	ENSG00000176788:[ENST00000322611(c.431A>G,gAg>gGg):ENSP00000319281(p.Glu144Gly)]	NA	NA	5.99E-04	2.15E-03	3.96E-03	5.99E-04	3.10E-03	D	BN	NA	MODERATE	U	1	48	47	0	43	22	21	48.84
Non-user	IOB_24	chr5	32712565	G	A	NPR3	MISSENSE	ENSG00000113389:[ENST00000265074(c.683G>A,gGt>gAt):ENSP00000265074(p.Gly228Asp)]	NA	NA	NA	1.65E-04	7.76E-04	NA	NA	T	BN	NA	MODERATE	NA	NA	319	319	0	243	122	120	49.38
Non-user	IOB_24	chr5	33452416	A	G	TARS	INTRONIC	ENSG00000113407:[ENST00000508361(c.139-978A>G)]	NA	NA	NA	1.31E-03	1.74E-03	NA	NA	NA	NA	NA	MODIFIER	NA	NA	96	96	0	104	61	43	41.35
Non-user	IOB_24	chr5	33453456	G	A	TARS	MISSENSE	ENSG00000113407:[ENST00000455217(c.491G>A,cGc>cAc):ENSP00000387710(p.Arg164His)]	NA	chr5*33453456*33453456*COSM185937*G*A*2*large_intestine,endometrium*1,1	NA	1.32E-04	7.28E-04	NA	NA	D	PrD	NA	MODERATE	D	1	138	127	0	113	48	55	48.67
Non-user	IOB_24	chr5	33457493	T	C	TARS	SILENT	ENSG00000113407:[ENST00000455217(c.1068T>C,caT>caC):ENSP00000387710(p.His356(=))]	NA	NA	NA	NA	NA	NA	NA	NA	NA	NA	MODIFIER	NA	NA	119	119	0	116	58	58	50
Non-user	IOB_24	chr5	33459830	G	A	TARS	MISSENSE	ENSG00000113407:[ENST00000414361(c.751G>A,Gta>Ata):ENSP00000394291(p.Val251Ile)]	NA	NA	NA	2.06E-04	1.33E-03	NA	NA	NA	NA	NA	MODIFIER	NA	NA	123	123	0	90	49	40	44.44
Non-user	IOB_24	chr5	33546329	A	T	ADAMTS12	INTRONIC	ENSG00000151388:[ENST00000352040(c.4048-22T>A)]	NA	NA	NA	1.15E-04	8.64E-04	NA	NA	NA	NA	NA	MODIFIER	NA	NA	21	21	0	14	5	9	64.29
Non-user	IOB_24	chr5	34124590	C	A	C1QTNF3-AMACR	EXONIC-NC	100534612:[NR_037951.1(n.44G>T)]	NA	NA	1.28E-02	2.51E-02	3.70E-02	1.28E-02	4.09E-02	NA	NA	NA	MODIFIER	NA	NA	49	49	0	42	29	13	30.95
Non-user	IOB_24	chr5	34124590	C	A	RP11-1084J3.4	5UTR	ENSG00000273294:[ENST00000382079(c.-32G>T)]	NA	NA	1.28E-02	2.51E-02	3.70E-02	1.28E-02	4.09E-02	NA	NA	NA	MODIFIER	NA	NA	49	49	0	42	29	13	30.95
Non-user	IOB_24	chr5	35709095	C	T	CTD-2113L7.1	NCT	ENSG00000248969:[ENST00000510433(n.174-10958G>A)]	NA	NA	NA	7.56E-01	6.99E-01	NA	NA	NA	NA	NA	MODIFIER	NA	NA	126	126	0	72	0	71	98.61
Non-user	IOB_24	chr5	35709095	C	T	SPEF2	MISSENSE	ENSG00000152582:[ENST00000440995(c.2696C>T,gCt>gTt):ENSP00000412125(p.Ala899Val)]	NA	NA	NA	7.56E-01	6.99E-01	NA	NA	NA	NA	NA	MODIFIER	NA	NA	126	126	0	72	0	71	98.61
Non-user	IOB_24	chr5	35709184	G	C	CTD-2113L7.1	NCT	ENSG00000248969:[ENST00000510433(n.174-11047C>G)]	NA	NA	NA	7.56E-01	6.99E-01	NA	NA	NA	NA	NA	MODIFIER	NA	NA	104	104	0	81	0	81	100
Non-user	IOB_24	chr5	35709184	G	C	SPEF2	MISSENSE	ENSG00000152582:[ENST00000440995(c.2785G>C,Gct>Cct):ENSP00000412125(p.Ala929Pro)]	NA	NA	NA	7.56E-01	6.99E-01	NA	NA	NA	NA	NA	MODIFIER	NA	NA	104	104	0	81	0	81	100
Non-user	IOB_24	chr5	38943051	T	C	OSMR	INTRONIC	ENSG00000145623:[ENST00000508882(c.75-1252T>C)]	NA	NA	NA	8.31E-06	6.09E-05	NA	NA	NA	NA	NA	MODIFIER	NA	NA	42	42	0	37	21	16	43.24
Non-user	IOB_24	chr5	38943051	T	C	RICTOR	MISSENSE	ENSG00000164327:[ENST00000357387(c.4936A>G,Aca>Gca):ENSP00000349959(p.Thr1646Ala)]	NA	NA	NA	8.31E-06	6.09E-05	NA	NA	NA	NA	NA	MODIFIER	NA	NA	42	42	0	37	21	16	43.24
Non-user	IOB_24	chr5	39364453	A	C	C9	NCT	ENSG00000113600:[ENST00000483232(n.120-22155T>G)]	NA	NA	1.20E-02	6.10E-03	4.35E-02	1.20E-02	6.13E-02	NA	NA	NA	MODIFIER	NA	NA	202	201	0	138	67	71	51.45
Non-user	IOB_24	chr5	40691893	G	A	PTGER4	MISSENSE	ENSG00000171522:[ENST00000302472(c.880G>A,Gtc>Atc):ENSP00000302846(p.Val294Ile)]	NA	NA	8.39E-03	1.70E-02	7.70E-03	8.39E-03	6.10E-03	NA	NA	NA	MODIFIER	NA	NA	287	284	1	213	103	106	49.77
Non-user	IOB_24	chr5	40834772	G	A	RPL37	INTRONIC	ENSG00000145592:[ENST00000274242(c.4-64C>T)]	NA	NA	9.78E-03	NA	NA	9.78E-03	2.45E-02	NA	NA	NA	MODIFIER	NA	NA	207	204	0	162	94	67	41.36
Non-user	IOB_24	chr5	42646392	A	C	GHR	INTRONIC	2690:[NM_001242399.2(c.157+17187A>C)]	NA	NA	2.26E-02	2.59E-02	2.76E-02	2.26E-02	1.84E-02	NA	NA	NA	MODIFIER	NA	NA	201	198	2	160	0	159	99.38
Non-user	IOB_24	chr5	42719082	C	T	GHR	SILENT	ENSG00000112964:[ENST00000357703(c.1407C>T,agC>agT):ENSP00000350335(p.Ser469(=))]	NA	NA	2.06E-02	2.26E-02	3.12E-02	2.06E-02	2.86E-02	NA	NA	NA	MODIFIER	NA	NA	198	196	0	159	79	79	49.69
Non-user	IOB_24	chr5	43298951	A	G	HMGCS1	SILENT	ENSG00000112972:[ENST00000325110(c.117T>C,taT>taC):ENSP00000322706(p.Tyr39(=))]	NA	chr5*43298951*43298951*COSM149824*A*G*1*stomach*1	1.98E-02	1.63E-02	2.04E-02	1.98E-02	1.23E-02	NA	NA	NA	LOW	NA	NA	463	460	0	370	201	166	44.86
Non-user	IOB_24	chr5	43509364	T	C	C5orf34	SILENT	ENSG00000172244:[ENST00000306862(c.78A>G,caA>caG):ENSP00000303490(p.Gln26(=))]	NA	chr5*43509364*43509364*COSM149826*T*C*1*stomach*1	1.64E-02	7.46E-03	2.12E-03	1.64E-02	3.10E-03	NA	NA	NA	MODIFIER	NA	NA	115	115	0	83	35	47	56.63
Non-user	IOB_24	chr5	43509364	T	C	RP11-159F24.3	EXONIC-NC	ENSG00000249492:[ENST00000505645(n.388T>C)]	NA	chr5*43509364*43509364*COSM149826*T*C*1*stomach*1	1.64E-02	7.46E-03	2.12E-03	1.64E-02	3.10E-03	NA	NA	NA	MODIFIER	NA	NA	115	115	0	83	35	47	56.63
Non-user	IOB_24	chr5	43613046	A	G	NNT	MISSENSE	ENSG00000112992:[ENST00000264663(c.188A>G,aAa>aGa):ENSP00000264663(p.Lys63Arg)]	NA	chr5*43613046*43613046*COSM149828*A*G*1*stomach*1	4.23E-02	4.60E-02	6.81E-02	4.23E-02	5.11E-02	NA	NA	NA	MODIFIER	NA	NA	132	131	0	130	63	65	50
Non-user	IOB_24	chr5	43644442	G	A	NNT	INTRONIC	ENSG00000112992:[ENST00000264663(c.1098+15G>A)]	NA	NA	4.31E-02	4.47E-02	6.68E-02	4.31E-02	5.11E-02	NA	NA	NA	MODIFIER	NA	NA	50	50	0	49	28	21	42.86
Non-user	IOB_24	chr5	43644481	G	A	NNT	INTRONIC	ENSG00000112992:[ENST00000512996(c.705+54G>A)]	NA	NA	4.31E-02	NA	NA	4.31E-02	5.11E-02	NA	NA	NA	MODIFIER	NA	NA	39	38	0	36	22	14	38.89
Non-user	IOB_24	chr5	43653243	C	T	NNT	MISSENSE	ENSG00000112992:[ENST00000264663(c.1987C>T,Ctc>Ttc):ENSP00000264663(p.Leu663Phe)]	NA	NA	4.33E-02	4.47E-02	6.68E-02	4.33E-02	5.11E-02	NA	NA	NA	MODIFIER	NA	NA	118	118	0	84	50	34	40.48
Non-user	IOB_24	chr5	52223323	C	T	ITGA1	NCT	ENSG00000213949:[ENST00000504669(n.5289-91C>T)]	NA	NA	NA	NA	NA	NA	NA	NA	NA	NA	MODIFIER	NA	NA	152	152	0	140	73	67	47.86
Non-user	IOB_24	chr5	52776728	G	C	FST	INTRONIC	ENSG00000134363:[ENST00000256759(c.85+22G>C)]	NA	NA	1.72E-02	3.67E-02	2.97E-02	1.72E-02	2.86E-02	NA	NA	NA	MODIFIER	NA	NA	100	100	0	61	29	32	52.46
Non-user	IOB_24	chr5	53815219	G	A	SNX18	SILENT	ENSG00000178996:[ENST00000326277(c.1437G>A,gaG>gaA):ENSP00000317332(p.Glu479(=))]	NA	NA	1.94E-02	3.44E-02	2.79E-02	1.94E-02	2.66E-02	NA	NA	NA	LOW	NA	1	298	296	0	251	135	112	44.62
Non-user	IOB_24	chr5	54468450	T	C	CDC20B	MISSENSE	ENSG00000164287:[ENST00000296733(c.92A>G,gAc>gGc):ENSP00000296733(p.Asp31Gly)]	NA	NA	6.19E-03	1.15E-02	1.36E-02	6.19E-03	9.20E-03	T	BN	NA	MODERATE	NA	NA	232	228	1	225	104	121	53.78
Non-user	IOB_24	chr5	55059937	C	T	DDX4	NCT	ENSG00000152670:[ENST00000508580(n.395+45C>T)]	NA	NA	2.00E-04	4.96E-05	3.65E-04	2.00E-04	1.00E-03	NA	NA	NA	MODIFIER	NA	NA	54	54	0	26	14	12	46.15
Non-user	IOB_24	chr5	55059937	C	T	NA	NCT	101928393:[XR_241793.1(n.10+181G>A)]	NA	NA	2.00E-04	4.96E-05	3.65E-04	2.00E-04	1.00E-03	NA	NA	NA	MODIFIER	NA	NA	54	54	0	26	14	12	46.15
Non-user	IOB_24	chr5	55059937	C	T	SLC38A9	NCT	ENSG00000177058:[ENST00000504880(n.114+8972G>A)]	NA	NA	2.00E-04	4.96E-05	3.65E-04	2.00E-04	1.00E-03	NA	NA	NA	MODIFIER	NA	NA	54	54	0	26	14	12	46.15
Non-user	IOB_24	chr5	56111704	G	A	MAP3K1	MISSENSE	ENSG00000095015:[ENST00000399503(c.304G>A,Ggg>Agg):ENSP00000382423(p.Gly102Arg)]	NA	NA	2.40E-03	2.31E-02	3.11E-02	2.40E-03	1.23E-02	D_lc	BN	NA	MODERATE	U	0.059	52	52	0	39	22	17	43.59
Non-user	IOB_24	chr5	56111794	G	C	MAP3K1	MISSENSE	ENSG00000095015:[ENST00000399503(c.394G>C,Gac>Cac):ENSP00000382423(p.Asp132His)]	NA	NA	chr5*56111794*56111794*G*A,C*rs557606535*NA*NA*NA	2.88E-02	3.21E-02	NA	NA	D_lc	PrD	NA	MODERATE	N	0.989	23	23	0	15	10	5	33.33
Non-user	IOB_24	chr5	56531795	G	A	GPBP1	SILENT-SS-PRX	ENSG00000062194:[ENST00000513732(n.22G>A)]	NA	chr5*56531795*56531795*COSM1069077*G*T*1*endometrium*1	6.79E-03	5.29E-03	3.87E-02	6.79E-03	3.48E-02	NA	NA	NA	LOW	NA	NA	73	72	0	65	34	31	47.69
Non-user	IOB_24	chr5	58272149	C	A	PDE4D	INTRONIC	5144:[NM_001197222.1(c.1158+28G>T)]	NA	NA	NA	1.92E-05	1.49E-04	NA	NA	NA	NA	NA	MODIFIER	NA	NA	214	213	0	181	101	80	44.2

Patel *et al.* , 2021. Whole-exome sequencing analysis of oral squamous cell carcinoma delineated by tobacco usage habits
Supplementary Table 4. List of somatic single nucleotide variants identified using whole exome sequencing of OSCC patients

Cohort	Sample	Chromosome	Genomic position	Reference allele	Alternate allele	Genesymbol	Variant type	Variant description	OncoMD	COSMIC	dbSNP	ExAC	ExAC_SAS	1000G	1000G_SAS	SIFT	Polyphen2	CONDEL	VEP	LRT	PhastCon7Way	Total reads in Normal	Reference read support in Normal	Alternative read support in Normal	Total reads in Tumor	Reference read support in Tumor	Alternate read support in Tumor	Alternate allele frequency in Tumor
Non-user	IOB_24	chr5	58297881	A	G	PDE4D	NCT	ENSG00000113448:[ENST00000405053(n.472-8589T>C)]	NA	NA	NA	8.66E-01	8.30E-01	NA	NA	NA	NA	NA	MODIFIER	NA	NA	215	214	0	150	53	96	64
Non-user	IOB_24	chr5	58571090	A	G	PDE4D	INTRONIC	ENSG00000113448:[ENST00000502484(c.273-59296T>C)]	NA	NA	1.20E-03	NA	NA	1.20E-03	6.10E-03	NA	NA	NA	MODIFIER	NA	NA	102	102	0	56	26	30	53.57
Non-user	IOB_24	chr5	64872697	A	G	PPWD1	NCT	ENSG00000113593:[ENST00000507608(n.940-9A>G)]	NA	NA	2.08E-02	3.21E-02	5.00E-02	2.08E-02	5.52E-02	NA	NA	NA	MODIFIER	NA	NA	203	203	0	171	87	83	48.54
Non-user	IOB_24	chr5	66427750	A	T	MAST4	SILENT	ENSG00000069020:[ENST00000405643(c.1527A>T,ggA>ggT):ENSP00000384099(p.Gly509(=))]	NA	NA	NA	4.97E-05	3.64E-04	NA	NA	NA	NA	NA	LOW	NA	NA	140	139	1	87	44	43	49.43
Non-user	IOB_24	chr5	66437935	C	G	MAST4	INTRONIC-SS-ACR-PRX	ENSG00000069020:[ENST00000405643(c.1958-8C>G)]	NA	NA	4.99E-03	2.65E-03	1.73E-02	4.99E-03	2.04E-02	NA	NA	NA	LOW	NA	NA	305	303	0	217	97	120	55.3
Non-user	IOB_24	chr5	70785472	T	C	BDP1	SILENT	ENSG00000145734:[ENST00000358731(c.1455T>C,acT>acC):ENSP00000351575(p.Thr485(=))]	NA	NA	NA	NA	NA	NA	NA	NA	NA	NA	MODIFIER	NA	NA	110	109	0	51	26	25	49.02
Non-user	IOB_24	chr5	70948576	C	T	MCCC2	SILENT	ENSG00000131844:[ENST00000340941(c.1569C>T,agC>agT):ENSP00000343657(p.Ser523(=))]	NA	NA	9.98E-04	1.25E-03	8.57E-03	9.98E-04	5.10E-03	NA	NA	NA	LOW	NA	1	87	87	0	51	32	19	37.25
Non-user	IOB_24	chr5	71756481	G	A	ZNF366	SILENT	ENSG00000178175:[ENST00000318442(c.843C>T,caC>caT):ENSP00000313158(p.His281(=))]	NA	NA	2.00E-04	9.06E-05	4.84E-04	2.00E-04	0.00E+00	NA	NA	NA	LOW	NA	1	246	246	0	167	75	91	54.49
Non-user	IOB_24	chr5	72861611	G	T	UTP15	SILENT	ENSG00000164338:[ENST00000509005(c.44G>T,cgG>cgT):ENSP00000421669(p.Arg15(=))]	NA	NA	NA	NA	NA	NA	NA	NA	NA	NA	MODIFIER	NA	NA	216	214	0	175	88	86	49.14
Non-user	IOB_24	chr5	74886186	A	G	POLK	MISSENSE	ENSG00000122008:[ENST00000380481(c.1007A>G,aAt>aGt):ENSP00000369848(p.Asn336Ser)]	NA	NA	NA	4.97E-05	3.71E-04	NA	NA	T	BN	NA	MODERATE	N	1	72	72	0	64	28	35	54.69
Non-user	IOB_24	chr5	75757530	C	T	IQGAP2	INTRONIC	10788:[XM_005248409.1(c.116+36C>T)]	NA	NA	4.79E-03	2.21E-03	1.59E-02	4.79E-03	2.35E-02	NA	NA	NA	MODIFIER	NA	NA	202	201	0	168	92	76	45.24
Non-user	IOB_24	chr5	76128909	G	T	F2RL1	MISSENSE	ENSG00000164251:[ENST00000296677(c.477G>T,atG>atT):ENSP00000296677(p.Met159Ile)]	NA	NA	3.99E-04	2.39E-04	1.76E-03	3.99E-04	2.00E-03	D	PrD	NA	MODERATE	NA	NA	329	327	0	284	128	156	54.93
Non-user	IOB_24	chr5	78378824	C	T	BHMT2	NCT	ENSG00000132840:[ENST00000523046(n.565+45C>T)]	NA	NA	4.11E-02	7.21E-02	5.37E-02	4.11E-02	6.34E-02	NA	NA	NA	MODIFIER	NA	NA	186	186	0	180	101	78	43.33
Non-user	IOB_24	chr5	78378824	C	T	DMGDH	NCT	ENSG00000132837:[ENST00000520388(n.606+21163G>A)]	NA	NA	4.11E-02	7.21E-02	5.37E-02	4.11E-02	6.34E-02	NA	NA	NA	MODIFIER	NA	NA	186	186	0	180	101	78	43.33
Non-user	IOB_24	chr5	78752802	T	C	HOMER1	SILENT	ENSG00000152413:[ENST00000282260(c.45A>G,caA>caG):ENSP00000282260(p.Gln15(=))]	NA	NA	4.05E-02	3.80E-02	2.02E-02	4.05E-02	1.84E-02	NA	NA	NA	LOW	NA	1	207	207	0	161	1	157	97.52
Non-user	IOB_24	chr5	78752845	C	A	HOMER1	INTRONIC-SS-ACR-PRX	ENSG00000152413:[ENST00000282260(c.6-4G>T)]	NA	NA	7.99E-03	1.81E-02	4.24E-03	7.99E-03	4.10E-03	NA	NA	NA	MODIFIER	NA	NA	193	192	0	166	77	88	53.01
Non-user	IOB_24	chr5	80390704	C	T	RASGRF2	SILENT	ENSG00000113319:[ENST00000265080(c.1648C>T,Ctg>Ttg):ENSP00000265080(p.Leu550(=))]	NA	NA	2.96E-02	1.36E-02	3.63E-02	2.96E-02	3.99E-02	NA	NA	NA	MODIFIER	NA	NA	120	119	0	111	60	50	45.05
Non-user	IOB_24	chr5	80409407	T	G	CTD-2193P3.2	EXONIC-NC	ENSG00000249772:[ENST00000508993(n.352A>C)]	NA	NA	2.58E-02	1.41E-02	3.68E-02	2.58E-02	3.89E-02	T_lc	PoD	NA	MODERATE	NA	NA	297	297	0	229	122	105	45.85
Non-user	IOB_24	chr5	80409407	T	G	RASGRF2	MISSENSE	ENSG00000113319:[ENST00000265080(c.2138T>G,tTg>tGg):ENSP00000265080(p.Leu713Trp)]	NA	NA	2.58E-02	1.41E-02	3.68E-02	2.58E-02	3.89E-02	T_lc	PoD	NA	MODERATE	NA	NA	297	297	0	229	122	105	45.85
Non-user	IOB_24	chr5	85586831	C	T	NBPF22P	EXONIC-NC	285622:[NR_003719.2(n.1134C>T)]	NA	NA	chr5*85586831*85586831*C*A,T*rs12654078*NA*NA*NA	1.16E-01	1.63E-01	NA	NA	NA	NA	NA	MODIFIER	NA	NA	100	99	0	68	42	26	38.24
Non-user	IOB_24	chr5	90072351	T	C	ADGRV1	EXONIC-NC	84059:[NR_003149.1(n.12498T>C)]	NA	chr5*90072351*90072351*COSM169460*T*C*1*large_intestine*1	NA	2.49E-05	0.00E+00	NA	NA	NA	NA	NA	MODIFIER	NA	NA	284	284	0	230	107	123	53.48
Non-user	IOB_24	chr5	90072351	T	C	GPR98	MISSENSE	ENSG00000164199:[ENST00000405460(c.12485T>C,aTt>aCt):ENSP00000384582(p.Ile4162Thr)]	NA	chr5*90072351*90072351*COSM169460*T*C*1*large_intestine*1	NA	2.49E-05	0.00E+00	NA	NA	NA	NA	NA	MODIFIER	NA	NA	284	284	0	230	107	123	53.48
Non-user	IOB_24	chr5	90111521	A	G	ADGRV1	EXONIC-NC	84059:[NR_003149.1(n.16177A>G)]	NA	NA	1.46E-02	3.11E-02	5.80E-02	1.46E-02	4.81E-02	NA	NA	NA	LOW	NA	0.005	104	104	0	76	39	36	47.37
Non-user	IOB_24	chr5	90111521	A	G	GPR98	SILENT	ENSG00000164199:[ENST00000425867(c.3147A>G,agA>agG):ENSP00000392618(p.Arg1049(=))]	NA	NA	1.46E-02	3.11E-02	5.80E-02	1.46E-02	4.81E-02	NA	NA	NA	LOW	NA	0.005	104	104	0	76	39	36	47.37
Non-user	IOB_24	chr5	93720171	T	C	KIAA0825	INTRONIC	285600:[NM_001145678.1(c.3620-8A>G)]	NA	NA	9.19E-03	2.08E-02	2.42E-02	9.19E-03	2.25E-02	NA	NA	NA	LOW	NA	NA	149	149	0	133	66	67	50.38
Non-user	IOB_24	chr5	95011111	C	G	RFESD	NCT	ENSG00000175449:[ENST00000508206(n.546+4902C>G)]	NA	NA	chr5*95011103*95011112*AGTATTCCCT*A*rs773629042*NA*NA*NA|chr5*95011111*95011111*C*A*rs376391160*NA*NA*NA	NA	NA	NA	NA	NA	NA	NA	MODIFIER	NA	NA	198	197	1	163	82	81	49.69
Non-user	IOB_24	chr5	95011111	C	G	SPATA9	NCT	ENSG00000145757:[ENST00000379990(n.125-11359G>C)]	NA	NA	chr5*95011103*95011112*AGTATTCCCT*A*rs773629042*NA*NA*NA|chr5*95011111*95011111*C*A*rs376391160*NA*NA*NA	NA	NA	NA	NA	NA	NA	NA	MODIFIER	NA	NA	198	197	1	163	82	81	49.69
Non-user	IOB_24	chr5	96518927	C	G	CTD-2215E18.1	MISSENSE	ENSG00000251606:[ENST00000509481(c.248C>G,cCg>cGg):ENSP00000424860(p.Pro83Arg)]	NA	NA	3.40E-02	5.86E-02	5.07E-02	3.39E-02	3.48E-02	NA	PoD	NA	MODERATE	NA	NA	405	404	0	293	184	107	36.52
Non-user	IOB_24	chr5	96518927	C	G	RIOK2	5UTR	ENSG00000058729:[ENST00000283109(c.-32G>C)]	NA	NA	3.40E-02	5.86E-02	5.07E-02	3.39E-02	3.48E-02	NA	PoD	NA	MODERATE	NA	NA	405	404	0	293	184	107	36.52
Non-user	IOB_24	chr5	104077210	A	C	RP11-6N13.1	NCT	ENSG00000251574:[ENST00000524336(n.190+145349T>G)]	NA	NA	1.60E-02	NA	NA	1.60E-02	8.18E-02	NA	NA	NA	MODIFIER	NA	NA	12	12	0	18	12	6	33.33
Non-user	IOB_24	chr5	110441839	G	A	WDR36	MISSENSE	ENSG00000134987:[ENST00000506538(c.1345G>A,Gca>Aca):ENSP00000423067(p.Ala449Thr)]	NA	NA	1.04E-02	8.52E-03	3.96E-02	1.04E-02	4.40E-02	T	BN	NA	MODERATE	D	1	301	299	0	246	125	121	49.19
Non-user	IOB_24	chr5	118324113	G	C	DTWD2	MISSENSE	ENSG00000169570:[ENST00000506980(c.94C>G,Cgg>Ggg):ENSP00000425016(p.Arg32Gly)]	NA	NA	1.32E-02	1.77E-02	4.69E-02	1.32E-02	3.17E-02	T_lc	BN	NA	MODERATE	N	0	140	140	0	128	58	70	54.69
Non-user	IOB_24	chr5	118533499	A	G	DMXL1	SILENT	ENSG00000172869:[ENST00000311085(c.7593A>G,caA>caG):ENSP00000309690(p.Gln2531(=))]	NA	NA	3.18E-02	1.57E-02	1.44E-02	3.17E-02	1.33E-02	NA	NA	NA	LOW	NA	0.098	194	194	0	174	85	87	50
Non-user	IOB_24	chr5	118582779	G	C	DMXL1	SILENT	ENSG00000172869:[ENST00000539542(c.9012G>C,gtG>gtC):ENSP00000439479(p.Val3004(=))]	NA	NA	4.73E-02	1.83E-02	4.30E-03	4.73E-02	5.10E-03	NA	NA	NA	LOW	NA	NA	285	284	0	196	113	83	42.35
Non-user	IOB_24	chr5	118604732	G	A	TNFAIP8	INTRONIC	ENSG00000145779:[ENST00000274456(c.1+130G>A)]	NA	NA	1.88E-02	NA	NA	1.88E-02	4.10E-03	NA	NA	NA	MODIFIER	NA	NA	173	172	1	135	76	57	42.22
Non-user	IOB_24	chr5	121487961	T	C	CTC-441N14.2	NCT	ENSG00000247311:[ENST00000504829(n.233+2358A>G)]	NA	NA	3.99E-04	3.39E-04	2.43E-04	3.99E-04	0.00E+00	NA	NA	NA	MODIFIER	NA	NA	112	112	0	88	52	36	40.91
Non-user	IOB_24	chr5	121487961	T	C	ZNF474	SILENT	ENSG00000164185:[ENST00000296600(c.276T>C,ccT>ccC):ENSP00000296600(p.Pro92(=))]	NA	NA	3.99E-04	3.39E-04	2.43E-04	3.99E-04	0.00E+00	NA	NA	NA	MODIFIER	NA	NA	112	112	0	88	52	36	40.91
Non-user	IOB_24	chr5	125696034	G	A	GRAMD3	MISSENSE	ENSG00000155324:[ENST00000513040(c.11G>A,aGa>aAa):ENSP00000426120(p.Arg4Lys)]	NA	NA	1.80E-03	1.85E-03	4.56E-03	1.80E-03	9.20E-03	T_lc	BN	NA	MODERATE	NA	NA	177	177	0	145	69	75	51.72
Non-user	IOB_24	chr5	125896685	G	A	ALDH7A1	NCT	ENSG00000164904:[ENST00000503281(n.502+90C>T)]	NA	NA	9.98E-04	NA	NA	9.98E-04	5.10E-03	NA	NA	NA	MODIFIER	NA	NA	84	84	0	71	46	25	35.21
Non-user	IOB_24	chr5	127507383	A	T	SLC12A2	SILENT	ENSG00000064651:[ENST00000262461(c.2748A>T,ggA>ggT):ENSP00000262461(p.Gly916(=))]	NA	NA	4.39E-03	2.82E-03	1.46E-02	4.39E-03	2.25E-02	NA	NA	NA	MODIFIER	NA	NA	63	61	2	70	46	20	28.57
Non-user	IOB_24	chr5	131553427	G	A	P4HA2	INTRONIC	ENSG00000072682:[ENST00000431054(c.275+18C>T)]	NA	NA	3.24E-02	5.73E-02	4.49E-02	3.23E-02	4.81E-02	NA	NA	NA	MODIFIER	NA	NA	61	60	1	62	31	29	46.77
Non-user	IOB_24	chr5	131714052	C	G	SLC22A5	INTRONIC	ENSG00000197375:[ENST00000415928(c.163-18C>G)]	NA	NA	2.00E-03	3.62E-04	1.82E-04	2.00E-03	0.00E+00	NA	NA	NA	MODIFIER	NA	NA	333	332	0	297	176	120	40.4
Non-user	IOB_24	chr5	132085115	G	C	CCNI2	INTRONIC-SS-DNR	645121:[NM_001287253.1(c.636+1G>C)]	NA	NA	4.99E-03	3.23E-03	1.63E-02	4.99E-03	2.35E-02	NA	NA	NA	HIGH	NA	NA	160	160	0	138	79	57	41.3
Non-user	IOB_24	chr5	132408967	G	A	HSPA4	MISSENSE	ENSG00000170606:[ENST00000304858(c.475G>A,Gca>Aca):ENSP00000302961(p.Ala159Thr)]	NA	NA	1.28E-02	1.87E-02	2.61E-02	1.28E-02	3.27E-02	D	BN	NA	MODERATE	N	1	177	172	0	162	85	69	42.59
Non-user	IOB_24	chr5	133481466	T	C	TCF7	SILENT	ENSG00000081059:[ENST00000321584(c.1110T>C,aaT>aaC):ENSP00000326540(p.Asn370(=))]	NA	NA	2.20E-03	4.81E-03	3.13E-03	2.20E-03	2.00E-03	NA	NA	NA	MODIFIER	NA	NA	127	126	0	92	44	48	52.17
Non-user	IOB_24	chr5	134210871	A	C	TXNDC15	MISSENSE	ENSG00000113621:[ENST00000508810(c.45A>C,caA>caC):ENSP00000424428(p.Gln15His)]	NA	NA	1.60E-03	4.65E-04	5.33E-04	1.60E-03	2.00E-03	NA	NA	NA	MODIFIER	NA	NA	144	141	1	88	45	43	48.86
Non-user	IOB_24	chr5	134724555	T	C	H2AFY	NCT	ENSG00000113648:[ENST00000508120(n.145+10197A>G)]	NA	NA	9.98E-04	NA	NA	9.98E-04	5.10E-03	NA	NA	NA	MODIFIER	NA	NA	74	74	0	66	30	35	53.03
Non-user	IOB_24	chr5	134724581	G	C	H2AFY	NCT	ENSG00000113648:[ENST00000360597(n.366+31C>G)]	NA	NA	2.58E-02	2.27E-02	5.46E-03	2.58E-02	7.20E-03	NA	NA	NA	MODIFIER	NA	NA	86	86	0	78	39	38	48.72
Non-user	IOB_24	chr5	138764284	C	A	DNAJC18	MISSENSE	ENSG00000170464:[ENST00000302060(c.316G>T,Gcc>Tcc):ENSP00000302843(p.Ala106Ser)]	NA	NA	chr5*138764284*138764284*C*T*rs772651526*NA*NA*NA	NA	NA	NA	NA	NA	NA	NA	LOW	NA	NA	223	222	0	165	159	5	3.03
Non-user	IOB_24	chr5	139838296	G	T	ANKHD1	SILENT	ENSG00000131503:[ENST00000297183(c.1329G>T,acG>acT):ENSP00000297183(p.Thr443(=))]	NA	NA	1.60E-03	8.10E-04	5.76E-03	1.60E-03	8.20E-03	NA	NA	NA	LOW	NA	NA	135	135	0	93	49	43	46.24
Non-user	IOB_24	chr5	139838296	G	T	ANKHD1-EIF4EBP3	SILENT	ENSG00000254996:[ENST00000532219(c.1329G>T,acG>acT):ENSP00000432016(p.Thr443(=))]	NA	NA	1.60E-03	8.10E-04	5.76E-03	1.60E-03	8.20E-03	NA	NA	NA	LOW	NA	NA	135	135	0	93	49	43	46.24
Non-user	IOB_24	chr5	139931629	C	G	SRA1	MISSENSE	ENSG00000213523:[ENST00000336283(c.328G>C,Gtg>Ctg):ENSP00000337513(p.Val110Leu)]	NA	chr5*139931629*139931629*COSM1166160*C*G*1*large_intestine*1	NA	9.90E-04	1.10E-03	NA	NA	NA	NA	NA	MODIFIER	NA	NA	178	178	0	82	1	79	96.34
Non-user	IOB_24	chr5	140168291	T	C	PCDHA1	MISSENSE	ENSG00000204970:[ENST00000378133(c.2416T>C,Tgg>Cgg):ENSP00000367373(p.Trp806Arg)]	NA	NA	7.59E-03	2.08E-02	8.46E-03	7.59E-03	4.10E-03	NA	NA	NA	MODIFIER	NA	NA	70	70	0	47	32	14	29.79
Non-user	IOB_24	chr5	140187102	A	G	PCDHA1	INTRONIC	ENSG00000204970:[ENST00000394633(c.1602+19625A>G)]	NA	NA	NA	5.24E-01	5.39E-01	NA	NA	NA	NA	NA	MODIFIER	NA	NA	97	97	0	79	0	79	100
Non-user	IOB_24	chr5	140187102	A	G	PCDHA2	INTRONIC	ENSG00000204969:[ENST00000526136(c.2388+10165A>G)]	NA	NA	NA	5.24E-01	5.39E-01	NA	NA	NA	NA	NA	MODIFIER	NA	NA	97	97	0	79	0	79	100
Non-user	IOB_24	chr5	140187102	A	G	PCDHA3	INTRONIC	ENSG00000255408:[ENST00000522353(c.2394+3926A>G)]	NA	NA	NA	5.24E-01	5.39E-01	NA	NA	NA	NA	NA	MODIFIER	NA	NA	97	97	0	79	0	79	100
Non-user	IOB_24	chr5	140187102	A	G	PCDHA4	SILENT	ENSG00000204967:[ENST00000356878(c.330A>G,gtA>gtG):ENSP00000349344(p.Val110(=))]	NA	NA	NA	5.24E-01	5.39E-01	NA	NA	NA	NA	NA	MODIFIER	NA	NA	97	97	0	79	0	79	100
Non-user	IOB_24	chr5	140201919	G	A	PCDHA1	INTRONIC	ENSG00000204970:[ENST00000394633(c.1602+34442G>A)]	NA	NA	7.59E-03	1.49E-02	6.00E-03	7.59E-03	4.10E-03	NA	NA	NA	MODIFIER	NA	NA	210	210	0	175	95	80	45.71
Non-user	IOB_24	chr5	140201919	G	A	PCDHA2	INTRONIC	ENSG00000204969:[ENST00000526136(c.2388+24982G>A)]	NA	NA	7.59E-03	1.49E-02	6.00E-03	7.59E-03	4.10E-03	NA	NA	NA	MODIFIER	NA	NA	210	210	0	175	95	80	45.71
Non-user	IOB_24	chr5	140201919	G	A	PCDHA3	INTRONIC	ENSG00000255408:[ENST00000522353(c.2394+18743G>A)]	NA	NA	7.59E-03	1.49E-02	6.00E-03	7.59E-03	4.10E-03	NA	NA	NA	MODIFIER	NA	NA	210	210	0	175	95	80	45.71
Non-user	IOB_24	chr5	140201919	G	A	PCDHA4	INTRONIC	ENSG00000204967:[ENST00000512229(c.2385+12762G>A)]	NA	NA	7.59E-03	1.49E-02	6.00E-03	7.59E-03	4.10E-03	NA	NA	NA	MODIFIER	NA	NA	210	210	0	175	95	80	45.71
Non-user	IOB_24	chr5	140201919	G	A	PCDHA5	MISSENSE	ENSG00000204965:[ENST00000378126(c.559G>A,Gaa>Aaa):ENSP00000367366(p.Glu187Lys)]	NA	NA	7.59E-03	1.49E-02	6.00E-03	7.59E-03	4.10E-03	NA	NA	NA	MODIFIER	NA	NA	210	210	0	175	95	80	45.71
Non-user	IOB_24	chr5	140229813	G	A	PCDHA1	INTRONIC	ENSG00000204970:[ENST00000504120(c.2394+61544G>A)]	NA	NA	1.40E-03	8.51E-04	6.04E-03	1.40E-03	7.20E-03	NA	NA	NA	MODIFIER	NA	NA	47	47	0	14	0	14	100
Non-user	IOB_24	chr5	140229813	G	A	PCDHA2	INTRONIC	ENSG00000204969:[ENST00000526136(c.2388+52876G>A)]	NA	NA	1.40E-03	8.51E-04	6.04E-03	1.40E-03	7.20E-03	NA	NA	NA	MODIFIER	NA	NA	47	47	0	14	0	14	100
Non-user	IOB_24	chr5	140229813	G	A	PCDHA3	INTRONIC	ENSG00000255408:[ENST00000522353(c.2394+46637G>A)]	NA	NA	1.40E-03	8.51E-04	6.04E-03	1.40E-03	7.20E-03	NA	NA	NA	MODIFIER	NA	NA	47	47	0	14	0	14	100
Non-user	IOB_24	chr5	140229813	G	A	PCDHA4	INTRONIC	ENSG00000204967:[ENST00000512229(c.2385+40656G>A)]	NA	NA	1.40E-03	8.51E-04	6.04E-03	1.40E-03	7.20E-03	NA	NA	NA	MODIFIER	NA	NA	47	47	0	14	0	14	100
Non-user	IOB_24	chr5	140229813	G	A	PCDHA5	INTRONIC	ENSG00000204965:[ENST00000529619(c.2352+26101G>A)]	NA	NA	1.40E-03	8.51E-04	6.04E-03	1.40E-03	7.20E-03	NA	NA	NA	MODIFIER	NA	NA	47	47	0	14	0	14	100
Non-user	IOB_24	chr5	140229813	G	A	PCDHA6	INTRONIC	ENSG00000081842:[ENST00000527624(c.1602+20535G>A)]	NA	NA	1.40E-03	8.51E-04	6.04E-03	1.40E-03	7.20E-03	NA	NA	NA	MODIFIER	NA	NA	47	47	0	14	0	14	100
Non-user	IOB_24	chr5	140229813	G	A	PCDHA7	INTRONIC	ENSG00000204963:[ENST00000525929(c.2355+13490G>A)]	NA	NA	1.40E-03	8.51E-04	6.04E-03	1.40E-03	7.20E-03	NA	NA	NA	MODIFIER	NA	NA	47	47	0	14	0	14	100
Non-user	IOB_24	chr5	140229813	G	A	PCDHA8	INTRONIC	ENSG00000204962:[ENST00000531613(c.2394+6513G>A)]	NA	NA	1.40E-03	8.51E-04	6.04E-03	1.40E-03	7.20E-03	NA	NA	NA	MODIFIER	NA	NA	47	47	0	14	0	14	100
Non-user	IOB_24	chr5	140229813	G	A	PCDHA9	MISSENSE	ENSG00000204961:[ENST00000378122(c.1733G>A,aGc>aAc):ENSP00000367362(p.Ser578Asn)]	NA	NA	1.40E-03	8.51E-04	6.04E-03	1.40E-03	7.20E-03	NA	NA	NA	MODIFIER	NA	NA	47	47	0	14	0	14	100
Non-user	IOB_24	chr5	140255578	T	G	PCDHA1	INTRONIC	ENSG00000204970:[ENST00000394633(c.1602+88101T>G)]	NA	NA	1.40E-03	8.38E-04	6.06E-03	1.40E-03	7.20E-03	D_lc	BN	NA	MODERATE	NA	NA	156	156	0	118	54	62	52.54
Non-user	IOB_24	chr5	140255578	T	G	PCDHA10	INTRONIC	ENSG00000250120:[ENST00000506939(c.1599+18346T>G)]	NA	NA	1.40E-03	8.38E-04	6.06E-03	1.40E-03	7.20E-03	D_lc	BN	NA	MODERATE	NA	NA	156	156	0	118	54	62	52.54
Non-user	IOB_24	chr5	140255578	T	G	PCDHA11	MISSENSE	56138:[XM_005268489.1(c.3053T>G,cTa>cGa):XP_005268546.1(p.Leu1018Arg)]	NA	NA	1.40E-03	8.38E-04	6.06E-03	1.40E-03	7.20E-03	D_lc	BN	NA	MODERATE	NA	NA	156	156	0	118	54	62	52.54
Non-user	IOB_24	chr5	140255578	T	G	PCDHA12	MISSENSE	ENSG00000251664:[ENST00000398631(c.521T>G,cTa>cGa):ENSP00000381628(p.Leu174Arg)]	NA	NA	1.40E-03	8.38E-04	6.06E-03	1.40E-03	7.20E-03	D_lc	BN	NA	MODERATE	NA	NA	156	156	0	118	54	62	52.54
Non-user	IOB_24	chr5	140255578	T	G	PCDHA2	INTRONIC	ENSG00000204969:[ENST00000526136(c.2388+78641T>G)]	NA	NA	1.40E-03	8.38E-04	6.06E-03	1.40E-03	7.20E-03	D_lc	BN	NA	MODERATE	NA	NA	156	156	0	118	54	62	52.54
Non-user	IOB_24	chr5	140255578	T	G	PCDHA3	INTRONIC	ENSG00000255408:[ENST00000522353(c.2394+72402T>G)]	NA	NA	1.40E-03	8.38E-04	6.06E-03	1.40E-03	7.20E-03	D_lc	BN	NA	MODERATE	NA	NA	156	156	0	118	54	62	52.54
Non-user	IOB_24	chr5	140255578	T	G	PCDHA4	INTRONIC	ENSG00000204967:[ENST00000512229(c.2385+66421T>G)]	NA	NA	1.40E-03	8.38E-04	6.06E-03	1.40E-03	7.20E-03	D_lc	BN	NA	MODERATE	NA	NA	156	156	0	118	54	62	52.54
Non-user	IOB_24	chr5	140255578	T	G	PCDHA5	INTRONIC	ENSG00000204965:[ENST00000529619(c.2352+51866T>G)]	NA	NA	1.40E-03	8.38E-04	6.06E-03	1.40E-03	7.20E-03	D_lc	BN	NA	MODERATE	NA	NA	156	156	0	118	54	62	52.54
Non-user	IOB_24	chr5	140255578	T	G	PCDHA6	INTRONIC	ENSG00000081842:[ENST00000529310(c.2394+45508T>G)]	NA	NA	1.40E-03	8.38E-04	6.06E-03	1.40E-03	7.20E-03	D_lc	BN	NA	MODERATE	NA	NA	156	156	0	118	54	62	52.54
Non-user	IOB_24	chr5	140255578	T	G	PCDHA7	INTRONIC	ENSG00000204963:[ENST00000525929(c.2355+39255T>G)]	NA	NA	1.40E-03	8.38E-04	6.06E-03	1.40E-03	7.20E-03	D_lc	BN	NA	MODERATE	NA	NA	156	156	0	118	54	62	52.54
Non-user	IOB_24	chr5	140255578	T	G	PCDHA8	INTRONIC	ENSG00000204962:[ENST00000531613(c.2394+32278T>G)]	NA	NA	1.40E-03	8.38E-04	6.06E-03	1.40E-03	7.20E-03	D_lc	BN	NA	MODERATE	NA	NA	156	156	0	118	54	62	52.54

Patel *et al.* , 2021. Whole-exome sequencing analysis of oral squamous cell carcinoma delineated by tobacco usage habits
Supplementary Table 4. List of somatic single nucleotide variants identified using whole exome sequencing of OSCC patients

Cohort	Sample	Chromosome	Genomic position	Reference allele	Alternate allele	Genesymbol	Variant type	Variant description	OncoMD	COSMIC	dbSNP	ExAC	ExAC_SAS	1000G	1000G_SAS	SIFT	Polyphen2	CONDEL	VEP	LRT	PhastCon7Way	Total reads in Normal	Reference read support in Normal	Alternative read support in Normal	Total reads in Tumor	Reference read support in Tumor	Alternate read support in Tumor	Alternate allele frequency in Tumor
Non-user	IOB_24	chr5	140255578	T	G	PCDHA9	INTRONIC	ENSG00000204961:[ENST00000532602(c.2394+25104T>G)]	NA	NA	1.40E-03	8.38E-04	6.06E-03	1.40E-03	7.20E-03	D_lc	BN	NA	MODERATE	NA	NA	156	156	0	118	54	62	52.54
Non-user	IOB_24	chr5	140256888	T	C	PCDHA1	INTRONIC	ENSG00000204970:[ENST00000504120(c.2394+88619T>C)]	NA	NA	1.40E-03	8.33E-04	6.06E-03	1.40E-03	7.20E-03	NA	NA	NA	MODIFIER	NA	NA	174	174	0	122	53	68	55.74
Non-user	IOB_24	chr5	140256888	T	C	PCDHA10	INTRONIC	ENSG00000250120:[ENST00000307360(c.2388+18867T>C)]	NA	NA	1.40E-03	8.33E-04	6.06E-03	1.40E-03	7.20E-03	NA	NA	NA	MODIFIER	NA	NA	174	174	0	122	53	68	55.74
Non-user	IOB_24	chr5	140256888	T	C	PCDHA11	SILENT	56138:[XM_005268489.1(c.4363T>C,Ttg>Ctg):XP_005268546.1(p.Leu1455(=))]	NA	NA	1.40E-03	8.33E-04	6.06E-03	1.40E-03	7.20E-03	NA	NA	NA	MODIFIER	NA	NA	174	174	0	122	53	68	55.74
Non-user	IOB_24	chr5	140256888	T	C	PCDHA12	SILENT	ENSG00000251664:[ENST00000398631(c.1831T>C,Ttg>Ctg):ENSP00000381628(p.Leu611(=))]	NA	NA	1.40E-03	8.33E-04	6.06E-03	1.40E-03	7.20E-03	NA	NA	NA	MODIFIER	NA	NA	174	174	0	122	53	68	55.74
Non-user	IOB_24	chr5	140256888	T	C	PCDHA2	INTRONIC	ENSG00000204969:[ENST00000526136(c.2388+79951T>C)]	NA	NA	1.40E-03	8.33E-04	6.06E-03	1.40E-03	7.20E-03	NA	NA	NA	MODIFIER	NA	NA	174	174	0	122	53	68	55.74
Non-user	IOB_24	chr5	140256888	T	C	PCDHA3	INTRONIC	ENSG00000255408:[ENST00000522353(c.2394+73712T>C)]	NA	NA	1.40E-03	8.33E-04	6.06E-03	1.40E-03	7.20E-03	NA	NA	NA	MODIFIER	NA	NA	174	174	0	122	53	68	55.74
Non-user	IOB_24	chr5	140256888	T	C	PCDHA4	INTRONIC	ENSG00000204967:[ENST00000512229(c.2385+67731T>C)]	NA	NA	1.40E-03	8.33E-04	6.06E-03	1.40E-03	7.20E-03	NA	NA	NA	MODIFIER	NA	NA	174	174	0	122	53	68	55.74
Non-user	IOB_24	chr5	140256888	T	C	PCDHA5	INTRONIC	ENSG00000204965:[ENST00000529619(c.2352+53176T>C)]	NA	NA	1.40E-03	8.33E-04	6.06E-03	1.40E-03	7.20E-03	NA	NA	NA	MODIFIER	NA	NA	174	174	0	122	53	68	55.74
Non-user	IOB_24	chr5	140256888	T	C	PCDHA6	INTRONIC	ENSG00000081842:[ENST00000529310(c.2394+46818T>C)]	NA	NA	1.40E-03	8.33E-04	6.06E-03	1.40E-03	7.20E-03	NA	NA	NA	MODIFIER	NA	NA	174	174	0	122	53	68	55.74
Non-user	IOB_24	chr5	140256888	T	C	PCDHA7	INTRONIC	ENSG00000204963:[ENST00000525929(c.2355+40565T>C)]	NA	NA	1.40E-03	8.33E-04	6.06E-03	1.40E-03	7.20E-03	NA	NA	NA	MODIFIER	NA	NA	174	174	0	122	53	68	55.74
Non-user	IOB_24	chr5	140256888	T	C	PCDHA8	INTRONIC	ENSG00000204962:[ENST00000531613(c.2394+33588T>C)]	NA	NA	1.40E-03	8.33E-04	6.06E-03	1.40E-03	7.20E-03	NA	NA	NA	MODIFIER	NA	NA	174	174	0	122	53	68	55.74
Non-user	IOB_24	chr5	140256888	T	C	PCDHA9	INTRONIC	ENSG00000204961:[ENST00000532602(c.2394+26414T>C)]	NA	NA	1.40E-03	8.33E-04	6.06E-03	1.40E-03	7.20E-03	NA	NA	NA	MODIFIER	NA	NA	174	174	0	122	53	68	55.74
Non-user	IOB_24	chr5	140557929	T	G	PCDHB8	MISSENSE	ENSG00000120322:[ENST00000239444(c.314T>G,cTa>cGa):ENSP00000239444(p.Leu105Arg)]	NA	NA	4.79E-03	2.83E-03	1.84E-02	4.79E-03	2.45E-02	D_lc	PoD	NA	MODERATE	NA	NA	168	168	0	64	28	36	56.25
Non-user	IOB_24	chr5	140562698	C	A	PCDHB16	SILENT	ENSG00000196963:[ENST00000361016(c.564C>A,ggC>ggA):ENSP00000354293(p.Gly188(=))]	NA	NA	NA	8.27E-06	6.06E-05	NA	NA	NA	NA	NA	LOW	NA	NA	283	282	1	209	203	6	2.87
Non-user	IOB_24	chr5	140574472	A	G	PCDHB10	MISSENSE	ENSG00000120324:[ENST00000239446(c.2347A>G,Agg>Ggg):ENSP00000239446(p.Arg783Gly)]	NA	NA	7.99E-04	5.96E-04	4.18E-03	7.99E-04	4.10E-03	T_lc	BN	NA	MODERATE	NA	NA	93	92	0	67	33	34	50.75
Non-user	IOB_24	chr5	140580451	T	C	PCDHB11	SILENT	ENSG00000197479:[ENST00000536699(c.9T>C,ttT>ttC):ENSP00000440344(p.Phe3(=))]	NA	NA	7.99E-04	7.00E-04	4.91E-03	7.99E-04	4.10E-03	NA	NA	NA	LOW	NA	NA	344	343	1	277	137	139	50.18
Non-user	IOB_24	chr5	140594442	A	G	PCDHB13	SILENT	ENSG00000187372:[ENST00000341948(c.747A>G,agA>agG):ENSP00000345491(p.Arg249(=))]	NA	NA	NA	1.55E-01	2.41E-01	NA	NA	NA	NA	NA	LOW	NA	NA	308	306	0	279	218	59	21.15
Non-user	IOB_24	chr5	140682891	A	C	SLC25A2	MISSENSE	ENSG00000120329:[ENST00000239451(c.542T>G,gTa>gGa):ENSP00000239451(p.Val181Gly)]	NA	NA	4.35E-02	3.20E-02	3.99E-02	4.35E-02	4.19E-02	D	PoD	NA	MODERATE	D	0.987	234	232	0	195	96	98	50.26
Non-user	IOB_24	chr5	140751288	C	T	PCDHGA1	INTRONIC	ENSG00000204956:[ENST00000517417(c.2421+38616C>T)]	NA	NA	2.40E-03	2.14E-03	1.53E-02	2.40E-03	1.23E-02	NA	NA	NA	MODIFIER	NA	NA	360	359	1	228	118	108	47.37
Non-user	IOB_24	chr5	140751288	C	T	PCDHGA2	INTRONIC	ENSG00000081853:[ENST00000394576(c.2424+30326C>T)]	NA	NA	2.40E-03	2.14E-03	1.53E-02	2.40E-03	1.23E-02	NA	NA	NA	MODIFIER	NA	NA	360	359	1	228	118	108	47.37
Non-user	IOB_24	chr5	140751288	C	T	PCDHGA3	INTRONIC	ENSG00000254245:[ENST00000253812(c.2424+25264C>T)]	NA	NA	2.40E-03	2.14E-03	1.53E-02	2.40E-03	1.23E-02	NA	NA	NA	MODIFIER	NA	NA	360	359	1	228	118	108	47.37
Non-user	IOB_24	chr5	140751288	C	T	PCDHGA4	INTRONIC	56111:[NM_018917.3(c.2514+14100C>T)]	NA	NA	2.40E-03	2.14E-03	1.53E-02	2.40E-03	1.23E-02	NA	NA	NA	MODIFIER	NA	NA	360	359	1	228	118	108	47.37
Non-user	IOB_24	chr5	140751288	C	T	PCDHGA5	INTRONIC	ENSG00000253485:[ENST00000518069(c.2421+4970C>T)]	NA	NA	2.40E-03	2.14E-03	1.53E-02	2.40E-03	1.23E-02	NA	NA	NA	MODIFIER	NA	NA	360	359	1	228	118	108	47.37
Non-user	IOB_24	chr5	140751288	C	T	PCDHGB1	INTRONIC	ENSG00000254221:[ENST00000523390(c.2409+19052C>T)]	NA	NA	2.40E-03	2.14E-03	1.53E-02	2.40E-03	1.23E-02	NA	NA	NA	MODIFIER	NA	NA	360	359	1	228	118	108	47.37
Non-user	IOB_24	chr5	140751288	C	T	PCDHGB2	INTRONIC	ENSG00000253910:[ENST00000522605(c.2421+9165C>T)]	NA	NA	2.40E-03	2.14E-03	1.53E-02	2.40E-03	1.23E-02	NA	NA	NA	MODIFIER	NA	NA	360	359	1	228	118	108	47.37
Non-user	IOB_24	chr5	140751288	C	T	PCDHGB3	MISSENSE	ENSG00000262209:[ENST00000576222(c.1327C>T,Ctt>Ttt):ENSP00000461862(p.Leu443Phe)]	NA	NA	2.40E-03	2.14E-03	1.53E-02	2.40E-03	1.23E-02	NA	NA	NA	MODIFIER	NA	NA	360	359	1	228	118	108	47.37
Non-user	IOB_24	chr5	140763100	C	T	PCDHGA1	INTRONIC	ENSG00000204956:[ENST00000517417(c.2421+50428C>T)]	NA	NA	4.85E-02	2.28E-02	3.83E-02	4.85E-02	5.52E-02	NA	NA	NA	MODIFIER	NA	NA	219	218	0	177	88	88	49.72
Non-user	IOB_24	chr5	140763100	C	T	PCDHGA2	INTRONIC	ENSG00000081853:[ENST00000394576(c.2424+42138C>T)]	NA	NA	4.85E-02	2.28E-02	3.83E-02	4.85E-02	5.52E-02	NA	NA	NA	MODIFIER	NA	NA	219	218	0	177	88	88	49.72
Non-user	IOB_24	chr5	140763100	C	T	PCDHGA3	INTRONIC	ENSG00000254245:[ENST00000253812(c.2424+37076C>T)]	NA	NA	4.85E-02	2.28E-02	3.83E-02	4.85E-02	5.52E-02	NA	NA	NA	MODIFIER	NA	NA	219	218	0	177	88	88	49.72
Non-user	IOB_24	chr5	140763100	C	T	PCDHGA4	INTRONIC	56111:[NM_018917.3(c.2514+25912C>T)]	NA	NA	4.85E-02	2.28E-02	3.83E-02	4.85E-02	5.52E-02	NA	NA	NA	MODIFIER	NA	NA	219	218	0	177	88	88	49.72
Non-user	IOB_24	chr5	140763100	C	T	PCDHGA5	INTRONIC	ENSG00000253485:[ENST00000518069(c.2421+16782C>T)]	NA	NA	4.85E-02	2.28E-02	3.83E-02	4.85E-02	5.52E-02	NA	NA	NA	MODIFIER	NA	NA	219	218	0	177	88	88	49.72
Non-user	IOB_24	chr5	140763100	C	T	PCDHGA6	INTRONIC	56109:[XM_005268482.1(c.4935+7026C>T)]	NA	NA	4.85E-02	2.28E-02	3.83E-02	4.85E-02	5.52E-02	NA	NA	NA	MODIFIER	NA	NA	219	218	0	177	88	88	49.72
Non-user	IOB_24	chr5	140763100	C	T	PCDHGA7	MISSENSE	ENSG00000253537:[ENST00000518325(c.634C>T,Ctc>Ttc):ENSP00000430024(p.Leu212Phe)]	NA	NA	4.85E-02	2.28E-02	3.83E-02	4.85E-02	5.52E-02	NA	NA	NA	MODIFIER	NA	NA	219	218	0	177	88	88	49.72
Non-user	IOB_24	chr5	140763100	C	T	PCDHGB1	INTRONIC	ENSG00000254221:[ENST00000523390(c.2409+30864C>T)]	NA	NA	4.85E-02	2.28E-02	3.83E-02	4.85E-02	5.52E-02	NA	NA	NA	MODIFIER	NA	NA	219	218	0	177	88	88	49.72
Non-user	IOB_24	chr5	140763100	C	T	PCDHGB2	INTRONIC	ENSG00000253910:[ENST00000522605(c.2421+20977C>T)]	NA	NA	4.85E-02	2.28E-02	3.83E-02	4.85E-02	5.52E-02	NA	NA	NA	MODIFIER	NA	NA	219	218	0	177	88	88	49.72
Non-user	IOB_24	chr5	140763100	C	T	PCDHGB3	INTRONIC	ENSG00000262209:[ENST00000576222(c.2415+10724C>T)]	NA	NA	4.85E-02	2.28E-02	3.83E-02	4.85E-02	5.52E-02	NA	NA	NA	MODIFIER	NA	NA	219	218	0	177	88	88	49.72
Non-user	IOB_24	chr5	140963581	G	T	DIAPH1	INTRONIC	ENSG00000131504:[ENST00000520569(c.240+110C>A)]	NA	NA	3.99E-03	NA	NA	3.99E-03	2.04E-02	NA	NA	NA	MODIFIER	NA	NA	93	92	0	60	28	32	53.33
Non-user	IOB_24	chr5	141019830	C	G	FCHSD1	EXONIC-NC	ENSG00000197948:[ENST00000520747(n.2221G>C)]	NA	NA	NA	2.03E-01	3.09E-01	NA	NA	NA	NA	NA	MODIFIER	NA	NA	226	225	0	187	118	68	36.36
Non-user	IOB_24	chr5	141019830	C	G	RELL2	MISSENSE	ENSG00000164620:[ENST00000297164(c.847C>G,Cag>Gag):ENSP00000297164(p.Gln283Glu)]	NA	NA	NA	2.03E-01	3.09E-01	NA	NA	NA	NA	NA	MODIFIER	NA	NA	226	225	0	187	118	68	36.36
Non-user	IOB_24	chr5	141029987	G	C	FCHSD1	INTRONIC	ENSG00000197948:[ENST00000522126(c.-103-16C>G)]	NA	NA	2.00E-04	8.28E-06	6.06E-05	2.00E-04	1.00E-03	NA	NA	NA	MODIFIER	NA	NA	57	57	0	41	28	12	29.27
Non-user	IOB_24	chr5	145616877	A	G	RBM27	SILENT	ENSG00000091009:[ENST00000265271(c.1161A>G,caA>caG):ENSP00000265271(p.Gln387(=))]	NA	NA	NA	7.48E-05	1.82E-04	NA	NA	NA	NA	NA	LOW	NA	1	240	239	0	194	103	90	46.39
Non-user	IOB_24	chr5	147481085	A	C	SPINK5	INTRONIC	ENSG00000133710:[ENST00000508733(c.1245+86A>C)]	NA	NA	4.97E-02	NA	NA	4.97E-02	6.03E-02	NA	NA	NA	MODIFIER	NA	NA	72	71	1	87	0	87	100
Non-user	IOB_24	chr5	148406584	C	T	SH3TC2	MISSENSE	ENSG00000169247:[ENST00000504690(c.2711G>A,cGa>cAa):ENSP00000425627(p.Arg904Gln)]	NA	NA	NA	1.65E-05	1.21E-04	NA	NA	NA	NA	NA	MODIFIER	NA	NA	93	93	0	74	42	32	43.24
Non-user	IOB_24	chr5	148680020	A	T	AFAP1L1	NCT	ENSG00000157510:[ENST00000522492(n.303+103A>T)]	NA	NA	2.86E-02	NA	NA	2.86E-02	2.56E-02	NA	NA	NA	MODIFIER	NA	NA	38	38	0	42	20	22	52.38
Non-user	IOB_24	chr5	149357444	A	C	SLC26A2	MISSENSE	ENSG00000155850:[ENST00000286298(c.229A>C,Aat>Cat):ENSP00000286298(p.Asn77His)]	NA	NA	1.02E-02	5.14E-03	3.52E-02	1.02E-02	4.70E-02	T	BN	NA	MODERATE	N	0.984	351	348	1	282	134	148	52.48
Non-user	IOB_24	chr5	149360566	A	G	SLC26A2	SILENT	ENSG00000155850:[ENST00000286298(c.1410A>G,gtA>gtG):ENSP00000286298(p.Val470(=))]	NA	NA	9.98E-03	5.05E-03	3.51E-02	9.98E-03	4.70E-02	NA	NA	NA	LOW	NA	NA	376	376	0	266	139	127	47.74
Non-user	IOB_24	chr5	149416450	G	A	HMGXB3	SILENT	ENSG00000113716:[ENST00000502717(c.2235G>A,ccG>ccA):ENSP00000421917(p.Pro745(=))]	NA	NA	9.59E-03	1.32E-02	3.48E-02	9.58E-03	4.29E-02	NA	NA	NA	MODIFIER	NA	NA	77	77	0	56	29	27	48.21
Non-user	IOB_24	chr5	149428203	A	G	HMGXB3	SILENT	ENSG00000113716:[ENST00000503427(c.3060A>G,ccA>ccG):ENSP00000422231(p.Pro1020(=))]	NA	NA	8.99E-03	1.25E-02	3.39E-02	8.99E-03	4.29E-02	NA	NA	NA	MODIFIER	NA	NA	69	69	0	51	25	26	50.98
Non-user	IOB_24	chr5	149504306	G	A	PDGFRB	SILENT	ENSG00000113721:[ENST00000261799(c.1896C>T,gcC>gcT):ENSP00000261799(p.Ala632(=))]	NA	NA	1.40E-03	6.69E-04	5.66E-03	1.40E-03	7.20E-03	NA	NA	NA	LOW	NA	NA	81	81	0	88	40	48	54.55
Non-user	IOB_24	chr5	149907779	A	G	NDST1	SILENT	ENSG00000070614:[ENST00000261797(c.927A>G,ccA>ccG):ENSP00000261797(p.Pro309(=))]	NA	NA	9.39E-03	7.61E-03	4.61E-02	9.38E-03	4.60E-02	NA	NA	NA	LOW	NA	NA	501	500	0	395	228	167	42.28
Non-user	IOB_24	chr5	150138388	C	A	DCTN4	INTRONIC	ENSG00000132912:[ENST00000446090(c.135+33G>T)]	NA	NA	NA	2.48E-05	1.83E-04	NA	NA	NA	NA	NA	MODIFIER	NA	NA	139	137	1	99	63	34	34.34
Non-user	IOB_24	chr5	150436422	C	T	TNIP1	MISSENSE	ENSG00000145901:[ENST00000315050(c.532G>A,Ggc>Agc):ENSP00000317891(p.Gly178Ser)]	NA	NA	1.64E-02	2.89E-02	6.67E-02	1.64E-02	4.60E-02	T	BN	NA	MODERATE	NA	NA	352	350	0	267	137	130	48.69
Non-user	IOB_24	chr5	150481162	G	A	ANXA6	INTRONIC	ENSG00000197043:[ENST00000356496(c.1945-94C>T)]	NA	NA	4.09E-02	NA	NA	4.09E-02	6.24E-02	NA	NA	NA	MODIFIER	NA	NA	124	124	0	131	61	70	53.44
Non-user	IOB_24	chr5	150663655	C	T	SLC36A3	SILENT	ENSG00000186334:[ENST00000377713(c.1047G>A,aaG>aaA):ENSP00000366942(p.Lys349(=))]	NA	chr5*150663655*150663655*COSM3697076*C*T*2*large_intestine*2	1.98E-02	3.33E-02	4.16E-02	1.98E-02	4.19E-02	NA	NA	NA	LOW	NA	0.979	87	84	0	75	31	43	57.33
Non-user	IOB_24	chr5	150666946	C	T	SLC36A3	MISSENSE	ENSG00000186334:[ENST00000377713(c.692G>A,cGt>cAt):ENSP00000366942(p.Arg231His)]	NA	chr5*150666946*150666946*COSM3697077*C*T*1*large_intestine*1	1.98E-02	3.34E-02	4.25E-02	1.98E-02	4.19E-02	T	PoD	NA	MODERATE	D	0.845	85	85	0	74	47	27	36.49
Non-user	IOB_24	chr5	150867719	C	T	SLC36A1	SILENT	ENSG00000123643:[ENST00000243389(c.1335C>T,ttC>ttT):ENSP00000243389(p.Phe445(=))]	NA	NA	3.77E-02	4.43E-02	3.91E-02	3.77E-02	3.48E-02	NA	NA	NA	LOW	NA	0	132	131	0	129	73	55	42.64
Non-user	IOB_24	chr5	150891772	C	G	CTC-251D13.1	EXONIC-NC	ENSG00000271795:[ENST00000606930(n.2759C>G)]	NA	NA	3.34E-02	3.80E-02	2.63E-02	3.33E-02	3.07E-02	D	BN	NA	MODERATE	NA	NA	108	108	0	92	55	37	40.22
Non-user	IOB_24	chr5	150891772	C	G	FAT2	MISSENSE	ENSG00000086570:[ENST00000261800(c.11859G>C,caG>caC):ENSP00000261800(p.Gln3953His)]	NA	NA	3.34E-02	3.80E-02	2.63E-02	3.33E-02	3.07E-02	D	BN	NA	MODERATE	NA	NA	108	108	0	92	55	37	40.22
Non-user	IOB_24	chr5	150931082	C	G	FAT2	SILENT	ENSG00000086570:[ENST00000261800(c.4242G>C,tcG>tcC):ENSP00000261800(p.Ser1414(=))]	NA	NA	1.44E-02	2.19E-02	5.41E-02	1.44E-02	3.78E-02	NA	NA	NA	LOW	NA	NA	74	74	0	47	17	30	63.83
Non-user	IOB_24	chr5	153405920	C	T	FAM114A2	INTRONIC	10827:[XM_005268359.1(c.931+39G>A)]	NA	NA	3.59E-02	3.27E-02	8.79E-02	3.59E-02	8.18E-02	NA	NA	NA	MODIFIER	NA	NA	37	37	0	32	11	21	65.62
Non-user	IOB_24	chr5	161528378	T	G	GABRG2	INTRONIC	ENSG00000113327:[ENST00000523372(c.*596+55T>G)]	NA	NA	9.98E-03	NA	NA	9.98E-03	7.20E-03	NA	NA	NA	MODIFIER	NA	NA	236	236	0	181	93	88	48.62
Non-user	IOB_24	chr5	162911075	C	A	HMMR	MISSENSE-SS-PRX	ENSG00000072571:[ENST00000432118(c.1525C>A,Cta>Ata):ENSP00000402673(p.Leu509Ile)]	NA	NA	3.47E-02	9.45E-02	4.54E-02	3.47E-02	3.68E-02	D	BN	NA	MODERATE	N	1	38	37	0	29	0	29	100
Non-user	IOB_24	chr5	162911075	C	A	RP11-80G7.1	NCT	ENSG00000251018:[ENST00000521666(n.593-797G>T)]	NA	NA	3.47E-02	9.45E-02	4.54E-02	3.47E-02	3.68E-02	D	BN	NA	MODERATE	N	1	38	37	0	29	0	29	100
Non-user	IOB_24	chr5	162918091	A	G	HMMR	INTRONIC	ENSG00000072571:[ENST00000393915(c.2126-27A>G)]	NA	NA	1.40E-03	3.61E-03	2.57E-03	1.40E-03	0.00E+00	NA	NA	NA	MODIFIER	NA	NA	29	29	0	51	22	29	56.86
Non-user	IOB_24	chr5	162918091	A	G	RP11-80G7.1	NCT	ENSG00000251018:[ENST00000514724(n.468-1702T>C)]	NA	NA	1.40E-03	3.61E-03	2.57E-03	1.40E-03	0.00E+00	NA	NA	NA	MODIFIER	NA	NA	29	29	0	51	22	29	56.86
Non-user	IOB_24	chr5	167835608	C	T	WWC1	SILENT	ENSG00000113645:[ENST00000524228(c.146C>T,Cta>Tta):ENSP00000429339(p.Leu50(=))]	NA	NA	1.12E-02	1.24E-02	2.94E-02	1.12E-02	3.78E-02	NA	NA	NA	LOW	NA	0.001	216	215	0	150	67	80	53.33
Non-user	IOB_24	chr5	167837016	C	T	WWC1	INTRONIC	ENSG00000113645:[ENST00000524228(c.270+16C>T)]	NA	NA	1.06E-02	8.16E-03	3.19E-02	1.06E-02	3.48E-02	NA	NA	NA	MODIFIER	NA	NA	111	111	0	96	58	38	39.58
Non-user	IOB_24	chr5	167929035	G	A	RARS	MISSENSE	ENSG00000113643:[ENST00000231572(c.982G>A,Gtc>Atc):ENSP00000231572(p.Val328Ile)]	NA	NA	1.04E-02	8.42E-03	3.57E-02	1.04E-02	4.09E-02	T	BN	NA	MODERATE	N	1	23	23	0	17	10	7	41.18
Non-user	IOB_24	chr5	169126570	C	T	DOCK2	NCT	ENSG00000134516:[ENST00000523684(n.327+108C>T)]	NA	NA	2.00E-03	NA	NA	2.00E-03	1.00E-03	NA	NA	NA	MODIFIER	NA	NA	63	62	0	29	17	12	41.38
Non-user	IOB_24	chr5	170338163	G	A	RANBP17	INTRONIC	ENSG00000204764:[ENST00000389118(c.760+25G>A)]	NA	NA	1.96E-02	3.81E-02	5.97E-02	1.96E-02	5.32E-02	NA	NA	NA	MODIFIER	NA	NA	39	39	0	35	21	14	40
Non-user	IOB_24	chr5	172395581	C	T	RPL26L1	SILENT	ENSG00000037241:[ENST00000265100(c.288C>T,caC>caT):ENSP00000265100(p.His96(=))]	NA	NA	2.96E-02	3.07E-02	7.22E-02	2.96E-02	7.67E-02	NA	NA	NA	LOW	NA	0.243	168	168	0	137	72	65	47.45
Non-user	IOB_24	chr5	173428228	T	C	C5orf47	EXONIC-NC	ENSG00000185056:[ENST00000522195(n.266T>C)]	NA	NA	3.06E-02	5.80E-02	5.84E-02	3.06E-02	4.40E-02	NA	NA	NA	MODIFIER	NA	NA	77	77	0	36	20	16	44.44
Non-user	IOB_24	chr5	175353627	A	G	RP11-91H12.4	NCT	ENSG00000250820:[ENST00000502813(n.88-19205T>C)]	NA	NA	NA	1.76E-01	1.84E-01	NA	NA	NA	NA	NA	MODIFIER	NA	NA	109	108	1	65	34	31	47.69
Non-user	IOB_24	chr5	175353627	A	G	THOC3	3UTR	ENSG00000051596:[ENST00000515016(c.*11T>C)]	NA	NA	NA	1.76E-01	1.84E-01	NA	NA	NA	NA	NA	MODIFIER	NA	NA	109	108	1	65	34	31	47.69
Non-user	IOB_24	chr5	175530214	C	T	FAM153B	INTRONIC	ENSG00000182230:[ENST00000253490(c.665-16C>T)]	NA	NA	NA	9.98E-02	9.68E-02	NA	NA	NA	NA	NA	MODIFIER	NA	NA	366	361	1	303	241	62	20.46
Non-user	IOB_24	chr5	175958677	C	T	RNF44	INTRONIC	ENSG00000146083:[ENST00000274811(c.298-46G>A)]	NA	NA	5.99E-04	2.73E-03	9.66E-05	5.99E-04	0.00E+00	NA	NA	NA	MODIFIER	NA	NA	112	112	0	103	58	43	41.75
Non-user	IOB_24	chr5	176637149	G	A	NSD1	SILENT	ENSG00000165671:[ENST00000361032(c.1440G>A,gaG>gaA):ENSP00000354310(p.Glu480(=))]	NA	NA	NA	1.76E-01	2.22E-01	NA	NA	NA	NA	NA	LOW	NA	1	224	224	0	153	77	75	49.02
Non-user	IOB_24	chr5	176637192	T	C	NSD1	SILENT	ENSG00000165671:[ENST00000347982(c.985T>C,Ttg>Ctg):ENSP00000343209(p.Leu329(=))]	NA	NA	1.34E-02	1.29E-02	5.82E-02	1.34E-02	5.73E-02	NA	NA	NA	LOW	NA	1	227	227	0	153	82	70	45.75
Non-user	IOB_24	chr5	176831544	G	A	F12	SILENT	ENSG00000131187:[ENST00000253496(c.756C>T,gcC>gcT):ENSP00000253496(p.Ala252(=))]	NA	NA	2.76E-02	2.26E-02	5.19E-02	2.76E-02	5.01E-02	NA	NA	NA	MODIFIER	NA	NA	246	246	0	183	102	81	44.26
Non-user	IOB_24	chr5	176831544	G	A	GRK6	5UTR	ENSG00000198055:[ENST00000506296(c.-59G>A)]	NA	NA	2.76E-02	2.26E-02	5.19E-02	2.76E-02	5.01E-02	NA	NA	NA	MODIFIER	NA	NA	246	246	0	183	102	81	44.26
Non-user	IOB_24	chr5	176832242	C	T	F12	INTRONIC	ENSG00000131187:[ENST00000253496(c.398-56G>A)]	NA	NA	1.12E-02	NA	NA	1.12E-02	5.01E-02	NA	NA	NA	MODIFIER	NA	NA	136	136	0	99	58	40	40.4
Non-user	IOB_24	chr5	176832242	C	T	GRK6	INTRONIC	ENSG00000198055:[ENST00000506296(c.-45+684C>T)]	NA	NA	1.12E-02	NA	NA	1.12E-02	5.01E-02	NA	NA	NA	MODIFIER	NA	NA	136	136	0	99	58	40	40.4
Non-user	IOB_24	chr5	177019695	T	C	TMED9	NCT	ENSG00000184840:[ENST00000507578(n.26+19T>C)]	NA	NA	NA	8.97E-04	1.11E-04	NA	NA	NA	NA	NA	MODIFIER	NA	NA	188	182	2	127	70	40	31.5
Non-user	IOB_24	chr5	177019698	T	C	TMED9	INTRONIC	ENSG00000184840:[ENST00000332598(c.285+22T>C)]	NA	NA	NA	6.63E-05	0.00E+00	NA	NA	NA	NA	NA	MODIFIER	NA	NA	187	182	1	130	74	38	29.23
Non-user	IOB_24	chr5	178310142	G	A	ZNF354B	MISSENSE	ENSG00000178338:[ENST00000322434(c.689G>A,cGt>cAt):ENSP00000327143(p.Arg230His)]	NA	NA	1.80E-03	5.13E-03	5.59E-03	1.80E-03	5.10E-03	T	PoD	NA	MODERATE	NA	NA	62	62	0	58	37	21	36.21
Non-user	IOB_24	chr5	178373819	G	A	ZNF454	NCT	ENSG00000178187:[ENST00000522827(n.525-79G>A)]	NA	NA	7.99E-04	NA	NA	7.99E-04	4.10E-03	NA	NA	NA	MODIFIER	NA	NA	212	211	0	157	95	62	39.49
Non-user	IOB_24	chr5	178507048	C	G	ZNF354C	MISSENSE	ENSG00000177932:[ENST00000315475(c.1615C>G,Ctt>Gtt):ENSP00000324064(p.Leu539Val)]	NA	NA	3.44E-02	5.32E-02	1.20E-01	3.43E-02	8.69E-02	D	BN	NA	MODERATE	NA	NA	200	200	0	90	59	31	34.44
Non-user	IOB_24	chr5	179195867	G	A	MAML1	MISSENSE	ENSG00000161021:[ENST00000292599(c.1748G>A,aGt>aAt):ENSP00000292599(p.Ser583Asn)]	NA	NA	2.32E-02	4.69E-02	6.64E-02	2.32E-02	5.73E-02	T	BN	NA	MODERATE	N	0	112	111	0	107	59	47	43.93
Non-user	IOB_24	chr5	179280379	T	C	C5orf45	MISSENSE	ENSG00000161010:-SS-PRX[ENST00000292586(c.125A>G,cAg>cGg):ENSP00000292586(p.Gln42Arg)]	NA	NA	NA	6.37E-01	6.22E-01	NA	NA	NA	NA	NA	LOW	NA	NA	136	135	0	85	41	43	50.59
Non-user	IOB_24	chr5	179779345	A	G	GFPT2	INTRONIC	9945:[XM_005266017.1(c.-457+866T>C)]	NA	NA	NA	5.60E-04	3.45E-04	NA	NA	NA	NA	NA	MODIFIER	NA	NA	49	40	0	44	31	6	13.64

Patel *et al.* , 2021. Whole-exome sequencing analysis of oral squamous cell carcinoma delineated by tobacco usage habits
Supplementary Table 4. List of somatic single nucleotide variants identified using whole exome sequencing of OSCC patients

Cohort	Sample	Chromosome	Genomic position	Reference allele	Alternate allele	Genesymbol	Variant type	Variant description	OncoMD	COSMIC	dbSNP	ExAC	ExAC_SAS	1000G	1000G_SAS	SIFT	Polyphen2	CONDEL	VEP	LRT	PhastCon7Way	Total reads in Normal	Reference read support in Normal	Alternative read support in Normal	Total reads in Tumor	Reference read support in Tumor	Alternate read support in Tumor	Alternate allele frequency in Tumor
Non-user	IOB_24	chr5	180288240	A	C	ZFP62	5UTR	ENSG00000196670:[ENST00000506439(c.-197T>G)]	NA	NA	3.59E-03	NA	NA	3.59E-03	5.10E-03	NA	NA	NA	MODIFIER	NA	NA	49	49	0	54	34	20	37.04
Non-user	IOB_24	chr5	180669370	A	G	GNB2L1	NCT	ENSG00000204628:[ENST00000502548(n.226-25T>C)]	NA	NA	chr5*180669366*180669370*GAAAA*G*rs748813655*NA*NA*NA|chr5*180669370*180669370*A*C,G*rs2287715*NA*NA*NA	3.32E-01	4.05E-01	NA	NA	NA	NA	NA	MODIFIER	NA	NA	67	66	0	66	32	34	51.52
Non-user	IOB_24	chr6	1312998	T	C	FOXQ1	MISSENSE	ENSG00000164379:[ENST00000296839(c.59T>C,cTg>cCg):ENSP00000296839(p.Leu20Pro)]	NA	NA	3.04E-02	5.48E-02	1.00E-01	3.04E-02	5.83E-02	T	PrD	NA	MODERATE	U	0	109	109	0	55	29	26	47.27
Non-user	IOB_24	chr6	4995464	C	T	RPP40	MISSENSE	ENSG00000124787:[ENST00000464646(c.760G>A,Gtt>Att):ENSP00000419431(p.Val254Ile)]	NA	chr6*4995464*4995464*COSM1444990*C*T*1*large_intestine*1	4.77E-02	4.29E-02	8.57E-02	4.77E-02	1.15E-01	D	PrD	NA	MODERATE	D	1	124	122	0	90	49	41	45.56
Non-user	IOB_24	chr6	4996543	C	T	RPP40	MISSENSE	ENSG00000124787:[ENST00000380051(c.671G>A,aGc>aAc):ENSP00000369391(p.Ser224Asn)]	NA	NA	4.65E-02	3.43E-02	5.02E-02	4.65E-02	5.32E-02	D	BN	NA	MODERATE	D	0.963	67	67	0	43	24	19	44.19
Non-user	IOB_24	chr6	5000031	C	T	RPP40	INTRONIC	ENSG00000124787:[ENST00000479782(c.*128+12G>A)]	NA	NA	2.78E-02	2.78E-02	4.85E-02	2.78E-02	5.11E-02	NA	NA	NA	MODIFIER	NA	NA	43	43	0	52	24	28	53.85
Non-user	IOB_24	chr6	10529449	C	G	GCNT2	MISSENSE	ENSG00000111846:[ENST00000379597(c.305C>G,aCc>aGc):ENSP00000368917(p.Thr102Ser)]	NA	NA	6.59E-03	9.92E-03	2.05E-02	6.59E-03	1.64E-02	NA	NA	NA	MODIFIER	NA	NA	274	273	0	181	90	91	50.28
Non-user	IOB_24	chr6	10874806	T	C	GCM2	MISSENSE	ENSG00000124827:[ENST00000379491(c.943A>G,Aat>Gat):ENSP00000368805(p.Asn315Asp)]	NA	NA	2.40E-03	3.49E-03	6.78E-03	2.40E-03	4.10E-03	NA	NA	NA	MODIFIER	NA	NA	341	340	1	237	120	115	48.52
Non-user	IOB_24	chr6	10874806	T	C	RP11-637O19.3	INTRONIC	ENSG00000272162:[ENST00000480294(c.101-16940T>C)]	NA	NA	2.40E-03	3.49E-03	6.78E-03	2.40E-03	4.10E-03	NA	NA	NA	MODIFIER	NA	NA	341	340	1	237	120	115	48.52
Non-user	IOB_24	chr6	10874806	T	C	SYCP2L	INTRONIC	ENSG00000153157:[ENST00000543878(c.-468-16940T>C)]	NA	NA	2.40E-03	3.49E-03	6.78E-03	2.40E-03	4.10E-03	NA	NA	NA	MODIFIER	NA	NA	341	340	1	237	120	115	48.52
Non-user	IOB_24	chr6	15524698	T	C	DTNBP1	SILENT	ENSG00000047579:[ENST00000338950(c.870A>G,ccA>ccG):ENSP00000344718(p.Pro290(=))]	NA	NA	3.24E-02	3.57E-02	7.16E-02	3.23E-02	8.38E-02	NA	NA	NA	MODIFIER	NA	NA	153	153	0	131	60	70	53.44
Non-user	IOB_24	chr6	16290641	C	T	GMPR	INTRONIC	ENSG00000137198:[ENST00000259727(c.698-52C>T)]	NA	NA	4.39E-03	NA	NA	4.39E-03	8.20E-03	NA	NA	NA	MODIFIER	NA	NA	142	141	0	122	71	51	41.8
Non-user	IOB_24	chr6	17601213	C	T	FAM8A1	SILENT	ENSG00000137414:[ENST00000259963(c.573C>T,gcC>gcT):ENSP00000259963(p.Ala191(=))]	NA	NA	9.78E-03	2.79E-02	1.62E-02	9.78E-03	1.33E-02	NA	NA	NA	LOW	NA	NA	143	143	0	133	65	67	50.38
Non-user	IOB_24	chr6	17675553	T	C	NUP153	SILENT	ENSG00000124789:[ENST00000262077(c.630A>G,ccA>ccG):ENSP00000262077(p.Pro210(=))]	NA	NA	1.38E-02	1.91E-02	1.48E-02	1.38E-02	1.43E-02	NA	NA	NA	LOW	NA	NA	156	155	1	100	53	42	42
Non-user	IOB_24	chr6	24570166	G	A	KIAA0319	SILENT	ENSG00000137261:[ENST00000430948(c.1821C>T,gaC>gaT):ENSP00000401086(p.Asp607(=))]	NA	NA	2.00E-04	9.90E-05	6.68E-04	2.00E-04	1.00E-03	NA	NA	NA	LOW	NA	1	112	112	0	87	47	40	45.98
Non-user	IOB_24	chr6	25851049	C	A	SLC17A3	MISSENSE	ENSG00000124564:[ENST00000360657(c.535G>T,Gtt>Ttt):ENSP00000353873(p.Val179Phe)]	NA	NA	2.00E-03	1.38E-03	5.21E-03	2.00E-03	7.20E-03	D	PoD	NA	MODERATE	N	0	177	177	0	109	63	45	41.28
Non-user	IOB_24	chr6	26204838	A	C	NA	INTERGENIC	NA	NA	NA	NA	5.37E-04	2.32E-03	NA	NA	NA	NA	NA	MODIFIER	NA	NA	169	167	0	113	61	52	46.02
Non-user	IOB_24	chr6	27278179	G	T	POM121L2	MISSENSE	ENSG00000158553:[ENST00000444565(c.1771C>A,Ccc>Acc):ENSP00000392726(p.Pro591Thr)]	NA	NA	1.92E-02	2.45E-02	2.05E-02	1.92E-02	1.94E-02	D	PrD	NA	MODERATE	N	0.056	182	181	1	150	92	57	38
Non-user	IOB_24	chr6	27279021	C	G	POM121L2	MISSENSE	ENSG00000158553:[ENST00000429945(c.71G>C,gGa>gCa):ENSP00000415181(p.Gly24Ala)]	NA	NA	2.02E-02	2.57E-02	2.05E-02	2.02E-02	1.94E-02	T	BN	NA	MODERATE	N	0	77	77	0	61	41	20	32.79
Non-user	IOB_24	chr6	27279805	G	T	POM121L2	MISSENSE	ENSG00000158553:[ENST00000377451(c.145C>A,Cct>Act):ENSP00000366671(p.Pro49Thr)]	NA	NA	1.96E-02	3.00E-02	3.26E-02	1.96E-02	1.94E-02	D	PoD	NA	MODERATE	N	0.709	293	292	0	238	133	104	43.7
Non-user	IOB_24	chr6	27293107	G	A	VN1R10P	EXONIC-NC	387316:[NR_045612.1(n.568G>A)]	NA	NA	1.98E-02	2.46E-02	1.98E-02	1.98E-02	1.94E-02	NA	NA	NA	MODIFIER	NA	NA	308	307	0	244	137	104	42.62
Non-user	IOB_24	chr6	27293114	T	A	VN1R10P	EXONIC-NC	387316:[NR_045612.1(n.575T>A)]	NA	NA	1.98E-02	2.46E-02	1.96E-02	1.98E-02	1.94E-02	NA	NA	NA	MODIFIER	NA	NA	293	291	1	235	132	103	43.83
Non-user	IOB_24	chr6	27293147	T	A	VN1R10P	EXONIC-NC	ENSG00000220758:[ENST00000447106(n.475T>A)]	NA	NA	1.98E-02	2.45E-02	1.94E-02	1.98E-02	1.94E-02	NA	NA	NA	MODIFIER	NA	NA	238	238	0	185	97	88	47.57
Non-user	IOB_24	chr6	27293478	T	C	VN1R10P	EXONIC-NC	ENSG00000220758:[ENST00000401407(n.900T>C)]	NA	NA	1.98E-02	2.54E-02	2.06E-02	1.98E-02	1.94E-02	NA	NA	NA	MODIFIER	NA	NA	125	125	0	97	53	43	44.33
Non-user	IOB_24	chr6	27368209	C	T	ZNF391	SILENT	ENSG00000124613:[ENST00000244576(c.60C>T,aaC>aaT):ENSP00000244576(p.Asn20(=))]	NA	NA	1.94E-02	2.44E-02	1.90E-02	1.94E-02	1.94E-02	NA	NA	NA	LOW	NA	NA	96	95	1	56	29	27	48.21
Non-user	IOB_24	chr6	27424566	G	A	ZNF184	NCT	7738:[NR_144688.1(n.583+39C>T)]	NA	NA	2.08E-02	2.48E-02	1.90E-02	2.08E-02	1.94E-02	NA	NA	NA	MODIFIER	NA	NA	77	77	0	39	22	17	43.59
Non-user	IOB_24	chr6	27776002	C	T	HIST1H2AI	SILENT	ENSG00000196747:[ENST00000358739(c.15C>T,ggC>ggT):ENSP00000351589(p.Gly5(=))]	NA	NA	NA	8.39E-02	7.25E-02	NA	NA	NA	NA	NA	LOW	NA	NA	186	185	0	210	182	28	13.33
Non-user	IOB_24	chr6	27792163	G	A	HIST1H4J	SILENT	ENSG00000197238:[ENST00000355057(c.261G>A,gtG>gtA):ENSP00000347168(p.Val87(=))]	NA	NA	4.41E-02	2.79E-02	5.90E-02	4.41E-02	5.42E-02	NA	NA	NA	LOW	NA	NA	212	207	0	179	126	49	27.37
Non-user	IOB_24	chr6	27792232	T	C	HIST1H4J	3UTR	ENSG00000197238:[ENST00000355057(c.*18T>C)]	NA	NA	3.69E-02	2.76E-02	6.19E-02	3.69E-02	5.42E-02	NA	NA	NA	MODIFIER	NA	NA	279	277	0	251	187	61	24.3
Non-user	IOB_24	chr6	28240525	C	T	RP5-874C20.3	EXONIC-NC	ENSG00000197062:[ENST00000423234(n.336C>T)]	NA	NA	4.39E-03	6.27E-03	3.77E-02	4.39E-03	1.94E-02	NA	NA	NA	MODIFIER	NA	NA	144	144	0	92	46	46	50
Non-user	IOB_24	chr6	28240525	C	T	ZSCAN26	MISSENSE	7741:[NM_152736.5(c.94C>T,Cgg>Tgg):NP_689949.3(p.Arg32Trp)]	NA	NA	4.39E-03	6.27E-03	3.77E-02	4.39E-03	1.94E-02	NA	NA	NA	MODIFIER	NA	NA	144	144	0	92	46	46	50
Non-user	IOB_24	chr6	28297279	C	T	ZSCAN31	MISSENSE	ENSG00000235109:[ENST00000344279(c.182G>A,cGa>cAa):ENSP00000345339(p.Arg61Gln)]	NA	NA	4.19E-03	5.72E-03	2.74E-02	4.19E-03	1.64E-02	NA	NA	NA	MODIFIER	NA	NA	98	96	0	58	33	23	39.66
Non-user	IOB_24	chr6	29142347	A	T	OR2J2	MISSENSE	ENSG00000204700:[ENST00000377167(c.935A>T,aAg>aTg):ENSP00000366372(p.Lys312Met)]	NA	NA	2.84E-02	3.11E-02	7.20E-02	2.84E-02	4.91E-02	D	BN	NA	MODERATE	NA	NA	88	88	0	88	49	37	42.05
Non-user	IOB_24	chr6	29691312	C	T	HCG4P11	EXONIC-NC	ENSG00000225864:[ENST00000427340(n.437G>A)]	NA	NA	NA	1.73E-05	0.00E+00	NA	NA	NA	NA	NA	MODIFIER	NA	NA	87	87	0	49	29	20	40.82
Non-user	IOB_24	chr6	29691312	C	T	HLA-F	NCT	ENSG00000204642:[ENST00000489502(n.28+81C>T)]	NA	NA	NA	1.73E-05	0.00E+00	NA	NA	NA	NA	NA	MODIFIER	NA	NA	87	87	0	49	29	20	40.82
Non-user	IOB_24	chr6	29913007	C	T	HLA-A	NCT-SS-PRX	ENSG00000206503:[ENST00000461903(n.1305-4C>T)]	NA	NA	4.19E-03	3.66E-03	1.42E-02	4.19E-03	1.64E-02	NA	NA	NA	LOW	NA	NA	256	256	0	152	72	79	51.97
Non-user	IOB_24	chr6	30080208	G	A	TRIM31	SILENT	ENSG00000204616:[ENST00000376734(c.375C>T,tcC>tcT):ENSP00000365924(p.Ser125(=))]	NA	NA	3.34E-02	2.28E-02	1.33E-01	3.33E-02	1.29E-01	NA	NA	NA	LOW	NA	NA	96	96	0	63	43	20	31.75
Non-user	IOB_24	chr6	30080208	G	A	TRIM31-AS1	NCT	ENSG00000231226:[ENST00000440874(n.395+592G>A)]	NA	NA	3.34E-02	2.28E-02	1.33E-01	3.33E-02	1.29E-01	NA	NA	NA	LOW	NA	NA	96	96	0	63	43	20	31.75
Non-user	IOB_24	chr6	30080405	C	T	TRIM31	MISSENSE	ENSG00000204616:[ENST00000376734(c.178G>A,Gta>Ata):ENSP00000365924(p.Val60Ile)]	NA	chr6*30080405*30080405*COSM1131938*C*T*1*prostate*1	3.36E-02	2.29E-02	1.31E-01	3.35E-02	1.29E-01	T	BN	NA	MODERATE	N	0	171	171	0	157	75	82	52.23
Non-user	IOB_24	chr6	30080405	C	T	TRIM31-AS1	NCT	ENSG00000231226:[ENST00000440874(n.395+789C>T)]	NA	chr6*30080405*30080405*COSM1131938*C*T*1*prostate*1	3.36E-02	2.29E-02	1.31E-01	3.35E-02	1.29E-01	T	BN	NA	MODERATE	N	0	171	171	0	157	75	82	52.23
Non-user	IOB_24	chr6	30121385	T	C	TRIM10	3UTR	ENSG00000204613:[ENST00000449742(c.*361A>G)]	NA	NA	3.38E-02	NA	NA	3.37E-02	1.31E-01	NA	NA	NA	MODIFIER	NA	NA	74	73	0	79	46	33	41.77
Non-user	IOB_24	chr6	30157154	C	T	TRIM26	INTRONIC	ENSG00000234127:[ENST00000437089(c.904+41G>A)]	NA	NA	3.12E-02	2.18E-02	1.24E-01	3.12E-02	1.24E-01	NA	NA	NA	MODIFIER	NA	NA	97	97	0	95	59	36	37.89
Non-user	IOB_24	chr6	30166475	C	T	TRIM26	MISSENSE	ENSG00000234127:[ENST00000416596(c.406G>A,Gtc>Atc):ENSP00000413673(p.Val136Ile)]	NA	NA	3.12E-02	2.21E-02	1.26E-01	3.12E-02	1.24E-01	T	BN	NA	MODERATE	N	0	163	160	0	131	62	68	51.91
Non-user	IOB_24	chr6	30166632	C	T	TRIM26	SILENT	ENSG00000234127:[ENST00000416596(c.249G>A,gtG>gtA):ENSP00000413673(p.Val83(=))]	NA	NA	3.12E-02	2.18E-02	1.24E-01	3.12E-02	1.24E-01	NA	NA	NA	MODIFIER	NA	NA	248	248	0	173	94	78	45.09
Non-user	IOB_24	chr6	30521203	T	C	GNL1	SILENT	ENSG00000204590:[ENST00000433809(c.726A>G,caA>caG):ENSP00000404728(p.Gln242(=))]	NA	NA	NA	NA	NA	NA	NA	NA	NA	NA	LOW	NA	0.116	38	38	0	33	18	15	45.45
Non-user	IOB_24	chr6	30546417	T	G	ABCF1	INTRONIC	ENSG00000204574:[ENST00000441867(c.492+63T>G)]	NA	NA	1.30E-02	NA	NA	1.30E-02	2.35E-02	NA	NA	NA	MODIFIER	NA	NA	88	87	1	64	32	32	50
Non-user	IOB_24	chr6	30574428	A	G	PPP1R10	INTRONIC-SS-ACR-PRX	ENSG00000204569:[ENST00000376511(c.461-10T>C)]	NA	NA	1.46E-02	9.81E-03	2.41E-02	1.46E-02	2.35E-02	NA	NA	NA	MODIFIER	NA	NA	54	53	0	39	17	21	53.85
Non-user	IOB_24	chr6	30594827	C	T	ATAT1	NCT	79969:[XR_241920.1(n.145+58C>T)]	NA	NA	1.30E-02	NA	NA	1.30E-02	2.35E-02	NA	NA	NA	MODIFIER	NA	NA	338	336	0	221	130	90	40.72
Non-user	IOB_24	chr6	30615213	C	T	C6orf136	SILENT	ENSG00000204564:[ENST00000446773(c.16C>T,Ctg>Ttg):ENSP00000415810(p.Leu6(=))]	NA	NA	1.72E-02	2.16E-02	4.44E-02	1.72E-02	2.35E-02	NA	NA	NA	LOW	NA	NA	125	125	0	70	41	28	40
Non-user	IOB_24	chr6	30615603	A	G	C6orf136	MISSENSE	ENSG00000204564:[ENST00000293604(c.595A>G,Agg>Ggg):ENSP00000293604(p.Arg199Gly)]	NA	NA	1.72E-02	2.93E-02	3.88E-02	1.72E-02	2.35E-02	NA	NA	NA	MODIFIER	NA	NA	72	71	0	59	32	27	45.76
Non-user	IOB_24	chr6	30620528	G	A	C6orf136	NCT	ENSG00000204564:[ENST00000460172(n.107+34G>A)]	NA	NA	1.72E-02	1.12E-02	2.49E-02	1.72E-02	2.35E-02	NA	NA	NA	MODIFIER	NA	NA	147	147	0	118	52	65	55.08
Non-user	IOB_24	chr6	30647209	G	A	PPP1R18	NCT	ENSG00000146112:[ENST00000467662(n.46-43C>T)]	NA	NA	1.64E-02	1.15E-02	2.72E-02	1.64E-02	2.25E-02	NA	NA	NA	MODIFIER	NA	NA	25	25	0	15	7	7	46.67
Non-user	IOB_24	chr6	30653141	C	G	PPP1R18	MISSENSE	ENSG00000146112:[ENST00000274853(c.655G>C,Gtg>Ctg):ENSP00000274853(p.Val219Leu)]	NA	NA	6.19E-03	6.13E-03	2.12E-02	6.19E-03	2.25E-02	NA	NA	NA	MODIFIER	NA	NA	360	359	0	271	137	134	49.45
Non-user	IOB_24	chr6	30656398	T	C	NRM	EXONIC-NC	11270:[NR_073072.1(n.650A>G)]	NA	NA	2.34E-02	1.74E-02	2.80E-02	2.34E-02	2.25E-02	NA	NA	NA	MODIFIER	NA	NA	34	32	0	17	10	5	29.41
Non-user	IOB_24	chr6	30658385	C	T	NRM	MISSENSE	ENSG00000137404:[ENST00000444096(c.116G>A,Gac>Aac):ENSP00000397892(p.Asp40Asn)]	NA	NA	4.63E-02	5.94E-02	8.82E-02	4.63E-02	9.10E-02	T_lc	BN	NA	MODERATE	NA	NA	64	64	0	59	24	35	59.32
Non-user	IOB_24	chr6	30671452	T	C	MDC1	SILENT	ENSG00000137337:[ENST00000376405(c.4716A>G,acA>acG):ENSP00000365587(p.Thr1572(=))]	NA	NA	1.66E-02	1.01E-02	2.56E-02	1.66E-02	2.25E-02	NA	NA	NA	LOW	NA	NA	200	198	0	135	70	64	47.41
Non-user	IOB_24	chr6	30671452	T	C	MDC1-AS1	NCT	ENSG00000224328:[ENST00000442150(n.127+482T>C)]	NA	NA	1.66E-02	1.01E-02	2.56E-02	1.66E-02	2.25E-02	NA	NA	NA	LOW	NA	NA	200	198	0	135	70	64	47.41
Non-user	IOB_24	chr6	30671760	C	T	MDC1	MISSENSE	ENSG00000137337:[ENST00000376406(c.5200G>A,Gca>Aca):ENSP00000365588(p.Ala1734Thr)]	NA	NA	1.66E-02	1.10E-02	2.47E-02	1.66E-02	2.25E-02	T	BN	NA	MODERATE	N	0	206	205	0	165	82	83	50.3
Non-user	IOB_24	chr6	30671760	C	T	MDC1-AS1	NCT	ENSG00000224328:[ENST00000442150(n.127+790C>T)]	NA	NA	1.66E-02	1.10E-02	2.47E-02	1.66E-02	2.25E-02	T	BN	NA	MODERATE	N	0	206	205	0	165	82	83	50.3
Non-user	IOB_24	chr6	30671969	G	C	MDC1	MISSENSE	ENSG00000137337:[ENST00000376405(c.4199C>G,aCc>aGc):ENSP00000365587(p.Thr1400Ser)]	NA	NA	1.68E-02	1.12E-02	2.58E-02	1.68E-02	2.25E-02	T	BN	NA	MODERATE	N	0	187	187	0	130	80	50	38.46
Non-user	IOB_24	chr6	30671969	G	C	MDC1-AS1	NCT	ENSG00000224328:[ENST00000442150(n.127+999G>C)]	NA	NA	1.68E-02	1.12E-02	2.58E-02	1.68E-02	2.25E-02	T	BN	NA	MODERATE	N	0	187	187	0	130	80	50	38.46
Non-user	IOB_24	chr6	30672718	G	A	MDC1	SILENT	ENSG00000137337:[ENST00000376405(c.3450C>T,ctC>ctT):ENSP00000365587(p.Leu1150(=))]	NA	NA	1.66E-02	1.11E-02	2.67E-02	1.66E-02	2.25E-02	NA	NA	NA	LOW	NA	NA	331	322	0	220	116	101	45.91
Non-user	IOB_24	chr6	30672718	G	A	MDC1-AS1	NCT	ENSG00000224328:[ENST00000442150(n.127+1748G>A)]	NA	NA	1.66E-02	1.11E-02	2.67E-02	1.66E-02	2.25E-02	NA	NA	NA	LOW	NA	NA	331	322	0	220	116	101	45.91
Non-user	IOB_24	chr6	30673113	G	T	MDC1	MISSENSE	ENSG00000137337:[ENST00000376406(c.3847C>A,Cca>Aca):ENSP00000365588(p.Pro1283Thr)]	NA	chr6*30673113*30673113*COSM1235522*G*T*2*haematopoietic_and_lymphoid_tissue*2|chr6*30673113*30673113*COSM1235523*G*T*2*haematopoietic_and_lymphoid_tissue*2	4.57E-02	1.26E-02	3.01E-02	4.57E-02	8.79E-02	NA	NA	NA	MODERATE	NA	0	289	285	2	183	114	62	33.88
Non-user	IOB_24	chr6	30673113	G	T	MDC1-AS1	NCT	ENSG00000224328:[ENST00000442150(n.127+2143G>T)]	NA	chr6*30673113*30673113*COSM1235522*G*T*2*haematopoietic_and_lymphoid_tissue*2|chr6*30673113*30673113*COSM1235523*G*T*2*haematopoietic_and_lymphoid_tissue*2	4.57E-02	1.26E-02	3.01E-02	4.57E-02	8.79E-02	NA	NA	NA	MODERATE	NA	0	289	285	2	183	114	62	33.88
Non-user	IOB_24	chr6	30673177	A	G	MDC1	SILENT	ENSG00000137337:[ENST00000376406(c.3783T>C,acT>acC):ENSP00000365588(p.Thr1261(=))]	NA	NA	NA	1.03E-02	2.12E-02	NA	NA	NA	NA	NA	LOW	NA	NA	303	276	8	200	114	65	32.5
Non-user	IOB_24	chr6	30673177	A	G	MDC1-AS1	NCT	ENSG00000224328:[ENST00000442150(n.127+2207A>G)]	NA	NA	NA	1.03E-02	2.12E-02	NA	NA	NA	NA	NA	LOW	NA	NA	303	276	8	200	114	65	32.5
Non-user	IOB_24	chr6	30673186	C	T	MDC1	SILENT	ENSG00000137337:[ENST00000376406(c.3774G>A,caG>caA):ENSP00000365588(p.Gln1258(=))]	NA	NA	4.37E-02	1.01E-02	2.09E-02	4.37E-02	8.38E-02	NA	NA	NA	MODIFIER	NA	NA	302	284	2	203	116	77	37.93
Non-user	IOB_24	chr6	30673186	C	T	MDC1-AS1	NCT	ENSG00000224328:[ENST00000442150(n.127+2216C>T)]	NA	NA	4.37E-02	1.01E-02	2.09E-02	4.37E-02	8.38E-02	NA	NA	NA	MODIFIER	NA	NA	302	284	2	203	116	77	37.93
Non-user	IOB_24	chr6	30676046	C	G	MDC1	SILENT	ENSG00000137337:[ENST00000376406(c.2310G>C,acG>acC):ENSP00000365588(p.Thr770(=))]	NA	NA	4.65E-02	3.23E-02	7.88E-02	4.65E-02	9.10E-02	NA	NA	NA	MODIFIER	NA	NA	167	166	0	112	65	47	41.96
Non-user	IOB_24	chr6	30676046	C	G	MDC1-AS1	NCT	ENSG00000224328:[ENST00000442150(n.128-4043C>G)]	NA	NA	4.65E-02	3.23E-02	7.88E-02	4.65E-02	9.10E-02	NA	NA	NA	MODIFIER	NA	NA	167	166	0	112	65	47	41.96
Non-user	IOB_24	chr6	30691490	G	A	TUBB	SILENT	ENSG00000196230:[ENST00000330914(c.435G>A,ctG>ctA):ENSP00000365578(p.Leu145(=))]	NA	NA	4.53E-02	2.50E-02	2.88E-02	4.53E-02	2.35E-02	NA	NA	NA	LOW	NA	NA	327	325	0	226	130	96	42.48
Non-user	IOB_24	chr6	30691490	G	A	XXbac-BPG252P9.9	EXONIC-NC	ENSG00000272540:[ENST00000607476(n.165C>T)]	NA	NA	4.53E-02	2.50E-02	2.88E-02	4.53E-02	2.35E-02	NA	NA	NA	LOW	NA	NA	327	325	0	226	130	96	42.48
Non-user	IOB_24	chr6	30697931	T	C	FLOT1	SILENT	ENSG00000137312:[ENST00000456573(c.978A>G,tcA>tcG):ENSP00000394375(p.Ser326(=))]	NA	NA	4.69E-02	2.57E-02	2.91E-02	4.69E-02	2.35E-02	NA	NA	NA	LOW	NA	NA	121	119	0	100	44	55	55

Patel *et al.* , 2021. Whole-exome sequencing analysis of oral squamous cell carcinoma delineated by tobacco usage habits
Supplementary Table 4. List of somatic single nucleotide variants identified using whole exome sequencing of OSCC patients

Cohort	Sample	Chromosome	Genomic position	Reference allele	Alternate allele	Genesymbol	Variant type	Variant description	OncoMD	COSMIC	dbSNP	ExAC	ExAC_SAS	1000G	1000G_SAS	SIFT	Polyphen2	CONDEL	VEP	LRT	PhastCon7Way	Total reads in Normal	Reference read support in Normal	Alternative read support in Normal	Total reads in Tumor	Reference read support in Tumor	Alternate read support in Tumor	Alternate allele frequency in Tumor
Non-user	IOB_24	chr6	30865279	C	T	DDR1	SILENT	ENSG00000204580:[ENST00000324771(c.2121C>T,ggC>ggT):ENSP00000318217(p.Gly707(=))]	NA	NA	2.48E-02	2.50E-02	3.15E-02	2.48E-02	3.27E-02	NA	NA	NA	LOW	NA	NA	148	147	1	128	53	74	57.81
Non-user	IOB_24	chr6	30887465	C	T	VARS2	NCT	ENSG00000137411:[ENST00000477288(n.3688-70C>T)]	NA	NA	2.48E-02	NA	NA	2.48E-02	3.27E-02	NA	NA	NA	MODIFIER	NA	NA	284	281	2	201	103	96	47.76
Non-user	IOB_24	chr6	30916705	A	G	DPCR1	MISSENSE	ENSG00000168631:[ENST00000462446(c.464A>G,aAa>aGa):ENSP00000417182(p.Lys155Arg)]	NA	NA	3.47E-02	3.99E-02	8.86E-02	3.47E-02	1.29E-01	T	PoD	NA	MODERATE	NA	NA	102	101	1	84	36	48	57.14
Non-user	IOB_24	chr6	30916705	A	G	HCG21	NCT	102723346:[NR_138040.1(n.257-2350T>C)]	NA	NA	3.47E-02	3.99E-02	8.86E-02	3.47E-02	1.29E-01	T	PoD	NA	MODERATE	NA	NA	102	101	1	84	36	48	57.14
Non-user	IOB_24	chr6	30920086	C	T	DPCR1	MISSENSE	ENSG00000168631:[ENST00000462446(c.3845C>T,aCg>aTg):ENSP00000417182(p.Thr1282Met)]	NA	NA	4.85E-02	4.39E-02	3.36E-02	4.85E-02	3.37E-02	T	BN	NA	MODERATE	NA	NA	185	182	0	164	71	92	56.1
Non-user	IOB_24	chr6	30920086	C	T	HCG21	NCT	ENSG00000233529:[ENST00000419481(n.224+758G>A)]	NA	NA	4.85E-02	4.39E-02	3.36E-02	4.85E-02	3.37E-02	T	BN	NA	MODERATE	NA	NA	185	182	0	164	71	92	56.1
Non-user	IOB_24	chr6	30996325	C	T	MUC22	SILENT	ENSG00000261272:[ENST00000561890(c.3117C>T,acC>acT):ENSP00000455906(p.Thr1039(=))]	NA	NA	1.46E-02	4.20E-02	5.10E-02	1.46E-02	4.29E-02	NA	NA	NA	LOW	NA	NA	819	806	1	563	273	283	50.27
Non-user	IOB_24	chr6	31116245	C	T	CCHCR1	MISSENSE	ENSG00000204536:[ENST00000376266(c.1250G>A,cGg>cAg):ENSP00000365442(p.Arg417Gln)]	NA	NA	3.61E-02	5.20E-02	8.28E-02	3.61E-02	7.77E-02	T	BN	NA	MODERATE	N	0.009	125	125	0	119	54	65	54.62
Non-user	IOB_24	chr6	31165507	G	T	XXbac-BPG299F13.17	EXONIC-NC	ENSG00000272501:[ENST00000606367(n.308C>A)]	NA	NA	3.51E-02	1.10E-01	9.37E-02	3.51E-02	7.16E-02	NA	NA	NA	MODIFIER	NA	NA	259	258	0	249	110	136	54.62
Non-user	IOB_24	chr6	31324128	C	T	HLA-B	SILENT	ENSG00000234745:[ENST00000434333(c.468G>A,aaG>aaA):ENSP00000405931(p.Lys156(=))]	NA	NA	NA	8.21E-02	1.66E-01	NA	NA	NA	NA	NA	MODIFIER	NA	NA	101	100	0	26	7	19	73.08
Non-user	IOB_24	chr6	31379118	G	A	HCP5	NCT	ENSG00000206337:[ENST00000414046(n.62+10578G>A)]	NA	NA	2.00E-03	5.35E-03	9.12E-03	2.00E-03	4.10E-03	T	BN	NA	MODERATE	NA	0	204	203	0	139	72	65	46.76
Non-user	IOB_24	chr6	31379118	G	A	MICA	MISSENSE	ENSG00000204520:[ENST00000449934(c.595G>A,Gta>Ata):ENSP00000413079(p.Val199Ile)]	NA	NA	2.00E-03	5.35E-03	9.12E-03	2.00E-03	4.10E-03	T	BN	NA	MODERATE	NA	0	204	203	0	139	72	65	46.76
Non-user	IOB_24	chr6	31465917	G	T	MICB	INTRONIC	ENSG00000204516:[ENST00000538442(c.-27+3145G>T)]	NA	NA	4.89E-02	NA	NA	4.89E-02	1.00E-01	NA	NA	NA	MODIFIER	NA	NA	96	96	0	82	47	33	40.24
Non-user	IOB_24	chr6	31474820	C	T	MICB	MISSENSE	ENSG00000204516:[ENST00000399150(c.506C>T,aCc>aTc):ENSP00000382103(p.Thr169Ile)]	NA	NA	4.87E-02	3.58E-02	6.10E-02	4.87E-02	1.00E-01	D	BN	NA	MODERATE	NA	NA	133	133	0	156	82	74	47.44
Non-user	IOB_24	chr6	31509980	G	T	ATP6V1G2-DDX39B	EXONIC-NC	ENSG00000254870:[ENST00000475917(n.519C>A)]	NA	NA	3.89E-02	NA	NA	3.89E-02	9.30E-02	NA	NA	NA	MODIFIER	NA	NA	288	284	1	206	103	103	50
Non-user	IOB_24	chr6	31509980	G	T	DDX39B	5UTR	ENSG00000198563:[ENST00000396172(c.-386C>A)]	NA	NA	3.89E-02	NA	NA	3.89E-02	9.30E-02	NA	NA	NA	MODIFIER	NA	NA	288	284	1	206	103	103	50
Non-user	IOB_24	chr6	31601689	C	T	PRRC2A	MISSENSE	ENSG00000204469:[ENST00000376007(c.4642C>T,Cgg>Tgg):ENSP00000365175(p.Arg1548Trp)]	NA	NA	NA	NA	NA	NA	NA	D_lc	BN	NA	MODERATE	N	0.716	54	53	0	69	34	33	47.83
Non-user	IOB_24	chr6	31679870	C	G	LY6G6E	3UTR	ENSG00000255552:[ENST00000409239(c.*9G>C)]	NA	NA	2.00E-04	2.00E-03	4.30E-03	2.00E-04	1.00E-03	NA	NA	NA	MODIFIER	NA	NA	223	223	0	107	57	50	46.73
Non-user	IOB_24	chr6	31679870	C	G	LY6G6F	INTRONIC	ENSG00000204424:[ENST00000556581(c.802+1912C>G)]	NA	NA	2.00E-04	2.00E-03	4.30E-03	2.00E-04	1.00E-03	NA	NA	NA	MODIFIER	NA	NA	223	223	0	107	57	50	46.73
Non-user	IOB_24	chr6	31679870	C	G	MEGT1	INTRONIC	ENSG00000250641:[ENST00000503322(c.802+1912C>G)]	NA	NA	2.00E-04	2.00E-03	4.30E-03	2.00E-04	1.00E-03	NA	NA	NA	MODIFIER	NA	NA	223	223	0	107	57	50	46.73
Non-user	IOB_24	chr6	31679870	C	G	XXbac-BPG32J3.20	3UTR	ENSG00000204422:[ENST00000461287(c.*9G>C)]	NA	NA	2.00E-04	2.00E-03	4.30E-03	2.00E-04	1.00E-03	NA	NA	NA	MODIFIER	NA	NA	223	223	0	107	57	50	46.73
Non-user	IOB_24	chr6	31692777	G	A	C6orf25	MISSENSE	ENSG00000204420:[ENST00000375814(c.520G>A,Gcc>Acc):ENSP00000364972(p.Ala174Thr)]	NA	NA	NA	3.45E-05	2.47E-04	NA	NA	NA	NA	NA	MODIFIER	NA	NA	139	138	0	116	56	59	50.86
Non-user	IOB_24	chr6	31861207	G	C	EHMT2	EXONIC-NC	ENSG00000204371:[ENST00000480912(n.475C>G)]	NA	NA	3.30E-02	4.45E-02	4.93E-02	3.29E-02	9.71E-02	NA	NA	NA	MODIFIER	NA	NA	27	27	0	31	18	13	41.94
Non-user	IOB_24	chr6	31864711	G	T	EHMT2	MISSENSE	ENSG00000204371:[ENST00000375528(c.269C>A,gCc>gAc):ENSP00000364678(p.Ala90Asp)]	NA	NA	1.40E-03	1.20E-03	6.90E-03	1.40E-03	5.10E-03	NA	NA	NA	MODIFIER	NA	NA	98	97	0	80	44	35	43.75
Non-user	IOB_24	chr6	31929808	C	T	SKIV2L	SILENT	ENSG00000204351:[ENST00000544581(c.462C>T,gcC>gcT):ENSP00000442645(p.Ala154(=))]	NA	NA	3.26E-02	2.87E-02	5.57E-02	3.25E-02	9.82E-02	NA	NA	NA	MODIFIER	NA	NA	156	155	0	163	67	96	58.9
Non-user	IOB_24	chr6	31937886	C	T	DXO	MISSENSE-SS-PRX	ENSG00000204348:[ENST00000478221(n.924G>A)]	NA	NA	NA	NA	NA	NA	NA	NA	NA	NA	MODIFIER	NA	NA	221	221	0	173	103	69	39.88
Non-user	IOB_24	chr6	31977230	C	T	TNXA	NCT	ENSG00000248290:[ENST00000507684(n.1611-15G>A)]	NA	NA	NA	2.71E-01	3.03E-01	NA	NA	NA	NA	NA	MODIFIER	NA	NA	14	14	0	15	10	5	33.33
Non-user	IOB_24	chr6	31997600	C	G	C4B	INTRONIC	ENSG00000224389:[ENST00000425700(c.3909+25C>G)]	NA	NA	chr6*31997599*31997600*GC*G*rs762874090*NA*NA*NA|chr6*31997600*31997600*C*A,G,T*rs2023615*NA*NA*NA	4.75E-01	4.72E-01	NA	NA	NA	NA	NA	MODIFIER	NA	NA	92	92	0	29	14	15	51.72
Non-user	IOB_24	chr6	32006337	C	A	CYP21A2	SILENT	ENSG00000231852:[ENST00000418967(c.138C>A,ccC>ccA):ENSP00000408860(p.Pro46(=))]	NA	NA	NA	6.64E-01	6.36E-01	NA	NA	NA	NA	NA	LOW	NA	NA	85	84	0	50	24	24	48
Non-user	IOB_24	chr6	32008451	C	T	CYP21A2	SILENT	ENSG00000231852:[ENST00000418967(c.1125C>T,tcC>tcT):ENSP00000408860(p.Ser375(=))]	NA	NA	chr6*32008451*32008451*C*A,G,T*rs6469*NA*NA*NA	2.17E-01	1.94E-01	NA	NA	NA	NA	NA	MODIFIER	NA	NA	187	187	0	79	28	51	64.56
Non-user	IOB_24	chr6	32039995	T	C	TNXB	MISSENSE	ENSG00000168477:[ENST00000375244(c.4762A>G,Ata>Gta):ENSP00000364393(p.Ile1588Val)]	NA	NA	7.99E-04	7.95E-04	5.74E-03	7.99E-04	4.10E-03	NA	BN	NA	MODERATE	N	0.001	321	321	0	233	109	123	52.79
Non-user	IOB_24	chr6	32085704	T	C	ATF6B	SILENT	ENSG00000213676:[ENST00000375201(c.1347A>G,gaA>gaG):ENSP00000364347(p.Glu449(=))]	NA	NA	NA	NA	NA	NA	NA	NA	NA	NA	LOW	NA	0	80	80	0	69	34	35	50.72
Non-user	IOB_24	chr6	32185796	C	T	NOTCH4	MISSENSE	ENSG00000204301:[ENST00000375023(c.1600G>A,Ggc>Agc):ENSP00000364163(p.Gly534Ser)]	NA	NA	3.12E-02	2.73E-02	5.91E-02	3.12E-02	9.82E-02	T	BN	NA	MODERATE	N	0	80	80	0	61	35	26	42.62
Non-user	IOB_24	chr6	32188823	G	A	NOTCH4	MISSENSE	ENSG00000204301:[ENST00000375023(c.731C>T,tCg>tTg):ENSP00000364163(p.Ser244Leu)]	NA	NA	2.22E-02	3.58E-02	3.03E-02	2.22E-02	2.66E-02	NA	NA	NA	MODIFIER	NA	NA	235	231	1	210	106	104	49.52
Non-user	IOB_24	chr6	32223516	T	C	LOC101929163	NCT	101929163:[NR_136246.1(n.242+325T>C)]	NA	NA	1.08E-02	2.14E-02	5.30E-02	1.08E-02	1.33E-02	NA	NA	NA	MODIFIER	NA	NA	49	48	0	29	17	12	41.38
Non-user	IOB_24	chr6	32223516	T	C	XXbac-BPG154L12.4	EXONIC-NC	ENSG00000225914:[ENST00000425033(n.29T>C)]	NA	NA	1.08E-02	2.14E-02	5.30E-02	1.08E-02	1.33E-02	NA	NA	NA	MODIFIER	NA	NA	49	48	0	29	17	12	41.38
Non-user	IOB_24	chr6	32223615	T	G	LOC101929163	NCT	101929163:[NR_136246.1(n.242+424T>G)]	NA	NA	1.06E-02	1.04E-02	1.43E-02	1.06E-02	1.33E-02	NA	NA	NA	MODIFIER	NA	NA	82	82	0	67	43	20	29.85
Non-user	IOB_24	chr6	32223615	T	G	XXbac-BPG154L12.4	EXONIC-NC	ENSG00000225914:[ENST00000425033(n.128T>G)]	NA	NA	1.06E-02	1.04E-02	1.43E-02	1.06E-02	1.33E-02	NA	NA	NA	MODIFIER	NA	NA	82	82	0	67	43	20	29.85
Non-user	IOB_24	chr6	32256486	G	A	C6orf10	INTRONIC	ENSG00000204296:[ENST00000442822(c.1360-40C>T)]	NA	NA	1.06E-02	1.17E-02	1.45E-02	1.06E-02	1.33E-02	NA	NA	NA	MODIFIER	NA	NA	186	183	0	133	59	72	54.14
Non-user	IOB_24	chr6	32256486	G	A	LOC101929163	NCT	101929163:[NR_136244.1(n.440+25900G>A)]	NA	NA	1.06E-02	1.17E-02	1.45E-02	1.06E-02	1.33E-02	NA	NA	NA	MODIFIER	NA	NA	186	183	0	133	59	72	54.14
Non-user	IOB_24	chr6	32281259	A	G	C6orf10	INTRONIC	ENSG00000204296:[ENST00000533191(c.574+2290T>C)]	NA	NA	1.06E-02	NA	NA	1.06E-02	1.33E-02	NA	NA	NA	MODIFIER	NA	NA	85	85	0	70	35	34	48.57
Non-user	IOB_24	chr6	32281259	A	G	LOC101929163	NCT	101929163:[NR_136246.1(n.243-36030A>G)]	NA	NA	1.06E-02	NA	NA	1.06E-02	1.33E-02	NA	NA	NA	MODIFIER	NA	NA	85	85	0	70	35	34	48.57
Non-user	IOB_24	chr6	32281446	T	G	C6orf10	INTRONIC	ENSG00000204296:[ENST00000447241(c.580+8817A>C)]	NA	NA	1.06E-02	NA	NA	1.06E-02	1.33E-02	NA	NA	NA	MODIFIER	NA	NA	218	218	0	164	70	92	56.1
Non-user	IOB_24	chr6	32281446	T	G	LOC101929163	NCT	101929163:[NR_136246.1(n.243-35843T>G)]	NA	NA	1.06E-02	NA	NA	1.06E-02	1.33E-02	NA	NA	NA	MODIFIER	NA	NA	218	218	0	164	70	92	56.1
Non-user	IOB_24	chr6	32299744	G	C	C6orf10	INTRONIC	ENSG00000204296:[ENST00000442822(c.424+67C>G)]	NA	NA	1.06E-02	NA	NA	1.06E-02	1.33E-02	NA	NA	NA	MODIFIER	NA	NA	135	134	0	104	44	60	57.69
Non-user	IOB_24	chr6	32299744	G	C	LOC101929163	NCT	101929163:[NR_136244.1(n.441-33813G>C)]	NA	NA	1.06E-02	NA	NA	1.06E-02	1.33E-02	NA	NA	NA	MODIFIER	NA	NA	135	134	0	104	44	60	57.69
Non-user	IOB_24	chr6	32301835	T	A	C6orf10	INTRONIC	ENSG00000204296:[ENST00000375007(c.466+1379A>T)]	NA	NA	3.79E-03	NA	NA	3.79E-03	5.10E-03	NA	NA	NA	MODIFIER	NA	NA	54	54	0	38	19	19	50
Non-user	IOB_24	chr6	32301835	T	A	LOC101929163	NCT	101929163:[NR_136244.1(n.441-31722T>A)]	NA	NA	3.79E-03	NA	NA	3.79E-03	5.10E-03	NA	NA	NA	MODIFIER	NA	NA	54	54	0	38	19	19	50
Non-user	IOB_24	chr6	32303707	C	T	C6orf10	MISSENSE-SS-PRX	ENSG00000204296:[ENST00000447241(c.433G>A,Gga>Aga):ENSP00000415517(p.Gly145Arg)]	NA	NA	1.08E-02	9.27E-03	1.70E-02	1.08E-02	1.33E-02	NA	NA	NA	MODIFIER	NA	NA	80	80	0	49	30	19	38.78
Non-user	IOB_24	chr6	32303707	C	T	LOC101929163	NCT	101929163:[NR_136245.1(n.243-29850C>T)]	NA	NA	1.08E-02	9.27E-03	1.70E-02	1.08E-02	1.33E-02	NA	NA	NA	MODIFIER	NA	NA	80	80	0	49	30	19	38.78
Non-user	IOB_24	chr6	32303749	A	G	C6orf10	INTRONIC	ENSG00000204296:[ENST00000375015(c.383-40T>C)]	NA	NA	1.06E-02	9.11E-03	1.69E-02	1.06E-02	1.33E-02	NA	NA	NA	MODIFIER	NA	NA	70	68	2	30	17	13	43.33
Non-user	IOB_24	chr6	32303749	A	G	LOC101929163	NCT	101929163:[NR_136244.1(n.441-29808A>G)]	NA	NA	1.06E-02	9.11E-03	1.69E-02	1.06E-02	1.33E-02	NA	NA	NA	MODIFIER	NA	NA	70	68	2	30	17	13	43.33
Non-user	IOB_24	chr6	32304433	A	G	C6orf10	INTRONIC	ENSG00000204296:[ENST00000375007(c.404-21T>C)]	NA	NA	1.06E-02	9.19E-03	1.71E-02	1.06E-02	1.33E-02	NA	NA	NA	MODIFIER	NA	NA	87	87	0	74	47	27	36.49
Non-user	IOB_24	chr6	32304433	A	G	LOC101929163	NCT	101929163:[NR_136246.1(n.243-12856A>G)]	NA	NA	1.06E-02	9.19E-03	1.71E-02	1.06E-02	1.33E-02	NA	NA	NA	MODIFIER	NA	NA	87	87	0	74	47	27	36.49
Non-user	IOB_24	chr6	32317568	G	A	C6orf10	MISSENSE	ENSG00000204296:[ENST00000375007(c.292C>T,Ctc>Ttc):ENSP00000364146(p.Leu98Phe)]	NA	NA	1.06E-02	9.03E-03	1.69E-02	1.06E-02	1.33E-02	T_lc	BN	NA	MODERATE	NA	NA	147	147	0	117	55	61	52.14
Non-user	IOB_24	chr6	32317568	G	A	LOC101929163	NCT	101929163:[NR_136244.1(n.441-15989G>A)]	NA	NA	1.06E-02	9.03E-03	1.69E-02	1.06E-02	1.33E-02	T_lc	BN	NA	MODERATE	NA	NA	147	147	0	117	55	61	52.14
Non-user	IOB_24	chr6	32333784	C	T	C6orf10	INTRONIC	ENSG00000204296:[ENST00000532023(c.229+266G>A)]	NA	NA	1.08E-02	1.09E-02	1.46E-02	1.08E-02	1.33E-02	NA	NA	NA	MODIFIER	NA	NA	56	56	0	55	36	19	34.55
Non-user	IOB_24	chr6	32333784	C	T	LOC101929163	NCT	101929163:[NR_136244.1(n.500+168C>T)]	NA	NA	1.08E-02	1.09E-02	1.46E-02	1.08E-02	1.33E-02	NA	NA	NA	MODIFIER	NA	NA	56	56	0	55	36	19	34.55
Non-user	IOB_24	chr6	32606762	T	G	HLA-DQA1	MISSENSE	ENSG00000196735:[ENST00000482745(c.156T>G,gaT>gaG):ENSP00000436546(p.Asp52Glu)]	NA	NA	1.80E-03	1.07E-02	1.43E-02	1.80E-03	3.10E-03	NA	NA	NA	MODIFIER	NA	NA	58	57	0	44	15	29	65.91
Non-user	IOB_24	chr6	32731252	C	T	HLA-DQB2	5UTR	ENSG00000232629:[ENST00000411527(c.-5G>A)]	NA	NA	1.98E-02	5.67E-02	1.26E-01	1.98E-02	5.52E-02	NA	NA	NA	MODIFIER	NA	NA	143	143	0	93	62	30	32.26
Non-user	IOB_24	chr6	32782897	C	T	HLA-DOB	SILENT	ENSG00000241106:[ENST00000447394(c.244G>A,gaG>gaA):ENSP00000394860(p.Glu82(=))]	NA	NA	2.78E-02	1.74E-02	8.56E-02	2.78E-02	1.02E-01	NA	NA	NA	MODIFIER	NA	NA	155	153	0	114	69	45	39.47
Non-user	IOB_24	chr6	32782897	C	T	TAP2	SILENT	ENSG00000250264:[ENST00000452392(c.2106G>A,gaG>gaA):ENSP00000391806(p.Glu702(=))]	NA	NA	2.78E-02	1.74E-02	8.56E-02	2.78E-02	1.02E-01	NA	NA	NA	MODIFIER	NA	NA	155	153	0	114	69	45	39.47
Non-user	IOB_24	chr6	32943229	T	C	BRD2	SILENT	ENSG00000204256:[ENST00000449085(c.261T>C,taT>taC):ENSP00000409145(p.Tyr87(=))]	NA	NA	6.99E-03	8.77E-03	3.00E-02	6.99E-03	1.43E-02	NA	NA	NA	MODIFIER	NA	NA	78	78	0	79	43	35	44.3
Non-user	IOB_24	chr6	32948287	T	G	BRD2	INTRONIC	ENSG00000204256:[ENST00000449025(c.2286+46T>G)]	NA	NA	4.09E-02	2.99E-02	3.09E-02	4.09E-02	1.43E-02	NA	NA	NA	MODIFIER	NA	NA	180	180	0	139	69	65	46.76
Non-user	IOB_24	chr6	32948288	T	C	BRD2	NCT	ENSG00000204256:[ENST00000482838(n.574+47T>C)]	NA	NA	4.09E-02	2.99E-02	3.09E-02	4.09E-02	1.43E-02	NA	NA	NA	MODIFIER	NA	NA	180	180	0	139	68	66	47.48
Non-user	IOB_24	chr6	32976056	A	G	HLA-DOA	EXONIC-NC	ENSG00000204252:[ENST00000467465(n.254T>C)]	NA	NA	6.39E-03	7.11E-03	4.96E-02	6.39E-03	2.76E-02	NA	NA	NA	MODIFIER	NA	NA	113	113	0	104	43	61	58.65
Non-user	IOB_24	chr6	32977298	C	T	HLA-DOA	MISSENSE	ENSG00000204252:[ENST00000229829(c.16G>A,Ggg>Agg):ENSP00000229829(p.Gly6Arg)]	NA	NA	6.39E-03	8.98E-03	5.82E-02	6.39E-03	2.76E-02	NA	NA	NA	MODIFIER	NA	NA	34	33	0	49	20	29	59.18
Non-user	IOB_24	chr6	32977419	T	G	NA	INTERGENIC	NA	NA	NA	5.79E-03	NA	NA	5.79E-03	2.76E-02	NA	NA	NA	MODIFIER	NA	NA	13	13	0	18	8	10	55.56
Non-user	IOB_24	chr6	33133838	C	A	COL11A2	INTRONIC	ENSG00000204248:[ENST00000361917(c.4108-77G>T)]	NA	NA	1.28E-02	NA	NA	1.28E-02	5.42E-02	NA	NA	NA	MODIFIER	NA	NA	131	131	0	124	62	60	48.39
Non-user	IOB_24	chr6	33135699	G	A	COL11A2	INTRONIC	ENSG00000204248:[ENST00000374714(c.3883-70C>T)]	NA	NA	9.78E-03	NA	NA	9.78E-03	4.70E-02	NA	NA	NA	MODIFIER	NA	NA	104	104	0	94	46	48	51.06
Non-user	IOB_24	chr6	33139202	A	G	COL11A2	INTRONIC	ENSG00000204248:[ENST00000374708(c.3000+42T>C)]	NA	NA	5.79E-03	4.72E-03	3.31E-02	5.79E-03	2.86E-02	NA	NA	NA	MODIFIER	NA	NA	115	114	1	113	63	49	43.36
Non-user	IOB_24	chr6	33157012	G	A	COL11A2	INTRONIC	ENSG00000204248:[ENST00000341947(c.233-47C>T)]	NA	NA	1.04E-02	8.58E-03	3.96E-02	1.04E-02	3.37E-02	NA	NA	NA	MODIFIER	NA	NA	172	171	0	160	82	76	47.5
Non-user	IOB_24	chr6	33167145	A	G	RXRB	MISSENSE	ENSG00000204231:[ENST00000374680(c.284T>C,gTc>gCc):ENSP00000363812(p.Val95Ala)]	NA	NA	1.40E-02	5.80E-03	3.33E-02	1.40E-02	3.37E-02	T_lc	BN	NA	MODERATE	N	0	30	30	0	24	11	11	45.83
Non-user	IOB_24	chr6	33172755	G	A	HSD17B8	SILENT	ENSG00000204228:[ENST00000374662(c.129G>A,ctG>ctA):ENSP00000363794(p.Leu43(=))]	NA	NA	9.98E-03	1.84E-02	1.48E-01	9.98E-03	3.37E-02	NA	NA	NA	LOW	NA	NA	92	92	0	64	31	33	51.56
Non-user	IOB_24	chr6	33382955	G	C	PHF1	INTRONIC	ENSG00000112511:[ENST00000427826(c.80+34G>C)]	NA	NA	3.97E-02	2.58E-02	9.16E-02	3.97E-02	1.20E-01	NA	NA	NA	MODIFIER	NA	NA	102	102	0	75	42	33	44
Non-user	IOB_24	chr6	33391204	C	T	SYNGAP1	NCT	ENSG00000197283:[ENST00000496374(n.23-50C>T)]	NA	NA	NA	9.06E-05	6.66E-04	NA	NA	NA	NA	NA	MODIFIER	NA	NA	65	65	0	48	27	21	43.75
Non-user	IOB_24	chr6	33762451	G	A	MLN	3UTR	ENSG00000096395:[ENST00000430124(c.*151C>T)]	NA	NA	2.74E-02	NA	NA	2.74E-02	5.83E-02	NA	NA	NA	MODIFIER	NA	NA	46	45	0	36	25	11	30.56
Non-user	IOB_24	chr6	35259432	G	A	ZNF76	SILENT	ENSG00000065029:[ENST00000339411(c.849G>A,agG>agA):ENSP00000344097(p.Arg283(=))]	NA	NA	NA	3.30E-05	2.42E-04	NA	NA	NA	NA	NA	LOW	NA	1	248	247	0	217	117	97	44.7
Non-user	IOB_24	chr6	35444080	G	A	TEAD3	MISSENSE-SS-PRX	7005:[XM_005249343.1(c.530C>T,aCg>aTg):XP_005249400.1(p.Thr177Met)]	NA	NA	NA	7.27E-05	4.40E-04	NA	NA	T	BN	NA	MODERATE	NA	NA	20	20	0	28	17	11	39.29
Non-user	IOB_24	chr6	35761336	T	C	CLPSL1	3UTR	ENSG00000204140:[ENST00000428710(c.*24T>C)]	NA	NA	NA	2.05E-01	2.03E-01	NA	NA	NA	NA	NA	MODIFIER	NA	NA	100	99	0	101	87	14	13.86
Non-user	IOB_24	chr6	35911856	G	A	SLC26A8	MISSENSE	ENSG00000112053:[ENST00000394602(c.2419C>T,Ccc>Tcc):ENSP00000378100(p.Pro807Ser)]	NA	NA	2.60E-03	1.03E-02	1.05E-02	2.60E-03	3.10E-03	NA	NA	NA	MODIFIER	NA	NA	166	165	0	128	72	55	42.97
Non-user	IOB_24	chr6	36353472	C	T	ETV7	INTRONIC	51513:[NM_001207037.1(c.-212-26G>A)]	NA	NA	3.51E-02	5.37E-02	5.85E-02	3.51E-02	5.83E-02	NA	NA	NA	MODIFIER	NA	NA	63	61	0	63	38	24	38.1
Non-user	IOB_24	chr6	36464481	G	A	STK38	INTRONIC-SS-DNR-PRX	ENSG00000112079:[ENST00000229812(c.1267+7C>T)]	NA	NA	3.14E-02	5.36E-02	4.09E-02	3.13E-02	3.68E-02	NA	NA	NA	LOW	NA	NA	164	164	0	145	81	64	44.14
Non-user	IOB_24	chr6	39353538	A	G	KIF6	INTRONIC	ENSG00000164627:[ENST00000287152(c.1811-90T>C)]	NA	NA	NA	NA	NA	NA	NA	NA	NA	NA	MODIFIER	NA	NA	56	42	0	45	20	23	51.11
Non-user	IOB_24	chr6	39513481	T	C	KIF6	INTRONIC	ENSG00000164627:[ENST00000287152(c.1182-17A>G)]	NA	NA	8.99E-03	2.42E-02	1.76E-02	8.99E-03	1.43E-02	NA	NA	NA	MODIFIER	NA	NA	86	86	0	68	39	29	42.65
Non-user	IOB_24	chr6	39607551	C	T	KIF6	NCT	ENSG00000164627:[ENST00000482238(n.503-18G>A)]	NA	NA	8.99E-03	2.09E-02	1.73E-02	8.99E-03	1.74E-02	NA	NA	NA	MODIFIER	NA	NA	178	178	0	118	63	54	45.76
Non-user	IOB_24	chr6	39865007	T	C	DAAM2	MISSENSE	ENSG00000146122:[ENST00000274867(c.2567T>C,aTt>aCt):ENSP00000274867(p.Ile856Thr)]	NA	NA	1.20E-03	3.88E-03	1.64E-03	1.20E-03	0.00E+00	T	BN	NA	MODERATE	D	1	168	168	0	125	64	59	47.2
Non-user	IOB_24	chr6	39865007	T	C	LOC100505635	EXONIC-NC	100505635:[XR_242014.1(n.195A>G)]	NA	NA	1.20E-03	3.88E-03	1.64E-03	1.20E-03	0.00E+00	T	BN	NA	MODERATE	D	1	168	168	0	125	64	59	47.2
Non-user	IOB_24	chr6	39865007	T	C	RP11-61I13.3	EXONIC-NC	ENSG00000235033:[ENST00000430595(n.123A>G)]	NA	NA	1.20E-03	3.88E-03	1.64E-03	1.20E-03	0.00E+00	T	BN	NA	MODERATE	D	1	168	168	0	125	64	59	47.2
Non-user	IOB_24	chr6	41533573	G	A	FOXP4	SILENT	ENSG00000137166:[ENST00000307972(c.75G>A,ggG>ggA):ENSP00000309823(p.Gly25(=))]	NA	NA	3.51E-02	4.84E-02	7.42E-02	3.51E-02	7.16E-02	NA	NA	NA	LOW	NA	NA	108	108	0	95	45	50	52.63
Non-user	IOB_24	chr6	42185631	G	A	MRPS10	5UTR	55173:[NM_018141.3(c.-44C>T)]	NA	NA	3.42E-02	6.36E-02	9.46E-02	3.41E-02	8.38E-02	NA	NA	NA	MODIFIER	NA	NA	134	133	0	115	46	69	60
Non-user	IOB_24	chr6	44217910	A	G	HSP90AB1	INTRONIC	3326:[NM_001271972.1(c.618+19A>G)]	NA	NA	8.39E-03	1.79E-02	6.55E-03	8.39E-03	4.10E-03	NA	NA	NA	MODIFIER	NA	NA	389	387	0	309	136	171	55.34

Patel *et al.* , 2021. Whole-exome sequencing analysis of oral squamous cell carcinoma delineated by tobacco usage habits
Supplementary Table 4. List of somatic single nucleotide variants identified using whole exome sequencing of OSCC patients

Cohort	Sample	Chromosome	Genomic position	Reference allele	Alternate allele	Genesymbol	Variant type	Variant description	OncoMD	COSMIC	dbSNP	ExAC	ExAC_SAS	1000G	1000G_SAS	SIFT	Polyphen2	CONDEL	VEP	LRT	PhastCon7Way	Total reads in Normal	Reference read support in Normal	Alternative read support in Normal	Total reads in Tumor	Reference read support in Tumor	Alternate read support in Tumor	Alternate allele frequency in Tumor
Non-user	IOB_24	chr6	44233372	G	A	NFKBIE	SILENT	ENSG00000146232:[ENST00000275015(c.129C>T,ggC>ggT):ENSP00000275015(p.Gly43(=))]	NA	NA	2.20E-03	2.98E-03	6.68E-04	2.20E-03	0.00E+00	NA	NA	NA	LOW	NA	NA	93	93	0	90	58	31	34.44
Non-user	IOB_24	chr6	44270933	G	A	AARS2	NCT	57505:[XR_241907.1(n.2179+90C>T)]	NA	NA	7.99E-04	2.57E-04	1.82E-03	7.99E-04	4.10E-03	NA	NA	NA	MODIFIER	NA	NA	124	123	0	95	49	46	48.42
Non-user	IOB_24	chr6	44270933	G	A	RP11-444E17.6	INTRONIC	ENSG00000272442:[ENST00000505802(c.314-3747G>A)]	NA	NA	7.99E-04	2.57E-04	1.82E-03	7.99E-04	4.10E-03	NA	NA	NA	MODIFIER	NA	NA	124	123	0	95	49	46	48.42
Non-user	IOB_24	chr6	44270933	G	A	TMEM151B	INTRONIC	ENSG00000178233:[ENST00000438774(c.577-3747G>A)]	NA	NA	7.99E-04	2.57E-04	1.82E-03	7.99E-04	4.10E-03	NA	NA	NA	MODIFIER	NA	NA	124	123	0	95	49	46	48.42
Non-user	IOB_24	chr6	44272382	C	T	AARS2	SILENT-SS-PRX	ENSG00000124608:[ENST00000244571(c.1752G>A,gaG>gaA):ENSP00000244571(p.Glu584(=))]	NA	NA	1.34E-02	8.77E-03	2.77E-02	1.34E-02	4.09E-02	NA	NA	NA	MODIFIER	NA	NA	215	213	1	184	84	99	53.8
Non-user	IOB_24	chr6	44272382	C	T	RP11-444E17.6	INTRONIC	ENSG00000272442:[ENST00000505802(c.314-2298C>T)]	NA	NA	1.34E-02	8.77E-03	2.77E-02	1.34E-02	4.09E-02	NA	NA	NA	MODIFIER	NA	NA	215	213	1	184	84	99	53.8
Non-user	IOB_24	chr6	44272382	C	T	TMEM151B	INTRONIC	ENSG00000178233:[ENST00000438774(c.577-2298C>T)]	NA	NA	1.34E-02	8.77E-03	2.77E-02	1.34E-02	4.09E-02	NA	NA	NA	MODIFIER	NA	NA	215	213	1	184	84	99	53.8
Non-user	IOB_24	chr6	44361330	A	C	CDC5L	INTRONIC	988:[XM_005249506.1(c.356+103A>C)]	NA	NA	2.20E-03	3.93E-03	2.66E-03	2.20E-03	1.00E-03	NA	NA	NA	MODIFIER	NA	NA	101	101	0	86	45	40	46.51
Non-user	IOB_24	chr6	47575772	G	T	CD2AP	NCT-SS-PRX	ENSG00000198087:[ENST00000486693(n.157+8G>T)]	NA	NA	1.88E-02	9.97E-03	2.24E-02	1.88E-02	2.04E-02	NA	NA	NA	LOW	NA	NA	199	197	1	196	108	87	44.39
Non-user	IOB_24	chr6	49986643	C	T	DEFB110	INTRONIC	ENSG00000203970:[ENST00000393660(c.55+2951G>A)]	NA	NA	5.79E-03	3.68E-03	2.61E-02	5.79E-03	2.76E-02	NA	NA	NA	MODIFIER	NA	NA	74	73	0	65	24	41	63.08
Non-user	IOB_24	chr6	50697058	G	C	TFAP2D	INTRONIC	ENSG00000008197:[ENST00000008391(c.883+33G>C)]	NA	NA	1.20E-03	4.31E-04	3.05E-03	1.20E-03	6.10E-03	NA	NA	NA	MODIFIER	NA	NA	168	168	0	125	64	60	48
Non-user	IOB_24	chr6	50805847	T	G	TFAP2B	INTRONIC	7021:[XM_005249348.1(c.982+41T>G)]	NA	NA	NA	5.77E-05	4.24E-04	NA	NA	NA	NA	NA	MODIFIER	NA	NA	67	67	0	56	23	33	58.93
Non-user	IOB_24	chr6	51920568	A	T	PKHD1	INTRONIC	ENSG00000170927:[ENST00000340994(c.1694-41T>A)]	NA	NA	9.98E-04	8.67E-04	6.25E-03	9.98E-04	5.10E-03	NA	NA	NA	MODIFIER	NA	NA	21	21	0	12	6	6	50
Non-user	IOB_24	chr6	52962574	C	A	FBXO9	SILENT	ENSG00000112146:[ENST00000473318(c.536C>A,ccC>ccA):ENSP00000417349(p.Pro179(=))]	NA	NA	1.48E-02	7.81E-03	5.77E-02	1.48E-02	7.57E-02	NA	NA	NA	LOW	NA	NA	138	137	1	141	82	58	41.13
Non-user	IOB_24	chr6	53152711	G	C	ELOVL5	SILENT	ENSG00000012660:[ENST00000541407(c.276C>G,gcC>gcG):ENSP00000438095(p.Ala92(=))]	NA	NA	3.59E-03	1.29E-02	1.81E-02	3.59E-03	1.64E-02	NA	NA	NA	LOW	NA	NA	117	114	0	94	64	30	31.91
Non-user	IOB_24	chr6	56394545	T	C	DST	MISSENSE	ENSG00000151914:[ENST00000244364(c.9427A>G,Aat>Gat):ENSP00000244364(p.Asn3143Asp)]	NA	NA	5.99E-04	2.32E-04	1.75E-03	5.99E-04	3.10E-03	NA	NA	NA	MODIFIER	NA	NA	207	206	1	111	53	58	52.25
Non-user	IOB_24	chr6	56485023	T	C	DST	MISSENSE	ENSG00000151914:[ENST00000370765(c.3809A>G,aAa>aGa):ENSP00000359801(p.Lys1270Arg)]	NA	NA	7.99E-03	2.20E-02	1.62E-02	7.99E-03	8.20E-03	NA	NA	NA	MODIFIER	NA	NA	137	136	0	106	53	53	50
Non-user	IOB_24	chr6	56880022	C	T	BEND6	SILENT	ENSG00000151917:[ENST00000370746(c.390C>T,tcC>tcT):ENSP00000359782(p.Ser130(=))]	NA	NA	NA	1.66E-05	6.06E-05	NA	NA	NA	NA	NA	MODIFIER	NA	NA	269	269	0	203	103	100	49.26
Non-user	IOB_24	chr6	56883328	C	T	BEND6	SILENT	ENSG00000151917:[ENST00000545789(c.528C>T,aaC>aaT):ENSP00000439320(p.Asn176(=))]	NA	NA	NA	2.57E-05	2.01E-04	NA	NA	NA	NA	NA	LOW	NA	0.997	50	50	0	45	25	20	44.44
Non-user	IOB_24	chr6	57012688	C	G	LOC101927211	NCT	101927211:[NR_110742.1(n.234+23115G>C)]	NA	NA	.	8.26E-06	6.06E-05	NA	NA	NA	NA	NA	MODIFIER	NA	NA	324	321	0	240	130	109	45.42
Non-user	IOB_24	chr6	57012688	C	G	RP11-203B9.4	NCT	ENSG00000226803:[ENST00000586053(n.213+3138G>C)]	NA	NA	.	8.26E-06	6.06E-05	NA	NA	NA	NA	NA	MODIFIER	NA	NA	324	321	0	240	130	109	45.42
Non-user	IOB_24	chr6	57012688	C	G	ZNF451	MISSENSE	ENSG00000112200:[ENST00000357489(c.1805C>G,cCg>cGg):ENSP00000350083(p.Pro602Arg)]	NA	NA	.	8.26E-06	6.06E-05	NA	NA	NA	NA	NA	MODIFIER	NA	NA	324	321	0	240	130	109	45.42
Non-user	IOB_24	chr6	57398207	G	T	NA	EXONIC-NC	100996481:[XR_171038.2(n.1004G>T)]	NA	NA	NA	3.62E-02	1.16E-01	NA	NA	NA	NA	NA	MODIFIER	NA	NA	556	547	0	492	433	51	10.37
Non-user	IOB_24	chr6	57398207	G	T	PRIM2	NONSENSE	ENSG00000146143:[ENST00000607273(c.910G>T,Gga>Tga):ENSP00000475738(p.Gly304Ter)]	NA	NA	NA	3.62E-02	1.16E-01	NA	NA	NA	NA	NA	MODIFIER	NA	NA	556	547	0	492	433	51	10.37
Non-user	IOB_24	chr6	57415172	C	T	NA	NCT	100996481:[XR_171038.2(n.1114+16855C>T)]	NA	NA	1.12E-02	NA	NA	1.12E-02	5.73E-02	NA	NA	NA	MODIFIER	NA	NA	524	521	0	380	313	63	16.58
Non-user	IOB_24	chr6	57415172	C	T	PRIM2	NCT	ENSG00000146143:[ENST00000550475(n.275+16855C>T)]	NA	NA	1.12E-02	NA	NA	1.12E-02	5.73E-02	NA	NA	NA	MODIFIER	NA	NA	524	521	0	380	313	63	16.58
Non-user	IOB_24	chr6	63990101	G	A	LGSN	MISSENSE	ENSG00000146166:[ENST00000370657(c.1355C>T,cCt>cTt):ENSP00000359691(p.Pro452Leu)]	NA	NA	4.39E-03	2.57E-03	6.66E-04	4.39E-03	0.00E+00	T	BN	NA	MODERATE	N	0.015	320	315	0	227	103	123	54.19
Non-user	IOB_24	chr6	65612302	A	G	EYS	SILENT	ENSG00000188107:[ENST00000370616(c.2733T>C,aaT>aaC):ENSP00000359650(p.Asn911(=))]	NA	chr6*65612302*65612302*COSM3697932*A*G*1*large intestine*1	1.62E-02	3.59E-02	5.25E-02	1.62E-02	3.78E-02	NA	NA	NA	LOW	NA	NA	170	170	0	159	74	85	53.46
Non-user	IOB_24	chr6	69703697	C	T	BAI3	MISSENSE	ENSG00000135298:[ENST00000370598(c.1772C>T,gCa>gTa):ENSP00000359630(p.Ala591Val)]	NA	NA	1.60E-03	4.62E-04	3.40E-03	1.60E-03	8.20E-03	T	PrD	NA	MODERATE	D	1	154	154	0	136	79	56	41.18
Non-user	IOB_24	chr6	70944285	C	T	COL9A1	SILENT	ENSG00000112280:[ENST00000357250(c.2271G>A,ccG>ccA):ENSP00000349790(p.Pro757(=))]	NA	NA	4.03E-02	2.59E-02	6.84E-02	4.03E-02	6.13E-02	NA	NA	NA	MODIFIER	NA	NA	249	246	1	194	102	92	47.42
Non-user	IOB_24	chr6	70944285	C	T	RP1-149L1.1	NCT	ENSG00000253809:[ENST00000522264(n.82-7402C>T)]	NA	NA	4.03E-02	2.59E-02	6.84E-02	4.03E-02	6.13E-02	NA	NA	NA	MODIFIER	NA	NA	249	246	1	194	102	92	47.42
Non-user	IOB_24	chr6	71469557	C	A	SMAP1	MISSENSE	ENSG00000112305:[ENST00000422334(c.365C>A,gCt>gAt):ENSP00000398541(p.Ala122Asp)]	NA	NA	7.99E-04	2.06E-03	2.79E-03	7.99E-04	4.10E-03	D_lc	BN	NA	MODERATE	NA	NA	242	241	1	187	107	80	42.78
Non-user	IOB_24	chr6	72968049	C	T	RIMS1	INTRONIC	22999:[XM_005248692.1(c.2894+65C>T)]	NA	NA	NA	NA	NA	NA	NA	NA	NA	NA	MODIFIER	NA	NA	72	72	0	38	20	18	47.37
Non-user	IOB_24	chr6	74331619	C	T	SLC17A5	MISSENSE	ENSG00000119899:[ENST00000355773(c.886G>A,Gtt>Att):ENSP00000348019(p.Val296Ile)]	NA	NA	3.99E-02	2.21E-02	1.85E-02	3.99E-02	1.23E-02	NA	NA	NA	MODERATE	NA	1	230	228	0	188	96	92	48.94
Non-user	IOB_24	chr6	76163835	C	T	FILIP1	INTRONIC	ENSG00000118407:[ENST00000237172(c.-6-39141G>A)]	NA	NA	2.98E-02	NA	NA	2.98E-02	6.54E-02	NA	NA	NA	MODIFIER	NA	NA	277	275	1	185	93	91	49.19
Non-user	IOB_24	chr6	76163835	C	T	LOC101928540	NCT	101928540:[NR_125859.1(n.300-738C>T)]	NA	NA	2.98E-02	NA	NA	2.98E-02	6.54E-02	NA	NA	NA	MODIFIER	NA	NA	277	275	1	185	93	91	49.19
Non-user	IOB_24	chr6	76751738	C	T	IMPG1	MISSENSE	ENSG00000112706:[ENST00000369950(c.173G>A,aGa>aAa):ENSP00000358966(p.Arg58Lys)]	NA	NA	3.40E-03	6.07E-03	1.76E-02	3.39E-03	1.23E-02	T	BN	NA	MODERATE	N	0.085	200	198	0	148	70	77	52.03
Non-user	IOB_24	chr6	80721466	A	G	TTK	MISSENSE	ENSG00000112742:[ENST00000230510(c.749A>G,gAt>gGt):ENSP00000230510(p.Asp250Gly)]	NA	NA	3.40E-03	2.80E-03	1.99E-02	3.39E-03	1.74E-02	T	BN	NA	MODERATE	D	1	74	74	0	64	2	61	95.31
Non-user	IOB_24	chr6	84233327	T	C	PRSS35	MISSENSE	ENSG00000146250:[ENST00000369700(c.167T>C,aTg>aCg):ENSP00000358714(p.Met56Thr)]	NA	NA	3.99E-04	7.41E-05	3.03E-04	3.99E-04	1.00E-03	T	BN	NA	MODERATE	N	0.02	312	312	0	251	119	132	52.59
Non-user	IOB_24	chr6	87725583	C	T	HTR1E	SILENT	ENSG00000168830:[ENST00000305344(c.531C>T,caC>caT):ENSP00000307766(p.His177(=))]	NA	NA	2.46E-02	5.64E-02	1.94E-02	2.46E-02	1.64E-02	NA	NA	NA	LOW	NA	0.253	216	216	0	128	67	60	46.88
Non-user	IOB_24	chr6	89909204	G	A	GABRR1	INTRONIC	ENSG00000146276:[ENST00000435811(c.230-56C>T)]	NA	NA	2.34E-02	NA	NA	2.34E-02	1.53E-02	NA	NA	NA	MODIFIER	NA	NA	133	132	0	79	43	35	44.3
Non-user	IOB_24	chr6	91246007	T	A	MAP3K7	NCT	ENSG00000135341:[ENST00000479630(n.882+49A>T)]	NA	NA	3.63E-02	2.97E-02	4.95E-02	3.63E-02	5.11E-02	NA	NA	NA	MODIFIER	NA	NA	113	112	0	97	53	44	45.36
Non-user	IOB_24	chr6	91269510	C	A	MAP3K7	INTRONIC	ENSG00000135341:[ENST00000369325(c.482+285G>T)]	NA	NA	4.57E-02	NA	NA	4.57E-02	5.01E-02	NA	NA	NA	MODIFIER	NA	NA	167	166	1	120	58	62	51.67
Non-user	IOB_24	chr6	94066676	G	C	EPHA7	MISSENSE	ENSG00000135333:[ENST00000369303(c.1083C>G,aaC>aaG):ENSP00000358309(p.Asn361Lys)]	NA	NA	2.00E-04	4.86E-04	1.57E-03	2.00E-04	0.00E+00	T	BN	NA	MODERATE	D	0.049	161	161	0	129	75	54	41.86
Non-user	IOB_24	chr6	97580539	A	G	KLHL32	SILENT	ENSG00000186231:[ENST00000539200(c.1410A>G,gaA>gaG):ENSP00000441527(p.Glu470(=))]	NA	NA	3.08E-02	3.59E-02	4.63E-02	3.08E-02	4.60E-02	NA	NA	NA	LOW	NA	1	185	185	0	177	97	79	44.63
Non-user	IOB_24	chr6	105781270	G	A	PREP	MISSENSE	ENSG00000085377:[ENST00000369110(c.934C>T,Cgc>Tgc):ENSP00000358106(p.Arg312Cys)]	NA	NA	NA	9.06E-05	4.84E-04	NA	NA	D	BN	NA	MODERATE	D	1	334	334	0	246	139	107	43.5
Non-user	IOB_24	chr6	106967346	G	A	AIM1	MISSENSE	ENSG00000112297:[ENST00000369066(c.1039G>A,Gtc>Atc):ENSP00000358062(p.Val347Ile)]	NA	NA	NA	1.32E-04	6.67E-04	NA	NA	T_lc	BN	NA	MODERATE	NA	0	123	122	0	83	44	39	46.99
Non-user	IOB_24	chr6	107031213	T	A	RTN4IP1	MISSENSE	ENSG00000130347:[ENST00000369063(c.1073A>T,gAt>gTt):ENSP00000358059(p.Asp358Val)]	NA	NA	1.60E-03	9.63E-04	7.11E-03	1.60E-03	8.20E-03	NA	NA	NA	MODIFIER	NA	NA	115	114	0	87	40	47	54.02
Non-user	IOB_24	chr6	108487936	C	T	NR2E1	5UTR	ENSG00000112333:[ENST00000368986(c.-34C>T)]	NA	NA	4.57E-02	1.05E-01	9.63E-02	4.57E-02	1.09E-01	NA	NA	NA	MODIFIER	NA	NA	82	81	0	65	29	36	55.38
Non-user	IOB_24	chr6	111583530	G	C	KIAA1919	MISSENSE	ENSG00000173214:[ENST00000368847(c.98G>C,aGt>aCt):ENSP00000357840(p.Ser33Thr)]	NA	NA	7.99E-03	1.80E-02	1.91E-02	7.99E-03	2.04E-02	T	BN	NA	MODERATE	D	1	370	370	0	318	157	161	50.63
Non-user	IOB_24	chr6	112020651	T	C	FYN	INTRONIC	ENSG00000010810:[ENST00000229470(c.706+67A>G)]	NA	NA	6.99E-03	NA	NA	6.99E-03	1.23E-02	NA	NA	NA	MODIFIER	NA	NA	87	87	0	67	0	66	98.51
Non-user	IOB_24	chr6	116864998	G	A	FAM26D	MISSENSE	ENSG00000164451:[ENST00000416171(c.22G>A,Gag>Aag):ENSP00000416976(p.Glu8Lys)]	NA	NA	4.19E-03	5.39E-03	1.60E-03	4.19E-03	2.00E-03	NA	NA	NA	MODIFIER	NA	NA	215	215	0	177	98	79	44.63
Non-user	IOB_24	chr6	116864998	G	A	TRAPPC3L	INTRONIC	ENSG00000173626:[ENST00000368602(c.43-435C>T)]	NA	NA	4.19E-03	5.39E-03	1.60E-03	4.19E-03	2.00E-03	NA	NA	NA	MODIFIER	NA	NA	215	215	0	177	98	79	44.63
Non-user	IOB_24	chr6	116943975	G	A	RSPH4A	MISSENSE	ENSG00000111834:[ENST00000229554(c.731G>A,cGt>cAt):ENSP00000229554(p.Arg244His)]	NA	NA	1.20E-02	1.96E-02	2.25E-02	1.20E-02	1.94E-02	D	BN	NA	MODERATE	D	1	52	52	0	55	26	29	52.73
Non-user	IOB_24	chr6	117730899	T	G	GOPC	NCT	ENSG00000047932:[ENST00000467125(n.548-88342A>C)]	NA	NA	4.97E-02	NA	NA	4.97E-02	5.73E-02	NA	NA	NA	MODIFIER	NA	NA	67	67	0	58	35	23	39.66
Non-user	IOB_24	chr6	117730899	T	G	ROS1	INTRONIC	ENSG00000047936:[ENST00000368508(c.229-94A>C)]	NA	NA	4.97E-02	NA	NA	4.97E-02	5.73E-02	NA	NA	NA	MODIFIER	NA	NA	67	67	0	58	35	23	39.66
Non-user	IOB_24	chr6	117730907	T	C	GOPC	NCT	ENSG00000047932:[ENST00000467125(n.548-88350A>G)]	NA	NA	8.99E-03	NA	NA	8.99E-03	2.76E-02	NA	NA	NA	MODIFIER	NA	NA	67	67	0	50	20	30	60
Non-user	IOB_24	chr6	117730907	T	C	ROS1	INTRONIC	ENSG00000047936:[ENST00000368508(c.229-102A>G)]	NA	NA	8.99E-03	NA	NA	8.99E-03	2.76E-02	NA	NA	NA	MODIFIER	NA	NA	67	67	0	50	20	30	60
Non-user	IOB_24	chr6	117853411	G	A	DCBLD1	NCT	ENSG00000164465:[ENST00000533453(n.609-12G>A)]	NA	NA	2.06E-02	3.07E-02	1.79E-02	2.06E-02	1.53E-02	NA	NA	NA	MODIFIER	NA	NA	112	111	0	113	1	111	98.23
Non-user	IOB_24	chr6	117853411	G	A	GOPC	NCT	ENSG00000047932:[ENST00000467125(n.547+34606C>T)]	NA	NA	2.06E-02	3.07E-02	1.79E-02	2.06E-02	1.53E-02	NA	NA	NA	MODIFIER	NA	NA	112	111	0	113	1	111	98.23
Non-user	IOB_24	chr6	117869530	G	A	DCBLD1	SILENT	ENSG00000164465:[ENST00000338728(c.2076G>A,acG>acA):ENSP00000342422(p.Thr692(=))]	NA	NA	2.42E-02	3.22E-02	2.41E-02	2.42E-02	2.97E-02	NA	NA	NA	MODIFIER	NA	NA	301	300	0	274	122	149	54.38
Non-user	IOB_24	chr6	117869530	G	A	GOPC	NCT	ENSG00000047932:[ENST00000467125(n.547+18487C>T)]	NA	NA	2.42E-02	3.22E-02	2.41E-02	2.42E-02	2.97E-02	NA	NA	NA	MODIFIER	NA	NA	301	300	0	274	122	149	54.38
Non-user	IOB_24	chr6	117896728	A	C	GOPC	INTRONIC	ENSG00000047932:[ENST00000052569(c.451-213T>G)]	NA	NA	3.20E-02	4.17E-02	3.37E-02	3.19E-02	4.29E-02	NA	NA	NA	MODIFIER	NA	NA	35	35	0	33	13	20	60.61
Non-user	IOB_24	chr6	119345240	T	C	FAM184A	MISSENSE	ENSG00000111879:[ENST00000352896(c.538A>G,Aaa>Gaa):ENSP00000326608(p.Lys180Glu)]	NA	NA	NA	NA	NA	NA	NA	NA	NA	NA	MODIFIER	NA	NA	176	176	0	154	74	80	51.95
Non-user	IOB_24	chr6	119345240	T	C	RP11-351A11.1	NCT	ENSG00000253194:[ENST00000518570(n.335-7330T>C)]	NA	NA	NA	NA	NA	NA	NA	NA	NA	NA	MODIFIER	NA	NA	176	176	0	154	74	80	51.95
Non-user	IOB_24	chr6	121491503	A	T	TBC1D32	NCT	ENSG00000146350:[ENST00000398197(n.210-9301T>A)]	NA	NA	1.26E-02	1.37E-02	4.32E-02	1.26E-02	3.07E-02	NA	NA	NA	MODIFIER	NA	NA	91	91	0	90	46	44	48.89
Non-user	IOB_24	chr6	121768710	G	A	GJA1	SILENT	ENSG00000152661:[ENST00000282561(c.717G>A,cgG>cgA):ENSP00000282561(p.Arg239(=))]	NA	NA	3.44E-02	1.99E-02	3.24E-02	3.43E-02	2.56E-02	NA	NA	NA	LOW	NA	NA	176	176	0	111	52	59	53.15
Non-user	IOB_24	chr6	123110451	G	C	SMPDL3A	5UTR	10924:[NM_001286138.1(c.-220G>C)]	NA	NA	1.40E-02	2.97E-02	2.75E-02	1.40E-02	1.74E-02	NA	NA	NA	MODIFIER	NA	NA	144	144	0	115	75	39	33.91
Non-user	IOB_24	chr6	123869689	C	A	TRDN	MISSENSE	ENSG00000186439:[ENST00000334268(c.301G>T,Gac>Tac):ENSP00000333984(p.Asp101Tyr)]	NA	NA	NA	NA	NA	NA	NA	D	PrD	NA	MODERATE	D	0.998	170	169	0	159	150	5	3.14
Non-user	IOB_24	chr6	125233709	C	A	RNF217-AS1	EXONIC-NC	7955:[NR_026876.1(n.1025G>T)]	NA	NA	NA	NA	NA	NA	NA	NA	NA	NA	MODIFIER	NA	NA	235	234	0	199	194	5	2.51
Non-user	IOB_24	chr6	125233709	C	A	RP11-510H23.1	EXONIC-NC	ENSG00000236548:[ENST00000439075(n.1037G>T)]	NA	NA	NA	NA	NA	NA	NA	NA	NA	NA	MODIFIER	NA	NA	235	234	0	199	194	5	2.51
Non-user	IOB_24	chr6	125284594	C	T	RNF217	NCT	ENSG00000146373:[ENST00000519971(n.178+22C>T)]	NA	NA	9.98E-04	1.33E-03	1.16E-02	9.98E-04	5.10E-03	NA	NA	NA	MODIFIER	NA	NA	157	156	0	118	67	50	42.37
Non-user	IOB_24	chr6	126073140	T	C	HEY2	INTRONIC	ENSG00000135547:[ENST00000368364(c.163-13T>C)]	NA	NA	3.79E-03	8.22E-03	3.82E-03	3.79E-03	1.00E-03	NA	NA	NA	MODIFIER	NA	NA	41	40	0	37	20	16	43.24
Non-user	IOB_24	chr6	129511468	G	A	LAMA2	MISSENSE	ENSG00000196569:[ENST00000421865(c.1586G>A,aGt>aAt):ENSP00000400365(p.Ser529Asn)]	NA	NA	8.19E-03	3.25E-03	2.37E-02	8.19E-03	4.19E-02	D	BN	NA	MODERATE	D	0.867	342	341	0	306	166	140	45.75
Non-user	IOB_24	chr6	135299909	A	G	HBS1L	MISSENSE	ENSG00000112339:[ENST00000445176(c.877T>C,Tgt>Cgt):ENSP00000415305(p.Cys293Arg)]	NA	NA	NA	NA	NA	NA	NA	NA	NA	NA	MODIFIER	NA	NA	156	156	0	134	128	6	4.48
Non-user	IOB_24	chr6	135502706	G	A	MYB	INTRONIC	ENSG00000118513:[ENST00000316528(c.23+32G>A)]	NA	NA	chr6*135502703*135502713*GGCGGGGGCGC*G*rs555253038*1*0.9992*0.0007987	NA	NA	NA	NA	NA	NA	NA	MODIFIER	NA	NA	33	33	0	22	13	9	40.91
Non-user	IOB_24	chr6	135518264	A	G	MYB	MISSENSE	ENSG00000118513:[ENST00000438901(c.1321A>G,Agt>Ggt):ENSP00000413011(p.Ser441Gly)]	NA	NA	NA	NA	NA	NA	NA	NA	NA	NA	MODIFIER	NA	NA	106	106	0	69	28	41	59.42
Non-user	IOB_24	chr6	138726268	C	T	HEBP2	INTRONIC	ENSG00000051620:[ENST00000448741(c.136-14C>T)]	NA	NA	2.04E-02	3.06E-02	4.13E-02	2.04E-02	4.60E-02	NA	NA	NA	MODIFIER	NA	NA	129	128	1	118	54	62	52.54
Non-user	IOB_24	chr6	139355420	C	A	ABRACL	SILENT	ENSG00000146386:[ENST00000461027(c.123C>A,ggC>ggA):ENSP00000418406(p.Gly41(=))]	NA	NA	4.99E-03	4.12E-03	2.88E-02	4.99E-03	2.56E-02	NA	NA	NA	LOW	NA	NA	107	107	0	81	39	42	51.85
Non-user	IOB_24	chr6	142519685	T	G	VTA1	SILENT	ENSG00000009844:[ENST00000367630(c.630T>G,acT>acG):ENSP00000356602(p.Thr210(=))]	NA	NA	NA	NA	NA	NA	NA	NA	NA	NA	LOW	NA	NA	141	140	0	107	57	49	45.79
Non-user	IOB_24	chr6	144780322	A	C	UTRN	MISSENSE	ENSG00000152818:[ENST00000367545(c.2539A>C,Aat>Cat):ENSP00000356515(p.Asn847His)]	NA	NA	2.00E-04	8.50E-04	4.26E-03	2.00E-04	0.00E+00	NA	NA	NA	MODERATE	NA	0	140	139	1	81	39	41	50.62
Non-user	IOB_24	chr6	145075963	A	G	UTRN	INTRONIC	ENSG00000152818:[ENST00000367545(c.8434+29A>G)]	NA	NA	3.18E-02	4.02E-02	3.50E-02	3.17E-02	4.09E-02	NA	NA	NA	MODIFIER	NA	NA	125	125	0	101	39	62	61.39
Non-user	IOB_24	chr6	145079033	A	G	UTRN	INTRONIC	7402:[XM_005267128.1(c.8447-32A>G)]	NA	NA	3.44E-02	4.18E-02	4.78E-02	3.43E-02	5.32E-02	NA	NA	NA	MODIFIER	NA	NA	316	314	1	221	122	98	44.34
Non-user	IOB_24	chr6	145093052	A	G	UTRN	SILENT	ENSG00000152818:[ENST00000367545(c.8505A>G,acA>acG):ENSP00000356515(p.Thr2835(=))]	NA	NA	3.42E-02	4.22E-02	4.78E-02	3.41E-02	5.42E-02	NA	NA	NA	LOW	NA	NA	56	55	0	57	35	20	35.09
Non-user	IOB_24	chr6	145103215	C	T	UTRN	SILENT	ENSG00000152818:[ENST00000367545(c.8790C>T,ctC>ctT):ENSP00000356515(p.Leu2930(=))]	NA	NA	4.13E-02	4.73E-02	7.74E-02	4.13E-02	8.90E-02	NA	NA	NA	LOW	NA	NA	226	225	0	180	89	90	50

Patel *et al.* , 2021. Whole-exome sequencing analysis of oral squamous cell carcinoma delineated by tobacco usage habits
Supplementary Table 4. List of somatic single nucleotide variants identified using whole exome sequencing of OSCC patients

Cohort	Sample	Chromosome	Genomic position	Reference allele	Alternate allele	Genesymbol	Variant type	Variant description	OncoMD	COSMIC	dbSNP	ExAC	ExAC_SAS	1000G	1000G_SAS	SIFT	Polyphen2	CONDEL	VEP	LRT	PhastCon7Way	Total reads in Normal	Reference read support in Normal	Alternative read support in Normal	Total reads in Tumor	Reference read support in Tumor	Alternate read support in Tumor	Alternate allele frequency in Tumor
Non-user	IOB_24	chr6	147049803	G	T	ADGB	MISSENSE	ENSG00000118492:[ENST00000397944(c.2446G>T,Gct>Tct):ENSP00000381036(p.Ala816Ser)]	NA	NA	1.80E-03	5.21E-03	1.28E-02	1.80E-03	9.20E-03	T	PrD	NA	MODERATE	NA	NA	188	188	0	151	81	70	46.36
Non-user	IOB_24	chr6	147055043	G	A	ADGB	MISSENSE	ENSG00000118492:[ENST00000480328(c.930G>A,Gtt>Att):ENSP00000429315(p.Val311Ile)]	NA	NA	5.59E-03	1.10E-02	2.09E-02	5.59E-03	1.64E-02	NA	NA	NA	MODIFIER	NA	NA	131	130	0	121	76	44	36.36
Non-user	IOB_24	chr6	147830212	G	C	SAMD5	MISSENSE	ENSG00000203727:[ENST00000367474(c.148G>C,Gcg>Ccg):ENSP00000356444(p.Ala50Pro)]	NA	NA	4.39E-02	7.15E-02	4.35E-02	4.39E-02	3.89E-02	T	BN	NA	MODERATE	N	1	329	327	0	283	128	154	54.42
Non-user	IOB_24	chr6	149691154	A	G	TAB2	SILENT	ENSG00000055208:[ENST00000286332(c.21A>G,caA>caG):ENSP00000286332(p.Gln7(=))]	NA	NA	4.39E-03	1.86E-03	1.31E-02	4.39E-03	2.15E-02	NA	NA	NA	MODIFIER	NA	NA	145	144	0	133	68	65	48.87
Non-user	IOB_24	chr6	149997325	G	A	LATS1	INTRONIC	ENSG00000131023:[ENST00000253339(c.2883+71C>T)]	NA	NA	5.99E-04	1.45E-03	5.98E-03	5.99E-04	3.10E-03	NA	NA	NA	MODIFIER	NA	NA	47	47	0	29	12	17	58.62
Non-user	IOB_24	chr6	150343148	A	C	RAET1L	MISSENSE	ENSG00000155918:[ENST00000286380(c.317T>G,cTt>cGt):ENSP00000286380(p.Leu106Arg)]	NA	NA	NA	3.59E-02	2.20E-02	NA	NA	T	BN	NA	MODERATE	NA	NA	138	129	9	33	0	33	100
Non-user	IOB_24	chr6	151121958	C	T	PLEKHG1	MISSENSE	ENSG00000120278:[ENST00000358517(c.733C>T,Ctc>Ttc):ENSP00000351318(p.Leu245Phe)]	NA	NA	1.20E-03	5.60E-04	1.94E-03	1.20E-03	1.00E-03	D	PrD	NA	MODERATE	NA	NA	86	86	0	78	28	50	64.1
Non-user	IOB_24	chr6	151203948	T	C	MTHFD1L	SILENT	ENSG00000120254:[ENST00000443074(c.3T>C,ctT>ctC):ENSP00000415039(p.Leu1(=))]	NA	NA	NA	NA	NA	NA	NA	NA	NA	NA	LOW	NA	NA	160	160	0	143	88	55	38.46
Non-user	IOB_24	chr6	151673070	G	A	AKAP12	MISSENSE	ENSG00000131016:[ENST00000253332(c.3544G>A,Gca>Aca):ENSP00000253332(p.Ala1182Thr)]	NA	chr6*151673070*151673070*COSM3702906*G*C*1*liver*1	6.39E-03	1.50E-02	3.28E-02	6.39E-03	1.94E-02	T	BN	NA	MODERATE	N	0	119	118	0	109	54	54	49.54
Non-user	IOB_24	chr6	151815365	T	C	CCDC170	INTRONIC	ENSG00000120262:[ENST00000239374(c.57+45T>C)]	NA	NA	3.40E-03	6.40E-03	8.78E-03	3.39E-03	1.74E-02	NA	NA	NA	MODIFIER	NA	NA	72	72	0	52	34	18	34.62
Non-user	IOB_24	chr6	157739931	T	C	TMEM242	SILENT	ENSG00000215712:[ENST00000367144(c.210A>G,gcA>gcG):ENSP00000356112(p.Ala70(=))]	NA	NA	1.48E-02	3.45E-02	2.28E-02	1.48E-02	1.53E-02	NA	NA	NA	LOW	NA	NA	116	116	0	101	51	50	49.5
Non-user	IOB_24	chr6	159655190	G	A	FNDC1	MISSENSE	ENSG00000164694:[ENST00000329629(c.3332G>A,Gat>Aat):ENSP00000333297(p.Asp1112Asn)]	NA	chr6*159655190*159655190*COSM1311774*G*A*1*urinary_tract*1	7.39E-03	1.66E-02	7.92E-03	7.39E-03	2.00E-03	T_lc	BN	NA	MODERATE	N	0.095	164	163	0	87	43	44	50.57
Non-user	IOB_24	chr6	160953642	A	G	LPA	MISSENSE	ENSG00000198670:[ENST00000316300(c.5882T>C,cTt>cCt):ENSP00000321334(p.Leu1961Pro)]	NA	NA	1.22E-02	2.07E-02	3.40E-02	1.22E-02	3.68E-02	NA	NA	NA	MODERATE	NA	NA	162	161	0	127	56	71	55.91
Non-user	IOB_24	chr6	161152242	T	A	PLG	MISSENSE	ENSG00000122194:[ENST00000308192(c.1416T>A,gaT>gaA):ENSP00000308938(p.Asp472Glu)]	NA	NA	NA	8.24E-05	4.24E-04	NA	NA	NA	NA	NA	MODIFIER	NA	NA	61	61	0	39	18	20	51.28
Non-user	IOB_24	chr6	162204609	G	A	PARK2	INTRONIC	ENSG00000185345:[ENST00000338468(c.298+2195C>T)]	NA	NA	1.30E-02	NA	NA	1.30E-02	3.89E-02	NA	NA	NA	MODIFIER	NA	NA	54	54	0	47	20	27	57.45
Non-user	IOB_24	chr6	162204641	A	C	PARK2	INTRONIC	ENSG00000185345:[ENST00000366897(c.787+2163T>G)]	NA	NA	3.22E-02	NA	NA	3.21E-02	3.89E-02	NA	NA	NA	MODIFIER	NA	NA	69	69	0	61	28	32	52.46
Non-user	IOB_24	chr6	166779464	G	A	MPC1	SILENT-SS-PRX	ENSG00000060762:[ENST00000475708(n.527C>T)]	NA	NA	3.20E-02	4.99E-02	8.39E-02	3.19E-02	7.16E-02	NA	NA	NA	LOW	NA	NA	105	105	0	61	33	28	45.9
Non-user	IOB_24	chr6	167352306	C	A	RNASET2	INTRONIC	ENSG00000026297:[ENST00000476238(c.446+77G>T)]	NA	NA	2.40E-03	4.05E-03	1.21E-02	2.40E-03	7.20E-03	NA	NA	NA	MODIFIER	NA	NA	148	148	0	127	70	57	44.88
Non-user	IOB_24	chr6	167352306	C	A	RP11-514O12.4	INTRONIC	ENSG00000249141:[ENST00000507747(c.388+77G>T)]	NA	NA	2.40E-03	4.05E-03	1.21E-02	2.40E-03	7.20E-03	NA	NA	NA	MODIFIER	NA	NA	148	148	0	127	70	57	44.88
Non-user	IOB_24	chr6	167411301	G	A	MIR3939	EXONIC-NC	ENSG00000265828:[ENST00000582614(n.100C>T)]	NA	NA	NA	2.88E-01	1.47E-01	NA	NA	NA	NA	NA	MODIFIER	NA	NA	66	66	0	106	6	5	4.72
Non-user	IOB_24	chr6	167411301	G	A	RP1-167A14.2	NCT	ENSG00000227598:[ENST00000444102(n.148+1105C>T)]	NA	NA	NA	2.88E-01	1.47E-01	NA	NA	NA	NA	NA	MODIFIER	NA	NA	66	66	0	106	6	5	4.72
Non-user	IOB_24	chr6	167411334	C	T	MIR3939	MATURE-MIRNA	ENSG00000265828:[ENST00000582614(n.67G>A)]	NA	NA	NA	1.00E-01	1.16E-02	NA	NA	NA	NA	NA	MODIFIER	NA	NA	86	86	0	130	95	29	22.31
Non-user	IOB_24	chr6	167411334	C	T	RP1-167A14.2	NCT	ENSG00000227598:[ENST00000444102(n.148+1072G>A)]	NA	NA	NA	1.00E-01	1.16E-02	NA	NA	NA	NA	NA	MODIFIER	NA	NA	86	86	0	130	95	29	22.31
Non-user	IOB_24	chr6	167411337	G	A	MIR3939	MATURE-MIRNA	ENSG00000265828:[ENST00000582614(n.64C>T)]	NA	NA	NA	8.09E-02	1.09E-02	NA	NA	NA	NA	NA	MODIFIER	NA	NA	88	87	0	133	100	29	21.8
Non-user	IOB_24	chr6	167411337	G	A	RP1-167A14.2	NCT	ENSG00000227598:[ENST00000444102(n.148+1069C>T)]	NA	NA	NA	8.09E-02	1.09E-02	NA	NA	NA	NA	NA	MODIFIER	NA	NA	88	87	0	133	100	29	21.8
Non-user	IOB_24	chr6	167411362	G	A	MIR3939	EXONIC-NC	ENSG00000265828:[ENST00000582614(n.39C>T)]	NA	NA	NA	3.39E-02	0.00E+00	NA	NA	NA	NA	NA	MODIFIER	NA	NA	115	115	0	159	127	31	19.5
Non-user	IOB_24	chr6	167411362	G	A	RP1-167A14.2	NCT	ENSG00000227598:[ENST00000444102(n.148+1044C>T)]	NA	NA	NA	3.39E-02	0.00E+00	NA	NA	NA	NA	NA	MODIFIER	NA	NA	115	115	0	159	127	31	19.5
Non-user	IOB_24	chr6	167411388	C	G	MIR3939	EXONIC-NC	ENSG00000265828:[ENST00000582614(n.13G>C)]	NA	NA	NA	2.96E-02	1.19E-02	NA	NA	NA	NA	NA	MODIFIER	NA	NA	130	130	0	173	140	33	19.08
Non-user	IOB_24	chr6	167411388	C	G	RP1-167A14.2	NCT	ENSG00000227598:[ENST00000444102(n.148+1018G>C)]	NA	NA	NA	2.96E-02	1.19E-02	NA	NA	NA	NA	NA	MODIFIER	NA	NA	130	130	0	173	140	33	19.08
Non-user	IOB_24	chr6	167411401	C	T	RP1-167A14.2	NCT	ENSG00000227598:[ENST00000444102(n.148+1005G>A)]	NA	NA	NA	2.19E-02	1.28E-02	NA	NA	NA	NA	NA	MODIFIER	NA	NA	139	139	0	168	134	32	19.05
Non-user	IOB_24	chr6	167591954	G	A	TCP10L2	MISSENSE	ENSG00000166984:[ENST00000283507(c.581G>A,cGt>cAt):ENSP00000283507(p.Arg194His)]	NA	NA	NA	1.81E-01	1.94E-01	NA	NA	NA	NA	NA	MODIFIER	NA	NA	100	100	0	142	120	21	14.79
Non-user	IOB_24	chr6	167591956	C	T	TCP10L2	NONSENSE	ENSG00000166984:[ENST00000283507(c.583C>T,Caa>Taa):ENSP00000283507(p.Gln195Ter)]	NA	NA	NA	1.59E-01	1.33E-01	NA	NA	NA	NA	NA	MODIFIER	NA	NA	101	101	0	143	111	31	21.68
Non-user	IOB_24	chr6	167791536	A	G	TCP10	SILENT	ENSG00000203690:[ENST00000366827(c.324T>C,gaT>gaC):ENSP00000355792(p.Asp108(=))]	NA	NA	2.32E-02	3.70E-02	4.39E-02	2.32E-02	2.56E-02	NA	NA	NA	LOW	NA	NA	86	85	0	100	36	63	63
Non-user	IOB_24	chr6	167794588	A	G	TCP10	SILENT	ENSG00000203690:[ENST00000476779(c.300T>C,tgT>tgC):ENSP00000427675(p.Cys100(=))]	NA	NA	4.39E-03	1.28E-02	7.16E-03	4.39E-03	5.10E-03	NA	NA	NA	MODIFIER	NA	NA	152	151	0	56	1	55	98.21
Non-user	IOB_24	chr6	168431498	G	A	KIF25	SILENT	ENSG00000125337:[ENST00000351261(c.138G>A,gtG>gtA):ENSP00000252688(p.Val46(=))]	NA	chr6*168431498*168431498*COSM85018*G*A*1*pancreas*1	4.39E-03	6.13E-03	2.93E-03	4.39E-03	0.00E+00	NA	NA	NA	MODIFIER	NA	NA	245	244	0	159	90	69	43.4
Non-user	IOB_24	chr6	168440829	G	A	KIF25	SILENT	ENSG00000125337:[ENST00000351261(c.579G>A,gcG>gcA):ENSP00000252688(p.Ala193(=))]	NA	NA	2.60E-02	2.56E-02	1.21E-02	2.60E-02	4.10E-03	NA	NA	NA	LOW	NA	NA	97	96	1	101	55	46	45.54
Non-user	IOB_24	chr6	168694549	G	A	DACT2	3UTR	ENSG00000164488:[ENST00000366796(c.*25C>T)]	NA	NA	1.60E-03	4.48E-03	2.23E-03	1.60E-03	0.00E+00	NA	NA	NA	MODIFIER	NA	NA	74	74	0	40	13	27	67.5
Non-user	IOB_24	chr7	299720	G	A	FAM20C	MISSENSE	ENSG00000177706:[ENST00000313766(c.1529G>A,cGt>cAt):ENSP00000322323(p.Arg510His)]	NA	NA	NA	6.15E-05	0.00E+00	NA	NA	T	PrD	NA	MODERATE	NA	NA	158	158	0	189	100	87	46.03
Non-user	IOB_24	chr7	821869	G	A	DNAAF5	NCT	54919:[NR_075098.1(n.2389+2088G>A)]	NA	NA	NA	NA	NA	NA	NA	NA	NA	NA	MODIFIER	NA	NA	191	172	5	210	156	41	19.52
Non-user	IOB_24	chr7	821869	G	A	HEATR2	INTRONIC	ENSG00000164818:[ENST00000440747(c.1835+2088G>A)]	NA	NA	NA	NA	NA	NA	NA	NA	NA	NA	MODIFIER	NA	NA	191	172	5	210	156	41	19.52
Non-user	IOB_24	chr7	1481903	C	A	MICALL2	MISSENSE	ENSG00000164877:[ENST00000413446(c.1510G>T,Ggc>Tgc):ENSP00000405415(p.Gly504Cys)]	NA	NA	3.40E-03	4.49E-03	1.78E-02	3.39E-03	1.53E-02	NA	NA	NA	MODIFIER	NA	NA	248	245	0	182	92	90	49.45
Non-user	IOB_24	chr7	1542487	G	A	INTS1	INTRONIC	ENSG00000164880:[ENST00000389470(c.733+50C>T)]	NA	NA	NA	9.21E-05	6.36E-04	NA	NA	NA	NA	NA	MODIFIER	NA	NA	114	114	0	113	58	54	47.79
Non-user	IOB_24	chr7	1784529	C	T	ELFN1	SILENT	ENSG00000225968:[ENST00000424383(c.297C>T,atC>atT):ENSP00000456548(p.Ile99(=))]	NA	NA	2.40E-03	7.34E-03	1.70E-02	2.40E-03	1.23E-02	NA	NA	NA	LOW	NA	NA	546	545	0	523	289	233	44.55
Non-user	IOB_24	chr7	2257681	A	G	MAD1L1	INTRONIC-SS-ACR-PRX	ENSG00000002822:[ENST00000265854(c.597-3T>C)]	NA	NA	NA	1.66E-05	6.06E-05	NA	NA	NA	NA	NA	LOW	NA	NA	66	66	0	58	35	23	39.66
Non-user	IOB_24	chr7	2649704	T	G	IQCE	MISSENSE	ENSG00000106012:[ENST00000404984(c.1843T>G,Ttg>Gtg):ENSP00000385945(p.Leu615Val)]	NA	NA	chr7*2649704*2649704*T*A,G*rs3735109*NA*NA*NA	4.56E-01	4.95E-01	NA	NA	NA	NA	NA	MODIFIER	NA	NA	235	235	0	185	77	108	58.38
Non-user	IOB_24	chr7	2691793	C	A	TTYH3	INTRONIC	ENSG00000136295:[ENST00000407643(c.776-28C>A)]	NA	NA	NA	NA	NA	NA	NA	NA	NA	NA	MODIFIER	NA	NA	111	109	0	101	96	5	4.95
Non-user	IOB_24	chr7	3186829	G	A	AC091801.1	INTRONIC	ENSG00000217455:[ENST00000458648(c.93-6899C>T)]	NA	NA	4.41E-02	7.51E-02	1.15E-01	4.41E-02	1.39E-01	NA	NA	NA	MODIFIER	NA	NA	119	118	0	93	0	93	100
Non-user	IOB_24	chr7	3186829	G	A	LOC100129603	NCT	100129603:[XR_242122.1(n.179-5995C>T)]	NA	NA	4.41E-02	7.51E-02	1.15E-01	4.41E-02	1.39E-01	NA	NA	NA	MODIFIER	NA	NA	119	118	0	93	0	93	100
Non-user	IOB_24	chr7	4791255	C	T	FOXK1	INTRONIC	ENSG00000164916:[ENST00000460979(c.166-2835C>T)]	NA	NA	5.59E-03	NA	NA	5.59E-03	9.20E-03	NA	NA	NA	MODIFIER	NA	NA	218	217	0	172	94	77	44.77
Non-user	IOB_24	chr7	4829449	C	T	AP5Z1	NCT	ENSG00000242802:[ENST00000469614(n.727-14C>T)]	NA	NA	1.36E-02	4.66E-02	4.64E-02	1.36E-02	3.07E-02	NA	NA	NA	MODIFIER	NA	NA	129	129	0	88	55	33	37.5
Non-user	IOB_24	chr7	4829458	C	T	AP5Z1	NCT-SS-PRX	ENSG00000242802:[ENST00000496303(n.1772-5C>T)]	NA	NA	5.59E-03	1.29E-02	1.40E-02	5.59E-03	8.20E-03	NA	NA	NA	LOW	NA	NA	139	139	0	91	58	33	36.26
Non-user	IOB_24	chr7	4839990	C	A	RADIL	INTRONIC	ENSG00000157927:[ENST00000445392(c.*1614-49G>T)]	NA	NA	3.81E-02	5.13E-02	6.57E-02	3.81E-02	5.83E-02	NA	NA	NA	MODIFIER	NA	NA	73	72	0	50	27	19	38
Non-user	IOB_24	chr7	4841596	G	C	RADIL	MISSENSE	ENSG00000157927:[ENST00000538469(c.1810C>G,Ctg>Gtg):ENSP00000442966(p.Leu604Val)]	NA	NA	NA	NA	NA	NA	NA	NA	NA	NA	MODIFIER	NA	NA	71	71	0	41	19	22	53.66
Non-user	IOB_24	chr7	5256333	G	C	WIPI2	NCT	ENSG00000157954:[ENST00000480238(n.176+43G>C)]	NA	NA	1.92E-02	2.43E-02	2.66E-02	1.92E-02	1.94E-02	NA	NA	NA	MODIFIER	NA	NA	101	101	0	81	46	33	40.74
Non-user	IOB_24	chr7	5372499	C	T	TNRC18	SILENT	ENSG00000182095:[ENST00000455076(c.11G>A,aaG>aaA):ENSP00000398809(p.Lys4(=))]	NA	NA	1.34E-02	3.49E-02	3.62E-02	1.34E-02	4.70E-02	NA	NA	NA	LOW	NA	0.997	87	86	0	80	38	42	52.5
Non-user	IOB_24	chr7	5770330	T	C	RNF216	INTRONIC	ENSG00000011275:[ENST00000389902(c.1224+16A>G)]	NA	NA	NA	3.06E-01	3.25E-01	NA	NA	NA	NA	NA	MODIFIER	NA	NA	48	47	0	103	69	33	32.04
Non-user	IOB_24	chr7	5984721	C	T	RSPH10B	SILENT	ENSG00000155026:[ENST00000337579(c.1503G>A,gcG>gcA):ENSP00000338556(p.Ala501(=))]	NA	NA	NA	3.64E-01	4.29E-01	NA	NA	NA	NA	NA	MODIFIER	NA	NA	70	69	0	26	17	9	34.62
Non-user	IOB_24	chr7	6006547	G	A	RSPH10B	SILENT	ENSG00000155026:[ENST00000337579(c.201C>T,aaC>aaT):ENSP00000338556(p.Asn67(=))]	NA	NA	NA	2.31E-03	1.54E-02	NA	NA	NA	NA	NA	LOW	NA	0	268	267	0	525	427	95	18.1
Non-user	IOB_24	chr7	6066116	A	T	EIF2AK1	MISSENSE	ENSG00000086232:[ENST00000422786(c.68T>A,Tgt>Agt):ENSP00000392734(p.Cys24Ser)]	NA	NA	NA	NA	NA	NA	NA	NA	UN	NA	MODERATE	NA	NA	182	182	0	93	54	39	41.94
Non-user	IOB_24	chr7	6078231	C	T	EIF2AK1	SILENT	ENSG00000086232:[ENST00000536084(c.819G>A,gaG>gaA):ENSP00000445784(p.Glu273(=))]	NA	NA	1.40E-03	1.35E-03	9.81E-03	1.40E-03	7.20E-03	NA	NA	NA	LOW	NA	0.952	92	92	0	95	50	45	47.37
Non-user	IOB_24	chr7	6098685	C	T	EIF2AK1	SILENT	ENSG00000086232:[ENST00000199389(c.30G>A,aaG>aaA):ENSP00000199389(p.Lys10(=))]	NA	NA	4.99E-03	2.92E-02	2.89E-02	4.99E-03	1.53E-02	NA	NA	NA	MODIFIER	NA	NA	98	98	0	93	53	40	43.01
Non-user	IOB_24	chr7	6797520	G	C	RSPH10B2	MISSENSE	ENSG00000169402:[ENST00000297186(c.212G>C,aGc>aCc):ENSP00000297186(p.Ser71Thr)]	NA	NA	NA	8.60E-06	0.00E+00	NA	NA	NA	NA	NA	MODIFIER	NA	NA	339	335	4	16	0	15	93.75
Non-user	IOB_24	chr7	8261042	G	C	ICA1	INTRONIC	ENSG00000003147:[ENST00000265577(c.254-14C>G)]	NA	NA	6.99E-03	1.49E-02	1.15E-02	6.99E-03	1.23E-02	NA	NA	NA	MODIFIER	NA	NA	161	160	0	146	81	65	44.52
Non-user	IOB_24	chr7	8261110	C	T	ICA1	NCT	ENSG00000003147:[ENST00000486677(n.52+40614G>A)]	NA	NA	8.19E-03	NA	NA	8.19E-03	1.33E-02	NA	NA	NA	MODIFIER	NA	NA	112	111	0	96	56	40	41.67
Non-user	IOB_24	chr7	15652075	C	T	MEOX2	SILENT	ENSG00000106511:[ENST00000262041(c.852G>A,ggG>ggA):ENSP00000262041(p.Gly284(=))]	NA	NA	NA	8.24E-06	0.00E+00	NA	NA	NA	NA	NA	LOW	NA	NA	76	74	1	67	35	32	47.76
Non-user	IOB_24	chr7	16793565	G	C	TSPAN13	5UTR	ENSG00000106537:[ENST00000262067(c.-28G>C)]	NA	NA	chr7*16793565*16793565*G*A,C*rs367827729*NA*NA*NA	1.13E-04	8.02E-04	NA	NA	NA	NA	NA	MODIFIER	NA	NA	138	138	0	135	70	65	48.15
Non-user	IOB_24	chr7	21468466	T	C	SP4	EXONIC-NC	6671:[NR_137166.1(n.397T>C)]	NA	NA	9.98E-04	NA	NA	9.98E-04	5.10E-03	NA	NA	NA	MODIFIER	NA	NA	53	52	0	40	29	11	27.5
Non-user	IOB_24	chr7	21631097	C	T	DNAH11	NONSENSE	ENSG00000105877:[ENST00000328843(c.2569C>T,Cga>Tga):ENSP00000330671(p.Arg857Ter)]	NA	NA	NA	1.67E-05	0.00E+00	NA	NA	NA	NA	NA	HIGH	NA	0.175	109	108	1	95	51	43	45.26
Non-user	IOB_24	chr7	21641247	A	G	DNAH11	INTRONIC	ENSG00000105877:[ENST00000328843(c.3648+11A>G)]	NA	NA	2.00E-04	3.23E-04	2.25E-03	2.00E-04	1.00E-03	NA	NA	NA	MODIFIER	NA	NA	72	72	0	65	29	36	55.38
Non-user	IOB_24	chr7	23757162	G	C	STK31	MISSENSE	ENSG00000196335:[ENST00000354639(c.144G>C,caG>caC):ENSP00000346660(p.Gln48His)]	NA	NA	NA	2.95E-01	3.57E-01	NA	NA	NA	NA	NA	MODIFIER	NA	NA	162	160	1	124	53	70	56.45
Non-user	IOB_24	chr7	24844066	C	G	OSBPL3	INTRONIC-SS-ACR-PRX	ENSG00000070882:[ENST00000409069(c.2244-10G>C)]	NA	NA	NA	1.86E-01	3.21E-01	NA	NA	NA	NA	NA	MODIFIER	NA	NA	44	43	0	31	11	19	61.29
Non-user	IOB_24	chr7	29332474	G	A	CHN2	INTRONIC	ENSG00000106069:[ENST00000222792(c.50-61767G>A)]	NA	NA	4.05E-02	NA	NA	4.05E-02	1.64E-02	NA	NA	NA	MODIFIER	NA	NA	192	191	0	142	91	50	35.21
Non-user	IOB_24	chr7	29332476	A	T	CHN2	INTRONIC	ENSG00000106069:[ENST00000222792(c.50-61765A>T)]	NA	NA	4.05E-02	NA	NA	4.05E-02	1.64E-02	NA	NA	NA	MODIFIER	NA	NA	196	194	0	141	91	50	35.46
Non-user	IOB_24	chr7	29771601	G	A	DPY19L2P3	EXONIC-NC	ENSG00000227855:[ENST00000414296(n.1691G>A)]	NA	NA	2.86E-02	NA	NA	2.86E-02	5.73E-02	NA	NA	NA	MODERATE	NA	NA	124	116	0	104	54	46	44.23
Non-user	IOB_24	chr7	29771601	G	A	LOC101928208	MISSENSE	101928208:[XM_005249924.1(c.169G>A,Gtg>Atg):XP_005249981.1(p.Val57Met)]	NA	NA	2.86E-02	NA	NA	2.86E-02	5.73E-02	NA	NA	NA	MODERATE	NA	NA	124	116	0	104	54	46	44.23
Non-user	IOB_24	chr7	29924041	C	G	WIPF3	MISSENSE	ENSG00000122574:[ENST00000242140(c.931C>G,Ccc>Gcc):ENSP00000242140(p.Pro311Ala)]	NA	NA	NA	8.58E-05	2.36E-04	NA	NA	T_lc	BN	NA	MODERATE	N	0.003	57	56	0	61	25	35	57.38
Non-user	IOB_24	chr7	30651875	T	G	GARS	INTRONIC	2617:[NM_001316772.1(c.869+14T>G)]	NA	NA	7.99E-04	2.01E-03	1.21E-03	7.99E-04	0.00E+00	NA	NA	NA	MODIFIER	NA	NA	234	233	0	177	96	81	45.76
Non-user	IOB_24	chr7	33312795	C	T	BBS9	MISSENSE	ENSG00000122507:[ENST00000425508(c.739C>T,Cca>Tca):ENSP00000405151(p.Pro247Ser)]	NA	NA	NA	NA	NA	NA	NA	T	PoD	NA	MODERATE	NA	NA	107	106	0	84	78	6	7.14
Non-user	IOB_24	chr7	36656001	C	T	AOAH	SILENT	ENSG00000136250:[ENST00000258749(c.831G>A,gcG>gcA):ENSP00000258749(p.Ala277(=))]	NA	NA	4.49E-02	2.74E-02	3.77E-02	4.49E-02	4.50E-02	NA	NA	NA	LOW	NA	NA	211	211	0	168	83	85	50.6
Non-user	IOB_24	chr7	38397867	G	C	TRG-AS1	NCT	100506776:[XR_242061.1(n.526-1186G>C)]	NA	NA	chr7*38397867*38397867*G*A,C*rs370983159*NA*NA*NA	2.12E-01	2.39E-01	NA	NA	NA	NA	NA	MODIFIER	NA	NA	33	33	0	36	24	12	33.33
Non-user	IOB_24	chr7	38534225	T	C	AMPH	INTRONIC	ENSG00000078053:[ENST00000325590(c.206-98A>G)]	NA	NA	2.56E-02	NA	NA	2.56E-02	1.09E-01	NA	NA	NA	MODIFIER	NA	NA	58	58	0	38	25	13	34.21
Non-user	IOB_24	chr7	39074090	C	T	POU6F2	INTRONIC	ENSG00000106536:[ENST00000403058(c.18+27623C>T)]	NA	NA	NA	2.24E-04	4.27E-04	NA	NA	T_lc	UN	NA	MODERATE	NA	NA	158	157	0	123	51	72	58.54
Non-user	IOB_24	chr7	43840111	G	A	BLVRA	MISSENSE	ENSG00000106605:[ENST00000265523(c.400G>A,Gct>Act):ENSP00000265523(p.Ala134Thr)]	NA	NA	1.20E-03	1.37E-03	6.48E-03	1.20E-03	6.10E-03	T	BN	NA	MODERATE	D	1	135	134	1	95	45	49	51.58

Patel *et al.* , 2021. Whole-exome sequencing analysis of oral squamous cell carcinoma delineated by tobacco usage habits
Supplementary Table 4. List of somatic single nucleotide variants identified using whole exome sequencing of OSCC patients

Cohort	Sample	Chromosome	Genomic position	Reference allele	Alternate allele	Genesymbol	Variant type	Variant description	OncoMD	COSMIC	dbSNP	ExAC	ExAC_SAS	1000G	1000G_SAS	SIFT	Polyphen2	CONDEL	VEP	LRT	PhastCon7Way	Total reads in Normal	Reference read support in Normal	Alternative read support in Normal	Total reads in Tumor	Reference read support in Tumor	Alternate read support in Tumor	Alternate allele frequency in Tumor
Non-user	IOB_24	chr7	43921261	T	C	URGCP	MISSENSE	ENSG00000106608:[ENST00000453200(c.199A>G,Aca>Gca):ENSP00000396918(p.Thr67Ala)]	NA	NA	9.39E-03	2.08E-02	2.65E-02	9.38E-03	1.84E-02	NA	NA	NA	MODIFIER	NA	NA	76	76	0	71	36	35	49.3
Non-user	IOB_24	chr7	43921261	T	C	URGCP-MRPS24	MISSENSE	ENSG00000270617:[ENST00000603700(c.172A>G,Aca>Gca):ENSP00000473871(p.Thr58Ala)]	NA	NA	9.39E-03	2.08E-02	2.65E-02	9.38E-03	1.84E-02	NA	NA	NA	MODIFIER	NA	NA	76	76	0	71	36	35	49.3
Non-user	IOB_24	chr7	44153614	C	G	AEBP1	SILENT	ENSG00000106624:[ENST00000450684(c.1956C>G,ggC>ggG):ENSP00000398878(p.Gly652(=))]	NA	chr7*44153614*44153614*COSM298987*C*G*1*large_intestine*1	4.57E-02	5.36E-02	1.61E-01	4.57E-02	1.52E-01	NA	NA	NA	LOW	NA	NA	172	166	0	185	93	89	48.11
Non-user	IOB_24	chr7	44442301	C	T	NUDCD3	NCT	ENSG00000015676:[ENST00000493613(n.195+1738G>A)]	NA	NA	chr7*44442301*44442301*C*A,T*rs764215413*NA*NA*NA	7.19E-04	1.31E-03	NA	NA	NA	NA	NA	MODIFIER	NA	NA	67	67	0	48	29	19	39.58
Non-user	IOB_24	chr7	47947684	C	T	HUS1	INTRONIC	ENSG00000136273:[ENST00000436444(c.*1170+56099G>A)]	NA	NA	5.99E-04	1.04E-03	5.23E-03	5.99E-04	1.00E-03	NA	NA	NA	MODIFIER	NA	NA	39	39	0	29	12	17	58.62
Non-user	IOB_24	chr7	47947684	C	T	PKD1L1	SILENT	ENSG00000158683:[ENST00000289672(c.1392G>A,gtG>gtA):ENSP00000289672(p.Val464(=))]	NA	NA	5.99E-04	1.04E-03	5.23E-03	5.99E-04	1.00E-03	NA	NA	NA	MODIFIER	NA	NA	39	39	0	29	12	17	58.62
Non-user	IOB_24	chr7	48048724	G	A	SUN3	INTRONIC	ENSG00000164744:[ENST00000449896(c.*117-52C>T)]	NA	NA	3.79E-03	3.31E-03	1.35E-03	3.79E-03	0.00E+00	NA	NA	NA	MODIFIER	NA	NA	25	25	0	22	8	14	63.64
Non-user	IOB_24	chr7	49842377	A	C	VWC2	MISSENSE	ENSG00000188730:[ENST00000340652(c.767A>C,cAg>cCg):ENSP00000341819(p.Gln256Pro)]	NA	NA	1.92E-02	3.95E-02	1.28E-02	1.92E-02	5.10E-03	NA	NA	NA	MODIFIER	NA	NA	158	157	0	142	62	78	54.93
Non-user	IOB_24	chr7	50560779	A	G	DDC	INTRONIC	1644:[NM_001242886.1(c.830+2269T>C)]	NA	NA	9.98E-04	NA	NA	9.98E-04	5.10E-03	NA	NA	NA	MODIFIER	NA	NA	72	72	0	73	35	38	52.05
Non-user	IOB_24	chr7	53104297	C	T	POM121L12	3UTR	ENSG00000221900:[ENST00000408890(c.*42C>T)]	NA	NA	1.46E-02	5.48E-03	4.65E-02	1.46E-02	6.03E-02	NA	NA	NA	MODIFIER	NA	NA	112	112	0	95	47	47	49.47
Non-user	IOB_24	chr7	55755533	G	A	FKBP9L	EXONIC-NC	ENSG00000176826:[ENST00000455909(n.474C>T)]	NA	NA	1.82E-02	3.39E-02	4.39E-02	1.82E-02	2.56E-02	NA	NA	NA	MODIFIER	NA	NA	283	281	0	233	116	115	49.36
Non-user	IOB_24	chr7	55755533	G	A	FKBP9P1	EXONIC-NC	360132:[NR_027340.1(n.193C>T)]	NA	NA	1.82E-02	3.39E-02	4.39E-02	1.82E-02	2.56E-02	NA	NA	NA	MODIFIER	NA	NA	283	281	0	233	116	115	49.36
Non-user	IOB_24	chr7	55779103	C	T	FKBP9L	EXONIC-NC	ENSG00000176826:[ENST00000441699(n.437G>A)]	NA	NA	NA	NA	NA	NA	NA	NA	NA	NA	MODIFIER	NA	NA	173	171	0	110	8	102	92.73
Non-user	IOB_24	chr7	55902230	T	C	SEPT14	MISSENSE	ENSG00000154997:[ENST00000388975(c.608A>G,gAt>gGt):ENSP00000373627(p.Asp203Gly)]	NA	NA	4.59E-03	1.38E-02	2.31E-03	4.59E-03	1.00E-03	D	PoD	NA	MODERATE	N	1	78	78	0	59	0	59	100
Non-user	IOB_24	chr7	56087300	C	T	PSPH	MISSENSE	ENSG00000146733:[ENST00000275605(c.268G>A,Ggc>Agc):ENSP00000275605(p.Gly90Ser)]	NA	NA	2.00E-04	5.06E-03	1.62E-02	2.00E-04	0.00E+00	NA	NA	NA	MODIFIER	NA	NA	105	103	0	116	77	12	10.34
Non-user	IOB_24	chr7	56087319	T	G	PSPH	MISSENSE	ENSG00000146733:[ENST00000275605(c.249A>C,caA>caC):ENSP00000275605(p.Gln83His)]	NA	NA	NA	8.51E-03	1.01E-02	NA	NA	T	BN	NA	MODERATE	NA	NA	114	111	2	142	94	45	31.69
Non-user	IOB_24	chr7	56087399	G	A	PSPH	MISSENSE	ENSG00000146733:[ENST00000275605(c.169C>T,Cct>Tct):ENSP00000275605(p.Pro57Ser)]	NA	NA	NA	1.20E-01	1.36E-01	NA	NA	T	BN	NA	MODERATE	NA	NA	83	82	0	144	89	53	36.81
Non-user	IOB_24	chr7	56087409	G	A	PSPH	MISSENSE	ENSG00000146733:[ENST00000427797(c.233C>T,gCg>gTg):ENSP00000403431(p.Ala78Val)]	NA	NA	2.00E-04	1.22E-01	1.36E-01	2.00E-04	0.00E+00	T_lc	BN	NA	MODERATE	NA	NA	69	69	0	131	76	51	38.93
Non-user	IOB_24	chr7	56087423	G	A	PSPH	MISSENSE	ENSG00000146733:[ENST00000275605(c.145C>T,Cgg>Tgg):ENSP00000275605(p.Arg49Trp)]	NA	chr7*56087423*56087423*COSM329573*G*A*1*haematopoietic_and_lymphoid_tissue*1	2.00E-04	1.47E-01	1.80E-01	2.00E-04	0.00E+00	NA	NA	NA	MODIFIER	NA	NA	67	67	0	119	68	50	42.02
Non-user	IOB_24	chr7	56088726	G	T	PSPH	INTRONIC	ENSG00000146733:[ENST00000275605(c.140+40C>A)]	NA	NA	NA	5.59E-04	7.09E-05	NA	NA	NA	NA	NA	MODIFIER	NA	NA	241	241	0	219	180	39	17.81
Non-user	IOB_24	chr7	56088780	G	A	PSPH	SILENT	ENSG00000146733:[ENST00000275605(c.126C>T,gaC>gaT):ENSP00000275605(p.Asp42(=))]	NA	NA	NA	7.83E-02	1.44E-01	NA	NA	NA	NA	NA	LOW	NA	NA	337	336	0	373	269	87	23.32
Non-user	IOB_24	chr7	56088786	A	T	PSPH	SILENT	ENSG00000146733:[ENST00000275605(c.120T>A,gtT>gtA):ENSP00000275605(p.Val40(=))]	NA	NA	NA	2.02E-01	2.68E-01	NA	NA	NA	NA	NA	LOW	NA	NA	343	343	0	380	273	88	23.16
Non-user	IOB_24	chr7	56088789	G	A	PSPH	SILENT	ENSG00000146733:[ENST00000275605(c.117C>T,ggC>ggT):ENSP00000275605(p.Gly39(=))]	NA	NA	NA	2.03E-01	2.70E-01	NA	NA	NA	NA	NA	LOW	NA	NA	341	340	0	380	272	91	23.95
Non-user	IOB_24	chr7	56088811	T	C	PSPH	MISSENSE	ENSG00000146733:[ENST00000275605(c.95A>G,gAt>gGt):ENSP00000275605(p.Asp32Gly)]	NA	NA	NA	1.74E-01	2.33E-01	NA	NA	NA	NA	NA	MODIFIER	NA	NA	332	331	0	372	258	111	29.84
Non-user	IOB_24	chr7	56088825	T	A	PSPH	MISSENSE	ENSG00000146733:[ENST00000275605(c.81A>T,agA>agT):ENSP00000275605(p.Arg27Ser)]	NA	NA	NA	1.84E-01	2.47E-01	NA	NA	NA	NA	NA	MODIFIER	NA	NA	307	305	0	352	236	116	32.95
Non-user	IOB_24	chr7	66648157	C	T	TYW1	SILENT	ENSG00000198874:[ENST00000359626(c.1743C>T,aaC>aaT):ENSP00000352645(p.Asn581(=))]	NA	NA	NA	2.24E-01	2.83E-01	NA	NA	NA	NA	NA	MODIFIER	NA	NA	191	190	1	144	71	71	49.31
Non-user	IOB_24	chr7	66648199	G	A	TYW1	SILENT	ENSG00000198874:[ENST00000359626(c.1785G>A,ggG>ggA):ENSP00000352645(p.Gly595(=))]	NA	NA	NA	2.35E-01	2.77E-01	NA	NA	NA	NA	NA	MODIFIER	NA	NA	186	185	0	144	73	69	47.92
Non-user	IOB_24	chr7	66660242	A	G	TYW1	MISSENSE	ENSG00000198874:[ENST00000359626(c.1895A>G,cAc>cGc):ENSP00000352645(p.His632Arg)]	NA	chr7*66660242*66660242*COSM150308*A*G*1*stomach*1	NA	2.08E-01	2.56E-01	NA	NA	NA	NA	NA	MODIFIER	NA	NA	288	287	1	115	93	22	19.13
Non-user	IOB_24	chr7	66660247	T	C	TYW1	SILENT	ENSG00000198874:[ENST00000359626(c.1900T>C,Ttg>Ctg):ENSP00000352645(p.Leu634(=))]	NA	chr7*66660247*66660247*COSM150309*T*C*1*stomach*1	NA	2.05E-01	2.51E-01	NA	NA	NA	NA	NA	LOW	NA	0.489	283	283	0	114	96	18	15.79
Non-user	IOB_24	chr7	72081774	A	G	TYW1B	MISSENSE	ENSG00000254184:[ENST00000435769(c.1669T>C,Tgt>Cgt):ENSP00000477053(p.Cys557Arg)]	NA	NA	NA	1.44E-01	2.06E-01	NA	NA	NA	NA	NA	MODIFIER	NA	NA	230	230	0	234	205	29	12.39
Non-user	IOB_24	chr7	72413874	T	C	POM121	SILENT	ENSG00000196313:[ENST00000257622(c.2547T>C,aaT>aaC):ENSP00000257622(p.Asn849(=))]	NA	NA	NA	3.26E-01	4.85E-01	NA	NA	NA	NA	NA	LOW	NA	0	70	68	2	34	23	10	29.41
Non-user	IOB_24	chr7	72440457	G	A	AC005488.11	NCT	ENSG00000205584:[ENST00000503899(n.58-31G>A)]	NA	NA	NA	NA	NA	NA	NA	NA	NA	NA	MODIFIER	NA	NA	20	19	0	19	0	18	94.74
Non-user	IOB_24	chr7	72440457	G	A	LOC100101148	NCT	100101148:[NR_104007.1(n.190-31G>A)]	NA	NA	NA	NA	NA	NA	NA	NA	NA	NA	MODIFIER	NA	NA	20	19	0	19	0	18	94.74
Non-user	IOB_24	chr7	72440457	G	A	STAG3L3	EXONIC-NC	ENSG00000174353:[ENST00000436857(n.803C>T)]	NA	NA	NA	NA	NA	NA	NA	NA	NA	NA	MODIFIER	NA	NA	20	19	0	19	0	18	94.74
Non-user	IOB_24	chr7	73020093	C	T	MLXIPL	MISSENSE	ENSG00000009950:[ENST00000395189(c.547G>A,Gtc>Atc):ENSP00000378616(p.Val183Ile)]	NA	NA	NA	2.75E-05	0.00E+00	NA	NA	NA	NA	NA	MODIFIER	NA	NA	92	91	1	77	41	36	46.75
Non-user	IOB_24	chr7	73117910	C	T	STX1A	NCT	ENSG00000106089:[ENST00000470878(n.689+175G>A)]	NA	NA	1.58E-02	NA	NA	1.58E-02	2.86E-02	NA	NA	NA	MODIFIER	NA	NA	64	64	0	57	27	29	50.88
Non-user	IOB_24	chr7	73117910	C	T	WBSCR22	3UTR	ENSG00000071462:[ENST00000423166(c.*496C>T)]	NA	NA	1.58E-02	NA	NA	1.58E-02	2.86E-02	NA	NA	NA	MODIFIER	NA	NA	64	64	0	57	27	29	50.88
Non-user	IOB_24	chr7	73152375	A	C	ABHD11	EXONIC-NC	ENSG00000106077:[ENST00000468998(n.420T>G)]	NA	NA	NA	NA	NA	NA	NA	NA	NA	NA	MODIFIER	NA	NA	154	154	0	123	67	55	44.72
Non-user	IOB_24	chr7	73442480	C	T	ELN	EXONIC-NC	ENSG00000049540:[ENST00000480728(n.7C>T)]	NA	NA	2.60E-02	2.11E-02	7.63E-02	2.60E-02	7.46E-02	NA	NA	NA	MODIFIER	NA	NA	90	90	0	67	34	33	49.25
Non-user	IOB_24	chr7	73471024	C	T	ELN	SILENT	ENSG00000049540:[ENST00000357036(c.1353C>T,gcC>gcT):ENSP00000349540(p.Ala451(=))]	NA	NA	3.99E-03	2.50E-03	1.78E-02	3.99E-03	1.94E-02	NA	NA	NA	LOW	NA	NA	159	158	1	85	44	40	47.06
Non-user	IOB_24	chr7	75168677	G	A	HIP1	SILENT	ENSG00000127946:[ENST00000434438(c.2874C>T,taC>taT):ENSP00000410300(p.Tyr958(=))]	NA	NA	3.20E-03	2.41E-03	1.44E-02	3.19E-03	1.64E-02	NA	NA	NA	LOW	NA	0.331	173	172	0	125	67	56	44.8
Non-user	IOB_24	chr7	75912246	G	C	SRRM3	INTRONIC	ENSG00000177679:[ENST00000326382(c.1596-36G>C)]	NA	NA	NA	NA	NA	NA	NA	NA	NA	NA	MODIFIER	NA	NA	70	70	0	38	26	12	31.58
Non-user	IOB_24	chr7	76058857	G	A	ZP3	MISSENSE	ENSG00000188372:[ENST00000336517(c.185G>A,aGc>aAc):ENSP00000337310(p.Ser62Asn)]	NA	NA	1.62E-02	6.58E-03	4.70E-02	1.62E-02	7.77E-02	D	BN	NA	MODERATE	N	0.959	216	215	0	177	80	97	54.8
Non-user	IOB_24	chr7	76111922	T	C	DTX2	SILENT	ENSG00000091073:[ENST00000307569(c.366T>C,acT>acC):ENSP00000305242(p.Thr122(=))]	NA	chr7*76111922*76111922*COSM602431*T*C*1*lung*1	1.62E-02	6.77E-03	4.80E-02	1.62E-02	7.67E-02	NA	NA	NA	LOW	NA	NA	59	59	0	86	38	48	55.81
Non-user	IOB_24	chr7	76134923	G	A	DTX2	EXONIC-NC	ENSG00000091073:[ENST00000468546(n.1053G>A)]	NA	NA	2.00E-03	9.27E-03	2.80E-02	2.00E-03	8.20E-03	NA	NA	NA	MODIFIER	NA	NA	256	252	1	207	101	103	49.76
Non-user	IOB_24	chr7	76239510	G	A	AC004980.7	NCT	ENSG00000205485:[ENST00000450661(n.605-15728G>A)]	NA	NA	2.20E-03	2.19E-03	1.32E-02	2.20E-03	9.20E-03	NA	NA	NA	MODIFIER	NA	NA	119	117	0	102	65	36	35.29
Non-user	IOB_24	chr7	76239510	G	A	LOC100133091	NCT	100133091:[NR_029411.1(n.625-15728G>A)]	NA	NA	2.20E-03	2.19E-03	1.32E-02	2.20E-03	9.20E-03	NA	NA	NA	MODIFIER	NA	NA	119	117	0	102	65	36	35.29
Non-user	IOB_24	chr7	76239510	G	A	LOC100505767	INTRONIC	100505767:[XM_005250741.1(c.830-69847C>T)]	NA	NA	2.20E-03	2.19E-03	1.32E-02	2.20E-03	9.20E-03	NA	NA	NA	MODIFIER	NA	NA	119	117	0	102	65	36	35.29
Non-user	IOB_24	chr7	76239510	G	A	POMZP3	3UTR	ENSG00000146707:[ENST00000275569(c.*20C>T)]	NA	NA	2.20E-03	2.19E-03	1.32E-02	2.20E-03	9.20E-03	NA	NA	NA	MODIFIER	NA	NA	119	117	0	102	65	36	35.29
Non-user	IOB_24	chr7	76239510	G	A	UPK3B	INTRONIC	ENSG00000243566:[ENST00000443097(c.*253-36597G>A)]	NA	NA	2.20E-03	2.19E-03	1.32E-02	2.20E-03	9.20E-03	NA	NA	NA	MODIFIER	NA	NA	119	117	0	102	65	36	35.29
Non-user	IOB_24	chr7	76240996	G	A	AC004980.7	NCT	ENSG00000205485:[ENST00000418663(n.606-14242G>A)]	NA	NA	NA	2.81E-02	5.63E-02	NA	NA	NA	NA	NA	MODIFIER	NA	NA	144	143	0	275	161	113	41.09
Non-user	IOB_24	chr7	76240996	G	A	LOC100133091	NCT	100133091:[NR_029411.1(n.625-14242G>A)]	NA	NA	NA	2.81E-02	5.63E-02	NA	NA	NA	NA	NA	MODIFIER	NA	NA	144	143	0	275	161	113	41.09
Non-user	IOB_24	chr7	76240996	G	A	LOC100505767	INTRONIC	100505767:[XM_005250741.1(c.830-71333C>T)]	NA	NA	NA	2.81E-02	5.63E-02	NA	NA	NA	NA	NA	MODIFIER	NA	NA	144	143	0	275	161	113	41.09
Non-user	IOB_24	chr7	76240996	G	A	POMZP3	INTRONIC	ENSG00000146707:[ENST00000275569(c.346-1464C>T)]	NA	NA	NA	2.81E-02	5.63E-02	NA	NA	NA	NA	NA	MODIFIER	NA	NA	144	143	0	275	161	113	41.09
Non-user	IOB_24	chr7	76240996	G	A	UPK3B	INTRONIC	ENSG00000243566:[ENST00000443097(c.*253-35111G>A)]	NA	NA	NA	2.81E-02	5.63E-02	NA	NA	NA	NA	NA	MODIFIER	NA	NA	144	143	0	275	161	113	41.09
Non-user	IOB_24	chr7	76619690	G	A	DTX2P1	NCT-SS-PRX	ENSG00000186704:[ENST00000425797(n.1017+4G>A)]	NA	NA	6.59E-03	NA	NA	6.59E-03	3.37E-02	NA	NA	NA	MODIFIER	NA	NA	38	38	0	19	11	8	42.11
Non-user	IOB_24	chr7	76619690	G	A	DTX2P1-UPK3BP1-PMS2P11	NCT-SS-PRX	ENSG00000265479:[ENST00000584900(n.413+4G>A)]	NA	NA	6.59E-03	NA	NA	6.59E-03	3.37E-02	NA	NA	NA	MODIFIER	NA	NA	38	38	0	19	11	8	42.11
Non-user	IOB_24	chr7	76619690	G	A	LOC100505767	INTRONIC	100505767:[XM_005250741.1(c.829+38860C>T)]	NA	NA	6.59E-03	NA	NA	6.59E-03	3.37E-02	NA	NA	NA	MODIFIER	NA	NA	38	38	0	19	11	8	42.11
Non-user	IOB_24	chr7	76619690	G	A	UPK3B	INTRONIC	ENSG00000243566:[ENST00000419923(c.*221-28451G>A)]	NA	NA	6.59E-03	NA	NA	6.59E-03	3.37E-02	NA	NA	NA	MODIFIER	NA	NA	38	38	0	19	11	8	42.11
Non-user	IOB_24	chr7	87190621	C	T	ABCB1	MISSENSE	ENSG00000085563:[ENST00000543898(c.593G>A,aGa>aAa):ENSP00000444095(p.Arg198Lys)]	NA	chr7*87190621*87190621*COSM1181290*C*A*1*large intestine*1	2.00E-04	1.16E-04	8.52E-04	2.00E-04	1.00E-03	D	BN	NA	MODERATE	D	1	194	193	0	151	84	67	44.37
Non-user	IOB_24	chr7	87822541	G	A	ADAM22	MISSENSE	ENSG00000008277:[ENST00000426930(c.959G>A,cGa>cAa):ENSP00000396233(p.Arg320Gln)]	NA	NA	3.99E-04	2.01E-04	7.38E-04	3.99E-04	2.00E-03	NA	NA	NA	MODIFIER	NA	NA	267	266	0	237	121	116	48.95
Non-user	IOB_24	chr7	89915616	G	C	C7orf63	MISSENSE	ENSG00000105792:[ENST00000316089(c.1559G>C,aGc>aCc):ENSP00000321753(p.Ser520Thr)]	NA	chr7*89915616*89915616*COSM747617*G*T*1*lung*1|chr7*89915616*89915616*COSM1150779*G*T*1*lung*1	chr7*89915613*89915617*TAAGC*T*rs777146307*NA*NA*NA|chr7*89915616*89915616*G*A*rs773263746*NA*NA*NA	NA	NA	NA	NA	T	BN	NA	MODERATE	NA	NA	45	45	0	45	18	27	60
Non-user	IOB_24	chr7	97361936	C	G	TAC1	SILENT	ENSG00000006128:[ENST00000319273(c.12C>G,ctC>ctG):ENSP00000321106(p.Leu4(=))]	NA	chr7*97361936*97361936*COSM453625*C*G*1*breast*1	5.59E-03	3.82E-03	2.80E-02	5.59E-03	2.86E-02	NA	NA	NA	LOW	NA	NA	290	287	0	234	139	95	40.6
Non-user	IOB_24	chr7	99690878	A	C	MCM7	INTRONIC	4176:[XM_005250348.1(c.1637+37T>G)]	NA	NA	7.39E-03	1.05E-02	2.29E-02	7.39E-03	2.56E-02	NA	NA	NA	MODIFIER	NA	NA	90	90	0	74	34	39	52.7
Non-user	IOB_24	chr7	99821725	T	G	GATS	NCT	ENSG00000239521:[ENST00000538588(n.159-27A>C)]	NA	NA	3.18E-02	4.54E-02	1.00E-01	3.17E-02	9.10E-02	NA	NA	NA	MODIFIER	NA	NA	55	55	0	56	32	23	41.07
Non-user	IOB_24	chr7	100033537	C	A	PPP1R35	MISSENSE	ENSG00000160813:[ENST00000292330(c.385G>T,Gcc>Tcc):ENSP00000292330(p.Ala129Ser)]	NA	NA	NA	NA	NA	NA	NA	NA	NA	NA	MODIFIER	NA	NA	176	176	0	170	99	71	41.76
Non-user	IOB_24	chr7	100033537	C	A	RP11-758P17.2	NCT	ENSG00000241357:[ENST00000492523(n.205+453C>A)]	NA	NA	NA	NA	NA	NA	NA	NA	NA	NA	MODIFIER	NA	NA	176	176	0	170	99	71	41.76
[truncated: 2,867,104 more chars]
